# Insights into psychosis risk from leukocyte microRNA expression

**DOI:** 10.1038/tp.2016.148

**Published:** 2016-12-13

**Authors:** C D Jeffries, D O Perkins, S D Chandler, T Stark, E Yeo, J Addington, C E Bearden, K S Cadenhead, T D Cannon, B A Cornblatt, D H Mathalon, T H McGlashan, L J Seidman, E F Walker, S W Woods, S J Glatt, M Tsuang

**Affiliations:** 1 Renaissance Computing Institute, University of North Carolina, Chapel Hill, NC, USA; 2 Department of Psychiatry, University of North Carolina, Chapel Hill, NC, USA; 3 Center for Behavioral Genomics, Department of Psychiatry, and the Institute of Genomic Medicine, University of California, San Diego, San Diego, CA, USA; 4 Division of Biological Sciences, University of California, San Diego, San Diego, CA, USA; 5 Department of Cellular and Molecular Medicine, University of California, San Diego, San Diego, CA, USA; 6 Hotchkiss Brain Institute, Department of Psychiatry, University of Calgary, Calgary, AB, Canada; 7 Department of Psychiatry and Biobehavioral Sciences, University of California, Los Angeles, Los Angeles, CA, USA; 8 Department of Psychology, University of California, Los Angeles, Los Angeles, CA, USA; 9 Department of Psychiatry, University of California, San Diego, San Diego, CA, USA; 10 Departments of Psychology and Psychiatry, Yale University, New Haven, CT, USA; 11 Department of Psychiatry, Zucker Hillside Hospital, Glen Oaks, NY, USA; 12 Department of Psychiatry, University of California, San Francisco, San Francisco, CA, USA; 13 Department of Psychiatry, Yale University, New Haven, CT, USA; 14 Department of Psychiatry, Harvard Medical School at Beth Israel Deaconess Medical Center and Massachusetts General Hospital, Boston, MA, USA; 15 Department of Psychology, Emory University, Atlanta, GA, USA; 16 Department of Psychiatry, Emory University, Atlanta, GA, USA; 17 Department of Psychiatry and Behavioral Sciences, SUNY Upstate Medical University, Syracuse, NY, USA

## Abstract

Dysregulation of immune system functions has been implicated in schizophrenia, suggesting that immune cells may be involved in the development of the disorder. With the goal of a biomarker assay for psychosis risk, we performed small RNA sequencing on RNA isolated from circulating immune cells. We compared baseline microRNA (miRNA) expression for persons who were unaffected (*n*=27) or who, over a subsequent 2-year period, were at clinical high risk but did not progress to psychosis (*n*=37), or were at high risk and did progress to psychosis (*n*=30). A greedy algorithm process led to selection of five miRNAs that when summed with +1 weights distinguished progressed from nonprogressed subjects with an area under the receiver operating characteristic curve of 0.86. Of the five, miR-941 is human-specific with incompletely understood functions, but the other four are prominent in multiple immune system pathways. Three of those four are downregulated in progressed vs. nonprogressed subjects (with weight -1 in a classifier function that increases with risk); all three have also been independently reported as downregulated in monocytes from schizophrenia patients vs. unaffected subjects. Importantly, these findings passed stringent randomization tests that minimized the risk of conclusions arising by chance. Regarding miRNA–miRNA correlations over the three groups, progressed subjects were found to have much weaker miRNA orchestration than nonprogressed or unaffected subjects. If independently verified, the leukocytic miRNA biomarker assay might improve accuracy of psychosis high-risk assessments and eventually help rationalize preventative intervention decisions.

## Introduction

Schizophrenia affects about 1% of the general population, typically emerges in late adolescence and early adulthood, and is usually chronic, relapsing and disabling.^1, 2^ However early identification and treatment of psychosis is associated with better clinical outcome,^3^ and interventions in persons experiencing high-risk symptoms show promise in preventing the development of psychosis.^4^ Clinical diagnostic criteria for the psychosis prodrome identify persons with a 13–22% 2-year psychosis risk.^5, 6, 7, 8, 9, 10, 11^ While much higher than the general population risk, this relatively low conversion rate hampers the development and implementation of preventative interventions. Thus, for persons at high risk a biomarker assay that improved risk prediction would be of great value. In addition, employed biomarkers may illuminate mechanisms involved in the emergence of schizophrenia and potentially point towards new therapeutic targets.

The tissue used for biomarker discovery should represent the disease pathology. Regarding schizophrenia, biomarker studies have often considered circulating immune cells as easily accessible proxies for the brain that may reflect an environmental or genetic vulnerability that is shared with the brain.^12, 13^ Moreover, peripheral immune cells are now known to regulate brain functions involved in schizophrenia, including cognition,^14^ behavioral responses to stress,^15^ neural plasticity^16^ and neurogenesis.^17, 18, 19^ In addition, converging reports from genetic, epidemiological, clinical and post-mortem studies implicate dysregulation of both the innate and adaptive immune systems in schizophrenia.^20^ In particular meta-analyses have found schizophrenia to be associated with elevations in blood levels of specific analytes,^21, 22^ as well as shifts in adaptive immune cell populations.^23^ We and others have found that alterations in plasma analyte levels predicted psychosis in persons meeting clinical criteria for psychosis risk.^24, 25^ Thus, peripheral immune cells may be of value for schizophrenia biomarker discovery because their dysregulation may be directly linked to emerging psychosis.

Here we report the results of small RNA sequencing on RNA isolated from circulating immune cells, emphasizing the comparison of expression in persons at clinical high risk for psychosis who progressed to psychosis (schizophrenia or a related disorder) vs. those who remained psychosis-free. We focused on microRNAs (miRNAs), ~22 nucleotide single-stranded RNA molecules that are now generally appreciated as regulators of mRNA processing in translation and doubtless involved in many developmental and pathological processes in animals.^26, 27, 28^ In particular, alterations in miRNA abundance may indicate a shift in immune system state. The present work offers a preliminary connection of immune cell miRNA levels and the likelihood of transition from clinical high risk to psychosis, providing further evidence of association of immune dysregulation.

## Materials and methods

As described previously, the North American Prodrome Longitudinal Study, Phase 2 (NAPLS 2)^29^ is an eight-site observational study of the predictors and mechanisms of conversion to psychosis in persons at elevated risk indicated by the Criteria of Psychosis-Risk States.^30^ The full NAPLS 2 cohort includes 764 high-risk and 280 demographically similar unaffected subjects between the ages of 12–35. The study was approved by the Institutional Review Board at each site, and each subject provided written informed consent or assent, with a parent or guardian consenting for subjects <18 years old.

In the present analysis, we included all high-risk subjects with RNA samples who had either progressed to psychosis within 2 years (*n*=30) or who remained nonprogressed at 2-year follow-up (*n*=37), as of February 2012. Also included were some unaffected comparison subjects (*n*=27) who did not meet high-risk criteria and had no personal or family history of a psychotic disorder.

### Assessments

Clinical assessments were done every 6 months and subjects followed for up to 2 years. Participants were screened using the Structured Interview for Psychosis-Risk Syndromes and rated with the Scale of Psychosis-Risk Symptoms as defined by the Criteria of Psychosis-Risk States: attenuated psychotic symptoms, brief intermittent psychotic symptoms, substantial functional decline combined with a first-degree relative with a psychotic disorder, or schizotypal personality disorder in individuals <18 years old.^30^ The Structured Clinical Interview for DSM-IV^31^ was used to determine psychiatric diagnoses.

Data on prescription medications were based on self-reports and/or parental reports. Socioeconomic status was estimated by maximum years of education of mother or father.

### Assays of leukocytes for miRNAs

Immediately after phlebotomy, leukocytes were isolated on a filter and RNA preserved with RNAlater (Qiagen, Venlo, The Netherlands). Samples were stored at −20 °C until processing. RNA was extracted using a modification of the LeukoLOCK procedure (Life Technologies, Foster City, CA, USA).^32^ Small RNA libraries were prepared with Illumina TruSeq kits (Illumina, San Diego, CA, USA) following manufacturer's protocol. Barcoded libraries were combined in equimolar amounts (10 nmol l^−1^ each), then diluted to 4 pmol l^−1^ for each flowcell lane and sequenced by Illumina HiSeq Sequencing Systems (Illumina).

The Illumina processing pipeline v1.5 (Illumina) was used for base-calling using the SCARF text format. Each of 2588 mature miRNA sequences from miRBase v21 was sought as an exact sequence match within each read. From an initial set of 100 samples, we excluded 6 (3 unaffected, 1 nonprogressed, 2 progressed) with low-abundance reads, leaving 27 unaffected and 67 high-risk subjects. The analysis was also repeated using trimmed subsequences of canonical miRNA sequences to account for isoform diversity.

### Normalisation

For all analyses, we included the 136 miRNAs that were robustly expressed, defined as ⩾10 000 total reads in the 94 subjects. However, different subjects sometimes had many miRNAs with high read counts or many with low read counts. For one pair of miRNAs, this skewness would already imply high correlations. Thus, to avoid spuriously high correlations from skewness, we divided read counts for each sample by the average of read counts for the top 30 miRNAs of that sample, forming quotients (Supplementary Materials and Methods) and reducing the ratios maximum:minimum among the top 30 miRNAs. For each miRNA, we then used the average and s.d. over all unaffected subjects' quotients for that miRNA to process all quotients into *z*-scores; final values for each miRNA were in a 4 × range over all 94 samples.

One nonprogressed sample was confidentially submitted in technical duplicates for sequencing. After normalization, the 136 robustly expressed miRNAs had a Pearson correlation of 0.61, achieving the 98.4th percentile of all 4371 possible correlations over 94 samples. If the 20 least-correlated miRNAs were dropped, the correlation rose to 0.80. Visually, the quality of this correlation as a test of the normalization process can be appreciated from a graph in Supplementary Materials and Methods. Numerically, the correlation in 136-dimensional space of two vectors having entries generated from a normal distributions would exceed 0.61 with probability 9.3E−15.

### Trimmed miRNA sequences

Each canonical mature miRNA sequence shown in miRBase is only a representative of multiple RNA species arising from the same precursor,^33^ and other isoforms might be important in cell functions.^34^ To investigate the potential impact of multiple isoforms, we trimmed two bases from the 5′ end and four bases from the 3′ end of each canonical sequence, completely retabulated matches, and reanalyzed read count data. This had the effect of multiplying the grand total of all miRNA matches by ~1.79. However, rerunning normalization and analyses with trimmed miRNA sequences led to similar choices of informative miRNAs for the full set of samples from trimmed vs. untrimmed data (Supplementary Materials and Methods). That is, the first, second, and fourth chosen miRNAs for the full data were, respectively, the first, second, fifth chosen for trimmed data, and so on.

### Construction of a classifier that best differentiated nonprogressed from progressed subjects

We detected ⩾1 reads for 1569 of the 2588 canonical miRNAs in miRBase v21 but limited our analyses to the 137 miRNAs with at least 10 000 reads over 94 subjects. One miRNA (miR-486-5p) was discarded as superabundant (62% of all reads), leaving 136 miRNAs.

We employed a greedy algorithm (Supplementary Materials and Methods) to develop our classifier. It selected first the one miRNA that best distinguished nonprogressed from progressed, based on the Student *t*-test *P*-value. The greedy algorithm then sought to add a weighted, second miRNA that best improved the overall Student *t*-test *P*-value, if possible. The greedy algorithm continued to add miRNAs to the sum until no improvement in the metric was possible or a limit was reached. We predicted (correctly) that classifier functions with low Student *t*-test *P*-values would also achieve high area under the curve of the receiver operating characteristic (AUC of ROC), thereby diversifying performance objectives.

Using the Student *P*-value as the selection metric was not logically the same as optimization of various geometric fits as in conventional linear regressions. That is, we sought to distinguish the groups as sets, not as abstract points in space separated by a hyperplane in some way. In our tests enforcing the same limits on the number of selected markers, the AUCs obtained were about the same or superior to those from standard geometric methods (data not shown).

**Figure 1:**
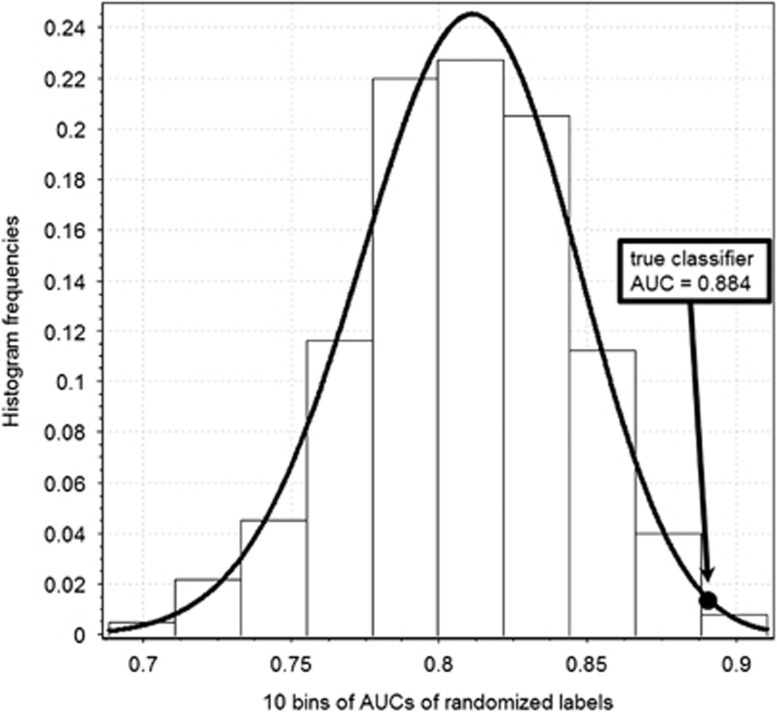
Histogram of one AUC from true data vs. 1000 AUCs of classifiers built by the same greedy algorithm applied to pseudo data (NP and P labels randomly permuted). Fitted with a beta distribution, the AUC from real data indicates a *P*-value of 0.012. Since 17 random AUCs of 1000 by chance exceed the true AUC, an algebraic method^36^ gives alternative *P*-value=0.018. Thus, the performance of the Greedy Algorithm limited to selection of at most six markers and applied to the full data set is unlikely to be chance. AUC, area under the curve of receiver operating characteristic.

Notably, the nonzero weights of miRNA values we used were all +1. In practice, the greedy algorithm typically terminated after ⩽10 iterations (selection of ⩽10 of 136 miRNAs, each weighted +1 in a classifier function). Considering its construction, we call this greedy algorithm ‘Coarse Approximation Linear Function' (CALF); a similar algorithm has been developed for distinguishing pairs of markers.^35^ CALF is now freely available at https://cran.r-project.org/web/packages/CALF/index.html. Although using real numbers as weights as in conventional regression would presumably yield better metric (and AUC) values, using +1 avoided instability. That is, setting aside a few samples and re-computing optimal real weights in conventional methods generally would not yield exactly the same real numbers, but stable (identical) weights would be more likely when limited to +1 (Supplementary Materials and Methods). In mathematical experiments with real data, we have found that in dimensions >5, using few +1 weights can approximate target functions almost as well as using the same number of real-valued weights. This surprising fact is due, in higher dimensions, to the exponentially increasing crowding of directions defined by all such coarse vectors (as rays from origin) among the directions defined by all real vectors as they penetrate the cube with vertex components +1 (Supplementary Materials and Methods). However, using any classifier algorithm that automatically selected a small subset of markers and otherwise passed rigorous randomization tests (as in Figure 1 above) would have been acceptable from the standpoint of prudent classifier construction processes that support reproducibility.

### Assay validation

Real time quantitative PCR validation of sequencing results was done with a total of 37 clinical high-risk subjects who did not progress to psychosis within 2 years and 30 others who did progress. Following conventional reverse transcription, cDNA synthesis, and preamplification steps, samples were assayed with high-throughput real-time qPCR (HT-PCR) using Fluidigm technology (San Francisco, CA, USA). A total of 21 miRNAs were assayed, including the 5 miRNAs selected in the classifier function (equation (1)). Data for 32 nonprogressed subjects and 24 progressed subjects could be compared with read counts. The 56 Spearman correlation of PCR values vs. RNA-seq read values averaged 0.64 (s.d. 0.11); minimum was 0.30. It follows that the 56 Spearman correlations with such values would arise by chance with probability <5.2E−30.

Furthermore, a simple (but likely suboptimal) way to modify the classifier (equation (1)) to use PCR data is to find for each of the subjects the average over the 21 miRNAs of Cq values. The averages can be subtracted from the raw Cq values to reduce gross sample-to-sample biases. A signal remains when selected miRNAs are relatively high within one group and low in the other. The Student *P*-value for such a classifier from equation (1) was 0.0012 and the AUC was 0.72.

In summary, PCR values and RNA-seq values were consistent. However, switching to PCR technology in an extension of the present work would properly assay anew a pool of the five selected miRNAs in equation (1), as well as other miRNAs that could be selected by CALF if the five were disallowed, all leading to a revised classifier function similar but not necessarily identical to equation (1).

### Randomization tests to assess significance of classification

Prudent case/control classifier construction can include six steps. First, a classifier algorithm is applied to true data, yielding a performance number (for example, AUC). Next, case and control memberships of all samples are randomly permuted, the model-building algorithm is re-applied to the pseudo data exactly as to the true data, and the performance number recorded—all repeated multiple times (for example, 1000 times). If such randomization tests indicate clear ability of the algorithm to distinguish case from control, further development of the classifier is indicated. Then the same algorithm is applied multiple times (for example, 1000 times) to random 80% subsets of true cases and controls. The resulting classifiers are integrated to produce a final classifier. The integrated classifier is tested with true data. If the integrated classifier performs well, then it is applied to external data (beyond the means and scope of the present pilot study). This general process and its many modern enhancements are widely used in drug design, development of cancer biomarkers, genomic research and other fields.^37, 38, 39, 40, 41^ It can be attempted with any classifier algorithm. We emphasise publication of histograms of randomization tests (see Figure 1) or the logical equivalent to reduce readers' scepticism. For example, a successful training set histogram would reduce likelihood of someone repeatedly adjusting an algorithm *post hoc* to optimize performance with both training and external data.

It should be emphasized that the randomization tests herein are applied to AUCs of true and pseudo classifiers; this is logically distinct from applying the true classifier to true and randomized data, as is often done.

Nonetheless, best practices do not insure that the only or best classifier has been developed. Furthermore, selected markers might only be surrogates for some deeper, causal markers unknown or inaccessible to the experimenter. It is always possible that systematic bias could have entered the analysis as misdiagnoses or some fundamental chemical bias in RNA-seq processing. Ultimately, the true utility of classification by miRNAs will rest with testing samples from additional subjects.

## Results

### Participant characteristics

Table 1 provides a description of subjects. All at high-risk met attenuated psychosis criteria. The 30 progressed included: 14 with schizophrenia; 11 with psychosis, not otherwise specified; 2 with major depression with psychotic features; and 1 each with schizoaffective, delusional and psychotic bipolar disorder.

### Using individual miRNAs

The smallest Student *t*-test *P*-value for a miRNA for nonprogressed vs progressed data was α=0.0053 (miR-941) (see Supplementary Materials and Methods). Thus, Bonferonni control for multiple comparisons over 136 miRNAs would declare no individual miRNA as a statistically significant biomarker. However, as explained by Fredrickson *et al.*,^42^ it can indeed happen that the association between a set of markers and phenotypes reaches a high level of reliability while individual markers in the same set fail to do so. This principle is heavily employed in the present work.

### Using sets of miRNAs for psychosis-risk prediction

**Figure 2:**
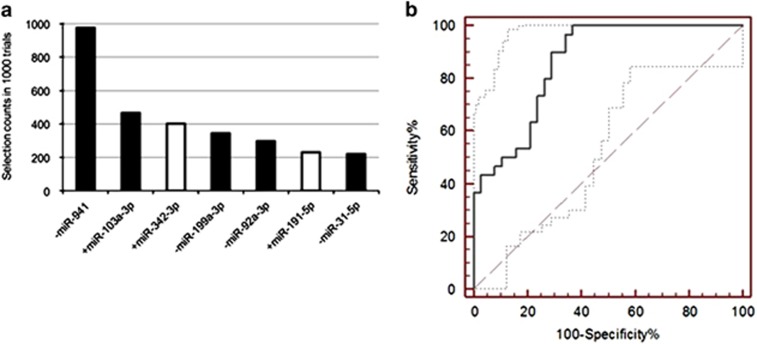
(**a**) The Greedy Algorithm was applied to 1000 selections of random 80% subsets of nonprogressed subjects and random 80% subsets of progressed subjects. Each time up to six markers could be chosen. The seven most frequently chosen markers are shown with their selection rates. The solid bars indicate the five markers that were also selected in the first six markers chosen by the Greedy Algorithm for the full data set (Figure 1), yielding as a sum of *z*-scores the classifier function in sum (equation (1)). This function applied to the full data yields AUC=0.86. (**b**) ROC of the five-miRNA classifier function (equation (1)). Dotted lines are 95% confidence levels, and the dashed line is hypothetical performance of a random classifier. AUC, area under the curve; miRNA, microRNA; ROC, receiving operating characteristics.

The performance of the miRNA classifier developed from all nonprogressed and progressed subjects using the sum of the first six miRNAs chosen by CALF was AUC=0.88. This value was superior to 983 of 1000 AUCs from exactly the same algorithm applied to randomized data (Figure 1). Next we applied CALF to 1000 random selections of 80% subsets of nonprogressed subjects and 80% subsets of progressed subjects. The 7 miRNAs that were selected in at least 225 of the 1000 trials are shown in Figure 2, and 5 of these were also among the six in the initial classifier developed from all subjects. Our integrated classifier was thus the sum of the five miRNAs chosen by both approaches, where the '+' means the value (*z*-score of normalized data) is added and the '–' means the value is subtracted from the final score:


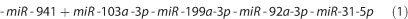


The classifier function (equation (1)) generally results in higher values for progressed (mean=1.24, s.d.=0.27) than nonprogressed subjects (mean=-0.78, s.d.=0.22), with AUC=0.86 (Figure 2). In addition (equation (1)) achieved AUC=0.75 on application to unaffected vs progressed subjects. Regarding ranks of total read counts among the 136 miRNAs, those in equation (1) ranked 39, 18, 119, 1, 115, respectively, implying the 5 miRNAs in equation (1) have diverse frequencies among the top 136.

Random permutations of true data will have chance patterns that algorithms can exploit to create seemingly convincing classifiers. As shown in Figure 1, many pseudo classifiers achieved AUCs well in excess of 0.5, the customary value of random classifiers using prior probabilities. However, as shown in Figure 1, only 17 of 1000 pseudo AUCs exceeded the true AUC, yielding^36^ a *P*-value of (17+1)/(1000+1)=0.018. Alternatively, fitting a beta distribution to a histogram of pseudo AUCs using EasyFit (MathWave, Dnepropetrovsk, Ukraine) led to an estimated *P*-value =0.012.

Prescription medication use is common in subjects at high-risk of psychosis^43^ (Table 1) and may affect leukocytic miRNA expression. To investigate the effects, the 5-miRNA sum (equation (1)) was applied to 1000 random selections of 25 samples from the set of nonprogressed and 25 samples from the set of progressed subjects. The average AUC was 0.86 with s.d.=0.029. We then selected 1000 times random subsets in 2 ways: to maximize or minimize the number of treated subjects plus a number of untreated subjects needed to make a total of 25. The resulting AUCs were in [0.83, 0.87]. This experiment therefore provided evidence of little influence of medications on the performance of the classifier (equation (1)) (Supplementary Materials and Methods, Supplementary Table S1).

### miRNA–miRNA correlation networks within groups

We next explored the degree of co-regulation among 136 robustly expressed miRNAs. We randomly selected 1000 times sets of 25 subjects from each of the 3 groups. From each selection, Pearson correlations of all 9180 distinct pairs of 136 miRNAs were calculated. Surprised by the consistent differences group vs. group, we redid all calculations with random subsets of 23 and 21 subjects instead of 25. This yielded essentially the same patterns (Supplementary Materials and Methods). Correlation graphs of miRNA data have been reported elsewhere in neuroscience.^44^

**Figure 3:**
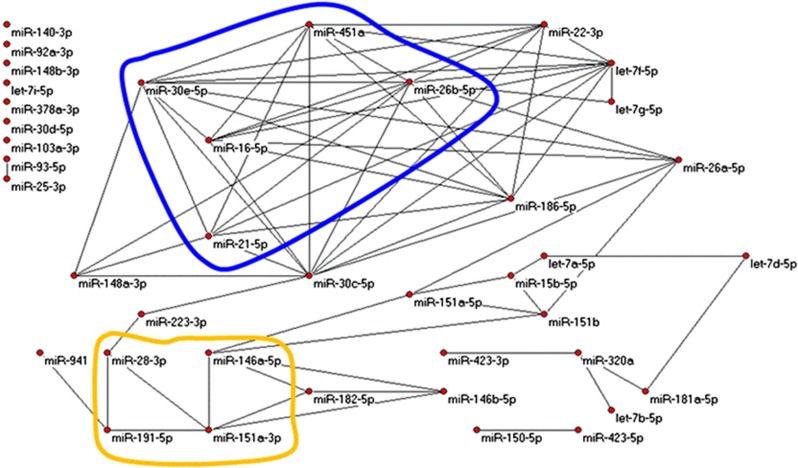
Graphs from miRNA–miRNA correlations. Edges represent strong correlations. Looped regions are common subgraphs. Networks shown are strongly correlated miRNAs among unaffected controls. miRNA, microRNA.

**Figure 4:**
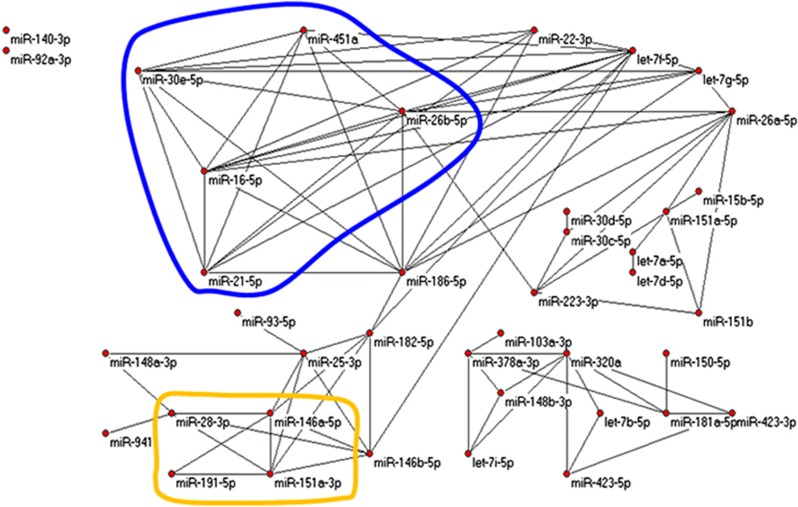
Graphs from miRNA–miRNA correlation with the same criteria as in Figure 3 but for nonprogressed subjects. This graph is similar to that in Figure 3. miRNA, microRNA.

**Figure 5:**
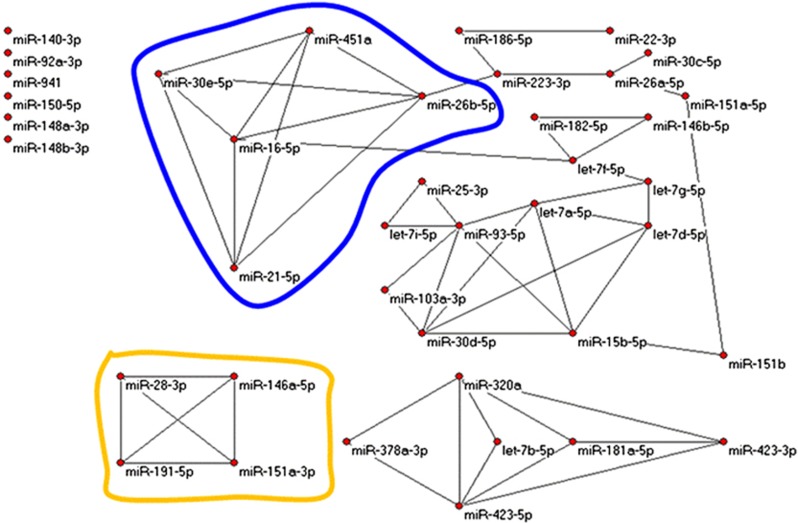
Graphs from miRNA–miRNA correlations with the same criteria as in Figures 3 and 4 but for progressed subjects. Evidently much organisation of miRNA networks is lost in subjects who eventually progressed to psychosis. miRNA, microRNA.

Restricting attention to the 40 most robustly expressed miRNAs (that include 3 miRNAs from equation (1), namely, miR-941, miR-103a-3p, and miR-92a-3p), we contrasted miRNA–miRNA correlation networks over the 3 groups by randomly selecting 1000 times subsets of 25 samples from each group and calculating all 780 Pearson correlations of pairs (from 40 miRNAs represented by 25-dimensional vectors). In each group and for each pair of miRNAs, we tabulated the number of times of 1000 possible that the correlation exceeded 0.5878 (*P*-value=~1.00E−3 for normally distributed 25-dimensional vectors, hence expecting 0.78 times among 780 random pairs to exceed that threshold). Such correlations were much more frequent than chance; those occurring in >500 of 1000 trials became edges in graphs in Figures 3, 4, 5 (drawn with Pajek http://pajek.imfm.si/doku.php). Clearly, highly correlated miRNAs were more numerous in nonprogressed and unaffected subjects than in progressed subjects. Notably, miR-941 and miR-103a-3p were both included in the correlation networks for unaffected and nonprogressed subjects but absent from the network for progressed subjects.

### Bioinformatic analyses

Although the seed region (nucleotides 2 through 8, numbered from 5′ end) was originally proposed to define the agency of miRNA targeting,^45, 46^ recent reports suggest that all of the mature miRNA sequence may be involved. Other types of targeting include ‘offset' (starting at base 3), ‘supplementary' (additional binding in a second region) and ‘compensatory' (supplementary targeting that tolerates limited mismatches in the seed).^47^ Also, ‘centered' sites were found as a class of miRNA target sites that lack both perfect seed pairing and 3′-compensatory pairing and instead have 11–12 contiguous Watson–Crick pairs near the center of the canonical miRNA.^48^ Regarding prevalence of non-seed binding, a transcriptome-wide survey for miR-155 found that ~40% of miR-155-dependent Argonaute (Ago) binding occurs at sites without perfect seed matches.^49^ In mouse brain, G-bulge sites (positions 5 or 6 in the seed) were found often bound and regulated by miR-124, and more generally, bulged sites comprise ⩾15% of all Ago-miRNA interactions.^50^ An analysis of 18 000 high-confidence miRNA–mRNA interactions found ~60% of seed interactions to be noncanonical, containing bulged or mismatched nucleotides.^51^ In summary, evidence suggests miRNA targeting is not necessarily a function of base pairing of the seed region.

Moreover, many canonical miRNAs are very similar as sequences. To filter our list of 136, we used Ingenuity (QIAGEN). The Ingenuity list typically represents sets of miRNAs with very similar sequences by just one from the set. We noted that consequently only 92 of the 136 robustly expressed miRNAs were in the Ingenuity miRNA targeting database (for example, the nine let-7 species in our 136 were represented by let-7a-5p). There are 5 miRNAs in equation (1), so 10 pairs. We calculated the Smith–Waterman sequence alignment score^52^ for all 10 pairs using weights: match +1, mismatch −1, gap −1. Pairs of the five miRNAs in equation (1) had strong similarities. For example, the score 11 − 0 − 2 = 9 is reached for miR-199a-3p 5′-ACAGUAGUCUGCACAU_UGGUUA-3' and miR-941 5'-CACCCGGCUGUGUGCACAUGUGC-3′ because they contain the common subsequence GU-UGCACAU-UG (Supplementary Materials and Methods).

The average of the 10 alignment scores was 6.4. Then we selected 1000 times a random set of 5 miRNAs from the 87 in Ingenuity excluding the 5 in equation (1) and calculated the 10 Smith–Waterman scores and their 1000 averages. The average was 5.1. A total of 19 times of 1000 the random averages were ⩾6.4. Thus, the Monte Carlo^36^ estimated *P*-value that the true sequence similarities are due to chance is 0.02 (Supplementary Materials and Methods). Other Smith–Waterman weight choices including extreme choices with mismatch or gap equal to −100 (so exact match subsequences) led to the same conclusions (data not shown). In summary, taken as full, canonical sequences, the five miRNAs selected in equation (1) from nonprogressed vs progressed analysis were as sequences more similar than would be expected by chance.

## Discussion

This study suggests that expression patterns of small, regulatory miRNAs in leukocytes differentiate persons at clinical high risk for psychosis who subsequently develop psychosis from those who do not. While no single miRNA exhibits statistically significant predictive power, we found that a sum of five abundantly expressed miRNAs produced a risk classifier that survives randomization testing. That is, stringent randomization tests have implied: original data likely contained miRNA information that distinguished nonprogressed from progressed; the normalization procedure we used likely did not obliterate that information; and the greedy algorithm (CALF) found a classifier function with AUC performance better than chance would readily allow. Randomization tests might seem obviously necessary, but many reports of classifier constructions in the scientific literature do not include them.

Our results are consistent with a study conducted by Gardiner *et al.*^53^ that compared miRNA expression in 112 schizophrenia subjects to that of 76 unaffected comparison subjects. They reported 83 miRNAs as downregulated, 30 of which were robustly expressed and thus considered in our analyses; remarkably, miR-92a-3p, miR-199a-3p, miR-31-5p in equation (1) were among these. There was, however, no apparent overlap between our study and a second investigation of monocyte miRNA expression in schizophrenia.^54^ Certain other studies did not consider the five miRNAs in equation (1) in their analyses.^55, 56, 57, 58^

miRNA regulation of gene expression in humans is based on imperfect base-pair binding of the mature miRNA to a targeted mRNA. The canonical sequences of the five miRNAs selected from nonprogressed vs. progressed analysis in equation (1) are significantly more similar than expected. This finding implies that these five miRNAs may in some way co-regulate gene expression and may be in themselves co-regulated. Finally, the remarkably different miRNA–miRNA correlation networks in Figure 3 suggest a shift in network orchestration in persons who progressed to psychosis.

Our findings require further investigation in terms of affected genes and cellular products. Most informative would be mRNA:miRNA co-expression analyses. Assuming correct and representative sampling and reproducibility of lab assays, and noting favorable outcomes of randomization tests, the classifiers and regulatory network patterns herein are unlikely to be due to chance. However, verification is needed with additional samples, possibly using an alternative assay technology such as HT-PCR.

## Figures and Tables

**Table 1:** Demographic and clinical characteristics of study subjects

	*Unaffected comparison (UC)* n*=29*	*Clinical high risk, not psychotic (CHR-NP)* n*=37*	*Clinical high risk, psychotic (CHR-P)* n*=30*
Age, average (s.d.)	19.3 (4.4)	18.1 (3.8)	18.7 (3.7)

*Ancestry*			
% Caucasian	61%,	65%	52%
% African	32%	13.5%	19%
% Asian	7%	13.5%	19%
% Mixed	0%	8%	10%
			
Sex, % male	68%	62%	74%
SES, average (s.d.)	7.5 (1.7)	6.5 (1.7)	6.2 (1.6)

*Peripheral blood mononuclear cells*			
Neutrophils %	56 (11)	55 (13)	55 (10)
Lymphocytes %	34 (9)	35 (11)	33 (9)
Monocytes %	8 (2)	7 (3)	8 (3)
Eosinophils %	2 (3)	2 (2)	2 (2)
Basophils %	1 (1)	1 (1)	1 (1)

*SOPS scores, average (*s.d.*)*			
Total^a^	4.8 (5.3)	36.8 (12.4)	45.0 (13.0)
Positive^a^	1.3 (1.8)	12.6 (4.4)	13.9 (3.7)
Negative^a^	1.3 (1.8)	11.5 (5.9)	14.0 (5.9)
Disorganized^a^	.8 (1.1)	4.9 (2.6)	6.2 (3.4)
			
General^a^^,^^b^	1.4 (1.7)	7.8 (4.5)	10.9 (4.7)

*Prescription medication*			
Antipsychotic^c^	0%	27%	13%
Antidepressant^d^	3%	24%	23%
Stimulant	0%	7%	6%
Mood stabilizer	0%	0%	3%
Benzodiazepine^e^	0%	3%	13%
NSAID	0%	0%	0%
Antibiotic	0%	0%	0%

*Substance use*			
Tobacco use^f^	7%	30%	39%
Alcohol use	41%	38%	35%
Marijuana use^g^	7%	24%	32%

Abbreviations: NSAID, non-steroidal anti-inflammatory drug; SES, socioeconomic status.

a CHR-P vs UC *t*-test *P*-value<0.0001, CHR-NP vs UC *t*-test *P*-value<0.0001.

b CHR-P vs CHR-NP *t*-test *P*-value=0.02.

c CHR-P vs UC FET *P*-value=0.047, CHR-NP vs UC FET *P*-value=0.001.

d CHR-P vs UC FET *P*-value=0.011, CHR-NP vs UC FET *P*-value=0.002.

e CHR-P vs UC FET *P*-value=0.047.

f CHR-P vs UC FET *P*-value=0.001, CHR-NP vs UC FET *P*-value=0.02.

g CHR-P vs UC FET *P*-value=0.020, CHR-NP vs UC FET *P*-value=0.056.
