# Supplementary material for: Insights into psychosis risk from leukocyte microRNA expression
Source: Transl Psychiatry. 2016 Dec 13;6(12):e981–. doi: 10.1038/tp.2016.148 (PMC5290334; doi:10.1038/tp.2016.148)
Supplement: Supplementary Information, Figures and Tables 1 and 2 [file tp2016148x2.docx]

Insights into Psychosis Risk from Leukocyte microRNA Expression

Supplementary Materials and Methods

Contents:

1. Normalization

2. Trimmed sequences

3. Greedy algorithm: Coarse Approximation Linear Function (CALF)

4. Selection of linear weights

5. Best practices for building and testing classifiers

6. Medications

7. Smith-Waterman similarity of selected mature miRNA sequences

8. Validation of RNA-seq by PCR

9. Data from raw read counts to z-scores (spreadsheet Table S3)

Figures:

Fig. S1. Data for two samples that were technical duplicates.

Fig. S2. Flow diagram of the CALF algorithm.

Fig. S3. Random labeling and random selection flowchart.

Fig. S4. A high-level flowchart adapted from Edgington(1) including randomization tests of proposed classifiers.

Fig. S5. A histogram of 1001 AUC values.

Fig. S6. Comparison of 1000 averages of Smith-Waterman scores with the Smith-Waterman score of the five miRNAs selected to differentiate nonprogressed *v* progressed subjects.

Tables:

Table S1. Comparison of AUCs that considers medications

Table S2. Summary of five chosen miRNAs in a classifier function and their Smith-Waterman alignment scores

Table S3. Data from raw read counts to z-scores (spreadsheet)

1. Normalization

RNA-Seq refers to measurements of populations of general transcription species, not just miRNAs as in the present work. As noted by Kozomara and Griffiths-Jones, even restriction to miRNAs carries complications because both canonical mature miRNAs and multiple isoforms per miRNA can be expected within reads(2). But setting aside for now such complications, the goal of normalization is selection of a simple process that preserves miRNA expression information based upon canonical miRBase sequences.

First, we noted miR-486-5p (as its canonical mature sequence exactly included as a subsequence in reads) accounted for 62% of the grand total of 3.63E8 miRNA instances (spreadsheet in Table S3). Since we sought informative sets of miRNAs, the overwhelming miR-486-5p levels were discarded. Retained otherwise were all miRNAs with at least 10 000 reads in total over all 94 samples, leaving 136 robustly expressed miRNAs for further analyses. However, processed as below, miR-486-5p was distinguished in none of the groups.

Many normalization methods are described in the literature, and some may be summarized as follows.

Smalheiser(3) et al. used division of miRNA read counts by the read counts for a certain endogenous miRNA, miR-139-5p. Several tests in their paper served to corroborate findings using miR-139-5p. This is a form of flattening samples or "library size normalization." A goal is to nullify the effects of pipetting variability, leaving relative sample read counts that can be meaningfully compared.

Other papers used published data on housekeeping genes or used spike-ins. The implication is that some aspects of the data should match after normalization, guiding the choice of normalization method.

Mortazavi et al.(4) used RPKM = (reads per kilobase of exon model)/(million mapped reads), another type of library size normalization. This was in the early days of 25-base reads. The goal was to infer relative molar concentrations of transcripts from segments of long transcripts from genes. They verified the approach with spike-in experiments.

Wang et al.(5) referred to Mortazavi and others as providing evidence that RNA-Seq can capture transcriptome dynamics without sophisticated normalization of data sets, that is, with simply counting reads.

Robinson and Oshlack(6) asserted that reproducibility in spike-in experiments can be itself difficult to achieve. They studied normalization by a trimmed mean of fold change values (TMM) (trimming = ignoring genes with zero counts). They found that TMM normalization better explained housekeeping gene data than library size normalization.

Drewe et al.(7) developed two methods to include effects of different isoforms with or without annotations. They employed steps logically equivalent to library normalization.

Dillies et al.(8) noted that the increasing number of RNA-Seq normalization methods made selection of a method challenging. They analyzed patterns from raw values versus patterns from seven normalization methods. Designing experiments to generate no difference in power among all eight methods, they found lower false positive rates with TMM and DESeq (a Bioconductor package); RPKM was especially prone to high false positive rates.

We chose a version of library size normalization, described as follows. Among the 136 robustly detected miRNAs in 94 samples, we observed highly skewed read counts. To avoid spuriously high correlations from skewness itself, we divided read counts for each sample by the average of read counts for the top 30 miRNAs of that sample, forming quotients. This flattened the miRNA numbers and made the maximum:minimum ratios of counts among the top 30 miRNAs for each sample less extreme. For each miRNA, we then used the average and standard deviation (sd) over all unaffected controls of those quotients to convert all quotients to z-scores; final values among the top 30 miRNAs were in a 4X range for each sample. Our process is available in detail from raw read counts to z-scores in a supplementary spreadsheet, Table S3.

In conclusion, although there is no universal agreement about RNA-Seq normalization methods, some form of library size normalization is probably acceptable for miRNA studies in which all sought patterns are completely within single reads. To some extent, the normalization method we chose was justified by comparison of duplicates (described below) and by the overall finding of classifiers that survived randomization tests.

Evidence for the preservation of information after our normalization

Technical duplicates of one nonprogressed sample were assayed twice as samples B_33 and C_2. The original tube of blood was divided into two equal aliquots and each portion was subsequently treated as an independent sample without awareness of the assaying laboratory.

After normalization as described above, correlation of the duplicates over all 136 miRNAs was 0.61; this was at the 98.4^th^ percentile of correlations of all 4465 possible pairs of 94 samples. Correlations >0.61 in 136-dimensional space would be very unlikely among random, normally distributed vectors; p-value would be 1.60E-15.

Considering correlations over all 136 samples, if the 20 miRNAs with lowest differences are dropped, the resulting two 116-dimensional vectors have correlation 0.80. The five miRNAs selected for the classifier function all are among the 116.

The quality of replication is suggested in the following Fig. S1.


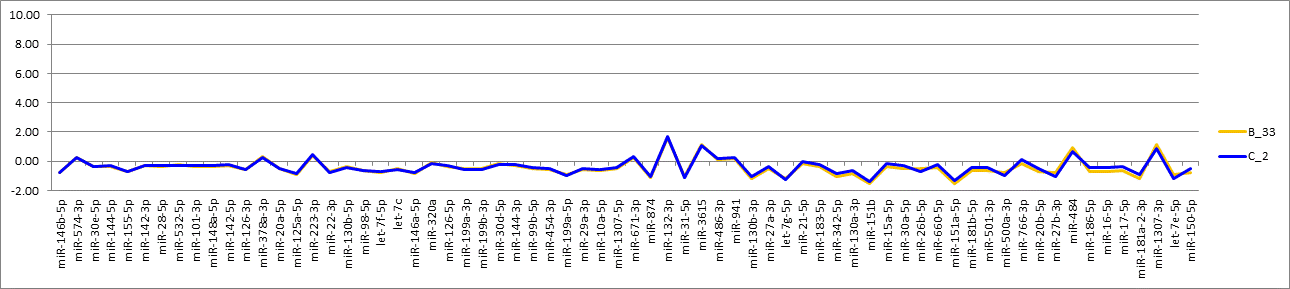


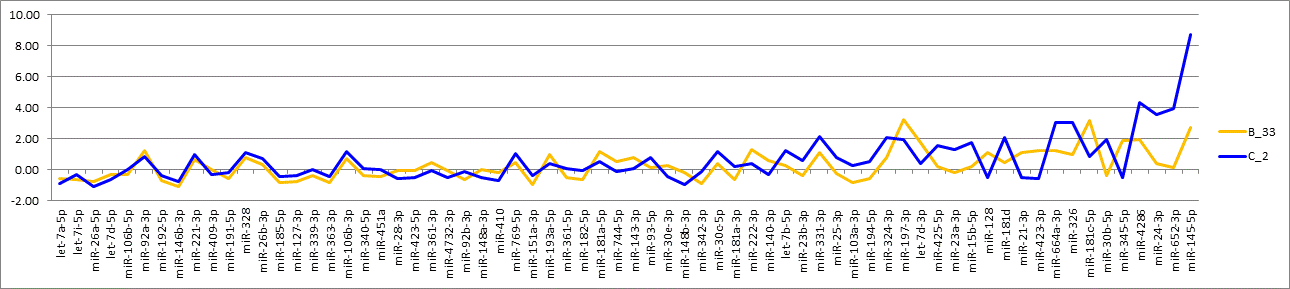


Fig. S1. Data for two samples that were technical duplicates. Shown are normalized z-scores of duplicated samples for 136 robustly expressed miRNAs, sorted by increasing absolute values of differences. For all but the last 20 or so miRNAs the duplication is excellent to good, implying that RNA-seq methods we used are reproducible for some but not all miRNA mature sequences.

2. Trimmed sequences

Different isoforms of one miRNA can appear in reads at different rates relative to the canonical miRBase mature sequence(9). That is, each canonical mature miRNA sequence shown in miRBase at http://www.mirbase.org/ actually represents multiple RNA species arising from the same precursor(10). The noncanonical isoforms of any miRNA might be important in cell functions. To investigate the consequences of seeking different isoforms in RNA-Seq data, we trimmed two bases from the 5’ end and four bases from the 3’ end of the canonical sequences and completely retabulated matches and reanalyzed data. We restricted classifier construction to the same 136 miRNAs. This had the effect of multiplying the grand total of all miRNA matches by 1.79. With this approach several shortened sequences embedded in reads were counted as equally significant for each miRNA. However, upon rerunning normalization and analysis with trimmed miRNA sequences, the first, second, and fourth chosen miRNAs were the same as the first, second, and fifth chosen with full mature sequences, respectively. Using trimmed sequences, the area under the curve (AUC) of the receiver operating characteristic (ROC) for the classifier function built with the first six miRNAs and applied to all nonprogressed *v* progressed samples was 0.90 (compared to 0.88 for canonical miRNAs).

Conventionally, qRT-PCR has been used to confirm at least the fold change directions from RNA-Seq data, and in some reports this yields good quantitative agreement(11). However, it is not practical to definitively assay the full range of all possible isoform molecules representing each of 2588 miRNA species(12).

In light of the above reasoning, we chose to base our analyses upon canonical miRBase mature sequences.

3. Greedy algorithm: Coarse Approximation Linear Function (CALF)

The Student t-test is applicable to two sets of numbers such as values of a classifier function over two groups of samples. It is derived from optimization of a fraction—the Student t-test statistic. The numerator is the absolute value of the difference of averages of two sets of values. The denominator is derived from the square root of a sum of the squares of standard deviations of the two sets of values. For given sample sizes, the larger the t-test value (the fraction), the smaller the p-value. Thus, we used Student t-test p-value as a score in a greedy algorithm. Importantly, this implies the selected classifier function will not generally optimize some geometric function and so will be different from conventional linear regression optimizations. We expected and eventually observed that improving (decreasing) this set-derived (not geometry-derived) score corresponded to improving (increasing) the AUC values of the classifier built by application of the algorithm.

We call the basic greedy algorithm (a simple version of forward selection linear regression) used herein Coarse Approximation Linear Function algorithm (CALF). It is shown as a flowchart in Fig. S2.


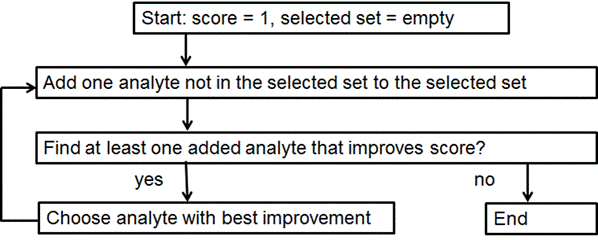


Fig. S2. Flow diagram of the greedy algorithm Coarse Approximation Linear Function (CALF).

A flowchart that is more detailed and that accommodates randomization tests follows in Fig. S3.


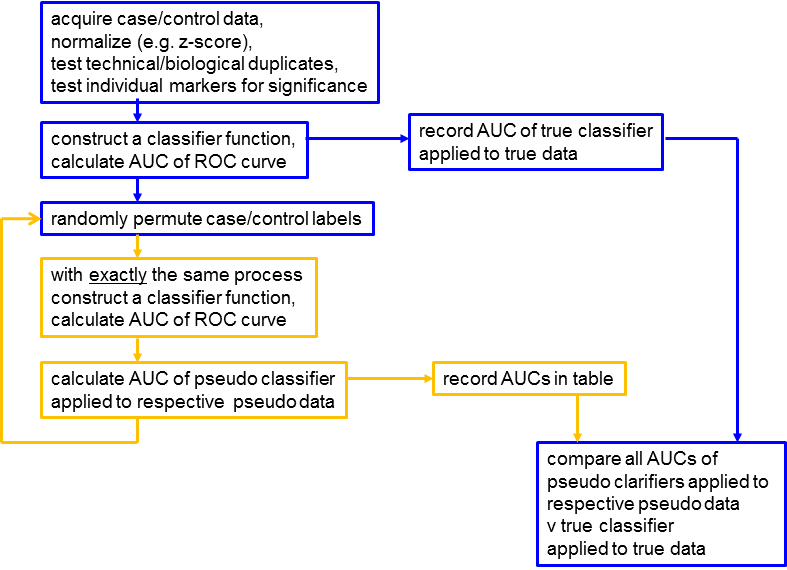


Fig. S3. Randomization testing and random selection flowchart. This method was used to verify that the CALF algorithm actually was capable of finding a function (sum) of miRNA z-scores that differentiated group types. The resulting histogram is shown in Fig. 1. Instead of AUC as figure of merit, other possibilities include the Pearson correlation with a desired, real-valued target vector.

We emphasize that we apply randomization tests to AUC values (arising from data and an algorithm). This is in contrast to conventional application of randomization tests to values of a classifier function, sometimes simply the normalized values of individual markers.

That is, our goal is not identification of single miRNAs as individually informative; rather, we seek to identify *sets or networks* of miRNAs that inform case *v* control. We can do so despite the computational explosion of numbers of possible sets of subsets precisely because: 1) CALF grows small sets deterministically and simply; and 2) randomization tests assure us (or not) that the quality of classification cannot be reasonably explained by chance.

In point of fact, the Student p-values of z-scores for our 136 miRNAs over 37 nonprogressed *v* 30 progressed samples had minimum value 5.2E-3 for miR-941. This p-value does not survive correction for multiple testing.

Theoretical reasoning has been applied by Cole et al. to attempt to answer the question, “How is it that the associations between gene sets and phenotypes can reach high levels of reliability when the associations between individual genes and phenotypes that they are derived from do not?”(13, 14) Cole’s analysis invokes the central limit theorem and concepts of contraction of dimension of search space. Also, Jaffe et al.(15) have discussed finding sets of markers in terms of bagging sets, and Zhang et al.(16) have explored using pairs of markers.

The goal of some classifier methods is minimization of a geometric difference between observed data and modeled predictions (minimal average distance, least angle regression, etc.) By contrast, the goal of CALF is optimization of choice of a function of markers that might not be geometric (e.g. Student p-value). This leads to AUCs that are in some cases better than AUCs of conventional methods.

We note that the same CALF algorithm and randomization tests were applied successfully in two recent NAPLS papers on classifiers that also distinguished nonprogressed from progressed subjects. The two papers were constructed from blood analyte data(17) and clinical symptom data(18).

An R implementation of CALF is freely available at https://cran.r-project.org/web/packages/CALF/index.html . An included example uses data from the supplementary spreadsheet of this paper.

4. Selection of linear weights

This section of the supplement is mathematical background for using discrete weights +1, -1, or 0 instead of the usual real number weights in a linear sum classifier function.

Linear regression uses multiple samples and multiple markers in a matrix. A vector of sample values is proposed as a target of a linear approximation. That is, weights for a linear combination of marker values are sought so that the function, sample by sample, approximates the target vector. Without loss of generality, one might seek weights so that some products (e.g. controls) have low values while others (e.g. cases) have high values. Classification is successful when the same weights used in training data produce a linear combination that is also a good approximation when applied to external test data.

If weights to be used in a linear regression are restricted to +1, -1, or 0, then there are a total of 3^n-1 combinations (excluding the all-0 combination). In n-space, an n-dimensional cube (n-cube) can be described by specifying its vertices as all 2^n combinations of coefficients +1, -1. The 3^n-1 weight vectors correspond to rays from the origin and passing through all the vertices or passing through all central points of all lower-dimensional cubes on the surface of the n-cube. The surface area of the n-cube can be shown to be n*(2^n). Thus, the number of such rays divided by the surface area of the n-cube is a ratio that ~exponentially increases with increasing n; in fact, the ratio increases like (3/2)^n. In 2-dimensional space, the box is an ordinary square and the ratio is 8/8 = 1. In 3-dimensional space, the box is an ordinary cube and the ratio is 26/24. In 5-, 10-, 15-, 20-, and 25-dimensional space, the ratios are ~1.51, ~5.77, ~29.19,~166.26, and ~1010.05. Thus, with increasing dimension, although the surface area of the n-cube grows rapidly, the number of such rays puncturing it grows even more rapidly, and, so to speak, the rays become ever more crowded.

Roughly speaking, the set of all such rays provides a discrete approximation of all the possible “directions” in n-dimensional space that could be perfectly described by the infinite set of real rays originating at the origin. The following simulation illustrates this point.

Suppose we generate two real 25-dimensional vectors A and B with uniformly random components selected from the real interval [-1, +1]. From A and for any fixed i = 1, 2,..., 25, let us form a “coarse” vector A such that the i^th^ component, i = 1, 2,..., 25 of A is:

if A_i_ > 0.33, then A_i_ = 1;

elseif A_i_ < -0.33, then A_i_ = -1;

else A_i_ = 0.

Thus, A is a coarse approximation of A in which the real values of components of A have been rounded to +1, -1, or 0.

Let us divide A, B, and A by their lengths, forming vectors of unit length A_u_, B_u_, and A_u_. Next we can form two dot products: A_u_*B_u_ and A_u_*B_u_. If we do generate random A and B and then the other vectors 1000 times, we get 1000 pairs of such dot products A_u_*B_u_ and A_u_*B_u_ in the range [-1, +1]. How do the pairs of dot products differ? In one simulation, the average absolute value in 1000 trials of the pairwise differences of dot products A_u_*B_u_ and A_u_*B_u_ was 0.054 (sd = 0.041). The correlation of the two 1000-dimensional vectors of 1000 dot products was 0.94.

In summary, the unit vectors A_u_ and (its coarse approximation) A_u_ typically have almost the same dot products with the random unit vectors B_u_, and the approximation improves with increasing dimension.

Conventional linear regression methods of approximations have two problems: the real number weights chosen generally have no straightforward interpretation; and the weights generally change if the test set changes in any way, such as discarding one control sample or one case sample. That is, the weights in conventional linear regression generally are inexplicable and unstable. By contrast, using +1,-1, or 0 as weights could be interpreted to mean "significant positive contribution (+1)," "significant negative contribution ( -1)," or "no significant contribution (0)."

5. Best practices for building and testing classifiers

Given tens of cases, tens of controls, and a few hundreds of markers, what function of a small subset of markers has values that differentiate cases and controls? The value of the classifier function can be appreciated from a randomization test (Fig. 1). When only one marker is available or when several markers are selected as independently distinguishing case from control, the ordinary Student p-value is frequently used without checking by randomization tests. If the numbers of cases and controls are sufficiently large and certain other conditions are met, then this is appropriate, according to Halsey et al(19).

However, our interest is in sets of markers that are collectively informative. As will be shown by examples, in this situation a good metric value with training data does not in itself justify confidence in the analysis because various popular algorithms can find seemingly convincing patterns in nonsense data, that is, apophenia. Required instead are many random permutations of samples into pseudo cases and controls and application of the same classifier construction algorithm to provide performance levels of pseudo classifiers. From a histogram comparing performance of the true classifier with that of many pseudo classifiers, it can be seen that either the true classifier is superior to almost all of the pseudo classifiers or not. If and only if the true classifier is superior is further development of the classifier warranted. Next steps include additional developments of many classifiers from random 80% subsets of (true) cases and controls, hybridization of all such classifiers, and application to an external test set.

Biological and medical journals are replete with a great number of classifiers, but reproducibility of some has been questioned(20), resulting in multiple proposals for remediation (e.g. Ioannidis(21)) that include more stringent methods of data analyses. For example, data analysis practices advocated by Halsey et al.(19) include appropriate use of the Student t-test p-value (as opposed to inappropriate application with low numbers of samples).

Modern randomization tests(1) can be traced to work in 1935 by Fisher(22). Many modern authors have enhanced and expanded the basic ideas, e.g. Suresh(23). A randomization test In this article assumes that an experimental program has produced a rectangular matrix in which rows correspond to samples, many columns contain numerical results of various assays, and a special column designates case or control (or in more general work a column that designates more than two discrete states, real values such as time elapsed until conversion from one state to another, a target vector meant to be approximated in correlation with classifier values, etc.).

There are tens of classifier methods, some invented, tested on a few data sets, published as breakthroughs, but then forgotten because they offer only the “illusion of progress” in classifier theory(24) and are not actually superior to simpler, well-documented methods. Perhaps as important as choices of algorithms are successful marker (feature) selection methods that reduce the risk of overfitting, such as might happen with many thousands of molecular species encountered in cancer research(25, 26). Logical reduction of the dimensionality of marker space is a crucial step, all the more so with thousands of dimensions(26, 27).

A flowchart for randomization tests is shown in Fig. S4.


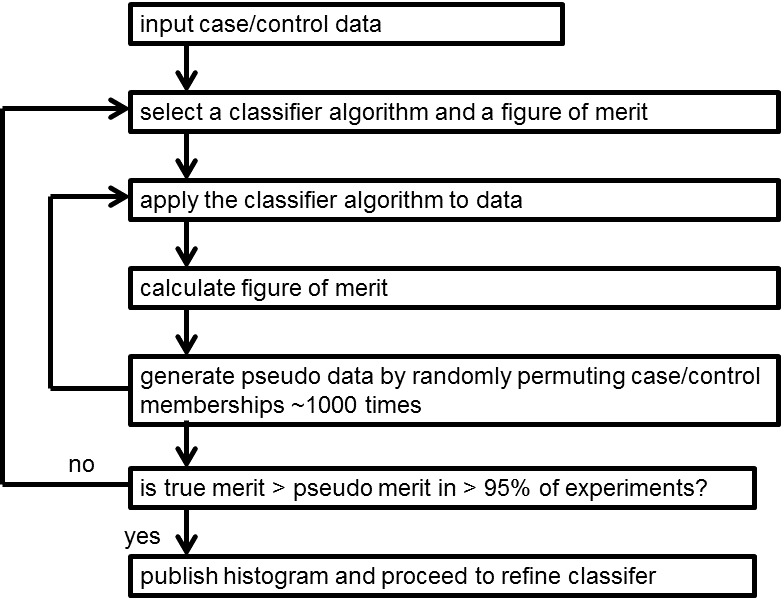


Fig. S4. A high-level flowchart adapted from Edgington(1) including randomization tests of proposed classifiers. If this algorithm terminates successfully, then further development of the classifier may be justified.

The source data used in the present work (Table S3, a spreadsheet) were RNA-seq detections corresponding to microRNAs (miRNAs) from leukocytes from 67 persons at risk of progression to schizophrenia. Within two years of assays, 30 progressed and 37 did not; 136 miRNAs achieved robust assay levels in all 67 samples.

Data on miRNA levels sometimes exhibit correlations or anticorrelations in keeping with supporting or opposing regulatory processes(28) and so may have multiple types and degrees of obvious or subtle interdependencies. This is typical of many kinds of biological data(26) and consequently an active topic of visualization research(29).

Real data with tens of cases, tens of controls, and hundreds of markers often cannot be easily approximated by standard distributions across samples or across markers.

Surprisingly, it can be easier with a simple classifier algorithm to build classifiers with good AUC from random data than from real miRNA data. So to speak, too many of the best markers in real data might be in lockstep, limiting the flexibility of parsimonious functions of combinations of small numbers of chosen markers. Elaboration of this paradox follows.

The output of a successful randomization test of a classifier applied to training data can be a histogram with a fitted distribution somewhat like that in Fig. S5 (modified from Fig. 1).


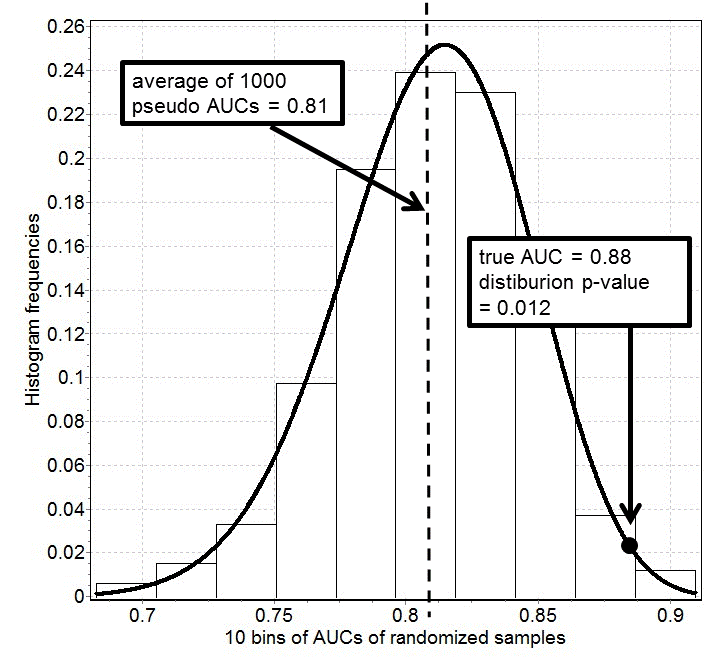


Fig. S5. A histogram of 1001 AUC values. One AUC (indicated) is that of the true classifier generated from the true data. Another 1000 AUCs are represented by histogram bins and are from pseudo classifiers generated by applying exactly the same classifier algorithm to pseudo data. Pseudo data are data in which case/control group sizes are kept but memberships of samples have been randomly reassigned. The shown beta distribution fits the histogram. The chosen classifier construction method (CALF) applied to true data yields an AUC = ~0.88, superior to most AUCs from exactly the same classifier applied to 1000 sets of pseudo data (AUC average = ~0.81, sd = ~0.02).

The beta distribution fit in Fig. S5 was prepared using EasyFit (MathWave, Dnepropetrovsk Ukraine) and itself yields a p-value of 0.012 from the true AUC value. Its shape reveals not only that the true value was higher than most pseudo values but also how much higher it was than the bulk of the pseudo values. However, the p-value of the true AUC can also be estimated from the 17 pseudo classifiers that by chance outperformed the true classifier; the estimated type I error rate(30, 31) is (17+1)/(1000+1) = ~0.018. Importantly, if the chosen six markers are disallowed, then rerunning CALF yields a second selection of six markers with an AUC of 0.83; this drop in AUC is evidence of the superior utility of the first six.

In summary, prudent construction of a classifier can include:

1. a tentative classifier applied to training data achieves seemingly good performance;

2. the same tentative classifier passes randomization tests (Fig. 3);

3. the same classifier applied to many 80% subsets of training data repeatedly selects mainly the same markers (Fig. 6);

4. the intersection of chosen markers in steps 1 and 3 is robust, leading to a hybrid classifier;

5. the hybrid classifier also exhibits strong performance for the full training set;

6. the hybrid classifier exhibits strong performance with an external test set.

6. Medications

There were six samples unsuitable for this study in the sense that total read counts were much lower than read counts of all other samples. Remaining were 94 samples: 27 unaffected controls, 37 nonprogressed subjects, and 30 progressed subjects. The 5-miRNA sum (equation (*1*)) was applied to all nonprogressed *v* progressed samples and achieved an AUC of 0.86. When applied 1000 times to random subsets of 25 nonprogressed and 25 progressed subjects, the average (sd) of the AUCs was 0.86 (0.029).

To investigate the effects of antidepressants, antipsychotics, benzodiazepines, mood stabilizers, stimulants, and use of any of these on classifier performance, we selected random subsets of 25 samples each from the nonprogressed and progressed groups in two ways:

(1) maximized treated subjects: all treated subjects in the group union a random set of untreated subjects sufficient to form a total of 25 subjects; and

(2) minimized treated subjects: all untreated subjects (or a random set of 25 if untreated subjects are sufficiently numerous) in the group union a random set of treated subjects sufficient to form a total of 25 subjects;

The results are shown as averages (sds) in Table S1.

Table S1. Comparison of AUCs that considers medications. The classifier with five miRNAs (*1*) was applied to subsets of 25 samples and the numbers of treated subjects within the random subsets were maximized or minimized. All twelve possible averages were in [0.83, 0.87].


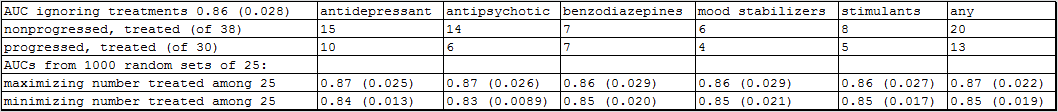


These experiments provided evidence that the performance of the five-miRNA classifier in equation (*1*) was largely independent of inclusion or exclusion of patients treated with the listed types of medications.

7. Smith-Waterman similarity of selected mature miRNA sequences

We also used Ingenuity (QIAGEN, Venlo, Netherlands) to study our selection of informative miRNAs. We noted that only 92 of the 136 robustly expressed miRNAs were in the Ingenuity miRNA targeting database due to similarities of mature sequences (e.g. nine let-7 species in the 136 were represented by hsa-let-7a-5p). There are 5 true miRNAs in (1), so 10 pairs. We calculated the Smith-Waterman sequence alignment score(32) for all 10 pairs using weights match = +1, mismatch = -1, gap = -1. The average of the ten alignment scores was 6.4.

An example of a Smith-Waterman alignment and score is:

miR-199a-3p ACAGUAGUCUGCACAU-UGGUUA

miR-941 CACCCGGCUGUGUGCACAUGUGC

** ******* **

Over a span of 13 bases there are 11 matches, 0 mismatches, and 2 gaps. With the given weights, the Smith-Waterman score is therefore 11-0-2=9.

Then we selected 1000 times a random set of five miRNAs from the 92 in Ingenuity and calculated ten Smith-Waterman scores and the 1000 averages. A total of 19 times of 1000 the random averages were greater than or equal to the true average. Thus, the Monte Carlo estimated p-value that the high sequence similarities are due to chance is 0.020 (31).

Table S2. Summary of five chosen miRNAs in a classifier function and their Smith-Waterman alignment scores.


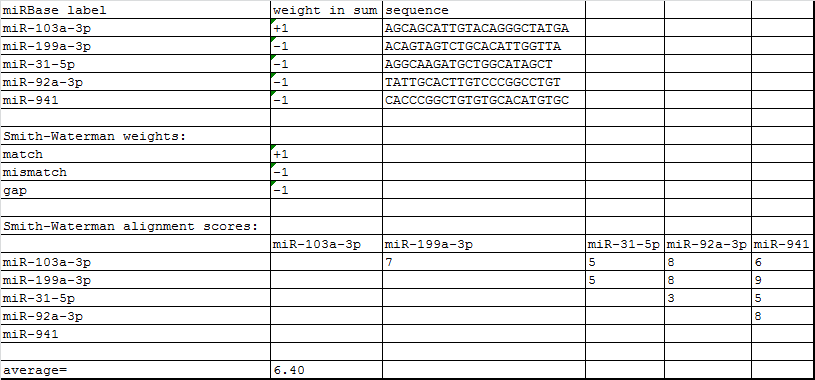


As an alternative to Monte Carlo, the 1000 averages from random selections of five miRNAs can be used in a histogram and approximated with a Log-Logistic distribution with three parameters: α=15.317, β=4.3866, γ=0.82437. The graph appears in Fig. S6.


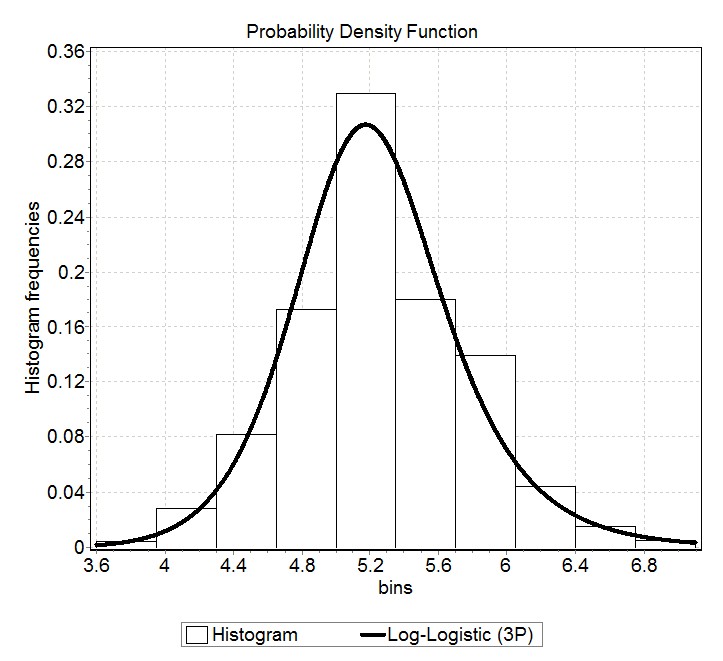


Fig. S6. Comparison of 1000 averages of Smith-Waterman scores with the Smith-Waterman score of the five miRNAs selected to differentiate nonprogressed *v* progressed subjects. The log-logistic distribution implies a p-value for the true score average 6.4 of 0.025. Thus, the high strengths of the various alignments among the five chosen miRNAs are unlikely to be due to chance.

The strongest four pairs of ten possible alignments are listed as follows.

miR-199a-3p ACAGUAGUCUGCACAUUGGUUA

miR-92a-3p UA U UGCAC UUGUCCCGGCCUGU

score = 8 ** * ***** ***

miR-199a-3p ACAGUAGUCUGCACAU UGGUUA

miR-941 CACCCGGCUGUGUGCACAUGUGC

score = 9 ** ******* **

miR-92a-3p UAUUGCACUUGUCCCGGCCUGU

miR-941 CACCCGGC UGUGUGCACAUGUGC

score = 8 ****** ***

miR-103a-3p AGCAGCAUUGUACAGGGCUAUGA

miR-92a-3p UAUUGCA C UUGUCCCGGCCUGU

score = 8 *** * **** * ** **

8. Validation of RNA-seq by PCR

We selected at random 12 samples from unaffected controls. The RNA was diluted to a uniform level and submitted to a core processing facility at UNC. Following conventional reverse transcription, cDNA synthesis, and preamplification steps, samples were assayed with high-throughput real-time qPCR (HT-PCR) using Fluidigm Corporation (San Francisco CA) technology controlled by a BioMark HD system.

Of the 12 samples, results from one had to be discarded because two of the five miRNAs were not detected and the other three were outliers relative to the other 11 (data not shown). In the remaining 11 samples, all miR-92a-3p was by far the most robustly detected, consistent with its high read levels. miR-103a-3p was in 10 assays the second most robust, again consistent with ranks of read values. The other three miRNAs were less consistent in terms of ranks. However, correlations of ranks between PCR and RNA-seq data (two five-vectors of 1,2,3,4,5 for each sample) had an average over 11 samples of 0.61. When 11 sets of random ranks were generated 10 000 times, no set of 11 correlations had an average exceeding 0.60, implying a p-value of consistency of < 0.0001.

This is not to say that PCR assays will necessarily lead to the same choice of five informative miRNAs as the RNA-seq assays. However, in HT-PCR development it would be prudent to start with assays of the top 30 or so miRNAs from RNA-seq work (including the five already used). In a new set of samples we plan to refine our classifier construction with HT-PCR, possibly leading to additional insights about the roles of chosen miRNAs.

References

1. Edgington ES, Onghena P. Randomization tests. 4th ed. Boca Raton, FL: Chapman & Hall/CRC; 2007. 345 p. p.

2. Kozomara A, Griffiths-Jones S. miRBase: annotating high confidence microRNAs using deep sequencing data. Nucleic Acids Res. 2014;42(D1):D68-D73.

3. Smalheiser NR, Lugli G, Thimmapuram J, Cook EH, Larson J. Endogenous siRNAs and noncoding RNA-derived small RNAs are expressed in adult mouse hippocampus and are up-regulated in olfactory discrimination training. Rna. 2011;17(1):166-81.

4. Mortazavi A, Williams BA, McCue K, Schaeffer L, Wold B. Mapping and quantifying mammalian transcriptomes by RNA-Seq. Nature methods. 2008;5(7):621-8.

5. Wang Z, Gerstein M, Snyder M. RNA-Seq: a revolutionary tool for transcriptomics. Nature reviews Genetics. 2009;10(1):57-63.

6. Robinson MD, Oshlack A. A scaling normalization method for differential expression analysis of RNA-seq data. Genome biology. 2010;11(3):R25.

7. Drewe P, Stegle O, Hartmann L, Kahles A, Bohnert R, Wachter A, et al. Accurate detection of differential RNA processing. Nucleic acids research. 2013;41(10):5189-98.

8. Dillies MA, Rau A, Aubert J, Hennequet-Antier C, Jeanmougin M, Servant N, et al. A comprehensive evaluation of normalization methods for Illumina high-throughput RNA sequencing data analysis. Briefings in bioinformatics. 2013;14(6):671-83.

9. Vaz C, Ahmad HM, Bharti R, Pandey P, Kumar L, Kulshreshtha R, et al. Analysis of the microRNA transcriptome and expression of different isomiRs in human peripheral blood mononuclear cells. BMC research notes. 2013;6:390.

10. Zhang W, Gao S, Zhou X, Xia J, Chellappan P, Zhou X, et al. Multiple distinct small RNAs originate from the same microRNA precursors. Genome biology. 2010;11(8):R81.

11. Chaudhry MA, Omaruddin RA, Brumbaugh CD, Tariq MA, Pourmand N. Identification of radiation-induced microRNA transcriptome by next-generation massively parallel sequencing. Journal of radiation research. 2013;54(5):808-22.

12. Schmittgen TD, Lee EJ, Jiang J, Sarkar A, Yang L, Elton TS, et al. Real-time PCR quantification of precursor and mature microRNA. Methods. 2008;44(1):31-8.

13. Fredrickson BL, Grewen KM, Coffey KA, Algoe SB, Firestine AM, Arevalo JM, et al. A functional genomic perspective on human well-being. Proceedings of the National Academy of Sciences of the United States of America. 2013;110(33):13684-9.

14. Cole SW. Elevating the perspective on human stress genomics. Psychoneuroendocrinology. 2010;35(7):955-62.

15. Jaffe AE, Storey JD, Ji H, Leek JT. Gene set bagging for estimating the probability a statistically significant result will replicate. BMC Bioinformatics. 2013;14:360.

16. Zhang W, Zeng T, Chen L. EdgeMarker: Identifying differentially correlated molecule pairs as edge-biomarkers. J Theor Biol. 2014;362:35-43.

17. Perkins DO, Jeffries CD, Addington J, Bearden CE, Cadenhead KS, Cannon TD, et al. Towards a psychosis risk blood diagnostic for persons experiencing high-risk symptoms: preliminary results from the NAPLS project. Schizophr Bull. 2015;41(2):419-28.

18. Perkins DO, Jeffries CD, Cornblatt BA, Woods SW, Addington J, Bearden CE, et al. Severity of thought disorder predicts psychosis in persons at clinical high-risk. Schizophr Res. 2015.

19. Halsey LG, Curran-Everett D, Vowler SL, Drummond GB. The fickle P value generates irreproducible results. Nat Methods. 2015;12(3):179-85.

20. Collins FS, Tabak LA. Policy: NIH plans to enhance reproducibility. Nature. 2014;505(7485):612-3.

21. Ioannidis JPA. How to Make More Published Research True. Plos Med. 2014;11(10).

22. Fisher RA. The Design of Experiments, 9th Edition: Macmillan; 1971.

23. Suresh K. An overview of randomization techniques: An unbiased assessment of outcome in clinical research. Journal of human reproductive sciences. 2011;4(1):8-11.

24. Hand DJ. Classifier technology and the illusion of progress. Stat Sci. 2006;21(1):1-14.

25. Ressom HW, Varghese RS, Zhang Z, Xuan J, Clarke R. Classification algorithms for phenotype prediction in genomics and proteomics. Frontiers in bioscience : a journal and virtual library. 2008;13:691-708.

26. Clarke R, Ressom HW, Wang A, Xuan J, Liu MC, Gehan EA, et al. The properties of high-dimensional data spaces: implications for exploring gene and protein expression data. Nature reviews Cancer. 2008;8(1):37-49.

27. Wang Y, Miller DJ, Clarke R. Approaches to working in high-dimensional data spaces: gene expression microarrays. British journal of cancer. 2008;98(6):1023-8.

28. Smalheiser NR, Lugli G, Rizavi HS, Torvik VI, Turecki G, Dwivedi Y. MicroRNA expression is down-regulated and reorganized in prefrontal cortex of depressed suicide subjects. PloS one. 2012;7(3):e33201.

29. Ray WC, Wolock SL, Callahan NW, Dong M, Li QQ, Liang C, et al. Addressing the unmet need for visualizing conditional random fields in biological data. BMC bioinformatics. 2014;15:202.

30. North BV, Curtis D, Sham PC. A note on the calculation of empirical P values from Monte Carlo procedures. American journal of human genetics. 2002;71(2):439-41.

31. North BV, Curtis D, Sham PC. A note on calculation of empirical P values from Monte Carlo procedure. American journal of human genetics. 2003;72(2):498-9.

32. Smith TF, Waterman MS. Identification of common molecular subsequences. Journal of molecular biology. 1981;147(1):195-7.
