# Supplementary material for: Insights into psychosis risk from leukocyte microRNA expression
Source: Transl Psychiatry. 2016 Dec 13;6(12):e981–. doi: 10.1038/tp.2016.148 (PMC5290334; doi:10.1038/tp.2016.148)
Supplement: Supplementary Table S3 [file tp2016148x1.pdf]

duplicate study results in DB1386 (frozen over all samples)
selection of 25 or more in C563
selection of 98 useful samples starts A563
quotients start in A1105
z-scores start in A1379
consistent sign test in GX1935
All 101 samples X 530 miRNAs 2.44E-04
30 UCs, 39 CHR-NPs, 32 CHR-Ps->29, 38, 31
counts shown are exact matches + inclusions
thresholds = 0.5498, corresponding to 2E-3
each miR ranked by total reads 530 miRs, 101 samples
Macro "rand_labels" randomly relabels all 101 samples.

530 mature miRNAs:	ID
TCCTGTACTGAGCTGCCCCGAG	miR-486-5p
TATTGCACTTGTCCCGGCCTGT	miR-92a-3p
CAACGGAATCCCAAAAGCAGCTG	miR-191-5p
TCTCCCAACCCTTGTACCAGTG	miR-150-5p
TGAGGTAGTAGGTTGTATAGTT	let-7a-5p
AACATTCAACGCTGTCGGTGAGT	miR-181a-5p
TAGCAGCACGTAAATATTGGCG	miR-16-5p
TTCAAGTAATCCAGGATAGGCT	miR-26a-5p
AAACCGTTACCATTACTGAGTT	miR-451a
AAGCTGCCAGTTGAAGAACTGT	miR-22-3p
CATTGCACTTGTCTCGGTCTGA	miR-25-3p
TGAGGTAGTAGTTTGTGCTGTT	let-7i-5p
TGTAAACATCCCCGACTGGAAG	miR-30d-5p
TGAGGTAGTAGTTTGTACAGTT	let-7g-5p
TGAGGTAGTAGGTTGTGTGGTT	let-7b-5p
TGAGGTAGTAGATTGTATAGTT	let-7f-5p
AGCTCGGTCTGAGGCCCCTCAGT	miR-423-3p
TTCAAGTAATTCAGGATAGGT	miR-26b-5p
AGCAGCATTGTACAGGGCTATGA	miR-103a-3p
CAAAGAATTCTCCTTTTGGGCT	miR-186-5p
CACTAGATTGTGAGCTCCTGGA	miR-28-3p
ACTGGACTTGGAGTCAGAAGG	miR-378a-3p
CAAAGTGCTGTTCGTGCAGGTAG	miR-93-5p
TCAGTGCACTACAGAACTTTGT	miR-148a-3p
TGTCAGTTTGTCAAATACCCCA	miR-223-3p
TGAGAACTGAATTCCATAGGCT	miR-146b-5p
TACCACAGGGTAGAACCACGG	miR-140-3p
AAAAGCTGGGTTGAGAGGGCGA	miR-320a
TTTGGCAATGGTAGAACTCACACT	miR-182-5p
TCGAGGAGCTCACAGTCT	miR-151b
TCAGTGCATCACAGAACTTTGT	miR-148b-3p
TGTAAACATCCTACACTCTCAGC	miR-30c-5p
AGAGGTAGTAGGTTGCATAGTT	let-7d-5p
TGTAAACATCCTTGACTGGAAG	miR-30e-5p
TAGCTTATCAGACTGATGTTGA	miR-21-5p
TCGAGGAGCTCACAGTCTAGT	miR-151a-5p
TAGCAGCACATCATGGTTTACA	miR-15b-5p
CTAGACTGAAGCTCCTTGAGG	miR-151a-3p
TGAGAACTGAATTCCATGGGTT	miR-146a-5p
CACCCGGCTGTGTGCACATGTGC	miR-941
TGAGGGGCAGAGAGCGAGACTTT	miR-423-5p
TTCACCACCTTCTCCACCCAGC	miR-197-3p
AACATTCATTGCTGTCGGTGGGT	miR-181b-5p
TCAGGCTCAGTCCCCTCCCGAT	miR-484
CTGACCTATGAATTGACAGCC	miR-192-5p
TCTCACACAGAAATCGCACCCGT	miR-342-3p

AGCTACATCTGGCTACTGGGT	miR-222-3p
TTCACAGTGGCTAAGTTCTGC	miR-27b-3p
TCTCTCGGCTCCTCGCGGCTC	miR-3615
GGATATCATCATATACTGTAAG	miR-144-5p
TGAGATGAAGCACTGTAGCTC	miR-143-3p
TCGGATCCGTCTGAGCTTGGCT	miR-127-3p
ATCACATTGCCAGGGATTTCC	miR-23a-3p
AATGGCGCCACTAGGGTTGTG	miR-652-3p
CTTTCAGTCGGATGTTTACAGC	miR-30e-3p
CAACACCAGTCGATGGGCTGT	miR-21-3p
TACAGTACTGTGATAACTGAA	miR-101-3p
AATGACACGATCACTCCCGTTGA	miR-425-5p
AACATTCAACCTGTCGGTGAGT	miR-181c-5p
TTAATGCTAATCGTGATAGGGGT	miR-155-5p
TCCCTGAGACCCTTTAACCTGTGA	miR-125a-5p
AGCTACATTGTCTGCTGGGTTTC	miR-221-3p
TGAGACCTCTGGGTTCTGAGCT	miR-769-5p
CATTATTACTTTTGGTACGCG	miR-126-5p
CATAAAGTAGAAAGCACTACT	miR-142-5p
GCCCTGACCTGTCCTGTTCTG	miR-4732-3p
CAAAGTGCTTACAGTGCAGGTAG	miR-17-5p
TGTAAACATCCTACACTCAGCT	miR-30b-5p
TAAAGTGCTTATAGTGCAGGTAG	miR-20a-5p
TGGCTCAGTTCAGCAGGAACAG	miR-24-3p
TATGGCACTGGTAGAATTCACT	miR-183-5p
CTATACGACCTGCTGCCTTTCT	let-7d-3p
CGGGGCAGCTCAGTACAGGAT	miR-486-3p
TGAGGTAGTAAGTTGTATTGTT	miR-98-5p
CATGCCTTGAGTGTAGGACCGT	miR-532-5p
TAGCACCATCTGAAATCGGTTA	miR-29a-3p
GAATGTTGCTCGGTGAACCCCT	miR-409-3p
ATGCACCTGGGCAAGGATTCTG	miR-500a-3p
CACCCGTAGAACCGACCTTGCG	miR-99b-5p
TGCGGGGCTAGGGCTAACAGCA	miR-744-5p
ATCACATTGCCAGGGATTACC	miR-23b-3p
AATTGCACGGTATCCATCTGTA	miR-363-3p
ACTCGGCGTGGCGTCGGTCGTG	miR-1307-3p
TATTGCACTCGTCCCGGCCTCC	miR-92b-3p
TTATAAAGCAATGAGACTGATT	miR-340-5p
TTCACAGTGGCTAAGTTCCGC	miR-27a-3p
CCTCTGGGCCCTTCCTCCAG	miR-326
CTGGCCCTCTCTGCCCTTCCGT	miR-328
TCGTACCGTGAGTAATAATGCG	miR-126-3p
TCACAGTGAACCGGTCTCTTT	miR-128
TGAGGTAGGAGGTTGTATAGTT	let-7e-5p
ACCACTGACCGTTGACTGTACC	miR-181a-2-3p
TACAGTATAGATGATGTACT	miR-144-3p
GCTGACTCCTAGTCCAGGGCTC	miR-345-5p
CAGTGCAATGTTAAAAGGGCAT	miR-130a-3p
TAGCAGCACATAATGGTTTGTG	miR-15a-5p
AAGGAGCTCACAGTCTATTGAG	miR-28-5p
TCCGGTTCTCAGGGCTCCACC	miR-671-3p
CCGCACTGTGGGTACTTGCTGC	miR-106b-3p
TGCCCTGTGGACTCAGTTCTGG	miR-146b-3p
TAGTGCAATATTGCTTATAGGGT	miR-454-3p
GCCCCTGGGCCTATCCTAGAA	miR-331-3p
CAGTGCAATGATGAAAGGGCAT	miR-130b-3p
AGGGGTGCTATCTGTGATTGA	miR-342-5p
TACCCTGTAGATCCGAATTTGTG	miR-10a-5p

TGTAACAGCAACTCCATGTGGA	miR-194-5p
TACCCATTGCATATCGGAGTTG	miR-660-5p
ACCCCACTCCTGGTACC	miR-4286
TTATCAGAATCTCCAGGGGTAC	miR-361-5p
TAAAGTGCTGACAGTGCAGAT	miR-106b-5p
TGGAGAGAAAGGCAGTTCCTGA	miR-185-5p
ACCATCGACCGTTGATTGTACC	miR-181a-3p
TCCCCCAGGTGTGATTCTGATTT	miR-361-3p
GTCCAGTTTTCCCAGGAATCCCT	miR-145-5p
CAAAGTGCTCATAGTGCAGGTAG	miR-20b-5p
AGGCAAGATGCTGGCATAGCT	miR-31-5p
TGAGGTAGTAGGTTGTATGGTT	let-7c
TGTAAACATCCTCGACTGGAAG	miR-30a-5p
ACAGTAGTCTGCACATTGGTTA	miR-199a-3p
ACAGTAGTCTGCACATTGGTTA	miR-199b-3p
TATTCATTTATCCCCAGCCTACA	miR-664a-3p
ACTGCCCCAGGTGCTGCTGG	miR-324-3p
TAACAGTCTACAGCCATGGTCG	miR-132-3p
AATATAACACAGATGGCCTGT	miR-410
AAAGTTCTGAGACACTCCGACT	miR-148a-5p
TGTAGTGTTTCCTACTTTATGGA	miR-142-3p
ACTCTTTCCCTGTTGCACTAC	miR-130b-5p
CTGCCCTGGCCCGAGGGACCGA	miR-874
TGAGCGCCTCGACGACAGAGCCG	miR-339-3p
ACTCCAGCCCCACAGCCTCAGC	miR-766-3p
TGGGTCTTTGCGGGCGAGATGA	miR-193a-5p
AATGCACCCGGGCAAGGATTCT	miR-501-3p
CCTGTTCTCCATTACTTGGCTC	miR-26b-3p
CCCAGTGTTCAGACTACCTGTTC	miR-199a-5p
CACGCTCATGCACACACCCACA	miR-574-3p
TCGACCGGACCTCGACCGGCT	miR-1307-5p
AACATTCATTGTTGTCGGTGGGT	miR-181d
TGGGTTTACGTTGGGAGAACT	miR-629-5p
CTGTGCGTGTGACAGCGGCTGA	miR-210
AAGTTCTGTTATACACTCAGGC	miR-148b-5p
ATCGGGAATGTCGTGTCCGCCC	miR-425-3p
TGAGAACCACGTCTGCTCTGAG	miR-589-5p
ACTGCTGAGCTAGCACTTCCCG	miR-93-3p
GTAGAGGAGATGGCGCAGGG	miR-877-5p
TCCGTCTCAGTTACTTTATAGC	miR-340-3p
TGTGCAAATCCATGCAAAACTGA	miR-19b-3p
TTTCCGGCTCGCGTGGGTGTGT	miR-1180
CAAAACGTGAGGCGCTGCTAT	miR-424-3p
ATCAACAGACATTAATTGGGCGC	miR-421
TCTTCTCTGTTTTGGCCATGTG	miR-942
CCTCCCACACCCAAGGCTTGCA	miR-532-3p
CGTGTATTTGACAAGCTGAGTT	miR-223-5p
AAAAGTGCTTACAGTGCAGGTAG	miR-106a-5p
TATACAAGGGCAAGCTCTCTGT	miR-381-3p
TGTCCTCTAGGGCCTGCAGTCT	miR-3909
ACTGTAAACGCTTTCTGATG	miR-3607-3p
TTTTGTGTCTCCCATTCCCCAG	miR-5010-3p
AATCCTTGGAACCTAGGTGTGAGT	miR-362-5p
TCTACAGTGCACGTGTCTCCAGT	miR-139-5p
TCCCTGTCCTCCAGGAGCTCACG	miR-339-5p
GGCGGGTGCGGGGGTGG	miR-3656
CAGTGCAATAGTATTGTCAAAGC	miR-301a-3p
CGTCAACACTTGCTGGTTTCCT	miR-505-3p
TTGGGGAAACGGCCGCTGAGTG	miR-2110

CAGTGGTTTTACCCTATGGTAG	miR-140-5p
TCCAGCATCAGTGATTTTGTTG	miR-338-3p
CCTCCTGCCCTCCTTGCTGT	miR-1976
TAATACTGCCGGGTAATGATGGA	miR-200c-3p
AATGGATTTTTGGAGCAGG	miR-1246
TAGCACCATTTGAAATCGGTTA	miR-29c-3p
CACCTTGCGCTACTCAGGTCTG	miR-3200-3p
AAACTCTACTTGTCCTTCTGAGT	miR-618
TAGGTAGTTTCCTGTTGTTGGG	miR-196b-5p
CTCCGTTTGCCTGTTTCGCTG	miR-1468
TCTAGTAAGAGTGGCAGTCGA	miR-628-3p
CCCAGTGTTTAGACTATCTGTTC	miR-199b-5p
ACGCCCTTCCCCCCCTTCTTCA	miR-1249
TGTGACTGGTTGACCAGAGGGG	miR-134
TCAGTGCATGACAGAACTTGG	miR-152
CTCCTGACTCCAGGTCCTGTGT	miR-378a-5p
GTCATACACGGCTCTCCTCTCT	miR-485-3p
TGTTCCTCTGTCTCCCAGAC	miR-4326
AAAAGTACTTGCGGATTT	miR-548av-5p
TCTTGGAGTAGGTCATTGGGTGG	miR-432-5p
ATTCTAATTTCTCCACGTCTTT	miR-576-5p
CAGGGCTGGCAGTGACATGGGT	miR-4446-3p
AGGCGGAGACTTGGGCAATTG	miR-25-5p
AAAAGTACTTGCGGATTTTGCT	miR-548k
TTGTACATGGTAGGCTTTCATT	miR-493-5p
AGGGGGAAAGTTCTATAGTCC	miR-625-5p
ACCTGGCATACAATGTAGATTT	miR-221-5p
GAGTTCTACAGTCAGAC	miR-3168
ATATAATACAACCTGCTAAGTG	miR-374b-5p
AGCAGCATTGTACAGGGCTATCA	miR-107
CCTCCGTGTTACCTGTCCTCTAG	miR-3605-3p
TTATGGTTTGCCTGGGACTGAG	miR-584-5p
TCCCTGAGACCCTAACTTGTGA	miR-125b-5p
TAACTGGTTGAACAACTGAACC	miR-582-3p
AAGGGCTTCCTCTCTGCAGGAC	miR-3158-3p
CGCATCCCCTAGGGCATTGGTGT	miR-324-5p
ACTGGCCTGGGACTACCGG	miR-3176
GGGGTTCCTGGGGATGGGATTT	miR-23a-5p
CAAGTCACTAGTGGTTCCGTT	miR-224-5p
TGAGTGTGTGTGTGTGAGTGTGT	miR-574-5p
TGAGGAGATCGTCGAGGTTGG	miR-3150b-3p
TAAGGTGCATCTAGTGCAGATAG	miR-18a-5p
GCCTGCTGGGGTGGAACCTGGT	miR-370
AGTGCCTGAGGGAGTAAGAG	miR-550a-3-5p
TCAGAACAAATGCCGGTTCCCAGA	miR-589-3p
TTTGGTCCCCTTCAACCAGCTG	miR-133a
TGTAGAGCAGGGAGCAGGAAGCT	miR-4732-5p
CAGCCCCACAGCCTCAGA	miR-4323
ACTCAAAAGATGGCGGCACTTT	miR-371b-5p
TAGTAGACCGTATAGCGTACG	miR-411-5p
CCTCACCATCCCTTCTGCCTGC	miR-6511a-3p
TCTGGGCAACAAAGTGAGACCT	miR-1285-3p
CTGGGAGAGGGTTGTTTACTCC	miR-30c-1-3p
TCTCTGGGCCTGTGTCTTAGGC	miR-330-5p
AGGGCTTAGCTGCTTGTGAGCA	miR-27a-5p
TGATATGTTTGATATATTAGGT	miR-190a
TCAGCTACTACCTCTATTAGG	miR-5690
AACCCGTAGATCCGAACTTGTG	miR-100-5p
ACTGCCCTAAGTGCTCCTTCTGG	miR-18a-3p

CTGTACAGGCCACTGCCTTGC	let-7g-3p
AATCCTTTGTCCCTGGGTGAGA	miR-501-5p
TGTCTTGCAGGCCGTCATGCA	miR-431-5p
AGGTTGGGATCGGTTGCAATGCT	miR-92a-1-5p
AAAAGCTGGGTTGAGAGGGCAA	miR-320b
GGGAGAAGGGTCGGGGC	miR-4516
TTGCAGCTGCCTGGGAGTGACTTC	miR-1301
AATCCTTGCTACCTGGGT	miR-500b
TTTTTCATTATTGCTCCTGACC	miR-335-3p
CCGGTCCCAGGAGAACCTGCAGA	miR-4746-5p
TTATAATACAACCTGATAAGTG	miR-374a-5p
TTAGGGCCCTGGCTCCATCTCC	miR-1296
ACTGCAGTGAAGGCACTTGTAG	miR-17-3p
AGGGCCCCCCCTCAATCCTGT	miR-296-5p
AACTGGCCCTCAAAGTCCCGCT	miR-193b-3p
TTACAGTTGTTCAACCAGTTACT	miR-582-5p
TGCCTACTGAGCTGAAACACAG	miR-24-2-5p
TGTCTTACTCCCTCAGGCACAT	miR-550a-3p
GAAGTTGTTCGTGGTGGATTCG	miR-382-5p
CTGCGCAAGCTACTGCCTTGCT	let-7i-3p
CGAATCATTATTTGCTGCTCTA	miR-15b-3p
TAATGCCCCTAAAAATCCTTAT	miR-365a-3p
TAATGCCCCTAAAAATCCTTAT	miR-365b-3p
ATCATGATGGGCTCCTCGGTGT	miR-433
CGTGCCACCCTTTTCCCCAG	miR-1227-3p
AATGCACCTGGGCAAGGATTCA	miR-502-3p
AATCGTACAGGGTCATCCACTT	miR-487b
TAATACTGCCTGGTAATGATGA	miR-200b-3p
TCGGGGATCATCATGTCACGAGA	miR-542-5p
TGATATGTTTGATATTGGGTT	miR-190b
AATCTGAGAAGGCGCACAAGGT	miR-3200-5p
CGGGCGTGGTGGTGGGGG	miR-1268a
CATCAGAATTCATGGAGGCTAG	miR-2115-3p
ACCTGGACCCAGCGTAGACAAAG	miR-3690
GACTATAGAACTTTCCCCCTCA	miR-625-3p
TAATCCTTGCTACCTGGGTGAGA	miR-500a-5p
TGTGCAAATCTATGCAAAACTGA	miR-19a-3p
CTGGTTTCACATGGTGGCTTAG	miR-29b-2-5p
AAAACTGCAGTTACTTTTGC	miR-548av-3p
AAAAGCTGGGTTGAGAGGA	miR-320d
TGTGCTTGCTCGTCCCGCCCGCA	miR-636
CCAAAACTGCAGTTACTTTTGC	miR-548o-3p
CAAGCTCGTGTCTGTGGGTCCG	miR-99b-3p
CGGCGGGGACGGCGATTGGTC	miR-1908
CATCCCTTGCATGGTGGAGGG	miR-188-5p
ACGTTGGCTCTGGTGGTG	miR-1306-3p
CTTATCAGATTGTATTGTAATT	miR-374a-3p
GTGGGGGAGAGGCTGTC	miR-1275
CCTCACCACCCCTTCTGCCTGCA	miR-6511b-3p
GCGGGGCTGGGCGCGCG	miR-4508
AAACATTCGCGGTGCACTTCTT	miR-543
AGGACCTTCCCTGAACCAAGGA	miR-659-5p
TGGAAGACTAGTGATTTTGTTGT	miR-7-5p
TTCATTTGCCTCCCAGCCTACA	miR-664b-3p
AAAAGTAATTGCGGTCTTT	miR-548ap-5p
TCTCACTGTAGCCTCGAACCCC	miR-1304-3p
AAAAGCTGGGTTGAGAGGGT	miR-320c
CCTGCAACTTTGCCTGATCAGA	miR-4772-3p
TCACCAGCCCTGTGTTCCCTAG	miR-1226-3p

AGAGGTTGCCCTTGGTGAATTC	miR-377-5p
TGCTGGATCAGTGGTTCGAGTC	miR-1287
TGAAGGTCTACTGTGTGCCAGG	miR-493-3p
AGTTGCCAGGGCTGCCTTTGGT	miR-6501-5p
ATCCCACCACTGCCACCAT	miR-1260b
TGCACGGCACTGGGGACACGT	miR-3177-3p
AAAAGTAATTGCGGTCTTTGGT	miR-548j
CACATTACACGGTCGACCTCT	miR-323a-3p
TTTGGCACTAGCACATTTTTGCT	miR-96-5p
ATGCTGACATATTTACTAGAGG	miR-628-5p
AACCCGTAGATCCGATCTTGTG	miR-99a-5p
CCACCTCCCCTGCAAACGTCCA	miR-1306-5p
TACGTCATCGTTGTCATCGTCA	miR-598
CTAAGAAGTTGACTGAAG	miR-3653
TGGTGGGCCGCAGAACATGTGC	miR-654-5p
ACCCTATCAATATTGTCTCTGC	miR-454-5p
ATGGCCAGAGCTCACACAGAGG	miR-4435
AGGAAGCCCTGGAGGGGCTGGAG	miR-671-5p
AAGGCAGGGCCCCCGCTCCCC	miR-940
TGAAACATACACGGGAAACCTC	miR-494
AGAGGCTGGCCGTGATGAATTC	miR-485-5p
CTGGGAGGTGGATGTTTACTTC	miR-30b-3p
GGAGGAACCTTGGAGCTTCGGC	miR-3928
GTGAGGACTCGGGAGGTGG	miR-1224-5p
AGAGCTTAGCTGATTGGTGAAC	miR-27b-5p
GGGAGCCAGGAAGTATTGATGT	miR-505-5p
GTCCCTCTCCAAATGTGTCTTG	miR-642a-5p
CGGCTCTGGGTCTGTGGGGA	miR-760
AGTGGGGAACCCTTCCATGAGG	miR-491-5p
CAGCAGCACACTGTGGTTTGT	miR-497-5p
AGAGTTGAGTCTGGACGTCCCG	miR-219-1-3p
TCTGTGAGACCAAAGAACTACT	miR-4677-3p
CAGTGCAATGATATTGTCAAAGC	miR-301b
TATGGAAAGACTTTGCCACTCT	miR-3688-3p
TCAAGTGTCATCTGTCCCTAG	miR-6513-3p
TCTCCCTTCCTGCCCTGGCTAG	miR-4685-3p
TAGTGGTCAGAGGGCTTATGA	miR-6718-5p
CTGGGATCTCCGGGGTCTTGGTT	miR-769-3p
AGGTCTGCATTCAAATCCCCAGA	miR-6503-5p
AGCGCGGGCTGAGCGCTGCCAGTC	miR-2277-5p
CTTTCAGTCAGATGTTTGCTGC	miR-30d-3p
ACTGGGCTTGGAGTCAGAAG	miR-378g
CAGGTCGTCTTGCAGGGCTTCT	miR-431-3p
CTCCTGGGGCCCGCACTCTCGC	miR-1343
TTTGTGACCTGGTCCACTAACC	miR-758-3p
AGCCCGCCCCAGCCGAGGTTCT	miR-4707-3p
GTTCCTGCTGAACTGAGCCAG	miR-3074-5p
CGGATGAGCAAAGAAAGTGGTT	miR-1255b-5p
TCACCCTGCATCCCGCACCCAG	miR-3620-3p
GCTGGTTTCATATGGTGGTTTAGA	miR-29b-1-5p
CTCTCACCACTGCCCTCCCACAG	miR-1229-3p
TAGCACCATTTGAAATCAGTGTT	miR-29b-3p
TGCCCTGCCTGTTTTCTCCTTT	miR-3173-5p
ATCCCCAGATACAATGGACAA	miR-2355-5p
CTGCCCTAGTCTAGCTGAAGCT	miR-3157-3p
ACTGGACTTGGAGCCAGAAG	miR-378f
TGAGCCCTGTCCTCCCGCAG	miR-1233-3p
CTTGGCACCTAGCAAGCACTCA	miR-1271-5p
CTGGAGATATGGAAGAGCTGTGT	miR-1270

CTTTCAGTCGGATGTTTGCAGC	miR-30a-3p
GCTGCGCTTGGATTTCGTCCCC	miR-191-3p
ACTGGACTTGGAGGCAGAA	miR-378b
GCAAAGCACACGGCCTGCAGAGA	miR-330-3p
CCACTTGGATCTGAAGGCTGCCC	miR-3614-5p
ACAGGTGAGGTTCTTGGGAGCC	miR-125a-3p
TTGGCCATGGGGCTGCGCGG	miR-3187-3p
TTAATATCGGACAACCATTGT	miR-889
ATCCTTGCTATCTGGGTGCTA	miR-502-5p
TAACAGTCTCCAGTCACGGCC	miR-212-3p
TGACAGCGCCCTGCCTGGCTC	miR-2277-3p
GGGACTAGGATGCAGACCTCC	miR-6503-3p
TTCCCTTTGTCATCCTATGCCT	miR-204-5p
CAGCAGCAATTCATGTTTTGAA	miR-424-5p
GGAGACTGATGAGTTCCCGGGA	miR-873-3p
AGCTTCTTTACAGTGCTGCCTTG	miR-103a-2-5p
ACTGATTTCTTTTGGTGTTCAG	miR-29a-5p
TTCTGCCTCTGTCCAGGTCCTT	miR-5001-3p
AGTTCTTCAGTGGCAAGCTTTA	miR-22-5p
TGACCGATTTCTCCTGGTGTTC	miR-29c-5p
ACTGGACTTGGAGTCAGAAA	miR-378d
AGCTTCCATGACTCCTGATGGA	miR-2115-5p
TTCAACGGGTATTTATTGAGCA	miR-95
TTTGTTCGTTCGGCTCGCGTGA	miR-375
TGGGGAGCTGAGGCTCTGGGGGTG	miR-939-5p
TCAGGTGTGGAAACTGAGGCAG	miR-3934-5p
TGATCAGGCAAAATTGCAGACT	miR-4772-5p
TTTTGCGATGTGTTCCTAATAT	miR-450a-5p
CCAGTGGGGCTGCTGTTATCTG	miR-194-3p
TTTAGAGACGGGGTCTTGCTCT	miR-1303
TGATTGGTACGTCTGTGGGTAG	miR-509-3p
TGTGAGGTTGGCATTGTTGTCT	miR-1294
TGGCAGTGTCTTAGCTGGTTGT	miR-34a-5p
TCATCCTCGTCTCCCTCCCAG	miR-5196-3p
GTGAGTCTCTAAGAAAAGAGGA	miR-627
TGTCACTCGGCTCGGCCCACTAC	miR-668
ACTGGACTTGGAGTCAGGA	miR-378e
GGGGCTGGGCGCGCGCC	miR-4492
TCCTTCTGCTCCGTCCCCCAG	miR-1237-3p
CCAGTCCTGTGCCTGCCGCCT	miR-1910
TGAGGATGGATAGCAAGGAAGCC	miR-3605-5p
AGCTGGTGTTGTGAATCAGGCCG	miR-138-5p
TATGTCTGCTGACCATCACCTT	miR-654-3p
GGTGCAGTGCTGCATCTCTGGT	miR-143-5p
CTGGTACAGGCCTGGGGGACAG	miR-150-3p
GCGACCCACTCTTGGTTTCCA	miR-551a
ACCGTGGCTTTCGATTGTTACT	miR-132-5p
CGGCCCGGGCTGCTGCTGTTCCT	miR-1538
ACTCAAAACCCTTCAGTGACTT	miR-616-5p
AAGGAGCTTACAATCTAGCTGGG	miR-708-5p
TCCTCTTCTCCCTCCTCCCAG	miR-877-3p
ATATACAGGGGGAGACTCTTAT	miR-1185-1-3p
TAGCAGCACAGAAATATTGGC	miR-195-5p
GTTCTCCCAACGTAAGCCCAGC	miR-629-3p
AAAGACATAGGATAGAGTCACCTC	miR-641
TACCCTGTAGAACCGAATTTGTG	miR-10b-5p
ACTGGACTAGGAGTCAGAAGG	miR-378i
CAGCCCTCCTCCCGCACCCAAA	miR-4687-5p
ACCTTCCTCTCCATGGGTCTTT	miR-3667-3p

GAAGAACTGTTGCATTTGCCCT	miR-4511
AGAGTCGGCGACGCCGCCAGC	miR-4785
AGCTCTAGAAAGATTGTTGACC	miR-6502-5p
GCAGGAACTTGTGAGTCTCCT	miR-873-5p
TGGCCCTGACTGAAGACCAGCAGT	miR-1291
TGGAGACGCGGCCCTGTTGGAGT	miR-139-3p
AGGGGCTGGCTTTCCTCTGGTC	miR-185-3p
AGGCAGTGTAGTTAGCTGATTGC	miR-34c-5p
AGCCTGGAAGCTGGAGCCTGCAGT	miR-1254
AACTAGCTCTGTGGATCCTGAC	miR-4661-5p
TCTTTGGTTATCTAGCTGTATGA	miR-9-5p
AAAAACTGAGACTACTTTTGCA	miR-548e
TAATTTTATGTATAAGCTAGT	miR-590-3p
AACTGGCCTACAAAGTCCCAGT	miR-193a-3p
CTGGGCCCGCGGCGGGCGTGGGG	miR-6724-5p
TTTGGGATTGACGCCACATGTCT	miR-6513-5p
ATTGTCCCTCTCCCTTCCCAG	miR-4646-3p
CACAGCAAGTGTAGACAGGCA	miR-3120-3p
TCAAGAGCAATAACGAAAAATGT	miR-335-5p
TGGTAGACTATGGAACGTAGG	miR-379-5p
TAGGTTATCCGTGTTGCCTTCG	miR-154-5p
CAACAAATCACAGTCTGCCATA	miR-7-1-3p
CTATACGGCCTCCTAGCTTTCC	let-7e-3p
AGGAGGAATTGGTGCTGGTCTT	miR-766-5p
AGGGGGCGGGCTCCGGCG	miR-4488
TAGATAAAATATTGGTACCTG	miR-577
GGCCAGCCACCAGGAGGGCTG	miR-3194-5p
AAAAGTATTTGCGGGTTTTGTC	miR-548l
TGCCTAGGCTGAGACTGCAGTG	miR-3135a
CTGTTGCCACTAACCTCAACCT	miR-744-3p
CTATACAATCTACTGTCTTTC	let-7a-3p
TCAGCACCAGGATATTGTTGGAG	miR-3065-3p
CTGACTGTTGCCGTCCTCCAG	miR-943
AGGGGGATGGCAGAGCAAAATT	miR-5010-5p
CCAATATTACTGTGCTGCTTTA	miR-16-2-3p
ATCAGGGCTTGTGGAATGGGAAG	miR-3127-5p
ATCCCACCTCTGCCACCA	miR-1260a
TGAGCTAAATGTGTGCTGGGA	miR-610
AACCATCGACCGTTGAGTGGAC	miR-181c-3p
GGAGAAATTATCCTTGGTGTGT	miR-539-5p
CATCATCGTCTCAAATGAGTCT	miR-136-3p
ATTGTCCTTGCTGTTTGGAGAT	miR-2355-3p
TGACCTGGGACTCGGACAGCTG	miR-3661
GCAGCCCAGCTGAGGCCTCTG	miR-4690-3p
CCAACCTAGGTGGTCAGAGTTG	miR-4714-3p
AATCATACAGGGACATCCAGTT	miR-487a
TTAGCCAATTGTCCATCTTTAG	miR-4662a-5p
TGCCGCCCTCTCGCTGCTCTAG	miR-4632-3p
CGCCCCTCCTGCCCCCACAG	miR-4749-3p
ACCTTGGCTCTAGACTGCTTACT	miR-212-5p
TACGCGCAGACCACAGGATGTC	miR-3939
AAAAGTTATTGCGGTTTTGGCT	miR-548at-5p
AGGGACGGGACGCGGTGCAGTG	miR-92b-5p
AGACAGTAGTTCTTGCCTGGTT	miR-4645-3p
ACCTCCTGTGTGCATGGATTA	miR-660-3p
TAGGATTACAAGTGTCGGCCAC	miR-3159
TTCAGCCAGGCTAGTGCAGTCT	miR-3157-5p
TGGGCAGGGGCTTATTGTAGGAG	miR-6721-5p
CAGCCCGGATCCCAGCCCACTT	miR-3940-3p

GGGCTCACATCACCCCAT	miR-4284
TGTCGTGGGGCTTGCTGGCTTG	miR-4440
CATCCGTCCGTCTGTCCAC	miR-4800-3p
TTTCCCTTTCCATCCTGGCAG	miR-5006-3p
AGTGCCTGAGGGAGTAAGAGCCC	miR-550a-5p
AAAAGTAATTGTGGTTTTTGC	miR-548ay-5p
TTGGAGGCGTGGGTTTT	miR-4443
CAGTTATCACAGTGCTGATGCT	miR-101-5p
CCAGGCTCTGCAGTGGGA	miR-3155b
AAACAAACATGGTGCACTTCTT	miR-495-3p
ATGGCATCGTCCCCTGGTGGCT	miR-4642
TCAGTTCCAGGCCAACCAGGCT	miR-584-3p
AAAAGTAATTGCGGTTTTTGC	miR-548au-5p
GGGTGCGGGCCGGCGGGG	miR-4466
TTACACAGCTGGACAGAGGCA	miR-4672
CTGCCAATTCCATAGGTCACAG	miR-192-3p
AAGTGCCCCCACAGTTTGAGTGC	miR-371b-3p
AGGTTACCCGAGCAACTTTGCAT	miR-409-5p
TTAATTTTTTGTTTCGGTCACT	miR-4775
TACCCAGTCTCCGGTGCAGCC	miR-3130-5p
GCTAAGGAAGTCCTGTGCTCAG	miR-4521
TAACGCATAATATGGACATGT	miR-3912
CTTAGCAGGTTGTATTATCATT	miR-374b-3p
CCAGAGCAGCCTGCGGTAACAGT	miR-6501-3p
CGGGTAGAGAGGGCAGTGGGAGG	miR-197-5p
TCTATACAGACCCTGGCTTTTC	miR-1284
AGAGCTGGCTGAAGGGCAG	miR-4487
TCGGCCTGACCACCCACCCCAC	miR-1234-3p
TGTGACAGATTGATAACTGAAA	miR-542-3p
TAAGGTGCATCTAGTGCAGTTAG	miR-18b-5p
TGAGTATTACATGGCCAATCTC	miR-496
GAGCCAGTGGTGAGACAGTGA	miR-4676-5p
TTTCTTCTTAGACATGGCAACG	miR-4659a-3p
AGACCCTGGTCTGCACTCTATC	miR-504
ACGGTGCTGGATGTGGCCTTT	miR-1250
TCGTGTCTTGTGTTGCAGCCGG	miR-187-3p
TGAGGCGGGGGGGCGAGC	miR-6087
TGAGGCTCTGTTAGCCTTGGCTC	miR-2467-5p
GATATCAGCTCAGTAGGCACCG	miR-3074-3p
CCAGTTACCGCTTCCGCTACCGC	miR-935
TTTGGGACTGATCTTGATGTCT	miR-3913-5p
CTCGGGCGGAGGTGGTTGAGTG	miR-4750-5p
CCTCCCATGCCAAGAACTCCC	miR-2116-3p
TAGGGGCAGCAGAGGACCTGGG	miR-4688
CCAGGCTCTGCAGTGGGAACT	miR-3155a
TATAAAATGAGGGCAGTAAGAC	miR-3163
TGAGGATATGGCAGGGAAGGGGA	miR-3679-5p
TGGACGGAGAACTGATAAGGGT	miR-184
AAAGCTGGGTTGAGAAGG	miR-320e
CTTCCTCGTCTGTCTGCCCC	miR-1238-3p
TCTGGCTCCGTGTCTTCACTCCC	miR-149-5p
AGGATGAGCAAAGAAAGTAGATT	miR-1255a
ATAGGACTCATATAGTGCCAG	miR-3117-3p
TAGGTAGTTTCATGTTGTTGGG	miR-196a-5p
CACTGTGGGTACATGCT	miR-4318
TTTGAGGCTACAGTGAGATGTG	miR-1304-5p
CTAGGTATGGTCCCAGGGATCC	miR-331-5p
AGGGACTTTCAGGGGCAGCTGT	miR-365b-5p
AGGAGATCCTGGGTT	miR-3676-5p

TCAACAAAATCACTGATGCTGGA	miR-3065-5p
TCTCTTCATCTACCCCCCAG	miR-6515-3p
TAGTGAGTTAGAGATGCAGAGCC	miR-3174
CTCGTGGGCTCTGGCCACGGCC	miR-3677-3p
CAAGCTCGCTTCTATGGGTCTG	miR-99a-3p
TTATTGTCACGTTCTGATT	miR-5701
ATCCGCGCTCTGACTCTCTGCC	miR-937-3p
CGTCCCACCCCCCACTCCTGT	miR-4433-5p
CCCCGGGGAGCCCGGCG	miR-4532
TTTCTATTTCTCAGTGGGGCTC	miR-4482-3p
TAAAGAGCCCTGTGGAGACA	miR-1276
TACTTTTCTAGGTTGTTGGGG	miR-5003-3p
TCCTGTACTGAGCTGCCCCGAG	miR-486-5p

min of sums of selected 95:	
21319	sample size limit
ave of sums of selected 95:	sum =
1469347	529 miRs
sd of sums of selected 95:	XX samples
1235774	
counts shown are exact matches + inclusions	
each miR ranked by total reads	
530 mature miRNAs:	ID
TATTGCACTTGTCCCGGCCTGT	miR-92a-3p
CAACGGAATCCCAAAAGCAGCTG	miR-191-5p
TCTCCCAACCCTTGTACCAGTG	miR-150-5p
TGAGGTAGTAGGTTGTATAGTT	let-7a-5p
AACATTCAACGCTGTCGGTGAGT	miR-181a-5p
TAGCAGCACGTAAATATTGGCG	miR-16-5p
TTCAAGTAATCCAGGATAGGCT	miR-26a-5p
AAACCGTTACCATTACTGAGTT	miR-451a
AAGCTGCCAGTTGAAGAACTGT	miR-22-3p
CATTGCACTTGTCTCGGTCTGA	miR-25-3p
TGAGGTAGTAGTTTGTGCTGTT	let-7i-5p
TGTAAACATCCCCGACTGGAAG	miR-30d-5p
TGAGGTAGTAGTTTGTACAGTT	let-7g-5p
TGAGGTAGTAGGTTGTGTGGTT	let-7b-5p
TGAGGTAGTAGATTGTATAGTT	let-7f-5p
AGCTCGGTCTGAGGCCCCTCAGT	miR-423-3p
TTCAAGTAATTCAGGATAGGT	miR-26b-5p
AGCAGCATTGTACAGGGCTATGA	miR-103a-3p
CAAAGAATTCTCCTTTTGGGCT	miR-186-5p

CACTAGATTGTGAGCTCCTGGA	miR-28-3p
ACTGGACTTGGAGTCAGAAGG	miR-378a-3p
CAAAGTGCTGTTCGTGCAGGTAG	miR-93-5p
TCAGTGCACTACAGAACTTTGT	miR-148a-3p
TGTCAGTTTGTCAAATACCCCA	miR-223-3p
TGAGAACTGAATTCCATAGGCT	miR-146b-5p
TACCACAGGGTAGAACCACGG	miR-140-3p
AAAAGCTGGGTTGAGAGGGCGA	miR-320a
TTTGGCAATGGTAGAACTCACACT	miR-182-5p
TCGAGGAGCTCACAGTCT	miR-151b
TCAGTGCATCACAGAACTTTGT	miR-148b-3p
TGTAAACATCCTACACTCTCAGC	miR-30c-5p
AGAGGTAGTAGGTTGCATAGTT	let-7d-5p
TGTAAACATCCTTGACTGGAAG	miR-30e-5p
TAGCTTATCAGACTGATGTTGA	miR-21-5p
TCGAGGAGCTCACAGTCTAGT	miR-151a-5p
TAGCAGCACATCATGGTTTACA	miR-15b-5p
CTAGACTGAAGCTCCTTGAGG	miR-151a-3p
TGAGAACTGAATTCCATGGGTT	miR-146a-5p
CACCCGGCTGTGTGCACATGTGC	miR-941
TGAGGGGCAGAGAGCGAGACTTT	miR-423-5p
TTCACCACCTTCTCCACCCAGC	miR-197-3p
AACATTCATTGCTGTCGGTGGGT	miR-181b-5p
TCAGGCTCAGTCCCCTCCCGAT	miR-484
CTGACCTATGAATTGACAGCC	miR-192-5p
TCTCACACAGAAATCGCACCCGT	miR-342-3p
AGCTACATCTGGCTACTGGGT	miR-222-3p
TTCACAGTGGCTAAGTTCTGC	miR-27b-3p
TCTCTCGGCTCCTCGCGGCTC	miR-3615
GGATATCATCATATACTGTAAG	miR-144-5p
TGAGATGAAGCACTGTAGCTC	miR-143-3p
TCGGATCCGTCTGAGCTTGGCT	miR-127-3p
ATCACATTGCCAGGGATTTCC	miR-23a-3p
AATGGCGCCACTAGGGTTGTG	miR-652-3p
CTTTCAGTCGGATGTTTACAGC	miR-30e-3p
CAACACCAGTCGATGGGCTGT	miR-21-3p
TACAGTACTGTGATAACTGAA	miR-101-3p
AATGACACGATCACTCCCGTTGA	miR-425-5p
AACATTCAACCTGTCGGTGAGT	miR-181c-5p
TTAATGCTAATCGTGATAGGGGT	miR-155-5p
TCCCTGAGACCCTTTAACCTGTGA	miR-125a-5p
AGCTACATTGTCTGCTGGGTTTC	miR-221-3p
TGAGACCTCTGGGTTCTGAGCT	miR-769-5p
CATTATTACTTTTGGTACGCG	miR-126-5p
CATAAAGTAGAAAGCACTACT	miR-142-5p
GCCCTGACCTGTCCTGTTCTG	miR-4732-3p
CAAAGTGCTTACAGTGCAGGTAG	miR-17-5p
TGTAAACATCCTACACTCAGCT	miR-30b-5p
TAAAGTGCTTATAGTGCAGGTAG	miR-20a-5p
TGGCTCAGTTCAGCAGGAACAG	miR-24-3p
TATGGCACTGGTAGAATTCACT	miR-183-5p
CTATACGACCTGCTGCCTTTCT	let-7d-3p
CGGGGCAGCTCAGTACAGGAT	miR-486-3p
TGAGGTAGTAAGTTGTATTGTT	miR-98-5p
CATGCCTTGAGTGTAGGACCGT	miR-532-5p
TAGCACCATCTGAAATCGGTTA	miR-29a-3p
GAATGTTGCTCGGTGAACCCCT	miR-409-3p
ATGCACCTGGGCAAGGATTCTG	miR-500a-3p
CACCCGTAGAACCGACCTTGCG	miR-99b-5p

TGCGGGGCTAGGGCTAACAGCA	miR-744-5p
ATCACATTGCCAGGGATTACC	miR-23b-3p
AATTGCACGGTATCCATCTGTA	miR-363-3p
ACTCGGCGTGGCGTCGGTCGTG	miR-1307-3p
TATTGCACTCGTCCCGGCCTCC	miR-92b-3p
TTATAAAGCAATGAGACTGATT	miR-340-5p
TTCACAGTGGCTAAGTTCCGC	miR-27a-3p
CCTCTGGGCCCTTCCTCCAG	miR-326
CTGGCCCTCTCTGCCCTTCCGT	miR-328
TCGTACCGTGAGTAATAATGCG	miR-126-3p
TCACAGTGAACCGGTCTCTTT	miR-128
TGAGGTAGGAGGTTGTATAGTT	let-7e-5p
ACCACTGACCGTTGACTGTACC	miR-181a-2-3p
TACAGTATAGATGATGTACT	miR-144-3p
GCTGACTCCTAGTCCAGGGCTC	miR-345-5p
CAGTGCAATGTTAAAAGGGCAT	miR-130a-3p
TAGCAGCACATAATGGTTTGTG	miR-15a-5p
AAGGAGCTCACAGTCTATTGAG	miR-28-5p
TCCGGTTCTCAGGGCTCCACC	miR-671-3p
CCGCACTGTGGGTACTTGCTGC	miR-106b-3p
TGCCCTGTGGACTCAGTTCTGG	miR-146b-3p
TAGTGCAATATTGCTTATAGGGT	miR-454-3p
GCCCCTGGGCCTATCCTAGAA	miR-331-3p
CAGTGCAATGATGAAAGGGCAT	miR-130b-3p
AGGGGTGCTATCTGTGATTGA	miR-342-5p
TACCCTGTAGATCCGAATTTGTG	miR-10a-5p
TGTAACAGCAACTCCATGTGGA	miR-194-5p
TACCCATTGCATATCGGAGTTG	miR-660-5p
ACCCCACTCCTGGTACC	miR-4286
TTATCAGAATCTCCAGGGGTAC	miR-361-5p
TAAAGTGCTGACAGTGCAGAT	miR-106b-5p
TGGAGAGAAAGGCAGTTCCTGA	miR-185-5p
ACCATCGACCGTTGATTGTACC	miR-181a-3p
TCCCCCAGGTGTGATTCTGATTT	miR-361-3p
GTCCAGTTTTCCCAGGAATCCCT	miR-145-5p
CAAAGTGCTCATAGTGCAGGTAG	miR-20b-5p
AGGCAAGATGCTGGCATAGCT	miR-31-5p
TGAGGTAGTAGGTTGTATGGTT	let-7c
TGTAAACATCCTCGACTGGAAG	miR-30a-5p
ACAGTAGTCTGCACATTGGTTA	miR-199a-3p
ACAGTAGTCTGCACATTGGTTA	miR-199b-3p
TATTCATTTATCCCCAGCCTACA	miR-664a-3p
ACTGCCCCAGGTGCTGCTGG	miR-324-3p
TAACAGTCTACAGCCATGGTCG	miR-132-3p
AATATAACACAGATGGCCTGT	miR-410
AAAGTTCTGAGACACTCCGACT	miR-148a-5p
TGTAGTGTTTCCTACTTTATGGA	miR-142-3p
ACTCTTTCCCTGTTGCACTAC	miR-130b-5p
CTGCCCTGGCCCGAGGGACCGA	miR-874
TGAGCGCCTCGACGACAGAGCCG	miR-339-3p
ACTCCAGCCCCACAGCCTCAGC	miR-766-3p
TGGGTCTTTGCGGGCGAGATGA	miR-193a-5p
AATGCACCCGGGCAAGGATTCT	miR-501-3p
CCTGTTCTCCATTACTTGGCTC	miR-26b-3p
CCCAGTGTTCAGACTACCTGTTC	miR-199a-5p
CACGCTCATGCACACACCCACA	miR-574-3p
TCGACCGGACCTCGACCGGCT	miR-1307-5p
AACATTCATTGTTGTCGGTGGGT	miR-181d
TGGGTTTACGTTGGGAGAACT	miR-629-5p

CTGTGCGTGTGACAGCGGCTGA	miR-210
AAGTTCTGTTATACACTCAGGC	miR-148b-5p
ATCGGGAATGTCGTGTCCGCCC	miR-425-3p
TGAGAACCACGTCTGCTCTGAG	miR-589-5p
ACTGCTGAGCTAGCACTTCCCG	miR-93-3p
GTAGAGGAGATGGCGCAGGG	miR-877-5p
TCCGTCTCAGTTACTTTATAGC	miR-340-3p
TGTGCAAATCCATGCAAAACTGA	miR-19b-3p
TTTCCGGCTCGCGTGGGTGTGT	miR-1180
CAAAACGTGAGGCGCTGCTAT	miR-424-3p
ATCAACAGACATTAATTGGGCGC	miR-421
TCTTCTCTGTTTTGGCCATGTG	miR-942
CCTCCCACACCCAAGGCTTGCA	miR-532-3p
CGTGTATTTGACAAGCTGAGTT	miR-223-5p
AAAAGTGCTTACAGTGCAGGTAG	miR-106a-5p
TATACAAGGGCAAGCTCTCTGT	miR-381-3p
TGTCCTCTAGGGCCTGCAGTCT	miR-3909
ACTGTAAACGCTTTCTGATG	miR-3607-3p
TTTTGTGTCTCCCATTCCCCAG	miR-5010-3p
AATCCTTGGAACCTAGGTGTGAGT	miR-362-5p
TCTACAGTGCACGTGTCTCCAGT	miR-139-5p
TCCCTGTCCTCCAGGAGCTCACG	miR-339-5p
GGCGGGTGCGGGGGTGG	miR-3656
CAGTGCAATAGTATTGTCAAAGC	miR-301a-3p
CGTCAACACTTGCTGGTTTCCT	miR-505-3p
TTGGGGAAACGGCCGCTGAGTG	miR-2110
CAGTGGTTTTACCCTATGGTAG	miR-140-5p
TCCAGCATCAGTGATTTTGTTG	miR-338-3p
CCTCCTGCCCTCCTTGCTGT	miR-1976
TAATACTGCCGGGTAATGATGGA	miR-200c-3p
AATGGATTTTTGGAGCAGG	miR-1246
TAGCACCATTTGAAATCGGTTA	miR-29c-3p
CACCTTGCGCTACTCAGGTCTG	miR-3200-3p
AAACTCTACTTGTCCTTCTGAGT	miR-618
TAGGTAGTTTCCTGTTGTTGGG	miR-196b-5p
CTCCGTTTGCCTGTTTCGCTG	miR-1468
TCTAGTAAGAGTGGCAGTCGA	miR-628-3p
CCCAGTGTTTAGACTATCTGTTC	miR-199b-5p
ACGCCCTTCCCCCCCTTCTTCA	miR-1249
TGTGACTGGTTGACCAGAGGGG	miR-134
TCAGTGCATGACAGAACTTGG	miR-152
CTCCTGACTCCAGGTCCTGTGT	miR-378a-5p
GTCATACACGGCTCTCCTCTCT	miR-485-3p
TGTTCCTCTGTCTCCCAGAC	miR-4326
AAAAGTACTTGCGGATTT	miR-548av-5p
TCTTGGAGTAGGTCATTGGGTGG	miR-432-5p
ATTCTAATTTCTCCACGTCTTT	miR-576-5p
CAGGGCTGGCAGTGACATGGGT	miR-4446-3p
AGGCGGAGACTTGGGCAATTG	miR-25-5p
AAAAGTACTTGCGGATTTTGCT	miR-548k
TTGTACATGGTAGGCTTTCATT	miR-493-5p
AGGGGGAAAGTTCTATAGTCC	miR-625-5p
ACCTGGCATACAATGTAGATTT	miR-221-5p
GAGTTCTACAGTCAGAC	miR-3168
ATATAATACAACCTGCTAAGTG	miR-374b-5p
AGCAGCATTGTACAGGGCTATCA	miR-107
CCTCCGTGTTACCTGTCCTCTAG	miR-3605-3p
TTATGGTTTGCCTGGGACTGAG	miR-584-5p
TCCCTGAGACCCTAACTTGTGA	miR-125b-5p

TAACTGGTTGAACAACTGAACC	miR-582-3p
AAGGGCTTCCTCTCTGCAGGAC	miR-3158-3p
CGCATCCCCTAGGGCATTGGTGT	miR-324-5p
ACTGGCCTGGGACTACCGG	miR-3176
GGGGTTCCTGGGGATGGGATTT	miR-23a-5p
CAAGTCACTAGTGGTTCCGTT	miR-224-5p
TGAGTGTGTGTGTGTGAGTGTGT	miR-574-5p
TGAGGAGATCGTCGAGGTTGG	miR-3150b-3p
TAAGGTGCATCTAGTGCAGATAG	miR-18a-5p
GCCTGCTGGGGTGGAACCTGGT	miR-370
AGTGCCTGAGGGAGTAAGAG	miR-550a-3-5p
TCAGAACAAATGCCGGTTCCCAGA	miR-589-3p
TTTGGTCCCCTTCAACCAGCTG	miR-133a
TGTAGAGCAGGGAGCAGGAAGCT	miR-4732-5p
CAGCCCCACAGCCTCAGA	miR-4323
ACTCAAAAGATGGCGGCACTTT	miR-371b-5p
TAGTAGACCGTATAGCGTACG	miR-411-5p
CCTCACCATCCCTTCTGCCTGC	miR-6511a-3p
TCTGGGCAACAAAGTGAGACCT	miR-1285-3p
CTGGGAGAGGGTTGTTTACTCC	miR-30c-1-3p
TCTCTGGGCCTGTGTCTTAGGC	miR-330-5p
AGGGCTTAGCTGCTTGTGAGCA	miR-27a-5p
TGATATGTTTGATATATTAGGT	miR-190a
TCAGCTACTACCTCTATTAGG	miR-5690
AACCCGTAGATCCGAACTTGTG	miR-100-5p
ACTGCCCTAAGTGCTCCTTCTGG	miR-18a-3p
CTGTACAGGCCACTGCCTTGC	let-7g-3p
AATCCTTTGTCCCTGGGTGAGA	miR-501-5p
TGTCTTGCAGGCCGTCATGCA	miR-431-5p
AGGTTGGGATCGGTTGCAATGCT	miR-92a-1-5p
AAAAGCTGGGTTGAGAGGGCAA	miR-320b
GGGAGAAGGGTCGGGGC	miR-4516
TTGCAGCTGCCTGGGAGTGACTTC	miR-1301
AATCCTTGCTACCTGGGT	miR-500b
TTTTTCATTATTGCTCCTGACC	miR-335-3p
CCGGTCCCAGGAGAACCTGCAGA	miR-4746-5p
TTATAATACAACCTGATAAGTG	miR-374a-5p
TTAGGGCCCTGGCTCCATCTCC	miR-1296
ACTGCAGTGAAGGCACTTGTAG	miR-17-3p
AGGGCCCCCCCTCAATCCTGT	miR-296-5p
AACTGGCCCTCAAAGTCCCGCT	miR-193b-3p
TTACAGTTGTTCAACCAGTTACT	miR-582-5p
TGCCTACTGAGCTGAAACACAG	miR-24-2-5p
TGTCTTACTCCCTCAGGCACAT	miR-550a-3p
GAAGTTGTTCGTGGTGGATTCG	miR-382-5p
CTGCGCAAGCTACTGCCTTGCT	let-7i-3p
CGAATCATTATTTGCTGCTCTA	miR-15b-3p
TAATGCCCCTAAAAATCCTTAT	miR-365a-3p
TAATGCCCCTAAAAATCCTTAT	miR-365b-3p
ATCATGATGGGCTCCTCGGTGT	miR-433
CGTGCCACCCTTTTCCCCAG	miR-1227-3p
AATGCACCTGGGCAAGGATTCA	miR-502-3p
AATCGTACAGGGTCATCCACTT	miR-487b
TAATACTGCCTGGTAATGATGA	miR-200b-3p
TCGGGGATCATCATGTCACGAGA	miR-542-5p
TGATATGTTTGATATTGGGTT	miR-190b
AATCTGAGAAGGCGCACAAGGT	miR-3200-5p
CGGGCGTGGTGGTGGGGG	miR-1268a
CATCAGAATTCATGGAGGCTAG	miR-2115-3p

ACCTGGACCCAGCGTAGACAAAG	miR-3690
GACTATAGAACTTTCCCCCTCA	miR-625-3p
TAATCCTTGCTACCTGGGTGAGA	miR-500a-5p
TGTGCAAATCTATGCAAAACTGA	miR-19a-3p
CTGGTTTCACATGGTGGCTTAG	miR-29b-2-5p
AAAACTGCAGTTACTTTTGC	miR-548av-3p
AAAAGCTGGGTTGAGAGGA	miR-320d
TGTGCTTGCTCGTCCCGCCCGCA	miR-636
CCAAAACTGCAGTTACTTTTGC	miR-548o-3p
CAAGCTCGTGTCTGTGGGTCCG	miR-99b-3p
CGGCGGGGACGGCGATTGGTC	miR-1908
CATCCCTTGCATGGTGGAGGG	miR-188-5p
ACGTTGGCTCTGGTGGTG	miR-1306-3p
CTTATCAGATTGTATTGTAATT	miR-374a-3p
GTGGGGGAGAGGCTGTC	miR-1275
CCTCACCACCCCTTCTGCCTGCA	miR-6511b-3p
GCGGGGCTGGGCGCGCG	miR-4508
AAACATTCGCGGTGCACTTCTT	miR-543
AGGACCTTCCCTGAACCAAGGA	miR-659-5p
TGGAAGACTAGTGATTTTGTTGT	miR-7-5p
TTCATTTGCCTCCCAGCCTACA	miR-664b-3p
AAAAGTAATTGCGGTCTTT	miR-548ap-5p
TCTCACTGTAGCCTCGAACCCC	miR-1304-3p
AAAAGCTGGGTTGAGAGGGT	miR-320c
CCTGCAACTTTGCCTGATCAGA	miR-4772-3p
TCACCAGCCCTGTGTTCCCTAG	miR-1226-3p
AGAGGTTGCCCTTGGTGAATTC	miR-377-5p
TGCTGGATCAGTGGTTCGAGTC	miR-1287
TGAAGGTCTACTGTGTGCCAGG	miR-493-3p
AGTTGCCAGGGCTGCCTTTGGT	miR-6501-5p
ATCCCACCACTGCCACCAT	miR-1260b
TGCACGGCACTGGGGACACGT	miR-3177-3p
AAAAGTAATTGCGGTCTTTGGT	miR-548j
CACATTACACGGTCGACCTCT	miR-323a-3p
TTTGGCACTAGCACATTTTTGCT	miR-96-5p
ATGCTGACATATTTACTAGAGG	miR-628-5p
AACCCGTAGATCCGATCTTGTG	miR-99a-5p
CCACCTCCCCTGCAAACGTCCA	miR-1306-5p
TACGTCATCGTTGTCATCGTCA	miR-598
CTAAGAAGTTGACTGAAG	miR-3653
TGGTGGGCCGCAGAACATGTGC	miR-654-5p
ACCCTATCAATATTGTCTCTGC	miR-454-5p
ATGGCCAGAGCTCACACAGAGG	miR-4435
AGGAAGCCCTGGAGGGGCTGGAG	miR-671-5p
AAGGCAGGGCCCCCGCTCCCC	miR-940
TGAAACATACACGGGAAACCTC	miR-494
AGAGGCTGGCCGTGATGAATTC	miR-485-5p
CTGGGAGGTGGATGTTTACTTC	miR-30b-3p
GGAGGAACCTTGGAGCTTCGGC	miR-3928
GTGAGGACTCGGGAGGTGG	miR-1224-5p
AGAGCTTAGCTGATTGGTGAAC	miR-27b-5p
GGGAGCCAGGAAGTATTGATGT	miR-505-5p
GTCCCTCTCCAAATGTGTCTTG	miR-642a-5p
CGGCTCTGGGTCTGTGGGGA	miR-760
AGTGGGGAACCCTTCCATGAGG	miR-491-5p
CAGCAGCACACTGTGGTTTGT	miR-497-5p
AGAGTTGAGTCTGGACGTCCCG	miR-219-1-3p
TCTGTGAGACCAAAGAACTACT	miR-4677-3p
CAGTGCAATGATATTGTCAAAGC	miR-301b

TATGGAAAGACTTTGCCACTCT	miR-3688-3p
TCAAGTGTCATCTGTCCCTAG	miR-6513-3p
TCTCCCTTCCTGCCCTGGCTAG	miR-4685-3p
TAGTGGTCAGAGGGCTTATGA	miR-6718-5p
CTGGGATCTCCGGGGTCTTGGTT	miR-769-3p
AGGTCTGCATTCAAATCCCCAGA	miR-6503-5p
AGCGCGGGCTGAGCGCTGCCAGTC	miR-2277-5p
CTTTCAGTCAGATGTTTGCTGC	miR-30d-3p
ACTGGGCTTGGAGTCAGAAG	miR-378g
CAGGTCGTCTTGCAGGGCTTCT	miR-431-3p
CTCCTGGGGCCCGCACTCTCGC	miR-1343
TTTGTGACCTGGTCCACTAACC	miR-758-3p
AGCCCGCCCCAGCCGAGGTTCT	miR-4707-3p
GTTCCTGCTGAACTGAGCCAG	miR-3074-5p
CGGATGAGCAAAGAAAGTGGTT	miR-1255b-5p
TCACCCTGCATCCCGCACCCAG	miR-3620-3p
GCTGGTTTCATATGGTGGTTTAGA	miR-29b-1-5p
CTCTCACCACTGCCCTCCCACAG	miR-1229-3p
TAGCACCATTTGAAATCAGTGTT	miR-29b-3p
TGCCCTGCCTGTTTTCTCCTTT	miR-3173-5p
ATCCCCAGATACAATGGACAA	miR-2355-5p
CTGCCCTAGTCTAGCTGAAGCT	miR-3157-3p
ACTGGACTTGGAGCCAGAAG	miR-378f
TGAGCCCTGTCCTCCCGCAG	miR-1233-3p
CTTGGCACCTAGCAAGCACTCA	miR-1271-5p
CTGGAGATATGGAAGAGCTGTGT	miR-1270
CTTTCAGTCGGATGTTTGCAGC	miR-30a-3p
GCTGCGCTTGGATTTCGTCCCC	miR-191-3p
ACTGGACTTGGAGGCAGAA	miR-378b
GCAAAGCACACGGCCTGCAGAGA	miR-330-3p
CCACTTGGATCTGAAGGCTGCCC	miR-3614-5p
ACAGGTGAGGTTCTTGGGAGCC	miR-125a-3p
TTGGCCATGGGGCTGCGCGG	miR-3187-3p
TTAATATCGGACAACCATTGT	miR-889
ATCCTTGCTATCTGGGTGCTA	miR-502-5p
TAACAGTCTCCAGTCACGGCC	miR-212-3p
TGACAGCGCCCTGCCTGGCTC	miR-2277-3p
GGGACTAGGATGCAGACCTCC	miR-6503-3p
TTCCCTTTGTCATCCTATGCCT	miR-204-5p
CAGCAGCAATTCATGTTTTGAA	miR-424-5p
GGAGACTGATGAGTTCCCGGGA	miR-873-3p
AGCTTCTTTACAGTGCTGCCTTG	miR-103a-2-5p
ACTGATTTCTTTTGGTGTTCAG	miR-29a-5p
TTCTGCCTCTGTCCAGGTCCTT	miR-5001-3p
AGTTCTTCAGTGGCAAGCTTTA	miR-22-5p
TGACCGATTTCTCCTGGTGTTC	miR-29c-5p
ACTGGACTTGGAGTCAGAAA	miR-378d
AGCTTCCATGACTCCTGATGGA	miR-2115-5p
TTCAACGGGTATTTATTGAGCA	miR-95
TTTGTTCGTTCGGCTCGCGTGA	miR-375
TGGGGAGCTGAGGCTCTGGGGGTG	miR-939-5p
TCAGGTGTGGAAACTGAGGCAG	miR-3934-5p
TGATCAGGCAAAATTGCAGACT	miR-4772-5p
TTTTGCGATGTGTTCCTAATAT	miR-450a-5p
CCAGTGGGGCTGCTGTTATCTG	miR-194-3p
TTTAGAGACGGGGTCTTGCTCT	miR-1303
TGATTGGTACGTCTGTGGGTAG	miR-509-3p
TGTGAGGTTGGCATTGTTGTCT	miR-1294
TGGCAGTGTCTTAGCTGGTTGT	miR-34a-5p

TCATCCTCGTCTCCCTCCCAG	miR-5196-3p
GTGAGTCTCTAAGAAAAGAGGA	miR-627
TGTCACTCGGCTCGGCCCACTAC	miR-668
ACTGGACTTGGAGTCAGGA	miR-378e
GGGGCTGGGCGCGCGCC	miR-4492
TCCTTCTGCTCCGTCCCCCAG	miR-1237-3p
CCAGTCCTGTGCCTGCCGCCT	miR-1910
TGAGGATGGATAGCAAGGAAGCC	miR-3605-5p
AGCTGGTGTTGTGAATCAGGCCG	miR-138-5p
TATGTCTGCTGACCATCACCTT	miR-654-3p
GGTGCAGTGCTGCATCTCTGGT	miR-143-5p
CTGGTACAGGCCTGGGGGACAG	miR-150-3p
GCGACCCACTCTTGGTTTCCA	miR-551a
ACCGTGGCTTTCGATTGTTACT	miR-132-5p
CGGCCCGGGCTGCTGCTGTTCCT	miR-1538
ACTCAAAACCCTTCAGTGACTT	miR-616-5p
AAGGAGCTTACAATCTAGCTGGG	miR-708-5p
TCCTCTTCTCCCTCCTCCCAG	miR-877-3p
ATATACAGGGGGAGACTCTTAT	miR-1185-1-3p
TAGCAGCACAGAAATATTGGC	miR-195-5p
GTTCTCCCAACGTAAGCCCAGC	miR-629-3p
AAAGACATAGGATAGAGTCACCTC	miR-641
TACCCTGTAGAACCGAATTTGTG	miR-10b-5p
ACTGGACTAGGAGTCAGAAGG	miR-378i
CAGCCCTCCTCCCGCACCCAAA	miR-4687-5p
ACCTTCCTCTCCATGGGTCTTT	miR-3667-3p
GAAGAACTGTTGCATTTGCCCT	miR-4511
AGAGTCGGCGACGCCGCCAGC	miR-4785
AGCTCTAGAAAGATTGTTGACC	miR-6502-5p
GCAGGAACTTGTGAGTCTCCT	miR-873-5p
TGGCCCTGACTGAAGACCAGCAGT	miR-1291
TGGAGACGCGGCCCTGTTGGAGT	miR-139-3p
AGGGGCTGGCTTTCCTCTGGTC	miR-185-3p
AGGCAGTGTAGTTAGCTGATTGC	miR-34c-5p
AGCCTGGAAGCTGGAGCCTGCAGT	miR-1254
AACTAGCTCTGTGGATCCTGAC	miR-4661-5p
TCTTTGGTTATCTAGCTGTATGA	miR-9-5p
AAAAACTGAGACTACTTTTGCA	miR-548e
TAATTTTATGTATAAGCTAGT	miR-590-3p
AACTGGCCTACAAAGTCCCAGT	miR-193a-3p
CTGGGCCCGCGGCGGGCGTGGGG	miR-6724-5p
TTTGGGATTGACGCCACATGTCT	miR-6513-5p
ATTGTCCCTCTCCCTTCCCAG	miR-4646-3p
CACAGCAAGTGTAGACAGGCA	miR-3120-3p
TCAAGAGCAATAACGAAAAATGT	miR-335-5p
TGGTAGACTATGGAACGTAGG	miR-379-5p
TAGGTTATCCGTGTTGCCTTCG	miR-154-5p
CAACAAATCACAGTCTGCCATA	miR-7-1-3p
CTATACGGCCTCCTAGCTTTCC	let-7e-3p
AGGAGGAATTGGTGCTGGTCTT	miR-766-5p
AGGGGGCGGGCTCCGGCG	miR-4488
TAGATAAAATATTGGTACCTG	miR-577
GGCCAGCCACCAGGAGGGCTG	miR-3194-5p
AAAAGTATTTGCGGGTTTTGTC	miR-548l
TGCCTAGGCTGAGACTGCAGTG	miR-3135a
CTGTTGCCACTAACCTCAACCT	miR-744-3p
CTATACAATCTACTGTCTTTC	let-7a-3p
TCAGCACCAGGATATTGTTGGAG	miR-3065-3p
CTGACTGTTGCCGTCCTCCAG	miR-943

AGGGGGATGGCAGAGCAAAATT	miR-5010-5p
CCAATATTACTGTGCTGCTTTA	miR-16-2-3p
ATCAGGGCTTGTGGAATGGGAAG	miR-3127-5p
ATCCCACCTCTGCCACCA	miR-1260a
TGAGCTAAATGTGTGCTGGGA	miR-610
AACCATCGACCGTTGAGTGGAC	miR-181c-3p
GGAGAAATTATCCTTGGTGTGT	miR-539-5p
CATCATCGTCTCAAATGAGTCT	miR-136-3p
ATTGTCCTTGCTGTTTGGAGAT	miR-2355-3p
TGACCTGGGACTCGGACAGCTG	miR-3661
GCAGCCCAGCTGAGGCCTCTG	miR-4690-3p
CCAACCTAGGTGGTCAGAGTTG	miR-4714-3p
AATCATACAGGGACATCCAGTT	miR-487a
TTAGCCAATTGTCCATCTTTAG	miR-4662a-5p
TGCCGCCCTCTCGCTGCTCTAG	miR-4632-3p
CGCCCCTCCTGCCCCCACAG	miR-4749-3p
ACCTTGGCTCTAGACTGCTTACT	miR-212-5p
TACGCGCAGACCACAGGATGTC	miR-3939
AAAAGTTATTGCGGTTTTGGCT	miR-548at-5p
AGGGACGGGACGCGGTGCAGTG	miR-92b-5p
AGACAGTAGTTCTTGCCTGGTT	miR-4645-3p
ACCTCCTGTGTGCATGGATTA	miR-660-3p
TAGGATTACAAGTGTCGGCCAC	miR-3159
TTCAGCCAGGCTAGTGCAGTCT	miR-3157-5p
TGGGCAGGGGCTTATTGTAGGAG	miR-6721-5p
CAGCCCGGATCCCAGCCCACTT	miR-3940-3p
GGGCTCACATCACCCCAT	miR-4284
TGTCGTGGGGCTTGCTGGCTTG	miR-4440
CATCCGTCCGTCTGTCCAC	miR-4800-3p
TTTCCCTTTCCATCCTGGCAG	miR-5006-3p
AGTGCCTGAGGGAGTAAGAGCCC	miR-550a-5p
AAAAGTAATTGTGGTTTTTGC	miR-548ay-5p
TTGGAGGCGTGGGTTTT	miR-4443
CAGTTATCACAGTGCTGATGCT	miR-101-5p
CCAGGCTCTGCAGTGGGA	miR-3155b
AAACAAACATGGTGCACTTCTT	miR-495-3p
ATGGCATCGTCCCCTGGTGGCT	miR-4642
TCAGTTCCAGGCCAACCAGGCT	miR-584-3p
AAAAGTAATTGCGGTTTTTGC	miR-548au-5p
GGGTGCGGGCCGGCGGGG	miR-4466
TTACACAGCTGGACAGAGGCA	miR-4672
CTGCCAATTCCATAGGTCACAG	miR-192-3p
AAGTGCCCCCACAGTTTGAGTGC	miR-371b-3p
AGGTTACCCGAGCAACTTTGCAT	miR-409-5p
TTAATTTTTTGTTTCGGTCACT	miR-4775
TACCCAGTCTCCGGTGCAGCC	miR-3130-5p
GCTAAGGAAGTCCTGTGCTCAG	miR-4521
TAACGCATAATATGGACATGT	miR-3912
CTTAGCAGGTTGTATTATCATT	miR-374b-3p
CCAGAGCAGCCTGCGGTAACAGT	miR-6501-3p
CGGGTAGAGAGGGCAGTGGGAGG	miR-197-5p
TCTATACAGACCCTGGCTTTTC	miR-1284
AGAGCTGGCTGAAGGGCAG	miR-4487
TCGGCCTGACCACCCACCCCAC	miR-1234-3p
TGTGACAGATTGATAACTGAAA	miR-542-3p
TAAGGTGCATCTAGTGCAGTTAG	miR-18b-5p
TGAGTATTACATGGCCAATCTC	miR-496
GAGCCAGTGGTGAGACAGTGA	miR-4676-5p
TTTCTTCTTAGACATGGCAACG	miR-4659a-3p

AGACCCTGGTCTGCACTCTATC	miR-504
ACGGTGCTGGATGTGGCCTTT	miR-1250
TCGTGTCTTGTGTTGCAGCCGG	miR-187-3p
TGAGGCGGGGGGGCGAGC	miR-6087
TGAGGCTCTGTTAGCCTTGGCTC	miR-2467-5p
GATATCAGCTCAGTAGGCACCG	miR-3074-3p
CCAGTTACCGCTTCCGCTACCGC	miR-935
TTTGGGACTGATCTTGATGTCT	miR-3913-5p
CTCGGGCGGAGGTGGTTGAGTG	miR-4750-5p
CCTCCCATGCCAAGAACTCCC	miR-2116-3p
TAGGGGCAGCAGAGGACCTGGG	miR-4688
CCAGGCTCTGCAGTGGGAACT	miR-3155a
TATAAAATGAGGGCAGTAAGAC	miR-3163
TGAGGATATGGCAGGGAAGGGGA	miR-3679-5p
TGGACGGAGAACTGATAAGGGT	miR-184
AAAGCTGGGTTGAGAAGG	miR-320e
CTTCCTCGTCTGTCTGCCCC	miR-1238-3p
TCTGGCTCCGTGTCTTCACTCCC	miR-149-5p
AGGATGAGCAAAGAAAGTAGATT	miR-1255a
ATAGGACTCATATAGTGCCAG	miR-3117-3p
TAGGTAGTTTCATGTTGTTGGG	miR-196a-5p
CACTGTGGGTACATGCT	miR-4318
TTTGAGGCTACAGTGAGATGTG	miR-1304-5p
CTAGGTATGGTCCCAGGGATCC	miR-331-5p
AGGGACTTTCAGGGGCAGCTGT	miR-365b-5p
AGGAGATCCTGGGTT	miR-3676-5p
TCAACAAAATCACTGATGCTGGA	miR-3065-5p
TCTCTTCATCTACCCCCCAG	miR-6515-3p
TAGTGAGTTAGAGATGCAGAGCC	miR-3174
CTCGTGGGCTCTGGCCACGGCC	miR-3677-3p
CAAGCTCGCTTCTATGGGTCTG	miR-99a-3p
TTATTGTCACGTTCTGATT	miR-5701
ATCCGCGCTCTGACTCTCTGCC	miR-937-3p
CGTCCCACCCCCCACTCCTGT	miR-4433-5p
CCCCGGGGAGCCCGGCG	miR-4532
TTTCTATTTCTCAGTGGGGCTC	miR-4482-3p
TAAAGAGCCCTGTGGAGACA	miR-1276
TACTTTTCTAGGTTGTTGGGG	miR-5003-3p

1644-1115 = 529 miRs >=100 reads tsample size limit

	sum =
	529 miRs
	XX samples

counts shown are exact matches + inclusions

each miR ranked by total reads

529 mature miRNAs:	ID
TATTGCACTTGTCCCGGCCTGT	miR-92a-3p
CAACGGAATCCCAAAAGCAGCTG	miR-191-5p
TCTCCCAACCCTTGTACCAGTG	miR-150-5p
TGAGGTAGTAGGTTGTATAGTT	let-7a-5p
AACATTCAACGCTGTCGGTGAGT	miR-181a-5p

TAGCAGCACGTAAATATTGGCG	miR-16-5p
TTCAAGTAATCCAGGATAGGCT	miR-26a-5p
AAACCGTTACCATTACTGAGTT	miR-451a
AAGCTGCCAGTTGAAGAACTGT	miR-22-3p
CATTGCACTTGTCTCGGTCTGA	miR-25-3p
TGAGGTAGTAGTTTGTGCTGTT	let-7i-5p
TGTAAACATCCCCGACTGGAAG	miR-30d-5p
TGAGGTAGTAGTTTGTACAGTT	let-7g-5p
TGAGGTAGTAGGTTGTGTGGTT	let-7b-5p
TGAGGTAGTAGATTGTATAGTT	let-7f-5p
AGCTCGGTCTGAGGCCCCTCAGT	miR-423-3p
TTCAAGTAATTCAGGATAGGT	miR-26b-5p
AGCAGCATTGTACAGGGCTATGA	miR-103a-3p
CAAAGAATTCTCCTTTTGGGCT	miR-186-5p
CACTAGATTGTGAGCTCCTGGA	miR-28-3p
ACTGGACTTGGAGTCAGAAGG	miR-378a-3p
CAAAGTGCTGTTCGTGCAGGTAG	miR-93-5p
TCAGTGCACTACAGAACTTTGT	miR-148a-3p
TGTCAGTTTGTCAAATACCCCA	miR-223-3p
TGAGAACTGAATTCCATAGGCT	miR-146b-5p
TACCACAGGGTAGAACCACGG	miR-140-3p
AAAAGCTGGGTTGAGAGGGCGA	miR-320a
TTTGGCAATGGTAGAACTCACACT	miR-182-5p
TCGAGGAGCTCACAGTCT	miR-151b
TCAGTGCATCACAGAACTTTGT	miR-148b-3p
TGTAAACATCCTACACTCTCAGC	miR-30c-5p
AGAGGTAGTAGGTTGCATAGTT	let-7d-5p
TGTAAACATCCTTGACTGGAAG	miR-30e-5p
TAGCTTATCAGACTGATGTTGA	miR-21-5p
TCGAGGAGCTCACAGTCTAGT	miR-151a-5p
TAGCAGCACATCATGGTTTACA	miR-15b-5p
CTAGACTGAAGCTCCTTGAGG	miR-151a-3p
TGAGAACTGAATTCCATGGGTT	miR-146a-5p
CACCCGGCTGTGTGCACATGTGC	miR-941
TGAGGGGCAGAGAGCGAGACTTT	miR-423-5p
TTCACCACCTTCTCCACCCAGC	miR-197-3p
AACATTCATTGCTGTCGGTGGGT	miR-181b-5p
TCAGGCTCAGTCCCCTCCCGAT	miR-484
CTGACCTATGAATTGACAGCC	miR-192-5p
TCTCACACAGAAATCGCACCCGT	miR-342-3p
AGCTACATCTGGCTACTGGGT	miR-222-3p
TTCACAGTGGCTAAGTTCTGC	miR-27b-3p
TCTCTCGGCTCCTCGCGGCTC	miR-3615
GGATATCATCATATACTGTAAG	miR-144-5p
TGAGATGAAGCACTGTAGCTC	miR-143-3p
TCGGATCCGTCTGAGCTTGGCT	miR-127-3p
ATCACATTGCCAGGGATTTCC	miR-23a-3p
AATGGCGCCACTAGGGTTGTG	miR-652-3p
CTTTCAGTCGGATGTTTACAGC	miR-30e-3p
CAACACCAGTCGATGGGCTGT	miR-21-3p
TACAGTACTGTGATAACTGAA	miR-101-3p
AATGACACGATCACTCCCGTTGA	miR-425-5p
AACATTCAACCTGTCGGTGAGT	miR-181c-5p
TTAATGCTAATCGTGATAGGGGT	miR-155-5p
TCCCTGAGACCCTTTAACCTGTGA	miR-125a-5p
AGCTACATTGTCTGCTGGGTTTC	miR-221-3p
TGAGACCTCTGGGTTCTGAGCT	miR-769-5p
CATTATTACTTTTGGTACGCG	miR-126-5p
CATAAAGTAGAAAGCACTACT	miR-142-5p

GCCCTGACCTGTCCTGTTCTG	miR-4732-3p
CAAAGTGCTTACAGTGCAGGTAG	miR-17-5p
TGTAAACATCCTACACTCAGCT	miR-30b-5p
TAAAGTGCTTATAGTGCAGGTAG	miR-20a-5p
TGGCTCAGTTCAGCAGGAACAG	miR-24-3p
TATGGCACTGGTAGAATTCACT	miR-183-5p
CTATACGACCTGCTGCCTTTCT	let-7d-3p
CGGGGCAGCTCAGTACAGGAT	miR-486-3p
TGAGGTAGTAAGTTGTATTGTT	miR-98-5p
CATGCCTTGAGTGTAGGACCGT	miR-532-5p
TAGCACCATCTGAAATCGGTTA	miR-29a-3p
GAATGTTGCTCGGTGAACCCCT	miR-409-3p
ATGCACCTGGGCAAGGATTCTG	miR-500a-3p
CACCCGTAGAACCGACCTTGCG	miR-99b-5p
TGCGGGGCTAGGGCTAACAGCA	miR-744-5p
ATCACATTGCCAGGGATTACC	miR-23b-3p
AATTGCACGGTATCCATCTGTA	miR-363-3p
ACTCGGCGTGGCGTCGGTCGTG	miR-1307-3p
TATTGCACTCGTCCCGGCCTCC	miR-92b-3p
TTATAAAGCAATGAGACTGATT	miR-340-5p
TTCACAGTGGCTAAGTTCCGC	miR-27a-3p
CCTCTGGGCCCTTCCTCCAG	miR-326
CTGGCCCTCTCTGCCCTTCCGT	miR-328
TCGTACCGTGAGTAATAATGCG	miR-126-3p
TCACAGTGAACCGGTCTCTTT	miR-128
TGAGGTAGGAGGTTGTATAGTT	let-7e-5p
ACCACTGACCGTTGACTGTACC	miR-181a-2-3p
TACAGTATAGATGATGTACT	miR-144-3p
GCTGACTCCTAGTCCAGGGCTC	miR-345-5p
CAGTGCAATGTTAAAAGGGCAT	miR-130a-3p
TAGCAGCACATAATGGTTTGTG	miR-15a-5p
AAGGAGCTCACAGTCTATTGAG	miR-28-5p
TCCGGTTCTCAGGGCTCCACC	miR-671-3p
CCGCACTGTGGGTACTTGCTGC	miR-106b-3p
TGCCCTGTGGACTCAGTTCTGG	miR-146b-3p
TAGTGCAATATTGCTTATAGGGT	miR-454-3p
GCCCCTGGGCCTATCCTAGAA	miR-331-3p
CAGTGCAATGATGAAAGGGCAT	miR-130b-3p
AGGGGTGCTATCTGTGATTGA	miR-342-5p
TACCCTGTAGATCCGAATTTGTG	miR-10a-5p
TGTAACAGCAACTCCATGTGGA	miR-194-5p
TACCCATTGCATATCGGAGTTG	miR-660-5p
ACCCCACTCCTGGTACC	miR-4286
TTATCAGAATCTCCAGGGGTAC	miR-361-5p
TAAAGTGCTGACAGTGCAGAT	miR-106b-5p
TGGAGAGAAAGGCAGTTCCTGA	miR-185-5p
ACCATCGACCGTTGATTGTACC	miR-181a-3p
TCCCCCAGGTGTGATTCTGATTT	miR-361-3p
GTCCAGTTTTCCCAGGAATCCCT	miR-145-5p
CAAAGTGCTCATAGTGCAGGTAG	miR-20b-5p
AGGCAAGATGCTGGCATAGCT	miR-31-5p
TGAGGTAGTAGGTTGTATGGTT	let-7c
TGTAAACATCCTCGACTGGAAG	miR-30a-5p
ACAGTAGTCTGCACATTGGTTA	miR-199a-3p
ACAGTAGTCTGCACATTGGTTA	miR-199b-3p
TATTCATTTATCCCCAGCCTACA	miR-664a-3p
ACTGCCCCAGGTGCTGCTGG	miR-324-3p
TAACAGTCTACAGCCATGGTCG	miR-132-3p
AATATAACACAGATGGCCTGT	miR-410

AAAGTTCTGAGACACTCCGACT	miR-148a-5p
TGTAGTGTTTCCTACTTTATGGA	miR-142-3p
ACTCTTTCCCTGTTGCACTAC	miR-130b-5p
CTGCCCTGGCCCGAGGGACCGA	miR-874
TGAGCGCCTCGACGACAGAGCCG	miR-339-3p
ACTCCAGCCCCACAGCCTCAGC	miR-766-3p
TGGGTCTTTGCGGGCGAGATGA	miR-193a-5p
AATGCACCCGGGCAAGGATTCT	miR-501-3p
CCTGTTCTCCATTACTTGGCTC	miR-26b-3p
CCCAGTGTTCAGACTACCTGTTC	miR-199a-5p
CACGCTCATGCACACACCCACA	miR-574-3p
TCGACCGGACCTCGACCGGCT	miR-1307-5p
AACATTCATTGTTGTCGGTGGGT	miR-181d
TGGGTTTACGTTGGGAGAACT	miR-629-5p
CTGTGCGTGTGACAGCGGCTGA	miR-210
AAGTTCTGTTATACACTCAGGC	miR-148b-5p
ATCGGGAATGTCGTGTCCGCCC	miR-425-3p
TGAGAACCACGTCTGCTCTGAG	miR-589-5p
ACTGCTGAGCTAGCACTTCCCG	miR-93-3p
GTAGAGGAGATGGCGCAGGG	miR-877-5p
TCCGTCTCAGTTACTTTATAGC	miR-340-3p
TGTGCAAATCCATGCAAAACTGA	miR-19b-3p
TTTCCGGCTCGCGTGGGTGTGT	miR-1180
CAAAACGTGAGGCGCTGCTAT	miR-424-3p
ATCAACAGACATTAATTGGGCGC	miR-421
TCTTCTCTGTTTTGGCCATGTG	miR-942
CCTCCCACACCCAAGGCTTGCA	miR-532-3p
CGTGTATTTGACAAGCTGAGTT	miR-223-5p
AAAAGTGCTTACAGTGCAGGTAG	miR-106a-5p
TATACAAGGGCAAGCTCTCTGT	miR-381-3p
TGTCCTCTAGGGCCTGCAGTCT	miR-3909
ACTGTAAACGCTTTCTGATG	miR-3607-3p
TTTTGTGTCTCCCATTCCCCAG	miR-5010-3p
AATCCTTGGAACCTAGGTGTGAGT	miR-362-5p
TCTACAGTGCACGTGTCTCCAGT	miR-139-5p
TCCCTGTCCTCCAGGAGCTCACG	miR-339-5p
GGCGGGTGCGGGGGTGG	miR-3656
CAGTGCAATAGTATTGTCAAAGC	miR-301a-3p
CGTCAACACTTGCTGGTTTCCT	miR-505-3p
TTGGGGAAACGGCCGCTGAGTG	miR-2110
CAGTGGTTTTACCCTATGGTAG	miR-140-5p
TCCAGCATCAGTGATTTTGTTG	miR-338-3p
CCTCCTGCCCTCCTTGCTGT	miR-1976
TAATACTGCCGGGTAATGATGGA	miR-200c-3p
AATGGATTTTTGGAGCAGG	miR-1246
TAGCACCATTTGAAATCGGTTA	miR-29c-3p
CACCTTGCGCTACTCAGGTCTG	miR-3200-3p
AAACTCTACTTGTCCTTCTGAGT	miR-618
TAGGTAGTTTCCTGTTGTTGGG	miR-196b-5p
CTCCGTTTGCCTGTTTCGCTG	miR-1468
TCTAGTAAGAGTGGCAGTCGA	miR-628-3p
CCCAGTGTTTAGACTATCTGTTC	miR-199b-5p
ACGCCCTTCCCCCCCTTCTTCA	miR-1249
TGTGACTGGTTGACCAGAGGGG	miR-134
TCAGTGCATGACAGAACTTGG	miR-152
CTCCTGACTCCAGGTCCTGTGT	miR-378a-5p
GTCATACACGGCTCTCCTCTCT	miR-485-3p
TGTTCCTCTGTCTCCCAGAC	miR-4326
AAAAGTACTTGCGGATTT	miR-548av-5p

TCTTGGAGTAGGTCATTGGGTGG	miR-432-5p
ATTCTAATTTCTCCACGTCTTT	miR-576-5p
CAGGGCTGGCAGTGACATGGGT	miR-4446-3p
AGGCGGAGACTTGGGCAATTG	miR-25-5p
AAAAGTACTTGCGGATTTTGCT	miR-548k
TTGTACATGGTAGGCTTTCATT	miR-493-5p
AGGGGGAAAGTTCTATAGTCC	miR-625-5p
ACCTGGCATACAATGTAGATTT	miR-221-5p
GAGTTCTACAGTCAGAC	miR-3168
ATATAATACAACCTGCTAAGTG	miR-374b-5p
AGCAGCATTGTACAGGGCTATCA	miR-107
CCTCCGTGTTACCTGTCCTCTAG	miR-3605-3p
TTATGGTTTGCCTGGGACTGAG	miR-584-5p
TCCCTGAGACCCTAACTTGTGA	miR-125b-5p
TAACTGGTTGAACAACTGAACC	miR-582-3p
AAGGGCTTCCTCTCTGCAGGAC	miR-3158-3p
CGCATCCCCTAGGGCATTGGTGT	miR-324-5p
ACTGGCCTGGGACTACCGG	miR-3176
GGGGTTCCTGGGGATGGGATTT	miR-23a-5p
CAAGTCACTAGTGGTTCCGTT	miR-224-5p
TGAGTGTGTGTGTGTGAGTGTGT	miR-574-5p
TGAGGAGATCGTCGAGGTTGG	miR-3150b-3p
TAAGGTGCATCTAGTGCAGATAG	miR-18a-5p
GCCTGCTGGGGTGGAACCTGGT	miR-370
AGTGCCTGAGGGAGTAAGAG	miR-550a-3-5p
TCAGAACAAATGCCGGTTCCCAGA	miR-589-3p
TTTGGTCCCCTTCAACCAGCTG	miR-133a
TGTAGAGCAGGGAGCAGGAAGCT	miR-4732-5p
CAGCCCCACAGCCTCAGA	miR-4323
ACTCAAAAGATGGCGGCACTTT	miR-371b-5p
TAGTAGACCGTATAGCGTACG	miR-411-5p
CCTCACCATCCCTTCTGCCTGC	miR-6511a-3p
TCTGGGCAACAAAGTGAGACCT	miR-1285-3p
CTGGGAGAGGGTTGTTTACTCC	miR-30c-1-3p
TCTCTGGGCCTGTGTCTTAGGC	miR-330-5p
AGGGCTTAGCTGCTTGTGAGCA	miR-27a-5p
TGATATGTTTGATATATTAGGT	miR-190a
TCAGCTACTACCTCTATTAGG	miR-5690
AACCCGTAGATCCGAACTTGTG	miR-100-5p
ACTGCCCTAAGTGCTCCTTCTGG	miR-18a-3p
CTGTACAGGCCACTGCCTTGC	let-7g-3p
AATCCTTTGTCCCTGGGTGAGA	miR-501-5p
TGTCTTGCAGGCCGTCATGCA	miR-431-5p
AGGTTGGGATCGGTTGCAATGCT	miR-92a-1-5p
AAAAGCTGGGTTGAGAGGGCAA	miR-320b
GGGAGAAGGGTCGGGGC	miR-4516
TTGCAGCTGCCTGGGAGTGACTTC	miR-1301
AATCCTTGCTACCTGGGT	miR-500b
TTTTTCATTATTGCTCCTGACC	miR-335-3p
CCGGTCCCAGGAGAACCTGCAGA	miR-4746-5p
TTATAATACAACCTGATAAGTG	miR-374a-5p
TTAGGGCCCTGGCTCCATCTCC	miR-1296
ACTGCAGTGAAGGCACTTGTAG	miR-17-3p
AGGGCCCCCCCTCAATCCTGT	miR-296-5p
AACTGGCCCTCAAAGTCCCGCT	miR-193b-3p
TTACAGTTGTTCAACCAGTTACT	miR-582-5p
TGCCTACTGAGCTGAAACACAG	miR-24-2-5p
TGTCTTACTCCCTCAGGCACAT	miR-550a-3p
GAAGTTGTTCGTGGTGGATTCG	miR-382-5p

CTGCGCAAGCTACTGCCTTGCT	let-7i-3p
CGAATCATTATTTGCTGCTCTA	miR-15b-3p
TAATGCCCCTAAAAATCCTTAT	miR-365a-3p
TAATGCCCCTAAAAATCCTTAT	miR-365b-3p
ATCATGATGGGCTCCTCGGTGT	miR-433
CGTGCCACCCTTTTCCCCAG	miR-1227-3p
AATGCACCTGGGCAAGGATTCA	miR-502-3p
AATCGTACAGGGTCATCCACTT	miR-487b
TAATACTGCCTGGTAATGATGA	miR-200b-3p
TCGGGGATCATCATGTCACGAGA	miR-542-5p
TGATATGTTTGATATTGGGTT	miR-190b
AATCTGAGAAGGCGCACAAGGT	miR-3200-5p
CGGGCGTGGTGGTGGGGG	miR-1268a
CATCAGAATTCATGGAGGCTAG	miR-2115-3p
ACCTGGACCCAGCGTAGACAAAG	miR-3690
GACTATAGAACTTTCCCCCTCA	miR-625-3p
TAATCCTTGCTACCTGGGTGAGA	miR-500a-5p
TGTGCAAATCTATGCAAAACTGA	miR-19a-3p
CTGGTTTCACATGGTGGCTTAG	miR-29b-2-5p
AAAACTGCAGTTACTTTTGC	miR-548av-3p
AAAAGCTGGGTTGAGAGGA	miR-320d
TGTGCTTGCTCGTCCCGCCCGCA	miR-636
CCAAAACTGCAGTTACTTTTGC	miR-548o-3p
CAAGCTCGTGTCTGTGGGTCCG	miR-99b-3p
CGGCGGGGACGGCGATTGGTC	miR-1908
CATCCCTTGCATGGTGGAGGG	miR-188-5p
ACGTTGGCTCTGGTGGTG	miR-1306-3p
CTTATCAGATTGTATTGTAATT	miR-374a-3p
GTGGGGGAGAGGCTGTC	miR-1275
CCTCACCACCCCTTCTGCCTGCA	miR-6511b-3p
GCGGGGCTGGGCGCGCG	miR-4508
AAACATTCGCGGTGCACTTCTT	miR-543
AGGACCTTCCCTGAACCAAGGA	miR-659-5p
TGGAAGACTAGTGATTTTGTTGT	miR-7-5p
TTCATTTGCCTCCCAGCCTACA	miR-664b-3p
AAAAGTAATTGCGGTCTTT	miR-548ap-5p
TCTCACTGTAGCCTCGAACCCC	miR-1304-3p
AAAAGCTGGGTTGAGAGGGT	miR-320c
CCTGCAACTTTGCCTGATCAGA	miR-4772-3p
TCACCAGCCCTGTGTTCCCTAG	miR-1226-3p
AGAGGTTGCCCTTGGTGAATTC	miR-377-5p
TGCTGGATCAGTGGTTCGAGTC	miR-1287
TGAAGGTCTACTGTGTGCCAGG	miR-493-3p
AGTTGCCAGGGCTGCCTTTGGT	miR-6501-5p
ATCCCACCACTGCCACCAT	miR-1260b
TGCACGGCACTGGGGACACGT	miR-3177-3p
AAAAGTAATTGCGGTCTTTGGT	miR-548j
CACATTACACGGTCGACCTCT	miR-323a-3p
TTTGGCACTAGCACATTTTTGCT	miR-96-5p
ATGCTGACATATTTACTAGAGG	miR-628-5p
AACCCGTAGATCCGATCTTGTG	miR-99a-5p
CCACCTCCCCTGCAAACGTCCA	miR-1306-5p
TACGTCATCGTTGTCATCGTCA	miR-598
CTAAGAAGTTGACTGAAG	miR-3653
TGGTGGGCCGCAGAACATGTGC	miR-654-5p
ACCCTATCAATATTGTCTCTGC	miR-454-5p
ATGGCCAGAGCTCACACAGAGG	miR-4435
AGGAAGCCCTGGAGGGGCTGGAG	miR-671-5p
AAGGCAGGGCCCCCGCTCCCC	miR-940

TGAAACATACACGGGAAACCTC	miR-494
AGAGGCTGGCCGTGATGAATTC	miR-485-5p
CTGGGAGGTGGATGTTTACTTC	miR-30b-3p
GGAGGAACCTTGGAGCTTCGGC	miR-3928
GTGAGGACTCGGGAGGTGG	miR-1224-5p
AGAGCTTAGCTGATTGGTGAAC	miR-27b-5p
GGGAGCCAGGAAGTATTGATGT	miR-505-5p
GTCCCTCTCCAAATGTGTCTTG	miR-642a-5p
CGGCTCTGGGTCTGTGGGGA	miR-760
AGTGGGGAACCCTTCCATGAGG	miR-491-5p
CAGCAGCACACTGTGGTTTGT	miR-497-5p
AGAGTTGAGTCTGGACGTCCCG	miR-219-1-3p
TCTGTGAGACCAAAGAACTACT	miR-4677-3p
CAGTGCAATGATATTGTCAAAGC	miR-301b
TATGGAAAGACTTTGCCACTCT	miR-3688-3p
TCAAGTGTCATCTGTCCCTAG	miR-6513-3p
TCTCCCTTCCTGCCCTGGCTAG	miR-4685-3p
TAGTGGTCAGAGGGCTTATGA	miR-6718-5p
CTGGGATCTCCGGGGTCTTGGTT	miR-769-3p
AGGTCTGCATTCAAATCCCCAGA	miR-6503-5p
AGCGCGGGCTGAGCGCTGCCAGTC	miR-2277-5p
CTTTCAGTCAGATGTTTGCTGC	miR-30d-3p
ACTGGGCTTGGAGTCAGAAG	miR-378g
CAGGTCGTCTTGCAGGGCTTCT	miR-431-3p
CTCCTGGGGCCCGCACTCTCGC	miR-1343
TTTGTGACCTGGTCCACTAACC	miR-758-3p
AGCCCGCCCCAGCCGAGGTTCT	miR-4707-3p
GTTCCTGCTGAACTGAGCCAG	miR-3074-5p
CGGATGAGCAAAGAAAGTGGTT	miR-1255b-5p
TCACCCTGCATCCCGCACCCAG	miR-3620-3p
GCTGGTTTCATATGGTGGTTTAGA	miR-29b-1-5p
CTCTCACCACTGCCCTCCCACAG	miR-1229-3p
TAGCACCATTTGAAATCAGTGTT	miR-29b-3p
TGCCCTGCCTGTTTTCTCCTTT	miR-3173-5p
ATCCCCAGATACAATGGACAA	miR-2355-5p
CTGCCCTAGTCTAGCTGAAGCT	miR-3157-3p
ACTGGACTTGGAGCCAGAAG	miR-378f
TGAGCCCTGTCCTCCCGCAG	miR-1233-3p
CTTGGCACCTAGCAAGCACTCA	miR-1271-5p
CTGGAGATATGGAAGAGCTGTGT	miR-1270
CTTTCAGTCGGATGTTTGCAGC	miR-30a-3p
GCTGCGCTTGGATTTCGTCCCC	miR-191-3p
ACTGGACTTGGAGGCAGAA	miR-378b
GCAAAGCACACGGCCTGCAGAGA	miR-330-3p
CCACTTGGATCTGAAGGCTGCCC	miR-3614-5p
ACAGGTGAGGTTCTTGGGAGCC	miR-125a-3p
TTGGCCATGGGGCTGCGCGG	miR-3187-3p
TTAATATCGGACAACCATTGT	miR-889
ATCCTTGCTATCTGGGTGCTA	miR-502-5p
TAACAGTCTCCAGTCACGGCC	miR-212-3p
TGACAGCGCCCTGCCTGGCTC	miR-2277-3p
GGGACTAGGATGCAGACCTCC	miR-6503-3p
TTCCCTTTGTCATCCTATGCCT	miR-204-5p
CAGCAGCAATTCATGTTTTGAA	miR-424-5p
GGAGACTGATGAGTTCCCGGGA	miR-873-3p
AGCTTCTTTACAGTGCTGCCTTG	miR-103a-2-5p
ACTGATTTCTTTTGGTGTTCAG	miR-29a-5p
TTCTGCCTCTGTCCAGGTCCTT	miR-5001-3p
AGTTCTTCAGTGGCAAGCTTTA	miR-22-5p

TGACCGATTTCTCCTGGTGTTC	miR-29c-5p
ACTGGACTTGGAGTCAGAAA	miR-378d
AGCTTCCATGACTCCTGATGGA	miR-2115-5p
TTCAACGGGTATTTATTGAGCA	miR-95
TTTGTTCGTTCGGCTCGCGTGA	miR-375
TGGGGAGCTGAGGCTCTGGGGGTG	miR-939-5p
TCAGGTGTGGAAACTGAGGCAG	miR-3934-5p
TGATCAGGCAAAATTGCAGACT	miR-4772-5p
TTTTGCGATGTGTTCCTAATAT	miR-450a-5p
CCAGTGGGGCTGCTGTTATCTG	miR-194-3p
TTTAGAGACGGGGTCTTGCTCT	miR-1303
TGATTGGTACGTCTGTGGGTAG	miR-509-3p
TGTGAGGTTGGCATTGTTGTCT	miR-1294
TGGCAGTGTCTTAGCTGGTTGT	miR-34a-5p
TCATCCTCGTCTCCCTCCCAG	miR-5196-3p
GTGAGTCTCTAAGAAAAGAGGA	miR-627
TGTCACTCGGCTCGGCCCACTAC	miR-668
ACTGGACTTGGAGTCAGGA	miR-378e
GGGGCTGGGCGCGCGCC	miR-4492
TCCTTCTGCTCCGTCCCCCAG	miR-1237-3p
CCAGTCCTGTGCCTGCCGCCT	miR-1910
TGAGGATGGATAGCAAGGAAGCC	miR-3605-5p
AGCTGGTGTTGTGAATCAGGCCG	miR-138-5p
TATGTCTGCTGACCATCACCTT	miR-654-3p
GGTGCAGTGCTGCATCTCTGGT	miR-143-5p
CTGGTACAGGCCTGGGGGACAG	miR-150-3p
GCGACCCACTCTTGGTTTCCA	miR-551a
ACCGTGGCTTTCGATTGTTACT	miR-132-5p
CGGCCCGGGCTGCTGCTGTTCCT	miR-1538
ACTCAAAACCCTTCAGTGACTT	miR-616-5p
AAGGAGCTTACAATCTAGCTGGG	miR-708-5p
TCCTCTTCTCCCTCCTCCCAG	miR-877-3p
ATATACAGGGGGAGACTCTTAT	miR-1185-1-3p
TAGCAGCACAGAAATATTGGC	miR-195-5p
GTTCTCCCAACGTAAGCCCAGC	miR-629-3p
AAAGACATAGGATAGAGTCACCTC	miR-641
TACCCTGTAGAACCGAATTTGTG	miR-10b-5p
ACTGGACTAGGAGTCAGAAGG	miR-378i
CAGCCCTCCTCCCGCACCCAAA	miR-4687-5p
ACCTTCCTCTCCATGGGTCTTT	miR-3667-3p
GAAGAACTGTTGCATTTGCCCT	miR-4511
AGAGTCGGCGACGCCGCCAGC	miR-4785
AGCTCTAGAAAGATTGTTGACC	miR-6502-5p
GCAGGAACTTGTGAGTCTCCT	miR-873-5p
TGGCCCTGACTGAAGACCAGCAGT	miR-1291
TGGAGACGCGGCCCTGTTGGAGT	miR-139-3p
AGGGGCTGGCTTTCCTCTGGTC	miR-185-3p
AGGCAGTGTAGTTAGCTGATTGC	miR-34c-5p
AGCCTGGAAGCTGGAGCCTGCAGT	miR-1254
AACTAGCTCTGTGGATCCTGAC	miR-4661-5p
TCTTTGGTTATCTAGCTGTATGA	miR-9-5p
AAAAACTGAGACTACTTTTGCA	miR-548e
TAATTTTATGTATAAGCTAGT	miR-590-3p
AACTGGCCTACAAAGTCCCAGT	miR-193a-3p
CTGGGCCCGCGGCGGGCGTGGGG	miR-6724-5p
TTTGGGATTGACGCCACATGTCT	miR-6513-5p
ATTGTCCCTCTCCCTTCCCAG	miR-4646-3p
CACAGCAAGTGTAGACAGGCA	miR-3120-3p
TCAAGAGCAATAACGAAAAATGT	miR-335-5p

TGGTAGACTATGGAACGTAGG	miR-379-5p
TAGGTTATCCGTGTTGCCTTCG	miR-154-5p
CAACAAATCACAGTCTGCCATA	miR-7-1-3p
CTATACGGCCTCCTAGCTTTCC	let-7e-3p
AGGAGGAATTGGTGCTGGTCTT	miR-766-5p
AGGGGGCGGGCTCCGGCG	miR-4488
TAGATAAAATATTGGTACCTG	miR-577
GGCCAGCCACCAGGAGGGCTG	miR-3194-5p
AAAAGTATTTGCGGGTTTTGTC	miR-548l
TGCCTAGGCTGAGACTGCAGTG	miR-3135a
CTGTTGCCACTAACCTCAACCT	miR-744-3p
CTATACAATCTACTGTCTTTC	let-7a-3p
TCAGCACCAGGATATTGTTGGAG	miR-3065-3p
CTGACTGTTGCCGTCCTCCAG	miR-943
AGGGGGATGGCAGAGCAAAATT	miR-5010-5p
CCAATATTACTGTGCTGCTTTA	miR-16-2-3p
ATCAGGGCTTGTGGAATGGGAAG	miR-3127-5p
ATCCCACCTCTGCCACCA	miR-1260a
TGAGCTAAATGTGTGCTGGGA	miR-610
AACCATCGACCGTTGAGTGGAC	miR-181c-3p
GGAGAAATTATCCTTGGTGTGT	miR-539-5p
CATCATCGTCTCAAATGAGTCT	miR-136-3p
ATTGTCCTTGCTGTTTGGAGAT	miR-2355-3p
TGACCTGGGACTCGGACAGCTG	miR-3661
GCAGCCCAGCTGAGGCCTCTG	miR-4690-3p
CCAACCTAGGTGGTCAGAGTTG	miR-4714-3p
AATCATACAGGGACATCCAGTT	miR-487a
TTAGCCAATTGTCCATCTTTAG	miR-4662a-5p
TGCCGCCCTCTCGCTGCTCTAG	miR-4632-3p
CGCCCCTCCTGCCCCCACAG	miR-4749-3p
ACCTTGGCTCTAGACTGCTTACT	miR-212-5p
TACGCGCAGACCACAGGATGTC	miR-3939
AAAAGTTATTGCGGTTTTGGCT	miR-548at-5p
AGGGACGGGACGCGGTGCAGTG	miR-92b-5p
AGACAGTAGTTCTTGCCTGGTT	miR-4645-3p
ACCTCCTGTGTGCATGGATTA	miR-660-3p
TAGGATTACAAGTGTCGGCCAC	miR-3159
TTCAGCCAGGCTAGTGCAGTCT	miR-3157-5p
TGGGCAGGGGCTTATTGTAGGAG	miR-6721-5p
CAGCCCGGATCCCAGCCCACTT	miR-3940-3p
GGGCTCACATCACCCCAT	miR-4284
TGTCGTGGGGCTTGCTGGCTTG	miR-4440
CATCCGTCCGTCTGTCCAC	miR-4800-3p
TTTCCCTTTCCATCCTGGCAG	miR-5006-3p
AGTGCCTGAGGGAGTAAGAGCCC	miR-550a-5p
AAAAGTAATTGTGGTTTTTGC	miR-548ay-5p
TTGGAGGCGTGGGTTTT	miR-4443
CAGTTATCACAGTGCTGATGCT	miR-101-5p
CCAGGCTCTGCAGTGGGA	miR-3155b
AAACAAACATGGTGCACTTCTT	miR-495-3p
ATGGCATCGTCCCCTGGTGGCT	miR-4642
TCAGTTCCAGGCCAACCAGGCT	miR-584-3p
AAAAGTAATTGCGGTTTTTGC	miR-548au-5p
GGGTGCGGGCCGGCGGGG	miR-4466
TTACACAGCTGGACAGAGGCA	miR-4672
CTGCCAATTCCATAGGTCACAG	miR-192-3p
AAGTGCCCCCACAGTTTGAGTGC	miR-371b-3p
AGGTTACCCGAGCAACTTTGCAT	miR-409-5p
TTAATTTTTTGTTTCGGTCACT	miR-4775

TACCCAGTCTCCGGTGCAGCC	miR-3130-5p
GCTAAGGAAGTCCTGTGCTCAG	miR-4521
TAACGCATAATATGGACATGT	miR-3912
CTTAGCAGGTTGTATTATCATT	miR-374b-3p
CCAGAGCAGCCTGCGGTAACAGT	miR-6501-3p
CGGGTAGAGAGGGCAGTGGGAGG	miR-197-5p
TCTATACAGACCCTGGCTTTTC	miR-1284
AGAGCTGGCTGAAGGGCAG	miR-4487
TCGGCCTGACCACCCACCCCAC	miR-1234-3p
TGTGACAGATTGATAACTGAAA	miR-542-3p
TAAGGTGCATCTAGTGCAGTTAG	miR-18b-5p
TGAGTATTACATGGCCAATCTC	miR-496
GAGCCAGTGGTGAGACAGTGA	miR-4676-5p
TTTCTTCTTAGACATGGCAACG	miR-4659a-3p
AGACCCTGGTCTGCACTCTATC	miR-504
ACGGTGCTGGATGTGGCCTTT	miR-1250
TCGTGTCTTGTGTTGCAGCCGG	miR-187-3p
TGAGGCGGGGGGGCGAGC	miR-6087
TGAGGCTCTGTTAGCCTTGGCTC	miR-2467-5p
GATATCAGCTCAGTAGGCACCG	miR-3074-3p
CCAGTTACCGCTTCCGCTACCGC	miR-935
TTTGGGACTGATCTTGATGTCT	miR-3913-5p
CTCGGGCGGAGGTGGTTGAGTG	miR-4750-5p
CCTCCCATGCCAAGAACTCCC	miR-2116-3p
TAGGGGCAGCAGAGGACCTGGG	miR-4688
CCAGGCTCTGCAGTGGGAACT	miR-3155a
TATAAAATGAGGGCAGTAAGAC	miR-3163
TGAGGATATGGCAGGGAAGGGGA	miR-3679-5p
TGGACGGAGAACTGATAAGGGT	miR-184
AAAGCTGGGTTGAGAAGG	miR-320e
CTTCCTCGTCTGTCTGCCCC	miR-1238-3p
TCTGGCTCCGTGTCTTCACTCCC	miR-149-5p
AGGATGAGCAAAGAAAGTAGATT	miR-1255a
ATAGGACTCATATAGTGCCAG	miR-3117-3p
TAGGTAGTTTCATGTTGTTGGG	miR-196a-5p
CACTGTGGGTACATGCT	miR-4318
TTTGAGGCTACAGTGAGATGTG	miR-1304-5p
CTAGGTATGGTCCCAGGGATCC	miR-331-5p
AGGGACTTTCAGGGGCAGCTGT	miR-365b-5p
AGGAGATCCTGGGTT	miR-3676-5p
TCAACAAAATCACTGATGCTGGA	miR-3065-5p
TCTCTTCATCTACCCCCCAG	miR-6515-3p
TAGTGAGTTAGAGATGCAGAGCC	miR-3174
CTCGTGGGCTCTGGCCACGGCC	miR-3677-3p
CAAGCTCGCTTCTATGGGTCTG	miR-99a-3p
TTATTGTCACGTTCTGATT	miR-5701
ATCCGCGCTCTGACTCTCTGCC	miR-937-3p
CGTCCCACCCCCCACTCCTGT	miR-4433-5p
CCCCGGGGAGCCCGGCG	miR-4532
TTTCTATTTCTCAGTGGGGCTC	miR-4482-3p
TAAAGAGCCCTGTGGAGACA	miR-1276
TACTTTTCTAGGTTGTTGGGG	miR-5003-3p

1917-1658 = 259 miRNAs

Selected from XX samples X 530 miRNAs

Instances/ave top 30 miRNAs	259 miRs
B_33vC_2	XX samples

0.90 sum <> "" =

C1102 samples included

XX UCs, XX CHR-NPs, XX CHR-Ps

counts shown are exact matches + inclusions

each miR ranked by total reads

259 mature miRNAs:	ID
TATTGCACTTGTCCCGGCCTGT	miR-92a-3p
CAACGGAATCCCAAAAGCAGCTG	miR-191-5p
TCTCCCAACCCTTGTACCAGTG	miR-150-5p
TGAGGTAGTAGGTTGTATAGTT	let-7a-5p
AACATTCAACGCTGTCGGTGAGT	miR-181a-5p
TAGCAGCACGTAAATATTGGCG	miR-16-5p
TTCAAGTAATCCAGGATAGGCT	miR-26a-5p
AAACCGTTACCATTACTGAGTT	miR-451a
AAGCTGCCAGTTGAAGAACTGT	miR-22-3p
CATTGCACTTGTCTCGGTCTGA	miR-25-3p
TGAGGTAGTAGTTTGTGCTGTT	let-7i-5p
TGTAAACATCCCCGACTGGAAG	miR-30d-5p
TGAGGTAGTAGTTTGTACAGTT	let-7g-5p
TGAGGTAGTAGGTTGTGTGGTT	let-7b-5p
TGAGGTAGTAGATTGTATAGTT	let-7f-5p
AGCTCGGTCTGAGGCCCCTCAGT	miR-423-3p
TTCAAGTAATTCAGGATAGGT	miR-26b-5p
AGCAGCATTGTACAGGGCTATGA	miR-103a-3p
CAAAGAATTCTCCTTTTGGGCT	miR-186-5p
CACTAGATTGTGAGCTCCTGGA	miR-28-3p
ACTGGACTTGGAGTCAGAAGG	miR-378a-3p
CAAAGTGCTGTTCGTGCAGGTAG	miR-93-5p
TCAGTGCACTACAGAACTTTGT	miR-148a-3p
TGTCAGTTTGTCAAATACCCCA	miR-223-3p
TGAGAACTGAATTCCATAGGCT	miR-146b-5p
TACCACAGGGTAGAACCACGG	miR-140-3p
AAAAGCTGGGTTGAGAGGGCGA	miR-320a
TTTGGCAATGGTAGAACTCACACT	miR-182-5p
TCGAGGAGCTCACAGTCT	miR-151b
TCAGTGCATCACAGAACTTTGT	miR-148b-3p
TGTAAACATCCTACACTCTCAGC	miR-30c-5p
AGAGGTAGTAGGTTGCATAGTT	let-7d-5p
TGTAAACATCCTTGACTGGAAG	miR-30e-5p
TAGCTTATCAGACTGATGTTGA	miR-21-5p
TCGAGGAGCTCACAGTCTAGT	miR-151a-5p
TAGCAGCACATCATGGTTTACA	miR-15b-5p
CTAGACTGAAGCTCCTTGAGG	miR-151a-3p
TGAGAACTGAATTCCATGGGTT	miR-146a-5p
CACCCGGCTGTGTGCACATGTGC	miR-941
TGAGGGGCAGAGAGCGAGACTTT	miR-423-5p
TTCACCACCTTCTCCACCCAGC	miR-197-3p
AACATTCATTGCTGTCGGTGGGT	miR-181b-5p
TCAGGCTCAGTCCCCTCCCGAT	miR-484
CTGACCTATGAATTGACAGCC	miR-192-5p
TCTCACACAGAAATCGCACCCGT	miR-342-3p
AGCTACATCTGGCTACTGGGT	miR-222-3p
TTCACAGTGGCTAAGTTCTGC	miR-27b-3p
TCTCTCGGCTCCTCGCGGCTC	miR-3615
GGATATCATCATATACTGTAAG	miR-144-5p
TGAGATGAAGCACTGTAGCTC	miR-143-3p
TCGGATCCGTCTGAGCTTGGCT	miR-127-3p
ATCACATTGCCAGGGATTTCC	miR-23a-3p

AATGGCGCCACTAGGGTTGTG	miR-652-3p
CTTTCAGTCGGATGTTTACAGC	miR-30e-3p
CAACACCAGTCGATGGGCTGT	miR-21-3p
TACAGTACTGTGATAACTGAA	miR-101-3p
AATGACACGATCACTCCCGTTGA	miR-425-5p
AACATTCAACCTGTCGGTGAGT	miR-181c-5p
TTAATGCTAATCGTGATAGGGGT	miR-155-5p
TCCCTGAGACCCTTTAACCTGTGA	miR-125a-5p
AGCTACATTGTCTGCTGGGTTTC	miR-221-3p
TGAGACCTCTGGGTTCTGAGCT	miR-769-5p
CATTATTACTTTTGGTACGCG	miR-126-5p
CATAAAGTAGAAAGCACTACT	miR-142-5p
GCCCTGACCTGTCCTGTTCTG	miR-4732-3p
CAAAGTGCTTACAGTGCAGGTAG	miR-17-5p
TGTAAACATCCTACACTCAGCT	miR-30b-5p
TAAAGTGCTTATAGTGCAGGTAG	miR-20a-5p
TGGCTCAGTTCAGCAGGAACAG	miR-24-3p
TATGGCACTGGTAGAATTCACT	miR-183-5p
CTATACGACCTGCTGCCTTTCT	let-7d-3p
CGGGGCAGCTCAGTACAGGAT	miR-486-3p
TGAGGTAGTAAGTTGTATTGTT	miR-98-5p
CATGCCTTGAGTGTAGGACCGT	miR-532-5p
TAGCACCATCTGAAATCGGTTA	miR-29a-3p
GAATGTTGCTCGGTGAACCCCT	miR-409-3p
ATGCACCTGGGCAAGGATTCTG	miR-500a-3p
CACCCGTAGAACCGACCTTGCG	miR-99b-5p
TGCGGGGCTAGGGCTAACAGCA	miR-744-5p
ATCACATTGCCAGGGATTACC	miR-23b-3p
AATTGCACGGTATCCATCTGTA	miR-363-3p
ACTCGGCGTGGCGTCGGTCGTG	miR-1307-3p
TATTGCACTCGTCCCGGCCTCC	miR-92b-3p
TTATAAAGCAATGAGACTGATT	miR-340-5p
TTCACAGTGGCTAAGTTCCGC	miR-27a-3p
CCTCTGGGCCCTTCCTCCAG	miR-326
CTGGCCCTCTCTGCCCTTCCGT	miR-328
TCGTACCGTGAGTAATAATGCG	miR-126-3p
TCACAGTGAACCGGTCTCTTT	miR-128
TGAGGTAGGAGGTTGTATAGTT	let-7e-5p
ACCACTGACCGTTGACTGTACC	miR-181a-2-3p
TACAGTATAGATGATGTACT	miR-144-3p
GCTGACTCCTAGTCCAGGGCTC	miR-345-5p
CAGTGCAATGTTAAAAGGGCAT	miR-130a-3p
TAGCAGCACATAATGGTTTGTG	miR-15a-5p
AAGGAGCTCACAGTCTATTGAG	miR-28-5p
TCCGGTTCTCAGGGCTCCACC	miR-671-3p
CCGCACTGTGGGTACTTGCTGC	miR-106b-3p
TGCCCTGTGGACTCAGTTCTGG	miR-146b-3p
TAGTGCAATATTGCTTATAGGGT	miR-454-3p
GCCCCTGGGCCTATCCTAGAA	miR-331-3p
CAGTGCAATGATGAAAGGGCAT	miR-130b-3p
AGGGGTGCTATCTGTGATTGA	miR-342-5p
TACCCTGTAGATCCGAATTTGTG	miR-10a-5p
TGTAACAGCAACTCCATGTGGA	miR-194-5p
TACCCATTGCATATCGGAGTTG	miR-660-5p
ACCCCACTCCTGGTACC	miR-4286
TTATCAGAATCTCCAGGGGTAC	miR-361-5p
TAAAGTGCTGACAGTGCAGAT	miR-106b-5p
TGGAGAGAAAGGCAGTTCCTGA	miR-185-5p
ACCATCGACCGTTGATTGTACC	miR-181a-3p

TCCCCCAGGTGTGATTCTGATTT	miR-361-3p
GTCCAGTTTTCCCAGGAATCCCT	miR-145-5p
CAAAGTGCTCATAGTGCAGGTAG	miR-20b-5p
AGGCAAGATGCTGGCATAGCT	miR-31-5p
TGAGGTAGTAGGTTGTATGGTT	let-7c
TGTAAACATCCTCGACTGGAAG	miR-30a-5p
ACAGTAGTCTGCACATTGGTTA	miR-199a-3p
ACAGTAGTCTGCACATTGGTTA	miR-199b-3p
TATTCATTTATCCCCAGCCTACA	miR-664a-3p
ACTGCCCCAGGTGCTGCTGG	miR-324-3p
TAACAGTCTACAGCCATGGTCG	miR-132-3p
AATATAACACAGATGGCCTGT	miR-410
AAAGTTCTGAGACACTCCGACT	miR-148a-5p
TGTAGTGTTTCCTACTTTATGGA	miR-142-3p
ACTCTTTCCCTGTTGCACTAC	miR-130b-5p
CTGCCCTGGCCCGAGGGACCGA	miR-874
TGAGCGCCTCGACGACAGAGCCG	miR-339-3p
ACTCCAGCCCCACAGCCTCAGC	miR-766-3p
TGGGTCTTTGCGGGCGAGATGA	miR-193a-5p
AATGCACCCGGGCAAGGATTCT	miR-501-3p
CCTGTTCTCCATTACTTGGCTC	miR-26b-3p
CCCAGTGTTCAGACTACCTGTTC	miR-199a-5p
CACGCTCATGCACACACCCACA	miR-574-3p
TCGACCGGACCTCGACCGGCT	miR-1307-5p
AACATTCATTGTTGTCGGTGGGT	miR-181d
TGGGTTTACGTTGGGAGAACT	miR-629-5p
CTGTGCGTGTGACAGCGGCTGA	miR-210
AAGTTCTGTTATACACTCAGGC	miR-148b-5p
ATCGGGAATGTCGTGTCCGCCC	miR-425-3p
TGAGAACCACGTCTGCTCTGAG	miR-589-5p
ACTGCTGAGCTAGCACTTCCCG	miR-93-3p
GTAGAGGAGATGGCGCAGGG	miR-877-5p
TCCGTCTCAGTTACTTTATAGC	miR-340-3p
TGTGCAAATCCATGCAAAACTGA	miR-19b-3p
TTTCCGGCTCGCGTGGGTGTGT	miR-1180
CAAAACGTGAGGCGCTGCTAT	miR-424-3p
ATCAACAGACATTAATTGGGCGC	miR-421
TCTTCTCTGTTTTGGCCATGTG	miR-942
CCTCCCACACCCAAGGCTTGCA	miR-532-3p
CGTGTATTTGACAAGCTGAGTT	miR-223-5p
AAAAGTGCTTACAGTGCAGGTAG	miR-106a-5p
TATACAAGGGCAAGCTCTCTGT	miR-381-3p
TGTCCTCTAGGGCCTGCAGTCT	miR-3909
ACTGTAAACGCTTTCTGATG	miR-3607-3p
TTTTGTGTCTCCCATTCCCCAG	miR-5010-3p
AATCCTTGGAACCTAGGTGTGAGT	miR-362-5p
TCTACAGTGCACGTGTCTCCAGT	miR-139-5p
TCCCTGTCCTCCAGGAGCTCACG	miR-339-5p
GGCGGGTGCGGGGGTGG	miR-3656
CAGTGCAATAGTATTGTCAAAGC	miR-301a-3p
CGTCAACACTTGCTGGTTTCCT	miR-505-3p
TTGGGGAAACGGCCGCTGAGTG	miR-2110
CAGTGGTTTTACCCTATGGTAG	miR-140-5p
TCCAGCATCAGTGATTTTGTTG	miR-338-3p
CCTCCTGCCCTCCTTGCTGT	miR-1976
TAATACTGCCGGGTAATGATGGA	miR-200c-3p
AATGGATTTTTGGAGCAGG	miR-1246
TAGCACCATTTGAAATCGGTTA	miR-29c-3p
CACCTTGCGCTACTCAGGTCTG	miR-3200-3p

AAACTCTACTTGTCCTTCTGAGT	miR-618
TAGGTAGTTTCCTGTTGTTGGG	miR-196b-5p
CTCCGTTTGCCTGTTTCGCTG	miR-1468
TCTAGTAAGAGTGGCAGTCGA	miR-628-3p
CCCAGTGTTTAGACTATCTGTTC	miR-199b-5p
ACGCCCTTCCCCCCCTTCTTCA	miR-1249
TGTGACTGGTTGACCAGAGGGG	miR-134
TCAGTGCATGACAGAACTTGG	miR-152
CTCCTGACTCCAGGTCCTGTGT	miR-378a-5p
GTCATACACGGCTCTCCTCTCT	miR-485-3p
TGTTCCTCTGTCTCCCAGAC	miR-4326
AAAAGTACTTGCGGATTT	miR-548av-5p
TCTTGGAGTAGGTCATTGGGTGG	miR-432-5p
ATTCTAATTTCTCCACGTCTTT	miR-576-5p
CAGGGCTGGCAGTGACATGGGT	miR-4446-3p
AGGCGGAGACTTGGGCAATTG	miR-25-5p
AAAAGTACTTGCGGATTTTGCT	miR-548k
TTGTACATGGTAGGCTTTCATT	miR-493-5p
AGGGGGAAAGTTCTATAGTCC	miR-625-5p
ACCTGGCATACAATGTAGATTT	miR-221-5p
GAGTTCTACAGTCAGAC	miR-3168
ATATAATACAACCTGCTAAGTG	miR-374b-5p
AGCAGCATTGTACAGGGCTATCA	miR-107
CCTCCGTGTTACCTGTCCTCTAG	miR-3605-3p
TTATGGTTTGCCTGGGACTGAG	miR-584-5p
TCCCTGAGACCCTAACTTGTGA	miR-125b-5p
TAACTGGTTGAACAACTGAACC	miR-582-3p
AAGGGCTTCCTCTCTGCAGGAC	miR-3158-3p
CGCATCCCCTAGGGCATTGGTGT	miR-324-5p
ACTGGCCTGGGACTACCGG	miR-3176
GGGGTTCCTGGGGATGGGATTT	miR-23a-5p
CAAGTCACTAGTGGTTCCGTT	miR-224-5p
TGAGTGTGTGTGTGTGAGTGTGT	miR-574-5p
TGAGGAGATCGTCGAGGTTGG	miR-3150b-3p
TAAGGTGCATCTAGTGCAGATAG	miR-18a-5p
GCCTGCTGGGGTGGAACCTGGT	miR-370
AGTGCCTGAGGGAGTAAGAG	miR-550a-3-5p
TCAGAACAAATGCCGGTTCCCAGA	miR-589-3p
TTTGGTCCCCTTCAACCAGCTG	miR-133a
TGTAGAGCAGGGAGCAGGAAGCT	miR-4732-5p
CAGCCCCACAGCCTCAGA	miR-4323
ACTCAAAAGATGGCGGCACTTT	miR-371b-5p
TAGTAGACCGTATAGCGTACG	miR-411-5p
CCTCACCATCCCTTCTGCCTGC	miR-6511a-3p
TCTGGGCAACAAAGTGAGACCT	miR-1285-3p
CTGGGAGAGGGTTGTTTACTCC	miR-30c-1-3p
TCTCTGGGCCTGTGTCTTAGGC	miR-330-5p
AGGGCTTAGCTGCTTGTGAGCA	miR-27a-5p
TGATATGTTTGATATATTAGGT	miR-190a
TCAGCTACTACCTCTATTAGG	miR-5690
AACCCGTAGATCCGAACTTGTG	miR-100-5p
ACTGCCCTAAGTGCTCCTTCTGG	miR-18a-3p
CTGTACAGGCCACTGCCTTGC	let-7g-3p
AATCCTTTGTCCCTGGGTGAGA	miR-501-5p
TGTCTTGCAGGCCGTCATGCA	miR-431-5p
AGGTTGGGATCGGTTGCAATGCT	miR-92a-1-5p
AAAAGCTGGGTTGAGAGGGCAA	miR-320b
GGGAGAAGGGTCGGGGC	miR-4516
TTGCAGCTGCCTGGGAGTGACTTC	miR-1301

AATCCTTGCTACCTGGGT	miR-500b
TTTTTCATTATTGCTCCTGACC	miR-335-3p
CCGGTCCCAGGAGAACCTGCAGA	miR-4746-5p
TTATAATACAACCTGATAAGTG	miR-374a-5p
TTAGGGCCCTGGCTCCATCTCC	miR-1296
ACTGCAGTGAAGGCACTTGTAG	miR-17-3p
AGGGCCCCCCCTCAATCCTGT	miR-296-5p
AACTGGCCCTCAAAGTCCCGCT	miR-193b-3p
TTACAGTTGTTCAACCAGTTACT	miR-582-5p
TGCCTACTGAGCTGAAACACAG	miR-24-2-5p
TGTCTTACTCCCTCAGGCACAT	miR-550a-3p
GAAGTTGTTCGTGGTGGATTCG	miR-382-5p
CTGCGCAAGCTACTGCCTTGCT	let-7i-3p
CGAATCATTATTTGCTGCTCTA	miR-15b-3p
TAATGCCCCTAAAAATCCTTAT	miR-365a-3p
TAATGCCCCTAAAAATCCTTAT	miR-365b-3p
ATCATGATGGGCTCCTCGGTGT	miR-433
CGTGCCACCCTTTTCCCCAG	miR-1227-3p
AATGCACCTGGGCAAGGATTCA	miR-502-3p
AATCGTACAGGGTCATCCACTT	miR-487b
TAATACTGCCTGGTAATGATGA	miR-200b-3p
TCGGGGATCATCATGTCACGAGA	miR-542-5p
TGATATGTTTGATATTGGGTT	miR-190b
AATCTGAGAAGGCGCACAAGGT	miR-3200-5p
CGGGCGTGGTGGTGGGGG	miR-1268a
CATCAGAATTCATGGAGGCTAG	miR-2115-3p
ACCTGGACCCAGCGTAGACAAAG	miR-3690
GACTATAGAACTTTCCCCCTCA	miR-625-3p
TAATCCTTGCTACCTGGGTGAGA	miR-500a-5p
TGTGCAAATCTATGCAAAACTGA	miR-19a-3p

2071-1935 = 136 miRs with at least 10000 reads total over 9

Selected 98 samples X 136 miRNAs	
correlation B_33 v C_2	
-0.12	2071-1935 = 136 miRs
	136 miRs
ALL SELECTED	XX samples
FROZEN	
TOP 136	
z-scores with aveUC, sdUC used	sum <> "" =

counts shown are exact matches + inclusions

each miR ranked by total reads	2071
use 136 miRNAs:	ID
TATTGCACTTGTCCCGGCCTGT	miR-92a-3p
CAACGGAATCCCAAAAGCAGCTG	miR-191-5p
TCTCCCAACCCTTGTACCAGTG	miR-150-5p
TGAGGTAGTAGGTTGTATAGTT	let-7a-5p
AACATTCAACGCTGTCGGTGAGT	miR-181a-5p
TAGCAGCACGTAAATATTGGCG	miR-16-5p
TTCAAGTAATCCAGGATAGGCT	miR-26a-5p
AAACCGTTACCATTACTGAGTT	miR-451a

AAGCTGCCAGTTGAAGAACTGT	miR-22-3p
CATTGCACTTGTCTCGGTCTGA	miR-25-3p
TGAGGTAGTAGTTTGTGCTGTT	let-7i-5p
TGTAAACATCCCCGACTGGAAG	miR-30d-5p
TGAGGTAGTAGTTTGTACAGTT	let-7g-5p
TGAGGTAGTAGGTTGTGTGGTT	let-7b-5p
TGAGGTAGTAGATTGTATAGTT	let-7f-5p
AGCTCGGTCTGAGGCCCCTCAGT	miR-423-3p
TTCAAGTAATTCAGGATAGGT	miR-26b-5p
AGCAGCATTGTACAGGGCTATGA	miR-103a-3p
CAAAGAATTCTCCTTTTGGGCT	miR-186-5p
CACTAGATTGTGAGCTCCTGGA	miR-28-3p
ACTGGACTTGGAGTCAGAAGG	miR-378a-3p
CAAAGTGCTGTTCGTGCAGGTAG	miR-93-5p
TCAGTGCACTACAGAACTTTGT	miR-148a-3p
TGTCAGTTTGTCAAATACCCCA	miR-223-3p
TGAGAACTGAATTCCATAGGCT	miR-146b-5p
TACCACAGGGTAGAACCACGG	miR-140-3p
AAAAGCTGGGTTGAGAGGGCGA	miR-320a
TTTGGCAATGGTAGAACTCACACT	miR-182-5p
TCGAGGAGCTCACAGTCT	miR-151b
TCAGTGCATCACAGAACTTTGT	miR-148b-3p
TGTAAACATCCTACACTCTCAGC	miR-30c-5p
AGAGGTAGTAGGTTGCATAGTT	let-7d-5p
TGTAAACATCCTTGACTGGAAG	miR-30e-5p
TAGCTTATCAGACTGATGTTGA	miR-21-5p
TCGAGGAGCTCACAGTCTAGT	miR-151a-5p
TAGCAGCACATCATGGTTTACA	miR-15b-5p
CTAGACTGAAGCTCCTTGAGG	miR-151a-3p
TGAGAACTGAATTCCATGGGTT	miR-146a-5p
CACCCGGCTGTGTGCACATGTGC	miR-941
TGAGGGGCAGAGAGCGAGACTTT	miR-423-5p
TTCACCACCTTCTCCACCCAGC	miR-197-3p
AACATTCATTGCTGTCGGTGGGT	miR-181b-5p
TCAGGCTCAGTCCCCTCCCGAT	miR-484
CTGACCTATGAATTGACAGCC	miR-192-5p
TCTCACACAGAAATCGCACCCGT	miR-342-3p
AGCTACATCTGGCTACTGGGT	miR-222-3p
TTCACAGTGGCTAAGTTCTGC	miR-27b-3p
TCTCTCGGCTCCTCGCGGCTC	miR-3615
GGATATCATCATATACTGTAAG	miR-144-5p
TGAGATGAAGCACTGTAGCTC	miR-143-3p
TCGGATCCGTCTGAGCTTGGCT	miR-127-3p
ATCACATTGCCAGGGATTTCC	miR-23a-3p
AATGGCGCCACTAGGGTTGTG	miR-652-3p
CTTTCAGTCGGATGTTTACAGC	miR-30e-3p
CAACACCAGTCGATGGGCTGT	miR-21-3p

TACAGTACTGTGATAACTGAA	miR-101-3p
AATGACACGATCACTCCCGTTGA	miR-425-5p
AACATTCAACCTGTCGGTGAGT	miR-181c-5p
TTAATGCTAATCGTGATAGGGGT	miR-155-5p
TCCCTGAGACCCTTTAACCTGTGA	miR-125a-5p
AGCTACATTGTCTGCTGGGTTTC	miR-221-3p
TGAGACCTCTGGGTTCTGAGCT	miR-769-5p
CATTATTACTTTTGGTACGCG	miR-126-5p
CATAAAGTAGAAAGCACTACT	miR-142-5p
GCCCTGACCTGTCCTGTTCTG	miR-4732-3p
CAAAGTGCTTACAGTGCAGGTAG	miR-17-5p
TGTAAACATCCTACACTCAGCT	miR-30b-5p
TAAAGTGCTTATAGTGCAGGTAG	miR-20a-5p
TGGCTCAGTTCAGCAGGAACAG	miR-24-3p
TATGGCACTGGTAGAATTCACT	miR-183-5p
CTATACGACCTGCTGCCTTTCT	let-7d-3p
CGGGGCAGCTCAGTACAGGAT	miR-486-3p
TGAGGTAGTAAGTTGTATTGTT	miR-98-5p
CATGCCTTGAGTGTAGGACCGT	miR-532-5p
TAGCACCATCTGAAATCGGTTA	miR-29a-3p
GAATGTTGCTCGGTGAACCCCT	miR-409-3p
ATGCACCTGGGCAAGGATTCTG	miR-500a-3p
CACCCGTAGAACCGACCTTGCG	miR-99b-5p
TGCGGGGCTAGGGCTAACAGCA	miR-744-5p
ATCACATTGCCAGGGATTACC	miR-23b-3p
AATTGCACGGTATCCATCTGTA	miR-363-3p
ACTCGGCGTGGCGTCGGTCGTG	miR-1307-3p
TATTGCACTCGTCCCGGCCTCC	miR-92b-3p
TTATAAAGCAATGAGACTGATT	miR-340-5p
TTCACAGTGGCTAAGTTCCGC	miR-27a-3p
CCTCTGGGCCCTTCCTCCAG	miR-326
CTGGCCCTCTCTGCCCTTCCGT	miR-328
TCGTACCGTGAGTAATAATGCG	miR-126-3p
TCACAGTGAACCGGTCTCTTT	miR-128
TGAGGTAGGAGGTTGTATAGTT	let-7e-5p
ACCACTGACCGTTGACTGTACC	miR-181a-2-3p
TACAGTATAGATGATGTACT	miR-144-3p
GCTGACTCCTAGTCCAGGGCTC	miR-345-5p
CAGTGCAATGTTAAAAGGGCAT	miR-130a-3p
TAGCAGCACATAATGGTTTGTG	miR-15a-5p
AAGGAGCTCACAGTCTATTGAG	miR-28-5p
TCCGGTTCTCAGGGCTCCACC	miR-671-3p
CCGCACTGTGGGTACTTGCTGC	miR-106b-3p
TGCCCTGTGGACTCAGTTCTGG	miR-146b-3p
TAGTGCAATATTGCTTATAGGGT	miR-454-3p
GCCCCTGGGCCTATCCTAGAA	miR-331-3p
CAGTGCAATGATGAAAGGGCAT	miR-130b-3p
AGGGGTGCTATCTGTGATTGA	miR-342-5p

TACCCTGTAGATCCGAATTTGTG	miR-10a-5p
TGTAACAGCAACTCCATGTGGA	miR-194-5p
TACCCATTGCATATCGGAGTTG	miR-660-5p
ACCCCACTCCTGGTACC	miR-4286
TTATCAGAATCTCCAGGGGTAC	miR-361-5p
TAAAGTGCTGACAGTGCAGAT	miR-106b-5p
TGGAGAGAAAGGCAGTTCCTGA	miR-185-5p
ACCATCGACCGTTGATTGTACC	miR-181a-3p
TCCCCCAGGTGTGATTCTGATTT	miR-361-3p
GTCCAGTTTTCCCAGGAATCCCT	miR-145-5p
CAAAGTGCTCATAGTGCAGGTAG	miR-20b-5p
AGGCAAGATGCTGGCATAGCT	miR-31-5p
TGAGGTAGTAGGTTGTATGGTT	let-7c
TGTAAACATCCTCGACTGGAAG	miR-30a-5p
ACAGTAGTCTGCACATTGGTTA	miR-199a-3p
ACAGTAGTCTGCACATTGGTTA	miR-199b-3p
TATTCATTTATCCCCAGCCTACA	miR-664a-3p
ACTGCCCCAGGTGCTGCTGG	miR-324-3p
TAACAGTCTACAGCCATGGTCG	miR-132-3p
AATATAACACAGATGGCCTGT	miR-410
AAAGTTCTGAGACACTCCGACT	miR-148a-5p
TGTAGTGTTTCCTACTTTATGGA	miR-142-3p
ACTCTTTCCCTGTTGCACTAC	miR-130b-5p
CTGCCCTGGCCCGAGGGACCGA	miR-874
TGAGCGCCTCGACGACAGAGCCG	miR-339-3p
ACTCCAGCCCCACAGCCTCAGC	miR-766-3p
TGGGTCTTTGCGGGCGAGATGA	miR-193a-5p
AATGCACCCGGGCAAGGATTCT	miR-501-3p
CCTGTTCTCCATTACTTGGCTC	miR-26b-3p
CCCAGTGTTCAGACTACCTGTTC	miR-199a-5p
CACGCTCATGCACACACCCACA	miR-574-3p
TCGACCGGACCTCGACCGGCT	miR-1307-5p
AACATTCATTGTTGTCGGTGGGT	miR-181d

Selected XX samples X 136 miRNAs	136 miRs (6 in red)
TRANSPOSE OF TOP 136	95 samples
z-scores with aveUC, sdUC used	0
sum <> "" =	0
97	index type:
0	1
0	2
0	3
0	4
0	6
0	7
0	8
0	9
0	10
0	11
0	12
0	13
0	14
0	15
0	17
0	18
0	19
0	20
0	21
0	22

0	23
0	24
0	25
0	26
0	27
0	28
0	29
0	1
0	2
0	3
0	4
0	5
0	6
0	7
0	8
0	9
0	10
0	11
0	12
0	13
0	14
0	15
0	16
0	17
0	18
0	19
0	20
0	21
0	22
0	23
0	24
0	25
0	26
0	27
0	28
0	29
0	30
0	31
0	32
0	33
0	34
0	35
0	36
0	37
0	38
0	1
0	2
0	3
0	4
0	5
0	6
0	8
0	9
0	10
0	11
0	12
0	13
0	14
0	15

0	16
0	17
0	18
0	19
0	20
0	21
0	22
0	23
0	24
0	25
0	26
0	27
0	28
0	29
0	30
0	31

CORRELATION OF B_33 WITH C_2	136 miRs
0.61	ALL 95 samples

z-scores with aveUC, sdUC used	0
sum <> "" =	0
97	index type:
0	1
0	2
0	3
0	4
0	6
0	7
0	8
0	9
0	10
0	11
0	12
0	13
0	14
0	15
0	17
0	18
0	19
0	20
0	21
0	22
0	23
0	24
0	25
0	26
0	27
0	28
0	29
0	1

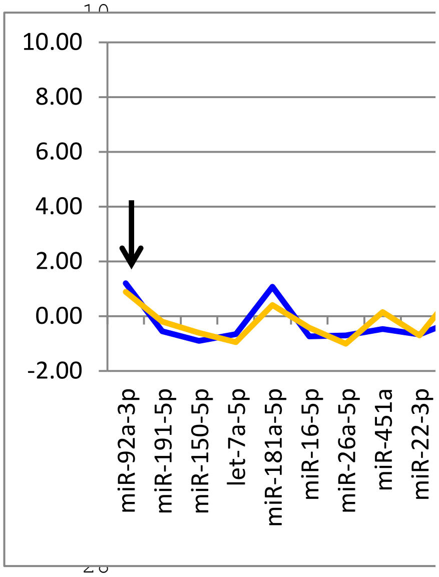

0	2
0	3
0	4
0	5
0	6
0	7
0	8
0	9
0	10
0	[illegible]
0	[illegible]
0	[illegible]
0	[illegible]
0	[illegible]
0	[illegible]
0	[illegible]
0	[illegible]
0	[illegible]
0	[illegible]
0	[illegible]
0	[illegible]
0	[illegible]
0	[illegible]
0	[illegible]
0	[illegible]
0	[illegible]
0	[illegible]
0	29
0	30
0	31
0	32
0	33
0	34
0	35
0	36
0	37
0	38
0	1
0	2
0	3
0	4
0	5
0	6
0	8
0	9
0	10
0	11
0	12
0	13
0	14
0	15
0	16
0	17
0	18
0	19
0	20
0	21
0	22
0	23

0	24
0	25
0	26
0	27
0	28
0	29
0	30
0	31

CORRELATION OF B_33 WITH C_2	136 miRs
0.61	95 samples
corr discard lowest 22 (thus keeping 103a-3p)	
0.80	

z-scores with aveUC, sdUC used	0
sum <> "" =	0
97	index type:
0	1
0	2
0	3
0	4
0	6
0	7
0	8
0	9
0	10
0	11
0	12
0	13
0	14
0	15
0	17
0	18
0	19
0	20
0	21
0	22
0	23
0	24
0	25
0	26
0	27
0	28
0	29
0	1
0	2
0	3
0	4
0	5
0	6
0	7
0	8
0	9
0	[illegible]
0	
0	

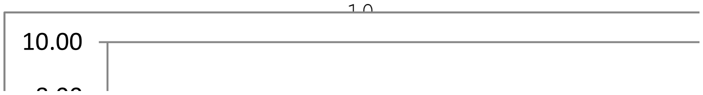

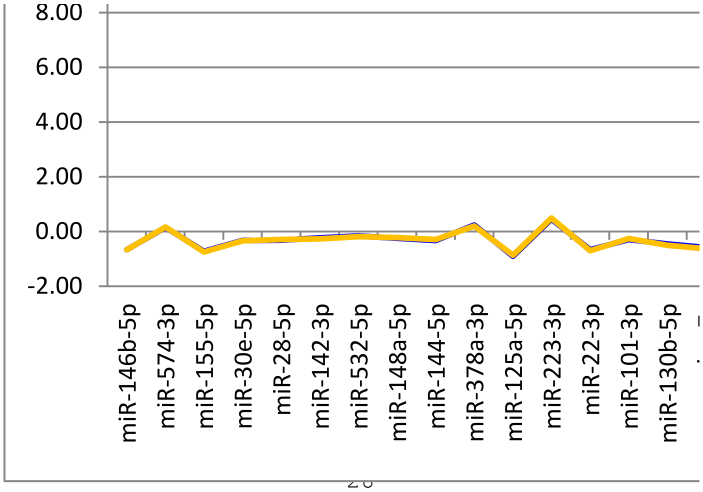

0	
0	
0	
0	
0	
0	
0	
0	
0	
0	
0	
0	
0	
0	
0	
0	[illegible]
0	29
0	30
0	31
0	32
0	33
0	34
0	35
0	36
0	37
0	38
0	1
0	2
0	3
0	4
0	5
0	6
0	8
0	9
0	10
0	11
0	12
0	13
0	14
0	15
0	16
0	17
0	18
0	19
0	20
0	21
0	22
0	23
0	24
0	25
0	26
0	27
0	28
0	29
0	30
0	31

	duplicate correlation
	0.61366
	8836
	park
-0.66	-0.24
-0.63	
-0.63	beta distribution
-0.62	a1=7.4722 a2=28.622 a=-
-0.60	
-0.57	p-value
-0.57	0.01644
-0.56	
-0.55	number of corrs:
-0.54	4465
-0.54	
-0.52	number of corrs exceeding
-0.52	72
-0.52	algebraic p-value
-0.51	0.01635
-0.51	
-0.50	
-0.50	
-0.50	
-0.50	
-0.50	
-0.50	
-0.50	
-0.50	
-0.49	
-0.49	
-0.49	
-0.49	
-0.48	
-0.48	
-0.48	
-0.47	
-0.47	
-0.47	
-0.47	
-0.47	
-0.47	
-0.47	
-0.47	
-0.47	
-0.47	
-0.47	
-0.47	
-0.47	
-0.47	
-0.47	
-0.47	
-0.47	
-0.46	
-0.46	

-0.46
-0.46
-0.46
-0.46
-0.46
-0.46
-0.46
-0.46
-0.46
-0.45
-0.45
-0.45
-0.45
-0.45
-0.45
-0.45
-0.45
-0.45
-0.44
-0.44
-0.44
-0.44
-0.44
-0.44
-0.44
-0.43
-0.43
-0.43
-0.43
-0.43
-0.43
-0.43
-0.43
-0.43
-0.43
-0.43
-0.43
-0.43
-0.43
-0.42
-0.42
-0.42
-0.42
-0.42
-0.42
-0.42
-0.42
-0.42
-0.42
-0.42
-0.42
-0.42
-0.42
-0.41
-0.41
-0.41
-0.41
-0.41
-0.41

-0.41
-0.41
-0.41
-0.41
-0.41
-0.41
-0.41
-0.41
-0.41
-0.41
-0.41
-0.41
-0.41
-0.41
-0.41
-0.41
-0.41
-0.41
-0.41
-0.40
-0.40
-0.40
-0.40
-0.40
-0.40
-0.40
-0.40
-0.40
-0.40
-0.40
-0.40
-0.40
-0.40
-0.40
-0.40
-0.40
-0.40
-0.40
-0.39
-0.39
-0.39
-0.39
-0.39
-0.39
-0.39
-0.39
-0.39
-0.39
-0.39
-0.39
-0.39
-0.38
-0.38
-0.38
-0.38
-0.38
-0.38
-0.38
-0.38

-0.38
-0.38
-0.38
-0.38
-0.38
-0.38
-0.38
-0.38
-0.38
-0.38
-0.38
-0.38
-0.38
-0.37
-0.37
-0.37
-0.37
-0.37
-0.37
-0.37
-0.37
-0.37
-0.37
-0.37
-0.37
-0.37
-0.37
-0.37
-0.37
-0.37
-0.37
-0.37
-0.37
-0.37
-0.37
-0.37
-0.37
-0.37
-0.37
-0.37
-0.37
-0.37
-0.36
-0.36
-0.36
-0.36
-0.36
-0.36
-0.36
-0.36
-0.36
-0.36
-0.36
-0.36
-0.36
-0.36
-0.36
-0.36
-0.36

-0.36
-0.36
-0.36
-0.36
-0.36
-0.36
-0.36
-0.36
-0.36
-0.36
-0.36
-0.36
-0.36
-0.36
-0.36
-0.36
-0.35
-0.35
-0.35
-0.35
-0.35
-0.35
-0.35
-0.35
-0.35
-0.35
-0.35
-0.35
-0.35
-0.35
-0.35
-0.35
-0.35
-0.35
-0.35
-0.35
-0.35
-0.35
-0.35
-0.35
-0.35
-0.35
-0.35
-0.35
-0.34
-0.34
-0.34
-0.34
-0.34
-0.34
-0.34
-0.34
-0.34
-0.34
-0.34
-0.34
-0.34
-0.34
-0.34

-0.34
-0.34
-0.34
-0.34
-0.34
-0.34
-0.34
-0.34
-0.34
-0.34
-0.34
-0.34
-0.34
-0.34
-0.34
-0.34
-0.34
-0.34
-0.34
-0.34
-0.34
-0.34
-0.34
-0.34
-0.34
-0.34
-0.34
-0.34
-0.34
-0.33
-0.33
-0.33
-0.33
-0.33
-0.33
-0.33
-0.33
-0.33
-0.33
-0.33
-0.33
-0.33
-0.33
-0.33
-0.33
-0.33
-0.33
-0.33
-0.33
-0.33
-0.33
-0.33
-0.33
-0.33
-0.33
-0.33
-0.33
-0.33
-0.33

-0.33
-0.33
-0.33
-0.33
-0.33
-0.33
-0.33
-0.33
-0.32
-0.32
-0.32
-0.32
-0.32
-0.32
-0.32
-0.32
-0.32
-0.32
-0.32
-0.32
-0.32
-0.32
-0.32
-0.32
-0.32
-0.32
-0.32
-0.32
-0.32
-0.32
-0.32
-0.32
-0.32
-0.32
-0.32
-0.32
-0.32
-0.32
-0.32
-0.32
-0.32
-0.32
-0.32
-0.31
-0.31
-0.31
-0.31
-0.31
-0.31
-0.31
-0.31
-0.31
-0.31
-0.31
-0.31
-0.31
-0.31
-0.31
-0.31

-0.31
-0.31
-0.31
-0.31
-0.31
-0.31
-0.31
-0.31
-0.31
-0.31
-0.31
-0.31
-0.31
-0.31
-0.31
-0.31
-0.31
-0.31
-0.31
-0.30
-0.30
-0.30
-0.30
-0.30
-0.30
-0.30
-0.30
-0.30
-0.30
-0.30
-0.30
-0.30
-0.30
-0.30
-0.30
-0.30
-0.30
-0.30
-0.30
-0.30
-0.30
-0.30
-0.30
-0.30
-0.30
-0.30
-0.30
-0.30
-0.30
-0.30
-0.30
-0.30
-0.30
-0.30
-0.30
-0.30
-0.30
-0.30
-0.30

-0.30
-0.30
-0.29
-0.29
-0.29
-0.29
-0.29
-0.29
-0.29
-0.29
-0.29
-0.29
-0.29
-0.29
-0.29
-0.29
-0.29
-0.29
-0.29
-0.29
-0.29
-0.29
-0.29
-0.29
-0.29
-0.29
-0.29
-0.29
-0.29
-0.29
-0.29
-0.29
-0.29
-0.29
-0.29
-0.29
-0.29
-0.29
-0.29
-0.29
-0.29
-0.29
-0.29
-0.29
-0.29
-0.29
-0.29
-0.29
-0.28
-0.28
-0.28
-0.28
-0.28
-0.28
-0.28
-0.28
-0.28
-0.28
-0.28

-0.28
-0.28
-0.28
-0.28
-0.28
-0.28
-0.28
-0.28
-0.28
-0.28
-0.28
-0.28
-0.28
-0.28
-0.28
-0.28
-0.28
-0.28
-0.28
-0.28
-0.28
-0.28
-0.28
-0.28
-0.28
-0.28
-0.28
-0.28
-0.28
-0.28
-0.28
-0.28
-0.28
-0.28
-0.28
-0.28
-0.28
-0.28
-0.27
-0.27
-0.27
-0.27
-0.27
-0.27
-0.27
-0.27
-0.27
-0.27
-0.27
-0.27
-0.27
-0.27
-0.27
-0.27
-0.27
-0.27
-0.27
-0.27
-0.27

-0.27
-0.27
-0.27
-0.27
-0.27
-0.27
-0.27
-0.27
-0.27
-0.27
-0.27
-0.27
-0.27
-0.27
-0.27
-0.27
-0.27
-0.27
-0.27
-0.26
-0.26
-0.26
-0.26
-0.26
-0.26
-0.26
-0.26
-0.26
-0.26
-0.26
-0.26
-0.26
-0.26
-0.26
-0.26
-0.26
-0.26
-0.26
-0.26
-0.26
-0.26
-0.26
-0.26
-0.26
-0.26
-0.26
-0.26
-0.26
-0.26
-0.26
-0.26
-0.26
-0.26
-0.26
-0.26
-0.26
-0.26
-0.26

-0.26
-0.26
-0.26
-0.26
-0.26
-0.26
-0.26
-0.26
-0.25
-0.25
-0.25
-0.25
-0.25
-0.25
-0.25
-0.25
-0.25
-0.25
-0.25
-0.25
-0.25
-0.25
-0.25
-0.25
-0.25
-0.25
-0.25
-0.25
-0.25
-0.25
-0.25
-0.25
-0.25
-0.25
-0.25
-0.25
-0.25
-0.25
-0.25
-0.25
-0.25
-0.25
-0.25
-0.25
-0.25
-0.25
-0.25
-0.25
-0.25
-0.25
-0.25
-0.25
-0.25
-0.25
-0.25
-0.25
-0.25
-0.25
-0.25

-0.25
-0.25
-0.25
-0.25
-0.24
-0.24
-0.24
-0.24
-0.24
-0.24
-0.24
-0.24
-0.24
-0.24
-0.24
-0.24
-0.24
-0.24
-0.24
-0.24
-0.24
-0.24
-0.24
-0.24
-0.24
-0.24
-0.24
-0.24
-0.24
-0.24
-0.24
-0.24
-0.24
-0.24
-0.24
-0.24
-0.24
-0.24
-0.24
-0.24
-0.24
-0.24
-0.24
-0.24
-0.24
-0.24
-0.24
-0.24
-0.24
-0.24
-0.24
-0.24
-0.24
-0.24
-0.24
-0.24
-0.24
-0.24
-0.24

-0.24
-0.24
-0.24
-0.24
-0.24
-0.24
-0.24
-0.23
-0.23
-0.23
-0.23
-0.23
-0.23
-0.23
-0.23
-0.23
-0.23
-0.23
-0.23
-0.23
-0.23
-0.23
-0.23
-0.23
-0.23
-0.23
-0.23
-0.23
-0.23
-0.23
-0.23
-0.23
-0.23
-0.23
-0.23
-0.23
-0.23
-0.23
-0.23
-0.23
-0.23
-0.23
-0.23
-0.23
-0.23
-0.23
-0.23
-0.23
-0.23
-0.23
-0.23
-0.23
-0.23
-0.23
-0.23
-0.23
-0.23
-0.22
-0.22

-0.22
-0.22
-0.22
-0.22
-0.22
-0.22
-0.22
-0.22
-0.22
-0.22
-0.22
-0.22
-0.22
-0.22
-0.22
-0.22
-0.22
-0.22
-0.22
-0.22
-0.22
-0.22
-0.22
-0.22
-0.22
-0.22
-0.22
-0.22
-0.22
-0.22
-0.22
-0.22
-0.22
-0.22
-0.22
-0.22
-0.22
-0.22
-0.22
-0.22
-0.22
-0.22
-0.22
-0.22
-0.22
-0.22
-0.22
-0.22
-0.22
-0.22
-0.22
-0.22
-0.21
-0.21
-0.21
-0.21
-0.21
-0.21
-0.21

-0.21
-0.21
-0.21
-0.21
-0.21
-0.21
-0.21
-0.21
-0.21
-0.21
-0.21
-0.21
-0.21
-0.21
-0.21
-0.21
-0.21
-0.21
-0.21
-0.21
-0.21
-0.21
-0.21
-0.21
-0.21
-0.21
-0.21
-0.21
-0.21
-0.21
-0.21
-0.21
-0.21
-0.21
-0.21
-0.21
-0.21
-0.21
-0.21
-0.21
-0.21
-0.21
-0.21
-0.21
-0.21
-0.20
-0.20
-0.20
-0.20
-0.20
-0.20
-0.20
-0.20
-0.20
-0.20
-0.20
-0.20
-0.20
-0.20

-0.20
-0.20
-0.20
-0.20
-0.20
-0.20
-0.20
-0.20
-0.20
-0.20
-0.20
-0.20
-0.20
-0.20
-0.20
-0.20
-0.20
-0.20
-0.20
-0.20
-0.20
-0.20
-0.20
-0.20
-0.20
-0.20
-0.20
-0.20
-0.20
-0.20
-0.20
-0.20
-0.20
-0.20
-0.20
-0.20
-0.20
-0.20
-0.20
-0.20
-0.20
-0.20
-0.20
-0.20
-0.20
-0.20
-0.20
-0.20
-0.19
-0.19
-0.19
-0.19
-0.19
-0.19
-0.19
-0.19
-0.19
-0.19
-0.19

-0.19
-0.19
-0.19
-0.19
-0.19
-0.19
-0.19
-0.19
-0.19
-0.19
-0.19
-0.19
-0.19
-0.19
-0.19
-0.19
-0.19
-0.19
-0.19
-0.19
-0.19
-0.19
-0.19
-0.19
-0.19
-0.19
-0.19
-0.19
-0.19
-0.19
-0.19
-0.19
-0.19
-0.19
-0.19
-0.19
-0.19
-0.19
-0.19
-0.19
-0.19
-0.19
-0.19
-0.19
-0.18
-0.18
-0.18
-0.18
-0.18
-0.18
-0.18
-0.18
-0.18
-0.18
-0.18
-0.18
-0.18
-0.18
-0.18

-0.18
-0.18
-0.18
-0.18
-0.18
-0.18
-0.18
-0.18
-0.18
-0.18
-0.18
-0.18
-0.18
-0.18
-0.18
-0.18
-0.18
-0.18
-0.18
-0.18
-0.18
-0.18
-0.18
-0.18
-0.18
-0.18
-0.18
-0.18
-0.18
-0.18
-0.18
-0.18
-0.18
-0.18
-0.18
-0.18
-0.18
-0.18
-0.18
-0.18
-0.18
-0.18
-0.18
-0.18
-0.18
-0.18
-0.18
-0.18
-0.18
-0.18
-0.17
-0.17
-0.17
-0.17
-0.17
-0.17
-0.17
-0.17
-0.17

-0.17
-0.17
-0.17
-0.17
-0.17
-0.17
-0.17
-0.17
-0.17
-0.17
-0.17
-0.17
-0.17
-0.17
-0.17
-0.17
-0.17
-0.17
-0.17
-0.17
-0.17
-0.17
-0.17
-0.17
-0.17
-0.17
-0.17
-0.17
-0.17
-0.17
-0.17
-0.17
-0.17
-0.17
-0.17
-0.17
-0.17
-0.17
-0.17
-0.17
-0.17
-0.17
-0.17
-0.17
-0.17
-0.17
-0.17
-0.17
-0.17
-0.17
-0.17
-0.17
-0.17
-0.17
-0.17
-0.17
-0.17
-0.16
-0.16

-0.16
-0.16
-0.16
-0.16
-0.16
-0.16
-0.16
-0.16
-0.16
-0.16
-0.16
-0.16
-0.16
-0.16
-0.16
-0.16
-0.16
-0.16
-0.16
-0.16
-0.16
-0.16
-0.16
-0.16
-0.16
-0.16
-0.16
-0.16
-0.16
-0.16
-0.16
-0.16
-0.16
-0.16
-0.16
-0.16
-0.16
-0.16
-0.16
-0.16
-0.16
-0.16
-0.16
-0.16
-0.16
-0.16
-0.16
-0.16
-0.16
-0.16
-0.16
-0.16
-0.16
-0.16
-0.16
-0.16
-0.16
-0.16
-0.16

-0.16
-0.15
-0.15
-0.15
-0.15
-0.15
-0.15
-0.15
-0.15
-0.15
-0.15
-0.15
-0.15
-0.15
-0.15
-0.15
-0.15
-0.15
-0.15
-0.15
-0.15
-0.15
-0.15
-0.15
-0.15
-0.15
-0.15
-0.15
-0.15
-0.15
-0.15
-0.15
-0.15
-0.15
-0.15
-0.15
-0.15
-0.15
-0.15
-0.15
-0.15
-0.15
-0.15
-0.15
-0.15
-0.15
-0.15
-0.15
-0.15
-0.15
-0.15
-0.15
-0.15
-0.15
-0.15
-0.15
-0.15
-0.15
-0.15

-0.15
-0.14
-0.14
-0.14
-0.14
-0.14
-0.14
-0.14
-0.14
-0.14
-0.14
-0.14
-0.14
-0.14
-0.14
-0.14
-0.14
-0.14
-0.14
-0.14
-0.14
-0.14
-0.14
-0.14
-0.14
-0.14
-0.14
-0.14
-0.14
-0.14
-0.14
-0.14
-0.14
-0.14
-0.14
-0.14
-0.14
-0.14
-0.14
-0.14
-0.14
-0.14
-0.14
-0.14
-0.14
-0.14
-0.14
-0.14
-0.14
-0.14
-0.14
-0.14
-0.14
-0.14
-0.14
-0.14
-0.14
-0.14
-0.14

-0.14
-0.14
-0.14
-0.14
-0.14
-0.14
-0.14
-0.14
-0.14
-0.14
-0.14
-0.14
-0.14
-0.14
-0.14
-0.13
-0.13
-0.13
-0.13
-0.13
-0.13
-0.13
-0.13
-0.13
-0.13
-0.13
-0.13
-0.13
-0.13
-0.13
-0.13
-0.13
-0.13
-0.13
-0.13
-0.13
-0.13
-0.13
-0.13
-0.13
-0.13
-0.13
-0.13
-0.13
-0.13
-0.13
-0.13
-0.13
-0.13
-0.13
-0.13
-0.13
-0.13
-0.13
-0.13
-0.13
-0.13
-0.13
-0.13

-0.13
-0.13
-0.13
-0.13
-0.13
-0.13
-0.13
-0.13
-0.13
-0.13
-0.13
-0.13
-0.13
-0.13
-0.13
-0.13
-0.13
-0.13
-0.13
-0.13
-0.13
-0.13
-0.13
-0.13
-0.13
-0.13
-0.13
-0.13
-0.13
-0.13
-0.13
-0.13
-0.13
-0.13
-0.13
-0.13
-0.13
-0.13
-0.13
-0.13
-0.13
-0.13
-0.13
-0.12
-0.12
-0.12
-0.12
-0.12
-0.12
-0.12
-0.12
-0.12
-0.12
-0.12
-0.12
-0.12
-0.12
-0.12
-0.12

-0.12
-0.12
-0.12
-0.12
-0.12
-0.12
-0.12
-0.12
-0.12
-0.12
-0.12
-0.12
-0.12
-0.12
-0.12
-0.12
-0.12
-0.12
-0.12
-0.12
-0.12
-0.12
-0.12
-0.12
-0.12
-0.12
-0.12
-0.12
-0.12
-0.12
-0.12
-0.12
-0.12
-0.12
-0.12
-0.11
-0.11
-0.11
-0.11
-0.11
-0.11
-0.11
-0.11
-0.11
-0.11
-0.11
-0.11
-0.11
-0.11
-0.11
-0.11
-0.11
-0.11
-0.11
-0.11
-0.11
-0.11
-0.11
-0.11

-0.11
-0.11
-0.11
-0.11
-0.11
-0.11
-0.11
-0.11
-0.11
-0.11
-0.11
-0.11
-0.11
-0.11
-0.11
-0.11
-0.11
-0.11
-0.11
-0.11
-0.11
-0.11
-0.11
-0.11
-0.11
-0.11
-0.11
-0.11
-0.11
-0.11
-0.11
-0.11
-0.11
-0.11
-0.11
-0.11
-0.11
-0.11
-0.11
-0.11
-0.11
-0.11
-0.11
-0.11
-0.11
-0.10
-0.10
-0.10
-0.10
-0.10
-0.10
-0.10
-0.10
-0.10
-0.10
-0.10
-0.10
-0.10
-0.10

-0.10
-0.10
-0.10
-0.10
-0.10
-0.10
-0.10
-0.10
-0.10
-0.10
-0.10
-0.10
-0.10
-0.10
-0.10
-0.10
-0.10
-0.10
-0.10
-0.10
-0.10
-0.10
-0.10
-0.10
-0.10
-0.10
-0.10
-0.10
-0.10
-0.10
-0.10
-0.10
-0.10
-0.10
-0.10
-0.10
-0.10
-0.10
-0.10
-0.10
-0.10
-0.10
-0.10
-0.10
-0.10
-0.10
-0.10
-0.10
-0.10
-0.10
-0.10
-0.10
-0.09
-0.09
-0.09
-0.09
-0.09
-0.09
-0.09

-0.09
-0.09
-0.09
-0.09
-0.09
-0.09
-0.09
-0.09
-0.09
-0.09
-0.09
-0.09
-0.09
-0.09
-0.09
-0.09
-0.09
-0.09
-0.09
-0.09
-0.09
-0.09
-0.09
-0.09
-0.09
-0.09
-0.09
-0.09
-0.09
-0.09
-0.09
-0.09
-0.09
-0.09
-0.09
-0.09
-0.09
-0.09
-0.09
-0.09
-0.09
-0.09
-0.09
-0.09
-0.09
-0.09
-0.09
-0.09
-0.09
-0.09
-0.09
-0.09
-0.08
-0.08
-0.08
-0.08
-0.08
-0.08
-0.08

-0.08
-0.08
-0.08
-0.08
-0.08
-0.08
-0.08
-0.08
-0.08
-0.08
-0.08
-0.08
-0.08
-0.08
-0.08
-0.08
-0.08
-0.08
-0.08
-0.08
-0.08
-0.08
-0.08
-0.08
-0.08
-0.08
-0.08
-0.08
-0.08
-0.08
-0.08
-0.08
-0.08
-0.08
-0.08
-0.08
-0.08
-0.08
-0.08
-0.08
-0.08
-0.08
-0.08
-0.08
-0.08
-0.08
-0.08
-0.08
-0.08
-0.08
-0.08
-0.08
-0.08
-0.07
-0.07
-0.07
-0.07
-0.07
-0.07

-0.07
-0.07
-0.07
-0.07
-0.07
-0.07
-0.07
-0.07
-0.07
-0.07
-0.07
-0.07
-0.07
-0.07
-0.07
-0.07
-0.07
-0.07
-0.07
-0.07
-0.07
-0.07
-0.07
-0.07
-0.07
-0.07
-0.07
-0.07
-0.07
-0.07
-0.07
-0.07
-0.07
-0.07
-0.07
-0.07
-0.07
-0.07
-0.07
-0.07
-0.07
-0.07
-0.07
-0.07
-0.07
-0.07
-0.07
-0.07
-0.07
-0.07
-0.07
-0.07
-0.07
-0.07
-0.07
-0.07
-0.07
-0.07
-0.07

-0.07
-0.07
-0.06
-0.06
-0.06
-0.06
-0.06
-0.06
-0.06
-0.06
-0.06
-0.06
-0.06
-0.06
-0.06
-0.06
-0.06
-0.06
-0.06
-0.06
-0.06
-0.06
-0.06
-0.06
-0.06
-0.06
-0.06
-0.06
-0.06
-0.06
-0.06
-0.06
-0.06
-0.06
-0.06
-0.06
-0.06
-0.06
-0.06
-0.06
-0.06
-0.06
-0.06
-0.06
-0.06
-0.06
-0.06
-0.06
-0.06
-0.06
-0.06
-0.06
-0.06
-0.06
-0.06
-0.06
-0.06
-0.06
-0.06

-0.06
-0.06
-0.06
-0.06
-0.06
-0.06
-0.06
-0.06
-0.06
-0.06
-0.06
-0.06
-0.06
-0.06
-0.06
-0.06
-0.06
-0.06
-0.06
-0.06
-0.06
-0.06
-0.06
-0.05
-0.05
-0.05
-0.05
-0.05
-0.05
-0.05
-0.05
-0.05
-0.05
-0.05
-0.05
-0.05
-0.05
-0.05
-0.05
-0.05
-0.05
-0.05
-0.05
-0.05
-0.05
-0.05
-0.05
-0.05
-0.05
-0.05
-0.05
-0.05
-0.05
-0.05
-0.05
-0.05
-0.05
-0.05
-0.05

-0.05
-0.05
-0.05
-0.05
-0.05
-0.05
-0.05
-0.05
-0.05
-0.05
-0.05
-0.05
-0.05
-0.05
-0.05
-0.05
-0.05
-0.05
-0.05
-0.05
-0.05
-0.05
-0.05
-0.05
-0.05
-0.05
-0.05
-0.05
-0.05
-0.04
-0.04
-0.04
-0.04
-0.04
-0.04
-0.04
-0.04
-0.04
-0.04
-0.04
-0.04
-0.04
-0.04
-0.04
-0.04
-0.04
-0.04
-0.04
-0.04
-0.04
-0.04
-0.04
-0.04
-0.04
-0.04
-0.04
-0.04
-0.04
-0.04

-0.04
-0.04
-0.04
-0.04
-0.04
-0.04
-0.04
-0.04
-0.04
-0.04
-0.04
-0.04
-0.04
-0.04
-0.04
-0.04
-0.04
-0.04
-0.04
-0.04
-0.04
-0.04
-0.04
-0.04
-0.04
-0.04
-0.04
-0.04
-0.04
-0.04
-0.04
-0.04
-0.04
-0.04
-0.04
-0.04
-0.04
-0.04
-0.04
-0.04
-0.04
-0.04
-0.03
-0.03
-0.03
-0.03
-0.03
-0.03
-0.03
-0.03
-0.03
-0.03
-0.03
-0.03
-0.03
-0.03
-0.03
-0.03
-0.03

-0.03
-0.03
-0.03
-0.03
-0.03
-0.03
-0.03
-0.03
-0.03
-0.03
-0.03
-0.03
-0.03
-0.03
-0.03
-0.03
-0.03
-0.03
-0.03
-0.03
-0.03
-0.03
-0.03
-0.03
-0.03
-0.03
-0.03
-0.03
-0.03
-0.03
-0.03
-0.03
-0.03
-0.03
-0.03
-0.03
-0.03
-0.03
-0.03
-0.03
-0.03
-0.03
-0.03
-0.03
-0.03
-0.03
-0.03
-0.03
-0.03
-0.03
-0.03
-0.03
-0.03
-0.02
-0.02
-0.02
-0.02
-0.02
-0.02

-0.02
-0.02
-0.02
-0.02
-0.02
-0.02
-0.02
-0.02
-0.02
-0.02
-0.02
-0.02
-0.02
-0.02
-0.02
-0.02
-0.02
-0.02
-0.02
-0.02
-0.02
-0.02
-0.02
-0.02
-0.02
-0.02
-0.02
-0.02
-0.02
-0.02
-0.02
-0.02
-0.02
-0.02
-0.02
-0.02
-0.02
-0.02
-0.02
-0.02
-0.02
-0.02
-0.02
-0.02
-0.02
-0.02
-0.02
-0.02
-0.02
-0.02
-0.02
-0.02
-0.02
-0.02
-0.02
-0.02
-0.02
-0.02
-0.02

-0.01
-0.01
-0.01
-0.01
-0.01
-0.01
-0.01
-0.01
-0.01
-0.01
-0.01
-0.01
-0.01
-0.01
-0.01
-0.01
-0.01
-0.01
-0.01
-0.01
-0.01
-0.01
-0.01
-0.01
-0.01
-0.01
-0.01
-0.01
-0.01
-0.01
-0.01
-0.01
-0.01
-0.01
-0.01
-0.01
-0.01
-0.01
-0.01
-0.01
-0.01
-0.01
-0.01
-0.01
-0.01
-0.01
-0.01
-0.01
-0.01
-0.01
-0.01
-0.01
-0.01
-0.01
-0.01
-0.01
-0.01
-0.01
-0.01

-0.01
-0.01
-0.01
-0.01
-0.01
-0.01
-0.01
-0.01
-0.01
-0.01
-0.01
-0.01
-0.01
0.00
0.00
0.00
0.00
0.00
0.00
0.00
0.00
0.00
0.00
0.00
0.00
0.00
0.00
0.00
0.00
0.00
0.00
0.00
0.00
0.00
0.00
0.00
0.00
0.00
0.00
0.00
0.00
0.00
0.00
0.00
0.00
0.00
0.00
0.00
0.00
0.00
0.00
0.00
0.00
0.00
0.00
0.00
0.00
0.00
0.00

0.00
0.00
0.00
0.00
0.00
0.00
0.00
0.00
0.00
0.00
0.00
0.00
0.00
0.00
0.00
0.00
0.00
0.00
0.00
0.00
0.00
0.00
0.00
0.00
0.00
0.00
0.00
0.01
0.01
0.01
0.01
0.01
0.01
0.01
0.01
0.01
0.01
0.01
0.01
0.01
0.01
0.01
0.01
0.01
0.01
0.01
0.01
0.01
0.01
0.01
0.01
0.01
0.01
0.01
0.01
0.01
0.01
0.01
0.01

0.01
0.01
0.01
0.01
0.01
0.01
0.01
0.01
0.01
0.01
0.01
0.01
0.01
0.01
0.01
0.01
0.01
0.01
0.01
0.01
0.01
0.01
0.01
0.01
0.01
0.01
0.01
0.01
0.01
0.01
0.01
0.01
0.01
0.01
0.01
0.01
0.01
0.01
0.01
0.01
0.01
0.01
0.01
0.01
0.01
0.01
0.01
0.01
0.01
0.02
0.02
0.02
0.02
0.02
0.02
0.02
0.02
0.02
0.02

0.02
0.02
0.02
0.02
0.02
0.02
0.02
0.02
0.02
0.02
0.02
0.02
0.02
0.02
0.02
0.02
0.02
0.02
0.02
0.02
0.02
0.02
0.02
0.02
0.02
0.02
0.02
0.02
0.02
0.02
0.02
0.02
0.02
0.02
0.02
0.02
0.02
0.02
0.02
0.02
0.02
0.02
0.02
0.02
0.02
0.02
0.02
0.02
0.02
0.03
0.03
0.03
0.03
0.03
0.03
0.03
0.03
0.03
0.03

0.03
0.03
0.03
0.03
0.03
0.03
0.03
0.03
0.03
0.03
0.03
0.03
0.03
0.03
0.03
0.03
0.03
0.03
0.03
0.03
0.03
0.03
0.03
0.03
0.03
0.03
0.03
0.03
0.03
0.03
0.03
0.03
0.03
0.03
0.03
0.03
0.03
0.03
0.03
0.03
0.03
0.03
0.03
0.03
0.03
0.03
0.03
0.03
0.03
0.03
0.03
0.03
0.03
0.03
0.03
0.03
0.03
0.03
0.03

0.03
0.03
0.03
0.04
0.04
0.04
0.04
0.04
0.04
0.04
0.04
0.04
0.04
0.04
0.04
0.04
0.04
0.04
0.04
0.04
0.04
0.04
0.04
0.04
0.04
0.04
0.04
0.04
0.04
0.04
0.04
0.04
0.04
0.04
0.04
0.04
0.04
0.04
0.04
0.04
0.04
0.04
0.04
0.04
0.04
0.04
0.04
0.04
0.04
0.04
0.04
0.04
0.04
0.04
0.04
0.04
0.04
0.04
0.04

0.04
0.04
0.04
0.04
0.04
0.04
0.04
0.04
0.05
0.05
0.05
0.05
0.05
0.05
0.05
0.05
0.05
0.05
0.05
0.05
0.05
0.05
0.05
0.05
0.05
0.05
0.05
0.05
0.05
0.05
0.05
0.05
0.05
0.05
0.05
0.05
0.05
0.05
0.05
0.05
0.05
0.05
0.05
0.05
0.05
0.05
0.05
0.05
0.05
0.05
0.05
0.05
0.05
0.05
0.05
0.05
0.05
0.05
0.05

0.05
0.05
0.05
0.05
0.05
0.05
0.05
0.05
0.05
0.05
0.05
0.05
0.05
0.06
0.06
0.06
0.06
0.06
0.06
0.06
0.06
0.06
0.06
0.06
0.06
0.06
0.06
0.06
0.06
0.06
0.06
0.06
0.06
0.06
0.06
0.06
0.06
0.06
0.06
0.06
0.06
0.06
0.06
0.06
0.06
0.06
0.06
0.06
0.06
0.06
0.06
0.06
0.06
0.06
0.06
0.06
0.06
0.06
0.06

0.06
0.06
0.06
0.06
0.06
0.06
0.06
0.06
0.07
0.07
0.07
0.07
0.07
0.07
0.07
0.07
0.07
0.07
0.07
0.07
0.07
0.07
0.07
0.07
0.07
0.07
0.07
0.07
0.07
0.07
0.07
0.07
0.07
0.07
0.07
0.07
0.07
0.07
0.07
0.07
0.07
0.07
0.07
0.07
0.07
0.07
0.07
0.07
0.07
0.07
0.07
0.07
0.07
0.07
0.07
0.07
0.07
0.07
0.07

0.07
0.07
0.07
0.07
0.07
0.07
0.07
0.07
0.07
0.07
0.07
0.07
0.07
0.07
0.07
0.07
0.07
0.07
0.07
0.07
0.07
0.07
0.07
0.07
0.07
0.08
0.08
0.08
0.08
0.08
0.08
0.08
0.08
0.08
0.08
0.08
0.08
0.08
0.08
0.08
0.08
0.08
0.08
0.08
0.08
0.08
0.08
0.08
0.08
0.08
0.08
0.08
0.08
0.08
0.08
0.08
0.08
0.08
0.08

0.08
0.08
0.08
0.08
0.08
0.08
0.08
0.08
0.08
0.08
0.08
0.08
0.08
0.08
0.08
0.08
0.08
0.08
0.08
0.08
0.08
0.08
0.08
0.08
0.08
0.08
0.08
0.08
0.08
0.08
0.09
0.09
0.09
0.09
0.09
0.09
0.09
0.09
0.09
0.09
0.09
0.09
0.09
0.09
0.09
0.09
0.09
0.09
0.09
0.09
0.09
0.09
0.09
0.09
0.09
0.09
0.09
0.09
0.09

0.09
0.09
0.09
0.09
0.09
0.09
0.09
0.09
0.09
0.09
0.09
0.09
0.09
0.09
0.09
0.09
0.09
0.09
0.09
0.09
0.09
0.09
0.09
0.09
0.09
0.09
0.09
0.09
0.09
0.09
0.09
0.09
0.09
0.09
0.09
0.10
0.10
0.10
0.10
0.10
0.10
0.10
0.10
0.10
0.10
0.10
0.10
0.10
0.10
0.10
0.10
0.10
0.10
0.10
0.10
0.10
0.10
0.10
0.10

0.10
0.10
0.10
0.10
0.10
0.10
0.10
0.10
0.10
0.10
0.10
0.10
0.10
0.10
0.10
0.10
0.10
0.10
0.10
0.10
0.10
0.10
0.10
0.10
0.10
0.10
0.10
0.11
0.11
0.11
0.11
0.11
0.11
0.11
0.11
0.11
0.11
0.11
0.11
0.11
0.11
0.11
0.11
0.11
0.11
0.11
0.11
0.11
0.11
0.11
0.11
0.11
0.11
0.11
0.11
0.11
0.11
0.11
0.11

0.11
0.11
0.11
0.11
0.11
0.11
0.11
0.11
0.11
0.11
0.11
0.11
0.11
0.11
0.11
0.11
0.11
0.11
0.11
0.11
0.11
0.11
0.11
0.11
0.11
0.11
0.11
0.11
0.11
0.12
0.12
0.12
0.12
0.12
0.12
0.12
0.12
0.12
0.12
0.12
0.12
0.12
0.12
0.12
0.12
0.12
0.12
0.12
0.12
0.12
0.12
0.12
0.12
0.12
0.12
0.12
0.12
0.12
0.12

0.12
0.12
0.12
0.12
0.12
0.12
0.12
0.12
0.12
0.12
0.12
0.12
0.12
0.12
0.12
0.12
0.12
0.13
0.13
0.13
0.13
0.13
0.13
0.13
0.13
0.13
0.13
0.13
0.13
0.13
0.13
0.13
0.13
0.13
0.13
0.13
0.13
0.13
0.13
0.13
0.13
0.13
0.13
0.13
0.13
0.13
0.13
0.13
0.13
0.13
0.13
0.13
0.13
0.13
0.13
0.13
0.13
0.13
0.13

0.13
0.14
0.14
0.14
0.14
0.14
0.14
0.14
0.14
0.14
0.14
0.14
0.14
0.14
0.14
0.14
0.14
0.14
0.14
0.14
0.14
0.14
0.14
0.14
0.14
0.14
0.14
0.14
0.14
0.14
0.14
0.14
0.14
0.14
0.14
0.14
0.14
0.14
0.14
0.14
0.14
0.14
0.14
0.14
0.14
0.14
0.14
0.14
0.14
0.14
0.14
0.14
0.14
0.14
0.14
0.14
0.14
0.14
0.14

0.15
0.15
0.15
0.15
0.15
0.15
0.15
0.15
0.15
0.15
0.15
0.15
0.15
0.15
0.15
0.15
0.15
0.15
0.15
0.15
0.15
0.15
0.15
0.15
0.15
0.15
0.15
0.15
0.15
0.15
0.15
0.15
0.15
0.15
0.15
0.15
0.15
0.15
0.15
0.15
0.15
0.15
0.15
0.15
0.15
0.15
0.15
0.15
0.15
0.15
0.15
0.15
0.15
0.15
0.15
0.15
0.15
0.15
0.16

0.16
0.16
0.16
0.16
0.16
0.16
0.16
0.16
0.16
0.16
0.16
0.16
0.16
0.16
0.16
0.16
0.16
0.16
0.16
0.16
0.16
0.16
0.16
0.16
0.16
0.16
0.16
0.16
0.16
0.16
0.16
0.16
0.16
0.16
0.16
0.16
0.16
0.16
0.16
0.16
0.16
0.16
0.16
0.16
0.16
0.16
0.16
0.16
0.16
0.16
0.16
0.16
0.16
0.16
0.16
0.16
0.16
0.16
0.16

0.16
0.16
0.16
0.16
0.16
0.16
0.16
0.17
0.17
0.17
0.17
0.17
0.17
0.17
0.17
0.17
0.17
0.17
0.17
0.17
0.17
0.17
0.17
0.17
0.17
0.17
0.17
0.17
0.17
0.17
0.17
0.17
0.17
0.17
0.17
0.17
0.17
0.17
0.17
0.17
0.17
0.17
0.17
0.17
0.17
0.17
0.17
0.17
0.17
0.17
0.17
0.17
0.17
0.17
0.17
0.17
0.17
0.18
0.18

0.18
0.18
0.18
0.18
0.18
0.18
0.18
0.18
0.18
0.18
0.18
0.18
0.18
0.18
0.18
0.18
0.18
0.18
0.18
0.18
0.18
0.18
0.18
0.18
0.18
0.18
0.18
0.18
0.18
0.18
0.18
0.18
0.18
0.18
0.18
0.18
0.18
0.18
0.18
0.18
0.18
0.18
0.18
0.18
0.18
0.18
0.18
0.18
0.18
0.19
0.19
0.19
0.19
0.19
0.19
0.19
0.19
0.19
0.19

0.19
0.19
0.19
0.19
0.19
0.19
0.19
0.19
0.19
0.19
0.19
0.19
0.19
0.19
0.19
0.19
0.19
0.19
0.19
0.19
0.19
0.19
0.19
0.19
0.19
0.19
0.19
0.19
0.19
0.19
0.19
0.19
0.19
0.19
0.19
0.19
0.19
0.19
0.19
0.19
0.19
0.20
0.20
0.20
0.20
0.20
0.20
0.20
0.20
0.20
0.20
0.20
0.20
0.20
0.20
0.20
0.20
0.20
0.20

0.20
0.20
0.20
0.20
0.20
0.20
0.20
0.20
0.20
0.20
0.20
0.20
0.20
0.20
0.20
0.20
0.20
0.20
0.20
0.20
0.20
0.20
0.20
0.20
0.20
0.20
0.20
0.20
0.20
0.20
0.20
0.20
0.20
0.20
0.20
0.20
0.20
0.20
0.20
0.20
0.20
0.20
0.21
0.21
0.21
0.21
0.21
0.21
0.21
0.21
0.21
0.21
0.21
0.21
0.21
0.21
0.21
0.21
0.21

0.21
0.21
0.21
0.21
0.21
0.21
0.21
0.21
0.21
0.21
0.21
0.21
0.21
0.21
0.21
0.21
0.21
0.21
0.21
0.21
0.21
0.21
0.21
0.21
0.21
0.21
0.21
0.21
0.21
0.21
0.21
0.21
0.21
0.21
0.22
0.22
0.22
0.22
0.22
0.22
0.22
0.22
0.22
0.22
0.22
0.22
0.22
0.22
0.22
0.22
0.22
0.22
0.22
0.22
0.22
0.22
0.22
0.22
0.22

0.22
0.22
0.22
0.22
0.22
0.22
0.22
0.22
0.22
0.22
0.22
0.22
0.22
0.23
0.23
0.23
0.23
0.23
0.23
0.23
0.23
0.23
0.23
0.23
0.23
0.23
0.23
0.23
0.23
0.23
0.23
0.23
0.23
0.23
0.23
0.23
0.23
0.23
0.23
0.23
0.23
0.23
0.23
0.23
0.23
0.23
0.23
0.23
0.23
0.23
0.23
0.23
0.23
0.23
0.23
0.23
0.23
0.23
0.24

0.24
0.24
0.24
0.24
0.24
0.24
0.24
0.24
0.24
0.24
0.24
0.24
0.24
0.24
0.24
0.24
0.24
0.24
0.24
0.24
0.24
0.24
0.24
0.24
0.24
0.24
0.24
0.24
0.24
0.24
0.24
0.24
0.24
0.24
0.24
0.24
0.24
0.24
0.24
0.25
0.25
0.25
0.25
0.25
0.25
0.25
0.25
0.25
0.25
0.25
0.25
0.25
0.25
0.25
0.25
0.25
0.25
0.25
0.25

0.25
0.25
0.25
0.25
0.25
0.25
0.25
0.25
0.25
0.25
0.26
0.26
0.26
0.26
0.26
0.26
0.26
0.26
0.26
0.26
0.26
0.26
0.26
0.26
0.26
0.26
0.26
0.26
0.26
0.26
0.26
0.26
0.26
0.26
0.26
0.26
0.26
0.26
0.26
0.26
0.26
0.26
0.26
0.26
0.26
0.27
0.27
0.27
0.27
0.27
0.27
0.27
0.27
0.27
0.27
0.27
0.27
0.27
0.27

0.27
0.27
0.27
0.27
0.27
0.27
0.27
0.27
0.27
0.27
0.27
0.27
0.27
0.27
0.27
0.27
0.27
0.27
0.27
0.27
0.27
0.27
0.27
0.27
0.27
0.27
0.27
0.27
0.27
0.27
0.27
0.27
0.28
0.28
0.28
0.28
0.28
0.28
0.28
0.28
0.28
0.28
0.28
0.28
0.28
0.28
0.28
0.28
0.28
0.28
0.28
0.28
0.28
0.28
0.28
0.28
0.28
0.28
0.28

0.28
0.28
0.29
0.29
0.29
0.29
0.29
0.29
0.29
0.29
0.29
0.29
0.29
0.29
0.29
0.29
0.29
0.29
0.29
0.29
0.29
0.29
0.29
0.29
0.29
0.29
0.29
0.29
0.30
0.30
0.30
0.30
0.30
0.30
0.30
0.30
0.30
0.30
0.30
0.30
0.30
0.30
0.30
0.30
0.30
0.30
0.30
0.30
0.30
0.30
0.30
0.30
0.30
0.30
0.30
0.30
0.30
0.30
0.30

0.30
0.30
0.30
0.30
0.30
0.30
0.31
0.31
0.31
0.31
0.31
0.31
0.31
0.31
0.31
0.31
0.31
0.31
0.31
0.31
0.31
0.31
0.31
0.31
0.31
0.31
0.31
0.31
0.31
0.31
0.31
0.31
0.31
0.31
0.31
0.31
0.32
0.32
0.32
0.32
0.32
0.32
0.32
0.32
0.32
0.32
0.32
0.32
0.32
0.32
0.32
0.32
0.32
0.32
0.32
0.32
0.32
0.32
0.32

0.32
0.32
0.32
0.32
0.32
0.33
0.33
0.33
0.33
0.33
0.33
0.33
0.33
0.33
0.33
0.33
0.33
0.33
0.33
0.33
0.33
0.33
0.33
0.33
0.33
0.33
0.33
0.33
0.33
0.33
0.33
0.33
0.33
0.33
0.33
0.34
0.34
0.34
0.34
0.34
0.34
0.34
0.34
0.34
0.34
0.34
0.34
0.34
0.34
0.34
0.34
0.34
0.34
0.34
0.34
0.34
0.34
0.34
0.35

0.35
0.35
0.35
0.35
0.35
0.35
0.35
0.35
0.35
0.35
0.35
0.35
0.35
0.35
0.35
0.35
0.35
0.35
0.35
0.35
0.35
0.35
0.35
0.35
0.35
0.35
0.36
0.36
0.36
0.36
0.36
0.36
0.36
0.36
0.36
0.36
0.36
0.36
0.36
0.36
0.36
0.36
0.36
0.36
0.36
0.36
0.36
0.36
0.36
0.37
0.37
0.37
0.37
0.37
0.37
0.37
0.37
0.37
0.37

0.37
0.37
0.37
0.37
0.37
0.37
0.37
0.37
0.37
0.37
0.37
0.37
0.38
0.38
0.38
0.38
0.38
0.38
0.38
0.38
0.38
0.38
0.38
0.38
0.38
0.38
0.38
0.38
0.38
0.38
0.38
0.38
0.38
0.38
0.38
0.38
0.38
0.39
0.39
0.39
0.39
0.39
0.39
0.39
0.39
0.39
0.39
0.39
0.39
0.39
0.39
0.39
0.39
0.39
0.39
0.39
0.39
0.39
0.39

0.39
0.39
0.39
0.39
0.40
0.40
0.40
0.40
0.40
0.40
0.40
0.40
0.40
0.40
0.40
0.40
0.40
0.40
0.40
0.40
0.40
0.40
0.40
0.40
0.41
0.41
0.41
0.41
0.41
0.41
0.41
0.41
0.41
0.41
0.41
0.41
0.41
0.41
0.41
0.41
0.41
0.41
0.41
0.41
0.41
0.41
0.41
0.41
0.41
0.42
0.42
0.42
0.42
0.42
0.42
0.42
0.42
0.42
0.42

0.42
0.42
0.42
0.42
0.42
0.42
0.42
0.42
0.42
0.43
0.43
0.43
0.43
0.43
0.43
0.43
0.43
0.43
0.43
0.43
0.43
0.43
0.43
0.43
0.43
0.43
0.44
0.44
0.44
0.44
0.44
0.44
0.44
0.44
0.44
0.44
0.44
0.44
0.44
0.44
0.44
0.44
0.44
0.44
0.44
0.44
0.44
0.44
0.44
0.45
0.45
0.45
0.45
0.45
0.45
0.45
0.45
0.45
0.45

0.45
0.45
0.45
0.46
0.46
0.46
0.46
0.46
0.46
0.46
0.46
0.46
0.46
0.46
0.46
0.46
0.46
0.46
0.46
0.46
0.46
0.46
0.46
0.46
0.46
0.46
0.47
0.47
0.47
0.47
0.47
0.47
0.47
0.47
0.47
0.47
0.47
0.48
0.48
0.48
0.48
0.48
0.48
0.48
0.48
0.48
0.48
0.49
0.49
0.49
0.49
0.49
0.49
0.49
0.49
0.49
0.49
0.50
0.50

0.50
0.50
0.50
0.50
0.50
0.50
0.50
0.50
0.50
0.51
0.51
0.51
0.51
0.51
0.51
0.51
0.52
0.52
0.52
0.52
0.52
0.52
0.52
0.52
0.52
0.52
0.52
0.52
0.53
0.53
0.53
0.53
0.53
0.53
0.53
0.54
0.54
0.54
0.54
0.54
0.54
0.54
0.54
0.54
0.54
0.54
0.55
0.55
0.55
0.55
0.55
0.55
0.55
0.55
0.55
0.55
0.56
0.56
0.56

0.56
0.56
0.56
0.56
0.56
0.56
0.56
0.56
0.56
0.57
0.57
0.57
0.57
0.57
0.57
0.57
0.58
0.58
0.58
0.58
0.58
0.58
0.59
0.59
0.59
0.59
0.59
0.60
0.60
0.60
0.61
0.61
0.61
0.61
0.61
0.61
0.61
0.61
0.62
0.62
0.63
0.63
0.63
0.64
0.64
0.64
0.64
0.64
0.64
0.64
0.64
0.65
0.65
0.65
0.65
0.65
0.65
0.66
0.66

0.66
0.66
0.67
0.67
0.67
0.67
0.69
0.69
0.69
0.69
0.70
0.70
0.70
0.71
0.72
0.74
0.74
0.75
0.76
0.76
0.77
0.77
0.78
0.78
0.78
0.79
0.80
0.80
0.81
0.82
0.82
0.82
0.83
0.83
0.83
0.83
0.85
0.86
0.86
0.86
0.87
0.87
0.87
0.88
0.89
0.89
0.92
0.93
0.96

20000	479320	21319	1102300
6			
omitted if all but 486-5p < C1	150000		
omitted if grand total < D3			
6			
6 omitted: A_7, D_15, F_2, C_5, A_8, A_9			
index all samples:	1	2	3
index type:	1	2	3
total of totals of =100:	A_11	A_16	A_2
sample size limit in C557	UC	UC	UC
0.62	1651246	515917	5577762
rand labels	0.61	0.66	0.30
total all samples >=100:	A_11	A_16	A_2
223271318	1171926	494598	4475462
46404581	253154	2382	397359
10498404	9303	9512	44303
8999274	18873	920	14220
8841680	25490	1159	106715
8662078	29662	380	50300
7941012	3705	835	68855
5954165	6768	639	39890
3763460	986	127	13562
3462919	5884	74	50176
3417096	17952	549	60322
2188329	12700	149	24235
2132403	9540	228	19335
1899806	3495	93	17356
1668110	12666	178	23277
1418729	613	120	10364
1094550	4504	10	3283
1034732	485	85	5773
1030984	3147	164	9946
979762	2840	117	8927
906387	4036	346	6751
738707	2679	37	4052
679466	3450	44	10712
664977	2212	10	2253
625033	1526	104	3905
614841	464	296	4671
606562	2756	13	4826
549934	2591	44	4258
505881	538	266	5655
501182	905	105	6388
497825	1676	24	2416
492738	1081	34	2754
463892	1154	24	5835
457435	543	12	2502
456927	546	19	660
454196	638	67	4333
436478	1372	96	7402
393632	1273	224	3273
339414	781	192	2198
313784	455	259	2413
312924	981	21	2657
286444	1281	84	967
270499	744	21	1168
261741	1162	66	2341
249097	60	70	1377
230103	1924	125	1897

221424	803	21	1544
209492	591	5	1275
198409	1039	6	1633
178964	45	2	406
177822	172	4	263
154499	1136	27	797
151962	278	15	679
143408	950	23	1106
140534	196	9	1627
137656	392	0	246
134572	90	0	190
127276	368	18	1773
125050	266	4	594
122438	661	20	820
115231	275	74	810
106322	669	18	481
102310	341	9	468
96763	24	5	189
94939	13	3	29
93447	399	14	966
87914	144	4	1248
82826	308	0	206
79690	55	9	720
78184	194	9	328
75851	51	260	1231
72033	740	5	223
70273	353	22	758
69125	40	11	417
68156	258	6	724
67492	126	2	371
67274	607	3	357
65133	238	2	427
56449	153	129	272
56432	155	3	227
53045	234	6	110
52339	135	8	859
51963	249	0	221
51269	63	33	438
50621	1073	0	55
49200	76	1	249
49163	317	0	137
48499	165	9	270
41327	12	4	271
40550	134	7	184
37949	99	5	228
36838	91	1	178
36676	9	0	14
32919	174	0	210
30545	50	1	186
29010	11	0	161
28172	32	6	170
28131	53	3	84
27406	112	6	140
27234	106	11	243
26772	8	0	98
26326	438	0	200
26245	37	0	171
26061	145	1	178
25828	36	40	236

25551	46	5	284
23957	24	2	155
23836	141	1	52
23463	58	10	122
23447	36	2	219
23408	26	1	180
23183	27	2	134
20724	111	3	158
20260	119	0	89
19723	25	0	290
18526	39	0	116
18158	76	5	208
16606	66	0	70
16193	28	1	71
16193	28	1	71
15377	95	2	106
15302	199	0	92
15272	72	0	73
14132	71	0	99
13689	36	1	97
13339	1	0	5
12974	68	1	60
12392	62	0	85
12317	69	0	55
12212	30	3	58
11818	12	1	78
11434	9	6	151
11121	26	0	48
11028	25	1	62
10806	56	2	51
10703	67	0	75
10605	29	0	39
9824	15	0	72
9782	64	0	159
9597	16	2	93
9488	45	0	37
9014	36	0	65
9012	40	2	56
8832	48	0	38
8236	24	0	53
8114	3	0	9
7859	23	1	128
7859	26	0	60
7834	7	0	61
7338	24	3	76
7318	21	0	47
7191	10	0	16
7081	12	0	110
7035	41	0	49
6797	16	1	39
6584	78	0	19
6466	40	0	52
6416	9	4	71
6377	69	0	19
6243	63	1	43
6242	34	0	63
6205	1	0	6
6134	28	0	22
6081	9	0	18

6064	22	0	31
6037	7	1	18
5856	23	0	59
5792	20	2	43
5689	132	0	6
5638	6	0	11
5485	55	0	125
5465	21	0	25
5452	14	0	37
5418	23	0	41
5411	15	1	20
5405	3	0	15
5398	11	0	14
5381	96	0	19
5381	11	1	23
5198	67	0	45
4983	56	0	20
4907	13	2	65
4692	1	0	24
4676	93	1	21
4641	3	1	34
4477	22	1	13
4443	18	2	24
4439	1	0	22
4400	60	0	30
4359	11	1	16
4323	1	0	12
4232	31	0	0
4205	0	0	6
4174	12	1	15
4157	9	3	18
4141	20	1	41
3995	12	1	28
3968	4	0	2
3955	14	0	48
3845	30	0	20
3785	45	1	63
3707	3	0	11
3686	12	0	14
3620	23	0	21
3618	9	0	14
3607	0	0	29
3546	52	0	15
3529	12	0	28
3504	17	0	24
3467	13	1	36
3415	39	0	49
3391	12	0	25
3235	6	0	4
3142	1	0	18
3042	8	0	25
3041	18	0	8
3023	5	1	14
2999	1	0	8
2816	6	0	10
2792	0	0	1
2699	4	0	35
2612	0	2	17
2540	19	0	24

2502	21	0	30
2419	34	0	25
2275	12	0	22
2252	18	0	11
2239	7	0	23
2235	12	0	23
2207	7	0	13
2170	14	0	42
2147	4	0	10
2119	4	0	20
2115	2	0	5
2034	15	1	10
2010	2	0	14
1960	19	0	23
1952	10	0	11
1929	3	0	1
1891	1	0	12
1888	7	0	19
1869	21	0	5
1862	15	0	6
1855	2	1	16
1845	10	1	13
1845	10	1	13
1751	19	0	6
1720	2	0	15
1718	2	0	29
1710	10	0	3
1707	1	0	12
1691	5	0	11
1646	3	0	12
1619	6	1	17
1607	6	0	5
1586	1	0	1
1585	2	1	22
1580	6	0	9
1576	11	0	30
1567	0	0	1
1552	7	0	18
1548	2	0	10
1508	3	0	7
1463	6	0	13
1443	2	0	10
1392	13	0	5
1382	7	0	7
1377	11	0	7
1351	10	0	15
1323	0	0	0
1311	7	0	5
1251	3	0	16
1239	2	0	14
1239	7	0	7
1226	4	0	12
1221	0	0	0
1216	8	1	19
1214	2	0	5
1212	5	0	3
1206	7	0	4
1162	8	1	8
1134	3	0	3

1131	16	0	2
1127	4	1	6
1118	10	0	3
1111	4	0	4
1104	3	0	5
1075	5	0	6
1072	2	0	5
1066	25	0	3
1053	0	1	3
1052	1	0	5
1048	0	0	4
1038	5	0	3
1038	6	1	6
1029	5	0	0
1005	13	0	3
1001	2	0	7
997	0	0	25
974	4	0	7
961	19	0	5
949	9	0	5
942	4	0	4
930	3	0	10
925	5	0	4
920	11	0	13
898	1	0	4
887	0	0	8
872	11	0	3
858	6	0	8
818	0	0	8
806	0	0	0
796	3	0	9
796	0	0	2
794	0	0	3
789	1	2	8
783	3	0	14
771	4	0	6
770	7	0	4
763	9	0	7
741	7	0	1
739	0	0	3
733	2	0	2
718	1	0	7
709	7	0	3
706	0	0	3
701	7	0	2
700	12	0	0
683	6	0	7
658	1	1	11
658	6	1	5
646	1	0	7
640	2	0	4
639	3	0	1
639	0	0	2
637	0	0	3
629	0	0	3
628	2	0	0
624	1	0	1
607	2	1	0
605	8	0	11

604	1	0	13
601	4	0	5
592	4	0	3
591	2	0	1
573	0	0	2
571	0	0	1
566	6	0	4
564	2	0	3
553	3	0	8
545	0	0	1
544	3	0	3
528	1	0	2
525	2	0	20
521	0	0	1
518	1	0	4
512	4	0	6
495	1	0	0
471	8	0	2
463	1	0	2
463	3	0	2
454	1	0	0
451	3	0	1
443	0	0	6
441	1	0	5
438	3	0	2
434	3	0	6
427	0	0	0
418	0	0	0
412	2	0	7
394	1	0	6
394	2	1	3
383	1	0	1
374	1	0	2
374	0	0	1
371	2	0	1
370	3	0	3
356	0	0	1
354	0	0	2
353	5	0	1
352	0	0	1
351	0	0	3
350	6	0	2
348	6	0	4
335	2	0	1
330	0	0	3
329	2	0	0
328	0	0	3
322	0	0	2
320	0	0	1
319	2	0	0
318	3	0	4
317	3	0	2
313	1	0	5
311	2	0	0
311	1	0	4
310	1	0	3
308	0	0	1
307	3	0	4
302	0	1	0

296	0	0	1
295	2	0	0
286	0	0	5
286	1	0	1
285	3	1	1
281	1	0	1
280	1	0	0
279	0	0	1
278	1	1	3
276	3	0	2
275	0	0	2
273	0	0	0
273	0	0	0
269	1	0	1
266	4	0	1
258	1	0	1
257	2	0	2
246	4	0	1
246	0	0	0
246	1	0	0
245	3	0	1
244	0	0	2
241	2	0	2
241	1	0	1
237	4	0	1
236	0	0	0
235	1	0	1
232	0	1	1
230	0	0	0
230	1	0	0
229	0	0	0
229	1	0	0
229	0	0	2
225	1	0	2
224	0	0	0
224	0	0	2
223	1	0	4
223	2	0	1
221	2	0	1
221	4	0	3
219	0	0	0
212	0	0	0
208	0	0	1
207	1	0	0
204	1	0	1
204	0	0	1
199	0	0	0
198	1	0	2
198	0	1	5
197	0	0	2
196	0	1	3
196	0	0	2
196	0	0	3
194	0	0	0
189	1	0	2
185	0	0	1
182	2	0	2
182	2	0	2
176	1	0	0

176	4	0	0
172	0	0	0
172	0	0	0
168	0	0	1
166	0	0	0
164	1	0	0
163	1	0	1
162	0	0	1
162	0	0	0
161	0	0	2
160	0	0	0
157	0	0	0
155	0	0	1
153	1	0	0
152	0	0	1
151	0	0	4
149	1	0	1
149	0	0	1
149	0	0	1
147	1	0	1
146	0	0	1
144	0	0	1
143	0	0	0
143	0	0	1
142	0	0	0
141	1	0	2
141	0	0	1
140	2	0	0
138	0	0	0
137	0	0	1
137	0	0	3
135	1	0	1
134	0	0	0
134	0	0	1
133	0	0	0
133	2	0	2
133	0	0	0
132	0	0	0
132	0	0	1
132	0	0	0
130	4	0	0
130	0	0	0
129	1	0	0
128	0	0	2
126	0	0	0
124	0	0	3
122	0	0	1
119	2	0	4
119	1	0	0
118	0	0	0
118	1	0	1
117	1	0	1
117	1	0	3
116	1	0	0
116	1	0	1
113	0	0	1
113	0	0	0
110	0	0	0
110	4	0	0

107	0	0	0
107	1	0	1
106	0	0	0
106	0	0	0
106	1	0	0
103	0	0	0
103	2	0	0
102	0	0	0
102	1	0	1
101	2	0	0
100	1	0	1
100	2	0	0
total reads <= 100 in top 30	0	9	0
	A_11	A_16	A_2
	UC	UC	UC
223271318	1171926	494598	4475462
divide ave top31	22	30	25
z-score	0.24	1.79	0.82
ttest UC v P	0.07		
ttest NP v P	0.93		

XX SAMPLES specified here:			
rand sampling numbers=	0.93	0.57	0.09
38	3	13	26
95	1	1	1
index all samples:	1	2	3
index type:	1	2	3
total of totals of =100:	A_11	A_16	A_2
139587919	UC	UC	UC
total all miRNA reads =100:	479320	21319	1102300
total all samples =100:	A_11	A_16	A_2
46389442	253154	2382	397359
10497180	9303	9512	44303
8994745	18873	920	14220
8839402	25490	1159	106715
8658137	29662	380	50300
7939871	3705	835	68855
5953261	6768	639	39890
3762724	986	127	13562
3462453	5884	74	50176
3413660	17952	549	60322
2186845	12700	149	24235
2131733	9540	228	19335
1899491	3495	93	17356
1666971	12666	178	23277
1418336	613	120	10364
1093922	4504	10	3283
1034642	485	85	5773
1030775	3147	164	9946
979581	2840	117	8927

903691	4036	346	6751
738230	2679	37	4052
679306	3450	44	10712
664224	2212	10	2253
624773	1526	104	3905
614674	464	296	4671
605614	2756	13	4826
549148	2591	44	4258
501331	538	266	5655
500966	905	105	6388
497718	1676	24	2416
492626	1081	34	2754
463804	1154	24	5835
457362	543	12	2502
456843	546	19	660
454020	638	67	4333
436288	1372	96	7402
393199	1273	224	3273
339140	781	192	2198
313699	455	259	2413
312449	981	21	2657
286288	1281	84	967
270366	744	21	1168
261666	1162	66	2341
248983	60	70	1377
229733	1924	125	1897
221365	803	21	1544
209441	591	5	1275
198175	1039	6	1633
178962	45	2	406
177234	172	4	263
154285	1136	27	797
151875	278	15	679
143334	950	23	1106
138740	196	9	1627
137573	392	0	246
134502	90	0	190
127210	368	18	1773
125038	266	4	594
122415	661	20	820
115160	275	74	810
106297	669	18	481
102201	341	9	468
96743	24	5	189
94911	13	3	29
93386	399	14	966
87898	144	4	1248
82798	308	0	206
79678	55	9	720
78156	194	9	328
75744	51	260	1231
71988	740	5	223
70256	353	22	758
69114	40	11	417
68018	258	6	724
67482	126	2	371
67182	607	3	357
65049	238	2	427
56245	153	129	272

56410	155	3	227
53026	234	6	110
52323	135	8	859
51847	249	0	221
51252	63	33	438
50588	1073	0	55
49172	76	1	249
49098	317	0	137
48416	165	9	270
41318	12	4	271
40544	134	7	184
37943	99	5	228
36605	91	1	178
36676	9	0	14
32906	174	0	210
30537	50	1	186
29005	11	0	161
28163	32	6	170
28118	53	3	84
27350	112	6	140
27199	106	11	243
26769	8	0	98
26325	438	0	200
26241	37	0	171
26016	145	1	178
25811	36	40	236
25540	46	5	284
23949	24	2	155
23822	141	1	52
23449	58	10	122
23446	36	2	219
23397	26	1	180
23176	27	2	134
20718	111	3	158
20235	119	0	89
19721	25	0	290
18520	39	0	116
18147	76	5	208
16585	66	0	70
16186	28	1	71
16186	28	1	71
15369	95	2	106
15277	199	0	92
15261	72	0	73
14130	71	0	99
13673	36	1	97
13338	1	0	5
12970	68	1	60
12358	62	0	85
12315	69	0	55
12195	30	3	58
11818	12	1	78
11405	9	6	151
11100	26	0	48
11007	25	1	62
10799	56	2	51
10674	67	0	75
10591	29	0	39
9795	15	0	72

9779	64	0	159
9597	16	2	93
9484	45	0	37
8988	36	0	65
9010	40	2	56
8799	48	0	38
8234	24	0	53
8112	3	0	9
7853	23	1	128
7857	26	0	60
7833	7	0	61
7337	24	3	76
7313	21	0	47
7188	10	0	16
7081	12	0	110
7031	41	0	49
6794	16	1	39
6526	78	0	19
6466	40	0	52
6415	9	4	71
6371	69	0	19
6235	63	1	43
6234	34	0	63
6205	1	0	6
6131	28	0	22
6075	9	0	18
6064	22	0	31
6037	7	1	18
5852	23	0	59
5791	20	2	43
5684	132	0	6
5638	6	0	11
5483	55	0	125
5462	21	0	25
5445	14	0	37
5410	23	0	41
5409	15	1	20
5404	3	0	15
5394	11	0	14
5376	96	0	19
5378	11	1	23
5198	67	0	45
4981	56	0	20
4906	13	2	65
4691	1	0	24
4664	93	1	21
4641	3	1	34
4449	22	1	13
4437	18	2	24
4439	1	0	22
4396	60	0	30
4359	11	1	16
4317	1	0	12
4227	31	0	0
4204	0	0	6
4173	12	1	15
4157	9	3	18
4113	20	1	41
3993	12	1	28

3954	4	0	2
3954	14	0	48
3836	30	0	20
3781	45	1	63
3706	3	0	11
3683	12	0	14
3618	23	0	21
3550	9	0	14
3607	0	0	29
3539	52	0	15
3527	12	0	28
3502	17	0	24
3467	13	1	36
3405	39	0	49
3388	12	0	25
3235	6	0	4
3139	1	0	18
3039	8	0	25
3036	18	0	8
3023	5	1	14
2999	1	0	8
2814	6	0	10
2792	0	0	1
2699	4	0	35
2607	0	2	17
2540	19	0	24
2500	21	0	30
2416	34	0	25
2275	12	0	22
2250	18	0	11
2236	7	0	23
2231	12	0	23
2207	7	0	13
2170	14	0	42
2146	4	0	10
2119	4	0	20
2114	2	0	5
2033	15	1	10
2009	2	0	14
1957	19	0	23
1952	10	0	11
1926	3	0	1
1890	1	0	12
1888	7	0	19
1866	21	0	5
1862	15	0	6
1854	2	1	16
1843	10	1	13
1843	10	1	13
1750	19	0	6
1719	2	0	15
1715	2	0	29
1709	10	0	3
1707	1	0	12
1690	5	0	11
1641	3	0	12
1619	6	1	17
1607	6	0	5
1586	1	0	1

1585	2	1	22
1577	6	0	9
1576	11	0	30
1567	0	0	1
1552	7	0	18
1548	2	0	10
1507	3	0	7
1463	6	0	13
1443	2	0	10
1383	13	0	5
1382	7	0	7
1377	11	0	7
1351	10	0	15
1322	0	0	0
1307	7	0	5
1250	3	0	16
1237	2	0	14
1239	7	0	7
1226	4	0	12
1221	0	0	0
1216	8	1	19
1214	2	0	5
1209	5	0	3
1206	7	0	4
1161	8	1	8
1134	3	0	3
1131	16	0	2
1126	4	1	6
1118	10	0	3
1109	4	0	4
1104	3	0	5
1074	5	0	6
1072	2	0	5
1064	25	0	3
1052	0	1	3
1051	1	0	5
1047	0	0	4
1037	5	0	3
1038	6	1	6
1026	5	0	0
998	13	0	3
1001	2	0	7
997	0	0	25
973	4	0	7
960	19	0	5
949	9	0	5
942	4	0	4
930	3	0	10
925	5	0	4
917	11	0	13
897	1	0	4
887	0	0	8
872	11	0	3
858	6	0	8
818	0	0	8
805	0	0	0
794	3	0	9
795	0	0	2
794	0	0	3

789	1	2	8
782	3	0	14
771	4	0	6
768	7	0	4
761	9	0	7
741	7	0	1
739	0	0	3
733	2	0	2
718	1	0	7
709	7	0	3
705	0	0	3
701	7	0	2
697	12	0	0
683	6	0	7
656	1	1	11
657	6	1	5
646	1	0	7
640	2	0	4
639	3	0	1
639	0	0	2
637	0	0	3
629	0	0	3
628	2	0	0
624	1	0	1
603	2	1	0
600	8	0	11
594	1	0	13
600	4	0	5
591	4	0	3
591	2	0	1
572	0	0	2
571	0	0	1
566	6	0	4
564	2	0	3
552	3	0	8
544	0	0	1
544	3	0	3
528	1	0	2
525	2	0	20
521	0	0	1
518	1	0	4
511	4	0	6
495	1	0	0
471	8	0	2
463	1	0	2
463	3	0	2
454	1	0	0
450	3	0	1
443	0	0	6
440	1	0	5
438	3	0	2
434	3	0	6
427	0	0	0
418	0	0	0
408	2	0	7
394	1	0	6
394	2	1	3
383	1	0	1
374	1	0	2

374	0	0	1
371	2	0	1
370	3	0	3
356	0	0	1
354	0	0	2
352	5	0	1
352	0	0	1
351	0	0	3
349	6	0	2
348	6	0	4
335	2	0	1
330	0	0	3
328	2	0	0
328	0	0	3
321	0	0	2
320	0	0	1
319	2	0	0
318	3	0	4
317	3	0	2
313	1	0	5
311	2	0	0
311	1	0	4
308	1	0	3
308	0	0	1
307	3	0	4
302	0	1	0
296	0	0	1
294	2	0	0
286	0	0	5
285	1	0	1
285	3	1	1
280	1	0	1
280	1	0	0
279	0	0	1
278	1	1	3
275	3	0	2
275	0	0	2
272	0	0	0
273	0	0	0
269	1	0	1
266	4	0	1
258	1	0	1
257	2	0	2
246	4	0	1
246	0	0	0
246	1	0	0
245	3	0	1
244	0	0	2
241	2	0	2
241	1	0	1
237	4	0	1
236	0	0	0
235	1	0	1
232	0	1	1
230	0	0	0
230	1	0	0
229	0	0	0
229	1	0	0
228	0	0	2

225	1	0	2
224	0	0	0
223	0	0	2
223	1	0	4
223	2	0	1
220	2	0	1
221	4	0	3
219	0	0	0
212	0	0	0
207	0	0	1
207	1	0	0
204	1	0	1
204	0	0	1
199	0	0	0
198	1	0	2
198	0	1	5
197	0	0	2
196	0	1	3
196	0	0	2
195	0	0	3
194	0	0	0
189	1	0	2
185	0	0	1
182	2	0	2
182	2	0	2
176	1	0	0
176	4	0	0
171	0	0	0
172	0	0	0
168	0	0	1
166	0	0	0
164	1	0	0
163	1	0	1
162	0	0	1
162	0	0	0
161	0	0	2
160	0	0	0
157	0	0	0
155	0	0	1
152	1	0	0
152	0	0	1
151	0	0	4
149	1	0	1
148	0	0	1
149	0	0	1
147	1	0	1
146	0	0	1
144	0	0	1
143	0	0	0
143	0	0	1
142	0	0	0
141	1	0	2
141	0	0	1
140	2	0	0
138	0	0	0
137	0	0	1
137	0	0	3
135	1	0	1
134	0	0	0

134	0	0	1
133	0	0	0
133	2	0	2
132	0	0	0
132	0	0	0
132	0	0	1
132	0	0	0
130	4	0	0
129	0	0	0
129	1	0	0
128	0	0	2
126	0	0	0
124	0	0	3
122	0	0	1
118	2	0	4
119	1	0	0
118	0	0	0
118	1	0	1
117	1	0	1
117	1	0	3
116	1	0	0
116	1	0	1
113	0	0	1
113	0	0	0
109	0	0	0
110	4	0	0
107	0	0	0
106	1	0	1
106	0	0	0
106	0	0	0
106	1	0	0
103	0	0	0
103	2	0	0
102	0	0	0
102	1	0	1
101	2	0	0
100	1	0	1
99	2	0	0

rand sampling numbers=	0.93	0.57	0.09
38	3	13	26
95	1	1	1
index all samples:	1	2	3
index type:	1	2	3
total of totals of =100:	A_11	A_16	A_2
139587919	UC	UC	UC
total all miRNA reads =100:	479320	21319	1102300
total all samples =100:	UC	UC	UC
46389442	253154	2382	397359
10497180	9303	9512	44303
8994745	18873	920	14220
8839402	25490	1159	106715
8658137	29662	380	50300

7939871	3705	835	68855
5953261	6768	639	39890
3762724	986	127	13562
3462453	5884	74	50176
3413660	17952	549	60322
2186845	12700	149	24235
2131733	9540	228	19335
1899491	3495	93	17356
1666971	12666	178	23277
1418336	613	120	10364
1093922	4504	10	3283
1034642	485	85	5773
1030775	3147	164	9946
979581	2840	117	8927
903691	4036	346	6751
738230	2679	37	4052
679306	3450	44	10712
664224	2212	10	2253
624773	1526	104	3905
614674	464	296	4671
605614	2756	13	4826
549148	2591	44	4258
501331	538	266	5655
500966	905	105	6388
497718	1676	24	2416
492626	1081	34	2754
463804	1154	24	5835
457362	543	12	2502
456843	546	19	660
454020	638	67	4333
436288	1372	96	7402
393199	1273	224	3273
339140	781	192	2198
313699	455	259	2413
312449	981	21	2657
286288	1281	84	967
270366	744	21	1168
261666	1162	66	2341
248983	60	70	1377
229733	1924	125	1897
221365	803	21	1544
209441	591	5	1275
198175	1039	6	1633
178962	45	2	406
177234	172	4	263
154285	1136	27	797
151875	278	15	679
143334	950	23	1106
138740	196	9	1627
137573	392	0	246
134502	90	0	190
127210	368	18	1773
125038	266	4	594
122415	661	20	820
115160	275	74	810
106297	669	18	481
102201	341	9	468
96743	24	5	189
94911	13	3	29

93386	399	14	966
87898	144	4	1248
82798	308	0	206
79678	55	9	720
78156	194	9	328
75744	51	260	1231
71988	740	5	223
70256	353	22	758
69114	40	11	417
68018	258	6	724
67482	126	2	371
67182	607	3	357
65049	238	2	427
56245	153	129	272
56410	155	3	227
53026	234	6	110
52323	135	8	859
51847	249	0	221
51252	63	33	438
50588	1073	0	55
49172	76	1	249
49098	317	0	137
48416	165	9	270
41318	12	4	271
40544	134	7	184
37943	99	5	228
36605	91	1	178
36676	9	0	14
32906	174	0	210
30537	50	1	186
29005	11	0	161
28163	32	6	170
28118	53	3	84
27350	112	6	140
27199	106	11	243
26769	8	0	98
26325	438	0	200
26241	37	0	171
26016	145	1	178
25811	36	40	236
25540	46	5	284
23949	24	2	155
23822	141	1	52
23449	58	10	122
23446	36	2	219
23397	26	1	180
23176	27	2	134
20718	111	3	158
20235	119	0	89
19721	25	0	290
18520	39	0	116
18147	76	5	208
16585	66	0	70
16186	28	1	71
16186	28	1	71
15369	95	2	106
15277	199	0	92
15261	72	0	73
14130	71	0	99

13673	36	1	97
13338	1	0	5
12970	68	1	60
12358	62	0	85
12315	69	0	55
12195	30	3	58
11818	12	1	78
11405	9	6	151
11100	26	0	48
11007	25	1	62
10799	56	2	51
10674	67	0	75
10591	29	0	39
9795	15	0	72
9779	64	0	159
9597	16	2	93
9484	45	0	37
8988	36	0	65
9010	40	2	56
8799	48	0	38
8234	24	0	53
8112	3	0	9
7853	23	1	128
7857	26	0	60
7833	7	0	61
7337	24	3	76
7313	21	0	47
7188	10	0	16
7081	12	0	110
7031	41	0	49
6794	16	1	39
6526	78	0	19
6466	40	0	52
6415	9	4	71
6371	69	0	19
6235	63	1	43
6234	34	0	63
6205	1	0	6
6131	28	0	22
6075	9	0	18
6064	22	0	31
6037	7	1	18
5852	23	0	59
5791	20	2	43
5684	132	0	6
5638	6	0	11
5483	55	0	125
5462	21	0	25
5445	14	0	37
5410	23	0	41
5409	15	1	20
5404	3	0	15
5394	11	0	14
5376	96	0	19
5378	11	1	23
5198	67	0	45
4981	56	0	20
4906	13	2	65
4691	1	0	24

4664	93	1	21
4641	3	1	34
4449	22	1	13
4437	18	2	24
4439	1	0	22
4396	60	0	30
4359	11	1	16
4317	1	0	12
4227	31	0	0
4204	0	0	6
4173	12	1	15
4157	9	3	18
4113	20	1	41
3993	12	1	28
3954	4	0	2
3954	14	0	48
3836	30	0	20
3781	45	1	63
3706	3	0	11
3683	12	0	14
3618	23	0	21
3550	9	0	14
3607	0	0	29
3539	52	0	15
3527	12	0	28
3502	17	0	24
3467	13	1	36
3405	39	0	49
3388	12	0	25
3235	6	0	4
3139	1	0	18
3039	8	0	25
3036	18	0	8
3023	5	1	14
2999	1	0	8
2814	6	0	10
2792	0	0	1
2699	4	0	35
2607	0	2	17
2540	19	0	24
2500	21	0	30
2416	34	0	25
2275	12	0	22
2250	18	0	11
2236	7	0	23
2231	12	0	23
2207	7	0	13
2170	14	0	42
2146	4	0	10
2119	4	0	20
2114	2	0	5
2033	15	1	10
2009	2	0	14
1957	19	0	23
1952	10	0	11
1926	3	0	1
1890	1	0	12
1888	7	0	19
1866	21	0	5

1862	15	0	6
1854	2	1	16
1843	10	1	13
1843	10	1	13
1750	19	0	6
1719	2	0	15
1715	2	0	29
1709	10	0	3
1707	1	0	12
1690	5	0	11
1641	3	0	12
1619	6	1	17
1607	6	0	5
1586	1	0	1
1585	2	1	22
1577	6	0	9
1576	11	0	30
1567	0	0	1
1552	7	0	18
1548	2	0	10
1507	3	0	7
1463	6	0	13
1443	2	0	10
1383	13	0	5
1382	7	0	7
1377	11	0	7
1351	10	0	15
1322	0	0	0
1307	7	0	5
1250	3	0	16
1237	2	0	14
1239	7	0	7
1226	4	0	12
1221	0	0	0
1216	8	1	19
1214	2	0	5
1209	5	0	3
1206	7	0	4
1161	8	1	8
1134	3	0	3
1131	16	0	2
1126	4	1	6
1118	10	0	3
1109	4	0	4
1104	3	0	5
1074	5	0	6
1072	2	0	5
1064	25	0	3
1052	0	1	3
1051	1	0	5
1047	0	0	4
1037	5	0	3
1038	6	1	6
1026	5	0	0
998	13	0	3
1001	2	0	7
997	0	0	25
973	4	0	7
960	19	0	5

949	9	0	5
942	4	0	4
930	3	0	10
925	5	0	4
917	11	0	13
897	1	0	4
887	0	0	8
872	11	0	3
858	6	0	8
818	0	0	8
805	0	0	0
794	3	0	9
795	0	0	2
794	0	0	3
789	1	2	8
782	3	0	14
771	4	0	6
768	7	0	4
761	9	0	7
741	7	0	1
739	0	0	3
733	2	0	2
718	1	0	7
709	7	0	3
705	0	0	3
701	7	0	2
697	12	0	0
683	6	0	7
656	1	1	11
657	6	1	5
646	1	0	7
640	2	0	4
639	3	0	1
639	0	0	2
637	0	0	3
629	0	0	3
628	2	0	0
624	1	0	1
603	2	1	0
600	8	0	11
594	1	0	13
600	4	0	5
591	4	0	3
591	2	0	1
572	0	0	2
571	0	0	1
566	6	0	4
564	2	0	3
552	3	0	8
544	0	0	1
544	3	0	3
528	1	0	2
525	2	0	20
521	0	0	1
518	1	0	4
511	4	0	6
495	1	0	0
471	8	0	2
463	1	0	2

463	3	0	2
454	1	0	0
450	3	0	1
443	0	0	6
440	1	0	5
438	3	0	2
434	3	0	6
427	0	0	0
418	0	0	0
408	2	0	7
394	1	0	6
394	2	1	3
383	1	0	1
374	1	0	2
374	0	0	1
371	2	0	1
370	3	0	3
356	0	0	1
354	0	0	2
352	5	0	1
352	0	0	1
351	0	0	3
349	6	0	2
348	6	0	4
335	2	0	1
330	0	0	3
328	2	0	0
328	0	0	3
321	0	0	2
320	0	0	1
319	2	0	0
318	3	0	4
317	3	0	2
313	1	0	5
311	2	0	0
311	1	0	4
308	1	0	3
308	0	0	1
307	3	0	4
302	0	1	0
296	0	0	1
294	2	0	0
286	0	0	5
285	1	0	1
285	3	1	1
280	1	0	1
280	1	0	0
279	0	0	1
278	1	1	3
275	3	0	2
275	0	0	2
272	0	0	0
273	0	0	0
269	1	0	1
266	4	0	1
258	1	0	1
257	2	0	2
246	4	0	1
246	0	0	0

246	1	0	0
245	3	0	1
244	0	0	2
241	2	0	2
241	1	0	1
237	4	0	1
236	0	0	0
235	1	0	1
232	0	1	1
230	0	0	0
230	1	0	0
229	0	0	0
229	1	0	0
228	0	0	2
225	1	0	2
224	0	0	0
223	0	0	2
223	1	0	4
223	2	0	1
220	2	0	1
221	4	0	3
219	0	0	0
212	0	0	0
207	0	0	1
207	1	0	0
204	1	0	1
204	0	0	1
199	0	0	0
198	1	0	2
198	0	1	5
197	0	0	2
196	0	1	3
196	0	0	2
195	0	0	3
194	0	0	0
189	1	0	2
185	0	0	1
182	2	0	2
182	2	0	2
176	1	0	0
176	4	0	0
171	0	0	0
172	0	0	0
168	0	0	1
166	0	0	0
164	1	0	0
163	1	0	1
162	0	0	1
162	0	0	0
161	0	0	2
160	0	0	0
157	0	0	0
155	0	0	1
152	1	0	0
152	0	0	1
151	0	0	4
149	1	0	1
148	0	0	1
149	0	0	1

147	1	0	1
146	0	0	1
144	0	0	1
143	0	0	0
143	0	0	1
142	0	0	0
141	1	0	2
141	0	0	1
140	2	0	0
138	0	0	0
137	0	0	1
137	0	0	3
135	1	0	1
134	0	0	0
134	0	0	1
133	0	0	0
133	2	0	2
132	0	0	0
132	0	0	0
132	0	0	1
132	0	0	0
130	4	0	0
129	0	0	0
129	1	0	0
128	0	0	2
126	0	0	0
124	0	0	3
122	0	0	1
118	2	0	4
119	1	0	0
118	0	0	0
118	1	0	1
117	1	0	1
117	1	0	3
116	1	0	0
116	1	0	1
113	0	0	1
113	0	0	0
109	0	0	0
110	4	0	0
107	0	0	0
106	1	0	1
106	0	0	0
106	0	0	0
106	1	0	0
103	0	0	0
103	2	0	0
102	0	0	0
102	1	0	1
101	2	0	0
100	1	0	1
99	2	0	0
ave top 30	14820	634	34136
sd top 30	45642	1745	72911
total reads <= 100 in all miRs	441	503	414
	UC	UC	UC
	UC	UC	UC

95			
index all samples:	1	2	3
index type:	1	2	3
total of totals of =100:	UC	UC	UC
139641984	A_11	A_16	A_2
total all miRNA reads =100:	479320	21319	1102300
total all samples =100:	UC	UC	UC
46389442	17.08	3.76	11.64
10497180	0.63	15.01	1.30
8994745	1.27	1.45	0.42
8839402	1.72	1.83	3.13
8658137	2.00	0.60	1.47
7939871	0.25	1.32	2.02
5953261	0.46	1.01	1.17
3762724	0.07	0.20	0.40
3462453	0.40	0.12	1.47
3413660	1.21	0.87	1.77
2186845	0.86	0.24	0.71
2131733	0.64	0.36	0.57
1899491	0.24	0.15	0.51
1666971	0.85	0.28	0.68
1418336	0.04	0.19	0.30
1093922	0.30	0.02	0.10
1034642	0.03	0.13	0.17
1030775	0.21	0.26	0.29
979581	0.19	0.18	0.26
903691	0.27	0.55	0.20
738230	0.18	0.06	0.12
679306	0.23	0.07	0.31
664224	0.15	0.02	0.07
624773	0.10	0.16	0.11
614674	0.03	0.47	0.14
605614	0.19	0.02	0.14
549148	0.17	0.07	0.12
501331	0.04	0.42	0.17
500966	0.06	0.17	0.19
497718	0.11	0.04	0.07
492626	0.07	0.05	0.08
463804	0.08	0.04	0.17
457362	0.04	0.02	0.07
456843	0.04	0.03	0.02
454020	0.04	0.11	0.13
436288	0.09	0.15	0.22
393199	0.09	0.35	0.10
339140	0.05	0.30	0.06
313699	0.03	0.41	0.07
312449	0.07	0.03	0.08
286288	0.09	0.13	0.03
270366	0.05	0.03	0.03
261666	0.08	0.10	0.07
248983	0.00	0.11	0.04
229733	0.13	0.20	0.06
221365	0.05	0.03	0.05
209441	0.04	0.01	0.04
198175	0.07	0.01	0.05
178962	0.00	0.00	0.01
177234	0.01	0.01	0.01
154285	0.08	0.04	0.02
151875	0.02	0.02	0.02

143334	0.06	0.04	0.03
138740	0.01	0.01	0.05
137573	0.03	0.00	0.01
134502	0.01	0.00	0.01
127210	0.02	0.03	0.05
125038	0.02	0.01	0.02
122415	0.04	0.03	0.02
115160	0.02	0.12	0.02
106297	0.05	0.03	0.01
102201	0.02	0.01	0.01
96743	0.00	0.01	0.01
94911	0.00	0.00	0.00
93386	0.03	0.02	0.03
87898	0.01	0.01	0.04
82798	0.02	0.00	0.01
79678	0.00	0.01	0.02
78156	0.01	0.01	0.01
75744	0.00	0.41	0.04
71988	0.05	0.01	0.01
70256	0.02	0.03	0.02
69114	0.00	0.02	0.01
68018	0.02	0.01	0.02
67482	0.01	0.00	0.01
67182	0.04	0.00	0.01
65049	0.02	0.00	0.01
56245	0.01	0.20	0.01
56410	0.01	0.00	0.01
53026	0.02	0.01	0.00
52323	0.01	0.01	0.03
51847	0.02	0.00	0.01
51252	0.00	0.05	0.01
50588	0.07	0.00	0.00
49172	0.01	0.00	0.01
49098	0.02	0.00	0.00
48416	0.01	0.01	0.01
41318	0.00	0.01	0.01
40544	0.01	0.01	0.01
37943	0.01	0.01	0.01
36605	0.01	0.00	0.01
36676	0.00	0.00	0.00
32906	0.01	0.00	0.01
30537	0.00	0.00	0.01
29005	0.00	0.00	0.00
28163	0.00	0.01	0.00
28118	0.00	0.00	0.00
27350	0.01	0.01	0.00
27199	0.01	0.02	0.01
26769	0.00	0.00	0.00
26325	0.03	0.00	0.01
26241	0.00	0.00	0.01
26016	0.01	0.00	0.01
25811	0.00	0.06	0.01
25540	0.00	0.01	0.01
23949	0.00	0.00	0.00
23822	0.01	0.00	0.00
23449	0.00	0.02	0.00
23446	0.00	0.00	0.01
23397	0.00	0.00	0.01
23176	0.00	0.00	0.00

20718	0.01	0.00	0.00
20235	0.01	0.00	0.00
19721	0.00	0.00	0.01
18520	0.00	0.00	0.00
18147	0.01	0.01	0.01
16585	0.00	0.00	0.00
16186	0.00	0.00	0.00
16186	0.00	0.00	0.00
15369	0.01	0.00	0.00
15277	0.01	0.00	0.00
15261	0.00	0.00	0.00
14130	0.00	0.00	0.00
13673	0.00	0.00	0.00
13338	0.00	0.00	0.00
12970	0.00	0.00	0.00
12358	0.00	0.00	0.00
12315	0.00	0.00	0.00
12195	0.00	0.00	0.00
11818	0.00	0.00	0.00
11405	0.00	0.01	0.00
11100	0.00	0.00	0.00
11007	0.00	0.00	0.00
10799	0.00	0.00	0.00
10674	0.00	0.00	0.00
10591	0.00	0.00	0.00
9795	0.00	0.00	0.00
9779	0.00	0.00	0.00
9597	0.00	0.00	0.00
9484	0.00	0.00	0.00
8988	0.00	0.00	0.00
9010	0.00	0.00	0.00
8799	0.00	0.00	0.00
8234	0.00	0.00	0.00
8112	0.00	0.00	0.00
7853	0.00	0.00	0.00
7857	0.00	0.00	0.00
7833	0.00	0.00	0.00
7337	0.00	0.00	0.00
7313	0.00	0.00	0.00
7188	0.00	0.00	0.00
7081	0.00	0.00	0.00
7031	0.00	0.00	0.00
6794	0.00	0.00	0.00
6526	0.01	0.00	0.00
6466	0.00	0.00	0.00
6415	0.00	0.01	0.00
6371	0.00	0.00	0.00
6235	0.00	0.00	0.00
6234	0.00	0.00	0.00
6205	0.00	0.00	0.00
6131	0.00	0.00	0.00
6075	0.00	0.00	0.00
6064	0.00	0.00	0.00
6037	0.00	0.00	0.00
5852	0.00	0.00	0.00
5791	0.00	0.00	0.00
5684	0.01	0.00	0.00
5638	0.00	0.00	0.00
5483	0.00	0.00	0.00

5462	0.00	0.00	0.00
5445	0.00	0.00	0.00
5410	0.00	0.00	0.00
5409	0.00	0.00	0.00
5404	0.00	0.00	0.00
5394	0.00	0.00	0.00
5376	0.01	0.00	0.00
5378	0.00	0.00	0.00
5198	0.00	0.00	0.00
4981	0.00	0.00	0.00
4906	0.00	0.00	0.00
4691	0.00	0.00	0.00
4664	0.01	0.00	0.00
4641	0.00	0.00	0.00
4449	0.00	0.00	0.00
4437	0.00	0.00	0.00
4439	0.00	0.00	0.00
4396	0.00	0.00	0.00
4359	0.00	0.00	0.00
4317	0.00	0.00	0.00
4227	0.00	0.00	0.00
4204	0.00	0.00	0.00
4173	0.00	0.00	0.00
4157	0.00	0.00	0.00
4113	0.00	0.00	0.00
3993	0.00	0.00	0.00
3954	0.00	0.00	0.00
3954	0.00	0.00	0.00
3836	0.00	0.00	0.00
3781	0.00	0.00	0.00
3706	0.00	0.00	0.00
3683	0.00	0.00	0.00
3618	0.00	0.00	0.00
3550	0.00	0.00	0.00
3607	0.00	0.00	0.00
3539	0.00	0.00	0.00
3527	0.00	0.00	0.00
3502	0.00	0.00	0.00
3467	0.00	0.00	0.00
3405	0.00	0.00	0.00
3388	0.00	0.00	0.00
3235	0.00	0.00	0.00
3139	0.00	0.00	0.00
3039	0.00	0.00	0.00
3036	0.00	0.00	0.00
3023	0.00	0.00	0.00
2999	0.00	0.00	0.00
2814	0.00	0.00	0.00
2792	0.00	0.00	0.00
2699	0.00	0.00	0.00
2607	0.00	0.00	0.00
2540	0.00	0.00	0.00
2500	0.00	0.00	0.00
2416	0.00	0.00	0.00
2275	0.00	0.00	0.00
2250	0.00	0.00	0.00
2236	0.00	0.00	0.00
2231	0.00	0.00	0.00
2207	0.00	0.00	0.00

2170	0.00	0.00	0.00
2146	0.00	0.00	0.00
2119	0.00	0.00	0.00
2114	0.00	0.00	0.00
2033	0.00	0.00	0.00
2009	0.00	0.00	0.00
1957	0.00	0.00	0.00
1952	0.00	0.00	0.00
1926	0.00	0.00	0.00
1890	0.00	0.00	0.00
1888	0.00	0.00	0.00
1866	0.00	0.00	0.00
1862	0.00	0.00	0.00
1854	0.00	0.00	0.00
1843	0.00	0.00	0.00
1843	0.00	0.00	0.00
1750	0.00	0.00	0.00
1719	0.00	0.00	0.00
1715	0.00	0.00	0.00
1709	0.00	0.00	0.00
1707	0.00	0.00	0.00
1690	0.00	0.00	0.00
1641	0.00	0.00	0.00
1619	0.00	0.00	0.00
1607	0.00	0.00	0.00
1586	0.00	0.00	0.00
1585	0.00	0.00	0.00
1577	0.00	0.00	0.00
1576	0.00	0.00	0.00
1567	0.00	0.00	0.00

95 samples:

max 30 miRs:
2
min 30 miRs:
2

	1	2	3
97			
index type:	1	2	3
total of totals of =100:	UC	UC	UC
139641984	A_11	A_16	A_2
total all miRNA reads =100:	479320	21319	1102300
total all samples =100:	A_11	A_16	A_2
46389442	1.23	-1.76	0.00
10497180	-0.77	3.44	-0.58
8994745	-0.63	-0.49	-1.27
8839402	-0.72	-0.61	0.67
8658137	-0.18	-1.73	-0.76
7939871	-0.85	0.11	0.75
5953261	-1.13	-0.25	0.00
3762724	-0.50	-0.26	0.10

3462453	-0.38	-0.94	1.75
3413660	1.16	0.27	2.58
2186845	1.45	-0.89	0.90
2131733	1.36	-1.18	0.67
1899491	-0.70	-1.21	0.87
1666971	2.23	-1.07	1.24
1418336	-0.82	-0.14	0.39
1093922	0.03	-1.22	-0.87
1034642	-0.79	-0.15	0.07
1030775	-0.02	0.44	0.76
979581	0.09	0.03	0.71
903691	0.42	3.14	-0.33
738230	0.38	-1.59	-0.62
679306	1.24	-1.25	2.48
664224	0.38	-1.27	-0.65
624773	-0.15	0.42	-0.05
614674	-0.58	2.00	0.04
605614	1.34	-1.78	0.50
549148	0.58	-1.10	-0.21
501331	-0.73	2.95	0.51
500966	-0.90	1.14	1.56
497718	0.26	-1.50	-0.73
492626	-0.41	-0.86	-0.23
463804	-0.85	-1.63	0.95
457362	-0.35	-0.61	0.21
456843	-0.20	-0.24	-0.29
454020	-1.15	0.25	0.73
436288	-0.31	0.05	0.45
393199	-0.32	2.84	-0.21
339140	-0.41	2.17	-0.29
313699	-0.82	3.71	-0.35
312449	-0.27	-0.54	-0.18
286288	0.21	1.16	-0.97
270366	-0.55	-1.07	-1.03
261666	0.57	1.61	0.17
248983	-0.83	1.81	0.07
229733	1.43	2.87	-0.15
221365	0.56	-0.96	-0.09
209441	-0.04	-1.33	-0.14
198175	0.68	-1.33	-0.06
178962	-0.29	-0.29	0.02
177234	-0.43	-0.55	-0.51
154285	2.23	0.39	-0.65
151875	-0.46	-0.31	-0.42
143334	2.20	0.05	-0.25
138740	-0.84	-0.78	0.98
137573	0.08	-1.38	-0.98

134502	-0.13	-0.38	-0.16
127210	-0.26	0.03	1.92
125038	-0.50	-1.47	-0.55
122415	1.04	0.07	-0.49
115160	-0.59	2.00	-0.45
106297	1.65	0.31	-0.84
102201	0.08	-0.65	-0.69
96743	-0.60	-0.11	-0.29
94911	-0.26	-0.08	-0.26
93386	-0.14	-0.42	-0.06
87898	-0.65	-0.98	1.93
82798	0.64	-0.89	-0.45
79678	-0.66	0.38	1.06
78156	0.04	0.17	-0.36
75744	-0.38	4.86	0.04
71988	2.00	-0.96	-1.06
70256	0.34	1.41	0.18
69114	-0.69	0.68	0.20
68018	0.47	-0.58	0.97
67482	-0.31	-0.98	-0.02
67182	1.96	-1.03	-0.56
65049	0.19	-1.38	-0.24
56245	-0.29	4.80	-0.35
56410	-0.65	-1.18	-1.00
53026	0.09	-0.50	-1.08
52323	-0.38	0.32	2.78
51847	0.64	-1.76	-0.83
51252	-1.14	3.28	-0.34
50588	2.86	-0.51	-0.44
49172	-0.47	-1.07	-0.11
49098	2.24	-1.55	-0.84
48416	-0.41	0.21	-1.05
41318	-0.88	0.03	0.30
40544	0.18	0.71	-0.79
37943	-0.64	-0.49	-0.64
36605	-0.46	-1.63	-0.70
36676	-0.20	-0.32	-0.24
32906	1.35	-1.77	-0.13
30537	-0.48	-0.85	-0.06
29005	-0.56	-0.70	0.20
28163	-0.76	0.69	-0.20
28118	-1.03	-0.76	-1.30
27350	0.24	0.97	-1.08
27199	-0.05	2.39	-0.05
26769	-0.69	-0.81	-0.15
26325	4.12	-1.02	0.00
26241	-1.01	-2.02	0.01
26016	0.47	-1.27	-0.50

25811	-0.49	4.54	-0.12
25540	-0.50	1.09	1.23
23949	-0.51	-0.07	0.33
23822	1.23	-1.16	-1.17
23449	-0.40	1.33	-0.45
23446	-0.42	-0.19	0.84
23397	-0.68	-0.74	0.66
23176	-0.85	-0.38	-0.11
20718	1.41	0.02	-0.04
20235	1.29	-1.36	-0.50
19721	-0.65	-1.33	2.10
18520	-0.50	-1.48	-0.21
18147	0.13	1.32	0.55
16585	0.81	-2.04	-0.73
16186	-0.55	-0.68	-0.47
16186	-0.55	-0.68	-0.47
15369	0.57	-0.33	-0.35
15277	3.54	-1.28	-0.31
15261	0.91	-2.06	-0.75
14130	0.77	-0.96	0.09
13673	-0.12	-0.50	0.06
13338	-0.37	-0.39	-0.34
12970	0.32	-0.98	-0.91
12358	0.68	-1.39	-0.16
12315	0.88	-1.52	-0.69
12195	-0.44	0.51	-0.56
11818	-0.63	-0.46	-0.30
11405	-0.50	3.21	1.10
11100	-0.35	-1.72	-0.62
11007	-0.34	-0.42	-0.25
10799	0.38	0.14	-0.52
10674	1.73	-0.86	0.40
10591	-0.30	-2.01	-1.01

counts shown are exact matches + inc	0	each miR ran	use 136 miRNAs:
0	0	2071	ID
total of totals of =100:	139641984	total all mi	total all samples =1(
UC	A_11	479320	A_11
UC	A_16	21319	A_16
UC	A_2	1102300	A_2
UC	A_4	1969971	A_4
UC	B_29	71054	B_29
UC	B_32	356562	B_32
UC	B_34	1363063	B_34
UC	C_1	824884	C_1
UC	C_40	2206918	C_40
UC	C_41	623719	C_41
UC	C_42	774447	C_42
UC	C_44	627333	C_44
UC	C_46	2772220	C_46
UC	C_47	1217409	C_47
UC	D_16	4032681	D_16
UC	D_17	1739788	D_17
UC	D_18	1227867	D_18
UC	D_36	121631	D_36
UC	D_4	2029037	D_4
UC	E_47	1734052	E_47

UC	F_43	1026638	F_43
UC	F_6	1873959	F_6
UC	F_7	1687678	F_7
UC	F_8	1238990	F_8
UC	G_33	121924	G_33
UC	G_34	416015	G_34
UC	G_8	1782397	G_8
CHR_NP	A_1	2570804	A_1
CHR_NP	A_13	371228	A_13
CHR_NP	A_14	359180	A_14
CHR_NP	A_15	111957	A_15
CHR_NP	A_17	117100	A_17
CHR_NP	B_30	148505	B_30
CHR_NP	B_33	1922117	B_33
CHR_NP	C_2	685397	C_2
CHR_NP	C_37	2915691	C_37
CHR_NP	C_38	932164	C_38
CHR_NP	C_39	148130	C_39
CHR_NP	C_43	2018570	C_43
CHR_NP	C_48	2072241	C_48
CHR_NP	D_10	727386	D_10
CHR_NP	D_12	206047	D_12
CHR_NP	D_13	2758841	D_13
CHR_NP	D_14	2173631	D_14
CHR_NP	D_3	852941	D_3
CHR_NP	D_9	239935	D_9
CHR_NP	E_20	1893208	E_20
CHR_NP	E_29	1240451	E_29
CHR_NP	E_4	2304775	E_4
CHR_NP	E_5	1597470	E_5
CHR_NP	E_6	1583268	E_6
CHR_NP	F_1	2592561	F_1
CHR_NP	F_35	3565545	F_35
CHR_NP	F_36	2585170	F_36
CHR_NP	F_37	976547	F_37
CHR_NP	F_38	1738984	F_38
CHR_NP	F_39	1856730	F_39
CHR_NP	G_3	2304707	G_3
CHR_NP	G_30	924323	G_30
CHR_NP	G_31	555209	G_31
CHR_NP	G_32	358914	G_32
CHR_NP	G_4	3926455	G_4
CHR_NP	G_5	1257195	G_5
CHR_NP	G_6	488065	G_6
CHR_NP	G_7	1421920	G_7
CHR_P	A_10	492624	A_10
CHR_P	A_12	721119	A_12
CHR_P	A_18	27468	A_18
CHR_P	A_3	816795	A_3
CHR_P	A_5	972175	A_5
CHR_P	A_6	1468719	A_6
CHR_P	B_31	409595	B_31
CHR_P	C_4	1862148	C_4
CHR_P	C_45	4820581	C_45
CHR_P	D_11	198165	D_11
CHR_P	D_35	2718285	D_35
CHR_P	D_5	905697	D_5
CHR_P	D_6	128375	D_6
CHR_P	E_19	4686947	E_19

CHR_P	E_21	1336672	E_21
CHR_P	E_22	1032612	E_22
CHR_P	E_23	1495548	E_23
CHR_P	E_3	331698	E_3
CHR_P	E_46	1653485	E_46
CHR_P	E_48	7479891	E_48
CHR_P	E_7	1160935	E_7
CHR_P	E_8	2099567	E_8
CHR_P	E_9	1323600	E_9
CHR_P	F_40	3571995	F_40
CHR_P	F_41	3076499	F_41
CHR_P	F_42	1136044	F_42
CHR_P	F_44	2580829	F_44
CHR_P	F_9	1797912	F_9
CHR_P	G_10	999373	G_10
CHR_P	G_9	336028	G_9
			UC ave
			UC sd
	min=	0.006	ttest NPvP

ALL SAMPLES FROZEN			
counts shown are exact matches + inc	0	each miR ran	use 136 miRNAs:
0	0	2071	ID
total of totals of =100:	139641984	total all mi	total all samples =1
UC	A_11	479320	A_11
UC	A_16	21319	A_16
UC	A_2	1102300	A_2
UC	A_4	1969971	A_4
UC	B_29	71054	B_29
UC	B_32	356562	B_32
UC	B_34	1363063	B_34
UC	C_1	824884	C_1
UC	C_40	2206918	C_40
UC	C_41	623719	C_41
UC	C_42	774447	C_42
UC	C_44	627333	C_44
UC	C_46	2772220	C_46
UC	C_47	1217409	C_47
UC	D_16	4032681	D_16
UC	D_17	1739788	D_17
UC	D_18	1227867	D_18
UC	D_36	121631	D_36
UC	D_4	2029037	D_4
UC	E_47	1734052	E_47
UC	F_43	1026638	F_43
UC	F_6	1873959	F_6
UC	F_7	1687678	F_7
UC	F_8	1238990	F_8
UC	G_33	121924	G_33
UC	G_34	416015	G_34
UC	G_8	1782397	G_8
CHR_NP	A_1	2570804	A_1

CHR_NP	A_13	371228	A_13
CHR_NP	A_14	359180	A_14
CHR_NP	A_15	111957	A_15
CHR_NP	A_17	117100	A_17
CHR_NP	B_30	148505	B_30
CHR_NP	B_33	1922117	B_33
CHR_NP	C_2	685397	C_2
CHR_NP	C_37	2915691	C_37
CHR_NP	[illegible]	[illegible]	[illegible]

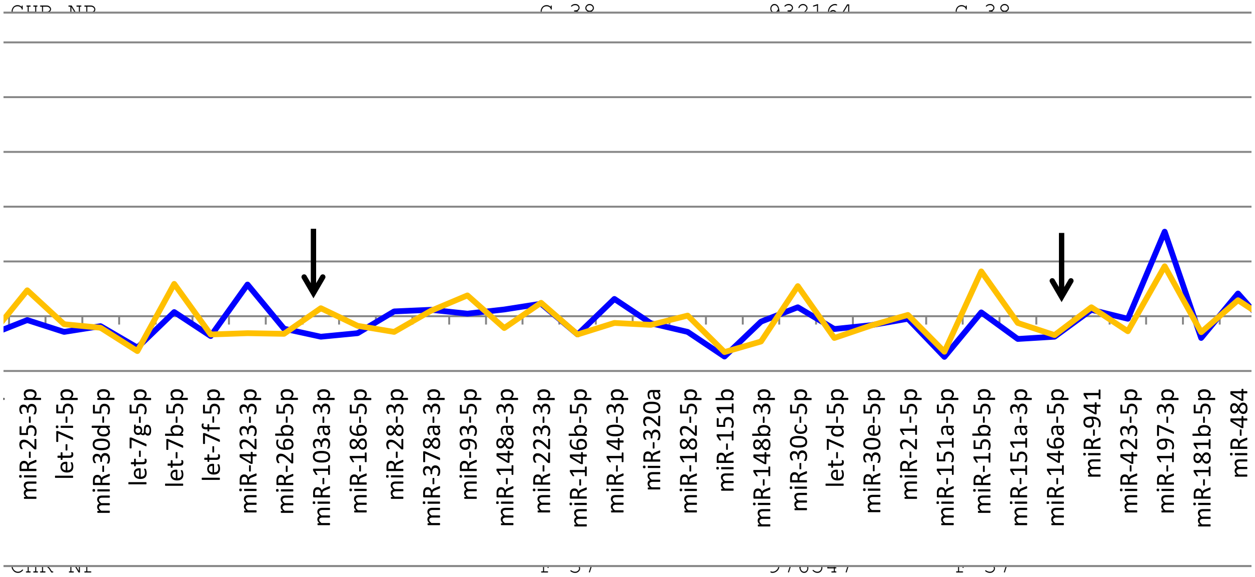

[illegible]	[illegible]	[illegible]	[illegible]
CHR_NP	F_38	1738984	F_38
CHR_NP	F_39	1856730	F_39
CHR_NP	G_3	2304707	G_3
CHR_NP	G_30	924323	G_30
CHR_NP	G_31	555209	G_31
CHR_NP	G_32	358914	G_32
CHR_NP	G_4	3926455	G_4
CHR_NP	G_5	1257195	G_5
CHR_NP	G_6	488065	G_6
CHR_NP	G_7	1421920	G_7
CHR_P	A_10	492624	A_10
CHR_P	A_12	721119	A_12
CHR_P	A_18	27468	A_18
CHR_P	A_3	816795	A_3
CHR_P	A_5	972175	A_5
CHR_P	A_6	1468719	A_6
CHR_P	B_31	409595	B_31
CHR_P	C_4	1862148	C_4
CHR_P	C_45	4820581	C_45
CHR_P	D_11	198165	D_11
CHR_P	D_35	2718285	D_35
CHR_P	D_5	905697	D_5
CHR_P	D_6	128375	D_6
CHR_P	E_19	4686947	E_19
CHR_P	E_21	1336672	E_21
CHR_P	E_22	1032612	E_22
CHR_P	E_23	1495548	E_23
CHR_P	E_3	331698	E_3
CHR_P	E_46	1653485	E_46
CHR_P	E_48	7479891	E_48
CHR_P	E_7	1160935	E_7
CHR_P	E_8	2099567	E_8

CHR_P	E_9	1323600	E_9
CHR_P	F_40	3571995	F_40
CHR_P	F_41	3076499	F_41
CHR_P	F_42	1136044	F_42
CHR_P	F_44	2580829	F_44
CHR_P	F_9	1797912	F_9
CHR_P	G_10	999373	G_10
CHR_P	G_9	336028	G_9

ALL SAMPLES FROZEN			
counts shown are exact matches + inc	0	each miR ran	use 136 miRNAs:
0	0	2071	ID
total of totals of =100:	139641984	total all mi	total all samples =1
UC	A_11	479320	A_11
UC	A_16	21319	A_16
UC	A_2	1102300	A_2
UC	A_4	1969971	A_4
UC	B_29	71054	B_29
UC	B_32	356562	B_32
UC	B_34	1363063	B_34
UC	C_1	824884	C_1
UC	C_40	2206918	C_40
UC	C_41	623719	C_41
UC	C_42	774447	C_42
UC	C_44	627333	C_44
UC	C_46	2772220	C_46
UC	C_47	1217409	C_47
UC	D_16	4032681	D_16
UC	D_17	1739788	D_17
UC	D_18	1227867	D_18
UC	D_36	121631	D_36
UC	D_4	2029037	D_4
UC	E_47	1734052	E_47
UC	F_43	1026638	F_43
UC	F_6	1873959	F_6
UC	F_7	1687678	F_7
UC	F_8	1238990	F_8
UC	G_33	121924	G_33
UC	G_34	416015	G_34
UC	G_8	1782397	G_8
CHR_NP	A_1	2570804	A_1
CHR_NP	A_13	371228	A_13
CHR_NP	A_14	359180	A_14
CHR_NP	A_15	111957	A_15
CHR_NP	A_17	117100	A_17
CHR_NP	B_30	148505	B_30
CHR_NP	B_33	1922117	B_33
CHR_NP	C_2	685397	C_2
CHR_NP	C_37	2915691	C_37
CHR_NP	C_38	932164	C_38

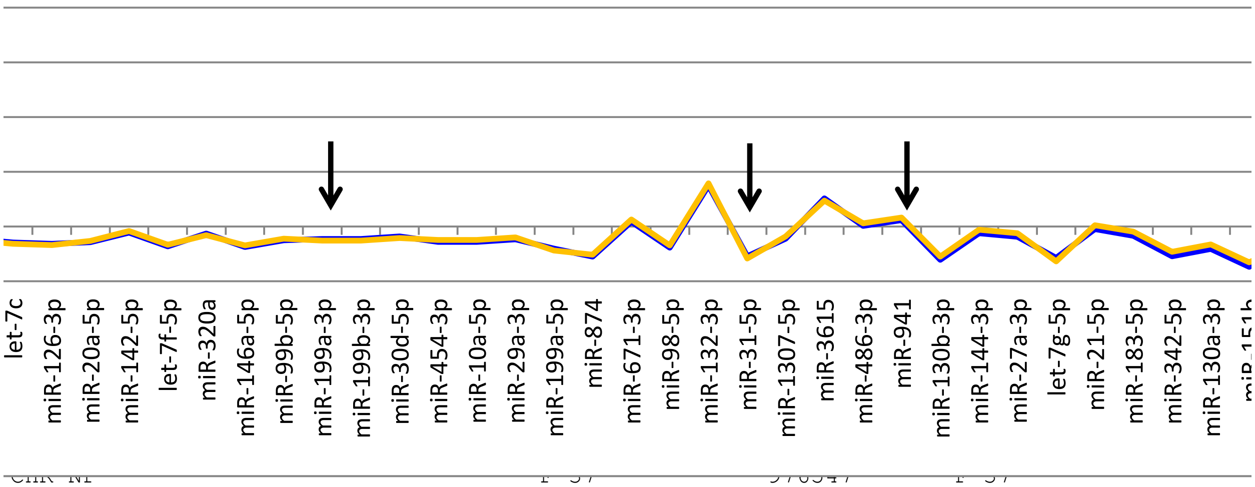

CHR_NP	F_37	[illegible]	F_37
CHR_NP	F_38	1738984	F_38
CHR_NP	F_39	1856730	F_39
CHR_NP	G_3	2304707	G_3
CHR_NP	G_30	924323	G_30
CHR_NP	G_31	555209	G_31
CHR_NP	G_32	358914	G_32
CHR_NP	G_4	3926455	G_4
CHR_NP	G_5	1257195	G_5
CHR_NP	G_6	488065	G_6
CHR_NP	G_7	1421920	G_7
CHR_P	A_10	492624	A_10
CHR_P	A_12	721119	A_12
CHR_P	A_18	27468	A_18
CHR_P	A_3	816795	A_3
CHR_P	A_5	972175	A_5
CHR_P	A_6	1468719	A_6
CHR_P	B_31	409595	B_31
CHR_P	C_4	1862148	C_4
CHR_P	C_45	4820581	C_45
CHR_P	D_11	198165	D_11
CHR_P	D_35	2718285	D_35
CHR_P	D_5	905697	D_5
CHR_P	D_6	128375	D_6
CHR_P	E_19	4686947	E_19
CHR_P	E_21	1336672	E_21
CHR_P	E_22	1032612	E_22
CHR_P	E_23	1495548	E_23
CHR_P	E_3	331698	E_3
CHR_P	E_46	1653485	E_46
CHR_P	E_48	7479891	E_48
CHR_P	E_7	1160935	E_7
CHR_P	E_8	2099567	E_8
CHR_P	E_9	1323600	E_9
CHR_P	F_40	3571995	F_40
CHR_P	F_41	3076499	F_41
CHR_P	F_42	1136044	F_42
CHR_P	F_44	2580829	F_44
CHR_P	F_9	1797912	F_9
CHR_P	G_10	999373	G_10
CHR_P	G_9	336028.00	G_9

21 are >= 1.1; the five are <= 1.0

abs delta
absdelt/(abs+abs)

-0.77215 b=3.0153

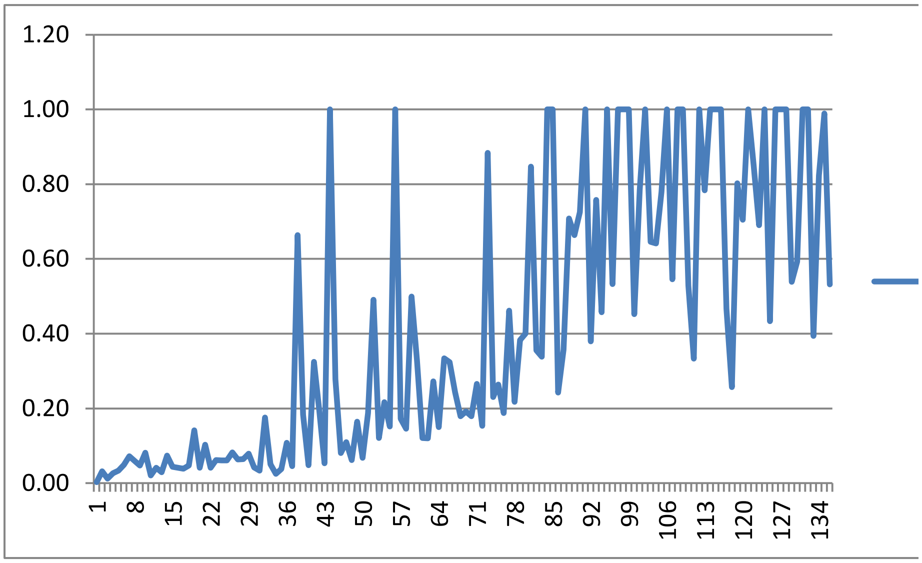

; dup:

1969971	14538	71054
	1	
	1	
4	5	6
4	5	6
A_4	A_7	B_29
UC	UC	UC
8888098	142388	216489
0.87	0.69	1.00
A_4	A_7	B_29
6918127	127850	145435
1083588	3496	3861
51322	288	11755
49632	1322	10912
143882	383	10803
65367	966	4310
34942	234	3715
36120	120	5832
7608	299	150
37604	104	180
80209	1957	824
50730	980	561
26927	181	928
33640	69	1497
40345	333	1133
8967	190	606
9272	98	108
6907	26	819
11271	82	730
6868	46	276
9093	124	357
7556	71	409
16114	66	186
5128	233	56
4274	104	416
3875	81	440
12666	144	44
7317	129	230
5860	673	73
5305	52	543
4367	13	84
3309	37	233
8149	24	372
2955	11	125
838	38	136
3526	39	493
7659	20	1914
2236	24	306
3355	149	505
3451	4	352
4517	29	43
2457	38	357
2054	40	174
3384	13	321
808	16	58
2367	171	394

2546	19	83
1557	12	57
3411	34	52
1115	0	4
421	107	7
909	7	98
602	33	338
1823	21	107
1682	183	32
1231	33	1
254	28	3
1262	3	125
758	5	56
1404	6	144
1169	18	299
801	4	64
509	11	35
164	6	11
32	1	4
1947	4	14
792	3	58
310	23	20
547	2	21
588	15	54
1901	3	39
385	7	96
1954	2	32
346	9	20
1125	14	0
361	4	70
354	8	31
666	6	1
248	32	60
449	4	77
176	4	93
1112	1	31
504	12	2
550	0	58
73	27	9
276	6	13
272	17	8
416	8	39
286	2	16
296	0	28
436	0	42
238	8	11
23	0	1
559	2	4
225	6	8
78	2	5
128	7	31
165	2	33
306	10	4
339	1	28
57	0	7
343	0	13
196	0	7
210	4	29
373	8	17

318	1	9
240	7	3
101	6	28
91	0	71
196	0	14
121	8	6
104	1	6
230	2	9
144	9	7
306	0	9
92	2	19
285	1	8
91	3	1
114	0	16
114	0	16
140	1	41
285	5	2
234	1	4
53	2	0
128	9	4
7	0	1
134	2	22
77	1	14
100	1	2
68	6	14
179	0	23
51	0	2
82	4	3
47	1	2
85	5	29
93	0	0
43	3	5
102	16	2
197	0	2
127	0	3
69	1	8
119	5	3
55	0	8
112	11	6
60	1	12
8	1	0
151	0	0
108	0	9
37	1	4
114	0	16
129	2	12
32	1	0
69	0	2
33	2	0
75	0	5
22	11	0
99	0	4
49	1	2
33	4	2
71	3	3
258	1	1
4	0	0
66	0	4
55	0	8

46	0	3
30	0	4
141	1	0
47	0	9
23	3	2
5	0	1
115	2	0
0	0	4
93	5	0
65	1	2
33	1	11
18	0	1
37	2	9
18	2	14
25	0	1
59	0	3
15	0	4
81	0	1
19	1	0
26	6	4
25	0	2
33	0	4
68	0	1
19	0	0
26	0	1
10	0	6
15	0	2
36	0	1
5	0	0
26	0	6
52	0	4
81	0	2
31	0	3
9	2	0
87	1	2
23	1	3
142	0	1
39	0	0
27	0	11
44	0	4
30	3	0
42	0	1
32	0	0
57	0	1
40	0	0
7	0	2
93	6	1
33	0	8
24	0	1
7	0	1
54	1	4
18	1	0
12	0	1
23	0	2
16	1	1
2	0	0
62	0	3
14	1	3
39	0	0

27	1	3
35	2	1
15	0	1
22	0	2
37	1	1
59	1	0
27	0	3
31	0	3
6	0	0
20	0	1
0	0	0
22	1	1
28	0	0
39	0	0
14	0	2
15	1	2
8	0	4
30	0	0
13	0	2
13	0	0
4	0	1
8	1	3
8	1	3
14	1	1
24	0	2
10	0	1
6	1	0
22	0	3
16	1	1
21	4	0
31	0	2
11	0	0
10	0	2
11	0	1
8	2	0
18	0	3
1	0	0
34	0	0
16	0	1
13	0	1
40	0	2
16	0	1
16	3	0
16	0	0
35	0	0
21	0	2
1	0	0
6	0	0
32	0	1
43	0	0
7	0	1
4	0	0
4	0	0
17	0	3
11	0	0
5	0	1
29	0	0
14	0	6
11	0	4

8	0	1
9	0	2
8	0	0
15	1	0
10	0	3
10	0	1
10	0	0
4	2	0
2	0	0
6	0	1
3	0	2
10	1	0
12	0	0
6	1	0
4	2	0
5	0	0
15	0	0
6	0	0
11	0	0
4	0	0
5	0	1
13	0	1
13	0	0
10	1	0
3	0	1
17	0	0
7	0	1
5	0	1
12	0	0
9	0	0
9	0	0
5	0	0
0	0	1
6	0	0
13	0	2
16	0	0
6	0	0
13	0	0
4	0	1
10	0	0
7	0	0
7	0	1
7	0	0
8	1	1
0	0	0
0	1	0
10	0	1
15	1	1
1	1	3
15	0	2
11	0	1
1	0	0
5	0	0
1	0	1
4	0	0
7	0	0
13	0	0
1	0	1
10	1	0

11	1	0
10	1	0
8	0	0
2	0	0
3	1	1
9	0	0
12	0	0
0	0	1
13	0	0
8	1	1
8	0	0
8	0	0
3	0	0
1	0	0
7	0	0
7	0	0
6	0	0
9	0	0
2	0	0
3	0	0
1	0	0
1	0	0
4	0	0
7	0	0
12	0	0
5	0	2
4	0	0
0	0	0
2	0	0
2	0	0
2	0	0
4	0	1
5	0	0
3	0	1
1	0	0
3	0	0
1	0	1
7	0	0
4	0	0
1	0	0
3	0	0
2	0	0
2	0	0
3	0	0
3	0	0
1	0	0
4	0	0
3	0	0
1	0	0
0	0	1
6	0	0
1	0	0
0	0	0
3	0	0
6	0	1
4	0	0
2	0	0
5	0	0
3	0	0

7	0	0
0	0	0
4	0	0
0	0	0
3	0	0
4	1	0
0	0	0
2	0	0
0	0	0
4	0	0
2	0	0
0	0	0
0	0	0
6	0	0
2	0	0
1	0	0
3	0	0
0	0	1
0	0	0
0	0	0
0	0	0
0	0	0
1	0	0
0	0	0
3	0	0
0	0	0
3	0	0
2	0	0
2	0	0
0	0	0
0	0	0
1	0	0
5	1	2
4	0	0
2	0	1
0	0	0
3	0	0
0	0	0
0	1	0
4	0	0
0	0	0
1	0	0
4	0	0
1	0	0
5	0	0
1	0	0
1	0	0
2	0	0
5	0	0
2	0	0
2	0	0
3	0	0
3	0	0
5	0	0
2	0	0
1	0	0
0	0	0
1	0	0
4	0	1

0	0	0
1	0	0
1	0	0
0	0	0
1	0	0
1	0	0
1	0	0
0	0	0
0	0	0
0	0	0
0	0	0
3	0	0
0	0	0
1	0	1
5	0	0
2	0	0
1	0	0
0	0	0
0	0	0
1	0	1
2	0	0
0	0	0
1	0	0
1	0	1
0	0	0
0	0	0
1	0	0
1	0	0
0	0	0
1	0	0
0	0	0
4	0	0
1	0	0
1	0	0
0	0	0
1	0	0
7	0	0
1	0	0
0	0	0
3	0	0
2	0	0
3	0	0
0	0	0
3	0	0
0	0	0
4	0	0
0	0	0
4	1	0
3	0	0
2	0	1
2	0	0
0	0	0
2	0	0
1	0	0
1	0	0
0	0	0
2	0	0
1	0	0
3	0	0

3	0	0
0	1	0
1	0	0
0	0	0
0	0	0
0	0	0
1	0	0
1	0	0
0	0	0
3	0	0
1	0	0
4	1	0
0	9	3
A_4	A_7	B_29
UC	UC	UC
6918127	127850	145435
24	28	22
0.64	1.43	0.08

0.59	0.75	0.23
12	8	23
1	1	1
4	5	6
4	5	6
A_4	B_29	B_32
UC	UC	UC
1969971	71054	356562
A_4	B_29	B_32
1083588	3861	165954
51322	11755	17347
49632	10912	11202
143882	10803	37642
65367	4310	19686
34942	3715	6653
36120	5832	16299
7608	150	126
37604	180	2890
80209	824	7519
50730	561	12213
26927	928	3540
33640	1497	4451
40345	1133	4350
8967	606	2342
9272	108	7215
6907	819	1156
11271	730	1114
6868	276	730

9093	357	2828
7556	409	1372
16114	186	725
5128	56	2398
4274	416	265
3875	440	703
12666	44	840
7317	230	1234
5860	73	129
5305	543	946
4367	84	1462
3309	233	444
8149	372	1040
2955	125	312
838	136	309
3526	493	855
7659	1914	922
2236	306	643
3355	505	980
3451	352	1148
4517	43	749
2457	357	182
2054	174	886
3384	321	560
808	58	105
2367	394	221
2546	83	726
1557	57	619
3411	52	398
1115	4	77
421	7	351
909	98	365
602	338	218
1823	107	276
1682	32	270
1231	1	339
254	3	26
1262	125	228
758	56	150
1404	144	571
1169	299	252
801	64	199
509	35	162
164	11	33
32	4	13
1947	14	603
792	58	64
310	20	26
547	21	26
588	54	35
1901	39	23
385	96	711
1954	32	277
346	20	55
1125	0	78
361	70	53
354	31	225
666	1	112
248	60	60

449	77	221
176	93	154
1112	31	74
504	2	94
550	58	171
73	9	77
276	13	48
272	8	36
416	39	181
286	16	12
296	28	155
436	42	126
238	11	62
23	1	5
559	4	70
225	8	28
78	5	15
128	31	65
165	33	183
306	4	62
339	28	34
57	7	16
343	13	2
196	7	71
210	29	140
373	17	52
318	9	49
240	3	11
101	28	28
91	71	55
196	14	32
121	6	12
104	6	9
230	9	18
144	7	10
306	9	10
92	19	22
285	8	40
91	1	25
114	16	34
114	16	34
140	41	15
285	2	25
234	4	49
53	0	9
128	4	13
7	1	2
134	22	102
77	14	14
100	2	16
68	14	5
179	23	18
51	2	0
82	3	25
47	2	21
85	29	22
93	0	9
43	5	41
102	2	27

197	2	3
127	3	12
69	8	10
119	3	50
55	8	9
112	6	22
60	12	25
8	0	2
151	0	18
108	9	35
37	4	8
114	16	34
129	12	7
32	0	3
69	2	4
33	0	7
75	5	27
22	0	2
99	4	15
49	2	1
33	2	2
71	3	3
258	1	5
4	0	8
66	4	6
55	8	18
46	3	6
30	4	6
141	0	34
47	9	4
23	2	14
5	1	1
115	0	8
0	4	6
93	0	25
65	2	27
33	11	23
18	1	6
37	9	9
18	14	28
25	1	20
59	3	2
15	4	3
81	1	0
19	0	2
26	4	2
25	2	1
33	4	5
68	1	19
19	0	2
26	1	17
10	6	36
15	2	8
36	1	0
5	0	3
26	6	4
52	4	50
81	2	4
31	3	2

9	0	2
87	2	6
23	3	2
142	1	0
39	0	0
27	11	31
44	4	2
30	0	7
42	1	3
32	0	9
57	1	4
40	0	5
7	2	1
93	1	6
33	8	4
24	1	15
7	1	3
54	4	19
18	0	5
12	1	21
23	2	6
16	1	7
2	0	0
62	3	1
14	3	1
39	0	4
27	3	1
35	1	4
15	1	3
22	2	12
37	1	5
59	0	33
27	3	6
31	3	2
6	0	1
20	1	13
0	0	0
22	1	2
28	0	5
39	0	0
14	2	1
15	2	0
8	4	2
30	0	4
13	2	5
13	0	4
4	1	0
8	3	2
8	3	2
14	1	1
24	2	0
10	1	1
6	0	1
22	3	4
16	1	4
21	0	3
31	2	1
11	0	1
10	2	5

11	1	4
8	0	3
18	3	2
1	0	0
34	0	5
16	1	0
13	1	4
40	2	7
16	1	0
16	0	2
16	0	1
35	0	1
21	2	9
1	0	0
6	0	3
32	1	13
43	0	3
7	1	1
4	0	1
4	0	0
17	3	2
11	0	3
5	1	0
29	0	1
14	6	1
11	4	0
8	1	0
9	2	3
8	0	4
15	0	7
10	3	4
10	1	3
10	0	3
4	0	4
2	0	0
6	1	1
3	2	0
10	0	1
12	0	3
6	0	0
4	0	3
5	0	2
15	0	1
6	0	1
11	0	1
4	0	2
5	1	5
13	1	2
13	0	0
10	0	1
3	1	3
17	0	1
7	1	5
5	1	2
12	0	4
9	0	1
9	0	0
5	0	0
0	1	0

6	0	0
13	2	3
16	0	1
6	0	0
13	0	1
4	1	1
10	0	2
7	0	3
7	1	3
7	0	2
8	1	3
0	0	3
0	0	0
10	1	3
15	1	4
1	3	2
15	2	1
11	1	1
1	0	0
5	0	5
1	1	0
4	0	1
7	0	0
13	0	3
1	1	0
10	0	1
11	0	2
10	0	6
8	0	0
2	0	2
3	1	4
9	0	0
12	0	2
0	1	0
13	0	0
8	1	1
8	0	2
8	0	2
3	0	0
1	0	0
7	0	3
7	0	1
6	0	1
9	0	0
2	0	0
3	0	0
1	0	0
1	0	0
4	0	1
7	0	2
12	0	2
5	2	7
4	0	0
0	0	1
2	0	0
2	0	1
2	0	0
4	1	2
5	0	0

3	1	0
1	0	0
3	0	3
1	1	1
7	0	1
4	0	5
1	0	0
3	0	2
2	0	0
2	0	0
3	0	1
3	0	0
1	0	0
4	0	0
3	0	1
1	0	0
0	1	0
6	0	1
1	0	0
0	0	0
3	0	0
6	1	2
4	0	0
2	0	1
5	0	0
3	0	0
7	0	0
0	0	0
4	0	2
0	0	0
3	0	0
4	0	0
0	0	0
2	0	5
0	0	1
4	0	0
2	0	0
0	0	0
0	0	0
6	0	0
2	0	1
1	0	1
3	0	2
0	1	3
0	0	0
0	0	0
0	0	0
0	0	1
1	0	0
0	0	2
3	0	0
0	0	0
3	0	0
2	0	2
2	0	0
0	0	0
0	0	0
1	0	0
5	2	2

4	0	0
2	1	0
0	0	1
3	0	2
0	0	0
0	0	0
4	0	1
0	0	0
1	0	2
4	0	3
1	0	0
5	0	2
1	0	0
1	0	1
2	0	0
5	0	0
2	0	0
2	0	1
3	0	0
3	0	0
5	0	1
2	0	0
1	0	1
0	0	0
1	0	0
4	1	0
0	0	0
1	0	0
1	0	2
0	0	0
1	0	0
1	0	1
1	0	0
0	0	0
0	0	0
0	0	0
0	0	0
3	0	0
0	0	0
1	1	1
5	0	0
2	0	0
1	0	0
0	0	0
0	0	0
1	1	0
2	0	0
0	0	0
1	0	0
1	1	1
0	0	3
0	0	0
1	0	0
1	0	0
0	0	0
1	0	0
0	0	0
4	0	0
1	0	0

1	0	0
0	0	0
1	0	0
7	0	0
1	0	0
0	0	0
3	0	1
2	0	1
3	0	0
0	0	1
3	0	0
0	0	0
4	0	0
0	0	1
4	0	0
3	0	0
2	1	0
2	0	1
0	0	0
2	0	0
1	0	1
1	0	0
0	0	0
2	0	0
1	0	0
3	0	1
3	0	0
0	0	0
1	0	0
0	0	0
0	0	0
0	0	0
1	0	0
1	0	0
0	0	0
3	0	4
1	0	0
4	0	1

0.59	0.75	0.23
12	8	23
1	1	1
4	5	6
4	5	6
A_4	B_29	B_32
UC	UC	UC
1969971	71054	356562
UC	UC	UC
1083588	3861	165954
51322	11755	17347
49632	10912	11202
143882	10803	37642
65367	4310	19686

34942	3715	6653
36120	5832	16299
7608	150	126
37604	180	2890
80209	824	7519
50730	561	12213
26927	928	3540
33640	1497	4451
40345	1133	4350
8967	606	2342
9272	108	7215
6907	819	1156
11271	730	1114
6868	276	730
9093	357	2828
7556	409	1372
16114	186	725
5128	56	2398
4274	416	265
3875	440	703
12666	44	840
7317	230	1234
5860	73	129
5305	543	946
4367	84	1462
3309	233	444
8149	372	1040
2955	125	312
838	136	309
3526	493	855
7659	1914	922
2236	306	643
3355	505	980
3451	352	1148
4517	43	749
2457	357	182
2054	174	886
3384	321	560
808	58	105
2367	394	221
2546	83	726
1557	57	619
3411	52	398
1115	4	77
421	7	351
909	98	365
602	338	218
1823	107	276
1682	32	270
1231	1	339
254	3	26
1262	125	228
758	56	150
1404	144	571
1169	299	252
801	64	199
509	35	162
164	11	33
32	4	13

1947	14	603
792	58	64
310	20	26
547	21	26
588	54	35
1901	39	23
385	96	711
1954	32	277
346	20	55
1125	0	78
361	70	53
354	31	225
666	1	112
248	60	60
449	77	221
176	93	154
1112	31	74
504	2	94
550	58	171
73	9	77
276	13	48
272	8	36
416	39	181
286	16	12
296	28	155
436	42	126
238	11	62
23	1	5
559	4	70
225	8	28
78	5	15
128	31	65
165	33	183
306	4	62
339	28	34
57	7	16
343	13	2
196	7	71
210	29	140
373	17	52
318	9	49
240	3	11
101	28	28
91	71	55
196	14	32
121	6	12
104	6	9
230	9	18
144	7	10
306	9	10
92	19	22
285	8	40
91	1	25
114	16	34
114	16	34
140	41	15
285	2	25
234	4	49
53	0	9

128	4	13
7	1	2
134	22	102
77	14	14
100	2	16
68	14	5
179	23	18
51	2	0
82	3	25
47	2	21
85	29	22
93	0	9
43	5	41
102	2	27
197	2	3
127	3	12
69	8	10
119	3	50
55	8	9
112	6	22
60	12	25
8	0	2
151	0	18
108	9	35
37	4	8
114	16	34
129	12	7
32	0	3
69	2	4
33	0	7
75	5	27
22	0	2
99	4	15
49	2	1
33	2	2
71	3	3
258	1	5
4	0	8
66	4	6
55	8	18
46	3	6
30	4	6
141	0	34
47	9	4
23	2	14
5	1	1
115	0	8
0	4	6
93	0	25
65	2	27
33	11	23
18	1	6
37	9	9
18	14	28
25	1	20
59	3	2
15	4	3
81	1	0
19	0	2

26	4	2
25	2	1
33	4	5
68	1	19
19	0	2
26	1	17
10	6	36
15	2	8
36	1	0
5	0	3
26	6	4
52	4	50
81	2	4
31	3	2
9	0	2
87	2	6
23	3	2
142	1	0
39	0	0
27	11	31
44	4	2
30	0	7
42	1	3
32	0	9
57	1	4
40	0	5
7	2	1
93	1	6
33	8	4
24	1	15
7	1	3
54	4	19
18	0	5
12	1	21
23	2	6
16	1	7
2	0	0
62	3	1
14	3	1
39	0	4
27	3	1
35	1	4
15	1	3
22	2	12
37	1	5
59	0	33
27	3	6
31	3	2
6	0	1
20	1	13
0	0	0
22	1	2
28	0	5
39	0	0
14	2	1
15	2	0
8	4	2
30	0	4
13	2	5

13	0	4
4	1	0
8	3	2
8	3	2
14	1	1
24	2	0
10	1	1
6	0	1
22	3	4
16	1	4
21	0	3
31	2	1
11	0	1
10	2	5
11	1	4
8	0	3
18	3	2
1	0	0
34	0	5
16	1	0
13	1	4
40	2	7
16	1	0
16	0	2
16	0	1
35	0	1
21	2	9
1	0	0
6	0	3
32	1	13
43	0	3
7	1	1
4	0	1
4	0	0
17	3	2
11	0	3
5	1	0
29	0	1
14	6	1
11	4	0
8	1	0
9	2	3
8	0	4
15	0	7
10	3	4
10	1	3
10	0	3
4	0	4
2	0	0
6	1	1
3	2	0
10	0	1
12	0	3
6	0	0
4	0	3
5	0	2
15	0	1
6	0	1
11	0	1

4	0	2
5	1	5
13	1	2
13	0	0
10	0	1
3	1	3
17	0	1
7	1	5
5	1	2
12	0	4
9	0	1
9	0	0
5	0	0
0	1	0
6	0	0
13	2	3
16	0	1
6	0	0
13	0	1
4	1	1
10	0	2
7	0	3
7	1	3
7	0	2
8	1	3
0	0	3
0	0	0
10	1	3
15	1	4
1	3	2
15	2	1
11	1	1
1	0	0
5	0	5
1	1	0
4	0	1
7	0	0
13	0	3
1	1	0
10	0	1
11	0	2
10	0	6
8	0	0
2	0	2
3	1	4
9	0	0
12	0	2
0	1	0
13	0	0
8	1	1
8	0	2
8	0	2
3	0	0
1	0	0
7	0	3
7	0	1
6	0	1
9	0	0
2	0	0

3	0	0
1	0	0
1	0	0
4	0	1
7	0	2
12	0	2
5	2	7
4	0	0
0	0	1
2	0	0
2	0	1
2	0	0
4	1	2
5	0	0
3	1	0
1	0	0
3	0	3
1	1	1
7	0	1
4	0	5
1	0	0
3	0	2
2	0	0
2	0	0
3	0	1
3	0	0
1	0	0
4	0	0
3	0	1
1	0	0
0	1	0
6	0	1
1	0	0
0	0	0
3	0	0
6	1	2
4	0	0
2	0	1
5	0	0
3	0	0
7	0	0
0	0	0
4	0	2
0	0	0
3	0	0
4	0	0
0	0	0
2	0	5
0	0	1
4	0	0
2	0	0
0	0	0
0	0	0
6	0	0
2	0	1
1	0	1
3	0	2
0	1	3
0	0	0

0	0	0
0	0	0
0	0	1
1	0	0
0	0	2
3	0	0
0	0	0
3	0	0
2	0	2
2	0	0
0	0	0
0	0	0
1	0	0
5	2	2
4	0	0
2	1	0
0	0	1
3	0	2
0	0	0
0	0	0
4	0	1
0	0	0
1	0	2
4	0	3
1	0	0
5	0	2
1	0	0
1	0	1
2	0	0
5	0	0
2	0	0
2	0	1
3	0	0
3	0	0
5	0	1
2	0	0
1	0	1
0	0	0
1	0	0
4	1	0
0	0	0
1	0	0
1	0	2
0	0	0
1	0	0
1	0	1
1	0	0
0	0	0
0	0	0
0	0	0
0	0	0
3	0	0
0	0	0
1	1	1
5	0	0
2	0	0
1	0	0
0	0	0
0	0	0

1	1	0
2	0	0
0	0	0
1	0	0
1	1	1
0	0	3
0	0	0
1	0	0
1	0	0
0	0	0
1	0	0
0	0	0
4	0	0
1	0	0
1	0	0
0	0	0
1	0	0
7	0	0
1	0	0
0	0	0
3	0	1
2	0	1
3	0	0
0	0	1
3	0	0
0	0	0
4	0	0
0	0	1
4	0	0
3	0	0
2	1	0
2	0	1
0	0	0
2	0	0
1	0	1
1	0	0
0	0	0
2	0	0
1	0	0
3	0	1
3	0	0
0	0	0
1	0	0
0	0	0
0	0	0
0	0	0
1	0	0
1	0	0
0	0	0
3	0	4
1	0	0
4	0	1
62225	2061	11178
195264	3404	30332
399	485	455
UC	UC	UC
UC	UC	UC

4	5	6
4	5	6
UC	UC	UC
A_4	B_29	B_32
1969971	71054	356562
UC	UC	UC
17.41	1.87	14.85
0.82	5.70	1.55
0.80	5.29	1.00
2.31	5.24	3.37
1.05	2.09	1.76
0.56	1.80	0.60
0.58	2.83	1.46
0.12	0.07	0.01
0.60	0.09	0.26
1.29	0.40	0.67
0.82	0.27	1.09
0.43	0.45	0.32
0.54	0.73	0.40
0.65	0.55	0.39
0.14	0.29	0.21
0.15	0.05	0.65
0.11	0.40	0.10
0.18	0.35	0.10
0.11	0.13	0.07
0.15	0.17	0.25
0.12	0.20	0.12
0.26	0.09	0.06
0.08	0.03	0.21
0.07	0.20	0.02
0.06	0.21	0.06
0.20	0.02	0.08
0.12	0.11	0.11
0.09	0.04	0.01
0.09	0.26	0.08
0.07	0.04	0.13
0.05	0.11	0.04
0.13	0.18	0.09
0.05	0.06	0.03
0.01	0.07	0.03
0.06	0.24	0.08
0.12	0.93	0.08
0.04	0.15	0.06
0.05	0.24	0.09
0.06	0.17	0.10
0.07	0.02	0.07
0.04	0.17	0.02
0.03	0.08	0.08
0.05	0.16	0.05
0.01	0.03	0.01
0.04	0.19	0.02
0.04	0.04	0.06
0.03	0.03	0.06
0.05	0.03	0.04
0.02	0.00	0.01
0.01	0.00	0.03
0.01	0.05	0.03
0.01	0.16	0.02

0.03	0.05	0.02
0.03	0.02	0.02
0.02	0.00	0.03
0.00	0.00	0.00
0.02	0.06	0.02
0.01	0.03	0.01
0.02	0.07	0.05
0.02	0.15	0.02
0.01	0.03	0.02
0.01	0.02	0.01
0.00	0.01	0.00
0.00	0.00	0.00
0.03	0.01	0.05
0.01	0.03	0.01
0.00	0.01	0.00
0.01	0.01	0.00
0.01	0.03	0.00
0.03	0.02	0.00
0.01	0.05	0.06
0.03	0.02	0.02
0.01	0.01	0.00
0.02	0.00	0.01
0.01	0.03	0.00
0.01	0.02	0.02
0.01	0.00	0.01
0.00	0.03	0.01
0.01	0.04	0.02
0.00	0.05	0.01
0.02	0.02	0.01
0.01	0.00	0.01
0.01	0.03	0.02
0.00	0.00	0.01
0.00	0.01	0.00
0.00	0.00	0.00
0.01	0.02	0.02
0.00	0.01	0.00
0.00	0.01	0.01
0.01	0.02	0.01
0.00	0.01	0.01
0.00	0.00	0.00
0.01	0.00	0.01
0.00	0.00	0.00
0.00	0.00	0.00
0.00	0.02	0.01
0.00	0.02	0.02
0.00	0.00	0.01
0.01	0.01	0.00
0.00	0.00	0.00
0.01	0.01	0.00
0.00	0.00	0.01
0.00	0.01	0.01
0.01	0.01	0.00
0.01	0.00	0.00
0.00	0.00	0.00
0.00	0.01	0.00
0.00	0.03	0.00
0.00	0.01	0.00
0.00	0.00	0.00
0.00	0.00	0.00

0.00	0.00	0.00
0.00	0.00	0.00
0.00	0.00	0.00
0.00	0.01	0.00
0.00	0.00	0.00
0.00	0.00	0.00
0.00	0.01	0.00
0.00	0.01	0.00
0.00	0.02	0.00
0.00	0.00	0.00
0.00	0.00	0.00
0.00	0.00	0.00
0.00	0.00	0.00
0.00	0.00	0.00
0.00	0.01	0.01
0.00	0.01	0.00
0.00	0.00	0.00
0.00	0.01	0.00
0.00	0.01	0.00
0.00	0.00	0.00
0.00	0.00	0.00
0.00	0.00	0.00
0.00	0.01	0.00
0.00	0.00	0.00
0.00	0.00	0.00
0.00	0.00	0.00
0.00	0.00	0.00
0.00	0.00	0.00
0.00	0.00	0.00
0.00	0.00	0.00
0.00	0.00	0.00
0.00	0.00	0.00
0.00	0.01	0.00
0.00	0.00	0.00
0.00	0.00	0.00
0.00	0.00	0.00
0.00	0.00	0.00
0.00	0.01	0.00
0.00	0.01	0.00
0.00	0.00	0.00
0.00	0.00	0.00
0.00	0.00	0.00
0.00	0.00	0.00
0.00	0.00	0.00
0.00	0.00	0.00
0.00	0.00	0.00
0.00	0.00	0.00
0.00	0.00	0.00
0.00	0.00	0.00
0.00	0.00	0.00
0.00	0.00	0.00
0.00	0.00	0.00
0.00	0.00	0.00
0.00	0.00	0.00
0.00	0.00	0.00
0.00	0.00	0.00
0.00	0.00	0.00
0.00	0.00	0.00
0.00	0.00	0.00

0.00	0.00	0.00
0.00	0.00	0.00
0.00	0.00	0.00
0.00	0.01	0.00
0.00	0.00	0.00
0.00	0.00	0.00
0.00	0.01	0.00
0.00	0.00	0.00
0.00	0.00	0.00
0.00	0.00	0.00
0.00	0.00	0.00
0.00	0.00	0.00
0.00	0.00	0.00
0.00	0.00	0.00
0.00	0.00	0.00
0.00	0.00	0.00
0.00	0.00	0.00
0.00	0.00	0.00
0.00	0.00	0.00
0.00	0.00	0.00
0.00	0.00	0.00
0.00	0.00	0.00
0.00	0.00	0.00
0.00	0.00	0.00
0.00	0.00	0.00
0.00	0.00	0.00
0.00	0.00	0.00
0.00	0.00	0.00
0.00	0.00	0.00
0.00	0.00	0.00
0.00	0.00	0.00
0.00	0.01	0.00
0.00	0.00	0.00
0.00	0.00	0.00
0.00	0.00	0.00
0.00	0.00	0.00
0.00	0.00	0.00
0.00	0.00	0.00
0.00	0.00	0.00
0.00	0.00	0.00
0.00	0.00	0.00
0.00	0.00	0.00
0.00	0.00	0.00
0.00	0.00	0.00
0.00	0.00	0.00
0.00	0.00	0.00
0.00	0.00	0.00
0.00	0.00	0.00
0.00	0.00	0.00
0.00	0.00	0.00
0.00	0.00	0.00
0.00	0.00	0.00
0.00	0.00	0.00
0.00	0.00	0.00
0.00	0.00	0.00
0.00	0.00	0.00
0.00	0.00	0.00
0.00	0.00	0.00
0.00	0.00	0.00

0.00	0.00	0.00
0.00	0.00	0.00
0.00	0.00	0.00
0.00	0.00	0.00
0.00	0.00	0.00
0.00	0.00	0.00
0.00	0.00	0.00
0.00	0.00	0.00
0.00	0.00	0.00
0.00	0.00	0.00
0.00	0.00	0.00
0.00	0.00	0.00
0.00	0.00	0.00
0.00	0.00	0.00
0.00	0.00	0.00
0.00	0.00	0.00
0.00	0.00	0.00
0.00	0.00	0.00
0.00	0.00	0.00
0.00	0.00	0.00
0.00	0.00	0.00
0.00	0.00	0.00
0.00	0.00	0.00
0.00	0.00	0.00
0.00	0.00	0.00
0.00	0.00	0.00
0.00	0.00	0.00
0.00	0.00	0.00
0.00	0.00	0.00
0.00	0.00	0.00

4	5	6
4	6	7
UC	UC	UC
A_4	B_29	B_32
1969971	71054	356562
A_4	B_29	B_32
1.30	-2.19	0.72
-0.72	0.71	-0.50
-0.99	2.40	-0.83
-0.13	2.76	0.91
-1.23	-0.08	-0.44
-0.57	0.55	-0.54
-0.93	2.65	0.47
-0.40	-0.49	-0.60

0.03	-1.00	-0.66
1.36	-0.92	-0.22
1.29	-0.75	2.33
-0.53	-0.37	-1.57
1.05	2.12	0.23
1.04	0.48	-0.45
-0.35	0.34	-0.04
-0.64	-1.06	1.52
-0.29	1.51	-0.34
-0.33	1.38	-1.13
-0.64	-0.43	-1.04
-0.84	-0.57	0.22
-0.58	0.66	-0.55
1.64	-0.94	-1.32
-0.45	-1.13	1.18
-0.48	0.78	-0.90
-0.40	0.49	-0.40
1.67	-1.76	-0.75
-0.33	-0.42	-0.44
-0.18	-0.74	-0.97
-0.43	3.04	-0.44
-0.75	-1.43	0.67
-0.87	0.52	-1.19
0.18	1.13	-0.56
-0.18	0.02	-0.48
-0.33	-0.04	-0.25
-0.85	3.24	-0.40
-0.12	4.79	-0.37
-0.91	0.42	-0.66
-0.40	1.57	-0.05
-0.53	0.86	0.04
-0.22	-0.64	-0.26
-0.75	1.99	-1.22
-1.07	0.47	0.32
-0.40	3.70	-0.57
-0.61	-0.23	-0.70
-0.52	2.74	-0.91
-0.40	-0.45	1.34
-0.64	-0.53	0.59
0.17	-0.81	-0.47
0.23	-0.33	-0.16
-0.54	-0.61	0.03
-1.12	0.66	-0.15
-0.72	3.79	-0.43
-0.49	1.26	-0.85
-0.11	-0.71	-0.26
-0.29	-1.36	0.30

-0.22	-0.32	-0.29
-0.63	2.63	-0.62
-0.98	0.26	-0.88
-0.60	2.92	1.53
-0.58	2.75	-0.48
-0.94	0.52	-0.54
-1.15	-0.42	-0.62
-0.52	-0.31	-0.50
-0.27	-0.21	-0.24
0.11	-1.30	1.41
-0.36	1.12	-1.04
-0.52	-0.18	-0.72
-0.16	-0.02	-0.80
-0.38	1.53	-1.10
-0.03	-0.18	-0.40
-1.08	1.76	2.96
1.09	-0.48	0.43
-0.42	-0.04	-0.48
0.55	-1.82	-0.91
-0.65	2.85	-0.78
-0.95	-0.18	0.24
-0.46	-1.70	-0.54
-0.46	0.21	-0.42
-0.95	1.81	0.20
-1.12	2.84	-0.09
1.35	0.79	-0.86
-0.60	-1.62	-0.56
-0.71	1.07	-0.12
-0.46	-0.31	-0.19
-0.59	-0.27	-0.61
-0.78	-0.87	-0.98
-1.30	1.15	0.60
-0.25	0.27	-0.83
-0.96	1.38	1.46
-0.60	1.06	-0.07
-1.06	-0.67	-0.61
-0.25	-0.22	-0.23
0.62	-1.25	-0.10
-0.43	-0.38	-0.66
-0.46	-0.24	-0.44
-0.78	1.80	-0.03
-1.25	1.93	2.02
-0.77	-1.91	-0.53
-0.45	1.49	-1.03
-0.60	-0.03	-0.48
-0.06	0.08	-0.99
-0.74	-0.64	0.55
-0.89	1.39	1.06

-0.20	-0.01	-0.31
0.17	-0.08	-0.08
0.13	-0.56	-0.70
-1.14	2.46	-0.88
-0.76	4.06	-0.25
-0.19	0.96	-0.28
-0.60	-0.24	-0.93
-0.91	-0.47	-1.21
-0.51	-0.17	-1.56
-0.60	-0.24	-1.07
0.65	0.43	-0.97
-0.93	1.96	-0.74
-0.11	-0.41	-0.54
-1.10	-1.73	-0.61
-0.58	1.93	-0.07
-0.58	1.93	-0.07
-0.58	4.31	-0.83
0.36	-0.93	-0.48
0.24	-0.87	0.62
-0.66	-0.96	-0.67
-0.29	-0.34	-0.69
-0.35	-0.22	-0.33
-0.74	2.95	2.28
-0.78	1.98	-0.77
-0.69	-1.02	-0.78
-0.77	1.23	-1.00
-0.16	1.71	-0.45
-0.41	-0.35	-0.76
-0.69	-0.58	0.02
-1.00	-0.84	-0.20
-0.57	4.45	-0.33
0.00	-0.86	-0.40
-1.41	0.11	1.20

1	2	3
1	3	4
TATTGCACTTGTCCCGGCCTGT	CAACGGAATCCCAAAAGCAGCTG	TCTCCCAACCCTTGTACCAGTG
miR-92a-3p	miR-191-5p	miR-150-5p
46389442	10497180	8994745
1.23	-0.77	-0.63
-1.76	3.44	-0.49
0.00	-0.58	-1.27
1.30	-0.72	-0.99
-2.19	0.71	2.40
0.72	-0.50	-0.83
0.63	-0.74	0.28
1.28	-0.53	0.32
1.11	-0.06	-1.21
0.95	-0.19	-0.67
-0.09	0.29	-0.36
0.50	-0.81	3.01
-1.08	-0.57	-0.41
1.05	-0.55	0.47
0.13	0.07	-0.71
-0.52	-0.14	0.03
-0.04	-0.02	0.22
-0.01	-0.53	0.35
-0.50	0.88	-0.09
-0.90	-0.49	-0.49

0.82	-0.47	-0.62
0.08	-0.34	1.20
0.16	-0.34	0.60
0.71	-0.55	0.35
-1.88	2.64	-0.41
-1.11	0.82	0.78
-0.60	0.06	-0.82
-1.04	-0.73	-1.39
0.35	-0.02	-0.78
0.96	0.40	-1.14
0.86	0.80	-1.30
-1.14	1.55	-1.18
-2.35	0.91	-0.42
1.20	-0.55	-0.91
0.88	-0.20	-0.62
0.16	-0.61	2.79
2.69	-0.85	-1.13
0.15	-0.66	0.68
-1.17	-0.72	-0.62
-0.53	-0.26	2.94
0.64	-0.64	0.64
-1.46	-0.61	1.75
0.61	-0.28	0.42
0.02	-0.14	-0.64
0.61	0.28	1.22
1.17	-0.78	-0.31
-1.08	0.36	0.20
-0.05	-0.28	0.48
0.21	-0.29	0.19
-0.15	-0.37	2.45
-0.52	-0.31	1.54
0.27	0.20	0.46
0.95	-0.06	-1.06
-0.39	0.14	0.27
0.87	-0.21	-0.33
0.59	0.14	-0.23
0.52	-0.27	-0.15
-1.52	-0.61	-0.81
-0.77	-0.03	1.47
-1.80	0.48	0.36
-2.04	0.42	1.88
-1.24	-0.54	-1.08
0.73	-0.22	-0.23
0.30	-0.32	-0.06
-0.59	-0.10	-0.96
0.21	-0.81	-1.09
0.01	-0.78	-1.33
-0.84	2.04	-0.90
-1.38	-0.54	-0.48
-1.06	-0.36	-0.25
1.33	-0.73	-0.74
-1.59	-0.94	5.34
0.61	-0.65	-0.74
-0.35	-0.66	1.06
0.15	-0.81	2.20
-0.16	-0.57	0.99
-0.60	1.00	0.65
-0.32	1.12	-0.39
-0.87	0.08	-0.71

-1.20	0.58	-0.81
0.04	0.86	-0.78
-0.75	0.14	0.59
0.11	-0.39	3.40
-1.40	-0.58	-0.28
-1.59	-0.68	-1.11
0.10	-0.19	1.55
-0.64	-0.07	1.55
0.04	-0.30	1.16
-0.33	0.03	-0.60
-0.22	0.18	1.32
-1.06	-0.16	-1.27
1.05	-0.17	-0.25
0.04	-0.31	0.12
-0.64	1.06	-1.24
-0.22	-0.22	-0.70
0	1	0
0.00	0.00	0.00
1.00	1.00	1.00
0.17	0.79	0.72

1	2	3
TATTGCACTTGTCCCGGCCTGT	CAACGGAATCCCAAAAGCAGCTG	TCTCCCAACCCTTGTACCAGTG
miR-92a-3p	miR-191-5p	miR-150-5p
46389442	10497180	8994745
1.23	-0.77	-0.63
-1.76	3.44	-0.49
0.00	-0.58	-1.27
1.30	-0.72	-0.99
-2.19	0.71	2.40
0.72	-0.50	-0.83
0.63	-0.74	0.28
1.28	-0.53	0.32
1.11	-0.06	-1.21
0.95	-0.19	-0.67
-0.09	0.29	-0.36
0.50	-0.81	3.01
-1.08	-0.57	-0.41
1.05	-0.55	0.47
0.13	0.07	-0.71
-0.52	-0.14	0.03
-0.04	-0.02	0.22
-0.01	-0.53	0.35
-0.50	0.88	-0.09
-0.90	-0.49	-0.49
0.82	-0.47	-0.62
0.08	-0.34	1.20
0.16	-0.34	0.60
0.71	-0.55	0.35
-1.88	2.64	-0.41
-1.11	0.82	0.78
-0.60	0.06	-0.82
-1.04	-0.73	-1.39

0.35	-0.02	-0.78
0.96	0.40	-1.14
0.86	0.80	-1.30
-1.14	1.55	-1.18
-2.35	0.91	-0.42
1.20	-0.55	-0.91
0.88	-0.20	-0.62
0.16	-0.61	2.79
[illegible]	[illegible]	[illegible]

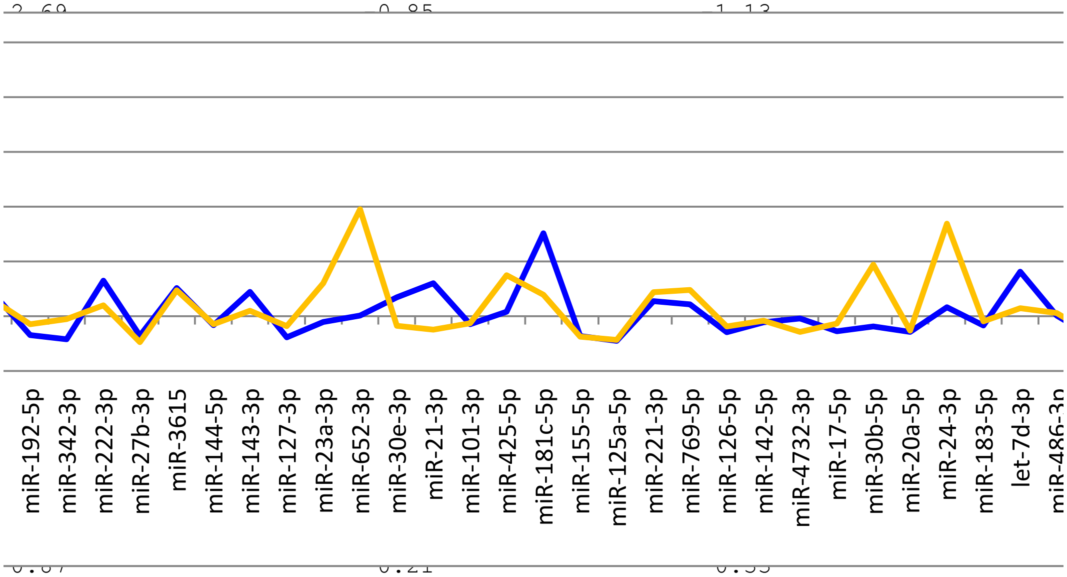

[illegible]	[illegible]	[illegible]
0.59	0.14	-0.23
0.52	-0.27	-0.15
-1.52	-0.61	-0.81
-0.77	-0.03	1.47
-1.80	0.48	0.36
-2.04	0.42	1.88
-1.24	-0.54	-1.08
0.73	-0.22	-0.23
0.30	-0.32	-0.06
-0.59	-0.10	-0.96
0.21	-0.81	-1.09
0.01	-0.78	-1.33
-0.84	2.04	-0.90
-1.38	-0.54	-0.48
-1.06	-0.36	-0.25
1.33	-0.73	-0.74
-1.59	-0.94	5.34
0.61	-0.65	-0.74
-0.35	-0.66	1.06
0.15	-0.81	2.20
-0.16	-0.57	0.99
-0.60	1.00	0.65
-0.32	1.12	-0.39
-0.87	0.08	-0.71
-1.20	0.58	-0.81
0.04	0.86	-0.78
-0.75	0.14	0.59
0.11	-0.39	3.40
-1.40	-0.58	-0.28
-1.59	-0.68	-1.11
0.10	-0.19	1.55
-0.64	-0.07	1.55

0.04	-0.30	1.16
-0.33	0.03	-0.60
-0.22	0.18	1.32
-1.06	-0.16	-1.27
1.05	-0.17	-0.25
0.04	-0.31	0.12
-0.64	1.06	-1.24
0	0	-1

1	2	3
25	134	59
TGAGAACTGAATTCCATAGGCT	CACGCTCATGCACACACCCACA	TTAATGCTAATCGTGATAGGGGT
miR-146b-5p	miR-574-3p	miR-155-5p
614674	10799	122415
-0.58	0.38	1.04
2.00	0.14	0.07
0.04	-0.52	-0.49
-0.40	-0.57	-0.60
0.49	4.45	2.92
-0.40	-0.33	1.53
-0.68	0.07	0.89
-0.71	-0.43	-1.16
-0.23	-0.33	-1.52
0.81	0.30	-0.63
3.99	-0.62	-0.39
-0.56	-0.95	0.37
0.27	0.59	0.30
-0.63	-0.28	-0.73
-0.41	-0.22	-0.20
-0.20	-0.35	0.31
-0.47	0.70	-0.36
-0.49	0.99	-0.07
0.20	-0.33	0.33
0.70	-0.38	-0.63
-0.37	-0.58	0.53
-0.63	0.03	-0.54
-0.62	-0.04	-0.13
-0.64	-0.17	-0.04
-0.41	-0.36	-1.33
-0.03	-0.30	1.75
-0.05	-0.88	-1.24
1.67	-0.70	-1.08
2.81	-0.58	-0.07
1.13	-0.79	-0.17
-0.57	-0.30	-1.06
6.40	-0.53	-0.69
0.09	2.24	-0.93
-0.67	0.15	-0.74
-0.67	0.16	-0.75
-0.36	0.15	1.14
-0.75	-0.73	-1.83

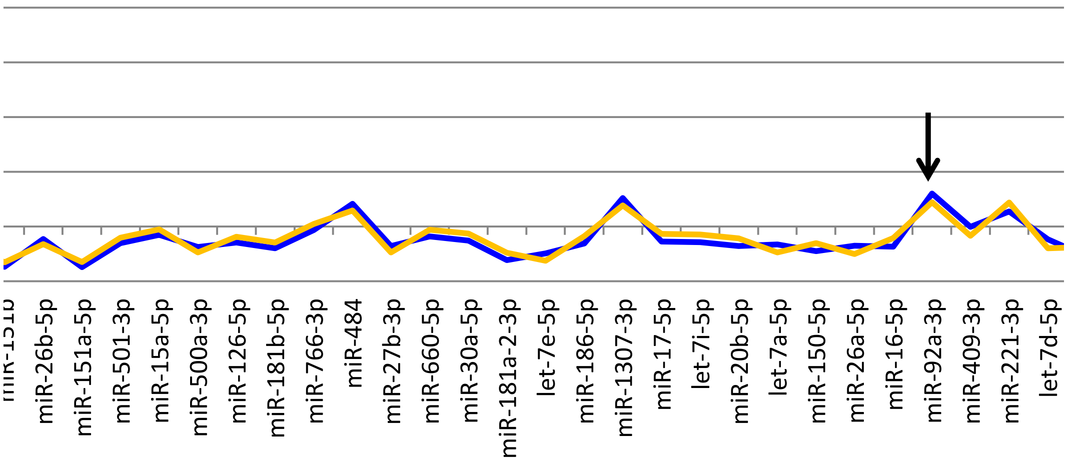

[illegible]	[illegible]	[illegible]
-0.57	0.13	-0.44
-0.49	-0.35	0.04
1.14	-0.92	0.09
0.02	-0.48	1.22
0.10	-0.35	2.41
0.77	-0.12	1.71
0.53	-0.84	-1.37
-0.61	-0.38	-0.63
-0.38	-0.90	-0.43
0.69	-0.84	-0.98
-0.14	-0.82	-0.21
0.02	-0.30	-0.92
1.40	0.82	1.08
-0.39	0.27	0.26
0.36	0.46	1.59
-0.49	-0.39	-0.31
0.49	-0.61	-1.09
-0.32	-0.56	-0.63
1.01	-0.49	0.63
-0.71	8.27	-1.36
-0.29	-0.51	0.56
1.03	-0.76	0.93
0.87	-0.71	0.30
0.96	0.35	0.19
0.48	0.09	-0.16
-0.52	0.60	-1.42
0.20	-0.01	0.91
-0.57	-0.53	-0.04
1.68	-0.14	0.21
0.83	-0.74	-1.29
-0.44	0.77	-0.94
-0.11	-0.12	0.30
-0.48	-0.32	0.25
0.85	-0.09	0.16
-0.21	0.58	0.31
3.24	-0.87	-0.19
-0.56	-0.48	0.38
-0.55	0.22	-0.30
0.45	-0.84	-0.89
-0.05	-0.31	0.16
0.00	0.01	0.02
0.00	0.03	0.01

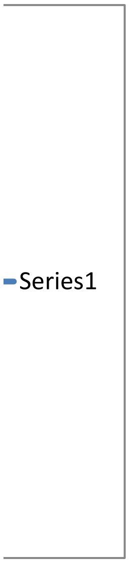
Series1

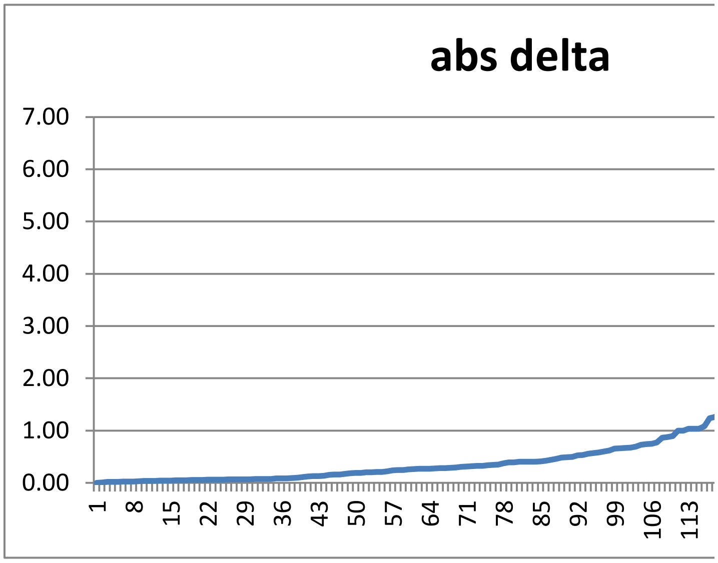
abs delta
7.00
6.00
5.00
4.00
3.00
2.00
1.00
0.00
1
8
15
22
29
36
43
50
57
64
71
78
85
92
99
106
113

356562	1363063	824884
7	8	9
7	8	9
B_32	B_34	C_1
UC	UC	UC
793095	2416278	2851220
0.64	0.61	0.04
B_32	B_34	C_1
436533	1053215	2026336
165954	611778	445855
17347	31223	37854
11202	105091	65314
37642	120189	45388
19686	131474	70868
6653	7446	5218
16299	33009	14027
126	913	844
2890	8942	3676
7519	15831	8819
12213	33498	5755
3540	22347	14870
4451	25473	3000
4350	16799	14150
2342	3788	838
7215	35649	9765
1156	4464	802
1114	8136	2552
730	2501	2161
2828	10410	3362
1372	8427	3475
725	7142	2556
2398	4485	1504
265	652	702
703	614	255
840	4063	2120
1234	8635	3018
129	1193	462
946	2888	721
1462	7026	1802
444	1773	1691
1040	5054	3070
312	1040	781
309	1682	352
855	2659	688
922	2979	2836
643	2827	848
980	1020	620
1148	2750	3200
749	3356	5842
182	3138	3340
886	4162	1789
560	2440	1510
105	240	142
221	1699	477

726	2940	869
619	1723	394
398	2245	2429
77	236	78
351	1293	281
365	2059	658
218	741	318
276	1306	704
270	1046	696
339	1367	166
26	166	41
228	599	439
150	880	636
571	1805	385
252	1212	597
199	1989	452
162	869	595
33	68	55
13	31	71
603	1234	1698
64	378	165
26	242	99
26	225	35
35	276	117
23	79	122
711	1830	741
277	775	970
55	138	36
78	414	161
53	178	112
225	1954	461
112	325	222
60	232	159
221	1633	645
154	896	170
74	206	165
94	817	462
171	446	894
77	755	28
48	135	71
36	527	221
181	947	601
12	51	38
155	525	132
126	996	210
62	370	176
5	7	27
70	487	203
28	147	38
15	67	18
65	204	90
183	592	339
62	295	313
34	205	143
16	38	15
2	213	83
71	133	83
140	427	137
52	88	27

49	98	62
11	63	29
28	210	222
55	116	66
32	91	30
12	46	25
9	80	80
18	242	83
10	147	148
10	45	17
22	253	70
40	287	64
25	157	71
34	166	30
34	166	30
15	80	97
25	183	85
49	257	125
9	105	18
13	48	30
2	12	4
102	188	139
14	41	28
16	179	112
5	81	94
18	161	60
0	9	21
25	59	68
21	47	14
22	127	44
9	50	14
41	199	46
27	122	19
3	77	32
12	57	26
10	58	45
50	103	49
9	38	40
22	165	140
25	80	45
2	9	4
18	48	126
35	114	74
8	26	25
34	59	77
7	40	31
3	26	10
4	43	5
7	85	9
27	88	48
2	102	8
15	58	58
1	32	15
2	80	46
3	118	23
5	39	49
8	5	5
6	69	38
18	51	85

6	39	10
6	18	15
34	77	60
4	52	39
14	13	47
1	5	3
8	44	65
6	50	26
25	74	11
27	70	54
23	38	32
6	7	9
9	153	40
28	150	29
20	25	8
2	36	26
3	114	39
0	24	2
2	8	7
2	65	19
1	7	5
5	107	33
19	59	55
2	8	7
17	192	20
36	33	33
8	21	5
0	0	0
3	7	2
4	33	10
50	59	122
4	19	23
2	8	4
2	4	0
6	27	35
2	50	13
0	9	26
0	18	76
31	89	13
2	46	26
7	30	27
3	8	9
9	31	7
4	15	47
5	49	24
1	60	10
6	24	26
4	34	31
15	14	30
3	9	7
19	43	33
5	56	6
21	31	15
6	10	20
7	34	10
0	2	1
1	16	6
1	0	1
4	32	34

1	10	6
4	26	7
3	33	13
12	46	25
5	26	16
33	24	27
6	18	11
2	14	3
1	11	6
13	20	17
0	0	1
2	34	12
5	11	6
0	13	6
1	9	0
0	6	1
2	15	4
4	10	20
5	62	5
4	12	8
0	0	3
2	0	5
2	0	5
1	29	39
0	15	22
1	3	7
1	44	7
4	16	0
4	18	14
3	30	7
1	8	3
1	15	2
5	4	9
4	2	7
3	14	5
2	12	2
0	2	0
5	22	6
0	4	2
4	9	10
7	12	19
0	2	2
2	42	11
1	24	3
1	6	4
9	27	11
0	2	1
3	26	7
13	13	11
3	8	23
1	33	8
1	6	2
0	0	0
2	6	7
3	5	3
0	4	62
1	7	5
1	8	10
0	8	4

0	18	10
3	8	10
4	18	7
7	12	9
4	21	12
3	23	16
3	5	3
4	41	7
0	2	0
1	3	0
0	1	2
1	11	9
3	8	0
0	33	9
3	22	5
2	2	1
1	8	11
1	11	0
1	11	5
2	12	2
5	20	13
2	14	3
0	9	5
1	12	13
3	11	2
1	6	9
5	15	2
2	21	4
4	9	3
1	9	4
0	5	7
0	0	0
0	1	0
0	5	6
3	3	3
1	10	8
0	21	9
1	4	8
1	9	2
2	9	5
3	1	0
3	11	3
2	8	2
3	5	11
3	4	3
0	12	7
3	3	6
4	4	1
2	13	11
1	11	7
1	20	6
0	1	0
5	8	2
0	1	1
1	3	4
0	3	4
3	17	6
0	0	0
1	18	5

2	16	3
6	15	5
0	1	1
2	6	7
4	4	7
0	8	2
2	5	5
0	1	2
0	3	2
1	7	4
2	10	1
2	3	4
0	1	4
0	0	0
3	17	5
1	1	2
1	2	3
0	3	1
0	0	0
0	4	2
0	4	0
0	5	1
1	4	2
2	5	7
2	6	3
7	8	0
0	1	0
1	1	0
0	6	6
1	2	1
0	2	1
2	10	4
0	1	0
0	12	4
0	1	0
3	16	3
1	4	1
1	1	7
5	10	11
0	2	3
2	5	6
0	4	0
0	8	0
1	9	0
0	3	1
0	1	4
0	2	2
1	5	1
0	0	0
0	0	1
1	10	4
0	5	0
0	0	0
0	1	2
2	0	0
0	2	0
1	3	3
0	6	1
0	1	0

0	0	1
0	4	3
2	0	0
0	0	0
0	2	4
0	1	0
0	3	7
5	0	0
1	4	3
0	2	2
0	2	0
0	1	0
0	0	0
0	0	1
1	5	1
1	8	4
2	5	3
3	3	2
0	0	0
0	0	0
0	3	1
1	1	0
0	2	1
2	3	0
0	4	1
0	0	0
0	2	0
2	3	0
0	0	0
0	1	1
0	0	0
0	5	1
2	6	1
0	2	6
0	1	1
1	5	0
2	1	2
0	3	0
0	1	1
1	6	0
0	1	1
2	1	2
3	4	1
0	0	2
2	3	1
0	0	0
1	0	0
0	0	6
0	2	4
0	0	1
1	2	1
0	0	0
0	1	0
1	1	0
0	1	0
1	0	0
0	0	1
0	5	2
0	0	0

0	2	2
0	3	3
2	2	2
0	0	0
0	0	1
1	0	0
0	4	2
0	1	0
0	3	5
0	1	0
0	2	7
0	0	0
0	1	0
1	5	0
0	0	0
0	1	0
0	0	1
0	0	0
0	0	0
0	2	0
0	0	0
0	0	0
0	0	0
1	1	4
3	3	0
0	1	1
0	2	3
0	3	0
0	0	0
0	0	1
0	0	0
0	0	3
0	0	0
0	2	1
0	2	1
0	2	0
0	0	0
0	0	0
0	2	0
1	2	1
1	0	0
0	2	2
1	3	0
0	4	1
0	3	4
0	0	0
1	0	4
0	0	4
0	1	1
0	2	1
1	2	2
0	1	0
0	0	0
1	0	0
0	2	1
0	0	0
0	1	0
0	1	3
1	0	0

0	0	1
0	1	0
0	0	0
0	3	0
0	0	0
0	3	2
0	0	0
0	0	1
0	0	0
4	1	3
0	1	2
1	2	0
0	0	0
B_32	B_34	C_1
UC	UC	UC
436533	1053215	2026336
18	14	22
-0.80	-1.54	0.23

0.27	0.25	0.68
20	21	9
1	1	1
7	8	9
7	8	9
B_34	C_1	C_40
UC	UC	UC
1363063	824884	2206918
B_34	C_1	C_40
611778	445855	1151739
31223	37854	212328
105091	65314	34851
120189	45388	161620
131474	70868	71100
7446	5218	69561
33009	14027	46792
913	844	14990
8942	3676	40102
15831	8819	78886
33498	5755	22597
22347	14870	26861
25473	3000	12589
16799	14150	27534
3788	838	8708
35649	9765	12161
4464	802	3895
8136	2552	7622
2501	2161	10910

10410	3362	9844
8427	3475	6819
7142	2556	9468
4485	1504	3495
652	702	3102
614	255	6392
4063	2120	5309
8635	3018	5386
1193	462	9240
2888	721	7772
7026	1802	5010
1773	1691	4682
5054	3070	5236
1040	781	1221
1682	352	461
2659	688	7293
2979	2836	7956
2827	848	10796
1020	620	6151
2750	3200	6179
3356	5842	3316
3138	3340	2883
4162	1789	1123
2440	1510	3965
240	142	2630
1699	477	1702
2940	869	1577
1723	394	1318
2245	2429	1707
236	78	265
1293	281	586
2059	658	3027
741	318	1030
1306	704	1275
1046	696	1197
1367	166	691
166	41	74
599	439	1963
880	636	801
1805	385	708
1212	597	1406
1989	452	643
869	595	560
68	55	288
31	71	49
1234	1698	3665
378	165	755
242	99	166
225	35	208
276	117	235
79	122	3061
1830	741	819
775	970	1051
138	36	424
414	161	1357
178	112	173
1954	461	1019
325	222	1271
232	159	751

1633	645	467
896	170	431
206	165	1370
817	462	459
446	894	2109
755	28	46
135	71	296
527	221	234
947	601	959
51	38	203
525	132	589
996	210	306
370	176	209
7	27	23
487	203	185
147	38	159
67	18	80
204	90	109
592	339	264
295	313	286
205	143	143
38	15	121
213	83	204
133	83	158
427	137	117
88	27	322
98	62	256
63	29	100
210	222	82
116	66	220
91	30	74
46	25	127
80	80	138
242	83	115
147	148	125
45	17	254
253	70	89
287	64	243
157	71	214
166	30	65
166	30	65
80	97	182
183	85	163
257	125	124
105	18	178
48	30	102
12	4	5
188	139	131
41	28	84
179	112	47
81	94	181
161	60	54
9	21	49
59	68	50
47	14	126
127	44	137
50	14	25
199	46	78
122	19	215

77	32	81
57	26	60
58	45	70
103	49	118
38	40	222
165	140	73
80	45	31
9	4	14
48	126	206
114	74	46
26	25	85
59	77	170
40	31	50
26	10	21
43	5	76
85	9	52
88	48	52
102	8	1
58	58	140
32	15	435
80	46	48
118	23	43
39	49	68
5	5	4
69	38	35
51	85	94
39	10	15
18	15	18
77	60	100
52	39	34
13	47	24
5	3	7
44	65	92
50	26	15
74	11	35
70	54	49
38	32	48
7	9	12
153	40	40
150	29	38
25	8	30
36	26	83
114	39	38
24	2	79
8	7	25
65	19	30
7	5	54
107	33	24
59	55	55
8	7	22
192	20	46
33	33	14
21	5	30
0	0	0
7	2	47
33	10	24
59	122	35
19	23	96
8	4	17

4	0	6
27	35	102
50	13	17
9	26	29
18	76	30
89	13	30
46	26	51
30	27	10
8	9	24
31	7	36
15	47	115
49	24	20
60	10	16
24	26	46
34	31	29
14	30	26
9	7	20
43	33	51
56	6	17
31	15	34
10	20	11
34	10	10
2	1	3
16	6	69
0	1	54
32	34	36
10	6	19
26	7	22
33	13	17
46	25	28
26	16	23
24	27	33
18	11	26
14	3	58
11	6	13
20	17	81
0	1	4
34	12	18
11	6	21
13	6	9
9	0	4
6	1	4
15	4	6
10	20	29
62	5	5
12	8	12
0	3	20
0	5	12
0	5	12
29	39	23
15	22	19
3	7	13
44	7	6
16	0	18
18	14	6
30	7	6
8	3	20
15	2	4
4	9	4

2	7	3
14	5	11
12	2	46
2	0	1
22	6	10
4	2	12
9	10	15
12	19	26
2	2	11
42	11	7
24	3	6
6	4	10
27	11	13
2	1	1
26	7	4
13	11	27
8	23	17
33	8	14
6	2	15
0	0	0
6	7	8
5	3	14
4	62	274
7	5	12
8	10	25
8	4	28
18	10	13
8	10	12
18	7	15
12	9	11
21	12	16
23	16	9
5	3	14
41	7	16
2	0	7
3	0	2
1	2	10
11	9	12
8	0	4
33	9	0
22	5	6
2	1	16
8	11	40
11	0	3
11	5	3
12	2	5
20	13	6
14	3	4
9	5	3
12	13	10
11	2	6
6	9	6
15	2	3
21	4	5
9	3	7
9	4	7
5	7	18
0	0	5
1	0	1

5	6	17
3	3	15
10	8	9
21	9	2
4	8	1
9	2	1
9	5	6
1	0	5
11	3	3
8	2	10
5	11	5
4	3	14
12	7	4
3	6	13
4	1	11
13	11	5
11	7	2
20	6	4
1	0	0
8	2	8
1	1	2
3	4	7
3	4	4
17	6	6
0	0	2
18	5	3
16	3	10
15	5	3
1	1	6
6	7	3
4	7	4
8	2	9
5	5	10
1	2	2
3	2	3
7	4	4
10	1	3
3	4	3
1	4	6
0	0	0
17	5	6
1	2	4
2	3	2
3	1	7
0	0	1
4	2	15
4	0	1
5	1	3
4	2	3
5	7	9
6	3	11
8	0	0
1	0	1
1	0	1
6	6	3
2	1	4
2	1	2
10	4	3
1	0	0

12	4	4
1	0	0
16	3	6
4	1	5
1	7	5
10	11	3
2	3	2
5	6	1
4	0	1
8	0	1
9	0	3
3	1	3
1	4	0
2	2	2
5	1	4
0	0	1
0	1	4
10	4	3
5	0	3
0	0	1
1	2	1
0	0	1
2	0	8
3	3	6
6	1	3
1	0	0
0	1	6
4	3	2
0	0	3
0	0	1
2	4	1
1	0	6
3	7	2
0	0	3
4	3	6
2	2	2
2	0	1
1	0	1
0	0	0
0	1	1
5	1	1
8	4	2
5	3	8
3	2	3
0	0	0
0	0	1
3	1	2
1	0	0
2	1	3
3	0	2
4	1	4
0	0	0
2	0	0
3	0	1
0	0	6
1	1	3
0	0	1
5	1	2
6	1	3

2	6	2
1	1	0
5	0	3
1	2	4
3	0	2
1	1	3
6	0	5
1	1	0
1	2	0
4	1	3
0	2	0
3	1	4
0	0	1
0	0	0
0	6	2
2	4	4
0	1	5
2	1	0
0	0	5
1	0	8
1	0	1
1	0	1
0	0	4
0	1	0
5	2	0
0	0	5
2	2	0
3	3	2
2	2	0
0	0	3
0	1	8
0	0	1
4	2	0
1	0	1
3	5	2
1	0	1
2	7	1
0	0	0
1	0	0
5	0	1
0	0	3
1	0	1
0	1	0
0	0	0
0	0	0
2	0	6
0	0	2
0	0	0
0	0	0
1	4	1
3	0	0
1	1	1
2	3	0
3	0	5
0	0	0
0	1	4
0	0	4
0	3	0
0	0	3

2	1	0
2	1	1
2	0	0
0	0	2
0	0	0
2	0	1
2	1	1
0	0	0
2	2	1
3	0	0
4	1	1
3	4	2
0	0	8
0	4	3
0	4	5
1	1	0
2	1	2
2	2	1
1	0	1
0	0	0
0	0	0
2	1	1
0	0	3
1	0	1
1	3	0
0	0	0
0	1	1
1	0	0
0	0	0
3	0	0
0	0	0
3	2	0
0	0	2
0	1	6
0	0	1
1	3	3
1	2	1
2	0	2

0.27	0.25	0.68
20	21	9
1	1	1
7	8	9
7	8	9
B_34	C_1	C_40
UC	UC	UC
1363063	824884	2206918
UC	UC	UC
611778	445855	1151739
31223	37854	212328
105091	65314	34851
120189	45388	161620
131474	70868	71100

7446	5218	69561
33009	14027	46792
913	844	14990
8942	3676	40102
15831	8819	78886
33498	5755	22597
22347	14870	26861
25473	3000	12589
16799	14150	27534
3788	838	8708
35649	9765	12161
4464	802	3895
8136	2552	7622
2501	2161	10910
10410	3362	9844
8427	3475	6819
7142	2556	9468
4485	1504	3495
652	702	3102
614	255	6392
4063	2120	5309
8635	3018	5386
1193	462	9240
2888	721	7772
7026	1802	5010
1773	1691	4682
5054	3070	5236
1040	781	1221
1682	352	461
2659	688	7293
2979	2836	7956
2827	848	10796
1020	620	6151
2750	3200	6179
3356	5842	3316
3138	3340	2883
4162	1789	1123
2440	1510	3965
240	142	2630
1699	477	1702
2940	869	1577
1723	394	1318
2245	2429	1707
236	78	265
1293	281	586
2059	658	3027
741	318	1030
1306	704	1275
1046	696	1197
1367	166	691
166	41	74
599	439	1963
880	636	801
1805	385	708
1212	597	1406
1989	452	643
869	595	560
68	55	288
31	71	49

1234	1698	3665
378	165	755
242	99	166
225	35	208
276	117	235
79	122	3061
1830	741	819
775	970	1051
138	36	424
414	161	1357
178	112	173
1954	461	1019
325	222	1271
232	159	751
1633	645	467
896	170	431
206	165	1370
817	462	459
446	894	2109
755	28	46
135	71	296
527	221	234
947	601	959
51	38	203
525	132	589
996	210	306
370	176	209
7	27	23
487	203	185
147	38	159
67	18	80
204	90	109
592	339	264
295	313	286
205	143	143
38	15	121
213	83	204
133	83	158
427	137	117
88	27	322
98	62	256
63	29	100
210	222	82
116	66	220
91	30	74
46	25	127
80	80	138
242	83	115
147	148	125
45	17	254
253	70	89
287	64	243
157	71	214
166	30	65
166	30	65
80	97	182
183	85	163
257	125	124
105	18	178

48	30	102
12	4	5
188	139	131
41	28	84
179	112	47
81	94	181
161	60	54
9	21	49
59	68	50
47	14	126
127	44	137
50	14	25
199	46	78
122	19	215
77	32	81
57	26	60
58	45	70
103	49	118
38	40	222
165	140	73
80	45	31
9	4	14
48	126	206
114	74	46
26	25	85
59	77	170
40	31	50
26	10	21
43	5	76
85	9	52
88	48	52
102	8	1
58	58	140
32	15	435
80	46	48
118	23	43
39	49	68
5	5	4
69	38	35
51	85	94
39	10	15
18	15	18
77	60	100
52	39	34
13	47	24
5	3	7
44	65	92
50	26	15
74	11	35
70	54	49
38	32	48
7	9	12
153	40	40
150	29	38
25	8	30
36	26	83
114	39	38
24	2	79
8	7	25

65	19	30
7	5	54
107	33	24
59	55	55
8	7	22
192	20	46
33	33	14
21	5	30
0	0	0
7	2	47
33	10	24
59	122	35
19	23	96
8	4	17
4	0	6
27	35	102
50	13	17
9	26	29
18	76	30
89	13	30
46	26	51
30	27	10
8	9	24
31	7	36
15	47	115
49	24	20
60	10	16
24	26	46
34	31	29
14	30	26
9	7	20
43	33	51
56	6	17
31	15	34
10	20	11
34	10	10
2	1	3
16	6	69
0	1	54
32	34	36
10	6	19
26	7	22
33	13	17
46	25	28
26	16	23
24	27	33
18	11	26
14	3	58
11	6	13
20	17	81
0	1	4
34	12	18
11	6	21
13	6	9
9	0	4
6	1	4
15	4	6
10	20	29
62	5	5

12	8	12
0	3	20
0	5	12
0	5	12
29	39	23
15	22	19
3	7	13
44	7	6
16	0	18
18	14	6
30	7	6
8	3	20
15	2	4
4	9	4
2	7	3
14	5	11
12	2	46
2	0	1
22	6	10
4	2	12
9	10	15
12	19	26
2	2	11
42	11	7
24	3	6
6	4	10
27	11	13
2	1	1
26	7	4
13	11	27
8	23	17
33	8	14
6	2	15
0	0	0
6	7	8
5	3	14
4	62	274
7	5	12
8	10	25
8	4	28
18	10	13
8	10	12
18	7	15
12	9	11
21	12	16
23	16	9
5	3	14
41	7	16
2	0	7
3	0	2
1	2	10
11	9	12
8	0	4
33	9	0
22	5	6
2	1	16
8	11	40
11	0	3
11	5	3

12	2	5
20	13	6
14	3	4
9	5	3
12	13	10
11	2	6
6	9	6
15	2	3
21	4	5
9	3	7
9	4	7
5	7	18
0	0	5
1	0	1
5	6	17
3	3	15
10	8	9
21	9	2
4	8	1
9	2	1
9	5	6
1	0	5
11	3	3
8	2	10
5	11	5
4	3	14
12	7	4
3	6	13
4	1	11
13	11	5
11	7	2
20	6	4
1	0	0
8	2	8
1	1	2
3	4	7
3	4	4
17	6	6
0	0	2
18	5	3
16	3	10
15	5	3
1	1	6
6	7	3
4	7	4
8	2	9
5	5	10
1	2	2
3	2	3
7	4	4
10	1	3
3	4	3
1	4	6
0	0	0
17	5	6
1	2	4
2	3	2
3	1	7
0	0	1

4	2	15
4	0	1
5	1	3
4	2	3
5	7	9
6	3	11
8	0	0
1	0	1
1	0	1
6	6	3
2	1	4
2	1	2
10	4	3
1	0	0
12	4	4
1	0	0
16	3	6
4	1	5
1	7	5
10	11	3
2	3	2
5	6	1
4	0	1
8	0	1
9	0	3
3	1	3
1	4	0
2	2	2
5	1	4
0	0	1
0	1	4
10	4	3
5	0	3
0	0	1
1	2	1
0	0	1
2	0	8
3	3	6
6	1	3
1	0	0
0	1	6
4	3	2
0	0	3
0	0	1
2	4	1
1	0	6
3	7	2
0	0	3
4	3	6
2	2	2
2	0	1
1	0	1
0	0	0
0	1	1
5	1	1
8	4	2
5	3	8
3	2	3
0	0	0

0	0	1
3	1	2
1	0	0
2	1	3
3	0	2
4	1	4
0	0	0
2	0	0
3	0	1
0	0	6
1	1	3
0	0	1
5	1	2
6	1	3
2	6	2
1	1	0
5	0	3
1	2	4
3	0	2
1	1	3
6	0	5
1	1	0
1	2	0
4	1	3
0	2	0
3	1	4
0	0	1
0	0	0
0	6	2
2	4	4
0	1	5
2	1	0
0	0	5
1	0	8
1	0	1
1	0	1
0	0	4
0	1	0
5	2	0
0	0	5
2	2	0
3	3	2
2	2	0
0	0	3
0	1	8
0	0	1
4	2	0
1	0	1
3	5	2
1	0	1
2	7	1
0	0	0
1	0	0
5	0	1
0	0	3
1	0	1
0	1	0
0	0	0
0	0	0

2	0	6
0	0	2
0	0	0
0	0	0
1	4	1
3	0	0
1	1	1
2	3	0
3	0	5
0	0	0
0	1	4
0	0	4
0	3	0
0	0	3
2	1	0
2	1	1
2	0	0
0	0	2
0	0	0
2	0	1
2	1	1
0	0	0
2	2	1
3	0	0
4	1	1
3	4	2
0	0	8
0	4	3
0	4	5
1	1	0
2	1	2
2	2	1
1	0	1
0	0	0
0	0	0
2	1	1
0	0	3
1	0	1
1	3	0
0	0	0
0	1	1
1	0	0
0	0	0
3	0	0
0	0	0
3	2	0
0	0	2
0	1	6
0	0	1
1	3	3
1	2	1
2	0	2
42470	25724	69556
112911	81506	209845
405	435	402
UC	UC	UC
UC	UC	UC

7	8	9
7	8	9
UC	UC	UC
B_34	C_1	C_40
1363063	824884	2206918
UC	UC	UC
14.41	17.33	16.56
0.74	1.47	3.05
2.47	2.54	0.50
2.83	1.76	2.32
3.10	2.75	1.02
0.18	0.20	1.00
0.78	0.55	0.67
0.02	0.03	0.22
0.21	0.14	0.58
0.37	0.34	1.13
0.79	0.22	0.32
0.53	0.58	0.39
0.60	0.12	0.18
0.40	0.55	0.40
0.09	0.03	0.13
0.84	0.38	0.17
0.11	0.03	0.06
0.19	0.10	0.11
0.06	0.08	0.16
0.25	0.13	0.14
0.20	0.14	0.10
0.17	0.10	0.14
0.11	0.06	0.05
0.02	0.03	0.04
0.01	0.01	0.09
0.10	0.08	0.08
0.20	0.12	0.08
0.03	0.02	0.13
0.07	0.03	0.11
0.17	0.07	0.07
0.04	0.07	0.07
0.12	0.12	0.08
0.02	0.03	0.02
0.04	0.01	0.01
0.06	0.03	0.10
0.07	0.11	0.11
0.07	0.03	0.16
0.02	0.02	0.09
0.06	0.12	0.09
0.08	0.23	0.05
0.07	0.13	0.04
0.10	0.07	0.02
0.06	0.06	0.06
0.01	0.01	0.04
0.04	0.02	0.02
0.07	0.03	0.02
0.04	0.02	0.02
0.05	0.09	0.02
0.01	0.00	0.00
0.03	0.01	0.01
0.05	0.03	0.04
0.02	0.01	0.01

0.03	0.03	0.02
0.02	0.03	0.02
0.03	0.01	0.01
0.00	0.00	0.00
0.01	0.02	0.03
0.02	0.02	0.01
0.04	0.01	0.01
0.03	0.02	0.02
0.05	0.02	0.01
0.02	0.02	0.01
0.00	0.00	0.00
0.00	0.00	0.00
0.03	0.07	0.05
0.01	0.01	0.01
0.01	0.00	0.00
0.01	0.00	0.00
0.01	0.00	0.00
0.00	0.00	0.04
0.04	0.03	0.01
0.02	0.04	0.02
0.00	0.00	0.01
0.01	0.01	0.02
0.00	0.00	0.00
0.05	0.02	0.01
0.01	0.01	0.02
0.01	0.01	0.01
0.04	0.03	0.01
0.02	0.01	0.01
0.00	0.01	0.02
0.02	0.02	0.01
0.01	0.03	0.03
0.02	0.00	0.00
0.00	0.00	0.00
0.01	0.01	0.00
0.02	0.02	0.01
0.00	0.00	0.00
0.01	0.01	0.01
0.02	0.01	0.00
0.01	0.01	0.00
0.00	0.00	0.00
0.01	0.01	0.00
0.00	0.00	0.00
0.00	0.00	0.00
0.00	0.00	0.00
0.01	0.01	0.00
0.01	0.01	0.00
0.00	0.01	0.00
0.00	0.00	0.00
0.01	0.00	0.00
0.00	0.00	0.00
0.01	0.01	0.00
0.00	0.00	0.00
0.00	0.00	0.00
0.00	0.00	0.00
0.00	0.01	0.00
0.00	0.00	0.00
0.00	0.00	0.00
0.00	0.00	0.00
0.00	0.00	0.00

0.01	0.00	0.00
0.00	0.01	0.00
0.00	0.00	0.00
0.01	0.00	0.00
0.01	0.00	0.00
0.00	0.00	0.00
0.00	0.00	0.00
0.00	0.00	0.00
0.00	0.00	0.00
0.00	0.00	0.00
0.01	0.00	0.00
0.00	0.00	0.00
0.00	0.00	0.00
0.00	0.00	0.00
0.00	0.01	0.00
0.00	0.00	0.00
0.00	0.00	0.00
0.00	0.00	0.00
0.00	0.00	0.00
0.00	0.00	0.00
0.00	0.00	0.00
0.00	0.00	0.00
0.00	0.00	0.00
0.00	0.00	0.00
0.00	0.00	0.00
0.00	0.00	0.00
0.00	0.00	0.00
0.00	0.00	0.00
0.00	0.00	0.00
0.00	0.00	0.00
0.00	0.00	0.00
0.00	0.01	0.00
0.00	0.00	0.00
0.00	0.00	0.00
0.00	0.00	0.00
0.00	0.00	0.00
0.00	0.00	0.00
0.00	0.00	0.00
0.00	0.00	0.00
0.00	0.00	0.00
0.00	0.00	0.00
0.00	0.00	0.00
0.00	0.00	0.00
0.00	0.00	0.00
0.00	0.00	0.00
0.00	0.00	0.01
0.00	0.00	0.00
0.00	0.00	0.00
0.00	0.00	0.00
0.00	0.00	0.00
0.00	0.00	0.00
0.00	0.00	0.00
0.00	0.00	0.00
0.00	0.00	0.00
0.00	0.00	0.00
0.00	0.00	0.00
0.00	0.00	0.00
0.00	0.00	0.00
0.00	0.00	0.00

0.00	0.00	0.00
0.00	0.00	0.00
0.00	0.00	0.00
0.00	0.00	0.00
0.00	0.00	0.00
0.00	0.00	0.00
0.00	0.00	0.00
0.00	0.00	0.00
0.00	0.00	0.00
0.00	0.00	0.00
0.00	0.00	0.00
0.00	0.00	0.00
0.00	0.00	0.00
0.00	0.00	0.00
0.00	0.00	0.00
0.00	0.00	0.00
0.00	0.00	0.00
0.00	0.00	0.00
0.00	0.00	0.00
0.00	0.00	0.00
0.00	0.00	0.00
0.00	0.00	0.00
0.00	0.00	0.00
0.00	0.00	0.00
0.00	0.00	0.00
0.00	0.00	0.00
0.00	0.00	0.00
0.00	0.00	0.00
0.00	0.00	0.00
0.00	0.00	0.00
0.00	0.00	0.00
0.00	0.00	0.00
0.00	0.00	0.00
0.00	0.00	0.00
0.00	0.00	0.00
0.00	0.00	0.00
0.00	0.00	0.00
0.00	0.00	0.00
0.00	0.00	0.00
0.00	0.00	0.00
0.00	0.00	0.00
0.00	0.00	0.00
0.00	0.00	0.00
0.00	0.00	0.00
0.00	0.00	0.00
0.00	0.00	0.00
0.00	0.00	0.00
0.00	0.00	0.00
0.00	0.00	0.00
0.00	0.00	0.00
0.00	0.00	0.00
0.00	0.00	0.00
0.00	0.00	0.00
0.00	0.00	0.00
0.00	0.00	0.00
0.00	0.00	0.00
0.00	0.00	0.00
0.00	0.00	0.00
0.00	0.00	0.00

0.00	0.00	0.00
0.00	0.00	0.00
0.00	0.00	0.00
0.00	0.00	0.00
0.00	0.00	0.00
0.00	0.00	0.00
0.00	0.00	0.00
0.00	0.00	0.00
0.00	0.00	0.00
0.00	0.00	0.00
0.00	0.00	0.00
0.00	0.00	0.00
0.00	0.00	0.00
0.00	0.00	0.00
0.00	0.00	0.00
0.00	0.00	0.00
0.00	0.00	0.00
0.00	0.00	0.00
0.00	0.00	0.00
0.00	0.00	0.00
0.00	0.00	0.00
0.00	0.00	0.00
0.00	0.00	0.00
0.00	0.00	0.00
0.00	0.00	0.00
0.00	0.00	0.00
0.00	0.00	0.00
0.00	0.00	0.00
0.00	0.00	0.00
0.00	0.00	0.00

7	8	9
8	9	10
UC	UC	UC
B_34	C_1	C_40
1363063	824884	2206918
B_34	C_1	C_40
0.63	1.28	1.11
-0.74	-0.53	-0.06
0.28	0.32	-1.21
0.38	-0.68	-0.12
1.03	0.65	-1.26
-0.92	-0.89	-0.17
-0.62	-0.99	-0.79
-0.58	-0.56	-0.23

-0.75	-0.89	-0.03
-0.99	-1.07	0.96
1.19	-0.93	-0.55
0.31	0.77	-0.94
1.39	-1.39	-1.02
-0.41	0.48	-0.41
-0.60	-0.86	-0.43
2.37	0.36	-0.53
-0.33	-0.80	-0.64
-0.23	-1.13	-1.03
-1.09	-0.87	-0.22
0.15	-0.99	-0.89
0.66	-0.36	-0.95
0.26	-0.80	-0.23
-0.16	-0.75	-0.85
-0.98	-0.87	-0.70
-0.68	-0.71	-0.23
-0.36	-0.61	-0.73
1.04	-0.33	-0.97
-0.81	-0.91	0.19
-0.76	-1.54	0.09
1.48	-0.75	-0.70
-1.14	-0.58	-0.54
-0.06	-0.05	-0.90
-0.53	-0.44	-0.63
-0.18	-0.32	-0.36
-0.71	-1.52	0.23
-0.45	-0.20	-0.18
-0.55	-0.95	0.50
-0.70	-0.70	-0.04
-0.42	0.30	-0.13
-0.17	1.03	-0.42
-0.04	1.10	-0.71
0.88	0.03	-1.58
-0.28	-0.23	-0.29
-0.79	-0.79	0.01
-0.48	-0.94	-0.81
1.65	-0.92	-1.72
-0.01	-1.03	-0.88
0.11	1.49	-0.83
-0.21	-0.29	-0.27
0.01	-0.44	-0.50
0.71	-0.53	0.44
-0.49	-0.64	-0.57
-0.38	-0.64	-1.34
-0.23	-0.11	-0.63
0.40	-1.03	-0.83

-0.22	-0.32	-0.34
-1.13	-0.89	0.01
-0.27	0.06	-1.04
0.89	-1.16	-1.52
-0.33	-0.47	-0.55
1.79	-0.56	-1.23
-0.13	0.09	-1.16
-0.60	-0.56	-0.40
-0.26	-0.17	-0.26
-0.02	2.11	1.34
-0.73	-0.97	-0.55
-0.47	-0.61	-0.71
-0.51	-0.90	-0.74
-0.71	-0.94	-1.07
-0.40	-0.37	0.14
1.52	0.51	-0.69
-0.21	1.71	-0.52
-0.64	-0.81	-0.37
-0.54	-1.00	0.74
-0.85	-0.83	-1.06
2.38	0.06	-0.21
-0.83	-0.71	0.46
-0.42	-0.40	-0.28
1.91	0.69	-1.00
0.59	-0.77	-0.80
-1.21	-0.90	1.71
0.99	0.81	-0.82
-0.56	1.68	1.27
0.32	-0.46	-0.48
-0.80	-0.87	-0.62
0.64	-0.03	-0.96
1.82	2.04	0.12
-0.81	-0.77	-0.53
1.06	-0.86	0.03
1.44	-0.46	-0.92
0.20	-0.28	-1.27
-0.28	-0.12	-0.25
1.28	0.33	-1.06
-0.46	-0.87	-0.70
-0.40	-0.57	-0.48
-0.24	-0.50	-0.88
1.44	1.26	-0.98
0.01	2.00	-1.08
-0.60	-0.43	-1.26
-0.61	-0.68	-0.41
-0.14	-0.46	-0.51
-0.75	-0.71	-1.10
0.53	-0.48	-1.25

-0.52	-0.61	-0.31
-0.76	-0.73	-0.31
-0.55	-0.65	-0.56
-0.14	0.97	-1.28
-0.57	-0.60	-0.51
-0.51	-0.82	-0.86
-0.93	-0.97	-0.65
-0.83	-0.40	-0.80
0.50	-0.74	-1.54
-0.22	0.54	-0.77
-0.90	-1.06	0.14
0.74	-0.46	-1.00
0.83	-1.01	-0.58
0.33	-0.27	-0.07
0.30	-0.86	-0.96
0.30	-0.86	-0.96
-0.68	-0.16	-0.48
0.27	-0.09	-0.44
1.64	0.91	-0.97
-0.07	-0.71	-0.04
-0.70	-0.69	-0.55
-0.29	-0.34	-0.37
0.25	0.67	-0.85
-0.91	-0.85	-0.79
0.66	0.73	-1.17
-0.48	0.13	-0.24
0.04	-0.29	-0.64
-0.67	-0.41	-0.46
-0.64	0.34	-1.16
-0.75	-1.14	-0.25
0.07	-0.43	-0.33
-0.19	-0.55	-0.66
2.09	-0.45	-1.03

4	5	6
5	6	7
TGAGGTAGTAGGTTGTATAGTT	AACATTCAACGCTGTCGGTGAGT	TAGCAGCACGTAAATATTGGCG
let-7a-5p	miR-181a-5p	miR-16-5p
8839402	8658137	7939871
-0.72	-0.18	-0.85
-0.61	-1.73	0.11
0.67	-0.76	0.75
-0.13	-1.23	-0.57
2.76	-0.08	0.55
0.91	-0.44	-0.54
0.38	1.03	-0.92
-0.68	0.65	-0.89
-0.12	-1.26	-0.17
-0.53	-1.64	-0.12
-0.86	-1.00	0.38
-0.32	0.27	-0.95
-0.44	-0.24	1.63
-0.21	0.88	-0.87
-0.39	-0.49	0.62
-0.71	-0.42	1.34
0.37	1.77	-0.72
-1.16	0.05	1.14
-1.28	0.11	0.06
-1.12	-0.29	2.80

-0.29	0.23	-0.30
0.24	1.38	-0.93
0.49	1.21	-0.82
0.22	1.19	-0.86
2.92	0.34	-0.78
0.43	1.77	-0.64
0.19	-1.13	1.56
-0.39	-1.79	4.09
-1.01	-1.47	-0.21
-1.33	-1.71	-0.58
-0.29	-1.76	-0.90
-0.71	-1.76	-0.12
2.11	-1.26	3.49
-0.67	1.07	-0.74
-0.96	0.40	-0.43
-0.39	0.31	-0.85
-1.16	-1.01	-0.96
-1.01	0.48	-0.44
-1.38	0.26	0.39
-0.65	2.00	-0.69
-1.87	2.23	-0.96
-2.05	4.38	-1.02
-0.61	0.78	-0.63
-1.31	-0.78	0.37
-1.60	0.14	-0.64
-2.14	3.61	-0.92
-0.89	-0.03	1.12
-0.25	1.96	-0.62
-1.33	0.15	0.55
-1.09	0.70	-0.29
-1.29	1.01	0.81
-0.01	-0.55	-0.82
0.61	-1.08	-0.62
0.11	-0.49	-0.18
0.00	-0.24	-0.64
-0.02	-0.11	-0.54
-0.27	-0.83	0.09
-0.71	-0.57	3.42
0.23	2.54	-0.75
1.34	1.40	0.22
1.35	1.09	0.54
-0.76	-0.97	4.07
0.26	0.62	-0.74
0.75	-0.19	-0.45
0.97	-0.36	0.97
0.04	1.09	-0.75
-1.79	-1.78	2.41
-0.94	-1.67	-0.58
4.47	0.55	-0.30
0.92	0.95	0.13
-0.46	-0.87	-0.73
-1.79	-1.31	4.40
-0.10	-0.99	0.63
-0.47	-0.06	0.70
-1.94	2.07	-1.07
-0.35	-0.32	0.52
-1.02	-0.77	0.00
-0.40	-0.82	0.10
0.12	-1.07	1.38

-0.62	-0.75	2.46
-0.23	-0.23	-0.25
0.00	-0.18	0.36
-1.32	0.81	-0.78
-1.33	0.29	2.10
-0.83	-1.15	4.85
-1.58	0.16	-0.11
-0.69	0.27	0.04
-0.53	-0.10	-0.08
-0.86	-0.51	1.10
-0.65	-0.44	-0.14
1.62	-0.33	0.45
-0.66	-0.42	-0.81
0.91	1.73	-0.78
0.57	-0.45	-0.12
-0.84	0.16	0.08
0	-1	-1
0.00	0.00	0.00
1.00	1.00	1.00
0.61	0.14	0.26

4	5	6
TGAGGTAGTAGGTTGTATAGTT	AACATTCAACGCTGTCGGTGAGT	TAGCAGCACGTAAATATTGGCG
let-7a-5p	miR-181a-5p	miR-16-5p
8839402	8658137	7939871
-0.72	-0.18	-0.85
-0.61	-1.73	0.11
0.67	-0.76	0.75
-0.13	-1.23	-0.57
2.76	-0.08	0.55
0.91	-0.44	-0.54
0.38	1.03	-0.92
-0.68	0.65	-0.89
-0.12	-1.26	-0.17
-0.53	-1.64	-0.12
-0.86	-1.00	0.38
-0.32	0.27	-0.95
-0.44	-0.24	1.63
-0.21	0.88	-0.87
-0.39	-0.49	0.62
-0.71	-0.42	1.34
0.37	1.77	-0.72
-1.16	0.05	1.14
-1.28	0.11	0.06
-1.12	-0.29	2.80
-0.29	0.23	-0.30
0.24	1.38	-0.93
0.49	1.21	-0.82
0.22	1.19	-0.86
2.92	0.34	-0.78
0.43	1.77	-0.64
0.19	-1.13	1.56
-0.39	-1.79	4.09

-1.01	-1.47	-0.21
-1.33	-1.71	-0.58
-0.29	-1.76	-0.90
-0.71	-1.76	-0.12
2.11	-1.26	3.49
-0.67	1.07	-0.74
-0.96	0.40	-0.43
-0.39	0.31	-0.85
[illegible]	[illegible]	[illegible]

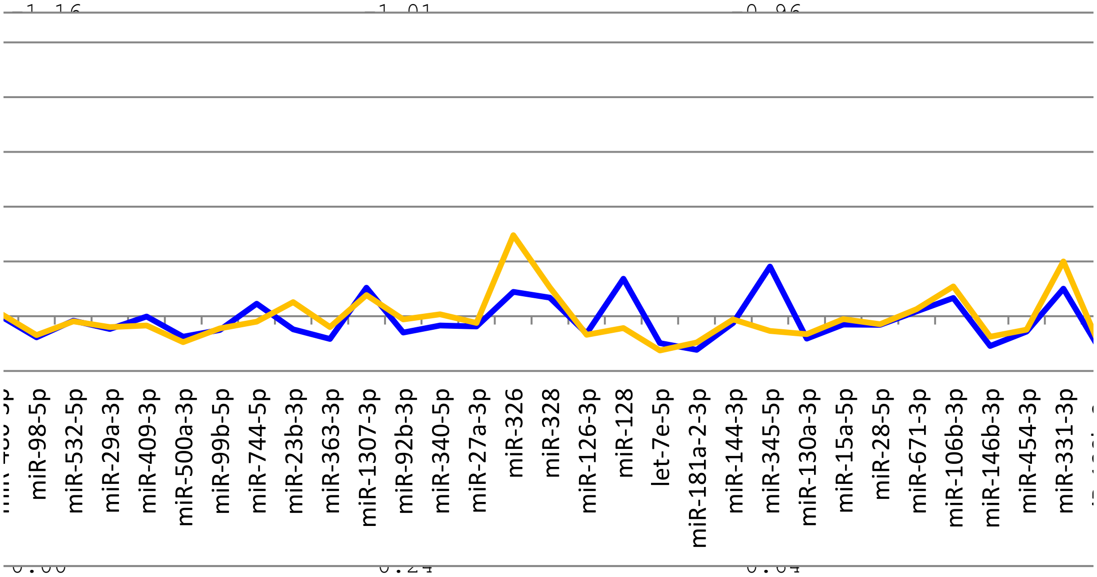

[illegible]	[illegible]	[illegible]
-0.02	-0.11	-0.54
-0.27	-0.83	0.09
-0.71	-0.57	3.42
0.23	2.54	-0.75
1.34	1.40	0.22
1.35	1.09	0.54
-0.76	-0.97	4.07
0.26	0.62	-0.74
0.75	-0.19	-0.45
0.97	-0.36	0.97
0.04	1.09	-0.75
-1.79	-1.78	2.41
-0.94	-1.67	-0.58
4.47	0.55	-0.30
0.92	0.95	0.13
-0.46	-0.87	-0.73
-1.79	-1.31	4.40
-0.10	-0.99	0.63
-0.47	-0.06	0.70
-1.94	2.07	-1.07
-0.35	-0.32	0.52
-1.02	-0.77	0.00
-0.40	-0.82	0.10
0.12	-1.07	1.38
-0.62	-0.75	2.46
-0.23	-0.23	-0.25
0.00	-0.18	0.36
-1.32	0.81	-0.78
-1.33	0.29	2.10
-0.83	-1.15	4.85
-1.58	0.16	-0.11
-0.69	0.27	0.04

-0.53	-0.10	-0.08
-0.86	-0.51	1.10
-0.65	-0.44	-0.14
1.62	-0.33	0.45
-0.66	-0.42	-0.81
0.91	1.73	-0.78
0.57	-0.45	-0.12
-1	0	0

4	5	6
33	96	125
TGTAAACATCCTTGACTGGAAG	AAGGAGCTCACAGTCTATTGAG	TGTAGTGTTTCCTACTTTATGGA
miR-30e-5p	miR-28-5p	miR-142-3p
457362	28163	13338
-0.35	-0.76	-0.37
-0.61	0.69	-0.39
0.21	-0.20	-0.34
-0.18	-0.78	-0.35
0.02	1.80	-0.22
-0.48	-0.03	-0.33
-0.53	-0.24	-0.29
-0.44	-0.50	-0.34
-0.63	-0.88	-0.37
-0.58	-0.73	-0.34
-0.16	0.54	-0.33
-0.51	-0.10	-0.32
3.39	3.20	1.68
-0.59	-0.81	-0.37
-0.35	-0.33	-0.23
0.86	1.85	0.41
-0.47	-0.46	-0.30
-0.16	0.08	-0.30
-0.14	-0.02	-0.27
2.91	1.51	4.54
-0.48	-0.39	-0.27
-0.47	-0.67	-0.35
-0.34	-0.41	-0.27
-0.55	-0.58	-0.30
-0.47	-0.60	-0.39
0.04	-0.69	0.04
1.07	-0.48	0.34
4.26	0.93	4.34
0.41	0.38	-0.23
-0.49	-0.84	-0.36
-0.70	-1.13	-0.39
0.03	-0.49	-0.39
0.25	1.90	-0.39
-0.33	-0.32	-0.24
-0.35	-0.30	-0.27
-0.21	-0.26	-0.05
[illegible]	[illegible]	[illegible]

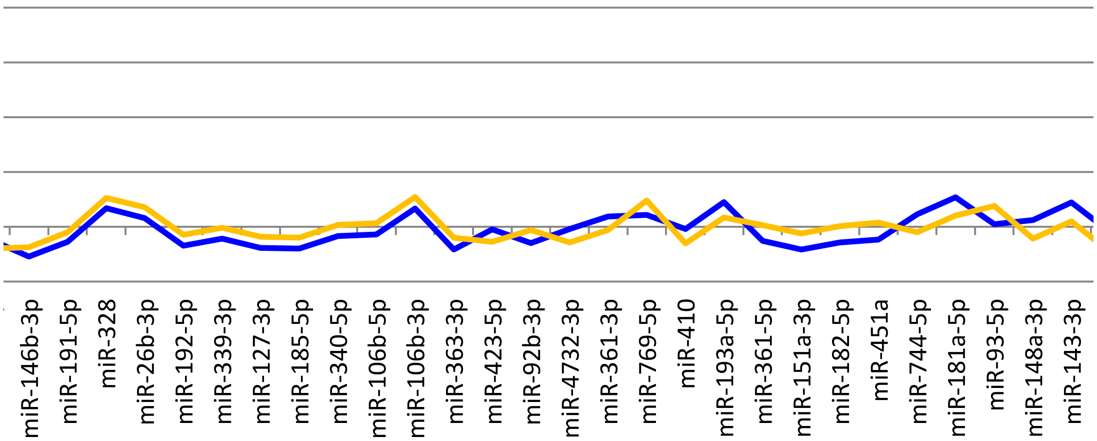

[illegible]	[illegible]	[illegible]
-0.54	-0.79	-0.29
-0.28	-0.38	-0.25
4.64	0.51	11.42
-0.43	-0.41	-0.30
0.64	1.07	-0.15
0.46	1.65	-0.19
4.03	-0.05	5.97
-0.32	-0.97	-0.23
-0.12	-0.79	-0.18
0.33	-0.65	-0.24
-0.36	-0.01	-0.37
1.24	-0.33	-0.34
-0.73	-0.22	-0.39
0.15	-0.28	-0.36
1.06	1.22	-0.33
-0.17	-0.66	-0.28
0.95	2.93	-0.25
-0.06	-0.10	-0.27
0.52	1.72	-0.07
-0.66	-1.15	-0.12
0.34	1.15	0.01
-0.36	-0.02	-0.29
-0.56	-0.44	-0.21
1.50	-0.28	-0.04
0.73	1.02	-0.20
-0.21	-0.77	-0.34
0.18	0.01	-0.17
-0.11	0.45	-0.18
4.21	1.57	6.52
5.16	0.43	6.10
0.05	0.03	-0.18
0.48	0.46	-0.22
0.02	-0.61	-0.22
0.22	0.55	-0.16
-0.03	0.06	-0.20
0.89	0.04	-0.25
-0.47	-0.86	-0.31
-0.02	-0.48	-0.14
-0.08	-0.68	-0.35
0.29	-0.69	-0.39
0.02	0.02	0.03
0.03	0.03	0.05

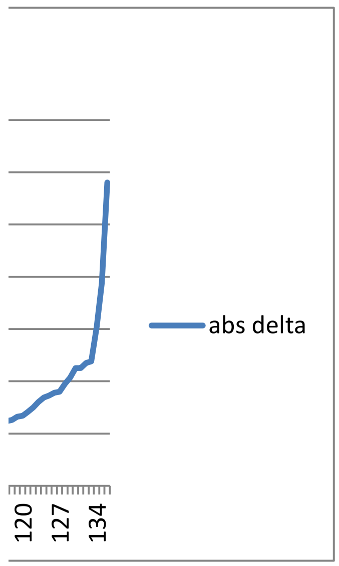
abs delta
120
127
134

2206918	623719	774447	627333	2772220
10	11	12	13	14
10	11	12	13	14
C_40	C_41	C_42	C_44	C_46
UC	UC	UC	UC	UC
17200243	4588607	2634187	1410256	4408275
0.33	0.80	0.73	0.63	0.00
C_40	C_41	C_42	C_44	C_46
14993325	3964888	1859740	782923	1636055
1151739	307256	262633	268518	548517
212328	50961	99661	9784	105685
34851	23541	38127	118333	125439
161620	37177	36863	41106	160662
71100	13196	29489	46656	156161
69561	20511	37797	2814	240682
46792	15577	43820	10515	172526
14990	6321	11878	883	137354
40102	8078	14047	4576	115851
78886	24650	18138	6193	64098
22597	9511	4895	10035	78871
26861	6339	10423	8175	39735
12589	5866	4634	6493	63899
27534	8294	5452	8195	25208
8708	5090	6269	4575	85702
12161	2335	4024	12753	17484
3895	1885	3731	944	56571
7622	2909	3032	2809	26495
10910	4625	7899	1763	37455
9844	4139	7982	2881	16454
6819	2331	2456	2873	23217
9468	3267	2123	1631	17176
3495	1170	2961	1222	26852
3102	1093	6547	325	15993
6392	5178	18805	686	14069
5309	1605	2430	1312	7665
5386	1741	1043	2842	5637
9240	4320	6596	368	8157
7772	1947	4366	1066	9427
5010	1359	3053	969	8717
4682	1287	2430	1563	15733
5236	1265	1977	3252	8322
1221	407	1145	497	22869
461	488	949	271	76995
7293	1770	4041	984	8871
7956	4187	1714	501	7546
10796	4263	8385	988	10993
6151	2995	10557	525	7827
6179	922	3605	576	4504
3316	381	1106	12046	2705
2883	1036	1343	674	6053
1123	485	887	2679	5165
3965	1230	1605	699	3430
2630	1565	2000	165	9282
1702	2131	2015	854	4660

1577	462	933	953	4707
1318	396	1629	293	5009
1707	243	428	2696	2910
265	65	64	72	1555
586	230	1196	310	14430
3027	568	614	125	228
1030	408	1128	225	9671
1275	735	459	153	2961
1197	267	1249	566	4930
691	196	653	348	3067
74	72	101	26	2354
1963	497	739	181	3173
801	122	572	478	4670
708	429	591	688	2787
1406	239	1147	1042	1855
643	345	488	250	5044
560	321	566	449	4143
288	224	600	31	2776
49	31	68	7	701
3665	580	292	1137	702
755	417	405	60	2624
166	358	249	111	5181
208	157	183	40	2302
235	232	291	84	3407
3061	1256	1359	44	427
819	252	390	308	1576
1051	261	244	538	677
424	150	281	79	3887
1357	443	342	141	1607
173	74	286	78	1464
1019	169	192	160	223
1271	276	501	150	880
751	336	930	110	390
467	86	160	758	1084
431	323	606	63	2441
1370	186	345	86	997
459	113	227	306	1046
2109	299	494	297	523
46	138	25	28	7527
296	97	310	35	1681
234	55	122	107	1084
959	144	270	328	511
203	83	229	34	1643
589	166	260	128	1415
306	68	299	368	535
209	120	140	206	512
23	13	3	1	193
185	41	80	229	656
159	49	122	39	755
80	58	81	20	1978
109	45	203	106	1775
264	59	115	194	465
286	79	81	129	472
143	50	179	106	244
121	124	109	11	1562
204	98	57	8	434
158	39	115	28	635
117	85	117	418	461
322	155	603	55	357

256	142	120	18	958
100	32	64	23	1094
82	55	85	78	609
220	101	177	28	587
74	82	52	15	1263
127	118	75	11	682
138	36	159	67	774
115	39	80	90	247
125	26	46	28	695
254	76	127	6	328
89	33	110	110	275
243	49	53	146	339
214	19	82	52	390
65	23	75	26	685
65	23	75	26	685
182	87	119	30	356
163	76	38	32	336
124	74	56	90	518
178	15	57	6	56
102	17	69	35	621
5	3	4	4	457
131	54	35	103	443
84	62	110	23	101
47	12	16	53	434
181	38	85	7	136
54	9	19	69	133
49	37	0	5	37
50	10	23	64	411
126	20	139	13	280
137	69	29	8	345
25	8	16	8	648
78	46	70	31	285
215	84	43	41	215
81	17	24	21	209
60	7	27	19	261
70	24	31	26	245
118	33	88	35	121
222	76	40	17	258
73	11	30	321	180
31	6	32	24	206
14	23	14	1	302
206	46	16	34	112
46	4	21	50	182
85	13	33	9	234
170	27	29	42	136
50	22	12	3	65
21	17	32	23	249
76	30	28	7	185
52	8	15	10	63
52	2	18	36	158
1	1	2	112	327
140	49	12	11	45
435	48	57	6	90
48	28	34	7	164
43	18	21	13	138
68	14	23	20	74
4	4	0	1	118
35	15	20	24	188
94	12	22	24	79

15	17	21	7	493
18	1	20	5	357
100	18	21	106	67
34	20	28	9	81
24	71	26	20	469
7	2	9	1	155
92	35	20	23	103
15	3	1	17	256
35	16	21	12	182
49	23	31	37	99
48	13	27	16	202
12	5	25	9	402
40	8	15	21	36
38	18	5	5	26
30	13	29	9	155
83	21	20	7	220
38	7	12	12	20
79	26	27	18	119
25	5	23	3	292
30	7	5	2	5
54	18	42	7	161
24	16	14	19	53
55	13	23	52	80
22	5	22	3	275
46	6	12	8	16
14	14	15	15	166
30	17	49	7	169
0	1	0	11	0
47	23	43	4	417
24	11	37	10	136
35	8	12	60	27
96	41	37	11	60
17	19	21	8	48
6	1	12	5	108
102	15	12	17	73
17	19	10	6	208
29	4	2	13	62
30	0	17	37	19
30	13	29	6	34
51	3	6	22	61
10	9	6	11	59
24	18	6	1	156
36	4	5	3	2
115	9	22	11	59
20	0	10	13	79
16	3	9	17	117
46	14	8	30	57
29	10	10	2	23
26	0	10	16	75
20	3	20	0	44
51	11	2	14	43
17	4	17	4	109
34	5	24	12	69
11	3	13	12	94
10	7	22	5	103
3	0	2	0	14
69	6	22	3	52
54	71	113	1	38
36	8	9	6	46

19	7	13	3	76
22	15	6	8	78
17	4	3	1	3
28	6	14	18	25
23	3	2	22	29
33	7	7	9	25
26	10	9	4	43
58	13	16	3	56
13	7	20	2	115
81	10	9	1	27
4	1	11	0	75
18	6	6	9	64
21	4	7	5	51
9	4	4	7	20
4	5	6	3	39
4	4	4	1	125
6	1	11	3	123
29	6	9	6	31
5	4	2	4	10
12	3	11	7	86
20	12	31	1	87
12	5	9	3	26
12	5	9	3	26
23	1	3	4	1
19	13	3	2	26
13	8	5	2	13
6	3	2	5	7
18	7	10	9	47
6	2	3	6	49
6	8	3	4	54
20	12	6	7	30
4	2	6	7	14
4	4	9	6	107
3	4	23	2	10
11	7	7	8	25
46	9	7	2	43
1	2	0	0	30
10	8	5	5	40
12	1	9	3	169
15	0	12	16	15
26	3	2	5	7
11	1	9	3	165
7	0	8	5	22
6	1	3	3	4
10	4	1	3	80
13	5	2	9	27
1	1	0	0	8
4	0	5	18	17
27	5	7	4	7
17	2	4	3	18
14	2	3	4	8
15	1	7	1	19
0	6	1	0	29
8	3	3	0	15
14	3	4	2	71
274	7	14	4	43
12	4	3	12	15
25	0	2	6	69
28	7	13	1	18

13	1	2	1	2
12	2	6	6	30
15	2	3	0	5
11	2	6	6	22
16	3	5	3	18
9	1	8	2	18
14	3	4	2	65
16	3	0	7	7
7	3	0	0	28
2	1	5	0	60
10	4	17	0	21
12	3	2	2	8
4	3	3	0	59
0	0	0	31	39
6	2	4	3	4
16	8	11	4	62
40	9	7	2	14
3	3	2	2	48
3	0	1	3	35
5	1	0	0	6
6	1	3	8	3
4	0	8	5	14
3	0	2	4	14
10	7	2	11	3
6	1	9	5	33
6	0	3	6	27
3	1	2	2	24
5	2	2	7	17
7	1	1	1	14
7	0	5	3	27
18	7	10	5	13
5	2	7	2	45
1	0	6	0	54
17	8	2	4	17
15	6	11	1	25
9	5	2	2	18
2	2	3	4	9
1	2	0	3	17
1	0	1	3	17
6	1	5	3	17
5	4	11	0	57
3	2	1	5	25
10	2	1	0	2
5	2	4	0	17
14	2	4	1	2
4	5	3	1	35
13	2	2	2	11
11	6	2	0	15
5	8	3	0	8
2	0	1	2	9
4	2	2	1	7
0	0	0	0	31
8	4	1	1	17
2	1	5	0	16
7	5	4	1	10
4	3	4	4	25
6	0	1	9	6
2	1	2	0	1
3	1	1	2	13

10	4	2	7	14
3	2	2	6	21
6	2	0	1	10
3	2	4	4	22
4	1	4	12	6
9	2	4	5	13
10	2	3	2	10
2	0	0	0	1
3	2	3	1	23
4	1	3	2	17
3	2	2	1	14
3	1	0	2	18
6	0	12	1	8
0	0	1	0	73
6	0	6	2	8
4	8	1	2	28
2	0	1	0	23
7	1	0	0	14
1	1	0	0	28
15	3	6	1	16
1	2	0	3	26
3	1	1	2	27
3	3	4	0	7
9	3	2	0	10
11	2	0	3	1
0	0	3	0	1
1	0	0	0	27
1	0	1	1	58
3	0	2	0	6
4	1	2	2	10
2	0	3	0	1
3	2	0	2	9
0	1	1	1	11
4	1	2	6	3
0	0	1	1	15
6	1	1	0	0
5	1	1	1	9
5	0	1	1	2
3	1	1	2	8
2	0	1	3	6
1	1	2	10	10
1	1	3	1	9
1	0	2	0	3
3	1	1	0	5
3	2	4	4	3
0	0	2	1	13
2	0	0	1	12
4	0	0	4	8
1	1	4	0	17
4	0	0	0	20
3	0	1	1	4
3	1	0	0	0
1	1	0	0	20
1	3	0	1	2
1	0	2	1	6
8	3	4	0	8
6	0	0	3	8
3	1	5	0	4
0	1	0	0	1

6	0	1	2	8
2	1	3	3	7
3	0	4	0	5
1	0	1	1	12
1	1	1	1	6
6	1	2	0	2
2	2	1	2	7
3	0	0	1	21
6	1	0	1	3
2	0	3	2	0
1	0	2	0	23
1	0	0	0	9
0	0	1	0	1
1	0	0	2	16
1	1	0	2	5
2	0	1	2	6
8	2	2	1	1
3	1	0	4	2
0	0	0	0	10
1	1	0	0	0
2	1	0	1	3
0	0	3	1	26
3	0	1	1	2
2	0	3	5	2
4	1	0	1	3
0	1	5	0	18
0	0	0	2	8
1	1	2	0	13
6	2	1	0	5
3	1	2	1	10
1	0	0	0	14
2	0	1	2	4
3	1	1	1	8
2	1	0	8	2
0	1	1	0	18
3	0	0	0	7
4	0	1	2	1
2	1	2	3	3
3	0	1	1	8
5	0	1	0	0
0	0	0	0	1
0	0	0	0	5
3	0	3	4	2
0	0	2	0	5
4	0	0	1	6
1	0	1	0	2
0	0	1	0	24
2	1	3	0	3
4	1	1	2	3
5	1	1	0	3
0	0	1	1	6
5	1	3	0	9
8	0	0	1	2
1	0	2	0	13
1	0	0	0	6
4	0	3	0	4
0	0	1	0	6
0	1	0	1	0
5	1	1	0	9

0	0	0	1	5
2	0	0	2	5
0	0	1	5	2
3	0	3	1	2
8	0	2	0	3
1	0	5	0	15
0	0	2	1	1
1	0	0	1	13
2	0	0	0	4
1	0	0	0	5
1	0	0	0	0
0	1	2	2	3
0	0	0	0	14
1	0	0	0	0
3	0	1	0	3
1	2	0	0	3
0	0	1	0	4
0	0	0	0	1
0	0	1	0	14
6	1	0	0	0
2	2	0	0	7
0	0	1	0	7
0	0	1	0	13
1	1	5	1	3
0	2	0	2	2
1	1	0	0	6
0	0	0	0	4
5	0	0	0	0
0	0	0	1	8
4	1	0	0	3
4	0	0	0	0
0	0	1	0	2
3	2	1	0	0
0	0	0	1	5
1	0	0	0	2
0	0	1	0	4
2	0	1	1	1
0	0	0	0	8
1	0	1	0	6
1	1	1	0	4
0	0	0	0	4
1	0	0	0	3
0	0	3	1	2
1	0	0	1	3
2	0	0	0	1
8	2	1	0	3
3	0	2	0	1
5	2	0	0	0
0	1	0	0	2
2	0	1	1	1
1	0	0	0	4
1	0	0	0	0
0	0	1	1	4
0	0	0	0	2
1	1	0	2	1
3	0	0	0	2
1	0	0	0	2
0	1	1	3	3
0	0	0	0	4

1	0	1	0	8
0	1	0	2	3
0	1	0	2	1
0	0	1	0	2
0	0	0	2	1
0	0	0	0	5
2	0	0	2	1
6	0	3	0	1
1	0	1	1	0
3	0	0	0	0
1	0	0	0	0
2	2	0	0	0
0	0	0	0	0
C_40	C_41	C_42	C_44	C_46
UC	UC	UC	UC	UC
14993325	3964888	1859740	782923	1636055
27	27	23	18	13
1.23	1.19	0.24	-0.75	-1.85

0.89	0.99	0.03	0.53	0.99
4	1	27	18	2
1	1	1	1	1
10	11	12	13	14
10	11	12	13	14
C_41	C_42	C_44	C_46	C_47
UC	UC	UC	UC	UC
623719	774447	627333	2772220	1217409
C_41	C_42	C_44	C_46	C_47
307256	262633	268518	548517	620936
50961	99661	9784	105685	53388
23541	38127	118333	125439	104165
37177	36863	41106	160662	85023
13196	29489	46656	156161	112601
20511	37797	2814	240682	8973
15577	43820	10515	172526	17934
6321	11878	883	137354	1841
8078	14047	4576	115851	5596
24650	18138	6193	64098	16782
9511	4895	10035	78871	6932
6339	10423	8175	39735	17594
5866	4634	6493	63899	5475
8294	5452	8195	25208	28330
5090	6269	4575	85702	1335
2335	4024	12753	17484	13042
1885	3731	944	56571	1022
2909	3032	2809	26495	4615
4625	7899	1763	37455	2969

4139	7982	2881	16454	4816
2331	2456	2873	23217	5000
3267	2123	1631	17176	4814
1170	2961	1222	26852	1009
1093	6547	325	15993	1373
5178	18805	686	14069	870
1605	2430	1312	7665	3686
1741	1043	2842	5637	6404
4320	6596	368	8157	1026
1947	4366	1066	9427	2036
1359	3053	969	8717	1998
1287	2430	1563	15733	1630
1265	1977	3252	8322	5761
407	1145	497	22869	788
488	949	271	76995	433
1770	4041	984	8871	1927
4187	1714	501	7546	4579
4263	8385	988	10993	2027
2995	10557	525	7827	1363
922	3605	576	4504	4960
381	1106	12046	2705	5636
1036	1343	674	6053	3358
485	887	2679	5165	3586
1230	1605	699	3430	2625
1565	2000	165	9282	300
2131	2015	854	4660	1427
462	933	953	4707	976
396	1629	293	5009	625
243	428	2696	2910	2186
65	64	72	1555	93
230	1196	310	14430	312
568	614	125	228	1635
408	1128	225	9671	438
735	459	153	2961	1098
267	1249	566	4930	780
196	653	348	3067	341
72	101	26	2354	37
497	739	181	3173	598
122	572	478	4670	676
429	591	688	2787	791
239	1147	1042	1855	1146
345	488	250	5044	612
321	566	449	4143	737
224	600	31	2776	68
31	68	7	701	42
580	292	1137	702	1509
417	405	60	2624	251
358	249	111	5181	117
157	183	40	2302	99
232	291	84	3407	241
1256	1359	44	427	398
252	390	308	1576	580
261	244	538	677	1487
150	281	79	3887	70
443	342	141	1607	250
74	286	78	1464	133
169	192	160	223	809
276	501	150	880	345
336	930	110	390	535

86	160	758	1084	817
323	606	63	2441	376
186	345	86	997	233
113	227	306	1046	766
299	494	297	523	845
138	25	28	7527	58
97	310	35	1681	71
55	122	107	1084	397
144	270	328	511	628
83	229	34	1643	59
166	260	128	1415	88
68	299	368	535	502
120	140	206	512	338
13	3	1	193	16
41	80	229	656	187
49	122	39	755	51
58	81	20	1978	37
45	203	106	1775	73
59	115	194	465	330
79	81	129	472	332
50	179	106	244	286
124	109	11	1562	32
98	57	8	434	247
39	115	28	635	105
85	117	418	461	299
155	603	55	357	79
142	120	18	958	72
32	64	23	1094	27
55	85	78	609	167
101	177	28	587	82
82	52	15	1263	33
118	75	11	682	47
36	159	67	774	92
39	80	90	247	177
26	46	28	695	214
76	127	6	328	39
33	110	110	275	127
49	53	146	339	141
19	82	52	390	220
23	75	26	685	30
23	75	26	685	30
87	119	30	356	217
76	38	32	336	111
74	56	90	518	208
15	57	6	56	49
17	69	35	621	68
3	4	4	457	2
54	35	103	443	113
62	110	23	101	48
12	16	53	434	144
38	85	7	136	232
9	19	69	133	167
37	0	5	37	49
10	23	64	411	46
20	139	13	280	50
69	29	8	345	80
8	16	8	648	14
46	70	31	285	76
84	43	41	215	45

17	24	21	209	58
7	27	19	261	24
24	31	26	245	80
33	88	35	121	109
76	40	17	258	50
11	30	321	180	163
6	32	24	206	21
23	14	1	302	6
46	16	34	112	177
4	21	50	182	68
13	33	9	234	16
27	29	42	136	28
22	12	3	65	55
17	32	23	249	14
30	28	7	185	17
8	15	10	63	21
2	18	36	158	64
1	2	112	327	43
49	12	11	45	69
48	57	6	90	44
28	34	7	164	70
18	21	13	138	43
14	23	20	74	88
4	0	1	118	1
15	20	24	188	46
12	22	24	79	107
17	21	7	493	3
1	20	5	357	7
18	21	106	67	83
20	28	9	81	45
71	26	20	469	121
2	9	1	155	4
35	20	23	103	97
3	1	17	256	0
16	21	12	182	8
23	31	37	99	61
13	27	16	202	29
5	25	9	402	9
8	15	21	36	46
18	5	5	26	21
13	29	9	155	10
21	20	7	220	54
7	12	12	20	67
26	27	18	119	97
5	23	3	292	5
7	5	2	5	40
18	42	7	161	0
16	14	19	53	40
13	23	52	80	72
5	22	3	275	5
6	12	8	16	42
14	15	15	166	24
17	49	7	169	8
1	0	11	0	2
23	43	4	417	3
11	37	10	136	19
8	12	60	27	42
41	37	11	60	32
19	21	8	48	15

1	12	5	108	1
15	12	17	73	34
19	10	6	208	17
4	2	13	62	26
0	17	37	19	57
13	29	6	34	18
3	6	22	61	80
9	6	11	59	22
18	6	1	156	3
4	5	3	2	34
9	22	11	59	59
0	10	13	79	40
3	9	17	117	23
14	8	30	57	56
10	10	2	23	48
0	10	16	75	17
3	20	0	44	5
11	2	14	43	66
4	17	4	109	6
5	24	12	69	23
3	13	12	94	11
7	22	5	103	12
0	2	0	14	1
6	22	3	52	16
71	113	1	38	7
8	9	6	46	28
7	13	3	76	9
15	6	8	78	19
4	3	1	3	7
6	14	18	25	47
3	2	22	29	14
7	7	9	25	27
10	9	4	43	42
13	16	3	56	9
7	20	2	115	6
10	9	1	27	27
1	11	0	75	1
6	6	9	64	32
4	7	5	51	2
4	4	7	20	20
5	6	3	39	19
4	4	1	125	2
1	11	3	123	2
6	9	6	31	6
4	2	4	10	4
3	11	7	86	1
12	31	1	87	0
5	9	3	26	4
5	9	3	26	4
1	3	4	1	27
13	3	2	26	12
8	5	2	13	7
3	2	5	7	10
7	10	9	47	11
2	3	6	49	11
8	3	4	54	3
12	6	7	30	11
2	6	7	14	10
4	9	6	107	6

4	23	2	10	6
7	7	8	25	9
9	7	2	43	6
2	0	0	30	0
8	5	5	40	12
1	9	3	169	4
0	12	16	15	18
3	2	5	7	30
1	9	3	165	4
0	8	5	22	15
1	3	3	4	16
4	1	3	80	7
5	2	9	27	12
1	0	0	8	0
0	5	18	17	17
5	7	4	7	34
2	4	3	18	26
2	3	4	8	10
1	7	1	19	2
6	1	0	29	0
3	3	0	15	17
3	4	2	71	2
7	14	4	43	95
4	3	12	15	15
0	2	6	69	9
7	13	1	18	12
1	2	1	2	27
2	6	6	30	10
2	3	0	5	9
2	6	6	22	10
3	5	3	18	13
1	8	2	18	14
3	4	2	65	2
3	0	7	7	13
3	0	0	28	1
1	5	0	60	3
4	17	0	21	0
3	2	2	8	5
3	3	0	59	0
0	0	31	39	22
2	4	3	4	7
8	11	4	62	3
9	7	2	14	13
3	2	2	48	8
0	1	3	35	4
1	0	0	6	2
1	3	8	3	10
0	8	5	14	19
0	2	4	14	10
7	2	11	3	21
1	9	5	33	3
0	3	6	27	15
1	2	2	24	23
2	2	7	17	17
1	1	1	14	11
0	5	3	27	5
7	10	5	13	7
2	7	2	45	0
0	6	0	54	3

8	2	4	17	6
6	11	1	25	2
5	2	2	18	10
2	3	4	9	1
2	0	3	17	6
0	1	3	17	5
1	5	3	17	6
4	11	0	57	1
2	1	5	25	4
2	1	0	2	3
2	4	0	17	8
2	4	1	2	4
5	3	1	35	4
2	2	2	11	10
6	2	0	15	2
8	3	0	8	15
0	1	2	9	3
2	2	1	7	14
0	0	0	31	0
4	1	1	17	2
1	5	0	16	2
5	4	1	10	2
3	4	4	25	6
0	1	9	6	9
1	2	0	1	0
1	1	2	13	9
4	2	7	14	4
2	2	6	21	16
2	0	1	10	1
2	4	4	22	5
1	4	12	6	5
2	4	5	13	2
2	3	2	10	15
0	0	0	1	0
2	3	1	23	4
1	3	2	17	7
2	2	1	14	3
1	0	2	18	0
0	12	1	8	9
0	1	0	73	0
0	6	2	8	13
8	1	2	28	6
0	1	0	23	2
1	0	0	14	1
1	0	0	28	1
3	6	1	16	2
2	0	3	26	4
1	1	2	27	2
3	4	0	7	5
3	2	0	10	4
2	0	3	1	5
0	3	0	1	1
0	0	0	27	0
0	1	1	58	0
0	2	0	6	6
1	2	2	10	3
0	3	0	1	6
2	0	2	9	1
1	1	1	11	4

1	2	6	3	5
0	1	1	15	0
1	1	0	0	6
1	1	1	9	2
0	1	1	2	10
1	1	2	8	8
0	1	3	6	2
1	2	10	10	1
1	3	1	9	0
0	2	0	3	2
1	1	0	5	10
2	4	4	3	4
0	2	1	13	2
0	0	1	12	0
0	0	4	8	4
1	4	0	17	1
0	0	0	20	1
0	1	1	4	4
1	0	0	0	0
1	0	0	20	2
3	0	1	2	3
0	2	1	6	5
3	4	0	8	2
0	0	3	8	3
1	5	0	4	6
1	0	0	1	2
0	1	2	8	2
1	3	3	7	0
0	4	0	5	0
0	1	1	12	1
1	1	1	6	0
1	2	0	2	3
2	1	2	7	5
0	0	1	21	0
1	0	1	3	6
0	3	2	0	6
0	2	0	23	0
0	0	0	9	1
0	1	0	1	0
0	0	2	16	0
1	0	2	5	3
0	1	2	6	1
2	2	1	1	5
1	0	4	2	0
0	0	0	10	0
1	0	0	0	1
1	0	1	3	2
0	3	1	26	1
0	1	1	2	4
0	3	5	2	3
1	0	1	3	1
1	5	0	18	0
0	0	2	8	1
1	2	0	13	1
2	1	0	5	4
1	2	1	10	1
0	0	0	14	0
0	1	2	4	0
1	1	1	8	3

1	0	8	2	8
1	1	0	18	4
0	0	0	7	2
0	1	2	1	0
1	2	3	3	0
0	1	1	8	1
0	1	0	0	1
0	0	0	1	0
0	0	0	5	0
0	3	4	2	2
0	2	0	5	0
0	0	1	6	3
0	1	0	2	0
0	1	0	24	0
1	3	0	3	7
1	1	2	3	2
1	1	0	3	4
0	1	1	6	0
1	3	0	9	0
0	0	1	2	13
0	2	0	13	1
0	0	0	6	0
0	3	0	4	1
0	1	0	6	0
1	0	1	0	0
1	1	0	9	2
0	0	1	5	1
0	0	2	5	3
0	1	5	2	4
0	3	1	2	1
0	2	0	3	0
0	5	0	15	0
0	2	1	1	0
0	0	1	13	0
0	0	0	4	1
0	0	0	5	0
0	0	0	0	3
1	2	2	3	1
0	0	0	14	0
0	0	0	0	1
0	1	0	3	1
2	0	0	3	0
0	1	0	4	1
0	0	0	1	0
0	1	0	14	0
1	0	0	0	1
2	0	0	7	0
0	1	0	7	0
0	1	0	13	0
1	5	1	3	3
2	0	2	2	3
1	0	0	6	0
0	0	0	4	6
0	0	0	0	2
0	0	1	8	0
1	0	0	3	0
0	0	0	0	0
0	1	0	2	3
2	1	0	0	0

0	0	1	5	0
0	0	0	2	2
0	1	0	4	1
0	1	1	1	1
0	0	0	8	0
0	1	0	6	0
1	1	0	4	7
0	0	0	4	0
0	0	0	3	2
0	3	1	2	1
0	0	1	3	1
0	0	0	1	0
2	1	0	3	0
0	2	0	1	1
2	0	0	0	3
1	0	0	2	1
0	1	1	1	5
0	0	0	4	0
0	0	0	0	0
0	1	1	4	0
0	0	0	2	0
1	0	2	1	1
0	0	0	2	3
0	0	0	2	0
1	1	3	3	1
0	0	0	4	0
0	1	0	8	0
1	0	2	3	1
1	0	2	1	0
0	1	0	2	0
0	0	2	1	0
0	0	0	5	1
0	0	2	1	3
0	3	0	1	2
0	1	1	0	0
0	0	0	0	1
0	0	0	0	1
2	0	0	0	1

0.89	0.99	0.03	0.53	0.99
4	1	27	18	2
1	1	1	1	1
10	11	12	13	14
10	11	12	13	14
C_41	C_42	C_44	C_46	C_47
UC	UC	UC	UC	UC
623719	774447	627333	2772220	1217409
UC	UC	UC	UC	UC
307256	262633	268518	548517	620936
50961	99661	9784	105685	53388
23541	38127	118333	125439	104165
37177	36863	41106	160662	85023
13196	29489	46656	156161	112601

20511	37797	2814	240682	8973
15577	43820	10515	172526	17934
6321	11878	883	137354	1841
8078	14047	4576	115851	5596
24650	18138	6193	64098	16782
9511	4895	10035	78871	6932
6339	10423	8175	39735	17594
5866	4634	6493	63899	5475
8294	5452	8195	25208	28330
5090	6269	4575	85702	1335
2335	4024	12753	17484	13042
1885	3731	944	56571	1022
2909	3032	2809	26495	4615
4625	7899	1763	37455	2969
4139	7982	2881	16454	4816
2331	2456	2873	23217	5000
3267	2123	1631	17176	4814
1170	2961	1222	26852	1009
1093	6547	325	15993	1373
5178	18805	686	14069	870
1605	2430	1312	7665	3686
1741	1043	2842	5637	6404
4320	6596	368	8157	1026
1947	4366	1066	9427	2036
1359	3053	969	8717	1998
1287	2430	1563	15733	1630
1265	1977	3252	8322	5761
407	1145	497	22869	788
488	949	271	76995	433
1770	4041	984	8871	1927
4187	1714	501	7546	4579
4263	8385	988	10993	2027
2995	10557	525	7827	1363
922	3605	576	4504	4960
381	1106	12046	2705	5636
1036	1343	674	6053	3358
485	887	2679	5165	3586
1230	1605	699	3430	2625
1565	2000	165	9282	300
2131	2015	854	4660	1427
462	933	953	4707	976
396	1629	293	5009	625
243	428	2696	2910	2186
65	64	72	1555	93
230	1196	310	14430	312
568	614	125	228	1635
408	1128	225	9671	438
735	459	153	2961	1098
267	1249	566	4930	780
196	653	348	3067	341
72	101	26	2354	37
497	739	181	3173	598
122	572	478	4670	676
429	591	688	2787	791
239	1147	1042	1855	1146
345	488	250	5044	612
321	566	449	4143	737
224	600	31	2776	68
31	68	7	701	42

580	292	1137	702	1509
417	405	60	2624	251
358	249	111	5181	117
157	183	40	2302	99
232	291	84	3407	241
1256	1359	44	427	398
252	390	308	1576	580
261	244	538	677	1487
150	281	79	3887	70
443	342	141	1607	250
74	286	78	1464	133
169	192	160	223	809
276	501	150	880	345
336	930	110	390	535
86	160	758	1084	817
323	606	63	2441	376
186	345	86	997	233
113	227	306	1046	766
299	494	297	523	845
138	25	28	7527	58
97	310	35	1681	71
55	122	107	1084	397
144	270	328	511	628
83	229	34	1643	59
166	260	128	1415	88
68	299	368	535	502
120	140	206	512	338
13	3	1	193	16
41	80	229	656	187
49	122	39	755	51
58	81	20	1978	37
45	203	106	1775	73
59	115	194	465	330
79	81	129	472	332
50	179	106	244	286
124	109	11	1562	32
98	57	8	434	247
39	115	28	635	105
85	117	418	461	299
155	603	55	357	79
142	120	18	958	72
32	64	23	1094	27
55	85	78	609	167
101	177	28	587	82
82	52	15	1263	33
118	75	11	682	47
36	159	67	774	92
39	80	90	247	177
26	46	28	695	214
76	127	6	328	39
33	110	110	275	127
49	53	146	339	141
19	82	52	390	220
23	75	26	685	30
23	75	26	685	30
87	119	30	356	217
76	38	32	336	111
74	56	90	518	208
15	57	6	56	49

17	69	35	621	68
3	4	4	457	2
54	35	103	443	113
62	110	23	101	48
12	16	53	434	144
38	85	7	136	232
9	19	69	133	167
37	0	5	37	49
10	23	64	411	46
20	139	13	280	50
69	29	8	345	80
8	16	8	648	14
46	70	31	285	76
84	43	41	215	45
17	24	21	209	58
7	27	19	261	24
24	31	26	245	80
33	88	35	121	109
76	40	17	258	50
11	30	321	180	163
6	32	24	206	21
23	14	1	302	6
46	16	34	112	177
4	21	50	182	68
13	33	9	234	16
27	29	42	136	28
22	12	3	65	55
17	32	23	249	14
30	28	7	185	17
8	15	10	63	21
2	18	36	158	64
1	2	112	327	43
49	12	11	45	69
48	57	6	90	44
28	34	7	164	70
18	21	13	138	43
14	23	20	74	88
4	0	1	118	1
15	20	24	188	46
12	22	24	79	107
17	21	7	493	3
1	20	5	357	7
18	21	106	67	83
20	28	9	81	45
71	26	20	469	121
2	9	1	155	4
35	20	23	103	97
3	1	17	256	0
16	21	12	182	8
23	31	37	99	61
13	27	16	202	29
5	25	9	402	9
8	15	21	36	46
18	5	5	26	21
13	29	9	155	10
21	20	7	220	54
7	12	12	20	67
26	27	18	119	97
5	23	3	292	5

7	5	2	5	40
18	42	7	161	0
16	14	19	53	40
13	23	52	80	72
5	22	3	275	5
6	12	8	16	42
14	15	15	166	24
17	49	7	169	8
1	0	11	0	2
23	43	4	417	3
11	37	10	136	19
8	12	60	27	42
41	37	11	60	32
19	21	8	48	15
1	12	5	108	1
15	12	17	73	34
19	10	6	208	17
4	2	13	62	26
0	17	37	19	57
13	29	6	34	18
3	6	22	61	80
9	6	11	59	22
18	6	1	156	3
4	5	3	2	34
9	22	11	59	59
0	10	13	79	40
3	9	17	117	23
14	8	30	57	56
10	10	2	23	48
0	10	16	75	17
3	20	0	44	5
11	2	14	43	66
4	17	4	109	6
5	24	12	69	23
3	13	12	94	11
7	22	5	103	12
0	2	0	14	1
6	22	3	52	16
71	113	1	38	7
8	9	6	46	28
7	13	3	76	9
15	6	8	78	19
4	3	1	3	7
6	14	18	25	47
3	2	22	29	14
7	7	9	25	27
10	9	4	43	42
13	16	3	56	9
7	20	2	115	6
10	9	1	27	27
1	11	0	75	1
6	6	9	64	32
4	7	5	51	2
4	4	7	20	20
5	6	3	39	19
4	4	1	125	2
1	11	3	123	2
6	9	6	31	6
4	2	4	10	4

3	11	7	86	1
12	31	1	87	0
5	9	3	26	4
5	9	3	26	4
1	3	4	1	27
13	3	2	26	12
8	5	2	13	7
3	2	5	7	10
7	10	9	47	11
2	3	6	49	11
8	3	4	54	3
12	6	7	30	11
2	6	7	14	10
4	9	6	107	6
4	23	2	10	6
7	7	8	25	9
9	7	2	43	6
2	0	0	30	0
8	5	5	40	12
1	9	3	169	4
0	12	16	15	18
3	2	5	7	30
1	9	3	165	4
0	8	5	22	15
1	3	3	4	16
4	1	3	80	7
5	2	9	27	12
1	0	0	8	0
0	5	18	17	17
5	7	4	7	34
2	4	3	18	26
2	3	4	8	10
1	7	1	19	2
6	1	0	29	0
3	3	0	15	17
3	4	2	71	2
7	14	4	43	95
4	3	12	15	15
0	2	6	69	9
7	13	1	18	12
1	2	1	2	27
2	6	6	30	10
2	3	0	5	9
2	6	6	22	10
3	5	3	18	13
1	8	2	18	14
3	4	2	65	2
3	0	7	7	13
3	0	0	28	1
1	5	0	60	3
4	17	0	21	0
3	2	2	8	5
3	3	0	59	0
0	0	31	39	22
2	4	3	4	7
8	11	4	62	3
9	7	2	14	13
3	2	2	48	8
0	1	3	35	4

1	0	0	6	2
1	3	8	3	10
0	8	5	14	19
0	2	4	14	10
7	2	11	3	21
1	9	5	33	3
0	3	6	27	15
1	2	2	24	23
2	2	7	17	17
1	1	1	14	11
0	5	3	27	5
7	10	5	13	7
2	7	2	45	0
0	6	0	54	3
8	2	4	17	6
6	11	1	25	2
5	2	2	18	10
2	3	4	9	1
2	0	3	17	6
0	1	3	17	5
1	5	3	17	6
4	11	0	57	1
2	1	5	25	4
2	1	0	2	3
2	4	0	17	8
2	4	1	2	4
5	3	1	35	4
2	2	2	11	10
6	2	0	15	2
8	3	0	8	15
0	1	2	9	3
2	2	1	7	14
0	0	0	31	0
4	1	1	17	2
1	5	0	16	2
5	4	1	10	2
3	4	4	25	6
0	1	9	6	9
1	2	0	1	0
1	1	2	13	9
4	2	7	14	4
2	2	6	21	16
2	0	1	10	1
2	4	4	22	5
1	4	12	6	5
2	4	5	13	2
2	3	2	10	15
0	0	0	1	0
2	3	1	23	4
1	3	2	17	7
2	2	1	14	3
1	0	2	18	0
0	12	1	8	9
0	1	0	73	0
0	6	2	8	13
8	1	2	28	6
0	1	0	23	2
1	0	0	14	1
1	0	0	28	1

3	6	1	16	2
2	0	3	26	4
1	1	2	27	2
3	4	0	7	5
3	2	0	10	4
2	0	3	1	5
0	3	0	1	1
0	0	0	27	0
0	1	1	58	0
0	2	0	6	6
1	2	2	10	3
0	3	0	1	6
2	0	2	9	1
1	1	1	11	4
1	2	6	3	5
0	1	1	15	0
1	1	0	0	6
1	1	1	9	2
0	1	1	2	10
1	1	2	8	8
0	1	3	6	2
1	2	10	10	1
1	3	1	9	0
0	2	0	3	2
1	1	0	5	10
2	4	4	3	4
0	2	1	13	2
0	0	1	12	0
0	0	4	8	4
1	4	0	17	1
0	0	0	20	1
0	1	1	4	4
1	0	0	0	0
1	0	0	20	2
3	0	1	2	3
0	2	1	6	5
3	4	0	8	2
0	0	3	8	3
1	5	0	4	6
1	0	0	1	2
0	1	2	8	2
1	3	3	7	0
0	4	0	5	0
0	1	1	12	1
1	1	1	6	0
1	2	0	2	3
2	1	2	7	5
0	0	1	21	0
1	0	1	3	6
0	3	2	0	6
0	2	0	23	0
0	0	0	9	1
0	1	0	1	0
0	0	2	16	0
1	0	2	5	3
0	1	2	6	1
2	2	1	1	5
1	0	4	2	0
0	0	0	10	0

1	0	0	0	1
1	0	1	3	2
0	3	1	26	1
0	1	1	2	4
0	3	5	2	3
1	0	1	3	1
1	5	0	18	0
0	0	2	8	1
1	2	0	13	1
2	1	0	5	4
1	2	1	10	1
0	0	0	14	0
0	1	2	4	0
1	1	1	8	3
1	0	8	2	8
1	1	0	18	4
0	0	0	7	2
0	1	2	1	0
1	2	3	3	0
0	1	1	8	1
0	1	0	0	1
0	0	0	1	0
0	0	0	5	0
0	3	4	2	2
0	2	0	5	0
0	0	1	6	3
0	1	0	2	0
0	1	0	24	0
1	3	0	3	7
1	1	2	3	2
1	1	0	3	4
0	1	1	6	0
1	3	0	9	0
0	0	1	2	13
0	2	0	13	1
0	0	0	6	0
0	3	0	4	1
0	1	0	6	0
1	0	1	0	0
1	1	0	9	2
0	0	1	5	1
0	0	2	5	3
0	1	5	2	4
0	3	1	2	1
0	2	0	3	0
0	5	0	15	0
0	2	1	1	0
0	0	1	13	0
0	0	0	4	1
0	0	0	5	0
0	0	0	0	3
1	2	2	3	1
0	0	0	14	0
0	0	0	0	1
0	1	0	3	1
2	0	0	3	0
0	1	0	4	1
0	0	0	1	0
0	1	0	14	0

1	0	0	0	1
2	0	0	7	0
0	1	0	7	0
0	1	0	13	0
1	5	1	3	3
2	0	2	2	3
1	0	0	6	0
0	0	0	4	6
0	0	0	0	2
0	0	1	8	0
1	0	0	3	0
0	0	0	0	0
0	1	0	2	3
2	1	0	0	0
0	0	1	5	0
0	0	0	2	2
0	1	0	4	1
0	1	1	1	1
0	0	0	8	0
0	1	0	6	0
1	1	0	4	7
0	0	0	4	0
0	0	0	3	2
0	3	1	2	1
0	0	1	3	1
0	0	0	1	0
2	1	0	3	0
0	2	0	1	1
2	0	0	0	3
1	0	0	2	1
0	1	1	1	5
0	0	0	4	0
0	0	0	0	0
0	1	1	4	0
0	0	0	2	0
1	0	2	1	1
0	0	0	2	3
0	0	0	2	0
1	1	3	3	1
0	0	0	4	0
0	1	0	8	0
1	0	2	3	1
1	0	2	1	0
0	1	0	2	0
0	0	2	1	0
0	0	0	5	1
0	0	2	1	3
0	3	0	1	2
0	1	1	0	0
0	0	0	0	1
0	0	0	0	1
2	0	0	0	1
19409	23372	19377	80392	38053
55578	49516	52354	107680	114166
442	424	441	343	423
UC	UC	UC	UC	UC
UC	UC	UC	UC	UC

10	11	12	13	14
10	11	12	13	14
UC	UC	UC	UC	UC
C_41	C_42	C_44	C_46	C_47
623719	774447	627333	2772220	1217409
UC	UC	UC	UC	UC
15.83	11.24	13.86	6.82	16.32
2.63	4.26	0.50	1.31	1.40
1.21	1.63	6.11	1.56	2.74
1.92	1.58	2.12	2.00	2.23
0.68	1.26	2.41	1.94	2.96
1.06	1.62	0.15	2.99	0.24
0.80	1.87	0.54	2.15	0.47
0.33	0.51	0.05	1.71	0.05
0.42	0.60	0.24	1.44	0.15
1.27	0.78	0.32	0.80	0.44
0.49	0.21	0.52	0.98	0.18
0.33	0.45	0.42	0.49	0.46
0.30	0.20	0.34	0.79	0.14
0.43	0.23	0.42	0.31	0.74
0.26	0.27	0.24	1.07	0.04
0.12	0.17	0.66	0.22	0.34
0.10	0.16	0.05	0.70	0.03
0.15	0.13	0.14	0.33	0.12
0.24	0.34	0.09	0.47	0.08
0.21	0.34	0.15	0.20	0.13
0.12	0.11	0.15	0.29	0.13
0.17	0.09	0.08	0.21	0.13
0.06	0.13	0.06	0.33	0.03
0.06	0.28	0.02	0.20	0.04
0.27	0.80	0.04	0.18	0.02
0.08	0.10	0.07	0.10	0.10
0.09	0.04	0.15	0.07	0.17
0.22	0.28	0.02	0.10	0.03
0.10	0.19	0.06	0.12	0.05
0.07	0.13	0.05	0.11	0.05
0.07	0.10	0.08	0.20	0.04
0.07	0.08	0.17	0.10	0.15
0.02	0.05	0.03	0.28	0.02
0.03	0.04	0.01	0.96	0.01
0.09	0.17	0.05	0.11	0.05
0.22	0.07	0.03	0.09	0.12
0.22	0.36	0.05	0.14	0.05
0.15	0.45	0.03	0.10	0.04
0.05	0.15	0.03	0.06	0.13
0.02	0.05	0.62	0.03	0.15
0.05	0.06	0.03	0.08	0.09
0.02	0.04	0.14	0.06	0.09
0.06	0.07	0.04	0.04	0.07
0.08	0.09	0.01	0.12	0.01
0.11	0.09	0.04	0.06	0.04
0.02	0.04	0.05	0.06	0.03
0.02	0.07	0.02	0.06	0.02
0.01	0.02	0.14	0.04	0.06
0.00	0.00	0.00	0.02	0.00
0.01	0.05	0.02	0.18	0.01
0.03	0.03	0.01	0.00	0.04
0.02	0.05	0.01	0.12	0.01

0.04	0.02	0.01	0.04	0.03
0.01	0.05	0.03	0.06	0.02
0.01	0.03	0.02	0.04	0.01
0.00	0.00	0.00	0.03	0.00
0.03	0.03	0.01	0.04	0.02
0.01	0.02	0.02	0.06	0.02
0.02	0.03	0.04	0.03	0.02
0.01	0.05	0.05	0.02	0.03
0.02	0.02	0.01	0.06	0.02
0.02	0.02	0.02	0.05	0.02
0.01	0.03	0.00	0.03	0.00
0.00	0.00	0.00	0.01	0.00
0.03	0.01	0.06	0.01	0.04
0.02	0.02	0.00	0.03	0.01
0.02	0.01	0.01	0.06	0.00
0.01	0.01	0.00	0.03	0.00
0.01	0.01	0.00	0.04	0.01
0.06	0.06	0.00	0.01	0.01
0.01	0.02	0.02	0.02	0.02
0.01	0.01	0.03	0.01	0.04
0.01	0.01	0.00	0.05	0.00
0.02	0.01	0.01	0.02	0.01
0.00	0.01	0.00	0.02	0.00
0.01	0.01	0.01	0.00	0.02
0.01	0.02	0.01	0.01	0.01
0.02	0.04	0.01	0.00	0.01
0.00	0.01	0.04	0.01	0.02
0.02	0.03	0.00	0.03	0.01
0.01	0.01	0.00	0.01	0.01
0.01	0.01	0.02	0.01	0.02
0.02	0.02	0.02	0.01	0.02
0.01	0.00	0.00	0.09	0.00
0.00	0.01	0.00	0.02	0.00
0.00	0.01	0.01	0.01	0.01
0.01	0.01	0.02	0.01	0.02
0.00	0.01	0.00	0.02	0.00
0.01	0.01	0.01	0.02	0.00
0.00	0.01	0.02	0.01	0.01
0.01	0.01	0.01	0.01	0.01
0.00	0.00	0.00	0.00	0.00
0.00	0.00	0.01	0.01	0.00
0.00	0.01	0.00	0.01	0.00
0.00	0.00	0.00	0.02	0.00
0.00	0.01	0.01	0.02	0.00
0.00	0.00	0.01	0.01	0.01
0.00	0.00	0.01	0.01	0.01
0.00	0.01	0.01	0.00	0.01
0.01	0.00	0.00	0.02	0.00
0.01	0.00	0.00	0.01	0.01
0.00	0.00	0.00	0.01	0.00
0.00	0.01	0.02	0.01	0.01
0.01	0.03	0.00	0.00	0.00
0.01	0.01	0.00	0.01	0.00
0.00	0.00	0.00	0.01	0.00
0.00	0.00	0.00	0.01	0.00
0.01	0.01	0.00	0.01	0.00
0.00	0.00	0.00	0.02	0.00
0.01	0.00	0.00	0.01	0.00
0.00	0.01	0.00	0.01	0.00

0.00	0.00	0.00	0.00	0.00
0.00	0.00	0.00	0.01	0.01
0.00	0.01	0.00	0.00	0.00
0.00	0.00	0.01	0.00	0.00
0.00	0.00	0.01	0.00	0.00
0.00	0.00	0.00	0.00	0.01
0.00	0.00	0.00	0.01	0.00
0.00	0.00	0.00	0.01	0.00
0.00	0.01	0.00	0.00	0.01
0.00	0.00	0.00	0.00	0.00
0.00	0.00	0.00	0.01	0.01
0.00	0.00	0.00	0.00	0.00
0.00	0.00	0.00	0.01	0.00
0.00	0.00	0.00	0.01	0.00
0.00	0.00	0.01	0.01	0.00
0.00	0.00	0.00	0.00	0.00
0.00	0.00	0.00	0.01	0.00
0.00	0.00	0.00	0.00	0.01
0.00	0.00	0.00	0.00	0.00
0.00	0.00	0.00	0.00	0.00
0.00	0.00	0.00	0.01	0.00
0.00	0.01	0.00	0.00	0.00
0.00	0.00	0.00	0.00	0.00
0.00	0.00	0.00	0.01	0.00
0.00	0.00	0.00	0.00	0.00
0.00	0.00	0.00	0.00	0.00
0.00	0.00	0.00	0.00	0.00
0.00	0.00	0.00	0.00	0.00
0.00	0.00	0.00	0.00	0.00
0.00	0.00	0.00	0.00	0.00
0.00	0.00	0.00	0.00	0.00
0.00	0.00	0.02	0.00	0.00
0.00	0.00	0.00	0.00	0.00
0.00	0.00	0.00	0.00	0.00
0.00	0.00	0.00	0.00	0.00
0.00	0.00	0.00	0.00	0.00
0.00	0.00	0.00	0.00	0.00
0.00	0.00	0.00	0.00	0.00
0.00	0.00	0.00	0.00	0.00
0.00	0.00	0.00	0.00	0.00
0.00	0.00	0.00	0.00	0.00
0.00	0.00	0.00	0.00	0.00
0.00	0.00	0.00	0.00	0.00
0.00	0.00	0.01	0.00	0.00
0.00	0.00	0.00	0.00	0.00
0.00	0.00	0.00	0.00	0.00
0.00	0.00	0.00	0.00	0.00
0.00	0.00	0.00	0.00	0.00
0.00	0.00	0.00	0.00	0.00
0.00	0.00	0.00	0.00	0.00
0.00	0.00	0.00	0.00	0.00
0.00	0.00	0.00	0.00	0.00
0.00	0.00	0.00	0.01	0.00
0.00	0.00	0.00	0.00	0.00
0.00	0.00	0.01	0.00	0.00
0.00	0.00	0.00	0.00	0.00
0.00	0.00	0.00	0.01	0.00
0.00	0.00	0.00	0.00	0.00
0.00	0.00	0.00	0.00	0.00

0.00	0.00	0.00	0.00	0.00
0.00	0.00	0.00	0.00	0.00
0.00	0.00	0.00	0.00	0.00
0.00	0.00	0.00	0.00	0.00
0.00	0.00	0.00	0.01	0.00
0.00	0.00	0.00	0.00	0.00
0.00	0.00	0.00	0.00	0.00
0.00	0.00	0.00	0.00	0.00
0.00	0.00	0.00	0.00	0.00
0.00	0.00	0.00	0.00	0.00
0.00	0.00	0.00	0.00	0.00
0.00	0.00	0.00	0.00	0.00
0.00	0.00	0.00	0.00	0.00
0.00	0.00	0.00	0.00	0.00
0.00	0.00	0.00	0.00	0.00
0.00	0.00	0.00	0.00	0.00
0.00	0.00	0.00	0.00	0.00
0.00	0.00	0.00	0.00	0.00
0.00	0.00	0.00	0.00	0.00
0.00	0.00	0.00	0.00	0.00
0.00	0.00	0.00	0.00	0.00
0.00	0.00	0.00	0.01	0.00
0.00	0.00	0.00	0.00	0.00
0.00	0.00	0.00	0.00	0.00
0.00	0.00	0.00	0.00	0.00
0.00	0.00	0.00	0.00	0.00
0.00	0.00	0.00	0.00	0.00
0.00	0.00	0.00	0.00	0.00
0.00	0.00	0.00	0.00	0.00
0.00	0.00	0.00	0.00	0.00
0.00	0.00	0.00	0.00	0.00
0.00	0.00	0.00	0.00	0.00
0.00	0.00	0.00	0.00	0.00
0.00	0.00	0.00	0.00	0.00
0.00	0.00	0.00	0.00	0.00
0.00	0.00	0.00	0.00	0.00
0.00	0.00	0.00	0.00	0.00
0.00	0.00	0.00	0.00	0.00
0.00	0.00	0.00	0.00	0.00
0.00	0.00	0.00	0.00	0.00
0.00	0.00	0.00	0.00	0.00
0.00	0.00	0.00	0.00	0.00
0.00	0.00	0.00	0.00	0.00
0.00	0.00	0.00	0.00	0.00
0.00	0.00	0.00	0.00	0.00
0.00	0.00	0.00	0.00	0.00
0.00	0.00	0.00	0.00	0.00
0.00	0.00	0.00	0.00	0.00
0.00	0.00	0.00	0.00	0.00
0.00	0.00	0.00	0.00	0.00
0.00	0.00	0.00	0.00	0.00
0.00	0.00	0.00	0.00	0.00
0.00	0.00	0.00	0.00	0.00
0.00	0.00	0.00	0.00	0.00
0.00	0.00	0.00	0.00	0.00
0.00	0.00	0.00	0.00	0.00
0.00	0.00	0.00	0.00	0.00
0.00	0.00	0.00	0.00	0.00
0.00	0.00	0.00	0.00	0.00

0.00	0.00	0.00	0.00	0.00
0.00	0.00	0.00	0.00	0.00
0.00	0.00	0.00	0.00	0.00
0.00	0.00	0.00	0.00	0.00
0.00	0.00	0.00	0.00	0.00
0.00	0.00	0.00	0.00	0.00
0.00	0.00	0.00	0.00	0.00
0.00	0.00	0.00	0.00	0.00
0.00	0.00	0.00	0.00	0.00
0.00	0.00	0.00	0.00	0.00
0.00	0.00	0.00	0.00	0.00
0.00	0.00	0.00	0.00	0.00
0.00	0.00	0.00	0.00	0.00
0.00	0.00	0.00	0.00	0.00
0.00	0.00	0.00	0.00	0.00
0.00	0.00	0.00	0.00	0.00
0.00	0.00	0.00	0.00	0.00
0.00	0.00	0.00	0.00	0.00
0.00	0.00	0.00	0.00	0.00
0.00	0.00	0.00	0.00	0.00
0.00	0.00	0.00	0.00	0.00
0.00	0.00	0.00	0.00	0.00
0.00	0.00	0.00	0.00	0.00
0.00	0.00	0.00	0.00	0.00
0.00	0.00	0.00	0.00	0.00
0.00	0.00	0.00	0.00	0.00
0.00	0.00	0.00	0.00	0.00
0.00	0.00	0.00	0.00	0.00
0.00	0.00	0.00	0.00	0.00
0.00	0.00	0.00	0.00	0.00

10	11	12	13	14
11	12	13	14	15
UC	UC	UC	UC	UC
C_41	C_42	C_44	C_46	C_47
623719	774447	627333	2772220	1217409
C_41	C_42	C_44	C_46	C_47
0.95	-0.09	0.50	-1.08	1.05
-0.19	0.29	-0.81	-0.57	-0.55
-0.67	-0.36	3.01	-0.41	0.47
-0.53	-0.86	-0.32	-0.44	-0.21
-1.64	-1.00	0.27	-0.24	0.88
-0.12	0.38	-0.95	1.63	-0.87
-0.58	1.13	-0.99	1.56	-1.11
-0.03	0.30	-0.54	2.47	-0.53

-0.35	0.02	-0.70	1.69	-0.88
1.31	0.04	-1.13	0.10	-0.82
0.07	-0.98	0.17	1.92	-1.09
-1.48	-0.41	-0.62	0.02	-0.26
-0.32	-0.92	-0.13	2.52	-1.23
-0.23	-1.34	-0.25	-0.88	1.60
0.20	0.23	0.08	3.89	-0.85
-0.77	-0.54	1.58	-0.34	0.20
-0.38	0.01	-0.69	3.45	-0.82
-0.64	-0.83	-0.68	1.13	-0.92
0.51	1.40	-0.81	2.54	-0.92
-0.17	1.11	-0.82	-0.26	-1.04
-0.60	-0.84	-0.14	2.12	-0.42
0.26	-0.93	-1.03	0.95	-0.38
-0.72	0.10	-0.69	2.66	-1.14
-0.59	1.52	-0.97	0.75	-0.78
0.81	3.99	-0.56	0.27	-0.63
-0.61	-0.21	-0.89	-0.37	-0.34
-0.77	-1.49	0.13	-1.08	0.48
1.05	1.63	-0.90	-0.11	-0.82
-0.13	1.55	-1.02	0.20	-1.05
-0.75	0.67	-1.22	0.15	-1.16
-0.57	0.31	-0.23	2.45	-1.11
-1.10	-0.72	0.89	-0.36	0.57
-0.58	-0.16	-0.51	3.39	-0.59
-0.26	-0.18	-0.32	4.83	-0.34
-0.07	1.75	-0.98	0.35	-0.98
0.44	-0.43	-0.72	-0.30	-0.14
1.26	2.90	-0.74	0.28	-0.71
0.64	3.69	-0.67	0.05	-0.58
-0.62	0.66	-0.84	-0.52	0.37
-0.65	-0.42	4.21	-0.53	0.39
-0.46	-0.38	-0.84	-0.01	0.25
-1.31	-0.92	2.09	-0.13	0.77
-0.04	0.18	-1.14	-0.87	0.19
1.07	1.19	-0.72	1.93	-0.73
1.01	0.50	-0.39	-0.10	-0.53
-1.64	-0.47	0.20	0.88	-1.51
-0.82	1.17	-1.04	0.87	-0.98
-1.23	-1.04	2.97	-0.45	0.26
-0.28	-0.30	-0.27	0.28	-0.31
-0.42	0.48	-0.32	3.41	-0.50
-0.33	-0.49	-1.56	-1.76	0.41
-0.39	0.41	-0.66	2.51	-0.67
0.17	-1.24	-2.15	0.09	-0.53
-0.81	1.28	0.01	1.70	-0.45
-0.82	0.16	-0.39	0.73	-0.89

-0.23	-0.21	-0.33	0.81	-0.34
-0.20	0.29	-1.51	0.92	-1.00
-1.47	0.04	0.05	2.83	-0.52
-0.63	-0.39	0.37	0.30	-0.73
-0.75	0.22	0.34	-0.47	-0.28
-0.55	-0.30	-0.94	3.07	-0.68
-0.45	0.18	0.09	2.44	-0.22
0.18	1.28	-0.60	1.97	-0.59
-0.22	-0.16	-0.28	0.10	-0.25
0.03	-0.98	1.69	-1.19	0.59
0.48	0.08	-1.29	1.55	-0.96
0.46	-0.11	-0.47	3.84	-0.66
-0.23	-0.26	-0.83	1.81	-0.77
-0.09	-0.03	-0.96	3.38	-0.73
0.41	0.32	-0.40	-0.36	-0.29
-0.60	-0.34	-0.40	-0.14	-0.45
-0.69	-0.98	0.73	-1.18	1.84
-0.22	0.18	-0.56	3.58	-0.77
1.18	0.10	-0.87	0.81	-0.96
-0.90	0.15	-0.87	0.89	-0.94
-0.70	-0.74	-0.74	-1.19	0.34
-0.03	0.84	-0.82	-0.43	-0.66
-0.10	0.49	-0.41	-0.43	-0.19
-1.21	-0.98	1.97	-0.38	0.36
0.17	1.04	-1.08	1.46	-0.46
-0.28	0.74	-1.29	0.27	-0.96
-0.93	-0.37	0.50	0.10	1.12
-0.11	0.42	-0.11	-0.93	0.52
-0.18	-0.46	-0.44	3.85	-0.44
-0.49	0.90	-1.03	2.19	-1.02
-1.05	-0.63	-0.57	0.84	0.29
-1.15	-0.32	0.75	-1.36	0.66
-0.30	0.61	-0.72	2.36	-0.75
0.05	0.73	-0.47	2.44	-1.60
-1.03	0.12	0.88	-0.64	0.17
-0.45	-0.50	0.70	-0.40	0.25
-0.19	-0.29	-0.31	0.13	-0.24
-1.20	-0.86	1.37	0.40	-0.46
-0.65	-0.11	-0.76	0.74	-0.89
-0.13	-0.04	-0.50	3.99	-0.51
-0.73	0.54	-0.10	3.20	-0.81
-1.16	-0.71	0.50	-0.51	0.18
-1.09	-1.32	-0.10	-0.40	0.69
-1.14	0.08	-0.45	-1.03	0.04
0.65	0.26	-0.68	3.65	-0.62
-0.14	-0.59	-0.95	-0.08	0.11
-1.20	-0.03	-1.43	1.17	-0.90
-0.68	-0.54	2.99	-0.39	0.06

-0.03	1.44	-0.46	-0.33	-0.52
0.90	0.17	-1.22	2.42	-0.90
-0.50	-0.19	-0.64	2.94	-0.77
-0.78	-0.54	-0.42	0.65	-0.31
-0.21	0.14	-0.76	0.10	-0.66
0.15	-0.49	-0.95	3.79	-0.92
0.96	-0.13	-1.13	1.87	-0.87
-0.84	0.90	-0.28	1.90	-0.64
-1.36	-0.64	-0.03	-0.82	-0.03
-0.92	-0.71	-0.88	1.49	0.50
0.25	0.86	-1.20	0.32	-0.91
-0.84	0.28	0.64	-0.20	-0.23
-1.00	-1.11	1.17	-0.27	-0.49
-1.41	0.21	-0.32	1.06	1.66
-0.85	0.00	-0.78	2.25	-1.02
-0.85	0.00	-0.78	2.25	-1.02
0.04	0.21	-0.78	0.02	0.37
0.13	-0.69	-0.69	0.22	-0.23
0.27	-0.59	0.78	1.88	1.28
-0.68	-0.08	-0.85	-0.71	-0.50
-0.82	0.11	-0.40	2.24	-0.41
-0.34	-0.33	-0.32	1.68	-0.37
-0.46	-1.02	0.63	0.71	-0.38
0.19	0.94	-0.80	-0.77	-0.77
-1.20	-1.17	-0.11	1.27	0.43
-0.47	0.12	-1.03	-0.56	0.98
-0.71	-0.63	-0.01	-0.44	0.18
0.04	-0.76	-0.65	-0.56	-0.22
-1.32	-0.95	0.86	2.27	-0.78
-0.80	2.66	-1.06	0.93	-0.60
0.30	-0.62	-0.95	0.59	-0.28
-0.62	-0.47	-0.62	3.76	-0.65
0.06	0.61	-0.61	1.09	-0.26

7	8	9	10	11
8	9	10	11	12
TTCAAGTAATCCAGGATAGGCT	AAACCGTTACCATTACTGAGTT	AAGCTGCCAGTT	CATTGCACTTGT	TGAGGTAGTAGT
miR-26a-5p	miR-451a	miR-22-3p	miR-25-3p	let-7i-5p
5953261	3762724	3462453	3413660	2186845
-1.13	-0.50	-0.38	1.16	1.45
-0.25	-0.26	-0.94	0.27	-0.89
0.00	0.10	1.75	2.58	0.90
-0.93	-0.40	0.03	1.36	1.29
2.65	-0.49	-1.00	-0.92	-0.75
0.47	-0.60	-0.66	-0.22	2.33
-0.62	-0.58	-0.75	-0.99	1.19
-0.99	-0.56	-0.89	-1.07	-0.93
-0.79	-0.23	-0.03	0.96	-0.55
-0.58	-0.03	-0.35	1.31	0.07
1.13	0.30	0.02	0.04	-0.98
-0.99	-0.54	-0.70	-1.13	0.17
1.56	2.47	1.69	0.10	1.92
-1.11	-0.53	-0.88	-0.82	-1.09
-0.63	-0.08	0.78	0.75	-0.14
0.67	0.07	2.19	-0.18	0.07
0.13	-0.52	-0.47	-0.68	-0.50
-0.49	0.68	1.23	0.77	-0.25
0.28	0.02	-0.17	0.59	-0.64
1.35	3.77	1.54	-0.34	-0.75

-0.72	-0.51	-0.32	0.24	1.39
-0.32	-0.56	-0.77	-1.04	-0.65
-0.47	-0.47	-0.60	-1.05	-0.30
-1.09	-0.54	-0.70	-1.29	-0.51
0.34	-0.52	-1.02	-1.05	-1.42
1.32	-0.49	-0.24	-0.41	-0.36
1.21	0.97	1.65	1.07	-0.07
0.90	5.14	2.15	1.12	0.27
0.95	-0.37	-0.08	1.07	0.10
-0.88	-0.14	-0.41	1.73	0.12
-1.42	-0.53	-0.94	1.03	0.76
-0.20	0.53	-0.57	3.37	0.97
3.89	0.31	-0.83	0.17	-0.85
-0.71	-0.47	-0.67	-0.15	-0.58
-1.02	0.14	-0.71	0.94	-0.29
-0.19	-0.52	-0.72	-0.23	-0.44
-1.64	-0.60	-1.04	-1.41	-0.94
0.45	0.00	-0.37	2.16	3.02
0.57	10.00	0.49	3.03	2.96
0.75	-0.41	-0.49	-1.00	-1.02
-0.21	-0.41	-0.78	-1.21	2.41
0.12	0.15	-0.25	-1.40	11.30
-0.25	-0.55	-0.55	-0.82	-0.23
0.63	0.38	0.53	1.96	1.88
-0.83	-0.51	-0.49	-0.90	-1.29
-0.51	-0.51	-0.78	-1.61	0.47
3.42	-0.04	0.93	-0.45	-0.81
0.48	-0.46	-0.12	-0.85	-0.60
0.12	0.28	1.04	-0.73	-0.72
0.64	-0.29	0.06	-0.88	-0.87
0.60	0.11	1.55	-0.61	-0.49
-0.04	-0.55	-0.64	-0.86	0.00
-0.56	-0.53	-0.61	-0.55	0.22
1.23	-0.42	0.51	-0.73	0.48
-0.46	-0.57	-0.48	-0.69	-0.06
-0.32	-0.55	-0.56	-0.73	-0.74
-0.11	-0.50	0.43	-0.26	0.94
1.57	6.18	2.70	0.10	0.26
0.56	-0.52	-0.20	-0.27	-0.56
2.77	-0.19	0.08	-0.41	0.22
3.06	-0.08	-0.48	-0.88	-0.76
1.56	4.07	2.45	0.80	0.09
-0.32	-0.45	-0.65	-0.70	-0.92
1.20	-0.41	-0.10	-0.60	0.66
1.23	0.00	2.74	0.61	0.58
-1.35	-0.09	-0.41	2.79	6.00
-0.74	1.39	3.23	7.24	2.28
-0.60	-0.36	-0.96	3.69	1.32
-0.40	-0.28	-0.62	1.97	2.35
1.01	-0.15	0.07	1.63	1.34
-0.65	-0.45	-0.29	0.99	1.24
0.38	-0.15	-0.89	0.07	2.54
-0.20	-0.20	0.38	1.55	1.56
0.25	1.13	0.67	-0.11	0.06
-1.54	-0.37	-0.92	-1.57	1.47
-0.20	-0.19	1.58	-0.42	1.23
0.27	-0.01	-0.43	0.93	-0.49
-0.27	-0.51	-0.31	0.96	-0.28
0.87	1.56	1.09	0.72	0.44

2.86	0.25	1.73	-0.20	-0.71
0.71	-0.39	-0.20	-0.61	-1.18
1.99	-0.17	0.43	0.00	-0.03
-0.11	-0.47	-0.39	-1.62	-0.97
1.73	7.12	1.80	-0.35	-0.08
0.68	8.65	1.25	0.14	-0.12
1.24	0.18	0.20	-0.72	-1.08
2.17	-0.35	0.53	-0.75	-0.44
1.26	-0.45	-0.02	-0.30	0.13
0.38	0.49	0.74	0.50	-0.15
0.78	-0.37	0.33	-0.47	-0.73
0.17	0.60	0.04	1.16	1.26
-0.80	-0.56	-0.71	0.14	0.58
-0.43	-0.55	-0.44	-0.69	-0.27
1.11	-0.31	0.40	0.58	-0.07
1.04	-0.14	0.47	3.33	2.97
-1	0	-1	0	-1
0.00	0.00	0.00	0.00	0.00
1.00	1.00	1.00	1.00	1.00
0.77	0.88	0.38	0.11	0.67

7	8	9	10	11
TTCAAGTAATCCAGGATAGGCT	AAACCGTTACCATTACTGAGTT	AAGCTGCCAGTT	CATTGCACTTGT	TGAGGTAGTAGT
miR-26a-5p	miR-451a	miR-22-3p	miR-25-3p	let-7i-5p
5953261	3762724	3462453	3413660	2186845
-1.13	-0.50	-0.38	1.16	1.45
-0.25	-0.26	-0.94	0.27	-0.89
0.00	0.10	1.75	2.58	0.90
-0.93	-0.40	0.03	1.36	1.29
2.65	-0.49	-1.00	-0.92	-0.75
0.47	-0.60	-0.66	-0.22	2.33
-0.62	-0.58	-0.75	-0.99	1.19
-0.99	-0.56	-0.89	-1.07	-0.93
-0.79	-0.23	-0.03	0.96	-0.55
-0.58	-0.03	-0.35	1.31	0.07
1.13	0.30	0.02	0.04	-0.98
-0.99	-0.54	-0.70	-1.13	0.17
1.56	2.47	1.69	0.10	1.92
-1.11	-0.53	-0.88	-0.82	-1.09
-0.63	-0.08	0.78	0.75	-0.14
0.67	0.07	2.19	-0.18	0.07
0.13	-0.52	-0.47	-0.68	-0.50
-0.49	0.68	1.23	0.77	-0.25
0.28	0.02	-0.17	0.59	-0.64
1.35	3.77	1.54	-0.34	-0.75
-0.72	-0.51	-0.32	0.24	1.39
-0.32	-0.56	-0.77	-1.04	-0.65
-0.47	-0.47	-0.60	-1.05	-0.30
-1.09	-0.54	-0.70	-1.29	-0.51
0.34	-0.52	-1.02	-1.05	-1.42
1.32	-0.49	-0.24	-0.41	-0.36
1.21	0.97	1.65	1.07	-0.07
0.90	5.14	2.15	1.12	0.27

0.95	-0.37	-0.08	1.07	0.10
-0.88	-0.14	-0.41	1.73	0.12
-1.42	-0.53	-0.94	1.03	0.76
-0.20	0.53	-0.57	3.37	0.97
3.89	0.31	-0.83	0.17	-0.85
-0.71	-0.47	-0.67	-0.15	-0.58
-1.02	0.14	-0.71	0.94	-0.29
-0.19	-0.52	-0.72	-0.23	-0.44
[illegible]	[illegible]	[illegible]	[illegible]	[illegible]

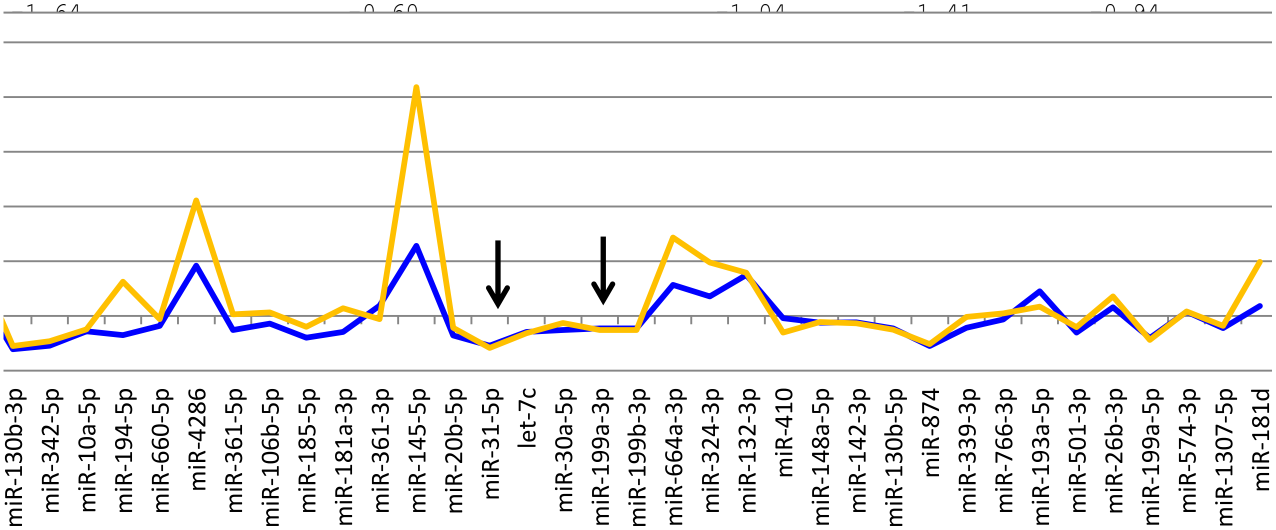

[illegible]	[illegible]	[illegible]	[illegible]	[illegible]
-0.32	-0.55	-0.56	-0.73	-0.74
-0.11	-0.50	0.43	-0.26	0.94
1.57	6.18	2.70	0.10	0.26
0.56	-0.52	-0.20	-0.27	-0.56
2.77	-0.19	0.08	-0.41	0.22
3.06	-0.08	-0.48	-0.88	-0.76
1.56	4.07	2.45	0.80	0.09
-0.32	-0.45	-0.65	-0.70	-0.92
1.20	-0.41	-0.10	-0.60	0.66
1.23	0.00	2.74	0.61	0.58
-1.35	-0.09	-0.41	2.79	6.00
-0.74	1.39	3.23	7.24	2.28
-0.60	-0.36	-0.96	3.69	1.32
-0.40	-0.28	-0.62	1.97	2.35
1.01	-0.15	0.07	1.63	1.34
-0.65	-0.45	-0.29	0.99	1.24
0.38	-0.15	-0.89	0.07	2.54
-0.20	-0.20	0.38	1.55	1.56
0.25	1.13	0.67	-0.11	0.06
-1.54	-0.37	-0.92	-1.57	1.47
-0.20	-0.19	1.58	-0.42	1.23
0.27	-0.01	-0.43	0.93	-0.49
-0.27	-0.51	-0.31	0.96	-0.28
0.87	1.56	1.09	0.72	0.44
2.86	0.25	1.73	-0.20	-0.71
0.71	-0.39	-0.20	-0.61	-1.18
1.99	-0.17	0.43	0.00	-0.03
-0.11	-0.47	-0.39	-1.62	-0.97
1.73	7.12	1.80	-0.35	-0.08
0.68	8.65	1.25	0.14	-0.12
1.24	0.18	0.20	-0.72	-1.08
2.17	-0.35	0.53	-0.75	-0.44

1.26	-0.45	-0.02	-0.30	0.13
0.38	0.49	0.74	0.50	-0.15
0.78	-0.37	0.33	-0.47	-0.73
0.17	0.60	0.04	1.16	1.26
-0.80	-0.56	-0.71	0.14	0.58
-0.43	-0.55	-0.44	-0.69	-0.27
1.11	-0.31	0.40	0.58	-0.07
1	0	0	3	3

7	8	9	10	11
74	124	49	21	60
CATGCCTTGAGTGTAGGACCGT	AAAGTTCTGAGACACTCCGACT	GGATATCATCATA	ACTGGACTTGGAG	TCCCTGAGACCCT
miR-532-5p	miR-148a-5p	miR-144-5p	miR-378a-3p	miR-125a-5p
68018	13673	178962	738230	115160
0.47	-0.12	-0.29	0.38	-0.59
-0.58	-0.50	-0.29	-1.59	2.00
0.97	0.06	0.02	-0.62	-0.45
0.55	-0.29	0.23	-0.58	-0.58
-1.82	-0.34	-0.33	0.66	2.75
-0.91	-0.69	-0.16	-0.55	-0.48
-0.54	-0.70	-0.21	0.66	-0.33
-1.00	-0.69	-0.29	-0.36	-0.47
0.74	-0.55	-0.27	-0.95	-0.55
1.18	-0.82	-0.28	-0.60	-0.75
0.10	0.11	-0.30	-0.84	0.22
-0.87	-0.40	-0.27	-0.14	0.34
0.81	2.24	0.28	2.12	-0.47
-0.96	-0.41	-0.31	-0.42	-0.28
1.51	0.05	-0.29	1.33	-0.34
0.48	0.89	-0.11	-0.23	-0.55
-0.06	-0.01	-0.34	1.83	-0.09
2.70	-0.97	-0.11	0.97	-0.66
0.35	3.48	-0.36	0.82	-0.70
0.67	1.35	4.92	0.33	-0.73
-0.08	-0.66	-0.13	-0.68	-0.59
-0.21	0.65	-0.34	1.08	0.63
-0.38	0.22	-0.24	1.15	0.11
-0.75	-0.49	-0.28	-0.14	-0.42
-1.54	-0.73	-0.29	-1.54	2.90
-1.00	-0.65	-0.26	-0.70	0.52
0.18	-0.04	0.30	-1.39	-0.43
0.35	0.44	7.55	-1.44	-0.82
0.59	1.50	-0.25	-0.55	-0.10
1.88	0.91	-0.33	-0.50	-0.31
-0.44	-0.69	-0.34	-0.84	0.66
0.10	2.19	-0.35	-0.24	-0.23
-1.48	-0.47	-0.11	-0.17	10.28
-0.17	-0.25	-0.33	0.23	-0.91
-0.19	-0.22	-0.30	0.20	-0.87
0.09	-0.20	-0.31	1.05	-0.64
-1.29	-1.10	-0.31	-1.85	-0.76

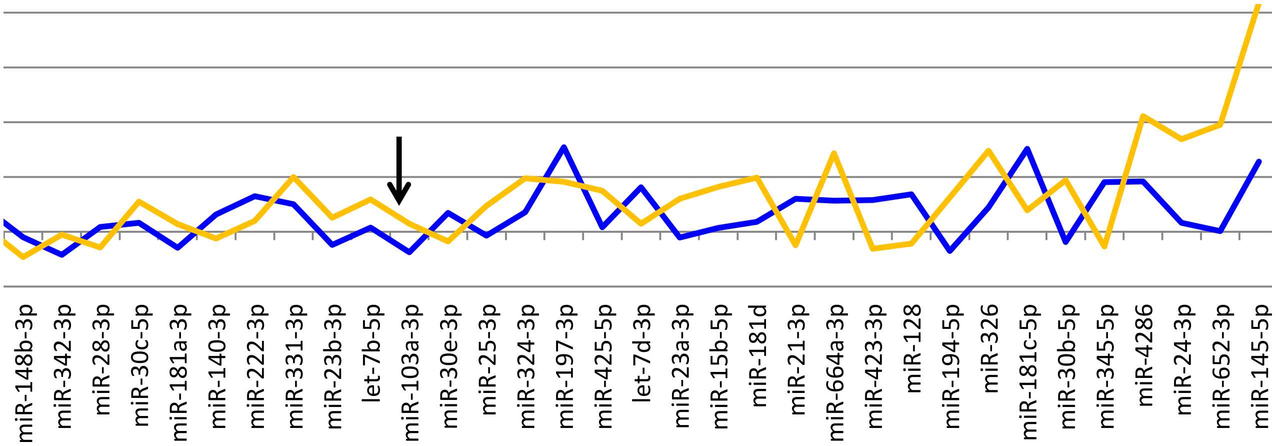

[illegible]	[illegible]	[illegible]	[illegible]	[illegible]
-0.19	-0.21	-0.33	0.59	0.94
0.17	-0.16	-0.15	-0.66	0.19
0.44	-0.56	7.33	-0.92	-0.78
-0.66	-0.42	-0.30	0.18	0.28
-0.97	0.13	-0.01	0.00	0.93
-1.25	-0.29	-0.11	-0.57	1.15
0.92	-0.18	5.92	-1.16	-0.85
-0.16	-0.58	-0.09	-0.95	0.08
-0.31	-0.77	0.03	-0.94	-0.08
0.62	-0.51	0.15	-1.03	-0.75
1.70	1.76	0.24	-0.22	-0.53
1.27	-0.11	-0.21	-0.75	-0.90
-0.54	1.52	-0.36	0.40	1.04
-0.18	-0.71	1.03	-0.30	-0.07
-0.29	0.51	0.48	0.84	0.14
0.23	-0.15	0.20	-0.90	-0.21
1.44	0.88	0.19	-0.39	-0.82
0.50	-0.54	0.18	0.11	-0.73
0.11	-0.02	0.01	0.69	-0.49
-0.97	-0.54	-0.37	5.89	-0.23
0.17	0.07	0.10	0.10	-0.45
-0.19	2.29	-0.37	1.10	-0.60
-1.09	0.71	-0.39	-0.49	-0.48
1.84	0.19	0.40	1.73	-0.82
0.00	0.65	-0.19	0.81	-0.33
-0.93	-0.26	-0.24	-0.94	0.26
-0.38	-0.08	0.16	0.44	-0.18
-1.02	0.22	-0.21	-0.47	0.13
0.71	1.72	4.72	1.20	-0.69
0.35	0.06	12.72	0.08	-0.97
-0.44	0.29	-0.30	-0.09	0.46
-0.24	0.26	-0.07	0.35	-0.61
-0.52	-0.17	0.05	-0.73	-0.35
2.45	2.06	-0.27	2.00	-0.91
0.50	-0.16	-0.27	0.09	0.16
1.02	2.43	0.71	0.77	-0.63
0.20	-0.29	-0.29	0.46	-0.53
-0.39	-0.09	-0.19	1.47	-0.11
-0.23	0.15	-0.11	-1.15	-0.24
0.38	-0.39	-0.16	-0.87	-0.77
0.03	0.03	0.03	0.04	0.04
0.07	0.06	0.05	0.08	0.02

1217409	19623	4032681	1739788	1227867	121631	2029037
	1					
	1					
15	16	17	18	19	20	21
15	16	17	18	19	20	21
C_47	D_15	D_16	D_17	D_18	D_36	D_4
UC	UC	UC	UC	UC	UC	UC
7784665	68312	11547045	3054238	4012517	291206	4268943
0.29	0.77	0.93	0.77	0.96	0.81	0.31
C_47	D_15	D_16	D_17	D_18	D_36	D_4
6567256	48689	7514364	1314450	2784650	169575	2239906
620936	3363	1531849	492299	434340	43427	587465
53388	438	439203	147428	121333	5509	392427
104165	992	146839	114004	91145	9645	124341
85023	242	257462	91626	107193	4797	72204
112601	191	216353	94252	142992	8299	141988
8973	850	236351	141379	15073	9190	79097
17934	630	96321	83961	47442	3216	84166
1841	379	37646	20264	2086	2689	22024
5596	64	123431	89451	13489	4537	31482
16782	477	131928	36434	18771	3976	62023
6932	89	54535	25861	12887	1522	18899
17594	264	56370	31108	22005	2555	23168
5475	153	38107	22508	7706	971	12274
28330	118	51226	11534	21866	1929	12074
1335	172	31921	26946	1105	878	11836
13042	41	25011	19800	10553	1128	4026
1022	41	20229	15122	1285	446	15501
4615	54	46387	16987	6462	1895	18873
2969	105	28660	20805	5612	488	16615
4816	2401	26135	12362	8659	600	32768
5000	88	30117	7562	10288	817	13028
4814	31	29467	7813	6300	967	7672
1009	290	12100	8758	2850	234	19089
1373	59	9499	7671	2046	566	28264
870	36	7633	5119	1917	178	10212
3686	333	20525	7101	7076	537	10933
6404	34	20654	5910	7129	645	4277
1026	3579	18939	7643	1260	364	10771
2036	110	12409	8787	2516	387	4527
1998	77	9324	6564	5175	240	6960
1630	50	12062	8726	3465	314	10061
5761	9	11175	4139	5507	350	3526
788	53	4610	6155	1070	183	3160
433	37	2976	6071	563	195	2703
1927	99	11404	8133	2321	358	4165
4579	118	15472	4644	5836	512	6346
2027	378	9964	6651	1732	412	11395
1363	76	5458	5447	1530	86	5968
4960	66	16089	2287	4079	147	2913
5636	23	3617	1490	1957	326	4804
3358	16	8931	2670	3233	198	3231
3586	7	5684	4329	4867	184	5708
2625	31	7925	2349	3039	188	2382
300	83	3066	2256	253	170	2355
1427	25	3568	2368	2760	171	3494

976	14	4992	3193	1653	129	3229
625	37	4186	6725	1846	117	2667
2186	11	6822	1728	1775	172	2486
93	1	383	435	64	31	80
312	362	2173	2128	395	126	4109
1635	18	3862	1835	1788	180	1747
438	47	4225	2672	1062	140	2942
1098	42	5394	1923	1901	222	2882
780	1079	4437	2415	575	78	5573
341	9	3705	2029	2316	76	1983
37	37	451	461	60	39	227
598	55	5246	2251	1394	113	1423
676	1	2867	2062	913	111	1974
791	5	3497	1840	978	111	2194
1146	15	3517	1056	1417	59	898
612	3	2243	1912	843	93	836
737	19	2703	1636	1016	88	3769
68	11	723	1288	51	23	386
42	0	601	274	74	6	84
1509	5	2844	1145	590	114	457
251	10	3331	1525	451	120	840
117	5	798	1235	314	42	704
99	10	1680	876	208	37	380
241	3	1830	858	519	57	1216
398	102	2905	960	452	16	2410
580	16	2014	1137	1058	56	805
1487	8	2019	587	646	87	454
70	0	1377	1240	58	38	564
250	107	3183	926	510	129	1034
133	5	1190	1295	434	52	959
809	5	1462	931	519	117	502
345	58	3552	1119	1401	66	1446
535	158	1422	632	537	32	587
817	1	1746	770	606	124	499
376	11	1062	2057	577	98	547
233	12	1547	766	487	57	349
766	1	1060	836	617	79	582
845	14	1501	543	433	21	517
58	4	627	710	365	13	243
71	18	888	703	387	25	683
397	1	1200	563	776	63	412
628	6	964	846	539	42	652
59	7	627	802	33	60	416
88	6	925	615	124	42	430
502	1	1082	436	289	45	300
338	143	676	397	582	43	694
16	0	258	79	13	1	4
187	0	859	406	528	23	268
51	2	1244	906	146	81	241
37	2	460	464	57	23	183
73	1	545	810	139	24	370
330	6	912	426	324	13	457
332	10	833	359	292	45	494
286	4	474	217	435	15	467
32	3	891	492	32	14	194
247	1	1137	179	324	50	811
105	3	522	481	278	33	502
299	1	556	387	344	20	218
79	4	418	760	149	22	399

72	9	528	485	91	25	253
27	1	508	312	59	41	292
167	1	536	324	213	43	357
82	14	599	331	221	11	509
33	1	412	327	106	26	127
47	2	829	245	88	23	188
92	4	723	411	142	40	585
177	0	653	253	248	15	345
214	3	495	167	252	18	656
39	2	771	252	106	21	163
127	0	252	200	234	19	109
141	1	421	205	216	12	116
220	17	505	191	215	14	293
30	4	212	520	39	12	102
30	4	212	520	39	12	102
217	1	548	228	185	10	410
111	1	399	198	207	39	125
208	0	327	218	156	15	234
49	0	534	230	129	33	190
68	3	352	248	102	2	657
2	1	56	116	10	1	21
113	0	231	244	116	9	117
48	1	242	149	247	13	130
144	0	372	201	162	29	180
232	0	346	155	158	9	213
167	0	343	86	134	8	78
49	29	192	80	102	1	89
46	0	299	198	97	16	197
50	17	277	315	100	3	106
80	0	283	102	174	20	123
14	0	117	163	41	6	63
76	1	342	225	86	8	187
45	2	161	108	69	1	63
58	0	292	117	118	18	63
24	0	211	160	66	4	71
80	0	189	132	125	18	65
109	8	216	100	135	4	94
50	1	353	120	97	11	81
163	0	135	88	105	38	124
21	0	165	137	76	1	55
6	1	41	76	5	3	22
177	4	347	63	73	6	61
68	1	196	86	67	0	106
16	0	192	116	62	2	90
28	1	136	81	49	2	58
55	0	292	70	96	15	110
14	1	308	209	19	9	266
17	0	208	93	25	10	47
21	0	166	149	30	23	51
64	0	104	56	78	2	100
43	24	34	47	16	20	24
69	0	228	86	61	7	42
44	0	224	71	89	3	101
70	0	103	111	89	6	82
43	0	137	76	101	9	64
88	0	222	82	87	8	78
1	0	23	43	6	1	18
46	0	149	64	90	7	109
107	2	168	51	53	8	80

3	0	129	107	16	6	71
7	0	131	180	17	1	114
83	1	155	94	51	7	36
45	0	146	55	59	5	129
121	0	54	66	20	3	152
4	0	69	72	77	3	47
97	0	176	77	68	11	29
0	0	110	2	108	1	94
8	0	117	87	43	2	36
61	1	167	88	62	5	106
29	1	312	102	25	4	198
9	1	134	56	14	3	80
46	0	219	38	58	3	57
21	0	195	87	39	6	14
10	3	90	82	71	8	121
54	0	145	38	70	11	49
67	0	100	56	76	9	23
97	0	121	40	46	4	36
5	0	106	82	8	2	50
40	0	139	60	35	3	19
0	0	110	103	18	3	53
40	18	117	66	37	2	50
72	0	116	49	28	10	34
5	0	101	81	6	2	48
42	0	107	31	18	5	22
24	0	82	171	24	2	54
8	6	97	114	42	0	44
2	0	37	48	65	0	1
3	1	92	90	7	9	63
19	0	136	89	30	6	99
42	0	118	60	23	5	54
32	28	133	55	48	4	69
15	2	63	61	35	1	39
1	10	61	37	8	1	37
34	0	139	40	55	8	23
17	1	109	52	44	10	38
26	0	142	70	26	0	11
57	0	97	38	35	3	72
18	3	92	75	25	6	70
80	0	90	28	60	4	59
22	47	147	54	38	0	50
3	0	75	47	10	1	24
34	0	62	34	49	4	12
59	0	101	29	23	5	68
40	2	132	51	37	2	72
23	0	69	14	58	3	57
56	0	112	21	31	4	17
48	0	100	33	25	7	38
17	0	121	77	34	4	135
5	3	40	48	3	13	37
66	0	123	38	54	7	23
6	0	78	47	21	2	41
23	0	89	50	37	0	82
11	0	68	62	34	0	41
12	0	100	44	21	10	36
1	0	29	13	0	0	2
16	0	59	22	30	3	31
7	4	53	13	16	0	62
28	0	110	41	27	4	29

9	0	62	28	30	0	32
19	0	127	35	38	1	18
7	0	54	41	15	5	13
47	0	43	21	28	1	23
14	0	53	23	31	3	19
27	1	49	35	36	5	33
42	0	51	25	23	3	28
9	0	84	24	43	0	25
6	1	48	46	8	3	54
27	0	87	22	34	2	39
1	1	14	19	0	1	8
32	0	41	34	34	0	11
2	1	54	50	13	2	9
20	0	71	4	29	4	19
19	0	30	20	31	1	12
2	1	21	28	4	0	32
2	0	53	39	7	1	35
6	0	57	29	20	1	15
4	0	52	26	8	5	5
1	0	41	32	14	2	15
0	1	42	23	4	1	47
4	1	34	11	11	0	37
4	1	34	11	11	0	37
27	0	42	15	10	1	6
12	0	55	14	20	1	23
7	2	51	12	14	1	17
10	0	45	31	8	3	0
11	0	29	16	12	0	19
11	0	44	28	26	0	51
3	0	51	29	4	1	13
11	0	54	14	10	3	6
10	0	32	17	27	0	8
6	0	58	14	2	2	60
6	0	95	7	59	0	16
9	0	21	33	13	2	5
6	0	69	13	33	0	19
0	0	5	2	0	0	0
12	0	45	21	15	0	20
4	0	31	40	1	0	18
18	0	30	22	16	0	14
30	0	60	15	22	0	8
4	0	30	40	1	0	17
15	0	50	29	13	4	10
16	0	41	9	32	3	6
7	0	38	21	14	3	13
12	0	36	14	13	1	5
0	1	8	1	1	0	2
17	0	19	9	12	7	11
34	0	57	10	14	3	15
26	0	33	6	20	0	12
10	0	23	15	11	3	8
2	0	36	14	15	1	42
0	0	8	8	0	0	3
17	0	33	10	9	0	14
2	0	22	29	2	1	22
95	0	1	0	0	7	0
15	0	29	12	16	0	9
9	0	24	16	13	2	10
12	0	40	9	12	0	26

27	0	39	10	12	5	6
10	0	16	23	26	4	8
9	0	19	18	9	3	9
10	0	23	12	15	2	19
13	0	18	23	10	4	0
14	0	38	11	22	2	26
2	0	21	27	2	1	21
13	0	7	12	5	2	5
1	1	12	26	1	0	5
3	1	7	13	0	1	16
0	1	17	27	12	0	32
5	0	35	8	20	1	19
0	0	11	16	9	2	10
22	0	6	7	2	4	6
7	0	20	17	9	3	10
3	0	28	21	1	3	11
13	0	48	19	8	0	13
8	0	22	17	15	0	14
4	0	17	17	16	0	13
2	0	20	7	5	3	5
10	0	20	12	8	2	5
19	0	16	10	11	0	7
10	0	30	7	8	0	11
21	0	20	23	21	3	6
3	1	23	22	3	2	36
15	0	9	3	11	3	25
23	0	10	21	19	0	8
17	0	7	20	7	1	6
11	0	14	11	9	3	19
5	1	18	27	6	0	11
7	0	24	3	2	1	18
0	1	11	10	0	1	13
3	0	5	28	1	1	20
6	0	21	8	9	0	3
2	0	33	9	3	1	6
10	0	34	6	6	0	8
1	1	36	6	18	0	13
6	0	31	9	17	0	13
5	0	18	12	21	0	4
6	0	23	15	9	2	1
1	0	12	22	4	1	8
4	0	25	12	8	0	19
3	0	17	5	6	1	1
8	0	29	12	14	0	4
4	0	22	13	4	1	10
4	0	16	22	8	3	2
10	0	15	12	8	0	3
2	0	25	2	6	0	4
15	0	17	5	11	0	2
3	0	15	7	5	0	17
14	0	22	5	4	1	5
0	0	11	9	1	0	5
2	0	13	7	1	0	4
2	0	12	12	0	0	12
2	0	33	7	14	1	5
6	0	17	4	7	0	5
9	0	13	12	9	2	9
0	4	1	1	1	0	0
9	1	30	6	6	0	8

4	9	13	14	5	1	15
16	0	13	11	10	0	5
1	0	19	6	8	1	8
5	0	15	11	10	2	9
5	0	7	11	6	0	19
2	0	26	3	6	2	6
15	0	24	5	7	0	12
0	0	5	10	1	2	7
4	0	26	6	4	0	1
7	0	7	11	7	1	5
3	0	13	11	5	2	5
0	0	12	4	8	0	6
9	0	9	6	10	0	15
0	0	4	10	1	0	3
13	0	5	6	5	0	8
6	0	7	6	3	0	14
2	0	8	5	1	1	7
1	0	18	8	5	1	9
1	0	14	3	1	1	7
2	0	11	5	7	0	7
4	0	15	1	2	0	10
2	1	20	6	1	0	10
5	0	7	2	2	0	1
4	1	12	7	4	2	7
5	0	21	4	1	0	8
1	0	11	4	4	2	4
0	0	3	3	0	0	7
0	0	4	3	0	0	2
6	0	24	5	3	0	3
3	0	14	5	9	0	4
6	0	3	2	5	0	1
1	0	14	3	8	0	3
4	0	9	1	3	0	4
5	0	11	6	5	0	8
0	0	7	6	1	0	2
6	0	6	2	3	0	1
2	0	21	5	3	0	1
10	0	14	2	4	0	6
8	0	9	9	1	0	2
2	0	4	5	5	1	11
1	0	5	2	1	1	30
0	0	3	3	4	1	5
2	0	12	5	5	1	4
10	0	5	3	3	0	1
4	0	7	2	5	0	7
2	0	9	1	2	3	12
0	0	5	6	1	0	4
4	0	11	10	6	0	4
1	0	4	7	2	0	6
1	0	3	8	7	0	1
4	0	8	4	1	2	2
0	0	4	4	1	0	4
2	0	10	5	0	1	3
3	0	12	0	2	0	7
5	0	2	9	5	0	5
2	0	6	4	0	0	16
3	0	13	1	7	0	5
6	0	8	1	5	0	10
2	0	18	6	2	1	3

2	0	11	5	0	0	4
0	0	9	4	6	1	2
0	0	8	9	0	2	23
1	0	5	5	1	0	3
0	0	9	3	1	0	1
3	0	7	3	4	0	3
5	0	14	7	2	0	8
0	0	8	0	1	0	4
6	0	8	2	2	0	0
6	1	2	2	2	0	1
0	0	7	7	0	0	7
1	1	2	4	0	0	0
0	0	1	0	0	0	0
0	0	4	4	0	0	2
3	0	11	2	8	0	0
1	0	3	5	2	1	2
5	0	5	3	1	0	1
0	0	6	8	3	0	2
0	0	1	2	1	0	1
1	0	2	5	1	0	1
2	0	5	3	1	1	5
1	0	3	6	0	0	3
4	0	17	5	3	0	1
3	0	2	2	1	1	2
1	0	9	2	7	1	1
0	0	0	3	0	0	2
1	0	6	1	3	1	0
1	0	9	2	2	0	7
4	0	9	1	4	0	1
1	0	2	5	3	0	5
0	0	1	2	0	0	0
0	0	11	9	0	0	5
3	0	7	1	6	0	2
8	0	3	2	3	0	0
4	0	0	2	0	0	7
2	0	15	3	6	0	4
0	0	6	2	2	0	0
0	0	4	5	1	0	1
1	0	5	3	2	0	6
1	0	3	2	0	2	0
0	0	1	0	0	1	0
0	0	3	3	1	0	1
2	0	4	3	4	0	3
0	0	14	2	3	0	2
3	0	9	3	2	0	1
0	0	2	2	0	0	1
0	0	2	5	0	0	1
7	0	5	1	4	0	3
2	0	7	3	2	0	2
4	0	6	2	7	0	3
0	0	7	5	2	1	10
0	0	7	5	0	0	3
13	0	5	2	1	2	1
1	0	1	2	0	0	6
0	0	10	5	2	0	0
1	0	3	3	0	0	6
0	0	10	5	5	0	4
0	0	12	1	4	1	2
2	0	2	5	5	0	1

1	0	2	1	0	0	2
3	0	4	1	2	0	1
4	0	6	1	1	2	3
1	0	7	3	0	0	2
0	0	13	0	1	1	2
0	0	1	1	0	0	1
0	0	6	2	1	1	2
0	0	0	2	1	0	0
1	0	5	2	1	1	3
0	0	3	6	1	0	1
3	0	7	3	0	0	0
1	0	7	2	1	0	1
0	0	3	5	0	0	1
1	0	5	1	0	0	0
1	0	3	1	0	0	1
0	0	4	2	2	0	1
1	0	6	3	2	0	1
0	0	1	0	0	0	0
0	0	0	3	0	0	0
1	0	2	0	1	0	7
0	0	3	4	3	0	2
0	0	0	1	0	0	0
0	0	4	0	0	0	0
3	0	3	2	1	1	4
3	0	3	1	0	0	4
0	0	2	4	3	0	1
6	0	4	0	2	1	1
2	0	2	0	2	0	1
0	0	1	0	0	0	0
0	0	2	2	0	0	0
0	0	4	0	0	0	0
3	0	3	1	1	1	2
0	0	4	0	0	0	1
0	0	3	1	1	0	1
2	0	2	1	2	0	3
1	0	4	0	3	0	1
1	0	2	1	0	0	0
0	0	4	1	1	0	0
0	0	5	2	4	0	1
7	0	3	3	6	1	2
0	0	2	4	0	0	2
2	1	7	4	0	0	1
1	0	5	2	1	0	1
1	0	2	0	3	0	2
0	0	3	2	1	0	3
0	0	3	0	1	0	0
1	0	2	0	2	0	2
3	0	9	0	0	0	0
1	0	4	0	2	1	0
5	0	2	1	1	0	1
0	0	3	5	3	0	1
0	0	1	0	1	1	1
0	0	0	0	1	0	0
0	0	2	2	0	0	0
1	0	5	2	0	0	2
3	0	7	2	0	0	2
0	0	5	3	1	0	1
1	0	2	0	0	0	2
0	0	3	4	0	0	3

0	0	2	4	0	0	1
1	0	3	3	3	0	2
0	0	9	3	0	0	3
0	0	5	0	1	0	4
0	0	3	4	0	0	0
1	0	4	0	2	0	1
3	0	5	1	1	0	0
2	0	10	0	0	0	0
0	0	4	2	1	0	3
1	0	0	4	1	0	13
1	0	4	1	1	0	0
1	0	5	2	2	0	2
0	10	0	0	0	0	0
C_47	D_15	D_16	D_17	D_18	D_36	D_4
UC	UC	UC	UC	UC	UC	UC
6567256	48689	7514364	1314450	2784650	169575	2239906
26	23	21	14	22	19	17
1.06	0.44	-0.15	-1.54	0.14	-0.57	-0.94

0.76	0.66	0.21	0.23	0.59	0.57	0.88
7	10	24	22	11	16	5
1	1	1	1	1	1	1
15	16	17	18	19	20	21
15	16	17	18	19	20	21
D_16	D_17	D_18	D_36	D_4	E_47	F_43
UC	UC	UC	UC	UC	UC	UC
4032681	1739788	1227867	121631	2029037	1734052	1026638
D_16	D_17	D_18	D_36	D_4	E_47	F_43
1531849	492299	434340	43427	587465	393385	494991
439203	147428	121333	5509	392427	82970	54523
146839	114004	91145	9645	124341	75041	41641
257462	91626	107193	4797	72204	67885	69737
216353	94252	142992	8299	141988	98228	76677
236351	141379	15073	9190	79097	221090	27687
96321	83961	47442	3216	84166	103902	23181
37646	20264	2086	2689	22024	125143	2006
123431	89451	13489	4537	31482	70550	13874
131928	36434	18771	3976	62023	32415	27676
54535	25861	12887	1522	18899	14010	27254
56370	31108	22005	2555	23168	33546	10282
38107	22508	7706	971	12274	20989	16434
51226	11534	21866	1929	12074	15421	11363
31921	26946	1105	878	11836	28940	6158
25011	19800	10553	1128	4026	13776	9179
20229	15122	1285	446	15501	24133	3570
46387	16987	6462	1895	18873	14832	6400
28660	20805	5612	488	16615	15649	3023

26135	12362	8659	600	32768	9752	7433
30117	7562	10288	817	13028	9166	3726
29467	7813	6300	967	7672	5987	4327
12100	8758	2850	234	19089	9506	6108
9499	7671	2046	566	28264	13140	808
7633	5119	1917	178	10212	12829	2190
20525	7101	7076	537	10933	9001	3253
20654	5910	7129	645	4277	5532	4049
18939	7643	1260	364	10771	10349	4272
12409	8787	2516	387	4527	7445	4213
9324	6564	5175	240	6960	3536	5401
12062	8726	3465	314	10061	9235	1715
11175	4139	5507	350	3526	4999	2298
4610	6155	1070	183	3160	13028	904
2976	6071	563	195	2703	12790	847
11404	8133	2321	358	4165	6853	3974
15472	4644	5836	512	6346	2996	1863
9964	6651	1732	412	11395	4826	2779
5458	5447	1530	86	5968	3812	2200
16089	2287	4079	147	2913	2967	1642
3617	1490	1957	326	4804	3747	695
8931	2670	3233	198	3231	2650	913
5684	4329	4867	184	5708	2571	2464
7925	2349	3039	188	2382	3028	1463
3066	2256	253	170	2355	6940	483
3568	2368	2760	171	3494	2855	645
4992	3193	1653	129	3229	2658	1824
4186	6725	1846	117	2667	2161	1155
6822	1728	1775	172	2486	2225	1273
383	435	64	31	80	7803	253
2173	2128	395	126	4109	8507	407
3862	1835	1788	180	1747	269	569
4225	2672	1062	140	2942	1870	521
5394	1923	1901	222	2882	1176	861
4437	2415	575	78	5573	3091	932
3705	2029	2316	76	1983	2787	324
451	461	60	39	227	6612	152
5246	2251	1394	113	1423	1483	484
2867	2062	913	111	1974	2234	308
3497	1840	978	111	2194	1143	1220
3517	1056	1417	59	898	686	600
2243	1912	843	93	836	901	674
2703	1636	1016	88	3769	1876	344
723	1288	51	23	386	2445	104
601	274	74	6	84	5997	46
2844	1145	590	114	457	925	2043
3331	1525	451	120	840	1480	376
798	1235	314	42	704	2074	215
1680	876	208	37	380	2037	207
1830	858	519	57	1216	1577	250
2905	960	452	16	2410	541	598
2014	1137	1058	56	805	582	500
2019	587	646	87	454	610	884
1377	1240	58	38	564	1348	248
3183	926	510	129	1034	977	430
1190	1295	434	52	959	1216	249
1462	931	519	117	502	175	263
3552	1119	1401	66	1446	784	264
1422	632	537	32	587	727	303

1746	770	606	124	499	460	304
1062	2057	577	98	547	296	382
1547	766	487	57	349	805	258
1060	836	617	79	582	578	409
1501	543	433	21	517	420	544
627	710	365	13	243	869	194
888	703	387	25	683	1038	100
1200	563	776	63	412	443	154
964	846	539	42	652	343	538
627	802	33	60	416	1028	104
925	615	124	42	430	664	304
1082	436	289	45	300	240	243
676	397	582	43	694	518	448
258	79	13	1	4	1448	37
859	406	528	23	268	648	103
1244	906	146	81	241	391	176
460	464	57	23	183	778	64
545	810	139	24	370	702	130
912	426	324	13	457	242	335
833	359	292	45	494	291	207
474	217	435	15	467	216	255
891	492	32	14	194	539	81
1137	179	324	50	811	185	47
522	481	278	33	502	375	195
556	387	344	20	218	183	247
418	760	149	22	399	250	84
528	485	91	25	253	370	136
508	312	59	41	292	529	42
536	324	213	43	357	215	87
599	331	221	11	509	349	128
412	327	106	26	127	429	73
829	245	88	23	188	575	49
723	411	142	40	585	503	117
653	253	248	15	345	207	145
495	167	252	18	656	131	36
771	252	106	21	163	374	70
252	200	234	19	109	234	43
421	205	216	12	116	109	136
505	191	215	14	293	307	63
212	520	39	12	102	275	92
212	520	39	12	102	275	92
548	228	185	10	410	129	43
399	198	207	39	125	137	75
327	218	156	15	234	126	114
534	230	129	33	190	85	13
352	248	102	2	657	295	40
56	116	10	1	21	698	11
231	244	116	9	117	132	118
242	149	247	13	130	94	71
372	201	162	29	180	123	85
346	155	158	9	213	112	42
343	86	134	8	78	75	44
192	80	102	1	89	404	53
299	198	97	16	197	174	41
277	315	100	3	106	106	62
283	102	174	20	123	95	43
117	163	41	6	63	209	23
342	225	86	8	187	88	78
161	108	69	1	63	169	77

292	117	118	18	63	131	59
211	160	66	4	71	169	41
189	132	125	18	65	119	41
216	100	135	4	94	212	41
353	120	97	11	81	111	43
135	88	105	38	124	77	12
165	137	76	1	55	154	32
41	76	5	3	22	256	12
347	63	73	6	61	66	99
196	86	67	0	106	89	39
192	116	62	2	90	225	35
136	81	49	2	58	74	77
292	70	96	15	110	67	34
308	209	19	9	266	192	40
208	93	25	10	47	150	28
166	149	30	23	51	71	18
104	56	78	2	100	96	24
34	47	16	20	24	151	6
228	86	61	7	42	46	83
224	71	89	3	101	36	28
103	111	89	6	82	33	42
137	76	101	9	64	70	40
222	82	87	8	78	101	23
23	43	6	1	18	337	9
149	64	90	7	109	34	14
168	51	53	8	80	32	46
129	107	16	6	71	124	22
131	180	17	1	114	87	25
155	94	51	7	36	75	62
146	55	59	5	129	54	31
54	66	20	3	152	53	26
69	72	77	3	47	259	4
176	77	68	11	29	44	36
110	2	108	1	94	62	19
117	87	43	2	36	47	73
167	88	62	5	106	33	62
312	102	25	4	198	85	29
134	56	14	3	80	247	6
219	38	58	3	57	56	30
195	87	39	6	14	4	20
90	82	71	8	121	100	39
145	38	70	11	49	52	9
100	56	76	9	23	7	16
121	40	46	4	36	52	42
106	82	8	2	50	101	17
139	60	35	3	19	9	23
110	103	18	3	53	134	13
117	66	37	2	50	21	71
116	49	28	10	34	55	20
101	81	6	2	48	96	16
107	31	18	5	22	6	18
82	171	24	2	54	41	21
97	114	42	0	44	79	28
37	48	65	0	1	10	1
92	90	7	9	63	67	11
136	89	30	6	99	54	25
118	60	23	5	54	31	25
133	55	48	4	69	34	42
63	61	35	1	39	72	16

61	37	8	1	37	330	4
139	40	55	8	23	58	29
109	52	44	10	38	36	10
142	70	26	0	11	29	23
97	38	35	3	72	55	9
92	75	25	6	70	27	43
90	28	60	4	59	47	8
147	54	38	0	50	42	29
75	47	10	1	24	52	4
62	34	49	4	12	8	12
101	29	23	5	68	33	17
132	51	37	2	72	39	11
69	14	58	3	57	25	15
112	21	31	4	17	65	22
100	33	25	7	38	39	14
121	77	34	4	135	30	10
40	48	3	13	37	36	14
123	38	54	7	23	22	7
78	47	21	2	41	61	20
89	50	37	0	82	38	17
68	62	34	0	41	48	19
100	44	21	10	36	42	10
29	13	0	0	2	96	0
59	22	30	3	31	30	4
53	13	16	0	62	108	5
110	41	27	4	29	16	22
62	28	30	0	32	41	7
127	35	38	1	18	16	6
54	41	15	5	13	3	7
43	21	28	1	23	28	17
53	23	31	3	19	19	16
49	35	36	5	33	28	10
51	25	23	3	28	24	13
84	24	43	0	25	19	10
48	46	8	3	54	45	10
87	22	34	2	39	34	24
14	19	0	1	8	85	2
41	34	34	0	11	19	8
54	50	13	2	9	35	11
71	4	29	4	19	28	18
30	20	31	1	12	22	6
21	28	4	0	32	30	0
53	39	7	1	35	25	7
57	29	20	1	15	24	6
52	26	8	5	5	4	10
41	32	14	2	15	14	6
42	23	4	1	47	57	1
34	11	11	0	37	35	1
34	11	11	0	37	35	1
42	15	10	1	6	3	7
55	14	20	1	23	21	9
51	12	14	1	17	49	5
45	31	8	3	0	4	5
29	16	12	0	19	48	6
44	28	26	0	51	21	4
51	29	4	1	13	8	17
54	14	10	3	6	27	3
32	17	27	0	8	29	4
58	14	2	2	60	27	2

95	7	59	0	16	17	13
21	33	13	2	5	37	5
69	13	33	0	19	14	5
5	2	0	0	0	61	0
45	21	15	0	20	22	8
31	40	1	0	18	23	11
30	22	16	0	14	13	8
60	15	22	0	8	7	15
30	40	1	0	17	21	10
50	29	13	4	10	7	10
41	9	32	3	6	17	7
38	21	14	3	13	10	3
36	14	13	1	5	11	11
8	1	1	0	2	73	1
19	9	12	7	11	16	8
57	10	14	3	15	14	4
33	6	20	0	12	11	3
23	15	11	3	8	3	3
36	14	15	1	42	29	7
8	8	0	0	3	36	0
33	10	9	0	14	12	3
22	29	2	1	22	18	3
1	0	0	7	0	0	0
29	12	16	0	9	15	14
24	16	13	2	10	11	17
40	9	12	0	26	8	3
39	10	12	5	6	2	1
16	23	26	4	8	5	12
19	18	9	3	9	4	4
23	12	15	2	19	11	4
18	23	10	4	0	4	14
38	11	22	2	26	10	10
21	27	2	1	21	14	3
7	12	5	2	5	4	3
12	26	1	0	5	14	3
7	13	0	1	16	34	3
17	27	12	0	32	6	5
35	8	20	1	19	6	5
11	16	9	2	10	21	5
6	7	2	4	6	12	0
20	17	9	3	10	3	3
28	21	1	3	11	34	3
48	19	8	0	13	10	10
22	17	15	0	14	18	0
17	17	16	0	13	12	6
20	7	5	3	5	4	0
20	12	8	2	5	0	5
16	10	11	0	7	14	0
30	7	8	0	11	17	0
20	23	21	3	6	2	7
23	22	3	2	36	11	5
9	3	11	3	25	14	1
10	21	19	0	8	10	9
7	20	7	1	6	6	8
14	11	9	3	19	4	2
18	27	6	0	11	13	3
24	3	2	1	18	6	11
11	10	0	1	13	33	2
5	28	1	1	20	19	5

21	8	9	0	3	8	2
33	9	3	1	6	10	4
34	6	6	0	8	5	5
36	6	18	0	13	8	2
31	9	17	0	13	8	2
18	12	21	0	4	21	3
23	15	9	2	1	12	2
12	22	4	1	8	14	2
25	12	8	0	19	9	2
17	5	6	1	1	0	5
29	12	14	0	4	5	3
22	13	4	1	10	3	0
16	22	8	3	2	0	3
15	12	8	0	3	12	6
25	2	6	0	4	9	8
17	5	11	0	2	3	10
15	7	5	0	17	5	3
22	5	4	1	5	2	7
11	9	1	0	5	25	1
13	7	1	0	4	7	3
12	12	0	0	12	18	3
33	7	14	1	5	10	2
17	4	7	0	5	9	3
13	12	9	2	9	4	3
1	1	1	0	0	29	4
30	6	6	0	8	6	4
13	14	5	1	15	7	9
13	11	10	0	5	7	5
19	6	8	1	8	8	6
15	11	10	2	9	6	2
7	11	6	0	19	3	5
26	3	6	2	6	7	2
24	5	7	0	12	5	2
5	10	1	2	7	6	3
26	6	4	0	1	6	1
7	11	7	1	5	8	2
13	11	5	2	5	7	3
12	4	8	0	6	21	0
9	6	10	0	15	7	5
4	10	1	0	3	17	0
5	6	5	0	8	7	3
7	6	3	0	14	4	0
8	5	1	1	7	4	1
18	8	5	1	9	2	5
14	3	1	1	7	12	0
11	5	7	0	7	3	0
15	1	2	0	10	10	5
20	6	1	0	10	9	2
7	2	2	0	1	4	6
12	7	4	2	7	7	10
21	4	1	0	8	4	6
11	4	4	2	4	4	3
3	3	0	0	7	13	3
4	3	0	0	2	19	1
24	5	3	0	3	5	1
14	5	9	0	4	3	0
3	2	5	0	1	2	6
14	3	8	0	3	6	0
9	1	3	0	4	4	0

11	6	5	0	8	3	6
7	6	1	0	2	9	0
6	2	3	0	1	2	2
21	5	3	0	1	7	2
14	2	4	0	6	3	3
9	9	1	0	2	2	4
4	5	5	1	11	3	4
5	2	1	1	30	2	0
3	3	4	1	5	2	2
12	5	5	1	4	0	0
5	3	3	0	1	4	3
7	2	5	0	7	5	2
9	1	2	3	12	5	0
5	6	1	0	4	6	1
11	10	6	0	4	6	3
4	7	2	0	6	9	0
3	8	7	0	1	1	1
8	4	1	2	2	2	0
4	4	1	0	4	3	2
10	5	0	1	3	14	0
12	0	2	0	7	3	1
2	9	5	0	5	10	2
6	4	0	0	16	3	0
13	1	7	0	5	6	1
8	1	5	0	10	2	1
18	6	2	1	3	10	5
11	5	0	0	4	5	1
9	4	6	1	2	0	2
8	9	0	2	23	7	1
5	5	1	0	3	3	1
9	3	1	0	1	3	1
7	3	4	0	3	1	3
14	7	2	0	8	4	4
8	0	1	0	4	8	0
8	2	2	0	0	4	0
2	2	2	0	1	3	1
7	7	0	0	7	2	0
2	4	0	0	0	16	0
1	0	0	0	0	14	0
4	4	0	0	2	7	0
11	2	8	0	0	10	1
3	5	2	1	2	0	1
5	3	1	0	1	2	3
6	8	3	0	2	2	1
1	2	1	0	1	16	0
2	5	1	0	1	3	1
5	3	1	1	5	2	0
3	6	0	0	3	8	0
17	5	3	0	1	0	1
2	2	1	1	2	4	2
9	2	7	1	1	5	0
0	3	0	0	2	18	0
6	1	3	1	0	10	1
9	2	2	0	7	3	2
9	1	4	0	1	4	2
2	5	3	0	5	6	1
1	2	0	0	0	10	0
11	9	0	0	5	5	2
7	1	6	0	2	2	2

3	2	3	0	0	5	0
0	2	0	0	7	7	1
15	3	6	0	4	3	1
6	2	2	0	0	6	1
4	5	1	0	1	3	1
5	3	2	0	6	7	1
3	2	0	2	0	0	0
1	0	0	1	0	6	1
3	3	1	0	1	1	1
4	3	4	0	3	4	2
14	2	3	0	2	2	0
9	3	2	0	1	2	0
2	2	0	0	1	1	1
2	5	0	0	1	6	1
5	1	4	0	3	3	1
7	3	2	0	2	0	2
6	2	7	0	3	2	2
7	5	2	1	10	3	2
7	5	0	0	3	0	0
5	2	1	2	1	2	1
1	2	0	0	6	2	0
10	5	2	0	0	3	0
3	3	0	0	6	4	0
10	5	5	0	4	0	1
12	1	4	1	2	2	1
2	5	5	0	1	0	2
2	1	0	0	2	4	3
4	1	2	0	1	1	1
6	1	1	2	3	2	2
7	3	0	0	2	1	2
13	0	1	1	2	3	2
1	1	0	0	1	6	0
6	2	1	1	2	1	1
0	2	1	0	0	3	1
5	2	1	1	3	0	0
3	6	1	0	1	1	1
7	3	0	0	0	1	0
7	2	1	0	1	4	0
3	5	0	0	1	4	1
5	1	0	0	0	2	0
3	1	0	0	1	6	0
4	2	2	0	1	2	0
6	3	2	0	1	4	0
1	0	0	0	0	1	0
0	3	0	0	0	6	0
2	0	1	0	7	1	0
3	4	3	0	2	3	1
0	1	0	0	0	3	1
4	0	0	0	0	6	0
3	2	1	1	4	1	1
3	1	0	0	4	1	0
2	4	3	0	1	1	1
4	0	2	1	1	2	0
2	0	2	0	1	0	2
1	0	0	0	0	4	0
2	2	0	0	0	4	0
4	0	0	0	0	0	0
3	1	1	1	2	1	0
4	0	0	0	1	4	0

3	1	1	0	1	0	1
2	1	2	0	3	1	0
4	0	3	0	1	1	0
2	1	0	0	0	2	0
4	1	1	0	0	4	0
5	2	4	0	1	3	0
3	3	6	1	2	1	1
2	4	0	0	2	1	0
7	4	0	0	1	2	0
5	2	1	0	1	2	1
2	0	3	0	2	1	0
3	2	1	0	3	0	0
3	0	1	0	0	2	1
2	0	2	0	2	1	0
9	0	0	0	0	1	0
4	0	2	1	0	2	3
2	1	1	0	1	2	1
3	5	3	0	1	1	0
1	0	1	1	1	3	1
0	0	1	0	0	1	1
2	2	0	0	0	2	0
5	2	0	0	2	0	0
7	2	0	0	2	1	0
5	3	1	0	1	1	0
2	0	0	0	2	1	0
3	4	0	0	3	2	0
2	4	0	0	1	1	1
3	3	3	0	2	2	1
9	3	0	0	3	2	0
5	0	1	0	4	2	0
3	4	0	0	0	3	1
4	0	2	0	1	0	0
5	1	1	0	0	1	0
10	0	0	0	0	0	0
4	2	1	0	3	0	0
0	4	1	0	13	0	0
4	1	1	0	0	1	0
5	2	2	0	2	0	2

0.76	0.66	0.21	0.23	0.59	0.57	0.88
7	10	24	22	11	16	5
1	1	1	1	1	1	1
15	16	17	18	19	20	21
15	16	17	18	19	20	21
D_16	D_17	D_18	D_36	D_4	E_47	F_43
UC	UC	UC	UC	UC	UC	UC
4032681	1739788	1227867	121631	2029037	1734052	1026638
UC	UC	UC	UC	UC	UC	UC
1531849	492299	434340	43427	587465	393385	494991
439203	147428	121333	5509	392427	82970	54523
146839	114004	91145	9645	124341	75041	41641
257462	91626	107193	4797	72204	67885	69737
216353	94252	142992	8299	141988	98228	76677

236351	141379	15073	9190	79097	221090	27687
96321	83961	47442	3216	84166	103902	23181
37646	20264	2086	2689	22024	125143	2006
123431	89451	13489	4537	31482	70550	13874
131928	36434	18771	3976	62023	32415	27676
54535	25861	12887	1522	18899	14010	27254
56370	31108	22005	2555	23168	33546	10282
38107	22508	7706	971	12274	20989	16434
51226	11534	21866	1929	12074	15421	11363
31921	26946	1105	878	11836	28940	6158
25011	19800	10553	1128	4026	13776	9179
20229	15122	1285	446	15501	24133	3570
46387	16987	6462	1895	18873	14832	6400
28660	20805	5612	488	16615	15649	3023
26135	12362	8659	600	32768	9752	7433
30117	7562	10288	817	13028	9166	3726
29467	7813	6300	967	7672	5987	4327
12100	8758	2850	234	19089	9506	6108
9499	7671	2046	566	28264	13140	808
7633	5119	1917	178	10212	12829	2190
20525	7101	7076	537	10933	9001	3253
20654	5910	7129	645	4277	5532	4049
18939	7643	1260	364	10771	10349	4272
12409	8787	2516	387	4527	7445	4213
9324	6564	5175	240	6960	3536	5401
12062	8726	3465	314	10061	9235	1715
11175	4139	5507	350	3526	4999	2298
4610	6155	1070	183	3160	13028	904
2976	6071	563	195	2703	12790	847
11404	8133	2321	358	4165	6853	3974
15472	4644	5836	512	6346	2996	1863
9964	6651	1732	412	11395	4826	2779
5458	5447	1530	86	5968	3812	2200
16089	2287	4079	147	2913	2967	1642
3617	1490	1957	326	4804	3747	695
8931	2670	3233	198	3231	2650	913
5684	4329	4867	184	5708	2571	2464
7925	2349	3039	188	2382	3028	1463
3066	2256	253	170	2355	6940	483
3568	2368	2760	171	3494	2855	645
4992	3193	1653	129	3229	2658	1824
4186	6725	1846	117	2667	2161	1155
6822	1728	1775	172	2486	2225	1273
383	435	64	31	80	7803	253
2173	2128	395	126	4109	8507	407
3862	1835	1788	180	1747	269	569
4225	2672	1062	140	2942	1870	521
5394	1923	1901	222	2882	1176	861
4437	2415	575	78	5573	3091	932
3705	2029	2316	76	1983	2787	324
451	461	60	39	227	6612	152
5246	2251	1394	113	1423	1483	484
2867	2062	913	111	1974	2234	308
3497	1840	978	111	2194	1143	1220
3517	1056	1417	59	898	686	600
2243	1912	843	93	836	901	674
2703	1636	1016	88	3769	1876	344
723	1288	51	23	386	2445	104
601	274	74	6	84	5997	46

2844	1145	590	114	457	925	2043
3331	1525	451	120	840	1480	376
798	1235	314	42	704	2074	215
1680	876	208	37	380	2037	207
1830	858	519	57	1216	1577	250
2905	960	452	16	2410	541	598
2014	1137	1058	56	805	582	500
2019	587	646	87	454	610	884
1377	1240	58	38	564	1348	248
3183	926	510	129	1034	977	430
1190	1295	434	52	959	1216	249
1462	931	519	117	502	175	263
3552	1119	1401	66	1446	784	264
1422	632	537	32	587	727	303
1746	770	606	124	499	460	304
1062	2057	577	98	547	296	382
1547	766	487	57	349	805	258
1060	836	617	79	582	578	409
1501	543	433	21	517	420	544
627	710	365	13	243	869	194
888	703	387	25	683	1038	100
1200	563	776	63	412	443	154
964	846	539	42	652	343	538
627	802	33	60	416	1028	104
925	615	124	42	430	664	304
1082	436	289	45	300	240	243
676	397	582	43	694	518	448
258	79	13	1	4	1448	37
859	406	528	23	268	648	103
1244	906	146	81	241	391	176
460	464	57	23	183	778	64
545	810	139	24	370	702	130
912	426	324	13	457	242	335
833	359	292	45	494	291	207
474	217	435	15	467	216	255
891	492	32	14	194	539	81
1137	179	324	50	811	185	47
522	481	278	33	502	375	195
556	387	344	20	218	183	247
418	760	149	22	399	250	84
528	485	91	25	253	370	136
508	312	59	41	292	529	42
536	324	213	43	357	215	87
599	331	221	11	509	349	128
412	327	106	26	127	429	73
829	245	88	23	188	575	49
723	411	142	40	585	503	117
653	253	248	15	345	207	145
495	167	252	18	656	131	36
771	252	106	21	163	374	70
252	200	234	19	109	234	43
421	205	216	12	116	109	136
505	191	215	14	293	307	63
212	520	39	12	102	275	92
212	520	39	12	102	275	92
548	228	185	10	410	129	43
399	198	207	39	125	137	75
327	218	156	15	234	126	114
534	230	129	33	190	85	13

352	248	102	2	657	295	40
56	116	10	1	21	698	11
231	244	116	9	117	132	118
242	149	247	13	130	94	71
372	201	162	29	180	123	85
346	155	158	9	213	112	42
343	86	134	8	78	75	44
192	80	102	1	89	404	53
299	198	97	16	197	174	41
277	315	100	3	106	106	62
283	102	174	20	123	95	43
117	163	41	6	63	209	23
342	225	86	8	187	88	78
161	108	69	1	63	169	77
292	117	118	18	63	131	59
211	160	66	4	71	169	41
189	132	125	18	65	119	41
216	100	135	4	94	212	41
353	120	97	11	81	111	43
135	88	105	38	124	77	12
165	137	76	1	55	154	32
41	76	5	3	22	256	12
347	63	73	6	61	66	99
196	86	67	0	106	89	39
192	116	62	2	90	225	35
136	81	49	2	58	74	77
292	70	96	15	110	67	34
308	209	19	9	266	192	40
208	93	25	10	47	150	28
166	149	30	23	51	71	18
104	56	78	2	100	96	24
34	47	16	20	24	151	6
228	86	61	7	42	46	83
224	71	89	3	101	36	28
103	111	89	6	82	33	42
137	76	101	9	64	70	40
222	82	87	8	78	101	23
23	43	6	1	18	337	9
149	64	90	7	109	34	14
168	51	53	8	80	32	46
129	107	16	6	71	124	22
131	180	17	1	114	87	25
155	94	51	7	36	75	62
146	55	59	5	129	54	31
54	66	20	3	152	53	26
69	72	77	3	47	259	4
176	77	68	11	29	44	36
110	2	108	1	94	62	19
117	87	43	2	36	47	73
167	88	62	5	106	33	62
312	102	25	4	198	85	29
134	56	14	3	80	247	6
219	38	58	3	57	56	30
195	87	39	6	14	4	20
90	82	71	8	121	100	39
145	38	70	11	49	52	9
100	56	76	9	23	7	16
121	40	46	4	36	52	42
106	82	8	2	50	101	17

139	60	35	3	19	9	23
110	103	18	3	53	134	13
117	66	37	2	50	21	71
116	49	28	10	34	55	20
101	81	6	2	48	96	16
107	31	18	5	22	6	18
82	171	24	2	54	41	21
97	114	42	0	44	79	28
37	48	65	0	1	10	1
92	90	7	9	63	67	11
136	89	30	6	99	54	25
118	60	23	5	54	31	25
133	55	48	4	69	34	42
63	61	35	1	39	72	16
61	37	8	1	37	330	4
139	40	55	8	23	58	29
109	52	44	10	38	36	10
142	70	26	0	11	29	23
97	38	35	3	72	55	9
92	75	25	6	70	27	43
90	28	60	4	59	47	8
147	54	38	0	50	42	29
75	47	10	1	24	52	4
62	34	49	4	12	8	12
101	29	23	5	68	33	17
132	51	37	2	72	39	11
69	14	58	3	57	25	15
112	21	31	4	17	65	22
100	33	25	7	38	39	14
121	77	34	4	135	30	10
40	48	3	13	37	36	14
123	38	54	7	23	22	7
78	47	21	2	41	61	20
89	50	37	0	82	38	17
68	62	34	0	41	48	19
100	44	21	10	36	42	10
29	13	0	0	2	96	0
59	22	30	3	31	30	4
53	13	16	0	62	108	5
110	41	27	4	29	16	22
62	28	30	0	32	41	7
127	35	38	1	18	16	6
54	41	15	5	13	3	7
43	21	28	1	23	28	17
53	23	31	3	19	19	16
49	35	36	5	33	28	10
51	25	23	3	28	24	13
84	24	43	0	25	19	10
48	46	8	3	54	45	10
87	22	34	2	39	34	24
14	19	0	1	8	85	2
41	34	34	0	11	19	8
54	50	13	2	9	35	11
71	4	29	4	19	28	18
30	20	31	1	12	22	6
21	28	4	0	32	30	0
53	39	7	1	35	25	7
57	29	20	1	15	24	6
52	26	8	5	5	4	10

41	32	14	2	15	14	6
42	23	4	1	47	57	1
34	11	11	0	37	35	1
34	11	11	0	37	35	1
42	15	10	1	6	3	7
55	14	20	1	23	21	9
51	12	14	1	17	49	5
45	31	8	3	0	4	5
29	16	12	0	19	48	6
44	28	26	0	51	21	4
51	29	4	1	13	8	17
54	14	10	3	6	27	3
32	17	27	0	8	29	4
58	14	2	2	60	27	2
95	7	59	0	16	17	13
21	33	13	2	5	37	5
69	13	33	0	19	14	5
5	2	0	0	0	61	0
45	21	15	0	20	22	8
31	40	1	0	18	23	11
30	22	16	0	14	13	8
60	15	22	0	8	7	15
30	40	1	0	17	21	10
50	29	13	4	10	7	10
41	9	32	3	6	17	7
38	21	14	3	13	10	3
36	14	13	1	5	11	11
8	1	1	0	2	73	1
19	9	12	7	11	16	8
57	10	14	3	15	14	4
33	6	20	0	12	11	3
23	15	11	3	8	3	3
36	14	15	1	42	29	7
8	8	0	0	3	36	0
33	10	9	0	14	12	3
22	29	2	1	22	18	3
1	0	0	7	0	0	0
29	12	16	0	9	15	14
24	16	13	2	10	11	17
40	9	12	0	26	8	3
39	10	12	5	6	2	1
16	23	26	4	8	5	12
19	18	9	3	9	4	4
23	12	15	2	19	11	4
18	23	10	4	0	4	14
38	11	22	2	26	10	10
21	27	2	1	21	14	3
7	12	5	2	5	4	3
12	26	1	0	5	14	3
7	13	0	1	16	34	3
17	27	12	0	32	6	5
35	8	20	1	19	6	5
11	16	9	2	10	21	5
6	7	2	4	6	12	0
20	17	9	3	10	3	3
28	21	1	3	11	34	3
48	19	8	0	13	10	10
22	17	15	0	14	18	0
17	17	16	0	13	12	6

20	7	5	3	5	4	0
20	12	8	2	5	0	5
16	10	11	0	7	14	0
30	7	8	0	11	17	0
20	23	21	3	6	2	7
23	22	3	2	36	11	5
9	3	11	3	25	14	1
10	21	19	0	8	10	9
7	20	7	1	6	6	8
14	11	9	3	19	4	2
18	27	6	0	11	13	3
24	3	2	1	18	6	11
11	10	0	1	13	33	2
5	28	1	1	20	19	5
21	8	9	0	3	8	2
33	9	3	1	6	10	4
34	6	6	0	8	5	5
36	6	18	0	13	8	2
31	9	17	0	13	8	2
18	12	21	0	4	21	3
23	15	9	2	1	12	2
12	22	4	1	8	14	2
25	12	8	0	19	9	2
17	5	6	1	1	0	5
29	12	14	0	4	5	3
22	13	4	1	10	3	0
16	22	8	3	2	0	3
15	12	8	0	3	12	6
25	2	6	0	4	9	8
17	5	11	0	2	3	10
15	7	5	0	17	5	3
22	5	4	1	5	2	7
11	9	1	0	5	25	1
13	7	1	0	4	7	3
12	12	0	0	12	18	3
33	7	14	1	5	10	2
17	4	7	0	5	9	3
13	12	9	2	9	4	3
1	1	1	0	0	29	4
30	6	6	0	8	6	4
13	14	5	1	15	7	9
13	11	10	0	5	7	5
19	6	8	1	8	8	6
15	11	10	2	9	6	2
7	11	6	0	19	3	5
26	3	6	2	6	7	2
24	5	7	0	12	5	2
5	10	1	2	7	6	3
26	6	4	0	1	6	1
7	11	7	1	5	8	2
13	11	5	2	5	7	3
12	4	8	0	6	21	0
9	6	10	0	15	7	5
4	10	1	0	3	17	0
5	6	5	0	8	7	3
7	6	3	0	14	4	0
8	5	1	1	7	4	1
18	8	5	1	9	2	5
14	3	1	1	7	12	0

11	5	7	0	7	3	0
15	1	2	0	10	10	5
20	6	1	0	10	9	2
7	2	2	0	1	4	6
12	7	4	2	7	7	10
21	4	1	0	8	4	6
11	4	4	2	4	4	3
3	3	0	0	7	13	3
4	3	0	0	2	19	1
24	5	3	0	3	5	1
14	5	9	0	4	3	0
3	2	5	0	1	2	6
14	3	8	0	3	6	0
9	1	3	0	4	4	0
11	6	5	0	8	3	6
7	6	1	0	2	9	0
6	2	3	0	1	2	2
21	5	3	0	1	7	2
14	2	4	0	6	3	3
9	9	1	0	2	2	4
4	5	5	1	11	3	4
5	2	1	1	30	2	0
3	3	4	1	5	2	2
12	5	5	1	4	0	0
5	3	3	0	1	4	3
7	2	5	0	7	5	2
9	1	2	3	12	5	0
5	6	1	0	4	6	1
11	10	6	0	4	6	3
4	7	2	0	6	9	0
3	8	7	0	1	1	1
8	4	1	2	2	2	0
4	4	1	0	4	3	2
10	5	0	1	3	14	0
12	0	2	0	7	3	1
2	9	5	0	5	10	2
6	4	0	0	16	3	0
13	1	7	0	5	6	1
8	1	5	0	10	2	1
18	6	2	1	3	10	5
11	5	0	0	4	5	1
9	4	6	1	2	0	2
8	9	0	2	23	7	1
5	5	1	0	3	3	1
9	3	1	0	1	3	1
7	3	4	0	3	1	3
14	7	2	0	8	4	4
8	0	1	0	4	8	0
8	2	2	0	0	4	0
2	2	2	0	1	3	1
7	7	0	0	7	2	0
2	4	0	0	0	16	0
1	0	0	0	0	14	0
4	4	0	0	2	7	0
11	2	8	0	0	10	1
3	5	2	1	2	0	1
5	3	1	0	1	2	3
6	8	3	0	2	2	1
1	2	1	0	1	16	0

2	5	1	0	1	3	1
5	3	1	1	5	2	0
3	6	0	0	3	8	0
17	5	3	0	1	0	1
2	2	1	1	2	4	2
9	2	7	1	1	5	0
0	3	0	0	2	18	0
6	1	3	1	0	10	1
9	2	2	0	7	3	2
9	1	4	0	1	4	2
2	5	3	0	5	6	1
1	2	0	0	0	10	0
11	9	0	0	5	5	2
7	1	6	0	2	2	2
3	2	3	0	0	5	0
0	2	0	0	7	7	1
15	3	6	0	4	3	1
6	2	2	0	0	6	1
4	5	1	0	1	3	1
5	3	2	0	6	7	1
3	2	0	2	0	0	0
1	0	0	1	0	6	1
3	3	1	0	1	1	1
4	3	4	0	3	4	2
14	2	3	0	2	2	0
9	3	2	0	1	2	0
2	2	0	0	1	1	1
2	5	0	0	1	6	1
5	1	4	0	3	3	1
7	3	2	0	2	0	2
6	2	7	0	3	2	2
7	5	2	1	10	3	2
7	5	0	0	3	0	0
5	2	1	2	1	2	1
1	2	0	0	6	2	0
10	5	2	0	0	3	0
3	3	0	0	6	4	0
10	5	5	0	4	0	1
12	1	4	1	2	2	1
2	5	5	0	1	0	2
2	1	0	0	2	4	3
4	1	2	0	1	1	1
6	1	1	2	3	2	2
7	3	0	0	2	1	2
13	0	1	1	2	3	2
1	1	0	0	1	6	0
6	2	1	1	2	1	1
0	2	1	0	0	3	1
5	2	1	1	3	0	0
3	6	1	0	1	1	1
7	3	0	0	0	1	0
7	2	1	0	1	4	0
3	5	0	0	1	4	1
5	1	0	0	0	2	0
3	1	0	0	1	6	0
4	2	2	0	1	2	0
6	3	2	0	1	4	0
1	0	0	0	0	1	0
0	3	0	0	0	6	0

2	0	1	0	7	1	0
3	4	3	0	2	3	1
0	1	0	0	0	3	1
4	0	0	0	0	6	0
3	2	1	1	4	1	1
3	1	0	0	4	1	0
2	4	3	0	1	1	1
4	0	2	1	1	2	0
2	0	2	0	1	0	2
1	0	0	0	0	4	0
2	2	0	0	0	4	0
4	0	0	0	0	0	0
3	1	1	1	2	1	0
4	0	0	0	1	4	0
3	1	1	0	1	0	1
2	1	2	0	3	1	0
4	0	3	0	1	1	0
2	1	0	0	0	2	0
4	1	1	0	0	4	0
5	2	4	0	1	3	0
3	3	6	1	2	1	1
2	4	0	0	2	1	0
7	4	0	0	1	2	0
5	2	1	0	1	2	1
2	0	3	0	2	1	0
3	2	1	0	3	0	0
3	0	1	0	0	2	1
2	0	2	0	2	1	0
9	0	0	0	0	1	0
4	0	2	1	0	2	3
2	1	1	0	1	2	1
3	5	3	0	1	1	0
1	0	1	1	1	3	1
0	0	1	0	0	1	1
2	2	0	0	0	2	0
5	2	0	0	2	0	0
7	2	0	0	2	1	0
5	3	1	0	1	1	0
2	0	0	0	2	1	0
3	4	0	0	3	2	0
2	4	0	0	1	1	1
3	3	3	0	2	2	1
9	3	0	0	3	2	0
5	0	1	0	4	2	0
3	4	0	0	0	3	1
4	0	2	0	1	0	0
5	1	1	0	0	1	0
10	0	0	0	0	0	0
4	2	1	0	3	0	0
0	4	1	0	13	0	0
4	1	1	0	0	1	0
5	2	2	0	2	0	2
125554	52902	37952	3754	62633	51605	32381
282834	93261	84112	7965	124019	80516	89622
335	380	406	470	387	377	425
UC	UC	UC	UC	UC	UC	UC
UC	UC	UC	UC	UC	UC	UC

15	16	17	18	19	20	21
15	16	17	18	19	20	21
UC	UC	UC	UC	UC	UC	UC
D_16	D_17	D_18	D_36	D_4	E_47	F_43
4032681	1739788	1227867	121631	2029037	1734052	1026638
UC	UC	UC	UC	UC	UC	UC
12.20	9.31	11.44	11.57	9.38	7.62	15.29
3.50	2.79	3.20	1.47	6.27	1.61	1.68
1.17	2.16	2.40	2.57	1.99	1.45	1.29
2.05	1.73	2.82	1.28	1.15	1.32	2.15
1.72	1.78	3.77	2.21	2.27	1.90	2.37
1.88	2.67	0.40	2.45	1.26	4.28	0.86
0.77	1.59	1.25	0.86	1.34	2.01	0.72
0.30	0.38	0.05	0.72	0.35	2.43	0.06
0.98	1.69	0.36	1.21	0.50	1.37	0.43
1.05	0.69	0.49	1.06	0.99	0.63	0.85
0.43	0.49	0.34	0.41	0.30	0.27	0.84
0.45	0.59	0.58	0.68	0.37	0.65	0.32
0.30	0.43	0.20	0.26	0.20	0.41	0.51
0.41	0.22	0.58	0.51	0.19	0.30	0.35
0.25	0.51	0.03	0.23	0.19	0.56	0.19
0.20	0.37	0.28	0.30	0.06	0.27	0.28
0.16	0.29	0.03	0.12	0.25	0.47	0.11
0.37	0.32	0.17	0.50	0.30	0.29	0.20
0.23	0.39	0.15	0.13	0.27	0.30	0.09
0.21	0.23	0.23	0.16	0.52	0.19	0.23
0.24	0.14	0.27	0.22	0.21	0.18	0.12
0.23	0.15	0.17	0.26	0.12	0.12	0.13
0.10	0.17	0.08	0.06	0.30	0.18	0.19
0.08	0.15	0.05	0.15	0.45	0.25	0.02
0.06	0.10	0.05	0.05	0.16	0.25	0.07
0.16	0.13	0.19	0.14	0.17	0.17	0.10
0.16	0.11	0.19	0.17	0.07	0.11	0.13
0.15	0.14	0.03	0.10	0.17	0.20	0.13
0.10	0.17	0.07	0.10	0.07	0.14	0.13
0.07	0.12	0.14	0.06	0.11	0.07	0.17
0.10	0.16	0.09	0.08	0.16	0.18	0.05
0.09	0.08	0.15	0.09	0.06	0.10	0.07
0.04	0.12	0.03	0.05	0.05	0.25	0.03
0.02	0.11	0.01	0.05	0.04	0.25	0.03
0.09	0.15	0.06	0.10	0.07	0.13	0.12
0.12	0.09	0.15	0.14	0.10	0.06	0.06
0.08	0.13	0.05	0.11	0.18	0.09	0.09
0.04	0.10	0.04	0.02	0.10	0.07	0.07
0.13	0.04	0.11	0.04	0.05	0.06	0.05
0.03	0.03	0.05	0.09	0.08	0.07	0.02
0.07	0.05	0.09	0.05	0.05	0.05	0.03
0.05	0.08	0.13	0.05	0.09	0.05	0.08
0.06	0.04	0.08	0.05	0.04	0.06	0.05
0.02	0.04	0.01	0.05	0.04	0.13	0.01
0.03	0.04	0.07	0.05	0.06	0.06	0.02
0.04	0.06	0.04	0.03	0.05	0.05	0.06
0.03	0.13	0.05	0.03	0.04	0.04	0.04
0.05	0.03	0.05	0.05	0.04	0.04	0.04
0.00	0.01	0.00	0.01	0.00	0.15	0.01
0.02	0.04	0.01	0.03	0.07	0.16	0.01
0.03	0.03	0.05	0.05	0.03	0.01	0.02
0.03	0.05	0.03	0.04	0.05	0.04	0.02

0.04	0.04	0.05	0.06	0.05	0.02	0.03
0.04	0.05	0.02	0.02	0.09	0.06	0.03
0.03	0.04	0.06	0.02	0.03	0.05	0.01
0.00	0.01	0.00	0.01	0.00	0.13	0.00
0.04	0.04	0.04	0.03	0.02	0.03	0.01
0.02	0.04	0.02	0.03	0.03	0.04	0.01
0.03	0.03	0.03	0.03	0.04	0.02	0.04
0.03	0.02	0.04	0.02	0.01	0.01	0.02
0.02	0.04	0.02	0.02	0.01	0.02	0.02
0.02	0.03	0.03	0.02	0.06	0.04	0.01
0.01	0.02	0.00	0.01	0.01	0.05	0.00
0.00	0.01	0.00	0.00	0.00	0.12	0.00
0.02	0.02	0.02	0.03	0.01	0.02	0.06
0.03	0.03	0.01	0.03	0.01	0.03	0.01
0.01	0.02	0.01	0.01	0.01	0.04	0.01
0.01	0.02	0.01	0.01	0.01	0.04	0.01
0.01	0.02	0.01	0.02	0.02	0.03	0.01
0.02	0.02	0.01	0.00	0.04	0.01	0.02
0.02	0.02	0.03	0.01	0.01	0.01	0.02
0.02	0.01	0.02	0.02	0.01	0.01	0.03
0.01	0.02	0.00	0.01	0.01	0.03	0.01
0.03	0.02	0.01	0.03	0.02	0.02	0.01
0.01	0.02	0.01	0.01	0.02	0.02	0.01
0.01	0.02	0.01	0.03	0.01	0.00	0.01
0.03	0.02	0.04	0.02	0.02	0.02	0.01
0.01	0.01	0.01	0.01	0.01	0.01	0.01
0.01	0.01	0.02	0.03	0.01	0.01	0.01
0.01	0.04	0.02	0.03	0.01	0.01	0.01
0.01	0.01	0.01	0.02	0.01	0.02	0.01
0.01	0.02	0.02	0.02	0.01	0.01	0.01
0.01	0.01	0.01	0.01	0.01	0.01	0.02
0.00	0.01	0.01	0.00	0.00	0.02	0.01
0.01	0.01	0.01	0.01	0.01	0.02	0.00
0.01	0.01	0.02	0.02	0.01	0.01	0.00
0.01	0.02	0.01	0.01	0.01	0.01	0.02
0.00	0.02	0.00	0.02	0.01	0.02	0.00
0.01	0.01	0.00	0.01	0.01	0.01	0.01
0.01	0.01	0.01	0.01	0.00	0.00	0.01
0.01	0.01	0.02	0.01	0.01	0.01	0.01
0.00	0.00	0.00	0.00	0.00	0.03	0.00
0.01	0.01	0.01	0.01	0.00	0.01	0.00
0.01	0.02	0.00	0.02	0.00	0.01	0.01
0.00	0.01	0.00	0.01	0.00	0.02	0.00
0.00	0.02	0.00	0.01	0.01	0.01	0.00
0.01	0.01	0.01	0.00	0.01	0.00	0.01
0.01	0.01	0.01	0.01	0.01	0.01	0.01
0.00	0.00	0.01	0.00	0.01	0.00	0.01
0.01	0.01	0.00	0.00	0.00	0.01	0.00
0.01	0.00	0.01	0.01	0.01	0.00	0.00
0.00	0.01	0.01	0.01	0.01	0.01	0.01
0.00	0.01	0.01	0.01	0.00	0.00	0.01
0.00	0.01	0.00	0.01	0.01	0.00	0.00
0.00	0.01	0.00	0.01	0.00	0.01	0.00
0.00	0.01	0.00	0.01	0.00	0.01	0.00
0.00	0.01	0.01	0.01	0.01	0.00	0.00
0.00	0.01	0.01	0.00	0.01	0.01	0.00
0.00	0.01	0.00	0.01	0.00	0.01	0.00
0.01	0.00	0.00	0.01	0.00	0.01	0.00
0.01	0.01	0.00	0.01	0.01	0.01	0.00

0.01	0.00	0.01	0.00	0.01	0.00	0.00
0.00	0.00	0.01	0.00	0.01	0.00	0.00
0.01	0.00	0.00	0.01	0.00	0.01	0.00
0.00	0.00	0.01	0.01	0.00	0.00	0.00
0.00	0.00	0.01	0.00	0.00	0.00	0.00
0.00	0.00	0.01	0.00	0.00	0.01	0.00
0.00	0.01	0.00	0.00	0.00	0.01	0.00
0.00	0.01	0.00	0.00	0.00	0.01	0.00
0.00	0.00	0.00	0.00	0.01	0.00	0.00
0.00	0.00	0.01	0.01	0.00	0.00	0.00
0.00	0.00	0.00	0.00	0.00	0.00	0.00
0.00	0.00	0.00	0.01	0.00	0.00	0.00
0.00	0.00	0.00	0.00	0.01	0.01	0.00
0.00	0.00	0.00	0.00	0.00	0.01	0.00
0.00	0.00	0.00	0.00	0.00	0.00	0.00
0.00	0.00	0.01	0.00	0.00	0.00	0.00
0.00	0.00	0.00	0.01	0.00	0.00	0.00
0.00	0.00	0.00	0.00	0.00	0.00	0.00
0.00	0.00	0.00	0.00	0.00	0.00	0.00
0.00	0.00	0.00	0.00	0.00	0.01	0.00
0.00	0.00	0.00	0.00	0.00	0.00	0.00
0.00	0.01	0.00	0.00	0.00	0.00	0.00
0.00	0.00	0.00	0.01	0.00	0.00	0.00
0.00	0.00	0.00	0.00	0.00	0.00	0.00
0.00	0.00	0.00	0.00	0.00	0.00	0.00
0.00	0.00	0.00	0.00	0.00	0.00	0.00
0.00	0.00	0.00	0.00	0.00	0.00	0.00
0.00	0.00	0.00	0.00	0.00	0.00	0.00
0.00	0.00	0.00	0.00	0.00	0.00	0.00
0.00	0.00	0.00	0.00	0.00	0.00	0.00
0.00	0.00	0.00	0.00	0.00	0.00	0.00
0.00	0.00	0.00	0.01	0.00	0.00	0.00
0.00	0.00	0.00	0.00	0.00	0.00	0.00
0.00	0.00	0.00	0.00	0.00	0.00	0.00
0.00	0.00	0.00	0.00	0.00	0.00	0.00
0.00	0.00	0.00	0.00	0.00	0.00	0.00
0.00	0.00	0.00	0.00	0.00	0.00	0.00
0.00	0.00	0.00	0.00	0.00	0.00	0.00
0.00	0.00	0.00	0.00	0.00	0.00	0.00
0.00	0.00	0.00	0.00	0.00	0.00	0.00
0.00	0.00	0.00	0.00	0.00	0.00	0.00
0.00	0.00	0.00	0.01	0.00	0.00	0.00
0.00	0.00	0.00	0.00	0.00	0.00	0.00
0.00	0.00	0.00	0.01	0.00	0.00	0.00
0.00	0.00	0.00	0.00	0.00	0.00	0.00
0.00	0.00	0.00	0.00	0.00	0.00	0.00
0.00	0.00	0.00	0.00	0.00	0.00	0.00
0.00	0.00	0.00	0.00	0.00	0.00	0.00
0.00	0.00	0.00	0.00	0.00	0.00	0.00
0.00	0.00	0.00	0.00	0.00	0.01	0.00
0.00	0.00	0.00	0.00	0.00	0.00	0.00
0.00	0.00	0.00	0.00	0.00	0.00	0.00
0.00	0.00	0.00	0.00	0.00	0.00	0.00
0.00	0.00	0.00	0.00	0.00	0.00	0.00
0.00	0.00	0.00	0.00	0.00	0.00	0.00
0.00	0.00	0.00	0.00	0.00	0.00	0.00
0.00	0.00	0.00	0.00	0.00	0.00	0.00
0.00	0.00	0.00	0.00	0.00	0.01	0.00
0.00	0.00	0.00	0.00	0.00	0.00	0.00

0.00	0.00	0.00	0.00	0.00	0.00	0.00
0.00	0.00	0.00	0.00	0.00	0.00	0.00
0.00	0.00	0.00	0.00	0.00	0.00	0.00
0.00	0.00	0.00	0.00	0.00	0.00	0.00
0.00	0.00	0.00	0.00	0.00	0.00	0.00
0.00	0.00	0.00	0.00	0.00	0.00	0.00
0.00	0.00	0.00	0.00	0.00	0.00	0.00
0.00	0.00	0.00	0.00	0.00	0.00	0.00
0.00	0.00	0.00	0.00	0.00	0.00	0.00
0.00	0.00	0.00	0.00	0.00	0.00	0.00
0.00	0.00	0.00	0.00	0.00	0.00	0.00
0.00	0.00	0.00	0.00	0.00	0.00	0.00
0.00	0.00	0.00	0.00	0.00	0.00	0.00
0.00	0.00	0.00	0.00	0.00	0.00	0.00
0.00	0.00	0.00	0.00	0.00	0.00	0.00
0.00	0.00	0.00	0.00	0.00	0.00	0.00
0.00	0.00	0.00	0.00	0.00	0.00	0.00
0.00	0.00	0.00	0.00	0.00	0.00	0.00
0.00	0.00	0.00	0.00	0.00	0.00	0.00
0.00	0.00	0.00	0.00	0.00	0.00	0.00
0.00	0.00	0.00	0.00	0.00	0.00	0.00
0.00	0.00	0.00	0.00	0.00	0.00	0.00
0.00	0.00	0.00	0.00	0.00	0.00	0.00
0.00	0.00	0.00	0.00	0.00	0.00	0.00
0.00	0.00	0.00	0.00	0.00	0.00	0.00
0.00	0.00	0.00	0.00	0.00	0.00	0.00
0.00	0.00	0.00	0.00	0.00	0.01	0.00
0.00	0.00	0.00	0.00	0.00	0.00	0.00
0.00	0.00	0.00	0.00	0.00	0.00	0.00
0.00	0.00	0.00	0.00	0.00	0.00	0.00
0.00	0.00	0.00	0.00	0.00	0.00	0.00
0.00	0.00	0.00	0.00	0.00	0.00	0.00
0.00	0.00	0.00	0.00	0.00	0.00	0.00
0.00	0.00	0.00	0.00	0.00	0.00	0.00
0.00	0.00	0.00	0.00	0.00	0.00	0.00
0.00	0.00	0.00	0.00	0.00	0.00	0.00
0.00	0.00	0.00	0.00	0.00	0.00	0.00
0.00	0.00	0.00	0.00	0.00	0.00	0.00
0.00	0.00	0.00	0.00	0.00	0.00	0.00
0.00	0.00	0.00	0.00	0.00	0.00	0.00
0.00	0.00	0.00	0.00	0.00	0.00	0.00
0.00	0.00	0.00	0.00	0.00	0.00	0.00
0.00	0.00	0.00	0.00	0.00	0.00	0.00
0.00	0.00	0.00	0.00	0.00	0.00	0.00
0.00	0.00	0.00	0.00	0.00	0.00	0.00
0.00	0.00	0.00	0.00	0.00	0.00	0.00
0.00	0.00	0.00	0.00	0.00	0.00	0.00
0.00	0.00	0.00	0.00	0.00	0.00	0.00
0.00	0.00	0.00	0.00	0.00	0.00	0.00
0.00	0.00	0.00	0.00	0.00	0.00	0.00
0.00	0.00	0.00	0.00	0.00	0.00	0.00
0.00	0.00	0.00	0.00	0.00	0.00	0.00
0.00	0.00	0.00	0.00	0.00	0.00	0.00
0.00	0.00	0.00	0.00	0.00	0.00	0.00
0.00	0.00	0.00	0.00	0.00	0.00	0.00
0.00	0.00	0.00	0.00	0.00	0.00	0.00
0.00	0.00	0.00	0.00	0.00	0.00	0.00
0.00	0.00	0.00	0.00	0.00	0.00	0.00
0.00	0.00	0.00	0.00	0.00	0.00	0.00

0.00	0.00	0.00	0.00	0.00	0.00	0.00
0.00	0.00	0.00	0.00	0.00	0.00	0.00
0.00	0.00	0.00	0.00	0.00	0.00	0.00
0.00	0.00	0.00	0.00	0.00	0.00	0.00
0.00	0.00	0.00	0.00	0.00	0.00	0.00
0.00	0.00	0.00	0.00	0.00	0.00	0.00
0.00	0.00	0.00	0.00	0.00	0.00	0.00
0.00	0.00	0.00	0.00	0.00	0.00	0.00
0.00	0.00	0.00	0.00	0.00	0.00	0.00
0.00	0.00	0.00	0.00	0.00	0.00	0.00
0.00	0.00	0.00	0.00	0.00	0.00	0.00
0.00	0.00	0.00	0.00	0.00	0.00	0.00
0.00	0.00	0.00	0.00	0.00	0.00	0.00
0.00	0.00	0.00	0.00	0.00	0.00	0.00
0.00	0.00	0.00	0.00	0.00	0.00	0.00
0.00	0.00	0.00	0.00	0.00	0.00	0.00
0.00	0.00	0.00	0.00	0.00	0.00	0.00
0.00	0.00	0.00	0.00	0.00	0.00	0.00
0.00	0.00	0.00	0.00	0.00	0.00	0.00
0.00	0.00	0.00	0.00	0.00	0.00	0.00
0.00	0.00	0.00	0.00	0.00	0.00	0.00
0.00	0.00	0.00	0.00	0.00	0.00	0.00
0.00	0.00	0.00	0.00	0.00	0.00	0.00
0.00	0.00	0.00	0.00	0.00	0.00	0.00
0.00	0.00	0.00	0.00	0.00	0.00	0.00
0.00	0.00	0.00	0.00	0.00	0.00	0.00
0.00	0.00	0.00	0.00	0.00	0.00	0.00
0.00	0.00	0.00	0.00	0.00	0.00	0.00
0.00	0.00	0.00	0.00	0.00	0.00	0.00
0.00	0.00	0.00	0.00	0.00	0.00	0.00

15	16	17	18	19	20	21
17	18	19	20	21	22	23
UC	UC	UC	UC	UC	UC	UC
D_16	D_17	D_18	D_36	D_4	E_47	F_43
4032681	1739788	1227867	121631	2029037	1734052	1026638
D_16	D_17	D_18	D_36	D_4	E_47	F_43
0.13	-0.52	-0.04	-0.01	-0.50	-0.90	0.82
0.07	-0.14	-0.02	-0.53	0.88	-0.49	-0.47
-0.71	0.03	0.22	0.35	-0.09	-0.49	-0.62
-0.39	-0.71	0.37	-1.16	-1.28	-1.12	-0.29
-0.49	-0.42	1.77	0.05	0.11	-0.29	0.23
0.62	1.34	-0.72	1.14	0.06	2.80	-0.30
-0.63	0.67	0.13	-0.49	0.28	1.35	-0.72
-0.08	0.07	-0.52	0.68	0.02	3.77	-0.51

0.78	2.19	-0.47	1.23	-0.17	1.54	-0.32
0.75	-0.18	-0.68	0.77	0.59	-0.34	0.24
-0.14	0.07	-0.50	-0.25	-0.64	-0.75	1.39
-0.38	0.86	0.79	1.69	-1.09	1.42	-1.56
-0.31	0.39	-0.89	-0.57	-0.93	0.28	0.86
-0.34	-1.43	0.63	0.27	-1.58	-0.97	-0.67
0.16	1.33	-0.87	0.07	-0.14	1.57	-0.13
-0.42	0.34	-0.08	0.02	-1.01	-0.13	-0.06
0.02	0.81	-0.78	-0.24	0.57	1.96	-0.30
1.53	1.05	-0.43	2.86	0.86	0.72	-0.17
0.42	1.89	-0.30	-0.46	0.75	1.09	-0.79
-0.22	0.03	-0.02	-0.70	2.92	-0.41	-0.01
1.33	-0.23	1.83	0.97	0.82	0.33	-0.68
1.27	-0.06	0.22	1.62	-0.44	-0.54	-0.27
-0.28	0.58	-0.54	-0.70	2.30	0.81	0.86
-0.41	0.24	-0.62	0.30	3.13	1.28	-0.89
-0.41	-0.20	-0.47	-0.49	0.20	0.70	-0.37
0.91	0.36	1.35	0.53	1.12	1.12	-0.27
0.42	-0.42	0.79	0.53	-1.11	-0.49	-0.21
0.37	0.30	-0.76	-0.15	0.57	0.84	0.18
-0.16	1.15	-0.80	-0.08	-0.68	0.72	0.45
-0.65	0.51	0.80	-0.89	0.21	-0.79	1.51
0.13	1.73	0.02	-0.16	1.63	2.06	-0.88
-0.64	-0.85	0.45	-0.56	-1.27	-0.49	-0.99
-0.35	0.86	-0.47	-0.16	-0.14	2.91	-0.48
-0.27	0.23	-0.32	-0.12	-0.16	0.96	-0.26
-0.08	1.33	-0.75	0.02	-0.63	0.86	0.63
-0.12	-0.34	0.06	-0.04	-0.26	-0.52	-0.52
-0.40	0.15	-0.80	-0.04	0.81	-0.23	-0.32
-0.50	0.11	-0.54	-0.72	0.03	-0.19	-0.25
0.34	-0.67	0.10	-0.72	-0.64	-0.50	-0.58
-0.57	-0.58	-0.39	-0.10	-0.19	-0.22	-0.63
-0.10	-0.52	0.19	-0.47	-0.50	-0.50	-0.98
-0.70	0.40	1.79	-0.59	0.68	-0.57	0.22
-0.05	-0.80	0.64	-0.58	-1.06	-0.23	-0.77
-0.32	0.13	-0.76	0.19	0.00	2.41	-0.56
-0.73	-0.38	0.22	-0.36	-0.14	-0.15	-0.91
-0.48	1.01	-0.21	-0.88	0.37	0.37	0.72
-0.30	3.48	0.32	-0.39	0.07	0.04	-0.21
0.16	-0.56	-0.09	-0.13	-0.33	-0.22	-0.34
-0.29	-0.11	-0.34	-0.11	-0.36	4.92	-0.13
-0.29	0.23	-0.45	0.08	0.81	3.08	-0.40
-0.25	-0.04	0.63	0.68	-0.40	-1.63	-0.96
-0.02	0.47	-0.19	0.09	0.37	0.06	-0.53
0.56	0.05	1.12	1.82	0.80	-1.00	-0.70
0.33	0.87	-0.73	-0.44	3.16	1.62	-0.02
0.25	0.74	2.00	-0.26	0.37	1.61	-0.83

-0.24	-0.03	-0.32	0.04	-0.23	4.85	-0.19
1.11	1.17	0.70	0.16	-0.43	0.05	-1.06
-0.10	1.24	0.00	0.46	0.62	1.60	-1.20
-0.20	0.31	-0.36	-0.07	0.33	-0.63	0.53
-0.34	-0.55	-0.09	-0.66	-0.70	-0.73	-0.59
-0.54	0.93	-0.19	0.02	-0.90	-0.57	-0.30
-0.04	0.74	0.39	0.12	3.16	1.19	-0.94
-0.28	1.18	-0.62	-0.25	-0.25	2.98	-0.48
-0.08	-0.06	-0.21	-0.22	-0.23	4.98	-0.23
-0.39	-0.45	-0.80	0.05	-1.28	-0.66	1.94
0.96	1.19	-0.45	1.49	-0.30	1.17	-0.47
-0.42	0.82	-0.28	-0.07	-0.06	2.06	-0.40
0.29	0.61	-0.49	-0.06	-0.43	2.88	-0.40
0.21	0.40	0.11	0.28	0.76	2.03	-0.57
-0.13	-0.19	-0.27	-0.37	0.07	-0.29	-0.19
-0.39	0.00	0.45	-0.47	-0.61	-0.72	-0.43
-0.43	-0.92	-0.33	0.27	-1.30	-0.85	0.68
0.08	1.25	-0.80	0.00	-0.10	1.50	-0.23
1.51	0.48	-0.06	2.70	0.35	0.67	-0.08
-0.19	1.67	0.05	0.35	0.53	1.56	-0.42
-0.46	0.03	-0.29	1.15	-0.76	-1.14	-0.75
1.68	0.81	2.72	0.37	1.04	0.09	-0.77
-0.26	-0.25	-0.19	-0.34	-0.31	-0.19	-0.31
-0.34	-0.28	-0.15	1.42	-0.88	-0.79	-0.75
-0.59	2.25	0.04	1.06	-0.57	-0.85	-0.28
0.26	0.68	0.36	0.82	-1.07	0.90	-0.60
-0.55	0.50	0.57	1.25	-0.43	-0.16	0.05
-0.42	-0.58	-0.47	-1.01	-0.77	-0.78	0.02
-0.28	0.11	-0.06	-0.35	-0.33	0.27	-0.23
-0.14	0.90	0.38	-0.21	0.50	2.05	-0.82
0.14	0.33	2.07	1.42	-0.39	-0.03	-0.71
-1.10	0.56	0.21	-0.40	-0.55	-1.30	0.69
-0.18	1.49	-0.87	1.63	0.09	2.28	-0.48
-0.26	0.86	-1.35	0.75	-0.40	1.19	0.27
-0.40	-0.45	-0.52	0.02	-0.87	-0.89	-0.54
-0.65	-0.11	1.91	0.91	0.81	0.54	1.52
0.07	-0.04	-0.25	-0.27	-0.30	4.95	-0.10
0.05	0.27	1.93	-0.14	-0.63	1.57	-0.92
0.84	2.30	-0.39	3.20	-0.39	0.37	-0.07
0.00	0.97	-0.41	0.47	-0.14	2.17	-0.32
-0.33	1.85	-0.46	0.08	-0.02	1.51	-0.39
-0.15	0.04	0.15	-1.06	-0.14	-0.77	0.58
-0.11	-0.05	0.29	1.93	0.37	-0.49	-0.20
-0.85	-0.77	0.98	-0.80	0.03	-0.75	0.13
0.82	1.32	-0.62	0.04	-0.10	1.58	-0.24
0.56	-0.43	0.47	1.30	1.24	-0.39	-0.77
-0.34	1.65	0.94	1.53	1.22	0.92	0.42
-0.67	-0.05	0.32	-0.47	-0.87	-0.85	0.02

-0.42	0.50	-0.37	-0.21	-0.17	-0.29	-0.48
-0.13	1.51	-0.73	0.68	-0.19	0.85	-0.14
0.19	0.72	-0.53	2.16	0.36	1.97	-0.61
-0.35	0.21	0.06	1.82	0.08	-0.38	-0.82
-0.27	-0.06	-0.12	-0.54	0.22	0.02	-0.39
-0.15	0.77	-0.31	1.00	-0.55	1.44	-0.48
1.16	0.41	-0.46	0.98	-0.20	2.88	-0.77
0.54	1.25	-0.18	2.26	1.80	1.94	-0.22
0.25	0.04	0.92	-0.36	0.41	-0.35	-0.11
-0.06	-0.32	0.83	0.22	2.10	-0.52	-0.99
1.15	0.59	-0.20	0.93	-0.28	1.59	-0.46
-0.73	-0.07	0.82	0.41	-0.83	0.21	-0.98
-0.64	-0.41	0.37	-0.71	-1.29	-1.18	-0.27
0.53	0.27	1.58	0.35	0.95	1.77	-0.79
-0.64	2.80	-0.92	0.00	-0.66	0.90	-0.15
-0.64	2.80	-0.92	0.00	-0.66	0.90	-0.15
0.00	-0.01	0.15	-0.47	0.61	-0.51	-0.84
-0.14	0.06	0.68	2.45	-0.56	-0.33	-0.45
-0.47	0.46	0.45	0.38	0.22	-0.57	0.09
0.58	0.61	0.27	2.22	0.14	-0.37	-0.82
0.05	0.89	-0.01	-0.97	3.48	1.35	-0.66
-0.23	0.41	-0.30	-0.30	-0.27	4.54	-0.27
-0.87	0.33	-0.35	-0.63	-0.86	-0.56	-0.09
-0.44	0.01	1.84	0.33	-0.36	-0.49	-0.30
0.01	0.44	0.68	2.47	-0.04	-0.29	-0.16
-0.19	-0.13	0.31	-0.31	0.04	-0.39	-0.70
-0.20	-0.44	-0.01	-0.33	-0.53	-0.48	-0.50
-0.12	-0.12	0.37	-0.64	-0.16	2.52	-0.07
0.14	1.20	0.27	1.60	0.73	0.91	-0.73
0.03	2.67	0.33	-0.96	-0.34	-0.08	-0.18
-0.22	-0.35	0.70	0.99	-0.33	-0.38	-0.58
-0.33	0.91	-0.24	0.06	-0.28	1.46	-0.45
0.37	1.71	-0.03	-0.15	0.60	-0.52	0.10

12	13	14	15	16	17	18
14	15	16	17	18	19	20
TGTAAACATCCC(	TGAGGTAGTAGTT	TGAGGTAGTAG(	TGAGGTAGTAGAT	AGCTCGGTCTGA(	TTCAAGTAATTCA	AGCAGCATTGTA(
miR-30d-5p	let-7g-5p	let-7b-5p	let-7f-5p	miR-423-3p	miR-26b-5p	miR-103a-3p
2131733	1899491	1666971	1418336	1093922	1034642	1030775
1.36	-0.70	2.23	-0.82	0.03	-0.79	-0.02
-1.18	-1.21	-1.07	-0.14	-1.22	-0.15	0.44
0.67	0.87	1.24	0.39	-0.87	0.07	0.76
-0.53	1.05	1.04	-0.35	-0.64	-0.29	-0.33
-0.37	2.12	0.48	0.34	-1.06	1.51	1.38
-1.57	0.23	-0.45	-0.04	1.52	-0.34	-1.13
0.31	1.39	-0.41	-0.60	2.37	-0.33	-0.23
0.77	-1.39	0.48	-0.86	0.36	-0.80	-1.13
-0.94	-1.02	-0.41	-0.43	-0.53	-0.64	-1.03
-1.48	-0.32	-0.23	0.20	-0.77	-0.38	-0.64
-0.41	-0.92	-1.34	0.23	-0.54	0.01	-0.83
-0.62	-0.13	-0.25	0.08	1.58	-0.69	-0.68
0.02	2.52	-0.88	3.89	-0.34	3.45	1.13
-0.26	-1.23	1.60	-0.85	0.20	-0.82	-0.92
-0.38	-0.31	-0.34	0.16	-0.42	0.02	1.53
0.86	0.39	-1.43	1.33	0.34	0.81	1.05
0.79	-0.89	0.63	-0.87	-0.08	-0.78	-0.43
1.69	-0.57	0.27	0.07	0.02	-0.24	2.86
-1.09	-0.93	-1.58	-0.14	-1.01	0.57	0.86
1.42	0.28	-0.97	1.57	-0.13	1.96	0.72

-1.56	0.86	-0.67	-0.13	-0.06	-0.30	-0.17
1.04	0.21	-0.06	-0.69	1.71	-0.73	-0.09
1.58	0.30	0.73	-0.60	1.01	-0.74	0.11
0.57	-0.56	1.83	-0.83	1.46	-0.86	-0.49
-0.97	-0.51	0.38	-0.57	-1.06	-0.34	-1.15
0.43	0.09	-0.14	-0.74	-0.90	-0.19	-0.73
-0.16	0.35	-0.67	0.41	-0.98	1.00	-0.82
-1.11	1.60	-0.97	3.67	-0.88	1.74	0.06
-1.90	0.14	-1.69	1.03	-0.80	0.70	-0.14
-2.45	-1.15	-1.30	-0.09	-1.13	-0.30	0.20
-1.11	-1.33	1.11	-0.74	-1.15	-0.80	-0.06
-2.10	-0.25	-1.17	1.39	-1.23	0.77	1.21
-0.74	1.21	-0.06	1.59	-1.14	3.42	1.17
-0.37	-1.15	0.14	-0.73	1.15	-0.46	-0.75
-0.43	-1.28	1.18	-0.67	-0.63	-0.65	0.28
-0.78	1.05	-1.14	-0.59	0.59	-0.32	-0.77
-2.02	-1.74	2.07	-0.90	0.71	-0.96	-1.84
-0.75	0.71	-1.12	0.17	-0.86	-0.05	0.40
-0.21	0.68	-1.06	1.16	-0.90	1.30	0.90
0.55	-0.09	-1.01	-0.28	-0.42	-0.08	-0.21
-0.79	-0.79	1.50	-0.88	0.49	-0.76	0.41
-0.73	-0.29	3.71	-0.84	2.47	-0.77	2.61
0.39	-0.36	-0.60	-0.75	0.55	-0.67	-0.28
-1.43	0.49	-1.59	0.18	-0.32	0.76	1.57
-0.60	-1.55	-1.23	-0.66	-0.34	-0.68	-0.39
-0.85	-1.30	1.47	-0.92	1.32	-0.85	-1.04
0.82	0.09	-1.66	-0.02	-0.19	0.85	0.06
1.89	-0.26	-0.98	-0.40	0.98	-0.42	0.33
3.59	-0.88	-0.77	-0.28	0.58	-0.14	0.95
1.84	-0.56	-0.95	-0.65	0.57	-0.36	0.15
2.95	-0.43	-1.23	-0.19	-0.12	0.19	1.00
0.67	-0.02	-0.59	-0.80	0.32	-0.58	-0.13
-0.78	-0.40	0.56	-0.70	-0.20	-0.64	-0.25
0.77	1.27	-0.76	0.01	0.43	0.24	1.50
-0.69	-0.09	-0.60	-0.31	0.59	-0.53	-0.67
-0.37	-0.95	0.13	-0.81	-0.53	-0.67	-0.59
0.20	0.74	-0.62	-0.20	-0.12	-0.23	-0.15
-1.13	2.98	-1.21	2.56	-0.98	3.28	-0.66
0.73	1.44	0.12	-0.59	-0.60	-0.37	-0.80
2.52	2.98	-0.12	0.82	-0.93	1.38	0.71
1.27	2.87	-0.54	0.45	-1.05	1.52	0.50
-1.15	1.90	-1.03	2.24	-1.00	2.50	-0.58
0.27	-0.78	1.15	-0.81	-0.46	-0.65	-1.46
-0.30	0.82	0.18	-0.46	-0.19	-0.61	-1.29
-0.57	1.59	0.18	0.15	-0.88	0.33	-1.08
0.49	1.66	0.24	2.44	0.38	-0.48	1.76
-0.81	-0.52	-0.27	-0.19	-0.97	0.16	-0.36
-2.17	-1.08	-1.02	0.05	-1.25	-0.29	2.03
5.34	5.24	6.59	0.77	-0.76	0.44	3.48
5.55	2.97	1.48	0.55	-0.70	1.84	3.75
-0.14	0.67	-0.02	-0.36	-0.12	-0.26	0.11
-1.03	2.51	-2.12	4.99	-1.16	0.45	-0.96
-0.74	1.06	0.21	0.60	-0.39	0.05	-0.55
1.01	0.47	0.87	1.39	-0.22	1.04	1.06
-1.12	-1.64	8.15	-0.97	2.03	-0.95	0.14
-0.36	2.20	-0.75	1.68	0.50	0.76	0.57
-1.54	-0.62	-1.54	-0.07	-1.14	0.23	0.84
-1.86	-1.17	-0.96	0.51	-1.03	-0.42	-0.12
0.57	0.70	-0.74	0.61	-0.80	2.07	2.68

0.66	-0.03	-1.32	0.28	-0.51	0.97	0.10
0.67	-1.44	0.20	-0.77	-0.34	-0.71	-0.84
-0.73	1.86	-0.79	0.32	-0.36	1.01	-0.11
1.28	-1.43	-1.22	-0.69	1.88	-0.73	-0.49
1.16	0.48	-1.32	2.11	-0.29	1.79	1.38
-0.74	1.91	-1.20	4.03	-0.87	3.05	0.40
0.70	-1.26	-1.22	-0.64	-0.04	-0.34	0.02
1.07	0.76	-1.02	-0.06	0.02	1.11	0.41
-0.31	0.21	-0.66	-0.54	0.00	-0.11	-0.34
-0.50	0.31	-1.48	1.49	-0.77	1.81	1.72
-0.30	-0.10	-1.02	-0.46	-0.15	-0.06	0.17
-0.11	7.32	-0.62	5.96	-0.34	1.52	1.07
-1.93	-0.21	-1.42	-0.58	-0.57	-0.32	-0.30
1.81	0.37	0.27	-0.65	1.13	-0.64	-0.31
-1.52	0.28	-1.02	0.08	-0.98	0.47	-0.82
-0.38	1.22	-0.16	-0.55	-0.86	-0.07	-0.71
-1	0	0	0	-1	-1	0
0.00	0.00	0.00	0.00	0.00	0.00	0.00
1.00	1.00	1.00	1.00	1.00	1.00	1.00
0.49	0.19	0.75	0.05	0.61	0.26	0.05

12	13	14	15	16	17	18
TGTAAACATCCC(	TGAGGTAGTAGTT	TGAGGTAGTAG(	TGAGGTAGTAGAT	AGCTCGGTCTGA(	TTCAAGTAATTCA	AGCAGCATTGTA(
miR-30d-5p	let-7g-5p	let-7b-5p	let-7f-5p	miR-423-3p	miR-26b-5p	miR-103a-3p
2131733	1899491	1666971	1418336	1093922	1034642	1030775
1.36	-0.70	2.23	-0.82	0.03	-0.79	-0.02
-1.18	-1.21	-1.07	-0.14	-1.22	-0.15	0.44
0.67	0.87	1.24	0.39	-0.87	0.07	0.76
-0.53	1.05	1.04	-0.35	-0.64	-0.29	-0.33
-0.37	2.12	0.48	0.34	-1.06	1.51	1.38
-1.57	0.23	-0.45	-0.04	1.52	-0.34	-1.13
0.31	1.39	-0.41	-0.60	2.37	-0.33	-0.23
0.77	-1.39	0.48	-0.86	0.36	-0.80	-1.13
-0.94	-1.02	-0.41	-0.43	-0.53	-0.64	-1.03
-1.48	-0.32	-0.23	0.20	-0.77	-0.38	-0.64
-0.41	-0.92	-1.34	0.23	-0.54	0.01	-0.83
-0.62	-0.13	-0.25	0.08	1.58	-0.69	-0.68
0.02	2.52	-0.88	3.89	-0.34	3.45	1.13
-0.26	-1.23	1.60	-0.85	0.20	-0.82	-0.92
-0.38	-0.31	-0.34	0.16	-0.42	0.02	1.53
0.86	0.39	-1.43	1.33	0.34	0.81	1.05
0.79	-0.89	0.63	-0.87	-0.08	-0.78	-0.43
1.69	-0.57	0.27	0.07	0.02	-0.24	2.86
-1.09	-0.93	-1.58	-0.14	-1.01	0.57	0.86
1.42	0.28	-0.97	1.57	-0.13	1.96	0.72
-1.56	0.86	-0.67	-0.13	-0.06	-0.30	-0.17
1.04	0.21	-0.06	-0.69	1.71	-0.73	-0.09
1.58	0.30	0.73	-0.60	1.01	-0.74	0.11
0.57	-0.56	1.83	-0.83	1.46	-0.86	-0.49
-0.97	-0.51	0.38	-0.57	-1.06	-0.34	-1.15
0.43	0.09	-0.14	-0.74	-0.90	-0.19	-0.73
-0.16	0.35	-0.67	0.41	-0.98	1.00	-0.82
-1.11	1.60	-0.97	3.67	-0.88	1.74	0.06

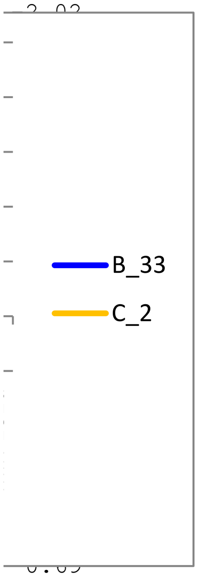

-1.90	0.14	-1.69	1.03	-0.80	0.70	-0.14
-2.45	-1.15	-1.30	-0.09	-1.13	-0.30	0.20
-1.11	-1.33	1.11	-0.74	-1.15	-0.80	-0.06
-2.10	-0.25	-1.17	1.39	-1.23	0.77	1.21
-0.74	1.21	-0.06	1.59	-1.14	3.42	1.17
-0.37	-1.15	0.14	-0.73	1.15	-0.46	-0.75
-0.43	-1.28	1.18	-0.67	-0.63	-0.65	0.28
-0.78	1.05	-1.14	-0.59	0.59	-0.32	-0.77
[illegible]	-1.74	2.07	-0.90	0.71	-0.96	-1.84
[illegible]	0.71	-1.12	0.17	-0.86	-0.05	0.40
[illegible]	0.68	-1.06	1.16	-0.90	1.30	0.90
[illegible]	-0.09	-1.01	-0.28	-0.42	-0.08	-0.21
[illegible]	-0.79	1.50	-0.88	0.49	-0.76	0.41
[illegible]	-0.29	3.71	-0.84	2.47	-0.77	2.61
[illegible]	-0.36	-0.60	-0.75	0.55	-0.67	-0.28
[illegible]	0.49	-1.59	0.18	-0.32	0.76	1.57
[illegible]	-1.55	-1.23	-0.66	-0.34	-0.68	-0.39
[illegible]	-1.30	1.47	-0.92	1.32	-0.85	-1.04
[illegible]	0.09	-1.66	-0.02	-0.19	0.85	0.06
[illegible]	-0.26	-0.98	-0.40	0.98	-0.42	0.33
[illegible]	-0.88	-0.77	-0.28	0.58	-0.14	0.95
[illegible]	-0.56	-0.95	-0.65	0.57	-0.36	0.15
[illegible]	-0.43	-1.23	-0.19	-0.12	0.19	1.00
[illegible]	-0.02	-0.59	-0.80	0.32	-0.58	-0.13
[illegible]	-0.40	0.56	-0.70	-0.20	-0.64	-0.25
[illegible]	1.27	-0.76	0.01	0.43	0.24	1.50
[illegible]	-0.09	-0.60	-0.31	0.59	-0.53	-0.67
-0.37	-0.95	0.13	-0.81	-0.53	-0.67	-0.59
0.20	0.74	-0.62	-0.20	-0.12	-0.23	-0.15
-1.13	2.98	-1.21	2.56	-0.98	3.28	-0.66
0.73	1.44	0.12	-0.59	-0.60	-0.37	-0.80
2.52	2.98	-0.12	0.82	-0.93	1.38	0.71
1.27	2.87	-0.54	0.45	-1.05	1.52	0.50
-1.15	1.90	-1.03	2.24	-1.00	2.50	-0.58
0.27	-0.78	1.15	-0.81	-0.46	-0.65	-1.46
-0.30	0.82	0.18	-0.46	-0.19	-0.61	-1.29
-0.57	1.59	0.18	0.15	-0.88	0.33	-1.08
0.49	1.66	0.24	2.44	0.38	-0.48	1.76
-0.81	-0.52	-0.27	-0.19	-0.97	0.16	-0.36
-2.17	-1.08	-1.02	0.05	-1.25	-0.29	2.03
5.34	5.24	6.59	0.77	-0.76	0.44	3.48
5.55	2.97	1.48	0.55	-0.70	1.84	3.75
-0.14	0.67	-0.02	-0.36	-0.12	-0.26	0.11
-1.03	2.51	-2.12	4.99	-1.16	0.45	-0.96
-0.74	1.06	0.21	0.60	-0.39	0.05	-0.55
1.01	0.47	0.87	1.39	-0.22	1.04	1.06
-1.12	-1.64	8.15	-0.97	2.03	-0.95	0.14
-0.36	2.20	-0.75	1.68	0.50	0.76	0.57
-1.54	-0.62	-1.54	-0.07	-1.14	0.23	0.84
-1.86	-1.17	-0.96	0.51	-1.03	-0.42	-0.12
0.57	0.70	-0.74	0.61	-0.80	2.07	2.68
0.66	-0.03	-1.32	0.28	-0.51	0.97	0.10
0.67	-1.44	0.20	-0.77	-0.34	-0.71	-0.84
-0.73	1.86	-0.79	0.32	-0.36	1.01	-0.11
1.28	-1.43	-1.22	-0.69	1.88	-0.73	-0.49
1.16	0.48	-1.32	2.11	-0.29	1.79	1.38
-0.74	1.91	-1.20	4.03	-0.87	3.05	0.40
0.70	-1.26	-1.22	-0.64	-0.04	-0.34	0.02
1.07	0.76	-1.02	-0.06	0.02	1.11	0.41

-0.31	0.21	-0.66	-0.54	0.00	-0.11	-0.34
-0.50	0.31	-1.48	1.49	-0.77	1.81	1.72
-0.30	-0.10	-1.02	-0.46	-0.15	-0.06	0.17
-0.11	7.32	-0.62	5.96	-0.34	1.52	1.07
-1.93	-0.21	-1.42	-0.58	-0.57	-0.32	-0.30
1.81	0.37	0.27	-0.65	1.13	-0.64	-0.31
-1.52	0.28	-1.02	0.08	-0.98	0.47	-0.82
0	1	0	-1	-1	0	-1

12	13	14	15	16	17	18
24	9	56	126	116	88	68
TGTCAGTTTGTCA	AAGCTGCCAGTTG	TACAGTACTGTG	ACTCTTTCCCTGT	TGAGGTAGTAGGT	TCGTACCGTGAGT	TAAAGTGCTTATA
miR-223-3p	miR-22-3p	miR-101-3p	miR-130b-5p	let-7c	miR-126-3p	miR-20a-5p
624773	3462453	134502	12970	18147	41318	79678
-0.15	-0.38	-0.13	0.32	0.13	-0.88	-0.66
0.42	-0.94	-0.38	-0.98	1.32	0.03	0.38
-0.05	1.75	-0.16	-0.91	0.55	0.30	1.06
-0.48	0.03	-0.22	-0.74	-0.11	-0.25	-0.16
0.78	-1.00	-0.32	2.95	-0.41	0.27	-0.02
-0.90	-0.66	-0.29	2.28	-0.54	-0.83	-0.80
-0.98	-0.75	-0.22	0.25	0.83	-0.81	-0.51
-0.87	-0.89	-0.32	0.67	-1.01	-0.77	-0.90
-0.70	-0.03	-0.34	-0.85	-0.58	-0.53	-0.74
-0.59	-0.35	-0.23	-0.46	-1.00	-0.30	-0.23
1.52	0.02	-0.21	-1.02	-1.11	0.61	-0.26
-0.97	-0.70	-0.33	0.63	1.17	-0.72	-0.83
0.75	1.69	0.81	0.71	-0.27	2.36	1.81
-0.78	-0.88	-0.34	-0.38	-0.49	-0.75	-0.77
-0.41	0.78	-0.24	-0.87	-0.64	-0.18	0.29
0.24	2.19	-0.03	0.33	-0.41	1.49	0.61
-0.62	-0.47	-0.32	-0.35	0.37	-0.87	-0.49
0.30	1.23	0.04	-0.63	-0.71	1.63	-0.06
3.13	-0.17	-0.23	-0.86	-1.29	0.09	-0.43
1.28	1.54	4.85	-0.56	-1.18	2.28	2.88
-0.89	-0.32	-0.19	-0.09	-0.27	-0.48	-0.40
-0.38	-0.77	-0.32	0.59	2.87	-0.73	-0.67
-0.41	-0.60	-0.25	1.14	0.97	-0.59	-0.40
-0.98	-0.70	-0.25	0.00	0.48	-0.85	-0.62
1.32	-1.02	-0.34	0.67	1.76	-0.61	-0.47
0.96	-0.24	-0.32	-0.92	0.32	-0.45	-0.20
-0.54	1.65	0.27	-0.89	-0.76	1.54	2.60
1.00	2.15	6.29	-1.01	-0.32	6.13	5.46
0.89	-0.08	-0.05	-0.43	-0.66	0.34	-0.23
-0.17	-0.41	-0.23	-1.36	-1.47	-0.58	-0.61
-0.97	-0.94	-0.33	-0.91	-0.70	-0.77	-0.48
0.21	-0.57	-0.07	-1.03	-0.95	0.10	1.46
4.39	-0.83	-0.33	-0.14	-0.26	1.59	1.67
0.45	-0.67	-0.30	-0.47	-0.59	-0.63	-0.58
0.49	-0.71	-0.26	-0.51	-0.64	-0.69	-0.53
0.23	-0.72	-0.29	-0.47	4.97	-0.64	-0.71
-1.10	-1.04	-0.36	-0.88	-0.38	-0.94	-1.00
[illegible]	-0.37	0.13	-0.06	-0.49	-0.47	-0.30
[illegible]	0.49	1.06	-0.59	-0.98	1.74	1.38

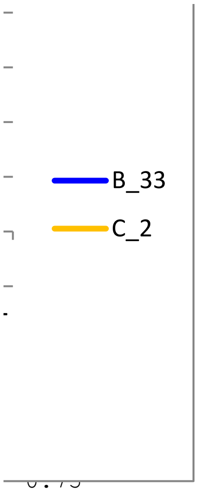

[illegible]	-0.49	-0.31	-0.26	-0.36	-0.43	-0.67
[illegible]	-0.78	-0.23	0.28	-0.91	-0.91	-0.49
[illegible]	-0.25	-0.17	3.76	0.59	-0.83	-0.65
[illegible]	-0.55	-0.31	0.09	-0.01	-0.55	-0.54
[illegible]	0.53	0.25	-0.16	-1.50	-0.31	0.34
[illegible]	-0.49	-0.35	-0.12	-0.69	-0.55	-0.85
[illegible]	-0.78	-0.31	0.05	-1.73	-0.85	-0.71
[illegible]	0.93	-0.21	-0.74	-1.07	0.54	-0.14
[illegible]	-0.12	-0.26	0.28	0.38	-0.51	-0.67
[illegible]	1.04	-0.16	0.20	-1.24	0.89	-0.10
[illegible]	0.06	-0.26	0.02	-1.23	-0.39	-0.49
[illegible]	1.55	-0.14	-0.70	-1.00	0.88	0.16
[illegible]	-0.64	-0.30	0.05	1.16	-0.79	-0.63
[illegible]	-0.61	-0.30	0.25	0.25	-0.76	-0.36
[illegible]	0.51	-0.24	0.75	0.59	-0.41	-0.02
[illegible]	-0.48	-0.30	1.40	0.36	-0.67	-0.77
-0.57	-0.56	-0.32	-0.63	0.09	-0.67	-0.66
-0.70	0.43	-0.25	-0.12	0.43	-0.24	-0.43
0.91	2.70	8.65	-1.02	-1.15	5.73	7.69
0.76	-0.20	-0.33	-0.35	1.64	-0.47	-0.52
1.94	0.08	-0.25	1.20	1.58	0.88	0.44
2.97	-0.48	-0.31	0.50	0.48	1.32	0.99
0.25	2.45	5.85	-1.07	-1.27	3.73	5.62
-0.26	-0.65	-0.27	-0.71	-1.04	-0.62	-0.51
-0.72	-0.10	-0.27	0.21	0.07	-0.53	-0.33
-0.19	2.74	-0.24	-1.06	-0.19	1.12	0.64
-0.59	-0.41	0.58	-1.13	0.78	0.60	0.84
0.24	3.23	0.08	-1.09	-1.28	1.95	0.43
0.13	-0.96	-0.23	-0.61	1.60	-0.81	-0.31
-0.34	-0.62	-0.31	1.45	2.96	0.93	1.20
1.83	0.07	-0.20	1.66	1.34	1.66	1.19
-0.39	-0.29	-0.19	-0.12	0.14	-0.30	-0.23
-0.62	-0.89	0.85	-0.88	-1.78	0.94	-0.22
-0.71	0.38	-0.24	-0.39	-0.83	0.36	-0.51
0.40	0.67	-0.01	-0.34	-0.55	1.87	0.78
-0.94	-0.92	-0.31	0.58	0.72	-0.79	-1.01
-0.51	1.58	0.02	-0.26	0.14	0.91	0.53
0.60	-0.43	-0.26	-1.17	-1.07	-0.30	-0.07
-0.58	-0.31	-0.27	-0.90	0.64	-0.09	-0.78
1.76	1.09	0.17	-1.14	-0.32	0.82	2.54
1.95	1.73	-0.28	-0.68	-0.94	1.13	-0.08
-0.30	-0.20	-0.31	-0.53	-0.94	-0.63	-0.54
0.64	0.43	-0.24	-0.58	-0.83	-0.13	-0.33
-0.79	-0.39	-0.22	0.51	-1.38	-0.58	-0.64
1.97	1.80	7.00	-0.62	-0.40	2.69	2.21
0.19	1.25	9.36	-1.18	-1.35	3.05	6.90
0.30	0.20	-0.28	-0.69	-1.14	0.24	-0.52
0.48	0.53	-0.25	-0.66	-0.52	0.21	-0.29
-0.40	-0.02	-0.26	-0.46	-0.22	-0.39	-0.51
0.80	0.74	-0.08	-0.73	-0.82	0.31	0.37
1.24	0.33	-0.30	0.01	-0.66	0.03	-0.38
1.03	0.04	0.32	-0.56	0.39	0.75	2.20
-0.79	-0.71	-0.28	-0.60	-1.22	-0.84	-0.71
-0.55	-0.44	-0.28	0.77	1.12	-0.76	-0.50
0.23	0.40	-0.26	-1.06	-0.68	-0.20	0.19
0.15	0.47	-0.04	-0.96	-0.05	-0.18	-0.39
0.04	0.04	0.04	0.04	0.05	0.05	0.05
0.04	0.03	0.07	0.04	0.04	0.04	0.05

1734052	139	1026638	1873959	1687678	1238990	121924
	1					
	1					
22	23	24	25	26	27	28
22	23	24	25	26	27	28
E_47	F_2	F_43	F_6	F_7	F_8	G_33
UC	UC	UC	UC	UC	UC	UC
3420100	899	2100117	3630351	3796767	3406036	987631
0.07	0.99	0.29	0.32	0.18	0.68	0.60
E_47	F_2	F_43	F_6	F_7	F_8	G_33
1686048	760	1073479	1756392	2109089	2167046	865707
393385	83	494991	687405	642121	571738	12006
82970	8	54523	121143	109242	54591	45542
75041	3	41641	212423	151317	99440	5799
67885	8	69737	154534	153536	103282	20023
98228	14	76677	195430	169720	125410	9164
221090	3	27687	9242	14625	9248	1222
103902	3	23181	55361	45205	18631	5116
125143	1	2006	1948	4365	1630	204
70550	1	13874	11730	15125	9109	290
32415	1	27676	20299	18237	9862	1294
14010	2	27254	17062	20395	12986	343
33546	1	10282	34885	34820	21490	1422
20989	0	16434	22591	21334	10088	995
15421	3	11363	26166	30912	30356	1976
28940	1	6158	3952	4627	1461	351
13776	1	9179	39459	27487	24425	196
24133	0	3570	2432	2128	840	387
14832	1	6400	11801	11726	6357	363
15649	0	3023	5932	5481	2466	284
9752	0	7433	14364	12199	6199	886
9166	0	3726	12856	11905	5736	228
5987	0	4327	7234	7197	4049	254
9506	1	6108	7834	6704	2699	243
13140	0	808	4539	3930	615	962
12829	1	2190	1382	1354	842	226
9001	0	3253	10683	7900	4933	51
5532	0	4049	10769	13171	13012	478
10349	0	4272	2890	2807	1132	133
7445	0	4213	4390	5071	2609	428
3536	0	5401	9991	7926	4721	281
9235	1	1715	5461	4259	1824	284
4999	0	2298	13143	10960	5985	808
13028	0	904	1640	1939	883	106
12790	0	847	581	1385	711	69
6853	0	3974	4171	4801	2471	402
2996	0	1863	3709	5120	4332	815
4826	0	2779	2742	3385	1427	470
3812	0	2200	1180	1672	977	290
2967	0	1642	3254	3569	2541	1069
3747	0	695	18645	5740	3502	485
2650	0	913	8974	6310	3723	756
2571	0	2464	5373	4777	4227	174
3028	0	1463	3363	2935	2596	385
6940	0	483	243	482	260	57
2855	0	645	3004	2416	1529	145

2658	0	1824	4100	3480	2045	123
2161	0	1155	2707	2062	1123	106
2225	0	1273	6038	3801	3568	67
7803	0	253	106	240	137	12
8507	0	407	746	1137	265	6
269	0	569	3158	1497	832	253
1870	0	521	1146	952	446	176
1176	0	861	1629	2190	1899	152
3091	0	932	1386	1082	370	36
2787	1	324	2003	2149	1287	1
6612	0	152	85	164	125	4
1483	0	484	855	997	559	129
2234	0	308	2417	1667	759	58
1143	0	1220	1339	1498	1161	47
686	0	600	3727	2354	959	559
901	0	674	1470	1318	736	103
1876	0	344	1493	1073	664	17
2445	0	104	37	57	30	16
5997	0	46	32	67	60	7
925	0	2043	1434	1742	1730	86
1480	0	376	360	531	286	41
2074	0	215	280	427	254	13
2037	0	207	207	330	159	21
1577	0	250	619	510	345	26
541	0	598	288	253	116	29
582	0	500	1329	1153	732	101
610	0	884	1378	1418	1478	45
1348	0	248	95	173	47	32
977	0	430	704	573	316	8
1216	0	249	567	509	180	41
175	1	263	2726	1012	608	98
784	0	264	1000	895	584	4
727	0	303	971	574	332	189
460	0	304	1677	1299	918	69
296	0	382	505	755	528	46
805	0	258	390	485	234	25
578	0	409	1436	1034	977	9
420	0	544	805	949	668	115
869	0	194	216	410	282	3
1038	0	100	263	236	156	37
443	0	154	966	716	481	10
343	0	538	829	798	807	49
1028	0	104	96	131	37	9
664	0	304	356	267	135	28
240	0	243	1704	805	366	137
518	0	448	970	706	377	20
1448	0	37	2	13	18	2
648	0	103	522	558	250	7
391	0	176	266	388	117	12
778	0	64	41	65	40	4
702	0	130	149	205	119	11
242	0	335	675	598	441	40
291	0	207	608	577	345	29
216	0	255	663	658	404	31
539	0	81	29	59	28	5
185	0	47	246	273	182	9
375	0	195	362	426	145	17
183	0	247	542	557	522	18
250	0	84	301	261	72	48

370	0	136	40	96	82	4
529	0	42	37	85	34	4
215	0	87	392	311	191	39
349	0	128	173	212	132	69
429	0	73	65	120	102	8
575	0	49	159	160	87	8
503	0	117	202	172	61	14
207	0	145	487	518	270	16
131	0	36	235	202	71	21
374	0	70	58	100	56	5
234	0	43	582	305	196	10
109	0	136	658	368	230	33
307	0	63	200	219	67	7
275	0	92	156	288	108	16
275	0	92	156	288	108	16
129	0	43	126	154	69	28
137	0	75	185	207	190	1
126	0	114	226	155	150	0
85	0	13	311	131	51	2
295	0	40	238	166	62	4
698	0	11	6	18	10	0
132	0	118	299	338	149	20
94	0	71	143	146	129	20
123	0	85	289	282	195	0
112	0	42	243	226	167	56
75	0	44	346	357	149	84
404	0	53	74	51	31	1
174	0	41	261	119	33	7
106	0	62	131	150	58	9
95	0	43	165	141	92	7
209	0	23	49	48	42	1
88	0	78	153	130	81	1
169	0	77	128	119	79	0
131	0	59	123	172	110	3
169	0	41	131	135	77	3
119	0	41	172	102	101	10
212	0	41	165	134	106	2
111	0	43	96	94	70	7
77	0	12	340	174	136	12
154	0	32	128	106	68	15
256	0	12	1	15	6	3
66	0	99	92	117	112	5
89	0	39	202	85	89	4
225	0	35	90	44	21	10
74	0	77	58	61	48	13
67	0	34	84	89	75	9
192	0	40	60	44	21	6
150	0	28	21	41	26	3
71	0	18	135	67	22	0
96	0	24	166	104	53	6
151	0	6	286	241	137	0
46	0	83	55	80	100	13
36	0	28	101	122	88	51
33	0	42	133	67	80	5
70	0	40	72	83	86	1
101	0	23	110	149	140	2
337	0	9	5	9	5	0
34	0	14	90	79	30	2
32	0	46	139	64	101	20

124	0	22	38	34	10	1
87	0	25	42	33	13	3
75	0	62	96	80	78	3
54	0	31	66	60	69	6
53	0	26	78	96	102	3
259	0	4	14	15	9	1
44	0	36	57	67	43	1
62	0	19	72	83	38	6
47	0	73	62	43	26	2
33	0	62	103	95	69	2
85	0	29	49	35	26	1
247	0	6	32	26	5	2
56	0	30	139	79	96	25
4	0	20	56	51	50	9
100	0	39	43	54	34	7
52	0	9	57	42	28	1
7	0	16	140	75	53	4
52	0	42	56	93	40	0
101	0	17	13	17	6	2
9	0	23	90	75	61	5
134	0	13	15	22	10	3
21	0	71	84	85	120	3
55	0	20	93	103	70	9
96	0	16	12	17	6	2
6	0	18	140	50	18	4
41	0	21	88	103	47	1
79	0	28	44	50	20	3
10	0	1	5	0	14	0
67	0	11	0	6	3	1
54	0	25	54	51	21	1
31	0	25	148	74	40	15
34	0	42	41	33	19	0
72	0	16	96	35	36	7
330	0	4	30	20	3	0
58	0	29	60	82	53	2
36	0	10	38	60	29	1
29	0	23	35	56	57	1
55	0	9	156	87	39	10
27	0	43	19	52	13	1
47	0	8	92	85	37	9
42	0	29	61	43	30	3
52	0	4	4	13	15	0
8	0	12	138	43	41	0
33	0	17	57	59	30	1
39	0	11	65	47	20	4
25	0	15	173	65	49	2
65	0	22	53	48	48	5
39	0	14	98	70	43	7
30	0	10	52	22	12	3
36	0	14	28	13	9	3
22	0	7	46	45	33	4
61	0	20	32	44	29	1
38	0	17	49	34	15	3
48	0	19	42	34	20	1
42	0	10	58	25	15	0
96	0	0	0	2	1	0
30	0	4	34	28	9	3
108	0	5	6	5	8	4
16	0	22	15	36	30	2

41	0	7	12	24	20	3
16	0	6	24	37	16	6
3	0	7	58	17	10	0
28	0	17	45	42	54	5
19	0	16	60	64	55	3
28	0	10	39	47	45	0
24	0	13	50	36	15	9
19	0	10	14	26	18	0
45	0	10	9	8	6	0
34	0	24	35	52	30	4
85	0	2	2	0	0	1
19	0	8	38	44	28	1
35	0	11	17	23	9	0
28	0	18	21	31	20	0
22	0	6	33	31	15	0
30	0	0	10	6	1	1
25	0	7	19	11	6	0
24	0	6	26	24	27	1
4	0	10	24	19	9	1
14	0	6	13	14	2	0
57	0	1	1	3	2	1
35	0	1	25	23	9	3
35	0	1	25	23	9	3
3	0	7	65	17	23	4
21	0	9	38	41	32	1
49	0	5	9	19	13	3
4	0	5	59	22	9	0
48	0	6	19	11	7	0
21	0	4	35	24	10	1
8	0	17	6	9	7	0
27	0	3	14	14	6	0
29	0	4	21	35	51	0
27	0	2	12	7	2	0
17	0	13	15	10	8	1
37	0	5	57	44	33	0
14	0	5	12	21	14	0
61	0	0	1	0	0	1
22	0	8	12	13	7	0
23	0	11	9	8	3	2
13	0	8	71	46	25	0
7	0	15	15	13	20	2
21	0	10	8	8	3	2
7	0	10	39	28	7	2
17	0	7	39	41	35	8
10	0	3	16	7	9	0
11	0	11	19	20	24	1
73	0	1	0	0	0	0
16	0	8	41	33	43	0
14	0	4	14	14	22	5
11	0	3	17	13	16	0
3	0	3	34	17	13	0
29	0	7	17	19	7	1
36	0	0	1	2	0	1
12	0	3	18	13	7	1
18	0	3	8	4	0	0
0	0	0	0	0	0	0
15	0	14	38	31	22	4
11	0	17	14	4	3	3
8	0	3	12	15	23	5

2	0	1	32	17	9	1
5	0	12	26	16	7	0
4	0	4	48	15	7	0
11	0	4	32	21	7	0
4	0	14	30	24	23	4
10	0	10	17	13	9	1
14	0	3	8	3	0	0
4	0	3	36	7	4	0
14	0	3	2	1	0	0
34	0	3	0	6	0	0
6	0	5	12	4	1	0
6	0	5	33	25	25	2
21	0	5	7	18	5	0
12	0	0	41	49	36	0
3	0	3	39	11	12	2
34	0	3	8	6	3	1
10	0	10	19	14	14	2
18	0	0	10	7	4	0
12	0	6	16	9	5	0
4	0	0	10	3	0	0
0	0	5	43	20	7	0
14	0	0	16	23	12	0
17	0	0	16	29	15	0
2	0	7	6	7	15	0
11	0	5	8	7	1	0
14	0	1	42	16	14	2
10	0	9	31	10	36	0
6	0	8	26	23	29	0
4	0	2	20	4	6	1
13	0	3	3	4	4	0
6	0	11	21	11	4	2
33	0	2	1	3	1	0
19	0	5	0	1	0	0
8	0	2	12	11	7	0
10	0	4	3	3	1	0
5	0	5	10	7	5	0
8	0	2	31	5	7	0
8	0	2	12	8	10	0
21	0	3	12	8	5	0
12	0	2	14	20	14	0
14	0	2	5	4	1	0
9	0	2	11	18	8	0
0	0	5	27	16	10	0
5	0	3	13	15	5	1
3	0	0	15	6	3	0
0	0	3	15	10	1	2
12	0	6	12	14	15	0
9	0	8	6	13	7	1
3	0	10	5	4	11	0
5	0	3	16	9	3	3
2	0	7	23	12	16	1
25	0	1	4	1	1	0
7	0	3	21	17	9	0
18	0	3	2	2	2	1
10	0	2	11	5	3	0
9	0	3	12	12	7	0
4	0	3	14	13	5	0
29	0	4	1	0	1	0
6	0	4	5	9	7	0

7	0	9	13	11	4	0
7	0	5	16	12	8	0
8	0	6	23	14	4	0
6	0	2	7	4	6	3
3	0	5	15	11	10	0
7	0	2	26	20	5	0
5	0	2	5	11	17	0
6	0	3	1	2	0	0
6	0	1	6	4	3	0
8	0	2	4	10	4	0
7	0	3	11	7	4	0
21	0	0	10	7	2	0
7	0	5	4	14	1	2
17	0	0	0	1	0	1
7	0	3	6	3	2	0
4	0	0	1	1	5	1
4	0	1	2	4	0	0
2	0	5	5	6	2	0
12	0	0	0	0	1	0
3	0	0	5	3	3	0
10	0	5	5	1	1	0
9	0	2	5	9	2	0
4	0	6	6	5	3	0
7	0	10	6	3	4	0
4	0	6	4	7	2	1
4	0	3	10	10	10	0
13	0	3	1	4	0	0
19	0	1	0	0	1	0
5	0	1	13	7	1	0
3	0	0	2	4	2	0
2	0	6	3	3	3	0
6	0	0	8	2	3	0
4	0	0	3	4	6	0
3	0	6	17	7	4	1
9	0	0	0	1	2	0
2	0	2	15	5	2	1
7	0	2	2	7	2	0
3	0	3	5	8	4	0
2	0	4	6	4	3	1
3	0	4	11	5	11	1
2	0	0	8	3	3	0
2	0	2	6	2	1	0
0	0	0	14	4	1	0
4	0	3	8	4	1	1
5	0	2	5	8	2	0
5	0	0	2	5	0	0
6	0	1	3	4	1	0
6	0	3	5	5	9	0
9	0	0	0	3	1	0
1	0	1	0	0	2	0
2	0	0	9	1	5	1
3	0	2	7	3	2	1
14	0	0	1	1	1	0
3	0	1	5	3	2	0
10	0	2	4	1	2	1
3	0	0	2	3	2	1
6	0	1	6	0	1	0
2	0	1	6	6	5	0
10	0	5	20	5	0	0

5	0	1	2	1	0	0
0	0	2	4	6	2	0
7	0	1	1	0	0	0
3	0	1	0	1	0	1
3	0	1	3	6	2	0
1	0	3	15	4	7	0
4	0	4	6	8	3	0
8	0	0	2	0	0	0
4	0	0	6	10	2	0
3	0	1	9	5	2	0
2	0	0	0	0	0	0
16	0	0	0	0	0	0
14	0	0	0	0	0	0
7	0	0	2	4	1	0
10	0	1	6	3	5	0
0	0	1	11	3	1	0
2	0	3	1	1	6	0
2	0	1	1	5	4	2
16	0	0	0	1	0	0
3	0	1	0	0	0	0
2	0	0	3	2	1	0
8	0	0	0	1	0	0
0	0	1	12	2	2	1
4	0	2	9	7	3	0
5	0	0	5	3	9	0
18	0	0	0	1	0	0
10	0	1	5	5	6	0
3	0	2	0	0	0	0
4	0	2	4	3	1	0
6	0	1	2	1	0	0
10	0	0	0	0	2	0
5	0	2	5	8	0	0
2	0	2	5	6	3	0
5	0	0	14	5	3	0
7	0	1	2	0	1	0
3	0	1	2	4	5	0
6	0	1	4	2	2	0
3	0	1	7	5	4	0
7	0	1	5	7	0	0
0	0	0	7	1	1	0
6	0	1	0	1	0	0
1	0	1	2	4	1	1
4	0	2	6	2	3	0
2	0	0	2	4	3	0
2	0	0	1	2	1	0
1	0	1	1	0	1	0
6	0	1	0	1	0	0
3	0	1	2	0	0	0
0	0	2	2	2	3	0
2	0	2	2	2	2	1
3	0	2	2	3	1	1
0	0	0	0	0	0	0
2	0	1	1	7	2	0
2	0	0	1	2	0	0
3	0	0	3	4	1	0
4	0	0	2	1	3	1
0	0	1	2	3	3	0
2	0	1	3	0	3	0
0	0	2	4	5	6	0

4	0	3	1	5	3	0
1	0	1	5	4	6	0
2	0	2	3	3	2	0
1	0	2	2	1	1	1
3	0	2	1	5	1	0
6	0	0	0	0	0	0
1	0	1	3	1	6	0
3	0	1	0	1	0	0
0	0	0	7	4	1	0
1	0	1	1	0	1	0
1	0	0	0	1	1	0
4	0	0	4	0	0	0
4	0	1	0	1	1	0
2	0	0	1	2	6	0
6	0	0	1	1	0	0
2	0	0	0	0	1	0
4	0	0	1	3	2	2
1	0	0	1	0	0	0
6	0	0	0	1	0	0
1	0	0	1	3	1	0
3	0	1	0	1	1	0
3	0	1	0	0	0	0
6	0	0	0	1	0	0
1	0	1	2	1	3	0
1	0	0	4	7	4	0
1	0	1	2	1	1	0
2	0	0	11	3	3	0
0	0	2	5	2	4	0
4	0	0	0	0	0	0
4	0	0	0	1	0	0
0	0	0	0	0	0	0
1	0	0	4	4	0	0
4	0	0	3	0	2	0
0	0	1	5	1	0	1
1	0	0	0	1	4	0
1	0	0	0	3	3	0
2	0	0	2	5	1	0
4	0	0	1	2	0	0
3	0	0	1	1	2	0
1	0	1	4	2	5	0
1	0	0	1	0	0	0
2	0	0	4	0	4	0
2	0	1	5	4	3	0
1	0	0	4	3	3	0
0	0	0	6	4	1	0
2	0	1	2	2	2	0
1	0	0	9	3	1	0
1	0	0	0	1	1	0
2	0	3	3	4	4	0
2	0	1	0	1	2	0
1	0	0	7	0	1	0
3	0	1	0	1	0	0
1	0	1	0	1	0	0
2	0	0	0	2	2	0
0	0	0	0	1	0	0
1	0	0	0	2	0	0
1	0	0	2	0	1	0
1	0	0	3	2	1	1
2	0	0	1	3	2	0

1	0	1	0	0	0	0
2	0	1	2	4	1	0
2	0	0	4	0	0	0
2	0	0	0	1	1	0
3	0	1	1	0	0	0
0	0	0	2	4	4	0
1	0	0	4	4	2	0
0	0	0	0	1	2	0
0	0	0	2	2	0	0
0	0	0	0	0	0	0
1	0	0	1	2	2	0
0	0	2	2	0	2	0
0	29	0	0	0	0	1
E_47	F_2	F_43	F_6	F_7	F_8	G_33
UC	UC	UC	UC	UC	UC	UC
1686048	760	1073479	1756392	2109089	2167046	865707
16	26	16	16	18	20	27
-1.09	1.04	-1.07	-1.20	-0.74	-0.24	1.29

0.03	0.57	0.03	0.27	0.57	0.82	0.56
28	15	29	19	14	6	17
1	1	1	1	1	1	1
22	23	24	25	26	27	28
22	23	24	25	26	27	1
F_6	F_7	F_8	G_33	G_34	G_8	A_1
UC	UC	UC	UC	UC	UC	CHR_NP
1873959	1687678	1238990	121924	416015	1782397	2570804
F_6	F_7	F_8	G_33	G_34	G_8	A_1
687405	642121	571738	12006	84942	490594	524718
121143	109242	54591	45542	77391	190767	57861
212423	151317	99440	5799	40120	56160	19787
154534	153536	103282	20023	36859	144853	154427
195430	169720	125410	9164	48028	62452	40902
9242	14625	9248	1222	6252	159965	429461
55361	45205	18631	5116	25442	105646	129967
1948	4365	1630	204	877	48162	239532
11730	15125	9109	290	6027	77934	125922
20299	18237	9862	1294	7638	64617	89917
17062	20395	12986	343	4804	24819	40910
34885	34820	21490	1422	6898	26015	27652
22591	21334	10088	995	4774	22981	47820
26166	30912	30356	1976	5644	19273	22471
3952	4627	1461	351	754	16947	76438
39459	27487	24425	196	1146	3916	7077
2432	2128	840	387	1626	17363	32560
11801	11726	6357	363	1789	7192	16561
5932	5481	2466	284	2298	19340	32924

14364	12199	6199	886	2965	10682	11662
12856	11905	5736	228	1450	3881	5082
7234	7197	4049	254	2096	10495	10374
7834	6704	2699	243	1725	11779	12831
4539	3930	615	962	2819	3401	16942
1382	1354	842	226	1591	6662	30968
10683	7900	4933	51	1149	5975	7914
10769	13171	13012	478	2192	5029	3956
2890	2807	1132	133	420	17452	20362
4390	5071	2609	428	1155	5432	14099
9991	7926	4721	281	2163	7464	4525
5461	4259	1824	284	1365	7197	4668
13143	10960	5985	808	2076	6021	7107
1640	1939	883	106	796	7148	25687
581	1385	711	69	307	2763	39709
4171	4801	2471	402	1062	4958	9505
3709	5120	4332	815	1773	5418	3867
2742	3385	1427	470	882	7719	10370
1180	1672	977	290	1205	4931	11825
3254	3569	2541	1069	1744	3394	3680
18645	5740	3502	485	997	1500	2655
8974	6310	3723	756	448	875	1553
5373	4777	4227	174	1375	1340	1182
3363	2935	2596	385	780	2474	2973
243	482	260	57	123	5969	15267
3004	2416	1529	145	1351	1744	2875
4100	3480	2045	123	672	2079	2339
2707	2062	1123	106	1033	3511	4959
6038	3801	3568	67	491	1780	1469
106	240	137	12	52	1090	16994
746	1137	265	6	151	1755	8702
3158	1497	832	253	739	2748	1046
1146	952	446	176	593	1203	1882
1629	2190	1899	152	574	1525	951
1386	1082	370	36	175	1301	3760
2003	2149	1287	1	212	3705	2971
85	164	125	4	19	870	12278
855	997	559	129	488	1698	2601
2417	1667	759	58	405	1378	1454
1339	1498	1161	47	690	766	1205
3727	2354	959	559	775	1354	724
1470	1318	736	103	529	1009	1246
1493	1073	664	17	296	895	966
37	57	30	16	38	2086	19396
32	67	60	7	23	397	13240
1434	1742	1730	86	61	1002	688
360	531	286	41	236	1815	3256
280	427	254	13	185	1317	1778
207	330	159	21	107	2009	4922
619	510	345	26	206	370	890
288	253	116	29	124	2669	2210
1329	1153	732	101	246	370	253
1378	1418	1478	45	93	305	737
95	173	47	32	38	1552	6757
704	573	316	8	80	836	1240
567	509	180	41	334	596	1151
2726	1012	608	98	229	1033	629
1000	895	584	4	181	1372	714
971	574	332	189	257	1310	364

1677	1299	918	69	311	451	322
505	755	528	46	250	283	255
390	485	234	25	108	631	1758
1436	1034	977	9	138	280	277
805	949	668	115	90	500	497
216	410	282	3	58	184	1045
263	236	156	37	226	1087	1734
966	716	481	10	103	377	318
829	798	807	49	135	291	282
96	131	37	9	43	849	3254
356	267	135	28	66	324	480
1704	805	366	137	212	402	518
970	706	377	20	79	184	201
2	13	18	2	1	171	4097
522	558	250	7	81	155	313
266	388	117	12	68	786	703
41	65	40	4	24	333	1680
149	205	119	11	32	196	801
675	598	441	40	71	196	115
608	577	345	29	68	256	155
663	658	404	31	208	205	151
29	59	28	5	21	495	1066
246	273	182	9	77	250	225
362	426	145	17	83	371	266
542	557	522	18	183	123	200
301	261	72	48	91	577	1037
40	96	82	4	25	560	2176
37	85	34	4	18	438	1580
392	311	191	39	115	83	110
173	212	132	69	139	251	184
65	120	102	8	39	301	1390
159	160	87	8	58	408	709
202	172	61	14	42	165	387
487	518	270	16	72	118	124
235	202	71	21	160	181	128
58	100	56	5	33	484	1041
582	305	196	10	126	69	212
658	368	230	33	71	169	308
200	219	67	7	41	215	908
156	288	108	16	31	209	577
156	288	108	16	31	209	577
126	154	69	28	97	171	197
185	207	190	1	52	102	133
226	155	150	0	28	48	101
311	131	51	2	44	664	466
238	166	62	4	16	144	278
6	18	10	0	15	111	976
299	338	149	20	22	98	114
143	146	129	20	106	99	122
289	282	195	0	29	126	108
243	226	167	56	21	71	99
346	357	149	84	42	123	70
74	51	31	1	7	334	83
261	119	33	7	32	148	81
131	150	58	9	40	245	382
165	141	92	7	26	32	78
49	48	42	1	14	154	604
153	130	81	1	53	105	93
128	119	79	0	18	133	114

123	172	110	3	21	122	247
131	135	77	3	18	126	289
172	102	101	10	33	76	52
165	134	106	2	25	126	166
96	94	70	7	10	61	51
340	174	136	12	24	80	39
128	106	68	15	47	90	193
1	15	6	3	13	68	632
92	117	112	5	10	93	55
202	85	89	4	19	31	44
90	44	21	10	17	74	131
58	61	48	13	17	102	76
84	89	75	9	31	71	68
60	44	21	6	14	34	105
21	41	26	3	14	155	207
135	67	22	0	8	220	314
166	104	53	6	21	43	59
286	241	137	0	9	8	27
55	80	100	13	21	59	57
101	122	88	51	46	119	72
133	67	80	5	28	20	29
72	83	86	1	28	28	43
110	149	140	2	4	76	166
5	9	5	0	2	37	646
90	79	30	2	34	31	22
139	64	101	20	12	79	11
38	34	10	1	17	71	217
42	33	13	3	31	51	79
96	80	78	3	14	54	63
66	60	69	6	31	35	30
78	96	102	3	6	13	26
14	15	9	1	8	67	337
57	67	43	1	4	65	109
72	83	38	6	14	16	20
62	43	26	2	10	92	173
103	95	69	2	24	60	44
49	35	26	1	16	48	31
32	26	5	2	8	71	268
139	79	96	25	15	45	25
56	51	50	9	11	48	35
43	54	34	7	14	60	99
57	42	28	1	10	16	35
140	75	53	4	31	47	24
56	93	40	0	17	102	62
13	17	6	2	6	103	217
90	75	61	5	17	52	24
15	22	10	3	6	88	141
84	85	120	3	10	32	27
93	103	70	9	16	30	37
12	17	6	2	6	95	201
140	50	18	4	10	36	36
88	103	47	1	30	43	35
44	50	20	3	16	73	42
5	0	14	0	85	59	17
0	6	3	1	3	103	90
54	51	21	1	13	36	72
148	74	40	15	8	29	16
41	33	19	0	6	103	46
96	35	36	7	34	41	70

30	20	3	0	1	28	199
60	82	53	2	6	73	60
38	60	29	1	7	28	24
35	56	57	1	6	63	140
156	87	39	10	16	49	33
19	52	13	1	9	35	24
92	85	37	9	17	43	25
61	43	30	3	7	41	16
4	13	15	0	6	80	194
138	43	41	0	6	67	36
57	59	30	1	10	41	36
65	47	20	4	17	47	17
173	65	49	2	32	54	141
53	48	48	5	12	44	48
98	70	43	7	11	10	30
52	22	12	3	9	23	16
28	13	9	3	2	131	221
46	45	33	4	11	41	12
32	44	29	1	2	70	31
49	34	15	3	7	40	48
42	34	20	1	12	49	53
58	25	15	0	5	39	44
0	2	1	0	0	14	288
34	28	9	3	11	52	53
6	5	8	4	6	56	84
15	36	30	2	9	36	26
12	24	20	3	8	14	54
24	37	16	6	11	12	20
58	17	10	0	7	104	50
45	42	54	5	14	8	8
60	64	55	3	11	18	19
39	47	45	0	5	16	27
50	36	15	9	5	14	22
14	26	18	0	6	8	40
9	8	6	0	8	26	171
35	52	30	4	4	24	14
2	0	0	1	0	22	223
38	44	28	1	4	16	21
17	23	9	0	1	31	72
21	31	20	0	5	29	22
33	31	15	0	5	12	16
10	6	1	1	8	17	41
19	11	6	0	5	38	86
26	24	27	1	5	19	16
24	19	9	1	2	29	17
13	14	2	0	4	10	19
1	3	2	1	4	21	92
25	23	9	3	4	22	31
25	23	9	3	4	22	31
65	17	23	4	4	12	10
38	41	32	1	1	8	11
9	19	13	3	8	30	27
59	22	9	0	9	30	21
19	11	7	0	7	12	15
35	24	10	1	6	15	17
6	9	7	0	3	4	17
14	14	6	0	4	22	33
21	35	51	0	8	9	15
12	7	2	0	4	12	29

15	10	8	1	31	4	7
57	44	33	0	4	11	22
12	21	14	0	4	6	24
1	0	0	1	0	9	149
12	13	7	0	7	12	13
9	8	3	2	3	29	47
71	46	25	0	8	7	9
15	13	20	2	1	19	13
8	8	3	2	3	22	42
39	28	7	2	6	11	12
39	41	35	8	2	22	9
16	7	9	0	2	34	31
19	20	24	1	6	15	6
0	0	0	0	0	4	113
41	33	43	0	6	8	14
14	14	22	5	2	6	9
17	13	16	0	1	3	27
34	17	13	0	2	10	11
17	19	7	1	4	30	13
1	2	0	1	0	9	39
18	13	7	1	15	14	17
8	4	0	0	1	9	14
0	0	0	0	0	0	3
38	31	22	4	3	15	13
14	4	3	3	0	8	13
12	15	23	5	6	12	11
32	17	9	1	10	16	9
26	16	7	0	4	10	3
48	15	7	0	4	24	18
32	21	7	0	1	15	8
30	24	23	4	2	4	8
17	13	9	1	7	10	11
8	3	0	0	0	8	11
36	7	4	0	6	10	9
2	1	0	0	0	78	58
0	6	0	0	0	10	72
12	4	1	0	3	28	20
33	25	25	2	5	10	5
7	18	5	0	3	19	31
41	49	36	0	7	1	3
39	11	12	2	5	5	12
8	6	3	1	2	3	39
19	14	14	2	4	19	20
10	7	4	0	7	10	12
16	9	5	0	1	4	11
10	3	0	0	1	36	15
43	20	7	0	2	8	5
16	23	12	0	4	2	15
16	29	15	0	3	3	0
6	7	15	0	4	13	4
8	7	1	0	1	7	13
42	16	14	2	4	2	3
31	10	36	0	1	2	2
26	23	29	0	2	3	7
20	4	6	1	6	5	5
3	4	4	0	8	7	10
21	11	4	2	4	8	3
1	3	1	0	1	14	52
0	1	0	0	0	20	56

12	11	7	0	1	19	7
3	3	1	0	3	19	26
10	7	5	0	1	5	4
31	5	7	0	4	7	1
12	8	10	0	1	2	5
12	8	5	0	0	2	0
14	20	14	0	1	3	11
5	4	1	0	2	5	71
11	18	8	0	3	3	3
27	16	10	0	2	13	5
13	15	5	1	1	4	6
15	6	3	0	2	13	13
15	10	1	2	0	1	0
12	14	15	0	1	5	8
6	13	7	1	1	2	14
5	4	11	0	2	4	4
16	9	3	3	2	7	6
23	12	16	1	0	13	4
4	1	1	0	1	5	33
21	17	9	0	1	3	2
2	2	2	1	0	14	28
11	5	3	0	1	8	5
12	12	7	0	1	1	0
14	13	5	0	0	2	4
1	0	1	0	0	22	4
5	9	7	0	2	9	1
13	11	4	0	2	5	11
16	12	8	0	1	2	2
23	14	4	0	0	2	1
7	4	6	3	1	1	6
15	11	10	0	2	5	0
26	20	5	0	4	5	3
5	11	17	0	2	7	2
1	2	0	0	1	23	61
6	4	3	0	0	9	8
4	10	4	0	0	3	3
11	7	4	0	2	3	5
10	7	2	0	2	5	5
4	14	1	2	1	0	9
0	1	0	1	1	10	23
6	3	2	0	1	0	7
1	1	5	1	2	9	11
2	4	0	0	3	5	13
5	6	2	0	0	5	5
0	0	1	0	2	8	26
5	3	3	0	1	8	7
5	1	1	0	1	1	8
5	9	2	0	1	2	8
6	5	3	0	4	3	5
6	3	4	0	1	2	1
4	7	2	1	0	8	10
10	10	10	0	5	1	2
1	4	0	0	0	4	23
0	0	1	0	1	4	12
13	7	1	0	3	2	0
2	4	2	0	0	0	2
3	3	3	0	0	4	2
8	2	3	0	2	3	4
3	4	6	0	1	1	8

17	7	4	1	0	1	2
0	1	2	0	0	6	14
15	5	2	1	1	6	3
2	7	2	0	0	4	1
5	8	4	0	0	1	5
6	4	3	1	2	1	3
11	5	11	1	1	1	4
8	3	3	0	2	1	2
6	2	1	0	1	1	3
14	4	1	0	2	4	6
8	4	1	1	0	2	8
5	8	2	0	4	3	1
2	5	0	0	5	7	4
3	4	1	0	1	1	2
5	5	9	0	0	3	1
0	3	1	0	0	6	5
0	0	2	0	0	0	0
9	1	5	1	2	2	5
7	3	2	1	1	5	8
1	1	1	0	0	6	9
5	3	2	0	1	1	2
4	1	2	1	1	2	5
2	3	2	1	0	13	10
6	0	1	0	0	0	2
6	6	5	0	1	4	4
20	5	0	0	2	0	2
2	1	0	0	0	4	5
4	6	2	0	0	0	2
1	0	0	0	0	0	6
0	1	0	1	1	0	12
3	6	2	0	3	4	4
15	4	7	0	0	2	2
6	8	3	0	0	0	0
2	0	0	0	1	3	13
6	10	2	0	1	2	0
9	5	2	0	1	3	3
0	0	0	0	0	0	24
0	0	0	0	0	1	18
0	0	0	0	0	1	17
2	4	1	0	0	8	2
6	3	5	0	0	4	2
11	3	1	0	1	1	0
1	1	6	0	0	1	3
1	5	4	2	1	4	2
0	1	0	0	0	2	40
0	0	0	0	0	3	6
3	2	1	0	0	2	4
0	1	0	0	0	1	18
12	2	2	1	1	5	3
9	7	3	0	0	0	0
5	3	9	0	0	3	1
0	1	0	0	0	2	10
5	5	6	0	0	0	10
0	0	0	0	0	1	2
4	3	1	0	0	0	1
2	1	0	0	0	1	4
0	0	2	0	0	1	18
5	8	0	0	0	6	4
5	6	3	0	1	2	1

14	5	3	0	0	1	2
2	0	1	0	2	0	8
2	4	5	0	1	1	0
4	2	2	0	0	0	4
7	5	4	0	0	4	3
5	7	0	0	1	3	8
7	1	1	0	0	4	4
0	1	0	0	0	8	41
2	4	1	1	3	1	3
6	2	3	0	2	2	0
2	4	3	0	0	3	3
1	2	1	0	0	1	0
1	0	1	0	0	5	8
0	1	0	0	0	9	11
2	0	0	0	1	2	0
2	2	3	0	0	2	0
2	2	2	1	0	1	2
2	3	1	1	1	4	4
0	0	0	0	0	3	7
1	7	2	0	1	5	2
1	2	0	0	0	1	6
3	4	1	0	0	2	2
2	1	3	1	0	1	2
2	3	3	0	2	4	2
3	0	3	0	1	4	0
4	5	6	0	1	5	0
1	5	3	0	0	0	1
5	4	6	0	1	1	1
3	3	2	0	0	1	2
2	1	1	1	0	0	1
1	5	1	0	0	7	2
0	0	0	0	0	2	6
3	1	6	0	0	0	2
0	1	0	0	0	2	17
7	4	1	0	1	0	1
1	0	1	0	0	1	8
0	1	1	0	0	0	6
4	0	0	0	0	8	2
0	1	1	0	0	3	3
1	2	6	0	0	0	3
1	1	0	0	0	4	7
0	0	1	0	0	1	3
1	3	2	2	1	0	0
1	0	0	0	0	10	2
0	1	0	0	0	2	12
1	3	1	0	1	0	0
0	1	1	0	0	0	1
0	0	0	0	0	2	13
0	1	0	0	0	3	14
2	1	3	0	1	0	0
4	7	4	0	1	0	0
2	1	1	0	0	2	2
11	3	3	0	1	0	0
5	2	4	0	0	1	1
0	0	0	0	0	3	12
0	1	0	0	1	0	10
0	0	0	0	0	3	6
4	4	0	0	0	1	1
3	0	2	0	0	3	3

5	1	0	1	1	0	0
0	1	4	0	0	2	0
0	3	3	0	0	2	3
2	5	1	0	0	1	2
1	2	0	0	0	1	4
1	1	2	0	0	3	0
4	2	5	0	1	1	1
1	0	0	0	0	3	5
4	0	4	0	0	3	0
5	4	3	0	0	0	1
4	3	3	0	1	3	0
6	4	1	0	1	0	0
2	2	2	0	0	1	2
9	3	1	0	1	2	1
0	1	1	0	0	3	1
3	4	4	0	1	2	1
0	1	2	0	0	2	0
7	0	1	0	0	0	1
0	1	0	0	0	1	11
0	1	0	0	0	0	2
0	2	2	0	0	0	9
0	1	0	0	0	2	1
0	2	0	0	1	0	2
2	0	1	0	0	1	1
3	2	1	1	0	0	0
1	3	2	0	0	0	5
0	0	0	0	0	0	2
2	4	1	0	0	0	2
4	0	0	0	0	0	1
0	1	1	0	0	4	4
1	0	0	0	0	0	1
2	4	4	0	0	0	3
4	4	2	0	1	2	1
0	1	2	0	1	0	0
2	2	0	0	0	1	0
0	0	0	0	0	1	0
1	2	2	0	1	2	0
2	0	2	0	0	3	4

0.03	0.57	0.03	0.27	0.57	0.82	0.56
28	15	29	19	14	6	17
1	1	1	1	1	1	1
22	23	24	25	26	27	28
22	23	24	25	26	27	1
F_6	F_7	F_8	G_33	G_34	G_8	A_1
UC	UC	UC	UC	UC	UC	CHR_NP
1873959	1687678	1238990	121924	416015	1782397	2570804
UC	UC	UC	UC	UC	UC	CHR_NP
687405	642121	571738	12006	84942	490594	524718
121143	109242	54591	45542	77391	190767	57861
212423	151317	99440	5799	40120	56160	19787
154534	153536	103282	20023	36859	144853	154427
195430	169720	125410	9164	48028	62452	40902

9242	14625	9248	1222	6252	159965	429461
55361	45205	18631	5116	25442	105646	129967
1948	4365	1630	204	877	48162	239532
11730	15125	9109	290	6027	77934	125922
20299	18237	9862	1294	7638	64617	89917
17062	20395	12986	343	4804	24819	40910
34885	34820	21490	1422	6898	26015	27652
22591	21334	10088	995	4774	22981	47820
26166	30912	30356	1976	5644	19273	22471
3952	4627	1461	351	754	16947	76438
39459	27487	24425	196	1146	3916	7077
2432	2128	840	387	1626	17363	32560
11801	11726	6357	363	1789	7192	16561
5932	5481	2466	284	2298	19340	32924
14364	12199	6199	886	2965	10682	11662
12856	11905	5736	228	1450	3881	5082
7234	7197	4049	254	2096	10495	10374
7834	6704	2699	243	1725	11779	12831
4539	3930	615	962	2819	3401	16942
1382	1354	842	226	1591	6662	30968
10683	7900	4933	51	1149	5975	7914
10769	13171	13012	478	2192	5029	3956
2890	2807	1132	133	420	17452	20362
4390	5071	2609	428	1155	5432	14099
9991	7926	4721	281	2163	7464	4525
5461	4259	1824	284	1365	7197	4668
13143	10960	5985	808	2076	6021	7107
1640	1939	883	106	796	7148	25687
581	1385	711	69	307	2763	39709
4171	4801	2471	402	1062	4958	9505
3709	5120	4332	815	1773	5418	3867
2742	3385	1427	470	882	7719	10370
1180	1672	977	290	1205	4931	11825
3254	3569	2541	1069	1744	3394	3680
18645	5740	3502	485	997	1500	2655
8974	6310	3723	756	448	875	1553
5373	4777	4227	174	1375	1340	1182
3363	2935	2596	385	780	2474	2973
243	482	260	57	123	5969	15267
3004	2416	1529	145	1351	1744	2875
4100	3480	2045	123	672	2079	2339
2707	2062	1123	106	1033	3511	4959
6038	3801	3568	67	491	1780	1469
106	240	137	12	52	1090	16994
746	1137	265	6	151	1755	8702
3158	1497	832	253	739	2748	1046
1146	952	446	176	593	1203	1882
1629	2190	1899	152	574	1525	951
1386	1082	370	36	175	1301	3760
2003	2149	1287	1	212	3705	2971
85	164	125	4	19	870	12278
855	997	559	129	488	1698	2601
2417	1667	759	58	405	1378	1454
1339	1498	1161	47	690	766	1205
3727	2354	959	559	775	1354	724
1470	1318	736	103	529	1009	1246
1493	1073	664	17	296	895	966
37	57	30	16	38	2086	19396
32	67	60	7	23	397	13240

1434	1742	1730	86	61	1002	688
360	531	286	41	236	1815	3256
280	427	254	13	185	1317	1778
207	330	159	21	107	2009	4922
619	510	345	26	206	370	890
288	253	116	29	124	2669	2210
1329	1153	732	101	246	370	253
1378	1418	1478	45	93	305	737
95	173	47	32	38	1552	6757
704	573	316	8	80	836	1240
567	509	180	41	334	596	1151
2726	1012	608	98	229	1033	629
1000	895	584	4	181	1372	714
971	574	332	189	257	1310	364
1677	1299	918	69	311	451	322
505	755	528	46	250	283	255
390	485	234	25	108	631	1758
1436	1034	977	9	138	280	277
805	949	668	115	90	500	497
216	410	282	3	58	184	1045
263	236	156	37	226	1087	1734
966	716	481	10	103	377	318
829	798	807	49	135	291	282
96	131	37	9	43	849	3254
356	267	135	28	66	324	480
1704	805	366	137	212	402	518
970	706	377	20	79	184	201
2	13	18	2	1	171	4097
522	558	250	7	81	155	313
266	388	117	12	68	786	703
41	65	40	4	24	333	1680
149	205	119	11	32	196	801
675	598	441	40	71	196	115
608	577	345	29	68	256	155
663	658	404	31	208	205	151
29	59	28	5	21	495	1066
246	273	182	9	77	250	225
362	426	145	17	83	371	266
542	557	522	18	183	123	200
301	261	72	48	91	577	1037
40	96	82	4	25	560	2176
37	85	34	4	18	438	1580
392	311	191	39	115	83	110
173	212	132	69	139	251	184
65	120	102	8	39	301	1390
159	160	87	8	58	408	709
202	172	61	14	42	165	387
487	518	270	16	72	118	124
235	202	71	21	160	181	128
58	100	56	5	33	484	1041
582	305	196	10	126	69	212
658	368	230	33	71	169	308
200	219	67	7	41	215	908
156	288	108	16	31	209	577
156	288	108	16	31	209	577
126	154	69	28	97	171	197
185	207	190	1	52	102	133
226	155	150	0	28	48	101
311	131	51	2	44	664	466

238	166	62	4	16	144	278
6	18	10	0	15	111	976
299	338	149	20	22	98	114
143	146	129	20	106	99	122
289	282	195	0	29	126	108
243	226	167	56	21	71	99
346	357	149	84	42	123	70
74	51	31	1	7	334	83
261	119	33	7	32	148	81
131	150	58	9	40	245	382
165	141	92	7	26	32	78
49	48	42	1	14	154	604
153	130	81	1	53	105	93
128	119	79	0	18	133	114
123	172	110	3	21	122	247
131	135	77	3	18	126	289
172	102	101	10	33	76	52
165	134	106	2	25	126	166
96	94	70	7	10	61	51
340	174	136	12	24	80	39
128	106	68	15	47	90	193
1	15	6	3	13	68	632
92	117	112	5	10	93	55
202	85	89	4	19	31	44
90	44	21	10	17	74	131
58	61	48	13	17	102	76
84	89	75	9	31	71	68
60	44	21	6	14	34	105
21	41	26	3	14	155	207
135	67	22	0	8	220	314
166	104	53	6	21	43	59
286	241	137	0	9	8	27
55	80	100	13	21	59	57
101	122	88	51	46	119	72
133	67	80	5	28	20	29
72	83	86	1	28	28	43
110	149	140	2	4	76	166
5	9	5	0	2	37	646
90	79	30	2	34	31	22
139	64	101	20	12	79	11
38	34	10	1	17	71	217
42	33	13	3	31	51	79
96	80	78	3	14	54	63
66	60	69	6	31	35	30
78	96	102	3	6	13	26
14	15	9	1	8	67	337
57	67	43	1	4	65	109
72	83	38	6	14	16	20
62	43	26	2	10	92	173
103	95	69	2	24	60	44
49	35	26	1	16	48	31
32	26	5	2	8	71	268
139	79	96	25	15	45	25
56	51	50	9	11	48	35
43	54	34	7	14	60	99
57	42	28	1	10	16	35
140	75	53	4	31	47	24
56	93	40	0	17	102	62
13	17	6	2	6	103	217

90	75	61	5	17	52	24
15	22	10	3	6	88	141
84	85	120	3	10	32	27
93	103	70	9	16	30	37
12	17	6	2	6	95	201
140	50	18	4	10	36	36
88	103	47	1	30	43	35
44	50	20	3	16	73	42
5	0	14	0	85	59	17
0	6	3	1	3	103	90
54	51	21	1	13	36	72
148	74	40	15	8	29	16
41	33	19	0	6	103	46
96	35	36	7	34	41	70
30	20	3	0	1	28	199
60	82	53	2	6	73	60
38	60	29	1	7	28	24
35	56	57	1	6	63	140
156	87	39	10	16	49	33
19	52	13	1	9	35	24
92	85	37	9	17	43	25
61	43	30	3	7	41	16
4	13	15	0	6	80	194
138	43	41	0	6	67	36
57	59	30	1	10	41	36
65	47	20	4	17	47	17
173	65	49	2	32	54	141
53	48	48	5	12	44	48
98	70	43	7	11	10	30
52	22	12	3	9	23	16
28	13	9	3	2	131	221
46	45	33	4	11	41	12
32	44	29	1	2	70	31
49	34	15	3	7	40	48
42	34	20	1	12	49	53
58	25	15	0	5	39	44
0	2	1	0	0	14	288
34	28	9	3	11	52	53
6	5	8	4	6	56	84
15	36	30	2	9	36	26
12	24	20	3	8	14	54
24	37	16	6	11	12	20
58	17	10	0	7	104	50
45	42	54	5	14	8	8
60	64	55	3	11	18	19
39	47	45	0	5	16	27
50	36	15	9	5	14	22
14	26	18	0	6	8	40
9	8	6	0	8	26	171
35	52	30	4	4	24	14
2	0	0	1	0	22	223
38	44	28	1	4	16	21
17	23	9	0	1	31	72
21	31	20	0	5	29	22
33	31	15	0	5	12	16
10	6	1	1	8	17	41
19	11	6	0	5	38	86
26	24	27	1	5	19	16
24	19	9	1	2	29	17

13	14	2	0	4	10	19
1	3	2	1	4	21	92
25	23	9	3	4	22	31
25	23	9	3	4	22	31
65	17	23	4	4	12	10
38	41	32	1	1	8	11
9	19	13	3	8	30	27
59	22	9	0	9	30	21
19	11	7	0	7	12	15
35	24	10	1	6	15	17
6	9	7	0	3	4	17
14	14	6	0	4	22	33
21	35	51	0	8	9	15
12	7	2	0	4	12	29
15	10	8	1	31	4	7
57	44	33	0	4	11	22
12	21	14	0	4	6	24
1	0	0	1	0	9	149
12	13	7	0	7	12	13
9	8	3	2	3	29	47
71	46	25	0	8	7	9
15	13	20	2	1	19	13
8	8	3	2	3	22	42
39	28	7	2	6	11	12
39	41	35	8	2	22	9
16	7	9	0	2	34	31
19	20	24	1	6	15	6
0	0	0	0	0	4	113
41	33	43	0	6	8	14
14	14	22	5	2	6	9
17	13	16	0	1	3	27
34	17	13	0	2	10	11
17	19	7	1	4	30	13
1	2	0	1	0	9	39
18	13	7	1	15	14	17
8	4	0	0	1	9	14
0	0	0	0	0	0	3
38	31	22	4	3	15	13
14	4	3	3	0	8	13
12	15	23	5	6	12	11
32	17	9	1	10	16	9
26	16	7	0	4	10	3
48	15	7	0	4	24	18
32	21	7	0	1	15	8
30	24	23	4	2	4	8
17	13	9	1	7	10	11
8	3	0	0	0	8	11
36	7	4	0	6	10	9
2	1	0	0	0	78	58
0	6	0	0	0	10	72
12	4	1	0	3	28	20
33	25	25	2	5	10	5
7	18	5	0	3	19	31
41	49	36	0	7	1	3
39	11	12	2	5	5	12
8	6	3	1	2	3	39
19	14	14	2	4	19	20
10	7	4	0	7	10	12
16	9	5	0	1	4	11

10	3	0	0	1	36	15
43	20	7	0	2	8	5
16	23	12	0	4	2	15
16	29	15	0	3	3	0
6	7	15	0	4	13	4
8	7	1	0	1	7	13
42	16	14	2	4	2	3
31	10	36	0	1	2	2
26	23	29	0	2	3	7
20	4	6	1	6	5	5
3	4	4	0	8	7	10
21	11	4	2	4	8	3
1	3	1	0	1	14	52
0	1	0	0	0	20	56
12	11	7	0	1	19	7
3	3	1	0	3	19	26
10	7	5	0	1	5	4
31	5	7	0	4	7	1
12	8	10	0	1	2	5
12	8	5	0	0	2	0
14	20	14	0	1	3	11
5	4	1	0	2	5	71
11	18	8	0	3	3	3
27	16	10	0	2	13	5
13	15	5	1	1	4	6
15	6	3	0	2	13	13
15	10	1	2	0	1	0
12	14	15	0	1	5	8
6	13	7	1	1	2	14
5	4	11	0	2	4	4
16	9	3	3	2	7	6
23	12	16	1	0	13	4
4	1	1	0	1	5	33
21	17	9	0	1	3	2
2	2	2	1	0	14	28
11	5	3	0	1	8	5
12	12	7	0	1	1	0
14	13	5	0	0	2	4
1	0	1	0	0	22	4
5	9	7	0	2	9	1
13	11	4	0	2	5	11
16	12	8	0	1	2	2
23	14	4	0	0	2	1
7	4	6	3	1	1	6
15	11	10	0	2	5	0
26	20	5	0	4	5	3
5	11	17	0	2	7	2
1	2	0	0	1	23	61
6	4	3	0	0	9	8
4	10	4	0	0	3	3
11	7	4	0	2	3	5
10	7	2	0	2	5	5
4	14	1	2	1	0	9
0	1	0	1	1	10	23
6	3	2	0	1	0	7
1	1	5	1	2	9	11
2	4	0	0	3	5	13
5	6	2	0	0	5	5
0	0	1	0	2	8	26

5	3	3	0	1	8	7
5	1	1	0	1	1	8
5	9	2	0	1	2	8
6	5	3	0	4	3	5
6	3	4	0	1	2	1
4	7	2	1	0	8	10
10	10	10	0	5	1	2
1	4	0	0	0	4	23
0	0	1	0	1	4	12
13	7	1	0	3	2	0
2	4	2	0	0	0	2
3	3	3	0	0	4	2
8	2	3	0	2	3	4
3	4	6	0	1	1	8
17	7	4	1	0	1	2
0	1	2	0	0	6	14
15	5	2	1	1	6	3
2	7	2	0	0	4	1
5	8	4	0	0	1	5
6	4	3	1	2	1	3
11	5	11	1	1	1	4
8	3	3	0	2	1	2
6	2	1	0	1	1	3
14	4	1	0	2	4	6
8	4	1	1	0	2	8
5	8	2	0	4	3	1
2	5	0	0	5	7	4
3	4	1	0	1	1	2
5	5	9	0	0	3	1
0	3	1	0	0	6	5
0	0	2	0	0	0	0
9	1	5	1	2	2	5
7	3	2	1	1	5	8
1	1	1	0	0	6	9
5	3	2	0	1	1	2
4	1	2	1	1	2	5
2	3	2	1	0	13	10
6	0	1	0	0	0	2
6	6	5	0	1	4	4
20	5	0	0	2	0	2
2	1	0	0	0	4	5
4	6	2	0	0	0	2
1	0	0	0	0	0	6
0	1	0	1	1	0	12
3	6	2	0	3	4	4
15	4	7	0	0	2	2
6	8	3	0	0	0	0
2	0	0	0	1	3	13
6	10	2	0	1	2	0
9	5	2	0	1	3	3
0	0	0	0	0	0	24
0	0	0	0	0	1	18
0	0	0	0	0	1	17
2	4	1	0	0	8	2
6	3	5	0	0	4	2
11	3	1	0	1	1	0
1	1	6	0	0	1	3
1	5	4	2	1	4	2
0	1	0	0	0	2	40

0	0	0	0	0	3	6
3	2	1	0	0	2	4
0	1	0	0	0	1	18
12	2	2	1	1	5	3
9	7	3	0	0	0	0
5	3	9	0	0	3	1
0	1	0	0	0	2	10
5	5	6	0	0	0	10
0	0	0	0	0	1	2
4	3	1	0	0	0	1
2	1	0	0	0	1	4
0	0	2	0	0	1	18
5	8	0	0	0	6	4
5	6	3	0	1	2	1
14	5	3	0	0	1	2
2	0	1	0	2	0	8
2	4	5	0	1	1	0
4	2	2	0	0	0	4
7	5	4	0	0	4	3
5	7	0	0	1	3	8
7	1	1	0	0	4	4
0	1	0	0	0	8	41
2	4	1	1	3	1	3
6	2	3	0	2	2	0
2	4	3	0	0	3	3
1	2	1	0	0	1	0
1	0	1	0	0	5	8
0	1	0	0	0	9	11
2	0	0	0	1	2	0
2	2	3	0	0	2	0
2	2	2	1	0	1	2
2	3	1	1	1	4	4
0	0	0	0	0	3	7
1	7	2	0	1	5	2
1	2	0	0	0	1	6
3	4	1	0	0	2	2
2	1	3	1	0	1	2
2	3	3	0	2	4	2
3	0	3	0	1	4	0
4	5	6	0	1	5	0
1	5	3	0	0	0	1
5	4	6	0	1	1	1
3	3	2	0	0	1	2
2	1	1	1	0	0	1
1	5	1	0	0	7	2
0	0	0	0	0	2	6
3	1	6	0	0	0	2
0	1	0	0	0	2	17
7	4	1	0	1	0	1
1	0	1	0	0	1	8
0	1	1	0	0	0	6
4	0	0	0	0	8	2
0	1	1	0	0	3	3
1	2	6	0	0	0	3
1	1	0	0	0	4	7
0	0	1	0	0	1	3
1	3	2	2	1	0	0
1	0	0	0	0	10	2
0	1	0	0	0	2	12

1	3	1	0	1	0	0
0	1	1	0	0	0	1
0	0	0	0	0	2	13
0	1	0	0	0	3	14
2	1	3	0	1	0	0
4	7	4	0	1	0	0
2	1	1	0	0	2	2
11	3	3	0	1	0	0
5	2	4	0	0	1	1
0	0	0	0	0	3	12
0	1	0	0	1	0	10
0	0	0	0	0	3	6
4	4	0	0	0	1	1
3	0	2	0	0	3	3
5	1	0	1	1	0	0
0	1	4	0	0	2	0
0	3	3	0	0	2	3
2	5	1	0	0	1	2
1	2	0	0	0	1	4
1	1	2	0	0	3	0
4	2	5	0	1	1	1
1	0	0	0	0	3	5
4	0	4	0	0	3	0
5	4	3	0	0	0	1
4	3	3	0	1	3	0
6	4	1	0	1	0	0
2	2	2	0	0	1	2
9	3	1	0	1	2	1
0	1	1	0	0	3	1
3	4	4	0	1	2	1
0	1	2	0	0	2	0
7	0	1	0	0	0	1
0	1	0	0	0	1	11
0	1	0	0	0	0	2
0	2	2	0	0	0	9
0	1	0	0	0	2	1
0	2	0	0	1	0	2
2	0	1	0	0	1	1
3	2	1	1	0	0	0
1	3	2	0	0	0	5
0	0	0	0	0	0	2
2	4	1	0	0	0	2
4	0	0	0	0	0	1
0	1	1	0	0	4	4
1	0	0	0	0	0	1
2	4	4	0	0	0	3
4	4	2	0	1	2	1
0	1	2	0	1	0	0
2	2	0	0	0	1	0
0	0	0	0	0	1	0
1	2	2	0	1	2	0
2	0	2	0	0	3	4
57358	52086	38665	3705	12768	54908	75187
131926	121159	105787	9030	22399	96148	122273
385	390	408	475	444	386	369
UC	UC	UC	UC	UC	UC	CHR_NP
UC	UC	UC	UC	UC	UC	CHR_NP

22	23	24	25	26	27	28
22	23	24	25	26	27	1
UC	UC	UC	UC	UC	UC	CHR_NP
F_6	F_7	F_8	G_33	G_34	G_8	A_1
1873959	1687678	1238990	121924	416015	1782397	2570804
UC	UC	UC	UC	UC	UC	CHR_NP
11.98	12.33	14.79	3.24	6.65	8.93	6.98
2.11	2.10	1.41	12.29	6.06	3.47	0.77
3.70	2.91	2.57	1.57	3.14	1.02	0.26
2.69	2.95	2.67	5.40	2.89	2.64	2.05
3.41	3.26	3.24	2.47	3.76	1.14	0.54
0.16	0.28	0.24	0.33	0.49	2.91	5.71
0.97	0.87	0.48	1.38	1.99	1.92	1.73
0.03	0.08	0.04	0.06	0.07	0.88	3.19
0.20	0.29	0.24	0.08	0.47	1.42	1.67
0.35	0.35	0.26	0.35	0.60	1.18	1.20
0.30	0.39	0.34	0.09	0.38	0.45	0.54
0.61	0.67	0.56	0.38	0.54	0.47	0.37
0.39	0.41	0.26	0.27	0.37	0.42	0.64
0.46	0.59	0.79	0.53	0.44	0.35	0.30
0.07	0.09	0.04	0.09	0.06	0.31	1.02
0.69	0.53	0.63	0.05	0.09	0.07	0.09
0.04	0.04	0.02	0.10	0.13	0.32	0.43
0.21	0.23	0.16	0.10	0.14	0.13	0.22
0.10	0.11	0.06	0.08	0.18	0.35	0.44
0.25	0.23	0.16	0.24	0.23	0.19	0.16
0.22	0.23	0.15	0.06	0.11	0.07	0.07
0.13	0.14	0.10	0.07	0.16	0.19	0.14
0.14	0.13	0.07	0.07	0.14	0.21	0.17
0.08	0.08	0.02	0.26	0.22	0.06	0.23
0.02	0.03	0.02	0.06	0.12	0.12	0.41
0.19	0.15	0.13	0.01	0.09	0.11	0.11
0.19	0.25	0.34	0.13	0.17	0.09	0.05
0.05	0.05	0.03	0.04	0.03	0.32	0.27
0.08	0.10	0.07	0.12	0.09	0.10	0.19
0.17	0.15	0.12	0.08	0.17	0.14	0.06
0.10	0.08	0.05	0.08	0.11	0.13	0.06
0.23	0.21	0.15	0.22	0.16	0.11	0.09
0.03	0.04	0.02	0.03	0.06	0.13	0.34
0.01	0.03	0.02	0.02	0.02	0.05	0.53
0.07	0.09	0.06	0.11	0.08	0.09	0.13
0.06	0.10	0.11	0.22	0.14	0.10	0.05
0.05	0.06	0.04	0.13	0.07	0.14	0.14
0.02	0.03	0.03	0.08	0.09	0.09	0.16
0.06	0.07	0.07	0.29	0.14	0.06	0.05
0.33	0.11	0.09	0.13	0.08	0.03	0.04
0.16	0.12	0.10	0.20	0.04	0.02	0.02
0.09	0.09	0.11	0.05	0.11	0.02	0.02
0.06	0.06	0.07	0.10	0.06	0.05	0.04
0.00	0.01	0.01	0.02	0.01	0.11	0.20
0.05	0.05	0.04	0.04	0.11	0.03	0.04
0.07	0.07	0.05	0.03	0.05	0.04	0.03
0.05	0.04	0.03	0.03	0.08	0.06	0.07
0.11	0.07	0.09	0.02	0.04	0.03	0.02
0.00	0.00	0.00	0.00	0.00	0.02	0.23
0.01	0.02	0.01	0.00	0.01	0.03	0.12
0.06	0.03	0.02	0.07	0.06	0.05	0.01
0.02	0.02	0.01	0.05	0.05	0.02	0.03

0.03	0.04	0.05	0.04	0.04	0.03	0.01
0.02	0.02	0.01	0.01	0.01	0.02	0.05
0.03	0.04	0.03	0.00	0.02	0.07	0.04
0.00	0.00	0.00	0.00	0.00	0.02	0.16
0.01	0.02	0.01	0.03	0.04	0.03	0.03
0.04	0.03	0.02	0.02	0.03	0.03	0.02
0.02	0.03	0.03	0.01	0.05	0.01	0.02
0.06	0.05	0.02	0.15	0.06	0.02	0.01
0.03	0.03	0.02	0.03	0.04	0.02	0.02
0.03	0.02	0.02	0.00	0.02	0.02	0.01
0.00	0.00	0.00	0.00	0.00	0.04	0.26
0.00	0.00	0.00	0.00	0.00	0.01	0.18
0.03	0.03	0.04	0.02	0.00	0.02	0.01
0.01	0.01	0.01	0.01	0.02	0.03	0.04
0.00	0.01	0.01	0.00	0.01	0.02	0.02
0.00	0.01	0.00	0.01	0.01	0.04	0.07
0.01	0.01	0.01	0.01	0.02	0.01	0.01
0.01	0.00	0.00	0.01	0.01	0.05	0.03
0.02	0.02	0.02	0.03	0.02	0.01	0.00
0.02	0.03	0.04	0.01	0.01	0.01	0.01
0.00	0.00	0.00	0.01	0.00	0.03	0.09
0.01	0.01	0.01	0.00	0.01	0.02	0.02
0.01	0.01	0.00	0.01	0.03	0.01	0.02
0.05	0.02	0.02	0.03	0.02	0.02	0.01
0.02	0.02	0.02	0.00	0.01	0.02	0.01
0.02	0.01	0.01	0.05	0.02	0.02	0.00
0.03	0.02	0.02	0.02	0.02	0.01	0.00
0.01	0.01	0.01	0.01	0.02	0.01	0.00
0.01	0.01	0.01	0.01	0.01	0.01	0.02
0.03	0.02	0.03	0.00	0.01	0.01	0.00
0.01	0.02	0.02	0.03	0.01	0.01	0.01
0.00	0.01	0.01	0.00	0.00	0.00	0.01
0.00	0.00	0.00	0.01	0.02	0.02	0.02
0.02	0.01	0.01	0.00	0.01	0.01	0.00
0.01	0.02	0.02	0.01	0.01	0.01	0.00
0.00	0.00	0.00	0.00	0.00	0.02	0.04
0.01	0.01	0.00	0.01	0.01	0.01	0.01
0.03	0.02	0.01	0.04	0.02	0.01	0.01
0.02	0.01	0.01	0.01	0.01	0.00	0.00
0.00	0.00	0.00	0.00	0.00	0.00	0.05
0.01	0.01	0.01	0.00	0.01	0.00	0.00
0.00	0.01	0.00	0.00	0.01	0.01	0.01
0.00	0.00	0.00	0.00	0.00	0.01	0.02
0.00	0.00	0.00	0.00	0.00	0.00	0.01
0.01	0.01	0.01	0.01	0.01	0.00	0.00
0.01	0.01	0.01	0.01	0.01	0.00	0.00
0.01	0.01	0.01	0.01	0.02	0.00	0.00
0.00	0.00	0.00	0.00	0.00	0.01	0.01
0.00	0.01	0.00	0.00	0.01	0.00	0.00
0.01	0.01	0.00	0.00	0.01	0.01	0.00
0.01	0.01	0.01	0.00	0.01	0.00	0.00
0.01	0.01	0.00	0.01	0.01	0.01	0.01
0.00	0.00	0.00	0.00	0.00	0.01	0.03
0.00	0.00	0.00	0.00	0.00	0.01	0.02
0.01	0.01	0.00	0.01	0.01	0.00	0.00
0.00	0.00	0.00	0.02	0.01	0.00	0.00
0.00	0.00	0.00	0.00	0.00	0.01	0.02
0.00	0.00	0.00	0.00	0.00	0.01	0.01
0.00	0.00	0.00	0.00	0.00	0.00	0.01

0.01	0.01	0.01	0.00	0.01	0.00	0.00
0.00	0.00	0.00	0.01	0.01	0.00	0.00
0.00	0.00	0.00	0.00	0.00	0.01	0.01
0.01	0.01	0.01	0.00	0.01	0.00	0.00
0.01	0.01	0.01	0.01	0.01	0.00	0.00
0.00	0.00	0.00	0.00	0.00	0.00	0.01
0.00	0.01	0.00	0.00	0.00	0.00	0.01
0.00	0.01	0.00	0.00	0.00	0.00	0.01
0.00	0.00	0.00	0.01	0.01	0.00	0.00
0.00	0.00	0.00	0.00	0.00	0.00	0.00
0.00	0.00	0.00	0.00	0.00	0.00	0.00
0.01	0.00	0.00	0.00	0.00	0.01	0.01
0.00	0.00	0.00	0.00	0.00	0.00	0.00
0.00	0.00	0.00	0.00	0.00	0.00	0.01
0.01	0.01	0.00	0.01	0.00	0.00	0.00
0.00	0.00	0.00	0.01	0.01	0.00	0.00
0.01	0.01	0.01	0.00	0.00	0.00	0.00
0.00	0.00	0.00	0.02	0.00	0.00	0.00
0.01	0.01	0.00	0.02	0.00	0.00	0.00
0.00	0.00	0.00	0.00	0.00	0.01	0.00
0.00	0.00	0.00	0.00	0.00	0.00	0.00
0.00	0.00	0.00	0.00	0.00	0.00	0.01
0.00	0.00	0.00	0.00	0.00	0.00	0.00
0.00	0.00	0.00	0.00	0.00	0.00	0.01
0.00	0.00	0.00	0.00	0.00	0.00	0.00
0.00	0.00	0.00	0.00	0.00	0.00	0.00
0.00	0.00	0.00	0.00	0.00	0.00	0.00
0.00	0.00	0.00	0.00	0.00	0.00	0.00
0.00	0.00	0.00	0.00	0.00	0.00	0.00
0.00	0.00	0.00	0.00	0.00	0.00	0.00
0.00	0.00	0.00	0.00	0.00	0.00	0.00
0.01	0.00	0.00	0.00	0.00	0.00	0.00
0.00	0.00	0.00	0.00	0.00	0.00	0.00
0.00	0.00	0.00	0.00	0.00	0.00	0.01
0.00	0.00	0.00	0.00	0.00	0.00	0.00
0.00	0.00	0.00	0.00	0.00	0.00	0.00
0.00	0.00	0.00	0.00	0.00	0.00	0.00
0.00	0.00	0.00	0.00	0.00	0.00	0.00
0.00	0.00	0.00	0.00	0.00	0.00	0.00
0.00	0.00	0.00	0.00	0.00	0.00	0.00
0.00	0.00	0.00	0.00	0.00	0.00	0.00
0.00	0.00	0.00	0.00	0.00	0.00	0.00
0.00	0.00	0.00	0.00	0.00	0.00	0.00
0.00	0.00	0.00	0.00	0.00	0.00	0.00
0.00	0.00	0.00	0.00	0.00	0.00	0.00
0.00	0.00	0.00	0.01	0.00	0.00	0.00
0.00	0.00	0.00	0.00	0.00	0.00	0.00
0.00	0.00	0.00	0.00	0.00	0.00	0.00
0.00	0.00	0.00	0.00	0.00	0.00	0.00
0.00	0.00	0.00	0.00	0.00	0.00	0.01
0.00	0.00	0.00	0.00	0.00	0.00	0.00
0.00	0.00	0.00	0.01	0.00	0.00	0.00
0.00	0.00	0.00	0.00	0.00	0.00	0.00
0.00	0.00	0.00	0.00	0.00	0.00	0.00
0.00	0.00	0.00	0.00	0.00	0.00	0.00
0.00	0.00	0.00	0.00	0.00	0.00	0.00
0.00	0.00	0.00	0.00	0.00	0.00	0.00
0.00	0.00	0.00	0.00	0.00	0.00	0.00
0.00	0.00	0.00	0.00	0.00	0.00	0.00

0.00	0.00	0.00	0.00	0.00	0.00	0.00
0.00	0.00	0.00	0.00	0.00	0.00	0.00
0.00	0.00	0.00	0.00	0.00	0.00	0.00
0.00	0.00	0.00	0.00	0.00	0.00	0.00
0.00	0.00	0.00	0.00	0.00	0.00	0.00
0.00	0.00	0.00	0.01	0.00	0.00	0.00
0.00	0.00	0.00	0.00	0.00	0.00	0.00
0.00	0.00	0.00	0.00	0.00	0.00	0.00
0.00	0.00	0.00	0.00	0.00	0.00	0.00
0.00	0.00	0.00	0.00	0.00	0.00	0.00
0.00	0.00	0.00	0.00	0.00	0.00	0.00
0.00	0.00	0.00	0.00	0.00	0.00	0.00
0.00	0.00	0.00	0.00	0.00	0.00	0.00
0.00	0.00	0.00	0.00	0.00	0.00	0.00
0.00	0.00	0.00	0.00	0.00	0.00	0.00
0.00	0.00	0.00	0.00	0.00	0.00	0.00
0.00	0.00	0.00	0.00	0.00	0.00	0.00
0.00	0.00	0.00	0.00	0.00	0.00	0.00
0.00	0.00	0.00	0.00	0.00	0.00	0.00
0.00	0.00	0.00	0.00	0.00	0.00	0.00
0.00	0.00	0.00	0.00	0.01	0.00	0.00
0.00	0.00	0.00	0.00	0.00	0.00	0.00
0.00	0.00	0.00	0.00	0.00	0.00	0.00
0.00	0.00	0.00	0.00	0.00	0.00	0.00
0.00	0.00	0.00	0.00	0.00	0.00	0.00
0.00	0.00	0.00	0.00	0.00	0.00	0.00
0.00	0.00	0.00	0.00	0.00	0.00	0.00
0.00	0.00	0.00	0.00	0.00	0.00	0.00
0.00	0.00	0.00	0.00	0.00	0.00	0.00
0.00	0.00	0.00	0.00	0.00	0.00	0.00
0.00	0.00	0.00	0.00	0.00	0.00	0.00
0.00	0.00	0.00	0.00	0.00	0.00	0.00
0.00	0.00	0.00	0.00	0.00	0.00	0.00
0.00	0.00	0.00	0.00	0.00	0.00	0.00
0.00	0.00	0.00	0.00	0.00	0.00	0.00
0.00	0.00	0.00	0.00	0.00	0.00	0.00
0.00	0.00	0.00	0.00	0.00	0.00	0.00
0.00	0.00	0.00	0.00	0.00	0.00	0.00
0.00	0.00	0.00	0.00	0.00	0.00	0.00
0.00	0.00	0.00	0.00	0.00	0.00	0.00
0.00	0.00	0.00	0.00	0.00	0.00	0.00
0.00	0.00	0.00	0.00	0.00	0.00	0.00
0.00	0.00	0.00	0.00	0.00	0.00	0.00
0.00	0.00	0.00	0.00	0.00	0.00	0.00
0.00	0.00	0.00	0.00	0.00	0.00	0.00
0.00	0.00	0.00	0.00	0.00	0.00	0.00
0.00	0.00	0.00	0.00	0.00	0.00	0.00
0.00	0.00	0.00	0.00	0.00	0.00	0.00
0.00	0.00	0.00	0.00	0.00	0.00	0.00
0.00	0.00	0.00	0.00	0.00	0.00	0.00
0.00	0.00	0.00	0.00	0.00	0.00	0.00
0.00	0.00	0.00	0.00	0.00	0.00	0.00
0.00	0.00	0.00	0.00	0.00	0.00	0.00
0.00	0.00	0.00	0.00	0.00	0.00	0.00
0.00	0.00	0.00	0.00	0.00	0.00	0.00
0.00	0.00	0.00	0.00	0.00	0.00	0.00
0.00	0.00	0.00	0.00	0.00	0.00	0.00
0.00	0.00	0.00	0.00	0.00	0.00	0.00
0.00	0.00	0.00	0.00	0.00	0.00	0.00

0.00	0.00	0.00	0.00	0.00	0.00	0.00
0.00	0.00	0.00	0.00	0.00	0.00	0.00
0.00	0.00	0.00	0.00	0.00	0.00	0.00
0.00	0.00	0.00	0.00	0.00	0.00	0.00
0.00	0.00	0.00	0.00	0.00	0.00	0.00
0.00	0.00	0.00	0.00	0.00	0.00	0.00
0.00	0.00	0.00	0.00	0.00	0.00	0.00
0.00	0.00	0.00	0.00	0.00	0.00	0.00
0.00	0.00	0.00	0.00	0.00	0.00	0.00
0.00	0.00	0.00	0.00	0.00	0.00	0.00
0.00	0.00	0.00	0.00	0.00	0.00	0.00
0.00	0.00	0.00	0.00	0.00	0.00	0.00
0.00	0.00	0.00	0.00	0.00	0.00	0.00
0.00	0.00	0.00	0.00	0.00	0.00	0.00
0.00	0.00	0.00	0.00	0.00	0.00	0.00
0.00	0.00	0.00	0.00	0.00	0.00	0.00
0.00	0.00	0.00	0.00	0.00	0.00	0.00
0.00	0.00	0.00	0.00	0.00	0.00	0.00
0.00	0.00	0.00	0.00	0.00	0.00	0.00
0.00	0.00	0.00	0.00	0.00	0.00	0.00
0.00	0.00	0.00	0.00	0.00	0.00	0.00
0.00	0.00	0.00	0.00	0.00	0.00	0.00
0.00	0.00	0.00	0.00	0.00	0.00	0.00
0.00	0.00	0.00	0.00	0.00	0.00	0.00
0.00	0.00	0.00	0.00	0.00	0.00	0.00
0.00	0.00	0.00	0.00	0.00	0.00	0.00
0.00	0.00	0.00	0.00	0.00	0.00	0.00
0.00	0.00	0.00	0.00	0.00	0.00	0.00
0.00	0.00	0.00	0.00	0.00	0.00	0.00
0.00	0.00	0.00	0.00	0.00	0.00	0.00

22	23	24	25	26	27	28
24	25	26	27	28	29	1
UC	UC	UC	UC	UC	UC	CHR_NP
F_6	F_7	F_8	G_33	G_34	G_8	A_1
1873959	1687678	1238990	121924	416015	1782397	2570804
F_6	F_7	F_8	G_33	G_34	G_8	A_1
0.08	0.16	0.71	-1.88	-1.11	-0.60	-1.04
-0.34	-0.34	-0.55	2.64	0.82	0.06	-0.73
1.20	0.60	0.35	-0.41	0.78	-0.82	-1.39
0.24	0.49	0.22	2.92	0.43	0.19	-0.39
1.38	1.21	1.19	0.34	1.77	-1.13	-1.79
-0.93	-0.82	-0.86	-0.78	-0.64	1.56	4.09
-0.32	-0.47	-1.09	0.34	1.32	1.21	0.90
-0.56	-0.47	-0.54	-0.52	-0.49	0.97	5.14

-0.77	-0.60	-0.70	-1.02	-0.24	1.65	2.15
-1.04	-1.05	-1.29	-1.05	-0.41	1.07	1.12
-0.65	-0.30	-0.51	-1.42	-0.36	-0.07	0.27
1.04	1.58	0.57	-0.97	0.43	-0.16	-1.11
0.21	0.30	-0.56	-0.51	0.09	0.35	1.60
-0.06	0.73	1.83	0.38	-0.14	-0.67	-0.97
-0.69	-0.60	-0.83	-0.57	-0.74	0.41	3.67
1.71	1.01	1.46	-1.06	-0.90	-0.98	-0.88
-0.73	-0.74	-0.86	-0.34	-0.19	1.00	1.74
-0.09	0.11	-0.49	-1.15	-0.73	-0.82	0.06
-0.70	-0.68	-1.05	-0.94	-0.01	1.52	2.29
0.20	0.04	-0.70	0.09	0.02	-0.36	-0.75
1.08	1.15	-0.14	-1.54	-0.70	-1.39	-1.44
-0.39	-0.20	-0.71	-1.27	0.19	0.61	-0.21
0.22	0.12	-0.61	-0.66	0.20	1.18	0.64
-0.38	-0.41	-0.98	1.32	0.96	-0.54	1.00
-0.63	-0.62	-0.64	-0.41	-0.03	-0.05	1.67
1.34	0.69	0.24	-1.91	-0.47	-0.12	-0.18
0.79	1.83	3.16	-0.15	0.53	-0.74	-1.36
-0.60	-0.56	-0.80	-0.74	-0.77	1.97	1.52
-0.60	-0.19	-0.77	0.16	-0.33	-0.16	1.56
1.68	1.17	0.47	-0.61	1.57	0.79	-0.98
0.11	-0.20	-1.01	-0.32	0.38	0.94	-0.66
2.08	1.72	0.64	1.86	0.79	-0.24	-0.53
-0.47	-0.34	-0.55	-0.47	0.04	1.07	4.26
-0.34	-0.25	-0.30	-0.30	-0.27	-0.12	2.49
-0.49	-0.05	-0.69	0.31	-0.25	-0.09	0.71
-0.48	-0.27	-0.19	0.47	-0.03	-0.27	-0.56
-0.77	-0.57	-0.90	0.16	-0.52	0.32	0.29
-0.74	-0.62	-0.69	-0.15	0.02	-0.03	0.67
-0.51	-0.37	-0.40	2.27	0.45	-0.45	-0.61
1.82	0.09	-0.07	0.25	-0.17	-0.58	-0.52
1.65	0.92	0.42	2.62	-0.84	-1.23	-1.13
0.75	0.69	1.22	-0.65	1.18	-1.33	-1.59
-0.23	-0.32	0.12	1.60	-0.13	-0.78	-1.00
-0.83	-0.70	-0.76	-0.55	-0.69	1.77	4.11
-0.22	-0.34	-0.49	-0.50	0.92	-0.65	-0.52
1.82	1.48	0.47	-0.96	0.45	-0.62	-1.11
0.26	-0.05	-0.47	-0.49	1.62	0.93	1.01
1.85	0.78	1.42	-1.05	-0.37	-0.57	-1.00
-0.34	-0.24	-0.28	-0.29	-0.26	0.30	7.55
-0.39	-0.19	-0.53	-0.65	-0.42	0.04	1.96
1.06	-0.36	-0.75	1.78	1.21	0.79	-1.16
-0.42	-0.47	-0.67	0.38	0.35	-0.36	-0.27
-0.56	0.49	1.04	0.41	0.72	-0.61	-1.78
-0.26	-0.44	-1.03	-1.02	-0.81	-0.28	1.10
0.55	0.90	0.46	-1.37	-0.46	2.35	0.80

-0.32	-0.25	-0.25	-0.34	-0.32	0.27	6.29
-1.06	-0.72	-1.10	0.54	0.82	0.23	0.53
1.50	0.66	-0.36	-0.69	0.64	0.09	-0.39
-0.54	-0.13	-0.04	-1.33	1.75	-1.24	-1.08
0.63	0.11	-0.42	2.90	0.52	-0.43	-0.82
0.08	0.06	-0.45	0.26	1.36	-0.50	-0.64
0.33	-0.12	-0.40	-1.44	0.10	-0.47	-0.76
-0.68	-0.64	-0.67	-0.39	-0.49	2.24	19.45
-0.27	-0.24	-0.23	-0.21	-0.21	0.03	7.70
-0.25	0.23	0.88	-0.36	-1.42	-0.64	-1.17
-0.99	-0.61	-0.88	-0.52	0.19	1.59	2.58
-0.53	-0.29	-0.41	-0.63	0.17	0.87	0.85
-0.67	-0.40	-0.62	-0.47	-0.20	2.60	5.46
-0.22	-0.34	-0.44	-0.65	0.39	-0.69	-0.10
-0.36	-0.37	-0.39	-0.33	-0.30	0.20	-0.05
0.11	0.04	-0.19	0.40	-0.16	-1.04	-1.28
0.36	0.67	1.76	-0.82	-1.30	-1.47	-1.05
-0.79	-0.63	-0.83	-0.13	-0.66	1.70	7.47
-0.21	-0.38	-0.75	-1.54	-1.00	0.18	0.35
-0.14	-0.16	-0.79	0.00	1.88	-0.02	0.53
2.50	0.18	-0.12	0.76	0.06	0.13	-0.73
0.36	0.33	0.07	-1.63	-0.04	1.27	-0.61
-0.11	-0.27	-0.33	0.78	-0.03	0.07	-0.43
1.07	0.67	0.57	0.10	0.62	-0.86	-1.22
-0.56	-0.03	-0.11	-0.22	0.45	-0.90	-1.07
-0.83	-0.34	-0.98	-0.84	-0.50	0.09	2.43
1.82	1.08	1.85	-1.41	-0.21	-1.03	-1.23
-0.23	0.15	0.07	1.34	-0.88	-0.69	-0.92
-0.34	-0.14	-0.17	-0.47	-0.30	-0.36	0.14
-0.56	-0.57	-0.66	0.35	1.65	2.00	2.55
1.43	0.88	0.65	-1.08	-0.12	-0.34	-0.80
0.26	0.43	1.54	0.01	-0.52	-1.57	-1.88
-0.73	-0.59	-0.85	-0.61	-0.45	1.54	6.13
-0.57	-0.86	-1.29	-0.21	-0.85	-0.65	-0.52
2.21	0.45	-0.30	3.11	0.59	-0.56	-0.61
2.31	1.45	0.47	-0.65	-0.45	-1.18	-1.35
-0.31	-0.27	-0.23	-0.21	-0.30	0.27	9.91
0.65	1.08	-0.05	-1.26	-0.08	-1.02	-0.66
-0.23	0.34	-0.55	-0.51	-0.09	1.73	0.73
-0.56	-0.46	-0.50	-0.49	-0.34	0.46	3.56
-0.67	-0.41	-0.58	-0.60	-0.69	-0.48	0.93
0.92	0.85	0.84	0.69	-0.56	-1.03	-1.52
1.40	1.59	0.76	0.34	-0.61	-0.86	-1.86
1.01	1.26	0.74	0.24	2.14	-0.86	-1.28
-0.70	-0.55	-0.65	-0.50	-0.44	1.26	2.44
-0.27	-0.11	-0.20	-0.59	0.03	-0.23	-0.50
0.53	1.29	-0.50	-0.16	0.61	0.71	-0.59
0.40	0.67	1.27	-0.57	1.44	-1.13	-1.04

-0.26	-0.28	-0.54	0.38	-0.10	0.18	0.45
-1.30	-0.92	-0.82	-1.17	-0.88	1.85	8.06
-0.79	-0.51	-0.73	-0.67	-0.57	1.32	5.07
0.43	0.17	-0.14	1.54	1.08	-1.18	-1.19
-0.53	-0.38	-0.47	1.75	0.62	-0.30	-0.61
-0.83	-0.46	-0.36	-0.51	-0.22	0.55	4.67
-0.29	-0.18	-0.49	-0.52	0.38	1.47	2.23
-0.25	-0.33	-0.94	-0.16	-0.34	-0.44	0.32
1.91	2.64	1.15	-0.19	0.47	-1.29	-1.54
-0.01	-0.08	-0.75	0.51	2.78	-0.27	-0.80
-0.92	-0.55	-0.74	-0.78	-0.29	2.22	4.25
2.31	0.71	0.41	-0.47	2.20	-1.01	-0.43
2.87	0.97	0.48	1.76	0.32	-0.76	-0.32
0.19	0.65	-0.93	-0.83	0.02	0.47	5.68
-0.20	0.98	-0.17	0.47	-0.33	0.26	1.89
-0.20	0.98	-0.17	0.47	-0.33	0.26	1.89
-0.60	-0.39	-0.71	0.89	0.90	-0.34	-0.48
-0.12	0.15	0.48	-1.18	0.18	-0.61	-0.64
0.35	-0.24	0.31	-2.06	-0.72	-1.52	-1.24
1.00	-0.05	-0.49	-0.77	0.29	3.42	1.28
0.65	0.22	-0.49	-0.73	-0.65	-0.04	0.44
-0.35	-0.27	-0.30	-0.39	0.04	0.34	4.34
0.59	1.14	0.00	0.67	-0.92	-0.89	-1.01
-0.16	0.00	0.26	1.29	2.72	-0.50	-0.59
1.08	1.28	1.08	-1.52	-0.35	-0.33	-0.78
0.33	0.37	0.36	4.15	-0.58	-0.70	-0.69
0.55	0.73	0.06	4.30	-0.07	-0.31	-0.60
-0.22	-0.35	-0.42	-0.64	-0.53	1.79	-0.29
1.83	0.06	-1.05	-0.25	0.23	0.38	-0.88
0.08	0.50	-0.47	0.18	0.68	1.62	2.05
0.03	-0.04	-0.17	-0.36	-0.30	-0.88	-0.70
-0.37	-0.33	-0.24	-0.71	-0.23	0.75	3.75
0.32	0.17	-0.18	-1.77	1.62	-0.34	-0.93

19	20	21	22	23	24	25
21	22	23	24	25	26	27
CAAAGAATTCTC(	CACTAGATTGTGA	ACTGGACTTGGA	CAAAGTGCTGTT	TCAGTGCACTACA	TGTCAGTTTGTCA	TGAGAACTGAAT
miR-186-5p	miR-28-3p	miR-378a-3p	miR-93-5p	miR-148a-3p	miR-223-3p	miR-146b-5p
979581	903691	738230	679306	664224	624773	614674
0.09	0.42	0.38	1.24	0.38	-0.15	-0.58
0.03	3.14	-1.59	-1.25	-1.27	0.42	2.00
0.71	-0.33	-0.62	2.48	-0.65	-0.05	0.04
-0.64	-0.84	-0.58	1.64	-0.45	-0.48	-0.40
-0.43	-0.57	0.66	-0.94	-1.13	0.78	0.49
-1.04	0.22	-0.55	-1.32	1.18	-0.90	-0.40
-1.09	0.15	0.66	0.26	-0.16	-0.98	-0.68
-0.87	-0.99	-0.36	-0.80	-0.75	-0.87	-0.71
-0.22	-0.89	-0.95	-0.23	-0.85	-0.70	-0.23
0.51	-0.17	-0.60	0.26	-0.72	-0.59	0.81
1.40	1.11	-0.84	-0.93	0.10	1.52	3.99
-0.81	-0.82	-0.14	-1.03	-0.69	-0.97	-0.56
2.54	-0.26	2.12	0.95	2.66	0.75	0.27
-0.92	-1.04	-0.42	-0.38	-1.14	-0.78	-0.63
0.42	-0.22	1.33	1.27	-0.28	-0.41	-0.41
1.89	0.03	-0.23	-0.06	0.58	0.24	-0.20
-0.30	-0.02	1.83	0.22	-0.54	-0.62	-0.47
-0.46	-0.70	0.97	1.62	-0.70	0.30	-0.49
0.75	2.92	0.82	-0.44	2.30	3.13	0.20
1.09	-0.41	0.33	-0.54	0.81	1.28	0.70

-0.79	-0.01	-0.68	-0.27	0.86	-0.89	-0.37
-0.70	0.20	1.08	-0.39	0.22	-0.38	-0.63
-0.68	0.04	1.15	-0.20	0.12	-0.41	-0.62
-1.05	-0.70	-0.14	-0.71	-0.61	-0.98	-0.64
-0.94	0.09	-1.54	-1.27	-0.66	1.32	-0.41
-0.01	0.02	-0.70	0.19	0.20	0.96	-0.03
1.52	-0.36	-1.39	0.61	1.18	-0.54	-0.05
2.29	-0.75	-1.44	-0.21	0.64	1.00	1.67
1.37	6.39	-0.55	-0.53	4.72	0.89	2.81
-0.14	3.15	-0.50	0.28	0.93	-0.17	1.13
-1.00	0.86	-0.84	1.77	-0.75	-0.97	-0.57
1.28	7.78	-0.24	0.04	2.64	0.21	6.40
0.34	-1.27	-0.17	-1.04	-0.90	4.39	0.09
-0.63	0.17	0.23	0.09	0.24	0.45	-0.67
-0.36	-0.58	0.20	0.76	-0.44	0.49	-0.67
-0.10	0.44	1.05	-0.65	0.05	0.23	-0.36
-1.40	-1.95	-1.85	-1.72	-1.35	-1.10	-0.75
0.17	1.25	1.05	0.49	1.63	-0.44	0.30
2.29	0.68	1.70	2.86	1.31	1.40	0.25
0.28	0.13	1.69	-0.56	-0.62	0.80	0.03
-1.37	0.78	2.06	-1.02	-0.90	-0.92	-0.44
-1.42	0.78	9.02	-0.94	-0.86	-0.73	-0.04
-0.52	-0.02	-0.89	-0.17	-0.56	-0.45	-0.50
1.34	1.61	0.67	1.30	4.26	0.74	-0.41
-0.53	0.55	-0.22	-1.23	-0.77	-0.58	-0.39
-1.41	-0.43	-0.69	-1.70	-1.32	-1.03	-0.62
1.57	0.34	0.47	-0.60	1.09	3.98	0.48
-0.49	0.89	0.77	-0.63	0.62	-0.10	-0.44
0.40	-1.03	-0.55	-0.22	-0.59	0.46	-0.50
-0.09	-0.28	1.25	-0.56	-0.63	-0.19	-0.39
0.40	-0.53	-0.08	-0.42	-0.48	-0.33	-0.09
-0.69	0.20	0.17	-0.24	0.65	-0.40	-0.57
-0.98	-0.67	-0.42	0.04	0.23	-0.68	-0.65
0.23	-0.30	0.65	0.46	1.01	0.88	-0.31
-0.82	-0.41	-0.74	-1.14	0.55	-0.75	-0.57
-0.74	0.06	0.59	-0.52	0.40	-0.57	-0.57
-0.32	-0.90	-0.66	-0.27	0.32	-0.70	-0.49
3.12	-0.74	-0.92	0.02	0.21	0.91	1.14
0.25	1.00	0.18	0.31	-0.07	0.76	0.02
1.17	0.70	0.00	0.22	-0.18	1.94	0.10
1.04	0.13	-0.57	-0.67	-0.77	2.97	0.77
3.62	-0.78	-1.16	0.60	0.77	0.25	0.53
-0.70	-1.04	-0.95	-0.01	-0.22	-0.26	-0.61
0.08	-0.79	-0.94	-0.18	-0.58	-0.72	-0.38
0.59	-0.82	-1.03	0.28	-0.11	-0.19	0.69
-0.68	-0.20	-0.22	2.75	-0.38	-0.59	-0.14
2.16	-0.48	-0.75	1.74	0.26	0.24	0.02
-0.32	7.04	0.40	0.80	0.43	0.13	1.40
-0.64	-1.10	-0.30	4.05	-1.02	-0.34	-0.39
1.01	0.12	0.84	3.41	0.07	1.83	0.36
-0.59	-0.35	-0.90	1.24	0.15	-0.39	-0.49
0.22	1.34	-0.39	-1.31	1.49	-0.62	0.49
0.08	-1.13	0.11	0.15	-0.51	-0.71	-0.32
1.05	-0.70	0.69	0.58	-0.82	0.40	1.01
-1.42	-0.71	5.89	-1.85	-1.38	-0.94	-0.71
0.30	-0.85	0.10	-0.09	0.02	-0.51	-0.29
0.15	2.53	1.10	-0.41	0.87	0.60	1.03
-0.21	3.15	-0.49	-1.23	0.81	-0.58	0.87
1.07	-0.71	1.73	1.72	0.85	1.76	0.96

1.18	0.23	0.81	-0.78	0.40	1.95	0.48
-0.51	-0.60	-0.94	-0.68	-0.41	-0.30	-0.52
0.15	0.17	0.44	-0.43	0.29	0.64	0.20
-0.74	-0.72	-0.47	-1.32	-0.55	-0.79	-0.57
1.32	-0.14	1.20	-0.58	0.88	1.97	1.68
0.86	-1.22	0.08	-0.40	0.86	0.19	0.83
0.40	-0.10	-0.09	-0.99	-0.61	0.30	-0.44
0.23	-0.15	0.35	-0.36	0.41	0.48	-0.11
-0.26	-0.70	-0.73	-0.29	0.05	-0.40	-0.48
1.24	0.94	2.00	-0.14	3.18	0.80	0.85
0.09	-0.35	0.09	-0.45	-0.07	1.24	-0.21
0.14	-0.93	0.77	0.38	1.13	1.03	3.24
-0.79	0.07	0.46	-0.40	3.98	-0.79	-0.56
-0.57	-0.18	1.47	-0.29	0.59	-0.55	-0.55
0.58	0.37	-1.15	0.40	2.75	0.23	0.45
2.62	0.05	-0.87	1.86	1.03	0.15	-0.05
-1	-1	-1	-1	1	-1	0
0.00	0.00	0.00	0.00	0.00	0.00	0.00
1.00	1.00	1.00	1.00	1.00	1.00	1.00
0.92	0.61	0.50	0.27	0.51	0.75	0.61

19	20	21	22	23	24	25
CAAAGAATTCTC(	CACTAGATTGTG/	ACTGGACTTGG/	CAAAGTGCTGT'	TCAGTGCACTAC/	TGTCAGTTTGTC/	TGAGAACTGAA'
miR-186-5p	miR-28-3p	miR-378a-3p	miR-93-5p	miR-148a-3p	miR-223-3p	miR-146b-5p
979581	903691	738230	679306	664224	624773	614674
0.09	0.42	0.38	1.24	0.38	-0.15	-0.58
0.03	3.14	-1.59	-1.25	-1.27	0.42	2.00
0.71	-0.33	-0.62	2.48	-0.65	-0.05	0.04
-0.64	-0.84	-0.58	1.64	-0.45	-0.48	-0.40
-0.43	-0.57	0.66	-0.94	-1.13	0.78	0.49
-1.04	0.22	-0.55	-1.32	1.18	-0.90	-0.40
-1.09	0.15	0.66	0.26	-0.16	-0.98	-0.68
-0.87	-0.99	-0.36	-0.80	-0.75	-0.87	-0.71
-0.22	-0.89	-0.95	-0.23	-0.85	-0.70	-0.23
0.51	-0.17	-0.60	0.26	-0.72	-0.59	0.81
1.40	1.11	-0.84	-0.93	0.10	1.52	3.99
-0.81	-0.82	-0.14	-1.03	-0.69	-0.97	-0.56
2.54	-0.26	2.12	0.95	2.66	0.75	0.27
-0.92	-1.04	-0.42	-0.38	-1.14	-0.78	-0.63
0.42	-0.22	1.33	1.27	-0.28	-0.41	-0.41
1.89	0.03	-0.23	-0.06	0.58	0.24	-0.20
-0.30	-0.02	1.83	0.22	-0.54	-0.62	-0.47
-0.46	-0.70	0.97	1.62	-0.70	0.30	-0.49
0.75	2.92	0.82	-0.44	2.30	3.13	0.20
1.09	-0.41	0.33	-0.54	0.81	1.28	0.70
-0.79	-0.01	-0.68	-0.27	0.86	-0.89	-0.37
-0.70	0.20	1.08	-0.39	0.22	-0.38	-0.63
-0.68	0.04	1.15	-0.20	0.12	-0.41	-0.62
-1.05	-0.70	-0.14	-0.71	-0.61	-0.98	-0.64
-0.94	0.09	-1.54	-1.27	-0.66	1.32	-0.41
-0.01	0.02	-0.70	0.19	0.20	0.96	-0.03
1.52	-0.36	-1.39	0.61	1.18	-0.54	-0.05
2.29	-0.75	-1.44	-0.21	0.64	1.00	1.67

1.37	6.39	-0.55	-0.53	4.72	0.89	2.81
-0.14	3.15	-0.50	0.28	0.93	-0.17	1.13
-1.00	0.86	-0.84	1.77	-0.75	-0.97	-0.57
1.28	7.78	-0.24	0.04	2.64	0.21	6.40
0.34	-1.27	-0.17	-1.04	-0.90	4.39	0.09
-0.63	0.17	0.23	0.09	0.24	0.45	-0.67
-0.36	-0.58	0.20	0.76	-0.44	0.49	-0.67
-0.10	0.44	1.05	-0.65	0.05	0.23	-0.36
-1.40	-1.95	-1.85	-1.72	-1.35	-1.10	-0.75
0.17	1.25	1.05	0.49	1.63	-0.44	0.30
2.29	0.68	1.70	2.86	1.31	1.40	0.25
0.28	0.13	1.69	-0.56	-0.62	0.80	0.03
-1.37	0.78	2.06	-1.02	-0.90	-0.92	-0.44
-1.42	0.78	9.02	-0.94	-0.86	-0.73	-0.04
-0.52	-0.02	-0.89	-0.17	-0.56	-0.45	-0.50
1.34	1.61	0.67	1.30	4.26	0.74	-0.41
-0.53	0.55	-0.22	-1.23	-0.77	-0.58	-0.39
-1.41	-0.43	-0.69	-1.70	-1.32	-1.03	-0.62
1.57	0.34	0.47	-0.60	1.09	3.98	0.48
-0.49	0.89	0.77	-0.63	0.62	-0.10	-0.44
0.40	-1.03	-0.55	-0.22	-0.59	0.46	-0.50
-0.09	-0.28	1.25	-0.56	-0.63	-0.19	-0.39
0.40	-0.53	-0.08	-0.42	-0.48	-0.33	-0.09
-0.69	0.20	0.17	-0.24	0.65	-0.40	-0.57
-0.98	-0.67	-0.42	0.04	0.23	-0.68	-0.65
0.23	-0.30	0.65	0.46	1.01	0.88	-0.31
-0.82	-0.41	-0.74	-1.14	0.55	-0.75	-0.57
-0.74	0.06	0.59	-0.52	0.40	-0.57	-0.57
-0.32	-0.90	-0.66	-0.27	0.32	-0.70	-0.49
3.12	-0.74	-0.92	0.02	0.21	0.91	1.14
0.25	1.00	0.18	0.31	-0.07	0.76	0.02
1.17	0.70	0.00	0.22	-0.18	1.94	0.10
1.04	0.13	-0.57	-0.67	-0.77	2.97	0.77
3.62	-0.78	-1.16	0.60	0.77	0.25	0.53
-0.70	-1.04	-0.95	-0.01	-0.22	-0.26	-0.61
0.08	-0.79	-0.94	-0.18	-0.58	-0.72	-0.38
0.59	-0.82	-1.03	0.28	-0.11	-0.19	0.69
-0.68	-0.20	-0.22	2.75	-0.38	-0.59	-0.14
2.16	-0.48	-0.75	1.74	0.26	0.24	0.02
-0.32	7.04	0.40	0.80	0.43	0.13	1.40
-0.64	-1.10	-0.30	4.05	-1.02	-0.34	-0.39
1.01	0.12	0.84	3.41	0.07	1.83	0.36
-0.59	-0.35	-0.90	1.24	0.15	-0.39	-0.49
0.22	1.34	-0.39	-1.31	1.49	-0.62	0.49
0.08	-1.13	0.11	0.15	-0.51	-0.71	-0.32
1.05	-0.70	0.69	0.58	-0.82	0.40	1.01
-1.42	-0.71	5.89	-1.85	-1.38	-0.94	-0.71
0.30	-0.85	0.10	-0.09	0.02	-0.51	-0.29
0.15	2.53	1.10	-0.41	0.87	0.60	1.03
-0.21	3.15	-0.49	-1.23	0.81	-0.58	0.87
1.07	-0.71	1.73	1.72	0.85	1.76	0.96
1.18	0.23	0.81	-0.78	0.40	1.95	0.48
-0.51	-0.60	-0.94	-0.68	-0.41	-0.30	-0.52
0.15	0.17	0.44	-0.43	0.29	0.64	0.20
-0.74	-0.72	-0.47	-1.32	-0.55	-0.79	-0.57
1.32	-0.14	1.20	-0.58	0.88	1.97	1.68
0.86	-1.22	0.08	-0.40	0.86	0.19	0.83
0.40	-0.10	-0.09	-0.99	-0.61	0.30	-0.44
0.23	-0.15	0.35	-0.36	0.41	0.48	-0.11

-0.26	-0.70	-0.73	-0.29	0.05	-0.40	-0.48
1.24	0.94	2.00	-0.14	3.18	0.80	0.85
0.09	-0.35	0.09	-0.45	-0.07	1.24	-0.21
0.14	-0.93	0.77	0.38	1.13	1.03	3.24
-0.79	0.07	0.46	-0.40	3.98	-0.79	-0.56
-0.57	-0.18	1.47	-0.29	0.59	-0.55	-0.55
0.58	0.37	-1.15	0.40	2.75	0.23	0.45
3	0	-1	2	1	0	0

19	20	21	22	23	24	25
64	15	27	38	78	118	119
CATAAAGTAGAAA	TGAGGTAGTAGAT	AAAAGCTGGGTT	TGAGAACTGAAT	CACCCGTAGAACC	ACAGTAGTCTGCA	ACAGTAGTCTGC
miR-142-5p	let-7f-5p	miR-320a	miR-146a-5p	miR-99b-5p	miR-199a-3p	miR-199b-3p
94911	1418336	549148	339140	56245	16186	16186
-0.26	-0.82	0.58	-0.41	-0.29	-0.55	-0.55
-0.08	-0.14	-1.10	2.17	4.80	-0.68	-0.68
-0.26	0.39	-0.21	-0.29	-0.35	-0.47	-0.47
-0.27	-0.35	-0.33	-0.40	-0.46	-0.58	-0.58
-0.21	0.34	-0.42	1.57	0.21	1.93	1.93
-0.24	-0.04	-0.44	-0.05	-0.42	-0.07	-0.07
-0.26	-0.60	1.04	-0.70	-0.42	0.30	0.30
-0.17	-0.86	-0.33	-0.70	-0.40	-0.86	-0.86
-0.26	-0.43	-0.97	-0.04	-0.28	-0.96	-0.96
-0.22	0.20	-0.77	0.64	-0.10	-0.85	-0.85
-0.16	0.23	-1.49	3.69	0.49	0.00	0.00
-0.28	0.08	0.13	-0.67	-0.41	-0.78	-0.78
0.10	3.89	-1.08	0.05	-0.43	2.25	2.25
-0.25	-0.85	0.48	-0.58	-0.19	-1.02	-1.02
-0.08	0.16	0.42	-0.50	-0.26	-0.64	-0.64
-0.06	1.33	-0.42	0.11	-0.25	2.80	2.80
-0.21	-0.87	0.79	-0.54	-0.19	-0.92	-0.92
-0.22	0.07	0.53	-0.72	-0.34	0.00	0.00
-0.23	-0.14	-1.11	0.03	-0.31	-0.66	-0.66
4.98	1.57	-0.49	-0.19	-0.19	0.90	0.90
-0.23	-0.13	-0.21	-0.25	-0.31	-0.15	-0.15
-0.27	-0.69	0.79	-0.74	-0.11	-0.20	-0.20
-0.24	-0.60	1.83	-0.62	-0.27	0.98	0.98
-0.23	-0.83	3.16	-0.69	-0.33	-0.17	-0.17
-0.21	-0.57	-0.15	-0.15	0.78	0.47	0.47
-0.21	-0.74	0.53	0.02	-0.03	-0.33	-0.33
0.03	0.41	-0.74	-0.03	0.07	0.26	0.26
7.70	3.67	-1.36	0.67	-0.43	1.89	1.89
-0.16	1.03	-1.48	2.56	-0.11	1.02	1.02
-0.25	-0.09	-1.36	1.77	-0.07	-0.86	-0.86
-0.24	-0.74	0.52	0.19	1.65	-0.98	-0.98
-0.20	1.39	-1.57	6.51	0.59	0.75	0.75
-0.11	1.59	-0.75	1.13	2.03	4.41	4.41
-0.23	-0.73	-0.26	-0.75	-0.51	-0.46	-0.46
-0.17	-0.67	-0.32	-0.69	-0.45	-0.52	-0.52
-0.08	-0.59	-0.49	-0.39	-0.44	-0.77	-0.77
-0.25	-0.90	0.52	-0.88	-0.48	-1.22	-1.22
-0.13	0.17	-1.15	0.07	-0.43	-0.34	-0.34
-0.06	1.16	-1.26	0.73	-0.28	0.64	0.64

-0.22	-0.28	-0.78	-0.39	-0.39	-0.61	-0.61
-0.24	-0.88	3.78	-0.58	-0.07	0.13	0.13
-0.19	-0.84	20.61	0.29	1.30	1.26	1.26
-0.16	-0.75	-0.32	-0.15	-0.09	-0.09	-0.09
-0.19	0.18	-1.18	-0.54	-0.20	2.03	2.03
-0.22	-0.66	-0.15	-0.46	-0.21	-0.76	-0.76
-0.16	-0.92	4.71	-0.70	-0.32	-0.54	-0.54
0.07	-0.02	-1.09	0.50	-0.14	0.93	0.93
-0.20	-0.40	0.45	-0.49	-0.39	0.16	0.16
-0.17	-0.28	-0.26	-0.60	-0.30	0.44	0.44
-0.13	-0.65	-0.22	-0.35	-0.41	-0.38	-0.38
-0.14	-0.19	-0.62	-0.01	-0.37	1.69	1.69
-0.20	-0.80	1.17	-0.31	0.21	0.17	0.17
-0.24	-0.70	1.00	-0.52	0.42	-0.17	-0.17
-0.24	0.01	0.23	-0.31	-0.02	2.07	2.07
-0.24	-0.31	0.22	-0.31	-0.20	0.18	0.18
-0.25	-0.81	0.28	-0.32	0.53	-0.43	-0.43
-0.24	-0.20	0.07	-0.22	0.32	0.58	0.58
4.81	2.56	-0.83	0.00	-0.27	1.95	1.95
-0.25	-0.59	1.87	-0.36	-0.21	-0.39	-0.39
-0.19	0.82	1.12	-0.08	-0.22	2.51	2.51
-0.06	0.45	0.80	0.97	-0.03	2.77	2.77
3.55	2.24	-0.97	-0.38	-0.29	0.92	0.92
-0.24	-0.81	0.87	-0.68	0.28	-0.93	-0.93
-0.25	-0.46	-0.15	-0.31	0.16	0.18	0.18
-0.21	0.15	-0.31	0.20	-0.18	-0.36	-0.36
-0.28	2.44	3.48	-0.22	-0.49	-0.55	-0.55
-0.17	-0.19	-1.09	0.76	-0.38	0.55	0.55
-0.24	0.05	-1.07	4.06	2.88	-0.84	-0.84
-0.28	0.77	3.76	-0.19	-0.44	0.80	0.80
-0.24	0.55	0.22	0.44	-0.33	1.87	1.87
-0.26	-0.36	-0.33	-0.46	-0.41	-0.45	-0.45
-0.01	4.99	-1.95	0.36	-0.54	2.16	2.16
-0.24	0.60	-0.66	-0.11	-0.40	-0.33	-0.33
-0.24	1.39	-0.61	0.39	-0.43	0.00	0.00
-0.25	-0.97	25.44	-0.70	-0.14	-1.12	-1.12
-0.22	1.68	0.66	-0.52	-0.41	0.77	0.77
-0.24	-0.07	-1.20	0.68	-0.16	-0.63	-0.63
-0.24	0.51	-1.21	0.92	-0.17	0.04	0.04
0.12	0.61	-0.67	0.49	-0.32	0.64	0.64
-0.04	0.28	-1.17	1.31	-0.12	0.69	0.69
-0.13	-0.77	-0.01	-0.50	0.18	-1.01	-1.01
-0.06	0.32	-0.50	0.72	-0.27	0.35	0.35
-0.18	-0.69	0.03	-0.69	-0.32	-0.33	-0.33
6.06	2.11	-0.62	-0.35	0.21	1.64	1.64
8.26	4.03	-1.11	-0.25	-0.42	0.28	0.28
-0.19	-0.64	0.11	-0.43	-0.01	-0.06	-0.06
-0.18	-0.06	-0.40	-0.21	-0.36	0.29	0.29
-0.20	-0.54	-0.14	-0.27	-0.36	-0.51	-0.51
-0.23	1.49	-1.03	-0.05	-0.40	0.13	0.13
-0.21	-0.46	-0.38	-0.31	0.19	-0.16	-0.16
-0.19	5.96	0.95	0.14	-0.35	0.02	0.02
-0.25	-0.58	-0.75	-0.61	-0.33	-0.59	-0.59
-0.21	-0.65	0.52	-0.63	-0.20	-0.18	-0.18
-0.24	0.08	-1.14	0.57	0.03	0.27	0.27
-0.22	-0.55	-0.36	0.26	-0.03	0.85	0.85
0.06	0.06	0.06	0.06	0.06	0.06	0.06
0.14	0.04	0.10	0.04	0.06	0.06	0.06

416015	1782397	2570804	371228	359180	111957	117100

29	30	31	32	33	34	35
29	30	1	2	3	4	5
G_34	G_8	A_1	A_13	A_14	A_15	A_17
UC	UC	CHR_NP	CHR_NP	CHR_NP	CHR_NP	CHR_NP
1225092	8081102	5806417	697662	2226184	4034201	718772
0.72	0.39	0.96	0.48	0.99	0.21	0.19
G_34	G_8	A_1	A_13	A_14	A_15	A_17
809077	6298705	3235613	326434	1867004	3922244	601672
84942	490594	524718	147840	177482	52963	22366
77391	190767	57861	35930	51767	20495	29235
40120	56160	19787	12104	6580	1299	1849
36859	144853	154427	16001	12349	7379	5916
48028	62452	40902	9346	6914	1958	1959
6252	159965	429461	10799	6151	691	3614
25442	105646	129967	19819	6870	944	3558
877	48162	239532	1527	2921	163	2170
6027	77934	125922	6157	4266	404	1038
7638	64617	89917	13201	15998	3982	7085
4804	24819	40910	5579	5618	2306	2495
6898	26015	27652	3134	2436	1260	877
4774	22981	47820	4281	1757	431	1073
5644	19273	22471	1945	2684	2259	900
754	16947	76438	4985	2226	199	1781
1146	3916	7077	1264	411	112	52
1626	17363	32560	3018	1229	106	957
1789	7192	16561	2248	2621	714	1153
2298	19340	32924	3758	1850	237	1112
2965	10682	11662	9783	6100	1083	3458
1450	3881	5082	1383	1406	359	487
2096	10495	10374	1314	1891	915	526
1725	11779	12831	5620	2161	199	1136
2819	3401	16942	2400	1130	56	484
1591	6662	30968	6796	3573	117	4142
1149	5975	7914	2005	975	141	139
2192	5029	3956	507	587	585	135
420	17452	20362	1135	2743	554	1838
1155	5432	14099	1375	1018	338	583
2163	7464	4525	1634	1050	476	473
1365	7197	4668	1176	613	83	382
2076	6021	7107	426	233	167	165
796	7148	25687	977	302	45	211
307	2763	39709	1253	256	39	320
1062	4958	9505	925	696	218	398
1773	5418	3867	554	984	787	356
882	7719	10370	2739	2759	690	2208
1205	4931	11825	3837	2951	380	2482
1744	3394	3680	3049	1325	876	1032
997	1500	2655	501	118	65	149
448	875	1553	897	502	298	168
1375	1340	1182	342	209	55	107
780	2474	2973	610	584	464	188
123	5969	15267	521	702	99	874
1351	1744	2875	753	768	109	319

672	2079	2339	738	377	94	183
1033	3511	4959	702	365	57	215
491	1780	1469	320	333	130	75
52	1090	16994	49	23	6	5
151	1755	8702	825	296	4	107
739	2748	1046	729	329	582	316
593	1203	1882	425	233	14	98
574	1525	951	283	357	204	128
175	1301	3760	1492	856	62	345
212	3705	2971	588	64	1	1
19	870	12278	90	41	4	26
488	1698	2601	272	171	55	78
405	1378	1454	160	92	6	26
690	766	1205	333	316	56	73
775	1354	724	416	324	226	110
529	1009	1246	183	110	40	66
296	895	966	257	174	16	88
38	2086	19396	149	96	10	123
23	397	13240	34	12	4	7
61	1002	688	146	319	563	95
236	1815	3256	192	176	63	79
185	1317	1778	88	33	4	39
107	2009	4922	91	48	19	86
206	370	890	114	113	11	44
124	2669	2210	262	1494	734	626
246	370	253	126	92	37	26
93	305	737	123	232	151	50
38	1552	6757	298	161	16	145
80	836	1240	206	314	36	50
334	596	1151	269	87	4	19
229	1033	629	195	83	62	75
181	1372	714	261	182	11	9
257	1310	364	191	208	288	149
311	451	322	63	58	19	13
250	283	255	78	40	21	11
108	631	1758	103	78	26	33
138	280	277	79	31	6	3
90	500	497	192	228	231	83
58	184	1045	124	33	4	21
226	1087	1734	183	116	21	129
103	377	318	27	21	4	5
135	291	282	99	130	95	29
43	849	3254	92	29	5	23
66	324	480	228	94	16	35
212	402	518	100	23	19	41
79	184	201	58	116	14	77
1	171	4097	1	3	2	0
81	155	313	78	21	1	8
68	786	703	28	24	2	9
24	333	1680	27	16	2	8
32	196	801	89	20	1	12
71	196	115	81	46	24	19
68	256	155	55	64	31	22
208	205	151	172	104	18	46
21	495	1066	22	15	3	7
77	250	225	41	60	11	19
83	371	266	50	19	4	18
183	123	200	83	18	4	5
91	577	1037	134	84	20	73

25	560	2176	95	77	10	39
18	438	1580	47	44	7	37
115	83	110	48	27	8	17
139	251	184	113	50	18	29
39	301	1390	37	19	4	10
58	408	709	22	21	3	12
42	165	387	97	45	4	22
72	118	124	49	33	15	10
160	181	128	36	35	5	4
33	484	1041	40	53	19	40
126	69	212	31	8	1	3
71	169	308	37	16	11	9
41	215	908	32	20	10	22
31	209	577	63	13	3	17
31	209	577	63	13	3	17
97	171	197	29	28	5	9
52	102	133	15	10	1	3
28	48	101	36	32	6	8
44	664	466	56	27	4	10
16	144	278	68	53	4	26
15	111	976	5	1	0	0
22	98	114	32	8	6	5
106	99	122	29	19	3	6
29	126	108	16	15	3	4
21	71	99	40	46	33	31
42	123	70	18	6	1	2
7	334	83	19	20	13	1
32	148	81	47	10	1	8
40	245	382	44	21	2	14
26	32	78	15	9	7	5
14	154	604	45	7	0	2
53	105	93	15	17	3	7
18	133	114	6	6	1	3
21	122	247	10	12	3	4
18	126	289	15	5	1	4
33	76	52	9	4	0	3
25	126	166	34	13	2	2
10	61	51	6	11	8	1
24	80	39	5	3	1	0
47	90	193	48	18	3	4
13	68	632	1	1	0	0
10	93	55	4	7	10	5
19	31	44	23	10	4	2
17	74	131	30	13	2	3
17	102	76	23	20	13	4
31	71	68	12	15	6	3
14	34	105	35	8	1	3
14	155	207	9	12	4	5
8	220	314	22	3	0	5
21	43	59	18	13	2	1
9	8	27	0	0	0	0
21	59	57	6	35	46	4
46	119	72	20	31	55	23
28	20	29	5	7	10	2
28	28	43	11	3	1	1
4	76	166	5	7	1	1
2	37	646	6	1	1	2
34	31	22	7	6	0	0
12	79	11	7	7	8	4

17	71	217	21	8	1	5
31	51	79	20	8	0	5
14	54	63	9	8	2	4
31	35	30	11	16	2	7
6	13	26	9	5	3	2
8	67	337	10	2	0	1
4	65	109	9	13	9	2
14	16	20	3	1	0	1
10	92	173	13	8	1	4
24	60	44	22	21	4	13
16	48	31	17	7	1	2
8	71	268	42	6	0	2
15	45	25	6	7	3	0
11	48	35	15	9	19	0
14	60	99	17	9	0	3
10	16	35	3	16	1	4
31	47	24	6	0	1	2
17	102	62	6	3	5	0
6	103	217	9	7	1	4
17	52	24	20	9	14	4
6	88	141	16	6	1	9
10	32	27	12	20	22	1
16	30	37	5	3	2	2
6	95	201	9	7	1	4
10	36	36	11	14	12	10
30	43	35	3	1	3	1
16	73	42	13	3	1	2
85	59	17	13	0	0	0
3	103	90	8	5	0	10
13	36	72	6	7	5	1
8	29	16	23	7	14	6
6	103	46	14	21	11	4
34	41	70	17	1	4	3
1	28	199	30	1	0	0
6	73	60	3	9	10	2
7	28	24	4	6	3	1
6	63	140	5	8	1	5
16	49	33	12	4	0	2
9	35	24	34	28	0	10
17	43	25	9	4	0	1
7	41	16	7	10	3	4
6	80	194	5	7	2	5
6	67	36	7	1	2	0
10	41	36	3	5	4	1
17	47	17	8	2	0	2
32	54	141	4	5	3	0
12	44	48	0	2	1	1
11	10	30	13	7	1	3
9	23	16	15	13	0	3
2	131	221	24	8	1	8
11	41	12	2	2	1	0
2	70	31	2	0	0	0
7	40	48	10	1	0	2
12	49	53	12	6	0	1
5	39	44	2	1	0	0
0	14	288	0	2	0	0
11	52	53	5	6	1	1
6	56	84	18	2	1	1
9	36	26	4	5	0	0

8	14	54	7	4	0	6
11	12	20	2	4	2	1
7	104	50	18	3	0	1
14	8	8	3	0	0	0
11	18	19	3	0	0	0
5	16	27	0	1	0	0
5	14	22	4	1	6	0
6	8	40	7	7	1	2
8	26	171	6	2	1	6
4	24	14	7	8	5	8
0	22	223	1	0	0	0
4	16	21	2	4	1	0
1	31	72	2	0	0	0
5	29	22	0	9	1	3
5	12	16	4	2	0	0
8	17	41	8	1	0	1
5	38	86	14	2	0	2
5	19	16	2	3	0	0
2	29	17	5	3	1	1
4	10	19	2	1	0	0
4	21	92	14	3	0	2
4	22	31	11	9	0	2
4	22	31	11	9	0	2
4	12	10	1	0	4	2
1	8	11	3	0	1	1
8	30	27	0	1	2	0
9	30	21	2	0	1	0
7	12	15	5	3	0	2
6	15	17	3	0	0	2
3	4	17	5	6	3	6
4	22	33	1	2	0	1
8	9	15	11	2	0	0
4	12	29	12	1	0	2
31	4	7	11	2	0	0
4	11	22	0	0	0	0
4	6	24	3	5	1	1
0	9	149	0	0	0	0
7	12	13	3	3	1	0
3	29	47	16	3	0	1
8	7	9	1	1	0	0
1	19	13	1	2	4	0
3	22	42	15	3	0	1
6	11	12	2	1	1	1
2	22	9	3	0	0	0
2	34	31	0	4	1	1
6	15	6	2	0	1	0
0	4	113	0	1	0	0
6	8	14	3	0	0	0
2	6	9	1	1	0	0
1	3	27	2	0	1	1
2	10	11	0	0	0	0
4	30	13	4	2	0	0
0	9	39	0	0	0	0
15	14	17	5	4	1	2
1	9	14	2	2	0	2
0	0	3	4	72	0	0
3	15	13	2	0	1	1
0	8	13	9	1	3	2
6	12	11	4	4	4	2

10	16	9	3	0	3	0
4	10	3	2	1	1	0
4	24	18	3	3	1	2
1	15	8	5	0	0	2
2	4	8	4	2	1	2
7	10	11	4	3	0	0
0	8	11	2	1	0	2
6	10	9	2	0	0	0
0	78	58	0	2	0	1
0	10	72	4	0	0	0
3	28	20	4	5	0	3
5	10	5	0	1	0	1
3	19	31	2	2	2	1
7	1	3	0	0	0	0
5	5	12	2	1	0	0
2	3	39	7	0	0	2
4	19	20	2	0	6	3
7	10	12	2	1	0	1
1	4	11	2	0	1	1
1	36	15	2	0	0	0
2	8	5	0	3	0	0
4	2	15	3	0	1	1
3	3	0	1	0	0	0
4	13	4	1	0	5	0
1	7	13	5	3	0	0
4	2	3	0	0	0	0
1	2	2	2	0	0	0
2	3	7	1	1	0	0
6	5	5	4	2	0	0
8	7	10	2	0	0	0
4	8	3	0	1	2	0
1	14	52	4	3	0	3
0	20	56	2	2	0	0
1	19	7	2	0	0	0
3	19	26	3	2	3	1
1	5	4	0	2	4	0
4	7	1	2	1	1	0
1	2	5	2	1	0	1
0	2	0	2	0	0	1
1	3	11	1	1	0	0
2	5	71	0	1	0	1
3	3	3	0	2	1	0
2	13	5	0	0	0	0
1	4	6	1	3	1	0
2	13	13	3	2	0	4
0	1	0	0	0	0	0
1	5	8	1	0	2	0
1	2	14	3	0	0	0
2	4	4	0	0	2	0
2	7	6	1	0	0	0
0	13	4	3	0	0	0
1	5	33	0	0	0	1
1	3	2	2	2	1	0
0	14	28	6	2	0	1
1	8	5	0	1	0	0
1	1	0	0	1	0	0
0	2	4	3	0	2	0
0	22	4	1	0	0	0
2	9	1	3	4	0	0

2	5	11	2	6	1	1
1	2	2	0	0	0	0
0	2	1	1	0	0	0
1	1	6	1	2	0	0
2	5	0	3	2	2	1
4	5	3	1	1	0	0
2	7	2	0	0	0	0
1	23	61	2	0	1	4
0	9	8	2	0	0	0
0	3	3	3	1	0	0
2	3	5	2	0	0	0
2	5	5	1	0	0	0
1	0	9	3	0	1	2
1	10	23	0	0	0	2
1	0	7	0	1	1	0
2	9	11	2	0	0	0
3	5	13	1	1	0	0
0	5	5	1	1	0	1
2	8	26	1	0	0	1
1	8	7	2	0	1	0
1	1	8	1	2	0	0
1	2	8	4	2	0	0
4	3	5	1	0	0	0
1	2	1	2	2	0	1
0	8	10	0	1	3	1
5	1	2	2	0	1	1
0	4	23	2	0	0	0
1	4	12	1	0	0	0
3	2	0	1	0	0	0
0	0	2	0	0	0	0
0	4	2	8	1	0	1
2	3	4	1	0	0	0
1	1	8	0	1	1	0
0	1	2	3	1	0	0
0	6	14	0	0	0	0
1	6	3	0	0	1	1
0	4	1	4	0	0	1
0	1	5	0	0	0	0
2	1	3	1	0	0	0
1	1	4	4	0	0	0
2	1	2	5	0	0	2
1	1	3	1	0	0	0
2	4	6	4	2	0	0
0	2	8	1	0	0	0
4	3	1	0	1	0	0
5	7	4	0	0	0	0
1	1	2	0	0	0	0
0	3	1	2	2	0	0
0	6	5	0	0	0	0
0	0	0	0	1	0	0
2	2	5	1	0	0	0
1	5	8	4	0	0	0
0	6	9	0	0	0	0
1	1	2	1	2	0	3
1	2	5	0	1	0	0
0	13	10	1	5	1	9
0	0	2	1	0	0	0
1	4	4	0	0	0	0
2	0	2	0	0	0	0

0	4	5	0	0	0	0
0	0	2	0	0	0	0
0	0	6	1	2	0	0
1	0	12	0	0	0	0
3	4	4	0	1	0	0
0	2	2	0	0	1	1
0	0	0	0	0	0	0
1	3	13	2	0	0	0
1	2	0	0	3	1	0
1	3	3	0	0	0	0
0	0	24	1	0	0	1
0	1	18	0	0	0	0
0	1	17	0	0	0	0
0	8	2	1	0	0	0
0	4	2	1	2	0	0
1	1	0	1	0	0	0
0	1	3	1	0	0	0
1	4	2	0	0	0	0
0	2	40	0	0	0	0
0	3	6	0	0	0	1
0	2	4	1	0	0	1
0	1	18	1	0	0	0
1	5	3	0	0	0	0
0	0	0	0	0	0	0
0	3	1	0	0	0	0
0	2	10	0	0	0	0
0	0	10	0	0	0	0
0	1	2	0	0	0	0
0	0	1	0	0	0	0
0	1	4	1	1	0	0
0	1	18	0	0	0	0
0	6	4	0	1	0	0
1	2	1	0	1	0	0
0	1	2	0	0	0	0
2	0	8	2	0	0	1
1	1	0	1	1	0	1
0	0	4	0	1	0	0
0	4	3	1	0	0	0
1	3	8	0	0	0	0
0	4	4	1	1	0	0
0	8	41	0	0	0	0
3	1	3	0	0	0	0
2	2	0	0	1	0	0
0	3	3	0	0	0	0
0	1	0	0	0	0	0
0	5	8	0	0	0	0
0	9	11	0	0	0	0
1	2	0	0	2	0	0
0	2	0	0	0	0	0
0	1	2	0	1	0	0
1	4	4	1	2	0	0
0	3	7	0	0	0	1
1	5	2	0	0	0	0
0	1	6	2	0	0	0
0	2	2	0	0	0	0
0	1	2	3	0	0	0
2	4	2	0	0	0	0
1	4	0	0	0	0	0
1	5	0	0	0	0	0

0	0	1	0	0	0	0
1	1	1	0	0	0	0
0	1	2	3	1	0	0
0	0	1	0	1	0	0
0	7	2	0	0	0	0
0	2	6	0	0	0	0
0	0	2	0	0	0	0
0	2	17	1	0	0	0
1	0	1	0	0	0	1
0	1	8	0	0	0	1
0	0	6	1	0	0	0
0	8	2	0	0	0	0
0	3	3	0	0	0	0
0	0	3	1	0	0	0
0	4	7	0	0	0	0
0	1	3	0	0	1	0
1	0	0	2	0	0	0
0	10	2	0	1	0	0
0	2	12	1	1	0	0
1	0	0	0	1	0	0
0	0	1	0	0	0	0
0	2	13	1	0	0	0
0	3	14	0	0	0	1
1	0	0	0	0	0	0
1	0	0	1	0	0	0
0	2	2	0	0	0	0
1	0	0	0	1	0	0
0	1	1	0	0	0	0
0	3	12	0	0	0	0
1	0	10	0	1	0	0
0	3	6	0	0	0	0
0	1	1	0	0	0	0
0	3	3	1	0	0	0
1	0	0	1	0	0	0
0	2	0	0	0	0	0
0	2	3	1	0	0	0
0	1	2	0	0	0	0
0	1	4	0	0	0	1
0	3	0	1	0	0	0
1	1	1	0	0	0	1
0	3	5	0	0	1	0
0	3	0	0	0	0	0
0	0	1	1	0	0	0
1	3	0	2	0	0	0
1	0	0	0	0	0	1
0	1	2	0	1	0	0
1	2	1	0	0	0	2
0	3	1	0	1	0	0
1	2	1	0	0	1	0
0	2	0	0	0	0	0
0	0	1	0	1	0	0
0	1	11	0	0	0	0
0	0	2	0	0	0	0
0	0	9	1	0	0	0
0	2	1	1	0	0	0
1	0	2	1	0	0	0
0	1	1	1	0	0	0
0	0	0	0	0	0	0
0	0	5	0	0	0	0

0	0	2	0	0	0	0
0	0	2	0	0	2	0
0	0	1	0	1	0	0
0	4	4	0	0	0	0
0	0	1	0	0	0	0
0	0	3	0	0	0	0
1	2	1	0	0	0	0
1	0	0	0	0	1	0
0	1	0	0	0	0	0
0	1	0	0	1	0	0
1	2	0	0	0	0	0
0	3	4	1	0	0	0

0	0	0	0	0	1	1
G_34	G_8	A_1	A_13	A_14	A_15	A_17
UC	UC	CHR_NP	CHR_NP	CHR_NP	CHR_NP	CHR_NP

809077	6298705	3235613	326434	1867004	3922244	601672
21	25	18	15	26	30	26
-0.07	0.68	-0.65	-1.28	1.04	1.86	1.08

0.15	0.02	0.31	0.94	0.02	0.54	0.27
25	38	22	5	36	18	24
1	1	1	1	1	1	1

29	30	31	32	33	34	35
2	3	4	5	6	7	8
A_13	A_14	A_15	A_17	B_30	B_33	C_2
CHR_NP	CHR_NP	CHR_NP	CHR_NP	CHR_NP	CHR_NP	CHR_NP
371228	359180	111957	117100	148505	1922117	685397
A_13	A_14	A_15	A_17	B_30	B_33	C_2
147840	177482	52963	22366	4948	1007604	324840
35930	51767	20495	29235	27066	82363	53864
12104	6580	1299	1849	6598	53526	26948
16001	12349	7379	5916	19460	105395	30924
9346	6914	1958	1959	4335	185758	52795
10799	6151	691	3614	21454	22114	14962
19819	6870	944	3558	15329	42853	11026
1527	2921	163	2170	2182	4727	8808
6157	4266	404	1038	731	14989	4841
13201	15998	3982	7085	3504	41754	23520
5579	5618	2306	2495	1040	18898	8215
3134	2436	1260	877	1737	26780	9268
4281	1757	431	1073	2414	9346	2814
1945	2684	2259	900	1939	29210	14032
4985	2226	199	1781	2405	3647	1546
1264	411	112	52	145	33307	3175
3018	1229	106	957	2972	4989	1148
2248	2621	714	1153	1417	8203	5081
3758	1850	237	1112	933	6588	2949

9783	6100	1083	3458	438	14685	3594
1383	1406	359	487	623	10198	3542
1314	1891	915	526	355	9348	4198
5620	2161	199	1136	195	8208	1746
2400	1130	56	484	2483	9903	3570
6796	3573	117	4142	618	960	351
2005	975	141	139	78	8786	2119
507	587	585	135	387	7245	2471
1135	2743	554	1838	209	3111	2406
1375	1018	338	583	1384	1840	832
1634	1050	476	473	118	5546	1298
1176	613	83	382	712	6198	2874
426	233	167	165	698	5805	1680
977	302	45	211	323	2251	767
1253	256	39	320	235	3245	1664
925	696	218	398	1268	1654	759
554	984	787	356	3972	9791	8590
2739	2759	690	2208	1051	2501	1914
3837	2951	380	2482	858	1145	524
3049	1325	876	1032	764	7023	2647
501	118	65	149	126	5223	653
897	502	298	168	602	13459	3447
342	209	55	107	64	2501	1026
610	584	464	188	608	5028	1644
521	702	99	874	608	572	534
753	768	109	319	314	1352	1203
738	377	94	183	93	3818	1085
702	365	57	215	134	1371	356
320	333	130	75	71	4788	1628
49	23	6	5	35	117	59
825	296	4	107	32	4093	805
729	329	582	316	129	1251	597
425	233	14	98	829	1597	1573
283	357	204	128	186	2136	1799
1492	856	62	345	89	2497	468
588	64	1	1	3	2771	336
90	41	4	26	5	119	63
272	171	55	78	374	1787	972
160	92	6	26	128	3593	697
333	316	56	73	77	1228	427
416	324	226	110	1831	389	166
183	110	40	66	177	1864	741
257	174	16	88	59	1616	701
149	96	10	123	78	105	93
34	12	4	7	17	92	58
146	319	563	95	42	1652	403
192	176	63	79	186	639	285
88	33	4	39	59	413	787
91	48	19	86	116	270	106
114	113	11	44	115	928	884
262	1494	734	626	34	375	377
126	92	37	26	125	2651	534
123	232	151	50	86	1226	451
298	161	16	145	88	106	54
206	314	36	50	11	748	259
269	87	4	19	178	427	162
195	83	62	75	39	1006	272
261	182	11	9	1	493	138
191	208	288	149	418	104	84

63	58	19	13	94	1336	321
78	40	21	11	141	567	423
103	78	26	33	137	401	188
79	31	6	3	5	1161	369
192	228	231	83	117	593	318
124	33	4	21	8	213	258
183	116	21	129	41	336	134
27	21	4	5	25	818	532
99	130	95	29	48	983	385
92	29	5	23	67	135	41
228	94	16	35	92	802	140
100	23	19	41	175	231	36
58	116	14	77	9	186	87
1	3	2	0	4	21	21
78	21	1	8	2	799	96
28	24	2	9	24	100	52
27	16	2	8	14	121	65
89	20	1	12	66	261	94
81	46	24	19	23	513	187
55	64	31	22	15	514	204
172	104	18	46	13	164	88
22	15	3	7	37	65	29
41	60	11	19	39	688	362
50	19	4	18	23	117	47
83	18	4	5	24	145	67
134	84	20	73	73	90	49
95	77	10	39	43	147	175
47	44	7	37	14	125	62
48	27	8	17	63	684	406
113	50	18	29	81	184	146
37	19	4	10	40	170	87
22	21	3	12	29	84	52
97	45	4	22	19	153	105
49	33	15	10	8	323	93
36	35	5	4	20	704	615
40	53	19	40	47	89	46
31	8	1	3	12	62	17
37	16	11	9	18	206	70
32	20	10	22	4	141	58
63	13	3	17	58	125	41
63	13	3	17	58	125	41
29	28	5	9	90	500	306
15	10	1	3	6	329	188
36	32	6	8	3	345	124
56	27	4	10	2	144	20
68	53	4	26	7	127	46
5	1	0	0	0	24	7
32	8	6	5	15	165	56
29	19	3	6	6	34	15
16	15	3	4	1	124	60
40	46	33	31	28	173	74
18	6	1	2	22	449	106
19	20	13	1	0	21	17
47	10	1	8	20	155	65
44	21	2	14	14	60	19
15	9	7	5	36	189	67
45	7	0	2	3	42	18
15	17	3	7	6	161	95
6	6	1	3	2	199	23

10	12	3	4	2	74	23
15	5	1	4	9	63	14
9	4	0	3	13	202	50
34	13	2	2	1	179	34
6	11	8	1	16	105	61
5	3	1	0	6	201	21
48	18	3	4	32	145	28
1	1	0	0	5	21	15
4	7	10	5	4	94	25
23	10	4	2	19	363	59
30	13	2	3	6	57	15
23	20	13	4	28	117	35
12	15	6	3	14	153	72
35	8	1	3	3	56	36
9	12	4	5	25	55	19
22	3	0	5	0	54	7
18	13	2	1	3	295	45
0	0	0	0	0	68	24
6	35	46	4	14	108	106
20	31	55	23	9	25	14
5	7	10	2	23	191	103
11	3	1	1	2	101	43
5	7	1	1	3	100	21
6	1	1	2	3	6	2
7	6	0	0	9	153	81
7	7	8	4	17	119	42
21	8	1	5	7	57	16
20	8	0	5	11	43	13
9	8	2	4	5	110	33
11	16	2	7	14	70	34
9	5	3	2	9	44	221
10	2	0	1	5	18	8
9	13	9	2	5	68	46
3	1	0	1	14	103	30
13	8	1	4	1	60	14
22	21	4	13	2	36	24
17	7	1	2	12	64	28
42	6	0	2	3	41	7
6	7	3	0	10	131	41
15	9	19	0	7	66	32
17	9	0	3	5	16	7
3	16	1	4	14	69	45
6	0	1	2	8	104	36
6	3	5	0	1	89	49
9	7	1	4	11	11	1
20	9	14	4	0	20	25
16	6	1	9	15	31	12
12	20	22	1	3	34	13
5	3	2	2	6	80	19
9	7	1	4	11	11	1
11	14	12	10	4	47	11
3	1	3	1	6	44	27
13	3	1	2	3	52	5
13	0	0	0	1	1	0
8	5	0	10	10	9	28
6	7	5	1	11	45	30
23	7	14	6	7	80	11
14	21	11	4	1	49	17
17	1	4	3	6	14	6

30	1	0	0	0	27	3
3	9	10	2	4	42	15
4	6	3	1	0	60	56
5	8	1	5	3	25	15
12	4	0	2	1	46	2
34	28	0	10	7	32	12
9	4	0	1	8	41	18
7	10	3	4	0	61	38
5	7	2	5	7	21	26
7	1	2	0	1	40	3
3	5	4	1	4	51	12
8	2	0	2	4	97	15
4	5	3	0	5	24	11
0	2	1	1	5	66	29
13	7	1	3	6	65	16
15	13	0	3	8	72	17
24	8	1	8	3	5	1
2	2	1	0	10	56	44
2	0	0	0	2	38	9
10	1	0	2	2	36	8
12	6	0	1	2	50	13
2	1	0	0	0	51	5
0	2	0	0	0	1	0
5	6	1	1	9	31	8
18	2	1	1	12	2	2
4	5	0	0	2	51	24
7	4	0	6	2	38	18
2	4	2	1	6	34	25
18	3	0	1	8	26	2
3	0	0	0	1	24	7
3	0	0	0	1	15	8
0	1	0	0	1	44	8
4	1	6	0	4	34	19
7	7	1	2	5	23	20
6	2	1	6	7	5	10
7	8	5	8	1	19	5
1	0	0	0	3	3	0
2	4	1	0	0	39	14
2	0	0	0	0	12	2
0	9	1	3	1	12	10
4	2	0	0	0	9	5
8	1	0	1	9	31	14
14	2	0	2	1	28	7
2	3	0	0	3	27	9
5	3	1	1	2	22	7
2	1	0	0	0	32	8
14	3	0	2	7	8	0
11	9	0	2	1	19	12
11	9	0	2	1	19	12
1	0	4	2	1	12	5
3	0	1	1	4	47	19
0	1	2	0	3	8	9
2	0	1	0	1	12	6
5	3	0	2	4	25	3
3	0	0	2	3	65	5
5	6	3	6	3	4	7
1	2	0	1	3	16	9
11	2	0	0	1	3	5
12	1	0	2	11	36	18

11	2	0	0	4	19	8
0	0	0	0	1	34	7
3	5	1	1	5	12	14
0	0	0	0	1	0	1
3	3	1	0	3	25	9
16	3	0	1	2	4	2
1	1	0	0	2	10	10
1	2	4	0	3	16	17
15	3	0	1	1	1	2
2	1	1	1	6	6	2
3	0	0	0	5	9	4
0	4	1	1	1	7	2
2	0	1	0	3	21	4
0	1	0	0	0	3	4
3	0	0	0	0	7	1
1	1	0	0	2	25	11
2	0	1	1	1	28	6
0	0	0	0	1	27	3
4	2	0	0	3	7	5
0	0	0	0	0	0	1
5	4	1	2	4	11	20
2	2	0	2	1	13	6
4	72	0	0	0	7	9
2	0	1	1	0	15	6
9	1	3	2	0	5	5
4	4	4	2	6	21	7
3	0	3	0	3	24	11
2	1	1	0	2	16	7
3	3	1	2	0	7	1
5	0	0	2	1	22	9
4	2	1	2	1	19	5
4	3	0	0	2	20	1
2	1	0	2	1	13	3
2	0	0	0	3	18	8
0	2	0	1	1	0	0
4	0	0	0	2	3	6
4	5	0	3	1	2	0
0	1	0	1	0	19	14
2	2	2	1	0	12	11
0	0	0	0	0	17	6
2	1	0	0	1	7	3
7	0	0	2	1	6	3
2	0	6	3	3	9	4
2	1	0	1	0	8	3
2	0	1	1	1	16	4
2	0	0	0	0	7	3
0	3	0	0	0	11	1
3	0	1	1	3	12	2
1	0	0	0	0	32	7
1	0	5	0	0	10	7
5	3	0	0	0	8	1
0	0	0	0	1	14	5
2	0	0	0	0	9	3
1	1	0	0	0	10	4
4	2	0	0	0	36	12
2	0	0	0	0	10	0
0	1	2	0	0	12	4
4	3	0	3	1	3	0
2	2	0	0	1	1	1

2	0	0	0	0	13	2
3	2	3	1	3	4	7
0	2	4	0	1	18	15
2	1	1	0	0	13	7
2	1	0	1	0	14	10
2	0	0	1	1	4	0
1	1	0	0	1	17	3
0	1	0	1	2	2	1
0	2	1	0	1	15	2
0	0	0	0	0	9	0
1	3	1	0	1	14	5
3	2	0	4	0	5	0
0	0	0	0	4	0	2
1	0	2	0	0	19	2
3	0	0	0	1	2	2
0	0	2	0	0	13	4
1	0	0	0	2	16	4
3	0	0	0	0	10	2
0	0	0	1	0	1	0
2	2	1	0	1	24	5
6	2	0	1	4	1	1
0	1	0	0	2	10	2
0	1	0	0	1	3	4
3	0	2	0	0	30	2
1	0	0	0	0	0	0
3	4	0	0	1	3	5
2	6	1	1	0	12	2
0	0	0	0	0	14	5
1	0	0	0	0	2	3
1	2	0	0	1	18	10
3	2	2	1	0	12	2
1	1	0	0	0	1	1
0	0	0	0	1	5	3
2	0	1	4	0	2	0
2	0	0	0	1	4	2
3	1	0	0	0	7	6
2	0	0	0	1	19	6
1	0	0	0	0	3	2
3	0	1	2	21	15	4
0	0	0	2	0	1	1
0	1	1	0	2	5	1
2	0	0	0	0	6	5
1	1	0	0	0	2	2
1	1	0	1	0	11	2
1	0	0	1	0	3	5
2	0	1	0	2	6	4
1	2	0	0	0	9	1
4	2	0	0	0	14	3
1	0	0	0	0	0	0
2	2	0	1	0	6	4
0	1	3	1	1	3	5
2	0	1	1	0	0	0
2	0	0	0	0	1	1
1	0	0	0	2	2	0
1	0	0	0	0	9	4
0	0	0	0	0	19	3
8	1	0	1	0	1	0
1	0	0	0	2	6	1
0	1	1	0	0	3	0

3	1	0	0	0	0	0
0	0	0	0	0	2	1
0	0	1	1	0	4	2
4	0	0	1	0	5	5
0	0	0	0	0	5	7
1	0	0	0	1	11	2
4	0	0	0	0	10	2
5	0	0	2	0	8	1
1	0	0	0	0	0	0
4	2	0	0	0	2	2
1	0	0	0	0	7	0
0	1	0	0	0	1	1
0	0	0	0	1	13	8
0	0	0	0	0	1	0
2	2	0	0	0	5	3
0	0	0	0	0	3	2
0	1	0	0	0	2	0
1	0	0	0	0	11	0
4	0	0	0	0	3	0
0	0	0	0	0	0	3
1	2	0	3	0	14	6
0	1	0	0	0	3	0
1	5	1	9	0	1	2
1	0	0	0	0	4	0
0	0	0	0	0	2	5
0	0	0	0	0	22	1
0	0	0	0	1	0	1
0	0	0	0	0	8	2
1	2	0	0	0	0	0
0	0	0	0	0	0	0
0	1	0	0	0	1	2
0	0	1	1	0	3	2
0	0	0	0	0	4	6
2	0	0	0	0	1	0
0	3	1	0	0	3	0
0	0	0	0	0	2	0
1	0	0	1	0	0	2
0	0	0	0	0	0	0
0	0	0	0	0	0	0
1	0	0	0	0	6	0
1	2	0	0	0	0	1
1	0	0	0	0	2	2
1	0	0	0	1	2	2
0	0	0	0	0	2	1
0	0	0	0	0	0	0
0	0	0	1	1	1	0
1	0	0	1	0	2	2
1	0	0	0	0	1	6
0	0	0	0	1	2	1
0	0	0	0	0	9	2
0	0	0	0	0	2	0
0	0	0	0	0	0	0
0	0	0	0	0	2	0
0	0	0	0	1	4	0
0	0	0	0	0	1	0
1	1	0	0	0	5	2
0	0	0	0	0	0	1
0	1	0	0	0	4	0
0	1	0	0	0	1	2

0	0	0	0	0	2	0
2	0	0	1	1	6	3
1	1	0	1	0	1	1
0	1	0	0	0	1	0
1	0	0	0	0	5	1
0	0	0	0	0	5	2
1	1	0	0	1	3	0
0	0	0	0	0	0	0
0	0	0	0	1	4	4
0	1	0	0	1	0	1
0	0	0	0	0	2	0
0	0	0	0	1	7	3
0	0	0	0	1	0	0
0	0	0	0	1	1	0
0	2	0	0	0	3	2
0	0	0	0	0	2	1
0	1	0	0	0	1	0
1	2	0	0	1	1	1
0	0	0	1	0	1	0
0	0	0	0	2	2	0
2	0	0	0	0	2	1
0	0	0	0	1	0	1
3	0	0	0	0	1	1
0	0	0	0	0	4	1
0	0	0	0	0	2	0
0	0	0	0	0	3	2
0	0	0	0	0	12	0
0	0	0	0	0	6	1
3	1	0	0	0	3	1
0	1	0	0	0	3	1
0	0	0	0	0	2	0
0	0	0	0	0	1	0
0	0	0	0	0	2	1
1	0	0	0	0	1	1
0	0	0	1	0	2	1
0	0	0	1	0	2	1
1	0	0	0	0	2	4
0	0	0	0	0	0	1
0	0	0	0	0	0	0
1	0	0	0	0	3	0
0	0	0	0	0	0	3
0	0	1	0	2	1	0
2	0	0	0	1	2	1
0	1	0	0	0	0	0
1	1	0	0	0	0	0
0	1	0	0	0	1	2
0	0	0	0	0	3	1
1	0	0	0	0	0	0
0	0	0	1	0	0	0
0	0	0	0	1	6	2
1	0	0	0	0	3	2
0	0	0	0	0	0	0
0	1	0	0	0	1	0
0	0	0	0	0	0	0
0	0	0	0	0	2	0
0	1	0	0	1	2	2
0	0	0	0	1	0	0
0	0	0	0	1	7	2
1	0	0	0	1	2	0

1	0	0	0	0	11	4
0	0	0	0	0	2	2
1	0	0	0	0	2	1
0	0	0	0	0	1	0
0	0	0	1	0	5	0
1	0	0	0	0	0	2
0	0	0	1	0	0	0
0	0	1	0	0	2	0
0	0	0	0	1	1	2
1	0	0	0	0	1	0
2	0	0	0	0	4	0
0	0	0	1	0	1	1
0	1	0	0	0	0	0
0	0	0	2	0	0	0
0	1	0	0	0	1	0
0	0	1	0	0	1	1
0	0	0	0	0	2	0
0	1	0	0	0	2	1
0	0	0	0	0	1	0
0	0	0	0	0	0	0
1	0	0	0	0	0	0
1	0	0	0	0	3	0
1	0	0	0	0	0	0
1	0	0	0	0	2	1
0	0	0	0	0	1	1
0	0	0	0	0	3	1
0	0	0	0	0	1	0
0	0	2	0	0	2	2
0	1	0	0	0	1	1
0	0	0	0	0	2	1
0	0	0	0	0	0	0
0	0	0	0	0	2	0
0	0	0	0	0	2	1
0	0	1	0	0	6	2
0	0	0	0	0	3	1
0	1	0	0	0	2	1
0	0	0	0	0	1	0
1	0	0	0	1	1	2

0.15	0.02	0.31	0.94	0.02	0.54	0.27
25	38	22	5	36	18	24
1	1	1	1	1	1	1
29	30	31	32	33	34	35
2	3	4	5	6	7	8
A_13	A_14	A_15	A_17	B_30	B_33	C_2
CHR_NP	CHR_NP	CHR_NP	CHR_NP	CHR_NP	CHR_NP	CHR_NP
371228	359180	111957	117100	148505	1922117	685397
CHR_NP	CHR_NP	CHR_NP	CHR_NP	CHR_NP	CHR_NP	CHR_NP
147840	177482	52963	22366	4948	1007604	324840
35930	51767	20495	29235	27066	82363	53864
12104	6580	1299	1849	6598	53526	26948
16001	12349	7379	5916	19460	105395	30924
9346	6914	1958	1959	4335	185758	52795

10799	6151	691	3614	21454	22114	14962
19819	6870	944	3558	15329	42853	11026
1527	2921	163	2170	2182	4727	8808
6157	4266	404	1038	731	14989	4841
13201	15998	3982	7085	3504	41754	23520
5579	5618	2306	2495	1040	18898	8215
3134	2436	1260	877	1737	26780	9268
4281	1757	431	1073	2414	9346	2814
1945	2684	2259	900	1939	29210	14032
4985	2226	199	1781	2405	3647	1546
1264	411	112	52	145	33307	3175
3018	1229	106	957	2972	4989	1148
2248	2621	714	1153	1417	8203	5081
3758	1850	237	1112	933	6588	2949
9783	6100	1083	3458	438	14685	3594
1383	1406	359	487	623	10198	3542
1314	1891	915	526	355	9348	4198
5620	2161	199	1136	195	8208	1746
2400	1130	56	484	2483	9903	3570
6796	3573	117	4142	618	960	351
2005	975	141	139	78	8786	2119
507	587	585	135	387	7245	2471
1135	2743	554	1838	209	3111	2406
1375	1018	338	583	1384	1840	832
1634	1050	476	473	118	5546	1298
1176	613	83	382	712	6198	2874
426	233	167	165	698	5805	1680
977	302	45	211	323	2251	767
1253	256	39	320	235	3245	1664
925	696	218	398	1268	1654	759
554	984	787	356	3972	9791	8590
2739	2759	690	2208	1051	2501	1914
3837	2951	380	2482	858	1145	524
3049	1325	876	1032	764	7023	2647
501	118	65	149	126	5223	653
897	502	298	168	602	13459	3447
342	209	55	107	64	2501	1026
610	584	464	188	608	5028	1644
521	702	99	874	608	572	534
753	768	109	319	314	1352	1203
738	377	94	183	93	3818	1085
702	365	57	215	134	1371	356
320	333	130	75	71	4788	1628
49	23	6	5	35	117	59
825	296	4	107	32	4093	805
729	329	582	316	129	1251	597
425	233	14	98	829	1597	1573
283	357	204	128	186	2136	1799
1492	856	62	345	89	2497	468
588	64	1	1	3	2771	336
90	41	4	26	5	119	63
272	171	55	78	374	1787	972
160	92	6	26	128	3593	697
333	316	56	73	77	1228	427
416	324	226	110	1831	389	166
183	110	40	66	177	1864	741
257	174	16	88	59	1616	701
149	96	10	123	78	105	93
34	12	4	7	17	92	58

146	319	563	95	42	1652	403
192	176	63	79	186	639	285
88	33	4	39	59	413	787
91	48	19	86	116	270	106
114	113	11	44	115	928	884
262	1494	734	626	34	375	377
126	92	37	26	125	2651	534
123	232	151	50	86	1226	451
298	161	16	145	88	106	54
206	314	36	50	11	748	259
269	87	4	19	178	427	162
195	83	62	75	39	1006	272
261	182	11	9	1	493	138
191	208	288	149	418	104	84
63	58	19	13	94	1336	321
78	40	21	11	141	567	423
103	78	26	33	137	401	188
79	31	6	3	5	1161	369
192	228	231	83	117	593	318
124	33	4	21	8	213	258
183	116	21	129	41	336	134
27	21	4	5	25	818	532
99	130	95	29	48	983	385
92	29	5	23	67	135	41
228	94	16	35	92	802	140
100	23	19	41	175	231	36
58	116	14	77	9	186	87
1	3	2	0	4	21	21
78	21	1	8	2	799	96
28	24	2	9	24	100	52
27	16	2	8	14	121	65
89	20	1	12	66	261	94
81	46	24	19	23	513	187
55	64	31	22	15	514	204
172	104	18	46	13	164	88
22	15	3	7	37	65	29
41	60	11	19	39	688	362
50	19	4	18	23	117	47
83	18	4	5	24	145	67
134	84	20	73	73	90	49
95	77	10	39	43	147	175
47	44	7	37	14	125	62
48	27	8	17	63	684	406
113	50	18	29	81	184	146
37	19	4	10	40	170	87
22	21	3	12	29	84	52
97	45	4	22	19	153	105
49	33	15	10	8	323	93
36	35	5	4	20	704	615
40	53	19	40	47	89	46
31	8	1	3	12	62	17
37	16	11	9	18	206	70
32	20	10	22	4	141	58
63	13	3	17	58	125	41
63	13	3	17	58	125	41
29	28	5	9	90	500	306
15	10	1	3	6	329	188
36	32	6	8	3	345	124
56	27	4	10	2	144	20

68	53	4	26	7	127	46
5	1	0	0	0	24	7
32	8	6	5	15	165	56
29	19	3	6	6	34	15
16	15	3	4	1	124	60
40	46	33	31	28	173	74
18	6	1	2	22	449	106
19	20	13	1	0	21	17
47	10	1	8	20	155	65
44	21	2	14	14	60	19
15	9	7	5	36	189	67
45	7	0	2	3	42	18
15	17	3	7	6	161	95
6	6	1	3	2	199	23
10	12	3	4	2	74	23
15	5	1	4	9	63	14
9	4	0	3	13	202	50
34	13	2	2	1	179	34
6	11	8	1	16	105	61
5	3	1	0	6	201	21
48	18	3	4	32	145	28
1	1	0	0	5	21	15
4	7	10	5	4	94	25
23	10	4	2	19	363	59
30	13	2	3	6	57	15
23	20	13	4	28	117	35
12	15	6	3	14	153	72
35	8	1	3	3	56	36
9	12	4	5	25	55	19
22	3	0	5	0	54	7
18	13	2	1	3	295	45
0	0	0	0	0	68	24
6	35	46	4	14	108	106
20	31	55	23	9	25	14
5	7	10	2	23	191	103
11	3	1	1	2	101	43
5	7	1	1	3	100	21
6	1	1	2	3	6	2
7	6	0	0	9	153	81
7	7	8	4	17	119	42
21	8	1	5	7	57	16
20	8	0	5	11	43	13
9	8	2	4	5	110	33
11	16	2	7	14	70	34
9	5	3	2	9	44	221
10	2	0	1	5	18	8
9	13	9	2	5	68	46
3	1	0	1	14	103	30
13	8	1	4	1	60	14
22	21	4	13	2	36	24
17	7	1	2	12	64	28
42	6	0	2	3	41	7
6	7	3	0	10	131	41
15	9	19	0	7	66	32
17	9	0	3	5	16	7
3	16	1	4	14	69	45
6	0	1	2	8	104	36
6	3	5	0	1	89	49
9	7	1	4	11	11	1

20	9	14	4	0	20	25
16	6	1	9	15	31	12
12	20	22	1	3	34	13
5	3	2	2	6	80	19
9	7	1	4	11	11	1
11	14	12	10	4	47	11
3	1	3	1	6	44	27
13	3	1	2	3	52	5
13	0	0	0	1	1	0
8	5	0	10	10	9	28
6	7	5	1	11	45	30
23	7	14	6	7	80	11
14	21	11	4	1	49	17
17	1	4	3	6	14	6
30	1	0	0	0	27	3
3	9	10	2	4	42	15
4	6	3	1	0	60	56
5	8	1	5	3	25	15
12	4	0	2	1	46	2
34	28	0	10	7	32	12
9	4	0	1	8	41	18
7	10	3	4	0	61	38
5	7	2	5	7	21	26
7	1	2	0	1	40	3
3	5	4	1	4	51	12
8	2	0	2	4	97	15
4	5	3	0	5	24	11
0	2	1	1	5	66	29
13	7	1	3	6	65	16
15	13	0	3	8	72	17
24	8	1	8	3	5	1
2	2	1	0	10	56	44
2	0	0	0	2	38	9
10	1	0	2	2	36	8
12	6	0	1	2	50	13
2	1	0	0	0	51	5
0	2	0	0	0	1	0
5	6	1	1	9	31	8
18	2	1	1	12	2	2
4	5	0	0	2	51	24
7	4	0	6	2	38	18
2	4	2	1	6	34	25
18	3	0	1	8	26	2
3	0	0	0	1	24	7
3	0	0	0	1	15	8
0	1	0	0	1	44	8
4	1	6	0	4	34	19
7	7	1	2	5	23	20
6	2	1	6	7	5	10
7	8	5	8	1	19	5
1	0	0	0	3	3	0
2	4	1	0	0	39	14
2	0	0	0	0	12	2
0	9	1	3	1	12	10
4	2	0	0	0	9	5
8	1	0	1	9	31	14
14	2	0	2	1	28	7
2	3	0	0	3	27	9
5	3	1	1	2	22	7

2	1	0	0	0	32	8
14	3	0	2	7	8	0
11	9	0	2	1	19	12
11	9	0	2	1	19	12
1	0	4	2	1	12	5
3	0	1	1	4	47	19
0	1	2	0	3	8	9
2	0	1	0	1	12	6
5	3	0	2	4	25	3
3	0	0	2	3	65	5
5	6	3	6	3	4	7
1	2	0	1	3	16	9
11	2	0	0	1	3	5
12	1	0	2	11	36	18
11	2	0	0	4	19	8
0	0	0	0	1	34	7
3	5	1	1	5	12	14
0	0	0	0	1	0	1
3	3	1	0	3	25	9
16	3	0	1	2	4	2
1	1	0	0	2	10	10
1	2	4	0	3	16	17
15	3	0	1	1	1	2
2	1	1	1	6	6	2
3	0	0	0	5	9	4
0	4	1	1	1	7	2
2	0	1	0	3	21	4
0	1	0	0	0	3	4
3	0	0	0	0	7	1
1	1	0	0	2	25	11
2	0	1	1	1	28	6
0	0	0	0	1	27	3
4	2	0	0	3	7	5
0	0	0	0	0	0	1
5	4	1	2	4	11	20
2	2	0	2	1	13	6
4	72	0	0	0	7	9
2	0	1	1	0	15	6
9	1	3	2	0	5	5
4	4	4	2	6	21	7
3	0	3	0	3	24	11
2	1	1	0	2	16	7
3	3	1	2	0	7	1
5	0	0	2	1	22	9
4	2	1	2	1	19	5
4	3	0	0	2	20	1
2	1	0	2	1	13	3
2	0	0	0	3	18	8
0	2	0	1	1	0	0
4	0	0	0	2	3	6
4	5	0	3	1	2	0
0	1	0	1	0	19	14
2	2	2	1	0	12	11
0	0	0	0	0	17	6
2	1	0	0	1	7	3
7	0	0	2	1	6	3
2	0	6	3	3	9	4
2	1	0	1	0	8	3
2	0	1	1	1	16	4

2	0	0	0	0	7	3
0	3	0	0	0	11	1
3	0	1	1	3	12	2
1	0	0	0	0	32	7
1	0	5	0	0	10	7
5	3	0	0	0	8	1
0	0	0	0	1	14	5
2	0	0	0	0	9	3
1	1	0	0	0	10	4
4	2	0	0	0	36	12
2	0	0	0	0	10	0
0	1	2	0	0	12	4
4	3	0	3	1	3	0
2	2	0	0	1	1	1
2	0	0	0	0	13	2
3	2	3	1	3	4	7
0	2	4	0	1	18	15
2	1	1	0	0	13	7
2	1	0	1	0	14	10
2	0	0	1	1	4	0
1	1	0	0	1	17	3
0	1	0	1	2	2	1
0	2	1	0	1	15	2
0	0	0	0	0	9	0
1	3	1	0	1	14	5
3	2	0	4	0	5	0
0	0	0	0	4	0	2
1	0	2	0	0	19	2
3	0	0	0	1	2	2
0	0	2	0	0	13	4
1	0	0	0	2	16	4
3	0	0	0	0	10	2
0	0	0	1	0	1	0
2	2	1	0	1	24	5
6	2	0	1	4	1	1
0	1	0	0	2	10	2
0	1	0	0	1	3	4
3	0	2	0	0	30	2
1	0	0	0	0	0	0
3	4	0	0	1	3	5
2	6	1	1	0	12	2
0	0	0	0	0	14	5
1	0	0	0	0	2	3
1	2	0	0	1	18	10
3	2	2	1	0	12	2
1	1	0	0	0	1	1
0	0	0	0	1	5	3
2	0	1	4	0	2	0
2	0	0	0	1	4	2
3	1	0	0	0	7	6
2	0	0	0	1	19	6
1	0	0	0	0	3	2
3	0	1	2	21	15	4
0	0	0	2	0	1	1
0	1	1	0	2	5	1
2	0	0	0	0	6	5
1	1	0	0	0	2	2
1	1	0	1	0	11	2
1	0	0	1	0	3	5

2	0	1	0	2	6	4
1	2	0	0	0	9	1
4	2	0	0	0	14	3
1	0	0	0	0	0	0
2	2	0	1	0	6	4
0	1	3	1	1	3	5
2	0	1	1	0	0	0
2	0	0	0	0	1	1
1	0	0	0	2	2	0
1	0	0	0	0	9	4
0	0	0	0	0	19	3
8	1	0	1	0	1	0
1	0	0	0	2	6	1
0	1	1	0	0	3	0
3	1	0	0	0	0	0
0	0	0	0	0	2	1
0	0	1	1	0	4	2
4	0	0	1	0	5	5
0	0	0	0	0	5	7
1	0	0	0	1	11	2
4	0	0	0	0	10	2
5	0	0	2	0	8	1
1	0	0	0	0	0	0
4	2	0	0	0	2	2
1	0	0	0	0	7	0
0	1	0	0	0	1	1
0	0	0	0	1	13	8
0	0	0	0	0	1	0
2	2	0	0	0	5	3
0	0	0	0	0	3	2
0	1	0	0	0	2	0
1	0	0	0	0	11	0
4	0	0	0	0	3	0
0	0	0	0	0	0	3
1	2	0	3	0	14	6
0	1	0	0	0	3	0
1	5	1	9	0	1	2
1	0	0	0	0	4	0
0	0	0	0	0	2	5
0	0	0	0	0	22	1
0	0	0	0	1	0	1
0	0	0	0	0	8	2
1	2	0	0	0	0	0
0	0	0	0	0	0	0
0	1	0	0	0	1	2
0	0	1	1	0	3	2
0	0	0	0	0	4	6
2	0	0	0	0	1	0
0	3	1	0	0	3	0
0	0	0	0	0	2	0
1	0	0	1	0	0	2
0	0	0	0	0	0	0
0	0	0	0	0	0	0
1	0	0	0	0	6	0
1	2	0	0	0	0	1
1	0	0	0	0	2	2
1	0	0	0	1	2	2
0	0	0	0	0	2	1
0	0	0	0	0	0	0

0	0	0	1	1	1	0
1	0	0	1	0	2	2
1	0	0	0	0	1	6
0	0	0	0	1	2	1
0	0	0	0	0	9	2
0	0	0	0	0	2	0
0	0	0	0	0	0	0
0	0	0	0	0	2	0
0	0	0	0	1	4	0
0	0	0	0	0	1	0
1	1	0	0	0	5	2
0	0	0	0	0	0	1
0	1	0	0	0	4	0
0	1	0	0	0	1	2
0	0	0	0	0	2	0
2	0	0	1	1	6	3
1	1	0	1	0	1	1
0	1	0	0	0	1	0
1	0	0	0	0	5	1
0	0	0	0	0	5	2
1	1	0	0	1	3	0
0	0	0	0	0	0	0
0	0	0	0	1	4	4
0	1	0	0	1	0	1
0	0	0	0	0	2	0
0	0	0	0	1	7	3
0	0	0	0	1	0	0
0	0	0	0	1	1	0
0	2	0	0	0	3	2
0	0	0	0	0	2	1
0	1	0	0	0	1	0
1	2	0	0	1	1	1
0	0	0	1	0	1	0
0	0	0	0	2	2	0
2	0	0	0	0	2	1
0	0	0	0	1	0	1
3	0	0	0	0	1	1
0	0	0	0	0	4	1
0	0	0	0	0	2	0
0	0	0	0	0	3	2
0	0	0	0	0	12	0
0	0	0	0	0	6	1
3	1	0	0	0	3	1
0	1	0	0	0	3	1
0	0	0	0	0	2	0
0	0	0	0	0	1	0
0	0	0	0	0	2	1
1	0	0	0	0	1	1
0	0	0	1	0	2	1
0	0	0	1	0	2	1
1	0	0	0	0	2	4
0	0	0	0	0	0	1
0	0	0	0	0	0	0
1	0	0	0	0	3	0
0	0	0	0	0	0	3
0	0	1	0	2	1	0
2	0	0	0	1	2	1
0	1	0	0	0	0	0
1	1	0	0	0	0	0

0	1	0	0	0	1	2
0	0	0	0	0	3	1
1	0	0	0	0	0	0
0	0	0	1	0	0	0
0	0	0	0	1	6	2
1	0	0	0	0	3	2
0	0	0	0	0	0	0
0	1	0	0	0	1	0
0	0	0	0	0	0	0
0	0	0	0	0	2	0
0	1	0	0	1	2	2
0	0	0	0	1	0	0
0	0	0	0	1	7	2
1	0	0	0	1	2	0
1	0	0	0	0	11	4
0	0	0	0	0	2	2
1	0	0	0	0	2	1
0	0	0	0	0	1	0
0	0	0	1	0	5	0
1	0	0	0	0	0	2
0	0	0	1	0	0	0
0	0	1	0	0	2	0
0	0	0	0	1	1	2
1	0	0	0	0	1	0
2	0	0	0	0	4	0
0	0	0	1	0	1	1
0	1	0	0	0	0	0
0	0	0	2	0	0	0
0	1	0	0	0	1	0
0	0	1	0	0	1	1
0	0	0	0	0	2	0
0	1	0	0	0	2	1
0	0	0	0	0	1	0
0	0	0	0	0	0	0
1	0	0	0	0	0	0
1	0	0	0	0	3	0
1	0	0	0	0	0	0
1	0	0	0	0	2	1
0	0	0	0	0	1	1
0	0	0	0	0	3	1
0	0	0	0	0	1	0
0	0	2	0	0	2	2
0	1	0	0	0	1	1
0	0	0	0	0	2	1
0	0	0	0	0	0	0
0	0	0	0	0	2	0
0	0	0	0	0	2	1
0	0	1	0	0	6	2
0	0	0	0	0	3	1
0	1	0	0	0	2	1
0	0	0	0	0	1	0
1	0	0	0	1	1	2
11230	11159	3424	3420	4250	59396	20896
26839	32796	10115	6376	6972	183174	59096
448	457	482	473	468	387	432
CHR_NP	CHR_NP	CHR_NP	CHR_NP	CHR_NP	CHR_NP	CHR_NP
CHR_NP	CHR_NP	CHR_NP	CHR_NP	CHR_NP	CHR_NP	CHR_NP

29	30	31	32	33	34	35
2	3	4	5	6	7	8
CHR_NP	CHR_NP	CHR_NP	CHR_NP	CHR_NP	CHR_NP	CHR_NP
A_13	A_14	A_15	A_17	B_30	B_33	C_2
371228	359180	111957	117100	148505	1922117	685397
CHR_NP	CHR_NP	CHR_NP	CHR_NP	CHR_NP	CHR_NP	CHR_NP
13.17	15.91	15.47	6.54	1.16	16.96	15.55
3.20	4.64	5.99	8.55	6.37	1.39	2.58
1.08	0.59	0.38	0.54	1.55	0.90	1.29
1.42	1.11	2.15	1.73	4.58	1.77	1.48
0.83	0.62	0.57	0.57	1.02	3.13	2.53
0.96	0.55	0.20	1.06	5.05	0.37	0.72
1.76	0.62	0.28	1.04	3.61	0.72	0.53
0.14	0.26	0.05	0.63	0.51	0.08	0.42
0.55	0.38	0.12	0.30	0.17	0.25	0.23
1.18	1.43	1.16	2.07	0.82	0.70	1.13
0.50	0.50	0.67	0.73	0.24	0.32	0.39
0.28	0.22	0.37	0.26	0.41	0.45	0.44
0.38	0.16	0.13	0.31	0.57	0.16	0.13
0.17	0.24	0.66	0.26	0.46	0.49	0.67
0.44	0.20	0.06	0.52	0.57	0.06	0.07
0.11	0.04	0.03	0.02	0.03	0.56	0.15
0.27	0.11	0.03	0.28	0.70	0.08	0.05
0.20	0.23	0.21	0.34	0.33	0.14	0.24
0.33	0.17	0.07	0.33	0.22	0.11	0.14
0.87	0.55	0.32	1.01	0.10	0.25	0.17
0.12	0.13	0.10	0.14	0.15	0.17	0.17
0.12	0.17	0.27	0.15	0.08	0.16	0.20
0.50	0.19	0.06	0.33	0.05	0.14	0.08
0.21	0.10	0.02	0.14	0.58	0.17	0.17
0.61	0.32	0.03	1.21	0.15	0.02	0.02
0.18	0.09	0.04	0.04	0.02	0.15	0.10
0.05	0.05	0.17	0.04	0.09	0.12	0.12
0.10	0.25	0.16	0.54	0.05	0.05	0.12
0.12	0.09	0.10	0.17	0.33	0.03	0.04
0.15	0.09	0.14	0.14	0.03	0.09	0.06
0.10	0.05	0.02	0.11	0.17	0.10	0.14
0.04	0.02	0.05	0.05	0.16	0.10	0.08
0.09	0.03	0.01	0.06	0.08	0.04	0.04
0.11	0.02	0.01	0.09	0.06	0.05	0.08
0.08	0.06	0.06	0.12	0.30	0.03	0.04
0.05	0.09	0.23	0.10	0.93	0.16	0.41
0.24	0.25	0.20	0.65	0.25	0.04	0.09
0.34	0.26	0.11	0.73	0.20	0.02	0.03
0.27	0.12	0.26	0.30	0.18	0.12	0.13
0.04	0.01	0.02	0.04	0.03	0.09	0.03
0.08	0.04	0.09	0.05	0.14	0.23	0.16
0.03	0.02	0.02	0.03	0.02	0.04	0.05
0.05	0.05	0.14	0.05	0.14	0.08	0.08
0.05	0.06	0.03	0.26	0.14	0.01	0.03
0.07	0.07	0.03	0.09	0.07	0.02	0.06
0.07	0.03	0.03	0.05	0.02	0.06	0.05
0.06	0.03	0.02	0.06	0.03	0.02	0.02
0.03	0.03	0.04	0.02	0.02	0.08	0.08
0.00	0.00	0.00	0.00	0.01	0.00	0.00
0.07	0.03	0.00	0.03	0.01	0.07	0.04
0.06	0.03	0.17	0.09	0.03	0.02	0.03
0.04	0.02	0.00	0.03	0.20	0.03	0.08

0.03	0.03	0.06	0.04	0.04	0.04	0.09
0.13	0.08	0.02	0.10	0.02	0.04	0.02
0.05	0.01	0.00	0.00	0.00	0.05	0.02
0.01	0.00	0.00	0.01	0.00	0.00	0.00
0.02	0.02	0.02	0.02	0.09	0.03	0.05
0.01	0.01	0.00	0.01	0.03	0.06	0.03
0.03	0.03	0.02	0.02	0.02	0.02	0.02
0.04	0.03	0.07	0.03	0.43	0.01	0.01
0.02	0.01	0.01	0.02	0.04	0.03	0.04
0.02	0.02	0.00	0.03	0.01	0.03	0.03
0.01	0.01	0.00	0.04	0.02	0.00	0.00
0.00	0.00	0.00	0.00	0.00	0.00	0.00
0.01	0.03	0.16	0.03	0.01	0.03	0.02
0.02	0.02	0.02	0.02	0.04	0.01	0.01
0.01	0.00	0.00	0.01	0.01	0.01	0.04
0.01	0.00	0.01	0.03	0.03	0.00	0.01
0.01	0.01	0.00	0.01	0.03	0.02	0.04
0.02	0.13	0.21	0.18	0.01	0.01	0.02
0.01	0.01	0.01	0.01	0.03	0.04	0.03
0.01	0.02	0.04	0.01	0.02	0.02	0.02
0.03	0.01	0.00	0.04	0.02	0.00	0.00
0.02	0.03	0.01	0.01	0.00	0.01	0.01
0.02	0.01	0.00	0.01	0.04	0.01	0.01
0.02	0.01	0.02	0.02	0.01	0.02	0.01
0.02	0.02	0.00	0.00	0.00	0.01	0.01
0.02	0.02	0.08	0.04	0.10	0.00	0.00
0.01	0.01	0.01	0.00	0.02	0.02	0.02
0.01	0.00	0.01	0.00	0.03	0.01	0.02
0.01	0.01	0.01	0.01	0.03	0.01	0.01
0.01	0.00	0.00	0.00	0.00	0.02	0.02
0.02	0.02	0.07	0.02	0.03	0.01	0.02
0.01	0.00	0.00	0.01	0.00	0.00	0.01
0.02	0.01	0.01	0.04	0.01	0.01	0.01
0.00	0.00	0.00	0.00	0.01	0.01	0.03
0.01	0.01	0.03	0.01	0.01	0.02	0.02
0.01	0.00	0.00	0.01	0.02	0.00	0.00
0.02	0.01	0.00	0.01	0.02	0.01	0.01
0.01	0.00	0.01	0.01	0.04	0.00	0.00
0.01	0.01	0.00	0.02	0.00	0.00	0.00
0.00	0.00	0.00	0.00	0.00	0.00	0.00
0.01	0.00	0.00	0.00	0.00	0.01	0.00
0.00	0.00	0.00	0.00	0.01	0.00	0.00
0.00	0.00	0.00	0.00	0.00	0.00	0.00
0.01	0.00	0.00	0.00	0.02	0.00	0.00
0.01	0.00	0.01	0.01	0.01	0.01	0.01
0.00	0.01	0.01	0.01	0.00	0.01	0.01
0.02	0.01	0.01	0.01	0.00	0.00	0.00
0.00	0.00	0.00	0.00	0.01	0.00	0.00
0.00	0.01	0.00	0.01	0.01	0.01	0.02
0.00	0.00	0.00	0.01	0.01	0.00	0.00
0.01	0.00	0.00	0.00	0.01	0.00	0.00
0.01	0.01	0.01	0.02	0.02	0.00	0.00
0.01	0.01	0.00	0.01	0.01	0.00	0.01
0.00	0.00	0.00	0.01	0.00	0.00	0.00
0.00	0.00	0.00	0.00	0.01	0.01	0.02
0.01	0.00	0.01	0.01	0.02	0.00	0.01
0.00	0.00	0.00	0.00	0.01	0.00	0.00
0.00	0.00	0.00	0.00	0.01	0.00	0.00
0.01	0.00	0.00	0.01	0.00	0.00	0.01

0.00	0.00	0.00	0.00	0.00	0.01	0.00
0.00	0.00	0.00	0.00	0.00	0.01	0.03
0.00	0.00	0.01	0.01	0.01	0.00	0.00
0.00	0.00	0.00	0.00	0.00	0.00	0.00
0.00	0.00	0.00	0.00	0.00	0.00	0.00
0.00	0.00	0.00	0.01	0.00	0.00	0.00
0.01	0.00	0.00	0.00	0.01	0.00	0.00
0.01	0.00	0.00	0.00	0.01	0.00	0.00
0.00	0.00	0.00	0.00	0.02	0.01	0.01
0.00	0.00	0.00	0.00	0.00	0.01	0.01
0.00	0.00	0.00	0.00	0.00	0.01	0.01
0.00	0.00	0.00	0.00	0.00	0.00	0.00
0.01	0.00	0.00	0.01	0.00	0.00	0.00
0.00	0.00	0.00	0.00	0.00	0.00	0.00
0.00	0.00	0.00	0.00	0.00	0.00	0.00
0.00	0.00	0.00	0.00	0.00	0.00	0.00
0.00	0.00	0.00	0.00	0.00	0.00	0.00
0.00	0.00	0.01	0.01	0.01	0.00	0.00
0.00	0.00	0.00	0.00	0.01	0.01	0.01
0.00	0.00	0.00	0.00	0.00	0.00	0.00
0.00	0.00	0.00	0.00	0.00	0.00	0.00
0.00	0.00	0.00	0.00	0.00	0.00	0.00
0.00	0.00	0.00	0.00	0.01	0.00	0.00
0.00	0.00	0.00	0.00	0.00	0.00	0.00
0.00	0.00	0.00	0.00	0.00	0.00	0.00
0.00	0.00	0.00	0.00	0.00	0.00	0.00
0.00	0.00	0.00	0.00	0.00	0.00	0.00
0.00	0.00	0.00	0.00	0.00	0.00	0.00
0.00	0.00	0.00	0.00	0.00	0.00	0.00
0.00	0.00	0.00	0.00	0.00	0.00	0.00
0.00	0.00	0.00	0.00	0.00	0.00	0.00
0.00	0.00	0.00	0.00	0.00	0.00	0.00
0.00	0.00	0.00	0.00	0.01	0.00	0.00
0.00	0.00	0.00	0.00	0.00	0.00	0.00
0.00	0.00	0.00	0.00	0.00	0.00	0.00
0.00	0.00	0.00	0.00	0.00	0.01	0.00
0.00	0.00	0.00	0.00	0.00	0.00	0.00
0.00	0.00	0.00	0.00	0.01	0.00	0.00
0.00	0.00	0.00	0.00	0.00	0.00	0.00
0.00	0.00	0.00	0.00	0.00	0.00	0.00
0.00	0.00	0.00	0.00	0.01	0.00	0.00
0.00	0.00	0.00	0.00	0.00	0.00	0.00
0.00	0.00	0.00	0.00	0.00	0.00	0.00
0.00	0.00	0.00	0.00	0.00	0.00	0.00
0.00	0.00	0.01	0.00	0.00	0.00	0.01
0.00	0.00	0.02	0.01	0.00	0.00	0.00
0.00	0.00	0.00	0.00	0.01	0.00	0.00
0.00	0.00	0.00	0.00	0.00	0.00	0.00
0.00	0.00	0.00	0.00	0.00	0.00	0.00
0.00	0.00	0.00	0.00	0.00	0.00	0.00
0.00	0.00	0.00	0.00	0.00	0.00	0.00
0.00	0.00	0.00	0.00	0.00	0.00	0.00
0.00	0.00	0.00	0.00	0.00	0.00	0.00
0.00	0.00	0.00	0.00	0.00	0.00	0.00
0.00	0.00	0.00	0.00	0.00	0.00	0.00
0.00	0.00	0.00	0.00	0.00	0.00	0.00
0.00	0.00	0.00	0.00	0.00	0.00	0.01
0.00	0.00	0.00	0.00	0.00	0.00	0.00
0.00	0.00	0.00	0.00	0.00	0.00	0.00

0.00	0.00	0.00	0.00	0.00	0.00	0.00
0.00	0.00	0.00	0.00	0.00	0.00	0.00
0.00	0.00	0.00	0.00	0.00	0.00	0.00
0.00	0.00	0.00	0.00	0.00	0.00	0.00
0.00	0.00	0.00	0.00	0.00	0.00	0.00
0.00	0.00	0.00	0.00	0.00	0.00	0.00
0.00	0.00	0.01	0.00	0.00	0.00	0.00
0.00	0.00	0.00	0.00	0.00	0.00	0.00
0.00	0.00	0.00	0.00	0.00	0.00	0.00
0.00	0.00	0.00	0.00	0.00	0.00	0.00
0.00	0.00	0.00	0.00	0.00	0.00	0.00
0.00	0.00	0.00	0.00	0.00	0.00	0.00
0.00	0.00	0.00	0.00	0.00	0.00	0.00
0.00	0.00	0.00	0.00	0.00	0.00	0.00
0.00	0.00	0.01	0.00	0.00	0.00	0.00
0.00	0.00	0.00	0.00	0.00	0.00	0.00
0.00	0.00	0.00	0.00	0.00	0.00	0.00
0.00	0.00	0.00	0.00	0.00	0.00	0.00
0.00	0.00	0.00	0.00	0.00	0.00	0.00
0.00	0.00	0.00	0.00	0.00	0.00	0.00
0.00	0.00	0.00	0.00	0.00	0.00	0.00
0.00	0.00	0.00	0.00	0.00	0.00	0.00
0.00	0.00	0.00	0.00	0.00	0.00	0.00
0.00	0.00	0.00	0.00	0.00	0.00	0.00
0.00	0.00	0.00	0.00	0.00	0.00	0.00
0.00	0.00	0.00	0.00	0.00	0.00	0.00
0.00	0.00	0.00	0.00	0.00	0.00	0.00
0.00	0.00	0.00	0.00	0.00	0.00	0.00
0.00	0.00	0.00	0.00	0.00	0.00	0.00
0.00	0.00	0.00	0.00	0.00	0.00	0.00
0.00	0.00	0.00	0.00	0.00	0.00	0.00
0.00	0.00	0.00	0.00	0.00	0.00	0.00
0.00	0.00	0.00	0.00	0.00	0.00	0.00
0.00	0.00	0.00	0.00	0.00	0.00	0.00
0.00	0.00	0.00	0.00	0.00	0.00	0.00
0.00	0.00	0.00	0.00	0.00	0.00	0.00
0.00	0.00	0.00	0.00	0.00	0.00	0.00
0.00	0.00	0.00	0.00	0.00	0.00	0.00
0.00	0.00	0.00	0.00	0.00	0.00	0.00
0.00	0.00	0.00	0.00	0.00	0.00	0.00
0.00	0.00	0.00	0.00	0.00	0.00	0.00
0.00	0.00	0.00	0.00	0.00	0.00	0.00
0.00	0.00	0.00	0.00	0.00	0.00	0.00
0.00	0.00	0.00	0.00	0.00	0.00	0.00
0.00	0.00	0.00	0.00	0.00	0.00	0.00
0.00	0.00	0.00	0.00	0.00	0.00	0.00
0.00	0.00	0.00	0.00	0.00	0.00	0.00
0.00	0.00	0.00	0.00	0.00	0.00	0.00
0.00	0.00	0.00	0.00	0.00	0.00	0.00
0.00	0.00	0.00	0.00	0.00	0.00	0.00
0.00	0.00	0.00	0.00	0.00	0.00	0.00
0.00	0.00	0.00	0.00	0.00	0.00	0.00
0.00	0.00	0.00	0.00	0.00	0.00	0.00
0.00	0.00	0.00	0.00	0.00	0.00	0.00
0.00	0.00	0.00	0.00	0.00	0.00	0.00
0.00	0.00	0.00	0.00	0.00	0.00	0.00
0.00	0.00	0.00	0.00	0.00	0.00	0.00
0.00	0.00	0.00	0.00	0.00	0.00	0.00
0.00	0.00	0.00	0.00	0.00	0.00	0.00

0.00	0.00	0.00	0.00	0.00	0.00	0.00
0.00	0.00	0.00	0.00	0.00	0.00	0.00
0.00	0.00	0.00	0.00	0.00	0.00	0.00
0.00	0.00	0.00	0.00	0.00	0.00	0.00
0.00	0.00	0.00	0.00	0.00	0.00	0.00
0.00	0.00	0.00	0.00	0.00	0.00	0.00
0.00	0.00	0.00	0.00	0.00	0.00	0.00
0.00	0.00	0.00	0.00	0.00	0.00	0.00
0.00	0.00	0.00	0.00	0.00	0.00	0.00
0.00	0.00	0.00	0.00	0.00	0.00	0.00
0.00	0.00	0.00	0.00	0.00	0.00	0.00
0.00	0.00	0.00	0.00	0.00	0.00	0.00
0.00	0.00	0.00	0.00	0.00	0.00	0.00
0.00	0.00	0.00	0.00	0.00	0.00	0.00
0.00	0.00	0.00	0.00	0.00	0.00	0.00
0.00	0.00	0.00	0.00	0.00	0.00	0.00
0.00	0.00	0.00	0.00	0.00	0.00	0.00
0.00	0.00	0.00	0.00	0.00	0.00	0.00
0.00	0.00	0.00	0.00	0.00	0.00	0.00
0.00	0.00	0.00	0.00	0.00	0.00	0.00
0.00	0.00	0.00	0.00	0.00	0.00	0.00
0.00	0.00	0.00	0.00	0.00	0.00	0.00
0.00	0.00	0.00	0.00	0.00	0.00	0.00
0.00	0.00	0.00	0.00	0.00	0.00	0.00
0.00	0.00	0.00	0.00	0.00	0.00	0.00
0.00	0.00	0.00	0.00	0.00	0.00	0.00
0.00	0.00	0.00	0.00	0.00	0.00	0.00
0.00	0.00	0.00	0.00	0.00	0.00	0.00
0.00	0.00	0.00	0.00	0.00	0.00	0.00
0.00	0.00	0.00	0.00	0.00	0.00	0.00

29	30	31	32	33	34	35
2<br>CHR_NP<br>A_13<br>371228	3<br>CHR_NP<br>A_14<br>359180	4<br>CHR_NP<br>A_15<br>111957	5<br>CHR_NP<br>A_17<br>117100	6<br>CHR_NP<br>B_30<br>148505	7<br>CHR_NP<br>B_33<br>1922117	8<br>CHR_NP<br>C_2<br>685397
A_13	A_14	A_15	A_17	B_30	B_33	C_2
0.35	0.96	0.86	-1.14	-2.35	1.20	0.88
-0.02	0.40	0.80	1.55	0.91	-0.55	-0.20
-0.78	-1.14	-1.30	-1.18	-0.42	-0.91	-0.62
-1.01	-1.33	-0.29	-0.71	2.11	-0.67	-0.96
-1.47	-1.71	-1.76	-1.76	-1.26	1.07	0.40
-0.21	-0.58	-0.90	-0.12	3.49	-0.74	-0.43
0.95	-0.88	-1.42	-0.20	3.89	-0.71	-1.02
-0.37	-0.14	-0.53	0.53	0.31	-0.47	0.14

-0.08	-0.41	-0.94	-0.57	-0.83	-0.67	-0.71
1.07	1.73	1.03	3.37	0.17	-0.15	0.94
0.10	0.12	0.76	0.97	-0.85	-0.58	-0.29
-1.90	-2.45	-1.11	-2.10	-0.74	-0.37	-0.43
0.14	-1.15	-1.33	-0.25	1.21	-1.15	-1.28
-1.69	-1.30	1.11	-1.17	-0.06	0.14	1.18
1.03	-0.09	-0.74	1.39	1.59	-0.73	-0.67
-0.80	-1.13	-1.15	-1.23	-1.14	1.15	-0.63
0.70	-0.30	-0.80	0.77	3.42	-0.46	-0.65
-0.14	0.20	-0.06	1.21	1.17	-0.75	0.28
1.37	-0.14	-1.00	1.28	0.34	-0.63	-0.36
6.39	3.15	0.86	7.78	-1.27	0.17	-0.58
-0.55	-0.50	-0.84	-0.24	-0.17	0.23	0.20
-0.53	0.28	1.77	0.04	-1.04	0.09	0.76
4.72	0.93	-0.75	2.64	-0.90	0.24	-0.44
0.89	-0.17	-0.97	0.21	4.39	0.45	0.49
2.81	1.13	-0.57	6.40	0.09	-0.67	-0.67
1.20	-0.52	-1.39	-1.40	-1.82	0.62	-0.26
-1.48	-1.36	0.52	-1.57	-0.75	-0.26	-0.32
-0.11	1.28	0.47	4.08	-0.61	-0.58	0.02
0.30	-0.31	-0.17	1.23	4.25	-1.48	-1.31
1.01	-0.19	0.86	0.85	-1.74	-0.20	-0.94
0.33	-0.83	-1.55	0.49	1.79	0.32	1.10
-1.63	-1.96	-1.42	-1.43	0.82	-0.47	-0.80
0.41	-0.49	-0.70	0.03	0.25	-0.33	-0.35
0.21	-0.27	-0.34	0.11	-0.10	-0.10	0.04
-0.27	-0.72	-0.69	0.49	4.56	-1.49	-1.30
-0.57	-0.34	0.53	-0.24	4.82	0.13	1.63
1.54	1.58	1.04	6.29	1.58	-0.84	-0.26
2.56	1.77	0.19	6.51	1.13	-0.75	-0.69
2.06	0.23	1.88	2.43	0.96	0.23	0.33
-0.44	-0.72	-0.65	-0.45	-0.56	-0.09	-0.55
0.08	-0.63	0.23	-0.55	1.34	3.08	1.82
-1.15	-1.50	-1.58	-1.12	-1.61	-0.80	-0.59
-0.40	-0.48	2.88	-0.38	3.19	0.82	0.58
0.22	0.63	-0.21	5.41	2.62	-0.69	-0.30
0.10	0.13	-0.65	0.65	0.24	-0.85	-0.11
1.40	-0.92	-1.38	0.51	-1.78	1.29	0.40
0.88	-0.33	-0.97	0.89	-0.37	-0.71	-0.96
-0.70	-0.66	-0.39	-0.92	-1.09	1.03	0.94
-0.25	-0.33	-0.34	-0.35	-0.11	-0.33	-0.30
0.99	-0.08	-0.66	0.02	-0.52	0.89	0.19
1.59	-0.32	7.26	3.08	-0.27	-0.77	-0.37
0.10	-0.39	-0.88	-0.17	4.70	-0.22	1.20
-0.81	-0.29	1.85	0.14	0.63	0.02	3.90
5.47	2.51	-0.58	3.78	-0.43	0.68	-0.35
1.52	-1.07	-1.37	-1.37	-1.34	1.20	-0.49

-0.05	-0.23	-0.33	-0.07	-0.33	-0.30	-0.26
-0.31	-1.03	-0.97	-0.42	4.83	0.16	1.49
-0.81	-1.31	-1.85	-1.36	0.51	3.03	0.77
-0.07	-0.17	-1.06	-0.69	-0.93	-0.74	-0.75
-0.10	-0.31	0.66	-0.23	10.28	-0.91	-0.87
-0.67	-1.18	-1.04	-0.42	1.37	0.55	0.88
0.07	-0.53	-1.44	0.31	-0.67	0.43	0.95
0.31	-0.05	-0.50	2.09	0.71	-0.59	-0.38
-0.16	-0.25	-0.24	-0.20	-0.11	-0.23	-0.17
-0.95	-0.05	7.78	-0.09	-1.13	-0.09	-0.58
0.06	-0.07	0.18	0.63	2.62	-0.55	-0.28
-0.31	-0.67	-0.80	-0.05	0.13	-0.38	1.87
-0.23	-0.61	-0.48	1.46	1.67	-0.58	-0.53
-0.30	-0.30	-1.09	0.01	1.63	0.33	3.37
-0.13	1.30	2.34	1.93	-0.33	-0.35	-0.20
-0.73	-0.94	-0.76	-0.98	0.55	1.63	0.28
-0.93	0.04	2.34	-0.57	-0.02	0.02	0.12
1.54	0.41	-0.51	3.02	0.99	-0.78	-0.70
0.59	1.88	-0.44	0.10	-1.48	-0.17	-0.19
1.60	-0.40	-1.23	-0.68	3.83	-0.48	-0.41
0.01	-0.81	0.08	0.39	-0.66	-0.02	-0.34
1.06	0.22	-1.37	-1.44	-1.73	-0.75	-0.96
-0.11	-0.07	1.65	0.59	2.03	-0.51	-0.45
-1.10	-1.14	-1.10	-1.26	0.42	0.45	-0.20
-0.73	-1.05	-0.81	-1.08	1.72	-0.49	0.51
-0.36	-0.79	-0.67	-0.27	4.17	-0.84	-0.40
-0.75	-1.36	-1.51	-1.63	-1.59	1.04	0.77
0.05	0.36	4.70	0.71	1.01	-0.61	-0.12
0.00	-0.37	-0.46	-0.23	-0.42	-0.34	0.06
1.41	0.42	-0.30	5.02	0.29	-0.38	-0.26
-1.13	-1.22	-1.35	-1.29	-0.51	0.89	2.96
-0.87	-0.30	2.91	-0.94	-0.38	0.67	1.05
0.34	-0.58	-0.77	0.10	1.59	-0.63	-0.69
3.16	0.02	-0.98	0.49	3.52	1.36	-0.44
-0.37	-1.21	-0.78	0.02	3.63	-0.99	-1.25
-0.71	0.63	-0.99	3.75	-1.49	-1.23	-0.97
-0.30	-0.27	-0.21	-0.32	-0.14	-0.25	-0.13
0.08	-1.27	-1.69	-1.14	-1.64	1.81	-0.54
-0.66	-0.73	-1.05	-0.63	-0.02	-0.82	-0.66
-0.24	-0.43	-0.59	-0.25	-0.07	-0.31	-0.11
0.38	-0.84	-1.13	-0.49	1.90	-0.32	-0.30
-0.16	-0.90	-0.21	-0.56	-0.59	0.18	0.25
-0.77	-0.45	0.81	-0.19	-1.30	0.66	1.08
1.91	0.47	-0.50	1.46	-1.02	-1.10	-0.75
-0.36	-0.50	-0.61	-0.34	1.18	-0.56	-0.49
-0.38	-0.08	-0.46	-0.05	0.58	1.00	2.00
-0.22	-1.33	-1.54	0.11	0.17	-1.22	-1.11
-0.04	-1.27	-1.36	-1.30	-0.41	-1.09	-0.93

0.29	-0.07	-0.21	1.08	0.73	-0.57	-0.50
1.27	0.76	-0.56	2.25	1.82	-0.71	1.25
0.23	0.16	-0.39	2.14	-0.03	-0.37	-0.12
-0.35	-0.90	-0.93	-0.14	2.83	1.84	4.22
0.50	-0.32	-0.20	0.27	1.81	-0.52	0.05
-0.15	-0.65	-0.82	-0.27	1.79	-0.29	0.13
-0.60	-0.63	-1.01	-0.01	1.24	-0.80	-0.40
1.55	-0.07	-1.09	0.77	0.08	-0.59	0.28
-0.17	-0.88	-0.16	-0.90	-1.42	0.37	-0.13
-0.30	-0.32	-0.88	-0.97	0.19	2.55	8.36
0.11	0.59	0.91	3.39	3.13	-0.72	-0.44
-0.45	-1.21	-1.37	-1.15	-0.43	-1.09	-1.18
-0.66	-1.47	-0.70	-0.95	-0.26	-0.59	-0.64
-0.22	-0.89	-0.17	2.08	-1.44	-0.52	-0.26
1.02	-0.86	-0.98	0.75	4.41	-0.46	-0.52
1.02	-0.86	-0.98	0.75	4.41	-0.46	-0.52
-0.49	-0.51	-0.80	-0.48	4.67	1.13	2.86
-0.80	-0.96	-1.17	-0.96	-0.77	0.71	1.95
-0.10	-0.31	-0.99	-0.63	-1.63	1.49	1.57
0.84	-0.09	-0.54	0.10	-0.79	-0.09	-0.62
1.50	0.91	-0.69	2.19	-0.47	-0.25	-0.22
-0.23	-0.36	-0.39	-0.39	-0.39	-0.24	-0.27
-0.43	-1.36	-0.91	-1.03	-0.14	-0.47	-0.51
-0.11	-0.55	-0.96	-0.52	-0.69	-1.11	-1.04
-0.78	-0.82	-1.07	-0.91	-1.40	-0.44	-0.04
0.10	0.29	2.23	2.03	1.16	-0.13	0.09
-0.45	-0.69	-0.75	-0.68	0.36	0.89	0.33
-0.05	-0.01	0.83	-0.63	-0.76	-0.61	-0.42
1.54	-1.02	-1.49	0.10	1.95	0.32	0.71
1.23	-0.20	-1.12	1.36	0.79	-0.82	-0.89
-0.58	-0.79	-0.30	-0.53	2.24	0.15	0.16
1.44	-0.50	-0.86	-0.53	-0.46	-0.46	-0.37
-0.84	-0.68	-1.24	-0.22	-0.78	0.36	1.97

26	27	28	29	30	31	32
28	29	30	31	32	33	34
TACCACAGGGTA(	AAAAGCTGGGTT(	TTTGGCAATGG	TCGAGGAGCTC	TCAGTGCATCAC	TGTAAACATCCT	AGAGGTAGTAGG
miR-140-3p	miR-320a	miR-182-5p	miR-151b	miR-148b-3p	miR-30c-5p	let-7d-5p
605614	549148	501331	500966	497718	492626	463804
1.34	0.58	-0.73	-0.90	0.26	-0.41	-0.85
-1.78	-1.10	2.95	1.14	-1.50	-0.86	-1.63
0.50	-0.21	0.51	1.56	-0.73	-0.23	0.95
1.67	-0.33	-0.18	-0.43	-0.75	-0.87	0.18
-1.76	-0.42	-0.74	3.04	-1.43	0.52	1.13
-0.75	-0.44	-0.97	-0.44	0.67	-1.19	-0.56
-0.36	1.04	-0.81	-0.76	1.48	-1.14	-0.06
-0.61	-0.33	-0.91	-1.54	-0.75	-0.58	-0.05
-0.73	-0.97	0.19	0.09	-0.70	-0.54	-0.90
-0.61	-0.77	1.05	-0.13	-0.75	-0.57	-1.10
-0.21	-1.49	1.63	1.55	0.67	0.31	-0.72
-0.89	0.13	-0.90	-1.02	-1.22	-0.23	0.89
-0.37	-1.08	-0.11	0.20	0.15	2.45	-0.36
-0.34	0.48	-0.82	-1.05	-1.16	-1.11	0.57
0.91	0.42	0.37	-0.16	-0.65	0.13	-0.64
0.36	-0.42	0.30	1.15	0.51	1.73	-0.85
1.35	0.79	-0.76	-0.80	0.80	0.02	0.45
0.53	0.53	-0.15	-0.08	-0.89	-0.16	-0.56
1.12	-1.11	0.57	-0.68	0.21	1.63	-1.27
1.12	-0.49	0.84	0.72	-0.79	2.06	-0.49

-0.27	-0.21	0.18	0.45	1.51	-0.88	-0.99
1.34	0.79	-0.60	-0.60	1.68	0.11	2.08
0.69	1.83	-0.56	-0.19	1.17	-0.20	1.72
0.24	3.16	-0.80	-0.77	0.47	-1.01	0.64
-1.91	-0.15	-0.74	0.16	-0.61	-0.32	1.86
-0.47	0.53	-0.77	-0.33	1.57	0.38	0.79
-0.12	-0.74	1.97	-0.16	0.79	0.94	-0.24
-0.18	-1.36	1.52	1.56	-0.98	-0.66	-0.53
1.20	-1.48	-0.11	0.30	1.01	0.33	-1.63
-0.52	-1.36	1.28	-0.31	-0.19	-0.83	-1.96
-1.39	0.52	0.47	-0.17	0.86	-1.55	-1.42
-1.40	-1.57	4.08	1.23	0.85	0.49	-1.43
-1.82	-0.75	-0.61	4.25	-1.74	1.79	0.82
0.62	-0.26	-0.58	-1.48	-0.20	0.32	-0.47
-0.26	-0.32	0.02	-1.31	-0.94	1.10	-0.80
1.10	-0.49	-0.99	-0.90	0.23	0.41	-0.13
-1.47	0.52	-1.00	-1.70	-1.72	-1.76	-0.21
1.04	-1.15	0.00	-1.12	-0.38	0.87	-1.57
-0.11	-1.26	0.77	-0.54	-0.27	0.83	-1.47
-0.17	-0.78	-0.76	-0.78	-0.91	1.28	-0.21
0.18	3.78	-0.46	-0.89	5.56	-0.94	-1.15
2.26	20.61	-0.36	-0.34	7.72	-0.99	-0.81
0.79	-0.32	-0.84	0.22	1.36	-0.81	-0.61
1.09	-1.18	0.25	-0.49	1.23	1.48	-1.62
-0.03	-0.15	-0.78	-0.93	-0.73	0.76	-1.22
-0.90	4.71	-0.79	-1.27	1.28	-1.56	-1.54
1.33	-1.09	-0.36	1.67	0.91	4.26	-0.61
0.71	0.45	-0.60	0.32	1.35	1.25	0.24
1.23	-0.26	0.17	0.47	-0.69	2.98	-1.08
1.94	-0.22	-0.48	-0.06	-0.52	2.36	-0.67
1.12	-0.62	-0.33	0.43	0.59	2.46	-1.13
1.49	1.17	-0.87	0.84	4.20	-0.72	-0.18
1.04	1.00	-0.40	0.96	3.01	-1.18	0.68
0.93	0.23	-0.70	1.31	1.71	1.54	0.07
0.30	0.22	-0.51	0.00	1.37	-0.52	-0.03
1.34	0.28	-0.95	0.26	1.36	-0.96	-0.02
0.62	0.07	-0.38	0.90	1.59	0.20	-0.72
-0.52	-0.83	1.33	-0.48	-0.83	0.51	-0.21
-0.79	1.87	-0.78	-0.75	0.97	0.33	0.87
-1.37	1.12	-0.33	0.58	0.65	3.47	0.79
-1.43	0.80	-0.08	0.86	-0.59	2.90	0.83
-0.95	-0.97	2.29	0.17	-0.76	0.17	-0.46
-0.48	0.87	-0.40	-0.99	0.30	-0.54	0.84
-0.93	-0.15	-0.24	-0.50	0.48	0.38	1.27
-0.22	-0.31	-0.66	0.45	-0.09	0.06	0.68
-0.29	3.48	2.73	-0.08	-0.83	-0.83	0.49
1.31	-1.09	0.19	-0.38	0.00	-0.50	-1.92
-1.66	-1.07	1.31	0.86	0.07	-1.06	-1.94
-0.87	3.76	0.00	3.26	-0.84	0.01	5.63
0.11	0.22	0.08	1.33	-0.06	2.01	1.24
1.16	-0.33	-0.68	-0.89	0.19	-0.47	-0.40
-0.07	-1.95	1.99	-1.65	-0.63	1.58	-1.70
-0.18	-0.66	0.23	-0.22	-0.63	-0.07	0.08
-0.02	-0.61	0.23	-0.14	-1.33	1.86	0.30
1.25	25.44	-0.94	-1.08	-0.57	-1.86	-0.98
-0.28	0.66	-0.41	-0.13	0.05	0.52	-0.34
0.21	-1.20	0.84	-0.26	-0.28	0.66	-1.37
-1.02	-1.21	1.12	-0.67	-0.17	-0.39	-0.47
2.55	-0.67	-0.22	1.82	1.21	1.93	-0.49

0.40	-1.17	0.04	1.78	0.97	2.51	-0.60
0.29	-0.01	-0.46	0.72	0.69	-0.34	-0.33
0.20	-0.50	-0.46	1.56	1.59	0.93	-0.17
-0.17	0.03	-0.89	-0.20	-0.40	2.45	-0.88
0.83	-0.62	0.61	0.40	-0.94	2.36	-1.09
0.10	-1.11	1.49	1.17	-1.06	-0.15	-0.54
1.08	0.11	-0.92	0.84	-0.22	2.33	-1.18
2.04	-0.40	-0.67	0.44	0.55	1.65	-0.31
1.24	-0.14	-0.21	1.64	0.07	0.61	-0.66
1.60	-1.03	0.40	-0.30	0.38	2.13	-0.97
0.87	-0.38	-0.66	1.07	0.71	1.22	-0.22
-0.74	0.95	3.43	0.30	-0.31	-0.92	1.14
1.02	-0.75	-0.79	-1.13	1.53	-0.90	-1.55
1.14	0.52	-0.86	-0.55	1.63	-0.17	2.16
-0.77	-1.14	0.41	-0.06	2.48	-0.04	-0.42
-0.39	-0.36	0.69	-0.35	3.96	-0.16	-1.26
-1	-1	-1	-1	0	0	-1
0.00	0.00	0.00	0.00	0.00	0.00	0.00
1.00	1.00	1.00	1.00	1.00	1.00	1.00
0.33	0.93	0.23	0.31	0.10	0.82	0.55

26	27	28	29	30	31	32
TACCACAGGGTA(	AAAAGCTGGGTT(	TTTGGCAATGG!	TCGAGGAGCTC/	TCAGTGCATCAC/	TGTAAACATCCT/	AGAGGTAGTAGGT
miR-140-3p	miR-320a	miR-182-5p	miR-151b	miR-148b-3p	miR-30c-5p	let-7d-5p
605614	549148	501331	500966	497718	492626	463804
1.34	0.58	-0.73	-0.90	0.26	-0.41	-0.85
-1.78	-1.10	2.95	1.14	-1.50	-0.86	-1.63
0.50	-0.21	0.51	1.56	-0.73	-0.23	0.95
1.67	-0.33	-0.18	-0.43	-0.75	-0.87	0.18
-1.76	-0.42	-0.74	3.04	-1.43	0.52	1.13
-0.75	-0.44	-0.97	-0.44	0.67	-1.19	-0.56
-0.36	1.04	-0.81	-0.76	1.48	-1.14	-0.06
-0.61	-0.33	-0.91	-1.54	-0.75	-0.58	-0.05
-0.73	-0.97	0.19	0.09	-0.70	-0.54	-0.90
-0.61	-0.77	1.05	-0.13	-0.75	-0.57	-1.10
-0.21	-1.49	1.63	1.55	0.67	0.31	-0.72
-0.89	0.13	-0.90	-1.02	-1.22	-0.23	0.89
-0.37	-1.08	-0.11	0.20	0.15	2.45	-0.36
-0.34	0.48	-0.82	-1.05	-1.16	-1.11	0.57
0.91	0.42	0.37	-0.16	-0.65	0.13	-0.64
0.36	-0.42	0.30	1.15	0.51	1.73	-0.85
1.35	0.79	-0.76	-0.80	0.80	0.02	0.45
0.53	0.53	-0.15	-0.08	-0.89	-0.16	-0.56
1.12	-1.11	0.57	-0.68	0.21	1.63	-1.27
1.12	-0.49	0.84	0.72	-0.79	2.06	-0.49
-0.27	-0.21	0.18	0.45	1.51	-0.88	-0.99
1.34	0.79	-0.60	-0.60	1.68	0.11	2.08
0.69	1.83	-0.56	-0.19	1.17	-0.20	1.72
0.24	3.16	-0.80	-0.77	0.47	-1.01	0.64
-1.91	-0.15	-0.74	0.16	-0.61	-0.32	1.86
-0.47	0.53	-0.77	-0.33	1.57	0.38	0.79
-0.12	-0.74	1.97	-0.16	0.79	0.94	-0.24
-0.18	-1.36	1.52	1.56	-0.98	-0.66	-0.53

1.20	-1.48	-0.11	0.30	1.01	0.33	-1.63
-0.52	-1.36	1.28	-0.31	-0.19	-0.83	-1.96
-1.39	0.52	0.47	-0.17	0.86	-1.55	-1.42
-1.40	-1.57	4.08	1.23	0.85	0.49	-1.43
-1.82	-0.75	-0.61	4.25	-1.74	1.79	0.82
0.62	-0.26	-0.58	-1.48	-0.20	0.32	-0.47
-0.26	-0.32	0.02	-1.31	-0.94	1.10	-0.80
1.10	-0.49	-0.99	-0.90	0.23	0.41	-0.13
-1.47	0.52	-1.00	-1.70	-1.72	-1.76	-0.21
1.04	-1.15	0.00	-1.12	-0.38	0.87	-1.57
-0.11	-1.26	0.77	-0.54	-0.27	0.83	-1.47
-0.17	-0.78	-0.76	-0.78	-0.91	1.28	-0.21
0.18	3.78	-0.46	-0.89	5.56	-0.94	-1.15
2.26	20.61	-0.36	-0.34	7.72	-0.99	-0.81
0.79	-0.32	-0.84	0.22	1.36	-0.81	-0.61
1.09	-1.18	0.25	-0.49	1.23	1.48	-1.62
-0.03	-0.15	-0.78	-0.93	-0.73	0.76	-1.22
-0.90	4.71	-0.79	-1.27	1.28	-1.56	-1.54
1.33	-1.09	-0.36	1.67	0.91	4.26	-0.61
0.71	0.45	-0.60	0.32	1.35	1.25	0.24
1.23	-0.26	0.17	0.47	-0.69	2.98	-1.08
1.94	-0.22	-0.48	-0.06	-0.52	2.36	-0.67
1.12	-0.62	-0.33	0.43	0.59	2.46	-1.13
1.49	1.17	-0.87	0.84	4.20	-0.72	-0.18
1.04	1.00	-0.40	0.96	3.01	-1.18	0.68
0.93	0.23	-0.70	1.31	1.71	1.54	0.07
0.30	0.22	-0.51	0.00	1.37	-0.52	-0.03
1.34	0.28	-0.95	0.26	1.36	-0.96	-0.02
0.62	0.07	-0.38	0.90	1.59	0.20	-0.72
-0.52	-0.83	1.33	-0.48	-0.83	0.51	-0.21
-0.79	1.87	-0.78	-0.75	0.97	0.33	0.87
-1.37	1.12	-0.33	0.58	0.65	3.47	0.79
-1.43	0.80	-0.08	0.86	-0.59	2.90	0.83
-0.95	-0.97	2.29	0.17	-0.76	0.17	-0.46
-0.48	0.87	-0.40	-0.99	0.30	-0.54	0.84
-0.93	-0.15	-0.24	-0.50	0.48	0.38	1.27
-0.22	-0.31	-0.66	0.45	-0.09	0.06	0.68
-0.29	3.48	2.73	-0.08	-0.83	-0.83	0.49
1.31	-1.09	0.19	-0.38	0.00	-0.50	-1.92
-1.66	-1.07	1.31	0.86	0.07	-1.06	-1.94
-0.87	3.76	0.00	3.26	-0.84	0.01	5.63
0.11	0.22	0.08	1.33	-0.06	2.01	1.24
1.16	-0.33	-0.68	-0.89	0.19	-0.47	-0.40
-0.07	-1.95	1.99	-1.65	-0.63	1.58	-1.70
-0.18	-0.66	0.23	-0.22	-0.63	-0.07	0.08
-0.02	-0.61	0.23	-0.14	-1.33	1.86	0.30
1.25	25.44	-0.94	-1.08	-0.57	-1.86	-0.98
-0.28	0.66	-0.41	-0.13	0.05	0.52	-0.34
0.21	-1.20	0.84	-0.26	-0.28	0.66	-1.37
-1.02	-1.21	1.12	-0.67	-0.17	-0.39	-0.47
2.55	-0.67	-0.22	1.82	1.21	1.93	-0.49
0.40	-1.17	0.04	1.78	0.97	2.51	-0.60
0.29	-0.01	-0.46	0.72	0.69	-0.34	-0.33
0.20	-0.50	-0.46	1.56	1.59	0.93	-0.17
-0.17	0.03	-0.89	-0.20	-0.40	2.45	-0.88
0.83	-0.62	0.61	0.40	-0.94	2.36	-1.09
0.10	-1.11	1.49	1.17	-1.06	-0.15	-0.54
1.08	0.11	-0.92	0.84	-0.22	2.33	-1.18
2.04	-0.40	-0.67	0.44	0.55	1.65	-0.31

1.24	-0.14	-0.21	1.64	0.07	0.61	-0.66
1.60	-1.03	0.40	-0.30	0.38	2.13	-0.97
0.87	-0.38	-0.66	1.07	0.71	1.22	-0.22
-0.74	0.95	3.43	0.30	-0.31	-0.92	1.14
1.02	-0.75	-0.79	-1.13	1.53	-0.90	-1.55
1.14	0.52	-0.86	-0.55	1.63	-0.17	2.16
-0.77	-1.14	0.41	-0.06	2.48	-0.04	-0.42
0	0	1	0	4	0	-1

26	27	28	29	30	31	32
12	100	104	75	133	127	97
TGTAAACATCCC(	TAGTGCAATATT(	TACCCTGTAGA'	TAGCACCATCT(	CCCAGTGTTCAG/	CTGCCCTGGCCC(	TCCGGTTCTCAG(
miR-30d-5p	miR-454-3p	miR-10a-5p	miR-29a-3p	miR-199a-5p	miR-874	miR-671-3p
2131733	2.68E+04	25811	67482	11007	12358	28118
1.36	-0.69	-0.49	-0.31	-0.34	0.68	-1.03
-1.18	-0.81	4.54	-0.98	-0.42	-1.39	-0.76
0.67	-0.15	-0.12	-0.02	-0.25	-0.16	-1.30
-0.53	-0.60	-0.20	-0.65	-1.00	-0.78	-1.25
-0.37	-0.03	-0.01	2.85	-0.84	1.98	1.93
-1.57	-0.48	-0.31	-0.78	-0.20	-0.77	2.02
0.31	-0.61	-0.52	-0.85	-0.75	-0.91	1.44
0.77	-0.68	-0.61	-0.83	-1.14	-0.85	1.26
-0.94	-0.41	-0.31	-1.06	-0.25	-0.79	-0.98
-1.48	0.65	-0.03	-0.90	-0.80	0.19	-1.16
-0.41	0.26	1.44	0.15	2.66	0.94	-0.71
-0.62	-0.68	-0.46	-0.87	-1.06	-0.80	0.50
0.02	3.65	-0.33	0.89	0.93	-0.77	-0.51
-0.26	-0.62	-0.52	-0.94	-0.60	-0.77	0.18
-0.38	0.82	-0.42	-0.19	0.03	-0.44	-0.15
0.86	1.32	0.50	1.67	2.67	0.01	0.04
0.79	-0.62	-0.37	0.05	0.33	1.84	0.15
1.69	0.04	-0.21	0.35	-0.96	0.33	-1.06
-1.09	-0.10	-0.17	0.53	-0.34	-0.36	-0.14
1.42	1.58	-0.29	1.56	-0.08	-0.49	-0.77
-1.56	-0.24	-0.48	-0.42	-0.18	-0.30	0.58
1.04	-0.70	-0.26	-0.14	0.08	-0.16	0.92
1.58	-0.55	-0.28	-0.16	0.50	0.00	0.85
0.57	-0.65	-0.54	-0.79	-0.47	0.26	0.84
-0.97	-0.50	0.38	0.00	0.18	1.29	0.69
0.43	-0.44	-0.10	1.88	0.68	2.72	-0.56
-0.16	1.26	0.18	-0.02	1.62	-0.50	-1.03
-1.11	2.44	0.45	0.53	2.05	-0.59	-1.52
-1.90	-0.36	0.29	1.60	1.23	-0.11	-0.16
-2.45	-0.50	-0.07	-0.40	-0.20	-0.55	-0.90
-1.11	-0.61	-0.21	-1.23	-1.12	-0.96	-0.21
-2.10	-0.34	1.08	-0.68	1.36	-0.52	-0.56
-0.74	1.18	0.73	3.83	0.79	-0.69	-0.59
-0.37	-0.56	-0.57	-0.48	-0.82	-1.11	0.18
-0.43	-0.49	-0.50	-0.41	-0.89	-1.04	0.25
-0.78	-0.60	-0.35	0.51	-0.56	0.13	0.25
-2.02	-0.77	-0.67	-1.29	-1.39	-1.09	0.03
-0.75	-0.06	-0.26	-0.50	0.16	-0.85	-0.64
-0.21	2.27	0.43	0.80	1.75	0.08	-1.46

0.55	-0.58	-0.09	0.79	-0.19	0.20	0.18
-0.79	-0.71	-0.59	-0.82	-0.54	7.67	0.80
-0.73	-0.65	-0.39	-0.92	4.25	54.32	3.37
0.39	-0.53	-0.33	-0.09	1.35	1.01	0.31
-1.43	1.26	-0.26	0.79	0.08	-0.42	-0.10
-0.60	-0.56	-0.43	-0.65	-0.86	-0.49	3.01
-0.85	-0.78	-0.65	-1.11	-1.53	7.11	1.26
0.82	-0.12	-0.25	5.19	1.79	0.78	-0.61
1.89	-0.61	-0.45	0.73	-0.08	-0.31	1.21
3.59	-0.11	-0.44	0.53	0.09	-0.56	-0.49
1.84	-0.59	-0.45	2.18	-0.49	0.22	-0.41
2.95	0.00	-0.29	1.43	1.41	-0.34	-0.45
0.67	-0.65	-0.43	0.38	1.74	0.91	0.75
-0.78	-0.55	-0.40	-0.70	0.73	-0.39	0.05
0.77	-0.12	-0.22	1.66	2.06	0.68	0.03
-0.69	-0.64	-0.47	-0.36	-0.56	-0.71	1.40
-0.37	-0.63	-0.45	0.14	0.23	1.78	0.02
0.20	-0.11	-0.28	0.03	0.42	0.11	-0.26
-1.13	5.38	0.72	2.18	0.66	-0.37	-1.38
0.73	-0.60	-0.23	0.74	0.28	0.49	0.58
2.52	0.34	0.01	3.60	1.59	0.33	1.25
1.27	0.19	0.61	5.59	0.37	0.96	0.18
-1.15	6.59	0.09	0.90	0.48	-0.66	-1.48
0.27	-0.64	-0.39	-0.39	-0.67	0.10	-0.75
-0.30	-0.48	-0.26	0.23	-0.37	0.27	-0.29
-0.57	0.21	-0.27	0.01	-0.17	-0.01	-1.23
0.49	-0.53	-0.11	-0.88	-0.46	-1.20	-0.44
-0.81	0.14	-0.45	-0.02	0.36	0.20	-1.61
-2.17	-0.81	1.23	-0.31	0.19	-0.18	-0.14
5.34	-0.44	-0.01	-0.20	-0.48	-0.47	-0.42
5.55	-0.29	0.18	2.06	0.55	-0.17	-0.11
-0.14	-0.66	-0.40	-0.52	-0.72	-0.85	-0.51
-1.03	0.38	-0.34	0.35	-1.25	-0.88	-1.41
-0.74	-0.12	-0.35	-0.57	-0.66	-0.80	-1.08
1.01	1.49	-0.15	-0.20	-0.47	-0.95	-0.87
-1.12	-0.69	-0.70	-0.75	0.17	59.63	4.27
-0.36	0.96	-0.42	0.61	-0.19	-0.73	-0.16
-1.54	0.04	-0.31	0.21	0.08	-0.26	0.05
-1.86	-0.41	-0.17	-0.77	-0.99	-0.64	2.58
0.57	0.68	0.36	2.76	2.25	0.73	-1.27
0.66	0.04	0.20	3.63	2.99	0.31	-0.68
0.67	-0.65	-0.30	0.20	0.32	1.25	-0.41
-0.73	-0.37	-0.16	2.88	0.42	1.07	-0.46
1.28	-0.57	-0.54	-0.08	-0.30	-0.28	0.52
1.16	1.76	-0.01	1.23	0.33	-0.02	-1.14
-0.74	4.35	-0.23	0.49	-0.56	-0.89	-1.51
0.70	-0.49	-0.41	1.27	2.97	0.70	0.01
1.07	-0.48	-0.08	2.79	-0.11	0.61	-0.49
-0.31	-0.57	-0.54	0.99	-0.55	0.55	-0.61
-0.50	1.26	-0.41	0.92	-0.79	-0.80	0.12
-0.30	-0.44	-0.37	2.22	1.12	0.26	-0.16
-0.11	1.60	0.83	0.16	-0.32	-0.45	-0.80
-1.93	-0.62	-0.51	-0.28	-1.15	-0.75	0.63
1.81	-0.63	-0.36	0.04	-0.14	0.21	0.98
-1.52	0.06	0.62	-0.03	0.84	-0.17	-0.75
-0.38	-0.15	0.07	0.32	1.67	1.86	-1.33

0.07	0.07	0.07	0.07	0.07	0.07	0.07
0.08	0.06	0.06	0.08	0.04	0.03	0.18

148505	1922117	685397	2915691	932164	148130	2018570

36	37	38	39	40	41	42
6	7	8	9	10	11	12
B_30	B_33	C_2	C_37	C_38	C_39	C_43
CHR_NP	CHR_NP	CHR_NP	CHR_NP	CHR_NP	CHR_NP	CHR_NP
852085	4579742	2339361	4568063	3630812	258656	5675202
0.39	0.91	0.50	0.52	0.44	0.13	0.17
B_30	B_33	C_2	C_37	C_38	C_39	C_43
703580	2657625	1653964	1652372	2698648	110526	3656632
4948	1007604	324840	1119107	698786	56414	382904
27066	82363	53864	107450	11085	4692	49604
6598	53526	26948	527415	18116	13820	77016
19460	105395	30924	186072	37780	6531	62996
4335	185758	52795	221867	36882	11931	143942
21454	22114	14962	22930	3941	3244	97556
15329	42853	11026	94903	4076	6644	91561
2182	4727	8808	5144	336	1560	352009
731	14989	4841	20714	1927	1860	50045
3504	41754	23520	60783	6245	7346	116265
1040	18898	8215	32180	6565	5855	75495
1737	26780	9268	36680	7874	1869	28048
2414	9346	2814	49017	1645	2208	28494
1939	29210	14032	24453	24432	1247	16962
2405	3647	1546	8257	694	1172	28299
145	33307	3175	39192	13559	450	5400
2972	4989	1148	9621	146	686	21808
1417	8203	5081	12394	826	1168	18331
933	6588	2949	15407	717	918	26233
438	14685	3594	24919	1038	1631	17880
623	10198	3542	20190	1251	1020	15754
355	9348	4198	9877	1143	841	20274
195	8208	1746	11176	292	1148	13470
2483	9903	3570	12983	77	333	16065
618	960	351	6261	89	832	10359
78	8786	2119	15706	1088	781	6546
387	7245	2471	9772	5049	303	3559
209	3111	2406	849	240	517	11574
1384	1840	832	5551	587	227	4758
118	5546	1298	10136	844	393	5438
712	6198	2874	9819	441	586	7562
698	5805	1680	10464	3285	188	2775
323	2251	767	4123	191	168	8470
235	3245	1664	2212	114	567	22075
1268	1654	759	5155	548	208	4446
3972	9791	8590	6463	2315	359	6902
1051	2501	1914	5083	425	812	14383
858	1145	524	4974	193	455	9827
764	7023	2647	8506	602	173	2542
126	5223	653	10639	14956	57	691
602	13459	3447	10287	568	251	3486
64	2501	1026	8284	774	317	3949
608	5028	1644	5680	1033	99	2057
608	572	534	672	66	204	5936
314	1352	1203	6637	148	185	10804

93	3818	1085	6740	861	199	2797
134	1371	356	3002	208	131	4229
71	4788	1628	3092	1921	111	879
35	117	59	227	80	7	485
32	4093	805	2038	43	333	5425
129	1251	597	741	308	177	1261
829	1597	1573	2395	53	267	5813
186	2136	1799	1818	379	160	2939
89	2497	468	2899	117	243	2661
3	2771	336	2193	110	149	1297
5	119	63	205	19	58	2116
374	1787	972	1974	277	83	3015
128	3593	697	2658	128	111	2724
77	1228	427	4160	175	194	3000
1831	389	166	1502	354	43	668
177	1864	741	3150	172	202	4586
59	1616	701	2575	92	164	3392
78	105	93	261	28	30	2396
17	92	58	431	30	17	309
42	1652	403	724	3378	35	294
186	639	285	640	51	63	2082
59	413	787	537	33	237	4285
116	270	106	292	9	34	1456
115	928	884	1126	12	88	2265
34	375	377	254	70	15	769
125	2651	534	2383	633	135	1280
86	1226	451	1528	1881	58	498
88	106	54	207	26	68	2189
11	748	259	1318	121	95	2107
178	427	162	1376	19	32	1048
39	1006	272	361	228	126	911
1	493	138	878	127	41	517
418	104	84	414	86	23	635
94	1336	321	1539	640	24	352
141	567	423	1370	103	194	2209
137	401	188	761	153	19	631
5	1161	369	1591	252	41	620
117	593	318	1085	713	24	169
8	213	258	341	8	209	5325
41	336	134	464	19	16	1258
25	818	532	1293	65	60	1721
48	983	385	1114	522	30	329
67	135	41	205	12	15	1000
92	802	140	1331	49	34	507
175	231	36	746	163	17	181
9	186	87	1017	64	31	490
4	21	21	56	22	4	158
2	799	96	1035	56	15	195
24	100	52	286	14	23	500
14	121	65	104	14	14	1025
66	261	94	426	16	28	540
23	513	187	812	237	24	107
15	514	204	433	184	33	231
13	164	88	831	69	35	232
37	65	29	84	6	15	806
39	688	362	879	13	41	884
23	117	47	389	32	24	220
24	145	67	1220	189	20	273
73	90	49	374	9	24	810

43	147	175	171	24	41	414
14	125	62	101	5	13	1136
63	684	406	555	47	13	291
81	184	146	307	15	28	486
40	170	87	154	14	13	586
29	84	52	112	8	10	272
19	153	105	367	20	26	707
8	323	93	770	55	20	263
20	704	615	460	15	36	724
47	89	46	58	8	9	324
12	62	17	1363	16	40	503
18	206	70	1481	117	17	154
4	141	58	236	43	17	521
58	125	41	125	9	11	283
58	125	41	125	9	11	283
90	500	306	504	19	20	740
6	329	188	227	70	17	353
3	345	124	498	53	12	279
2	144	20	39	10	25	182
7	127	46	205	7	20	422
0	24	7	85	4	3	100
15	165	56	250	54	17	149
6	34	15	279	18	5	178
1	124	60	308	21	13	139
28	173	74	293	12	3	139
22	449	106	301	20	1	59
0	21	17	42	10	1	70
20	155	65	169	3	8	215
14	60	19	124	6	11	279
36	189	67	288	28	21	200
3	42	18	112	2	4	183
6	161	95	215	16	18	405
2	199	23	200	30	14	206
2	74	23	71	18	5	349
9	63	14	116	4	4	98
13	202	50	202	22	6	175
1	179	34	225	55	5	67
16	105	61	159	38	9	176
6	201	21	229	267	7	36
32	145	28	177	7	4	65
5	21	15	26	4	0	197
4	94	25	50	118	4	57
19	363	59	392	26	6	69
6	57	15	99	6	4	67
28	117	35	99	64	5	45
14	153	72	131	19	3	61
3	56	36	121	6	12	205
25	55	19	43	11	4	177
0	54	7	11	2	9	167
3	295	45	230	20	2	38
0	68	24	102	8	4	155
14	108	106	78	73	2	116
9	25	14	57	5	0	103
23	191	103	160	17	9	214
2	101	43	210	10	8	94
3	100	21	49	61	6	38
3	6	2	20	0	1	26
9	153	81	211	13	13	210
17	119	42	130	63	4	61

7	57	16	93	1	13	169
11	43	13	78	1	4	159
5	110	33	79	154	7	73
14	70	34	172	14	2	86
9	44	221	37	28	15	153
5	18	8	31	0	3	95
5	68	46	40	21	1	118
14	103	30	149	1	9	110
1	60	14	133	4	13	146
2	36	24	133	36	8	21
12	64	28	113	4	6	84
3	41	7	57	0	4	86
10	131	41	176	27	2	17
7	66	32	13	9	12	81
5	16	7	77	3	6	97
14	69	45	111	1	9	312
8	104	36	28	11	8	94
1	89	49	146	44	6	132
11	11	1	39	1	1	128
0	20	25	5	12	11	49
15	31	12	49	2	4	124
3	34	13	61	43	5	50
6	80	19	126	89	3	30
11	11	1	39	1	0	112
4	47	11	33	3	16	95
6	44	27	93	11	5	112
3	52	5	109	5	0	98
1	1	0	0	3	0	0
10	9	28	11	3	5	589
11	45	30	83	0	5	87
7	80	11	83	52	3	5
1	49	17	83	20	5	65
6	14	6	287	4	3	51
0	27	3	6	0	4	52
4	42	15	29	26	2	49
0	60	56	64	1	7	151
3	25	15	64	2	5	41
1	46	2	96	20	0	3
7	32	12	172	2	5	53
8	41	18	108	12	0	17
0	61	38	57	5	6	63
7	21	26	19	4	2	174
1	40	3	17	3	2	7
4	51	12	44	42	1	21
4	97	15	100	12	3	46
5	24	11	56	6	6	60
5	66	29	41	66	5	31
6	65	16	149	8	2	29
8	72	17	79	1	1	33
3	5	1	3	1	4	60
10	56	44	59	54	2	24
2	38	9	13	3	6	42
2	36	8	75	8	0	14
2	50	13	65	7	3	31
0	51	5	70	3	3	21
0	1	0	4	0	0	18
9	31	8	78	8	3	36
12	2	2	2	0	2	64
2	51	24	32	11	2	64

2	38	18	75	0	3	129
6	34	25	43	10	4	123
8	26	2	11	2	3	14
1	24	7	86	24	1	19
1	15	8	60	22	1	5
1	44	8	18	25	0	6
4	34	19	75	10	0	21
5	23	20	36	4	2	88
7	5	10	22	0	9	124
1	19	5	31	15	0	9
3	3	0	10	0	0	60
0	39	14	59	11	5	33
0	12	2	33	3	2	13
1	12	10	25	9	0	32
0	9	5	40	2	3	39
9	31	14	47	2	4	148
1	28	7	24	0	1	37
3	27	9	32	7	3	12
2	22	7	9	1	9	18
0	32	8	57	0	2	120
7	8	0	12	1	2	65
1	19	12	16	0	2	52
1	19	12	16	0	2	52
1	12	5	11	8	2	14
4	47	19	49	7	0	10
3	8	9	19	1	0	22
1	12	6	6	0	2	12
4	25	3	42	1	0	13
3	65	5	35	2	1	26
3	4	7	33	2	6	64
3	16	9	20	13	2	14
1	3	5	48	4	1	8
11	36	18	24	1	2	16
4	19	8	27	1	0	3
1	34	7	94	12	5	39
5	12	14	26	3	2	59
1	0	1	1	2	0	15
3	25	9	22	3	0	41
2	4	2	24	1	0	30
2	10	10	50	8	0	8
3	16	17	14	47	0	10
1	1	2	22	1	0	28
6	6	2	15	4	0	32
5	9	4	20	4	0	7
1	7	2	18	1	1	34
3	21	4	23	25	0	23
0	3	4	1	1	0	13
0	7	1	71	8	0	3
2	25	11	22	27	0	10
1	28	6	8	20	0	19
1	27	3	12	4	2	13
3	7	5	17	0	2	23
0	0	1	1	0	0	10
4	11	20	33	1	2	8
1	13	6	22	0	2	56
0	7	9	32	64	3	43
0	15	6	21	15	0	9
0	5	5	51	3	1	31
6	21	7	26	8	1	12

3	24	11	12	1	0	13
2	16	7	30	1	0	18
0	7	1	7	4	5	7
1	22	9	33	5	2	15
1	19	5	26	4	1	11
2	20	1	31	7	0	5
1	13	3	21	0	1	47
3	18	8	8	3	3	30
1	0	0	2	0	1	23
2	3	6	9	0	2	10
1	2	0	91	0	1	13
0	19	14	14	5	3	1
0	12	11	19	0	4	71
0	17	6	22	4	1	15
1	7	3	6	3	0	5
1	6	3	7	1	0	32
3	9	4	9	16	0	4
0	8	3	27	1	1	18
1	16	4	24	2	3	12
0	7	3	7	0	3	13
0	11	1	6	10	0	6
3	12	2	35	3	0	4
0	32	7	28	3	0	6
0	10	7	10	40	0	8
0	8	1	20	2	0	17
1	14	5	36	7	0	8
0	9	3	36	1	0	16
0	10	4	26	9	2	22
0	36	12	28	0	0	16
0	10	0	20	2	0	15
0	12	4	6	7	1	10
1	3	0	2	2	0	14
1	1	1	0	0	0	16
0	13	2	3	1	2	25
3	4	7	13	1	0	11
1	18	15	9	9	2	19
0	13	7	22	0	1	17
0	14	10	13	0	0	7
1	4	0	9	3	0	5
1	17	3	34	1	0	4
2	2	1	10	0	1	18
1	15	2	19	1	0	6
0	9	0	2	1	2	1
1	14	5	14	4	0	3
0	5	0	4	1	0	16
4	0	2	7	8	4	42
0	19	2	12	10	1	17
1	2	2	1	0	3	13
0	13	4	9	8	1	16
2	16	4	33	3	2	5
0	10	2	5	5	2	1
0	1	0	3	0	0	27
1	24	5	15	8	0	9
4	1	1	3	0	0	13
2	10	2	12	3	0	3
1	3	4	20	3	1	15
0	30	2	18	3	0	2
0	0	0	1	0	0	4
1	3	5	9	3	0	6

0	12	2	9	7	1	18
0	14	5	14	6	0	2
0	2	3	19	2	2	10
1	18	10	14	2	1	13
0	12	2	6	2	2	6
0	1	1	14	1	1	2
1	5	3	15	6	1	7
0	2	0	0	0	0	18
1	4	2	6	0	0	21
0	7	6	13	0	0	11
1	19	6	8	1	2	9
0	3	2	14	0	0	3
21	15	4	17	3	1	11
0	1	1	1	0	0	48
2	5	1	12	1	0	2
0	6	5	4	1	1	33
0	2	2	19	0	0	14
0	11	2	8	1	1	7
0	3	5	5	0	0	21
2	6	4	10	0	0	22
0	9	1	17	1	2	26
0	14	3	11	0	1	12
0	0	0	16	0	0	5
0	6	4	13	5	0	6
1	3	5	3	5	0	4
0	0	0	15	4	0	11
0	1	1	3	0	0	5
2	2	0	6	0	0	18
0	9	4	10	0	2	4
0	19	3	8	0	0	0
0	1	0	1	2	0	7
2	6	1	9	3	0	4
0	3	0	8	0	0	6
0	0	0	9	7	0	2
0	2	1	4	0	0	2
0	4	2	2	2	1	4
0	5	5	8	0	0	9
0	5	7	3	7	0	2
1	11	2	6	1	0	3
0	10	2	11	4	0	0
0	8	1	12	3	0	0
0	0	0	5	1	0	4
0	2	2	3	0	1	4
0	7	0	6	2	0	2
0	1	1	22	0	1	6
1	13	8	5	1	0	7
0	1	0	9	1	0	7
0	5	3	7	5	0	1
0	3	2	5	0	1	12
0	2	0	1	0	0	0
0	11	0	10	1	0	4
0	3	0	0	0	0	2
0	0	3	1	0	0	15
0	14	6	9	1	1	10
0	3	0	7	0	0	3
0	1	2	2	0	0	12
0	4	0	7	0	1	6
0	2	5	9	1	0	5
0	22	1	0	0	0	0

1	0	1	0	0	0	5
0	8	2	4	0	0	11
0	0	0	3	0	1	3
0	0	0	5	0	0	1
0	1	2	5	1	0	4
0	3	2	5	6	0	0
0	4	6	4	4	1	5
0	1	0	6	0	1	4
0	3	0	6	4	0	1
0	2	0	5	0	2	6
0	0	2	1	0	1	22
0	0	0	0	0	0	0
0	0	0	0	0	0	0
0	6	0	2	0	0	8
0	0	1	9	1	0	3
0	2	2	1	0	0	9
1	2	2	3	6	1	1
0	2	1	1	1	0	7
0	0	0	0	0	0	1
1	1	0	1	0	0	7
0	2	2	0	0	0	14
0	1	6	1	0	0	18
1	2	1	5	2	0	0
0	9	2	3	1	0	2
0	2	0	6	3	2	0
0	0	0	0	0	0	3
0	2	0	5	0	0	6
1	4	0	1	0	0	4
0	1	0	1	3	0	2
0	5	2	1	0	0	3
0	0	1	3	0	0	14
0	4	0	5	0	0	1
0	1	2	3	3	0	4
0	2	0	3	24	0	0
1	6	3	4	1	0	4
0	1	1	3	0	0	4
0	1	0	8	3	0	0
0	5	1	2	1	0	3
0	5	2	4	0	1	6
1	3	0	0	1	0	5
0	0	0	0	0	0	3
1	4	4	4	1	0	7
1	0	1	3	1	0	5
0	2	0	3	2	0	0
1	7	3	3	1	1	6
1	0	0	0	0	0	3
1	1	0	0	0	0	3
0	3	2	6	0	0	3
0	2	1	2	2	0	0
0	1	0	7	0	0	4
1	1	1	4	0	0	1
0	1	0	0	1	0	6
2	2	0	5	1	0	0
0	2	1	1	0	1	6
1	0	1	3	0	0	2
0	1	1	14	0	0	4
0	4	1	0	0	0	7
0	2	0	1	1	0	0
0	3	2	3	0	0	2

0	12	0	2	0	0	0
0	6	1	6	3	0	0
0	3	1	3	1	2	0
0	3	1	9	0	0	14
0	2	0	1	0	0	2
0	1	0	1	0	0	10
0	2	1	7	0	0	0
0	1	1	0	0	0	1
0	2	1	3	0	0	2
0	2	1	0	0	0	18
0	2	4	0	3	1	1
0	0	1	0	0	0	2
0	0	0	2	0	0	9
0	3	0	2	3	1	0
0	0	3	0	1	0	2
2	1	0	2	0	0	4
1	2	1	1	0	0	3
0	0	0	0	0	0	1
0	0	0	0	0	0	3
0	1	2	4	1	0	6
0	3	1	2	0	0	6
0	0	0	2	0	0	6
0	0	0	1	0	0	5
1	6	2	4	0	0	1
0	3	2	6	0	0	0
0	0	0	4	0	0	1
0	1	0	2	1	0	1
0	0	0	5	1	0	0
0	2	0	0	0	0	2
1	2	2	0	0	0	10
1	0	0	0	0	0	2
1	7	2	1	2	0	1
1	2	0	4	0	0	1
0	11	4	1	0	0	0
0	2	2	4	0	1	3
0	2	1	1	0	0	6
0	1	0	2	1	0	0
0	5	0	0	0	0	3
0	0	2	4	0	0	2
0	0	0	10	0	0	0
0	2	0	4	0	1	13
1	1	2	1	2	0	2
0	1	0	1	1	0	1
0	4	0	6	2	0	0
0	1	1	2	0	0	2
0	0	0	1	2	0	0
0	0	0	3	0	0	1
0	1	0	1	1	0	0
0	1	1	1	1	0	0
0	2	0	3	8	0	2
0	2	1	6	1	0	1
0	1	0	4	0	0	0
0	0	0	0	1	0	10
0	0	0	8	0	0	12
0	3	0	1	2	0	2
0	0	0	0	1	0	2
0	2	1	5	1	0	1
0	1	1	4	0	0	0
0	3	1	0	0	0	4

0	1	0	10	0	0	3
0	2	2	4	0	0	2
0	1	1	0	0	0	0
0	2	1	1	1	0	0
0	0	0	11	0	0	0
0	2	0	2	1	0	1
0	2	1	2	1	0	0
0	6	2	1	1	0	1
0	3	1	0	2	0	2
0	2	1	1	1	0	0
0	1	0	8	2	0	1
1	1	2	0	1	0	1
1	0	0	0	2	0	0
B_30	B_33	C_2	C_37	C_38	C_39	C_43
CHR_NP	CHR_NP	CHR_NP	CHR_NP	CHR_NP	CHR_NP	CHR_NP
703580	2657625	1653964	1652372	2698648	110526	3656632
26	19	22	12	23	14	21
1.03	-0.59	0.24	-2.03	0.42	-1.59	-0.12

0.90	0.12	0.08	0.02	0.34	0.93	0.94
8	30	31	37	20	6	4
1	1	1	1	1	1	1
36	37	38	39	40	41	42
9	10	11	12	13	14	15
C_37	C_38	C_39	C_43	C_48	D_10	D_12
CHR_NP	CHR_NP	CHR_NP	CHR_NP	CHR_NP	CHR_NP	CHR_NP
2915691	932164	148130	2018570	2072241	727386	206047
C_37	C_38	C_39	C_43	C_48	D_10	D_12
1119107	698786	56414	382904	593526	308682	28125
107450	11085	4692	49604	152869	23073	6563
527415	18116	13820	77016	385992	63225	24309
186072	37780	6531	62996	114740	11822	2047
221867	36882	11931	143942	254736	89228	33653
22930	3941	3244	97556	27400	2763	381
94903	4076	6644	91561	104908	22052	6808
5144	336	1560	352009	7545	2512	2325
20714	1927	1860	50045	22013	4185	2552
60783	6245	7346	116265	23827	6144	1172
32180	6565	5855	75495	12756	23750	19091
36680	7874	1869	28048	35505	8613	2253
49017	1645	2208	28494	21950	4686	1683
24453	24432	1247	16962	18735	15532	6102
8257	694	1172	28299	10207	613	202
39192	13559	450	5400	12774	8711	4731
9621	146	686	21808	9339	790	192
12394	826	1168	18331	12358	5454	2635
15407	717	918	26233	13684	609	126

24919	1038	1631	17880	15631	6584	1697
20190	1251	1020	15754	16830	6083	3941
9877	1143	841	20274	7357	1802	494
11176	292	1148	13470	4425	990	270
12983	77	333	16065	13111	467	230
6261	89	832	10359	8660	1201	676
15706	1088	781	6546	6792	2657	1291
9772	5049	303	3559	5710	8014	7864
849	240	517	11574	2119	1372	413
5551	587	227	4758	4318	1315	494
10136	844	393	5438	4042	7254	2374
9819	441	586	7562	9334	1073	264
10464	3285	188	2775	7112	1333	440
4123	191	168	8470	3775	1146	644
2212	114	567	22075	2250	973	258
5155	548	208	4446	4019	1204	459
6463	2315	359	6902	5146	2115	172
5083	425	812	14383	4433	1060	646
4974	193	455	9827	3480	769	665
8506	602	173	2542	4769	6449	1347
10639	14956	57	691	7744	9122	3042
10287	568	251	3486	6633	10386	2733
8284	774	317	3949	8448	3106	999
5680	1033	99	2057	2888	3292	742
672	66	204	5936	1156	220	90
6637	148	185	10804	7260	1532	1263
6740	861	199	2797	3352	978	839
3002	208	131	4229	2009	1205	1306
3092	1921	111	879	3270	1891	1190
227	80	7	485	135	32	32
2038	43	333	5425	1169	758	464
741	308	177	1261	918	1652	870
2395	53	267	5813	3210	754	231
1818	379	160	2939	1434	3074	2195
2899	117	243	2661	2549	85	37
2193	110	149	1297	1239	1707	698
205	19	58	2116	113	79	29
1974	277	83	3015	1573	657	362
2658	128	111	2724	2705	111	65
4160	175	194	3000	2566	219	72
1502	354	43	668	2358	658	360
3150	172	202	4586	1390	1142	1023
2575	92	164	3392	2349	809	669
261	28	30	2396	150	8	15
431	30	17	309	108	25	13
724	3378	35	294	515	234	92
640	51	63	2082	552	174	42
537	33	237	4285	745	176	85
292	9	34	1456	232	116	21
1126	12	88	2265	899	1027	513
254	70	15	769	331	143	14
2383	633	135	1280	1080	831	161
1528	1881	58	498	412	791	212
207	26	68	2189	265	61	48
1318	121	95	2107	512	224	76
1376	19	32	1048	1116	95	20
361	228	126	911	618	491	265
878	127	41	517	747	835	328
414	86	23	635	416	400	388

1539	640	24	352	921	780	301
1370	103	194	2209	834	208	124
761	153	19	631	813	107	59
1591	252	41	620	834	855	493
1085	713	24	169	787	147	11
341	8	209	5325	197	33	4
464	19	16	1258	400	175	197
1293	65	60	1721	702	2554	2880
1114	522	30	329	564	1136	1039
205	12	15	1000	224	13	6
1331	49	34	507	516	65	45
746	163	17	181	448	304	145
1017	64	31	490	856	347	152
56	22	4	158	9	21	16
1035	56	15	195	633	291	130
286	14	23	500	181	24	15
104	14	14	1025	107	41	8
426	16	28	540	618	132	46
812	237	24	107	554	240	121
433	184	33	231	429	423	274
831	69	35	232	824	448	124
84	6	15	806	65	10	4
879	13	41	884	492	887	245
389	32	24	220	304	45	23
1220	189	20	273	688	510	399
374	9	24	810	465	28	20
171	24	41	414	200	31	9
101	5	13	1136	106	26	14
555	47	13	291	436	998	754
307	15	28	486	387	66	11
154	14	13	586	110	98	18
112	8	10	272	117	345	105
367	20	26	707	502	6	9
770	55	20	263	386	167	57
460	15	36	724	310	670	329
58	8	9	324	86	28	6
1363	16	40	503	524	70	109
1481	117	17	154	256	58	34
236	43	17	521	329	31	20
125	9	11	283	113	75	34
125	9	11	283	113	75	34
504	19	20	740	613	17	4
227	70	17	353	128	332	286
498	53	12	279	276	17	8
39	10	25	182	88	9	0
205	7	20	422	266	30	19
85	4	3	100	28	39	21
250	54	17	149	209	96	69
279	18	5	178	206	390	617
308	21	13	139	260	58	28
293	12	3	139	126	282	245
301	20	1	59	158	79	13
42	10	1	70	41	180	45
169	3	8	215	232	84	41
124	6	11	279	122	30	45
288	28	21	200	103	385	217
112	2	4	183	64	169	229
215	16	18	405	188	62	60
200	30	14	206	70	36	30

71	18	5	349	58	88	147
116	4	4	98	86	5	13
202	22	6	175	119	145	139
225	55	5	67	136	118	49
159	38	9	176	124	38	19
229	267	7	36	250	168	81
177	7	4	65	80	36	12
26	4	0	197	10	19	26
50	118	4	57	57	67	39
392	26	6	69	201	31	2
99	6	4	67	77	4	1
99	64	5	45	117	21	10
131	19	3	61	61	217	78
121	6	12	205	114	50	18
43	11	4	177	46	24	20
11	2	9	167	36	9	0
230	20	2	38	161	45	12
102	8	4	155	226	9	2
78	73	2	116	30	92	63
57	5	0	103	81	27	25
160	17	9	214	64	193	86
210	10	8	94	82	119	92
49	61	6	38	19	69	71
20	0	1	26	3	3	3
211	13	13	210	156	214	116
130	63	4	61	72	138	71
93	1	13	169	66	22	6
78	1	4	159	74	27	33
79	154	7	73	62	89	31
172	14	2	86	84	13	1
37	28	15	153	95	312	223
31	0	3	95	54	2	0
40	21	1	118	25	21	10
149	1	9	110	151	271	79
133	4	13	146	51	124	23
133	36	8	21	166	16	12
113	4	6	84	102	17	1
57	0	4	86	88	12	1
176	27	2	17	77	389	116
13	9	12	81	17	58	48
77	3	6	97	76	35	24
111	1	9	312	64	105	62
28	11	8	94	40	157	29
146	44	6	132	103	22	12
39	1	1	128	22	9	6
5	12	11	49	18	93	40
49	2	4	124	52	4	1
61	43	5	50	28	41	20
126	89	3	30	82	115	118
39	1	0	112	22	9	5
33	3	16	95	29	35	7
93	11	5	112	70	31	69
109	5	0	98	76	14	7
0	3	0	0	2	36	51
11	3	5	589	24	1	0
83	0	5	87	77	24	18
83	52	3	5	83	13	2
83	20	5	65	31	20	13
287	4	3	51	49	34	28

6	0	4	52	16	6	0
29	26	2	49	29	22	9
64	1	7	151	33	115	95
64	2	5	41	61	52	46
96	20	0	3	101	22	11
172	2	5	53	57	3	2
108	12	0	17	46	102	66
57	5	6	63	32	38	18
19	4	2	174	19	27	5
17	3	2	7	20	51	50
44	42	1	21	58	49	34
100	12	3	46	69	14	2
56	6	6	60	29	11	21
41	66	5	31	19	35	24
149	8	2	29	75	45	30
79	1	1	33	95	23	4
3	1	4	60	14	1	0
59	54	2	24	54	68	41
13	3	6	42	19	37	21
75	8	0	14	80	44	37
65	7	3	31	61	14	8
70	3	3	21	61	16	13
4	0	0	18	1	1	1
78	8	3	36	45	6	0
2	0	2	64	8	2	0
32	11	2	64	35	39	34
75	0	3	129	40	13	21
43	10	4	123	29	56	26
11	2	3	14	25	4	2
86	24	1	19	48	44	25
60	22	1	5	26	33	29
18	25	0	6	16	14	15
75	10	0	21	36	35	16
36	4	2	88	25	9	2
22	0	9	124	19	6	6
31	15	0	9	15	20	10
10	0	0	60	9	1	0
59	11	5	33	21	59	34
33	3	2	13	18	5	6
25	9	0	32	12	39	56
40	2	3	39	43	108	118
47	2	4	148	14	0	2
24	0	1	37	34	14	6
32	7	3	12	31	7	5
9	1	9	18	6	21	8
57	0	2	120	23	31	26
12	1	2	65	13	2	1
16	0	2	52	15	3	0
16	0	2	52	15	3	0
11	8	2	14	21	25	7
49	7	0	10	32	35	19
19	1	0	22	14	22	10
6	0	2	12	14	9	4
42	1	0	13	30	1	0
35	2	1	26	44	16	15
33	2	6	64	11	68	22
20	13	2	14	13	5	1
48	4	1	8	28	46	160
24	1	2	16	52	5	2

27	1	0	3	70	13	0
94	12	5	39	24	0	3
26	3	2	59	21	9	2
1	2	0	15	0	1	4
22	3	0	41	15	20	8
24	1	0	30	17	3	0
50	8	0	8	32	32	13
14	47	0	10	11	12	12
22	1	0	28	17	3	0
15	4	0	32	22	23	6
20	4	0	7	12	11	7
18	1	1	34	11	7	4
23	25	0	23	10	19	13
1	1	0	13	2	4	1
71	8	0	3	34	43	78
22	27	0	10	25	18	9
8	20	0	19	10	15	21
12	4	2	13	9	4	2
17	0	2	23	14	2	1
1	0	0	10	1	1	1
33	1	2	8	21	10	1
22	0	2	56	29	2	1
32	64	3	43	17	0	23
21	15	0	9	12	24	31
51	3	1	31	18	0	2
26	8	1	12	23	34	9
12	1	0	13	7	38	17
30	1	0	18	17	4	0
7	4	5	7	12	11	8
33	5	2	15	24	20	12
26	4	1	11	5	10	11
31	7	0	5	18	14	3
21	0	1	47	27	1	1
8	3	3	30	15	2	1
2	0	1	23	1	3	0
9	0	2	10	5	0	0
91	0	1	13	21	0	1
14	5	3	1	12	30	7
19	0	4	71	7	14	4
22	4	1	15	41	8	6
6	3	0	5	2	15	5
7	1	0	32	11	0	1
9	16	0	4	10	8	4
27	1	1	18	14	36	21
24	2	3	12	14	39	27
7	0	3	13	7	0	2
6	10	0	6	8	10	6
35	3	0	4	14	7	2
28	3	0	6	18	27	9
10	40	0	8	2	2	15
20	2	0	17	12	2	0
36	7	0	8	20	9	5
36	1	0	16	23	19	10
26	9	2	22	6	27	37
28	0	0	16	16	19	32
20	2	0	15	22	2	4
6	7	1	10	10	1	0
2	2	0	14	10	0	1
0	0	0	16	5	0	0

3	1	2	25	4	1	1
13	1	0	11	11	0	0
9	9	2	19	7	21	10
22	0	1	17	10	3	1
13	0	0	7	2	13	27
9	3	0	5	13	9	1
34	1	0	4	18	6	3
10	0	1	18	8	5	4
19	1	0	6	20	3	8
2	1	2	1	5	8	2
14	4	0	3	11	4	5
4	1	0	16	8	0	3
7	8	4	42	16	0	5
12	10	1	17	7	9	7
1	0	3	13	1	5	2
9	8	1	16	8	26	11
33	3	2	5	18	5	1
5	5	2	1	8	12	12
3	0	0	27	0	0	2
15	8	0	9	12	29	5
3	0	0	13	3	1	0
12	3	0	3	10	6	5
20	3	1	15	23	8	1
18	3	0	2	11	5	5
1	0	0	4	1	0	0
9	3	0	6	0	9	1
9	7	1	18	7	1	3
14	6	0	2	2	3	7
19	2	2	10	18	6	3
14	2	1	13	14	4	2
6	2	2	6	7	5	3
14	1	1	2	16	8	9
15	6	1	7	5	6	13
0	0	0	18	0	1	0
6	0	0	21	7	7	4
13	0	0	11	9	3	0
8	1	2	9	9	7	8
14	0	0	3	20	2	1
17	3	1	11	8	0	8
1	0	0	48	3	0	0
12	1	0	2	14	4	1
4	1	1	33	5	5	5
19	0	0	14	13	2	2
8	1	1	7	3	10	4
5	0	0	21	3	2	6
10	0	0	22	9	19	6
17	1	2	26	11	1	5
11	0	1	12	12	1	1
16	0	0	5	6	0	0
13	5	0	6	3	1	0
3	5	0	4	0	2	8
15	4	0	11	8	9	6
3	0	0	5	4	0	1
6	0	0	18	3	4	0
10	0	2	4	4	6	1
8	0	0	0	5	3	0
1	2	0	7	26	0	6
9	3	0	4	0	7	2
8	0	0	6	5	5	1

9	7	0	2	12	2	1
4	0	0	2	0	0	1
2	2	1	4	2	5	1
8	0	0	9	7	1	3
3	7	0	2	6	16	6
6	1	0	3	5	12	3
11	4	0	0	12	6	0
12	3	0	0	12	9	9
5	1	0	4	11	2	2
3	0	1	4	7	1	0
6	2	0	2	4	5	0
22	0	1	6	7	5	4
5	1	0	7	13	10	2
9	1	0	7	1	0	2
7	5	0	1	2	5	3
5	0	1	12	2	0	0
1	0	0	0	6	2	2
10	1	0	4	4	13	3
0	0	0	2	2	3	0
1	0	0	15	5	2	0
9	1	1	10	3	9	9
7	0	0	3	8	0	0
2	0	0	12	2	0	0
7	0	1	6	6	2	1
9	1	0	5	7	4	1
0	0	0	0	1	1	12
0	0	0	5	2	0	0
4	0	0	11	1	2	3
3	0	1	3	7	0	1
5	0	0	1	6	0	2
5	1	0	4	9	6	4
5	6	0	0	1	1	4
4	4	1	5	8	4	3
6	0	1	4	4	6	8
6	4	0	1	4	1	1
5	0	2	6	3	3	0
1	0	1	22	1	0	0
0	0	0	0	0	0	0
0	0	0	0	0	0	0
2	0	0	8	3	1	0
9	1	0	3	9	7	8
1	0	0	9	1	10	5
3	6	1	1	1	3	6
1	1	0	7	1	0	1
0	0	0	1	0	0	0
1	0	0	7	0	1	1
0	0	0	14	2	1	4
1	0	0	18	2	0	0
5	2	0	0	2	3	0
3	1	0	2	4	3	9
6	3	2	0	1	5	1
0	0	0	3	0	0	0
5	0	0	6	3	4	2
1	0	0	4	4	0	0
1	3	0	2	0	2	0
1	0	0	3	5	0	0
3	0	0	14	0	0	1
5	0	0	1	3	0	0
3	3	0	4	2	0	1

3	24	0	0	1	4	2
4	1	0	4	2	1	0
3	0	0	4	3	1	0
8	3	0	0	5	6	1
2	1	0	3	4	1	1
4	0	1	6	6	1	0
0	1	0	5	1	0	0
0	0	0	3	0	0	0
4	1	0	7	4	1	0
3	1	0	5	2	1	0
3	2	0	0	0	0	3
3	1	1	6	1	0	0
0	0	0	3	0	0	0
0	0	0	3	1	0	0
6	0	0	3	10	5	1
2	2	0	0	5	13	2
7	0	0	4	3	0	0
4	0	0	1	4	1	0
0	1	0	6	1	1	0
5	1	0	0	4	1	1
1	0	1	6	5	1	4
3	0	0	2	5	0	0
14	0	0	4	3	0	0
0	0	0	7	1	4	1
1	1	0	0	0	4	3
3	0	0	2	0	10	1
2	0	0	0	3	22	0
6	3	0	0	2	2	2
3	1	2	0	4	1	0
9	0	0	14	2	2	1
1	0	0	2	0	1	1
1	0	0	10	3	1	0
7	0	0	0	2	3	0
0	0	0	1	0	1	0
3	0	0	2	4	1	1
0	0	0	18	0	0	0
0	3	1	1	0	0	4
0	0	0	2	1	3	1
2	0	0	9	0	0	1
2	3	1	0	0	0	1
0	1	0	2	0	0	0
2	0	0	4	1	0	0
1	0	0	3	1	3	0
0	0	0	1	0	0	0
0	0	0	3	1	0	0
4	1	0	6	2	1	0
2	0	0	6	2	3	0
2	0	0	6	0	1	0
1	0	0	5	0	0	0
4	0	0	1	3	3	1
6	0	0	0	5	6	2
4	0	0	1	7	0	0
2	1	0	1	6	1	0
5	1	0	0	2	1	1
0	0	0	2	0	0	0
0	0	0	10	0	3	0
0	0	0	2	0	0	0
1	2	0	1	3	3	1
4	0	0	1	0	1	0

1	0	0	0	1	8	0
4	0	1	3	4	5	1
1	0	0	6	2	2	3
2	1	0	0	0	16	5
0	0	0	3	0	3	1
4	0	0	2	2	2	0
10	0	0	0	4	1	0
4	0	1	13	0	0	0
1	2	0	2	2	3	0
1	1	0	1	2	0	0
6	2	0	0	2	2	1
2	0	0	2	4	0	0
1	2	0	0	3	0	0
3	0	0	1	1	6	1
1	1	0	0	0	1	0
1	1	0	0	1	4	3
3	8	0	2	0	2	2
6	1	0	1	2	3	2
4	0	0	0	0	1	0
0	1	0	10	0	0	0
8	0	0	12	1	1	0
1	2	0	2	0	2	1
0	1	0	2	4	2	1
5	1	0	1	2	1	0
4	0	0	0	5	3	0
0	0	0	4	0	0	0
10	0	0	3	1	0	0
4	0	0	2	3	0	1
0	0	0	0	1	2	0
1	1	0	0	3	0	0
11	0	0	0	0	0	0
2	1	0	1	1	1	0
2	1	0	0	2	3	1
1	1	0	1	0	1	1
0	2	0	2	2	7	2
1	1	0	0	0	0	1
8	2	0	1	2	4	0
0	1	0	1	0	3	0

0.90	0.12	0.08	0.02	0.34	0.93	0.94
8	30	31	37	20	6	4
1	1	1	1	1	1	1
36	37	38	39	40	41	42
9	10	11	12	13	14	15
C_37	C_38	C_39	C_43	C_48	D_10	D_12
CHR_NP	CHR_NP	CHR_NP	CHR_NP	CHR_NP	CHR_NP	CHR_NP
2915691	932164	148130	2018570	2072241	727386	206047
CHR_NP	CHR_NP	CHR_NP	CHR_NP	CHR_NP	CHR_NP	CHR_NP
1119107	698786	56414	382904	593526	308682	28125
107450	11085	4692	49604	152869	23073	6563
527415	18116	13820	77016	385992	63225	24309
186072	37780	6531	62996	114740	11822	2047
221867	36882	11931	143942	254736	89228	33653

22930	3941	3244	97556	27400	2763	381
94903	4076	6644	91561	104908	22052	6808
5144	336	1560	352009	7545	2512	2325
20714	1927	1860	50045	22013	4185	2552
60783	6245	7346	116265	23827	6144	1172
32180	6565	5855	75495	12756	23750	19091
36680	7874	1869	28048	35505	8613	2253
49017	1645	2208	28494	21950	4686	1683
24453	24432	1247	16962	18735	15532	6102
8257	694	1172	28299	10207	613	202
39192	13559	450	5400	12774	8711	4731
9621	146	686	21808	9339	790	192
12394	826	1168	18331	12358	5454	2635
15407	717	918	26233	13684	609	126
24919	1038	1631	17880	15631	6584	1697
20190	1251	1020	15754	16830	6083	3941
9877	1143	841	20274	7357	1802	494
11176	292	1148	13470	4425	990	270
12983	77	333	16065	13111	467	230
6261	89	832	10359	8660	1201	676
15706	1088	781	6546	6792	2657	1291
9772	5049	303	3559	5710	8014	7864
849	240	517	11574	2119	1372	413
5551	587	227	4758	4318	1315	494
10136	844	393	5438	4042	7254	2374
9819	441	586	7562	9334	1073	264
10464	3285	188	2775	7112	1333	440
4123	191	168	8470	3775	1146	644
2212	114	567	22075	2250	973	258
5155	548	208	4446	4019	1204	459
6463	2315	359	6902	5146	2115	172
5083	425	812	14383	4433	1060	646
4974	193	455	9827	3480	769	665
8506	602	173	2542	4769	6449	1347
10639	14956	57	691	7744	9122	3042
10287	568	251	3486	6633	10386	2733
8284	774	317	3949	8448	3106	999
5680	1033	99	2057	2888	3292	742
672	66	204	5936	1156	220	90
6637	148	185	10804	7260	1532	1263
6740	861	199	2797	3352	978	839
3002	208	131	4229	2009	1205	1306
3092	1921	111	879	3270	1891	1190
227	80	7	485	135	32	32
2038	43	333	5425	1169	758	464
741	308	177	1261	918	1652	870
2395	53	267	5813	3210	754	231
1818	379	160	2939	1434	3074	2195
2899	117	243	2661	2549	85	37
2193	110	149	1297	1239	1707	698
205	19	58	2116	113	79	29
1974	277	83	3015	1573	657	362
2658	128	111	2724	2705	111	65
4160	175	194	3000	2566	219	72
1502	354	43	668	2358	658	360
3150	172	202	4586	1390	1142	1023
2575	92	164	3392	2349	809	669
261	28	30	2396	150	8	15
431	30	17	309	108	25	13

724	3378	35	294	515	234	92
640	51	63	2082	552	174	42
537	33	237	4285	745	176	85
292	9	34	1456	232	116	21
1126	12	88	2265	899	1027	513
254	70	15	769	331	143	14
2383	633	135	1280	1080	831	161
1528	1881	58	498	412	791	212
207	26	68	2189	265	61	48
1318	121	95	2107	512	224	76
1376	19	32	1048	1116	95	20
361	228	126	911	618	491	265
878	127	41	517	747	835	328
414	86	23	635	416	400	388
1539	640	24	352	921	780	301
1370	103	194	2209	834	208	124
761	153	19	631	813	107	59
1591	252	41	620	834	855	493
1085	713	24	169	787	147	11
341	8	209	5325	197	33	4
464	19	16	1258	400	175	197
1293	65	60	1721	702	2554	2880
1114	522	30	329	564	1136	1039
205	12	15	1000	224	13	6
1331	49	34	507	516	65	45
746	163	17	181	448	304	145
1017	64	31	490	856	347	152
56	22	4	158	9	21	16
1035	56	15	195	633	291	130
286	14	23	500	181	24	15
104	14	14	1025	107	41	8
426	16	28	540	618	132	46
812	237	24	107	554	240	121
433	184	33	231	429	423	274
831	69	35	232	824	448	124
84	6	15	806	65	10	4
879	13	41	884	492	887	245
389	32	24	220	304	45	23
1220	189	20	273	688	510	399
374	9	24	810	465	28	20
171	24	41	414	200	31	9
101	5	13	1136	106	26	14
555	47	13	291	436	998	754
307	15	28	486	387	66	11
154	14	13	586	110	98	18
112	8	10	272	117	345	105
367	20	26	707	502	6	9
770	55	20	263	386	167	57
460	15	36	724	310	670	329
58	8	9	324	86	28	6
1363	16	40	503	524	70	109
1481	117	17	154	256	58	34
236	43	17	521	329	31	20
125	9	11	283	113	75	34
125	9	11	283	113	75	34
504	19	20	740	613	17	4
227	70	17	353	128	332	286
498	53	12	279	276	17	8
39	10	25	182	88	9	0

205	7	20	422	266	30	19
85	4	3	100	28	39	21
250	54	17	149	209	96	69
279	18	5	178	206	390	617
308	21	13	139	260	58	28
293	12	3	139	126	282	245
301	20	1	59	158	79	13
42	10	1	70	41	180	45
169	3	8	215	232	84	41
124	6	11	279	122	30	45
288	28	21	200	103	385	217
112	2	4	183	64	169	229
215	16	18	405	188	62	60
200	30	14	206	70	36	30
71	18	5	349	58	88	147
116	4	4	98	86	5	13
202	22	6	175	119	145	139
225	55	5	67	136	118	49
159	38	9	176	124	38	19
229	267	7	36	250	168	81
177	7	4	65	80	36	12
26	4	0	197	10	19	26
50	118	4	57	57	67	39
392	26	6	69	201	31	2
99	6	4	67	77	4	1
99	64	5	45	117	21	10
131	19	3	61	61	217	78
121	6	12	205	114	50	18
43	11	4	177	46	24	20
11	2	9	167	36	9	0
230	20	2	38	161	45	12
102	8	4	155	226	9	2
78	73	2	116	30	92	63
57	5	0	103	81	27	25
160	17	9	214	64	193	86
210	10	8	94	82	119	92
49	61	6	38	19	69	71
20	0	1	26	3	3	3
211	13	13	210	156	214	116
130	63	4	61	72	138	71
93	1	13	169	66	22	6
78	1	4	159	74	27	33
79	154	7	73	62	89	31
172	14	2	86	84	13	1
37	28	15	153	95	312	223
31	0	3	95	54	2	0
40	21	1	118	25	21	10
149	1	9	110	151	271	79
133	4	13	146	51	124	23
133	36	8	21	166	16	12
113	4	6	84	102	17	1
57	0	4	86	88	12	1
176	27	2	17	77	389	116
13	9	12	81	17	58	48
77	3	6	97	76	35	24
111	1	9	312	64	105	62
28	11	8	94	40	157	29
146	44	6	132	103	22	12
39	1	1	128	22	9	6

5	12	11	49	18	93	40
49	2	4	124	52	4	1
61	43	5	50	28	41	20
126	89	3	30	82	115	118
39	1	0	112	22	9	5
33	3	16	95	29	35	7
93	11	5	112	70	31	69
109	5	0	98	76	14	7
0	3	0	0	2	36	51
11	3	5	589	24	1	0
83	0	5	87	77	24	18
83	52	3	5	83	13	2
83	20	5	65	31	20	13
287	4	3	51	49	34	28
6	0	4	52	16	6	0
29	26	2	49	29	22	9
64	1	7	151	33	115	95
64	2	5	41	61	52	46
96	20	0	3	101	22	11
172	2	5	53	57	3	2
108	12	0	17	46	102	66
57	5	6	63	32	38	18
19	4	2	174	19	27	5
17	3	2	7	20	51	50
44	42	1	21	58	49	34
100	12	3	46	69	14	2
56	6	6	60	29	11	21
41	66	5	31	19	35	24
149	8	2	29	75	45	30
79	1	1	33	95	23	4
3	1	4	60	14	1	0
59	54	2	24	54	68	41
13	3	6	42	19	37	21
75	8	0	14	80	44	37
65	7	3	31	61	14	8
70	3	3	21	61	16	13
4	0	0	18	1	1	1
78	8	3	36	45	6	0
2	0	2	64	8	2	0
32	11	2	64	35	39	34
75	0	3	129	40	13	21
43	10	4	123	29	56	26
11	2	3	14	25	4	2
86	24	1	19	48	44	25
60	22	1	5	26	33	29
18	25	0	6	16	14	15
75	10	0	21	36	35	16
36	4	2	88	25	9	2
22	0	9	124	19	6	6
31	15	0	9	15	20	10
10	0	0	60	9	1	0
59	11	5	33	21	59	34
33	3	2	13	18	5	6
25	9	0	32	12	39	56
40	2	3	39	43	108	118
47	2	4	148	14	0	2
24	0	1	37	34	14	6
32	7	3	12	31	7	5
9	1	9	18	6	21	8

57	0	2	120	23	31	26
12	1	2	65	13	2	1
16	0	2	52	15	3	0
16	0	2	52	15	3	0
11	8	2	14	21	25	7
49	7	0	10	32	35	19
19	1	0	22	14	22	10
6	0	2	12	14	9	4
42	1	0	13	30	1	0
35	2	1	26	44	16	15
33	2	6	64	11	68	22
20	13	2	14	13	5	1
48	4	1	8	28	46	160
24	1	2	16	52	5	2
27	1	0	3	70	13	0
94	12	5	39	24	0	3
26	3	2	59	21	9	2
1	2	0	15	0	1	4
22	3	0	41	15	20	8
24	1	0	30	17	3	0
50	8	0	8	32	32	13
14	47	0	10	11	12	12
22	1	0	28	17	3	0
15	4	0	32	22	23	6
20	4	0	7	12	11	7
18	1	1	34	11	7	4
23	25	0	23	10	19	13
1	1	0	13	2	4	1
71	8	0	3	34	43	78
22	27	0	10	25	18	9
8	20	0	19	10	15	21
12	4	2	13	9	4	2
17	0	2	23	14	2	1
1	0	0	10	1	1	1
33	1	2	8	21	10	1
22	0	2	56	29	2	1
32	64	3	43	17	0	23
21	15	0	9	12	24	31
51	3	1	31	18	0	2
26	8	1	12	23	34	9
12	1	0	13	7	38	17
30	1	0	18	17	4	0
7	4	5	7	12	11	8
33	5	2	15	24	20	12
26	4	1	11	5	10	11
31	7	0	5	18	14	3
21	0	1	47	27	1	1
8	3	3	30	15	2	1
2	0	1	23	1	3	0
9	0	2	10	5	0	0
91	0	1	13	21	0	1
14	5	3	1	12	30	7
19	0	4	71	7	14	4
22	4	1	15	41	8	6
6	3	0	5	2	15	5
7	1	0	32	11	0	1
9	16	0	4	10	8	4
27	1	1	18	14	36	21
24	2	3	12	14	39	27

7	0	3	13	7	0	2
6	10	0	6	8	10	6
35	3	0	4	14	7	2
28	3	0	6	18	27	9
10	40	0	8	2	2	15
20	2	0	17	12	2	0
36	7	0	8	20	9	5
36	1	0	16	23	19	10
26	9	2	22	6	27	37
28	0	0	16	16	19	32
20	2	0	15	22	2	4
6	7	1	10	10	1	0
2	2	0	14	10	0	1
0	0	0	16	5	0	0
3	1	2	25	4	1	1
13	1	0	11	11	0	0
9	9	2	19	7	21	10
22	0	1	17	10	3	1
13	0	0	7	2	13	27
9	3	0	5	13	9	1
34	1	0	4	18	6	3
10	0	1	18	8	5	4
19	1	0	6	20	3	8
2	1	2	1	5	8	2
14	4	0	3	11	4	5
4	1	0	16	8	0	3
7	8	4	42	16	0	5
12	10	1	17	7	9	7
1	0	3	13	1	5	2
9	8	1	16	8	26	11
33	3	2	5	18	5	1
5	5	2	1	8	12	12
3	0	0	27	0	0	2
15	8	0	9	12	29	5
3	0	0	13	3	1	0
12	3	0	3	10	6	5
20	3	1	15	23	8	1
18	3	0	2	11	5	5
1	0	0	4	1	0	0
9	3	0	6	0	9	1
9	7	1	18	7	1	3
14	6	0	2	2	3	7
19	2	2	10	18	6	3
14	2	1	13	14	4	2
6	2	2	6	7	5	3
14	1	1	2	16	8	9
15	6	1	7	5	6	13
0	0	0	18	0	1	0
6	0	0	21	7	7	4
13	0	0	11	9	3	0
8	1	2	9	9	7	8
14	0	0	3	20	2	1
17	3	1	11	8	0	8
1	0	0	48	3	0	0
12	1	0	2	14	4	1
4	1	1	33	5	5	5
19	0	0	14	13	2	2
8	1	1	7	3	10	4
5	0	0	21	3	2	6

10	0	0	22	9	19	6
17	1	2	26	11	1	5
11	0	1	12	12	1	1
16	0	0	5	6	0	0
13	5	0	6	3	1	0
3	5	0	4	0	2	8
15	4	0	11	8	9	6
3	0	0	5	4	0	1
6	0	0	18	3	4	0
10	0	2	4	4	6	1
8	0	0	0	5	3	0
1	2	0	7	26	0	6
9	3	0	4	0	7	2
8	0	0	6	5	5	1
9	7	0	2	12	2	1
4	0	0	2	0	0	1
2	2	1	4	2	5	1
8	0	0	9	7	1	3
3	7	0	2	6	16	6
6	1	0	3	5	12	3
11	4	0	0	12	6	0
12	3	0	0	12	9	9
5	1	0	4	11	2	2
3	0	1	4	7	1	0
6	2	0	2	4	5	0
22	0	1	6	7	5	4
5	1	0	7	13	10	2
9	1	0	7	1	0	2
7	5	0	1	2	5	3
5	0	1	12	2	0	0
1	0	0	0	6	2	2
10	1	0	4	4	13	3
0	0	0	2	2	3	0
1	0	0	15	5	2	0
9	1	1	10	3	9	9
7	0	0	3	8	0	0
2	0	0	12	2	0	0
7	0	1	6	6	2	1
9	1	0	5	7	4	1
0	0	0	0	1	1	12
0	0	0	5	2	0	0
4	0	0	11	1	2	3
3	0	1	3	7	0	1
5	0	0	1	6	0	2
5	1	0	4	9	6	4
5	6	0	0	1	1	4
4	4	1	5	8	4	3
6	0	1	4	4	6	8
6	4	0	1	4	1	1
5	0	2	6	3	3	0
1	0	1	22	1	0	0
0	0	0	0	0	0	0
0	0	0	0	0	0	0
2	0	0	8	3	1	0
9	1	0	3	9	7	8
1	0	0	9	1	10	5
3	6	1	1	1	3	6
1	1	0	7	1	0	1
0	0	0	1	0	0	0

1	0	0	7	0	1	1
0	0	0	14	2	1	4
1	0	0	18	2	0	0
5	2	0	0	2	3	0
3	1	0	2	4	3	9
6	3	2	0	1	5	1
0	0	0	3	0	0	0
5	0	0	6	3	4	2
1	0	0	4	4	0	0
1	3	0	2	0	2	0
1	0	0	3	5	0	0
3	0	0	14	0	0	1
5	0	0	1	3	0	0
3	3	0	4	2	0	1
3	24	0	0	1	4	2
4	1	0	4	2	1	0
3	0	0	4	3	1	0
8	3	0	0	5	6	1
2	1	0	3	4	1	1
4	0	1	6	6	1	0
0	1	0	5	1	0	0
0	0	0	3	0	0	0
4	1	0	7	4	1	0
3	1	0	5	2	1	0
3	2	0	0	0	0	3
3	1	1	6	1	0	0
0	0	0	3	0	0	0
0	0	0	3	1	0	0
6	0	0	3	10	5	1
2	2	0	0	5	13	2
7	0	0	4	3	0	0
4	0	0	1	4	1	0
0	1	0	6	1	1	0
5	1	0	0	4	1	1
1	0	1	6	5	1	4
3	0	0	2	5	0	0
14	0	0	4	3	0	0
0	0	0	7	1	4	1
1	1	0	0	0	4	3
3	0	0	2	0	10	1
2	0	0	0	3	22	0
6	3	0	0	2	2	2
3	1	2	0	4	1	0
9	0	0	14	2	2	1
1	0	0	2	0	1	1
1	0	0	10	3	1	0
7	0	0	0	2	3	0
0	0	0	1	0	1	0
3	0	0	2	4	1	1
0	0	0	18	0	0	0
0	3	1	1	0	0	4
0	0	0	2	1	3	1
2	0	0	9	0	0	1
2	3	1	0	0	0	1
0	1	0	2	0	0	0
2	0	0	4	1	0	0
1	0	0	3	1	3	0
0	0	0	1	0	0	0
0	0	0	3	1	0	0

4	1	0	6	2	1	0
2	0	0	6	2	3	0
2	0	0	6	0	1	0
1	0	0	5	0	0	0
4	0	0	1	3	3	1
6	0	0	0	5	6	2
4	0	0	1	7	0	0
2	1	0	1	6	1	0
5	1	0	0	2	1	1
0	0	0	2	0	0	0
0	0	0	10	0	3	0
0	0	0	2	0	0	0
1	2	0	1	3	3	1
4	0	0	1	0	1	0
1	0	0	0	1	8	0
4	0	1	3	4	5	1
1	0	0	6	2	2	3
2	1	0	0	0	16	5
0	0	0	3	0	3	1
4	0	0	2	2	2	0
10	0	0	0	4	1	0
4	0	1	13	0	0	0
1	2	0	2	2	3	0
1	1	0	1	2	0	0
6	2	0	0	2	2	1
2	0	0	2	4	0	0
1	2	0	0	3	0	0
3	0	0	1	1	6	1
1	1	0	0	0	1	0
1	1	0	0	1	4	3
3	8	0	2	0	2	2
6	1	0	1	2	3	2
4	0	0	0	0	1	0
0	1	0	10	0	0	0
8	0	0	12	1	1	0
1	2	0	2	0	2	1
0	1	0	2	4	2	1
5	1	0	1	2	1	0
4	0	0	0	5	3	0
0	0	0	4	0	0	0
10	0	0	3	1	0	0
4	0	0	2	3	0	1
0	0	0	0	1	2	0
1	1	0	0	3	0	0
11	0	0	0	0	0	0
2	1	0	1	1	1	0
2	1	0	0	2	3	1
1	1	0	1	0	1	1
0	2	0	2	2	7	2
1	1	0	0	0	0	1
8	2	0	1	2	4	0
0	1	0	1	0	3	0
90700	29578	4588	59955	64129	21339	5490
219894	126803	10372	91232	130793	57545	8799
369	455	468	363	385	413	436
CHR_NP	CHR_NP	CHR_NP	CHR_NP	CHR_NP	CHR_NP	CHR_NP
CHR_NP	CHR_NP	CHR_NP	CHR_NP	CHR_NP	CHR_NP	CHR_NP

36	37	38	39	40	41	42
9	10	11	12	13	14	15
CHR_NP	CHR_NP	CHR_NP	CHR_NP	CHR_NP	CHR_NP	CHR_NP
C_37	C_38	C_39	C_43	C_48	D_10	D_12
2915691	932164	148130	2018570	2072241	727386	206047
CHR_NP	CHR_NP	CHR_NP	CHR_NP	CHR_NP	CHR_NP	CHR_NP
12.34	23.63	12.30	6.39	9.26	14.47	5.12
1.18	0.37	1.02	0.83	2.38	1.08	1.20
5.81	0.61	3.01	1.28	6.02	2.96	4.43
2.05	1.28	1.42	1.05	1.79	0.55	0.37
2.45	1.25	2.60	2.40	3.97	4.18	6.13
0.25	0.13	0.71	1.63	0.43	0.13	0.07
1.05	0.14	1.45	1.53	1.64	1.03	1.24
0.06	0.01	0.34	5.87	0.12	0.12	0.42
0.23	0.07	0.41	0.83	0.34	0.20	0.46
0.67	0.21	1.60	1.94	0.37	0.29	0.21
0.35	0.22	1.28	1.26	0.20	1.11	3.48
0.40	0.27	0.41	0.47	0.55	0.40	0.41
0.54	0.06	0.48	0.48	0.34	0.22	0.31
0.27	0.83	0.27	0.28	0.29	0.73	1.11
0.09	0.02	0.26	0.47	0.16	0.03	0.04
0.43	0.46	0.10	0.09	0.20	0.41	0.86
0.11	0.00	0.15	0.36	0.15	0.04	0.03
0.14	0.03	0.25	0.31	0.19	0.26	0.48
0.17	0.02	0.20	0.44	0.21	0.03	0.02
0.27	0.04	0.36	0.30	0.24	0.31	0.31
0.22	0.04	0.22	0.26	0.26	0.29	0.72
0.11	0.04	0.18	0.34	0.11	0.08	0.09
0.12	0.01	0.25	0.22	0.07	0.05	0.05
0.14	0.00	0.07	0.27	0.20	0.02	0.04
0.07	0.00	0.18	0.17	0.14	0.06	0.12
0.17	0.04	0.17	0.11	0.11	0.12	0.24
0.11	0.17	0.07	0.06	0.09	0.38	1.43
0.01	0.01	0.11	0.19	0.03	0.06	0.08
0.06	0.02	0.05	0.08	0.07	0.06	0.09
0.11	0.03	0.09	0.09	0.06	0.34	0.43
0.11	0.01	0.13	0.13	0.15	0.05	0.05
0.12	0.11	0.04	0.05	0.11	0.06	0.08
0.05	0.01	0.04	0.14	0.06	0.05	0.12
0.02	0.00	0.12	0.37	0.04	0.05	0.05
0.06	0.02	0.05	0.07	0.06	0.06	0.08
0.07	0.08	0.08	0.12	0.08	0.10	0.03
0.06	0.01	0.18	0.24	0.07	0.05	0.12
0.05	0.01	0.10	0.16	0.05	0.04	0.12
0.09	0.02	0.04	0.04	0.07	0.30	0.25
0.12	0.51	0.01	0.01	0.12	0.43	0.55
0.11	0.02	0.05	0.06	0.10	0.49	0.50
0.09	0.03	0.07	0.07	0.13	0.15	0.18
0.06	0.03	0.02	0.03	0.05	0.15	0.14
0.01	0.00	0.04	0.10	0.02	0.01	0.02
0.07	0.01	0.04	0.18	0.11	0.07	0.23
0.07	0.03	0.04	0.05	0.05	0.05	0.15
0.03	0.01	0.03	0.07	0.03	0.06	0.24
0.03	0.06	0.02	0.01	0.05	0.09	0.22
0.00	0.00	0.00	0.01	0.00	0.00	0.01
0.02	0.00	0.07	0.09	0.02	0.04	0.08
0.01	0.01	0.04	0.02	0.01	0.08	0.16
0.03	0.00	0.06	0.10	0.05	0.04	0.04

0.02	0.01	0.03	0.05	0.02	0.14	0.40
0.03	0.00	0.05	0.04	0.04	0.00	0.01
0.02	0.00	0.03	0.02	0.02	0.08	0.13
0.00	0.00	0.01	0.04	0.00	0.00	0.01
0.02	0.01	0.02	0.05	0.02	0.03	0.07
0.03	0.00	0.02	0.05	0.04	0.01	0.01
0.05	0.01	0.04	0.05	0.04	0.01	0.01
0.02	0.01	0.01	0.01	0.04	0.03	0.07
0.03	0.01	0.04	0.08	0.02	0.05	0.19
0.03	0.00	0.04	0.06	0.04	0.04	0.12
0.00	0.00	0.01	0.04	0.00	0.00	0.00
0.00	0.00	0.00	0.01	0.00	0.00	0.00
0.01	0.11	0.01	0.00	0.01	0.01	0.02
0.01	0.00	0.01	0.03	0.01	0.01	0.01
0.01	0.00	0.05	0.07	0.01	0.01	0.02
0.00	0.00	0.01	0.02	0.00	0.01	0.00
0.01	0.00	0.02	0.04	0.01	0.05	0.09
0.00	0.00	0.00	0.01	0.01	0.01	0.00
0.03	0.02	0.03	0.02	0.02	0.04	0.03
0.02	0.06	0.01	0.01	0.01	0.04	0.04
0.00	0.00	0.01	0.04	0.00	0.00	0.01
0.01	0.00	0.02	0.04	0.01	0.01	0.01
0.02	0.00	0.01	0.02	0.02	0.00	0.00
0.00	0.01	0.03	0.02	0.01	0.02	0.05
0.01	0.00	0.01	0.01	0.01	0.04	0.06
0.00	0.00	0.01	0.01	0.01	0.02	0.07
0.02	0.02	0.01	0.01	0.01	0.04	0.05
0.02	0.00	0.04	0.04	0.01	0.01	0.02
0.01	0.01	0.00	0.01	0.01	0.01	0.01
0.02	0.01	0.01	0.01	0.01	0.04	0.09
0.01	0.02	0.01	0.00	0.01	0.01	0.00
0.00	0.00	0.05	0.09	0.00	0.00	0.00
0.01	0.00	0.00	0.02	0.01	0.01	0.04
0.01	0.00	0.01	0.03	0.01	0.12	0.52
0.01	0.02	0.01	0.01	0.01	0.05	0.19
0.00	0.00	0.00	0.02	0.00	0.00	0.00
0.01	0.00	0.01	0.01	0.01	0.00	0.01
0.01	0.01	0.00	0.00	0.01	0.01	0.03
0.01	0.00	0.01	0.01	0.01	0.02	0.03
0.00	0.00	0.00	0.00	0.00	0.00	0.00
0.01	0.00	0.00	0.00	0.01	0.01	0.02
0.00	0.00	0.01	0.01	0.00	0.00	0.00
0.00	0.00	0.00	0.02	0.00	0.00	0.00
0.00	0.00	0.01	0.01	0.01	0.01	0.01
0.01	0.01	0.01	0.00	0.01	0.01	0.02
0.00	0.01	0.01	0.00	0.01	0.02	0.05
0.01	0.00	0.01	0.00	0.01	0.02	0.02
0.00	0.00	0.00	0.01	0.00	0.00	0.00
0.01	0.00	0.01	0.01	0.01	0.04	0.04
0.00	0.00	0.01	0.00	0.00	0.00	0.00
0.01	0.01	0.00	0.00	0.01	0.02	0.07
0.00	0.00	0.01	0.01	0.01	0.00	0.00
0.00	0.00	0.01	0.01	0.00	0.00	0.00
0.00	0.00	0.00	0.02	0.00	0.00	0.00
0.01	0.00	0.00	0.00	0.01	0.05	0.14
0.00	0.00	0.01	0.01	0.01	0.00	0.00
0.00	0.00	0.00	0.01	0.00	0.00	0.00
0.00	0.00	0.00	0.00	0.00	0.02	0.02
0.00	0.00	0.01	0.01	0.01	0.00	0.00

0.01	0.00	0.00	0.00	0.01	0.01	0.01
0.01	0.00	0.01	0.01	0.00	0.03	0.06
0.00	0.00	0.00	0.01	0.00	0.00	0.00
0.02	0.00	0.01	0.01	0.01	0.00	0.02
0.02	0.00	0.00	0.00	0.00	0.00	0.01
0.00	0.00	0.00	0.01	0.01	0.00	0.00
0.00	0.00	0.00	0.00	0.00	0.00	0.01
0.00	0.00	0.00	0.00	0.00	0.00	0.01
0.01	0.00	0.00	0.01	0.01	0.00	0.00
0.00	0.00	0.00	0.01	0.00	0.02	0.05
0.01	0.00	0.00	0.00	0.00	0.00	0.00
0.00	0.00	0.01	0.00	0.00	0.00	0.00
0.00	0.00	0.00	0.01	0.00	0.00	0.00
0.00	0.00	0.00	0.00	0.00	0.00	0.00
0.00	0.00	0.00	0.00	0.00	0.00	0.01
0.00	0.00	0.00	0.00	0.00	0.02	0.11
0.00	0.00	0.00	0.00	0.00	0.00	0.01
0.00	0.00	0.00	0.00	0.00	0.01	0.04
0.00	0.00	0.00	0.00	0.00	0.00	0.00
0.00	0.00	0.00	0.00	0.00	0.01	0.01
0.00	0.00	0.00	0.00	0.00	0.00	0.01
0.00	0.00	0.00	0.00	0.00	0.00	0.01
0.00	0.00	0.00	0.00	0.00	0.02	0.04
0.00	0.00	0.00	0.00	0.00	0.01	0.04
0.00	0.00	0.00	0.01	0.00	0.00	0.01
0.00	0.00	0.00	0.00	0.00	0.00	0.01
0.00	0.00	0.00	0.01	0.00	0.00	0.03
0.00	0.00	0.00	0.00	0.00	0.00	0.00
0.00	0.00	0.00	0.00	0.00	0.01	0.03
0.00	0.00	0.00	0.00	0.00	0.01	0.01
0.00	0.00	0.00	0.00	0.00	0.00	0.00
0.00	0.01	0.00	0.00	0.00	0.01	0.01
0.00	0.00	0.00	0.00	0.00	0.00	0.00
0.00	0.00	0.00	0.00	0.00	0.00	0.00
0.00	0.00	0.00	0.00	0.00	0.00	0.01
0.00	0.00	0.00	0.00	0.00	0.00	0.00
0.00	0.00	0.00	0.00	0.00	0.00	0.00
0.00	0.00	0.00	0.00	0.00	0.00	0.00
0.00	0.00	0.00	0.00	0.00	0.01	0.01
0.00	0.00	0.00	0.00	0.00	0.00	0.00
0.00	0.00	0.00	0.00	0.00	0.00	0.00
0.00	0.00	0.00	0.00	0.00	0.00	0.00
0.00	0.00	0.00	0.00	0.00	0.00	0.00
0.00	0.00	0.00	0.00	0.00	0.00	0.00
0.00	0.00	0.00	0.00	0.00	0.00	0.01
0.00	0.00	0.00	0.00	0.00	0.00	0.00
0.00	0.00	0.00	0.00	0.00	0.01	0.02
0.00	0.00	0.00	0.00	0.00	0.01	0.02
0.00	0.00	0.00	0.00	0.00	0.00	0.01
0.00	0.00	0.00	0.00	0.00	0.00	0.00
0.00	0.00	0.00	0.00	0.00	0.01	0.02
0.00	0.00	0.00	0.00	0.00	0.01	0.01
0.00	0.00	0.00	0.00	0.00	0.00	0.00
0.00	0.00	0.00	0.00	0.00	0.00	0.01
0.00	0.01	0.00	0.00	0.00	0.00	0.01
0.00	0.00	0.00	0.00	0.00	0.00	0.00
0.00	0.00	0.00	0.00	0.00	0.01	0.04
0.00	0.00	0.00	0.00	0.00	0.00	0.00
0.00	0.00	0.00	0.00	0.00	0.00	0.00

0.00	0.00	0.00	0.00	0.00	0.01	0.01
0.00	0.00	0.00	0.00	0.00	0.01	0.00
0.00	0.00	0.00	0.00	0.00	0.00	0.00
0.00	0.00	0.00	0.00	0.00	0.00	0.00
0.00	0.00	0.00	0.00	0.00	0.00	0.00
0.00	0.00	0.00	0.00	0.00	0.02	0.02
0.00	0.00	0.00	0.00	0.00	0.00	0.01
0.00	0.00	0.00	0.00	0.00	0.00	0.00
0.00	0.00	0.00	0.01	0.00	0.00	0.01
0.00	0.00	0.00	0.00	0.00	0.01	0.01
0.00	0.00	0.00	0.00	0.00	0.00	0.00
0.00	0.00	0.00	0.00	0.00	0.00	0.00
0.00	0.00	0.00	0.00	0.00	0.00	0.01
0.00	0.00	0.00	0.00	0.00	0.00	0.00
0.00	0.00	0.00	0.00	0.00	0.00	0.00
0.00	0.00	0.00	0.00	0.00	0.01	0.02
0.00	0.00	0.00	0.00	0.00	0.00	0.00
0.00	0.00	0.00	0.00	0.00	0.00	0.00
0.00	0.00	0.00	0.00	0.00	0.00	0.01
0.00	0.00	0.00	0.00	0.00	0.00	0.00
0.00	0.00	0.00	0.00	0.00	0.00	0.01
0.00	0.00	0.00	0.01	0.00	0.00	0.00
0.00	0.00	0.00	0.00	0.00	0.00	0.00
0.00	0.00	0.00	0.00	0.00	0.00	0.00
0.00	0.00	0.00	0.00	0.00	0.00	0.00
0.00	0.00	0.00	0.00	0.00	0.00	0.01
0.00	0.00	0.00	0.00	0.00	0.00	0.00
0.00	0.00	0.00	0.00	0.00	0.00	0.00
0.00	0.00	0.00	0.00	0.00	0.01	0.02
0.00	0.00	0.00	0.00	0.00	0.00	0.01
0.00	0.00	0.00	0.00	0.00	0.00	0.00
0.00	0.00	0.00	0.00	0.00	0.00	0.00
0.00	0.00	0.00	0.00	0.00	0.00	0.01
0.00	0.00	0.00	0.00	0.00	0.00	0.00
0.00	0.00	0.00	0.00	0.00	0.00	0.00
0.00	0.00	0.00	0.00	0.00	0.00	0.01
0.00	0.00	0.00	0.00	0.00	0.00	0.01
0.00	0.00	0.00	0.00	0.00	0.00	0.00
0.00	0.00	0.00	0.00	0.00	0.00	0.00
0.00	0.00	0.00	0.00	0.00	0.00	0.00
0.00	0.00	0.00	0.00	0.00	0.00	0.01
0.00	0.00	0.00	0.00	0.00	0.00	0.00
0.00	0.00	0.00	0.00	0.00	0.00	0.00
0.00	0.00	0.00	0.00	0.00	0.00	0.01
0.00	0.00	0.00	0.00	0.00	0.00	0.00
0.00	0.00	0.00	0.00	0.00	0.00	0.01
0.00	0.00	0.00	0.00	0.00	0.00	0.00
0.00	0.00	0.00	0.00	0.00	0.00	0.00
0.00	0.00	0.00	0.00	0.00	0.00	0.00
0.00	0.00	0.00	0.00	0.00	0.00	0.00
0.00	0.00	0.00	0.00	0.00	0.00	0.00
0.00	0.00	0.00	0.00	0.00	0.00	0.01
0.00	0.00	0.00	0.00	0.00	0.00	0.00
0.00	0.00	0.00	0.00	0.00	0.00	0.00
0.00	0.00	0.00	0.00	0.00	0.00	0.00
0.00	0.00	0.00	0.00	0.00	0.00	0.00
0.00	0.00	0.00	0.00	0.00	0.00	0.01
0.00	0.00	0.00	0.00	0.00	0.00	0.00
0.00	0.00	0.00	0.00	0.00	0.00	0.00

0.00	0.00	0.00	0.00	0.00	0.00	0.00
0.00	0.00	0.00	0.00	0.00	0.00	0.00
0.00	0.00	0.00	0.00	0.00	0.00	0.00
0.00	0.00	0.00	0.00	0.00	0.00	0.00
0.00	0.00	0.00	0.00	0.00	0.00	0.01
0.00	0.00	0.00	0.00	0.00	0.00	0.00
0.00	0.00	0.00	0.00	0.00	0.00	0.01
0.00	0.00	0.00	0.00	0.00	0.01	0.02
0.00	0.00	0.00	0.00	0.00	0.00	0.00
0.00	0.00	0.00	0.00	0.00	0.00	0.00
0.00	0.00	0.00	0.00	0.00	0.00	0.00
0.00	0.00	0.00	0.00	0.00	0.00	0.00
0.00	0.00	0.00	0.00	0.00	0.00	0.00
0.00	0.00	0.00	0.00	0.00	0.00	0.00
0.00	0.00	0.00	0.00	0.00	0.00	0.00
0.00	0.00	0.00	0.00	0.00	0.00	0.00
0.00	0.00	0.00	0.00	0.00	0.00	0.00
0.00	0.00	0.00	0.00	0.00	0.00	0.00
0.00	0.00	0.00	0.00	0.00	0.00	0.00
0.00	0.00	0.00	0.00	0.00	0.00	0.00
0.00	0.00	0.00	0.00	0.00	0.00	0.00
0.00	0.00	0.00	0.00	0.00	0.00	0.00
0.00	0.00	0.00	0.00	0.00	0.00	0.00
0.00	0.00	0.00	0.00	0.00	0.00	0.00
0.00	0.00	0.00	0.00	0.00	0.00	0.03
0.00	0.00	0.00	0.00	0.00	0.00	0.00
0.00	0.00	0.00	0.00	0.00	0.00	0.00
0.00	0.00	0.00	0.00	0.00	0.00	0.00
0.00	0.00	0.00	0.00	0.00	0.00	0.00
0.00	0.00	0.00	0.00	0.00	0.00	0.00

36	37	38	39	40	41	42
9	10	11	12	13	14	15
CHR_NP	CHR_NP	CHR_NP	CHR_NP	CHR_NP	CHR_NP	CHR_NP
C_37	C_38	C_39	C_43	C_48	D_10	D_12
2915691	932164	148130	2018570	2072241	727386	206047
C_37	C_38	C_39	C_43	C_48	D_10	D_12
0.16	2.69	0.15	-1.17	-0.53	0.64	-1.46
-0.61	-0.85	-0.66	-0.72	-0.26	-0.64	-0.61
2.79	-1.13	0.68	-0.62	2.94	0.64	1.75
-0.39	-1.16	-1.01	-1.38	-0.65	-1.87	-2.05
0.31	-1.01	0.48	0.26	2.00	2.23	4.38
-0.85	-0.96	-0.44	0.39	-0.69	-0.96	-1.02
-0.19	-1.64	0.45	0.57	0.75	-0.21	0.12
-0.52	-0.60	0.00	10.00	-0.41	-0.41	0.15

-0.72	-1.04	-0.37	0.49	-0.49	-0.78	-0.25
-0.23	-1.41	2.16	3.03	-1.00	-1.21	-1.40
-0.44	-0.94	3.02	2.96	-1.02	2.41	11.30
-0.78	-2.02	-0.75	-0.21	0.55	-0.79	-0.73
1.05	-1.74	0.71	0.68	-0.09	-0.79	-0.29
-1.14	2.07	-1.12	-1.06	-1.01	1.50	3.71
-0.59	-0.90	0.17	1.16	-0.28	-0.88	-0.84
0.59	0.71	-0.86	-0.90	-0.42	0.49	2.47
-0.32	-0.96	-0.05	1.30	-0.08	-0.76	-0.77
-0.77	-1.84	0.40	0.90	-0.21	0.41	2.61
-0.10	-1.40	0.17	2.29	0.28	-1.37	-1.42
0.44	-1.95	1.25	0.68	0.13	0.78	0.78
1.05	-1.85	1.05	1.70	1.69	2.06	9.02
-0.65	-1.72	0.49	2.86	-0.56	-1.02	-0.94
0.05	-1.35	1.63	1.31	-0.62	-0.90	-0.86
0.23	-1.10	-0.44	1.40	0.80	-0.92	-0.73
-0.36	-0.75	0.30	0.25	0.03	-0.44	-0.04
1.10	-1.47	1.04	-0.11	-0.17	0.18	2.26
-0.49	0.52	-1.15	-1.26	-0.78	3.78	20.61
-0.99	-1.00	0.00	0.77	-0.76	-0.46	-0.36
-0.90	-1.70	-1.12	-0.54	-0.78	-0.89	-0.34
0.23	-1.72	-0.38	-0.27	-0.91	5.56	7.72
0.41	-1.76	0.87	0.83	1.28	-0.94	-0.99
-0.13	-0.21	-1.57	-1.47	-0.21	-1.15	-0.81
-0.21	-0.80	-0.35	1.23	-0.01	-0.09	0.87
-0.27	-0.38	0.28	1.61	-0.21	-0.15	-0.14
-0.84	-1.70	-1.10	-0.46	-0.71	-0.85	-0.24
-0.44	-0.40	-0.40	-0.17	-0.38	-0.27	-0.68
-0.68	-1.17	0.75	1.50	-0.52	-0.75	0.05
-0.39	-0.88	0.07	0.73	-0.39	-0.58	0.29
-0.07	-0.95	-0.74	-0.68	-0.30	2.43	1.75
0.14	3.28	-0.70	-0.71	0.17	2.65	3.67
0.77	-1.16	-0.43	-0.36	0.56	8.40	8.63
0.68	-1.28	0.01	-0.08	1.90	2.31	3.41
-0.07	-1.19	-1.73	-1.21	-0.78	3.64	2.87
-0.75	-0.87	0.17	1.53	-0.48	-0.67	-0.52
0.23	-1.22	-0.47	2.50	1.08	0.20	3.57
2.02	-1.26	-0.22	0.02	0.42	-0.04	7.71
-0.31	-1.36	-0.49	1.20	-0.38	0.63	7.95
-0.52	0.51	-0.84	-1.16	0.05	1.30	5.55
-0.31	-0.31	-0.35	-0.12	-0.33	-0.35	-0.20
-0.18	-0.66	0.97	1.38	-0.27	0.12	1.24
-1.47	-1.35	0.17	-0.77	-1.14	2.27	6.64
-0.23	-0.95	0.70	1.83	0.46	0.03	0.23
-1.21	-1.77	-0.06	1.03	-1.03	8.39	28.19
0.15	-1.32	1.26	0.81	0.56	-1.32	-1.18
-0.04	-1.18	0.41	-0.19	-0.31	3.05	5.66

-0.29	-0.36	0.13	1.06	-0.31	-0.23	-0.17
-0.51	-1.51	-0.80	1.79	-0.28	0.22	3.05
0.44	-1.63	0.01	1.78	1.51	-1.56	-1.01
1.14	-1.83	0.87	1.45	0.70	-1.51	-1.30
-0.64	-0.76	-0.83	-0.78	-0.11	-0.27	0.65
0.82	-1.51	1.56	4.18	-0.23	2.33	13.01
0.53	-1.57	1.14	2.86	1.21	1.32	8.27
-0.50	-0.65	-0.22	2.40	-0.54	-0.70	-0.51
-0.08	-0.25	-0.13	-0.06	-0.22	-0.24	-0.19
-1.24	4.89	-1.26	-1.41	-1.23	-1.06	-0.73
-0.91	-1.42	-0.27	1.75	-0.76	-0.81	-0.85
-0.45	-0.81	2.90	4.35	-0.04	-0.28	0.25
-0.71	-1.00	-0.30	1.38	-0.67	-0.49	-0.65
-0.04	-1.41	0.73	2.86	0.14	4.04	9.21
-0.39	-0.40	-0.39	-0.26	-0.36	-0.34	-0.40
0.33	-0.01	0.55	-0.02	-0.33	1.22	0.55
-0.35	4.26	-0.77	-1.19	-1.38	1.65	1.80
-0.73	-0.86	0.44	2.47	-0.56	-0.68	-0.12
0.09	-1.29	0.90	2.80	-0.77	-0.44	0.00
0.51	-1.29	-0.50	0.80	0.79	-0.82	-0.92
-1.09	-0.78	0.85	-0.17	-0.62	0.48	2.56
-0.58	-1.24	-0.67	-0.71	-0.34	2.99	5.49
-0.44	-0.48	-0.43	-0.28	-0.39	-0.07	1.30
-0.06	0.37	-1.13	-1.07	-0.29	1.74	3.41
0.03	-1.06	2.57	2.06	-0.17	-0.47	0.73
-0.52	-1.15	-1.35	-0.10	0.33	-1.18	-0.05
0.75	-0.54	-0.48	-0.28	0.10	3.97	11.09
-0.42	0.70	-1.04	-1.27	-0.39	-0.89	-1.34
-0.34	-0.50	1.61	3.62	-0.37	-0.44	-0.48
-0.47	-1.23	-0.75	2.20	-0.28	0.05	4.71
0.97	-1.16	0.76	3.53	0.39	19.65	91.37
-0.18	0.89	-1.33	-1.54	-0.88	8.00	35.18
-0.64	-0.94	-0.47	1.74	-0.43	-0.91	-0.83
1.67	-1.78	-0.25	0.02	-0.08	-1.41	-0.04
-0.45	-0.79	-1.01	-1.09	-0.60	0.30	1.80
0.85	-1.48	-0.30	0.06	1.39	2.14	5.08
-0.20	-0.18	-0.15	0.18	-0.29	-0.13	0.23
1.27	-1.26	-0.90	-0.90	0.86	1.86	4.53
-0.53	-1.07	-0.15	0.52	-0.59	-0.94	-0.61
-0.48	-0.61	-0.12	2.56	-0.38	-0.33	-0.42
-0.26	-1.08	0.02	0.60	0.73	0.04	0.48
0.25	0.03	-0.64	-1.46	0.18	0.80	3.37
-0.82	-0.27	0.10	-1.17	-0.09	4.93	16.43
0.43	-1.20	0.07	-0.83	1.32	3.26	3.64
-0.60	-0.77	-0.06	2.27	-0.58	-0.71	-0.65
0.67	-0.94	0.54	1.55	0.32	6.21	6.75
-0.28	-1.58	0.10	-0.53	-0.10	-1.16	-0.32
1.26	-0.25	-0.68	-0.64	0.68	3.48	13.87

-0.35	-0.67	-0.26	0.43	-0.09	-0.59	-0.39
-0.90	-1.26	1.43	0.76	-0.49	-1.05	-0.98
-0.66	-0.93	-0.16	4.48	-0.50	-0.63	-0.24
0.21	-1.15	-0.78	-0.17	0.42	12.46	39.75
-0.48	-0.90	-0.08	0.21	-0.09	-0.52	-0.68
-0.65	-1.04	-0.29	1.91	-0.65	0.26	-0.15
-0.87	-1.24	-0.51	0.38	-0.65	4.78	5.90
-0.07	-1.26	0.50	2.67	1.27	-1.40	-0.92
1.91	-1.43	-0.17	-0.16	0.66	1.57	2.86
0.31	-1.19	1.23	2.63	0.24	9.01	18.44
-1.07	-1.22	-0.54	0.85	-0.79	-0.80	-0.89
4.13	-1.28	1.78	1.65	1.57	-0.25	5.93
4.97	-0.38	-0.49	-0.98	-0.36	-0.91	0.59
-0.37	-1.11	0.33	3.52	1.24	-1.11	0.29
-0.77	-1.22	-0.34	0.64	-0.61	0.13	1.26
-0.77	-1.22	-0.34	0.64	-0.61	0.13	1.26
0.33	-1.03	0.00	2.22	1.44	-0.99	-1.00
-0.38	-0.43	0.05	0.83	-0.56	4.30	17.41
1.30	-0.96	-0.46	0.79	0.57	-1.57	-1.17
-0.81	-0.84	1.01	0.14	-0.47	-0.81	-0.96
-0.20	-1.10	0.74	1.94	0.65	-0.58	0.34
-0.05	-0.34	-0.15	0.22	-0.23	0.27	1.00
-0.47	-0.88	-0.06	-0.59	-0.26	0.28	3.76
0.13	-1.09	-0.85	0.08	0.20	7.67	54.32
0.23	-1.15	-0.06	-0.32	0.57	-0.12	1.11
-0.02	-1.01	-0.92	-0.34	-0.46	3.48	14.49
-0.06	-0.66	-0.76	-0.59	-0.26	0.02	-0.28
-0.56	-0.61	-0.67	-0.27	-0.49	2.77	2.67
-0.27	-1.64	-0.36	1.08	1.10	1.35	4.10
-0.56	-1.39	0.16	1.75	-0.19	-0.54	4.25
0.15	-0.73	0.70	0.21	-0.47	6.01	14.50
-0.15	-0.82	-0.36	0.89	-0.29	3.68	23.08
0.06	-1.54	1.42	3.90	0.55	0.53	7.55

33	34	1	2	3	4	5
35	36	37	38	39	40	41
TGTAAACATCCTI	TAGCTTATCAG/	TCGAGGAGCTCAC	TAGCAGCACATC/	CTAGACTGAAGCI	TGAGAACTGAATI	CACCCGGCTGTGI
miR-30e-5p	miR-21-5p	miR-151a-5p	miR-15b-5p	miR-151a-3p	miR-146a-5p	miR-941
457362	456843	454020	436288	393199	339140	313699
-0.35	-0.20	-1.15	-0.31	-0.32	-0.41	-0.82
-0.61	-0.24	0.25	0.05	2.84	2.17	3.71
0.21	-0.29	0.73	0.45	-0.21	-0.29	-0.35
-0.18	-0.33	-0.85	-0.12	-0.91	-0.40	-0.53
0.02	-0.04	3.24	4.79	0.42	1.57	0.86
-0.48	-0.25	-0.40	-0.37	-0.66	-0.05	0.04
-0.53	-0.18	-0.71	-0.45	-0.55	-0.70	-0.42
-0.44	-0.32	-1.52	-0.20	-0.95	-0.70	0.30
-0.63	-0.36	0.23	-0.18	0.50	-0.04	-0.13
-0.58	-0.26	-0.07	0.44	1.26	0.64	-0.62
-0.16	-0.18	1.75	-0.43	2.90	3.69	0.66
-0.51	-0.32	-0.98	-0.72	-0.74	-0.67	-0.84
3.39	4.83	0.35	-0.30	0.28	0.05	-0.52
-0.59	-0.34	-0.98	-0.14	-0.71	-0.58	0.37
-0.35	-0.27	-0.08	-0.12	-0.40	-0.50	0.34
0.86	0.23	1.33	-0.34	0.15	0.11	-0.67
-0.47	-0.32	-0.75	0.06	-0.80	-0.54	0.10
-0.16	-0.12	0.02	-0.04	-0.04	-0.72	-0.72
-0.14	-0.16	-0.63	-0.26	0.81	0.03	-0.64
2.91	0.96	0.86	-0.52	-0.23	-0.19	-0.50

-0.48	-0.26	0.63	-0.52	-0.32	-0.25	-0.58
-0.47	-0.34	-0.49	-0.48	-0.77	-0.74	-0.51
-0.34	-0.25	-0.05	-0.27	-0.57	-0.62	-0.37
-0.55	-0.30	-0.69	-0.19	-0.90	-0.69	-0.40
-0.47	-0.30	0.31	0.47	0.16	-0.15	2.27
0.04	-0.27	-0.25	-0.03	-0.52	0.02	0.45
1.07	-0.12	-0.09	-0.27	0.32	-0.03	-0.45
4.26	2.49	0.71	-0.56	0.29	0.67	-0.61
0.41	0.21	-0.27	-0.57	1.54	2.56	2.06
-0.49	-0.27	-0.72	-0.34	1.58	1.77	0.23
-0.70	-0.34	-0.69	0.53	1.04	0.19	1.88
0.03	0.11	0.49	-0.24	6.29	6.51	2.43
0.25	-0.10	4.56	4.82	1.58	1.13	0.96
-0.33	-0.10	-1.49	0.13	-0.84	-0.75	0.23
-0.35	0.04	-1.30	1.63	-0.26	-0.69	0.33
-0.21	-0.27	-0.84	-0.44	-0.68	-0.39	-0.07
-0.80	-0.38	-1.70	-0.40	-1.17	-0.88	-0.95
-0.35	0.28	-1.10	-0.40	0.75	0.07	-0.74
1.23	1.61	-0.46	-0.17	1.50	0.73	-0.68
-0.01	-0.21	-0.71	-0.38	-0.52	-0.39	-0.30
-0.09	-0.15	-0.85	-0.27	-0.75	-0.58	2.43
0.87	-0.14	-0.24	-0.68	0.05	0.29	1.75
-0.47	-0.33	0.36	-0.18	-0.61	-0.15	0.14
0.67	0.72	-0.44	0.02	0.01	-0.54	0.10
-0.65	-0.32	-0.89	-0.60	-0.04	-0.46	0.38
-0.46	-0.31	-1.26	-0.40	-1.03	-0.70	0.48
1.22	-0.19	1.95	-0.28	-0.11	0.50	-0.43
-0.20	-0.23	0.51	-0.39	-0.17	-0.49	0.15
0.14	-0.29	0.66	-0.31	-0.39	-0.60	-0.66
0.01	-0.32	0.07	-0.46	-0.66	-0.35	-0.57
0.48	-0.23	0.61	-0.46	-0.01	-0.01	-1.03
-0.42	-0.29	1.09	-0.34	-0.54	-0.31	0.92
-0.56	-0.30	1.22	0.03	-0.47	-0.52	0.03
0.17	-0.17	1.63	-0.09	-0.58	-0.31	-0.35
-0.51	-0.30	0.10	-0.61	-0.53	-0.31	-0.42
-0.54	-0.34	0.44	-0.01	-0.61	-0.32	0.50
-0.28	-0.30	1.06	-0.31	-0.48	-0.22	-0.75
4.64	1.96	-0.49	-0.56	-0.48	0.00	-0.93
-0.43	-0.32	-0.67	-0.19	-0.52	-0.36	0.37
0.64	0.00	0.77	0.11	0.45	-0.08	0.28
0.46	-0.12	1.02	0.83	0.30	0.97	-0.17
4.03	0.79	0.25	-0.36	-0.19	-0.38	-0.71
-0.32	-0.36	-0.96	0.21	-0.98	-0.68	0.06
-0.12	-0.28	-0.45	-0.30	-0.81	-0.31	-0.39
0.33	-0.33	0.50	-0.25	-0.22	0.20	-0.71
-0.36	-0.14	-0.48	-0.55	-0.51	-0.22	-1.05
1.24	0.24	-0.80	-0.20	0.44	0.76	-0.94
-0.73	-0.19	0.23	0.56	5.63	4.06	0.93
0.15	-0.28	2.18	2.96	0.04	-0.19	-0.74
1.06	-0.02	0.59	0.29	0.19	0.44	-0.12
-0.17	-0.27	-1.17	-0.33	-0.78	-0.46	-0.31
0.95	0.34	-1.69	-0.65	0.70	0.36	-1.03
-0.06	-0.21	-0.15	-0.22	-0.58	-0.11	-0.70
0.52	-0.01	-0.03	-0.30	-0.11	0.39	-0.56
-0.66	-0.38	-1.05	-0.72	-1.00	-0.70	1.07
0.34	0.04	-0.07	-0.54	-0.58	-0.52	-0.83
-0.36	-0.24	-0.19	-0.30	1.53	0.68	0.67
-0.56	-0.25	-0.61	-0.63	2.28	0.92	-0.34
1.50	-0.03	2.09	-0.12	0.36	0.49	-0.41

0.73	-0.19	2.10	-0.34	-0.01	1.31	-0.68
-0.21	-0.35	0.93	0.13	-0.57	-0.50	0.44
0.18	-0.24	1.81	-0.24	-0.60	0.72	0.12
-0.11	-0.30	-0.03	-0.65	-0.87	-0.69	-0.84
4.21	2.02	0.54	-0.52	-0.30	-0.35	-0.66
5.16	1.76	1.31	-0.52	-0.46	-0.25	-0.89
0.05	-0.34	1.06	-0.47	0.15	-0.43	-0.67
0.48	-0.27	0.59	-0.43	-0.63	-0.21	-0.37
0.02	-0.33	1.84	-0.08	-0.68	-0.27	-0.35
0.22	0.25	-0.21	-0.53	-0.09	-0.05	-0.12
-0.03	-0.29	1.32	-0.26	-0.63	-0.31	-0.67
0.89	0.22	0.41	-0.56	-0.47	0.14	-0.56
-0.47	-0.29	-1.10	-0.60	-0.81	-0.61	-0.17
-0.02	-0.26	-0.46	-0.26	-0.94	-0.63	-0.72
-0.08	-0.27	0.01	-0.27	0.53	0.57	-0.54
0.29	-0.06	-0.31	0.01	0.60	0.26	-0.60
0	0	-1	-1	-1	-1	0
0.00	0.00	0.00	0.00	0.00	0.00	0.00
1.00	1.00	1.00	1.00	1.00	1.00	1.00
0.59	0.82	0.37	0.41	0.99	0.93	0.01

33	34	35	36	37	38	39
TGTAAACATCCTT	TAGCTTATCAGA	TCGAGGAGCTCAC	TAGCAGCACATCA	CTAGACTGAAGCT	TGAGAACTGAATT	CACCCGGCTGTGT
miR-30e-5p	miR-21-5p	miR-151a-5p	miR-15b-5p	miR-151a-3p	miR-146a-5p	miR-941
457362	456843	454020	436288	393199	339140	313699
-0.35	-0.20	-1.15	-0.31	-0.32	-0.41	-0.82
-0.61	-0.24	0.25	0.05	2.84	2.17	3.71
0.21	-0.29	0.73	0.45	-0.21	-0.29	-0.35
-0.18	-0.33	-0.85	-0.12	-0.91	-0.40	-0.53
0.02	-0.04	3.24	4.79	0.42	1.57	0.86
-0.48	-0.25	-0.40	-0.37	-0.66	-0.05	0.04
-0.53	-0.18	-0.71	-0.45	-0.55	-0.70	-0.42
-0.44	-0.32	-1.52	-0.20	-0.95	-0.70	0.30
-0.63	-0.36	0.23	-0.18	0.50	-0.04	-0.13
-0.58	-0.26	-0.07	0.44	1.26	0.64	-0.62
-0.16	-0.18	1.75	-0.43	2.90	3.69	0.66
-0.51	-0.32	-0.98	-0.72	-0.74	-0.67	-0.84
3.39	4.83	0.35	-0.30	0.28	0.05	-0.52
-0.59	-0.34	-0.98	-0.14	-0.71	-0.58	0.37
-0.35	-0.27	-0.08	-0.12	-0.40	-0.50	0.34
0.86	0.23	1.33	-0.34	0.15	0.11	-0.67
-0.47	-0.32	-0.75	0.06	-0.80	-0.54	0.10
-0.16	-0.12	0.02	-0.04	-0.04	-0.72	-0.72
-0.14	-0.16	-0.63	-0.26	0.81	0.03	-0.64
2.91	0.96	0.86	-0.52	-0.23	-0.19	-0.50
-0.48	-0.26	0.63	-0.52	-0.32	-0.25	-0.58
-0.47	-0.34	-0.49	-0.48	-0.77	-0.74	-0.51
-0.34	-0.25	-0.05	-0.27	-0.57	-0.62	-0.37
-0.55	-0.30	-0.69	-0.19	-0.90	-0.69	-0.40
-0.47	-0.30	0.31	0.47	0.16	-0.15	2.27
0.04	-0.27	-0.25	-0.03	-0.52	0.02	0.45
1.07	-0.12	-0.09	-0.27	0.32	-0.03	-0.45
4.26	2.49	0.71	-0.56	0.29	0.67	-0.61

0.41	0.21	-0.27	-0.57	1.54	2.56	2.06
-0.49	-0.27	-0.72	-0.34	1.58	1.77	0.23
-0.70	-0.34	-0.69	0.53	1.04	0.19	1.88
0.03	0.11	0.49	-0.24	6.29	6.51	2.43
0.25	-0.10	4.56	4.82	1.58	1.13	0.96
-0.33	-0.10	-1.49	0.13	-0.84	-0.75	0.23
-0.35	0.04	-1.30	1.63	-0.26	-0.69	0.33
-0.21	-0.27	-0.84	-0.44	-0.68	-0.39	-0.07
-0.80	-0.38	-1.70	-0.40	-1.17	-0.88	-0.95
-0.35	0.28	-1.10	-0.40	0.75	0.07	-0.74
1.23	1.61	-0.46	-0.17	1.50	0.73	-0.68
-0.01	-0.21	-0.71	-0.38	-0.52	-0.39	-0.30
-0.09	-0.15	-0.85	-0.27	-0.75	-0.58	2.43
0.87	-0.14	-0.24	-0.68	0.05	0.29	1.75
-0.47	-0.33	0.36	-0.18	-0.61	-0.15	0.14
0.67	0.72	-0.44	0.02	0.01	-0.54	0.10
-0.65	-0.32	-0.89	-0.60	-0.04	-0.46	0.38
-0.46	-0.31	-1.26	-0.40	-1.03	-0.70	0.48
1.22	-0.19	1.95	-0.28	-0.11	0.50	-0.43
-0.20	-0.23	0.51	-0.39	-0.17	-0.49	0.15
0.14	-0.29	0.66	-0.31	-0.39	-0.60	-0.66
0.01	-0.32	0.07	-0.46	-0.66	-0.35	-0.57
0.48	-0.23	0.61	-0.46	-0.01	-0.01	-1.03
-0.42	-0.29	1.09	-0.34	-0.54	-0.31	0.92
-0.56	-0.30	1.22	0.03	-0.47	-0.52	0.03
0.17	-0.17	1.63	-0.09	-0.58	-0.31	-0.35
-0.51	-0.30	0.10	-0.61	-0.53	-0.31	-0.42
-0.54	-0.34	0.44	-0.01	-0.61	-0.32	0.50
-0.28	-0.30	1.06	-0.31	-0.48	-0.22	-0.75
4.64	1.96	-0.49	-0.56	-0.48	0.00	-0.93
-0.43	-0.32	-0.67	-0.19	-0.52	-0.36	0.37
0.64	0.00	0.77	0.11	0.45	-0.08	0.28
0.46	-0.12	1.02	0.83	0.30	0.97	-0.17
4.03	0.79	0.25	-0.36	-0.19	-0.38	-0.71
-0.32	-0.36	-0.96	0.21	-0.98	-0.68	0.06
-0.12	-0.28	-0.45	-0.30	-0.81	-0.31	-0.39
0.33	-0.33	0.50	-0.25	-0.22	0.20	-0.71
-0.36	-0.14	-0.48	-0.55	-0.51	-0.22	-1.05
1.24	0.24	-0.80	-0.20	0.44	0.76	-0.94
-0.73	-0.19	0.23	0.56	5.63	4.06	0.93
0.15	-0.28	2.18	2.96	0.04	-0.19	-0.74
1.06	-0.02	0.59	0.29	0.19	0.44	-0.12
-0.17	-0.27	-1.17	-0.33	-0.78	-0.46	-0.31
0.95	0.34	-1.69	-0.65	0.70	0.36	-1.03
-0.06	-0.21	-0.15	-0.22	-0.58	-0.11	-0.70
0.52	-0.01	-0.03	-0.30	-0.11	0.39	-0.56
-0.66	-0.38	-1.05	-0.72	-1.00	-0.70	1.07
0.34	0.04	-0.07	-0.54	-0.58	-0.52	-0.83
-0.36	-0.24	-0.19	-0.30	1.53	0.68	0.67
-0.56	-0.25	-0.61	-0.63	2.28	0.92	-0.34
1.50	-0.03	2.09	-0.12	0.36	0.49	-0.41
0.73	-0.19	2.10	-0.34	-0.01	1.31	-0.68
-0.21	-0.35	0.93	0.13	-0.57	-0.50	0.44
0.18	-0.24	1.81	-0.24	-0.60	0.72	0.12
-0.11	-0.30	-0.03	-0.65	-0.87	-0.69	-0.84
4.21	2.02	0.54	-0.52	-0.30	-0.35	-0.66
5.16	1.76	1.31	-0.52	-0.46	-0.25	-0.89
0.05	-0.34	1.06	-0.47	0.15	-0.43	-0.67
0.48	-0.27	0.59	-0.43	-0.63	-0.21	-0.37

0.02	-0.33	1.84	-0.08	-0.68	-0.27	-0.35
0.22	0.25	-0.21	-0.53	-0.09	-0.05	-0.12
-0.03	-0.29	1.32	-0.26	-0.63	-0.31	-0.67
0.89	0.22	0.41	-0.56	-0.47	0.14	-0.56
-0.47	-0.29	-1.10	-0.60	-0.81	-0.61	-0.17
-0.02	-0.26	-0.46	-0.26	-0.94	-0.63	-0.72
-0.08	-0.27	0.01	-0.27	0.53	0.57	-0.54
0	0	0	0	1	0	-1

33	34	35	36	37	38	39
73	122	115	135	48	72	39
TGAGGTAGTAAGT	TAACAGTCTACA	AGGCAAGATGCTG	TCGACCGGACCTG	TCTCTCGGCTCCT	CGGGGCAGCTCAG	CACCCGGCTGTGT
miR-98-5p	miR-132-3p	miR-31-5p	miR-1307-5p	miR-3615	miR-486-3p	miR-941
69114	15261	18520	10674	198175	70256	313699
-0.69	0.91	-0.50	1.73	0.68	0.34	-0.82
0.68	-2.06	-1.48	-0.86	-1.33	1.41	3.71
0.20	-0.75	-0.21	0.40	-0.06	0.18	-0.35
-0.42	0.24	-0.93	0.00	0.17	1.09	-0.53
-0.04	-0.87	1.96	-0.86	-0.81	-0.48	0.86
-0.48	0.62	-0.74	-0.40	-0.47	0.43	0.04
-0.64	1.64	0.74	-0.19	0.11	-0.21	-0.42
-0.81	0.91	-0.46	-0.55	1.49	1.71	0.30
-0.37	-0.97	-1.00	-0.66	-0.83	-0.52	-0.13
-0.22	0.27	-0.84	-0.62	-1.23	-0.69	-0.62
0.18	-0.59	0.28	-0.47	-1.04	-0.98	0.66
-0.56	0.78	0.64	-0.62	2.97	0.73	-0.84
3.58	1.88	-0.20	3.76	-0.45	-1.18	-0.52
-0.77	1.28	-0.23	-0.65	0.26	1.84	0.37
0.08	-0.47	-0.73	-0.33	0.16	-0.43	0.34
1.25	0.46	-0.07	0.91	-0.56	-0.92	-0.67
-0.80	0.45	0.82	-0.24	-0.09	-0.33	0.10
0.00	0.38	0.41	0.06	-0.13	0.27	-0.72
-0.10	0.22	-0.83	-0.28	-0.33	-1.30	-0.64
1.50	-0.57	0.21	1.46	-0.22	-0.85	-0.50
-0.23	0.09	-0.98	-0.45	-0.34	0.68	-0.58
-0.79	0.35	2.31	-0.37	1.85	0.36	-0.51
-0.63	-0.24	0.71	-0.33	0.78	0.67	-0.37
-0.83	0.31	0.41	-0.24	1.42	1.76	-0.40
-0.13	-2.06	-0.47	-0.71	-1.05	-0.82	2.27
-0.66	-0.72	2.20	-0.23	-0.37	-1.30	0.45
1.70	-1.52	-1.01	0.75	-0.57	-1.47	-0.45
7.47	-1.24	-0.43	3.75	-1.00	-1.05	-0.61
1.54	-0.10	-0.45	1.44	-0.70	-0.93	2.06
0.41	-0.31	-1.21	-0.50	-0.66	0.04	0.23
-0.51	-0.99	-1.37	-0.86	-0.39	2.34	1.88
3.02	-0.63	-1.15	-0.53	-0.92	-0.57	2.43
0.99	-1.63	-0.43	-0.46	-1.09	-0.02	0.96
-0.78	1.49	-1.09	-0.46	1.03	0.02	0.23
-0.70	1.57	-1.18	-0.37	0.94	0.12	0.33
-0.73	1.30	4.13	-0.15	-0.52	-0.35	-0.07
-0.86	-0.96	-1.28	-0.82	0.51	4.26	-0.95
0.44	-0.46	1.78	-0.36	-0.84	-0.77	-0.74
2.47	0.79	1.65	0.89	-1.16	-1.19	-0.68

-0.56	0.57	1.57	-0.29	0.05	-1.38	-0.30
-0.68	-1.57	-0.25	3.68	1.30	1.65	2.43
-0.12	-1.17	5.93	23.08	5.55	1.80	1.75
-0.62	0.33	-0.44	-0.43	0.25	-0.84	0.14
0.54	0.76	-1.00	0.57	0.30	-1.21	0.10
-0.55	1.00	-0.17	-0.62	1.52	-0.34	0.38
-0.88	-1.55	-0.51	1.27	1.74	0.83	0.48
-0.12	-0.04	0.00	1.23	-0.79	-1.36	-0.43
-0.55	1.09	1.19	0.27	1.29	-0.63	0.15
-0.48	1.03	-0.20	-0.12	0.57	0.04	-0.66
-0.77	1.08	2.30	-0.02	0.27	-0.51	-0.57
-0.31	1.43	1.10	0.51	-0.08	-0.61	-1.03
-0.75	-0.70	0.22	-0.17	0.07	-0.57	0.92
-0.63	0.75	-1.19	-0.57	-0.33	0.43	0.03
-0.26	1.32	0.43	0.04	0.29	-0.60	-0.35
-0.59	1.58	-0.40	-0.28	0.43	1.69	-0.42
-0.80	0.34	-0.30	-0.60	-0.06	0.44	0.50
-0.41	0.08	-0.03	-0.13	-0.27	-0.02	-0.75
4.07	-1.54	0.81	3.36	-0.99	-1.58	-0.93
-0.61	-0.77	2.36	-0.40	0.69	-0.58	0.37
0.31	-1.22	1.36	-0.21	0.02	-1.25	0.28
0.12	-1.15	1.12	-0.54	-0.80	-1.28	-0.17
3.39	-1.83	-0.05	1.84	-0.74	-1.61	-0.71
-0.77	-0.97	-0.47	-0.34	0.33	-0.92	0.06
-0.34	-1.33	0.04	0.08	-0.15	-1.00	-0.39
0.17	-1.15	-0.94	0.00	-0.72	-1.29	-0.71
0.72	1.76	-0.96	-0.26	2.07	0.75	-1.05
0.99	-0.12	-1.25	2.98	-0.88	-0.74	-0.94
0.88	0.18	-0.11	-0.86	-0.68	-0.93	0.93
0.19	-0.11	-0.71	-0.36	0.17	5.61	-0.74
0.57	1.86	0.41	1.70	1.20	1.25	-0.12
-0.42	1.47	-0.72	-0.23	0.83	0.90	-0.31
-0.40	0.02	-0.89	-0.36	-0.21	-0.92	-1.03
-0.05	0.53	-0.61	-0.31	0.04	-0.13	-0.70
0.64	2.38	0.43	-0.14	-0.21	-0.72	-0.56
-0.87	-1.60	-0.09	12.56	6.29	1.98	1.07
0.71	0.37	1.16	0.48	0.21	0.02	-0.83
0.03	-0.16	0.44	-0.53	-0.62	-1.15	0.67
0.81	-0.82	-1.29	-0.72	-0.35	-0.59	-0.34
1.50	-0.38	0.16	2.73	-1.11	-0.68	-0.41
0.42	-1.13	-0.43	0.56	-0.92	-1.16	-0.68
-0.72	-0.69	-0.04	-0.32	0.14	-0.24	0.44
0.35	-0.34	-0.18	0.20	-0.65	-0.97	0.12
-0.72	1.80	0.69	-0.08	1.86	0.20	-0.84
2.76	-0.61	1.83	1.18	0.09	-1.08	-0.66
3.98	-1.40	0.24	3.22	-1.03	-1.23	-0.89
-0.68	-0.30	-0.08	0.05	0.11	-0.42	-0.67
-0.47	0.43	0.88	0.87	-0.39	-1.05	-0.37
-0.63	0.01	0.78	-0.14	-0.10	-0.10	-0.35
0.66	0.19	0.01	0.31	-0.27	-0.49	-0.12
-0.62	0.93	0.82	-0.15	-0.43	-0.10	-0.67
3.61	-1.32	-0.94	-0.22	-0.55	-0.41	-0.56
-0.62	0.88	-0.86	-0.40	0.20	-0.22	-0.17
-0.72	0.24	0.55	0.54	0.09	0.44	-0.72
1.54	-1.12	-1.13	-0.11	-0.71	-1.53	-0.54
0.16	-0.76	-0.98	0.69	-0.42	-1.33	-0.60
0.07	0.08	0.09	0.09	0.09	0.09	0.10
0.05	0.03	0.04	0.11	0.05	0.66	0.18

2072241	4157	727386	206047	2758841	2173631	852941
	1					
	1					
43	44	45	46	47	48	49
13	14	15	16	17	18	19
C_48	C_5	D_10	D_12	D_13	D_14	D_3
CHR_NP	CHR_NP	CHR_NP	CHR_NP	CHR_NP	CHR_NP	CHR_NP
3193519	141023	2632177	1206683	6374876	4715753	1806730
0.18	0.47	0.89	0.71	0.66	0.72	0.77
C_48	C_5	D_10	D_12	D_13	D_14	D_3
1121278	136866	1904791	1000636	3616035	2542122	953789
593526	288	308682	28125	1230163	772506	380980
152869	15	23073	6563	199880	185575	112586
385992	95	63225	24309	229684	83193	98937
114740	6	11822	2047	157501	74283	21941
254736	1400	89228	33653	246324	96288	60780
27400	1	2763	381	42920	105430	12798
104908	6	22052	6808	86557	103298	17153
7545	36	2512	2325	3400	36567	1632
22013	267	4185	2552	27028	56592	9145
23827	25	6144	1172	37748	100568	10874
12756	102	23750	19091	35245	64172	3387
35505	19	8613	2253	45989	21913	11262
21950	7	4686	1683	25349	29263	2341
18735	61	15532	6102	31222	12564	6726
10207	0	613	202	4748	17127	2018
12774	106	8711	4731	36283	14749	5776
9339	5	790	192	4484	18325	1316
12358	5	5454	2635	15964	24721	4646
13684	4	609	126	10630	21944	3246
15631	13	6584	1697	19659	25876	7572
16830	249	6083	3941	8759	13123	3804
7357	2	1802	494	12074	15624	1878
4425	0	990	270	6338	30600	1503
13111	1	467	230	6183	13026	1523
8660	1	1201	676	3848	4041	1720
6792	5	2657	1291	13498	11428	3012
5710	392	8014	7864	10138	4225	3424
2119	7	1372	413	2137	9154	844
4318	4	1315	494	10180	5410	1580
4042	0	7254	2374	13760	10216	1876
9334	0	1073	264	4796	10179	3273
7112	0	1333	440	7773	2541	1568
3775	3	1146	644	2420	6848	432
2250	3	973	258	1060	13550	385
4019	4	1204	459	9511	4939	1450
5146	0	2115	172	9755	9678	1195
4433	15	1060	646	5290	7562	2910
3480	10	769	665	6660	2609	1267
4769	7	6449	1347	9551	7122	3472
7744	72	9122	3042	2286	837	6494
6633	4	10386	2733	5644	5686	1881
8448	15	3106	999	6226	4133	2206
2888	4	3292	742	6045	3709	913
1156	12	220	90	439	2491	342
7260	8	1532	1263	3435	1792	1277

3352	4	978	839	4376	2161	1208
2009	1	1205	1306	5353	4293	505
3270	123	1891	1190	4920	3871	2530
135	0	32	32	295	367	27
1169	6	758	464	803	5082	435
918	179	1652	870	4985	1132	481
3210	0	754	231	1978	5798	547
1434	2	3074	2195	3694	4232	836
2549	0	85	37	1468	3465	1250
1239	4	1707	698	2379	3983	266
113	2	79	29	150	1017	19
1573	3	657	362	2561	2812	490
2705	3	111	65	1562	1896	481
2566	0	219	72	1939	2073	756
2358	7	658	360	4233	2077	1003
1390	17	1142	1023	3305	1620	401
2349	63	809	669	1297	2676	615
150	1	8	15	447	721	69
108	27	25	13	253	160	43
515	33	234	92	1463	1046	801
552	0	174	42	892	1651	176
745	0	176	85	339	1657	115
232	0	116	21	430	916	50
899	0	1027	513	1412	2151	114
331	0	143	14	722	1093	162
1080	7	831	161	2138	1361	662
412	3	791	212	1020	540	450
265	1	61	48	294	1046	112
512	1	224	76	563	1584	203
1116	0	95	20	886	1147	155
618	29	491	265	1580	487	409
747	2	835	328	1295	1575	347
416	3	400	388	1538	912	354
921	1	780	301	1692	668	606
834	1	208	124	1425	1187	228
813	0	107	59	706	521	80
834	50	855	493	1520	847	319
787	0	147	11	1322	780	341
197	1	33	4	296	6428	22
400	1	175	197	504	836	55
702	35	2554	2880	1238	692	154
564	60	1136	1039	1672	666	660
224	0	13	6	239	279	74
516	0	65	45	335	716	123
448	0	304	145	888	254	319
856	5	347	152	880	392	425
9	0	21	16	81	50	4
633	4	291	130	568	381	99
181	0	24	15	549	466	63
107	0	41	8	146	596	16
618	0	132	46	267	566	120
554	0	240	121	789	493	545
429	7	423	274	707	604	343
824	3	448	124	755	252	412
65	0	10	4	107	598	29
492	0	887	245	468	609	69
304	0	45	23	477	373	106
688	4	510	399	661	184	211
465	0	28	20	383	350	86

200	1	31	9	152	656	91
106	0	26	14	68	327	40
436	3	998	754	594	523	218
387	0	66	11	418	652	117
110	0	98	18	125	581	12
117	1	345	105	118	665	25
502	0	6	9	272	383	200
386	0	167	57	470	337	168
310	3	670	329	648	650	233
86	0	28	6	157	247	17
524	0	70	109	240	84	93
256	0	58	34	413	90	86
329	0	31	20	294	505	35
113	0	75	34	256	529	37
113	0	75	34	256	529	37
613	0	17	4	262	340	123
128	16	332	286	398	316	48
276	0	17	8	336	305	133
88	0	9	0	136	79	62
266	1	30	19	125	405	113
28	0	39	21	50	124	14
209	1	96	69	350	230	95
206	31	390	617	417	130	48
260	0	58	28	326	175	127
126	2	282	245	382	144	127
158	0	79	13	353	174	116
41	0	180	45	162	39	14
232	1	84	41	174	255	153
122	0	30	45	351	151	25
103	0	385	217	156	319	42
64	29	169	229	65	165	11
188	6	62	60	300	302	57
70	0	36	30	155	161	23
58	1	88	147	134	242	21
86	0	5	13	193	112	19
119	2	145	139	391	119	28
136	6	118	49	225	137	35
124	1	38	19	144	200	28
250	1	168	81	111	29	147
80	0	36	12	179	198	17
10	0	19	26	23	75	6
57	1	67	39	174	81	40
201	0	31	2	143	178	31
77	0	4	1	101	112	44
117	0	21	10	203	200	31
61	1	217	78	112	142	24
114	0	50	18	85	117	86
46	0	24	20	58	101	3
36	0	9	0	61	64	14
161	1	45	12	153	118	47
226	0	9	2	46	20	13
30	0	92	63	179	178	39
81	0	27	25	67	161	7
64	0	193	86	222	79	47
82	2	119	92	153	89	32
19	1	69	71	150	134	32
3	0	3	3	19	35	2
156	2	214	116	143	165	24
72	3	138	71	122	74	46

66	0	22	6	54	186	18
74	0	27	33	61	359	2
62	1	89	31	139	72	43
84	0	13	1	152	113	22
95	1	312	223	57	107	81
54	0	2	0	37	32	7
25	0	21	10	79	107	12
151	2	271	79	142	78	22
51	0	124	23	80	410	5
166	1	16	12	217	89	65
102	0	17	1	76	101	60
88	0	12	1	36	165	26
77	1	389	116	115	127	31
17	2	58	48	188	64	13
76	0	35	24	98	65	33
64	0	105	62	55	140	12
40	0	157	29	180	37	28
103	0	22	12	89	67	41
22	0	9	6	21	93	8
18	0	93	40	183	26	4
52	0	4	1	23	78	31
28	0	41	20	179	40	37
82	2	115	118	55	36	24
22	0	9	5	21	90	8
29	0	35	7	91	41	5
70	0	31	69	76	102	19
76	0	14	7	82	62	21
2	4	36	51	520	1	22
24	0	1	0	7	98	24
77	1	24	18	81	107	18
83	0	13	2	112	83	97
31	0	20	13	161	23	25
49	0	34	28	83	21	17
16	0	6	0	9	77	6
29	0	22	9	87	83	25
33	6	115	95	93	129	6
61	4	52	46	57	48	10
101	1	22	11	53	15	62
57	0	3	2	87	120	70
46	0	102	66	54	18	37
32	1	38	18	108	129	27
19	0	27	5	27	78	1
20	6	51	50	144	12	3
58	2	49	34	32	42	14
69	0	14	2	65	68	36
29	0	11	21	124	62	19
19	0	35	24	46	22	21
75	0	45	30	81	33	28
95	0	23	4	59	62	52
14	0	1	0	18	19	11
54	0	68	41	73	47	9
19	4	37	21	53	75	12
80	0	44	37	43	32	42
61	0	14	8	56	76	9
61	0	16	13	25	93	15
1	0	1	1	1	1	0
45	0	6	0	39	59	5
8	0	2	0	3	9	3
35	0	39	34	62	47	14

40	0	13	21	39	23	2
29	1	56	26	19	69	7
25	0	4	2	49	8	11
48	0	44	25	47	20	21
26	0	33	29	45	12	11
16	1	14	15	49	22	13
36	0	35	16	64	33	15
25	0	9	2	32	49	3
19	0	6	6	14	45	5
15	0	20	10	34	30	27
9	0	1	0	5	19	0
21	0	59	34	65	32	10
18	0	5	6	27	26	3
12	2	39	56	45	8	3
43	0	108	118	51	17	4
14	0	0	2	8	116	1
34	1	14	6	10	51	6
31	0	7	5	26	31	13
6	0	21	8	56	18	4
23	0	31	26	15	47	1
13	0	2	1	5	33	0
15	0	3	0	24	59	5
15	0	3	0	24	59	5
21	0	25	7	59	7	4
32	0	35	19	32	26	15
14	0	22	10	21	26	2
14	0	9	4	41	12	8
30	0	1	0	17	19	10
44	0	16	15	42	57	8
11	0	68	22	21	96	0
13	0	5	1	17	23	8
28	0	46	160	26	11	3
52	0	5	2	12	26	18
70	0	13	0	61	83	16
24	0	0	3	19	16	7
21	0	9	2	23	39	2
0	0	1	4	2	5	0
15	0	20	8	36	25	7
17	0	3	0	12	27	6
32	1	32	13	22	6	16
11	0	12	12	39	19	10
17	0	3	0	9	27	4
22	1	23	6	61	28	17
12	0	11	7	44	6	2
11	0	7	4	14	19	6
10	0	19	13	23	30	2
2	0	4	1	2	3	2
34	1	43	78	20	2	12
25	1	18	9	25	9	8
10	2	15	21	44	6	2
9	0	4	2	40	7	1
14	0	2	1	16	33	7
1	0	1	1	2	10	0
21	0	10	1	23	16	6
29	0	2	1	10	56	7
17	0	0	23	1	159	0
12	0	24	31	14	9	9
18	0	0	2	14	24	4
23	0	34	9	24	16	5

7	0	38	17	33	4	5
17	0	4	0	38	14	11
12	0	11	8	18	6	3
24	1	20	12	38	23	3
5	0	10	11	36	13	10
18	0	14	3	36	17	24
27	0	1	1	10	50	7
15	0	2	1	17	9	6
1	0	3	0	3	12	0
5	0	0	0	2	23	3
21	0	0	1	16	9	12
12	0	30	7	26	36	6
7	0	14	4	16	50	0
41	0	8	6	7	6	6
2	0	15	5	24	2	9
11	0	0	1	2	23	7
10	0	8	4	34	10	10
14	1	36	21	11	13	4
14	1	39	27	15	11	1
7	0	0	2	5	3	4
8	0	10	6	22	2	3
14	0	7	2	21	4	8
18	0	27	9	30	3	15
2	2	2	15	31	14	7
12	0	2	0	19	9	8
20	0	9	5	9	10	9
23	0	19	10	21	3	26
6	0	27	37	17	7	7
16	0	19	32	19	7	3
22	0	2	4	15	17	1
10	1	1	0	8	10	6
10	0	0	1	5	14	5
5	0	0	0	1	10	1
4	0	1	1	9	19	1
11	0	0	0	10	8	3
7	0	21	10	17	19	8
10	0	3	1	18	13	3
2	2	13	27	21	8	4
13	0	9	1	16	7	4
18	0	6	3	21	18	9
8	0	5	4	5	15	2
20	0	3	8	11	4	2
5	0	8	2	21	5	2
11	0	4	5	11	10	4
8	0	0	3	20	10	2
16	1	0	5	23	50	0
7	0	9	7	15	5	6
1	0	5	2	6	11	1
8	0	26	11	14	16	2
18	0	5	1	15	9	3
8	0	12	12	15	2	7
0	0	0	2	3	10	0
12	0	29	5	10	14	3
3	0	1	0	2	11	1
10	0	6	5	17	11	2
23	0	8	1	4	6	1
11	0	5	5	17	7	6
1	0	0	0	2	2	0
0	0	9	1	13	5	4

7	0	1	3	19	5	2
2	0	3	7	16	5	7
18	1	6	3	5	2	7
14	0	4	2	15	10	3
7	0	5	3	11	16	8
16	0	8	9	16	5	3
5	0	6	13	12	8	1
0	0	1	0	5	9	0
7	0	7	4	10	10	0
9	0	3	0	19	7	2
9	0	7	8	19	18	5
20	0	2	1	3	6	2
8	0	0	8	5	1	8
3	0	0	0	1	12	0
14	0	4	1	9	3	2
5	0	5	5	7	10	1
13	0	2	2	8	14	1
3	0	10	4	13	6	3
3	0	2	6	6	12	1
9	0	19	6	12	13	0
11	0	1	5	1	9	3
12	0	1	1	9	29	4
6	0	0	0	10	5	1
3	0	1	0	14	9	4
0	0	2	8	14	6	0
8	0	9	6	14	8	1
4	0	0	1	0	3	0
3	0	4	0	1	17	0
4	0	6	1	12	11	5
5	0	3	0	5	6	0
26	0	0	6	5	18	1
0	0	7	2	9	4	1
5	0	5	1	4	6	0
12	0	2	1	4	9	8
0	0	0	1	1	3	1
2	0	5	1	17	3	1
7	0	1	3	3	1	4
6	0	16	6	6	2	0
5	1	12	3	8	5	7
12	0	6	0	9	6	3
12	0	9	9	1	1	14
11	0	2	2	2	3	0
7	0	1	0	4	3	0
4	0	5	0	8	3	1
7	0	5	4	2	3	1
13	1	10	2	8	11	1
1	0	0	2	8	4	1
2	1	5	3	14	6	7
2	0	0	0	1	7	2
6	0	2	2	2	0	0
4	0	13	3	10	7	2
2	0	3	0	9	3	0
5	0	2	0	0	8	1
3	0	9	9	1	4	0
8	0	0	0	4	4	3
2	0	0	0	1	2	2
6	0	2	1	2	7	0
7	0	4	1	6	8	0
1	0	1	12	0	13	1

2	0	0	0	1	9	1
1	0	2	3	8	8	0
7	0	0	1	2	1	5
6	1	0	2	1	1	4
9	0	6	4	5	3	1
1	0	1	4	10	0	1
8	0	4	3	7	5	0
4	0	6	8	2	5	0
4	0	1	1	3	3	2
3	0	3	0	2	4	2
1	0	0	0	0	12	1
0	0	0	0	0	2	0
0	0	0	0	0	2	0
3	0	1	0	0	1	1
9	0	7	8	8	6	5
1	0	10	5	9	1	3
1	0	3	6	7	4	0
1	0	0	1	10	4	3
0	0	0	0	0	0	0
0	0	1	1	9	5	0
2	0	1	4	6	2	0
2	0	0	0	1	6	0
2	0	3	0	6	8	2
4	0	3	9	4	1	1
1	0	5	1	0	3	2
0	0	0	0	0	2	0
3	0	4	2	9	2	0
4	0	0	0	0	10	1
0	0	2	0	9	3	1
5	0	0	0	7	6	3
0	0	0	1	0	0	0
3	0	0	0	6	5	1
2	0	0	1	2	5	2
1	0	4	2	2	0	3
2	0	1	0	2	3	1
3	0	1	0	6	6	3
5	0	6	1	5	0	2
4	0	1	1	8	5	0
6	0	1	0	6	1	1
1	0	0	0	7	1	1
0	0	0	0	0	1	0
4	0	1	0	4	4	1
2	0	1	0	7	4	2
0	0	0	3	3	3	0
1	0	0	0	1	12	2
0	0	0	0	1	2	0
1	0	0	0	1	3	0
10	0	5	1	6	4	0
5	0	13	2	4	9	1
3	0	0	0	7	0	0
4	0	1	0	3	0	2
1	0	1	0	0	0	1
4	1	1	1	5	1	2
5	0	1	4	2	12	0
5	0	0	0	5	2	1
3	0	0	0	4	4	0
1	0	4	1	2	5	0
0	0	4	3	3	3	1
0	0	10	1	6	2	1

3	0	22	0	4	2	0
2	0	2	2	9	2	0
4	0	1	0	2	4	2
2	0	2	1	3	8	2
0	0	1	1	3	4	0
3	0	1	0	0	3	0
2	0	3	0	8	2	0
0	0	1	0	0	1	1
4	0	1	1	2	4	2
0	0	0	0	0	0	0
0	0	0	4	0	0	1
1	0	3	1	2	2	4
0	0	0	1	1	6	0
0	1	0	1	1	0	1
0	0	0	0	1	0	1
1	0	0	0	2	5	0
1	0	3	0	4	2	1
0	0	0	0	1	0	0
1	0	0	0	0	0	1
2	0	1	0	0	3	1
2	0	3	0	2	2	1
0	0	1	0	0	1	0
0	0	0	0	0	4	2
3	0	3	1	2	3	1
5	0	6	2	0	1	2
7	0	0	0	2	3	2
6	0	1	0	1	2	5
2	0	1	1	3	1	1
0	0	0	0	0	2	0
0	0	3	0	1	1	0
0	0	0	0	2	0	0
3	0	3	1	2	4	3
0	0	1	0	2	2	1
1	0	8	0	7	1	0
4	0	5	1	7	2	0
2	0	2	3	2	4	0
0	1	16	5	4	3	1
0	0	3	1	1	3	1
2	0	2	0	0	2	0
4	0	1	0	0	0	4
0	0	0	0	1	5	0
2	0	3	0	7	1	1
2	0	0	0	1	0	1
2	0	2	1	5	0	3
4	0	0	0	2	4	2
3	0	0	0	2	0	1
1	0	6	1	3	0	4
0	0	1	0	3	0	0
1	0	4	3	0	1	0
0	0	2	2	4	5	1
2	0	3	2	4	3	2
0	0	1	0	0	1	0
0	0	0	0	1	3	0
1	0	1	0	0	4	0
0	0	2	1	1	2	1
4	0	2	1	1	1	1
2	0	1	0	2	9	4
5	1	3	0	1	5	2
0	0	0	0	0	1	1

1	0	0	0	1	3	0
3	0	0	1	0	1	2
1	0	2	0	0	1	0
3	0	0	0	2	0	0
0	0	0	0	6	3	0
1	0	1	0	1	1	0
2	0	3	1	1	3	6
0	0	1	1	6	0	1
2	0	7	2	1	4	2
0	0	0	1	0	10	1
2	0	4	0	0	1	0
0	0	3	0	0	1	0
0	22	0	0	0	0	0
C_48	C_5	D_10	D_12	D_13	D_14	D_3
CHR_NP	CHR_NP	CHR_NP	CHR_NP	CHR_NP	CHR_NP	CHR_NP
1121278	136866	1904791	1000636	3616035	2542122	953789
11	30	23	27	18	17	17
-2.09	1.88	0.39	1.11	-0.68	-0.83	-0.94

0.78	0.82	0.36	0.07	0.20	0.25	0.31
12	11	19	32	29	26	21
1	1	1	1	1	1	1
43	44	45	46	47	48	49
16	17	18	19	20	21	22
D_13	D_14	D_3	D_9	E_20	E_29	E_4
CHR_NP	CHR_NP	CHR_NP	CHR_NP	CHR_NP	CHR_NP	CHR_NP
2758841	2173631	852941	239935	1893208	1240451	2304775
D_13	D_14	D_3	D_9	E_20	E_29	E_4
1230163	772506	380980	122679	389983	434200	899048
199880	185575	112586	4360	256917	87950	162314
229684	83193	98937	12343	135742	104686	168898
157501	74283	21941	2068	88459	83637	78820
246324	96288	60780	39570	121914	149724	164506
42920	105430	12798	1297	138924	19072	128682
86557	103298	17153	6148	189457	55740	89051
3400	36567	1632	453	18376	3407	35682
27028	56592	9145	1444	60543	20112	79855
37748	100568	10874	949	33423	16296	33869
35245	64172	3387	4340	14649	11862	19996
45989	21913	11262	2892	33311	26726	63887
25349	29263	2341	966	21311	11884	14690
31222	12564	6726	5265	10244	11251	23877
4748	17127	2018	146	12249	5069	11310
36283	14749	5776	4360	14452	19783	30744
4484	18325	1316	165	16723	3485	9674
15964	24721	4646	794	12614	9448	22251
10630	21944	3246	172	20440	4826	16228

19659	25876	7572	1368	15097	12160	9087
8759	13123	3804	832	10639	7806	8818
12074	15624	1878	295	6410	4200	9798
6338	30600	1503	89	11806	6426	5072
6183	13026	1523	74	30945	4146	12014
3848	4041	1720	181	12014	2145	3273
13498	11428	3012	491	10595	5810	12898
10138	4225	3424	3164	3971	6324	8727
2137	9154	844	225	4317	1897	9309
10180	5410	1580	306	11047	4712	9403
13760	10216	1876	1144	8071	6087	5189
4796	10179	3273	173	15625	5490	15629
7773	2541	1568	311	5170	5106	4727
2420	6848	432	211	8030	1759	4918
1060	13550	385	117	2224	1179	1443
9511	4939	1450	280	10370	4460	8887
9755	9678	1195	563	5536	3045	6668
5290	7562	2910	190	5926	3775	5773
6660	2609	1267	176	8080	1710	2449
9551	7122	3472	1015	3615	4264	3159
2286	837	6494	3700	3582	2940	2314
5644	5686	1881	2104	5684	4438	4198
6226	4133	2206	905	3620	3242	3567
6045	3709	913	840	4933	2476	5529
439	2491	342	29	1420	336	1352
3435	1792	1277	212	4902	2611	3671
4376	2161	1208	210	5572	3584	4328
5353	4293	505	243	4614	1861	2385
4920	3871	2530	744	1477	3362	4775
295	367	27	23	338	162	616
803	5082	435	51	3054	1345	1917
4985	1132	481	311	2097	2013	605
1978	5798	547	69	3858	1336	2036
3694	4232	836	855	1586	1244	2444
1468	3465	1250	17	2990	1379	1240
2379	3983	266	477	2996	1905	1145
150	1017	19	13	247	111	382
2561	2812	490	116	2901	835	2672
1562	1896	481	43	3742	2007	2472
1939	2073	756	81	1716	988	1041
4233	2077	1003	168	2103	660	2449
3305	1620	401	158	1268	1255	1628
1297	2676	615	137	1917	1288	1637
447	721	69	11	394	49	481
253	160	43	22	463	83	204
1463	1046	801	158	375	991	2321
892	1651	176	43	1159	357	1198
339	1657	115	19	962	450	1277
430	916	50	24	516	139	671
1412	2151	114	117	1805	550	1314
722	1093	162	33	1023	176	1219
2138	1361	662	276	1048	1071	1559
1020	540	450	210	379	532	1490
294	1046	112	5	501	159	354
563	1584	203	31	633	375	992
886	1147	155	15	3022	643	1094
1580	487	409	90	423	988	238
1295	1575	347	238	1196	593	967
1538	912	354	66	921	242	714

1692	668	606	257	446	743	867
1425	1187	228	33	616	697	859
706	521	80	22	1099	316	825
1520	847	319	228	646	794	1116
1322	780	341	67	526	571	923
296	6428	22	2	435	474	213
504	836	55	18	1648	250	272
1238	692	154	421	671	482	839
1672	666	660	389	477	668	1088
239	279	74	7	537	116	823
335	716	123	7	996	476	842
888	254	319	67	674	325	306
880	392	425	60	651	464	429
81	50	4	6	28	15	97
568	381	99	67	967	382	1112
549	466	63	2	572	212	1030
146	596	16	10	333	78	348
267	566	120	26	642	259	492
789	493	545	96	306	494	418
707	604	343	124	312	511	626
755	252	412	118	404	357	350
107	598	29	1	172	34	218
468	609	69	142	260	169	277
477	373	106	17	749	334	525
661	184	211	233	277	324	271
383	350	86	4	310	111	220
152	656	91	13	291	87	432
68	327	40	2	133	52	171
594	523	218	282	422	296	537
418	652	117	10	811	290	369
125	581	12	20	249	99	315
118	665	25	71	164	118	281
272	383	200	1	392	231	454
470	337	168	45	308	247	330
648	650	233	55	272	152	309
157	247	17	6	186	40	184
240	84	93	19	226	272	246
413	90	86	6	135	217	140
294	505	35	9	266	133	223
256	529	37	14	309	136	304
256	529	37	14	309	136	304
262	340	123	5	170	80	208
398	316	48	111	177	117	427
336	305	133	6	189	196	362
136	79	62	5	362	243	90
125	405	113	3	240	186	249
50	124	14	3	65	22	42
350	230	95	29	123	171	309
417	130	48	125	250	83	120
326	175	127	12	206	172	274
382	144	127	59	208	105	158
353	174	116	40	184	145	171
162	39	14	34	106	38	88
174	255	153	13	190	122	208
351	151	25	0	269	78	164
156	319	42	118	201	99	162
65	165	11	27	208	75	92
300	302	57	16	207	108	122
155	161	23	16	157	66	182

134	242	21	26	78	83	308
193	112	19	0	208	77	173
391	119	28	30	185	88	212
225	137	35	34	195	145	141
144	200	28	5	197	84	256
111	29	147	82	81	79	157
179	198	17	12	236	91	135
23	75	6	1	14	2	24
174	81	40	18	38	66	214
143	178	31	8	125	119	176
101	112	44	1	347	125	212
203	200	31	11	99	37	154
112	142	24	68	87	70	155
85	117	86	7	109	37	68
58	101	3	2	76	32	86
61	64	14	1	99	81	36
153	118	47	11	149	92	143
46	20	13	3	93	108	137
179	178	39	32	47	50	143
67	161	7	6	62	38	36
222	79	47	29	49	78	122
153	89	32	16	73	51	135
150	134	32	28	32	48	159
19	35	2	0	24	4	11
143	165	24	38	113	53	87
122	74	46	39	91	74	122
54	186	18	1	93	20	52
61	359	2	6	248	44	69
139	72	43	25	48	81	166
152	113	22	1	116	68	95
57	107	81	77	31	50	22
37	32	7	1	79	15	79
79	107	12	5	43	31	125
142	78	22	40	207	90	137
80	410	5	18	50	30	85
217	89	65	6	67	71	54
76	101	60	4	110	38	79
36	165	26	1	128	31	69
115	127	31	87	45	114	57
188	64	13	7	28	67	48
98	65	33	4	72	42	90
55	140	12	14	59	39	78
180	37	28	33	42	57	29
89	67	41	9	63	0	76
21	93	8	0	69	16	66
183	26	4	28	16	60	25
23	78	31	1	120	17	115
179	40	37	8	30	47	84
55	36	24	34	53	49	64
21	90	8	0	67	14	62
91	41	5	9	27	51	13
76	102	19	17	67	45	134
82	62	21	2	88	24	29
520	1	22	2	9	7	130
7	98	24	0	55	11	45
81	107	18	0	64	35	71
112	83	97	7	69	66	109
161	23	25	4	33	17	42
83	21	17	10	63	60	39

9	77	6	0	62	14	25
87	83	25	10	18	29	119
93	129	6	20	40	34	57
57	48	10	9	91	45	55
53	15	62	26	109	66	22
87	120	70	2	49	38	63
54	18	37	22	53	35	31
108	129	27	9	39	49	62
27	78	1	0	24	6	41
144	12	3	21	53	59	26
32	42	14	22	36	29	56
65	68	36	0	52	33	85
124	62	19	4	42	35	77
46	22	21	14	16	21	56
81	33	28	14	95	51	48
59	62	52	7	88	32	61
18	19	11	0	60	10	13
73	47	9	21	20	26	49
53	75	12	6	42	31	85
43	32	42	21	73	32	37
56	76	9	3	97	34	48
25	93	15	0	63	29	19
1	1	0	0	5	0	7
39	59	5	1	40	12	28
3	9	3	0	9	11	6
62	47	14	15	18	20	83
39	23	2	2	68	20	33
19	69	7	16	14	14	48
49	8	11	0	46	21	21
47	20	21	10	28	37	26
45	12	11	19	24	33	24
49	22	13	6	12	16	130
64	33	15	8	39	20	31
32	49	3	4	36	10	28
14	45	5	2	37	16	26
34	30	27	7	27	32	29
5	19	0	0	17	1	6
65	32	10	15	22	16	36
27	26	3	1	54	8	47
45	8	3	13	8	17	44
51	17	4	14	31	16	19
8	116	1	0	47	6	9
10	51	6	1	52	11	18
26	31	13	7	37	24	55
56	18	4	6	17	17	7
15	47	1	4	35	9	26
5	33	0	1	27	5	10
24	59	5	2	101	17	41
24	59	5	2	101	17	41
59	7	4	9	10	13	12
32	26	15	11	26	25	32
21	26	2	5	35	11	16
41	12	8	2	37	23	12
17	19	10	0	59	15	31
42	57	8	7	35	10	45
21	96	0	6	24	6	20
17	23	8	0	28	3	19
26	11	3	9	27	17	18
12	26	18	1	64	15	39

61	83	16	0	81	14	7
19	16	7	2	18	24	41
23	39	2	3	30	8	18
2	5	0	1	4	0	1
36	25	7	1	17	10	38
12	27	6	1	55	5	29
22	6	16	12	25	33	14
39	19	10	10	11	12	36
9	27	4	1	52	5	28
61	28	17	4	14	6	32
44	6	2	6	24	17	27
14	19	6	1	17	6	17
23	30	2	6	9	9	35
2	3	2	0	0	0	0
20	2	12	25	12	21	7
25	9	8	12	14	14	29
44	6	2	4	6	9	27
40	7	1	3	14	19	12
16	33	7	0	31	8	22
2	10	0	0	1	0	3
23	16	6	2	20	3	19
10	56	7	0	16	4	26
1	159	0	0	0	0	0
14	9	9	11	14	11	10
14	24	4	2	13	5	10
24	16	5	10	23	5	13
33	4	5	8	8	24	7
38	14	11	0	13	17	14
18	6	3	2	9	15	3
38	23	3	4	16	14	19
36	13	10	7	16	30	22
36	17	24	8	13	22	16
10	50	7	0	15	4	24
17	9	6	0	12	8	5
3	12	0	0	6	2	11
2	23	3	0	18	6	17
16	9	12	0	21	9	3
26	36	6	4	11	11	20
16	50	0	3	12	6	10
7	6	6	3	6	20	32
24	2	9	8	10	11	10
2	23	7	0	16	7	18
34	10	10	0	6	6	10
11	13	4	16	13	6	10
15	11	1	5	12	11	14
5	3	4	0	11	1	11
22	2	3	2	11	16	7
21	4	8	2	19	11	21
30	3	15	11	8	17	16
31	14	7	4	1	7	18
19	9	8	0	11	11	13
9	10	9	5	10	9	18
21	3	26	7	11	12	16
17	7	7	10	7	9	14
19	7	3	4	13	10	9
15	17	1	1	16	10	14
8	10	6	2	8	9	13
5	14	5	0	10	3	5
1	10	1	0	18	2	7

9	19	1	0	9	5	10
10	8	3	0	13	4	10
17	19	8	8	3	8	9
18	13	3	1	6	14	11
21	8	4	3	12	7	12
16	7	4	1	14	11	18
21	18	9	3	12	15	8
5	15	2	1	15	6	2
11	4	2	1	8	7	6
21	5	2	5	13	10	6
11	10	4	4	9	9	14
20	10	2	0	25	7	6
23	50	0	2	7	5	16
15	5	6	3	5	7	13
6	11	1	0	4	4	12
14	16	2	12	9	3	9
15	9	3	1	16	6	3
15	2	7	10	10	5	20
3	10	0	0	8	1	1
10	14	3	3	9	8	13
2	11	1	0	22	1	11
17	11	2	2	14	3	9
4	6	1	0	10	9	8
17	7	6	2	7	11	17
2	2	0	1	5	0	2
13	5	4	2	8	9	10
19	5	2	1	6	5	7
16	5	7	1	13	9	9
5	2	7	0	8	3	9
15	10	3	1	5	7	16
11	16	8	4	10	17	18
16	5	3	1	18	0	14
12	8	1	3	6	4	14
5	9	0	0	6	1	2
10	10	0	0	11	3	7
19	7	2	0	15	8	9
19	18	5	3	9	5	15
3	6	2	4	13	7	12
5	1	8	0	4	7	2
1	12	0	0	2	2	0
9	3	2	4	24	5	9
7	10	1	0	3	0	7
8	14	1	0	10	3	9
13	6	3	3	7	4	10
6	12	1	0	13	1	4
12	13	0	3	12	1	4
1	9	3	0	10	5	3
9	29	4	0	10	2	11
10	5	1	0	10	7	4
14	9	4	2	5	6	4
14	6	0	3	6	5	7
14	8	1	0	4	6	6
0	3	0	0	8	1	7
1	17	0	2	7	2	3
12	11	5	0	6	1	9
5	6	0	0	15	7	12
5	18	1	5	5	5	3
9	4	1	0	6	5	4
4	6	0	1	18	5	2

4	9	8	2	2	6	5
1	3	1	0	5	2	2
17	3	1	1	3	5	3
3	1	4	0	5	1	7
6	2	0	4	1	1	2
8	5	7	2	3	7	2
9	6	3	0	5	3	9
1	1	14	3	11	3	0
2	3	0	0	8	5	0
4	3	0	0	4	6	4
8	3	1	0	8	2	6
2	3	1	1	5	2	6
8	11	1	2	4	2	6
8	4	1	0	14	6	8
14	6	7	1	0	5	5
1	7	2	0	7	2	26
2	0	0	0	3	3	7
10	7	2	4	2	4	7
9	3	0	0	7	6	2
0	8	1	1	6	1	3
1	4	0	1	10	2	8
4	4	3	0	12	5	7
1	2	2	0	3	2	3
2	7	0	1	7	5	2
6	8	0	1	6	3	5
0	13	1	0	6	15	5
1	9	1	0	5	0	4
8	8	0	2	11	6	3
2	1	5	0	6	1	7
1	1	4	0	5	1	8
5	3	1	3	6	3	1
10	0	1	0	1	0	6
7	5	0	2	3	5	7
2	5	0	1	11	3	2
3	3	2	3	5	3	8
2	4	2	0	61	4	6
0	12	1	0	1	0	1
0	2	0	0	1	0	0
0	2	0	0	0	0	1
0	1	1	0	7	0	3
8	6	5	1	4	3	3
9	1	3	1	9	8	7
7	4	0	1	1	1	9
10	4	3	0	0	5	4
0	0	0	0	1	0	1
9	5	0	0	5	1	0
6	2	0	0	2	1	1
1	6	0	0	8	0	3
6	8	2	0	1	1	5
4	1	1	1	2	4	1
0	3	2	0	1	1	6
0	2	0	0	0	1	0
9	2	0	2	4	4	2
0	10	1	0	2	1	4
9	3	1	0	3	3	4
7	6	3	1	9	1	4
0	0	0	0	3	0	2
6	5	1	0	5	2	5
2	5	2	1	2	3	6

2	0	3	2	2	2	3
2	3	1	0	3	0	2
6	6	3	1	5	1	4
5	0	2	2	5	7	3
8	5	0	1	0	4	9
6	1	1	0	6	1	8
7	1	1	0	1	5	0
0	1	0	0	0	0	0
4	4	1	0	7	2	6
7	4	2	0	1	1	7
3	3	0	0	4	2	6
1	12	2	0	2	2	6
1	2	0	0	7	3	0
1	3	0	0	2	0	2
6	4	0	2	1	1	3
4	9	1	5	2	3	2
7	0	0	1	1	2	0
3	0	2	0	5	2	1
0	0	1	0	5	2	2
5	1	2	0	1	1	4
2	12	0	1	3	1	5
5	2	1	0	4	2	5
4	4	0	0	7	1	2
2	5	0	0	6	1	3
3	3	1	2	0	3	5
6	2	1	3	3	2	11
4	2	0	0	3	4	5
9	2	0	1	0	5	2
2	4	2	0	2	4	1
3	8	2	2	1	2	1
3	4	0	0	4	4	2
0	3	0	1	1	0	0
8	2	0	1	1	1	2
0	1	1	1	1	0	2
2	4	2	1	1	3	2
0	0	0	0	5	2	0
0	0	1	0	1	0	4
2	2	4	1	2	0	8
1	6	0	0	4	0	1
1	0	1	2	2	3	1
1	0	1	0	1	0	4
2	5	0	0	4	2	1
4	2	1	0	5	4	2
1	0	0	0	2	0	1
0	0	1	0	0	0	1
0	3	1	2	6	2	3
2	2	1	0	2	1	3
0	1	0	0	1	0	0
0	4	2	0	2	0	1
2	3	1	0	4	1	3
0	1	2	3	3	1	1
2	3	2	0	4	0	3
1	2	5	1	3	3	4
3	1	1	1	2	2	5
0	2	0	0	0	0	1
1	1	0	0	0	0	0
2	0	0	0	2	0	2
2	4	3	0	2	0	0
2	2	1	0	0	2	2

7	1	0	0	1	0	6
7	2	0	0	2	0	3
2	4	0	0	2	0	0
4	3	1	7	0	2	0
1	3	1	0	3	2	0
0	2	0	0	4	1	0
0	0	4	0	0	2	1
1	5	0	0	0	0	0
7	1	1	0	0	0	3
1	0	1	0	6	3	0
5	0	3	1	2	2	2
2	4	2	1	1	3	2
2	0	1	0	1	1	2
3	0	4	1	1	0	1
3	0	0	0	0	0	1
0	1	0	0	0	0	2
4	5	1	0	0	0	2
4	3	2	0	1	0	3
0	1	0	0	1	0	1
1	3	0	0	1	0	2
0	4	0	0	0	0	5
1	2	1	0	0	0	4
1	1	1	0	1	0	1
2	9	4	0	0	3	2
1	5	2	2	3	0	1
0	1	1	1	0	1	1
1	3	0	0	2	1	1
0	1	2	0	4	0	1
0	1	0	1	2	1	0
2	0	0	0	5	1	1
6	3	0	0	7	1	0
1	1	0	0	1	1	1
1	3	6	0	1	3	1
6	0	1	3	0	1	2
1	4	2	0	0	1	3
0	10	1	0	2	1	0
0	1	0	0	2	1	2
0	1	0	0	0	0	1

0.78	0.82	0.36	0.07	0.20	0.25	0.31
12	11	19	32	29	26	21
1	1	1	1	1	1	1
43	44	45	46	47	48	49
16	17	18	19	20	21	22
D_13	D_14	D_3	D_9	E_20	E_29	E_4
CHR_NP	CHR_NP	CHR_NP	CHR_NP	CHR_NP	CHR_NP	CHR_NP
2758841	2173631	852941	239935	1893208	1240451	2304775
CHR_NP	CHR_NP	CHR_NP	CHR_NP	CHR_NP	CHR_NP	CHR_NP
1230163	772506	380980	122679	389983	434200	899048
199880	185575	112586	4360	256917	87950	162314
229684	83193	98937	12343	135742	104686	168898
157501	74283	21941	2068	88459	83637	78820
246324	96288	60780	39570	121914	149724	164506

42920	105430	12798	1297	138924	19072	128682
86557	103298	17153	6148	189457	55740	89051
3400	36567	1632	453	18376	3407	35682
27028	56592	9145	1444	60543	20112	79855
37748	100568	10874	949	33423	16296	33869
35245	64172	3387	4340	14649	11862	19996
45989	21913	11262	2892	33311	26726	63887
25349	29263	2341	966	21311	11884	14690
31222	12564	6726	5265	10244	11251	23877
4748	17127	2018	146	12249	5069	11310
36283	14749	5776	4360	14452	19783	30744
4484	18325	1316	165	16723	3485	9674
15964	24721	4646	794	12614	9448	22251
10630	21944	3246	172	20440	4826	16228
19659	25876	7572	1368	15097	12160	9087
8759	13123	3804	832	10639	7806	8818
12074	15624	1878	295	6410	4200	9798
6338	30600	1503	89	11806	6426	5072
6183	13026	1523	74	30945	4146	12014
3848	4041	1720	181	12014	2145	3273
13498	11428	3012	491	10595	5810	12898
10138	4225	3424	3164	3971	6324	8727
2137	9154	844	225	4317	1897	9309
10180	5410	1580	306	11047	4712	9403
13760	10216	1876	1144	8071	6087	5189
4796	10179	3273	173	15625	5490	15629
7773	2541	1568	311	5170	5106	4727
2420	6848	432	211	8030	1759	4918
1060	13550	385	117	2224	1179	1443
9511	4939	1450	280	10370	4460	8887
9755	9678	1195	563	5536	3045	6668
5290	7562	2910	190	5926	3775	5773
6660	2609	1267	176	8080	1710	2449
9551	7122	3472	1015	3615	4264	3159
2286	837	6494	3700	3582	2940	2314
5644	5686	1881	2104	5684	4438	4198
6226	4133	2206	905	3620	3242	3567
6045	3709	913	840	4933	2476	5529
439	2491	342	29	1420	336	1352
3435	1792	1277	212	4902	2611	3671
4376	2161	1208	210	5572	3584	4328
5353	4293	505	243	4614	1861	2385
4920	3871	2530	744	1477	3362	4775
295	367	27	23	338	162	616
803	5082	435	51	3054	1345	1917
4985	1132	481	311	2097	2013	605
1978	5798	547	69	3858	1336	2036
3694	4232	836	855	1586	1244	2444
1468	3465	1250	17	2990	1379	1240
2379	3983	266	477	2996	1905	1145
150	1017	19	13	247	111	382
2561	2812	490	116	2901	835	2672
1562	1896	481	43	3742	2007	2472
1939	2073	756	81	1716	988	1041
4233	2077	1003	168	2103	660	2449
3305	1620	401	158	1268	1255	1628
1297	2676	615	137	1917	1288	1637
447	721	69	11	394	49	481
253	160	43	22	463	83	204

1463	1046	801	158	375	991	2321
892	1651	176	43	1159	357	1198
339	1657	115	19	962	450	1277
430	916	50	24	516	139	671
1412	2151	114	117	1805	550	1314
722	1093	162	33	1023	176	1219
2138	1361	662	276	1048	1071	1559
1020	540	450	210	379	532	1490
294	1046	112	5	501	159	354
563	1584	203	31	633	375	992
886	1147	155	15	3022	643	1094
1580	487	409	90	423	988	238
1295	1575	347	238	1196	593	967
1538	912	354	66	921	242	714
1692	668	606	257	446	743	867
1425	1187	228	33	616	697	859
706	521	80	22	1099	316	825
1520	847	319	228	646	794	1116
1322	780	341	67	526	571	923
296	6428	22	2	435	474	213
504	836	55	18	1648	250	272
1238	692	154	421	671	482	839
1672	666	660	389	477	668	1088
239	279	74	7	537	116	823
335	716	123	7	996	476	842
888	254	319	67	674	325	306
880	392	425	60	651	464	429
81	50	4	6	28	15	97
568	381	99	67	967	382	1112
549	466	63	2	572	212	1030
146	596	16	10	333	78	348
267	566	120	26	642	259	492
789	493	545	96	306	494	418
707	604	343	124	312	511	626
755	252	412	118	404	357	350
107	598	29	1	172	34	218
468	609	69	142	260	169	277
477	373	106	17	749	334	525
661	184	211	233	277	324	271
383	350	86	4	310	111	220
152	656	91	13	291	87	432
68	327	40	2	133	52	171
594	523	218	282	422	296	537
418	652	117	10	811	290	369
125	581	12	20	249	99	315
118	665	25	71	164	118	281
272	383	200	1	392	231	454
470	337	168	45	308	247	330
648	650	233	55	272	152	309
157	247	17	6	186	40	184
240	84	93	19	226	272	246
413	90	86	6	135	217	140
294	505	35	9	266	133	223
256	529	37	14	309	136	304
256	529	37	14	309	136	304
262	340	123	5	170	80	208
398	316	48	111	177	117	427
336	305	133	6	189	196	362
136	79	62	5	362	243	90

125	405	113	3	240	186	249
50	124	14	3	65	22	42
350	230	95	29	123	171	309
417	130	48	125	250	83	120
326	175	127	12	206	172	274
382	144	127	59	208	105	158
353	174	116	40	184	145	171
162	39	14	34	106	38	88
174	255	153	13	190	122	208
351	151	25	0	269	78	164
156	319	42	118	201	99	162
65	165	11	27	208	75	92
300	302	57	16	207	108	122
155	161	23	16	157	66	182
134	242	21	26	78	83	308
193	112	19	0	208	77	173
391	119	28	30	185	88	212
225	137	35	34	195	145	141
144	200	28	5	197	84	256
111	29	147	82	81	79	157
179	198	17	12	236	91	135
23	75	6	1	14	2	24
174	81	40	18	38	66	214
143	178	31	8	125	119	176
101	112	44	1	347	125	212
203	200	31	11	99	37	154
112	142	24	68	87	70	155
85	117	86	7	109	37	68
58	101	3	2	76	32	86
61	64	14	1	99	81	36
153	118	47	11	149	92	143
46	20	13	3	93	108	137
179	178	39	32	47	50	143
67	161	7	6	62	38	36
222	79	47	29	49	78	122
153	89	32	16	73	51	135
150	134	32	28	32	48	159
19	35	2	0	24	4	11
143	165	24	38	113	53	87
122	74	46	39	91	74	122
54	186	18	1	93	20	52
61	359	2	6	248	44	69
139	72	43	25	48	81	166
152	113	22	1	116	68	95
57	107	81	77	31	50	22
37	32	7	1	79	15	79
79	107	12	5	43	31	125
142	78	22	40	207	90	137
80	410	5	18	50	30	85
217	89	65	6	67	71	54
76	101	60	4	110	38	79
36	165	26	1	128	31	69
115	127	31	87	45	114	57
188	64	13	7	28	67	48
98	65	33	4	72	42	90
55	140	12	14	59	39	78
180	37	28	33	42	57	29
89	67	41	9	63	0	76
21	93	8	0	69	16	66

183	26	4	28	16	60	25
23	78	31	1	120	17	115
179	40	37	8	30	47	84
55	36	24	34	53	49	64
21	90	8	0	67	14	62
91	41	5	9	27	51	13
76	102	19	17	67	45	134
82	62	21	2	88	24	29
520	1	22	2	9	7	130
7	98	24	0	55	11	45
81	107	18	0	64	35	71
112	83	97	7	69	66	109
161	23	25	4	33	17	42
83	21	17	10	63	60	39
9	77	6	0	62	14	25
87	83	25	10	18	29	119
93	129	6	20	40	34	57
57	48	10	9	91	45	55
53	15	62	26	109	66	22
87	120	70	2	49	38	63
54	18	37	22	53	35	31
108	129	27	9	39	49	62
27	78	1	0	24	6	41
144	12	3	21	53	59	26
32	42	14	22	36	29	56
65	68	36	0	52	33	85
124	62	19	4	42	35	77
46	22	21	14	16	21	56
81	33	28	14	95	51	48
59	62	52	7	88	32	61
18	19	11	0	60	10	13
73	47	9	21	20	26	49
53	75	12	6	42	31	85
43	32	42	21	73	32	37
56	76	9	3	97	34	48
25	93	15	0	63	29	19
1	1	0	0	5	0	7
39	59	5	1	40	12	28
3	9	3	0	9	11	6
62	47	14	15	18	20	83
39	23	2	2	68	20	33
19	69	7	16	14	14	48
49	8	11	0	46	21	21
47	20	21	10	28	37	26
45	12	11	19	24	33	24
49	22	13	6	12	16	130
64	33	15	8	39	20	31
32	49	3	4	36	10	28
14	45	5	2	37	16	26
34	30	27	7	27	32	29
5	19	0	0	17	1	6
65	32	10	15	22	16	36
27	26	3	1	54	8	47
45	8	3	13	8	17	44
51	17	4	14	31	16	19
8	116	1	0	47	6	9
10	51	6	1	52	11	18
26	31	13	7	37	24	55
56	18	4	6	17	17	7

15	47	1	4	35	9	26
5	33	0	1	27	5	10
24	59	5	2	101	17	41
24	59	5	2	101	17	41
59	7	4	9	10	13	12
32	26	15	11	26	25	32
21	26	2	5	35	11	16
41	12	8	2	37	23	12
17	19	10	0	59	15	31
42	57	8	7	35	10	45
21	96	0	6	24	6	20
17	23	8	0	28	3	19
26	11	3	9	27	17	18
12	26	18	1	64	15	39
61	83	16	0	81	14	7
19	16	7	2	18	24	41
23	39	2	3	30	8	18
2	5	0	1	4	0	1
36	25	7	1	17	10	38
12	27	6	1	55	5	29
22	6	16	12	25	33	14
39	19	10	10	11	12	36
9	27	4	1	52	5	28
61	28	17	4	14	6	32
44	6	2	6	24	17	27
14	19	6	1	17	6	17
23	30	2	6	9	9	35
2	3	2	0	0	0	0
20	2	12	25	12	21	7
25	9	8	12	14	14	29
44	6	2	4	6	9	27
40	7	1	3	14	19	12
16	33	7	0	31	8	22
2	10	0	0	1	0	3
23	16	6	2	20	3	19
10	56	7	0	16	4	26
1	159	0	0	0	0	0
14	9	9	11	14	11	10
14	24	4	2	13	5	10
24	16	5	10	23	5	13
33	4	5	8	8	24	7
38	14	11	0	13	17	14
18	6	3	2	9	15	3
38	23	3	4	16	14	19
36	13	10	7	16	30	22
36	17	24	8	13	22	16
10	50	7	0	15	4	24
17	9	6	0	12	8	5
3	12	0	0	6	2	11
2	23	3	0	18	6	17
16	9	12	0	21	9	3
26	36	6	4	11	11	20
16	50	0	3	12	6	10
7	6	6	3	6	20	32
24	2	9	8	10	11	10
2	23	7	0	16	7	18
34	10	10	0	6	6	10
11	13	4	16	13	6	10
15	11	1	5	12	11	14

5	3	4	0	11	1	11
22	2	3	2	11	16	7
21	4	8	2	19	11	21
30	3	15	11	8	17	16
31	14	7	4	1	7	18
19	9	8	0	11	11	13
9	10	9	5	10	9	18
21	3	26	7	11	12	16
17	7	7	10	7	9	14
19	7	3	4	13	10	9
15	17	1	1	16	10	14
8	10	6	2	8	9	13
5	14	5	0	10	3	5
1	10	1	0	18	2	7
9	19	1	0	9	5	10
10	8	3	0	13	4	10
17	19	8	8	3	8	9
18	13	3	1	6	14	11
21	8	4	3	12	7	12
16	7	4	1	14	11	18
21	18	9	3	12	15	8
5	15	2	1	15	6	2
11	4	2	1	8	7	6
21	5	2	5	13	10	6
11	10	4	4	9	9	14
20	10	2	0	25	7	6
23	50	0	2	7	5	16
15	5	6	3	5	7	13
6	11	1	0	4	4	12
14	16	2	12	9	3	9
15	9	3	1	16	6	3
15	2	7	10	10	5	20
3	10	0	0	8	1	1
10	14	3	3	9	8	13
2	11	1	0	22	1	11
17	11	2	2	14	3	9
4	6	1	0	10	9	8
17	7	6	2	7	11	17
2	2	0	1	5	0	2
13	5	4	2	8	9	10
19	5	2	1	6	5	7
16	5	7	1	13	9	9
5	2	7	0	8	3	9
15	10	3	1	5	7	16
11	16	8	4	10	17	18
16	5	3	1	18	0	14
12	8	1	3	6	4	14
5	9	0	0	6	1	2
10	10	0	0	11	3	7
19	7	2	0	15	8	9
19	18	5	3	9	5	15
3	6	2	4	13	7	12
5	1	8	0	4	7	2
1	12	0	0	2	2	0
9	3	2	4	24	5	9
7	10	1	0	3	0	7
8	14	1	0	10	3	9
13	6	3	3	7	4	10
6	12	1	0	13	1	4

12	13	0	3	12	1	4
1	9	3	0	10	5	3
9	29	4	0	10	2	11
10	5	1	0	10	7	4
14	9	4	2	5	6	4
14	6	0	3	6	5	7
14	8	1	0	4	6	6
0	3	0	0	8	1	7
1	17	0	2	7	2	3
12	11	5	0	6	1	9
5	6	0	0	15	7	12
5	18	1	5	5	5	3
9	4	1	0	6	5	4
4	6	0	1	18	5	2
4	9	8	2	2	6	5
1	3	1	0	5	2	2
17	3	1	1	3	5	3
3	1	4	0	5	1	7
6	2	0	4	1	1	2
8	5	7	2	3	7	2
9	6	3	0	5	3	9
1	1	14	3	11	3	0
2	3	0	0	8	5	0
4	3	0	0	4	6	4
8	3	1	0	8	2	6
2	3	1	1	5	2	6
8	11	1	2	4	2	6
8	4	1	0	14	6	8
14	6	7	1	0	5	5
1	7	2	0	7	2	26
2	0	0	0	3	3	7
10	7	2	4	2	4	7
9	3	0	0	7	6	2
0	8	1	1	6	1	3
1	4	0	1	10	2	8
4	4	3	0	12	5	7
1	2	2	0	3	2	3
2	7	0	1	7	5	2
6	8	0	1	6	3	5
0	13	1	0	6	15	5
1	9	1	0	5	0	4
8	8	0	2	11	6	3
2	1	5	0	6	1	7
1	1	4	0	5	1	8
5	3	1	3	6	3	1
10	0	1	0	1	0	6
7	5	0	2	3	5	7
2	5	0	1	11	3	2
3	3	2	3	5	3	8
2	4	2	0	61	4	6
0	12	1	0	1	0	1
0	2	0	0	1	0	0
0	2	0	0	0	0	1
0	1	1	0	7	0	3
8	6	5	1	4	3	3
9	1	3	1	9	8	7
7	4	0	1	1	1	9
10	4	3	0	0	5	4
0	0	0	0	1	0	1

9	5	0	0	5	1	0
6	2	0	0	2	1	1
1	6	0	0	8	0	3
6	8	2	0	1	1	5
4	1	1	1	2	4	1
0	3	2	0	1	1	6
0	2	0	0	0	1	0
9	2	0	2	4	4	2
0	10	1	0	2	1	4
9	3	1	0	3	3	4
7	6	3	1	9	1	4
0	0	0	0	3	0	2
6	5	1	0	5	2	5
2	5	2	1	2	3	6
2	0	3	2	2	2	3
2	3	1	0	3	0	2
6	6	3	1	5	1	4
5	0	2	2	5	7	3
8	5	0	1	0	4	9
6	1	1	0	6	1	8
7	1	1	0	1	5	0
0	1	0	0	0	0	0
4	4	1	0	7	2	6
7	4	2	0	1	1	7
3	3	0	0	4	2	6
1	12	2	0	2	2	6
1	2	0	0	7	3	0
1	3	0	0	2	0	2
6	4	0	2	1	1	3
4	9	1	5	2	3	2
7	0	0	1	1	2	0
3	0	2	0	5	2	1
0	0	1	0	5	2	2
5	1	2	0	1	1	4
2	12	0	1	3	1	5
5	2	1	0	4	2	5
4	4	0	0	7	1	2
2	5	0	0	6	1	3
3	3	1	2	0	3	5
6	2	1	3	3	2	11
4	2	0	0	3	4	5
9	2	0	1	0	5	2
2	4	2	0	2	4	1
3	8	2	2	1	2	1
3	4	0	0	4	4	2
0	3	0	1	1	0	0
8	2	0	1	1	1	2
0	1	1	1	1	0	2
2	4	2	1	1	3	2
0	0	0	0	5	2	0
0	0	1	0	1	0	4
2	2	4	1	2	0	8
1	6	0	0	4	0	1
1	0	1	2	2	3	1
1	0	1	0	1	0	4
2	5	0	0	4	2	1
4	2	1	0	5	4	2
1	0	0	0	2	0	1
0	0	1	0	0	0	1

0	3	1	2	6	2	3
2	2	1	0	2	1	3
0	1	0	0	1	0	0
0	4	2	0	2	0	1
2	3	1	0	4	1	3
0	1	2	3	3	1	1
2	3	2	0	4	0	3
1	2	5	1	3	3	4
3	1	1	1	2	2	5
0	2	0	0	0	0	1
1	1	0	0	0	0	0
2	0	0	0	2	0	2
2	4	3	0	2	0	0
2	2	1	0	0	2	2
7	1	0	0	1	0	6
7	2	0	0	2	0	3
2	4	0	0	2	0	0
4	3	1	7	0	2	0
1	3	1	0	3	2	0
0	2	0	0	4	1	0
0	0	4	0	0	2	1
1	5	0	0	0	0	0
7	1	1	0	0	0	3
1	0	1	0	6	3	0
5	0	3	1	2	2	2
2	4	2	1	1	3	2
2	0	1	0	1	1	2
3	0	4	1	1	0	1
3	0	0	0	0	0	1
0	1	0	0	0	0	2
4	5	1	0	0	0	2
4	3	2	0	1	0	3
0	1	0	0	1	0	1
1	3	0	0	1	0	2
0	4	0	0	0	0	5
1	2	1	0	0	0	4
1	1	1	0	1	0	1
2	9	4	0	0	3	2
1	5	2	2	3	0	1
0	1	1	1	0	1	1
1	3	0	0	2	1	1
0	1	2	0	4	0	1
0	1	0	1	2	1	0
2	0	0	0	5	1	1
6	3	0	0	7	1	0
1	1	0	0	1	1	1
1	3	6	0	1	3	1
6	0	1	3	0	1	2
1	4	2	0	0	1	3
0	10	1	0	2	1	0
0	1	0	0	2	1	2
0	1	0	0	0	0	1
85923	66060	26543	7286	57155	38029	71566
226632	140020	72322	22993	88391	82940	164437
362	363	428	460	375	404	369
CHR_NP	CHR_NP	CHR_NP	CHR_NP	CHR_NP	CHR_NP	CHR_NP
CHR_NP	CHR_NP	CHR_NP	CHR_NP	CHR_NP	CHR_NP	CHR_NP

43	44	45	46	47	48	49
16	17	18	19	20	21	22
CHR_NP	CHR_NP	CHR_NP	CHR_NP	CHR_NP	CHR_NP	CHR_NP
D_13	D_14	D_3	D_9	E_20	E_29	E_4
2758841	2173631	852941	239935	1893208	1240451	2304775
CHR_NP	CHR_NP	CHR_NP	CHR_NP	CHR_NP	CHR_NP	CHR_NP
14.32	11.69	14.35	16.84	6.82	11.42	12.56
2.33	2.81	4.24	0.60	4.50	2.31	2.27
2.67	1.26	3.73	1.69	2.37	2.75	2.36
1.83	1.12	0.83	0.28	1.55	2.20	1.10
2.87	1.46	2.29	5.43	2.13	3.94	2.30
0.50	1.60	0.48	0.18	2.43	0.50	1.80
1.01	1.56	0.65	0.84	3.31	1.47	1.24
0.04	0.55	0.06	0.06	0.32	0.09	0.50
0.31	0.86	0.34	0.20	1.06	0.53	1.12
0.44	1.52	0.41	0.13	0.58	0.43	0.47
0.41	0.97	0.13	0.60	0.26	0.31	0.28
0.54	0.33	0.42	0.40	0.58	0.70	0.89
0.30	0.44	0.09	0.13	0.37	0.31	0.21
0.36	0.19	0.25	0.72	0.18	0.30	0.33
0.06	0.26	0.08	0.02	0.21	0.13	0.16
0.42	0.22	0.22	0.60	0.25	0.52	0.43
0.05	0.28	0.05	0.02	0.29	0.09	0.14
0.19	0.37	0.18	0.11	0.22	0.25	0.31
0.12	0.33	0.12	0.02	0.36	0.13	0.23
0.23	0.39	0.29	0.19	0.26	0.32	0.13
0.10	0.20	0.14	0.11	0.19	0.21	0.12
0.14	0.24	0.07	0.04	0.11	0.11	0.14
0.07	0.46	0.06	0.01	0.21	0.17	0.07
0.07	0.20	0.06	0.01	0.54	0.11	0.17
0.04	0.06	0.06	0.02	0.21	0.06	0.05
0.16	0.17	0.11	0.07	0.19	0.15	0.18
0.12	0.06	0.13	0.43	0.07	0.17	0.12
0.02	0.14	0.03	0.03	0.08	0.05	0.13
0.12	0.08	0.06	0.04	0.19	0.12	0.13
0.16	0.15	0.07	0.16	0.14	0.16	0.07
0.06	0.15	0.12	0.02	0.27	0.14	0.22
0.09	0.04	0.06	0.04	0.09	0.13	0.07
0.03	0.10	0.02	0.03	0.14	0.05	0.07
0.01	0.21	0.01	0.02	0.04	0.03	0.02
0.11	0.07	0.05	0.04	0.18	0.12	0.12
0.11	0.15	0.05	0.08	0.10	0.08	0.09
0.06	0.11	0.11	0.03	0.10	0.10	0.08
0.08	0.04	0.05	0.02	0.14	0.04	0.03
0.11	0.11	0.13	0.14	0.06	0.11	0.04
0.03	0.01	0.24	0.51	0.06	0.08	0.03
0.07	0.09	0.07	0.29	0.10	0.12	0.06
0.07	0.06	0.08	0.12	0.06	0.09	0.05
0.07	0.06	0.03	0.12	0.09	0.07	0.08
0.01	0.04	0.01	0.00	0.02	0.01	0.02
0.04	0.03	0.05	0.03	0.09	0.07	0.05
0.05	0.03	0.05	0.03	0.10	0.09	0.06
0.06	0.06	0.02	0.03	0.08	0.05	0.03
0.06	0.06	0.10	0.10	0.03	0.09	0.07
0.00	0.01	0.00	0.00	0.01	0.00	0.01
0.01	0.08	0.02	0.01	0.05	0.04	0.03
0.06	0.02	0.02	0.04	0.04	0.05	0.01
0.02	0.09	0.02	0.01	0.07	0.04	0.03

0.04	0.06	0.03	0.12	0.03	0.03	0.03
0.02	0.05	0.05	0.00	0.05	0.04	0.02
0.03	0.06	0.01	0.07	0.05	0.05	0.02
0.00	0.02	0.00	0.00	0.00	0.00	0.01
0.03	0.04	0.02	0.02	0.05	0.02	0.04
0.02	0.03	0.02	0.01	0.07	0.05	0.03
0.02	0.03	0.03	0.01	0.03	0.03	0.01
0.05	0.03	0.04	0.02	0.04	0.02	0.03
0.04	0.02	0.02	0.02	0.02	0.03	0.02
0.02	0.04	0.02	0.02	0.03	0.03	0.02
0.01	0.01	0.00	0.00	0.01	0.00	0.01
0.00	0.00	0.00	0.00	0.01	0.00	0.00
0.02	0.02	0.03	0.02	0.01	0.03	0.03
0.01	0.02	0.01	0.01	0.02	0.01	0.02
0.00	0.03	0.00	0.00	0.02	0.01	0.02
0.01	0.01	0.00	0.00	0.01	0.00	0.01
0.02	0.03	0.00	0.02	0.03	0.01	0.02
0.01	0.02	0.01	0.00	0.02	0.00	0.02
0.02	0.02	0.02	0.04	0.02	0.03	0.02
0.01	0.01	0.02	0.03	0.01	0.01	0.02
0.00	0.02	0.00	0.00	0.01	0.00	0.00
0.01	0.02	0.01	0.00	0.01	0.01	0.01
0.01	0.02	0.01	0.00	0.05	0.02	0.02
0.02	0.01	0.02	0.01	0.01	0.03	0.00
0.02	0.02	0.01	0.03	0.02	0.02	0.01
0.02	0.01	0.01	0.01	0.02	0.01	0.01
0.02	0.01	0.02	0.04	0.01	0.02	0.01
0.02	0.02	0.01	0.00	0.01	0.02	0.01
0.01	0.01	0.00	0.00	0.02	0.01	0.01
0.02	0.01	0.01	0.03	0.01	0.02	0.02
0.02	0.01	0.01	0.01	0.01	0.02	0.01
0.00	0.10	0.00	0.00	0.01	0.01	0.00
0.01	0.01	0.00	0.00	0.03	0.01	0.00
0.01	0.01	0.01	0.06	0.01	0.01	0.01
0.02	0.01	0.02	0.05	0.01	0.02	0.02
0.00	0.00	0.00	0.00	0.01	0.00	0.01
0.00	0.01	0.00	0.00	0.02	0.01	0.01
0.01	0.00	0.01	0.01	0.01	0.01	0.00
0.01	0.01	0.02	0.01	0.01	0.01	0.01
0.00	0.00	0.00	0.00	0.00	0.00	0.00
0.01	0.01	0.00	0.01	0.02	0.01	0.02
0.01	0.01	0.00	0.00	0.01	0.01	0.01
0.00	0.01	0.00	0.00	0.01	0.00	0.00
0.00	0.01	0.00	0.00	0.01	0.01	0.01
0.01	0.01	0.02	0.01	0.01	0.01	0.01
0.01	0.01	0.01	0.02	0.01	0.01	0.01
0.01	0.00	0.02	0.02	0.01	0.01	0.00
0.00	0.01	0.00	0.00	0.00	0.00	0.00
0.01	0.01	0.00	0.02	0.00	0.00	0.00
0.01	0.01	0.00	0.00	0.01	0.01	0.01
0.01	0.00	0.01	0.03	0.00	0.01	0.00
0.00	0.01	0.00	0.00	0.01	0.00	0.00
0.00	0.01	0.00	0.00	0.01	0.00	0.01
0.00	0.00	0.00	0.00	0.00	0.00	0.00
0.01	0.01	0.01	0.04	0.01	0.01	0.01
0.00	0.01	0.00	0.00	0.01	0.01	0.01
0.00	0.01	0.00	0.00	0.00	0.00	0.00
0.00	0.01	0.00	0.01	0.00	0.00	0.00
0.00	0.01	0.01	0.00	0.01	0.01	0.01

0.01	0.01	0.01	0.01	0.01	0.01	0.00
0.01	0.01	0.01	0.01	0.00	0.00	0.00
0.00	0.00	0.00	0.00	0.00	0.00	0.00
0.00	0.00	0.00	0.00	0.00	0.01	0.00
0.00	0.00	0.00	0.00	0.00	0.01	0.00
0.00	0.01	0.00	0.00	0.00	0.00	0.00
0.00	0.01	0.00	0.00	0.01	0.00	0.00
0.00	0.01	0.00	0.00	0.01	0.00	0.00
0.00	0.01	0.00	0.00	0.00	0.00	0.00
0.00	0.00	0.00	0.02	0.00	0.00	0.01
0.00	0.00	0.01	0.00	0.00	0.01	0.01
0.00	0.00	0.00	0.00	0.01	0.01	0.00
0.00	0.01	0.00	0.00	0.00	0.00	0.00
0.00	0.00	0.00	0.00	0.00	0.00	0.00
0.00	0.00	0.00	0.00	0.00	0.00	0.00
0.00	0.00	0.00	0.02	0.00	0.00	0.00
0.00	0.00	0.00	0.00	0.00	0.00	0.00
0.00	0.00	0.00	0.01	0.00	0.00	0.00
0.00	0.00	0.00	0.01	0.00	0.00	0.00
0.00	0.00	0.00	0.00	0.00	0.00	0.00
0.00	0.00	0.01	0.00	0.00	0.00	0.00
0.00	0.00	0.00	0.00	0.00	0.00	0.00
0.00	0.00	0.00	0.02	0.00	0.00	0.00
0.00	0.00	0.00	0.00	0.00	0.00	0.00
0.00	0.00	0.00	0.00	0.00	0.00	0.00
0.00	0.00	0.00	0.00	0.00	0.00	0.00
0.00	0.00	0.00	0.00	0.00	0.00	0.00
0.00	0.00	0.00	0.00	0.00	0.00	0.00
0.00	0.00	0.00	0.00	0.00	0.00	0.00
0.00	0.00	0.00	0.00	0.00	0.00	0.00
0.00	0.00	0.00	0.00	0.00	0.00	0.00
0.00	0.00	0.01	0.01	0.00	0.00	0.00
0.00	0.00	0.00	0.00	0.00	0.00	0.00
0.00	0.00	0.00	0.00	0.00	0.00	0.00
0.00	0.00	0.00	0.00	0.00	0.00	0.00
0.00	0.00	0.00	0.00	0.00	0.00	0.00
0.00	0.00	0.00	0.00	0.01	0.00	0.00
0.00	0.00	0.00	0.00	0.00	0.00	0.00
0.00	0.00	0.00	0.01	0.00	0.00	0.00
0.00	0.00	0.00	0.00	0.00	0.00	0.00
0.00	0.00	0.00	0.00	0.00	0.00	0.00
0.00	0.00	0.00	0.00	0.00	0.00	0.00
0.00	0.00	0.00	0.00	0.00	0.00	0.00
0.00	0.00	0.00	0.00	0.00	0.00	0.00
0.00	0.00	0.00	0.00	0.00	0.00	0.00
0.00	0.00	0.00	0.00	0.00	0.00	0.00
0.00	0.00	0.00	0.00	0.00	0.00	0.00
0.00	0.00	0.00	0.00	0.00	0.00	0.00
0.00	0.00	0.00	0.00	0.00	0.00	0.00
0.00	0.00	0.00	0.00	0.00	0.00	0.00
0.00	0.00	0.00	0.01	0.00	0.00	0.00
0.00	0.00	0.00	0.01	0.00	0.00	0.00
0.00	0.00	0.00	0.00	0.00	0.00	0.00
0.00	0.01	0.00	0.00	0.00	0.00	0.00
0.00	0.00	0.00	0.00	0.00	0.00	0.00
0.00	0.00	0.00	0.00	0.00	0.00	0.00
0.00	0.00	0.00	0.01	0.00	0.00	0.00
0.00	0.00	0.00	0.00	0.00	0.00	0.00
0.00	0.00	0.00	0.00	0.00	0.00	0.00

0.00	0.00	0.00	0.01	0.00	0.00	0.00
0.00	0.01	0.00	0.00	0.00	0.00	0.00
0.00	0.00	0.00	0.00	0.00	0.00	0.00
0.00	0.00	0.00	0.00	0.00	0.00	0.00
0.00	0.00	0.00	0.00	0.00	0.00	0.00
0.00	0.00	0.00	0.01	0.00	0.00	0.00
0.00	0.00	0.00	0.00	0.00	0.00	0.00
0.00	0.00	0.00	0.00	0.00	0.00	0.00
0.00	0.00	0.00	0.00	0.00	0.00	0.00
0.00	0.00	0.00	0.00	0.00	0.00	0.00
0.00	0.00	0.00	0.00	0.00	0.00	0.00
0.00	0.00	0.00	0.00	0.00	0.00	0.00
0.00	0.00	0.00	0.00	0.00	0.00	0.00
0.00	0.00	0.00	0.00	0.00	0.00	0.00
0.00	0.00	0.00	0.00	0.00	0.00	0.00
0.00	0.00	0.00	0.00	0.00	0.00	0.00
0.00	0.00	0.00	0.00	0.00	0.00	0.00
0.00	0.00	0.00	0.00	0.00	0.00	0.00
0.00	0.00	0.00	0.00	0.00	0.00	0.00
0.00	0.00	0.00	0.00	0.00	0.00	0.00
0.01	0.00	0.00	0.00	0.00	0.00	0.00
0.00	0.00	0.00	0.00	0.00	0.00	0.00
0.00	0.00	0.00	0.00	0.00	0.00	0.00
0.00	0.00	0.00	0.00	0.00	0.00	0.00
0.00	0.00	0.00	0.00	0.00	0.00	0.00
0.00	0.00	0.00	0.00	0.00	0.00	0.00
0.00	0.00	0.00	0.00	0.00	0.00	0.00
0.00	0.00	0.00	0.00	0.00	0.00	0.00
0.00	0.00	0.00	0.00	0.00	0.00	0.00
0.00	0.00	0.00	0.00	0.00	0.00	0.00
0.00	0.00	0.00	0.00	0.00	0.00	0.00
0.00	0.00	0.00	0.00	0.00	0.00	0.00
0.00	0.00	0.00	0.00	0.00	0.00	0.00
0.00	0.00	0.00	0.00	0.00	0.00	0.00
0.00	0.00	0.00	0.00	0.00	0.00	0.00
0.00	0.00	0.00	0.00	0.00	0.00	0.00
0.00	0.00	0.00	0.00	0.00	0.00	0.00
0.00	0.00	0.00	0.00	0.00	0.00	0.00
0.00	0.00	0.00	0.00	0.00	0.00	0.00
0.00	0.00	0.00	0.00	0.00	0.00	0.00
0.00	0.00	0.00	0.00	0.00	0.00	0.00
0.00	0.00	0.00	0.00	0.00	0.00	0.00
0.00	0.00	0.00	0.00	0.00	0.00	0.00
0.00	0.00	0.00	0.00	0.00	0.00	0.00
0.00	0.00	0.00	0.00	0.00	0.00	0.00
0.00	0.00	0.00	0.00	0.00	0.00	0.00
0.00	0.00	0.00	0.00	0.00	0.00	0.00
0.00	0.00	0.00	0.00	0.00	0.00	0.00
0.00	0.00	0.00	0.00	0.00	0.00	0.00
0.00	0.00	0.00	0.00	0.00	0.00	0.00
0.00	0.00	0.00	0.00	0.00	0.00	0.00
0.00	0.00	0.00	0.00	0.00	0.00	0.00
0.00	0.00	0.00	0.00	0.00	0.00	0.00
0.00	0.00	0.00	0.00	0.00	0.00	0.00
0.00	0.00	0.00	0.00	0.00	0.00	0.00
0.00	0.00	0.00	0.00	0.00	0.00	0.00
0.00	0.00	0.00	0.00	0.00	0.00	0.00
0.00	0.00	0.00	0.00	0.00	0.00	0.00
0.00	0.00	0.00	0.00	0.00	0.00	0.00

0.00	0.00	0.00	0.00	0.00	0.00	0.00
0.00	0.00	0.00	0.00	0.00	0.00	0.00
0.00	0.00	0.00	0.00	0.00	0.00	0.00
0.00	0.00	0.00	0.00	0.00	0.00	0.00
0.00	0.00	0.00	0.00	0.00	0.00	0.00
0.00	0.00	0.00	0.00	0.00	0.00	0.00
0.00	0.00	0.00	0.00	0.00	0.00	0.00
0.00	0.00	0.00	0.00	0.00	0.00	0.00
0.00	0.00	0.00	0.00	0.00	0.00	0.00
0.00	0.00	0.00	0.00	0.00	0.00	0.00
0.00	0.00	0.00	0.00	0.00	0.00	0.00
0.00	0.00	0.00	0.00	0.00	0.00	0.00
0.00	0.00	0.00	0.00	0.00	0.00	0.00
0.00	0.00	0.00	0.00	0.00	0.00	0.00
0.00	0.00	0.00	0.00	0.00	0.00	0.00
0.00	0.00	0.00	0.00	0.00	0.00	0.00
0.00	0.00	0.00	0.00	0.00	0.00	0.00
0.00	0.00	0.00	0.00	0.00	0.00	0.00
0.00	0.00	0.00	0.00	0.00	0.00	0.00
0.00	0.00	0.00	0.00	0.00	0.00	0.00
0.00	0.00	0.00	0.00	0.00	0.00	0.00
0.00	0.00	0.00	0.00	0.00	0.00	0.00
0.00	0.00	0.00	0.00	0.00	0.00	0.00
0.00	0.00	0.00	0.00	0.00	0.00	0.00
0.00	0.00	0.00	0.00	0.00	0.00	0.00
0.00	0.00	0.00	0.00	0.00	0.00	0.00
0.00	0.00	0.00	0.00	0.00	0.00	0.00
0.00	0.00	0.00	0.00	0.00	0.00	0.00
0.00	0.00	0.00	0.00	0.00	0.00	0.00
0.00	0.00	0.00	0.00	0.00	0.00	0.00

43 44 45 46 47 48 49

16	17	18	19	20	21	22
CHR_NP	CHR_NP	CHR_NP	CHR_NP	CHR_NP	CHR_NP	CHR_NP
D_13	D_14	D_3	D_9	E_20	E_29	E_4
2758841	2173631	852941	239935	1893208	1240451	2304775
D_13	D_14	D_3	D_9	E_20	E_29	E_4
0.61	0.02	0.61	1.17	-1.08	-0.05	0.21
-0.28	-0.14	0.28	-0.78	0.36	-0.28	-0.29
0.42	-0.64	1.22	-0.31	0.20	0.48	0.19
-0.61	-1.31	-1.60	-2.14	-0.89	-0.25	-1.33
0.78	-0.78	0.14	3.61	-0.03	1.96	0.15
-0.63	0.37	-0.64	-0.92	1.12	-0.62	0.55
-0.25	0.63	-0.83	-0.51	3.42	0.48	0.12
-0.55	0.38	-0.51	-0.51	-0.04	-0.46	0.28

-0.55	0.53	-0.49	-0.78	0.93	-0.12	1.04
-0.82	1.96	-0.90	-1.61	-0.45	-0.85	-0.73
-0.23	1.88	-1.29	0.47	-0.81	-0.60	-0.72
0.39	-1.43	-0.60	-0.85	0.82	1.89	3.59
-0.36	0.49	-1.55	-1.30	0.09	-0.26	-0.88
-0.60	-1.59	-1.23	1.47	-1.66	-0.98	-0.77
-0.75	0.18	-0.66	-0.92	-0.02	-0.40	-0.28
0.55	-0.32	-0.34	1.32	-0.19	0.98	0.58
-0.67	0.76	-0.68	-0.85	0.85	-0.42	-0.14
-0.28	1.57	-0.39	-1.04	0.06	0.33	0.95
-0.52	1.34	-0.53	-1.41	1.57	-0.49	0.40
-0.02	1.61	0.55	-0.43	0.34	0.89	-1.03
-0.89	0.67	-0.22	-0.69	0.47	0.77	-0.55
-0.17	1.30	-1.23	-1.70	-0.60	-0.63	-0.22
-0.56	4.26	-0.77	-1.32	1.09	0.62	-0.59
-0.45	0.74	-0.58	-1.03	3.98	-0.10	0.46
-0.50	-0.41	-0.39	-0.62	0.48	-0.44	-0.50
0.79	1.09	-0.03	-0.90	1.33	0.71	1.23
-0.32	-1.18	-0.15	4.71	-1.09	0.45	-0.26
-0.84	0.25	-0.78	-0.79	-0.36	-0.60	0.17
0.22	-0.49	-0.93	-1.27	1.67	0.32	0.47
1.36	1.23	-0.73	1.28	0.91	1.35	-0.69
-0.81	1.48	0.76	-1.56	4.26	1.25	2.98
-0.61	-1.62	-1.22	-1.54	-0.61	0.24	-1.08
-0.47	0.67	-0.65	-0.46	1.22	-0.20	0.14
-0.33	0.72	-0.32	-0.31	-0.19	-0.23	-0.29
0.36	-0.44	-0.89	-1.26	1.95	0.51	0.66
-0.18	0.02	-0.60	-0.40	-0.28	-0.39	-0.31
-0.61	0.01	-0.04	-1.03	-0.11	-0.17	-0.39
-0.15	-0.54	-0.46	-0.70	0.50	-0.49	-0.60
0.14	0.10	0.38	0.48	-0.43	0.15	-0.66
-0.59	-0.70	1.17	3.29	-0.30	-0.18	-0.54
-0.21	0.21	-0.10	4.35	0.48	0.83	-0.35
0.12	-0.18	0.44	1.67	-0.16	0.50	-0.56
0.24	-0.33	-1.21	2.06	0.89	0.03	0.52
-0.80	0.01	-0.61	-0.83	-0.31	-0.71	-0.46
-0.48	-0.75	-0.31	-0.71	0.49	0.13	-0.24
0.33	-0.99	-0.07	-1.28	3.70	3.47	1.02
0.87	0.98	-0.88	-0.30	1.61	0.33	-0.30
0.25	0.30	1.52	1.74	-0.79	1.29	0.57
-0.28	-0.21	-0.36	-0.29	-0.19	-0.25	-0.10
-0.48	1.07	-0.32	-0.53	0.53	0.12	-0.08
1.22	-0.99	-0.93	0.39	0.07	0.95	-1.45
-0.33	1.56	-0.40	-0.73	0.97	0.02	-0.17
0.57	2.20	-0.32	6.32	-0.61	-0.23	-0.12
-0.63	1.23	0.95	-1.41	1.22	0.38	-0.62
0.15	1.96	-0.83	2.24	1.52	1.39	-0.50

-0.31	0.25	-0.35	-0.31	-0.21	-0.26	-0.16
0.14	1.17	-0.77	-0.98	1.83	-0.49	0.75
-0.49	0.39	-0.49	-1.50	3.44	2.39	0.87
-0.60	0.06	-0.16	-1.45	-0.04	-0.34	-1.19
0.22	-0.25	-0.08	-0.47	-0.11	-0.62	-0.18
1.12	0.00	-0.76	-0.23	-0.19	0.68	-0.15
-0.57	1.53	0.09	-0.27	0.95	0.98	0.07
-0.32	0.13	-0.52	-0.61	-0.19	-0.63	-0.20
-0.16	-0.19	-0.22	-0.16	0.07	-0.20	-0.17
-0.71	-0.78	0.04	-0.45	-1.32	-0.19	0.17
-0.59	0.82	-0.95	-1.02	0.36	-0.69	0.02
-0.60	0.95	-0.57	-0.70	0.35	-0.02	0.42
-0.54	0.34	-0.85	-0.71	-0.14	-0.67	-0.10
0.42	2.26	-0.96	0.38	2.15	0.20	0.64
-0.32	-0.21	-0.35	-0.37	-0.20	-0.37	-0.21
0.23	-0.07	0.24	1.15	-0.23	0.47	0.02
-0.84	-1.21	-0.34	0.83	-1.36	-0.63	0.04
-0.62	0.54	-0.55	-0.88	-0.12	-0.55	-0.48
-0.96	1.33	-0.82	-1.26	-0.37	-0.53	0.00
-0.09	0.79	-0.65	-1.11	5.19	0.73	0.53
0.10	-0.81	-0.15	-0.40	-0.81	0.72	-1.14
0.07	1.14	-0.17	2.21	0.78	0.13	-0.12
-0.09	-0.20	-0.21	-0.32	-0.14	-0.39	-0.30
0.19	-0.68	0.48	1.62	-0.90	0.18	-0.50
0.17	0.30	-0.58	-0.96	-0.37	0.33	-0.26
-0.55	-0.62	-1.57	-1.57	1.61	-0.53	0.10
0.77	0.07	-0.04	2.72	-0.14	1.23	0.47
-0.11	-0.44	-0.34	-0.68	-0.68	-0.14	-0.34
-0.35	4.02	-0.47	-0.50	-0.16	0.07	-0.37
-0.35	0.80	-0.99	-0.92	3.52	-0.23	-0.70
1.00	0.30	-0.53	8.68	0.53	0.69	0.52
1.26	-0.62	2.34	8.03	-0.96	0.88	0.41
-0.55	-0.31	-0.55	-0.85	0.54	-0.51	0.89
-1.18	0.65	-0.99	-1.96	2.40	1.10	0.90
-0.19	-0.99	0.02	-0.33	-0.01	-0.41	-0.94
0.60	-0.51	2.08	0.08	0.89	1.10	-0.50
-0.14	-0.17	-0.29	-0.16	-0.22	-0.24	-0.06
-0.01	-0.23	-0.77	0.68	2.73	0.90	2.36
0.13	0.26	-0.69	-1.11	0.86	-0.04	1.75
-0.38	1.02	-0.58	-0.44	0.41	-0.31	0.23
-0.57	0.51	-0.29	-0.48	1.04	0.16	0.18
0.31	-0.10	3.01	1.26	-0.61	1.21	-0.49
0.50	0.85	2.29	3.86	-0.56	2.49	0.70
0.34	-0.84	1.95	2.12	-0.07	0.49	-0.59
-0.53	1.26	-0.56	-0.78	-0.12	-0.61	-0.11
-0.07	0.59	-0.57	2.37	-0.23	-0.24	-0.34
0.23	0.26	-0.40	-1.07	3.27	1.53	0.95
0.03	-1.02	0.08	5.20	-0.58	0.21	-0.80

-0.33	-0.26	-0.43	-0.65	-0.25	-0.45	-0.44
-0.94	1.76	-0.39	-0.94	0.16	-0.77	0.47
-0.75	0.45	-0.54	-0.90	-0.31	-0.59	-0.29
0.45	0.75	0.84	10.03	0.59	0.71	0.63
-0.26	0.47	-0.33	-0.77	1.10	0.14	-0.22
-0.73	1.60	-1.05	-0.32	0.19	-0.37	0.20
-0.82	2.47	-0.98	2.35	-0.25	-0.17	0.15
-0.38	0.55	1.16	-1.45	0.92	0.65	0.74
0.39	0.20	0.82	0.74	0.35	0.90	-0.05
1.13	1.89	1.54	1.13	0.21	-0.04	0.07
-0.59	0.18	-1.07	-1.00	-0.02	-0.90	-0.29
-0.44	-1.00	-0.17	-0.51	0.00	1.19	-0.20
-0.01	-1.50	-0.69	-1.73	-1.07	0.38	-1.24
0.15	2.85	-1.20	-1.25	0.94	0.20	-0.05
-0.09	2.03	-0.76	-0.54	0.93	0.16	0.44
-0.09	2.03	-0.76	-0.54	0.93	0.16	0.44
-0.36	0.22	0.08	-1.02	-0.38	-0.62	-0.40
0.38	0.44	-0.63	4.19	-0.17	-0.17	0.86
0.33	0.76	1.00	-1.55	-0.04	1.09	1.03
-0.39	-0.53	-0.12	-0.72	1.33	1.35	-0.51
-0.56	1.53	0.69	-1.02	0.67	0.98	0.35
-0.18	0.29	-0.20	-0.24	0.02	-0.18	-0.18
0.09	-0.16	-0.12	0.05	-0.74	0.28	0.20
1.01	-0.42	-0.49	7.11	0.78	-0.31	-0.56
0.44	-0.15	0.95	-0.67	0.34	0.82	0.46
0.41	-0.39	0.52	1.69	0.12	-0.18	-0.38
0.12	-0.22	0.17	0.43	-0.09	0.05	-0.27
0.03	-0.51	-0.54	1.20	0.02	-0.34	-0.24
-0.14	1.29	2.78	-0.33	0.87	0.78	0.55
1.35	0.08	-0.86	-1.53	1.79	-0.08	0.09
-0.39	0.80	-0.48	5.29	0.28	-0.08	-0.21
-0.43	0.57	-0.62	1.27	1.23	0.27	-0.12
1.04	1.99	-0.13	-0.09	1.16	0.47	-0.52

6	7	8	9	10	11	12
42	43	44	45	46	47	48
TGAGGGGCAGAGA	TTCACCACCTTC	AACATTCATTGC	TCAGGCTCAGTC	CTGACCTATGAA	TCTCACACAGAAA	AGCTACATCTGC
miR-423-5p	miR-197-3p	miR-181b-5p	miR-484	miR-192-5p	miR-342-3p	miR-222-3p
312449	286288	270366	261666	248983	229733	221365
-0.27	0.21	-0.55	0.57	-0.83	1.43	0.56
-0.54	1.16	-1.07	1.61	1.81	2.87	-0.96
-0.18	-0.97	-1.03	0.17	0.07	-0.15	-0.09
-0.22	-0.75	-1.07	-0.40	-0.61	-0.52	-0.40
-0.64	1.99	0.47	3.70	-0.23	2.74	-0.45
-0.26	-1.22	0.32	-0.57	-0.70	-0.91	1.34
-0.17	-0.04	0.88	-0.28	-0.79	-0.48	1.65
1.03	1.10	0.03	-0.23	-0.79	-0.94	-0.92
-0.42	-0.71	-1.58	-0.29	0.01	-0.81	-1.72
-0.65	-0.46	-1.31	-0.04	1.07	1.01	-1.64
-0.42	-0.38	-0.92	0.18	1.19	0.50	-0.47
4.21	-0.84	2.09	-1.14	-0.72	-0.39	0.20
-0.53	-0.01	-0.13	-0.87	1.93	-0.10	0.88
0.39	0.25	0.77	0.19	-0.73	-0.53	-1.51
-0.57	-0.10	-0.70	-0.05	-0.32	-0.73	-0.48
-0.58	-0.52	0.40	-0.80	0.13	-0.38	1.01
-0.39	0.19	1.79	0.64	-0.76	0.22	-0.21
-0.10	-0.47	-0.59	-0.58	0.19	-0.36	-0.88
-0.19	-0.50	0.68	-1.06	0.00	-0.14	0.37
-0.22	-0.50	-0.57	-0.23	2.41	-0.15	0.37

-0.63	-0.98	0.22	-0.77	-0.56	-0.91	0.72
1.82	1.65	0.75	-0.23	-0.83	-0.22	1.82
0.09	0.92	0.69	-0.32	-0.70	-0.34	1.48
-0.07	0.42	1.22	0.12	-0.76	-0.49	0.47
0.25	2.62	-0.65	1.60	-0.55	-0.50	-0.96
-0.17	-0.84	1.18	-0.13	-0.69	0.92	0.45
-0.58	-1.23	-1.33	-0.78	1.77	-0.65	-0.62
-0.52	-1.13	-1.59	-1.00	4.11	-0.52	-1.11
-0.44	0.08	-1.15	-0.40	0.22	0.10	1.40
-0.72	-0.63	-1.50	-0.48	0.63	0.13	-0.92
-0.65	0.23	-1.58	2.88	-0.21	-0.65	-1.38
-0.45	-0.55	-1.12	-0.38	5.41	0.65	0.51
-0.56	1.34	-1.61	3.19	2.62	0.24	-1.78
-0.09	3.08	-0.80	0.82	-0.69	-0.85	1.29
-0.55	1.82	-0.59	0.58	-0.30	-0.11	0.40
0.14	0.77	0.68	-0.07	-0.75	0.23	2.02
3.28	-1.16	-1.28	-1.19	-0.87	-1.22	-1.26
-0.70	-0.43	0.01	-1.73	0.17	-0.47	-0.22
-0.71	-0.36	-0.08	-1.21	1.53	2.50	0.02
0.17	0.56	1.90	-0.78	-0.48	1.08	0.42
2.65	8.40	2.31	3.64	-0.67	0.20	-0.04
3.67	8.63	3.41	2.87	-0.52	3.57	7.71
-0.59	-0.21	0.12	0.24	-0.80	-0.48	0.33
-0.70	0.21	-0.18	-0.33	0.01	-0.75	-0.99
1.17	-0.10	0.44	-1.21	-0.61	-0.31	-0.07
3.29	4.35	1.67	2.06	-0.83	-0.71	-1.28
-0.30	0.48	-0.16	0.89	-0.31	0.49	3.70
-0.18	0.83	0.50	0.03	-0.71	0.13	3.47
-0.54	-0.35	-0.56	0.52	-0.46	-0.24	1.02
-0.29	-0.23	1.05	0.45	-0.71	0.82	1.87
-0.57	-0.68	0.83	-0.24	-0.34	0.83	2.38
-0.40	0.00	-0.56	0.83	-0.82	-0.54	3.13
-0.45	-0.22	-1.15	0.84	-0.82	-0.98	1.13
-0.49	0.06	-0.45	0.70	-0.65	-0.08	1.27
0.61	-0.84	0.08	-0.62	-0.76	-0.91	4.19
-0.37	-0.50	-0.46	1.00	-0.80	-0.49	0.59
-0.63	-0.89	-0.81	-0.20	-0.67	-0.76	0.81
-0.35	-1.33	0.05	-1.47	3.52	-0.58	-1.18
0.40	0.37	1.69	-0.03	-0.79	0.01	1.34
-0.33	0.33	1.93	0.38	-0.09	-0.23	3.04
-0.45	0.60	1.39	0.84	-0.05	0.44	0.71
-0.30	-1.31	-1.11	-1.29	5.44	-0.88	-1.74
0.62	-0.58	-0.18	0.36	-0.78	-0.74	-1.13
0.45	-1.09	-0.23	-0.49	-0.59	-0.61	0.12
-0.16	-1.18	-0.73	-0.17	-0.16	-0.63	-1.15
0.66	-0.80	2.04	-0.99	0.07	0.03	-0.33
-0.63	-0.90	-1.60	-0.77	0.08	-0.06	-0.94
-0.69	1.29	-1.70	0.51	1.46	1.34	-0.36
0.67	1.37	0.20	3.77	-0.20	-0.06	0.79
-0.22	2.17	0.98	2.80	-0.32	0.49	4.54
-0.21	0.09	-0.83	-0.01	-0.67	-0.45	0.98
-0.28	-1.43	-0.94	-0.67	2.05	-0.33	-0.94
-0.39	-1.05	-0.99	-0.85	0.27	-0.83	-1.21
-0.39	0.00	0.23	-0.73	0.66	0.04	-0.07
6.68	15.37	3.15	7.94	-0.84	2.81	2.97
-0.40	-0.85	0.54	-1.14	-0.03	-0.51	-0.06
-0.62	-0.70	-0.52	-1.35	0.82	0.15	-0.84
1.22	-1.01	0.94	-1.76	0.97	-0.66	-1.04
-0.68	-0.64	-0.28	1.72	0.38	0.51	1.23

-0.54	-0.50	-0.65	0.68	-0.17	0.44	0.93
-0.47	-0.26	-0.85	2.17	-0.69	0.12	-0.42
-0.49	-0.30	-0.32	0.81	-0.39	0.95	1.56
0.11	-0.64	0.76	0.28	-0.74	0.57	0.27
-0.55	-0.45	0.11	-0.14	3.65	1.77	-0.07
-0.53	-1.23	-1.13	-1.27	6.22	-0.59	-1.45
0.09	0.19	-0.32	1.37	-0.61	1.03	2.03
-0.33	-0.09	0.13	0.08	-0.63	0.86	1.79
-0.59	-0.10	-0.35	0.35	-0.67	0.11	0.40
-0.36	-0.61	-0.05	-1.26	0.24	-0.71	0.69
-0.16	0.04	-0.26	0.86	-0.64	0.13	1.11
-0.25	-1.22	0.02	-1.51	5.00	-0.71	-1.58
-0.68	-0.68	-0.10	-1.07	-0.68	-1.01	-0.23
0.45	1.11	1.75	-0.18	-0.77	-0.55	1.56
-0.64	-1.08	-0.88	-1.18	-0.18	-0.84	-1.16
-0.59	-0.98	-0.12	-0.62	-0.15	0.32	-0.89
0	-1	-1	-1	0	-1	0
0.00	0.00	0.00	0.00	0.00	0.00	0.00
1.00	1.00	1.00	1.00	1.00	1.00	1.00
0.73	0.72	0.90	0.94	0.52	0.37	0.33

40	41	42	43	44	45	46
TGAGGGGCAGAG/	TTCACCACCTT(	AACATTCATTG(	TCAGGCTCAGT(	CTGACCTATGA/	TCTCACACAGA/	AGCTACATCTG(
miR-423-5p	miR-197-3p	miR-181b-5p	miR-484	miR-192-5p	miR-342-3p	miR-222-3p
312449	286288	270366	261666	248983	229733	221365
-0.27	0.21	-0.55	0.57	-0.83	1.43	0.56
-0.54	1.16	-1.07	1.61	1.81	2.87	-0.96
-0.18	-0.97	-1.03	0.17	0.07	-0.15	-0.09
-0.22	-0.75	-1.07	-0.40	-0.61	-0.52	-0.40
-0.64	1.99	0.47	3.70	-0.23	2.74	-0.45
-0.26	-1.22	0.32	-0.57	-0.70	-0.91	1.34
-0.17	-0.04	0.88	-0.28	-0.79	-0.48	1.65
1.03	1.10	0.03	-0.23	-0.79	-0.94	-0.92
-0.42	-0.71	-1.58	-0.29	0.01	-0.81	-1.72
-0.65	-0.46	-1.31	-0.04	1.07	1.01	-1.64
-0.42	-0.38	-0.92	0.18	1.19	0.50	-0.47
4.21	-0.84	2.09	-1.14	-0.72	-0.39	0.20
-0.53	-0.01	-0.13	-0.87	1.93	-0.10	0.88
0.39	0.25	0.77	0.19	-0.73	-0.53	-1.51
-0.57	-0.10	-0.70	-0.05	-0.32	-0.73	-0.48
-0.58	-0.52	0.40	-0.80	0.13	-0.38	1.01
-0.39	0.19	1.79	0.64	-0.76	0.22	-0.21
-0.10	-0.47	-0.59	-0.58	0.19	-0.36	-0.88
-0.19	-0.50	0.68	-1.06	0.00	-0.14	0.37
-0.22	-0.50	-0.57	-0.23	2.41	-0.15	0.37
-0.63	-0.98	0.22	-0.77	-0.56	-0.91	0.72
1.82	1.65	0.75	-0.23	-0.83	-0.22	1.82
0.09	0.92	0.69	-0.32	-0.70	-0.34	1.48
-0.07	0.42	1.22	0.12	-0.76	-0.49	0.47
0.25	2.62	-0.65	1.60	-0.55	-0.50	-0.96
-0.17	-0.84	1.18	-0.13	-0.69	0.92	0.45
-0.58	-1.23	-1.33	-0.78	1.77	-0.65	-0.62
-0.52	-1.13	-1.59	-1.00	4.11	-0.52	-1.11

-0.44	0.08	-1.15	-0.40	0.22	0.10	1.40
-0.72	-0.63	-1.50	-0.48	0.63	0.13	-0.92
-0.65	0.23	-1.58	2.88	-0.21	-0.65	-1.38
-0.45	-0.55	-1.12	-0.38	5.41	0.65	0.51
-0.56	1.34	-1.61	3.19	2.62	0.24	-1.78
-0.09	3.08	-0.80	0.82	-0.69	-0.85	1.29
-0.55	1.82	-0.59	0.58	-0.30	-0.11	0.40
0.14	0.77	0.68	-0.07	-0.75	0.23	2.02
3.28	-1.16	-1.28	-1.19	-0.87	-1.22	-1.26
-0.70	-0.43	0.01	-1.73	0.17	-0.47	-0.22
-0.71	-0.36	-0.08	-1.21	1.53	2.50	0.02
0.17	0.56	1.90	-0.78	-0.48	1.08	0.42
2.65	8.40	2.31	3.64	-0.67	0.20	-0.04
3.67	8.63	3.41	2.87	-0.52	3.57	7.71
-0.59	-0.21	0.12	0.24	-0.80	-0.48	0.33
-0.70	0.21	-0.18	-0.33	0.01	-0.75	-0.99
1.17	-0.10	0.44	-1.21	-0.61	-0.31	-0.07
3.29	4.35	1.67	2.06	-0.83	-0.71	-1.28
-0.30	0.48	-0.16	0.89	-0.31	0.49	3.70
-0.18	0.83	0.50	0.03	-0.71	0.13	3.47
-0.54	-0.35	-0.56	0.52	-0.46	-0.24	1.02
-0.29	-0.23	1.05	0.45	-0.71	0.82	1.87
-0.57	-0.68	0.83	-0.24	-0.34	0.83	2.38
-0.40	0.00	-0.56	0.83	-0.82	-0.54	3.13
-0.45	-0.22	-1.15	0.84	-0.82	-0.98	1.13
-0.49	0.06	-0.45	0.70	-0.65	-0.08	1.27
0.61	-0.84	0.08	-0.62	-0.76	-0.91	4.19
-0.37	-0.50	-0.46	1.00	-0.80	-0.49	0.59
-0.63	-0.89	-0.81	-0.20	-0.67	-0.76	0.81
-0.35	-1.33	0.05	-1.47	3.52	-0.58	-1.18
0.40	0.37	1.69	-0.03	-0.79	0.01	1.34
-0.33	0.33	1.93	0.38	-0.09	-0.23	3.04
-0.45	0.60	1.39	0.84	-0.05	0.44	0.71
-0.30	-1.31	-1.11	-1.29	5.44	-0.88	-1.74
0.62	-0.58	-0.18	0.36	-0.78	-0.74	-1.13
0.45	-1.09	-0.23	-0.49	-0.59	-0.61	0.12
-0.16	-1.18	-0.73	-0.17	-0.16	-0.63	-1.15
0.66	-0.80	2.04	-0.99	0.07	0.03	-0.33
-0.63	-0.90	-1.60	-0.77	0.08	-0.06	-0.94
-0.69	1.29	-1.70	0.51	1.46	1.34	-0.36
0.67	1.37	0.20	3.77	-0.20	-0.06	0.79
-0.22	2.17	0.98	2.80	-0.32	0.49	4.54
-0.21	0.09	-0.83	-0.01	-0.67	-0.45	0.98
-0.28	-1.43	-0.94	-0.67	2.05	-0.33	-0.94
-0.39	-1.05	-0.99	-0.85	0.27	-0.83	-1.21
-0.39	0.00	0.23	-0.73	0.66	0.04	-0.07
6.68	15.37	3.15	7.94	-0.84	2.81	2.97
-0.40	-0.85	0.54	-1.14	-0.03	-0.51	-0.06
-0.62	-0.70	-0.52	-1.35	0.82	0.15	-0.84
1.22	-1.01	0.94	-1.76	0.97	-0.66	-1.04
-0.68	-0.64	-0.28	1.72	0.38	0.51	1.23
-0.54	-0.50	-0.65	0.68	-0.17	0.44	0.93
-0.47	-0.26	-0.85	2.17	-0.69	0.12	-0.42
-0.49	-0.30	-0.32	0.81	-0.39	0.95	1.56
0.11	-0.64	0.76	0.28	-0.74	0.57	0.27
-0.55	-0.45	0.11	-0.14	3.65	1.77	-0.07
-0.53	-1.23	-1.13	-1.27	6.22	-0.59	-1.45
0.09	0.19	-0.32	1.37	-0.61	1.03	2.03
-0.33	-0.09	0.13	0.08	-0.63	0.86	1.79

-0.59	-0.10	-0.35	0.35	-0.67	0.11	0.40
-0.36	-0.61	-0.05	-1.26	0.24	-0.71	0.69
-0.16	0.04	-0.26	0.86	-0.64	0.13	1.11
-0.25	-1.22	0.02	-1.51	5.00	-0.71	-1.58
-0.68	-0.68	-0.10	-1.07	-0.68	-1.01	-0.23
0.45	1.11	1.75	-0.18	-0.77	-0.55	1.56
-0.64	-1.08	-0.88	-1.18	-0.18	-0.84	-1.16
-1	-1	0	-1	0	0	-1

40	41	42	43	44	45	46
102	92	85	13	34	70	103
CAGTGCAATGAT(	TACAGTATAGA	TTCACAGTGGC	TGAGGTAGTAG	TAGCTTATCAG	TATGGCACTGGT	AGGGGTGCTAT(
miR-130b-3p	miR-144-3p	miR-27a-3p	let-7g-5p	miR-21-5p	miR-183-5p	miR-342-5p
26241.00	36676	49172	1899491	456843	75744	2.60E+04
-1.01	-0.20	-0.47	-0.70	-0.20	-0.38	0.47
-2.02	-0.32	-1.07	-1.21	-0.24	4.86	-1.27
0.01	-0.24	-0.11	0.87	-0.29	0.04	-0.50
-0.74	-0.25	-0.59	1.05	-0.33	-0.03	-0.89
-0.64	-0.22	-0.27	2.12	-0.04	-0.18	1.39
0.55	-0.23	-0.61	0.23	-0.25	-0.40	1.06
-0.75	-0.28	-0.80	1.39	-0.18	-0.40	0.53
-0.71	-0.12	-0.87	-1.39	-0.32	-0.37	-0.48
-1.10	-0.25	-0.62	-1.02	-0.36	0.14	-1.25
-1.20	-0.19	-0.49	-0.32	-0.26	0.41	-0.68
-0.03	-0.29	0.90	-0.92	-0.18	0.32	-0.54
-1.43	-0.31	-1.03	-0.13	-0.32	-0.40	2.99
1.17	0.13	2.19	2.52	4.83	-0.36	-0.39
-0.90	-0.24	-1.02	-1.23	-0.34	-0.29	0.06
-0.34	0.07	-0.14	-0.31	-0.27	-0.13	-0.67
1.65	-0.04	0.90	0.39	0.23	-0.19	-0.05
0.94	-0.25	0.38	-0.89	-0.32	-0.27	0.32
1.53	-0.27	-0.21	-0.57	-0.12	-0.37	-0.47
1.22	-0.30	0.50	-0.93	-0.16	0.07	-0.87
0.92	4.95	2.05	0.28	0.96	-0.29	-0.85
0.42	-0.10	-0.82	0.86	-0.26	-0.19	0.02
0.53	-0.31	-0.56	0.21	-0.34	-0.36	0.40
1.29	-0.27	-0.57	0.30	-0.25	-0.37	0.67
-0.50	-0.23	-0.66	-0.56	-0.30	-0.39	1.27
-0.16	-0.21	0.35	-0.51	-0.30	-0.33	-0.57
0.61	-0.30	1.65	0.09	-0.27	-0.30	1.44
0.71	0.27	2.00	0.35	-0.12	0.20	-1.13
-0.59	9.91	2.55	1.60	2.49	-0.05	-1.04
-0.22	-0.30	1.41	0.14	0.21	-0.13	-0.04
-1.33	-0.27	0.42	-1.15	-0.27	1.30	-1.27
-1.54	-0.21	-0.30	-1.33	-0.34	2.34	-1.36
0.11	-0.32	5.02	-0.25	0.11	1.93	-1.30
0.17	-0.14	0.29	1.21	-0.10	-0.33	-0.41
-1.22	-0.25	-0.38	-1.15	-0.10	-0.35	-1.09
-1.11	-0.13	-0.26	-1.28	0.04	-0.20	-0.93
-0.28	-0.20	-0.47	1.05	-0.27	-0.39	1.26
-1.58	-0.18	-1.23	-1.74	-0.38	-0.40	-0.25
0.10	-0.15	-0.75	0.71	0.28	-0.39	-0.68
-0.53	0.18	2.20	0.68	1.61	-0.26	-0.64

-0.10	-0.29	-0.28	-0.09	-0.21	-0.36	0.68
-1.16	-0.13	0.05	-0.79	-0.15	-0.34	3.48
-0.32	0.23	4.71	-0.29	-0.14	-0.40	13.87
0.23	-0.14	-0.35	-0.36	-0.33	-0.32	0.03
0.26	-0.17	0.80	0.49	0.72	-0.21	-1.02
-0.40	-0.29	-0.99	-1.55	-0.32	-0.35	0.08
-1.07	-0.16	-0.92	-1.30	-0.31	-0.37	5.20
3.27	-0.22	3.52	0.09	-0.19	-0.20	-0.58
1.53	-0.24	-0.23	-0.26	-0.23	-0.37	0.21
0.95	-0.06	-0.70	-0.88	-0.29	-0.21	-0.80
1.47	-0.23	-0.32	-0.56	-0.32	-0.28	0.00
1.40	-0.18	-0.36	-0.43	-0.23	-0.28	-0.46
1.56	-0.23	0.62	-0.02	-0.29	-0.25	0.24
1.66	-0.17	-0.28	-0.40	-0.30	-0.14	-0.88
1.87	-0.26	0.64	1.27	-0.17	-0.33	0.22
0.37	-0.27	-0.45	-0.09	-0.30	-0.19	0.27
1.43	-0.26	0.31	-0.95	-0.34	-0.31	-0.41
-0.19	-0.24	-0.35	0.74	-0.30	-0.10	-0.31
0.44	8.69	2.32	2.98	1.96	-0.27	-0.59
1.03	-0.28	-0.62	1.44	-0.32	-0.37	1.55
2.11	-0.24	1.06	2.98	0.00	-0.35	1.19
0.31	-0.19	0.43	2.87	-0.12	-0.34	1.42
0.84	8.22	1.39	1.90	0.79	-0.21	-1.03
0.09	-0.06	-0.05	-0.78	-0.36	-0.19	-0.46
0.35	-0.19	-0.22	0.82	-0.28	-0.25	0.77
1.18	-0.14	0.57	1.59	-0.33	-0.24	-0.82
-1.80	-0.24	-0.64	1.66	-0.14	-0.32	-0.42
-0.14	-0.17	0.46	-0.52	0.24	0.08	-0.86
-1.52	-0.32	1.34	-1.08	-0.19	1.32	-1.35
-0.46	-0.17	-0.65	5.24	-0.28	-0.22	0.17
0.53	-0.26	0.81	2.97	-0.02	-0.16	0.06
-0.93	-0.23	-0.69	0.67	-0.27	-0.31	-0.63
2.17	-0.21	-0.67	2.51	0.34	-0.40	-1.04
0.17	-0.12	-0.61	1.06	-0.21	-0.12	-0.56
-0.85	-0.16	-0.59	0.47	-0.01	-0.29	-0.28
-1.79	0.07	-0.77	-1.64	-0.38	-0.38	9.27
-0.15	-0.13	-0.41	2.20	0.04	-0.37	0.48
-0.37	-0.30	0.50	-0.62	-0.24	0.25	-0.66
0.94	-0.27	-0.48	-1.17	-0.25	0.09	0.54
0.65	0.30	6.11	0.70	-0.03	0.01	-1.02
4.04	-0.20	3.61	-0.03	-0.19	0.00	-0.93
2.05	-0.22	1.01	-1.44	-0.35	0.25	-1.03
2.34	-0.10	1.84	1.86	-0.24	-0.19	-0.25
0.81	-0.21	-0.68	-1.43	-0.30	-0.40	1.02
0.65	6.69	2.04	0.48	2.02	-0.31	-0.24
-0.16	14.75	1.47	1.91	1.76	-0.28	-1.20
1.18	-0.24	0.64	-1.26	-0.34	-0.38	-0.47
1.92	-0.19	1.13	0.76	-0.27	-0.29	-0.43
0.47	-0.16	0.04	0.21	-0.33	-0.03	-0.35
2.20	-0.28	0.14	0.31	0.25	-0.13	-0.85
1.93	-0.22	0.89	-0.10	-0.29	-0.20	-0.21
-0.78	-0.27	0.94	7.32	0.22	-0.26	-0.69
-0.42	-0.28	-0.38	-0.21	-0.29	-0.25	-0.65
1.07	-0.29	0.34	0.37	-0.26	-0.37	0.37
-0.45	-0.21	2.05	0.28	-0.27	0.23	-1.25
-0.03	-0.08	0.45	1.22	-0.06	0.14	-0.03
0.11	0.12	0.13	0.13	0.14	0.15	0.16
0.05	0.32	0.20	0.05	1.00	0.28	0.08

239935	1893208	1240451	2304775	1597470	1583268	2592561

50	51	52	53	54	55	56
20	21	22	23	24	25	26
D_9	E_20	E_29	E_4	E_5	E_6	F_1
CHR_NP	CHR_NP	CHR_NP	CHR_NP	CHR_NP	CHR_NP	CHR_NP
971042	2600064	2094850	4830515	2551385	2616413	3930264
0.92	0.68	0.29	0.24	0.49	0.84	1.00
D_9	E_20	E_29	E_4	E_5	E_6	F_1
731107	706856	854399	2525740	953915	1033145	1337703
122679	389983	434200	899048	539941	452076	1019114
4360	256917	87950	162314	99635	107343	314034
12343	135742	104686	168898	264828	202140	216370
2068	88459	83637	78820	66480	55579	193721
39570	121914	149724	164506	138176	149924	132168
1297	138924	19072	128682	43150	101571	22591
6148	189457	55740	89051	77544	75208	90366
453	18376	3407	35682	9097	19477	2885
1444	60543	20112	79855	30750	66644	21216
949	33423	16296	33869	20533	25326	33662
4340	14649	11862	19996	11806	16643	37442
2892	33311	26726	63887	34432	40006	45021
966	21311	11884	14690	12897	13780	28108
5265	10244	11251	23877	14905	12339	28906
146	12249	5069	11310	3826	8625	3619
4360	14452	19783	30744	21105	13046	29277
165	16723	3485	9674	4982	9159	5221
794	12614	9448	22251	11358	15361	16006
172	20440	4826	16228	8451	11039	8233
1368	15097	12160	9087	10001	8617	19882
832	10639	7806	8818	11595	7424	13305
295	6410	4200	9798	5656	6032	10784
89	11806	6426	5072	3364	3884	13575
74	30945	4146	12014	4908	4100	6108
181	12014	2145	3273	3201	5623	2676
491	10595	5810	12898	10754	8504	15410
3164	3971	6324	8727	6129	4824	16793
225	4317	1897	9309	3090	3810	1751
306	11047	4712	9403	5156	6291	11932
1144	8071	6087	5189	3944	6196	22369
173	15625	5490	15629	9462	9551	4731
311	5170	5106	4727	4324	3085	8943
211	8030	1759	4918	2963	4434	2540
117	2224	1179	1443	722	1495	1575
280	10370	4460	8887	4813	5923	11383
563	5536	3045	6668	3362	3311	6902
190	5926	3775	5773	2828	5461	5362
176	8080	1710	2449	2907	4450	4987
1015	3615	4264	3159	2550	655	13960
3700	3582	2940	2314	3146	1415	3960
2104	5684	4438	4198	3186	2075	6041
905	3620	3242	3567	5113	4677	3960
840	4933	2476	5529	3727	2842	6730
29	1420	336	1352	447	1157	341
212	4902	2611	3671	4984	4932	2963

210	5572	3584	4328	3568	3862	7118
243	4614	1861	2385	1755	3380	7296
744	1477	3362	4775	2856	2302	4118
23	338	162	616	238	324	192
51	3054	1345	1917	1056	961	742
311	2097	2013	605	2139	3436	17555
69	3858	1336	2036	1369	1445	2132
855	1586	1244	2444	1628	1537	3421
17	2990	1379	1240	1017	892	1714
477	2996	1905	1145	2342	1151	2854
13	247	111	382	144	292	154
116	2901	835	2672	1736	1442	2190
43	3742	2007	2472	2808	1498	1234
81	1716	988	1041	2131	1524	3269
168	2103	660	2449	982	1044	3947
158	1268	1255	1628	882	1673	3917
137	1917	1288	1637	1766	1312	894
11	394	49	481	66	408	193
22	463	83	204	182	169	162
158	375	991	2321	924	817	889
43	1159	357	1198	641	1102	773
19	962	450	1277	617	1420	495
24	516	139	671	271	586	321
117	1805	550	1314	924	1020	1142
33	1023	176	1219	552	550	1102
276	1048	1071	1559	846	907	2058
210	379	532	1490	755	691	1163
5	501	159	354	92	329	162
31	633	375	992	682	722	977
15	3022	643	1094	1412	1097	1119
90	423	988	238	734	1217	4399
238	1196	593	967	776	646	1496
66	921	242	714	277	349	2328
257	446	743	867	529	508	1474
33	616	697	859	424	988	2117
22	1099	316	825	620	644	478
228	646	794	1116	857	692	1845
67	526	571	923	583	485	1575
2	435	474	213	285	449	384
18	1648	250	272	298	282	920
421	671	482	839	547	646	1285
389	477	668	1088	565	552	1561
7	537	116	823	186	557	106
7	996	476	842	593	601	622
67	674	325	306	209	213	1078
60	651	464	429	381	918	693
6	28	15	97	23	36	36
67	967	382	1112	629	374	640
2	572	212	1030	339	996	490
10	333	78	348	121	219	84
26	642	259	492	448	482	196
96	306	494	418	305	293	877
124	312	511	626	344	285	517
118	404	357	350	428	350	727
1	172	34	218	47	173	58
142	260	169	277	188	191	683
17	749	334	525	427	412	703
233	277	324	271	373	262	690
4	310	111	220	147	240	251

13	291	87	432	137	291	117
2	133	52	171	82	135	69
282	422	296	537	302	315	655
10	811	290	369	295	297	449
20	249	99	315	145	247	181
71	164	118	281	130	238	132
1	392	231	454	348	524	163
45	308	247	330	477	272	542
55	272	152	309	141	135	382
6	186	40	184	103	179	94
19	226	272	246	500	337	362
6	135	217	140	98	123	596
9	266	133	223	144	226	119
14	309	136	304	113	351	286
14	309	136	304	113	351	286
5	170	80	208	109	118	203
111	177	117	427	228	194	233
6	189	196	362	254	278	176
5	362	243	90	355	632	386
3	240	186	249	160	148	121
3	65	22	42	26	45	33
29	123	171	309	193	109	316
125	250	83	120	161	103	368
12	206	172	274	247	261	310
59	208	105	158	106	149	628
40	184	145	171	112	85	313
34	106	38	88	67	48	453
13	190	122	208	120	208	156
0	269	78	164	73	203	368
118	201	99	162	115	113	339
27	208	75	92	72	116	95
16	207	108	122	114	110	268
16	157	66	182	112	85	107
26	78	83	308	142	132	97
0	208	77	173	113	153	215
30	185	88	212	120	143	302
34	195	145	141	124	100	191
5	197	84	256	134	111	184
82	81	79	157	82	90	123
12	236	91	135	113	93	193
1	14	2	24	7	15	18
18	38	66	214	97	93	90
8	125	119	176	160	55	153
1	347	125	212	121	158	98
11	99	37	154	54	69	93
68	87	70	155	125	101	149
7	109	37	68	48	51	62
2	76	32	86	58	92	31
1	99	81	36	119	278	193
11	149	92	143	97	35	159
3	93	108	137	399	120	22
32	47	50	143	61	59	116
6	62	38	36	41	30	134
29	49	78	122	58	226	314
16	73	51	135	133	109	158
28	32	48	159	57	55	112
0	24	4	11	12	9	19
38	113	53	87	49	53	147
39	91	74	122	76	60	155

1	93	20	52	33	46	48
6	248	44	69	30	43	63
25	48	81	166	73	56	96
1	116	68	95	118	69	241
77	31	50	22	35	57	30
1	79	15	79	40	82	35
5	43	31	125	54	69	50
40	207	90	137	103	55	157
18	50	30	85	33	52	77
6	67	71	54	53	70	127
4	110	38	79	49	74	89
1	128	31	69	16	35	35
87	45	114	57	68	22	81
7	28	67	48	43	114	413
4	72	42	90	45	102	68
14	59	39	78	68	45	120
33	42	57	29	53	104	338
9	63	0	76	41	53	143
0	69	16	66	21	22	22
28	16	60	25	61	99	641
1	120	17	115	42	83	13
8	30	47	84	32	66	351
34	53	49	64	64	45	84
0	67	14	62	21	21	21
9	27	51	13	58	101	409
17	67	45	134	72	99	117
2	88	24	29	30	35	169
2	9	7	130	52	105	366
0	55	11	45	26	55	4
0	64	35	71	44	73	103
7	69	66	109	40	21	71
4	33	17	42	14	36	261
10	63	60	39	37	38	94
0	62	14	25	20	33	12
10	18	29	119	50	47	31
20	40	34	57	33	61	84
9	91	45	55	75	30	33
26	109	66	22	62	20	77
2	49	38	63	43	75	37
22	53	35	31	22	24	97
9	39	49	62	28	26	125
0	24	6	41	10	37	12
21	53	59	26	74	104	327
22	36	29	56	37	33	46
0	52	33	85	51	48	80
4	42	35	77	43	120	120
14	16	21	56	37	23	38
14	95	51	48	47	47	109
7	88	32	61	57	37	52
0	60	10	13	27	80	67
21	20	26	49	29	53	61
6	42	31	85	31	55	44
21	73	32	37	34	38	78
3	97	34	48	46	38	87
0	63	29	19	30	25	18
0	5	0	7	1	3	0
1	40	12	28	36	24	29
0	9	11	6	5	11	6
15	18	20	83	25	24	37

2	68	20	33	51	34	55
16	14	14	48	16	31	40
0	46	21	21	62	159	96
10	28	37	26	51	21	61
19	24	33	24	32	11	45
6	12	16	130	28	34	50
8	39	20	31	16	21	102
4	36	10	28	23	28	35
2	37	16	26	5	33	25
7	27	32	29	13	18	47
0	17	1	6	1	4	6
15	22	16	36	19	29	90
1	54	8	47	37	45	43
13	8	17	44	42	14	26
14	31	16	19	39	19	44
0	47	6	9	5	12	27
1	52	11	18	11	18	14
7	37	24	55	27	27	48
6	17	17	7	13	49	111
4	35	9	26	26	15	39
1	27	5	10	7	15	2
2	101	17	41	25	22	22
2	101	17	41	25	22	22
9	10	13	12	19	47	209
11	26	25	32	13	16	35
5	35	11	16	15	14	36
2	37	23	12	50	74	101
0	59	15	31	25	24	22
7	35	10	45	31	14	26
6	24	6	20	9	17	25
0	28	3	19	22	22	10
9	27	17	18	48	18	42
1	64	15	39	24	12	5
0	81	14	7	15	4	90
2	18	24	41	23	32	17
3	30	8	18	17	22	28
1	4	0	1	1	1	4
1	17	10	38	21	24	22
1	55	5	29	16	11	11
12	25	33	14	9	8	38
10	11	12	36	16	16	21
1	52	5	28	15	11	11
4	14	6	32	8	3	47
6	24	17	27	13	22	70
1	17	6	17	14	35	18
6	9	9	35	10	20	23
0	0	0	0	3	1	0
25	12	21	7	31	12	18
12	14	14	29	7	13	15
4	6	9	27	11	1	26
3	14	19	12	15	25	97
0	31	8	22	23	25	9
0	1	0	3	3	1	1
2	20	3	19	18	14	28
0	16	4	26	12	22	6
0	0	0	0	0	0	0
11	14	11	10	8	14	16
2	13	5	10	9	4	20
10	23	5	13	10	8	36

8	8	24	7	14	18	82
0	13	17	14	12	16	27
2	9	15	3	16	30	101
4	16	14	19	15	3	14
7	16	30	22	20	16	45
8	13	22	16	8	14	38
0	15	4	24	12	19	5
0	12	8	5	28	42	48
0	6	2	11	1	1	0
0	18	6	17	1	14	4
0	21	9	3	6	7	15
4	11	11	20	18	8	29
3	12	6	10	5	7	14
3	6	20	32	49	12	3
8	10	11	10	20	28	106
0	16	7	18	6	19	3
0	6	6	10	8	9	38
16	13	6	10	13	9	19
5	12	11	14	20	23	22
0	11	1	11	17	70	11
2	11	16	7	17	18	85
2	19	11	21	17	10	25
11	8	17	16	8	12	25
4	1	7	18	6	7	6
0	11	11	13	7	23	11
5	10	9	18	14	11	9
7	11	12	16	15	17	11
10	7	9	14	8	8	33
4	13	10	9	7	5	18
1	16	10	14	10	24	14
2	8	9	13	10	10	26
0	10	3	5	4	7	2
0	18	2	7	1	5	6
0	9	5	10	7	7	4
0	13	4	10	3	6	17
8	3	8	9	8	5	9
1	6	14	11	6	3	29
3	12	7	12	8	24	18
1	14	11	18	12	6	13
3	12	15	8	10	8	25
1	15	6	2	2	7	4
1	8	7	6	11	8	12
5	13	10	6	18	16	81
4	9	9	14	6	7	27
0	25	7	6	19	25	38
2	7	5	16	9	5	1
3	5	7	13	8	7	20
0	4	4	12	2	8	6
12	9	3	9	4	5	14
1	16	6	3	16	2	7
10	10	5	20	7	10	17
0	8	1	1	5	2	4
3	9	8	13	8	6	17
0	22	1	11	5	15	4
2	14	3	9	8	5	9
0	10	9	8	10	13	14
2	7	11	17	5	8	16
1	5	0	2	4	0	33
2	8	9	10	8	18	10

1	6	5	7	4	9	5
1	13	9	9	5	7	10
0	8	3	9	10	7	18
1	5	7	16	7	7	9
4	10	17	18	7	0	8
1	18	0	14	5	7	19
3	6	4	14	8	4	3
0	6	1	2	4	6	8
0	11	3	7	7	7	5
0	15	8	9	5	11	8
3	9	5	15	5	4	16
4	13	7	12	6	8	5
0	4	7	2	2	0	3
0	2	2	0	0	3	1
4	24	5	9	2	4	3
0	3	0	7	10	4	4
0	10	3	9	6	8	8
3	7	4	10	5	9	16
0	13	1	4	1	16	7
3	12	1	4	3	2	6
0	10	5	3	4	5	9
0	10	2	11	6	1	2
0	10	7	4	8	4	20
2	5	6	4	4	6	11
3	6	5	7	2	6	2
0	4	6	6	5	5	17
0	8	1	7	3	4	1
2	7	2	3	1	1	2
0	6	1	9	7	3	13
0	15	7	12	2	8	5
5	5	5	3	1	14	26
0	6	5	4	3	3	10
1	18	5	2	3	5	8
2	2	6	5	9	5	3
0	5	2	2	2	1	1
1	3	5	3	9	7	26
0	5	1	7	7	3	7
4	1	1	2	4	1	12
2	3	7	2	3	3	9
0	5	3	9	4	3	10
3	11	3	0	3	1	5
0	8	5	0	3	8	4
0	4	6	4	6	6	32
0	8	2	6	0	1	8
1	5	2	6	6	1	10
2	4	2	6	6	4	4
0	14	6	8	3	4	2
1	0	5	5	2	5	9
0	7	2	26	7	3	0
0	3	3	7	0	0	4
4	2	4	7	1	2	4
0	7	6	2	5	18	11
1	6	1	3	2	5	5
1	10	2	8	4	2	6
0	12	5	7	4	8	13
0	3	2	3	1	1	0
1	7	5	2	5	6	7
1	6	3	5	6	1	5
0	6	15	5	12	0	4

0	5	0	4	5	4	1
2	11	6	3	1	1	10
0	6	1	7	4	2	0
0	5	1	8	1	4	1
3	6	3	1	4	2	10
0	1	0	6	1	4	20
2	3	5	7	2	4	10
1	11	3	2	4	1	3
3	5	3	8	3	3	11
0	61	4	6	4	0	7
0	1	0	1	0	1	0
0	1	0	0	0	0	2
0	0	0	1	0	1	0
0	7	0	3	3	2	4
1	4	3	3	7	6	3
1	9	8	7	3	6	11
1	1	1	9	2	5	3
0	0	5	4	1	6	11
0	1	0	1	0	1	0
0	5	1	0	3	13	4
0	2	1	1	3	5	17
0	8	0	3	0	2	3
0	1	1	5	1	3	8
1	2	4	1	3	3	8
0	1	1	6	3	1	7
0	0	1	0	2	1	0
2	4	4	2	12	4	10
0	2	1	4	0	3	2
0	3	3	4	4	1	5
1	9	1	4	2	6	7
0	3	0	2	0	2	0
0	5	2	5	3	9	6
1	2	3	6	6	3	4
2	2	2	3	2	1	2
0	3	0	2	7	5	3
1	5	1	4	2	0	9
2	5	7	3	4	0	5
1	0	4	9	3	3	2
0	6	1	8	3	3	6
0	1	5	0	1	3	7
0	0	0	0	0	1	2
0	7	2	6	1	0	4
0	1	1	7	1	2	8
0	4	2	6	3	4	7
0	2	2	6	4	2	2
0	7	3	0	0	9	5
0	2	0	2	1	4	1
2	1	1	3	5	1	3
5	2	3	2	2	0	4
1	1	2	0	1	1	3
0	5	2	1	1	5	5
0	5	2	2	0	3	2
0	1	1	4	0	6	5
1	3	1	5	0	0	2
0	4	2	5	8	4	3
0	7	1	2	2	0	0
0	6	1	3	1	0	3
2	0	3	5	2	1	6
3	3	2	11	1	2	5

0	3	4	5	8	1	3
1	0	5	2	2	1	4
0	2	4	1	6	2	1
2	1	2	1	2	1	3
0	4	4	2	1	4	4
1	1	0	0	0	1	0
1	1	1	2	3	2	6
1	1	0	2	1	3	3
1	1	3	2	1	0	5
0	5	2	0	0	2	3
0	1	0	4	0	0	8
1	2	0	8	0	1	5
0	4	0	1	1	2	1
2	2	3	1	2	2	1
0	1	0	4	2	3	0
0	4	2	1	5	2	4
0	5	4	2	0	0	5
0	2	0	1	0	1	17
0	0	0	1	0	0	0
2	6	2	3	2	0	1
0	2	1	3	3	1	1
0	1	0	0	0	0	0
0	2	0	1	0	1	0
0	4	1	3	1	0	1
3	3	1	1	1	1	4
0	4	0	3	0	3	3
1	3	3	4	2	1	4
1	2	2	5	2	2	8
0	0	0	1	0	0	1
0	0	0	0	0	0	0
0	2	0	2	2	4	2
0	2	0	0	0	2	5
0	0	2	2	1	0	1
0	1	0	6	0	0	2
0	2	0	3	0	0	4
0	2	0	0	1	2	1
7	0	2	0	1	1	1
0	3	2	0	1	2	2
0	4	1	0	1	5	6
0	0	2	1	1	3	1
0	0	0	0	0	2	0
0	0	0	3	0	2	9
0	6	3	0	5	0	3
1	2	2	2	3	0	6
1	1	3	2	1	0	4
0	1	1	2	1	0	0
1	1	0	1	0	1	4
0	0	0	1	0	0	1
0	0	0	2	1	1	1
0	0	0	2	0	1	5
0	1	0	3	2	3	1
0	1	0	1	0	0	0
0	1	0	2	0	1	0
0	0	0	5	3	0	1
0	0	0	4	1	1	1
0	1	0	1	0	3	0
0	0	3	2	3	4	6
2	3	0	1	4	3	2
1	0	1	1	2	2	2

0	2	1	1	1	1	2
0	4	0	1	4	0	2
1	2	1	0	3	0	0
0	5	1	1	2	0	2
0	7	1	0	1	1	1
0	1	1	1	6	0	1
0	1	3	1	3	3	2
3	0	1	2	7	0	1
0	0	1	3	7	1	3
0	2	1	0	3	0	0
0	2	1	2	0	3	3
0	0	0	1	1	1	2
2	0	0	0	0	0	0
D_9	E_20	E_29	E_4	E_5	E_6	F_1
CHR_NP	CHR_NP	CHR_NP	CHR_NP	CHR_NP	CHR_NP	CHR_NP
731107	706856	854399	2525740	953915	1033145	1337703
24	9	13	17	12	13	11
0.53	-2.59	-1.70	-0.97	-1.94	-1.79	-2.15

0.64	0.04	0.25	0.89	0.04	0.21	0.21
15	34	25	9	35	27	28
1	1	1	1	1	1	1
50	51	52	53	54	55	56
23	24	25	26	27	28	29
E_5	E_6	F_1	F_35	F_36	F_37	F_38
CHR_NP	CHR_NP	CHR_NP	CHR_NP	CHR_NP	CHR_NP	CHR_NP
1597470	1583268	2592561	3565545	2585170	976547	1738984
E_5	E_6	F_1	F_35	F_36	F_37	F_38
539941	452076	1019114	1755132	781741	473210	768215
99635	107343	314034	340046	296230	77703	201610
264828	202140	216370	77296	194779	50911	97414
66480	55579	193721	338825	202546	74728	131317
138176	149924	132168	131189	135975	59576	111341
43150	101571	22591	56016	78288	14801	32212
77544	75208	90366	90072	153035	26823	51941
9097	19477	2885	5235	8559	768	2104
30750	66644	21216	31586	66828	10588	16579
20533	25326	33662	60382	37679	15012	25734
11806	16643	37442	58644	47412	13953	14827
34432	40006	45021	44732	45670	12668	24311
12897	13780	28108	31789	45628	10437	10434
14905	12339	28906	62313	26495	11105	26421
3826	8625	3619	7407	17517	4647	2353
21105	13046	29277	27797	31237	13167	9460
4982	9159	5221	6277	15435	2270	2745
11358	15361	16006	20862	29022	4473	8323
8451	11039	8233	7956	16360	2743	5304

10001	8617	19882	18075	15799	5800	12796
11595	7424	13305	14518	15608	3396	10456
5656	6032	10784	17013	14339	2358	6331
3364	3884	13575	15150	15827	4989	8178
4908	4100	6108	5207	16756	1222	3150
3201	5623	2676	2223	6081	1028	1837
10754	8504	15410	18819	12991	3995	10054
6129	4824	16793	22216	12082	4650	8405
3090	3810	1751	7872	3113	1833	766
5156	6291	11932	17314	13790	3271	6517
3944	6196	22369	25514	13858	4918	8664
9462	9551	4731	4426	12369	2081	2658
4324	3085	8943	17338	9925	3677	6526
2963	4434	2540	2521	5618	786	1282
722	1495	1575	2065	3334	537	549
4813	5923	11383	16461	13215	3026	6156
3362	3311	6902	16417	10148	1342	7627
2828	5461	5362	8173	5067	2086	3342
2907	4450	4987	4628	4906	1909	3313
2550	655	13960	11242	5566	1962	7611
3146	1415	3960	4905	3092	5354	2915
3186	2075	6041	7186	6246	1073	2774
5113	4677	3960	3350	4246	2177	2881
3727	2842	6730	9416	6452	1494	4811
447	1157	341	504	891	212	289
4984	4932	2963	1799	4632	598	2122
3568	3862	7118	6858	5056	3186	2944
1755	3380	7296	6820	6085	1422	2590
2856	2302	4118	4403	4612	1915	2580
238	324	192	202	302	126	111
1056	961	742	1113	1973	318	373
2139	3436	17555	14345	2557	601	5781
1369	1445	2132	2976	6477	409	1087
1628	1537	3421	5234	3637	565	2223
1017	892	1714	1390	2111	949	1193
2342	1151	2854	2421	1850	546	1509
144	292	154	214	272	61	88
1736	1442	2190	2551	3851	530	1617
2808	1498	1234	1030	2129	438	803
2131	1524	3269	1644	3523	844	1334
982	1044	3947	3551	3532	566	4142
882	1673	3917	3851	3053	630	1099
1766	1312	894	1101	1675	297	668
66	408	193	269	194	49	145
182	169	162	147	103	36	52
924	817	889	2726	739	1151	933
641	1102	773	1478	1802	176	462
617	1420	495	426	1076	132	207
271	586	321	754	809	80	200
924	1020	1142	1539	2826	156	618
552	550	1102	2470	619	557	497
846	907	2058	2575	1968	800	1021
755	691	1163	2735	1136	1147	1340
92	329	162	370	576	114	85
682	722	977	1460	1111	336	672
1412	1097	1119	601	1931	249	659
734	1217	4399	4764	1152	298	1655
776	646	1496	1583	1483	332	1087
277	349	2328	4121	1625	424	2238

529	508	1474	1671	1732	531	635
424	988	2117	2383	1721	236	768
620	644	478	748	824	200	493
857	692	1845	1459	1299	405	586
583	485	1575	2105	859	519	984
285	449	384	315	1205	104	101
298	282	920	694	926	161	528
547	646	1285	1177	1147	120	431
565	552	1561	1502	1142	677	586
186	557	106	170	286	62	110
593	601	622	620	1201	397	272
209	213	1078	1863	773	509	749
381	918	693	825	668	446	661
23	36	36	84	25	8	17
629	374	640	441	827	179	274
339	996	490	617	691	103	277
121	219	84	167	329	31	64
448	482	196	215	689	105	109
305	293	877	897	634	421	432
344	285	517	697	524	260	312
428	350	727	523	454	179	461
47	173	58	125	237	23	42
188	191	683	962	465	50	359
427	412	703	1006	760	181	461
373	262	690	379	677	270	303
147	240	251	398	452	84	161
137	291	117	193	272	62	105
82	135	69	100	131	27	53
302	315	655	721	723	139	230
295	297	449	589	953	97	265
145	247	181	313	421	47	98
130	238	132	213	405	24	84
348	524	163	131	270	96	174
477	272	542	512	688	158	264
141	135	382	512	578	29	211
103	179	94	282	211	26	125
500	337	362	87	404	88	171
98	123	596	599	490	173	272
144	226	119	202	194	57	81
113	351	286	309	640	111	118
113	351	286	309	640	111	118
109	118	203	279	305	27	85
228	194	233	373	339	52	165
254	278	176	508	437	182	212
355	632	386	277	162	35	160
160	148	121	142	312	51	120
26	45	33	22	71	5	15
193	109	316	490	442	217	130
161	103	368	223	330	42	345
247	261	310	326	367	76	178
106	149	628	632	248	48	184
112	85	313	675	304	145	168
67	48	453	736	436	77	457
120	208	156	110	319	76	61
73	203	368	354	402	42	135
115	113	339	426	331	43	169
72	116	95	57	124	31	25
114	110	268	239	282	60	99
112	85	107	263	89	12	89

142	132	97	144	132	39	75
113	153	215	158	240	42	92
120	143	302	287	252	26	131
124	100	191	267	166	42	125
134	111	184	258	366	48	146
82	90	123	122	93	61	61
113	93	193	118	252	57	100
7	15	18	21	37	1	17
97	93	90	208	110	48	83
160	55	153	250	289	78	88
121	158	98	133	243	57	56
54	69	93	232	213	59	90
125	101	149	204	128	23	97
48	51	62	73	144	32	37
58	92	31	97	84	13	37
119	278	193	159	86	9	73
97	35	159	207	187	39	87
399	120	22	5	87	3	12
61	59	116	341	135	55	80
41	30	134	233	147	13	86
58	226	314	409	140	27	57
133	109	158	173	173	23	100
57	55	112	234	105	48	91
12	9	19	11	18	1	5
49	53	147	199	194	14	64
76	60	155	237	137	48	102
33	46	48	58	104	10	28
30	43	63	84	233	10	32
73	56	96	127	91	63	66
118	69	241	227	185	26	115
35	57	30	105	48	12	24
40	82	35	10	26	7	12
54	69	50	125	78	18	37
103	55	157	118	151	30	96
33	52	77	111	163	20	18
53	70	127	114	152	36	82
49	74	89	139	130	39	48
16	35	35	36	141	6	11
68	22	81	106	95	28	42
43	114	413	441	130	14	106
45	102	68	141	140	40	44
68	45	120	69	122	14	64
53	104	338	405	104	9	103
41	53	143	233	73	28	97
21	22	22	20	45	9	14
61	99	641	482	98	15	131
42	83	13	16	46	16	16
32	66	351	283	157	47	68
64	45	84	82	85	45	50
21	21	21	19	44	9	13
58	101	409	350	58	16	70
72	99	117	90	141	20	10
30	35	169	150	148	30	117
52	105	366	93	516	55	4
26	55	4	11	31	1	5
44	73	103	148	148	17	28
40	21	71	83	68	90	63
14	36	261	263	69	21	120
37	38	94	69	193	11	62

20	33	12	11	34	6	12
50	47	31	70	46	20	52
33	61	84	142	137	11	42
75	30	33	64	66	26	67
62	20	77	97	43	23	57
43	75	37	259	52	23	35
22	24	97	75	49	23	78
28	26	125	150	163	15	54
10	37	12	51	40	3	10
74	104	327	275	60	11	107
37	33	46	95	49	15	49
51	48	80	82	74	13	35
43	120	120	143	173	20	40
37	23	38	115	34	17	32
47	47	109	74	99	14	37
57	37	52	42	115	20	36
27	80	67	39	15	2	18
29	53	61	82	43	20	24
31	55	44	36	62	12	18
34	38	78	68	79	38	52
46	38	87	62	71	15	29
30	25	18	33	95	8	13
1	3	0	4	4	0	3
36	24	29	60	40	8	30
5	11	6	46	61	1	8
25	24	37	42	60	8	21
51	34	55	25	41	3	33
16	31	40	51	63	4	23
62	159	96	68	44	4	24
51	21	61	29	66	19	41
32	11	45	60	46	10	45
28	34	50	53	36	20	30
16	21	102	145	54	21	47
23	28	35	49	44	2	27
5	33	25	27	42	1	8
13	18	47	63	29	16	46
1	4	6	5	7	0	1
19	29	90	86	88	10	30
37	45	43	32	53	9	23
42	14	26	48	32	1	38
39	19	44	30	62	5	26
5	12	27	17	75	6	9
11	18	14	23	57	8	10
27	27	48	44	57	9	18
13	49	111	132	43	3	26
26	15	39	31	36	9	14
7	15	2	10	13	1	6
25	22	22	41	58	4	14
25	22	22	41	58	4	14
19	47	209	189	33	14	47
13	16	35	42	21	3	21
15	14	36	51	50	6	31
50	74	101	65	30	5	11
25	24	22	35	54	9	17
31	14	26	28	82	10	18
9	17	25	42	47	4	7
22	22	10	28	24	19	16
48	18	42	29	18	4	21
24	12	5	10	50	14	6

15	4	90	93	37	6	18
23	32	17	15	31	0	2
17	22	28	25	38	2	21
1	1	4	2	3	0	0
21	24	22	29	35	3	8
16	11	11	24	32	2	7
9	8	38	41	42	14	23
16	16	21	51	37	9	20
15	11	11	23	32	2	7
8	3	47	64	37	10	30
13	22	70	91	26	5	15
14	35	18	11	10	9	3
10	20	23	46	22	8	23
3	1	0	1	2	0	0
31	12	18	13	25	5	19
7	13	15	26	12	7	8
11	1	26	45	14	5	11
15	25	97	88	30	2	23
23	25	9	9	18	4	11
3	1	1	4	1	0	0
18	14	28	23	18	2	10
12	22	6	10	29	5	2
0	0	0	0	0	0	0
8	14	16	40	11	11	20
9	4	20	25	44	3	11
10	8	36	45	33	3	30
14	18	82	73	10	5	36
12	16	27	49	19	7	21
16	30	101	62	20	2	23
15	3	14	35	20	3	20
20	16	45	44	35	10	5
8	14	38	40	21	11	18
12	19	5	8	28	5	2
28	42	48	28	10	3	15
1	1	0	4	2	1	1
1	14	4	6	11	2	5
6	7	15	11	31	2	7
18	8	29	42	34	6	14
5	7	14	9	14	6	3
49	12	3	1	17	1	2
20	28	106	61	22	8	20
6	19	3	11	13	5	0
8	9	38	34	8	8	15
13	9	19	10	18	2	4
20	23	22	24	29	4	16
17	70	11	6	13	0	6
17	18	85	72	13	2	24
17	10	25	26	13	7	12
8	12	25	24	31	9	20
6	7	6	23	7	13	12
7	23	11	24	15	8	9
14	11	9	17	12	6	14
15	17	11	9	10	4	4
8	8	33	17	15	8	12
7	5	18	12	21	0	11
10	24	14	21	22	3	7
10	10	26	28	9	6	8
4	7	2	2	4	1	1
1	5	6	4	6	1	3

7	7	4	13	8	2	6
3	6	17	18	17	2	10
8	5	9	18	10	6	6
6	3	29	21	15	3	13
8	24	18	14	13	0	7
12	6	13	14	32	7	8
10	8	25	26	18	9	10
2	7	4	8	9	2	3
11	8	12	21	17	4	14
18	16	81	54	14	3	19
6	7	27	33	16	15	18
19	25	38	20	14	2	15
9	5	1	11	21	0	0
8	7	20	18	27	6	8
2	8	6	19	21	4	10
4	5	14	27	25	1	12
16	2	7	9	19	7	8
7	10	17	22	17	1	10
5	2	4	1	5	0	1
8	6	17	19	22	8	5
5	15	4	7	6	0	0
8	5	9	29	8	6	9
10	13	14	19	19	8	6
5	8	16	15	20	8	5
4	0	33	31	37	5	43
8	18	10	18	7	1	5
4	9	5	20	6	2	5
5	7	10	12	20	15	6
10	7	18	14	19	2	14
7	7	9	20	15	4	15
7	0	8	22	13	10	8
5	7	19	9	21	4	36
8	4	3	11	15	1	4
4	6	8	12	3	1	1
7	7	5	8	6	0	5
5	11	8	11	25	7	2
5	4	16	19	14	7	9
6	8	5	3	11	2	7
2	0	3	6	13	6	13
0	3	1	1	3	0	2
2	4	3	5	6	7	5
10	4	4	10	11	1	5
6	8	8	3	13	2	1
5	9	16	13	14	5	9
1	16	7	1	7	1	1
3	2	6	6	12	1	3
4	5	9	5	7	0	2
6	1	2	3	11	1	3
8	4	20	5	9	0	3
4	6	11	10	7	5	4
2	6	2	10	3	1	6
5	5	17	15	11	2	4
3	4	1	1	5	0	0
1	1	2	1	7	0	0
7	3	13	4	12	3	7
2	8	5	9	9	0	4
1	14	26	2	12	4	1
3	3	10	11	11	4	2
3	5	8	9	9	2	4

9	5	3	1	3	8	3
2	1	1	3	1	0	0
9	7	26	35	1	4	8
7	3	7	8	10	2	4
4	1	12	14	2	4	5
3	3	9	11	5	3	1
4	3	10	11	6	10	6
3	1	5	7	8	7	8
3	8	4	6	7	3	1
6	6	32	22	6	1	5
0	1	8	9	14	2	2
6	1	10	7	8	6	6
6	4	4	1	6	2	1
3	4	2	7	8	2	9
2	5	9	8	10	3	3
7	3	0	0	12	1	0
0	0	4	6	30	0	0
1	2	4	7	3	2	4
5	18	11	14	5	1	3
2	5	5	1	2	1	2
4	2	6	11	5	0	3
4	8	13	10	10	4	2
1	1	0	0	3	0	2
5	6	7	5	6	1	2
6	1	5	10	11	0	5
12	0	4	4	7	0	1
5	4	1	3	7	2	0
1	1	10	9	14	3	9
4	2	0	3	6	0	1
1	4	1	1	1	2	2
4	2	10	6	12	0	10
1	4	20	25	4	1	5
2	4	10	8	6	2	5
4	1	3	4	8	3	1
3	3	11	5	2	6	3
4	0	7	1	5	2	3
0	1	0	1	1	0	0
0	0	2	0	2	0	0
0	1	0	0	0	0	0
3	2	4	1	2	0	0
7	6	3	3	7	1	1
3	6	11	15	6	0	6
2	5	3	13	6	0	9
1	6	11	8	10	5	4
0	1	0	0	0	0	0
3	13	4	8	6	0	1
3	5	17	13	7	0	1
0	2	3	0	3	0	0
1	3	8	6	8	1	4
3	3	8	12	5	1	4
3	1	7	6	2	1	3
2	1	0	0	0	0	0
12	4	10	0	3	1	1
0	3	2	4	3	2	1
4	1	5	8	3	1	5
2	6	7	2	3	0	1
0	2	0	0	0	0	0
3	9	6	3	4	1	2
6	3	4	9	4	0	2

2	1	2	2	2	2	0
7	5	3	3	3	2	2
2	0	9	10	5	1	12
4	0	5	7	5	4	1
3	3	2	3	3	2	1
3	3	6	0	3	2	1
1	3	7	20	6	0	3
0	1	2	6	0	0	1
1	0	4	5	8	1	2
1	2	8	10	2	3	7
3	4	7	2	10	0	2
4	2	2	5	2	2	4
0	9	5	7	6	0	1
1	4	1	0	1	1	0
5	1	3	4	6	0	3
2	0	4	4	1	2	1
1	1	3	5	12	0	3
1	5	5	5	3	2	6
0	3	2	0	2	1	1
0	6	5	6	1	0	3
0	0	2	3	3	2	3
8	4	3	3	7	2	2
2	0	0	4	6	0	3
1	0	3	7	4	0	3
2	1	6	15	12	2	4
1	2	5	10	1	3	4
8	1	3	3	7	1	1
2	1	4	4	5	2	1
6	2	1	1	7	3	1
2	1	3	5	1	0	3
1	4	4	4	1	1	1
0	1	0	1	0	0	0
3	2	6	8	11	0	2
1	3	3	1	1	0	1
1	0	5	3	2	2	1
0	2	3	2	4	0	0
0	0	8	0	3	2	1
0	1	5	7	2	2	2
1	2	1	0	1	0	1
2	2	1	3	0	0	1
2	3	0	2	3	0	4
5	2	4	0	2	0	2
0	0	5	9	9	2	3
0	1	17	16	5	0	8
0	0	0	1	3	1	0
2	0	1	5	4	0	0
3	1	1	0	2	0	0
0	0	0	0	0	0	0
0	1	0	0	6	0	0
1	0	1	5	4	0	2
1	1	4	1	7	0	6
0	3	3	5	4	1	2
2	1	4	7	3	1	2
2	2	8	5	3	1	4
0	0	1	0	0	0	0
0	0	0	1	1	1	0
2	4	2	1	2	1	2
0	2	5	4	2	2	3
1	0	1	2	1	2	5

0	0	2	9	3	0	7
0	0	4	5	4	0	1
1	2	1	5	7	2	1
1	1	1	1	2	0	1
1	2	2	1	1	1	1
1	5	6	4	5	0	0
1	3	1	4	1	0	0
0	2	0	2	2	1	0
0	2	9	6	6	1	1
5	0	3	1	5	3	4
3	0	6	4	2	1	1
1	0	4	3	2	1	1
1	0	0	2	1	0	3
0	1	4	5	2	5	0
0	0	1	1	0	1	2
1	1	1	2	2	1	2
0	1	5	3	5	1	1
2	3	1	5	2	0	0
0	0	0	1	0	0	0
0	1	0	4	0	0	1
3	0	1	1	3	1	0
1	1	1	1	2	2	2
0	3	0	1	2	1	1
3	4	6	2	5	0	3
4	3	2	4	3	2	0
2	2	2	5	7	2	0
1	1	2	4	4	0	0
4	0	2	3	3	0	1
3	0	0	1	5	0	1
2	0	2	2	0	0	0
1	1	1	0	4	0	0
6	0	1	0	0	0	0
3	3	2	3	1	1	2
7	0	1	0	1	0	2
7	1	3	1	0	2	1
3	0	0	10	0	0	0
0	3	3	3	2	0	1
1	1	2	1	1	0	2

0.64	0.04	0.25	0.89	0.04	0.21	0.21
15	34	25	9	35	27	28
1	1	1	1	1	1	1
50	51	52	53	54	55	56
23	24	25	26	27	28	29
E_5	E_6	F_1	F_35	F_36	F_37	F_38
CHR_NP	CHR_NP	CHR_NP	CHR_NP	CHR_NP	CHR_NP	CHR_NP
1597470	1583268	2592561	3565545	2585170	976547	1738984
CHR_NP	CHR_NP	CHR_NP	CHR_NP	CHR_NP	CHR_NP	CHR_NP
539941	452076	1019114	1755132	781741	473210	768215
99635	107343	314034	340046	296230	77703	201610
264828	202140	216370	77296	194779	50911	97414
66480	55579	193721	338825	202546	74728	131317
138176	149924	132168	131189	135975	59576	111341

43150	101571	22591	56016	78288	14801	32212
77544	75208	90366	90072	153035	26823	51941
9097	19477	2885	5235	8559	768	2104
30750	66644	21216	31586	66828	10588	16579
20533	25326	33662	60382	37679	15012	25734
11806	16643	37442	58644	47412	13953	14827
34432	40006	45021	44732	45670	12668	24311
12897	13780	28108	31789	45628	10437	10434
14905	12339	28906	62313	26495	11105	26421
3826	8625	3619	7407	17517	4647	2353
21105	13046	29277	27797	31237	13167	9460
4982	9159	5221	6277	15435	2270	2745
11358	15361	16006	20862	29022	4473	8323
8451	11039	8233	7956	16360	2743	5304
10001	8617	19882	18075	15799	5800	12796
11595	7424	13305	14518	15608	3396	10456
5656	6032	10784	17013	14339	2358	6331
3364	3884	13575	15150	15827	4989	8178
4908	4100	6108	5207	16756	1222	3150
3201	5623	2676	2223	6081	1028	1837
10754	8504	15410	18819	12991	3995	10054
6129	4824	16793	22216	12082	4650	8405
3090	3810	1751	7872	3113	1833	766
5156	6291	11932	17314	13790	3271	6517
3944	6196	22369	25514	13858	4918	8664
9462	9551	4731	4426	12369	2081	2658
4324	3085	8943	17338	9925	3677	6526
2963	4434	2540	2521	5618	786	1282
722	1495	1575	2065	3334	537	549
4813	5923	11383	16461	13215	3026	6156
3362	3311	6902	16417	10148	1342	7627
2828	5461	5362	8173	5067	2086	3342
2907	4450	4987	4628	4906	1909	3313
2550	655	13960	11242	5566	1962	7611
3146	1415	3960	4905	3092	5354	2915
3186	2075	6041	7186	6246	1073	2774
5113	4677	3960	3350	4246	2177	2881
3727	2842	6730	9416	6452	1494	4811
447	1157	341	504	891	212	289
4984	4932	2963	1799	4632	598	2122
3568	3862	7118	6858	5056	3186	2944
1755	3380	7296	6820	6085	1422	2590
2856	2302	4118	4403	4612	1915	2580
238	324	192	202	302	126	111
1056	961	742	1113	1973	318	373
2139	3436	17555	14345	2557	601	5781
1369	1445	2132	2976	6477	409	1087
1628	1537	3421	5234	3637	565	2223
1017	892	1714	1390	2111	949	1193
2342	1151	2854	2421	1850	546	1509
144	292	154	214	272	61	88
1736	1442	2190	2551	3851	530	1617
2808	1498	1234	1030	2129	438	803
2131	1524	3269	1644	3523	844	1334
982	1044	3947	3551	3532	566	4142
882	1673	3917	3851	3053	630	1099
1766	1312	894	1101	1675	297	668
66	408	193	269	194	49	145
182	169	162	147	103	36	52

924	817	889	2726	739	1151	933
641	1102	773	1478	1802	176	462
617	1420	495	426	1076	132	207
271	586	321	754	809	80	200
924	1020	1142	1539	2826	156	618
552	550	1102	2470	619	557	497
846	907	2058	2575	1968	800	1021
755	691	1163	2735	1136	1147	1340
92	329	162	370	576	114	85
682	722	977	1460	1111	336	672
1412	1097	1119	601	1931	249	659
734	1217	4399	4764	1152	298	1655
776	646	1496	1583	1483	332	1087
277	349	2328	4121	1625	424	2238
529	508	1474	1671	1732	531	635
424	988	2117	2383	1721	236	768
620	644	478	748	824	200	493
857	692	1845	1459	1299	405	586
583	485	1575	2105	859	519	984
285	449	384	315	1205	104	101
298	282	920	694	926	161	528
547	646	1285	1177	1147	120	431
565	552	1561	1502	1142	677	586
186	557	106	170	286	62	110
593	601	622	620	1201	397	272
209	213	1078	1863	773	509	749
381	918	693	825	668	446	661
23	36	36	84	25	8	17
629	374	640	441	827	179	274
339	996	490	617	691	103	277
121	219	84	167	329	31	64
448	482	196	215	689	105	109
305	293	877	897	634	421	432
344	285	517	697	524	260	312
428	350	727	523	454	179	461
47	173	58	125	237	23	42
188	191	683	962	465	50	359
427	412	703	1006	760	181	461
373	262	690	379	677	270	303
147	240	251	398	452	84	161
137	291	117	193	272	62	105
82	135	69	100	131	27	53
302	315	655	721	723	139	230
295	297	449	589	953	97	265
145	247	181	313	421	47	98
130	238	132	213	405	24	84
348	524	163	131	270	96	174
477	272	542	512	688	158	264
141	135	382	512	578	29	211
103	179	94	282	211	26	125
500	337	362	87	404	88	171
98	123	596	599	490	173	272
144	226	119	202	194	57	81
113	351	286	309	640	111	118
113	351	286	309	640	111	118
109	118	203	279	305	27	85
228	194	233	373	339	52	165
254	278	176	508	437	182	212
355	632	386	277	162	35	160

160	148	121	142	312	51	120
26	45	33	22	71	5	15
193	109	316	490	442	217	130
161	103	368	223	330	42	345
247	261	310	326	367	76	178
106	149	628	632	248	48	184
112	85	313	675	304	145	168
67	48	453	736	436	77	457
120	208	156	110	319	76	61
73	203	368	354	402	42	135
115	113	339	426	331	43	169
72	116	95	57	124	31	25
114	110	268	239	282	60	99
112	85	107	263	89	12	89
142	132	97	144	132	39	75
113	153	215	158	240	42	92
120	143	302	287	252	26	131
124	100	191	267	166	42	125
134	111	184	258	366	48	146
82	90	123	122	93	61	61
113	93	193	118	252	57	100
7	15	18	21	37	1	17
97	93	90	208	110	48	83
160	55	153	250	289	78	88
121	158	98	133	243	57	56
54	69	93	232	213	59	90
125	101	149	204	128	23	97
48	51	62	73	144	32	37
58	92	31	97	84	13	37
119	278	193	159	86	9	73
97	35	159	207	187	39	87
399	120	22	5	87	3	12
61	59	116	341	135	55	80
41	30	134	233	147	13	86
58	226	314	409	140	27	57
133	109	158	173	173	23	100
57	55	112	234	105	48	91
12	9	19	11	18	1	5
49	53	147	199	194	14	64
76	60	155	237	137	48	102
33	46	48	58	104	10	28
30	43	63	84	233	10	32
73	56	96	127	91	63	66
118	69	241	227	185	26	115
35	57	30	105	48	12	24
40	82	35	10	26	7	12
54	69	50	125	78	18	37
103	55	157	118	151	30	96
33	52	77	111	163	20	18
53	70	127	114	152	36	82
49	74	89	139	130	39	48
16	35	35	36	141	6	11
68	22	81	106	95	28	42
43	114	413	441	130	14	106
45	102	68	141	140	40	44
68	45	120	69	122	14	64
53	104	338	405	104	9	103
41	53	143	233	73	28	97
21	22	22	20	45	9	14

61	99	641	482	98	15	131
42	83	13	16	46	16	16
32	66	351	283	157	47	68
64	45	84	82	85	45	50
21	21	21	19	44	9	13
58	101	409	350	58	16	70
72	99	117	90	141	20	10
30	35	169	150	148	30	117
52	105	366	93	516	55	4
26	55	4	11	31	1	5
44	73	103	148	148	17	28
40	21	71	83	68	90	63
14	36	261	263	69	21	120
37	38	94	69	193	11	62
20	33	12	11	34	6	12
50	47	31	70	46	20	52
33	61	84	142	137	11	42
75	30	33	64	66	26	67
62	20	77	97	43	23	57
43	75	37	259	52	23	35
22	24	97	75	49	23	78
28	26	125	150	163	15	54
10	37	12	51	40	3	10
74	104	327	275	60	11	107
37	33	46	95	49	15	49
51	48	80	82	74	13	35
43	120	120	143	173	20	40
37	23	38	115	34	17	32
47	47	109	74	99	14	37
57	37	52	42	115	20	36
27	80	67	39	15	2	18
29	53	61	82	43	20	24
31	55	44	36	62	12	18
34	38	78	68	79	38	52
46	38	87	62	71	15	29
30	25	18	33	95	8	13
1	3	0	4	4	0	3
36	24	29	60	40	8	30
5	11	6	46	61	1	8
25	24	37	42	60	8	21
51	34	55	25	41	3	33
16	31	40	51	63	4	23
62	159	96	68	44	4	24
51	21	61	29	66	19	41
32	11	45	60	46	10	45
28	34	50	53	36	20	30
16	21	102	145	54	21	47
23	28	35	49	44	2	27
5	33	25	27	42	1	8
13	18	47	63	29	16	46
1	4	6	5	7	0	1
19	29	90	86	88	10	30
37	45	43	32	53	9	23
42	14	26	48	32	1	38
39	19	44	30	62	5	26
5	12	27	17	75	6	9
11	18	14	23	57	8	10
27	27	48	44	57	9	18
13	49	111	132	43	3	26

26	15	39	31	36	9	14
7	15	2	10	13	1	6
25	22	22	41	58	4	14
25	22	22	41	58	4	14
19	47	209	189	33	14	47
13	16	35	42	21	3	21
15	14	36	51	50	6	31
50	74	101	65	30	5	11
25	24	22	35	54	9	17
31	14	26	28	82	10	18
9	17	25	42	47	4	7
22	22	10	28	24	19	16
48	18	42	29	18	4	21
24	12	5	10	50	14	6
15	4	90	93	37	6	18
23	32	17	15	31	0	2
17	22	28	25	38	2	21
1	1	4	2	3	0	0
21	24	22	29	35	3	8
16	11	11	24	32	2	7
9	8	38	41	42	14	23
16	16	21	51	37	9	20
15	11	11	23	32	2	7
8	3	47	64	37	10	30
13	22	70	91	26	5	15
14	35	18	11	10	9	3
10	20	23	46	22	8	23
3	1	0	1	2	0	0
31	12	18	13	25	5	19
7	13	15	26	12	7	8
11	1	26	45	14	5	11
15	25	97	88	30	2	23
23	25	9	9	18	4	11
3	1	1	4	1	0	0
18	14	28	23	18	2	10
12	22	6	10	29	5	2
0	0	0	0	0	0	0
8	14	16	40	11	11	20
9	4	20	25	44	3	11
10	8	36	45	33	3	30
14	18	82	73	10	5	36
12	16	27	49	19	7	21
16	30	101	62	20	2	23
15	3	14	35	20	3	20
20	16	45	44	35	10	5
8	14	38	40	21	11	18
12	19	5	8	28	5	2
28	42	48	28	10	3	15
1	1	0	4	2	1	1
1	14	4	6	11	2	5
6	7	15	11	31	2	7
18	8	29	42	34	6	14
5	7	14	9	14	6	3
49	12	3	1	17	1	2
20	28	106	61	22	8	20
6	19	3	11	13	5	0
8	9	38	34	8	8	15
13	9	19	10	18	2	4
20	23	22	24	29	4	16

17	70	11	6	13	0	6
17	18	85	72	13	2	24
17	10	25	26	13	7	12
8	12	25	24	31	9	20
6	7	6	23	7	13	12
7	23	11	24	15	8	9
14	11	9	17	12	6	14
15	17	11	9	10	4	4
8	8	33	17	15	8	12
7	5	18	12	21	0	11
10	24	14	21	22	3	7
10	10	26	28	9	6	8
4	7	2	2	4	1	1
1	5	6	4	6	1	3
7	7	4	13	8	2	6
3	6	17	18	17	2	10
8	5	9	18	10	6	6
6	3	29	21	15	3	13
8	24	18	14	13	0	7
12	6	13	14	32	7	8
10	8	25	26	18	9	10
2	7	4	8	9	2	3
11	8	12	21	17	4	14
18	16	81	54	14	3	19
6	7	27	33	16	15	18
19	25	38	20	14	2	15
9	5	1	11	21	0	0
8	7	20	18	27	6	8
2	8	6	19	21	4	10
4	5	14	27	25	1	12
16	2	7	9	19	7	8
7	10	17	22	17	1	10
5	2	4	1	5	0	1
8	6	17	19	22	8	5
5	15	4	7	6	0	0
8	5	9	29	8	6	9
10	13	14	19	19	8	6
5	8	16	15	20	8	5
4	0	33	31	37	5	43
8	18	10	18	7	1	5
4	9	5	20	6	2	5
5	7	10	12	20	15	6
10	7	18	14	19	2	14
7	7	9	20	15	4	15
7	0	8	22	13	10	8
5	7	19	9	21	4	36
8	4	3	11	15	1	4
4	6	8	12	3	1	1
7	7	5	8	6	0	5
5	11	8	11	25	7	2
5	4	16	19	14	7	9
6	8	5	3	11	2	7
2	0	3	6	13	6	13
0	3	1	1	3	0	2
2	4	3	5	6	7	5
10	4	4	10	11	1	5
6	8	8	3	13	2	1
5	9	16	13	14	5	9
1	16	7	1	7	1	1

3	2	6	6	12	1	3
4	5	9	5	7	0	2
6	1	2	3	11	1	3
8	4	20	5	9	0	3
4	6	11	10	7	5	4
2	6	2	10	3	1	6
5	5	17	15	11	2	4
3	4	1	1	5	0	0
1	1	2	1	7	0	0
7	3	13	4	12	3	7
2	8	5	9	9	0	4
1	14	26	2	12	4	1
3	3	10	11	11	4	2
3	5	8	9	9	2	4
9	5	3	1	3	8	3
2	1	1	3	1	0	0
9	7	26	35	1	4	8
7	3	7	8	10	2	4
4	1	12	14	2	4	5
3	3	9	11	5	3	1
4	3	10	11	6	10	6
3	1	5	7	8	7	8
3	8	4	6	7	3	1
6	6	32	22	6	1	5
0	1	8	9	14	2	2
6	1	10	7	8	6	6
6	4	4	1	6	2	1
3	4	2	7	8	2	9
2	5	9	8	10	3	3
7	3	0	0	12	1	0
0	0	4	6	30	0	0
1	2	4	7	3	2	4
5	18	11	14	5	1	3
2	5	5	1	2	1	2
4	2	6	11	5	0	3
4	8	13	10	10	4	2
1	1	0	0	3	0	2
5	6	7	5	6	1	2
6	1	5	10	11	0	5
12	0	4	4	7	0	1
5	4	1	3	7	2	0
1	1	10	9	14	3	9
4	2	0	3	6	0	1
1	4	1	1	1	2	2
4	2	10	6	12	0	10
1	4	20	25	4	1	5
2	4	10	8	6	2	5
4	1	3	4	8	3	1
3	3	11	5	2	6	3
4	0	7	1	5	2	3
0	1	0	1	1	0	0
0	0	2	0	2	0	0
0	1	0	0	0	0	0
3	2	4	1	2	0	0
7	6	3	3	7	1	1
3	6	11	15	6	0	6
2	5	3	13	6	0	9
1	6	11	8	10	5	4
0	1	0	0	0	0	0

3	13	4	8	6	0	1
3	5	17	13	7	0	1
0	2	3	0	3	0	0
1	3	8	6	8	1	4
3	3	8	12	5	1	4
3	1	7	6	2	1	3
2	1	0	0	0	0	0
12	4	10	0	3	1	1
0	3	2	4	3	2	1
4	1	5	8	3	1	5
2	6	7	2	3	0	1
0	2	0	0	0	0	0
3	9	6	3	4	1	2
6	3	4	9	4	0	2
2	1	2	2	2	2	0
7	5	3	3	3	2	2
2	0	9	10	5	1	12
4	0	5	7	5	4	1
3	3	2	3	3	2	1
3	3	6	0	3	2	1
1	3	7	20	6	0	3
0	1	2	6	0	0	1
1	0	4	5	8	1	2
1	2	8	10	2	3	7
3	4	7	2	10	0	2
4	2	2	5	2	2	4
0	9	5	7	6	0	1
1	4	1	0	1	1	0
5	1	3	4	6	0	3
2	0	4	4	1	2	1
1	1	3	5	12	0	3
1	5	5	5	3	2	6
0	3	2	0	2	1	1
0	6	5	6	1	0	3
0	0	2	3	3	2	3
8	4	3	3	7	2	2
2	0	0	4	6	0	3
1	0	3	7	4	0	3
2	1	6	15	12	2	4
1	2	5	10	1	3	4
8	1	3	3	7	1	1
2	1	4	4	5	2	1
6	2	1	1	7	3	1
2	1	3	5	1	0	3
1	4	4	4	1	1	1
0	1	0	1	0	0	0
3	2	6	8	11	0	2
1	3	3	1	1	0	1
1	0	5	3	2	2	1
0	2	3	2	4	0	0
0	0	8	0	3	2	1
0	1	5	7	2	2	2
1	2	1	0	1	0	1
2	2	1	3	0	0	1
2	3	0	2	3	0	4
5	2	4	0	2	0	2
0	0	5	9	9	2	3
0	1	17	16	5	0	8
0	0	0	1	3	1	0

2	0	1	5	4	0	0
3	1	1	0	2	0	0
0	0	0	0	0	0	0
0	1	0	0	6	0	0
1	0	1	5	4	0	2
1	1	4	1	7	0	6
0	3	3	5	4	1	2
2	1	4	7	3	1	2
2	2	8	5	3	1	4
0	0	1	0	0	0	0
0	0	0	1	1	1	0
2	4	2	1	2	1	2
0	2	5	4	2	2	3
1	0	1	2	1	2	5
0	0	2	9	3	0	7
0	0	4	5	4	0	1
1	2	1	5	7	2	1
1	1	1	1	2	0	1
1	2	2	1	1	1	1
1	5	6	4	5	0	0
1	3	1	4	1	0	0
0	2	0	2	2	1	0
0	2	9	6	6	1	1
5	0	3	1	5	3	4
3	0	6	4	2	1	1
1	0	4	3	2	1	1
1	0	0	2	1	0	3
0	1	4	5	2	5	0
0	0	1	1	0	1	2
1	1	1	2	2	1	2
0	1	5	3	5	1	1
2	3	1	5	2	0	0
0	0	0	1	0	0	0
0	1	0	4	0	0	1
3	0	1	1	3	1	0
1	1	1	1	2	2	2
0	3	0	1	2	1	1
3	4	6	2	5	0	3
4	3	2	4	3	2	0
2	2	2	5	7	2	0
1	1	2	4	4	0	0
4	0	2	3	3	0	1
3	0	0	1	5	0	1
2	0	2	2	0	0	0
1	1	1	0	4	0	0
6	0	1	0	0	0	0
3	3	2	3	1	1	2
7	0	1	0	1	0	2
7	1	3	1	0	2	1
3	0	0	10	0	0	0
0	3	3	3	2	0	1
1	1	2	1	1	0	2
49390	48686	79418	110583	79023	30568	53993
107346	90205	191944	321517	150427	86284	142593
388	379	357	348	353	427	397
CHR_NP	CHR_NP	CHR_NP	CHR_NP	CHR_NP	CHR_NP	CHR_NP
CHR_NP	CHR_NP	CHR_NP	CHR_NP	CHR_NP	CHR_NP	CHR_NP

50	51	52	53	54	55	56
23	24	25	26	27	28	29
CHR_NP	CHR_NP	CHR_NP	CHR_NP	CHR_NP	CHR_NP	CHR_NP
E_5	E_6	F_1	F_35	F_36	F_37	F_38
1597470	1583268	2592561	3565545	2585170	976547	1738984
CHR_NP	CHR_NP	CHR_NP	CHR_NP	CHR_NP	CHR_NP	CHR_NP
10.93	9.29	12.83	15.87	9.89	15.48	14.23
2.02	2.20	3.95	3.08	3.75	2.54	3.73
5.36	4.15	2.72	0.70	2.46	1.67	1.80
1.35	1.14	2.44	3.06	2.56	2.44	2.43
2.80	3.08	1.66	1.19	1.72	1.95	2.06
0.87	2.09	0.28	0.51	0.99	0.48	0.60
1.57	1.54	1.14	0.81	1.94	0.88	0.96
0.18	0.40	0.04	0.05	0.11	0.03	0.04
0.62	1.37	0.27	0.29	0.85	0.35	0.31
0.42	0.52	0.42	0.55	0.48	0.49	0.48
0.24	0.34	0.47	0.53	0.60	0.46	0.27
0.70	0.82	0.57	0.40	0.58	0.41	0.45
0.26	0.28	0.35	0.29	0.58	0.34	0.19
0.30	0.25	0.36	0.56	0.34	0.36	0.49
0.08	0.18	0.05	0.07	0.22	0.15	0.04
0.43	0.27	0.37	0.25	0.40	0.43	0.18
0.10	0.19	0.07	0.06	0.20	0.07	0.05
0.23	0.32	0.20	0.19	0.37	0.15	0.15
0.17	0.23	0.10	0.07	0.21	0.09	0.10
0.20	0.18	0.25	0.16	0.20	0.19	0.24
0.23	0.15	0.17	0.13	0.20	0.11	0.19
0.11	0.12	0.14	0.15	0.18	0.08	0.12
0.07	0.08	0.17	0.14	0.20	0.16	0.15
0.10	0.08	0.08	0.05	0.21	0.04	0.06
0.06	0.12	0.03	0.02	0.08	0.03	0.03
0.22	0.17	0.19	0.17	0.16	0.13	0.19
0.12	0.10	0.21	0.20	0.15	0.15	0.16
0.06	0.08	0.02	0.07	0.04	0.06	0.01
0.10	0.13	0.15	0.16	0.17	0.11	0.12
0.08	0.13	0.28	0.23	0.18	0.16	0.16
0.19	0.20	0.06	0.04	0.16	0.07	0.05
0.09	0.06	0.11	0.16	0.13	0.12	0.12
0.06	0.09	0.03	0.02	0.07	0.03	0.02
0.01	0.03	0.02	0.02	0.04	0.02	0.01
0.10	0.12	0.14	0.15	0.17	0.10	0.11
0.07	0.07	0.09	0.15	0.13	0.04	0.14
0.06	0.11	0.07	0.07	0.06	0.07	0.06
0.06	0.09	0.06	0.04	0.06	0.06	0.06
0.05	0.01	0.18	0.10	0.07	0.06	0.14
0.06	0.03	0.05	0.04	0.04	0.18	0.05
0.06	0.04	0.08	0.06	0.08	0.04	0.05
0.10	0.10	0.05	0.03	0.05	0.07	0.05
0.08	0.06	0.08	0.09	0.08	0.05	0.09
0.01	0.02	0.00	0.00	0.01	0.01	0.01
0.10	0.10	0.04	0.02	0.06	0.02	0.04
0.07	0.08	0.09	0.06	0.06	0.10	0.05
0.04	0.07	0.09	0.06	0.08	0.05	0.05
0.06	0.05	0.05	0.04	0.06	0.06	0.05
0.00	0.01	0.00	0.00	0.00	0.00	0.00
0.02	0.02	0.01	0.01	0.02	0.01	0.01
0.04	0.07	0.22	0.13	0.03	0.02	0.11
0.03	0.03	0.03	0.03	0.08	0.01	0.02

0.03	0.03	0.04	0.05	0.05	0.02	0.04
0.02	0.02	0.02	0.01	0.03	0.03	0.02
0.05	0.02	0.04	0.02	0.02	0.02	0.03
0.00	0.01	0.00	0.00	0.00	0.00	0.00
0.04	0.03	0.03	0.02	0.05	0.02	0.03
0.06	0.03	0.02	0.01	0.03	0.01	0.01
0.04	0.03	0.04	0.01	0.04	0.03	0.02
0.02	0.02	0.05	0.03	0.04	0.02	0.08
0.02	0.03	0.05	0.03	0.04	0.02	0.02
0.04	0.03	0.01	0.01	0.02	0.01	0.01
0.00	0.01	0.00	0.00	0.00	0.00	0.00
0.00	0.00	0.00	0.00	0.00	0.00	0.00
0.02	0.02	0.01	0.02	0.01	0.04	0.02
0.01	0.02	0.01	0.01	0.02	0.01	0.01
0.01	0.03	0.01	0.00	0.01	0.00	0.00
0.01	0.01	0.00	0.01	0.01	0.00	0.00
0.02	0.02	0.01	0.01	0.04	0.01	0.01
0.01	0.01	0.01	0.02	0.01	0.02	0.01
0.02	0.02	0.03	0.02	0.02	0.03	0.02
0.02	0.01	0.01	0.02	0.01	0.04	0.02
0.00	0.01	0.00	0.00	0.01	0.00	0.00
0.01	0.01	0.01	0.01	0.01	0.01	0.01
0.03	0.02	0.01	0.01	0.02	0.01	0.01
0.01	0.02	0.06	0.04	0.01	0.01	0.03
0.02	0.01	0.02	0.01	0.02	0.01	0.02
0.01	0.01	0.03	0.04	0.02	0.01	0.04
0.01	0.01	0.02	0.02	0.02	0.02	0.01
0.01	0.02	0.03	0.02	0.02	0.01	0.01
0.01	0.01	0.01	0.01	0.01	0.01	0.01
0.02	0.01	0.02	0.01	0.02	0.01	0.01
0.01	0.01	0.02	0.02	0.01	0.02	0.02
0.01	0.01	0.00	0.00	0.02	0.00	0.00
0.01	0.01	0.01	0.01	0.01	0.01	0.01
0.01	0.01	0.02	0.01	0.01	0.00	0.01
0.01	0.01	0.02	0.01	0.01	0.02	0.01
0.00	0.01	0.00	0.00	0.00	0.00	0.00
0.01	0.01	0.01	0.01	0.02	0.01	0.01
0.00	0.00	0.01	0.02	0.01	0.02	0.01
0.01	0.02	0.01	0.01	0.01	0.01	0.01
0.00	0.00	0.00	0.00	0.00	0.00	0.00
0.01	0.01	0.01	0.00	0.01	0.01	0.01
0.01	0.02	0.01	0.01	0.01	0.00	0.01
0.00	0.00	0.00	0.00	0.00	0.00	0.00
0.01	0.01	0.00	0.00	0.01	0.00	0.00
0.01	0.01	0.01	0.01	0.01	0.01	0.01
0.01	0.01	0.01	0.01	0.01	0.01	0.01
0.01	0.01	0.01	0.00	0.01	0.01	0.01
0.00	0.00	0.00	0.00	0.00	0.00	0.00
0.00	0.00	0.01	0.01	0.01	0.00	0.01
0.01	0.01	0.01	0.01	0.01	0.01	0.01
0.01	0.01	0.01	0.00	0.01	0.01	0.01
0.00	0.00	0.00	0.00	0.01	0.00	0.00
0.00	0.01	0.00	0.00	0.00	0.00	0.00
0.00	0.00	0.00	0.00	0.00	0.00	0.00
0.01	0.01	0.01	0.01	0.01	0.00	0.00
0.01	0.01	0.01	0.01	0.01	0.00	0.00
0.00	0.01	0.00	0.00	0.01	0.00	0.00
0.00	0.00	0.00	0.00	0.01	0.00	0.00
0.01	0.01	0.00	0.00	0.00	0.00	0.00

0.01	0.01	0.01	0.00	0.01	0.01	0.00
0.00	0.00	0.00	0.00	0.01	0.00	0.00
0.00	0.00	0.00	0.00	0.00	0.00	0.00
0.01	0.01	0.00	0.00	0.01	0.00	0.00
0.00	0.00	0.01	0.01	0.01	0.01	0.01
0.00	0.00	0.00	0.00	0.00	0.00	0.00
0.00	0.01	0.00	0.00	0.01	0.00	0.00
0.00	0.01	0.00	0.00	0.01	0.00	0.00
0.00	0.00	0.00	0.00	0.00	0.00	0.00
0.00	0.00	0.00	0.00	0.00	0.00	0.00
0.01	0.01	0.00	0.00	0.01	0.01	0.00
0.01	0.01	0.00	0.00	0.00	0.00	0.00
0.00	0.00	0.00	0.00	0.00	0.00	0.00
0.00	0.00	0.00	0.00	0.00	0.00	0.00
0.00	0.00	0.00	0.00	0.01	0.01	0.00
0.00	0.00	0.00	0.00	0.00	0.00	0.01
0.01	0.01	0.00	0.00	0.00	0.00	0.00
0.00	0.00	0.01	0.01	0.00	0.00	0.00
0.00	0.00	0.00	0.01	0.00	0.00	0.00
0.00	0.00	0.01	0.01	0.01	0.00	0.01
0.00	0.00	0.00	0.00	0.00	0.00	0.00
0.00	0.00	0.00	0.00	0.01	0.00	0.00
0.00	0.00	0.00	0.00	0.00	0.00	0.00
0.00	0.00	0.00	0.00	0.00	0.00	0.00
0.00	0.00	0.00	0.00	0.00	0.00	0.00
0.00	0.00	0.00	0.00	0.00	0.00	0.00
0.00	0.00	0.00	0.00	0.00	0.00	0.00
0.00	0.00	0.00	0.00	0.00	0.00	0.00
0.00	0.00	0.00	0.00	0.00	0.00	0.00
0.00	0.00	0.00	0.00	0.00	0.00	0.00
0.00	0.00	0.00	0.00	0.00	0.00	0.00
0.00	0.00	0.00	0.00	0.00	0.00	0.00
0.00	0.00	0.00	0.00	0.00	0.00	0.00
0.00	0.00	0.00	0.00	0.00	0.00	0.00
0.00	0.00	0.00	0.00	0.00	0.00	0.00
0.00	0.00	0.00	0.00	0.00	0.00	0.00
0.00	0.00	0.00	0.00	0.00	0.00	0.00
0.00	0.00	0.00	0.00	0.00	0.00	0.00
0.00	0.00	0.00	0.00	0.00	0.00	0.00
0.00	0.00	0.00	0.00	0.00	0.00	0.00
0.00	0.00	0.00	0.00	0.00	0.00	0.00
0.00	0.01	0.00	0.00	0.00	0.00	0.00
0.00	0.00	0.00	0.00	0.00	0.00	0.00
0.01	0.00	0.00	0.00	0.00	0.00	0.00
0.00	0.00	0.00	0.00	0.00	0.00	0.00
0.00	0.00	0.00	0.00	0.00	0.00	0.00
0.00	0.00	0.00	0.00	0.00	0.00	0.00
0.00	0.00	0.00	0.00	0.00	0.00	0.00
0.00	0.00	0.00	0.00	0.00	0.00	0.00
0.00	0.00	0.00	0.00	0.00	0.00	0.00
0.00	0.00	0.00	0.00	0.00	0.00	0.00
0.00	0.00	0.00	0.00	0.00	0.00	0.00
0.00	0.00	0.00	0.00	0.00	0.00	0.00
0.00	0.00	0.00	0.00	0.00	0.00	0.00
0.00	0.00	0.00	0.00	0.00	0.00	0.00
0.00	0.00	0.00	0.00	0.00	0.00	0.00
0.00	0.00	0.00	0.00	0.00	0.00	0.00
0.00	0.00	0.00	0.00	0.00	0.00	0.00
0.00	0.00	0.00	0.00	0.00	0.00	0.00

0.00	0.00	0.00	0.00	0.00	0.00	0.00
0.00	0.00	0.00	0.00	0.00	0.00	0.00
0.00	0.00	0.00	0.00	0.00	0.00	0.00
0.00	0.00	0.00	0.00	0.00	0.00	0.00
0.00	0.00	0.00	0.00	0.00	0.00	0.00
0.00	0.00	0.00	0.00	0.00	0.00	0.00
0.00	0.00	0.01	0.00	0.00	0.00	0.00
0.00	0.00	0.00	0.00	0.00	0.00	0.00
0.00	0.00	0.00	0.00	0.00	0.00	0.00
0.00	0.00	0.00	0.00	0.00	0.00	0.00
0.00	0.00	0.00	0.00	0.00	0.00	0.00
0.00	0.00	0.00	0.00	0.00	0.00	0.00
0.00	0.00	0.01	0.00	0.00	0.00	0.00
0.00	0.00	0.00	0.00	0.00	0.00	0.00
0.00	0.00	0.00	0.00	0.00	0.00	0.00
0.00	0.00	0.00	0.00	0.00	0.00	0.00
0.00	0.00	0.00	0.00	0.00	0.00	0.00
0.00	0.00	0.01	0.00	0.00	0.00	0.00
0.00	0.00	0.00	0.00	0.00	0.00	0.00
0.00	0.00	0.00	0.00	0.00	0.00	0.00
0.00	0.00	0.00	0.00	0.01	0.00	0.00
0.00	0.00	0.00	0.00	0.00	0.00	0.00
0.00	0.00	0.00	0.00	0.00	0.00	0.00
0.00	0.00	0.00	0.00	0.00	0.00	0.00
0.00	0.00	0.00	0.00	0.00	0.00	0.00
0.00	0.00	0.00	0.00	0.00	0.00	0.00
0.00	0.00	0.00	0.00	0.00	0.00	0.00
0.00	0.00	0.00	0.00	0.00	0.00	0.00
0.00	0.00	0.00	0.00	0.00	0.00	0.00
0.00	0.00	0.00	0.00	0.00	0.00	0.00
0.00	0.00	0.00	0.00	0.00	0.00	0.00
0.00	0.00	0.00	0.00	0.00	0.00	0.00
0.00	0.00	0.00	0.00	0.00	0.00	0.00
0.00	0.00	0.00	0.00	0.00	0.00	0.00
0.00	0.00	0.00	0.00	0.00	0.00	0.00
0.00	0.00	0.00	0.00	0.00	0.00	0.00
0.00	0.00	0.00	0.00	0.00	0.00	0.00
0.00	0.00	0.00	0.00	0.00	0.00	0.00
0.00	0.00	0.00	0.00	0.00	0.00	0.00
0.00	0.00	0.00	0.00	0.00	0.00	0.00
0.00	0.00	0.00	0.00	0.00	0.00	0.00
0.00	0.00	0.00	0.00	0.00	0.00	0.00
0.00	0.00	0.00	0.00	0.00	0.00	0.00
0.00	0.00	0.00	0.00	0.00	0.00	0.00
0.00	0.00	0.00	0.00	0.00	0.00	0.00
0.00	0.00	0.00	0.00	0.00	0.00	0.00
0.00	0.00	0.00	0.00	0.00	0.00	0.00
0.00	0.00	0.00	0.00	0.00	0.00	0.00
0.00	0.00	0.00	0.00	0.00	0.00	0.00
0.00	0.00	0.00	0.00	0.00	0.00	0.00
0.00	0.00	0.00	0.00	0.00	0.00	0.00
0.00	0.00	0.00	0.00	0.00	0.00	0.00
0.00	0.00	0.00	0.00	0.00	0.00	0.00
0.00	0.00	0.00	0.00	0.00	0.00	0.00
0.00	0.00	0.00	0.00	0.00	0.00	0.00
0.00	0.00	0.00	0.00	0.00	0.00	0.00
0.00	0.00	0.00	0.00	0.00	0.00	0.00
0.00	0.00	0.00	0.00	0.00	0.00	0.00
0.00	0.00	0.00	0.00	0.00	0.00	0.00

0.00	0.00	0.00	0.00	0.00	0.00	0.00
0.00	0.00	0.00	0.00	0.00	0.00	0.00
0.00	0.00	0.00	0.00	0.00	0.00	0.00
0.00	0.00	0.00	0.00	0.00	0.00	0.00
0.00	0.00	0.00	0.00	0.00	0.00	0.00
0.00	0.00	0.00	0.00	0.00	0.00	0.00
0.00	0.00	0.00	0.00	0.00	0.00	0.00
0.00	0.00	0.00	0.00	0.00	0.00	0.00
0.00	0.00	0.00	0.00	0.00	0.00	0.00
0.00	0.00	0.00	0.00	0.00	0.00	0.00
0.00	0.00	0.00	0.00	0.00	0.00	0.00
0.00	0.00	0.00	0.00	0.00	0.00	0.00
0.00	0.00	0.00	0.00	0.00	0.00	0.00
0.00	0.00	0.00	0.00	0.00	0.00	0.00
0.00	0.00	0.00	0.00	0.00	0.00	0.00
0.00	0.00	0.00	0.00	0.00	0.00	0.00
0.00	0.00	0.00	0.00	0.00	0.00	0.00
0.00	0.00	0.00	0.00	0.00	0.00	0.00
0.00	0.00	0.00	0.00	0.00	0.00	0.00
0.00	0.00	0.00	0.00	0.00	0.00	0.00
0.00	0.00	0.00	0.00	0.00	0.00	0.00
0.00	0.00	0.00	0.00	0.00	0.00	0.00
0.00	0.00	0.00	0.00	0.00	0.00	0.00
0.00	0.00	0.00	0.00	0.00	0.00	0.00
0.00	0.00	0.00	0.00	0.00	0.00	0.00
0.00	0.00	0.00	0.00	0.00	0.00	0.00
0.00	0.00	0.00	0.00	0.00	0.00	0.00
0.00	0.00	0.00	0.00	0.00	0.00	0.00
0.00	0.00	0.00	0.00	0.00	0.00	0.00
0.00	0.00	0.00	0.00	0.00	0.00	0.00

50	51	52	53	54	55	56
23	24	25	26	27	28	29
CHR_NP	CHR_NP	CHR_NP	CHR_NP	CHR_NP	CHR_NP	CHR_NP
E_5	E_6	F_1	F_35	F_36	F_37	F_38
1597470	1583268	2592561	3565545	2585170	976547	1738984
E_5	E_6	F_1	F_35	F_36	F_37	F_38
-0.15	-0.52	0.27	0.95	-0.39	0.87	0.59
-0.37	-0.31	0.20	-0.06	0.14	-0.21	0.14
2.45	1.54	0.46	-1.06	0.27	-0.33	-0.23
-1.09	-1.29	-0.01	0.61	0.11	0.00	-0.02
0.70	1.01	-0.55	-1.08	-0.49	-0.24	-0.11
-0.29	0.81	-0.82	-0.62	-0.18	-0.64	-0.54
0.64	0.60	-0.04	-0.56	1.23	-0.46	-0.32
-0.29	0.11	-0.55	-0.53	-0.42	-0.57	-0.55

0.06	1.55	-0.64	-0.61	0.51	-0.48	-0.56
-0.88	-0.61	-0.86	-0.55	-0.73	-0.69	-0.73
-0.87	-0.49	0.00	0.22	0.48	-0.06	-0.74
1.84	2.95	0.67	-0.78	0.77	-0.69	-0.37
-0.56	-0.43	-0.02	-0.40	1.27	-0.09	-0.95
-0.95	-1.23	-0.59	0.56	-0.76	-0.60	0.13
-0.65	-0.19	-0.80	-0.70	0.01	-0.31	-0.81
0.57	-0.12	0.32	-0.20	0.43	0.59	-0.53
-0.36	0.19	-0.58	-0.64	0.24	-0.53	-0.67
0.15	1.00	-0.13	-0.25	1.50	-0.67	-0.59
-0.09	0.40	-0.69	-0.98	0.23	-0.82	-0.74
-0.28	-0.53	0.20	-0.67	-0.30	-0.41	0.06
1.25	-0.08	0.17	-0.42	0.65	-0.74	0.59
-0.56	-0.42	-0.24	0.04	0.46	-1.14	-0.52
-0.63	-0.48	0.65	0.23	1.01	0.55	0.40
-0.19	-0.33	-0.40	-0.68	0.88	-0.75	-0.57
-0.39	-0.09	-0.57	-0.65	-0.31	-0.57	-0.57
1.94	1.12	1.49	1.04	0.93	0.30	1.34
-0.22	-0.62	1.17	1.00	0.23	0.22	0.28
-0.48	-0.33	-0.87	-0.40	-0.70	-0.51	-0.95
-0.06	0.43	0.84	0.96	1.31	0.00	0.26
-0.52	0.59	4.20	3.01	1.71	1.37	1.36
2.36	2.46	-0.72	-1.18	1.54	-0.52	-0.96
-0.67	-1.13	-0.18	0.68	0.07	-0.03	-0.02
0.01	0.48	-0.42	-0.56	0.17	-0.51	-0.54
-0.32	-0.23	-0.29	-0.30	-0.17	-0.30	-0.34
0.07	0.61	1.09	1.22	1.63	0.10	0.44
-0.46	-0.46	-0.34	0.03	-0.09	-0.61	-0.01
-0.66	-0.01	-0.54	-0.47	-0.58	-0.53	-0.61
-0.35	-0.01	-0.31	-0.52	-0.31	-0.31	-0.32
-0.57	-1.03	0.92	0.03	-0.35	-0.42	0.50
-0.29	-0.57	-0.40	-0.45	-0.49	0.61	-0.37
-0.23	-0.68	0.00	-0.22	0.06	-0.84	-0.50
1.05	0.83	-0.56	-1.15	-0.45	0.08	-0.46
0.45	-0.24	0.83	0.84	0.70	-0.62	1.00
-0.71	-0.34	-0.82	-0.82	-0.65	-0.76	-0.80
0.82	0.83	-0.54	-0.98	-0.08	-0.91	-0.49
1.87	2.38	3.13	1.13	1.27	4.19	0.59
-0.21	1.15	2.06	0.84	1.46	0.23	0.29
0.27	-0.08	0.07	-0.33	0.29	0.43	-0.06
-0.23	-0.17	-0.32	-0.34	-0.27	-0.26	-0.33
-0.20	-0.24	-0.48	-0.46	-0.12	-0.45	-0.53
0.43	1.90	10.02	5.09	-0.16	-0.85	3.87
-0.19	-0.14	-0.22	-0.22	1.39	-0.61	-0.42
-0.21	-0.32	0.57	0.90	0.80	-1.33	0.42
-0.45	-0.57	-0.40	-0.87	-0.12	0.10	-0.37
1.24	-0.07	0.61	-0.17	-0.09	-0.39	0.16

-0.26	-0.14	-0.30	-0.30	-0.24	-0.30	-0.32
0.57	0.13	-0.04	-0.40	1.67	-0.86	0.15
2.72	0.56	-0.70	-1.22	0.24	-0.80	-0.76
0.94	0.05	0.79	-1.17	1.04	-0.22	-0.44
-0.55	-0.51	0.23	-0.23	0.10	-0.59	0.94
-0.54	0.79	1.99	0.82	1.13	-0.32	-0.34
1.14	0.41	-0.89	-1.00	-0.07	-1.02	-0.80
-0.62	-0.07	-0.54	-0.54	-0.54	-0.60	-0.52
-0.13	-0.14	-0.20	-0.24	-0.24	-0.24	-0.25
-0.62	-0.73	-1.05	-0.28	-1.16	0.47	-0.70
-0.34	0.59	-0.65	-0.30	0.61	-1.04	-0.77
0.03	1.25	-0.43	-0.61	0.11	-0.57	-0.61
-0.49	0.16	-0.63	-0.36	-0.02	-0.77	-0.66
0.68	0.94	0.19	0.13	2.62	-0.87	-0.15
-0.28	-0.28	-0.25	-0.14	-0.33	-0.19	-0.31
-0.31	-0.21	0.31	0.12	0.24	0.32	-0.19
-0.51	-0.61	-0.57	0.43	-0.60	1.69	0.44
-0.77	-0.31	-0.75	-0.63	-0.26	-0.59	-0.80
-0.01	0.13	-0.21	-0.09	0.03	-0.38	-0.19
2.18	1.43	0.38	-0.70	1.66	-0.36	0.14
-0.19	0.64	3.15	2.14	-0.22	-0.61	1.11
0.15	-0.15	0.53	-0.02	0.52	-0.44	0.68
-0.41	-0.37	0.21	0.42	-0.02	-0.20	0.53
-0.63	-0.65	0.09	-0.23	0.40	-0.02	-0.53
-0.58	0.52	1.11	0.63	0.65	-0.66	-0.05
0.30	0.43	-0.98	-0.84	-0.12	-0.88	-0.37
0.72	0.27	1.56	0.13	0.59	0.14	-0.21
-0.44	-0.61	0.30	0.23	-0.52	0.04	0.16
-0.24	-0.08	-0.29	-0.38	0.20	-0.35	-0.42
-0.32	-0.36	0.62	-0.28	0.64	-0.45	0.31
0.41	0.80	1.31	0.33	1.02	-0.86	-0.14
-0.35	-0.37	1.29	0.08	0.25	1.79	-0.46
-0.39	0.88	-0.79	-0.76	-0.41	-0.67	-0.67
0.96	1.05	-0.14	-0.73	1.81	1.22	-0.88
-0.94	-0.93	0.21	0.62	-0.26	0.59	0.25
-0.05	2.81	0.21	-0.12	0.14	1.71	1.11
-0.23	-0.18	-0.23	-0.17	-0.26	-0.27	-0.26
1.62	0.28	0.38	-0.71	1.01	-0.21	-0.42
0.22	2.98	0.08	-0.04	0.61	-0.48	-0.13
-0.23	0.16	-0.50	-0.41	0.09	-0.51	-0.47
0.61	0.78	-0.70	-0.80	0.54	-0.51	-0.79
-0.41	-0.45	0.75	0.05	0.03	1.40	0.02
0.02	-0.41	-0.16	-0.24	-0.11	0.60	-0.44
0.32	-0.04	0.43	-0.62	-0.38	-0.36	0.29
-0.59	0.00	-0.65	-0.55	-0.12	-0.64	-0.63
-0.36	-0.33	0.48	0.50	0.01	-0.73	0.14
1.47	1.40	1.56	1.66	1.87	0.37	1.43
0.00	-0.46	0.24	-0.88	0.22	0.27	-0.41

-0.45	-0.29	-0.43	-0.40	-0.22	-0.47	-0.45
-0.61	0.45	-1.04	-0.95	-0.39	-0.86	-0.88
-0.50	-0.18	-0.73	-0.72	-0.50	-0.72	-0.70
0.21	0.32	0.85	0.33	1.12	-0.26	-0.35
-0.10	-0.08	-0.14	-0.19	0.79	-0.51	-0.25
-0.26	0.42	-0.47	-0.30	0.50	-0.71	-0.62
-0.34	0.51	-0.71	-0.61	0.60	-1.04	-0.75
0.99	2.30	-0.77	-1.08	-0.29	-0.39	-0.36
2.50	0.45	1.07	-0.04	2.02	0.24	0.09
-0.42	-0.45	0.23	0.17	1.06	-1.05	-0.07
-0.49	0.15	-0.85	-0.30	-0.25	-0.98	-0.39
2.30	1.10	0.22	-1.19	0.43	-0.40	-0.30
-1.23	-1.00	1.16	0.25	0.59	0.36	0.09
-0.17	0.93	-1.08	-0.87	-0.47	-0.85	-1.08
-0.38	1.69	0.17	-0.17	2.07	0.18	-0.43
-0.38	1.69	0.17	-0.17	2.07	0.18	-0.43
-0.59	-0.53	-0.50	-0.51	-0.14	-0.96	-0.77
0.38	0.15	-0.23	-0.07	0.26	-0.67	-0.18
1.08	1.43	-0.70	0.75	1.32	1.58	0.34
1.64	3.74	0.80	-0.06	-0.22	-0.55	0.11
0.24	0.15	-0.53	-0.63	0.56	-0.46	-0.21
-0.20	-0.06	-0.24	-0.32	-0.06	-0.33	-0.29
0.02	-0.70	0.05	0.25	0.75	1.40	-0.63
0.22	-0.34	0.91	-0.39	0.68	-0.71	1.78
1.06	1.25	0.50	0.00	0.88	-0.24	0.18
-0.40	-0.08	1.62	0.85	-0.05	-0.60	0.04
-0.30	-0.42	0.08	0.57	0.06	0.26	-0.11
-0.19	-0.34	1.63	2.03	1.55	0.30	2.79
0.17	1.61	-0.19	-0.94	1.43	0.22	-0.84
-0.49	1.41	1.74	0.73	2.06	-0.56	0.23
-0.19	-0.19	0.58	0.41	0.55	-0.55	0.13
-0.02	0.51	-0.17	-0.57	0.04	-0.28	-0.60
0.01	-0.03	0.94	-0.12	1.11	-0.29	-0.41

13	14	15	16	17	18	19
49	50	51	52	53	54	55
TTCACAGTGGCTA	TCTCTCGGCTCCT	GGATATCATCATA	TGAGATGAAGCAC	TCGGATCCGTCT	ATCACATTGCCAC	AATGGCGCCACTA
miR-27b-3p	miR-3615	miR-144-5p	miR-143-3p	miR-127-3p	miR-23a-3p	miR-652-3p
209441	198175	178962	177234	154285	151875	143334
-0.04	0.68	-0.29	-0.43	2.23	-0.46	2.20
-1.33	-1.33	-0.29	-0.55	0.39	-0.31	0.05
-0.14	-0.06	0.02	-0.51	-0.65	-0.42	-0.25
-0.64	0.17	0.23	-0.54	-1.12	-0.72	-0.49
-0.53	-0.81	-0.33	-0.61	0.66	3.79	1.26
0.59	-0.47	-0.16	0.03	-0.15	-0.43	-0.85
-0.01	0.11	-0.21	0.01	0.71	-0.49	-0.38
-1.03	1.49	-0.29	-0.44	-0.53	-0.64	-0.64
-0.88	-0.83	-0.27	-0.50	0.44	-0.57	-1.34
-0.82	-1.23	-0.28	-0.42	-0.33	-0.39	0.17
1.17	-1.04	-0.30	0.48	-0.49	0.41	-1.24
-1.04	2.97	-0.27	-0.32	-1.56	-0.66	-2.15
0.87	-0.45	0.28	3.41	-1.76	2.51	0.09
-0.98	0.26	-0.31	-0.50	0.41	-0.67	-0.53
-0.30	0.16	-0.29	-0.29	-0.25	-0.02	0.56
3.48	-0.56	-0.11	0.23	-0.04	0.47	0.05
0.32	-0.09	-0.34	-0.45	0.63	-0.19	1.12
-0.39	-0.13	-0.11	0.08	0.68	0.09	1.82
0.07	-0.33	-0.36	0.81	-0.40	0.37	0.80
0.04	-0.22	4.92	3.08	-1.63	0.06	-1.00

-0.21	-0.34	-0.13	-0.40	-0.96	-0.53	-0.70
0.26	1.85	-0.34	-0.39	1.06	-0.42	-0.56
-0.05	0.78	-0.24	-0.19	-0.36	-0.47	0.49
-0.47	1.42	-0.28	-0.53	-0.75	-0.67	1.04
-0.49	-1.05	-0.29	-0.65	1.78	0.38	0.41
1.62	-0.37	-0.26	-0.42	1.21	0.35	0.72
0.93	-0.57	0.30	0.04	0.79	-0.36	-0.61
1.01	-1.00	7.55	1.96	-1.16	-0.27	-1.78
0.88	-0.70	-0.25	0.99	1.59	0.10	-0.81
-0.33	-0.66	-0.33	-0.08	-0.32	-0.39	-0.29
-0.97	-0.39	-0.34	-0.66	7.26	-0.88	1.85
0.89	-0.92	-0.35	0.02	3.08	-0.17	0.14
-0.37	-1.09	-0.11	-0.52	-0.27	4.70	0.63
-0.71	1.03	-0.33	0.89	-0.77	-0.22	0.02
-0.96	0.94	-0.30	0.19	-0.37	1.20	3.90
-0.31	-0.52	-0.31	-0.18	-1.47	-0.23	-1.21
-1.36	0.51	-0.31	-0.66	-1.35	-0.95	-1.77
-0.49	-0.84	-0.35	0.97	0.17	0.70	-0.06
1.20	-1.16	-0.12	1.38	-0.77	1.83	1.03
-0.38	0.05	-0.33	-0.27	-1.14	0.46	-1.03
0.63	1.30	-0.35	0.12	2.27	0.03	8.39
7.95	5.55	-0.20	1.24	6.64	0.23	28.19
0.87	0.25	-0.28	-0.48	1.22	-0.33	0.57
0.98	0.30	-0.21	1.07	-0.99	1.56	2.20
-0.88	1.52	-0.36	-0.32	-0.93	-0.40	-0.32
-0.30	1.74	-0.29	-0.53	0.39	-0.73	6.32
1.61	-0.79	-0.19	0.53	0.07	0.97	-0.61
0.33	1.29	-0.25	0.12	0.95	0.02	-0.23
-0.30	0.57	-0.10	-0.08	-1.45	-0.17	-0.12
-0.21	0.27	-0.23	-0.20	0.43	-0.19	-0.21
1.15	-0.08	-0.17	-0.24	1.90	-0.14	-0.32
2.06	0.07	-0.32	-0.48	10.02	-0.22	0.57
0.84	-0.33	-0.34	-0.46	5.09	-0.22	0.90
1.46	0.29	-0.27	-0.12	-0.16	1.39	0.80
0.23	0.43	-0.26	-0.45	-0.85	-0.61	-1.33
0.29	-0.06	-0.33	-0.53	3.87	-0.42	0.42
0.66	-0.27	-0.15	-0.51	4.53	-0.41	0.02
0.78	-0.99	7.33	0.77	-1.76	-0.33	-1.10
-0.35	0.69	-0.30	-0.53	0.82	-0.19	0.24
0.99	0.02	-0.01	-0.44	-1.17	0.86	1.20
0.07	-0.80	-0.11	-0.58	-0.98	1.11	0.66
0.38	-0.74	5.92	0.96	-1.71	-0.52	-1.50
-0.11	0.33	-0.09	-0.55	-1.35	-0.72	0.44
0.84	-0.15	0.03	-0.50	-0.22	-0.61	-0.76
0.13	-0.72	0.15	-0.46	-1.58	-0.46	-0.83
-1.11	2.07	0.24	1.64	-1.59	-0.68	-1.46
0.16	-0.88	-0.21	-0.33	-0.31	-0.34	1.19
-0.86	-0.68	-0.36	-0.47	-0.99	0.10	3.19
-0.21	0.17	1.03	-0.65	0.71	-0.20	0.96
0.68	1.20	0.48	-0.37	1.52	0.60	0.63
-0.30	0.83	0.20	-0.38	0.29	-0.70	-1.14
-1.05	-0.21	0.19	4.63	-1.85	-0.61	-1.92
-0.61	0.04	0.18	-0.48	-1.24	-0.23	-0.82
-0.71	-0.21	0.01	0.72	-0.67	0.58	-0.19
-0.38	6.29	-0.37	-0.56	4.92	0.02	36.59
0.14	0.21	0.10	0.01	0.71	-0.27	-0.72
-0.43	-0.62	-0.37	0.06	-0.24	0.40	0.87
-0.28	-0.35	-0.39	-0.08	1.99	-0.42	-1.49
1.39	-1.11	0.40	-0.17	0.65	0.95	0.12

2.31	-0.92	-0.19	-0.08	-0.66	0.94	-0.83
0.70	0.14	-0.24	-0.53	-1.53	-0.42	0.69
0.94	-0.65	0.16	-0.42	1.90	0.55	-0.49
-0.19	1.86	-0.21	0.06	-0.23	-0.38	-1.72
0.45	0.09	4.72	3.71	-0.97	0.88	-0.81
-0.13	-1.03	12.72	1.40	-1.61	-0.41	-1.72
0.38	0.11	-0.30	0.29	-1.04	0.08	-0.60
0.80	-0.39	-0.07	-0.10	0.01	0.32	-0.82
0.25	-0.10	0.05	-0.38	-1.45	-0.22	-0.57
0.29	-0.27	-0.27	1.48	-1.20	0.19	-1.06
0.74	-0.43	-0.27	-0.39	-1.21	0.29	-0.28
-0.02	-0.55	0.71	1.87	-0.79	-0.28	-1.49
-0.32	0.20	-0.29	-0.23	0.19	-0.57	-1.06
0.49	0.09	-0.19	-0.29	0.80	-0.44	-0.53
1.91	-0.71	-0.11	-0.29	-0.76	-0.21	-0.60
3.44	-0.42	-0.16	-0.43	1.12	-0.28	2.02
1	1	TATTGCACTTGT(	miR-92a-3p	46389442	1	-2
0.00	0.00	0.00	0.00	0.00	0.00	0.00
1.00	1.00	1.00	1.00	1.00	1.00	1.00
0.29	0.97	0.64	0.26	0.08	0.41	0.82

47	48	49	50	51	52	53
TTCACAGTGGCT/	TCTCTCGGCTCC1	GGATATCATCAT/	TGAGATGAAGCA(	TCGGATCCGTC1	ATCACATTGCCA(	AATGGCGCCACT/
miR-27b-3p	miR-3615	miR-144-5p	miR-143-3p	miR-127-3p	miR-23a-3p	miR-652-3p
209441	198175	178962	177234	154285	151875	143334
-0.04	0.68	-0.29	-0.43	2.23	-0.46	2.20
-1.33	-1.33	-0.29	-0.55	0.39	-0.31	0.05
-0.14	-0.06	0.02	-0.51	-0.65	-0.42	-0.25
-0.64	0.17	0.23	-0.54	-1.12	-0.72	-0.49
-0.53	-0.81	-0.33	-0.61	0.66	3.79	1.26
0.59	-0.47	-0.16	0.03	-0.15	-0.43	-0.85
-0.01	0.11	-0.21	0.01	0.71	-0.49	-0.38
-1.03	1.49	-0.29	-0.44	-0.53	-0.64	-0.64
-0.88	-0.83	-0.27	-0.50	0.44	-0.57	-1.34
-0.82	-1.23	-0.28	-0.42	-0.33	-0.39	0.17
1.17	-1.04	-0.30	0.48	-0.49	0.41	-1.24
-1.04	2.97	-0.27	-0.32	-1.56	-0.66	-2.15
0.87	-0.45	0.28	3.41	-1.76	2.51	0.09
-0.98	0.26	-0.31	-0.50	0.41	-0.67	-0.53
-0.30	0.16	-0.29	-0.29	-0.25	-0.02	0.56
3.48	-0.56	-0.11	0.23	-0.04	0.47	0.05
0.32	-0.09	-0.34	-0.45	0.63	-0.19	1.12
-0.39	-0.13	-0.11	0.08	0.68	0.09	1.82
0.07	-0.33	-0.36	0.81	-0.40	0.37	0.80
0.04	-0.22	4.92	3.08	-1.63	0.06	-1.00
-0.21	-0.34	-0.13	-0.40	-0.96	-0.53	-0.70
0.26	1.85	-0.34	-0.39	1.06	-0.42	-0.56
-0.05	0.78	-0.24	-0.19	-0.36	-0.47	0.49
-0.47	1.42	-0.28	-0.53	-0.75	-0.67	1.04
-0.49	-1.05	-0.29	-0.65	1.78	0.38	0.41
1.62	-0.37	-0.26	-0.42	1.21	0.35	0.72
0.93	-0.57	0.30	0.04	0.79	-0.36	-0.61
1.01	-1.00	7.55	1.96	-1.16	-0.27	-1.78

0.88	-0.70	-0.25	0.99	1.59	0.10	-0.81
-0.33	-0.66	-0.33	-0.08	-0.32	-0.39	-0.29
-0.97	-0.39	-0.34	-0.66	7.26	-0.88	1.85
0.89	-0.92	-0.35	0.02	3.08	-0.17	0.14
-0.37	-1.09	-0.11	-0.52	-0.27	4.70	0.63
-0.71	1.03	-0.33	0.89	-0.77	-0.22	0.02
-0.96	0.94	-0.30	0.19	-0.37	1.20	3.90
-0.31	-0.52	-0.31	-0.18	-1.47	-0.23	-1.21
-1.36	0.51	-0.31	-0.66	-1.35	-0.95	-1.77
-0.49	-0.84	-0.35	0.97	0.17	0.70	-0.06
1.20	-1.16	-0.12	1.38	-0.77	1.83	1.03
-0.38	0.05	-0.33	-0.27	-1.14	0.46	-1.03
0.63	1.30	-0.35	0.12	2.27	0.03	8.39
7.95	5.55	-0.20	1.24	6.64	0.23	28.19
0.87	0.25	-0.28	-0.48	1.22	-0.33	0.57
0.98	0.30	-0.21	1.07	-0.99	1.56	2.20
-0.88	1.52	-0.36	-0.32	-0.93	-0.40	-0.32
-0.30	1.74	-0.29	-0.53	0.39	-0.73	6.32
1.61	-0.79	-0.19	0.53	0.07	0.97	-0.61
0.33	1.29	-0.25	0.12	0.95	0.02	-0.23
-0.30	0.57	-0.10	-0.08	-1.45	-0.17	-0.12
-0.21	0.27	-0.23	-0.20	0.43	-0.19	-0.21
1.15	-0.08	-0.17	-0.24	1.90	-0.14	-0.32
2.06	0.07	-0.32	-0.48	10.02	-0.22	0.57
0.84	-0.33	-0.34	-0.46	5.09	-0.22	0.90
1.46	0.29	-0.27	-0.12	-0.16	1.39	0.80
0.23	0.43	-0.26	-0.45	-0.85	-0.61	-1.33
0.29	-0.06	-0.33	-0.53	3.87	-0.42	0.42
0.66	-0.27	-0.15	-0.51	4.53	-0.41	0.02
0.78	-0.99	7.33	0.77	-1.76	-0.33	-1.10
-0.35	0.69	-0.30	-0.53	0.82	-0.19	0.24
0.99	0.02	-0.01	-0.44	-1.17	0.86	1.20
0.07	-0.80	-0.11	-0.58	-0.98	1.11	0.66
0.38	-0.74	5.92	0.96	-1.71	-0.52	-1.50
-0.11	0.33	-0.09	-0.55	-1.35	-0.72	0.44
0.84	-0.15	0.03	-0.50	-0.22	-0.61	-0.76
0.13	-0.72	0.15	-0.46	-1.58	-0.46	-0.83
-1.11	2.07	0.24	1.64	-1.59	-0.68	-1.46
0.16	-0.88	-0.21	-0.33	-0.31	-0.34	1.19
-0.86	-0.68	-0.36	-0.47	-0.99	0.10	3.19
-0.21	0.17	1.03	-0.65	0.71	-0.20	0.96
0.68	1.20	0.48	-0.37	1.52	0.60	0.63
-0.30	0.83	0.20	-0.38	0.29	-0.70	-1.14
-1.05	-0.21	0.19	4.63	-1.85	-0.61	-1.92
-0.61	0.04	0.18	-0.48	-1.24	-0.23	-0.82
-0.71	-0.21	0.01	0.72	-0.67	0.58	-0.19
-0.38	6.29	-0.37	-0.56	4.92	0.02	36.59
0.14	0.21	0.10	0.01	0.71	-0.27	-0.72
-0.43	-0.62	-0.37	0.06	-0.24	0.40	0.87
-0.28	-0.35	-0.39	-0.08	1.99	-0.42	-1.49
1.39	-1.11	0.40	-0.17	0.65	0.95	0.12
2.31	-0.92	-0.19	-0.08	-0.66	0.94	-0.83
0.70	0.14	-0.24	-0.53	-1.53	-0.42	0.69
0.94	-0.65	0.16	-0.42	1.90	0.55	-0.49
-0.19	1.86	-0.21	0.06	-0.23	-0.38	-1.72
0.45	0.09	4.72	3.71	-0.97	0.88	-0.81
-0.13	-1.03	12.72	1.40	-1.61	-0.41	-1.72
0.38	0.11	-0.30	0.29	-1.04	0.08	-0.60
0.80	-0.39	-0.07	-0.10	0.01	0.32	-0.82

0.25	-0.10	0.05	-0.38	-1.45	-0.22	-0.57
0.29	-0.27	-0.27	1.48	-1.20	0.19	-1.06
0.74	-0.43	-0.27	-0.39	-1.21	0.29	-0.28
-0.02	-0.55	0.71	1.87	-0.79	-0.28	-1.49
-0.32	0.20	-0.29	-0.23	0.19	-0.57	-1.06
0.49	0.09	-0.19	-0.29	0.80	-0.44	-0.53
1.91	-0.71	-0.11	-0.29	-0.76	-0.21	-0.60
3	0	0	0	1	0	2

47	48	49	50	51	52	53
94	29	17	35	131	95	77
CAGTGCAATGTTA	TCGAGGAGCTCAC	TTCAAGTAATTCA	TCGAGGAGCTCAC	AATGCACCCGGC	TAGCAGCACATAA	ATGCACCTGGGCA
miR-130a-3p	miR-151b	miR-26b-5p	miR-151a-5p	miR-501-3p	miR-15a-5p	miR-500a-3p
30537	500966	1034642	454020	11405	29005	65049
-0.48	-0.90	-0.79	-1.15	-0.50	-0.56	0.19
-0.85	1.14	-0.15	0.25	3.21	-0.70	-1.38
-0.06	1.56	0.07	0.73	1.10	0.20	-0.24
-0.43	-0.43	-0.29	-0.85	-0.41	-0.46	-0.46
-0.38	3.04	1.51	3.24	-0.35	-0.24	-1.70
-0.66	-0.44	-0.34	-0.40	-0.76	-0.44	-0.54
-0.46	-0.76	-0.33	-0.71	-0.67	-0.40	-0.83
-0.87	-1.54	-0.80	-1.52	-0.41	-0.57	-0.71
-0.70	0.09	-0.64	0.23	-0.46	-0.48	0.46
-0.65	-0.13	-0.38	-0.07	0.04	-0.13	-0.03
-0.11	1.55	0.01	1.75	-0.76	-0.04	0.84
-0.76	-1.02	-0.69	-0.98	-0.65	-0.50	-0.82
0.74	0.20	3.45	0.35	-0.56	3.99	-0.43
-0.89	-1.05	-0.82	-0.98	-0.22	-0.51	-0.66
0.84	-0.16	0.02	-0.08	-0.12	0.00	1.68
2.30	1.15	0.81	1.33	-0.12	0.97	0.81
-0.39	-0.80	-0.78	-0.75	0.37	-0.41	2.72
3.20	-0.08	-0.24	0.02	-0.64	0.47	0.37
-0.39	-0.68	0.57	-0.63	-0.16	-0.14	1.04
0.37	0.72	1.96	0.86	2.52	2.17	0.09
-0.07	0.45	-0.30	0.63	-0.07	-0.32	-0.77
-0.23	-0.60	-0.73	-0.49	-0.22	-0.56	0.36
0.34	-0.19	-0.74	-0.05	-0.35	-0.46	0.33
-0.55	-0.77	-0.86	-0.69	-0.42	-0.50	0.07
-0.51	0.16	-0.34	0.31	-0.64	-0.49	-1.63
-0.09	-0.33	-0.19	-0.25	-0.53	-0.34	-0.04
1.73	-0.16	1.00	-0.09	1.79	0.46	1.27
0.73	1.56	1.74	0.71	-0.29	3.56	-0.61
-0.66	0.30	0.70	-0.27	-0.05	-0.24	1.06
-0.73	-0.31	-0.30	-0.72	-0.01	-0.43	0.22
-1.05	-0.17	-0.80	-0.69	0.83	-0.59	-1.37
-0.63	1.23	0.77	0.49	-0.63	-0.25	-1.44
-0.02	4.25	3.42	4.56	-0.76	-0.07	-1.73
-0.82	-1.48	-0.46	-1.49	-0.61	-0.31	-0.75
-0.66	-1.31	-0.65	-1.30	-0.42	-0.11	-0.96
-0.53	-0.90	-0.32	-0.84	-0.56	-0.48	-0.58
-1.07	-1.70	-0.96	-1.70	-0.61	-0.61	-1.24
-0.15	-1.12	-0.05	-1.10	-0.67	-0.12	-0.67
0.52	-0.54	1.30	-0.46	-0.27	2.56	-0.71

-0.59	-0.78	-0.08	-0.71	-0.49	-0.38	-0.34
-0.94	-0.89	-0.76	-0.85	2.77	-0.33	2.99
-0.61	-0.34	-0.77	-0.24	2.67	-0.42	5.49
0.13	0.22	-0.67	0.36	0.03	-0.38	0.07
0.26	-0.49	0.76	-0.44	-0.51	1.02	1.14
-0.69	-0.93	-0.68	-0.89	-0.54	-0.58	-0.17
-1.11	-1.27	-0.85	-1.26	1.20	-0.44	2.21
0.86	1.67	0.85	1.95	0.02	0.41	0.78
-0.04	0.32	-0.42	0.51	-0.34	-0.31	0.13
1.75	0.47	-0.14	0.66	-0.24	0.23	-0.12
0.22	-0.06	-0.36	0.07	-0.19	-0.23	0.15
2.98	0.43	0.19	0.61	-0.34	0.16	-0.15
0.08	0.84	-0.58	1.09	1.63	-0.50	0.53
-0.04	0.96	-0.64	1.22	2.03	-0.41	-0.02
0.61	1.31	0.24	1.63	1.55	0.09	0.52
-0.48	0.00	-0.53	0.10	0.30	-0.51	-0.44
-0.13	0.26	-0.67	0.44	2.79	-0.47	0.68
0.50	0.90	-0.23	1.06	0.58	-0.22	-0.34
1.25	-0.48	3.28	-0.49	0.92	2.75	-0.28
-0.29	-0.75	-0.37	-0.67	-0.55	-0.62	-0.83
0.47	0.58	1.38	0.77	-0.71	-0.29	-1.50
0.47	0.86	1.52	1.02	-0.76	-0.19	-1.52
0.78	0.17	2.50	0.25	1.92	3.23	0.07
-0.62	-0.99	-0.65	-0.96	5.07	-0.52	0.87
-0.27	-0.50	-0.61	-0.45	3.01	-0.39	1.22
0.34	0.45	0.33	0.50	2.10	-0.18	1.34
-0.63	-0.08	-0.48	-0.48	-0.67	-0.25	-0.27
1.26	-0.38	0.16	-0.80	-0.38	0.65	-0.20
0.32	0.86	-0.29	0.23	-0.25	-0.70	-1.76
-0.49	3.26	0.44	2.18	-0.69	-0.36	-1.68
0.34	1.33	1.84	0.59	-0.68	0.01	-1.58
-0.32	-0.89	-0.26	-1.17	-0.52	-0.40	-0.68
-0.24	-1.65	0.45	-1.69	-0.69	2.99	-1.39
-0.35	-0.22	0.05	-0.15	-0.06	0.11	-0.38
0.18	-0.14	1.04	-0.03	-0.46	1.07	-0.90
-0.90	-1.08	-0.95	-1.05	4.14	-0.66	2.88
1.13	-0.13	0.76	-0.07	-0.58	0.76	-0.24
-0.54	-0.26	0.23	-0.19	-0.38	-0.39	-0.29
-0.60	-0.67	-0.42	-0.61	-0.23	-0.51	-0.56
2.24	1.82	2.07	2.09	0.16	1.52	0.96
2.25	1.78	0.97	2.10	0.13	0.42	1.41
-0.05	0.72	-0.71	0.93	0.43	-0.29	0.70
0.34	1.56	1.01	1.81	-0.12	0.16	-0.18
0.17	-0.20	-0.73	-0.03	-0.31	-0.33	-0.12
0.73	0.40	1.79	0.54	3.29	2.83	0.09
0.36	1.17	3.05	1.31	1.67	4.61	-0.38
0.64	0.84	-0.34	1.06	-0.32	0.07	0.28
0.37	0.44	1.11	0.59	-0.29	-0.08	0.25
0.31	1.64	-0.11	1.84	-0.25	-0.14	-0.30
0.25	-0.30	1.81	-0.21	0.25	0.56	0.40
0.37	1.07	-0.06	1.32	0.81	-0.17	-0.11
-0.82	0.30	1.52	0.41	-0.35	-0.05	-0.12
-0.75	-1.13	-0.32	-1.10	-0.19	-0.52	-0.57
-0.15	-0.55	-0.64	-0.46	-0.17	-0.47	0.53
-0.18	-0.06	0.47	0.01	1.83	-0.29	1.73
-0.23	-0.35	-0.07	-0.31	4.53	0.02	2.33

0.16	0.17	0.18	0.19	0.19	0.20	0.21
0.11	0.06	0.16	0.07	0.19	0.49	0.12

3565545	2585170	976547	1738984	1856730	2304707	924323

57	58	59	60	61	62	63
27	28	29	30	31	32	33
F_35	F_36	F_37	F_38	F_39	G_3	G_30
CHR_NP	CHR_NP	CHR_NP	CHR_NP	CHR_NP	CHR_NP	CHR_NP
8303947	3677962	1611445	4566325	3689974	4454265	2666236
0.12	0.01	0.45	0.82	0.90	0.11	0.67
F_35	F_36	F_37	F_38	F_39	G_3	G_30
4738402	1092792	634898	2827341	1833244	2149558	1741913
1755132	781741	473210	768215	806882	330186	233445
340046	296230	77703	201610	135146	80730	89825
77296	194779	50911	97414	110065	70536	115590
338825	202546	74728	131317	125806	117238	76499
131189	135975	59576	111341	81711	112000	127053
56016	78288	14801	32212	74839	337970	10312
90072	153035	26823	51941	63278	146448	43235
5235	8559	768	2104	3826	255607	1558
31586	66828	10588	16579	46674	132537	13936
60382	37679	15012	25734	38110	54366	18696
58644	47412	13953	14827	41760	36692	9162
44732	45670	12668	24311	29721	24843	16347
31789	45628	10437	10434	28155	59508	17309
62313	26495	11105	26421	20767	17432	13921
7407	17517	4647	2353	10166	52787	2598
27797	31237	13167	9460	15521	4803	4535
6277	15435	2270	2745	6990	46001	2812
20862	29022	4473	8323	11550	10033	3807
7956	16360	2743	5304	8434	36055	5974
18075	15799	5800	12796	8110	10606	9427
14518	15608	3396	10456	6725	6812	4791
17013	14339	2358	6331	7740	10373	4887
15150	15827	4989	8178	8362	9213	3233
5207	16756	1222	3150	2613	14634	5704
2223	6081	1028	1837	2730	21942	3822
18819	12991	3995	10054	8563	5931	2074
22216	12082	4650	8405	8258	5835	7276
7872	3113	1833	766	4240	17088	905
17314	13790	3271	6517	8869	5601	1955
25514	13858	4918	8664	9835	4533	4091
4426	12369	2081	2658	5727	7641	2978
17338	9925	3677	6526	4902	7556	4750
2521	5618	786	1282	2358	24953	888
2065	3334	537	549	1062	29276	428
16461	13215	3026	6156	8196	4940	1840
16417	10148	1342	7627	5392	3475	3209
8173	5067	2086	3342	4191	4920	1965
4628	4906	1909	3313	4112	6314	1628
11242	5566	1962	7611	2147	1471	3720
4905	3092	5354	2915	1231	3845	4246
7186	6246	1073	2774	1878	754	2685
3350	4246	2177	2881	2415	4776	3557
9416	6452	1494	4811	3437	1907	1814
504	891	212	289	602	12193	165
1799	4632	598	2122	1550	2408	1799

6858	5056	3186	2944	3330	2052	1851
6820	6085	1422	2590	3312	4089	912
4403	4612	1915	2580	2395	1343	2003
202	302	126	111	417	14936	82
1113	1973	318	373	468	4329	205
14345	2557	601	5781	6902	192	1441
2976	6477	409	1087	1175	1566	789
5234	3637	565	2223	2076	1458	1106
1390	2111	949	1193	1007	1047	484
2421	1850	546	1509	1036	3036	98
214	272	61	88	182	15017	37
2551	3851	530	1617	1500	1967	888
1030	2129	438	803	576	2395	874
1644	3523	844	1334	1803	2163	1338
3551	3532	566	4142	2782	756	1465
3851	3053	630	1099	1315	841	728
1101	1675	297	668	548	1838	569
269	194	49	145	361	10590	42
147	103	36	52	68	7637	30
2726	739	1151	933	2237	529	501
1478	1802	176	462	711	2897	425
426	1076	132	207	642	3817	206
754	809	80	200	352	5979	146
1539	2826	156	618	431	1258	273
2470	619	557	497	1460	822	128
2575	1968	800	1021	934	276	482
2735	1136	1147	1340	1169	301	413
370	576	114	85	330	3638	101
1460	1111	336	672	875	1171	252
601	1931	249	659	654	1941	485
4764	1152	298	1655	3828	158	929
1583	1483	332	1087	674	826	218
4121	1625	424	2238	1943	744	374
1671	1732	531	635	594	510	643
2383	1721	236	768	636	333	244
748	824	200	493	683	901	175
1459	1299	405	586	535	321	329
2105	859	519	984	579	162	352
315	1205	104	101	261	1138	52
694	926	161	528	339	1474	121
1177	1147	120	431	278	359	223
1502	1142	677	586	750	243	411
170	286	62	110	269	2775	93
620	1201	397	272	558	533	309
1863	773	509	749	764	421	581
825	668	446	661	472	413	257
84	25	8	17	22	3259	5
441	827	179	274	189	359	249
617	691	103	277	476	810	123
167	329	31	64	145	1231	12
215	689	105	109	235	580	112
897	634	421	432	395	143	294
697	524	260	312	342	168	221
523	454	179	461	321	200	569
125	237	23	42	176	1834	26
962	465	50	359	143	156	132
1006	760	181	461	261	414	215
379	677	270	303	352	326	422
398	452	84	161	286	1161	160

193	272	62	105	297	721	38
100	131	27	53	92	1600	45
721	723	139	230	260	212	140
589	953	97	265	298	248	223
313	421	47	98	205	1242	39
213	405	24	84	136	1682	111
131	270	96	174	122	556	176
512	688	158	264	233	223	224
512	578	29	211	112	114	258
282	211	26	125	132	821	55
87	404	88	171	225	417	293
599	490	173	272	337	147	246
202	194	57	81	125	683	58
309	640	111	118	264	531	65
309	640	111	118	264	531	65
279	305	27	85	97	228	101
373	339	52	165	229	152	72
508	437	182	212	202	58	60
277	162	35	160	384	101	86
142	312	51	120	135	99	50
22	71	5	15	23	2202	7
490	442	217	130	207	101	87
223	330	42	345	175	140	108
326	367	76	178	204	101	77
632	248	48	184	80	29	63
675	304	145	168	150	104	236
736	436	77	457	184	272	14
110	319	76	61	122	132	82
354	402	42	135	160	211	73
426	331	43	169	111	33	45
57	124	31	25	74	500	23
239	282	60	99	126	173	71
263	89	12	89	106	166	71
144	132	39	75	129	159	35
158	240	42	92	129	165	22
287	252	26	131	77	93	93
267	166	42	125	77	109	36
258	366	48	146	158	52	38
122	93	61	61	32	87	94
118	252	57	100	79	132	65
21	37	1	17	19	1255	3
208	110	48	83	120	55	47
250	289	78	88	55	39	85
133	243	57	56	83	104	79
232	213	59	90	180	68	45
204	128	23	97	70	87	78
73	144	32	37	55	266	43
97	84	13	37	55	286	33
159	86	9	73	150	48	17
207	187	39	87	50	46	103
5	87	3	12	14	208	119
341	135	55	80	142	63	34
233	147	13	86	40	100	65
409	140	27	57	99	16	38
173	173	23	100	86	74	47
234	105	48	91	116	67	21
11	18	1	5	6	701	4
199	194	14	64	28	53	50
237	137	48	102	83	41	48

58	104	10	28	30	248	24
84	233	10	32	22	168	45
127	91	63	66	101	48	44
227	185	26	115	62	36	50
105	48	12	24	27	80	23
10	26	7	12	15	641	6
125	78	18	37	97	21	12
118	151	30	96	49	72	56
111	163	20	18	108	117	17
114	152	36	82	96	34	56
139	130	39	48	65	93	29
36	141	6	11	19	182	25
106	95	28	42	6	38	62
441	130	14	106	441	5	32
141	140	40	44	78	159	17
69	122	14	64	31	21	19
405	104	9	103	304	5	35
233	73	28	97	51	56	39
20	45	9	14	36	266	9
482	98	15	131	302	5	43
16	46	16	16	28	146	19
283	157	47	68	119	16	47
82	85	45	50	35	36	51
19	44	9	13	35	255	9
350	58	16	70	207	3	23
90	141	20	10	93	39	47
150	148	30	117	60	79	18
93	516	55	4	6	338	2
11	31	1	5	15	215	11
148	148	17	28	40	20	20
83	68	90	63	62	14	46
263	69	21	120	86	41	15
69	193	11	62	70	67	63
11	34	6	12	1	51	3
70	46	20	52	41	66	24
142	137	11	42	31	35	13
64	66	26	67	88	48	20
97	43	23	57	14	70	73
259	52	23	35	42	22	40
75	49	23	78	22	48	50
150	163	15	54	76	24	42
51	40	3	10	19	206	9
275	60	11	107	134	11	26
95	49	15	49	23	35	35
82	74	13	35	44	43	49
143	173	20	40	27	26	30
115	34	17	32	54	47	20
74	99	14	37	21	24	38
42	115	20	36	40	28	34
39	15	2	18	46	27	9
82	43	20	24	48	27	18
36	62	12	18	54	49	8
68	79	38	52	32	22	25
62	71	15	29	43	54	8
33	95	8	13	22	110	19
4	4	0	3	3	233	0
60	40	8	30	18	63	25
46	61	1	8	11	222	2
42	60	8	21	40	17	11

25	41	3	33	15	39	4
51	63	4	23	26	30	22
68	44	4	24	111	10	21
29	66	19	41	42	24	33
60	46	10	45	29	32	33
53	36	20	30	29	25	24
145	54	21	47	30	12	8
49	44	2	27	31	33	13
27	42	1	8	29	69	9
63	29	16	46	37	15	14
5	7	0	1	2	251	0
86	88	10	30	20	7	15
32	53	9	23	22	63	1
48	32	1	38	16	32	12
30	62	5	26	15	14	14
17	75	6	9	4	29	10
23	57	8	10	23	67	9
44	57	9	18	22	23	19
132	43	3	26	132	8	5
31	36	9	14	16	18	2
10	13	1	6	2	74	5
41	58	4	14	19	15	17
41	58	4	14	19	15	17
189	33	14	47	73	2	17
42	21	3	21	27	11	20
51	50	6	31	29	55	7
65	30	5	11	86	3	7
35	54	9	17	15	26	6
28	82	10	18	8	19	4
42	47	4	7	32	15	1
28	24	19	16	29	39	12
29	18	4	21	10	31	11
10	50	14	6	4	20	14
93	37	6	18	2	56	8
15	31	0	2	12	30	22
25	38	2	21	21	26	5
2	3	0	0	2	269	1
29	35	3	8	16	17	11
24	32	2	7	8	35	14
41	42	14	23	10	16	25
51	37	9	20	23	19	5
23	32	2	7	8	28	13
64	37	10	30	40	9	13
91	26	5	15	5	5	13
11	10	9	3	21	37	1
46	22	8	23	18	12	13
1	2	0	0	0	145	0
13	25	5	19	4	19	16
26	12	7	8	18	17	9
45	14	5	11	8	19	10
88	30	2	23	60	3	12
9	18	4	11	14	44	7
4	1	0	0	0	120	3
23	18	2	10	5	18	4
10	29	5	2	12	47	2
0	0	0	0	0	0	0
40	11	11	20	8	14	11
25	44	3	11	24	12	13
45	33	3	30	11	8	11

73	10	5	36	56	1	15
49	19	7	21	15	12	14
62	20	2	23	54	0	11
35	20	3	20	21	21	11
44	35	10	5	13	1	13
40	21	11	18	15	1	12
8	28	5	2	12	36	2
28	10	3	15	39	3	7
4	2	1	1	2	88	0
6	11	2	5	6	62	8
11	31	2	7	20	14	8
42	34	6	14	12	12	7
9	14	6	3	11	29	0
1	17	1	2	8	11	11
61	22	8	20	37	4	6
11	13	5	0	10	25	1
34	8	8	15	12	9	3
10	18	2	4	7	28	7
24	29	4	16	8	13	3
6	13	0	6	29	6	7
72	13	2	24	56	1	8
26	13	7	12	9	7	13
24	31	9	20	7	6	11
23	7	13	12	18	8	6
24	15	8	9	6	15	3
17	12	6	14	3	10	7
9	10	4	4	13	8	2
17	15	8	12	15	3	5
12	21	0	11	7	3	15
21	22	3	7	11	10	0
28	9	6	8	16	5	7
2	4	1	1	0	24	0
4	6	1	3	0	41	0
13	8	2	6	9	20	4
18	17	2	10	6	10	7
18	10	6	6	17	6	7
21	15	3	13	7	7	15
14	13	0	7	2	14	7
14	32	7	8	9	7	6
26	18	9	10	3	1	1
8	9	2	3	8	24	0
21	17	4	14	7	5	4
54	14	3	19	29	4	8
33	16	15	18	14	1	5
20	14	2	15	34	2	4
11	21	0	0	7	0	8
18	27	6	8	7	7	2
19	21	4	10	22	9	4
27	25	1	12	7	4	5
9	19	7	8	6	8	5
22	17	1	10	6	5	4
1	5	0	1	0	101	1
19	22	8	5	13	4	9
7	6	0	0	4	25	5
29	8	6	9	18	9	7
19	19	8	6	8	3	1
15	20	8	5	8	4	7
31	37	5	43	16	27	1
18	7	1	5	3	4	1

20	6	2	5	8	2	0
12	20	15	6	11	3	0
14	19	2	14	6	2	0
20	15	4	15	8	3	9
22	13	10	8	3	2	7
9	21	4	36	4	6	4
11	15	1	4	8	3	2
12	3	1	1	8	20	2
8	6	0	5	7	6	0
11	25	7	2	7	7	2
19	14	7	9	4	2	5
3	11	2	7	7	12	3
6	13	6	13	10	7	0
1	3	0	2	1	39	1
5	6	7	5	2	30	4
10	11	1	5	3	7	0
3	13	2	1	1	23	3
13	14	5	9	5	3	8
1	7	1	1	0	20	2
6	12	1	3	2	6	1
5	7	0	2	0	6	3
3	11	1	3	2	10	4
5	9	0	3	3	17	4
10	7	5	4	5	3	2
10	3	1	6	11	3	3
15	11	2	4	14	5	3
1	5	0	0	2	17	0
1	7	0	0	1	32	0
4	12	3	7	4	2	6
9	9	0	4	1	2	7
2	12	4	1	9	5	4
11	11	4	2	5	2	0
9	9	2	4	3	14	0
1	3	8	3	13	3	2
3	1	0	0	1	27	0
35	1	4	8	15	1	7
8	10	2	4	3	1	5
14	2	4	5	6	5	1
11	5	3	1	5	3	3
11	6	10	6	5	0	1
7	8	7	8	2	2	0
6	7	3	1	7	14	1
22	6	1	5	13	1	2
9	14	2	2	2	1	5
7	8	6	6	7	5	3
1	6	2	1	1	1	5
7	8	2	9	6	7	1
8	10	3	3	4	4	4
0	12	1	0	1	9	4
6	30	0	0	3	9	0
7	3	2	4	1	3	4
14	5	1	3	23	0	2
1	2	1	2	2	10	0
11	5	0	3	2	0	1
10	10	4	2	2	1	1
0	3	0	2	1	33	0
5	6	1	2	3	2	3
10	11	0	5	3	5	1
4	7	0	1	0	1	7

3	7	2	0	5	7	5
9	14	3	9	3	1	0
3	6	0	1	0	6	2
1	1	2	2	0	69	1
6	12	0	10	3	6	5
25	4	1	5	4	0	1
8	6	2	5	4	0	0
4	8	3	1	3	10	0
5	2	6	3	3	2	3
1	5	2	3	0	3	1
1	1	0	0	1	11	0
0	2	0	0	1	33	1
0	0	0	0	0	33	0
1	2	0	0	2	7	1
3	7	1	1	2	1	2
15	6	0	6	2	2	1
13	6	0	9	3	2	2
8	10	5	4	6	3	3
0	0	0	0	1	32	0
8	6	0	1	11	3	1
13	7	0	1	11	1	2
0	3	0	0	0	8	0
6	8	1	4	4	2	3
12	5	1	4	0	2	1
6	2	1	3	6	5	2
0	0	0	0	0	24	0
0	3	1	1	0	4	0
4	3	2	1	5	3	1
8	3	1	5	1	2	3
2	3	0	1	2	4	0
0	0	0	0	0	14	1
3	4	1	2	3	2	2
9	4	0	2	2	2	1
2	2	2	0	2	2	3
3	3	2	2	0	6	8
10	5	1	12	3	4	3
7	5	4	1	1	8	1
3	3	2	1	7	3	0
0	3	2	1	0	1	1
20	6	0	3	8	0	3
6	0	0	1	4	4	1
5	8	1	2	5	8	1
10	2	3	7	0	1	3
2	10	0	2	4	2	2
5	2	2	4	1	2	3
7	6	0	1	14	1	0
0	1	1	0	3	12	0
4	6	0	3	0	2	0
4	1	2	1	1	3	1
5	12	0	3	4	3	3
5	3	2	6	3	2	3
0	2	1	1	2	15	1
6	1	0	3	2	0	7
3	3	2	3	1	2	2
3	7	2	2	0	2	1
4	6	0	3	2	3	1
7	4	0	3	0	1	1
15	12	2	4	2	0	4
10	1	3	4	1	0	1

3	7	1	1	3	3	1
4	5	2	1	0	5	2
1	7	3	1	4	1	2
5	1	0	3	4	0	1
4	1	1	1	2	2	0
1	0	0	0	0	15	0
8	11	0	2	1	3	1
1	1	0	1	3	6	1
3	2	2	1	0	2	2
2	4	0	0	4	0	1
0	3	2	1	0	1	0
7	2	2	2	1	2	0
0	1	0	1	1	18	0
3	0	0	1	1	5	1
2	3	0	4	1	6	1
0	2	0	2	3	1	1
9	9	2	3	1	0	2
16	5	0	8	11	1	0
1	3	1	0	0	4	0
5	4	0	0	2	0	7
0	2	0	0	0	5	1
0	0	0	0	0	11	0
0	6	0	0	1	6	0
5	4	0	2	1	0	1
1	7	0	6	2	0	1
5	4	1	2	0	0	3
7	3	1	2	1	0	2
5	3	1	4	0	0	2
0	0	0	0	0	16	0
1	1	1	0	1	5	0
1	2	1	2	5	2	0
4	2	2	3	1	1	4
2	1	2	5	1	2	1
9	3	0	7	0	0	1
5	4	0	1	0	1	0
5	7	2	1	1	0	3
1	2	0	1	0	2	3
1	1	1	1	0	3	1
4	5	0	0	5	1	0
4	1	0	0	2	2	1
2	2	1	0	1	4	0
6	6	1	1	1	2	1
1	5	3	4	2	0	2
4	2	1	1	0	1	1
3	2	1	1	0	1	2
2	1	0	3	2	1	1
5	2	5	0	1	3	3
1	0	1	2	5	1	0
2	2	1	2	1	2	3
3	5	1	1	1	2	0
5	2	0	0	1	1	0
1	0	0	0	1	5	0
4	0	0	1	3	3	0
1	3	1	0	1	2	1
1	2	2	2	3	0	0
1	2	1	1	2	2	1
2	5	0	3	5	2	0
4	3	2	0	0	1	1
5	7	2	0	1	4	0

4	4	0	0	1	1	0
3	3	0	1	0	1	1
1	5	0	1	1	2	1
2	0	0	0	1	2	0
0	4	0	0	2	1	1
0	0	0	0	2	12	2
3	1	1	2	2	1	0
0	1	0	2	0	0	0
1	0	2	1	3	1	0
10	0	0	0	1	0	0
3	2	0	1	0	0	0
1	1	0	2	4	0	0
0	0	0	0	0	0	0
F_35	F_36	F_37	F_38	F_39	G_3	G_30
CHR_NP	CHR_NP	CHR_NP	CHR_NP	CHR_NP	CHR_NP	CHR_NP
4738402	1092792	634898	2827341	1833244	2149558	1741913
18	10	13	20	16	16	21
-0.66	-2.43	-1.82	-0.34	-1.14	-1.14	-0.12

0.95	0.06	0.63	0.98	0.62	0.98	0.76
3	33	16	1	17	2	14
1	1	1	1	1	1	1
57	58	59	60	61	62	63
30	31	32	33	34	35	36
F_39	G_3	G_30	G_31	G_32	G_4	G_5
CHR_NP	CHR_NP	CHR_NP	CHR_NP	CHR_NP	CHR_NP	CHR_NP
1856730	2304707	924323	555209	358914	3926455	1257195
F_39	G_3	G_30	G_31	G_32	G_4	G_5
806882	330186	233445	60054	27156	715360	584807
135146	80730	89825	81885	50732	166580	98444
110065	70536	115590	43191	49624	78461	70682
125806	117238	76499	63556	41067	197923	106696
81711	112000	127053	57366	33951	151060	106970
74839	337970	10312	24074	19272	669358	14568
63278	146448	43235	48602	33276	252380	37877
3826	255607	1558	3955	3195	304888	3580
46674	132537	13936	10556	3738	214919	10351
38110	54366	18696	10050	4479	125897	19095
41760	36692	9162	8857	2892	58207	8865
29721	24843	16347	12921	6828	42738	20501
28155	59508	17309	14640	9224	80893	8750
20767	17432	13921	7451	4023	33867	26210
10166	52787	2598	6652	3420	83123	1659
15521	4803	4535	1400	588	7967	7460
6990	46001	2812	6284	4297	65216	2153
11550	10033	3807	4796	2853	18322	2596
8434	36055	5974	5219	3205	69159	4040

8110	10606	9427	5029	2619	17863	4956
6725	6812	4791	2626	1310	9992	3862
7740	10373	4887	2776	1160	22460	5937
8362	9213	3233	1742	613	21293	3969
2613	14634	5704	5429	4673	17203	3609
2730	21942	3822	2457	2791	25787	1081
8563	5931	2074	704	418	7579	3527
8258	5835	7276	3489	2027	9094	7577
4240	17088	905	1307	1126	41338	2796
8869	5601	1955	2292	1628	13657	2219
9835	4533	4091	2173	827	8205	4514
5727	7641	2978	4003	2313	11523	2645
4902	7556	4750	2719	1774	11574	6496
2358	24953	888	1704	969	38405	1508
1062	29276	428	1210	558	25718	253
8196	4940	1840	2158	1509	12423	2036
5392	3475	3209	2703	3005	9914	6992
4191	4920	1965	2524	1494	11445	1205
4112	6314	1628	1408	2015	6517	1049
2147	1471	3720	2049	920	4721	4100
1231	3845	4246	974	466	7388	6950
1878	754	2685	1538	1136	1409	1880
2415	4776	3557	2218	1235	3723	2467
3437	1907	1814	1232	915	3808	2879
602	12193	165	563	382	30204	243
1550	2408	1799	868	896	2494	1098
3330	2052	1851	1477	605	2644	1214
3312	4089	912	1093	458	5914	1492
2395	1343	2003	842	276	3222	2347
417	14936	82	187	88	21157	350
468	4329	205	184	51	8480	243
6902	192	1441	228	186	444	406
1175	1566	789	1067	777	1933	385
2076	1458	1106	856	476	1923	1624
1007	1047	484	310	95	2091	325
1036	3036	98	18	4	4507	859
182	15017	37	55	19	17962	108
1500	1967	888	872	667	3703	1109
576	2395	874	791	386	2376	683
1803	2163	1338	1052	576	1430	869
2782	756	1465	1276	911	1024	1720
1315	841	728	977	534	1021	605
548	1838	569	399	172	1929	663
361	10590	42	94	50	14472	107
68	7637	30	39	55	9973	52
2237	529	501	368	143	1498	1372
711	2897	425	447	361	5759	343
642	3817	206	352	185	4518	217
352	5979	146	249	220	7899	207
431	1258	273	255	301	731	172
1460	822	128	98	77	2021	730
934	276	482	474	288	343	551
1169	301	413	130	80	483	436
330	3638	101	223	122	5449	73
875	1171	252	108	47	2458	498
654	1941	485	669	604	2152	311
3828	158	929	143	469	1176	209
674	826	218	36	21	1775	853
1943	744	374	213	217	1197	1260

594	510	643	428	234	653	605
636	333	244	341	219	299	147
683	901	175	216	144	1996	315
535	321	329	55	20	436	401
579	162	352	283	141	790	627
261	1138	52	197	37	1224	50
339	1474	121	238	113	1906	299
278	359	223	80	50	339	258
750	243	411	253	151	470	427
269	2775	93	191	152	3382	92
558	533	309	323	199	855	176
764	421	581	292	173	403	334
472	413	257	128	66	613	346
22	3259	5	7	7	5354	54
189	359	249	68	37	351	200
476	810	123	135	87	1133	106
145	1231	12	36	29	2426	37
235	580	112	190	154	674	44
395	143	294	220	93	200	187
342	168	221	157	50	353	290
321	200	569	221	162	244	250
176	1834	26	84	47	3797	30
143	156	132	69	67	250	153
261	414	215	171	62	833	205
352	326	422	220	153	318	212
286	1161	160	142	169	1119	143
297	721	38	103	39	1575	41
92	1600	45	57	37	2189	69
260	212	140	137	80	153	106
298	248	223	208	190	385	142
205	1242	39	112	77	1598	66
136	1682	111	99	81	2438	85
122	556	176	141	75	574	33
233	223	224	90	68	236	132
112	114	258	131	94	236	268
132	821	55	65	43	2178	73
225	417	293	127	75	450	106
337	147	246	142	64	222	95
125	683	58	50	18	351	51
264	531	65	153	105	633	39
264	531	65	153	105	633	39
97	228	101	107	120	125	81
229	152	72	37	10	173	170
202	58	60	23	16	44	70
384	101	86	10	19	742	17
135	99	50	50	22	270	55
23	2202	7	11	6	2055	17
207	101	87	111	54	162	87
175	140	108	58	51	174	118
204	101	77	35	12	116	49
80	29	63	29	13	38	61
150	104	236	134	68	65	72
184	272	14	2	0	753	547
122	132	82	101	49	165	79
160	211	73	74	29	336	48
111	33	45	32	27	79	73
74	500	23	19	6	555	36
126	173	71	53	26	152	46
106	166	71	11	3	320	62

129	159	35	18	14	257	60
129	165	22	35	31	392	47
77	93	93	26	40	76	86
77	109	36	13	9	161	94
158	52	38	32	23	129	69
32	87	94	32	24	108	96
79	132	65	82	56	223	69
19	1255	3	14	7	1503	8
120	55	47	22	19	225	103
55	39	85	51	27	46	66
83	104	79	79	46	239	42
180	68	45	41	31	260	128
70	87	78	32	45	118	116
55	266	43	26	28	337	26
55	286	33	24	25	619	36
150	48	17	2	4	138	5
50	46	103	50	18	105	94
14	208	119	35	4	186	44
142	63	34	24	30	79	84
40	100	65	53	36	152	71
99	16	38	17	41	39	41
86	74	47	22	18	62	33
116	67	21	4	2	119	110
6	701	4	13	9	1268	1
28	53	50	43	31	65	57
83	41	48	33	42	83	70
30	248	24	15	10	300	19
22	168	45	33	36	193	18
101	48	44	18	6	99	69
62	36	50	29	34	39	50
27	80	23	16	16	15	33
15	641	6	22	21	572	11
97	21	12	9	3	144	45
49	72	56	52	13	89	32
108	117	17	15	7	189	19
96	34	56	40	19	78	36
65	93	29	26	13	148	26
19	182	25	43	10	347	13
6	38	62	54	31	45	49
441	5	32	20	81	50	8
78	159	17	45	22	117	17
31	21	19	7	11	30	20
304	5	35	7	56	45	9
51	56	39	8	7	89	95
36	266	9	20	13	451	13
302	5	43	10	8	12	6
28	146	19	36	22	338	16
119	16	47	33	8	27	20
35	36	51	18	15	64	54
35	255	9	17	12	423	13
207	3	23	1	5	6	2
93	39	47	43	14	112	16
60	79	18	13	12	138	38
6	338	2	0	222	2	23
15	215	11	23	24	217	1
40	20	20	26	13	31	8
62	14	46	53	15	38	55
86	41	15	2	3	98	37
70	67	63	51	39	100	40

1	51	3	1	0	225	3
41	66	24	14	7	183	40
31	35	13	24	14	33	10
88	48	20	8	7	130	10
14	70	73	13	5	51	88
42	22	40	10	7	61	9
22	48	50	22	17	53	35
76	24	42	9	5	26	20
19	206	9	15	8	237	6
134	11	26	3	1	15	16
23	35	35	14	10	164	77
44	43	49	17	12	66	40
27	26	30	15	23	27	11
54	47	20	9	6	77	49
21	24	38	18	11	15	16
40	28	34	17	14	32	35
46	27	9	0	9	114	1
48	27	18	13	8	36	38
54	49	8	9	4	111	8
32	22	25	28	13	63	21
43	54	8	3	3	85	23
22	110	19	14	2	94	16
3	233	0	0	0	360	1
18	63	25	8	17	77	28
11	222	2	22	9	420	69
40	17	11	7	3	63	39
15	39	4	5	3	54	5
26	30	22	8	7	51	17
111	10	21	13	3	20	2
42	24	33	14	13	22	17
29	32	33	28	11	34	27
29	25	24	7	1	54	44
30	12	8	6	8	20	20
31	33	13	7	9	39	9
29	69	9	18	11	77	1
37	15	14	9	5	31	16
2	251	0	3	3	325	2
20	7	15	8	1	11	23
22	63	1	3	0	106	9
16	32	12	4	6	40	38
15	14	14	4	3	13	16
4	29	10	11	10	102	11
23	67	9	13	11	67	2
22	23	19	5	1	58	20
132	8	5	7	6	29	6
16	18	2	1	0	25	3
2	74	5	10	9	132	0
19	15	17	22	10	42	10
19	15	17	22	10	42	10
73	2	17	3	3	2	3
27	11	20	14	6	25	30
29	55	7	14	5	127	31
86	3	7	7	5	21	3
15	26	6	14	13	53	12
8	19	4	8	2	23	12
32	15	1	6	2	22	4
29	39	12	7	9	93	24
10	31	11	2	1	22	9
4	20	14	7	14	21	4

2	56	8	6	4	69	15
12	30	22	5	2	32	12
21	26	5	5	9	25	7
2	269	1	0	1	342	0
16	17	11	7	4	24	18
8	35	14	23	8	58	4
10	16	25	8	10	12	22
23	19	5	5	7	28	23
8	28	13	21	8	53	4
40	9	13	9	0	9	8
5	5	13	8	5	3	8
21	37	1	2	1	50	10
18	12	13	6	5	19	10
0	145	0	1	0	212	1
4	19	16	8	5	10	12
18	17	9	1	3	22	27
8	19	10	4	2	20	28
60	3	12	0	5	9	3
14	44	7	13	7	64	7
0	120	3	1	1	167	1
5	18	4	3	5	15	8
12	47	2	8	8	67	6
0	0	0	0	0	0	0
8	14	11	7	6	20	22
24	12	13	8	16	16	7
11	8	11	4	4	16	14
56	1	15	2	16	10	3
15	12	14	5	1	16	5
54	0	11	3	3	6	1
21	21	11	4	3	17	7
13	1	13	10	3	3	17
15	1	12	7	1	3	9
12	36	2	7	8	49	6
39	3	7	0	2	5	4
2	88	0	2	1	183	3
6	62	8	6	5	100	4
20	14	8	7	3	33	0
12	12	7	7	2	6	9
11	29	0	4	2	36	6
8	11	11	4	0	4	5
37	4	6	2	4	3	1
10	25	1	13	4	29	1
12	9	3	8	3	17	10
7	28	7	3	1	23	4
8	13	3	1	0	8	7
29	6	7	1	1	8	0
56	1	8	1	11	18	8
9	7	13	4	1	1	3
7	6	11	5	4	10	10
18	8	6	1	2	15	16
6	15	3	7	2	19	1
3	10	7	0	1	24	15
13	8	2	5	3	5	5
15	3	5	2	2	6	4
7	3	15	3	1	4	11
11	10	0	4	4	18	2
16	5	7	3	3	19	12
0	24	0	2	1	54	0
0	41	0	4	4	54	0

9	20	4	2	2	50	9
6	10	7	2	3	25	3
17	6	7	2	0	6	6
7	7	15	8	2	8	1
2	14	7	0	1	2	10
9	7	6	10	7	16	11
3	1	1	3	1	2	6
8	24	0	2	3	17	0
7	5	4	6	0	7	5
29	4	8	1	0	3	3
14	1	5	2	6	7	2
34	2	4	0	1	4	1
7	0	8	2	0	3	3
7	7	2	2	0	10	2
22	9	4	3	2	15	2
7	4	5	0	2	6	7
6	8	5	4	4	4	4
6	5	4	6	3	4	4
0	101	1	2	1	63	0
13	4	9	9	5	7	6
4	25	5	5	2	31	2
18	9	7	7	0	18	6
8	3	1	2	1	1	3
8	4	7	0	1	5	5
16	27	1	1	0	46	27
3	4	1	4	0	23	6
8	2	0	0	0	6	1
11	3	0	0	0	0	1
6	2	0	1	1	3	1
8	3	9	6	1	7	2
3	2	7	1	0	7	11
4	6	4	3	0	4	6
8	3	2	0	1	12	5
8	20	2	1	3	51	0
7	6	0	0	3	21	2
7	7	2	1	2	6	2
4	2	5	2	0	3	4
7	12	3	1	0	10	5
10	7	0	2	0	2	2
1	39	1	3	2	53	0
2	30	4	0	2	3	7
3	7	0	1	0	9	0
1	23	3	3	1	9	6
5	3	8	4	3	3	4
0	20	2	2	1	17	0
2	6	1	2	0	12	3
0	6	3	0	3	6	1
2	10	4	2	0	8	5
3	17	4	11	0	4	2
5	3	2	0	0	8	6
11	3	3	1	0	9	2
14	5	3	2	1	3	3
2	17	0	0	1	26	1
1	32	0	2	1	31	2
4	2	6	0	0	3	8
1	2	7	1	1	6	4
9	5	4	1	2	8	28
5	2	0	2	0	3	4
3	14	0	0	0	11	0

13	3	2	1	0	0	1
1	27	0	0	1	41	0
15	1	7	1	3	3	2
3	1	5	3	0	7	1
6	5	1	0	1	5	4
5	3	3	1	3	3	4
5	0	1	0	2	5	8
2	2	0	1	1	7	3
7	14	1	3	1	7	0
13	1	2	0	0	5	1
2	1	5	3	2	7	2
7	5	3	0	0	3	6
1	1	5	2	0	1	0
6	7	1	2	0	8	3
4	4	4	1	1	2	5
1	9	4	6	1	13	1
3	9	0	0	0	4	0
1	3	4	1	1	4	4
23	0	2	1	0	8	2
2	10	0	0	2	16	0
2	0	1	0	4	0	1
2	1	1	0	1	3	2
1	33	0	0	2	16	0
3	2	3	1	0	7	2
3	5	1	0	2	1	1
0	1	7	0	3	11	1
5	7	5	2	1	18	1
3	1	0	0	0	1	3
0	6	2	0	1	6	0
0	69	1	0	0	9	0
3	6	5	1	2	6	4
4	0	1	1	0	4	6
4	0	0	0	0	0	0
3	10	0	3	0	10	3
3	2	3	3	0	8	1
0	3	1	0	0	5	0
1	11	0	3	0	13	0
1	33	1	1	0	58	1
0	33	0	0	0	43	0
2	7	1	0	0	6	1
2	1	2	0	0	1	2
2	2	1	2	1	7	0
3	2	2	0	0	1	2
6	3	3	5	5	6	3
1	32	0	0	0	40	0
11	3	1	0	1	7	1
11	1	2	1	2	9	0
0	8	0	1	0	2	1
4	2	3	3	0	1	0
0	2	1	2	1	2	0
6	5	2	0	0	4	5
0	24	0	0	0	24	0
0	4	0	0	0	2	0
5	3	1	2	1	10	1
1	2	3	0	2	8	3
2	4	0	1	0	6	1
0	14	1	1	1	19	0
3	2	2	0	1	4	0
2	2	1	1	0	2	1

2	2	3	0	0	1	2
0	6	8	1	1	4	1
3	4	3	1	0	4	2
1	8	1	1	0	4	4
7	3	0	1	0	8	1
0	1	1	1	0	2	0
8	0	3	0	3	0	0
4	4	1	1	0	12	0
5	8	1	1	1	2	0
0	1	3	1	2	1	1
4	2	2	0	0	3	0
1	2	3	1	1	5	0
14	1	0	0	2	14	0
3	12	0	1	0	22	0
0	2	0	0	0	2	0
1	3	1	4	0	4	1
4	3	3	1	1	1	1
3	2	3	0	1	2	1
2	15	1	0	0	16	1
2	0	7	1	0	5	1
1	2	2	0	2	3	1
0	2	1	1	1	4	1
2	3	1	1	2	9	2
0	1	1	0	1	5	2
2	0	4	0	0	2	1
1	0	1	0	1	1	1
3	3	1	1	0	1	1
0	5	2	1	0	2	0
4	1	2	1	0	3	0
4	0	1	0	0	2	3
2	2	0	1	0	8	2
0	15	0	1	0	18	1
1	3	1	0	0	2	1
3	6	1	0	0	11	0
0	2	2	0	1	4	1
4	0	1	1	0	6	1
0	1	0	0	0	1	0
1	2	0	0	0	5	2
1	18	0	1	2	14	0
1	5	1	0	0	6	2
1	6	1	1	0	9	3
3	1	1	1	0	4	1
1	0	2	2	0	1	3
11	1	0	0	0	19	3
0	4	0	0	0	14	0
2	0	7	0	2	2	2
0	5	1	0	0	8	0
0	11	0	0	0	17	0
1	6	0	1	0	10	0
1	0	1	0	1	0	1
2	0	1	2	0	0	0
0	0	3	3	1	3	0
1	0	2	1	0	0	0
0	0	2	1	0	0	1
0	16	0	0	0	12	0
1	5	0	0	0	18	1
5	2	0	1	5	11	0
1	1	4	0	1	2	2
1	2	1	1	1	4	2

0	0	1	0	0	2	1
0	1	0	2	1	1	1
1	0	3	0	1	3	0
0	2	3	0	1	1	2
0	3	1	0	0	3	1
5	1	0	1	0	2	1
2	2	1	2	0	0	1
1	4	0	0	0	7	0
1	2	1	2	0	0	0
2	0	2	1	0	1	0
0	1	1	0	0	2	4
0	1	2	0	0	4	1
2	1	1	0	2	4	2
1	3	3	0	1	2	1
5	1	0	0	0	1	0
1	2	3	1	0	1	0
1	2	0	1	0	2	1
1	1	0	1	1	0	0
1	5	0	1	0	10	0
3	3	0	0	0	6	1
1	2	1	0	0	2	0
3	0	0	1	0	5	2
2	2	1	1	0	2	2
5	2	0	0	0	1	2
0	1	1	0	0	0	1
1	4	0	0	0	1	0
1	1	0	1	1	2	0
0	1	1	1	0	0	1
1	2	1	0	0	4	0
1	2	0	0	0	3	1
2	1	1	0	0	3	0
2	12	2	4	1	6	1
2	1	0	1	0	1	0
0	0	0	0	0	3	1
3	1	0	0	0	2	0
1	0	0	1	0	0	4
0	0	0	1	3	1	0
4	0	0	0	0	2	2

0.95	0.06	0.63	0.98	0.62	0.98	0.76
3	33	16	1	17	2	14
1	1	1	1	1	1	1
57	58	59	60	61	62	63
30	31	32	33	34	35	36
F_39	G_3	G_30	G_31	G_32	G_4	G_5
CHR_NP	CHR_NP	CHR_NP	CHR_NP	CHR_NP	CHR_NP	CHR_NP
1856730	2304707	924323	555209	358914	3926455	1257195
CHR_NP	CHR_NP	CHR_NP	CHR_NP	CHR_NP	CHR_NP	CHR_NP
806882	330186	233445	60054	27156	715360	584807
135146	80730	89825	81885	50732	166580	98444
110065	70536	115590	43191	49624	78461	70682
125806	117238	76499	63556	41067	197923	106696
81711	112000	127053	57366	33951	151060	106970

74839	337970	10312	24074	19272	669358	14568
63278	146448	43235	48602	33276	252380	37877
3826	255607	1558	3955	3195	304888	3580
46674	132537	13936	10556	3738	214919	10351
38110	54366	18696	10050	4479	125897	19095
41760	36692	9162	8857	2892	58207	8865
29721	24843	16347	12921	6828	42738	20501
28155	59508	17309	14640	9224	80893	8750
20767	17432	13921	7451	4023	33867	26210
10166	52787	2598	6652	3420	83123	1659
15521	4803	4535	1400	588	7967	7460
6990	46001	2812	6284	4297	65216	2153
11550	10033	3807	4796	2853	18322	2596
8434	36055	5974	5219	3205	69159	4040
8110	10606	9427	5029	2619	17863	4956
6725	6812	4791	2626	1310	9992	3862
7740	10373	4887	2776	1160	22460	5937
8362	9213	3233	1742	613	21293	3969
2613	14634	5704	5429	4673	17203	3609
2730	21942	3822	2457	2791	25787	1081
8563	5931	2074	704	418	7579	3527
8258	5835	7276	3489	2027	9094	7577
4240	17088	905	1307	1126	41338	2796
8869	5601	1955	2292	1628	13657	2219
9835	4533	4091	2173	827	8205	4514
5727	7641	2978	4003	2313	11523	2645
4902	7556	4750	2719	1774	11574	6496
2358	24953	888	1704	969	38405	1508
1062	29276	428	1210	558	25718	253
8196	4940	1840	2158	1509	12423	2036
5392	3475	3209	2703	3005	9914	6992
4191	4920	1965	2524	1494	11445	1205
4112	6314	1628	1408	2015	6517	1049
2147	1471	3720	2049	920	4721	4100
1231	3845	4246	974	466	7388	6950
1878	754	2685	1538	1136	1409	1880
2415	4776	3557	2218	1235	3723	2467
3437	1907	1814	1232	915	3808	2879
602	12193	165	563	382	30204	243
1550	2408	1799	868	896	2494	1098
3330	2052	1851	1477	605	2644	1214
3312	4089	912	1093	458	5914	1492
2395	1343	2003	842	276	3222	2347
417	14936	82	187	88	21157	350
468	4329	205	184	51	8480	243
6902	192	1441	228	186	444	406
1175	1566	789	1067	777	1933	385
2076	1458	1106	856	476	1923	1624
1007	1047	484	310	95	2091	325
1036	3036	98	18	4	4507	859
182	15017	37	55	19	17962	108
1500	1967	888	872	667	3703	1109
576	2395	874	791	386	2376	683
1803	2163	1338	1052	576	1430	869
2782	756	1465	1276	911	1024	1720
1315	841	728	977	534	1021	605
548	1838	569	399	172	1929	663
361	10590	42	94	50	14472	107
68	7637	30	39	55	9973	52

2237	529	501	368	143	1498	1372
711	2897	425	447	361	5759	343
642	3817	206	352	185	4518	217
352	5979	146	249	220	7899	207
431	1258	273	255	301	731	172
1460	822	128	98	77	2021	730
934	276	482	474	288	343	551
1169	301	413	130	80	483	436
330	3638	101	223	122	5449	73
875	1171	252	108	47	2458	498
654	1941	485	669	604	2152	311
3828	158	929	143	469	1176	209
674	826	218	36	21	1775	853
1943	744	374	213	217	1197	1260
594	510	643	428	234	653	605
636	333	244	341	219	299	147
683	901	175	216	144	1996	315
535	321	329	55	20	436	401
579	162	352	283	141	790	627
261	1138	52	197	37	1224	50
339	1474	121	238	113	1906	299
278	359	223	80	50	339	258
750	243	411	253	151	470	427
269	2775	93	191	152	3382	92
558	533	309	323	199	855	176
764	421	581	292	173	403	334
472	413	257	128	66	613	346
22	3259	5	7	7	5354	54
189	359	249	68	37	351	200
476	810	123	135	87	1133	106
145	1231	12	36	29	2426	37
235	580	112	190	154	674	44
395	143	294	220	93	200	187
342	168	221	157	50	353	290
321	200	569	221	162	244	250
176	1834	26	84	47	3797	30
143	156	132	69	67	250	153
261	414	215	171	62	833	205
352	326	422	220	153	318	212
286	1161	160	142	169	1119	143
297	721	38	103	39	1575	41
92	1600	45	57	37	2189	69
260	212	140	137	80	153	106
298	248	223	208	190	385	142
205	1242	39	112	77	1598	66
136	1682	111	99	81	2438	85
122	556	176	141	75	574	33
233	223	224	90	68	236	132
112	114	258	131	94	236	268
132	821	55	65	43	2178	73
225	417	293	127	75	450	106
337	147	246	142	64	222	95
125	683	58	50	18	351	51
264	531	65	153	105	633	39
264	531	65	153	105	633	39
97	228	101	107	120	125	81
229	152	72	37	10	173	170
202	58	60	23	16	44	70
384	101	86	10	19	742	17

135	99	50	50	22	270	55
23	2202	7	11	6	2055	17
207	101	87	111	54	162	87
175	140	108	58	51	174	118
204	101	77	35	12	116	49
80	29	63	29	13	38	61
150	104	236	134	68	65	72
184	272	14	2	0	753	547
122	132	82	101	49	165	79
160	211	73	74	29	336	48
111	33	45	32	27	79	73
74	500	23	19	6	555	36
126	173	71	53	26	152	46
106	166	71	11	3	320	62
129	159	35	18	14	257	60
129	165	22	35	31	392	47
77	93	93	26	40	76	86
77	109	36	13	9	161	94
158	52	38	32	23	129	69
32	87	94	32	24	108	96
79	132	65	82	56	223	69
19	1255	3	14	7	1503	8
120	55	47	22	19	225	103
55	39	85	51	27	46	66
83	104	79	79	46	239	42
180	68	45	41	31	260	128
70	87	78	32	45	118	116
55	266	43	26	28	337	26
55	286	33	24	25	619	36
150	48	17	2	4	138	5
50	46	103	50	18	105	94
14	208	119	35	4	186	44
142	63	34	24	30	79	84
40	100	65	53	36	152	71
99	16	38	17	41	39	41
86	74	47	22	18	62	33
116	67	21	4	2	119	110
6	701	4	13	9	1268	1
28	53	50	43	31	65	57
83	41	48	33	42	83	70
30	248	24	15	10	300	19
22	168	45	33	36	193	18
101	48	44	18	6	99	69
62	36	50	29	34	39	50
27	80	23	16	16	15	33
15	641	6	22	21	572	11
97	21	12	9	3	144	45
49	72	56	52	13	89	32
108	117	17	15	7	189	19
96	34	56	40	19	78	36
65	93	29	26	13	148	26
19	182	25	43	10	347	13
6	38	62	54	31	45	49
441	5	32	20	81	50	8
78	159	17	45	22	117	17
31	21	19	7	11	30	20
304	5	35	7	56	45	9
51	56	39	8	7	89	95
36	266	9	20	13	451	13

302	5	43	10	8	12	6
28	146	19	36	22	338	16
119	16	47	33	8	27	20
35	36	51	18	15	64	54
35	255	9	17	12	423	13
207	3	23	1	5	6	2
93	39	47	43	14	112	16
60	79	18	13	12	138	38
6	338	2	0	222	2	23
15	215	11	23	24	217	1
40	20	20	26	13	31	8
62	14	46	53	15	38	55
86	41	15	2	3	98	37
70	67	63	51	39	100	40
1	51	3	1	0	225	3
41	66	24	14	7	183	40
31	35	13	24	14	33	10
88	48	20	8	7	130	10
14	70	73	13	5	51	88
42	22	40	10	7	61	9
22	48	50	22	17	53	35
76	24	42	9	5	26	20
19	206	9	15	8	237	6
134	11	26	3	1	15	16
23	35	35	14	10	164	77
44	43	49	17	12	66	40
27	26	30	15	23	27	11
54	47	20	9	6	77	49
21	24	38	18	11	15	16
40	28	34	17	14	32	35
46	27	9	0	9	114	1
48	27	18	13	8	36	38
54	49	8	9	4	111	8
32	22	25	28	13	63	21
43	54	8	3	3	85	23
22	110	19	14	2	94	16
3	233	0	0	0	360	1
18	63	25	8	17	77	28
11	222	2	22	9	420	69
40	17	11	7	3	63	39
15	39	4	5	3	54	5
26	30	22	8	7	51	17
111	10	21	13	3	20	2
42	24	33	14	13	22	17
29	32	33	28	11	34	27
29	25	24	7	1	54	44
30	12	8	6	8	20	20
31	33	13	7	9	39	9
29	69	9	18	11	77	1
37	15	14	9	5	31	16
2	251	0	3	3	325	2
20	7	15	8	1	11	23
22	63	1	3	0	106	9
16	32	12	4	6	40	38
15	14	14	4	3	13	16
4	29	10	11	10	102	11
23	67	9	13	11	67	2
22	23	19	5	1	58	20
132	8	5	7	6	29	6

16	18	2	1	0	25	3
2	74	5	10	9	132	0
19	15	17	22	10	42	10
19	15	17	22	10	42	10
73	2	17	3	3	2	3
27	11	20	14	6	25	30
29	55	7	14	5	127	31
86	3	7	7	5	21	3
15	26	6	14	13	53	12
8	19	4	8	2	23	12
32	15	1	6	2	22	4
29	39	12	7	9	93	24
10	31	11	2	1	22	9
4	20	14	7	14	21	4
2	56	8	6	4	69	15
12	30	22	5	2	32	12
21	26	5	5	9	25	7
2	269	1	0	1	342	0
16	17	11	7	4	24	18
8	35	14	23	8	58	4
10	16	25	8	10	12	22
23	19	5	5	7	28	23
8	28	13	21	8	53	4
40	9	13	9	0	9	8
5	5	13	8	5	3	8
21	37	1	2	1	50	10
18	12	13	6	5	19	10
0	145	0	1	0	212	1
4	19	16	8	5	10	12
18	17	9	1	3	22	27
8	19	10	4	2	20	28
60	3	12	0	5	9	3
14	44	7	13	7	64	7
0	120	3	1	1	167	1
5	18	4	3	5	15	8
12	47	2	8	8	67	6
0	0	0	0	0	0	0
8	14	11	7	6	20	22
24	12	13	8	16	16	7
11	8	11	4	4	16	14
56	1	15	2	16	10	3
15	12	14	5	1	16	5
54	0	11	3	3	6	1
21	21	11	4	3	17	7
13	1	13	10	3	3	17
15	1	12	7	1	3	9
12	36	2	7	8	49	6
39	3	7	0	2	5	4
2	88	0	2	1	183	3
6	62	8	6	5	100	4
20	14	8	7	3	33	0
12	12	7	7	2	6	9
11	29	0	4	2	36	6
8	11	11	4	0	4	5
37	4	6	2	4	3	1
10	25	1	13	4	29	1
12	9	3	8	3	17	10
7	28	7	3	1	23	4
8	13	3	1	0	8	7

29	6	7	1	1	8	0
56	1	8	1	11	18	8
9	7	13	4	1	1	3
7	6	11	5	4	10	10
18	8	6	1	2	15	16
6	15	3	7	2	19	1
3	10	7	0	1	24	15
13	8	2	5	3	5	5
15	3	5	2	2	6	4
7	3	15	3	1	4	11
11	10	0	4	4	18	2
16	5	7	3	3	19	12
0	24	0	2	1	54	0
0	41	0	4	4	54	0
9	20	4	2	2	50	9
6	10	7	2	3	25	3
17	6	7	2	0	6	6
7	7	15	8	2	8	1
2	14	7	0	1	2	10
9	7	6	10	7	16	11
3	1	1	3	1	2	6
8	24	0	2	3	17	0
7	5	4	6	0	7	5
29	4	8	1	0	3	3
14	1	5	2	6	7	2
34	2	4	0	1	4	1
7	0	8	2	0	3	3
7	7	2	2	0	10	2
22	9	4	3	2	15	2
7	4	5	0	2	6	7
6	8	5	4	4	4	4
6	5	4	6	3	4	4
0	101	1	2	1	63	0
13	4	9	9	5	7	6
4	25	5	5	2	31	2
18	9	7	7	0	18	6
8	3	1	2	1	1	3
8	4	7	0	1	5	5
16	27	1	1	0	46	27
3	4	1	4	0	23	6
8	2	0	0	0	6	1
11	3	0	0	0	0	1
6	2	0	1	1	3	1
8	3	9	6	1	7	2
3	2	7	1	0	7	11
4	6	4	3	0	4	6
8	3	2	0	1	12	5
8	20	2	1	3	51	0
7	6	0	0	3	21	2
7	7	2	1	2	6	2
4	2	5	2	0	3	4
7	12	3	1	0	10	5
10	7	0	2	0	2	2
1	39	1	3	2	53	0
2	30	4	0	2	3	7
3	7	0	1	0	9	0
1	23	3	3	1	9	6
5	3	8	4	3	3	4
0	20	2	2	1	17	0

2	6	1	2	0	12	3
0	6	3	0	3	6	1
2	10	4	2	0	8	5
3	17	4	11	0	4	2
5	3	2	0	0	8	6
11	3	3	1	0	9	2
14	5	3	2	1	3	3
2	17	0	0	1	26	1
1	32	0	2	1	31	2
4	2	6	0	0	3	8
1	2	7	1	1	6	4
9	5	4	1	2	8	28
5	2	0	2	0	3	4
3	14	0	0	0	11	0
13	3	2	1	0	0	1
1	27	0	0	1	41	0
15	1	7	1	3	3	2
3	1	5	3	0	7	1
6	5	1	0	1	5	4
5	3	3	1	3	3	4
5	0	1	0	2	5	8
2	2	0	1	1	7	3
7	14	1	3	1	7	0
13	1	2	0	0	5	1
2	1	5	3	2	7	2
7	5	3	0	0	3	6
1	1	5	2	0	1	0
6	7	1	2	0	8	3
4	4	4	1	1	2	5
1	9	4	6	1	13	1
3	9	0	0	0	4	0
1	3	4	1	1	4	4
23	0	2	1	0	8	2
2	10	0	0	2	16	0
2	0	1	0	4	0	1
2	1	1	0	1	3	2
1	33	0	0	2	16	0
3	2	3	1	0	7	2
3	5	1	0	2	1	1
0	1	7	0	3	11	1
5	7	5	2	1	18	1
3	1	0	0	0	1	3
0	6	2	0	1	6	0
0	69	1	0	0	9	0
3	6	5	1	2	6	4
4	0	1	1	0	4	6
4	0	0	0	0	0	0
3	10	0	3	0	10	3
3	2	3	3	0	8	1
0	3	1	0	0	5	0
1	11	0	3	0	13	0
1	33	1	1	0	58	1
0	33	0	0	0	43	0
2	7	1	0	0	6	1
2	1	2	0	0	1	2
2	2	1	2	1	7	0
3	2	2	0	0	1	2
6	3	3	5	5	6	3
1	32	0	0	0	40	0

11	3	1	0	1	7	1
11	1	2	1	2	9	0
0	8	0	1	0	2	1
4	2	3	3	0	1	0
0	2	1	2	1	2	0
6	5	2	0	0	4	5
0	24	0	0	0	24	0
0	4	0	0	0	2	0
5	3	1	2	1	10	1
1	2	3	0	2	8	3
2	4	0	1	0	6	1
0	14	1	1	1	19	0
3	2	2	0	1	4	0
2	2	1	1	0	2	1
2	2	3	0	0	1	2
0	6	8	1	1	4	1
3	4	3	1	0	4	2
1	8	1	1	0	4	4
7	3	0	1	0	8	1
0	1	1	1	0	2	0
8	0	3	0	3	0	0
4	4	1	1	0	12	0
5	8	1	1	1	2	0
0	1	3	1	2	1	1
4	2	2	0	0	3	0
1	2	3	1	1	5	0
14	1	0	0	2	14	0
3	12	0	1	0	22	0
0	2	0	0	0	2	0
1	3	1	4	0	4	1
4	3	3	1	1	1	1
3	2	3	0	1	2	1
2	15	1	0	0	16	1
2	0	7	1	0	5	1
1	2	2	0	2	3	1
0	2	1	1	1	4	1
2	3	1	1	2	9	2
0	1	1	0	1	5	2
2	0	4	0	0	2	1
1	0	1	0	1	1	1
3	3	1	1	0	1	1
0	5	2	1	0	2	0
4	1	2	1	0	3	0
4	0	1	0	0	2	3
2	2	0	1	0	8	2
0	15	0	1	0	18	1
1	3	1	0	0	2	1
3	6	1	0	0	11	0
0	2	2	0	1	4	1
4	0	1	1	0	6	1
0	1	0	0	0	1	0
1	2	0	0	0	5	2
1	18	0	1	2	14	0
1	5	1	0	0	6	2
1	6	1	1	0	9	3
3	1	1	1	0	4	1
1	0	2	2	0	1	3
11	1	0	0	0	19	3
0	4	0	0	0	14	0

2	0	7	0	2	2	2
0	5	1	0	0	8	0
0	11	0	0	0	17	0
1	6	0	1	0	10	0
1	0	1	0	1	0	1
2	0	1	2	0	0	0
0	0	3	3	1	3	0
1	0	2	1	0	0	0
0	0	2	1	0	0	1
0	16	0	0	0	12	0
1	5	0	0	0	18	1
5	2	0	1	5	11	0
1	1	4	0	1	2	2
1	2	1	1	1	4	2
0	0	1	0	0	2	1
0	1	0	2	1	1	1
1	0	3	0	1	3	0
0	2	3	0	1	1	2
0	3	1	0	0	3	1
5	1	0	1	0	2	1
2	2	1	2	0	0	1
1	4	0	0	0	7	0
1	2	1	2	0	0	0
2	0	2	1	0	1	0
0	1	1	0	0	2	4
0	1	2	0	0	4	1
2	1	1	0	2	4	2
1	3	3	0	1	2	1
5	1	0	0	0	1	0
1	2	3	1	0	1	0
1	2	0	1	0	2	1
1	1	0	1	1	0	0
1	5	0	1	0	10	0
3	3	0	0	0	6	1
1	2	1	0	0	2	0
3	0	0	1	0	5	2
2	2	1	1	0	2	2
5	2	0	0	0	1	2
0	1	1	0	0	0	1
1	4	0	0	0	1	0
1	1	0	1	1	2	0
0	1	1	1	0	0	1
1	2	1	0	0	4	0
1	2	0	0	0	3	1
2	1	1	0	0	3	0
2	12	2	4	1	6	1
2	1	0	1	0	1	0
0	0	0	0	0	3	1
3	1	0	0	0	2	0
1	0	0	1	0	0	4
0	0	0	1	3	1	0
4	0	0	0	0	2	2
57848	67945	28493	16718	10767	117693	39312
146433	91620	51625	22778	15438	175432	107773
381	366	422	424	444	345	421
CHR_NP	CHR_NP	CHR_NP	CHR_NP	CHR_NP	CHR_NP	CHR_NP
CHR_NP	CHR_NP	CHR_NP	CHR_NP	CHR_NP	CHR_NP	CHR_NP

57	58	59	60	61	62	63
30	31	32	33	34	35	36
CHR_NP	CHR_NP	CHR_NP	CHR_NP	CHR_NP	CHR_NP	CHR_NP
F_39	G_3	G_30	G_31	G_32	G_4	G_5
1856730	2304707	924323	555209	358914	3926455	1257195
CHR_NP	CHR_NP	CHR_NP	CHR_NP	CHR_NP	CHR_NP	CHR_NP
13.95	4.86	8.19	3.59	2.52	6.08	14.88
2.34	1.19	3.15	4.90	4.71	1.42	2.50
1.90	1.04	4.06	2.58	4.61	0.67	1.80
2.17	1.73	2.68	3.80	3.81	1.68	2.71
1.41	1.65	4.46	3.43	3.15	1.28	2.72
1.29	4.97	0.36	1.44	1.79	5.69	0.37
1.09	2.16	1.52	2.91	3.09	2.14	0.96
0.07	3.76	0.05	0.24	0.30	2.59	0.09
0.81	1.95	0.49	0.63	0.35	1.83	0.26
0.66	0.80	0.66	0.60	0.42	1.07	0.49
0.72	0.54	0.32	0.53	0.27	0.49	0.23
0.51	0.37	0.57	0.77	0.63	0.36	0.52
0.49	0.88	0.61	0.88	0.86	0.69	0.22
0.36	0.26	0.49	0.45	0.37	0.29	0.67
0.18	0.78	0.09	0.40	0.32	0.71	0.04
0.27	0.07	0.16	0.08	0.05	0.07	0.19
0.12	0.68	0.10	0.38	0.40	0.55	0.05
0.20	0.15	0.13	0.29	0.26	0.16	0.07
0.15	0.53	0.21	0.31	0.30	0.59	0.10
0.14	0.16	0.33	0.30	0.24	0.15	0.13
0.12	0.10	0.17	0.16	0.12	0.08	0.10
0.13	0.15	0.17	0.17	0.11	0.19	0.15
0.14	0.14	0.11	0.10	0.06	0.18	0.10
0.05	0.22	0.20	0.32	0.43	0.15	0.09
0.05	0.32	0.13	0.15	0.26	0.22	0.03
0.15	0.09	0.07	0.04	0.04	0.06	0.09
0.14	0.09	0.26	0.21	0.19	0.08	0.19
0.07	0.25	0.03	0.08	0.10	0.35	0.07
0.15	0.08	0.07	0.14	0.15	0.12	0.06
0.17	0.07	0.14	0.13	0.08	0.07	0.11
0.10	0.11	0.10	0.24	0.21	0.10	0.07
0.08	0.11	0.17	0.16	0.16	0.10	0.17
0.04	0.37	0.03	0.10	0.09	0.33	0.04
0.02	0.43	0.02	0.07	0.05	0.22	0.01
0.14	0.07	0.06	0.13	0.14	0.11	0.05
0.09	0.05	0.11	0.16	0.28	0.08	0.18
0.07	0.07	0.07	0.15	0.14	0.10	0.03
0.07	0.09	0.06	0.08	0.19	0.06	0.03
0.04	0.02	0.13	0.12	0.09	0.04	0.10
0.02	0.06	0.15	0.06	0.04	0.06	0.18
0.03	0.01	0.09	0.09	0.11	0.01	0.05
0.04	0.07	0.12	0.13	0.11	0.03	0.06
0.06	0.03	0.06	0.07	0.08	0.03	0.07
0.01	0.18	0.01	0.03	0.04	0.26	0.01
0.03	0.04	0.06	0.05	0.08	0.02	0.03
0.06	0.03	0.06	0.09	0.06	0.02	0.03
0.06	0.06	0.03	0.07	0.04	0.05	0.04
0.04	0.02	0.07	0.05	0.03	0.03	0.06
0.01	0.22	0.00	0.01	0.01	0.18	0.01
0.01	0.06	0.01	0.01	0.00	0.07	0.01
0.12	0.00	0.05	0.01	0.02	0.00	0.01
0.02	0.02	0.03	0.06	0.07	0.02	0.01

0.04	0.02	0.04	0.05	0.04	0.02	0.04
0.02	0.02	0.02	0.02	0.01	0.02	0.01
0.02	0.04	0.00	0.00	0.00	0.04	0.02
0.00	0.22	0.00	0.00	0.00	0.15	0.00
0.03	0.03	0.03	0.05	0.06	0.03	0.03
0.01	0.04	0.03	0.05	0.04	0.02	0.02
0.03	0.03	0.05	0.06	0.05	0.01	0.02
0.05	0.01	0.05	0.08	0.08	0.01	0.04
0.02	0.01	0.03	0.06	0.05	0.01	0.02
0.01	0.03	0.02	0.02	0.02	0.02	0.02
0.01	0.16	0.00	0.01	0.00	0.12	0.00
0.00	0.11	0.00	0.00	0.01	0.08	0.00
0.04	0.01	0.02	0.02	0.01	0.01	0.03
0.01	0.04	0.01	0.03	0.03	0.05	0.01
0.01	0.06	0.01	0.02	0.02	0.04	0.01
0.01	0.09	0.01	0.01	0.02	0.07	0.01
0.01	0.02	0.01	0.02	0.03	0.01	0.00
0.03	0.01	0.00	0.01	0.01	0.02	0.02
0.02	0.00	0.02	0.03	0.03	0.00	0.01
0.02	0.00	0.01	0.01	0.01	0.00	0.01
0.01	0.05	0.00	0.01	0.01	0.05	0.00
0.02	0.02	0.01	0.01	0.00	0.02	0.01
0.01	0.03	0.02	0.04	0.06	0.02	0.01
0.07	0.00	0.03	0.01	0.04	0.01	0.01
0.01	0.01	0.01	0.00	0.00	0.02	0.02
0.03	0.01	0.01	0.01	0.02	0.01	0.03
0.01	0.01	0.02	0.03	0.02	0.01	0.02
0.01	0.00	0.01	0.02	0.02	0.00	0.00
0.01	0.01	0.01	0.01	0.01	0.02	0.01
0.01	0.00	0.01	0.00	0.00	0.00	0.01
0.01	0.00	0.01	0.02	0.01	0.01	0.02
0.00	0.02	0.00	0.01	0.00	0.01	0.00
0.01	0.02	0.00	0.01	0.01	0.02	0.01
0.00	0.01	0.01	0.00	0.00	0.00	0.01
0.01	0.00	0.01	0.02	0.01	0.00	0.01
0.00	0.04	0.00	0.01	0.01	0.03	0.00
0.01	0.01	0.01	0.02	0.02	0.01	0.00
0.01	0.01	0.02	0.02	0.02	0.00	0.01
0.01	0.01	0.01	0.01	0.01	0.01	0.01
0.00	0.05	0.00	0.00	0.00	0.05	0.00
0.00	0.01	0.01	0.00	0.00	0.00	0.01
0.01	0.01	0.00	0.01	0.01	0.01	0.00
0.00	0.02	0.00	0.00	0.00	0.02	0.00
0.00	0.01	0.00	0.01	0.01	0.01	0.00
0.01	0.00	0.01	0.01	0.01	0.00	0.00
0.01	0.00	0.01	0.01	0.00	0.00	0.01
0.01	0.00	0.02	0.01	0.02	0.00	0.01
0.00	0.03	0.00	0.01	0.00	0.03	0.00
0.00	0.00	0.00	0.00	0.01	0.00	0.00
0.00	0.01	0.01	0.01	0.01	0.01	0.01
0.01	0.00	0.01	0.01	0.01	0.00	0.01
0.00	0.02	0.01	0.01	0.02	0.01	0.00
0.01	0.01	0.00	0.01	0.00	0.01	0.00
0.00	0.02	0.00	0.00	0.00	0.02	0.00
0.00	0.00	0.00	0.01	0.01	0.00	0.00
0.01	0.00	0.01	0.01	0.02	0.00	0.00
0.00	0.02	0.00	0.01	0.01	0.01	0.00
0.00	0.02	0.00	0.01	0.01	0.02	0.00
0.00	0.01	0.01	0.01	0.01	0.00	0.00

0.00	0.00	0.01	0.01	0.01	0.00	0.00
0.00	0.00	0.01	0.01	0.01	0.00	0.01
0.00	0.01	0.00	0.00	0.00	0.02	0.00
0.00	0.01	0.01	0.01	0.01	0.00	0.00
0.01	0.00	0.01	0.01	0.01	0.00	0.00
0.00	0.01	0.00	0.00	0.00	0.00	0.00
0.00	0.01	0.00	0.01	0.01	0.01	0.00
0.00	0.01	0.00	0.01	0.01	0.01	0.00
0.00	0.00	0.00	0.01	0.01	0.00	0.00
0.00	0.00	0.00	0.00	0.00	0.00	0.00
0.00	0.00	0.00	0.00	0.00	0.00	0.00
0.01	0.00	0.00	0.00	0.00	0.01	0.00
0.00	0.00	0.00	0.00	0.00	0.00	0.00
0.00	0.03	0.00	0.00	0.00	0.02	0.00
0.00	0.00	0.00	0.01	0.01	0.00	0.00
0.00	0.00	0.00	0.00	0.00	0.00	0.00
0.00	0.00	0.00	0.00	0.00	0.00	0.00
0.00	0.00	0.00	0.00	0.00	0.00	0.00
0.00	0.00	0.01	0.01	0.01	0.00	0.00
0.00	0.00	0.00	0.00	0.00	0.01	0.01
0.00	0.00	0.00	0.01	0.00	0.00	0.00
0.00	0.00	0.00	0.00	0.00	0.00	0.00
0.00	0.00	0.00	0.00	0.00	0.00	0.00
0.00	0.01	0.00	0.00	0.00	0.00	0.00
0.00	0.00	0.00	0.00	0.00	0.00	0.00
0.00	0.00	0.00	0.00	0.00	0.00	0.00
0.00	0.00	0.00	0.00	0.00	0.00	0.00
0.00	0.00	0.00	0.00	0.00	0.00	0.00
0.00	0.00	0.00	0.00	0.00	0.00	0.00
0.00	0.00	0.00	0.00	0.00	0.00	0.00
0.00	0.00	0.00	0.00	0.00	0.00	0.00
0.00	0.00	0.00	0.00	0.00	0.00	0.00
0.00	0.00	0.00	0.00	0.01	0.00	0.00
0.00	0.02	0.00	0.00	0.00	0.01	0.00
0.00	0.00	0.00	0.00	0.00	0.00	0.00
0.00	0.00	0.00	0.00	0.00	0.00	0.00
0.00	0.00	0.00	0.00	0.00	0.00	0.00
0.00	0.00	0.00	0.00	0.00	0.00	0.00
0.00	0.00	0.00	0.00	0.00	0.00	0.00
0.00	0.00	0.00	0.00	0.00	0.00	0.00
0.00	0.00	0.00	0.00	0.00	0.01	0.00
0.00	0.00	0.00	0.00	0.00	0.00	0.00
0.00	0.00	0.00	0.00	0.00	0.00	0.00
0.00	0.00	0.00	0.00	0.00	0.00	0.00
0.00	0.00	0.00	0.00	0.00	0.00	0.00
0.00	0.00	0.00	0.00	0.00	0.00	0.00
0.00	0.00	0.00	0.00	0.00	0.00	0.00
0.00	0.00	0.00	0.00	0.00	0.00	0.00
0.00	0.00	0.00	0.00	0.00	0.00	0.00
0.00	0.01	0.00	0.00	0.00	0.01	0.00
0.00	0.00	0.00	0.00	0.00	0.00	0.00
0.00	0.00	0.00	0.00	0.00	0.00	0.00
0.00	0.00	0.00	0.00	0.00	0.00	0.00
0.00	0.00	0.00	0.00	0.00	0.00	0.00
0.00	0.00	0.00	0.00	0.00	0.00	0.00
0.00	0.00	0.00	0.00	0.00	0.00	0.00
0.00	0.00	0.00	0.00	0.00	0.00	0.00
0.00	0.01	0.00	0.00	0.00	0.00	0.00
0.00	0.00	0.00	0.00	0.00	0.00	0.00

0.00	0.00	0.00	0.00	0.00	0.00	0.00
0.00	0.00	0.00	0.00	0.00	0.00	0.00
0.00	0.00	0.00	0.00	0.00	0.00	0.00
0.00	0.00	0.00	0.00	0.00	0.00	0.00
0.00	0.00	0.00	0.00	0.00	0.00	0.00
0.00	0.00	0.00	0.00	0.00	0.00	0.00
0.01	0.00	0.00	0.00	0.01	0.00	0.00
0.00	0.00	0.00	0.00	0.00	0.00	0.00
0.00	0.00	0.00	0.00	0.00	0.00	0.00
0.01	0.00	0.00	0.00	0.01	0.00	0.00
0.00	0.00	0.00	0.00	0.00	0.00	0.00
0.00	0.00	0.00	0.00	0.00	0.00	0.00
0.01	0.00	0.00	0.00	0.00	0.00	0.00
0.00	0.00	0.00	0.00	0.00	0.00	0.00
0.00	0.00	0.00	0.00	0.00	0.00	0.00
0.00	0.00	0.00	0.00	0.00	0.00	0.00
0.00	0.00	0.00	0.00	0.00	0.00	0.00
0.00	0.00	0.00	0.00	0.00	0.00	0.00
0.00	0.00	0.00	0.00	0.00	0.00	0.00
0.00	0.00	0.00	0.00	0.00	0.00	0.00
0.00	0.00	0.00	0.00	0.02	0.00	0.00
0.00	0.00	0.00	0.00	0.00	0.00	0.00
0.00	0.00	0.00	0.00	0.00	0.00	0.00
0.00	0.00	0.00	0.00	0.00	0.00	0.00
0.00	0.00	0.00	0.00	0.00	0.00	0.00
0.00	0.00	0.00	0.00	0.00	0.00	0.00
0.00	0.00	0.00	0.00	0.00	0.00	0.00
0.00	0.00	0.00	0.00	0.00	0.00	0.00
0.00	0.00	0.00	0.00	0.00	0.00	0.00
0.00	0.00	0.00	0.00	0.00	0.00	0.00
0.00	0.00	0.00	0.00	0.00	0.00	0.00
0.00	0.00	0.00	0.00	0.00	0.00	0.00
0.00	0.00	0.00	0.00	0.00	0.00	0.00
0.00	0.00	0.00	0.00	0.00	0.00	0.00
0.00	0.00	0.00	0.00	0.00	0.00	0.00
0.00	0.00	0.00	0.00	0.00	0.00	0.00
0.00	0.00	0.00	0.00	0.00	0.00	0.00
0.00	0.00	0.00	0.00	0.00	0.00	0.00
0.00	0.00	0.00	0.00	0.00	0.00	0.00
0.00	0.00	0.00	0.00	0.00	0.00	0.00
0.00	0.00	0.00	0.00	0.00	0.00	0.00
0.00	0.00	0.00	0.00	0.00	0.00	0.00
0.00	0.00	0.00	0.00	0.00	0.00	0.00
0.00	0.00	0.00	0.00	0.00	0.00	0.00
0.00	0.00	0.00	0.00	0.00	0.00	0.00
0.00	0.00	0.00	0.00	0.00	0.00	0.00
0.00	0.00	0.00	0.00	0.00	0.00	0.00
0.00	0.00	0.00	0.00	0.00	0.00	0.00
0.00	0.00	0.00	0.00	0.00	0.00	0.00
0.00	0.00	0.00	0.00	0.00	0.00	0.00
0.00	0.00	0.00	0.00	0.00	0.00	0.00
0.00	0.00	0.00	0.00	0.00	0.00	0.00
0.00	0.00	0.00	0.00	0.00	0.00	0.00
0.00	0.00	0.00	0.00	0.00	0.00	0.00
0.00	0.00	0.00	0.00	0.00	0.00	0.00
0.00	0.00	0.00	0.00	0.00	0.00	0.00
0.00	0.00	0.00	0.00	0.00	0.00	0.00
0.00	0.00	0.00	0.00	0.00	0.00	0.00
0.00	0.00	0.00	0.00	0.00	0.00	0.00

0.00	0.00	0.00	0.00	0.00	0.00	0.00
0.00	0.00	0.00	0.00	0.00	0.00	0.00
0.00	0.00	0.00	0.00	0.00	0.00	0.00
0.00	0.00	0.00	0.00	0.00	0.00	0.00
0.00	0.00	0.00	0.00	0.00	0.00	0.00
0.00	0.00	0.00	0.00	0.00	0.00	0.00
0.00	0.00	0.00	0.00	0.00	0.00	0.00
0.00	0.00	0.00	0.00	0.00	0.00	0.00
0.00	0.00	0.00	0.00	0.00	0.00	0.00
0.00	0.00	0.00	0.00	0.00	0.00	0.00
0.00	0.00	0.00	0.00	0.00	0.00	0.00
0.00	0.00	0.00	0.00	0.00	0.00	0.00
0.00	0.00	0.00	0.00	0.00	0.00	0.00
0.00	0.00	0.00	0.00	0.00	0.00	0.00
0.00	0.00	0.00	0.00	0.00	0.00	0.00
0.00	0.00	0.00	0.00	0.00	0.00	0.00
0.00	0.00	0.00	0.00	0.00	0.00	0.00
0.00	0.00	0.00	0.00	0.00	0.00	0.00
0.00	0.00	0.00	0.00	0.00	0.00	0.00
0.00	0.00	0.00	0.00	0.00	0.00	0.00
0.00	0.00	0.00	0.00	0.00	0.00	0.00
0.00	0.00	0.00	0.00	0.00	0.00	0.00
0.00	0.00	0.00	0.00	0.00	0.00	0.00
0.00	0.00	0.00	0.00	0.00	0.00	0.00
0.00	0.00	0.00	0.00	0.00	0.00	0.00
0.00	0.00	0.00	0.00	0.00	0.00	0.00
0.00	0.00	0.00	0.00	0.00	0.00	0.00
0.00	0.00	0.00	0.00	0.00	0.00	0.00
0.00	0.00	0.00	0.00	0.00	0.00	0.00
0.00	0.00	0.00	0.00	0.00	0.00	0.00

57	58	59	60	61	62	63
30	31	32	33	34	35	36
CHR_NP	CHR_NP	CHR_NP	CHR_NP	CHR_NP	CHR_NP	CHR_NP
F_39	G_3	G_30	G_31	G_32	G_4	G_5
1856730	2304707	924323	555209	358914	3926455	1257195
F_39	G_3	G_30	G_31	G_32	G_4	G_5
0.52	-1.52	-0.77	-1.80	-2.04	-1.24	0.73
-0.27	-0.61	-0.03	0.48	0.42	-0.54	-0.22
-0.15	-0.81	1.47	0.36	1.88	-1.08	-0.23
-0.27	-0.71	0.23	1.34	1.35	-0.76	0.26
-0.83	-0.57	2.54	1.40	1.09	-0.97	0.62
0.09	3.42	-0.75	0.22	0.54	4.07	-0.74
-0.11	1.57	0.56	2.77	3.06	1.56	-0.32
-0.50	6.18	-0.52	-0.19	-0.08	4.07	-0.45

0.43	2.70	-0.20	0.08	-0.48	2.45	-0.65
-0.26	0.10	-0.27	-0.41	-0.88	0.80	-0.70
0.94	0.26	-0.56	0.22	-0.76	0.09	-0.92
0.20	-1.13	0.73	2.52	1.27	-1.15	0.27
0.74	2.98	1.44	2.98	2.87	1.90	-0.78
-0.62	-1.21	0.12	-0.12	-0.54	-1.03	1.15
-0.20	2.56	-0.59	0.82	0.45	2.24	-0.81
-0.12	-0.98	-0.60	-0.93	-1.05	-1.00	-0.46
-0.23	3.28	-0.37	1.38	1.52	2.50	-0.65
-0.15	-0.66	-0.80	0.71	0.50	-0.58	-1.46
-0.32	3.12	0.25	1.17	1.04	3.62	-0.70
-0.90	-0.74	1.00	0.70	0.13	-0.78	-1.04
-0.66	-0.92	0.18	0.00	-0.57	-1.16	-0.95
-0.27	0.02	0.31	0.22	-0.67	0.60	-0.01
0.32	0.21	-0.07	-0.18	-0.77	0.77	-0.22
-0.70	0.91	0.76	1.94	2.97	0.25	-0.26
-0.49	1.14	0.02	0.10	0.77	0.53	-0.61
0.62	-0.52	-0.79	-1.37	-1.43	-0.95	-0.48
0.07	-0.83	1.87	1.12	0.80	-0.97	0.87
-0.38	1.33	-0.78	-0.33	-0.08	2.29	-0.40
0.90	-0.48	-0.75	0.58	0.86	0.17	-0.99
1.59	-0.83	0.97	0.65	-0.59	-0.76	0.30
0.20	0.51	0.33	3.47	2.90	0.17	-0.54
-0.72	-0.21	0.87	0.79	0.83	-0.46	0.84
-0.28	4.64	-0.43	0.64	0.46	4.03	-0.32
-0.30	1.96	-0.32	0.00	-0.12	0.79	-0.36
1.06	-0.49	-0.67	0.77	1.02	0.25	-0.96
-0.31	-0.56	-0.19	0.11	0.83	-0.36	0.21
-0.48	-0.48	-0.52	0.45	0.30	-0.19	-0.98
-0.22	0.00	-0.36	-0.08	0.97	-0.38	-0.68
-0.75	-0.93	0.37	0.28	-0.17	-0.71	0.06
-0.63	-0.35	0.40	-0.33	-0.45	-0.30	0.62
-0.89	-1.33	0.37	0.33	0.60	-1.31	-0.58
-0.81	0.05	1.69	1.93	1.39	-1.11	-0.18
-0.20	-1.47	-0.03	0.38	0.84	-1.29	0.36
-0.67	3.52	-0.79	-0.09	-0.05	5.44	-0.78
-0.76	-0.58	0.01	-0.23	0.44	-0.88	-0.74
0.81	-1.18	1.34	3.04	0.71	-1.74	-1.13
0.66	0.78	-0.35	0.99	0.07	0.38	-0.11
-0.27	-0.99	0.69	0.02	-0.80	-0.74	0.33
-0.15	7.33	-0.30	-0.01	-0.11	5.92	-0.09
-0.51	0.77	-0.53	-0.44	-0.58	0.96	-0.55
4.53	-1.76	0.82	-1.17	-0.98	-1.71	-1.35
-0.41	-0.33	-0.19	0.86	1.11	-0.52	-0.72
0.02	-1.10	0.24	1.20	0.66	-1.50	0.44
-0.61	-0.72	-0.64	-0.56	-1.07	-0.60	-1.10
-0.39	1.09	-1.19	-1.32	-1.36	0.74	-0.17

-0.25	8.65	-0.33	-0.25	-0.31	5.85	-0.27
-0.17	0.07	0.25	1.94	2.73	0.27	0.01
-1.17	0.93	0.55	1.93	0.98	-0.32	-0.55
0.04	0.09	1.22	2.41	1.71	-1.37	-0.63
0.19	-0.78	0.28	0.93	1.15	-0.85	0.08
-0.15	-0.98	0.08	2.72	2.01	-1.28	-0.74
-1.04	0.42	-0.17	0.15	-0.50	-0.47	-0.43
-0.24	11.46	-0.61	-0.29	-0.36	8.89	-0.51
-0.24	4.81	-0.25	-0.19	-0.06	3.55	-0.24
0.53	-1.25	-0.68	-0.43	-0.93	-0.96	0.32
-0.41	2.52	-0.15	0.98	1.64	3.12	-0.75
-0.07	3.23	-0.36	0.66	0.37	1.93	-0.48
-0.43	7.69	-0.52	0.44	0.99	5.62	-0.51
-0.60	0.66	-0.36	0.29	1.73	-0.75	-0.96
-0.10	-0.27	-0.37	-0.35	-0.34	-0.21	-0.19
-0.38	-1.23	-0.33	0.48	0.37	-1.31	-0.53
-0.02	-1.58	-0.58	-1.25	-1.28	-1.61	-0.92
-0.41	4.07	-0.61	0.31	0.12	3.39	-0.77
0.17	0.44	-0.66	-0.97	-1.25	0.92	-0.16
0.03	2.18	0.74	3.60	5.59	0.90	-0.39
4.04	-1.23	1.27	-0.71	2.18	-0.59	-0.98
-0.34	-0.28	-0.83	-1.50	-1.52	0.07	0.87
0.32	-0.27	-0.21	-0.22	-0.03	-0.29	0.28
-0.67	-0.92	0.46	0.74	0.38	-1.10	-0.20
-0.35	-0.92	-0.58	0.53	0.52	-1.15	-1.03
0.15	0.44	-0.96	0.37	0.46	1.17	-0.59
-0.44	-1.08	-0.11	-1.29	-1.49	-1.23	-0.30
-0.60	-1.31	-0.39	0.04	-0.32	-0.91	-0.06
-0.30	0.27	-0.43	0.04	-0.35	-0.03	-0.45
-0.35	2.32	-0.62	1.06	0.43	1.39	-0.05
-0.70	-0.62	-0.17	-0.71	-0.73	-1.04	-0.39
-0.04	-1.92	0.25	0.39	0.17	-1.83	-0.46
-0.24	5.73	-0.47	0.88	1.32	3.73	-0.62
0.34	-0.14	0.66	2.90	2.68	-0.29	-1.03
0.17	-0.70	1.06	0.70	0.52	-1.04	-0.42
0.06	-0.48	0.28	-0.07	-0.46	-0.70	0.22
-0.24	8.69	-0.28	-0.24	-0.19	8.22	-0.06
-0.90	-0.36	0.56	-0.68	-0.85	-0.97	-0.41
0.50	1.25	-0.29	0.47	0.47	0.78	-0.62
-0.22	2.75	-0.62	-0.29	-0.19	3.23	-0.52
-0.38	0.51	-0.41	1.07	1.65	-0.05	-0.97
-0.26	-1.38	0.58	1.25	0.18	-1.48	-0.75
-0.39	-1.70	0.32	0.94	-0.87	-1.50	0.17
-0.43	-1.05	3.02	1.40	1.84	-1.26	-0.24
-0.11	5.38	-0.60	0.34	0.19	6.59	-0.64
-0.59	-0.62	-0.21	-0.30	0.07	-0.65	-0.34
-0.19	0.44	1.03	2.11	0.31	0.84	0.09
-0.31	-0.59	1.55	1.19	1.42	-1.03	-0.46

-0.28	0.72	-0.23	0.01	0.61	0.09	-0.39
0.17	1.99	-1.09	0.51	-0.33	2.90	-1.18
-0.52	5.80	-0.52	0.00	0.01	4.37	-0.47
-0.28	-0.69	-0.15	0.84	0.61	-1.24	-0.82
-0.22	-0.44	0.17	0.85	1.61	-0.49	-0.44
-0.07	4.61	-0.76	0.93	1.08	3.12	-0.66
-0.45	8.04	0.14	0.90	1.51	6.51	-0.52
-0.75	1.39	0.68	1.48	0.96	0.22	-1.20
-0.34	-0.72	1.59	0.34	0.81	-1.36	-0.68
-0.72	-0.81	1.63	1.23	1.52	-0.70	0.89
-0.41	3.54	-0.55	0.24	0.28	6.13	-0.58
-0.03	0.81	2.36	1.36	1.12	-0.05	-0.47
0.43	-1.15	1.64	1.58	0.48	-1.27	-1.04
-0.66	4.39	-0.74	-0.13	-0.97	-0.13	-1.21
0.58	1.95	-0.39	2.51	2.77	0.92	-0.93
0.58	1.95	-0.39	2.51	2.77	0.92	-0.93
-0.74	-0.28	-0.22	0.57	1.88	-0.91	-0.64
0.14	-0.48	-0.37	-0.48	-0.95	-0.75	0.27
0.08	-1.54	-0.77	-1.22	-1.15	-1.83	-0.97
1.44	-0.43	0.13	-0.75	-0.32	1.32	-0.81
-0.16	-0.56	-0.42	0.13	-0.29	-0.18	-0.58
-0.25	11.42	-0.30	-0.15	-0.19	5.97	-0.23
-0.12	-1.02	-0.35	1.20	0.50	-1.07	-0.71
0.11	-0.37	0.49	0.33	0.96	-0.66	0.10
0.30	-0.75	-0.12	-0.44	-0.94	-1.01	-0.88
-0.67	-1.00	-0.38	-0.54	-0.73	-1.04	-0.61
-0.23	-0.47	1.06	1.00	0.61	-0.69	-0.40
0.58	0.92	-0.55	-0.71	-0.76	1.92	5.07
-0.08	-0.20	0.52	2.99	1.83	-0.63	-0.15
0.42	0.66	0.28	1.59	0.37	0.48	-0.67
-0.35	-0.92	-0.48	-0.35	-0.12	-0.84	-0.38
-0.13	3.36	-0.40	-0.21	-0.54	1.84	-0.34
-0.11	0.22	0.17	0.76	0.10	-0.88	-0.99

20	21	22	23	24	25	26
56	57	58	59	60	61	62
CTTTCAGTCGGAT	CAACACCAGTCGA	TACAGTACTGT	AATGACACGAT	AACATTCAACCT	TTAATGCTAATC	TCCCTGAGACC
miR-30e-3p	miR-21-3p	miR-101-3p	miR-425-5p	miR-181c-5p	miR-155-5p	miR-125a-5p
138740	137573	134502	127210	125038	122415	115160
-0.84	0.08	-0.13	-0.26	-0.50	1.04	-0.59
-0.78	-1.38	-0.38	0.03	-1.47	0.07	2.00
0.98	-0.98	-0.16	1.92	-0.55	-0.49	-0.45
-0.11	-0.29	-0.22	-0.63	-0.98	-0.60	-0.58
-0.71	-1.36	-0.32	2.63	0.26	2.92	2.75
-0.26	0.30	-0.29	-0.62	-0.88	1.53	-0.48
-0.23	0.40	-0.22	-1.13	-0.27	0.89	-0.33
-0.11	-1.03	-0.32	-0.89	0.06	-1.16	-0.47
-0.63	-0.83	-0.34	0.01	-1.04	-1.52	-0.55
-0.81	-0.82	-0.23	-0.20	-1.47	-0.63	-0.75
1.28	0.16	-0.21	0.29	0.04	-0.39	0.22
0.01	-0.39	-0.33	-1.51	0.05	0.37	0.34
1.70	0.73	0.81	0.92	2.83	0.30	-0.47
-0.45	-0.89	-0.34	-1.00	-0.52	-0.73	-0.28
0.33	0.25	-0.24	1.11	-0.10	-0.20	-0.34
0.87	0.74	-0.03	1.17	1.24	0.31	-0.55
-0.73	2.00	-0.32	0.70	0.00	-0.36	-0.09
-0.44	-0.26	0.04	0.16	0.46	-0.07	-0.66
3.16	0.37	-0.23	-0.43	0.62	0.33	-0.70
1.62	1.61	4.85	0.05	1.60	-0.63	-0.73

-0.02	-0.83	-0.19	-1.06	-1.20	0.53	-0.59
-0.26	0.55	-0.32	-1.06	1.50	-0.54	0.63
-0.44	0.90	-0.25	-0.72	0.66	-0.13	0.11
-1.03	0.46	-0.25	-1.10	-0.36	-0.04	-0.42
-1.02	-1.37	-0.34	0.54	-0.69	-1.33	2.90
-0.81	-0.46	-0.32	0.82	0.64	1.75	0.52
-0.28	2.35	0.27	0.23	0.09	-1.24	-0.43
1.10	0.80	6.29	0.53	-0.39	-1.08	-0.82
5.47	1.52	-0.05	-0.31	-0.81	-0.07	-0.10
2.51	-1.07	-0.23	-1.03	-1.31	-0.17	-0.31
-0.58	-1.37	-0.33	-0.97	-1.85	-1.06	0.66
3.78	-1.37	-0.07	-0.42	-1.36	-0.69	-0.23
-0.43	-1.34	-0.33	4.83	0.51	-0.93	10.28
0.68	1.20	-0.30	0.16	3.03	-0.74	-0.91
-0.35	-0.49	-0.26	1.49	0.77	-0.75	-0.87
0.15	-0.04	-0.29	-0.51	0.44	1.14	-0.64
-1.32	-1.18	-0.36	-1.51	-1.63	-1.83	-0.76
1.26	0.41	0.13	-0.80	0.01	0.87	-0.83
0.81	-0.19	1.06	1.79	1.78	1.45	-0.78
0.56	-0.31	-0.31	-0.28	1.51	0.70	-0.11
-1.32	3.05	-0.23	0.22	-1.56	-1.51	-0.27
-1.18	5.66	-0.17	3.05	-1.01	-1.30	0.65
-0.63	0.15	-0.31	0.14	-0.49	-0.60	0.22
1.23	1.96	0.25	1.17	0.39	0.06	-0.25
0.95	-0.83	-0.35	-0.77	-0.49	-0.16	-0.08
-1.41	2.24	-0.31	-0.98	-1.50	-1.45	-0.47
1.22	1.52	-0.21	1.83	3.44	-0.04	-0.11
0.38	1.39	-0.26	-0.49	2.39	-0.34	-0.62
-0.62	-0.50	-0.16	0.75	0.87	-1.19	-0.18
-0.45	1.24	-0.26	0.57	2.72	0.94	-0.55
-0.57	-0.07	-0.14	0.13	0.56	0.05	-0.51
-0.40	0.61	-0.30	-0.04	-0.70	0.79	0.23
-0.87	-0.17	-0.30	-0.40	-1.22	-1.17	-0.23
-0.12	-0.09	-0.24	1.67	0.24	1.04	0.10
0.10	-0.39	-0.30	-0.86	-0.80	-0.22	-0.59
-0.37	0.16	-0.32	0.15	-0.76	-0.44	0.94
-0.61	-0.39	-0.25	-0.17	-1.17	0.04	0.19
-0.72	1.09	8.65	0.07	0.93	0.09	-0.78
-0.64	-1.19	-0.33	0.25	0.55	1.22	0.28
-0.56	-1.32	-0.25	1.94	1.93	2.41	0.93
-1.07	-1.36	-0.31	2.73	0.98	1.71	1.15
-0.60	0.74	5.85	0.27	-0.32	-1.37	-0.85
-1.10	-0.17	-0.27	0.01	-0.55	-0.63	0.08
-1.16	1.03	-0.27	-0.09	-1.02	-0.43	-0.08
-0.93	1.13	-0.24	0.87	-0.54	-0.98	-0.75
5.98	-0.44	0.58	-1.72	-0.60	-0.21	-0.53
-0.63	-0.44	0.08	0.94	-1.08	-0.92	-0.90
1.81	-1.38	-0.23	0.10	-1.28	1.08	1.04
-0.39	-1.36	-0.31	0.88	-0.73	0.26	-0.07
2.28	-1.30	-0.20	1.67	1.85	1.59	0.14
0.65	-0.49	-0.19	-1.09	-0.67	-0.31	-0.21
5.13	-1.00	0.85	0.47	-0.96	-1.09	-0.82
-0.17	-0.77	-0.24	-0.34	-0.67	-0.63	-0.73
0.11	-0.90	-0.01	0.28	0.92	0.63	-0.49
-1.28	-0.43	-0.31	-0.39	-1.84	-1.36	-0.23
0.05	-0.25	0.02	-0.40	0.47	0.56	-0.45
2.62	-0.60	-0.26	-0.34	-0.13	0.93	-0.60
3.69	-0.99	-0.27	-1.32	-0.71	0.30	-0.48
-0.11	2.80	0.17	1.33	2.33	0.19	-0.82

0.80	1.46	-0.28	1.98	2.48	-0.16	-0.33
-0.82	-0.38	-0.31	1.68	1.12	-1.42	0.26
0.08	-0.89	-0.24	0.21	0.81	0.91	-0.18
-1.03	0.01	-0.22	-0.99	1.16	-0.04	0.13
0.12	1.27	7.00	-0.29	2.75	0.21	-0.69
-0.27	2.29	9.36	-0.40	0.03	-1.29	-0.97
-0.05	0.03	-0.28	0.59	1.59	-0.94	0.46
0.26	1.07	-0.25	0.09	2.45	0.30	-0.61
-0.43	-0.17	-0.26	0.21	-0.01	0.25	-0.35
2.31	0.79	-0.08	-0.41	0.77	0.16	-0.91
0.20	-0.66	-0.30	0.42	0.39	0.31	0.16
1.71	0.03	0.32	-1.21	2.24	-0.19	-0.63
0.67	-0.46	-0.28	-1.52	-0.87	0.38	-0.53
-0.19	1.95	-0.28	-0.48	1.01	-0.30	-0.11
-0.01	0.30	-0.26	-0.23	-0.12	-0.89	-0.24
-1.20	0.51	-0.04	-0.39	-0.95	0.16	-0.77
0	1	-2	1	1	1	1
0.00	0.00	0.00	0.00	0.00	0.00	0.00
1.00	1.00	1.00	1.00	1.00	1.00	1.00
0.07	0.27	0.86	0.15	0.30	0.59	0.18

54	55	56	57	58	59	60
CTTTCAGTCGGAT	CAACACCAGTCGA	TACAGTACTGTG	AATGACACGATC	AACATTCAACCTG	TTAATGCTAATCG	TCCCTGAGACCC
miR-30e-3p	miR-21-3p	miR-101-3p	miR-425-5p	miR-181c-5p	miR-155-5p	miR-125a-5p
138740	137573	134502	127210	125038	122415	115160
-0.84	0.08	-0.13	-0.26	-0.50	1.04	-0.59
-0.78	-1.38	-0.38	0.03	-1.47	0.07	2.00
0.98	-0.98	-0.16	1.92	-0.55	-0.49	-0.45
-0.11	-0.29	-0.22	-0.63	-0.98	-0.60	-0.58
-0.71	-1.36	-0.32	2.63	0.26	2.92	2.75
-0.26	0.30	-0.29	-0.62	-0.88	1.53	-0.48
-0.23	0.40	-0.22	-1.13	-0.27	0.89	-0.33
-0.11	-1.03	-0.32	-0.89	0.06	-1.16	-0.47
-0.63	-0.83	-0.34	0.01	-1.04	-1.52	-0.55
-0.81	-0.82	-0.23	-0.20	-1.47	-0.63	-0.75
1.28	0.16	-0.21	0.29	0.04	-0.39	0.22
0.01	-0.39	-0.33	-1.51	0.05	0.37	0.34
1.70	0.73	0.81	0.92	2.83	0.30	-0.47
-0.45	-0.89	-0.34	-1.00	-0.52	-0.73	-0.28
0.33	0.25	-0.24	1.11	-0.10	-0.20	-0.34
0.87	0.74	-0.03	1.17	1.24	0.31	-0.55
-0.73	2.00	-0.32	0.70	0.00	-0.36	-0.09
-0.44	-0.26	0.04	0.16	0.46	-0.07	-0.66
3.16	0.37	-0.23	-0.43	0.62	0.33	-0.70
1.62	1.61	4.85	0.05	1.60	-0.63	-0.73
-0.02	-0.83	-0.19	-1.06	-1.20	0.53	-0.59
-0.26	0.55	-0.32	-1.06	1.50	-0.54	0.63
-0.44	0.90	-0.25	-0.72	0.66	-0.13	0.11
-1.03	0.46	-0.25	-1.10	-0.36	-0.04	-0.42
-1.02	-1.37	-0.34	0.54	-0.69	-1.33	2.90
-0.81	-0.46	-0.32	0.82	0.64	1.75	0.52
-0.28	2.35	0.27	0.23	0.09	-1.24	-0.43
1.10	0.80	6.29	0.53	-0.39	-1.08	-0.82

5.47	1.52	-0.05	-0.31	-0.81	-0.07	-0.10
2.51	-1.07	-0.23	-1.03	-1.31	-0.17	-0.31
-0.58	-1.37	-0.33	-0.97	-1.85	-1.06	0.66
3.78	-1.37	-0.07	-0.42	-1.36	-0.69	-0.23
-0.43	-1.34	-0.33	4.83	0.51	-0.93	10.28
0.68	1.20	-0.30	0.16	3.03	-0.74	-0.91
-0.35	-0.49	-0.26	1.49	0.77	-0.75	-0.87
0.15	-0.04	-0.29	-0.51	0.44	1.14	-0.64
-1.32	-1.18	-0.36	-1.51	-1.63	-1.83	-0.76
1.26	0.41	0.13	-0.80	0.01	0.87	-0.83
0.81	-0.19	1.06	1.79	1.78	1.45	-0.78
0.56	-0.31	-0.31	-0.28	1.51	0.70	-0.11
-1.32	3.05	-0.23	0.22	-1.56	-1.51	-0.27
-1.18	5.66	-0.17	3.05	-1.01	-1.30	0.65
-0.63	0.15	-0.31	0.14	-0.49	-0.60	0.22
1.23	1.96	0.25	1.17	0.39	0.06	-0.25
0.95	-0.83	-0.35	-0.77	-0.49	-0.16	-0.08
-1.41	2.24	-0.31	-0.98	-1.50	-1.45	-0.47
1.22	1.52	-0.21	1.83	3.44	-0.04	-0.11
0.38	1.39	-0.26	-0.49	2.39	-0.34	-0.62
-0.62	-0.50	-0.16	0.75	0.87	-1.19	-0.18
-0.45	1.24	-0.26	0.57	2.72	0.94	-0.55
-0.57	-0.07	-0.14	0.13	0.56	0.05	-0.51
-0.40	0.61	-0.30	-0.04	-0.70	0.79	0.23
-0.87	-0.17	-0.30	-0.40	-1.22	-1.17	-0.23
-0.12	-0.09	-0.24	1.67	0.24	1.04	0.10
0.10	-0.39	-0.30	-0.86	-0.80	-0.22	-0.59
-0.37	0.16	-0.32	0.15	-0.76	-0.44	0.94
-0.61	-0.39	-0.25	-0.17	-1.17	0.04	0.19
-0.72	1.09	8.65	0.07	0.93	0.09	-0.78
-0.64	-1.19	-0.33	0.25	0.55	1.22	0.28
-0.56	-1.32	-0.25	1.94	1.93	2.41	0.93
-1.07	-1.36	-0.31	2.73	0.98	1.71	1.15
-0.60	0.74	5.85	0.27	-0.32	-1.37	-0.85
-1.10	-0.17	-0.27	0.01	-0.55	-0.63	0.08
-1.16	1.03	-0.27	-0.09	-1.02	-0.43	-0.08
-0.93	1.13	-0.24	0.87	-0.54	-0.98	-0.75
5.98	-0.44	0.58	-1.72	-0.60	-0.21	-0.53
-0.63	-0.44	0.08	0.94	-1.08	-0.92	-0.90
1.81	-1.38	-0.23	0.10	-1.28	1.08	1.04
-0.39	-1.36	-0.31	0.88	-0.73	0.26	-0.07
2.28	-1.30	-0.20	1.67	1.85	1.59	0.14
0.65	-0.49	-0.19	-1.09	-0.67	-0.31	-0.21
5.13	-1.00	0.85	0.47	-0.96	-1.09	-0.82
-0.17	-0.77	-0.24	-0.34	-0.67	-0.63	-0.73
0.11	-0.90	-0.01	0.28	0.92	0.63	-0.49
-1.28	-0.43	-0.31	-0.39	-1.84	-1.36	-0.23
0.05	-0.25	0.02	-0.40	0.47	0.56	-0.45
2.62	-0.60	-0.26	-0.34	-0.13	0.93	-0.60
3.69	-0.99	-0.27	-1.32	-0.71	0.30	-0.48
-0.11	2.80	0.17	1.33	2.33	0.19	-0.82
0.80	1.46	-0.28	1.98	2.48	-0.16	-0.33
-0.82	-0.38	-0.31	1.68	1.12	-1.42	0.26
0.08	-0.89	-0.24	0.21	0.81	0.91	-0.18
-1.03	0.01	-0.22	-0.99	1.16	-0.04	0.13
0.12	1.27	7.00	-0.29	2.75	0.21	-0.69
-0.27	2.29	9.36	-0.40	0.03	-1.29	-0.97
-0.05	0.03	-0.28	0.59	1.59	-0.94	0.46
0.26	1.07	-0.25	0.09	2.45	0.30	-0.61

-0.43	-0.17	-0.26	0.21	-0.01	0.25	-0.35
2.31	0.79	-0.08	-0.41	0.77	0.16	-0.91
0.20	-0.66	-0.30	0.42	0.39	0.31	0.16
1.71	0.03	0.32	-1.21	2.24	-0.19	-0.63
0.67	-0.46	-0.28	-1.52	-0.87	0.38	-0.53
-0.19	1.95	-0.28	-0.48	1.01	-0.30	-0.11
-0.01	0.30	-0.26	-0.23	-0.12	-0.89	-0.24
-1	1	0	0	-1	0	-1

54	55	56	57	58	59	60
63	42	129	43	47	106	117
CATTATTACTTTT	AACATTCATTGCT	ACTCCAGCCCCA	TCAGGCTCAGTC	TTCACAGTGGCTA	TACCCATTGCATA	TGTAAACATCCT
miR-126-5p	miR-181b-5p	miR-766-3p	miR-484	miR-27b-3p	miR-660-5p	miR-30a-5p
96743	270366	12195.00	261666	209441	23949	16585
-0.60	-0.55	-0.44	0.57	-0.04	-0.51	0.81
-0.11	-1.07	0.51	1.61	-1.33	-0.07	-2.04
-0.29	-1.03	-0.56	0.17	-0.14	0.33	-0.73
-0.52	-1.07	-0.77	-0.40	-0.64	0.13	-1.10
-0.31	0.47	1.23	3.70	-0.53	-0.56	-1.73
-0.50	0.32	-1.00	-0.57	0.59	-0.70	-0.61
-0.60	0.88	-0.48	-0.28	-0.01	-0.55	0.33
-0.56	0.03	0.13	-0.23	-1.03	-0.65	-0.27
-0.40	-1.58	-0.24	-0.29	-0.88	-0.56	-0.07
0.18	-1.31	-0.47	-0.04	-0.82	-0.50	-1.41
1.28	-0.92	0.12	0.18	1.17	-0.19	0.21
-0.60	2.09	-1.03	-1.14	-1.04	-0.64	-0.32
1.97	-0.13	-0.56	-0.87	0.87	2.94	1.06
-0.59	0.77	0.98	0.19	-0.98	-0.77	1.66
-0.28	-0.70	-0.19	-0.05	-0.30	0.19	0.53
1.18	0.40	-0.13	-0.80	3.48	0.72	0.27
-0.62	1.79	0.31	0.64	0.32	-0.53	1.58
-0.25	-0.59	-0.31	-0.58	-0.39	2.16	0.35
-0.25	0.68	0.04	-1.06	0.07	0.36	0.95
2.98	-0.57	-0.39	-0.23	0.04	1.97	1.77
-0.48	0.22	-0.70	-0.77	-0.21	-0.61	-0.79
-0.68	0.75	0.33	-0.23	0.26	-0.79	0.19
-0.64	0.69	0.37	-0.32	-0.05	-0.51	0.65
-0.67	1.22	0.36	0.12	-0.47	-0.73	-0.93
-0.39	-0.65	4.15	1.60	-0.49	-0.67	-0.83
-0.49	1.18	-0.58	-0.13	1.62	-0.57	0.02
2.24	-1.33	-0.70	-0.78	0.93	1.32	0.47
19.45	-1.59	-0.69	-1.00	1.01	5.07	5.68
0.31	-1.15	0.10	-0.40	0.88	0.23	-0.22
-0.05	-1.50	0.29	-0.48	-0.33	0.16	-0.89
-0.50	-1.58	2.23	2.88	-0.97	-0.39	-0.17
2.09	-1.12	2.03	-0.38	0.89	2.14	2.08
0.71	-1.61	1.16	3.19	-0.37	-0.03	-1.44
-0.59	-0.80	-0.13	0.82	-0.71	-0.37	-0.52
-0.38	-0.59	0.09	0.58	-0.96	-0.12	-0.26
-0.50	0.68	-0.02	-0.07	-0.31	-0.66	-0.37
-0.65	-1.28	-1.01	-1.19	-1.36	-0.93	-1.11
-0.22	0.01	-0.92	-1.73	-0.49	-0.16	0.33
2.40	-0.08	-0.34	-1.21	1.20	4.48	3.52

-0.54	1.90	-0.46	-0.78	-0.38	-0.50	1.24
-0.70	2.31	3.48	3.64	0.63	-0.63	-1.11
-0.51	3.41	14.49	2.87	7.95	-0.24	0.29
-0.32	0.12	0.41	0.24	0.87	-0.75	0.15
0.13	-0.18	-0.39	-0.33	0.98	0.45	2.85
-0.52	0.44	0.52	-1.21	-0.88	-0.54	-1.20
-0.61	1.67	1.69	2.06	-0.30	-0.90	-1.25
-0.19	-0.16	0.12	0.89	1.61	-0.31	0.94
-0.63	0.50	-0.18	0.03	0.33	-0.59	0.20
-0.20	-0.56	-0.38	0.52	-0.30	-0.29	-0.05
-0.62	1.05	-0.40	0.45	-0.21	-0.50	-0.17
-0.07	0.83	-0.08	-0.24	1.15	-0.18	0.93
-0.54	-0.56	1.62	0.83	2.06	-0.73	-1.08
-0.54	-1.15	0.85	0.84	0.84	-0.72	-0.87
-0.54	-0.45	-0.05	0.70	1.46	-0.50	-0.47
-0.60	0.08	-0.60	-0.62	0.23	-0.72	-0.85
-0.52	-0.46	0.04	1.00	0.29	-0.70	-1.08
-0.24	-0.81	-0.67	-0.20	0.66	-0.52	-0.66
11.46	0.05	-1.00	-1.47	0.78	5.80	4.39
-0.61	1.69	-0.38	-0.03	-0.35	-0.52	-0.74
-0.29	1.93	-0.54	0.38	0.99	0.00	-0.13
-0.36	1.39	-0.73	0.84	0.07	0.01	-0.97
8.89	-1.11	-1.04	-1.29	0.38	4.37	-0.13
-0.51	-0.18	-0.61	0.36	-0.11	-0.47	-1.21
-0.52	-0.23	-0.92	-0.49	0.84	-0.52	-0.82
-0.07	-0.73	-0.98	-0.17	0.13	0.22	4.46
-0.57	2.04	-0.65	-0.99	-1.11	4.09	4.44
2.04	-1.60	-0.65	-0.77	0.16	2.19	-0.61
0.13	-1.70	0.99	0.51	-0.86	0.08	0.30
-0.42	0.20	1.32	3.77	-0.21	0.48	0.11
-0.17	0.98	0.81	2.80	0.68	0.67	1.46
-0.51	-0.83	-0.27	-0.01	-0.30	-0.19	-0.71
-0.07	-0.94	-1.10	-0.67	-1.05	-0.59	-0.12
-0.15	-0.99	-0.80	-0.85	-0.61	-0.13	-1.13
0.17	0.23	-0.57	-0.73	-0.71	0.92	-0.20
-0.60	3.15	17.98	7.94	-0.38	-0.82	-1.56
-0.02	0.54	-0.82	-1.14	0.14	0.49	1.81
-0.23	-0.52	0.05	-1.35	-0.43	0.14	0.29
0.18	0.94	-0.89	-1.76	-0.28	-0.40	-1.39
0.75	-0.28	0.30	1.72	1.39	1.57	0.79
0.03	-0.65	0.21	0.68	2.31	-0.09	1.89
-0.42	-0.85	1.56	2.17	0.70	-0.66	2.55
-0.32	-0.32	-0.03	0.81	0.94	-0.36	-0.70
-0.64	0.76	-0.54	0.28	-0.19	-0.73	0.51
4.01	0.11	-0.71	-0.14	0.45	3.40	0.62
6.09	-1.13	-0.90	-1.27	-0.13	4.13	-0.14
-0.51	-0.32	0.16	1.37	0.38	-0.32	1.18
-0.48	0.13	-0.23	0.08	0.80	-0.38	0.13
-0.50	-0.35	-0.50	0.35	0.25	-0.54	-0.27
-0.18	-0.05	-0.55	-1.26	0.29	1.17	-0.47
-0.48	-0.26	0.30	0.86	0.74	-0.52	1.35
-0.21	0.02	-0.68	-1.51	-0.02	1.63	5.62
-0.58	-0.10	-0.31	-1.07	-0.32	-0.53	-0.82
-0.63	1.75	0.32	-0.18	0.49	-0.63	-0.77
-0.09	-0.88	-0.28	-1.18	1.91	-0.15	4.11
0.29	-0.12	-0.61	-0.62	3.44	-0.23	-0.74

0.21	0.21	0.22	0.24	0.24	0.25	0.26
0.22	0.15	1.00	0.17	0.15	0.50	0.33

555209	358914	3926455	1257195	488065	1421920	492624

64	65	66	67	68	69	70
34	35	36	37	38	39	1
G_31	G_32	G_4	G_5	G_6	G_7	A_10
CHR_NP	CHR_NP	CHR_NP	CHR_NP	CHR_NP	CHR_NP	CHR_P
1166964	863736	11268615	6409791	1457364	6651247	3902181
0.71	0.28	0.55	0.76	0.94	0.73	0.12
G_31	G_32	G_4	G_5	G_6	G_7	A_10
611755	504822	7342160	5152596	969299	5229327	3409557
60054	27156	715360	584807	196834	399578	190797
81885	50732	166580	98444	33091	131009	7668
43191	49624	78461	70682	30921	36819	10054
63556	41067	197923	106696	48784	152679	37879
57366	33951	151060	106970	30262	81663	47947
24074	19272	669358	14568	10574	100931	5499
48602	33276	252380	37877	29185	86225	4865
3955	3195	304888	3580	1738	15269	4480
10556	3738	214919	10351	8204	87617	5810
10050	4479	125897	19095	7982	44347	28115
8857	2892	58207	8865	9832	27797	31440
12921	6828	42738	20501	6971	19045	8308
14640	9224	80893	8750	7614	28174	9824
7451	4023	33867	26210	7585	22198	7741
6652	3420	83123	1659	1801	11236	11411
1400	588	7967	7460	3840	4218	5821
6284	4297	65216	2153	932	9345	1238
4796	2853	18322	2596	1264	4671	5981
5219	3205	69159	4040	2901	11037	1600
5029	2619	17863	4956	2305	6613	3207
2626	1310	9992	3862	1506	4148	2184
2776	1160	22460	5937	2124	7540	5042
1742	613	21293	3969	1093	4874	1341
5429	4673	17203	3609	655	4418	858
2457	2791	25787	1081	999	10956	1605
704	418	7579	3527	997	4583	1511
3489	2027	9094	7577	1962	5290	5431
1307	1126	41338	2796	1329	1938	6034
2292	1628	13657	2219	1244	5804	1572
2173	827	8205	4514	1864	4373	1013
4003	2313	11523	2645	1624	4144	838
2719	1774	11574	6496	2848	6975	2236
1704	969	38405	1508	789	3611	544
1210	558	25718	253	342	591	715
2158	1509	12423	2036	1134	5192	1112
2703	3005	9914	6992	1422	4545	796
2524	1494	11445	1205	686	4225	1073
1408	2015	6517	1049	942	4989	1075
2049	920	4721	4100	1021	1788	178
974	466	7388	6950	2366	3556	2757
1538	1136	1409	1880	348	817	563
2218	1235	3723	2467	928	1980	2074
1232	915	3808	2879	794	2672	607
563	382	30204	243	210	1372	613
868	896	2494	1098	514	1473	974

1477	605	2644	1214	731	1358	637
1093	458	5914	1492	937	1954	201
842	276	3222	2347	687	1241	1701
187	88	21157	350	187	702	277
184	51	8480	243	124	447	1549
228	186	444	406	477	270	91
1067	777	1933	385	203	826	167
856	476	1923	1624	394	1111	255
310	95	2091	325	107	509	2169
18	4	4507	859	663	2020	258
55	19	17962	108	42	155	358
872	667	3703	1109	409	1731	103
791	386	2376	683	179	781	256
1052	576	1430	869	378	776	421
1276	911	1024	1720	576	556	317
977	534	1021	605	295	481	231
399	172	1929	663	176	567	569
94	50	14472	107	40	372	31
39	55	9973	52	14	80	5
368	143	1498	1372	498	875	1075
447	361	5759	343	222	1097	173
352	185	4518	217	167	346	118
249	220	7899	207	107	752	287
255	301	731	172	70	433	155
98	77	2021	730	208	634	130
474	288	343	551	275	313	86
130	80	483	436	156	327	426
223	122	5449	73	98	531	270
108	47	2458	498	175	827	407
669	604	2152	311	196	495	60
143	469	1176	209	225	116	206
36	21	1775	853	373	1134	187
213	217	1197	1260	417	650	41
428	234	653	605	252	356	432
341	219	299	147	80	141	23
216	144	1996	315	122	698	120
55	20	436	401	108	173	352
283	141	790	627	218	310	57
197	37	1224	50	61	54	45
238	113	1906	299	101	502	63
80	50	339	258	75	150	92
253	151	470	427	160	189	70
191	152	3382	92	44	574	148
323	199	855	176	78	328	71
292	173	403	334	171	260	183
128	66	613	346	78	253	282
7	7	5354	54	10	41	6
68	37	351	200	99	174	89
135	87	1133	106	67	331	40
36	29	2426	37	25	121	36
190	154	674	44	31	121	90
220	93	200	187	102	123	92
157	50	353	290	95	138	241
221	162	244	250	115	210	120
84	47	3797	30	22	199	19
69	67	250	153	32	99	36
171	62	833	205	89	352	8
220	153	318	212	170	165	85
142	169	1119	143	80	228	108

103	39	1575	41	45	225	27
57	37	2189	69	24	185	268
137	80	153	106	58	71	18
208	190	385	142	45	201	12
112	77	1598	66	43	182	31
99	81	2438	85	65	341	80
141	75	574	33	29	154	126
90	68	236	132	51	102	98
131	94	236	268	40	98	46
65	43	2178	73	38	314	42
127	75	450	106	62	64	21
142	64	222	95	76	195	101
50	18	351	51	29	452	154
153	105	633	39	55	104	29
153	105	633	39	55	104	29
107	120	125	81	45	87	37
37	10	173	170	35	136	49
23	16	44	70	18	66	95
10	19	742	17	42	32	22
50	22	270	55	15	69	101
11	6	2055	17	9	19	1
111	54	162	87	66	62	19
58	51	174	118	51	124	6
35	12	116	49	34	39	30
29	13	38	61	10	22	22
134	68	65	72	29	88	65
2	0	753	547	137	304	3
101	49	165	79	42	51	78
74	29	336	48	25	86	23
32	27	79	73	8	30	11
19	6	555	36	25	67	16
53	26	152	46	13	61	54
11	3	320	62	26	161	126
18	14	257	60	25	65	31
35	31	392	47	27	68	21
26	40	76	86	26	86	13
13	9	161	94	38	97	50
32	23	129	69	23	58	11
32	24	108	96	32	46	122
82	56	223	69	46	60	21
14	7	1503	8	4	23	1
22	19	225	103	50	73	18
51	27	46	66	22	38	23
79	46	239	42	28	76	10
41	31	260	128	40	104	23
32	45	118	116	22	56	17
26	28	337	26	13	39	37
24	25	619	36	23	117	27
2	4	138	5	10	9	44
50	18	105	94	19	43	15
35	4	186	44	20	15	142
24	30	79	84	27	56	8
53	36	152	71	21	94	23
17	41	39	41	9	10	13
22	18	62	33	10	36	12
4	2	119	110	25	107	44
13	9	1268	1	2	8	1
43	31	65	57	16	20	5
33	42	83	70	25	62	15

15	10	300	19	9	16	11
33	36	193	18	8	22	12
18	6	99	69	41	47	30
29	34	39	50	8	34	10
16	16	15	33	64	25	6
22	21	572	11	10	21	5
9	3	144	45	13	33	45
52	13	89	32	13	18	8
15	7	189	19	24	65	61
40	19	78	36	19	29	21
26	13	148	26	4	39	13
43	10	347	13	7	20	6
54	31	45	49	19	30	16
20	81	50	8	10	11	4
45	22	117	17	13	42	16
7	11	30	20	5	16	6
7	56	45	9	21	8	1
8	7	89	95	30	1	14
20	13	451	13	10	41	11
10	8	12	6	15	4	14
36	22	338	16	10	52	11
33	8	27	20	13	10	50
18	15	64	54	20	27	27
17	12	423	13	10	41	9
1	5	6	2	4	4	14
43	14	112	16	20	26	10
13	12	138	38	14	46	6
0	222	2	23	91	120	0
23	24	217	1	2	28	10
26	13	31	8	2	7	9
53	15	38	55	23	29	29
2	3	98	37	16	49	7
51	39	100	40	27	68	4
1	0	225	3	0	6	36
14	7	183	40	17	59	27
24	14	33	10	3	15	5
8	7	130	10	23	30	4
13	5	51	88	20	34	5
10	7	61	9	32	75	29
22	17	53	35	9	27	17
9	5	26	20	9	14	12
15	8	237	6	6	29	8
3	1	15	16	24	6	4
14	10	164	77	31	43	4
17	12	66	40	9	22	11
15	23	27	11	5	9	11
9	6	77	49	10	39	61
18	11	15	16	5	12	8
17	14	32	35	6	17	1
0	9	114	1	4	7	3
13	8	36	38	12	19	20
9	4	111	8	5	25	24
28	13	63	21	12	21	2
3	3	85	23	13	31	2
14	2	94	16	7	30	13
0	0	360	1	0	2	2
8	17	77	28	4	49	1
22	9	420	69	32	143	0
7	3	63	39	10	20	4

5	3	54	5	5	16	2
8	7	51	17	8	17	14
13	3	20	2	10	6	2
14	13	22	17	11	14	5
28	11	34	27	13	32	13
7	1	54	44	8	40	14
6	8	20	20	9	11	6
7	9	39	9	4	19	3
18	11	77	1	2	9	2
9	5	31	16	4	17	6
3	3	325	2	0	4	0
8	1	11	23	9	5	6
3	0	106	9	7	28	5
4	6	40	38	10	16	5
4	3	13	16	5	5	3
11	10	102	11	2	6	7
13	11	67	2	5	14	4
5	1	58	20	16	17	1
7	6	29	6	5	4	5
1	0	25	3	1	9	1
10	9	132	0	2	20	2
22	10	42	10	5	13	3
22	10	42	10	5	13	3
3	3	2	3	10	1	2
14	6	25	30	3	18	7
14	5	127	31	11	19	1
7	5	21	3	7	2	1
14	13	53	12	4	13	0
8	2	23	12	4	10	6
6	2	22	4	4	12	17
7	9	93	24	6	34	9
2	1	22	9	3	9	4
7	14	21	4	1	10	3
6	4	69	15	2	24	2
5	2	32	12	10	11	12
5	9	25	7	4	13	2
0	1	342	0	1	2	0
7	4	24	18	5	9	7
23	8	58	4	4	17	3
8	10	12	22	7	13	1
5	7	28	23	7	7	6
21	8	53	4	4	17	3
9	0	9	8	8	5	14
8	5	3	8	5	7	9
2	1	50	10	1	9	0
6	5	19	10	1	10	11
1	0	212	1	0	0	0
8	5	10	12	4	6	7
1	3	22	27	8	8	13
4	2	20	28	6	11	6
0	5	9	3	1	1	4
13	7	64	7	6	23	7
1	1	167	1	1	0	2
3	5	15	8	1	9	4
8	8	67	6	1	13	7
0	0	0	0	0	0	1
7	6	20	22	5	13	6
8	16	16	7	5	6	7
4	4	16	14	2	8	0

2	16	10	3	0	1	1
5	1	16	5	3	11	6
3	3	6	1	3	0	1
4	3	17	7	0	13	2
10	3	3	17	3	5	2
7	1	3	9	7	8	2
7	8	49	6	1	13	6
0	2	5	4	2	1	2
2	1	183	3	0	8	3
6	5	100	4	0	2	0
7	3	33	0	4	24	0
7	2	6	9	7	4	1
4	2	36	6	6	6	2
4	0	4	5	3	1	27
2	4	3	1	2	1	7
13	4	29	1	1	7	3
8	3	17	10	5	14	4
3	1	23	4	5	5	1
1	0	8	7	2	5	2
1	1	8	0	7	1	2
1	11	18	8	5	1	0
4	1	1	3	1	7	2
5	4	10	10	0	2	4
1	2	15	16	2	4	15
7	2	19	1	4	6	9
0	1	24	15	5	3	1
5	3	5	5	2	0	1
2	2	6	4	1	2	6
3	1	4	11	2	4	3
4	4	18	2	3	5	0
3	3	19	12	3	12	3
2	1	54	0	1	0	15
4	4	54	0	2	4	0
2	2	50	9	2	10	2
2	3	25	3	1	7	1
2	0	6	6	3	9	1
8	2	8	1	3	1	6
0	1	2	10	0	5	3
10	7	16	11	7	5	1
3	1	2	6	4	2	3
2	3	17	0	0	1	0
6	0	7	5	5	1	3
1	0	3	3	2	0	0
2	6	7	2	0	2	0
0	1	4	1	1	1	0
2	0	3	3	1	3	0
2	0	10	2	3	2	0
3	2	15	2	2	10	2
0	2	6	7	1	5	0
4	4	4	4	0	3	6
6	3	4	4	2	3	5
2	1	63	0	0	1	2
9	5	7	6	3	2	0
5	2	31	2	0	4	2
7	0	18	6	2	10	0
2	1	1	3	0	2	2
0	1	5	5	2	3	5
1	0	46	27	11	14	0
4	0	23	6	3	9	5

0	0	6	1	0	2	19
0	0	0	1	3	0	1
1	1	3	1	0	2	1
6	1	7	2	1	2	0
1	0	7	11	1	5	4
3	0	4	6	3	5	0
0	1	12	5	0	9	0
1	3	51	0	3	0	3
0	3	21	2	1	6	1
1	2	6	2	0	2	0
2	0	3	4	2	2	0
1	0	10	5	3	4	1
2	0	2	2	2	0	8
3	2	53	0	0	0	0
0	2	3	7	0	6	4
1	0	9	0	1	4	1
3	1	9	6	3	2	2
4	3	3	4	0	2	0
2	1	17	0	0	5	2
2	0	12	3	1	3	0
0	3	6	1	2	2	1
2	0	8	5	0	3	0
11	0	4	2	3	4	2
0	0	8	6	0	6	4
1	0	9	2	1	1	6
2	1	3	3	1	1	0
0	1	26	1	1	6	2
2	1	31	2	1	3	0
0	0	3	8	0	1	7
1	1	6	4	3	4	0
1	2	8	28	1	0	1
2	0	3	4	1	4	5
0	0	11	0	1	4	0
1	0	0	1	1	1	1
0	1	41	0	0	1	1
1	3	3	2	0	0	0
3	0	7	1	0	3	0
0	1	5	4	0	2	1
1	3	3	4	1	3	2
0	2	5	8	2	1	3
1	1	7	3	3	2	2
3	1	7	0	0	2	4
0	0	5	1	2	1	0
3	2	7	2	2	2	2
0	0	3	6	0	1	0
2	0	1	0	1	2	2
2	0	8	3	1	0	0
1	1	2	5	1	3	4
6	1	13	1	0	3	0
0	0	4	0	1	0	0
1	1	4	4	1	0	0
1	0	8	2	2	0	0
0	2	16	0	0	2	0
0	4	0	1	0	1	1
0	1	3	2	0	1	1
0	2	16	0	0	2	3
1	0	7	2	0	2	0
0	2	1	1	0	2	1
0	3	11	1	0	1	0

2	1	18	1	0	3	3
0	0	1	3	3	0	1
0	1	6	0	0	1	1
0	0	9	0	1	1	2
1	2	6	4	4	0	1
1	0	4	6	0	2	2
0	0	0	0	0	0	1
3	0	10	3	1	1	0
3	0	8	1	2	2	1
0	0	5	0	1	3	2
3	0	13	0	0	2	0
1	0	58	1	0	0	0
0	0	43	0	0	0	0
0	0	6	1	0	0	4
0	0	1	2	0	0	1
2	1	7	0	0	2	1
0	0	1	2	0	4	2
5	5	6	3	1	1	1
0	0	40	0	0	0	0
0	1	7	1	0	0	0
1	2	9	0	0	0	0
1	0	2	1	0	0	0
3	0	1	0	0	0	0
2	1	2	0	1	3	1
0	0	4	5	2	1	4
0	0	24	0	0	0	0
0	0	2	0	1	0	0
2	1	10	1	0	1	1
0	2	8	3	0	7	1
1	0	6	1	1	3	1
1	1	19	0	0	0	0
0	1	4	0	0	1	3
1	0	2	1	1	2	0
0	0	1	2	0	4	3
1	1	4	1	0	0	0
1	0	4	2	0	1	3
1	0	4	4	3	0	0
1	0	8	1	0	5	5
1	0	2	0	0	1	0
0	3	0	0	0	0	0
1	0	12	0	0	1	1
1	1	2	0	0	1	0
1	2	1	1	1	1	1
0	0	3	0	0	0	0
1	1	5	0	0	2	0
0	2	14	0	2	1	0
1	0	22	0	0	0	0
0	0	2	0	0	0	0
4	0	4	1	2	2	0
1	1	1	1	0	3	0
0	1	2	1	1	1	0
0	0	16	1	1	3	0
1	0	5	1	0	0	0
0	2	3	1	0	0	1
1	1	4	1	0	3	0
1	2	9	2	0	3	1
0	1	5	2	0	2	0
0	0	2	1	0	0	2
0	1	1	1	1	0	0

1	0	1	1	0	0	2
1	0	2	0	0	0	0
1	0	3	0	1	0	0
0	0	2	3	0	1	1
1	0	8	2	4	1	0
1	0	18	1	0	0	0
0	0	2	1	1	0	0
0	0	11	0	0	0	1
0	1	4	1	2	0	2
1	0	6	1	0	0	0
0	0	1	0	0	0	0
0	0	5	2	0	3	0
1	2	14	0	0	0	0
0	0	6	2	1	0	1
1	0	9	3	0	5	1
1	0	4	1	0	3	0
2	0	1	3	0	0	0
0	0	19	3	0	0	0
0	0	14	0	0	1	0
0	2	2	2	0	1	0
0	0	8	0	0	2	0
0	0	17	0	0	0	0
1	0	10	0	0	0	0
0	1	0	1	2	1	1
2	0	0	0	0	0	1
3	1	3	0	0	0	0
1	0	0	0	0	0	0
1	0	0	1	0	0	0
0	0	12	0	1	0	0
0	0	18	1	0	4	0
1	5	11	0	1	0	1
0	1	2	2	0	1	1
1	1	4	2	0	3	0
0	0	2	1	0	1	0
2	1	1	1	1	0	0
0	1	3	0	0	0	0
0	1	1	2	0	0	1
0	0	3	1	0	2	2
1	0	2	1	0	0	0
2	0	0	1	0	0	0
0	0	7	0	0	1	0
2	0	0	0	0	0	0
1	0	1	0	0	0	1
0	0	2	4	0	1	1
0	0	4	1	2	0	2
0	2	4	2	1	2	1
0	1	2	1	0	1	1
0	0	1	0	0	2	1
1	0	1	0	0	1	0
1	0	2	1	0	0	1
1	1	0	0	0	0	1
1	0	10	0	0	0	0
0	0	6	1	0	0	1
0	0	2	0	0	2	1
1	0	5	2	1	3	1
1	0	2	2	0	2	1
0	0	1	2	0	0	0
0	0	0	1	1	0	0
0	0	1	0	0	0	0

1	1	2	0	0	1	1
1	0	0	1	1	1	2
0	0	4	0	1	0	2
0	0	3	1	0	1	0
0	0	3	0	2	1	0
4	1	6	1	2	0	2
1	0	1	0	1	0	1
0	0	3	1	1	2	0
0	0	2	0	0	0	0
1	0	0	4	0	0	0
1	3	1	0	0	1	2
0	0	2	2	0	0	1
0	0	0	0	0	0	0
G_31	G_32	G_4	G_5	G_6	G_7	A_10
CHR_NP	CHR_NP	CHR_NP	CHR_NP	CHR_NP	CHR_NP	CHR_P
611755	504822	7342160	5152596	969299	5229327	3409557
17	19	21	25	21	25	27
-0.91	-0.51	-0.09	0.82	-0.06	0.70	1.26

0.27	0.77	0.85	0.91	0.31	0.22	0.11
23	13	10	7	16	19	22
1	1	1	1	1	1	1
64	65	66	67	68	69	70
37	38	1	2	3	4	5
G_6	G_7	A_10	A_12	A_18	A_3	A_5
CHR_NP	CHR_NP	CHR_P	CHR_P	CHR_P	CHR_P	CHR_P
488065	1421920	492624	721119	27468	816795	972175
G_6	G_7	A_10	A_12	A_18	A_3	A_5
196834	399578	190797	261344	6469	132111	198821
33091	131009	7668	13505	8375	34328	58994
30921	36819	10054	7691	750	35614	51330
48784	152679	37879	14227	1228	168094	97543
30262	81663	47947	12291	536	64203	87260
10574	100931	5499	86372	452	20677	38724
29185	86225	4865	15656	647	22001	51972
1738	15269	4480	24861	117	4457	7468
8204	87617	5810	49674	88	6761	18115
7982	44347	28115	80322	1803	36876	40293
9832	27797	31440	24136	674	26458	23921
6971	19045	8308	8992	204	26259	32101
7614	28174	9824	5991	139	30575	25249
7585	22198	7741	9409	238	38899	20915
1801	11236	11411	3972	189	9342	9807
3840	4218	5821	1658	7	2939	3906
932	9345	1238	4083	91	5496	12964
1264	4671	5981	3990	345	13701	17197
2901	11037	1600	9484	119	2645	8526

2305	6613	3207	4091	768	2897	7001
1506	4148	2184	2473	149	3338	6047
2124	7540	5042	5947	167	10042	10806
1093	4874	1341	3135	126	869	3590
655	4418	858	3250	109	2006	9031
999	10956	1605	2971	300	1547	5495
997	4583	1511	4140	22	1660	3495
1962	5290	5431	1563	58	9033	4396
1329	1938	6034	2972	204	2727	3487
1244	5804	1572	1962	124	6624	5075
1864	4373	1013	2288	86	1596	2878
1624	4144	838	1545	37	2190	5104
2848	6975	2236	513	18	9944	5368
789	3611	544	3180	9	1671	3742
342	591	715	2604	32	547	1978
1134	5192	1112	1315	86	4632	3492
1422	4545	796	2471	193	15156	5512
686	4225	1073	3383	483	2811	3748
942	4989	1075	3737	399	1795	3909
1021	1788	178	479	145	911	2595
2366	3556	2757	470	12	4393	2097
348	817	563	718	114	3445	5257
928	1980	2074	344	10	1815	2919
794	2672	607	1017	63	3797	3856
210	1372	613	909	79	711	712
514	1473	974	1334	103	1435	2476
731	1358	637	750	34	1382	3150
937	1954	201	1003	16	858	1667
687	1241	1701	515	24	1322	2479
187	702	277	124	1	981	727
124	447	1549	350	8	45	410
477	270	91	664	14	1172	1836
203	826	167	509	31	663	1584
394	1111	255	1145	63	1159	1265
107	509	2169	382	52	524	2087
663	2020	258	382	0	12	46
42	155	358	255	3	43	126
409	1731	103	890	24	939	1409
179	781	256	247	7	368	1337
378	776	421	409	37	823	1500
576	556	317	147	66	925	1334
295	481	231	578	16	1649	1798
176	567	569	275	6	243	634
40	372	31	792	9	94	207
14	80	5	61	1	7	37
498	875	1075	198	31	2408	869
222	1097	173	747	19	1011	1035
167	346	118	637	3	199	376
107	752	287	330	6	544	646
70	433	155	307	8	420	957
208	634	130	888	111	387	591
275	313	86	309	21	537	548
156	327	426	289	9	1862	954
98	531	270	464	16	291	468
175	827	407	527	8	301	336
196	495	60	244	7	228	798
225	116	206	179	5	741	1033
373	1134	187	288	0	16	43
417	650	41	157	107	109	254

252	356	432	49	2	659	614
80	141	23	231	15	347	262
122	698	120	385	4	658	949
108	173	352	111	0	142	322
218	310	57	88	28	1066	638
61	54	45	423	3	33	138
101	502	63	239	13	98	367
75	150	92	162	0	189	346
160	189	70	116	10	367	406
44	574	148	402	1	283	467
78	328	71	273	5	282	579
171	260	183	38	6	413	439
78	253	282	43	6	184	232
10	41	6	17	0	19	9
99	174	89	111	1	91	322
67	331	40	269	6	80	215
25	121	36	159	0	43	107
31	121	90	97	4	111	350
102	123	92	26	6	148	215
95	138	241	58	11	245	323
115	210	120	48	15	343	406
22	199	19	93	0	39	66
32	99	36	238	2	548	373
89	352	8	104	1	93	182
170	165	85	79	1	201	227
80	228	108	65	19	200	306
45	225	27	287	4	147	300
24	185	268	247	3	122	165
58	71	18	66	8	112	234
45	201	12	86	7	85	159
43	182	31	144	0	154	203
65	341	80	93	0	81	133
29	154	126	57	4	67	237
51	102	98	48	3	187	260
40	98	46	80	2	200	293
38	314	42	126	7	230	314
62	64	21	14	3	50	146
76	195	101	42	7	282	229
29	452	154	50	3	81	158
55	104	29	101	1	123	220
55	104	29	101	1	123	220
45	87	37	116	4	211	195
35	136	49	271	2	81	98
18	66	95	71	3	77	185
42	32	22	69	1	15	72
15	69	101	55	5	27	111
9	19	1	3	0	2	5
66	62	19	30	2	174	222
51	124	6	72	2	45	71
34	39	30	34	0	68	115
10	22	22	32	5	170	162
29	88	65	4	3	71	160
137	304	3	20	1	4	5
42	51	78	48	2	74	221
25	86	23	60	2	36	85
8	30	11	46	4	84	115
25	67	16	150	0	21	129
13	61	54	22	0	29	98
26	161	126	43	0	21	29

25	65	31	88	0	89	82
27	68	21	34	1	54	94
26	86	13	26	2	66	85
38	97	50	22	1	12	25
23	58	11	65	1	42	52
32	46	122	13	0	187	93
46	60	21	11	1	59	154
4	23	1	28	0	9	9
50	73	18	30	0	220	73
22	38	23	15	0	82	196
28	76	10	20	1	21	80
40	104	23	38	0	128	105
22	56	17	32	2	273	128
13	39	37	51	1	5	27
23	117	27	57	0	96	108
10	9	44	49	0	4	35
19	43	15	11	0	33	99
20	15	142	4	0	13	52
27	56	8	41	2	120	58
21	94	23	15	4	77	44
9	10	13	37	1	75	39
10	36	12	32	0	48	95
25	107	44	31	1	23	22
2	8	1	4	0	4	7
16	20	5	17	1	43	74
25	62	15	10	2	63	46
9	16	11	38	0	17	52
8	22	12	14	1	11	68
41	47	30	16	2	82	51
8	34	10	11	0	48	65
64	25	6	238	2	14	14
10	21	5	18	1	2	10
13	33	45	43	1	65	68
13	18	8	6	0	17	25
24	65	61	54	0	8	35
19	29	21	15	2	28	32
4	39	13	15	0	16	31
7	20	6	14	1	2	46
19	30	16	9	0	53	60
10	11	4	45	0	83	96
13	42	16	22	1	23	46
5	16	6	56	2	24	34
21	8	1	19	0	58	56
30	1	14	0	1	41	47
10	41	11	20	0	13	22
15	4	14	28	0	30	76
10	52	11	67	1	30	28
13	10	50	28	5	48	48
20	27	27	17	0	49	55
10	41	9	17	0	13	21
4	4	14	25	0	106	112
20	26	10	8	0	23	30
14	46	6	4	0	2	12
91	120	0	99	2	16	29
2	28	10	32	1	5	7
2	7	9	17	1	52	80
23	29	29	4	1	36	75
16	49	7	38	0	17	21
27	68	4	4	2	25	35

0	6	36	7	0	1	9
17	59	27	15	0	66	46
3	15	5	22	1	21	50
23	30	4	13	0	17	64
20	34	5	0	0	24	50
32	75	29	21	0	11	32
9	27	17	4	1	96	44
9	14	12	6	0	2	36
6	29	8	34	1	49	37
24	6	4	19	0	16	26
31	43	4	11	0	38	30
9	22	11	10	0	52	60
5	9	11	11	0	15	46
10	39	61	30	0	101	29
5	12	8	9	1	64	63
6	17	1	6	0	8	41
4	7	3	20	0	22	32
12	19	20	5	0	65	44
5	25	24	8	0	4	9
12	21	2	3	1	9	25
13	31	2	4	0	4	9
7	30	13	3	2	0	23
0	2	2	0	0	1	0
4	49	1	4	0	28	30
32	143	0	17	1	1	7
10	20	4	27	0	22	29
5	16	2	18	0	14	35
8	17	14	30	1	38	26
10	6	2	11	0	43	66
11	14	5	3	0	8	14
13	32	13	4	0	40	25
8	40	14	6	0	16	18
9	11	6	4	0	43	39
4	19	3	27	3	20	16
2	9	2	21	0	3	11
4	17	6	8	2	35	16
0	4	0	8	0	2	1
9	5	6	7	0	9	16
7	28	5	5	0	1	8
10	16	5	12	0	23	17
5	5	3	3	0	9	13
2	6	7	5	0	8	17
5	14	4	5	0	4	16
16	17	1	3	0	10	20
5	4	5	13	1	27	42
1	9	1	21	0	5	8
2	20	2	14	0	6	18
5	13	3	12	0	3	14
5	13	3	12	0	3	14
10	1	2	4	0	25	19
3	18	7	6	0	44	20
11	19	1	2	0	11	9
7	2	1	4	0	16	20
4	13	0	4	0	5	12
4	10	6	3	0	4	23
4	12	17	10	0	6	22
6	34	9	9	0	14	9
3	9	4	1	0	5	3
1	10	3	5	1	5	23

2	24	2	1	0	2	6
10	11	12	5	0	15	19
4	13	2	19	1	14	11
1	2	0	3	0	0	1
5	9	7	10	0	27	24
4	17	3	10	0	7	28
7	13	1	1	0	10	14
7	7	6	4	0	43	25
4	17	3	10	0	4	24
8	5	14	2	1	7	20
5	7	9	5	0	17	20
1	9	0	13	0	7	13
1	10	11	1	0	17	4
0	0	0	0	0	0	1
4	6	7	1	0	3	6
8	8	13	3	1	39	15
6	11	6	5	0	51	18
1	1	4	3	0	20	22
6	23	7	6	0	11	14
1	0	2	0	0	2	1
1	9	4	6	0	51	31
1	13	7	7	0	3	12
0	0	1	4	0	69	1
5	13	6	1	1	49	8
5	6	7	6	0	9	23
2	8	0	4	1	15	9
0	1	1	7	0	15	13
3	11	6	3	0	16	8
3	0	1	5	0	16	30
0	13	2	4	0	1	5
3	5	2	6	1	20	10
7	8	2	2	0	5	11
1	13	6	5	0	2	11
2	1	2	8	0	12	17
0	8	3	5	0	0	4
0	2	0	4	0	9	4
4	24	0	2	1	1	3
7	4	1	1	0	20	15
6	6	2	12	0	5	9
3	1	27	0	0	2	7
2	1	7	6	0	8	13
1	7	3	9	0	7	7
5	14	4	1	1	15	11
5	5	1	5	0	4	5
2	5	2	6	0	1	4
7	1	2	13	0	9	17
5	1	0	1	0	3	8
1	7	2	4	0	17	18
0	2	4	0	0	1	10
2	4	15	4	0	17	7
4	6	9	3	0	8	4
5	3	1	0	0	11	5
2	0	1	6	1	14	11
1	2	6	2	0	4	10
2	4	3	5	0	4	21
3	5	0	0	0	3	7
3	12	3	2	0	6	6
1	0	15	4	0	2	6
2	4	0	2	0	0	1

2	10	2	4	2	10	12
1	7	1	4	1	15	9
3	9	1	8	2	14	9
3	1	6	2	0	3	7
0	5	3	2	0	2	5
7	5	1	1	0	7	6
4	2	3	1	0	1	3
0	1	0	6	0	2	7
5	1	3	1	0	4	11
2	0	0	2	0	4	6
0	2	0	0	0	3	7
1	1	0	4	0	10	16
1	3	0	11	0	8	7
3	2	0	5	0	12	6
2	10	2	10	0	14	9
1	5	0	3	0	10	7
0	3	6	0	0	4	16
2	3	5	0	0	14	6
0	1	2	6	0	0	1
3	2	0	1	0	7	9
0	4	2	7	1	0	5
2	10	0	2	0	1	3
0	2	2	1	0	1	3
2	3	5	0	0	6	6
11	14	0	1	0	0	0
3	9	5	2	0	3	4
0	2	19	4	0	1	6
3	0	1	2	0	10	8
0	2	1	1	0	1	3
1	2	0	1	0	3	4
1	5	4	2	0	2	4
3	5	0	0	0	0	1
0	9	0	7	0	6	6
3	0	3	3	0	2	7
1	6	1	8	0	14	8
0	2	0	3	0	5	8
2	2	0	1	0	1	5
3	4	1	3	0	2	7
2	0	8	2	4	6	13
0	0	0	1	0	0	0
0	6	4	0	0	7	26
1	4	1	9	0	2	8
3	2	2	0	0	5	14
0	2	0	5	1	3	4
0	5	2	2	0	2	3
1	3	0	2	1	1	11
2	2	1	0	0	0	4
0	3	0	0	0	0	5
3	4	2	2	0	6	7
0	6	4	3	0	10	7
1	1	6	0	0	5	9
1	1	0	0	0	3	9
1	6	2	1	0	0	5
1	3	0	1	0	0	2
0	1	7	1	0	1	4
3	4	0	1	0	1	3
1	0	1	2	0	2	2
1	4	5	2	0	8	5
1	4	0	1	0	1	3

1	1	1	3	0	3	4
0	1	1	2	0	3	0
0	0	0	3	0	6	3
0	3	0	0	0	1	0
0	2	1	2	0	7	4
1	3	2	2	0	2	4
2	1	3	0	1	4	4
3	2	2	0	0	3	2
0	2	4	0	0	2	5
2	1	0	2	0	2	9
2	2	2	1	0	1	4
0	1	0	1	0	0	0
1	2	2	2	0	1	4
1	0	0	0	0	4	5
1	3	4	0	0	1	2
0	3	0	1	0	0	2
1	0	0	0	0	0	4
1	0	0	0	0	12	5
2	0	0	0	0	1	8
0	2	0	5	0	0	1
0	1	1	2	0	3	10
0	1	1	0	0	1	8
0	2	3	3	0	0	4
0	2	0	1	0	0	3
0	2	1	1	1	0	7
0	1	0	2	0	3	3
0	3	3	1	0	4	5
3	0	1	3	0	1	1
0	1	1	0	0	0	8
1	1	2	0	0	1	5
4	0	1	1	0	0	5
0	2	2	2	0	7	3
0	0	1	1	0	0	0
1	1	0	0	0	1	4
2	2	1	0	0	3	7
1	3	2	0	0	0	1
0	2	0	0	0	0	1
0	0	0	1	0	0	1
0	0	0	0	0	0	1
0	0	4	0	0	0	2
0	0	1	0	0	1	0
0	2	1	1	0	2	2
0	4	2	1	1	5	4
1	1	1	1	0	5	2
0	0	0	0	0	0	0
0	0	0	3	0	2	3
0	0	0	0	0	4	8
0	0	0	3	0	1	2
0	0	0	0	0	0	0
1	3	1	1	0	1	1
2	1	4	2	0	2	1
0	0	0	0	0	0	0
1	0	0	0	0	0	0
0	1	1	1	1	1	3
0	7	1	0	0	5	0
1	3	1	0	0	4	1
0	0	0	0	0	0	0
0	1	3	2	0	0	3
1	2	0	1	0	4	5

0	4	3	1	0	5	1
0	0	0	0	0	2	3
0	1	3	1	0	2	2
3	0	0	3	0	5	1
0	5	5	1	0	0	1
0	1	0	1	0	0	1
0	0	0	2	0	3	2
0	1	1	1	0	0	2
0	1	0	0	0	0	0
1	1	1	1	0	1	7
0	0	0	1	0	0	0
0	2	0	2	0	2	0
2	1	0	1	0	0	4
0	0	0	0	0	2	0
0	0	0	0	0	2	2
2	2	0	0	0	2	1
0	3	0	0	0	0	0
1	1	0	1	0	0	3
1	3	0	0	0	2	2
0	0	0	0	0	2	5
0	0	1	3	1	0	2
0	3	0	1	0	2	1
0	3	1	1	0	0	2
0	2	0	2	0	0	0
0	0	2	1	0	4	3
1	0	0	1	0	2	0
0	0	2	2	0	0	3
0	0	0	3	0	0	1
1	0	0	0	0	1	1
0	1	1	7	0	1	1
4	1	0	0	0	1	1
0	0	0	1	0	0	2
1	0	0	0	0	0	0
0	0	1	3	0	0	2
2	0	2	1	0	0	3
0	0	0	1	0	3	0
0	0	0	0	0	1	0
0	3	0	1	0	1	0
0	0	0	1	0	0	0
1	0	1	0	0	1	2
0	5	1	1	0	1	2
0	3	0	0	0	1	3
0	0	0	0	0	2	1
0	0	0	0	0	1	0
0	1	0	1	0	0	0
0	1	0	2	0	2	1
0	2	0	4	0	0	2
0	0	0	1	0	0	0
0	0	0	0	0	0	0
2	1	1	0	0	1	2
0	0	1	0	0	0	0
0	0	0	1	0	1	0
0	0	0	1	0	0	0
0	0	0	0	0	2	2
1	0	0	1	0	0	0
0	4	0	4	0	3	1
1	0	1	3	0	1	4
0	1	1	0	0	3	2
0	3	0	1	0	1	4

0	1	0	2	0	0	3
1	0	0	1	0	0	1
0	0	0	4	0	3	2
0	0	1	2	0	1	0
0	2	2	0	0	0	1
0	0	0	0	0	0	0
0	0	0	0	0	1	0
0	1	0	2	0	0	0
0	0	0	0	0	1	2
0	0	1	0	0	1	1
0	1	1	0	0	0	0
2	0	2	1	0	0	2
1	2	1	0	0	3	1
0	1	1	1	0	2	1
0	2	1	0	0	2	0
0	1	0	0	0	1	4
0	0	1	0	0	0	0
0	0	1	0	0	0	1
0	0	0	0	0	0	2
0	0	1	1	0	1	1
0	2	1	0	0	0	0
1	3	1	0	0	3	1
0	2	1	1	0	1	2
0	0	0	0	0	0	1
1	0	0	0	0	0	0
0	0	0	1	0	0	0
0	1	1	0	0	0	2
1	1	2	0	0	1	0
1	0	2	1	0	0	1
0	1	0	0	0	0	0
2	1	0	0	0	2	0
2	0	2	0	0	1	2
1	0	1	0	0	0	0
1	2	0	1	0	0	0
0	0	0	3	0	0	0
0	0	0	0	0	1	0
0	1	2	0	0	2	3
0	0	1	0	0	1	1

0.27	0.77	0.85	0.91	0.31	0.22	0.11
23	13	10	7	16	19	22
1	1	1	1	1	1	1

64	65	66	67	68	69	70
37	38	1	2	3	4	5
G_6	G_7	A_10	A_12	A_18	A_3	A_5
CHR_NP	CHR_NP	CHR_P	CHR_P	CHR_P	CHR_P	CHR_P
488065	1421920	492624	721119	27468	816795	972175
CHR_NP	CHR_NP	CHR_P	CHR_P	CHR_P	CHR_P	CHR_P
196834	399578	190797	261344	6469	132111	198821
33091	131009	7668	13505	8375	34328	58994
30921	36819	10054	7691	750	35614	51330
48784	152679	37879	14227	1228	168094	97543
30262	81663	47947	12291	536	64203	87260

10574	100931	5499	86372	452	20677	38724
29185	86225	4865	15656	647	22001	51972
1738	15269	4480	24861	117	4457	7468
8204	87617	5810	49674	88	6761	18115
7982	44347	28115	80322	1803	36876	40293
9832	27797	31440	24136	674	26458	23921
6971	19045	8308	8992	204	26259	32101
7614	28174	9824	5991	139	30575	25249
7585	22198	7741	9409	238	38899	20915
1801	11236	11411	3972	189	9342	9807
3840	4218	5821	1658	7	2939	3906
932	9345	1238	4083	91	5496	12964
1264	4671	5981	3990	345	13701	17197
2901	11037	1600	9484	119	2645	8526
2305	6613	3207	4091	768	2897	7001
1506	4148	2184	2473	149	3338	6047
2124	7540	5042	5947	167	10042	10806
1093	4874	1341	3135	126	869	3590
655	4418	858	3250	109	2006	9031
999	10956	1605	2971	300	1547	5495
997	4583	1511	4140	22	1660	3495
1962	5290	5431	1563	58	9033	4396
1329	1938	6034	2972	204	2727	3487
1244	5804	1572	1962	124	6624	5075
1864	4373	1013	2288	86	1596	2878
1624	4144	838	1545	37	2190	5104
2848	6975	2236	513	18	9944	5368
789	3611	544	3180	9	1671	3742
342	591	715	2604	32	547	1978
1134	5192	1112	1315	86	4632	3492
1422	4545	796	2471	193	15156	5512
686	4225	1073	3383	483	2811	3748
942	4989	1075	3737	399	1795	3909
1021	1788	178	479	145	911	2595
2366	3556	2757	470	12	4393	2097
348	817	563	718	114	3445	5257
928	1980	2074	344	10	1815	2919
794	2672	607	1017	63	3797	3856
210	1372	613	909	79	711	712
514	1473	974	1334	103	1435	2476
731	1358	637	750	34	1382	3150
937	1954	201	1003	16	858	1667
687	1241	1701	515	24	1322	2479
187	702	277	124	1	981	727
124	447	1549	350	8	45	410
477	270	91	664	14	1172	1836
203	826	167	509	31	663	1584
394	1111	255	1145	63	1159	1265
107	509	2169	382	52	524	2087
663	2020	258	382	0	12	46
42	155	358	255	3	43	126
409	1731	103	890	24	939	1409
179	781	256	247	7	368	1337
378	776	421	409	37	823	1500
576	556	317	147	66	925	1334
295	481	231	578	16	1649	1798
176	567	569	275	6	243	634
40	372	31	792	9	94	207
14	80	5	61	1	7	37

498	875	1075	198	31	2408	869
222	1097	173	747	19	1011	1035
167	346	118	637	3	199	376
107	752	287	330	6	544	646
70	433	155	307	8	420	957
208	634	130	888	111	387	591
275	313	86	309	21	537	548
156	327	426	289	9	1862	954
98	531	270	464	16	291	468
175	827	407	527	8	301	336
196	495	60	244	7	228	798
225	116	206	179	5	741	1033
373	1134	187	288	0	16	43
417	650	41	157	107	109	254
252	356	432	49	2	659	614
80	141	23	231	15	347	262
122	698	120	385	4	658	949
108	173	352	111	0	142	322
218	310	57	88	28	1066	638
61	54	45	423	3	33	138
101	502	63	239	13	98	367
75	150	92	162	0	189	346
160	189	70	116	10	367	406
44	574	148	402	1	283	467
78	328	71	273	5	282	579
171	260	183	38	6	413	439
78	253	282	43	6	184	232
10	41	6	17	0	19	9
99	174	89	111	1	91	322
67	331	40	269	6	80	215
25	121	36	159	0	43	107
31	121	90	97	4	111	350
102	123	92	26	6	148	215
95	138	241	58	11	245	323
115	210	120	48	15	343	406
22	199	19	93	0	39	66
32	99	36	238	2	548	373
89	352	8	104	1	93	182
170	165	85	79	1	201	227
80	228	108	65	19	200	306
45	225	27	287	4	147	300
24	185	268	247	3	122	165
58	71	18	66	8	112	234
45	201	12	86	7	85	159
43	182	31	144	0	154	203
65	341	80	93	0	81	133
29	154	126	57	4	67	237
51	102	98	48	3	187	260
40	98	46	80	2	200	293
38	314	42	126	7	230	314
62	64	21	14	3	50	146
76	195	101	42	7	282	229
29	452	154	50	3	81	158
55	104	29	101	1	123	220
55	104	29	101	1	123	220
45	87	37	116	4	211	195
35	136	49	271	2	81	98
18	66	95	71	3	77	185
42	32	22	69	1	15	72

15	69	101	55	5	27	111
9	19	1	3	0	2	5
66	62	19	30	2	174	222
51	124	6	72	2	45	71
34	39	30	34	0	68	115
10	22	22	32	5	170	162
29	88	65	4	3	71	160
137	304	3	20	1	4	5
42	51	78	48	2	74	221
25	86	23	60	2	36	85
8	30	11	46	4	84	115
25	67	16	150	0	21	129
13	61	54	22	0	29	98
26	161	126	43	0	21	29
25	65	31	88	0	89	82
27	68	21	34	1	54	94
26	86	13	26	2	66	85
38	97	50	22	1	12	25
23	58	11	65	1	42	52
32	46	122	13	0	187	93
46	60	21	11	1	59	154
4	23	1	28	0	9	9
50	73	18	30	0	220	73
22	38	23	15	0	82	196
28	76	10	20	1	21	80
40	104	23	38	0	128	105
22	56	17	32	2	273	128
13	39	37	51	1	5	27
23	117	27	57	0	96	108
10	9	44	49	0	4	35
19	43	15	11	0	33	99
20	15	142	4	0	13	52
27	56	8	41	2	120	58
21	94	23	15	4	77	44
9	10	13	37	1	75	39
10	36	12	32	0	48	95
25	107	44	31	1	23	22
2	8	1	4	0	4	7
16	20	5	17	1	43	74
25	62	15	10	2	63	46
9	16	11	38	0	17	52
8	22	12	14	1	11	68
41	47	30	16	2	82	51
8	34	10	11	0	48	65
64	25	6	238	2	14	14
10	21	5	18	1	2	10
13	33	45	43	1	65	68
13	18	8	6	0	17	25
24	65	61	54	0	8	35
19	29	21	15	2	28	32
4	39	13	15	0	16	31
7	20	6	14	1	2	46
19	30	16	9	0	53	60
10	11	4	45	0	83	96
13	42	16	22	1	23	46
5	16	6	56	2	24	34
21	8	1	19	0	58	56
30	1	14	0	1	41	47
10	41	11	20	0	13	22

15	4	14	28	0	30	76
10	52	11	67	1	30	28
13	10	50	28	5	48	48
20	27	27	17	0	49	55
10	41	9	17	0	13	21
4	4	14	25	0	106	112
20	26	10	8	0	23	30
14	46	6	4	0	2	12
91	120	0	99	2	16	29
2	28	10	32	1	5	7
2	7	9	17	1	52	80
23	29	29	4	1	36	75
16	49	7	38	0	17	21
27	68	4	4	2	25	35
0	6	36	7	0	1	9
17	59	27	15	0	66	46
3	15	5	22	1	21	50
23	30	4	13	0	17	64
20	34	5	0	0	24	50
32	75	29	21	0	11	32
9	27	17	4	1	96	44
9	14	12	6	0	2	36
6	29	8	34	1	49	37
24	6	4	19	0	16	26
31	43	4	11	0	38	30
9	22	11	10	0	52	60
5	9	11	11	0	15	46
10	39	61	30	0	101	29
5	12	8	9	1	64	63
6	17	1	6	0	8	41
4	7	3	20	0	22	32
12	19	20	5	0	65	44
5	25	24	8	0	4	9
12	21	2	3	1	9	25
13	31	2	4	0	4	9
7	30	13	3	2	0	23
0	2	2	0	0	1	0
4	49	1	4	0	28	30
32	143	0	17	1	1	7
10	20	4	27	0	22	29
5	16	2	18	0	14	35
8	17	14	30	1	38	26
10	6	2	11	0	43	66
11	14	5	3	0	8	14
13	32	13	4	0	40	25
8	40	14	6	0	16	18
9	11	6	4	0	43	39
4	19	3	27	3	20	16
2	9	2	21	0	3	11
4	17	6	8	2	35	16
0	4	0	8	0	2	1
9	5	6	7	0	9	16
7	28	5	5	0	1	8
10	16	5	12	0	23	17
5	5	3	3	0	9	13
2	6	7	5	0	8	17
5	14	4	5	0	4	16
16	17	1	3	0	10	20
5	4	5	13	1	27	42

1	9	1	21	0	5	8
2	20	2	14	0	6	18
5	13	3	12	0	3	14
5	13	3	12	0	3	14
10	1	2	4	0	25	19
3	18	7	6	0	44	20
11	19	1	2	0	11	9
7	2	1	4	0	16	20
4	13	0	4	0	5	12
4	10	6	3	0	4	23
4	12	17	10	0	6	22
6	34	9	9	0	14	9
3	9	4	1	0	5	3
1	10	3	5	1	5	23
2	24	2	1	0	2	6
10	11	12	5	0	15	19
4	13	2	19	1	14	11
1	2	0	3	0	0	1
5	9	7	10	0	27	24
4	17	3	10	0	7	28
7	13	1	1	0	10	14
7	7	6	4	0	43	25
4	17	3	10	0	4	24
8	5	14	2	1	7	20
5	7	9	5	0	17	20
1	9	0	13	0	7	13
1	10	11	1	0	17	4
0	0	0	0	0	0	1
4	6	7	1	0	3	6
8	8	13	3	1	39	15
6	11	6	5	0	51	18
1	1	4	3	0	20	22
6	23	7	6	0	11	14
1	0	2	0	0	2	1
1	9	4	6	0	51	31
1	13	7	7	0	3	12
0	0	1	4	0	69	1
5	13	6	1	1	49	8
5	6	7	6	0	9	23
2	8	0	4	1	15	9
0	1	1	7	0	15	13
3	11	6	3	0	16	8
3	0	1	5	0	16	30
0	13	2	4	0	1	5
3	5	2	6	1	20	10
7	8	2	2	0	5	11
1	13	6	5	0	2	11
2	1	2	8	0	12	17
0	8	3	5	0	0	4
0	2	0	4	0	9	4
4	24	0	2	1	1	3
7	4	1	1	0	20	15
6	6	2	12	0	5	9
3	1	27	0	0	2	7
2	1	7	6	0	8	13
1	7	3	9	0	7	7
5	14	4	1	1	15	11
5	5	1	5	0	4	5
2	5	2	6	0	1	4

7	1	2	13	0	9	17
5	1	0	1	0	3	8
1	7	2	4	0	17	18
0	2	4	0	0	1	10
2	4	15	4	0	17	7
4	6	9	3	0	8	4
5	3	1	0	0	11	5
2	0	1	6	1	14	11
1	2	6	2	0	4	10
2	4	3	5	0	4	21
3	5	0	0	0	3	7
3	12	3	2	0	6	6
1	0	15	4	0	2	6
2	4	0	2	0	0	1
2	10	2	4	2	10	12
1	7	1	4	1	15	9
3	9	1	8	2	14	9
3	1	6	2	0	3	7
0	5	3	2	0	2	5
7	5	1	1	0	7	6
4	2	3	1	0	1	3
0	1	0	6	0	2	7
5	1	3	1	0	4	11
2	0	0	2	0	4	6
0	2	0	0	0	3	7
1	1	0	4	0	10	16
1	3	0	11	0	8	7
3	2	0	5	0	12	6
2	10	2	10	0	14	9
1	5	0	3	0	10	7
0	3	6	0	0	4	16
2	3	5	0	0	14	6
0	1	2	6	0	0	1
3	2	0	1	0	7	9
0	4	2	7	1	0	5
2	10	0	2	0	1	3
0	2	2	1	0	1	3
2	3	5	0	0	6	6
11	14	0	1	0	0	0
3	9	5	2	0	3	4
0	2	19	4	0	1	6
3	0	1	2	0	10	8
0	2	1	1	0	1	3
1	2	0	1	0	3	4
1	5	4	2	0	2	4
3	5	0	0	0	0	1
0	9	0	7	0	6	6
3	0	3	3	0	2	7
1	6	1	8	0	14	8
0	2	0	3	0	5	8
2	2	0	1	0	1	5
3	4	1	3	0	2	7
2	0	8	2	4	6	13
0	0	0	1	0	0	0
0	6	4	0	0	7	26
1	4	1	9	0	2	8
3	2	2	0	0	5	14
0	2	0	5	1	3	4
0	5	2	2	0	2	3

1	3	0	2	1	1	11
2	2	1	0	0	0	4
0	3	0	0	0	0	5
3	4	2	2	0	6	7
0	6	4	3	0	10	7
1	1	6	0	0	5	9
1	1	0	0	0	3	9
1	6	2	1	0	0	5
1	3	0	1	0	0	2
0	1	7	1	0	1	4
3	4	0	1	0	1	3
1	0	1	2	0	2	2
1	4	5	2	0	8	5
1	4	0	1	0	1	3
1	1	1	3	0	3	4
0	1	1	2	0	3	0
0	0	0	3	0	6	3
0	3	0	0	0	1	0
0	2	1	2	0	7	4
1	3	2	2	0	2	4
2	1	3	0	1	4	4
3	2	2	0	0	3	2
0	2	4	0	0	2	5
2	1	0	2	0	2	9
2	2	2	1	0	1	4
0	1	0	1	0	0	0
1	2	2	2	0	1	4
1	0	0	0	0	4	5
1	3	4	0	0	1	2
0	3	0	1	0	0	2
1	0	0	0	0	0	4
1	0	0	0	0	12	5
2	0	0	0	0	1	8
0	2	0	5	0	0	1
0	1	1	2	0	3	10
0	1	1	0	0	1	8
0	2	3	3	0	0	4
0	2	0	1	0	0	3
0	2	1	1	1	0	7
0	1	0	2	0	3	3
0	3	3	1	0	4	5
3	0	1	3	0	1	1
0	1	1	0	0	0	8
1	1	2	0	0	1	5
4	0	1	1	0	0	5
0	2	2	2	0	7	3
0	0	1	1	0	0	0
1	1	0	0	0	1	4
2	2	1	0	0	3	7
1	3	2	0	0	0	1
0	2	0	0	0	0	1
0	0	0	1	0	0	1
0	0	0	0	0	0	1
0	0	4	0	0	0	2
0	0	1	0	0	1	0
0	2	1	1	0	2	2
0	4	2	1	1	5	4
1	1	1	1	0	5	2
0	0	0	0	0	0	0

0	0	0	3	0	2	3
0	0	0	0	0	4	8
0	0	0	3	0	1	2
0	0	0	0	0	0	0
1	3	1	1	0	1	1
2	1	4	2	0	2	1
0	0	0	0	0	0	0
1	0	0	0	0	0	0
0	1	1	1	1	1	3
0	7	1	0	0	5	0
1	3	1	0	0	4	1
0	0	0	0	0	0	0
0	1	3	2	0	0	3
1	2	0	1	0	4	5
0	4	3	1	0	5	1
0	0	0	0	0	2	3
0	1	3	1	0	2	2
3	0	0	3	0	5	1
0	5	5	1	0	0	1
0	1	0	1	0	0	1
0	0	0	2	0	3	2
0	1	1	1	0	0	2
0	1	0	0	0	0	0
1	1	1	1	0	1	7
0	0	0	1	0	0	0
0	2	0	2	0	2	0
2	1	0	1	0	0	4
0	0	0	0	0	2	0
0	0	0	0	0	2	2
2	2	0	0	0	2	1
0	3	0	0	0	0	0
1	1	0	1	0	0	3
1	3	0	0	0	2	2
0	0	0	0	0	2	5
0	0	1	3	1	0	2
0	3	0	1	0	2	1
0	3	1	1	0	0	2
0	2	0	2	0	0	0
0	0	2	1	0	4	3
1	0	0	1	0	2	0
0	0	2	2	0	0	3
0	0	0	3	0	0	1
1	0	0	0	0	1	1
0	1	1	7	0	1	1
4	1	0	0	0	1	1
0	0	0	1	0	0	2
1	0	0	0	0	0	0
0	0	1	3	0	0	2
2	0	2	1	0	0	3
0	0	0	1	0	3	0
0	0	0	0	0	1	0
0	3	0	1	0	1	0
0	0	0	1	0	0	0
1	0	1	0	0	1	2
0	5	1	1	0	1	2
0	3	0	0	0	1	3
0	0	0	0	0	2	1
0	0	0	0	0	1	0
0	1	0	1	0	0	0

0	1	0	2	0	2	1
0	2	0	4	0	0	2
0	0	0	1	0	0	0
0	0	0	0	0	0	0
2	1	1	0	0	1	2
0	0	1	0	0	0	0
0	0	0	1	0	1	0
0	0	0	1	0	0	0
0	0	0	0	0	2	2
1	0	0	1	0	0	0
0	4	0	4	0	3	1
1	0	1	3	0	1	4
0	1	1	0	0	3	2
0	3	0	1	0	1	4
0	1	0	2	0	0	3
1	0	0	1	0	0	1
0	0	0	4	0	3	2
0	0	1	2	0	1	0
0	2	2	0	0	0	1
0	0	0	0	0	0	0
0	0	0	0	0	1	0
0	1	0	2	0	0	0
0	0	0	0	0	1	2
0	0	1	0	0	1	1
0	1	1	0	0	0	0
2	0	2	1	0	0	2
1	2	1	0	0	3	1
0	1	1	1	0	2	1
0	2	1	0	0	2	0
0	1	0	0	0	1	4
0	0	1	0	0	0	0
0	0	1	0	0	0	1
0	0	0	0	0	0	2
0	0	1	1	0	1	1
0	2	1	0	0	0	0
1	3	1	0	0	3	1
0	2	1	1	0	1	2
0	0	0	0	0	0	1
1	0	0	0	0	0	0
0	0	0	1	0	0	0
0	1	1	0	0	0	2
1	1	2	0	0	1	0
1	0	2	1	0	0	1
0	1	0	0	0	0	0
2	1	0	0	0	2	0
2	0	2	0	0	1	2
1	0	1	0	0	0	0
1	2	0	1	0	0	0
0	0	0	3	0	0	0
0	0	0	0	0	1	0
0	1	2	0	0	2	3
0	0	1	0	0	1	1
15213	44480	15209	22415	819	24126	28880
36465	78672	35131	49868	1854	37864	40552
445	408	440	428	497	419	399
CHR_NP	CHR_NP	CHR_P	CHR_P	CHR_P	CHR_P	CHR_P
CHR_NP	CHR_NP	CHR_P	CHR_P	CHR_P	CHR_P	CHR_P

64	65	66	67	68	69	70
37	38	1	2	3	4	5
CHR_NP	CHR_NP	CHR_P	CHR_P	CHR_P	CHR_P	CHR_P
G_6	G_7	A_10	A_12	A_18	A_3	A_5
488065	1421920	492624	721119	27468	816795	972175
CHR_NP	CHR_NP	CHR_P	CHR_P	CHR_P	CHR_P	CHR_P
12.94	8.98	12.54	11.66	7.89	5.48	6.88
2.18	2.95	0.50	0.60	10.22	1.42	2.04
2.03	0.83	0.66	0.34	0.92	1.48	1.78
3.21	3.43	2.49	0.63	1.50	6.97	3.38
1.99	1.84	3.15	0.55	0.65	2.66	3.02
0.70	2.27	0.36	3.85	0.55	0.86	1.34
1.92	1.94	0.32	0.70	0.79	0.91	1.80
0.11	0.34	0.29	1.11	0.14	0.18	0.26
0.54	1.97	0.38	2.22	0.11	0.28	0.63
0.52	1.00	1.85	3.58	2.20	1.53	1.40
0.65	0.62	2.07	1.08	0.82	1.10	0.83
0.46	0.43	0.55	0.40	0.25	1.09	1.11
0.50	0.63	0.65	0.27	0.17	1.27	0.87
0.50	0.50	0.51	0.42	0.29	1.61	0.72
0.12	0.25	0.75	0.18	0.23	0.39	0.34
0.25	0.09	0.38	0.07	0.01	0.12	0.14
0.06	0.21	0.08	0.18	0.11	0.23	0.45
0.08	0.11	0.39	0.18	0.42	0.57	0.60
0.19	0.25	0.11	0.42	0.15	0.11	0.30
0.15	0.15	0.21	0.18	0.94	0.12	0.24
0.10	0.09	0.14	0.11	0.18	0.14	0.21
0.14	0.17	0.33	0.27	0.20	0.42	0.37
0.07	0.11	0.09	0.14	0.15	0.04	0.12
0.04	0.10	0.06	0.14	0.13	0.08	0.31
0.07	0.25	0.11	0.13	0.37	0.06	0.19
0.07	0.10	0.10	0.18	0.03	0.07	0.12
0.13	0.12	0.36	0.07	0.07	0.37	0.15
0.09	0.04	0.40	0.13	0.25	0.11	0.12
0.08	0.13	0.10	0.09	0.15	0.27	0.18
0.12	0.10	0.07	0.10	0.10	0.07	0.10
0.11	0.09	0.06	0.07	0.05	0.09	0.18
0.19	0.16	0.15	0.02	0.02	0.41	0.19
0.05	0.08	0.04	0.14	0.01	0.07	0.13
0.02	0.01	0.05	0.12	0.04	0.02	0.07
0.07	0.12	0.07	0.06	0.10	0.19	0.12
0.09	0.10	0.05	0.11	0.24	0.63	0.19
0.05	0.09	0.07	0.15	0.59	0.12	0.13
0.06	0.11	0.07	0.17	0.49	0.07	0.14
0.07	0.04	0.01	0.02	0.18	0.04	0.09
0.16	0.08	0.18	0.02	0.01	0.18	0.07
0.02	0.02	0.04	0.03	0.14	0.14	0.18
0.06	0.04	0.14	0.02	0.01	0.08	0.10
0.05	0.06	0.04	0.05	0.08	0.16	0.13
0.01	0.03	0.04	0.04	0.10	0.03	0.02
0.03	0.03	0.06	0.06	0.13	0.06	0.09
0.05	0.03	0.04	0.03	0.04	0.06	0.11
0.06	0.04	0.01	0.04	0.02	0.04	0.06
0.05	0.03	0.11	0.02	0.03	0.05	0.09
0.01	0.02	0.02	0.01	0.00	0.04	0.03
0.01	0.01	0.10	0.02	0.01	0.00	0.01
0.03	0.01	0.01	0.03	0.02	0.05	0.06
0.01	0.02	0.01	0.02	0.04	0.03	0.05

0.03	0.02	0.02	0.05	0.08	0.05	0.04
0.01	0.01	0.14	0.02	0.06	0.02	0.07
0.04	0.05	0.02	0.02	0.00	0.00	0.00
0.00	0.00	0.02	0.01	0.00	0.00	0.00
0.03	0.04	0.01	0.04	0.03	0.04	0.05
0.01	0.02	0.02	0.01	0.01	0.02	0.05
0.02	0.02	0.03	0.02	0.05	0.03	0.05
0.04	0.01	0.02	0.01	0.08	0.04	0.05
0.02	0.01	0.02	0.03	0.02	0.07	0.06
0.01	0.01	0.04	0.01	0.01	0.01	0.02
0.00	0.01	0.00	0.04	0.01	0.00	0.01
0.00	0.00	0.00	0.00	0.00	0.00	0.00
0.03	0.02	0.07	0.01	0.04	0.10	0.03
0.01	0.02	0.01	0.03	0.02	0.04	0.04
0.01	0.01	0.01	0.03	0.00	0.01	0.01
0.01	0.02	0.02	0.01	0.01	0.02	0.02
0.00	0.01	0.01	0.01	0.01	0.02	0.03
0.01	0.01	0.01	0.04	0.14	0.02	0.02
0.02	0.01	0.01	0.01	0.03	0.02	0.02
0.01	0.01	0.03	0.01	0.01	0.08	0.03
0.01	0.01	0.02	0.02	0.02	0.01	0.02
0.01	0.02	0.03	0.02	0.01	0.01	0.01
0.01	0.01	0.00	0.01	0.01	0.01	0.03
0.01	0.00	0.01	0.01	0.01	0.03	0.04
0.02	0.03	0.01	0.01	0.00	0.00	0.00
0.03	0.01	0.00	0.01	0.13	0.00	0.01
0.02	0.01	0.03	0.00	0.00	0.03	0.02
0.01	0.00	0.00	0.01	0.02	0.01	0.01
0.01	0.02	0.01	0.02	0.00	0.03	0.03
0.01	0.00	0.02	0.00	0.00	0.01	0.01
0.01	0.01	0.00	0.00	0.03	0.04	0.02
0.00	0.00	0.00	0.02	0.00	0.00	0.00
0.01	0.01	0.00	0.01	0.02	0.00	0.01
0.00	0.00	0.01	0.01	0.00	0.01	0.01
0.01	0.00	0.00	0.01	0.01	0.02	0.01
0.00	0.01	0.01	0.02	0.00	0.01	0.02
0.01	0.01	0.00	0.01	0.01	0.01	0.02
0.01	0.01	0.01	0.00	0.01	0.02	0.02
0.01	0.01	0.02	0.00	0.01	0.01	0.01
0.00	0.00	0.00	0.00	0.00	0.00	0.00
0.01	0.00	0.01	0.00	0.00	0.00	0.01
0.00	0.01	0.00	0.01	0.01	0.00	0.01
0.00	0.00	0.00	0.01	0.00	0.00	0.00
0.00	0.00	0.01	0.00	0.00	0.00	0.01
0.01	0.00	0.01	0.00	0.01	0.01	0.01
0.01	0.00	0.02	0.00	0.01	0.01	0.01
0.01	0.00	0.01	0.00	0.02	0.01	0.01
0.00	0.00	0.00	0.00	0.00	0.00	0.00
0.00	0.00	0.00	0.01	0.00	0.02	0.01
0.01	0.01	0.00	0.00	0.00	0.00	0.01
0.01	0.00	0.01	0.00	0.00	0.01	0.01
0.01	0.01	0.01	0.00	0.02	0.01	0.01
0.00	0.01	0.00	0.01	0.00	0.01	0.01
0.00	0.00	0.02	0.01	0.00	0.01	0.01
0.00	0.00	0.00	0.00	0.01	0.00	0.01
0.00	0.00	0.00	0.00	0.01	0.00	0.01
0.00	0.00	0.00	0.01	0.00	0.01	0.01
0.00	0.01	0.01	0.00	0.00	0.00	0.00
0.00	0.00	0.01	0.00	0.00	0.00	0.01

0.00	0.00	0.01	0.00	0.00	0.01	0.01
0.00	0.00	0.00	0.00	0.00	0.01	0.01
0.00	0.01	0.00	0.01	0.01	0.01	0.01
0.00	0.00	0.00	0.00	0.00	0.00	0.01
0.00	0.00	0.01	0.00	0.01	0.01	0.01
0.00	0.01	0.01	0.00	0.00	0.00	0.01
0.00	0.00	0.00	0.00	0.00	0.01	0.01
0.00	0.00	0.00	0.00	0.00	0.01	0.01
0.00	0.00	0.00	0.01	0.00	0.01	0.01
0.00	0.00	0.00	0.01	0.00	0.00	0.00
0.00	0.00	0.01	0.00	0.00	0.00	0.01
0.00	0.00	0.00	0.00	0.00	0.00	0.00
0.00	0.00	0.01	0.00	0.01	0.00	0.00
0.00	0.00	0.00	0.00	0.00	0.00	0.00
0.00	0.00	0.00	0.00	0.00	0.01	0.01
0.00	0.00	0.00	0.00	0.00	0.00	0.00
0.00	0.00	0.00	0.00	0.00	0.00	0.00
0.00	0.00	0.00	0.00	0.01	0.01	0.01
0.00	0.00	0.00	0.00	0.00	0.00	0.01
0.01	0.01	0.00	0.00	0.00	0.00	0.00
0.00	0.00	0.01	0.00	0.00	0.00	0.01
0.00	0.00	0.00	0.00	0.00	0.00	0.00
0.00	0.00	0.00	0.00	0.00	0.00	0.00
0.00	0.00	0.00	0.01	0.00	0.00	0.00
0.00	0.00	0.00	0.00	0.00	0.00	0.00
0.00	0.00	0.01	0.00	0.00	0.00	0.00
0.00	0.00	0.00	0.00	0.00	0.00	0.00
0.00	0.00	0.00	0.00	0.00	0.00	0.00
0.00	0.00	0.00	0.00	0.00	0.00	0.00
0.00	0.00	0.00	0.00	0.00	0.00	0.00
0.00	0.00	0.00	0.00	0.00	0.00	0.00
0.00	0.00	0.01	0.00	0.00	0.01	0.00
0.00	0.00	0.00	0.00	0.00	0.00	0.01
0.00	0.00	0.00	0.00	0.00	0.00	0.00
0.00	0.00	0.00	0.00	0.00	0.01	0.00
0.00	0.00	0.00	0.00	0.00	0.00	0.01
0.00	0.00	0.00	0.00	0.00	0.00	0.00
0.00	0.00	0.00	0.00	0.00	0.01	0.00
0.00	0.00	0.00	0.00	0.00	0.01	0.00
0.00	0.00	0.00	0.00	0.00	0.00	0.00
0.00	0.00	0.00	0.00	0.00	0.00	0.00
0.00	0.00	0.00	0.00	0.00	0.00	0.00
0.00	0.00	0.00	0.00	0.00	0.00	0.00
0.00	0.00	0.01	0.00	0.00	0.00	0.00
0.00	0.00	0.00	0.00	0.00	0.00	0.00
0.00	0.00	0.00	0.00	0.00	0.00	0.00
0.00	0.00	0.00	0.00	0.00	0.00	0.00
0.00	0.00	0.00	0.00	0.00	0.00	0.00
0.00	0.00	0.00	0.00	0.00	0.00	0.00
0.00	0.00	0.00	0.00	0.00	0.00	0.00
0.00	0.00	0.00	0.00	0.00	0.00	0.00
0.00	0.00	0.00	0.00	0.00	0.00	0.00
0.00	0.00	0.00	0.00	0.00	0.00	0.00
0.00	0.00	0.00	0.00	0.00	0.00	0.00
0.00	0.00	0.00	0.00	0.00	0.00	0.00
0.00	0.00	0.00	0.00	0.00	0.00	0.00
0.00	0.00	0.00	0.01	0.00	0.00	0.00
0.00	0.00	0.00	0.00	0.00	0.00	0.00
0.00	0.00	0.00	0.00	0.00	0.00	0.00

0.00	0.00	0.00	0.00	0.00	0.00	0.00
0.00	0.00	0.00	0.00	0.00	0.00	0.00
0.00	0.00	0.00	0.00	0.00	0.00	0.00
0.00	0.00	0.00	0.00	0.00	0.00	0.00
0.00	0.00	0.00	0.00	0.00	0.00	0.00
0.00	0.00	0.00	0.00	0.00	0.00	0.00
0.00	0.00	0.00	0.00	0.00	0.00	0.00
0.00	0.00	0.00	0.00	0.00	0.00	0.00
0.00	0.00	0.00	0.00	0.00	0.00	0.00
0.00	0.00	0.00	0.00	0.00	0.00	0.00
0.00	0.00	0.00	0.00	0.00	0.00	0.00
0.00	0.00	0.00	0.00	0.00	0.00	0.00
0.00	0.00	0.00	0.00	0.00	0.00	0.00
0.00	0.00	0.00	0.00	0.00	0.00	0.00
0.00	0.00	0.00	0.00	0.01	0.00	0.00
0.00	0.00	0.00	0.00	0.00	0.00	0.00
0.00	0.00	0.00	0.00	0.00	0.00	0.00
0.00	0.00	0.00	0.00	0.00	0.00	0.00
0.00	0.00	0.00	0.00	0.00	0.00	0.00
0.00	0.00	0.00	0.00	0.00	0.00	0.00
0.01	0.00	0.00	0.00	0.00	0.00	0.00
0.00	0.00	0.00	0.00	0.00	0.00	0.00
0.00	0.00	0.00	0.00	0.00	0.00	0.00
0.00	0.00	0.00	0.00	0.00	0.00	0.00
0.00	0.00	0.00	0.00	0.00	0.00	0.00
0.00	0.00	0.00	0.00	0.00	0.00	0.00
0.00	0.00	0.00	0.00	0.00	0.00	0.00
0.00	0.00	0.00	0.00	0.00	0.00	0.00
0.00	0.00	0.00	0.00	0.00	0.00	0.00
0.00	0.00	0.00	0.00	0.00	0.00	0.00
0.00	0.00	0.00	0.00	0.00	0.00	0.00
0.00	0.00	0.00	0.00	0.00	0.00	0.00
0.00	0.00	0.00	0.00	0.00	0.00	0.00
0.00	0.00	0.00	0.00	0.00	0.00	0.00
0.00	0.00	0.00	0.00	0.00	0.00	0.00
0.00	0.00	0.00	0.00	0.00	0.00	0.00
0.00	0.00	0.00	0.00	0.00	0.00	0.00
0.00	0.00	0.00	0.00	0.00	0.00	0.00
0.00	0.00	0.00	0.00	0.00	0.00	0.00
0.00	0.00	0.00	0.00	0.00	0.00	0.00
0.00	0.00	0.00	0.00	0.00	0.00	0.00
0.00	0.00	0.00	0.00	0.00	0.00	0.00
0.00	0.00	0.00	0.00	0.00	0.00	0.00
0.00	0.00	0.00	0.00	0.00	0.00	0.00
0.00	0.00	0.00	0.00	0.00	0.00	0.00
0.00	0.00	0.00	0.00	0.00	0.00	0.00
0.00	0.00	0.00	0.00	0.00	0.00	0.00
0.00	0.00	0.00	0.00	0.00	0.00	0.00
0.00	0.00	0.00	0.00	0.00	0.00	0.00
0.00	0.00	0.00	0.00	0.00	0.00	0.00
0.00	0.00	0.00	0.00	0.00	0.00	0.00
0.00	0.00	0.00	0.00	0.00	0.00	0.00
0.00	0.00	0.00	0.00	0.00	0.00	0.00
0.00	0.00	0.00	0.00	0.00	0.00	0.00
0.00	0.00	0.00	0.00	0.00	0.00	0.00
0.00	0.00	0.00	0.00	0.00	0.00	0.00
0.00	0.00	0.00	0.00	0.00	0.00	0.00
0.00	0.00	0.00	0.00	0.00	0.00	0.00
0.00	0.00	0.00	0.00	0.00	0.00	0.00

0.00	0.00	0.00	0.00	0.00	0.00	0.00
0.00	0.00	0.00	0.00	0.00	0.00	0.00
0.00	0.00	0.00	0.00	0.00	0.00	0.00
0.00	0.00	0.00	0.00	0.00	0.00	0.00
0.00	0.00	0.00	0.00	0.00	0.00	0.00
0.00	0.00	0.00	0.00	0.00	0.00	0.00
0.00	0.00	0.00	0.00	0.00	0.00	0.00
0.00	0.00	0.00	0.00	0.00	0.00	0.00
0.00	0.00	0.00	0.00	0.00	0.00	0.00
0.00	0.00	0.00	0.00	0.00	0.00	0.00
0.00	0.00	0.00	0.00	0.00	0.00	0.00
0.00	0.00	0.00	0.00	0.00	0.00	0.00
0.00	0.00	0.00	0.00	0.00	0.00	0.00
0.00	0.00	0.00	0.00	0.00	0.00	0.00
0.00	0.00	0.00	0.00	0.00	0.00	0.00
0.00	0.00	0.00	0.00	0.00	0.00	0.00
0.00	0.00	0.00	0.00	0.00	0.00	0.00
0.00	0.00	0.00	0.00	0.00	0.00	0.00
0.00	0.00	0.00	0.00	0.00	0.00	0.00
0.00	0.00	0.00	0.00	0.00	0.00	0.00
0.00	0.00	0.00	0.00	0.00	0.00	0.00
0.00	0.00	0.00	0.00	0.00	0.00	0.00
0.00	0.00	0.00	0.00	0.00	0.00	0.00
0.00	0.00	0.00	0.00	0.00	0.00	0.00
0.00	0.00	0.00	0.00	0.00	0.00	0.00
0.00	0.00	0.00	0.00	0.00	0.00	0.00
0.00	0.00	0.00	0.00	0.00	0.00	0.00
0.00	0.00	0.00	0.00	0.00	0.00	0.00
0.00	0.00	0.00	0.00	0.00	0.00	0.00
0.00	0.00	0.00	0.00	0.00	0.00	0.00

64	65	66	67	68	69	70
37 CHR_NP G_6 488065	38 CHR_NP G_7 1421920	1 CHR_P A_10 492624	2 CHR_P A_12 721119	3 CHR_P A_18 27468	4 CHR_P A_3 816795	5 CHR_P A_5 972175
G_6	G_7	A_10	A_12	A_18	A_3	A_5
0.30	-0.59	0.21	0.01	-0.84	-1.38	-1.06
-0.32	-0.10	-0.81	-0.78	2.04	-0.54	-0.36
-0.06	-0.96	-1.09	-1.33	-0.90	-0.48	-0.25
0.75	0.97	0.04	-1.79	-0.94	4.47	0.92
-0.19	-0.36	1.09	-1.78	-1.67	0.55	0.95
-0.45	0.97	-0.75	2.41	-0.58	-0.30	0.13
1.20	1.23	-1.35	-0.74	-0.60	-0.40	1.01
-0.41	0.00	-0.09	1.39	-0.36	-0.28	-0.15

-0.10	2.74	-0.41	3.23	-0.96	-0.62	0.07
-0.60	0.61	2.79	7.24	3.69	1.97	1.63
0.66	0.58	6.00	2.28	1.32	2.35	1.34
-0.30	-0.57	0.49	-0.81	-2.17	5.34	5.55
0.82	1.59	1.66	-0.52	-1.08	5.24	2.97
0.18	0.18	0.24	-0.27	-1.02	6.59	1.48
-0.46	0.15	2.44	-0.19	0.05	0.77	0.55
-0.19	-0.88	0.38	-0.97	-1.25	-0.76	-0.70
-0.61	0.33	-0.48	0.16	-0.29	0.44	1.84
-1.29	-1.08	1.76	-0.36	2.03	3.48	3.75
0.08	0.59	-0.68	2.16	-0.32	-0.64	1.01
-0.79	-0.82	-0.20	-0.48	7.04	-1.10	0.12
-0.94	-1.03	-0.22	-0.75	0.40	-0.30	0.84
-0.18	0.28	2.75	1.74	0.80	4.05	3.41
-0.58	-0.11	-0.38	0.26	0.43	-1.02	0.07
-0.72	-0.19	-0.59	0.24	0.13	-0.34	1.83
-0.38	0.69	-0.14	0.02	1.40	-0.39	0.36
-0.93	-0.22	-0.29	1.31	-1.66	-0.87	0.11
-0.15	-0.31	3.48	-1.09	-1.07	3.76	0.22
-0.24	-0.66	2.73	0.19	1.31	0.00	0.08
-0.50	0.45	-0.08	-0.38	0.86	3.26	1.33
0.48	-0.09	-0.83	0.00	0.07	-0.84	-0.06
0.38	0.06	-0.83	-0.50	-1.06	0.01	2.01
1.27	0.68	0.49	-1.92	-1.94	5.63	1.24
-0.12	0.33	-0.36	1.24	-0.73	0.15	1.06
-0.28	-0.33	-0.14	0.24	-0.19	-0.28	-0.02
-0.45	0.50	-0.48	-0.80	0.23	2.18	0.59
-0.30	-0.25	-0.55	-0.20	0.56	2.96	0.29
-0.81	-0.22	-0.51	0.44	5.63	0.04	0.19
-0.31	0.20	-0.22	0.76	4.06	-0.19	0.44
-0.39	-0.71	-1.05	-0.94	0.93	-0.74	-0.12
0.45	-0.16	0.66	-0.63	-0.69	0.67	-0.22
-1.09	-1.18	-0.80	-0.90	1.29	1.37	2.17
-0.23	-0.73	2.04	-1.60	-1.70	0.20	0.98
-0.49	-0.17	-0.99	-0.77	0.51	3.77	2.80
-0.59	-0.16	0.07	0.08	1.46	-0.20	-0.32
-0.61	-0.63	0.03	-0.06	1.34	-0.06	0.49
0.12	-1.15	-0.33	-0.94	-0.36	0.79	4.54
0.84	0.13	-1.11	0.16	-0.86	-0.21	0.68
-0.15	-0.72	2.07	-0.88	-0.68	0.17	1.20
0.03	0.15	0.24	-0.21	-0.36	1.03	0.48
-0.50	-0.46	1.64	-0.33	-0.47	-0.65	-0.37
-0.22	-1.58	-1.59	-0.31	-0.99	0.71	1.52
-0.61	-0.46	-0.68	-0.34	0.10	-0.20	0.60
-0.76	-0.83	-1.46	1.19	3.19	0.96	0.63
-1.16	-0.93	5.98	-0.63	1.81	-0.39	2.28
1.03	1.13	-0.44	-0.44	-1.38	-1.36	-1.30

-0.27	-0.24	0.58	0.08	-0.23	-0.31	-0.20
-0.09	0.87	-1.72	0.94	0.10	0.88	1.67
-1.02	-0.54	-0.60	-1.08	-1.28	-0.73	1.85
-0.43	-0.98	-0.21	-0.92	1.08	0.26	1.59
-0.08	-0.75	-0.53	-0.90	1.04	-0.07	0.14
-0.42	-1.11	-0.76	0.10	-0.41	3.52	3.03
-0.87	-0.77	1.27	-0.81	-1.22	-0.99	-0.01
-0.52	-0.07	-0.57	2.04	0.13	-0.42	-0.17
-0.25	-0.21	-0.28	-0.17	-0.24	-0.28	-0.24
0.19	-0.56	2.38	-1.19	0.48	4.06	0.04
-0.19	0.78	-0.50	1.62	0.64	2.45	1.86
-0.08	-0.32	-0.32	1.20	-0.62	-0.28	0.07
-0.33	0.64	0.84	0.43	-0.31	1.20	1.19
-0.93	-0.34	-0.29	0.11	-0.34	0.53	2.33
-0.25	-0.24	-0.32	0.08	1.32	-0.22	-0.16
-0.25	-1.02	-1.12	-0.55	0.29	0.05	-0.18
-1.00	-1.29	0.75	-0.74	-0.93	5.61	1.25
-0.34	0.17	0.72	0.99	0.88	0.19	0.57
-0.31	0.62	1.70	1.27	-0.54	-0.18	-0.29
0.23	0.01	-0.88	-0.02	-0.31	-0.20	2.06
-0.20	-1.20	-0.30	-0.76	-0.92	1.12	1.53
1.22	1.34	-0.27	-0.20	-1.76	-1.68	-1.58
0.16	-0.18	-0.49	-0.38	2.88	-0.44	-0.33
-0.09	-0.88	0.99	-1.41	-1.39	0.89	0.34
-0.89	-1.09	-1.24	-0.42	0.33	-0.04	-0.53
-0.59	0.92	-0.61	1.21	-1.21	3.19	4.29
-0.74	-1.20	1.55	-1.05	-1.76	-0.92	-0.17
-0.20	-0.88	-1.18	-1.17	1.63	2.55	0.51
-0.32	-0.45	-0.37	0.37	-0.34	-0.45	-0.29
-0.22	0.57	-0.64	0.46	1.34	-0.65	0.81
-0.68	-0.96	-0.48	-0.27	-1.55	-0.17	0.57
-0.53	-1.78	-1.71	-1.60	-0.19	0.41	0.18
-0.53	1.12	0.60	1.95	-0.81	0.93	1.66
-0.86	-0.26	-0.98	1.01	-0.60	0.88	3.09
-0.08	-0.74	0.02	-1.26	-0.56	0.65	0.41
-0.72	-0.58	2.73	-1.55	-0.16	-0.08	0.03
-0.19	-0.14	-0.24	-0.17	-0.32	-0.17	-0.26
-0.04	-0.73	-0.21	-0.45	-1.44	-0.76	1.20
-0.27	0.34	-0.63	1.26	0.32	-0.49	0.34
-0.39	-0.18	-0.25	0.65	-0.70	-0.36	0.01
-0.79	-0.65	-0.01	-0.33	-0.22	-0.28	1.22
-0.29	-1.23	-0.44	-1.61	-0.14	-0.42	-0.11
-0.26	-1.46	3.41	-1.66	2.48	1.23	1.63
0.05	-0.63	0.13	-1.24	2.62	1.64	1.60
-0.48	0.21	-0.53	0.14	-0.81	-0.44	-0.29
-0.65	-0.63	-0.61	0.83	-0.59	2.93	1.23
0.35	1.18	-1.80	-0.14	-1.52	-0.46	0.53
0.77	-0.82	-0.42	-0.86	-1.35	0.17	0.06

-0.26	-0.27	-0.11	-0.45	1.23	-0.01	0.18
-0.55	0.15	-0.94	2.71	0.09	0.49	1.91
-0.52	0.22	4.09	2.19	0.08	0.48	0.67
-0.48	-1.15	-1.28	-0.75	1.31	-0.23	0.81
-0.54	-0.31	-0.85	-0.41	0.28	-0.46	-0.17
-0.30	0.11	-0.55	0.85	-1.19	0.83	1.04
0.28	1.56	0.65	0.23	-1.34	-0.07	0.40
-0.82	-0.28	1.43	-0.60	0.23	-0.52	1.40
-0.68	-1.21	0.88	-1.29	-0.52	1.54	2.17
-0.49	-0.63	-0.36	-0.18	-0.55	1.38	1.99
-0.32	1.52	-0.21	0.94	2.11	2.51	3.05
0.04	-0.94	-0.96	-1.25	-0.11	-0.71	0.41
0.07	-0.19	0.78	-1.28	1.60	2.96	1.34
-0.82	4.46	4.44	-0.61	0.30	0.11	1.46
0.18	-0.36	-0.55	0.55	-0.84	0.80	1.87
0.18	-0.36	-0.55	0.55	-0.84	0.80	1.87
-0.39	-0.66	-0.53	0.23	0.15	1.22	0.67
-0.45	-0.18	-0.12	3.06	-0.40	-0.07	-0.06
-1.33	-1.15	1.76	-0.12	0.18	-0.11	1.86
0.04	-0.70	-0.44	0.15	-0.52	-0.74	-0.06
-0.77	-0.51	1.76	-0.11	1.52	-0.71	0.51
-0.18	-0.24	-0.37	-0.34	-0.39	-0.36	-0.33
0.21	-1.06	-1.13	-1.09	-0.61	1.45	1.66
0.27	-0.01	-1.20	0.20	-0.18	-0.47	-0.17
-0.37	-1.07	-0.50	-0.74	-1.52	-0.06	0.54
-0.92	-0.98	-0.65	-0.65	0.99	1.32	0.81
-0.38	-0.37	0.15	-0.77	0.01	-0.15	0.44
3.01	2.10	-0.67	-0.38	-0.25	-0.69	-0.68
0.43	-0.83	2.28	-0.05	0.18	0.67	4.25
-0.37	-0.17	-0.46	0.36	0.19	-0.48	0.55
-0.90	-0.84	-0.82	-0.30	0.82	0.27	0.46
0.08	0.00	-0.26	2.98	-0.86	-0.36	1.70
-1.26	-0.81	1.10	-1.15	-2.01	-0.96	0.96

27	28	29	30	31	32	33
63	64	65	66	67	68	69
AGCTACATTGTCT	TGAGACCTCTGGG	CATTATTACTTTT	CATAAAGTAGAAA	GCCCTGACCTGTC	CAAAGTGCTTACA	TGTAAACATCCT
miR-221-3p	miR-769-5p	miR-126-5p	miR-142-5p	miR-4732-3p	miR-17-5p	miR-30b-5p
106297	102201	96743	94911	93386	87898	82798
1.65	0.08	-0.60	-0.26	-0.14	-0.65	0.64
0.31	-0.65	-0.11	-0.08	-0.42	-0.98	-0.89
-0.84	-0.69	-0.29	-0.26	-0.06	1.93	-0.45
-0.94	-1.15	-0.52	-0.27	0.11	-0.36	-0.52
0.52	-0.42	-0.31	-0.21	-1.30	1.12	-0.18
-0.54	-0.62	-0.50	-0.24	1.41	-1.04	-0.72
1.79	-0.13	-0.60	-0.26	-0.02	-0.73	-0.47
-0.56	0.09	-0.56	-0.17	2.11	-0.97	-0.61
-1.23	-1.16	-0.40	-0.26	1.34	-0.55	-0.71
-0.55	-0.45	0.18	-0.22	0.03	0.48	0.46
-0.30	0.18	1.28	-0.16	-0.98	0.08	-0.11
-0.94	0.09	-0.60	-0.28	1.69	-1.29	-0.47
3.07	2.44	1.97	0.10	-1.19	1.55	3.84
-0.68	-0.22	-0.59	-0.25	0.59	-0.96	-0.66
-0.54	-0.04	-0.28	-0.08	-0.39	0.96	-0.42
0.93	0.74	1.18	-0.06	-0.45	1.19	0.82
-0.19	0.39	-0.62	-0.21	-0.80	-0.45	-0.28
0.02	0.12	-0.25	-0.22	0.05	1.49	-0.07
-0.90	3.16	-0.25	-0.23	-1.28	-0.30	-0.06
-0.57	1.19	2.98	4.98	-0.66	1.17	2.06

-0.30	-0.94	-0.48	-0.23	1.94	-0.47	-0.40
0.08	0.33	-0.68	-0.27	-0.25	-0.99	-0.53
0.06	-0.12	-0.64	-0.24	0.23	-0.61	-0.29
-0.45	-0.40	-0.67	-0.23	0.88	-0.88	-0.41
0.26	-1.44	-0.39	-0.21	-0.36	-0.52	-0.63
1.36	0.10	-0.49	-0.21	-1.42	0.19	0.17
-0.50	-0.47	2.24	0.03	-0.64	1.59	0.87
-0.64	-0.76	19.45	7.70	-1.17	2.58	0.85
-0.67	0.07	0.31	-0.16	-0.95	0.06	-0.31
-1.18	-0.53	-0.05	-0.25	-0.05	-0.07	-0.67
-1.04	-1.44	-0.50	-0.24	7.78	0.18	-0.80
-0.42	0.31	2.09	-0.20	-0.09	0.63	-0.05
1.37	-0.67	0.71	-0.11	-1.13	2.62	0.13
0.55	0.43	-0.59	-0.23	-0.09	-0.55	-0.38
0.88	0.95	-0.38	-0.17	-0.58	-0.28	1.87
0.82	0.53	-0.50	-0.08	-1.24	-0.91	-0.45
-1.51	-1.57	-0.65	-0.25	4.89	-1.42	-0.81
1.56	1.14	-0.22	-0.13	-1.26	-0.27	2.90
4.18	2.86	2.40	-0.06	-1.41	1.75	4.35
-0.23	1.21	-0.54	-0.22	-1.23	-0.76	-0.04
2.33	1.32	-0.70	-0.24	-1.06	-0.81	-0.28
13.01	8.27	-0.51	-0.19	-0.73	-0.85	0.25
1.12	-0.57	-0.32	-0.16	-0.71	-0.59	-0.60
0.00	1.53	0.13	-0.19	-0.78	0.82	0.95
-0.76	0.09	-0.52	-0.22	0.04	-0.95	-0.57
-0.23	-0.27	-0.61	-0.16	-0.45	-1.02	-0.70
-0.19	0.95	-0.19	0.07	-1.32	0.36	0.35
0.68	0.98	-0.63	-0.20	-0.19	-0.69	-0.02
-0.15	0.07	-0.20	-0.17	0.17	0.02	0.42
-0.54	1.14	-0.62	-0.13	-0.62	-0.34	0.03
0.79	0.41	-0.07	-0.14	-0.73	0.59	1.25
1.99	-0.89	-0.54	-0.20	-1.05	-0.65	-0.43
0.82	-1.00	-0.54	-0.24	-0.28	-0.30	-0.61
1.13	-0.07	-0.54	-0.24	-1.16	0.61	0.11
-0.32	-1.02	-0.60	-0.24	0.47	-1.04	-0.57
-0.34	-0.80	-0.52	-0.25	-0.70	-0.77	-0.61
-0.15	-1.04	-0.24	-0.24	0.53	-0.41	-0.07
-0.98	0.42	11.46	4.81	-1.25	2.52	3.23
0.08	-0.17	-0.61	-0.25	-0.68	-0.15	-0.36
2.72	0.15	-0.29	-0.19	-0.43	0.98	0.66
2.01	-0.50	-0.36	-0.06	-0.93	1.64	0.37
-1.28	-0.47	8.89	3.55	-0.96	3.12	1.93
-0.74	-0.43	-0.51	-0.24	0.32	-0.75	-0.48
-0.42	-0.87	-0.52	-0.25	0.19	-0.19	-0.08
-1.11	-0.77	-0.07	-0.21	-0.56	0.78	-0.32
-0.76	1.27	-0.57	-0.28	2.38	-0.50	-0.32
0.10	-0.81	2.04	-0.17	-1.19	1.62	1.20
-0.41	-1.22	0.13	-0.24	0.48	0.64	-0.62
3.52	-0.99	-0.42	-0.28	4.06	2.45	-0.28
3.03	-0.01	-0.17	-0.24	0.04	1.86	0.07
0.21	-0.84	-0.51	-0.26	1.30	-0.29	-0.52
-1.21	-0.68	-0.07	-0.01	0.30	0.21	1.79
-0.72	-1.01	-0.15	-0.24	-0.10	0.02	-0.01
0.42	0.80	0.17	-0.24	0.01	0.45	1.35
3.88	2.31	-0.60	-0.25	-0.21	-1.55	-0.72
-0.54	0.22	-0.02	-0.22	-0.25	0.34	0.19
-0.99	1.40	-0.23	-0.24	-1.25	-0.02	-0.02
-1.31	-0.11	0.18	-0.24	-0.24	-1.13	-0.59
0.51	0.88	0.75	0.12	-1.36	2.84	1.60

-0.38	0.65	0.03	-0.04	-1.38	0.39	0.18
-0.86	-0.20	-0.42	-0.13	-0.68	-0.62	-0.58
-0.25	-0.18	-0.32	-0.06	-0.92	-0.06	0.03
-0.95	0.59	-0.64	-0.18	-0.47	-0.81	0.58
-0.03	1.82	4.01	6.06	-1.30	0.80	5.08
-1.29	-0.22	6.09	8.26	-1.03	2.14	3.34
0.03	0.52	-0.51	-0.19	-0.37	-0.49	0.03
-0.08	0.53	-0.48	-0.18	-1.06	-0.18	0.30
-0.44	-0.77	-0.50	-0.20	-0.09	-0.53	-0.09
-0.52	1.02	-0.18	-0.23	-0.96	0.73	0.61
-0.15	-0.11	-0.48	-0.21	-0.83	-0.18	-0.15
-1.14	0.80	-0.21	-0.19	-0.30	0.12	-0.30
-0.89	-0.90	-0.58	-0.25	-0.03	-1.01	-0.58
-0.26	-0.05	-0.63	-0.21	-0.46	-0.82	-0.30
-0.49	0.09	-0.09	-0.24	-0.70	0.19	-0.45
0.47	-0.61	0.29	-0.22	-0.83	0.20	2.50
1	0	1	-1	1	0	-1
0.00	0.00	0.00	0.00	0.00	0.00	0.00
1.00	1.00	1.00	1.00	1.00	1.00	1.00
0.15	0.82	0.35	0.88	0.96	0.76	0.75

61	62	63	64	65	66	67
AGCTACATTGTCT	TGAGACCTCTGGG	CATTATTACTTTT	CATAAAGTAGAAA	GCCCTGACCTGTC	CAAAGTGCTTACA	TGTAAACATCCT
miR-221-3p	miR-769-5p	miR-126-5p	miR-142-5p	miR-4732-3p	miR-17-5p	miR-30b-5p
106297	102201	96743	94911	93386	87898	82798
1.65	0.08	-0.60	-0.26	-0.14	-0.65	0.64
0.31	-0.65	-0.11	-0.08	-0.42	-0.98	-0.89
-0.84	-0.69	-0.29	-0.26	-0.06	1.93	-0.45
-0.94	-1.15	-0.52	-0.27	0.11	-0.36	-0.52
0.52	-0.42	-0.31	-0.21	-1.30	1.12	-0.18
-0.54	-0.62	-0.50	-0.24	1.41	-1.04	-0.72
1.79	-0.13	-0.60	-0.26	-0.02	-0.73	-0.47
-0.56	0.09	-0.56	-0.17	2.11	-0.97	-0.61
-1.23	-1.16	-0.40	-0.26	1.34	-0.55	-0.71
-0.55	-0.45	0.18	-0.22	0.03	0.48	0.46
-0.30	0.18	1.28	-0.16	-0.98	0.08	-0.11
-0.94	0.09	-0.60	-0.28	1.69	-1.29	-0.47
3.07	2.44	1.97	0.10	-1.19	1.55	3.84
-0.68	-0.22	-0.59	-0.25	0.59	-0.96	-0.66
-0.54	-0.04	-0.28	-0.08	-0.39	0.96	-0.42
0.93	0.74	1.18	-0.06	-0.45	1.19	0.82
-0.19	0.39	-0.62	-0.21	-0.80	-0.45	-0.28
0.02	0.12	-0.25	-0.22	0.05	1.49	-0.07
-0.90	3.16	-0.25	-0.23	-1.28	-0.30	-0.06
-0.57	1.19	2.98	4.98	-0.66	1.17	2.06
-0.30	-0.94	-0.48	-0.23	1.94	-0.47	-0.40
0.08	0.33	-0.68	-0.27	-0.25	-0.99	-0.53
0.06	-0.12	-0.64	-0.24	0.23	-0.61	-0.29
-0.45	-0.40	-0.67	-0.23	0.88	-0.88	-0.41
0.26	-1.44	-0.39	-0.21	-0.36	-0.52	-0.63
1.36	0.10	-0.49	-0.21	-1.42	0.19	0.17
-0.50	-0.47	2.24	0.03	-0.64	1.59	0.87
-0.64	-0.76	19.45	7.70	-1.17	2.58	0.85

-0.67	0.07	0.31	-0.16	-0.95	0.06	-0.31
-1.18	-0.53	-0.05	-0.25	-0.05	-0.07	-0.67
-1.04	-1.44	-0.50	-0.24	7.78	0.18	-0.80
-0.42	0.31	2.09	-0.20	-0.09	0.63	-0.05
1.37	-0.67	0.71	-0.11	-1.13	2.62	0.13
0.55	0.43	-0.59	-0.23	-0.09	-0.55	-0.38
0.88	0.95	-0.38	-0.17	-0.58	-0.28	1.87
0.82	0.53	-0.50	-0.08	-1.24	-0.91	-0.45
-1.51	-1.57	-0.65	-0.25	4.89	-1.42	-0.81
1.56	1.14	-0.22	-0.13	-1.26	-0.27	2.90
4.18	2.86	2.40	-0.06	-1.41	1.75	4.35
-0.23	1.21	-0.54	-0.22	-1.23	-0.76	-0.04
2.33	1.32	-0.70	-0.24	-1.06	-0.81	-0.28
13.01	8.27	-0.51	-0.19	-0.73	-0.85	0.25
1.12	-0.57	-0.32	-0.16	-0.71	-0.59	-0.60
0.00	1.53	0.13	-0.19	-0.78	0.82	0.95
-0.76	0.09	-0.52	-0.22	0.04	-0.95	-0.57
-0.23	-0.27	-0.61	-0.16	-0.45	-1.02	-0.70
-0.19	0.95	-0.19	0.07	-1.32	0.36	0.35
0.68	0.98	-0.63	-0.20	-0.19	-0.69	-0.02
-0.15	0.07	-0.20	-0.17	0.17	0.02	0.42
-0.54	1.14	-0.62	-0.13	-0.62	-0.34	0.03
0.79	0.41	-0.07	-0.14	-0.73	0.59	1.25
1.99	-0.89	-0.54	-0.20	-1.05	-0.65	-0.43
0.82	-1.00	-0.54	-0.24	-0.28	-0.30	-0.61
1.13	-0.07	-0.54	-0.24	-1.16	0.61	0.11
-0.32	-1.02	-0.60	-0.24	0.47	-1.04	-0.57
-0.34	-0.80	-0.52	-0.25	-0.70	-0.77	-0.61
-0.15	-1.04	-0.24	-0.24	0.53	-0.41	-0.07
-0.98	0.42	11.46	4.81	-1.25	2.52	3.23
0.08	-0.17	-0.61	-0.25	-0.68	-0.15	-0.36
2.72	0.15	-0.29	-0.19	-0.43	0.98	0.66
2.01	-0.50	-0.36	-0.06	-0.93	1.64	0.37
-1.28	-0.47	8.89	3.55	-0.96	3.12	1.93
-0.74	-0.43	-0.51	-0.24	0.32	-0.75	-0.48
-0.42	-0.87	-0.52	-0.25	0.19	-0.19	-0.08
-1.11	-0.77	-0.07	-0.21	-0.56	0.78	-0.32
-0.76	1.27	-0.57	-0.28	2.38	-0.50	-0.32
0.10	-0.81	2.04	-0.17	-1.19	1.62	1.20
-0.41	-1.22	0.13	-0.24	0.48	0.64	-0.62
3.52	-0.99	-0.42	-0.28	4.06	2.45	-0.28
3.03	-0.01	-0.17	-0.24	0.04	1.86	0.07
0.21	-0.84	-0.51	-0.26	1.30	-0.29	-0.52
-1.21	-0.68	-0.07	-0.01	0.30	0.21	1.79
-0.72	-1.01	-0.15	-0.24	-0.10	0.02	-0.01
0.42	0.80	0.17	-0.24	0.01	0.45	1.35
3.88	2.31	-0.60	-0.25	-0.21	-1.55	-0.72
-0.54	0.22	-0.02	-0.22	-0.25	0.34	0.19
-0.99	1.40	-0.23	-0.24	-1.25	-0.02	-0.02
-1.31	-0.11	0.18	-0.24	-0.24	-1.13	-0.59
0.51	0.88	0.75	0.12	-1.36	2.84	1.60
-0.38	0.65	0.03	-0.04	-1.38	0.39	0.18
-0.86	-0.20	-0.42	-0.13	-0.68	-0.62	-0.58
-0.25	-0.18	-0.32	-0.06	-0.92	-0.06	0.03
-0.95	0.59	-0.64	-0.18	-0.47	-0.81	0.58
-0.03	1.82	4.01	6.06	-1.30	0.80	5.08
-1.29	-0.22	6.09	8.26	-1.03	2.14	3.34
0.03	0.52	-0.51	-0.19	-0.37	-0.49	0.03
-0.08	0.53	-0.48	-0.18	-1.06	-0.18	0.30

-0.44	-0.77	-0.50	-0.20	-0.09	-0.53	-0.09
-0.52	1.02	-0.18	-0.23	-0.96	0.73	0.61
-0.15	-0.11	-0.48	-0.21	-0.83	-0.18	-0.15
-1.14	0.80	-0.21	-0.19	-0.30	0.12	-0.30
-0.89	-0.90	-0.58	-0.25	-0.03	-1.01	-0.58
-0.26	-0.05	-0.63	-0.21	-0.46	-0.82	-0.30
-0.49	0.09	-0.09	-0.24	-0.70	0.19	-0.45
0	-1	0	0	-1	0	3

61	62	63	64	65	66	67
91	90	19	82	66	11	114
ACCACTGACCGTT	TGAGGTAGGAGGT	CAAAGAATTCTCC	ACTCGGCGTGGCC	CAAAGTGCTTACA	TGAGGTAGTAGTT	CAAAGTGCTCAT
miR-181a-2-3p	let-7e-5p	miR-186-5p	miR-1307-3p	miR-17-5p	let-7i-5p	miR-20b-5p
36605	37943	979581	51847	87898	2186845	19721
-0.46	-0.64	0.09	0.64	-0.65	1.45	-0.65
-1.63	-0.49	0.03	-1.76	-0.98	-0.89	-1.33
-0.70	-0.64	0.71	-0.83	1.93	0.90	2.10
-1.06	-0.60	-0.64	-0.60	-0.36	1.29	0.65
-0.67	1.06	-0.43	-1.62	1.12	-0.75	0.43
-0.61	-0.07	-1.04	-0.56	-1.04	2.33	-0.97
0.20	1.44	-1.09	0.99	-0.73	1.19	-0.90
-0.28	-0.46	-0.87	0.81	-0.97	-0.93	-1.06
-1.27	-0.92	-0.22	-0.82	-0.55	-0.55	0.14
-0.45	-1.03	0.51	-0.93	0.48	0.07	0.25
-0.50	0.12	1.40	-0.37	0.08	-0.98	0.86
0.70	0.88	-0.81	0.50	-1.29	0.17	-1.20
-0.40	-0.64	2.54	0.10	1.55	1.92	0.32
0.25	0.17	-0.92	1.12	-0.96	-1.09	-0.91
-0.65	-0.40	0.42	-0.55	0.96	-0.14	1.15
-0.11	-0.45	1.89	0.50	1.19	0.07	0.59
1.91	-0.52	-0.30	0.57	-0.45	-0.50	-0.20
0.91	0.02	-0.46	1.25	1.49	-0.25	0.93
0.81	-0.87	0.75	-0.43	-0.30	-0.64	-0.28
0.54	-0.89	1.09	-0.16	1.17	-0.75	1.59
1.52	-0.54	-0.79	0.05	-0.47	1.39	-0.46
2.31	2.21	-0.70	1.82	-0.99	-0.65	-0.92
1.45	0.45	-0.68	1.08	-0.61	-0.30	-0.55
0.47	-0.30	-1.05	1.85	-0.88	-0.51	-0.74
-0.65	3.11	-0.94	-1.41	-0.52	-1.42	-0.78
-0.45	0.59	-0.01	-0.21	0.19	-0.36	-0.29
-1.18	-0.56	1.52	-1.03	1.59	-0.07	2.22
-1.35	-0.61	2.29	-1.23	2.58	0.27	4.25
-0.71	-0.37	1.37	-0.75	0.06	0.10	0.11
0.63	-1.21	-0.14	-1.36	-0.07	0.12	0.59
-0.99	-0.78	-1.00	-1.51	0.18	0.76	0.91
3.75	0.02	1.28	-1.63	0.63	0.97	3.39
-1.49	3.63	0.34	-1.59	2.62	-0.85	3.13
-1.23	-0.99	-0.63	1.04	-0.55	-0.58	-0.72
-0.97	-1.25	-0.36	0.77	-0.28	-0.29	-0.44
0.85	-0.45	-0.10	0.75	-0.91	-0.44	-1.07
-1.48	-0.79	-1.40	-0.54	-1.42	-0.94	-1.22
-0.30	-1.01	0.17	-0.48	-0.27	3.02	-0.54
0.06	-1.09	2.29	-0.28	1.75	2.96	0.85

1.39	-0.60	0.28	0.10	-0.76	-1.02	-0.79
2.14	0.30	-1.37	3.97	-0.81	2.41	-0.80
5.08	1.80	-1.42	11.09	-0.85	11.30	-0.89
0.60	-0.19	-0.52	0.77	-0.59	-0.23	-0.59
-0.51	-0.99	1.34	0.07	0.82	1.88	0.18
2.08	0.02	-0.53	-0.04	-0.95	-1.29	-1.07
0.08	-0.33	-1.41	2.72	-1.02	0.47	-1.00
0.89	-0.01	1.57	-0.14	0.36	-0.81	-0.02
1.10	-0.41	-0.49	1.23	-0.69	-0.60	-0.90
-0.50	-0.94	0.40	0.47	0.02	-0.72	-0.29
-0.05	-0.94	-0.09	0.72	-0.34	-0.87	-0.49
2.81	-0.93	0.40	0.27	0.59	-0.49	0.15
0.21	0.21	-0.69	1.56	-0.65	0.00	-0.85
-0.12	0.62	-0.98	0.13	-0.30	0.22	-0.30
0.14	-0.26	0.23	0.59	0.61	0.48	-0.25
1.71	0.59	-0.82	0.14	-1.04	-0.06	-0.98
1.11	0.25	-0.74	-0.21	-0.77	-0.74	-0.39
0.06	0.17	-0.32	-0.44	-0.41	0.94	-0.41
-0.48	-0.70	3.12	-1.08	2.52	0.26	3.54
0.28	1.06	0.25	-0.11	-0.15	-0.56	-0.55
-0.07	0.70	1.17	-1.29	0.98	0.22	0.24
-0.46	0.52	1.04	-1.49	1.64	-0.76	0.28
-0.70	-1.04	3.62	-1.23	3.12	0.09	6.13
0.22	-0.42	-0.70	-0.30	-0.75	-0.92	-0.58
-0.72	-0.08	0.08	-0.74	-0.19	0.66	-0.32
-0.58	-0.74	0.59	-1.20	0.78	0.58	1.52
2.73	0.02	-0.68	1.55	-0.50	6.00	-0.21
-1.55	-1.26	2.16	-1.05	1.62	2.28	0.94
-0.16	-0.56	-0.32	-1.76	0.64	1.32	2.11
-0.08	0.65	-0.64	-0.92	2.45	2.35	2.51
0.03	0.41	1.01	-0.17	1.86	1.34	3.05
-0.16	0.04	-0.59	-0.13	-0.29	1.24	-0.43
2.38	-1.12	0.22	-1.53	0.21	2.54	-0.56
-0.41	-0.72	0.08	-0.82	0.02	1.56	-0.37
0.21	-0.53	1.05	0.11	0.45	0.06	-0.07
1.49	-0.59	-1.42	9.10	-1.55	1.47	-1.33
-0.27	0.19	0.30	-0.03	0.34	1.23	0.04
-0.60	-0.63	0.15	-1.08	-0.02	-0.49	0.59
1.47	0.72	-0.21	-1.47	-1.13	-0.28	-1.02
0.15	-0.93	1.07	0.19	2.84	0.44	3.65
0.33	-0.18	1.18	-0.47	0.39	-0.71	-0.04
-0.65	-0.23	-0.51	0.26	-0.62	-1.18	0.02
0.45	-0.42	0.15	-0.13	-0.06	-0.03	-0.16
0.03	-0.57	-0.74	1.59	-0.81	-0.97	-0.78
0.42	-0.93	1.32	-0.49	0.80	-0.08	0.64
-1.12	-1.14	0.86	-1.20	2.14	-0.12	3.00
0.84	-0.39	0.40	0.65	-0.49	-1.08	-0.56
0.17	-0.53	0.23	0.05	-0.18	-0.44	-0.42
-2.04	-0.80	-0.26	-0.40	-0.53	0.13	-0.41
0.99	-0.92	1.24	-0.19	0.73	-0.15	0.45
0.62	0.37	0.09	0.13	-0.18	-0.73	-0.29
1.24	0.80	0.14	-1.00	0.12	1.26	0.77
2.90	-0.86	-0.79	0.00	-1.01	0.58	-0.83
3.08	1.34	-0.57	0.86	-0.82	-0.27	-0.77
-0.08	-0.43	0.58	-0.81	0.19	-0.07	1.39
0.05	-1.18	2.62	-0.55	0.20	2.97	-0.16
0.27	0.27	0.27	0.27	0.28	0.28	0.28
0.12	0.12	0.27	0.15	0.33	0.32	0.24

721119	27468	816795	972175	1468719	887	19904
					1	1
					1	1
71	72	73	74	75	76	77
2	3	4	5	6	7	8
A_12	A_18	A_3	A_5	A_6	A_8	A_9
CHR_P	CHR_P	CHR_P	CHR_P	CHR_P	CHR_P	CHR_P
2271250	356761	5807356	3122435	4486274	3080	82291
0.07	0.24	0.03	0.85	0.97	0.24	0.12
A_12	A_18	A_3	A_5	A_6	A_8	A_9
1550131	329293	4990561	2150260	3017555	2193	62387
261344	6469	132111	198821	807275	155	7754
13505	8375	34328	58994	36332	44	431
7691	750	35614	51330	51941	53	2064
14227	1228	168094	97543	91415	92	1547
12291	536	64203	87260	63497	60	1310
86372	452	20677	38724	17647	43	10
15656	647	22001	51972	34873	51	94
24861	117	4457	7468	4352	11	10
49674	88	6761	18115	20373	13	17
80322	1803	36876	40293	52728	42	934
24136	674	26458	23921	36895	23	288
8992	204	26259	32101	21912	27	178
5991	139	30575	25249	21818	23	63
9409	238	38899	20915	21332	15	609
3972	189	9342	9807	6502	13	17
1658	7	2939	3906	12372	2	380
4083	91	5496	12964	5382	10	8
3990	345	13701	17197	10384	13	54
9484	119	2645	8526	5314	9	17
4091	768	2897	7001	9014	6	152
2473	149	3338	6047	4666	7	62
5947	167	10042	10806	10705	11	50
3135	126	869	3590	6023	3	226
3250	109	2006	9031	3612	4	92
2971	300	1547	5495	2202	6	42
4140	22	1660	3495	8134	7	459
1563	58	9033	4396	5415	2	229
2972	204	2727	3487	1953	26	265
1962	124	6624	5075	2833	4	46
2288	86	1596	2878	5088	3	14
1545	37	2190	5104	3250	4	20
513	18	9944	5368	4663	8	47
3180	9	1671	3742	2217	6	0
2604	32	547	1978	1123	4	2
1315	86	4632	3492	1949	1	33
2471	193	15156	5512	4119	4	48
3383	483	2811	3748	2187	5	11
3737	399	1795	3909	2191	1	38
479	145	911	2595	3401	2	6
470	12	4393	2097	3374	5	346
718	114	3445	5257	3705	4	94
344	10	1815	2919	1895	1	70
1017	63	3797	3856	2949	4	23
909	79	711	712	491	0	3
1334	103	1435	2476	1914	5	161

750	34	1382	3150	2764	2	20
1003	16	858	1667	1540	0	1
515	24	1322	2479	3433	3	63
124	1	981	727	791	0	1
350	8	45	410	628	0	113
664	14	1172	1836	1881	6	4
509	31	663	1584	478	1	6
1145	63	1159	1265	966	1	8
382	52	524	2087	1910	6	526
382	0	12	46	747	0	36
255	3	43	126	213	0	3
890	24	939	1409	670	1	4
247	7	368	1337	733	2	1
409	37	823	1500	1214	0	12
147	66	925	1334	1512	1	30
578	16	1649	1798	1255	0	1
275	6	243	634	550	2	14
792	9	94	207	129	0	2
61	1	7	37	41	0	0
198	31	2408	869	2392	0	19
747	19	1011	1035	622	3	0
637	3	199	376	232	0	0
330	6	544	646	372	0	0
307	8	420	957	366	2	8
888	111	387	591	411	0	2
309	21	537	548	538	0	15
289	9	1862	954	1361	3	1
464	16	291	468	257	0	1
527	8	301	336	718	2	14
244	7	228	798	314	1	0
179	5	741	1033	816	0	49
288	0	16	43	409	0	18
157	107	109	254	256	0	11
49	2	659	614	566	1	15
231	15	347	262	158	0	3
385	4	658	949	398	2	1
111	0	142	322	526	0	53
88	28	1066	638	675	0	3
423	3	33	138	113	0	1
239	13	98	367	177	0	3
162	0	189	346	384	2	10
116	10	367	406	416	0	9
402	1	283	467	199	0	0
273	5	282	579	312	0	0
38	6	413	439	560	0	5
43	6	184	232	337	2	75
17	0	19	9	22	0	0
111	1	91	322	382	0	7
269	6	80	215	192	0	0
159	0	43	107	72	0	1
97	4	111	350	122	0	1
26	6	148	215	265	0	5
58	11	245	323	352	0	29
48	15	343	406	296	0	27
93	0	39	66	31	0	0
238	2	548	373	221	0	0
104	1	93	182	124	0	1
79	1	201	227	212	0	36
65	19	200	306	162	0	5

287	4	147	300	198	0	0
247	3	122	165	126	0	0
66	8	112	234	170	0	4
86	7	85	159	91	0	0
144	0	154	203	124	0	0
93	0	81	133	106	0	0
57	4	67	237	82	1	1
48	3	187	260	209	1	3
80	2	200	293	186	1	9
126	7	230	314	102	0	0
14	3	50	146	94	1	3
42	7	282	229	237	0	9
50	3	81	158	96	0	1
101	1	123	220	98	1	2
101	1	123	220	98	1	2
116	4	211	195	87	0	6
271	2	81	98	161	0	3
71	3	77	185	266	0	10
69	1	15	72	99	0	0
55	5	27	111	109	0	3
3	0	2	5	14	0	0
30	2	174	222	165	0	1
72	2	45	71	50	0	1
34	0	68	115	153	0	1
32	5	170	162	116	0	9
4	3	71	160	113	0	0
20	1	4	5	26	0	0
48	2	74	221	151	0	16
60	2	36	85	53	0	3
46	4	84	115	84	0	2
150	0	21	129	51	0	0
22	0	29	98	83	0	4
43	0	21	29	64	0	11
88	0	89	82	152	1	1
34	1	54	94	78	0	0
26	2	66	85	74	1	0
22	1	12	25	124	0	7
65	1	42	52	43	0	0
13	0	187	93	132	0	21
11	1	59	154	86	0	1
28	0	9	9	4	0	0
30	0	220	73	148	0	1
15	0	82	196	93	0	1
20	1	21	80	50	0	0
38	0	128	105	109	0	0
32	2	273	128	104	0	2
51	1	5	27	26	0	1
57	0	96	108	31	0	0
49	0	4	35	68	0	2
11	0	33	99	80	0	2
4	0	13	52	38	0	23
41	2	120	58	54	0	0
15	4	77	44	33	0	0
37	1	75	39	27	0	2
32	0	48	95	66	0	3
31	1	23	22	102	0	6
4	0	4	7	12	0	0
17	1	43	74	43	0	1
10	2	63	46	34	0	1

38	0	17	52	43	0	0
14	1	11	68	18	0	0
16	2	82	51	112	0	1
11	0	48	65	45	0	1
238	2	14	14	29	0	1
18	1	2	10	5	0	0
43	1	65	68	109	0	0
6	0	17	25	13	0	1
54	0	8	35	49	0	2
15	2	28	32	50	0	5
15	0	16	31	22	0	0
14	1	2	46	35	0	0
9	0	53	60	63	1	0
45	0	83	96	63	0	1
22	1	23	46	16	0	0
56	2	24	34	19	0	0
19	0	58	56	44	0	2
0	1	41	47	31	0	1
20	0	13	22	15	0	0
28	0	30	76	54	0	6
67	1	30	28	12	0	0
28	5	48	48	36	0	10
17	0	49	55	45	0	4
17	0	13	21	13	0	0
25	0	106	112	88	0	4
8	0	23	30	23	0	0
4	0	2	12	17	0	0
99	2	16	29	62	0	1
32	1	5	7	1	0	0
17	1	52	80	20	0	0
4	1	36	75	48	0	0
38	0	17	21	58	0	0
4	2	25	35	23	0	0
7	0	1	9	11	0	2
15	0	66	46	38	0	0
22	1	21	50	21	0	1
13	0	17	64	27	0	0
0	0	24	50	23	0	0
21	0	11	32	32	0	0
4	1	96	44	64	0	2
6	0	2	36	24	0	17
34	1	49	37	11	0	0
19	0	16	26	80	0	1
11	0	38	30	26	0	0
10	0	52	60	43	0	0
11	0	15	46	20	0	0
30	0	101	29	60	0	4
9	1	64	63	65	0	3
6	0	8	41	15	0	0
20	0	22	32	24	0	0
5	0	65	44	35	0	2
8	0	4	9	13	0	0
3	1	9	25	23	0	0
4	0	4	9	16	0	0
3	2	0	23	21	0	1
0	0	1	0	0	0	0
4	0	28	30	32	0	0
17	1	1	7	11	0	0
27	0	22	29	35	0	0

18	0	14	35	14	0	1
30	1	38	26	25	0	0
11	0	43	66	44	0	0
3	0	8	14	21	1	1
4	0	40	25	20	0	2
6	0	16	18	37	0	1
4	0	43	39	29	0	0
27	3	20	16	17	0	0
21	0	3	11	2	0	0
8	2	35	16	26	0	0
8	0	2	1	2	0	0
7	0	9	16	24	0	0
5	0	1	8	20	0	0
12	0	23	17	30	0	1
3	0	9	13	8	0	0
5	0	8	17	10	0	1
5	0	4	16	8	0	0
3	0	10	20	25	0	0
13	1	27	42	35	0	3
21	0	5	8	16	0	0
14	0	6	18	5	0	0
12	0	3	14	10	0	0
12	0	3	14	10	0	0
4	0	25	19	22	0	0
6	0	44	20	10	0	1
2	0	11	9	5	0	1
4	0	16	20	18	0	0
4	0	5	12	9	0	0
3	0	4	23	11	0	0
10	0	6	22	15	0	1
9	0	14	9	13	0	0
1	0	5	3	21	0	0
5	1	5	23	5	0	0
1	0	2	6	13	0	0
5	0	15	19	11	0	1
19	1	14	11	11	0	0
3	0	0	1	1	0	0
10	0	27	24	20	0	0
10	0	7	28	8	0	0
1	0	10	14	6	0	0
4	0	43	25	16	0	0
10	0	4	24	8	0	0
2	1	7	20	18	0	5
5	0	17	20	7	0	0
13	0	7	13	15	0	0
1	0	17	4	15	0	0
0	0	0	1	0	0	0
1	0	3	6	8	0	3
3	1	39	15	20	0	0
5	0	51	18	18	0	0
3	0	20	22	30	0	0
6	0	11	14	10	0	0
0	0	2	1	1	0	0
6	0	51	31	13	0	0
7	0	3	12	16	0	0
4	0	69	1	4	0	3
1	1	49	8	14	0	0
6	0	9	23	5	0	1
4	1	15	9	7	0	0

7	0	15	13	7	0	0
3	0	16	8	13	0	1
5	0	16	30	30	0	0
4	0	1	5	10	0	0
6	1	20	10	13	0	0
2	0	5	11	11	0	1
5	0	2	11	15	0	0
8	0	12	17	17	0	0
5	0	0	4	0	0	0
4	0	9	4	5	0	0
2	1	1	3	0	0	0
1	0	20	15	14	0	0
12	0	5	9	5	0	0
0	0	2	7	18	1	1
6	0	8	13	14	0	5
9	0	7	7	1	0	0
1	1	15	11	20	0	0
5	0	4	5	6	0	0
6	0	1	4	13	0	0
13	0	9	17	7	0	0
1	0	3	8	10	0	0
4	0	17	18	9	0	0
0	0	1	10	11	0	0
4	0	17	7	11	0	0
3	0	8	4	11	0	0
0	0	11	5	5	0	0
6	1	14	11	20	0	0
2	0	4	10	5	0	0
5	0	4	21	8	0	0
0	0	3	7	7	0	0
2	0	6	6	7	0	1
4	0	2	6	1	0	0
2	0	0	1	2	0	0
4	2	10	12	10	0	0
4	1	15	9	3	0	1
8	2	14	9	16	0	0
2	0	3	7	6	0	1
2	0	2	5	8	0	0
1	0	7	6	6	0	0
1	0	1	3	9	0	0
6	0	2	7	6	0	0
1	0	4	11	7	0	0
2	0	4	6	12	0	0
0	0	3	7	12	0	0
4	0	10	16	12	0	0
11	0	8	7	0	0	1
5	0	12	6	4	0	0
10	0	14	9	10	0	1
3	0	10	7	4	0	0
0	0	4	16	9	0	0
0	0	14	6	10	0	0
6	0	0	1	2	0	0
1	0	7	9	7	0	0
7	1	0	5	2	0	0
2	0	1	3	3	0	0
1	0	1	3	1	0	0
0	0	6	6	7	0	0
1	0	0	0	0	0	0
2	0	3	4	17	0	3

4	0	1	6	6	0	0
2	0	10	8	5	0	0
1	0	1	3	3	0	0
1	0	3	4	3	0	0
2	0	2	4	6	0	0
0	0	0	1	5	0	0
7	0	6	6	5	0	0
3	0	2	7	3	0	0
8	0	14	8	6	0	1
3	0	5	8	6	0	0
1	0	1	5	5	0	0
3	0	2	7	2	0	0
2	4	6	13	1	0	0
1	0	0	0	0	0	0
0	0	7	26	2	0	0
9	0	2	8	7	0	1
0	0	5	14	4	0	0
5	1	3	4	5	0	0
2	0	2	3	3	0	0
2	1	1	11	6	0	0
0	0	0	4	3	0	0
0	0	0	5	3	0	0
2	0	6	7	1	0	0
3	0	10	7	2	0	0
0	0	5	9	5	0	0
0	0	3	9	6	0	0
1	0	0	5	0	0	0
1	0	0	2	1	0	0
1	0	1	4	5	0	4
1	0	1	3	2	0	0
2	0	2	2	3	0	0
2	0	8	5	4	0	0
1	0	1	3	2	0	0
3	0	3	4	4	0	0
2	0	3	0	1	0	0
3	0	6	3	7	0	0
0	0	1	0	1	0	0
2	0	7	4	7	0	0
2	0	2	4	6	0	0
0	1	4	4	6	0	0
0	0	3	2	1	0	0
0	0	2	5	5	1	0
2	0	2	9	5	0	0
1	0	1	4	4	0	0
1	0	0	0	2	0	0
2	0	1	4	0	0	0
0	0	4	5	3	0	0
0	0	1	2	2	0	0
1	0	0	2	1	0	0
0	0	0	4	3	0	0
0	0	12	5	8	0	0
0	0	1	8	2	0	0
5	0	0	1	1	0	0
2	0	3	10	7	0	0
0	0	1	8	3	0	0
3	0	0	4	0	0	2
1	0	0	3	0	0	0
1	1	0	7	5	0	0
2	0	3	3	0	0	0

1	0	4	5	4	0	0
3	0	1	1	1	0	1
0	0	0	8	0	0	0
0	0	1	5	1	0	0
1	0	0	5	4	0	0
2	0	7	3	3	0	0
1	0	0	0	1	0	0
0	0	1	4	1	0	0
0	0	3	7	0	0	0
0	0	0	1	5	0	0
0	0	0	1	4	0	0
1	0	0	1	1	0	0
0	0	0	1	2	0	0
0	0	0	2	3	0	0
0	0	1	0	3	0	0
1	0	2	2	3	0	0
1	1	5	4	1	0	0
1	0	5	2	1	0	0
0	0	0	0	0	0	0
3	0	2	3	0	0	0
0	0	4	8	0	0	0
3	0	1	2	0	0	0
0	0	0	0	6	0	0
1	0	1	1	2	0	0
2	0	2	1	6	0	0
0	0	0	0	0	0	0
0	0	0	0	1	0	0
1	1	1	3	2	0	0
0	0	5	0	1	0	0
0	0	4	1	2	0	0
0	0	0	0	0	0	0
2	0	0	3	4	0	0
1	0	4	5	2	0	0
1	0	5	1	4	0	0
0	0	2	3	5	0	0
1	0	2	2	0	0	1
3	0	5	1	6	0	0
1	0	0	1	4	0	0
1	0	0	1	3	0	0
2	0	3	2	7	0	0
1	0	0	2	1	0	0
0	0	0	0	4	0	0
1	0	1	7	0	0	1
1	0	0	0	0	0	0
2	0	2	0	5	0	0
1	0	0	4	2	0	0
0	0	2	0	2	0	0
0	0	2	2	0	0	0
0	0	2	1	4	0	0
0	0	0	0	2	0	0
1	0	0	3	1	0	0
0	0	2	2	0	0	0
0	0	2	5	3	0	0
3	1	0	2	3	0	0
1	0	2	1	1	0	0
1	0	0	2	0	0	0
2	0	0	0	1	0	0
1	0	4	3	3	0	0
1	0	2	0	2	0	0

2	0	0	3	1	0	0
3	0	0	1	1	0	1
0	0	1	1	2	0	0
7	0	1	1	3	0	0
0	0	1	1	0	0	0
1	0	0	2	1	0	0
0	0	0	0	2	0	0
3	0	0	2	0	0	0
1	0	0	3	1	0	0
1	0	3	0	0	0	0
0	0	1	0	7	0	0
1	0	1	0	2	0	0
1	0	0	0	0	0	0
0	0	1	2	1	0	0
1	0	1	2	3	0	0
0	0	1	3	1	0	0
0	0	2	1	1	0	0
0	0	1	0	0	0	1
1	0	0	0	0	0	0
2	0	2	1	1	0	0
4	0	0	2	2	0	0
1	0	0	0	0	0	0
0	0	0	0	0	0	0
0	0	1	2	0	0	0
0	0	0	0	1	0	0
1	0	1	0	0	0	0
1	0	0	0	1	0	0
0	0	2	2	0	0	0
1	0	0	0	0	0	0
4	0	3	1	0	0	0
3	0	1	4	0	0	0
0	0	3	2	2	0	0
1	0	1	4	1	0	0
2	0	0	3	2	0	0
1	0	0	1	0	0	0
4	0	3	2	1	0	0
2	0	1	0	0	0	0
0	0	0	1	1	0	0
0	0	0	0	0	0	0
0	0	1	0	1	0	0
2	0	0	0	1	0	0
0	0	1	2	1	0	0
0	0	1	1	1	0	0
0	0	0	0	2	0	0
1	0	0	2	0	0	0
0	0	3	1	1	0	0
1	0	2	1	2	0	0
0	0	2	0	1	0	0
0	0	1	4	1	0	0
0	0	0	0	3	0	0
0	0	0	1	4	0	0
0	0	0	2	0	0	0
1	0	1	1	2	0	0
0	0	0	0	6	0	0
0	0	3	1	0	0	0
1	0	1	2	0	0	0
0	0	0	1	3	0	0
0	0	0	0	2	0	0
1	0	0	0	1	0	0

0	0	0	2	0	0	0
0	0	1	0	0	0	0
1	0	0	1	1	0	0
0	0	0	0	3	0	0
0	0	2	0	2	0	0
0	0	1	2	1	0	0
0	0	0	0	0	0	0
1	0	0	0	0	0	0
3	0	0	0	3	0	0
0	0	1	0	0	0	0
0	0	2	3	0	0	0
0	0	1	1	3	0	0
0	5	0	0	0	28	14
A_12	A_18	A_3	A_5	A_6	A_8	A_9
CHR_P	CHR_P	CHR_P	CHR_P	CHR_P	CHR_P	CHR_P
1550131	329293	4990561	2150260	3017555	2193	62387
22	29	27	22	21	23	24
0.06	1.58	1.20	0.16	-0.02	0.32	0.61

0.85	0.30	0.51	0.48	0.05	0.43	0.20
6	17	11	12	25	13	20
1	1	1	1	1	1	1

71	72	73	74	75	76	77
6	7	8	9	10	11	12
A_6	B_31	C_4	C_45	D_11	D_35	D_5
CHR_P	CHR_P	CHR_P	CHR_P	CHR_P	CHR_P	CHR_P
1468719	409595	1862148	4820581	198165	2718285	905697
A_6	B_31	C_4	C_45	D_11	D_35	D_5
807275	57543	839593	1485363	66104	922039	250879
36332	782	62135	149104	2808	110593	187485
51941	116387	66190	519025	27131	288601	83289
91415	8068	137605	290995	2601	177243	39675
63497	12377	74324	311607	21738	157984	41080
17647	76530	110612	290912	76	148973	33455
34873	17767	60894	195271	1079	87662	37482
4352	3301	13437	142980	751	19838	9389
20373	1786	45753	137089	680	116931	10484
52728	9961	80032	105854	790	50422	31476
36895	14513	52008	71950	4647	67416	9519
21912	4756	23945	89230	1976	38096	8959
21818	10040	31706	64809	394	62420	7016
21332	1245	29523	91349	10147	28410	5598
6502	16498	20456	76934	42	49422	5698
12372	391	12155	36167	4096	34739	976
5382	2884	9723	47570	42	23519	5442
10384	1475	9271	47481	1230	22958	8395
5314	2600	11194	44142	118	18192	5563

9014	4618	6833	23589	858	12258	13575
4666	1685	9605	29496	2819	13803	6319
10705	834	9464	27925	164	12294	3499
6023	3021	4524	7791	39	10145	5295
3612	673	2563	23854	103	5539	5107
2202	2687	4476	44370	52	6827	8514
8134	1409	6167	16802	977	8451	3533
5415	197	5688	14709	9351	15189	1765
1953	4048	8002	20128	78	5939	5604
2833	284	5632	14766	278	8474	2631
5088	948	4409	6685	418	8789	2520
3250	2002	5124	25164	59	9530	3332
4663	433	7383	20268	385	8809	1435
2217	1549	3256	13895	86	6943	1007
1123	1700	1982	10549	18	6747	794
1949	239	5156	13744	257	7733	2413
4119	458	6282	13928	136	4661	2640
2187	2186	3772	15343	153	5402	6796
2191	1609	4793	19198	132	3571	4428
3401	167	2424	7797	1014	2545	4355
3374	825	2996	7643	4999	4253	645
3705	78	1428	11190	4456	2903	1173
1895	471	2099	11262	934	7323	1439
2949	602	2529	6823	1404	3054	868
491	1521	2832	9459	19	3067	1973
1914	595	1373	9487	1047	3258	1950
2764	424	1740	6714	471	3847	975
1540	186	1506	3436	169	3733	841
3433	549	2982	6373	1287	4733	865
791	212	959	1740	4	1212	28
628	2941	539	9094	31	2585	920
1881	14	728	3394	682	4094	865
478	172	1546	8012	189	2108	1343
966	138	1472	4898	2738	2224	1314
1910	1599	1514	4602	26	2533	2209
747	88	651	1278	93	1729	394
213	382	207	1338	10	837	81
670	428	1397	4655	125	1954	667
733	157	936	5182	10	2506	630
1214	202	1298	5766	66	3213	1208
1512	123	765	3285	174	2007	507
1255	120	918	4397	392	1512	344
550	175	579	4681	269	2088	1090
129	106	433	1690	9	767	178
41	79	66	195	6	144	36
2392	438	1622	4366	139	2114	216
622	236	979	3131	2	1689	458
232	461	698	4507	12	1244	331
372	103	311	2696	1	1333	271
366	131	598	7405	81	781	465
411	29	1410	1546	20	395	1464
538	442	640	2261	346	1351	322
1361	140	1117	1937	218	1738	245
257	73	558	2504	4	1489	291
718	314	1035	2168	35	1279	349
314	175	376	1387	27	1345	357
816	146	433	3090	170	2575	217
409	38	665	1039	206	1059	340
256	9	366	703	87	482	428

566	56	559	2219	331	1650	185
158	30	634	2223	119	849	231
398	28	1130	1364	27	1025	146
526	20	383	1929	409	1023	133
675	36	695	1086	22	756	296
113	69	163	712	1	1101	87
177	50	251	656	18	464	305
384	2	260	1491	2711	571	138
416	160	450	947	1111	995	260
199	149	486	2576	7	980	121
312	319	474	1826	2	1036	142
560	36	356	1112	38	1129	190
337	217	370	1293	74	581	157
22	7	61	126	11	84	3
382	26	361	1762	96	710	97
192	58	236	982	7	959	86
72	245	249	1371	1	647	45
122	262	322	2157	1	996	165
265	25	197	628	139	612	227
352	160	312	944	212	510	189
296	81	185	818	165	368	332
31	66	176	1483	3	651	104
221	6	125	584	526	170	224
124	131	317	427	3	391	114
212	34	288	921	275	827	125
162	54	247	971	0	285	129
198	244	362	2134	0	396	165
126	17	173	973	3	430	109
170	48	135	921	694	331	117
91	61	165	676	9	235	206
124	68	187	919	1	501	39
106	26	179	1203	14	513	61
82	108	156	1025	6	450	256
209	24	80	588	111	440	123
186	32	85	978	413	192	183
102	24	139	459	0	286	133
94	20	137	753	20	598	144
237	9	170	525	35	436	66
96	38	83	424	4	508	102
98	105	142	472	3	425	48
98	105	142	472	3	425	48
87	14	184	1078	7	177	136
161	30	205	911	345	247	28
266	43	248	1072	4	336	87
99	22	63	492	0	491	66
109	59	87	391	8	241	219
14	5	19	131	4	94	8
165	23	173	453	28	275	32
50	13	70	132	663	112	64
153	36	121	518	30	157	53
116	2	59	244	294	79	96
113	3	63	307	45	220	69
26	2	98	106	63	36	25
151	115	106	657	27	328	79
53	5	72	222	13	160	64
84	16	81	230	128	129	25
51	11	56	185	126	198	16
83	2	89	335	30	271	96
64	4	209	327	10	250	29

152	6	83	331	45	355	35
78	19	86	246	0	174	26
74	14	93	759	107	120	36
124	5	72	219	20	75	30
43	19	128	359	19	156	30
132	11	108	661	83	257	22
86	22	41	198	1	127	37
4	11	33	200	4	62	2
148	40	193	453	51	145	22
93	7	45	250	0	169	32
50	37	51	286	0	130	44
109	66	215	314	3	195	25
104	2	50	168	109	87	35
26	25	70	227	2	159	65
31	18	81	269	3	152	29
68	6	32	427	0	519	31
80	1	39	226	13	71	19
38	14	17	782	2	215	6
54	21	99	180	74	81	19
33	1	33	86	9	69	44
27	7	33	226	50	84	19
66	23	54	297	90	84	31
102	3	89	85	103	182	16
12	3	8	50	1	34	11
43	8	33	241	95	74	42
34	10	71	269	83	94	23
43	16	32	421	1	134	39
18	4	27	182	0	121	40
112	17	97	210	65	195	12
45	3	45	176	2	51	35
29	5	33	280	265	27	33
5	37	5	101	0	56	8
109	16	217	254	11	176	12
13	32	34	234	12	25	28
49	3	111	153	3	160	21
50	3	62	150	2	124	71
22	7	61	271	2	134	61
35	7	25	277	1	80	26
63	5	45	76	186	138	19
63	5	31	178	12	208	16
16	38	44	219	2	116	36
19	4	55	227	47	44	22
44	19	33	236	19	181	19
31	2	97	131	16	123	28
15	8	45	186	0	100	19
54	0	15	158	27	117	9
12	12	61	225	0	98	24
36	4	25	91	21	78	27
45	5	56	172	107	91	7
13	7	41	172	0	98	17
88	22	25	228	2	204	11
23	13	16	168	23	108	3
17	4	33	169	5	55	18
62	2	20	48	179	2	1
1	17	29	359	0	90	16
20	1	21	177	6	78	47
48	12	24	100	4	113	21
58	5	62	99	14	65	31
23	4	16	29	13	44	13

11	19	10	81	0	31	8
38	11	95	171	4	80	21
21	1	20	186	90	55	9
27	14	63	119	18	53	34
23	0	16	85	3	50	20
32	10	13	301	1	113	49
64	0	46	104	55	28	14
24	5	28	49	32	61	36
11	1	34	106	0	54	8
80	2	15	123	22	144	3
26	3	61	118	30	56	17
43	29	35	142	0	62	31
20	1	18	140	4	134	1
60	3	54	204	21	35	5
65	4	16	57	52	35	22
15	8	30	138	2	74	21
24	22	18	123	0	142	21
35	1	53	124	63	73	17
13	9	35	108	5	122	11
23	28	32	84	26	47	30
16	8	30	74	5	52	12
21	4	25	138	5	73	15
0	0	2	16	0	13	0
32	0	41	68	0	51	4
11	1	2	11	0	34	0
35	5	61	116	22	58	9
14	3	12	59	7	24	12
25	3	33	125	24	42	8
44	6	20	152	0	129	8
21	0	16	77	22	58	13
20	2	21	43	32	59	10
37	2	42	41	24	83	3
29	2	22	58	13	27	9
17	2	25	77	0	22	10
2	7	23	71	0	43	15
26	4	27	34	15	44	18
2	9	8	44	0	21	2
24	2	16	65	40	39	4
20	3	22	69	0	40	2
30	5	22	49	97	29	7
8	2	4	35	102	24	11
10	1	4	54	0	12	13
8	27	9	117	1	43	14
25	4	22	89	3	24	1
35	2	21	140	1	82	8
16	4	23	36	5	31	1
5	2	20	71	0	24	8
10	4	2	45	0	22	15
10	4	2	45	0	22	15
22	0	6	74	3	69	5
10	0	18	70	26	31	8
5	2	10	38	11	20	10
18	6	14	94	2	115	4
9	5	16	98	0	25	9
11	3	11	78	3	36	6
15	6	14	36	3	23	4
13	0	75	106	1	56	0
21	0	11	32	78	12	2
5	10	11	102	0	23	11

13	0	21	13	5	10	8
11	5	5	63	2	39	0
11	0	19	56	0	17	7
1	2	4	11	2	9	0
20	2	23	113	5	15	8
8	6	22	36	0	24	12
6	1	12	32	11	36	8
16	1	35	48	18	41	3
8	5	20	34	0	23	11
18	0	14	32	4	49	8
7	0	8	31	23	41	2
15	5	16	68	1	52	4
15	1	14	77	14	32	4
0	0	0	0	0	2	1
8	1	2	25	73	23	5
20	2	24	48	16	22	5
18	0	8	27	32	29	5
30	1	11	112	1	49	0
10	2	13	24	0	15	7
1	0	1	32	0	11	0
13	1	3	40	3	11	10
16	0	14	91	0	31	5
4	1	14	46	0	0	0
14	0	19	30	12	22	1
5	1	30	83	2	36	1
7	1	6	24	20	7	7
7	0	3	55	5	27	2
13	0	22	52	4	29	5
30	8	11	46	1	63	3
10	0	13	29	20	21	2
13	0	6	31	14	24	3
11	1	19	22	4	20	8
15	0	11	81	0	28	4
17	1	10	145	1	51	0
0	2	19	76	0	2	4
5	13	4	24	0	28	2
0	2	5	10	1	20	25
14	0	4	20	17	14	1
5	2	8	33	0	20	8
18	5	1	71	3	69	0
14	3	10	33	9	41	4
1	6	12	75	0	20	3
20	2	12	39	2	12	7
6	1	9	28	10	25	2
13	3	5	31	46	6	3
7	18	5	242	0	76	2
10	0	2	41	2	49	1
9	0	6	28	1	14	5
11	0	8	22	33	22	2
11	3	13	40	18	52	3
11	6	11	48	1	19	7
5	2	12	48	5	9	2
20	4	7	39	17	24	1
5	0	5	28	33	17	5
8	0	5	35	26	9	2
7	9	15	37	0	27	7
7	0	9	27	5	13	5
1	3	1	18	0	15	2
2	1	6	13	0	14	6

10	13	18	37	1	22	5
3	6	19	31	0	15	5
16	0	10	53	17	13	4
6	2	7	25	0	9	5
8	0	5	48	15	12	7
6	14	4	21	1	19	2
9	0	8	32	6	18	1
6	1	8	16	0	12	2
7	4	12	17	2	18	5
12	0	2	21	2	23	0
12	0	6	16	3	17	3
12	0	6	37	1	19	2
0	2	7	49	13	28	0
4	0	6	15	5	9	1
10	1	9	34	0	8	3
4	0	8	42	19	5	2
9	0	2	32	1	24	9
10	1	7	26	9	5	2
2	1	0	4	1	5	2
7	5	7	9	10	14	2
2	3	2	15	0	15	2
3	1	11	17	2	15	3
1	1	10	27	0	9	1
7	3	4	20	2	13	1
0	0	5	4	0	2	1
17	0	8	17	2	19	3
6	4	13	14	0	11	1
5	4	5	31	2	14	2
3	0	15	14	3	5	3
3	1	8	33	1	17	3
6	1	7	10	6	2	5
5	1	1	15	1	11	3
5	1	4	37	7	14	2
3	2	4	10	0	24	3
6	0	12	23	0	8	2
6	2	6	20	1	15	4
5	2	7	28	14	5	4
2	0	10	38	0	5	0
1	1	7	23	0	11	3
0	1	0	7	0	6	1
2	2	5	20	2	9	0
7	0	4	36	3	10	2
4	0	3	40	0	19	2
5	0	0	12	5	10	0
3	1	0	19	0	4	1
6	0	5	12	4	5	2
3	0	2	12	0	10	9
3	0	2	17	3	4	8
1	1	3	8	1	10	1
2	2	6	21	1	13	0
5	0	5	9	11	8	7
6	1	6	12	4	9	2
0	3	4	58	0	9	0
1	0	3	17	0	5	1
5	0	2	15	5	10	4
2	0	5	9	0	6	4
3	1	6	16	1	2	1
4	1	2	21	0	4	5
2	0	1	12	1	6	0

4	1	4	10	2	21	1
1	5	1	14	0	6	2
7	1	2	15	0	7	0
1	0	1	14	1	8	9
7	0	2	5	10	10	0
6	0	2	12	4	9	0
6	1	2	6	0	8	2
1	1	1	8	2	4	2
5	0	0	11	2	23	3
5	1	3	11	0	14	2
4	0	8	9	1	12	0
2	1	2	10	7	2	3
0	0	3	28	1	3	6
3	0	4	13	3	8	0
2	1	1	16	7	8	0
1	1	5	31	0	5	1
3	0	0	1	0	2	0
8	1	2	11	8	8	0
2	0	1	23	1	17	0
1	5	0	10	0	6	1
7	0	0	8	15	1	3
3	2	4	13	0	9	2
0	2	4	7	0	3	2
0	0	3	7	3	7	4
5	0	4	10	2	6	3
0	0	0	8	4	5	1
4	1	12	17	0	12	1
1	0	5	10	1	8	0
0	2	3	3	0	8	4
1	3	2	11	0	5	0
4	1	3	3	5	6	1
3	0	0	5	5	6	2
1	0	3	16	3	9	1
1	1	0	11	0	8	0
0	1	2	4	1	6	0
5	1	4	8	0	4	1
4	5	1	4	0	11	1
1	0	0	3	0	1	0
2	0	0	0	0	0	0
3	1	1	12	0	5	1
3	0	4	9	23	3	2
3	0	3	8	5	2	0
1	2	5	7	7	3	1
1	0	2	4	0	7	0
0	1	0	2	0	0	0
0	0	2	26	0	8	2
0	0	3	11	1	11	1
0	0	1	13	0	1	1
6	0	1	5	1	9	1
2	0	3	11	2	7	0
6	0	4	7	4	8	1
0	2	0	6	0	2	0
1	0	1	8	0	4	0
2	2	1	20	0	6	0
1	0	3	8	1	5	1
2	0	0	11	0	3	2
0	2	0	4	0	2	0
4	1	0	8	0	5	2
2	0	4	4	1	5	3

4	0	1	6	2	9	0
5	1	5	8	0	3	1
0	0	1	5	1	3	1
6	0	2	6	5	3	0
4	0	6	6	2	9	0
3	0	0	12	0	2	2
7	1	0	19	0	19	2
1	0	0	3	0	7	0
4	2	2	7	0	6	1
0	0	2	4	0	7	3
0	0	3	4	0	7	1
5	0	2	9	1	5	0
2	0	2	10	0	2	0
2	0	7	2	0	1	2
0	0	1	7	5	2	3
4	0	2	3	2	0	0
2	0	2	10	2	5	0
1	0	1	8	0	4	3
0	0	8	6	0	6	0
3	0	0	18	5	2	0
3	0	3	9	0	3	1
1	0	0	5	0	3	2
0	0	2	6	0	0	0
1	0	2	6	2	4	2
3	0	1	4	3	7	1
2	0	0	4	2	1	0
1	1	0	9	0	1	0
1	0	2	8	0	2	0
2	0	1	2	0	4	1
3	0	2	8	2	1	0
0	0	0	3	0	2	0
1	0	3	10	0	6	0
2	1	1	4	3	3	0
0	0	0	3	0	0	0
1	0	1	3	0	3	1
0	0	1	19	0	5	1
7	0	6	9	1	14	0
2	1	1	15	1	1	1
0	1	1	5	0	6	0
1	0	2	0	8	3	0
3	0	2	7	0	7	0
1	0	4	5	0	2	3
1	0	0	2	0	1	0
0	0	0	2	0	2	0
0	0	3	1	0	2	0
1	0	4	7	1	0	2
2	0	0	10	0	2	0
0	1	1	3	0	2	1
0	3	2	4	0	2	1
0	0	1	7	3	4	1
1	1	1	2	2	3	1
0	0	1	12	0	6	1
1	2	0	2	3	3	1
0	0	1	2	3	3	1
0	0	0	1	0	0	0
0	0	3	2	0	0	1
0	0	1	15	0	7	2
2	0	1	4	1	0	0
1	0	4	8	0	3	0

2	0	0	1	2	2	0
0	0	1	10	1	2	1
1	0	1	7	1	0	0
0	0	2	3	6	2	0
1	1	0	7	0	3	1
0	0	3	4	1	0	1
1	0	0	4	6	1	0
1	3	2	7	0	4	1
1	0	0	6	3	0	0
1	0	1	2	0	3	0
2	0	1	1	0	2	0
0	0	1	2	0	3	1
1	0	4	5	0	2	0
2	1	0	1	0	1	0
1	0	4	6	0	3	2
1	0	0	5	1	5	1
3	0	0	6	0	3	1
4	0	1	3	1	3	0
0	0	0	15	0	2	1
2	0	0	12	0	1	0
6	1	0	0	0	1	0
0	3	3	7	1	3	0
0	3	3	7	0	5	2
3	0	0	0	0	2	0
2	0	1	1	1	1	0
1	0	2	2	0	1	0
0	1	1	5	0	1	0
0	1	0	4	2	2	0
1	0	0	1	0	2	0
3	0	0	1	1	5	1
2	0	0	0	0	3	0
1	0	0	6	0	3	0
0	1	0	2	4	0	1
0	0	1	1	8	2	0
3	0	0	1	0	2	0
0	0	0	0	0	9	0
0	0	1	2	0	1	0
3	0	5	2	1	3	0

0.85	0.30	0.51	0.48	0.05	0.43	0.20
6	17	11	12	25	13	20
1	1	1	1	1	1	1

71	72	73	74	75	76	77
6	7	8	9	10	11	12
A_6	B_31	C_4	C_45	D_11	D_35	D_5
CHR_P	CHR_P	CHR_P	CHR_P	CHR_P	CHR_P	CHR_P
1468719	409595	1862148	4820581	198165	2718285	905697
CHR_P	CHR_P	CHR_P	CHR_P	CHR_P	CHR_P	CHR_P
807275	57543	839593	1485363	66104	922039	250879
36332	782	62135	149104	2808	110593	187485
51941	116387	66190	519025	27131	288601	83289
91415	8068	137605	290995	2601	177243	39675
63497	12377	74324	311607	21738	157984	41080

17647	76530	110612	290912	76	148973	33455
34873	17767	60894	195271	1079	87662	37482
4352	3301	13437	142980	751	19838	9389
20373	1786	45753	137089	680	116931	10484
52728	9961	80032	105854	790	50422	31476
36895	14513	52008	71950	4647	67416	9519
21912	4756	23945	89230	1976	38096	8959
21818	10040	31706	64809	394	62420	7016
21332	1245	29523	91349	10147	28410	5598
6502	16498	20456	76934	42	49422	5698
12372	391	12155	36167	4096	34739	976
5382	2884	9723	47570	42	23519	5442
10384	1475	9271	47481	1230	22958	8395
5314	2600	11194	44142	118	18192	5563
9014	4618	6833	23589	858	12258	13575
4666	1685	9605	29496	2819	13803	6319
10705	834	9464	27925	164	12294	3499
6023	3021	4524	7791	39	10145	5295
3612	673	2563	23854	103	5539	5107
2202	2687	4476	44370	52	6827	8514
8134	1409	6167	16802	977	8451	3533
5415	197	5688	14709	9351	15189	1765
1953	4048	8002	20128	78	5939	5604
2833	284	5632	14766	278	8474	2631
5088	948	4409	6685	418	8789	2520
3250	2002	5124	25164	59	9530	3332
4663	433	7383	20268	385	8809	1435
2217	1549	3256	13895	86	6943	1007
1123	1700	1982	10549	18	6747	794
1949	239	5156	13744	257	7733	2413
4119	458	6282	13928	136	4661	2640
2187	2186	3772	15343	153	5402	6796
2191	1609	4793	19198	132	3571	4428
3401	167	2424	7797	1014	2545	4355
3374	825	2996	7643	4999	4253	645
3705	78	1428	11190	4456	2903	1173
1895	471	2099	11262	934	7323	1439
2949	602	2529	6823	1404	3054	868
491	1521	2832	9459	19	3067	1973
1914	595	1373	9487	1047	3258	1950
2764	424	1740	6714	471	3847	975
1540	186	1506	3436	169	3733	841
3433	549	2982	6373	1287	4733	865
791	212	959	1740	4	1212	28
628	2941	539	9094	31	2585	920
1881	14	728	3394	682	4094	865
478	172	1546	8012	189	2108	1343
966	138	1472	4898	2738	2224	1314
1910	1599	1514	4602	26	2533	2209
747	88	651	1278	93	1729	394
213	382	207	1338	10	837	81
670	428	1397	4655	125	1954	667
733	157	936	5182	10	2506	630
1214	202	1298	5766	66	3213	1208
1512	123	765	3285	174	2007	507
1255	120	918	4397	392	1512	344
550	175	579	4681	269	2088	1090
129	106	433	1690	9	767	178
41	79	66	195	6	144	36

2392	438	1622	4366	139	2114	216
622	236	979	3131	2	1689	458
232	461	698	4507	12	1244	331
372	103	311	2696	1	1333	271
366	131	598	7405	81	781	465
411	29	1410	1546	20	395	1464
538	442	640	2261	346	1351	322
1361	140	1117	1937	218	1738	245
257	73	558	2504	4	1489	291
718	314	1035	2168	35	1279	349
314	175	376	1387	27	1345	357
816	146	433	3090	170	2575	217
409	38	665	1039	206	1059	340
256	9	366	703	87	482	428
566	56	559	2219	331	1650	185
158	30	634	2223	119	849	231
398	28	1130	1364	27	1025	146
526	20	383	1929	409	1023	133
675	36	695	1086	22	756	296
113	69	163	712	1	1101	87
177	50	251	656	18	464	305
384	2	260	1491	2711	571	138
416	160	450	947	1111	995	260
199	149	486	2576	7	980	121
312	319	474	1826	2	1036	142
560	36	356	1112	38	1129	190
337	217	370	1293	74	581	157
22	7	61	126	11	84	3
382	26	361	1762	96	710	97
192	58	236	982	7	959	86
72	245	249	1371	1	647	45
122	262	322	2157	1	996	165
265	25	197	628	139	612	227
352	160	312	944	212	510	189
296	81	185	818	165	368	332
31	66	176	1483	3	651	104
221	6	125	584	526	170	224
124	131	317	427	3	391	114
212	34	288	921	275	827	125
162	54	247	971	0	285	129
198	244	362	2134	0	396	165
126	17	173	973	3	430	109
170	48	135	921	694	331	117
91	61	165	676	9	235	206
124	68	187	919	1	501	39
106	26	179	1203	14	513	61
82	108	156	1025	6	450	256
209	24	80	588	111	440	123
186	32	85	978	413	192	183
102	24	139	459	0	286	133
94	20	137	753	20	598	144
237	9	170	525	35	436	66
96	38	83	424	4	508	102
98	105	142	472	3	425	48
98	105	142	472	3	425	48
87	14	184	1078	7	177	136
161	30	205	911	345	247	28
266	43	248	1072	4	336	87
99	22	63	492	0	491	66

109	59	87	391	8	241	219
14	5	19	131	4	94	8
165	23	173	453	28	275	32
50	13	70	132	663	112	64
153	36	121	518	30	157	53
116	2	59	244	294	79	96
113	3	63	307	45	220	69
26	2	98	106	63	36	25
151	115	106	657	27	328	79
53	5	72	222	13	160	64
84	16	81	230	128	129	25
51	11	56	185	126	198	16
83	2	89	335	30	271	96
64	4	209	327	10	250	29
152	6	83	331	45	355	35
78	19	86	246	0	174	26
74	14	93	759	107	120	36
124	5	72	219	20	75	30
43	19	128	359	19	156	30
132	11	108	661	83	257	22
86	22	41	198	1	127	37
4	11	33	200	4	62	2
148	40	193	453	51	145	22
93	7	45	250	0	169	32
50	37	51	286	0	130	44
109	66	215	314	3	195	25
104	2	50	168	109	87	35
26	25	70	227	2	159	65
31	18	81	269	3	152	29
68	6	32	427	0	519	31
80	1	39	226	13	71	19
38	14	17	782	2	215	6
54	21	99	180	74	81	19
33	1	33	86	9	69	44
27	7	33	226	50	84	19
66	23	54	297	90	84	31
102	3	89	85	103	182	16
12	3	8	50	1	34	11
43	8	33	241	95	74	42
34	10	71	269	83	94	23
43	16	32	421	1	134	39
18	4	27	182	0	121	40
112	17	97	210	65	195	12
45	3	45	176	2	51	35
29	5	33	280	265	27	33
5	37	5	101	0	56	8
109	16	217	254	11	176	12
13	32	34	234	12	25	28
49	3	111	153	3	160	21
50	3	62	150	2	124	71
22	7	61	271	2	134	61
35	7	25	277	1	80	26
63	5	45	76	186	138	19
63	5	31	178	12	208	16
16	38	44	219	2	116	36
19	4	55	227	47	44	22
44	19	33	236	19	181	19
31	2	97	131	16	123	28
15	8	45	186	0	100	19

54	0	15	158	27	117	9
12	12	61	225	0	98	24
36	4	25	91	21	78	27
45	5	56	172	107	91	7
13	7	41	172	0	98	17
88	22	25	228	2	204	11
23	13	16	168	23	108	3
17	4	33	169	5	55	18
62	2	20	48	179	2	1
1	17	29	359	0	90	16
20	1	21	177	6	78	47
48	12	24	100	4	113	21
58	5	62	99	14	65	31
23	4	16	29	13	44	13
11	19	10	81	0	31	8
38	11	95	171	4	80	21
21	1	20	186	90	55	9
27	14	63	119	18	53	34
23	0	16	85	3	50	20
32	10	13	301	1	113	49
64	0	46	104	55	28	14
24	5	28	49	32	61	36
11	1	34	106	0	54	8
80	2	15	123	22	144	3
26	3	61	118	30	56	17
43	29	35	142	0	62	31
20	1	18	140	4	134	1
60	3	54	204	21	35	5
65	4	16	57	52	35	22
15	8	30	138	2	74	21
24	22	18	123	0	142	21
35	1	53	124	63	73	17
13	9	35	108	5	122	11
23	28	32	84	26	47	30
16	8	30	74	5	52	12
21	4	25	138	5	73	15
0	0	2	16	0	13	0
32	0	41	68	0	51	4
11	1	2	11	0	34	0
35	5	61	116	22	58	9
14	3	12	59	7	24	12
25	3	33	125	24	42	8
44	6	20	152	0	129	8
21	0	16	77	22	58	13
20	2	21	43	32	59	10
37	2	42	41	24	83	3
29	2	22	58	13	27	9
17	2	25	77	0	22	10
2	7	23	71	0	43	15
26	4	27	34	15	44	18
2	9	8	44	0	21	2
24	2	16	65	40	39	4
20	3	22	69	0	40	2
30	5	22	49	97	29	7
8	2	4	35	102	24	11
10	1	4	54	0	12	13
8	27	9	117	1	43	14
25	4	22	89	3	24	1
35	2	21	140	1	82	8

16	4	23	36	5	31	1
5	2	20	71	0	24	8
10	4	2	45	0	22	15
10	4	2	45	0	22	15
22	0	6	74	3	69	5
10	0	18	70	26	31	8
5	2	10	38	11	20	10
18	6	14	94	2	115	4
9	5	16	98	0	25	9
11	3	11	78	3	36	6
15	6	14	36	3	23	4
13	0	75	106	1	56	0
21	0	11	32	78	12	2
5	10	11	102	0	23	11
13	0	21	13	5	10	8
11	5	5	63	2	39	0
11	0	19	56	0	17	7
1	2	4	11	2	9	0
20	2	23	113	5	15	8
8	6	22	36	0	24	12
6	1	12	32	11	36	8
16	1	35	48	18	41	3
8	5	20	34	0	23	11
18	0	14	32	4	49	8
7	0	8	31	23	41	2
15	5	16	68	1	52	4
15	1	14	77	14	32	4
0	0	0	0	0	2	1
8	1	2	25	73	23	5
20	2	24	48	16	22	5
18	0	8	27	32	29	5
30	1	11	112	1	49	0
10	2	13	24	0	15	7
1	0	1	32	0	11	0
13	1	3	40	3	11	10
16	0	14	91	0	31	5
4	1	14	46	0	0	0
14	0	19	30	12	22	1
5	1	30	83	2	36	1
7	1	6	24	20	7	7
7	0	3	55	5	27	2
13	0	22	52	4	29	5
30	8	11	46	1	63	3
10	0	13	29	20	21	2
13	0	6	31	14	24	3
11	1	19	22	4	20	8
15	0	11	81	0	28	4
17	1	10	145	1	51	0
0	2	19	76	0	2	4
5	13	4	24	0	28	2
0	2	5	10	1	20	25
14	0	4	20	17	14	1
5	2	8	33	0	20	8
18	5	1	71	3	69	0
14	3	10	33	9	41	4
1	6	12	75	0	20	3
20	2	12	39	2	12	7
6	1	9	28	10	25	2
13	3	5	31	46	6	3

7	18	5	242	0	76	2
10	0	2	41	2	49	1
9	0	6	28	1	14	5
11	0	8	22	33	22	2
11	3	13	40	18	52	3
11	6	11	48	1	19	7
5	2	12	48	5	9	2
20	4	7	39	17	24	1
5	0	5	28	33	17	5
8	0	5	35	26	9	2
7	9	15	37	0	27	7
7	0	9	27	5	13	5
1	3	1	18	0	15	2
2	1	6	13	0	14	6
10	13	18	37	1	22	5
3	6	19	31	0	15	5
16	0	10	53	17	13	4
6	2	7	25	0	9	5
8	0	5	48	15	12	7
6	14	4	21	1	19	2
9	0	8	32	6	18	1
6	1	8	16	0	12	2
7	4	12	17	2	18	5
12	0	2	21	2	23	0
12	0	6	16	3	17	3
12	0	6	37	1	19	2
0	2	7	49	13	28	0
4	0	6	15	5	9	1
10	1	9	34	0	8	3
4	0	8	42	19	5	2
9	0	2	32	1	24	9
10	1	7	26	9	5	2
2	1	0	4	1	5	2
7	5	7	9	10	14	2
2	3	2	15	0	15	2
3	1	11	17	2	15	3
1	1	10	27	0	9	1
7	3	4	20	2	13	1
0	0	5	4	0	2	1
17	0	8	17	2	19	3
6	4	13	14	0	11	1
5	4	5	31	2	14	2
3	0	15	14	3	5	3
3	1	8	33	1	17	3
6	1	7	10	6	2	5
5	1	1	15	1	11	3
5	1	4	37	7	14	2
3	2	4	10	0	24	3
6	0	12	23	0	8	2
6	2	6	20	1	15	4
5	2	7	28	14	5	4
2	0	10	38	0	5	0
1	1	7	23	0	11	3
0	1	0	7	0	6	1
2	2	5	20	2	9	0
7	0	4	36	3	10	2
4	0	3	40	0	19	2
5	0	0	12	5	10	0
3	1	0	19	0	4	1

6	0	5	12	4	5	2
3	0	2	12	0	10	9
3	0	2	17	3	4	8
1	1	3	8	1	10	1
2	2	6	21	1	13	0
5	0	5	9	11	8	7
6	1	6	12	4	9	2
0	3	4	58	0	9	0
1	0	3	17	0	5	1
5	0	2	15	5	10	4
2	0	5	9	0	6	4
3	1	6	16	1	2	1
4	1	2	21	0	4	5
2	0	1	12	1	6	0
4	1	4	10	2	21	1
1	5	1	14	0	6	2
7	1	2	15	0	7	0
1	0	1	14	1	8	9
7	0	2	5	10	10	0
6	0	2	12	4	9	0
6	1	2	6	0	8	2
1	1	1	8	2	4	2
5	0	0	11	2	23	3
5	1	3	11	0	14	2
4	0	8	9	1	12	0
2	1	2	10	7	2	3
0	0	3	28	1	3	6
3	0	4	13	3	8	0
2	1	1	16	7	8	0
1	1	5	31	0	5	1
3	0	0	1	0	2	0
8	1	2	11	8	8	0
2	0	1	23	1	17	0
1	5	0	10	0	6	1
7	0	0	8	15	1	3
3	2	4	13	0	9	2
0	2	4	7	0	3	2
0	0	3	7	3	7	4
5	0	4	10	2	6	3
0	0	0	8	4	5	1
4	1	12	17	0	12	1
1	0	5	10	1	8	0
0	2	3	3	0	8	4
1	3	2	11	0	5	0
4	1	3	3	5	6	1
3	0	0	5	5	6	2
1	0	3	16	3	9	1
1	1	0	11	0	8	0
0	1	2	4	1	6	0
5	1	4	8	0	4	1
4	5	1	4	0	11	1
1	0	0	3	0	1	0
2	0	0	0	0	0	0
3	1	1	12	0	5	1
3	0	4	9	23	3	2
3	0	3	8	5	2	0
1	2	5	7	7	3	1
1	0	2	4	0	7	0
0	1	0	2	0	0	0

0	0	2	26	0	8	2
0	0	3	11	1	11	1
0	0	1	13	0	1	1
6	0	1	5	1	9	1
2	0	3	11	2	7	0
6	0	4	7	4	8	1
0	2	0	6	0	2	0
1	0	1	8	0	4	0
2	2	1	20	0	6	0
1	0	3	8	1	5	1
2	0	0	11	0	3	2
0	2	0	4	0	2	0
4	1	0	8	0	5	2
2	0	4	4	1	5	3
4	0	1	6	2	9	0
5	1	5	8	0	3	1
0	0	1	5	1	3	1
6	0	2	6	5	3	0
4	0	6	6	2	9	0
3	0	0	12	0	2	2
7	1	0	19	0	19	2
1	0	0	3	0	7	0
4	2	2	7	0	6	1
0	0	2	4	0	7	3
0	0	3	4	0	7	1
5	0	2	9	1	5	0
2	0	2	10	0	2	0
2	0	7	2	0	1	2
0	0	1	7	5	2	3
4	0	2	3	2	0	0
2	0	2	10	2	5	0
1	0	1	8	0	4	3
0	0	8	6	0	6	0
3	0	0	18	5	2	0
3	0	3	9	0	3	1
1	0	0	5	0	3	2
0	0	2	6	0	0	0
1	0	2	6	2	4	2
3	0	1	4	3	7	1
2	0	0	4	2	1	0
1	1	0	9	0	1	0
1	0	2	8	0	2	0
2	0	1	2	0	4	1
3	0	2	8	2	1	0
0	0	0	3	0	2	0
1	0	3	10	0	6	0
2	1	1	4	3	3	0
0	0	0	3	0	0	0
1	0	1	3	0	3	1
0	0	1	19	0	5	1
7	0	6	9	1	14	0
2	1	1	15	1	1	1
0	1	1	5	0	6	0
1	0	2	0	8	3	0
3	0	2	7	0	7	0
1	0	4	5	0	2	3
1	0	0	2	0	1	0
0	0	0	2	0	2	0
0	0	3	1	0	2	0

1	0	4	7	1	0	2
2	0	0	10	0	2	0
0	1	1	3	0	2	1
0	3	2	4	0	2	1
0	0	1	7	3	4	1
1	1	1	2	2	3	1
0	0	1	12	0	6	1
1	2	0	2	3	3	1
0	0	1	2	3	3	1
0	0	0	1	0	0	0
0	0	3	2	0	0	1
0	0	1	15	0	7	2
2	0	1	4	1	0	0
1	0	4	8	0	3	0
2	0	0	1	2	2	0
0	0	1	10	1	2	1
1	0	1	7	1	0	0
0	0	2	3	6	2	0
1	1	0	7	0	3	1
0	0	3	4	1	0	1
1	0	0	4	6	1	0
1	3	2	7	0	4	1
1	0	0	6	3	0	0
1	0	1	2	0	3	0
2	0	1	1	0	2	0
0	0	1	2	0	3	1
1	0	4	5	0	2	0
2	1	0	1	0	1	0
1	0	4	6	0	3	2
1	0	0	5	1	5	1
3	0	0	6	0	3	1
4	0	1	3	1	3	0
0	0	0	15	0	2	1
2	0	0	12	0	1	0
6	1	0	0	0	1	0
0	3	3	7	1	3	0
0	3	3	7	0	5	2
3	0	0	0	0	2	0
2	0	1	1	1	1	0
1	0	2	2	0	1	0
0	1	1	5	0	1	0
0	1	0	4	2	2	0
1	0	0	1	0	2	0
3	0	0	1	1	5	1
2	0	0	0	0	3	0
1	0	0	6	0	3	0
0	1	0	2	4	0	1
0	0	1	1	8	2	0
3	0	0	1	0	2	0
0	0	0	0	0	9	0
0	0	1	2	0	1	0
3	0	5	2	1	3	0
46066	12644	58597	147598	5386	84439	28007
145355	25766	151512	278214	13091	171360	55494
403	446	402	315	453	358	421
CHR_P	CHR_P	CHR_P	CHR_P	CHR_P	CHR_P	CHR_P
CHR_P	CHR_P	CHR_P	CHR_P	CHR_P	CHR_P	CHR_P

71	72	73	74	75	76	77
6	7	8	9	10	11	12
CHR_P	CHR_P	CHR_P	CHR_P	CHR_P	CHR_P	CHR_P
A_6	B_31	C_4	C_45	D_11	D_35	D_5
1468719	409595	1862148	4820581	198165	2718285	905697
CHR_P	CHR_P	CHR_P	CHR_P	CHR_P	CHR_P	CHR_P
17.52	4.55	14.33	10.06	12.27	10.92	8.96
0.79	0.06	1.06	1.01	0.52	1.31	6.69
1.13	9.21	1.13	3.52	5.04	3.42	2.97
1.98	0.64	2.35	1.97	0.48	2.10	1.42
1.38	0.98	1.27	2.11	4.04	1.87	1.47
0.38	6.05	1.89	1.97	0.01	1.76	1.19
0.76	1.41	1.04	1.32	0.20	1.04	1.34
0.09	0.26	0.23	0.97	0.14	0.23	0.34
0.44	0.14	0.78	0.93	0.13	1.38	0.37
1.14	0.79	1.37	0.72	0.15	0.60	1.12
0.80	1.15	0.89	0.49	0.86	0.80	0.34
0.48	0.38	0.41	0.60	0.37	0.45	0.32
0.47	0.79	0.54	0.44	0.07	0.74	0.25
0.46	0.10	0.50	0.62	1.88	0.34	0.20
0.14	1.30	0.35	0.52	0.01	0.59	0.20
0.27	0.03	0.21	0.25	0.76	0.41	0.03
0.12	0.23	0.17	0.32	0.01	0.28	0.19
0.23	0.12	0.16	0.32	0.23	0.27	0.30
0.12	0.21	0.19	0.30	0.02	0.22	0.20
0.20	0.37	0.12	0.16	0.16	0.15	0.48
0.10	0.13	0.16	0.20	0.52	0.16	0.23
0.23	0.07	0.16	0.19	0.03	0.15	0.12
0.13	0.24	0.08	0.05	0.01	0.12	0.19
0.08	0.05	0.04	0.16	0.02	0.07	0.18
0.05	0.21	0.08	0.30	0.01	0.08	0.30
0.18	0.11	0.11	0.11	0.18	0.10	0.13
0.12	0.02	0.10	0.10	1.74	0.18	0.06
0.04	0.32	0.14	0.14	0.01	0.07	0.20
0.06	0.02	0.10	0.10	0.05	0.10	0.09
0.11	0.07	0.08	0.05	0.08	0.10	0.09
0.07	0.16	0.09	0.17	0.01	0.11	0.12
0.10	0.03	0.13	0.14	0.07	0.10	0.05
0.05	0.12	0.06	0.09	0.02	0.08	0.04
0.02	0.13	0.03	0.07	0.00	0.08	0.03
0.04	0.02	0.09	0.09	0.05	0.09	0.09
0.09	0.04	0.11	0.09	0.03	0.06	0.09
0.05	0.17	0.06	0.10	0.03	0.06	0.24
0.05	0.13	0.08	0.13	0.02	0.04	0.16
0.07	0.01	0.04	0.05	0.19	0.03	0.16
0.07	0.07	0.05	0.05	0.93	0.05	0.02
0.08	0.01	0.02	0.08	0.83	0.03	0.04
0.04	0.04	0.04	0.08	0.17	0.09	0.05
0.06	0.05	0.04	0.05	0.26	0.04	0.03
0.01	0.12	0.05	0.06	0.00	0.04	0.07
0.04	0.05	0.02	0.06	0.19	0.04	0.07
0.06	0.03	0.03	0.05	0.09	0.05	0.03
0.03	0.01	0.03	0.02	0.03	0.04	0.03
0.07	0.04	0.05	0.04	0.24	0.06	0.03
0.02	0.02	0.02	0.01	0.00	0.01	0.00
0.01	0.23	0.01	0.06	0.01	0.03	0.03
0.04	0.00	0.01	0.02	0.13	0.05	0.03
0.01	0.01	0.03	0.05	0.04	0.02	0.05

0.02	0.01	0.03	0.03	0.51	0.03	0.05
0.04	0.13	0.03	0.03	0.00	0.03	0.08
0.02	0.01	0.01	0.01	0.02	0.02	0.01
0.00	0.03	0.00	0.01	0.00	0.01	0.00
0.01	0.03	0.02	0.03	0.02	0.02	0.02
0.02	0.01	0.02	0.04	0.00	0.03	0.02
0.03	0.02	0.02	0.04	0.01	0.04	0.04
0.03	0.01	0.01	0.02	0.03	0.02	0.02
0.03	0.01	0.02	0.03	0.07	0.02	0.01
0.01	0.01	0.01	0.03	0.05	0.02	0.04
0.00	0.01	0.01	0.01	0.00	0.01	0.01
0.00	0.01	0.00	0.00	0.00	0.00	0.00
0.05	0.03	0.03	0.03	0.03	0.03	0.01
0.01	0.02	0.02	0.02	0.00	0.02	0.02
0.01	0.04	0.01	0.03	0.00	0.01	0.01
0.01	0.01	0.01	0.02	0.00	0.02	0.01
0.01	0.01	0.01	0.05	0.02	0.01	0.02
0.01	0.00	0.02	0.01	0.00	0.00	0.05
0.01	0.03	0.01	0.02	0.06	0.02	0.01
0.03	0.01	0.02	0.01	0.04	0.02	0.01
0.01	0.01	0.01	0.02	0.00	0.02	0.01
0.02	0.02	0.02	0.01	0.01	0.02	0.01
0.01	0.01	0.01	0.01	0.01	0.02	0.01
0.02	0.01	0.01	0.02	0.03	0.03	0.01
0.01	0.00	0.01	0.01	0.04	0.01	0.01
0.01	0.00	0.01	0.00	0.02	0.01	0.02
0.01	0.00	0.01	0.02	0.06	0.02	0.01
0.00	0.00	0.01	0.02	0.02	0.01	0.01
0.01	0.00	0.02	0.01	0.01	0.01	0.01
0.01	0.00	0.01	0.01	0.08	0.01	0.00
0.01	0.00	0.01	0.01	0.00	0.01	0.01
0.00	0.01	0.00	0.00	0.00	0.01	0.00
0.00	0.00	0.00	0.00	0.00	0.01	0.01
0.01	0.00	0.00	0.01	0.50	0.01	0.00
0.01	0.01	0.01	0.01	0.21	0.01	0.01
0.00	0.01	0.01	0.02	0.00	0.01	0.00
0.01	0.03	0.01	0.01	0.00	0.01	0.01
0.01	0.00	0.01	0.01	0.01	0.01	0.01
0.01	0.02	0.01	0.01	0.01	0.01	0.01
0.00	0.00	0.00	0.00	0.00	0.00	0.00
0.01	0.00	0.01	0.01	0.02	0.01	0.00
0.00	0.00	0.00	0.01	0.00	0.01	0.00
0.00	0.02	0.00	0.01	0.00	0.01	0.00
0.00	0.02	0.01	0.01	0.00	0.01	0.01
0.01	0.00	0.00	0.00	0.03	0.01	0.01
0.01	0.01	0.01	0.01	0.04	0.01	0.01
0.01	0.01	0.00	0.01	0.03	0.00	0.01
0.00	0.01	0.00	0.01	0.00	0.01	0.00
0.00	0.00	0.00	0.00	0.10	0.00	0.01
0.00	0.01	0.01	0.00	0.00	0.00	0.00
0.00	0.00	0.00	0.01	0.05	0.01	0.00
0.00	0.00	0.00	0.01	0.00	0.00	0.00
0.00	0.02	0.01	0.01	0.00	0.00	0.01
0.00	0.00	0.00	0.01	0.00	0.01	0.00
0.00	0.00	0.00	0.01	0.13	0.00	0.00
0.00	0.00	0.00	0.00	0.00	0.00	0.01
0.00	0.01	0.00	0.01	0.00	0.01	0.00
0.00	0.00	0.00	0.01	0.00	0.01	0.00
0.00	0.01	0.00	0.01	0.00	0.01	0.01

0.00	0.00	0.00	0.00	0.02	0.01	0.00
0.00	0.00	0.00	0.01	0.08	0.00	0.01
0.00	0.00	0.00	0.00	0.00	0.00	0.00
0.00	0.00	0.00	0.01	0.00	0.01	0.01
0.01	0.00	0.00	0.00	0.01	0.01	0.00
0.00	0.00	0.00	0.00	0.00	0.01	0.00
0.00	0.01	0.00	0.00	0.00	0.01	0.00
0.00	0.01	0.00	0.00	0.00	0.01	0.00
0.00	0.00	0.00	0.01	0.00	0.00	0.00
0.00	0.00	0.00	0.01	0.06	0.00	0.00
0.01	0.00	0.00	0.01	0.00	0.00	0.00
0.00	0.00	0.00	0.00	0.00	0.01	0.00
0.00	0.00	0.00	0.00	0.00	0.00	0.01
0.00	0.00	0.00	0.00	0.00	0.00	0.00
0.00	0.00	0.00	0.00	0.01	0.00	0.00
0.00	0.00	0.00	0.00	0.12	0.00	0.00
0.00	0.00	0.00	0.00	0.01	0.00	0.00
0.00	0.00	0.00	0.00	0.05	0.00	0.00
0.00	0.00	0.00	0.00	0.01	0.00	0.00
0.00	0.00	0.00	0.00	0.01	0.00	0.00
0.00	0.01	0.00	0.00	0.01	0.00	0.00
0.00	0.00	0.00	0.00	0.00	0.00	0.00
0.00	0.00	0.00	0.00	0.02	0.00	0.00
0.00	0.00	0.00	0.00	0.02	0.00	0.00
0.00	0.00	0.00	0.00	0.01	0.00	0.00
0.00	0.00	0.00	0.00	0.00	0.00	0.00
0.00	0.00	0.00	0.00	0.01	0.00	0.00
0.00	0.00	0.00	0.00	0.00	0.00	0.00
0.00	0.00	0.00	0.01	0.02	0.00	0.00
0.00	0.00	0.00	0.00	0.00	0.00	0.00
0.00	0.00	0.00	0.00	0.00	0.00	0.00
0.00	0.00	0.00	0.00	0.02	0.00	0.00
0.00	0.00	0.00	0.00	0.00	0.00	0.00
0.00	0.00	0.00	0.00	0.00	0.00	0.00
0.00	0.00	0.00	0.00	0.01	0.00	0.00
0.00	0.00	0.00	0.00	0.00	0.00	0.00
0.00	0.00	0.00	0.00	0.00	0.00	0.00
0.00	0.01	0.00	0.00	0.00	0.00	0.00
0.00	0.00	0.00	0.00	0.02	0.00	0.00
0.00	0.00	0.00	0.00	0.00	0.00	0.00
0.00	0.00	0.00	0.00	0.00	0.00	0.00
0.00	0.00	0.00	0.00	0.00	0.01	0.00
0.00	0.00	0.00	0.00	0.00	0.00	0.00
0.00	0.00	0.00	0.01	0.00	0.00	0.00
0.00	0.00	0.00	0.00	0.01	0.00	0.00
0.00	0.00	0.00	0.00	0.00	0.00	0.00
0.00	0.00	0.00	0.00	0.01	0.00	0.00
0.00	0.00	0.00	0.00	0.02	0.00	0.00
0.00	0.00	0.00	0.00	0.02	0.00	0.00
0.00	0.00	0.00	0.00	0.00	0.00	0.00
0.00	0.00	0.00	0.00	0.02	0.00	0.00
0.00	0.00	0.00	0.00	0.02	0.00	0.00
0.00	0.00	0.00	0.00	0.00	0.00	0.00
0.00	0.00	0.00	0.00	0.00	0.00	0.00
0.00	0.00	0.00	0.00	0.01	0.00	0.00
0.00	0.00	0.00	0.00	0.00	0.00	0.00
0.00	0.00	0.00	0.00	0.05	0.00	0.00
0.00	0.00	0.00	0.00	0.00	0.00	0.00
0.00	0.00	0.00	0.00	0.00	0.00	0.00

0.00	0.00	0.00	0.00	0.00	0.00	0.00
0.00	0.00	0.00	0.00	0.00	0.00	0.00
0.00	0.00	0.00	0.00	0.00	0.00	0.00
0.00	0.00	0.00	0.00	0.00	0.00	0.00
0.00	0.00	0.00	0.00	0.00	0.00	0.00
0.00	0.00	0.00	0.00	0.03	0.00	0.00
0.00	0.00	0.00	0.00	0.00	0.00	0.00
0.00	0.00	0.00	0.00	0.00	0.00	0.00
0.00	0.00	0.00	0.00	0.01	0.00	0.00
0.00	0.00	0.00	0.00	0.00	0.00	0.00
0.00	0.00	0.00	0.00	0.00	0.00	0.00
0.00	0.00	0.00	0.00	0.00	0.00	0.00
0.00	0.00	0.00	0.00	0.01	0.00	0.00
0.00	0.00	0.00	0.00	0.00	0.00	0.00
0.00	0.00	0.00	0.00	0.00	0.00	0.00
0.00	0.00	0.00	0.00	0.02	0.00	0.00
0.00	0.00	0.00	0.00	0.00	0.00	0.00
0.00	0.00	0.00	0.00	0.00	0.00	0.00
0.00	0.00	0.00	0.00	0.00	0.00	0.00
0.00	0.00	0.00	0.00	0.00	0.00	0.00
0.00	0.00	0.00	0.00	0.03	0.00	0.00
0.00	0.00	0.00	0.00	0.00	0.00	0.00
0.00	0.00	0.00	0.00	0.00	0.00	0.00
0.00	0.00	0.00	0.00	0.00	0.00	0.00
0.00	0.00	0.00	0.00	0.00	0.00	0.00
0.00	0.00	0.00	0.00	0.00	0.00	0.00
0.00	0.00	0.00	0.00	0.00	0.00	0.00
0.00	0.00	0.00	0.00	0.00	0.00	0.00
0.00	0.00	0.00	0.00	0.02	0.00	0.00
0.00	0.00	0.00	0.00	0.00	0.00	0.00
0.00	0.00	0.00	0.00	0.00	0.00	0.00
0.00	0.00	0.00	0.00	0.00	0.00	0.00
0.00	0.00	0.00	0.00	0.01	0.00	0.00
0.00	0.00	0.00	0.00	0.01	0.00	0.00
0.00	0.00	0.00	0.00	0.00	0.00	0.00
0.00	0.00	0.00	0.00	0.00	0.00	0.00
0.00	0.00	0.00	0.00	0.01	0.00	0.00
0.00	0.00	0.00	0.00	0.00	0.00	0.00
0.00	0.00	0.00	0.00	0.00	0.00	0.00
0.00	0.00	0.00	0.00	0.00	0.00	0.00
0.00	0.00	0.00	0.00	0.01	0.00	0.00
0.00	0.00	0.00	0.00	0.00	0.00	0.00
0.00	0.00	0.00	0.00	0.00	0.00	0.00
0.00	0.00	0.00	0.00	0.01	0.00	0.00
0.00	0.00	0.00	0.00	0.00	0.00	0.00
0.00	0.00	0.00	0.00	0.00	0.00	0.00
0.00	0.00	0.00	0.00	0.00	0.00	0.00
0.00	0.00	0.00	0.00	0.00	0.00	0.00
0.00	0.00	0.00	0.00	0.00	0.00	0.00
0.00	0.00	0.00	0.00	0.00	0.00	0.00
0.00	0.00	0.00	0.00	0.00	0.00	0.00
0.00	0.00	0.00	0.00	0.00	0.00	0.00
0.00	0.00	0.00	0.00	0.00	0.00	0.00
0.00	0.00	0.00	0.00	0.00	0.00	0.00
0.00	0.00	0.00	0.00	0.00	0.00	0.00
0.00	0.00	0.00	0.00	0.00	0.00	0.00
0.00	0.00	0.00	0.00	0.01	0.00	0.00
0.00	0.00	0.00	0.00	0.00	0.00	0.00
0.00	0.00	0.00	0.00	0.00	0.00	0.00

0.00	0.00	0.00	0.00	0.00	0.00	0.00
0.00	0.00	0.00	0.00	0.00	0.00	0.00
0.00	0.00	0.00	0.00	0.00	0.00	0.00
0.00	0.00	0.00	0.00	0.00	0.00	0.00
0.00	0.00	0.00	0.00	0.01	0.00	0.00
0.00	0.00	0.00	0.00	0.00	0.00	0.00
0.00	0.00	0.00	0.00	0.02	0.00	0.00
0.00	0.00	0.00	0.00	0.02	0.00	0.00
0.00	0.00	0.00	0.00	0.00	0.00	0.00
0.00	0.00	0.00	0.00	0.00	0.00	0.00
0.00	0.00	0.00	0.00	0.00	0.00	0.00
0.00	0.00	0.00	0.00	0.00	0.00	0.00
0.00	0.00	0.00	0.00	0.00	0.00	0.00
0.00	0.00	0.00	0.00	0.00	0.00	0.00
0.00	0.00	0.00	0.00	0.00	0.00	0.00
0.00	0.00	0.00	0.00	0.00	0.00	0.00
0.00	0.00	0.00	0.00	0.00	0.00	0.00
0.00	0.00	0.00	0.00	0.00	0.00	0.00
0.00	0.00	0.00	0.00	0.00	0.00	0.00
0.00	0.00	0.00	0.00	0.00	0.00	0.00
0.00	0.00	0.00	0.00	0.00	0.00	0.00
0.00	0.00	0.00	0.00	0.00	0.00	0.00
0.00	0.00	0.00	0.00	0.00	0.00	0.00
0.00	0.00	0.00	0.00	0.00	0.00	0.00
0.00	0.00	0.00	0.00	0.01	0.00	0.00
0.00	0.00	0.00	0.00	0.00	0.00	0.00
0.00	0.00	0.00	0.00	0.00	0.00	0.00
0.00	0.00	0.00	0.00	0.00	0.00	0.00
0.00	0.00	0.00	0.00	0.00	0.00	0.00
0.00	0.00	0.00	0.00	0.00	0.00	0.00

71	72	73	74	75	76	77
6<br>CHR_P<br>A_6<br>1468719	8<br>CHR_P<br>B_31<br>409595	9<br>CHR_P<br>C_4<br>1862148	10<br>CHR_P<br>C_45<br>4820581	11<br>CHR_P<br>D_11<br>198165	12<br>CHR_P<br>D_35<br>2718285	13<br>CHR_P<br>D_5<br>905697
A_6	B_31	C_4	C_45	D_11	D_35	D_5
1.33	-1.59	0.61	-0.35	0.15	-0.16	-0.60
-0.73	-0.94	-0.65	-0.66	-0.81	-0.57	1.00
-0.74	5.34	-0.74	1.06	2.20	0.99	0.65
-0.46	-1.79	-0.10	-0.47	-1.94	-0.35	-1.02
-0.87	-1.31	-0.99	-0.06	2.07	-0.32	-0.77
-0.73	4.40	0.63	0.70	-1.07	0.52	0.00
-0.65	0.38	-0.20	0.25	-1.54	-0.20	0.27
-0.45	-0.15	-0.20	1.13	-0.37	-0.19	-0.01

-0.29	-0.89	0.38	0.67	-0.92	1.58	-0.43
0.99	0.07	1.55	-0.11	-1.57	-0.42	0.93
1.24	2.54	1.56	0.06	1.47	1.23	-0.49
-0.14	-1.03	-0.74	1.01	-1.12	-0.36	-1.54
0.67	2.51	1.06	0.47	-1.64	2.20	-0.62
-0.02	-2.12	0.21	0.87	8.15	-0.75	-1.54
-0.36	4.99	0.60	1.39	-0.97	1.68	-0.07
-0.12	-1.16	-0.39	-0.22	2.03	0.50	-1.14
-0.26	0.45	0.05	1.04	-0.95	0.76	0.23
0.11	-0.96	-0.55	1.06	0.14	0.57	0.84
-0.59	0.22	0.08	1.05	-1.42	0.30	0.15
-0.35	1.34	-1.13	-0.70	-0.71	-0.85	2.53
-0.90	-0.39	0.11	0.69	5.89	0.10	1.10
1.24	-1.31	0.15	0.58	-1.85	-0.09	-0.41
0.15	1.49	-0.51	-0.82	-1.38	0.02	0.87
-0.39	-0.62	-0.71	0.40	-0.94	-0.51	0.60
-0.49	0.49	-0.32	1.01	-0.71	-0.29	1.03
1.16	-0.07	-0.18	-0.02	1.25	-0.28	0.21
-0.33	-1.95	-0.66	-0.61	25.44	0.66	-1.20
-0.68	1.99	0.23	0.23	-0.94	-0.41	0.84
-0.89	-1.65	-0.22	-0.14	-1.08	-0.13	-0.26
0.19	-0.63	-0.63	-1.33	-0.57	0.05	-0.28
-0.47	1.58	-0.07	1.86	-1.86	0.52	0.66
-0.40	-1.70	0.08	0.30	-0.98	-0.34	-1.37
-0.17	0.95	-0.06	0.52	-0.66	0.34	-0.36
-0.27	0.34	-0.21	-0.01	-0.38	0.04	-0.24
-1.17	-1.69	-0.15	-0.03	-1.05	-0.07	-0.19
-0.33	-0.65	-0.22	-0.30	-0.72	-0.54	-0.30
-0.78	0.70	-0.58	-0.11	-1.00	-0.58	1.53
-0.46	0.36	-0.11	0.39	-0.70	-0.52	0.68
-0.31	-1.03	-0.70	-0.56	1.07	-0.83	0.67
-0.21	-0.28	-0.39	-0.39	6.68	-0.40	-0.62
0.09	-1.43	-1.05	0.00	15.37	-0.85	-0.70
-0.83	-0.94	-0.99	0.23	3.15	0.54	-0.52
-0.01	-0.67	-0.85	-0.73	7.94	-1.14	-1.35
-0.67	2.05	0.27	0.66	-0.84	-0.03	0.82
-0.45	-0.33	-0.83	0.04	2.81	-0.51	0.15
0.98	-0.94	-1.21	-0.07	2.97	-0.06	-0.84
-0.30	-1.05	-0.61	-0.71	-0.38	0.14	-0.43
0.83	-0.21	0.04	-0.21	6.29	0.21	-0.62
0.20	0.19	0.18	0.01	-0.37	0.10	-0.37
-0.38	4.63	-0.48	0.72	-0.56	0.01	0.06
0.29	-1.85	-1.24	-0.67	4.92	0.71	-0.24
-0.70	-0.61	-0.23	0.58	0.02	-0.27	0.40
-1.14	-1.92	-0.82	-0.19	36.59	-0.72	0.87
0.65	5.13	-0.17	0.11	-1.28	0.05	2.62
-0.49	-1.00	-0.77	-0.90	-0.43	-0.25	-0.60

-0.19	0.85	-0.24	-0.01	-0.31	0.02	-0.26
-1.09	0.47	-0.34	0.28	-0.39	-0.40	-0.34
-0.67	-0.96	-0.67	0.92	-1.84	0.47	-0.13
-0.31	-1.09	-0.63	0.63	-1.36	0.56	0.93
-0.21	-0.82	-0.73	-0.49	-0.23	-0.45	-0.60
0.21	-1.21	-0.72	0.42	3.88	-0.54	-0.99
-0.84	-0.68	-1.01	0.80	2.31	0.22	1.40
-0.51	-0.07	-0.15	0.17	-0.60	-0.02	-0.23
-0.26	-0.01	-0.24	-0.24	-0.25	-0.22	-0.24
1.30	0.30	-0.10	0.01	-0.21	-0.25	-1.25
-0.29	0.21	0.02	0.45	-1.55	0.34	-0.02
-0.52	1.79	-0.01	1.35	-0.72	0.19	-0.02
-0.23	-0.22	-0.51	0.78	-1.01	0.53	-0.07
-0.55	-0.27	-0.29	4.27	0.26	-0.40	0.44
-0.31	-0.40	-0.12	-0.29	-0.38	-0.37	0.25
-0.70	0.94	-0.75	-0.44	3.01	-0.39	-0.71
0.90	-0.92	-0.13	-0.72	1.98	0.02	-1.15
-0.42	-0.40	-0.05	0.64	-0.87	0.71	0.03
0.23	1.44	0.50	0.11	-0.97	0.17	-0.19
-0.52	0.35	-0.57	-0.20	-0.75	0.61	0.21
0.04	-0.47	-0.81	0.31	1.19	1.10	-0.78
-0.68	-1.39	-0.38	-0.90	2.88	-0.24	-0.29
-0.41	-0.54	-0.40	-0.43	-0.14	-0.41	-0.16
-0.49	-1.21	-0.74	-0.23	4.02	0.18	-1.01
-1.06	-1.16	-0.37	0.03	0.68	-0.44	-0.61
-0.47	-1.73	1.62	-0.35	-1.18	0.22	-1.14
-0.13	-1.53	-0.82	0.11	9.10	-0.03	-1.08
-0.17	-1.26	-0.43	-0.85	-1.15	-0.70	-0.55
-0.40	-0.26	-0.38	-0.29	-0.50	0.10	-0.37
-0.69	-0.67	-0.61	-0.59	-0.77	-0.41	0.50
-0.08	-1.53	-0.77	0.24	87.60	-0.36	-0.68
-0.83	-0.10	-1.10	-1.35	38.57	-0.28	-0.78
-0.30	0.94	0.36	1.87	-0.79	0.91	-0.30
-0.42	4.46	-0.07	1.06	-2.12	1.03	-0.87
0.04	-1.12	-0.72	-0.53	-0.59	0.19	-0.63
-0.16	2.38	-0.41	0.21	1.49	-0.27	-0.60
-0.23	-0.21	-0.12	-0.16	0.07	-0.13	-0.30
0.44	-1.22	-0.13	1.41	2.97	0.47	-0.85
-0.32	-0.24	-0.35	0.18	-0.90	1.13	-0.54
-0.40	2.99	0.11	1.07	-0.66	0.76	-0.39
-0.66	2.93	-0.10	1.72	-1.15	1.15	-0.02
-0.51	-1.41	-1.08	-0.87	4.27	-0.16	0.05
0.27	2.19	-0.61	-0.20	12.40	-0.34	-0.07
-0.22	-0.22	-1.00	-0.43	5.57	-0.71	1.08
-0.66	0.38	-0.12	1.49	-0.69	0.96	0.04
-0.18	-0.93	-0.65	-0.33	15.97	-0.67	0.37
-0.93	2.17	0.17	-0.85	-1.79	-0.15	-0.37
-0.63	-1.04	-0.56	-0.28	9.27	0.48	-0.66

-0.40	-0.34	-0.35	-0.15	-0.70	-0.42	-0.31
-0.10	4.86	0.52	3.26	-1.53	0.03	0.42
-0.19	-0.59	-0.13	0.92	-0.82	0.49	0.14
-0.52	-0.49	-0.94	0.25	37.19	-0.45	-0.37
-0.68	-0.27	-0.56	-0.30	-0.73	-0.56	0.10
-0.34	0.51	-0.18	0.78	-1.13	0.69	-0.75
-0.47	-0.56	-0.18	1.75	-0.36	0.96	-0.52
-0.87	1.52	-0.56	0.95	-1.10	0.38	1.73
-0.08	-1.41	-1.68	-0.36	8.02	0.26	-0.16
-0.03	-0.52	-0.88	0.83	23.97	-0.61	0.80
-0.43	-0.56	-0.37	-0.07	-1.33	0.04	0.59
-0.72	-0.89	-0.61	0.43	-0.09	1.16	0.44
0.14	-1.78	-0.83	-0.55	0.72	0.14	-1.07
-0.71	-0.12	-1.13	-0.20	-1.56	1.81	0.29
-0.45	2.16	-0.33	0.00	-1.12	0.77	-0.63
-0.45	2.16	-0.33	0.00	-1.12	0.77	-0.63
-0.68	-0.90	-0.34	0.82	-0.85	-0.63	0.14
-0.02	-0.43	-0.02	0.94	21.70	-0.23	-0.92
1.47	0.02	0.53	2.38	-1.60	0.37	-0.16
-0.18	-0.33	-0.57	0.24	-0.96	1.14	-0.11
-0.15	0.88	-0.54	-0.02	-0.54	0.07	2.29
-0.28	-0.25	-0.27	-0.07	-0.12	0.01	-0.29
-0.12	-0.88	-0.39	-0.34	0.58	-0.26	-1.17
-0.85	-0.88	-0.80	-0.95	59.63	-0.73	-0.26
0.20	-0.05	-0.45	0.29	1.36	-0.56	-0.54
-0.27	-1.10	-0.80	-0.57	17.98	-0.82	0.05
-0.26	-0.76	-0.57	-0.34	1.07	-0.22	-0.26
-0.52	-0.69	-0.06	-0.46	4.14	-0.58	-0.38
0.84	5.37	-0.31	1.75	2.19	1.31	0.48
-0.72	-1.25	-0.66	-0.47	0.17	-0.19	0.08
-0.39	-0.61	-0.56	-0.49	8.27	-0.51	-0.76
-0.23	-0.36	-0.31	-0.14	12.56	0.48	-0.53
-0.43	-1.87	-0.68	-0.02	2.86	0.80	0.99

34	1	2	3	4	5	6
70	72	73	74	75	76	77
TAAAGTGCTTAT	TGGCTCAGTTCA	TATGGCACTGGT	CTATACGACCTG	CGGGGCAGCTCA	TGAGGTAGTAA	CATGCCTTGAGT
miR-20a-5p	miR-24-3p	miR-183-5p	let-7d-3p	miR-486-3p	miR-98-5p	miR-532-5p
79678	78156	75744	71988	70256	69114	68018
-0.66	0.04	-0.38	2.00	0.34	-0.69	0.47
0.38	0.17	4.86	-0.96	1.41	0.68	-0.58
1.06	-0.36	0.04	-1.06	0.18	0.20	0.97
-0.16	-0.38	-0.03	-1.08	1.09	-0.42	0.55
-0.02	1.53	-0.18	1.76	-0.48	-0.04	-1.82
-0.80	-1.10	-0.40	2.96	0.43	-0.48	-0.91
-0.51	-0.71	-0.40	1.52	-0.21	-0.64	-0.54
-0.90	-0.94	-0.37	0.51	1.71	-0.81	-1.00
-0.74	-1.07	0.14	-0.69	-0.52	-0.37	0.74
-0.23	-0.09	0.41	-0.60	-0.69	-0.22	1.18
-0.26	-0.03	0.32	-0.34	-0.98	0.18	0.10
-0.83	-0.96	-0.40	-0.40	0.73	-0.56	-0.87
1.81	3.38	-0.36	-0.14	-1.18	3.58	0.81
-0.77	-0.73	-0.29	-0.45	1.84	-0.77	-0.96
0.29	0.21	-0.13	-0.39	-0.43	0.08	1.51
0.61	0.40	-0.19	0.00	-0.92	1.25	0.48
-0.49	0.11	-0.27	0.45	-0.33	-0.80	-0.06
-0.06	0.28	-0.37	-0.47	0.27	0.00	2.70
-0.43	0.76	0.07	-0.61	-1.30	-0.10	0.35
2.88	2.03	-0.29	-0.72	-0.85	1.50	0.67

-0.40	-0.57	-0.19	-0.43	0.68	-0.23	-0.08
-0.67	-0.22	-0.36	0.11	0.36	-0.79	-0.21
-0.40	-0.34	-0.37	0.04	0.67	-0.63	-0.38
-0.62	-0.44	-0.39	-0.19	1.76	-0.83	-0.75
-0.47	-0.65	-0.33	0.40	-0.82	-0.13	-1.54
-0.20	0.39	-0.30	-0.16	-1.30	-0.66	-1.00
2.60	-0.69	0.20	-1.04	-1.47	1.70	0.18
5.46	-0.10	-0.05	-1.28	-1.05	7.47	0.35
-0.23	-0.30	-0.13	-0.73	-0.93	1.54	0.59
-0.61	-0.30	1.30	-0.94	0.04	0.41	1.88
-0.48	-1.09	2.34	-0.76	2.34	-0.51	-0.44
1.46	0.01	1.93	-0.98	-0.57	3.02	0.10
1.67	1.63	-0.33	0.55	-0.02	0.99	-1.48
-0.58	0.33	-0.35	1.63	0.02	-0.78	-0.17
-0.53	3.37	-0.20	0.28	0.12	-0.70	-0.19
-0.71	-0.04	-0.39	0.33	-0.35	-0.73	0.09
-1.00	-1.41	-0.40	-0.01	4.26	-0.86	-1.29
-0.30	0.73	-0.39	0.55	-0.77	0.44	0.90
1.38	2.86	-0.26	-0.02	-1.19	2.47	2.80
-0.67	0.14	-0.36	-0.33	-1.38	-0.56	-0.77
-0.49	4.04	-0.34	1.22	1.65	-0.68	-0.44
-0.65	9.21	-0.40	0.55	1.80	-0.12	0.00
-0.54	0.42	-0.32	0.23	-0.84	-0.62	-0.96
0.34	2.26	-0.21	-0.07	-1.21	0.54	1.33
-0.85	-0.96	-0.35	0.24	-0.34	-0.55	-0.82
-0.71	0.38	-0.37	1.15	0.83	-0.88	-1.26
-0.14	2.15	-0.20	-0.23	-1.36	-0.12	-0.37
-0.67	0.20	-0.37	0.47	-0.63	-0.55	-0.53
-0.10	0.64	-0.21	0.02	0.04	-0.48	0.00
-0.49	0.68	-0.28	-0.31	-0.51	-0.77	-0.01
0.16	0.94	-0.28	-0.21	-0.61	-0.31	0.13
-0.63	0.19	-0.25	0.31	-0.57	-0.75	-0.21
-0.36	0.13	-0.14	0.12	0.43	-0.63	-0.09
-0.02	2.62	-0.33	0.24	-0.60	-0.26	0.03
-0.77	-0.87	-0.19	0.32	1.69	-0.59	-0.38
-0.66	-0.15	-0.31	-0.19	0.44	-0.80	-0.19
-0.43	-0.60	-0.10	-0.38	-0.02	-0.41	0.17
7.69	0.66	-0.27	-1.23	-1.58	4.07	0.44
-0.52	-0.36	-0.37	-0.33	-0.58	-0.61	-0.66
0.44	0.29	-0.35	0.48	-1.25	0.31	-0.97
0.99	1.73	-0.34	0.37	-1.28	0.12	-1.25
5.62	-0.75	-0.21	-1.31	-1.61	3.39	0.92
-0.51	-0.96	-0.19	-0.53	-0.92	-0.77	-0.16
-0.33	-0.93	-0.25	-0.25	-1.00	-0.34	-0.31
0.64	-0.34	-0.24	-1.02	-1.29	0.17	0.62
0.84	-0.29	-0.32	-1.12	0.75	0.72	1.70
0.43	0.11	0.08	-0.55	-0.74	0.99	1.27
-0.31	-0.34	1.32	0.29	-0.93	0.88	-0.54
1.20	0.53	-0.22	0.05	5.61	0.19	-0.18
1.19	2.33	-0.16	-0.18	1.25	0.57	-0.29
-0.23	-0.55	-0.31	-0.70	0.90	-0.42	0.23
-0.22	-0.27	-0.40	0.94	-0.92	-0.40	1.44
-0.51	-0.29	-0.12	-0.75	-0.13	-0.05	0.50
0.78	4.27	-0.29	-0.44	-0.72	0.64	0.11
-1.01	0.26	-0.38	3.01	1.98	-0.87	-0.97
0.53	-0.40	-0.37	-0.39	0.02	0.71	0.17
-0.07	0.44	0.25	-0.71	-1.15	0.03	-0.19
-0.78	-1.22	0.09	0.03	-0.59	0.81	-1.09
2.54	2.70	0.01	-0.95	-0.68	1.50	1.84

-0.08	1.54	0.00	-0.64	-1.16	0.42	0.00
-0.54	-0.27	0.25	-0.36	-0.24	-0.72	-0.93
-0.33	0.54	-0.19	-0.72	-0.97	0.35	-0.38
-0.64	-0.31	-0.40	2.09	0.20	-0.72	-1.02
2.21	2.41	-0.31	-0.63	-1.08	2.76	0.71
6.90	0.95	-0.28	-1.22	-1.23	3.98	0.35
-0.52	0.53	-0.38	0.02	-0.42	-0.68	-0.44
-0.29	1.32	-0.29	-0.36	-1.05	-0.47	-0.24
-0.51	0.13	-0.03	-0.56	-0.10	-0.63	-0.52
0.37	1.14	-0.13	-0.64	-0.49	0.66	2.45
-0.38	1.19	-0.20	-0.36	-0.10	-0.62	0.50
2.20	0.11	-0.26	-1.24	-0.41	3.61	1.02
-0.71	-0.67	-0.25	-0.50	-0.22	-0.62	0.20
-0.50	0.23	-0.37	0.04	0.44	-0.72	-0.39
0.19	-0.64	0.23	-0.91	-1.53	1.54	-0.23
-0.39	-0.57	0.14	0.11	-1.33	0.16	0.38
0	0	-1	-1	1	0	0
0.00	0.00	0.00	0.00	0.00	0.00	0.00
1.00	1.00	1.00	1.00	1.00	1.00	1.00
0.83	0.67	0.94	0.32	0.75	0.61	0.27

68	69	70	71	72	73	74
TAAAGTGCTTATA	TGGCTCAGTTCAG	TATGGCACTGGTA	CTATACGACCTGC	CGGGGCAGCTCAG	TGAGGTAGTAAG	CATGCCTTGAGTG
miR-20a-5p	miR-24-3p	miR-183-5p	let-7d-3p	miR-486-3p	miR-98-5p	miR-532-5p
79678	78156	75744	71988	70256	69114	68018
-0.66	0.04	-0.38	2.00	0.34	-0.69	0.47
0.38	0.17	4.86	-0.96	1.41	0.68	-0.58
1.06	-0.36	0.04	-1.06	0.18	0.20	0.97
-0.16	-0.38	-0.03	-1.08	1.09	-0.42	0.55
-0.02	1.53	-0.18	1.76	-0.48	-0.04	-1.82
-0.80	-1.10	-0.40	2.96	0.43	-0.48	-0.91
-0.51	-0.71	-0.40	1.52	-0.21	-0.64	-0.54
-0.90	-0.94	-0.37	0.51	1.71	-0.81	-1.00
-0.74	-1.07	0.14	-0.69	-0.52	-0.37	0.74
-0.23	-0.09	0.41	-0.60	-0.69	-0.22	1.18
-0.26	-0.03	0.32	-0.34	-0.98	0.18	0.10
-0.83	-0.96	-0.40	-0.40	0.73	-0.56	-0.87
1.81	3.38	-0.36	-0.14	-1.18	3.58	0.81
-0.77	-0.73	-0.29	-0.45	1.84	-0.77	-0.96
0.29	0.21	-0.13	-0.39	-0.43	0.08	1.51
0.61	0.40	-0.19	0.00	-0.92	1.25	0.48
-0.49	0.11	-0.27	0.45	-0.33	-0.80	-0.06
-0.06	0.28	-0.37	-0.47	0.27	0.00	2.70
-0.43	0.76	0.07	-0.61	-1.30	-0.10	0.35
2.88	2.03	-0.29	-0.72	-0.85	1.50	0.67
-0.40	-0.57	-0.19	-0.43	0.68	-0.23	-0.08
-0.67	-0.22	-0.36	0.11	0.36	-0.79	-0.21
-0.40	-0.34	-0.37	0.04	0.67	-0.63	-0.38
-0.62	-0.44	-0.39	-0.19	1.76	-0.83	-0.75
-0.47	-0.65	-0.33	0.40	-0.82	-0.13	-1.54
-0.20	0.39	-0.30	-0.16	-1.30	-0.66	-1.00
2.60	-0.69	0.20	-1.04	-1.47	1.70	0.18
5.46	-0.10	-0.05	-1.28	-1.05	7.47	0.35

-0.23	-0.30	-0.13	-0.73	-0.93	1.54	0.59
-0.61	-0.30	1.30	-0.94	0.04	0.41	1.88
-0.48	-1.09	2.34	-0.76	2.34	-0.51	-0.44
1.46	0.01	1.93	-0.98	-0.57	3.02	0.10
1.67	1.63	-0.33	0.55	-0.02	0.99	-1.48
-0.58	0.33	-0.35	1.63	0.02	-0.78	-0.17
-0.53	3.37	-0.20	0.28	0.12	-0.70	-0.19
-0.71	-0.04	-0.39	0.33	-0.35	-0.73	0.09
-1.00	-1.41	-0.40	-0.01	4.26	-0.86	-1.29
-0.30	0.73	-0.39	0.55	-0.77	0.44	0.90
1.38	2.86	-0.26	-0.02	-1.19	2.47	2.80
-0.67	0.14	-0.36	-0.33	-1.38	-0.56	-0.77
-0.49	4.04	-0.34	1.22	1.65	-0.68	-0.44
-0.65	9.21	-0.40	0.55	1.80	-0.12	0.00
-0.54	0.42	-0.32	0.23	-0.84	-0.62	-0.96
0.34	2.26	-0.21	-0.07	-1.21	0.54	1.33
-0.85	-0.96	-0.35	0.24	-0.34	-0.55	-0.82
-0.71	0.38	-0.37	1.15	0.83	-0.88	-1.26
-0.14	2.15	-0.20	-0.23	-1.36	-0.12	-0.37
-0.67	0.20	-0.37	0.47	-0.63	-0.55	-0.53
-0.10	0.64	-0.21	0.02	0.04	-0.48	0.00
-0.49	0.68	-0.28	-0.31	-0.51	-0.77	-0.01
0.16	0.94	-0.28	-0.21	-0.61	-0.31	0.13
-0.63	0.19	-0.25	0.31	-0.57	-0.75	-0.21
-0.36	0.13	-0.14	0.12	0.43	-0.63	-0.09
-0.02	2.62	-0.33	0.24	-0.60	-0.26	0.03
-0.77	-0.87	-0.19	0.32	1.69	-0.59	-0.38
-0.66	-0.15	-0.31	-0.19	0.44	-0.80	-0.19
-0.43	-0.60	-0.10	-0.38	-0.02	-0.41	0.17
7.69	0.66	-0.27	-1.23	-1.58	4.07	0.44
-0.52	-0.36	-0.37	-0.33	-0.58	-0.61	-0.66
0.44	0.29	-0.35	0.48	-1.25	0.31	-0.97
0.99	1.73	-0.34	0.37	-1.28	0.12	-1.25
5.62	-0.75	-0.21	-1.31	-1.61	3.39	0.92
-0.51	-0.96	-0.19	-0.53	-0.92	-0.77	-0.16
-0.33	-0.93	-0.25	-0.25	-1.00	-0.34	-0.31
0.64	-0.34	-0.24	-1.02	-1.29	0.17	0.62
0.84	-0.29	-0.32	-1.12	0.75	0.72	1.70
0.43	0.11	0.08	-0.55	-0.74	0.99	1.27
-0.31	-0.34	1.32	0.29	-0.93	0.88	-0.54
1.20	0.53	-0.22	0.05	5.61	0.19	-0.18
1.19	2.33	-0.16	-0.18	1.25	0.57	-0.29
-0.23	-0.55	-0.31	-0.70	0.90	-0.42	0.23
-0.22	-0.27	-0.40	0.94	-0.92	-0.40	1.44
-0.51	-0.29	-0.12	-0.75	-0.13	-0.05	0.50
0.78	4.27	-0.29	-0.44	-0.72	0.64	0.11
-1.01	0.26	-0.38	3.01	1.98	-0.87	-0.97
0.53	-0.40	-0.37	-0.39	0.02	0.71	0.17
-0.07	0.44	0.25	-0.71	-1.15	0.03	-0.19
-0.78	-1.22	0.09	0.03	-0.59	0.81	-1.09
2.54	2.70	0.01	-0.95	-0.68	1.50	1.84
-0.08	1.54	0.00	-0.64	-1.16	0.42	0.00
-0.54	-0.27	0.25	-0.36	-0.24	-0.72	-0.93
-0.33	0.54	-0.19	-0.72	-0.97	0.35	-0.38
-0.64	-0.31	-0.40	2.09	0.20	-0.72	-1.02
2.21	2.41	-0.31	-0.63	-1.08	2.76	0.71
6.90	0.95	-0.28	-1.22	-1.23	3.98	0.35
-0.52	0.53	-0.38	0.02	-0.42	-0.68	-0.44
-0.29	1.32	-0.29	-0.36	-1.05	-0.47	-0.24

-0.51	0.13	-0.03	-0.56	-0.10	-0.63	-0.52
0.37	1.14	-0.13	-0.64	-0.49	0.66	2.45
-0.38	1.19	-0.20	-0.36	-0.10	-0.62	0.50
2.20	0.11	-0.26	-1.24	-0.41	3.61	1.02
-0.71	-0.67	-0.25	-0.50	-0.22	-0.62	0.20
-0.50	0.23	-0.37	0.04	0.44	-0.72	-0.39
0.19	-0.64	0.23	-0.91	-1.53	1.54	-0.23
0	-1	0	0	-1	0	0

68	69	70	71	72	73	74
4	3	7	6	1	76	61
TGAGGTAGTAGGT	TCTCCCAACCCTT	TTCAAGTAATCCA	TAGCAGCACGTAA	TATTGCACTTGTC	GAATGTTGCTCG	AGCTACATTGTCT
let-7a-5p	miR-150-5p	miR-26a-5p	miR-16-5p	miR-92a-3p	miR-409-3p	miR-221-3p
8839402	8994745	5953261	7939871	46389442	67182	106297
-0.72	-0.63	-1.13	-0.85	1.23	1.96	1.65
-0.61	-0.49	-0.25	0.11	-1.76	-1.03	0.31
0.67	-1.27	0.00	0.75	0.00	-0.56	-0.84
-0.13	-0.99	-0.93	-0.57	1.30	-0.95	-0.94
2.76	2.40	2.65	0.55	-2.19	-0.18	0.52
0.91	-0.83	0.47	-0.54	0.72	0.24	-0.54
0.38	0.28	-0.62	-0.92	0.63	2.38	1.79
-0.68	0.32	-0.99	-0.89	1.28	0.06	-0.56
-0.12	-1.21	-0.79	-0.17	1.11	-0.21	-1.23
-0.53	-0.67	-0.58	-0.12	0.95	-0.70	-0.55
-0.86	-0.36	1.13	0.38	-0.09	-0.74	-0.30
-0.32	3.01	-0.99	-0.95	0.50	-0.74	-0.94
-0.44	-0.41	1.56	1.63	-1.08	-1.19	3.07
-0.21	0.47	-1.11	-0.87	1.05	0.34	-0.68
-0.39	-0.71	-0.63	0.62	0.13	-0.46	-0.54
-0.71	0.03	0.67	1.34	-0.52	0.03	0.93
0.37	0.22	0.13	-0.72	-0.04	-0.29	-0.19
-1.16	0.35	-0.49	1.14	-0.01	1.15	0.02
-1.28	-0.09	0.28	0.06	-0.50	-0.76	-0.90
-1.12	-0.49	1.35	2.80	-0.90	-1.14	-0.57
-0.29	-0.62	-0.72	-0.30	0.82	-0.75	-0.30
0.24	1.20	-0.32	-0.93	0.08	2.50	0.08
0.49	0.60	-0.47	-0.82	0.16	0.18	0.06
0.22	0.35	-1.09	-0.86	0.71	-0.12	-0.45
2.92	-0.41	0.34	-0.78	-1.88	0.76	0.26
0.43	0.78	1.32	-0.64	-1.11	0.06	1.36
0.19	-0.82	1.21	1.56	-0.60	0.13	-0.50
-0.39	-1.39	0.90	4.09	-1.04	-0.73	-0.64
-1.01	-0.78	0.95	-0.21	0.35	0.01	-0.67
-1.33	-1.14	-0.88	-0.58	0.96	-0.81	-1.18
-0.29	-1.30	-1.42	-0.90	0.86	0.08	-1.04
-0.71	-1.18	-0.20	-0.12	-1.14	0.39	-0.42
2.11	-0.42	3.89	3.49	-2.35	-0.66	1.37
-0.67	-0.91	-0.71	-0.74	1.20	-0.02	0.55
-0.96	-0.62	-1.02	-0.43	0.88	-0.34	0.88
-0.39	2.79	-0.19	-0.85	0.16	-1.09	0.82
-1.16	-1.13	-1.64	-0.96	2.69	-0.78	-1.51
-1.01	0.68	0.45	-0.44	0.15	0.85	1.56
-1.38	-0.62	0.57	0.39	-1.17	-0.17	4.18

-0.65	2.94	0.75	-0.69	-0.53	-0.62	-0.23
-1.87	0.64	-0.21	-0.96	0.64	0.48	2.33
-2.05	1.75	0.12	-1.02	-1.46	2.56	13.01
-0.61	0.42	-0.25	-0.63	0.61	0.10	1.12
-1.31	-0.64	0.63	0.37	0.02	-0.81	0.00
-1.60	1.22	-0.83	-0.64	0.61	-0.15	-0.76
-2.14	-0.31	-0.51	-0.92	1.17	-0.40	-0.23
-0.89	0.20	3.42	1.12	-1.08	-0.81	-0.19
-0.25	0.48	0.48	-0.62	-0.05	0.72	0.68
-1.33	0.19	0.12	0.55	0.21	-1.14	-0.15
-1.09	2.45	0.64	-0.29	-0.15	-0.19	-0.54
-1.29	1.54	0.60	0.81	-0.52	0.64	0.79
-0.01	0.46	-0.04	-0.82	0.27	3.15	1.99
0.61	-1.06	-0.56	-0.62	0.95	2.14	0.82
0.11	0.27	1.23	-0.18	-0.39	-0.22	1.13
0.00	-0.33	-0.46	-0.64	0.87	-0.61	-0.32
-0.02	-0.23	-0.32	-0.54	0.59	1.11	-0.34
-0.27	-0.15	-0.11	0.09	0.52	4.04	-0.15
-0.71	-0.81	1.57	3.42	-1.52	-1.23	-0.98
0.23	1.47	0.56	-0.75	-0.77	1.27	0.08
1.34	0.36	2.77	0.22	-1.80	-0.71	2.72
1.35	1.88	3.06	0.54	-2.04	2.18	2.01
-0.76	-1.08	1.56	4.07	-1.24	-0.59	-1.28
0.26	-0.23	-0.32	-0.74	0.73	-0.98	-0.74
0.75	-0.06	1.20	-0.45	0.30	-0.20	-0.42
0.97	-0.96	1.23	0.97	-0.59	-1.20	-1.11
0.04	-1.09	-1.35	-0.75	0.21	-0.30	-0.76
-1.79	-1.33	-0.74	2.41	0.01	-0.76	0.10
-0.94	-0.90	-0.60	-0.58	-0.84	-0.92	-0.41
4.47	-0.48	-0.40	-0.30	-1.38	1.12	3.52
0.92	-0.25	1.01	0.13	-1.06	1.53	3.03
-0.46	-0.74	-0.65	-0.73	1.33	0.04	0.21
-1.79	5.34	0.38	4.40	-1.59	-0.47	-1.21
-0.10	-0.74	-0.20	0.63	0.61	-0.81	-0.72
-0.47	1.06	0.25	0.70	-0.35	0.31	0.42
-1.94	2.20	-1.54	-1.07	0.15	1.19	3.88
-0.35	0.99	-0.20	0.52	-0.16	1.10	-0.54
-1.02	0.65	0.27	0.00	-0.60	-0.78	-0.99
-0.40	-0.39	-0.27	0.10	-0.32	1.67	-1.31
0.12	-0.71	0.87	1.38	-0.87	-0.74	0.51
-0.62	-0.81	2.86	2.46	-1.20	-1.04	-0.38
-0.23	-0.78	0.71	-0.25	0.04	-1.27	-0.86
0.00	0.59	1.99	0.36	-0.75	-0.40	-0.25
-1.32	3.40	-0.11	-0.78	0.11	-0.37	-0.95
-1.33	-0.28	1.73	2.10	-1.40	-0.45	-0.03
-0.83	-1.11	0.68	4.85	-1.59	-0.90	-1.29
-1.58	1.55	1.24	-0.11	0.10	-1.01	0.03
-0.69	1.55	2.17	0.04	-0.64	-0.59	-0.08
-0.53	1.16	1.26	-0.08	0.04	-1.22	-0.44
-0.86	-0.60	0.38	1.10	-0.33	-0.94	-0.52
-0.65	1.32	0.78	-0.14	-0.22	-0.96	-0.15
1.62	-1.27	0.17	0.45	-1.06	0.35	-1.14
-0.66	-0.25	-0.80	-0.81	1.05	-0.31	-0.89
0.91	0.12	-0.43	-0.78	0.04	1.29	-0.26
0.57	-1.24	1.11	-0.12	-0.64	-0.97	-0.49
-0.84	-0.70	1.04	0.08	-0.22	-0.17	0.47
0.29	0.29	0.31	0.31	0.32	0.32	0.33
0.18	0.19	0.18	0.27	0.15	0.88	0.23

409595	1862148	4820581	198165	2718285	905697	128375

78	79	80	81	82	83	84
9	10	11	12	13	14	15
B_31	C_4	C_45	D_11	D_35	D_5	D_6
CHR_P	CHR_P	CHR_P	CHR_P	CHR_P	CHR_P	CHR_P
1100319	5438511	11385676	1530796	5410445	2500630	376992
0.83	0.34	0.56	0.53	0.43	0.71	0.12
B_31	C_4	C_45	D_11	D_35	D_5	D_6
690724	3576363	6565095	1332631	2692160	1594933	248617
57543	839593	1485363	66104	922039	250879	40263
782	62135	149104	2808	110593	187485	28011
116387	66190	519025	27131	288601	83289	6316
8068	137605	290995	2601	177243	39675	8077
12377	74324	311607	21738	157984	41080	5640
76530	110612	290912	76	148973	33455	5133
17767	60894	195271	1079	87662	37482	3941
3301	13437	142980	751	19838	9389	239
1786	45753	137089	680	116931	10484	1718
9961	80032	105854	790	50422	31476	4480
14513	52008	71950	4647	67416	9519	1574
4756	23945	89230	1976	38096	8959	1123
10040	31706	64809	394	62420	7016	608
1245	29523	91349	10147	28410	5598	1187
16498	20456	76934	42	49422	5698	1306
391	12155	36167	4096	34739	976	241
2884	9723	47570	42	23519	5442	359
1475	9271	47481	1230	22958	8395	799
2600	11194	44142	118	18192	5563	624
4618	6833	23589	858	12258	13575	2164
1685	9605	29496	2819	13803	6319	502
834	9464	27925	164	12294	3499	282
3021	4524	7791	39	10145	5295	728
673	2563	23854	103	5539	5107	230
2687	4476	44370	52	6827	8514	1096
1409	6167	16802	977	8451	3533	240
197	5688	14709	9351	15189	1765	246
4048	8002	20128	78	5939	5604	908
284	5632	14766	278	8474	2631	288
948	4409	6685	418	8789	2520	375
2002	5124	25164	59	9530	3332	292
433	7383	20268	385	8809	1435	387
1549	3256	13895	86	6943	1007	90
1700	1982	10549	18	6747	794	107
239	5156	13744	257	7733	2413	267
458	6282	13928	136	4661	2640	158
2186	3772	15343	153	5402	6796	1211
1609	4793	19198	132	3571	4428	721
167	2424	7797	1014	2545	4355	280
825	2996	7643	4999	4253	645	994
78	1428	11190	4456	2903	1173	106
471	2099	11262	934	7323	1439	395
602	2529	6823	1404	3054	868	82
1521	2832	9459	19	3067	1973	303
595	1373	9487	1047	3258	1950	124

424	1740	6714	471	3847	975	127
186	1506	3436	169	3733	841	134
549	2982	6373	1287	4733	865	155
212	959	1740	4	1212	28	1
2941	539	9094	31	2585	920	105
14	728	3394	682	4094	865	286
172	1546	8012	189	2108	1343	79
138	1472	4898	2738	2224	1314	65
1599	1514	4602	26	2533	2209	392
88	651	1278	93	1729	394	28
382	207	1338	10	837	81	11
428	1397	4655	125	1954	667	46
157	936	5182	10	2506	630	61
202	1298	5766	66	3213	1208	137
123	765	3285	174	2007	507	89
120	918	4397	392	1512	344	33
175	579	4681	269	2088	1090	82
106	433	1690	9	767	178	46
79	66	195	6	144	36	5
438	1622	4366	139	2114	216	100
236	979	3131	2	1689	458	19
461	698	4507	12	1244	331	16
103	311	2696	1	1333	271	10
131	598	7405	81	781	465	8
29	1410	1546	20	395	1464	158
442	640	2261	346	1351	322	87
140	1117	1937	218	1738	245	57
73	558	2504	4	1489	291	74
314	1035	2168	35	1279	349	22
175	376	1387	27	1345	357	19
146	433	3090	170	2575	217	148
38	665	1039	206	1059	340	39
9	366	703	87	482	428	58
56	559	2219	331	1650	185	33
30	634	2223	119	849	231	27
28	1130	1364	27	1025	146	10
20	383	1929	409	1023	133	8
36	695	1086	22	756	296	91
69	163	712	1	1101	87	4
50	251	656	18	464	305	20
2	260	1491	2711	571	138	6
160	450	947	1111	995	260	55
149	486	2576	7	980	121	22
319	474	1826	2	1036	142	18
36	356	1112	38	1129	190	70
217	370	1293	74	581	157	54
7	61	126	11	84	3	1
26	361	1762	96	710	97	5
58	236	982	7	959	86	11
245	249	1371	1	647	45	4
262	322	2157	1	996	165	15
25	197	628	139	612	227	74
160	312	944	212	510	189	49
81	185	818	165	368	332	39
66	176	1483	3	651	104	7
6	125	584	526	170	224	1
131	317	427	3	391	114	29
34	288	921	275	827	125	40
54	247	971	0	285	129	25

244	362	2134	0	396	165	33
17	173	973	3	430	109	8
48	135	921	694	331	117	13
61	165	676	9	235	206	17
68	187	919	1	501	39	2
26	179	1203	14	513	61	6
108	156	1025	6	450	256	21
24	80	588	111	440	123	14
32	85	978	413	192	183	4
24	139	459	0	286	133	3
20	137	753	20	598	144	2
9	170	525	35	436	66	25
38	83	424	4	508	102	4
105	142	472	3	425	48	13
105	142	472	3	425	48	13
14	184	1078	7	177	136	7
30	205	911	345	247	28	0
43	248	1072	4	336	87	8
22	63	492	0	491	66	41
59	87	391	8	241	219	17
5	19	131	4	94	8	2
23	173	453	28	275	32	7
13	70	132	663	112	64	6
36	121	518	30	157	53	16
2	59	244	294	79	96	3
3	63	307	45	220	69	8
2	98	106	63	36	25	5
115	106	657	27	328	79	21
5	72	222	13	160	64	3
16	81	230	128	129	25	4
11	56	185	126	198	16	1
2	89	335	30	271	96	8
4	209	327	10	250	29	3
6	83	331	45	355	35	2
19	86	246	0	174	26	7
14	93	759	107	120	36	2
5	72	219	20	75	30	2
19	128	359	19	156	30	3
11	108	661	83	257	22	8
22	41	198	1	127	37	3
11	33	200	4	62	2	0
40	193	453	51	145	22	7
7	45	250	0	169	32	2
37	51	286	0	130	44	6
66	215	314	3	195	25	8
2	50	168	109	87	35	1
25	70	227	2	159	65	10
18	81	269	3	152	29	0
6	32	427	0	519	31	13
1	39	226	13	71	19	0
14	17	782	2	215	6	0
21	99	180	74	81	19	3
1	33	86	9	69	44	0
7	33	226	50	84	19	1
23	54	297	90	84	31	2
3	89	85	103	182	16	4
3	8	50	1	34	11	0
8	33	241	95	74	42	1
10	71	269	83	94	23	0

16	32	421	1	134	39	0
4	27	182	0	121	40	2
17	97	210	65	195	12	2
3	45	176	2	51	35	1
5	33	280	265	27	33	13
37	5	101	0	56	8	3
16	217	254	11	176	12	2
32	34	234	12	25	28	1
3	111	153	3	160	21	1
3	62	150	2	124	71	14
7	61	271	2	134	61	6
7	25	277	1	80	26	0
5	45	76	186	138	19	1
5	31	178	12	208	16	5
38	44	219	2	116	36	8
4	55	227	47	44	22	0
19	33	236	19	181	19	6
2	97	131	16	123	28	4
8	45	186	0	100	19	5
0	15	158	27	117	9	8
12	61	225	0	98	24	3
4	25	91	21	78	27	5
5	56	172	107	91	7	4
7	41	172	0	98	17	5
22	25	228	2	204	11	5
13	16	168	23	108	3	1
4	33	169	5	55	18	2
2	20	48	179	2	1	0
17	29	359	0	90	16	0
1	21	177	6	78	47	1
12	24	100	4	113	21	11
5	62	99	14	65	31	8
4	16	29	13	44	13	2
19	10	81	0	31	8	2
11	95	171	4	80	21	1
1	20	186	90	55	9	2
14	63	119	18	53	34	1
0	16	85	3	50	20	4
10	13	301	1	113	49	6
0	46	104	55	28	14	7
5	28	49	32	61	36	1
1	34	106	0	54	8	0
2	15	123	22	144	3	1
3	61	118	30	56	17	2
29	35	142	0	62	31	3
1	18	140	4	134	1	1
3	54	204	21	35	5	1
4	16	57	52	35	22	1
8	30	138	2	74	21	5
22	18	123	0	142	21	15
1	53	124	63	73	17	3
9	35	108	5	122	11	0
28	32	84	26	47	30	13
8	30	74	5	52	12	5
4	25	138	5	73	15	1
0	2	16	0	13	0	0
0	41	68	0	51	4	0
1	2	11	0	34	0	6
5	61	116	22	58	9	0

3	12	59	7	24	12	0
3	33	125	24	42	8	1
6	20	152	0	129	8	2
0	16	77	22	58	13	1
2	21	43	32	59	10	0
2	42	41	24	83	3	0
2	22	58	13	27	9	0
2	25	77	0	22	10	0
7	23	71	0	43	15	1
4	27	34	15	44	18	3
9	8	44	0	21	2	0
2	16	65	40	39	4	0
3	22	69	0	40	2	1
5	22	49	97	29	7	1
2	4	35	102	24	11	1
1	4	54	0	12	13	1
27	9	117	1	43	14	1
4	22	89	3	24	1	1
2	21	140	1	82	8	2
4	23	36	5	31	1	0
2	20	71	0	24	8	0
4	2	45	0	22	15	1
4	2	45	0	22	15	1
0	6	74	3	69	5	2
0	18	70	26	31	8	0
2	10	38	11	20	10	0
6	14	94	2	115	4	1
5	16	98	0	25	9	0
3	11	78	3	36	6	1
6	14	36	3	23	4	1
0	75	106	1	56	0	1
0	11	32	78	12	2	0
10	11	102	0	23	11	5
0	21	13	5	10	8	0
5	5	63	2	39	0	0
0	19	56	0	17	7	0
2	4	11	2	9	0	0
2	23	113	5	15	8	1
6	22	36	0	24	12	1
1	12	32	11	36	8	1
1	35	48	18	41	3	2
5	20	34	0	23	11	1
0	14	32	4	49	8	2
0	8	31	23	41	2	0
5	16	68	1	52	4	0
1	14	77	14	32	4	0
0	0	0	0	2	1	0
1	2	25	73	23	5	1
2	24	48	16	22	5	0
0	8	27	32	29	5	0
1	11	112	1	49	0	0
2	13	24	0	15	7	6
0	1	32	0	11	0	0
1	3	40	3	11	10	0
0	14	91	0	31	5	1
1	14	46	0	0	0	0
0	19	30	12	22	1	2
1	30	83	2	36	1	1
1	6	24	20	7	7	0

0	3	55	5	27	2	1
0	22	52	4	29	5	1
8	11	46	1	63	3	2
0	13	29	20	21	2	0
0	6	31	14	24	3	0
1	19	22	4	20	8	1
0	11	81	0	28	4	1
1	10	145	1	51	0	2
2	19	76	0	2	4	0
13	4	24	0	28	2	0
2	5	10	1	20	25	0
0	4	20	17	14	1	0
2	8	33	0	20	8	1
5	1	71	3	69	0	0
3	10	33	9	41	4	5
6	12	75	0	20	3	0
2	12	39	2	12	7	3
1	9	28	10	25	2	0
3	5	31	46	6	3	0
18	5	242	0	76	2	3
0	2	41	2	49	1	3
0	6	28	1	14	5	0
0	8	22	33	22	2	0
3	13	40	18	52	3	2
6	11	48	1	19	7	3
2	12	48	5	9	2	0
4	7	39	17	24	1	0
0	5	28	33	17	5	1
0	5	35	26	9	2	0
9	15	37	0	27	7	0
0	9	27	5	13	5	2
3	1	18	0	15	2	0
1	6	13	0	14	6	0
13	18	37	1	22	5	0
6	19	31	0	15	5	2
0	10	53	17	13	4	0
2	7	25	0	9	5	0
0	5	48	15	12	7	0
14	4	21	1	19	2	0
0	8	32	6	18	1	0
1	8	16	0	12	2	0
4	12	17	2	18	5	0
0	2	21	2	23	0	0
0	6	16	3	17	3	0
0	6	37	1	19	2	0
2	7	49	13	28	0	0
0	6	15	5	9	1	0
1	9	34	0	8	3	0
0	8	42	19	5	2	2
0	2	32	1	24	9	0
1	7	26	9	5	2	0
1	0	4	1	5	2	0
5	7	9	10	14	2	0
3	2	15	0	15	2	1
1	11	17	2	15	3	0
1	10	27	0	9	1	0
3	4	20	2	13	1	1
0	5	4	0	2	1	0
0	8	17	2	19	3	0

4	13	14	0	11	1	0
4	5	31	2	14	2	0
0	15	14	3	5	3	0
1	8	33	1	17	3	1
1	7	10	6	2	5	2
1	1	15	1	11	3	0
1	4	37	7	14	2	0
2	4	10	0	24	3	0
0	12	23	0	8	2	0
2	6	20	1	15	4	1
2	7	28	14	5	4	0
0	10	38	0	5	0	1
1	7	23	0	11	3	0
1	0	7	0	6	1	0
2	5	20	2	9	0	0
0	4	36	3	10	2	0
0	3	40	0	19	2	0
0	0	12	5	10	0	0
1	0	19	0	4	1	0
0	5	12	4	5	2	0
0	2	12	0	10	9	0
0	2	17	3	4	8	0
1	3	8	1	10	1	0
2	6	21	1	13	0	1
0	5	9	11	8	7	1
1	6	12	4	9	2	0
3	4	58	0	9	0	0
0	3	17	0	5	1	0
0	2	15	5	10	4	0
0	5	9	0	6	4	1
1	6	16	1	2	1	0
1	2	21	0	4	5	0
0	1	12	1	6	0	0
1	4	10	2	21	1	0
5	1	14	0	6	2	0
1	2	15	0	7	0	0
0	1	14	1	8	9	0
0	2	5	10	10	0	0
0	2	12	4	9	0	0
1	2	6	0	8	2	1
1	1	8	2	4	2	3
0	0	11	2	23	3	0
1	3	11	0	14	2	0
0	8	9	1	12	0	0
1	2	10	7	2	3	0
0	3	28	1	3	6	0
0	4	13	3	8	0	0
1	1	16	7	8	0	0
1	5	31	0	5	1	0
0	0	1	0	2	0	0
1	2	11	8	8	0	0
0	1	23	1	17	0	0
5	0	10	0	6	1	0
0	0	8	15	1	3	0
2	4	13	0	9	2	0
2	4	7	0	3	2	0
0	3	7	3	7	4	0
0	4	10	2	6	3	0
0	0	8	4	5	1	0

1	12	17	0	12	1	0
0	5	10	1	8	0	0
2	3	3	0	8	4	0
3	2	11	0	5	0	0
1	3	3	5	6	1	1
0	0	5	5	6	2	0
0	3	16	3	9	1	1
1	0	11	0	8	0	0
1	2	4	1	6	0	0
1	4	8	0	4	1	0
5	1	4	0	11	1	0
0	0	3	0	1	0	0
0	0	0	0	0	0	0
1	1	12	0	5	1	0
0	4	9	23	3	2	0
0	3	8	5	2	0	0
2	5	7	7	3	1	0
0	2	4	0	7	0	0
1	0	2	0	0	0	0
0	2	26	0	8	2	0
0	3	11	1	11	1	0
0	1	13	0	1	1	0
0	1	5	1	9	1	0
0	3	11	2	7	0	0
0	4	7	4	8	1	1
2	0	6	0	2	0	0
0	1	8	0	4	0	0
2	1	20	0	6	0	0
0	3	8	1	5	1	0
0	0	11	0	3	2	0
2	0	4	0	2	0	0
1	0	8	0	5	2	0
0	4	4	1	5	3	0
0	1	6	2	9	0	0
1	5	8	0	3	1	0
0	1	5	1	3	1	0
0	2	6	5	3	0	0
0	6	6	2	9	0	0
0	0	12	0	2	2	0
1	0	19	0	19	2	1
0	0	3	0	7	0	0
2	2	7	0	6	1	0
0	2	4	0	7	3	0
0	3	4	0	7	1	0
0	2	9	1	5	0	0
0	2	10	0	2	0	0
0	7	2	0	1	2	0
0	1	7	5	2	3	0
0	2	3	2	0	0	0
0	2	10	2	5	0	0
0	1	8	0	4	3	0
0	8	6	0	6	0	0
0	0	18	5	2	0	2
0	3	9	0	3	1	1
0	0	5	0	3	2	0
0	2	6	0	0	0	0
0	2	6	2	4	2	0
0	1	4	3	7	1	0
0	0	4	2	1	0	0

1	0	9	0	1	0	0
0	2	8	0	2	0	0
0	1	2	0	4	1	1
0	2	8	2	1	0	0
0	0	3	0	2	0	0
0	3	10	0	6	0	0
1	1	4	3	3	0	0
0	0	3	0	0	0	0
0	1	3	0	3	1	0
0	1	19	0	5	1	0
0	6	9	1	14	0	1
1	1	15	1	1	1	0
1	1	5	0	6	0	0
0	2	0	8	3	0	0
0	2	7	0	7	0	0
0	4	5	0	2	3	1
0	0	2	0	1	0	0
0	0	2	0	2	0	0
0	3	1	0	2	0	0
0	4	7	1	0	2	0
0	0	10	0	2	0	0
1	1	3	0	2	1	0
3	2	4	0	2	1	0
0	1	7	3	4	1	1
1	1	2	2	3	1	0
0	1	12	0	6	1	0
2	0	2	3	3	1	1
0	1	2	3	3	1	0
0	0	1	0	0	0	0
0	3	2	0	0	1	0
0	1	15	0	7	2	0
0	1	4	1	0	0	0
0	4	8	0	3	0	0
0	0	1	2	2	0	0
0	1	10	1	2	1	0
0	1	7	1	0	0	0
0	2	3	6	2	0	0
1	0	7	0	3	1	0
0	3	4	1	0	1	0
0	0	4	6	1	0	0
3	2	7	0	4	1	0
0	0	6	3	0	0	1
0	1	2	0	3	0	0
0	1	1	0	2	0	0
0	1	2	0	3	1	0
0	4	5	0	2	0	0
1	0	1	0	1	0	0
0	4	6	0	3	2	0
0	0	5	1	5	1	0
0	0	6	0	3	1	0
0	1	3	1	3	0	0
0	0	15	0	2	1	0
0	0	12	0	1	0	0
1	0	0	0	1	0	0
3	3	7	1	3	0	0
3	3	7	0	5	2	0
0	0	0	0	2	0	0
0	1	1	1	1	0	0
0	2	2	0	1	0	0

1	1	5	0	1	0	0
1	0	4	2	2	0	0
0	0	1	0	2	0	2
0	0	1	1	5	1	0
0	0	0	0	3	0	0
0	0	6	0	3	0	0
1	0	2	4	0	1	0
0	1	1	8	2	0	0
0	0	1	0	2	0	0
0	0	0	0	9	0	0
0	1	2	0	1	0	0
0	5	2	1	3	0	0
0	0	0	6	0	0	0
B_31	C_4	C_45	D_11	D_35	D_5	D_6
CHR_P	CHR_P	CHR_P	CHR_P	CHR_P	CHR_P	CHR_P
690724	3576363	6565095	1332631	2692160	1594933	248617
20	21	19	28	16	20	21
-0.28	-0.12	-0.60	1.32	-1.13	-0.22	-0.08

0.18	0.09	0.04	0.53	0.35	0.75	0.63
21	24	28	10	15	7	9
1	1	1	1	1	1	1
78	79	80	81	82	83	84
13	14	15	16	17	18	19
D_6	E_19	E_21	E_22	E_23	E_3	E_46
CHR_P	CHR_P	CHR_P	CHR_P	CHR_P	CHR_P	CHR_P
128375	4686947	1336672	1032612	1495548	331698	1653485
D_6	E_19	E_21	E_22	E_23	E_3	E_46
40263	1091684	255189	377862	379984	124977	258293
28011	502157	213241	199130	172634	19968	62806
6316	164902	42264	34304	133004	68294	83643
8077	363634	74226	71113	112232	11484	53131
5640	168227	60585	62714	91720	29872	116972
5133	384358	159415	29437	72873	3409	169046
3941	241542	120763	51665	110884	11308	108609
239	170486	19447	3984	11486	855	206272
1718	160818	59440	15638	37107	4040	72117
4480	146719	27738	16747	34871	1335	30042
1574	83057	11529	5049	21295	2197	21733
1123	78420	23046	18190	18821	6539	29913
608	67728	14380	3469	31235	1127	21234
1187	47756	9645	16109	15141	2620	11470
1306	49746	11428	1692	13245	723	32632
241	15960	7283	7002	9764	7497	11053
359	68639	12706	1450	14569	432	21244
799	68787	9163	4133	9311	1693	17088
624	42508	12757	4006	9113	1012	15857

2164	22498	10337	5460	11379	1634	10414
502	37407	8453	3170	8471	1321	11161
282	37223	4087	3436	5650	671	5481
728	26441	6142	2757	6541	768	9153
230	43123	13258	2801	8605	361	15787
1096	41338	8570	1361	7492	350	19954
240	35378	5537	4177	5771	1090	7670
246	13570	2631	4416	4918	1441	4771
908	12615	4758	2076	2971	210	8490
288	28349	8096	4620	8607	1001	6149
375	21739	5854	4227	7817	878	2975
292	24465	8071	2430	5991	2016	9244
387	13614	3701	3365	5191	787	3153
90	22482	4396	1462	3296	542	16316
107	9431	1539	315	1303	187	21344
267	26556	7663	4366	8052	962	5712
158	17481	3592	5270	4749	375	2813
1211	20296	4581	2089	2878	410	4251
721	19821	8938	1413	7464	263	2794
280	9234	1750	4360	5035	303	2132
994	2083	1343	1343	1802	1161	1533
106	6288	2100	2021	2809	461	2590
395	8362	1914	1295	2664	966	3483
82	15094	3308	3782	3872	735	2936
303	7445	1250	307	1006	77	8899
124	12184	3381	2185	4911	922	7026
127	8964	2412	1302	3118	517	2193
134	10643	3997	1868	2942	373	2509
155	2285	887	1727	1377	1089	2514
1	3209	249	142	737	55	7014
105	3186	1092	221	537	337	9277
286	6693	940	226	3241	320	843
79	9428	2710	646	2436	221	3106
65	5262	1015	1432	1345	139	1216
392	3803	1802	434	1409	98	1507
28	10682	2089	583	406	260	2310
11	1912	105	56	159	42	8704
46	6295	2142	1567	1409	163	1180
61	7356	2196	1203	1553	392	2756
137	4687	1156	368	1965	310	1606
89	1394	1149	1634	1572	472	712
33	4365	807	446	987	131	1164
82	4609	1215	630	910	301	2118
46	2675	394	128	238	12	2919
5	1293	226	118	235	27	6738
100	832	225	565	622	220	332
19	6500	838	323	732	83	1197
16	4792	595	134	572	206	3920
10	5083	392	158	326	41	1577
8	5139	1069	333	801	103	1631
158	4768	1358	1688	855	25	450
87	1148	508	530	523	528	610
57	1909	353	577	485	231	456
74	3695	594	77	635	25	1907
22	3930	566	218	505	63	929
19	4700	1642	406	1573	107	1008
148	1158	185	57	566	131	563
39	3160	1065	650	596	139	735
58	1282	676	901	513	93	1403

33	936	263	315	385	279	374
27	3055	613	391	500	116	987
10	3718	680	546	745	76	665
8	1928	367	453	524	241	428
91	1251	391	610	539	141	204
4	1104	241	54	164	162	2150
20	6239	1194	447	866	40	965
6	3066	404	328	350	90	715
55	818	275	419	448	245	261
22	1568	529	74	245	27	1080
18	1279	438	117	537	77	618
70	608	424	321	389	75	209
54	1204	375	173	445	83	460
1	460	25	16	54	6	1799
5	1169	347	303	654	106	437
11	2372	686	176	341	68	450
4	1641	240	69	207	20	892
15	649	453	68	278	85	668
74	364	206	198	274	104	151
49	518	141	188	202	121	229
39	534	162	201	292	122	296
7	916	152	23	88	11	540
1	2445	174	178	181	7	259
29	931	611	323	496	72	318
40	394	130	87	294	127	309
25	1789	440	152	296	19	395
33	876	229	97	144	30	436
8	1251	126	35	98	9	733
13	726	176	126	218	88	274
17	1460	525	192	478	47	341
2	1264	154	58	124	34	596
6	1129	126	75	120	20	559
21	1499	285	70	249	28	595
14	601	168	131	213	59	251
4	425	102	50	110	49	203
3	1745	130	107	133	14	235
2	620	114	124	160	60	427
25	579	108	85	134	17	188
4	625	250	230	96	41	200
13	666	197	26	185	25	341
13	666	197	26	185	25	341
7	853	134	88	111	14	309
0	569	99	137	140	53	191
8	387	62	72	129	65	114
41	936	99	10	188	25	313
17	441	169	68	116	33	316
2	137	21	5	28	6	913
7	171	93	84	115	52	117
6	605	140	171	228	23	133
16	310	87	50	143	49	121
3	585	158	248	147	18	61
8	385	71	148	84	7	31
5	309	86	91	70	11	466
21	245	92	31	127	46	174
3	757	261	84	127	18	127
4	522	124	139	128	15	118
1	885	101	30	85	14	171
8	481	126	60	116	23	129
3	627	96	70	142	7	143

2	653	58	68	54	22	195
7	635	128	59	131	12	140
2	318	99	57	112	20	106
2	353	141	100	94	28	135
3	370	103	102	119	27	128
8	81	31	21	39	46	66
3	318	130	66	99	24	122
0	130	8	5	21	1	319
7	221	26	60	59	25	37
2	115	50	49	74	42	87
6	341	216	38	133	29	161
8	97	60	46	55	24	37
1	422	68	60	91	18	91
10	241	95	29	81	3	309
0	456	38	28	42	11	135
13	324	39	2	59	13	379
0	127	66	68	105	31	55
0	97	14	13	42	38	269
3	255	35	84	66	16	26
0	594	82	85	70	2	36
1	239	35	52	29	5	85
2	273	40	20	67	6	84
4	151	36	42	80	8	54
0	71	10	4	8	0	373
1	209	61	39	62	16	79
0	193	39	68	77	18	47
0	209	41	8	46	3	139
2	419	108	31	94	5	108
2	83	34	50	53	35	46
1	415	59	69	93	11	53
13	31	10	12	10	25	266
3	178	47	12	37	5	307
2	275	47	40	44	6	34
1	159	85	68	34	12	60
1	139	30	22	27	13	72
14	59	48	43	65	19	31
6	143	91	33	49	8	89
0	140	77	5	46	7	180
1	151	46	23	72	8	50
5	224	10	9	74	12	55
8	309	94	21	57	11	108
0	611	40	77	45	4	114
6	111	15	7	57	22	65
4	179	25	0	60	19	49
5	221	59	12	50	4	135
8	118	10	0	75	7	43
3	165	63	10	39	6	120
5	205	27	36	42	15	27
4	83	24	30	48	22	44
5	208	53	12	50	4	130
5	156	18	3	53	9	46
1	153	34	13	34	33	88
2	231	63	28	38	5	88
0	20	0	0	12	103	16
0	207	43	3	18	1	133
1	322	49	15	45	4	67
11	29	15	29	40	39	21
8	124	17	23	26	4	16
2	131	39	38	39	9	137

2	107	11	7	10	3	382
1	124	25	25	20	8	45
2	268	30	14	33	4	60
1	39	21	17	40	2	38
4	114	66	70	64	4	19
6	63	93	17	24	9	31
7	93	22	31	51	2	17
1	144	17	25	53	6	13
0	314	16	11	22	2	114
1	94	19	0	57	10	31
2	83	31	43	34	12	26
3	116	37	16	36	9	35
1	136	17	23	50	19	7
1	88	8	26	28	9	40
1	91	37	41	40	5	26
5	91	66	34	19	10	34
15	173	26	0	50	3	185
3	102	17	25	17	5	16
0	95	15	4	11	21	55
13	49	47	20	29	11	29
5	149	61	27	44	4	26
1	100	33	12	38	2	62
0	45	3	0	2	2	64
0	282	27	16	36	2	22
6	28	45	18	11	4	83
0	90	8	21	29	8	17
0	168	46	30	27	10	57
1	96	7	19	10	4	38
2	45	11	0	37	5	33
1	49	7	15	33	7	35
0	46	9	9	25	10	17
0	39	6	22	11	19	27
0	70	26	25	21	3	16
0	191	26	29	29	4	24
1	121	20	7	15	2	47
3	90	13	31	24	10	15
0	66	11	3	11	0	53
0	46	17	10	18	7	14
1	121	23	9	16	8	23
1	78	16	28	28	2	20
1	126	6	21	27	7	68
1	89	10	4	28	1	73
1	64	35	6	16	4	30
1	44	17	10	21	6	29
2	77	7	1	22	2	25
0	237	33	16	17	3	31
0	112	29	4	16	0	48
1	173	57	26	17	4	26
1	173	57	26	17	4	26
2	47	8	0	24	6	11
0	33	9	11	11	2	6
0	94	15	16	20	5	42
1	68	13	2	41	6	42
0	82	29	11	48	6	40
1	47	14	12	23	7	12
1	125	10	3	20	4	25
1	52	15	19	15	2	1
0	54	19	10	27	2	26
5	30	34	4	11	3	20

0	18	8	17	3	0	10
0	74	12	4	6	7	70
0	135	21	22	24	3	18
0	20	0	0	2	0	68
1	102	15	9	21	6	12
1	85	31	2	35	1	12
1	31	7	15	20	6	11
2	29	13	7	20	5	10
1	81	30	2	34	1	11
2	23	11	9	10	5	6
0	73	26	28	11	1	8
0	82	8	5	9	5	23
0	51	14	9	15	4	13
0	5	0	2	0	0	100
1	17	5	10	20	3	12
0	44	8	10	11	5	6
0	34	3	6	18	4	16
0	20	2	3	20	5	15
6	33	9	11	12	1	16
0	18	0	0	2	0	53
0	151	14	14	21	2	11
1	52	11	0	16	0	18
0	0	0	0	0	0	0
2	34	7	4	11	2	6
1	21	11	9	18	8	10
0	65	11	9	12	0	6
1	34	2	1	11	1	6
1	34	14	3	18	3	21
2	22	6	2	15	4	14
0	36	8	12	10	1	11
0	27	13	22	15	4	10
1	15	20	6	13	5	10
1	44	11	0	16	0	14
2	28	5	1	16	5	16
0	40	7	1	3	0	31
0	21	11	1	10	2	39
0	75	19	9	14	0	37
0	49	2	10	13	4	11
1	40	14	6	11	0	28
0	7	1	4	5	10	21
5	22	3	3	11	9	14
0	22	8	1	6	0	32
3	16	8	9	8	0	3
0	49	5	3	7	2	26
0	38	6	11	6	1	20
3	19	4	0	8	6	32
3	14	3	2	13	0	8
0	36	9	11	26	0	9
0	20	6	3	19	7	7
2	34	4	9	2	0	6
3	29	8	2	16	1	17
0	13	7	8	6	1	17
0	7	5	4	8	7	5
1	25	5	6	13	1	7
0	21	8	12	8	0	5
0	36	15	5	13	4	6
2	25	4	4	6	3	11
0	26	3	2	3	0	54
0	66	21	1	9	0	26

0	24	2	4	2	0	9
2	32	9	6	15	0	5
0	21	5	6	3	8	6
0	54	7	4	10	0	7
0	22	4	7	7	10	12
0	27	10	3	17	3	22
0	10	6	6	9	1	9
0	41	10	0	7	0	19
0	24	10	4	5	2	8
0	36	4	1	9	3	7
0	16	4	7	5	4	8
0	31	4	0	17	0	10
0	20	0	6	2	8	42
0	13	5	14	11	0	10
0	35	7	3	6	0	12
2	18	2	9	6	0	8
0	14	5	5	9	2	2
0	27	5	12	5	2	5
0	30	0	0	1	2	44
0	13	5	1	3	1	12
1	43	13	1	6	0	18
0	24	6	8	6	2	3
0	37	6	1	7	2	5
1	1	2	3	10	1	4
0	11	0	0	1	0	41
0	20	1	2	4	0	5
0	18	8	9	12	1	7
0	4	3	4	12	4	7
0	29	4	3	8	1	6
1	16	6	11	6	6	3
2	5	6	6	9	3	8
0	9	1	5	10	1	7
0	27	4	2	2	2	1
0	13	2	1	1	0	34
0	33	9	0	9	0	3
1	23	8	3	8	0	10
0	16	4	5	3	2	2
1	18	8	3	8	1	9
0	15	3	4	11	0	1
0	14	0	2	1	1	66
0	26	15	12	0	2	6
0	42	4	1	2	0	10
0	20	3	2	5	0	15
0	14	3	3	7	0	5
0	29	3	2	6	0	19
0	36	2	5	4	1	4
0	29	4	1	5	1	12
0	8	7	3	3	1	4
0	27	6	3	9	0	9
1	7	4	3	7	1	1
1	26	3	4	7	0	2
0	24	1	3	5	2	4
0	15	0	0	3	1	19
0	10	3	0	5	1	16
0	7	2	2	5	1	1
1	30	9	2	5	2	5
0	7	1	1	10	0	4
0	9	3	0	4	1	4
0	20	2	0	13	0	13

0	4	1	1	5	0	5
0	3	3	1	2	0	30
0	8	0	1	6	2	2
0	22	0	3	3	2	7
0	5	0	5	4	1	6
0	9	0	1	5	1	5
1	3	4	2	8	1	3
3	1	4	0	2	1	4
0	14	1	1	10	3	6
0	14	3	1	6	2	3
0	22	3	2	0	0	3
0	9	0	0	2	1	5
0	20	1	2	0	0	2
0	21	4	3	6	0	5
0	4	2	0	5	0	2
0	8	4	0	2	4	10
0	68	0	3	2	0	0
0	7	2	4	2	1	2
0	14	1	0	6	0	4
0	14	4	0	4	0	10
0	13	2	4	1	0	4
0	12	5	2	3	1	10
0	6	1	0	1	0	9
0	19	4	1	1	1	12
0	5	4	2	2	0	3
0	6	6	5	2	2	5
0	15	0	0	1	1	0
0	11	5	3	5	0	12
0	17	10	2	4	1	7
0	18	4	0	1	0	14
1	7	5	3	4	0	3
0	16	2	2	2	0	1
1	6	1	0	1	0	2
0	4	2	4	6	0	7
0	11	3	7	6	1	1
0	5	0	1	4	0	5
0	5	10	0	3	0	8
0	1	0	0	0	0	19
0	0	0	0	1	0	27
0	19	2	0	3	1	16
0	6	1	2	4	1	5
0	2	4	1	4	1	2
0	5	0	2	4	2	1
0	8	1	0	1	3	1
0	1	2	1	0	0	23
0	14	0	0	7	0	16
0	12	0	0	5	0	5
0	10	1	2	4	0	10
0	1	3	1	4	1	3
0	7	2	1	3	1	2
1	6	0	0	2	0	6
0	5	0	2	0	0	27
0	9	3	1	2	3	6
0	4	2	1	1	0	4
0	9	1	4	3	0	2
0	8	9	2	2	1	6
0	0	1	0	2	1	23
0	3	2	2	3	0	1
0	7	0	2	1	0	2

0	0	4	2	0	1	2
0	3	1	2	5	0	4
0	4	2	0	2	0	2
0	4	1	1	7	3	2
0	7	2	0	1	1	2
0	7	2	3	3	1	6
1	9	1	0	4	2	1
0	7	0	1	2	0	42
0	16	2	0	1	0	1
0	4	0	0	3	0	0
0	11	0	1	1	0	4
0	4	0	1	1	0	0
0	9	1	0	4	0	7
0	4	5	0	3	0	9
0	7	2	9	2	1	1
0	1	3	1	3	0	2
0	8	5	4	7	0	2
0	8	1	0	2	0	1
0	3	4	0	6	1	5
2	3	2	5	0	0	1
1	8	0	0	4	1	3
0	11	4	2	2	1	2
0	11	3	1	2	0	1
0	13	2	2	5	0	5
0	5	1	2	2	0	0
0	6	2	1	0	0	0
0	5	1	0	1	0	3
0	6	2	1	3	0	2
1	5	5	2	2	0	3
0	7	2	2	1	0	1
0	5	3	2	2	0	0
0	2	0	0	0	0	9
0	5	2	0	2	0	4
0	10	3	0	1	0	7
0	5	1	3	1	0	1
0	9	0	0	2	0	8
1	2	4	0	0	2	0
0	7	0	0	0	1	2
0	1	1	0	1	0	2
0	12	0	1	1	0	5
0	4	0	0	0	0	2
1	9	5	1	2	0	3
0	3	1	2	1	0	0
0	3	1	0	1	0	9
0	4	0	0	0	0	10
0	13	1	0	6	0	2
0	13	3	2	2	0	5
0	1	1	0	0	0	6
0	6	1	0	1	0	6
1	0	1	2	1	2	1
0	0	0	2	2	0	2
0	3	1	3	0	0	3
1	2	0	0	1	1	0
0	3	0	3	3	0	4
0	1	0	0	0	0	27
0	13	0	2	0	0	2
0	11	1	0	4	0	2
0	3	0	2	0	0	2
0	5	3	0	3	0	1

0	4	1	1	0	0	2
0	11	0	2	1	0	4
0	8	1	0	3	0	0
0	3	1	1	1	0	2
0	9	0	0	2	1	4
0	7	4	5	1	0	3
0	5	0	2	2	0	0
0	7	0	0	0	0	3
1	3	0	0	3	0	1
0	1	0	0	3	1	3
0	2	1	3	2	0	1
0	3	0	2	1	0	1
0	2	0	0	1	0	2
0	2	4	0	0	0	1
0	3	0	1	0	0	1
0	8	1	1	0	0	2
0	3	0	3	1	0	1
0	3	0	0	1	0	2
0	0	0	0	0	0	8
0	8	1	0	0	0	6
0	2	5	1	4	0	1
0	3	1	0	1	0	2
0	4	1	1	1	2	1
0	2	1	0	1	1	0
0	4	1	2	1	0	1
0	1	0	0	0	0	2
0	3	1	1	3	0	0
0	4	0	1	0	0	4
2	5	3	0	1	0	5
0	15	5	1	1	0	1
0	8	1	1	1	0	5
0	0	0	0	0	0	0
0	1	0	1	1	1	1
0	2	0	2	0	0	0
0	2	1	0	1	0	1
0	1	1	0	0	1	0
0	4	1	0	0	1	1
0	4	0	0	0	0	0

0.18	0.09	0.04	0.53	0.35	0.75	0.63
21	24	28	10	15	7	9
1	1	1	1	1	1	1
78	79	80	81	82	83	84
13	14	15	16	17	18	19
D_6	E_19	E_21	E_22	E_23	E_3	E_46
CHR_P	CHR_P	CHR_P	CHR_P	CHR_P	CHR_P	CHR_P
128375	4686947	1336672	1032612	1495548	331698	1653485
CHR_P	CHR_P	CHR_P	CHR_P	CHR_P	CHR_P	CHR_P
40263	1091684	255189	377862	379984	124977	258293
28011	502157	213241	199130	172634	19968	62806
6316	164902	42264	34304	133004	68294	83643
8077	363634	74226	71113	112232	11484	53131
5640	168227	60585	62714	91720	29872	116972

5133	384358	159415	29437	72873	3409	169046
3941	241542	120763	51665	110884	11308	108609
239	170486	19447	3984	11486	855	206272
1718	160818	59440	15638	37107	4040	72117
4480	146719	27738	16747	34871	1335	30042
1574	83057	11529	5049	21295	2197	21733
1123	78420	23046	18190	18821	6539	29913
608	67728	14380	3469	31235	1127	21234
1187	47756	9645	16109	15141	2620	11470
1306	49746	11428	1692	13245	723	32632
241	15960	7283	7002	9764	7497	11053
359	68639	12706	1450	14569	432	21244
799	68787	9163	4133	9311	1693	17088
624	42508	12757	4006	9113	1012	15857
2164	22498	10337	5460	11379	1634	10414
502	37407	8453	3170	8471	1321	11161
282	37223	4087	3436	5650	671	5481
728	26441	6142	2757	6541	768	9153
230	43123	13258	2801	8605	361	15787
1096	41338	8570	1361	7492	350	19954
240	35378	5537	4177	5771	1090	7670
246	13570	2631	4416	4918	1441	4771
908	12615	4758	2076	2971	210	8490
288	28349	8096	4620	8607	1001	6149
375	21739	5854	4227	7817	878	2975
292	24465	8071	2430	5991	2016	9244
387	13614	3701	3365	5191	787	3153
90	22482	4396	1462	3296	542	16316
107	9431	1539	315	1303	187	21344
267	26556	7663	4366	8052	962	5712
158	17481	3592	5270	4749	375	2813
1211	20296	4581	2089	2878	410	4251
721	19821	8938	1413	7464	263	2794
280	9234	1750	4360	5035	303	2132
994	2083	1343	1343	1802	1161	1533
106	6288	2100	2021	2809	461	2590
395	8362	1914	1295	2664	966	3483
82	15094	3308	3782	3872	735	2936
303	7445	1250	307	1006	77	8899
124	12184	3381	2185	4911	922	7026
127	8964	2412	1302	3118	517	2193
134	10643	3997	1868	2942	373	2509
155	2285	887	1727	1377	1089	2514
1	3209	249	142	737	55	7014
105	3186	1092	221	537	337	9277
286	6693	940	226	3241	320	843
79	9428	2710	646	2436	221	3106
65	5262	1015	1432	1345	139	1216
392	3803	1802	434	1409	98	1507
28	10682	2089	583	406	260	2310
11	1912	105	56	159	42	8704
46	6295	2142	1567	1409	163	1180
61	7356	2196	1203	1553	392	2756
137	4687	1156	368	1965	310	1606
89	1394	1149	1634	1572	472	712
33	4365	807	446	987	131	1164
82	4609	1215	630	910	301	2118
46	2675	394	128	238	12	2919
5	1293	226	118	235	27	6738

100	832	225	565	622	220	332
19	6500	838	323	732	83	1197
16	4792	595	134	572	206	3920
10	5083	392	158	326	41	1577
8	5139	1069	333	801	103	1631
158	4768	1358	1688	855	25	450
87	1148	508	530	523	528	610
57	1909	353	577	485	231	456
74	3695	594	77	635	25	1907
22	3930	566	218	505	63	929
19	4700	1642	406	1573	107	1008
148	1158	185	57	566	131	563
39	3160	1065	650	596	139	735
58	1282	676	901	513	93	1403
33	936	263	315	385	279	374
27	3055	613	391	500	116	987
10	3718	680	546	745	76	665
8	1928	367	453	524	241	428
91	1251	391	610	539	141	204
4	1104	241	54	164	162	2150
20	6239	1194	447	866	40	965
6	3066	404	328	350	90	715
55	818	275	419	448	245	261
22	1568	529	74	245	27	1080
18	1279	438	117	537	77	618
70	608	424	321	389	75	209
54	1204	375	173	445	83	460
1	460	25	16	54	6	1799
5	1169	347	303	654	106	437
11	2372	686	176	341	68	450
4	1641	240	69	207	20	892
15	649	453	68	278	85	668
74	364	206	198	274	104	151
49	518	141	188	202	121	229
39	534	162	201	292	122	296
7	916	152	23	88	11	540
1	2445	174	178	181	7	259
29	931	611	323	496	72	318
40	394	130	87	294	127	309
25	1789	440	152	296	19	395
33	876	229	97	144	30	436
8	1251	126	35	98	9	733
13	726	176	126	218	88	274
17	1460	525	192	478	47	341
2	1264	154	58	124	34	596
6	1129	126	75	120	20	559
21	1499	285	70	249	28	595
14	601	168	131	213	59	251
4	425	102	50	110	49	203
3	1745	130	107	133	14	235
2	620	114	124	160	60	427
25	579	108	85	134	17	188
4	625	250	230	96	41	200
13	666	197	26	185	25	341
13	666	197	26	185	25	341
7	853	134	88	111	14	309
0	569	99	137	140	53	191
8	387	62	72	129	65	114
41	936	99	10	188	25	313

17	441	169	68	116	33	316
2	137	21	5	28	6	913
7	171	93	84	115	52	117
6	605	140	171	228	23	133
16	310	87	50	143	49	121
3	585	158	248	147	18	61
8	385	71	148	84	7	31
5	309	86	91	70	11	466
21	245	92	31	127	46	174
3	757	261	84	127	18	127
4	522	124	139	128	15	118
1	885	101	30	85	14	171
8	481	126	60	116	23	129
3	627	96	70	142	7	143
2	653	58	68	54	22	195
7	635	128	59	131	12	140
2	318	99	57	112	20	106
2	353	141	100	94	28	135
3	370	103	102	119	27	128
8	81	31	21	39	46	66
3	318	130	66	99	24	122
0	130	8	5	21	1	319
7	221	26	60	59	25	37
2	115	50	49	74	42	87
6	341	216	38	133	29	161
8	97	60	46	55	24	37
1	422	68	60	91	18	91
10	241	95	29	81	3	309
0	456	38	28	42	11	135
13	324	39	2	59	13	379
0	127	66	68	105	31	55
0	97	14	13	42	38	269
3	255	35	84	66	16	26
0	594	82	85	70	2	36
1	239	35	52	29	5	85
2	273	40	20	67	6	84
4	151	36	42	80	8	54
0	71	10	4	8	0	373
1	209	61	39	62	16	79
0	193	39	68	77	18	47
0	209	41	8	46	3	139
2	419	108	31	94	5	108
2	83	34	50	53	35	46
1	415	59	69	93	11	53
13	31	10	12	10	25	266
3	178	47	12	37	5	307
2	275	47	40	44	6	34
1	159	85	68	34	12	60
1	139	30	22	27	13	72
14	59	48	43	65	19	31
6	143	91	33	49	8	89
0	140	77	5	46	7	180
1	151	46	23	72	8	50
5	224	10	9	74	12	55
8	309	94	21	57	11	108
0	611	40	77	45	4	114
6	111	15	7	57	22	65
4	179	25	0	60	19	49
5	221	59	12	50	4	135

8	118	10	0	75	7	43
3	165	63	10	39	6	120
5	205	27	36	42	15	27
4	83	24	30	48	22	44
5	208	53	12	50	4	130
5	156	18	3	53	9	46
1	153	34	13	34	33	88
2	231	63	28	38	5	88
0	20	0	0	12	103	16
0	207	43	3	18	1	133
1	322	49	15	45	4	67
11	29	15	29	40	39	21
8	124	17	23	26	4	16
2	131	39	38	39	9	137
2	107	11	7	10	3	382
1	124	25	25	20	8	45
2	268	30	14	33	4	60
1	39	21	17	40	2	38
4	114	66	70	64	4	19
6	63	93	17	24	9	31
7	93	22	31	51	2	17
1	144	17	25	53	6	13
0	314	16	11	22	2	114
1	94	19	0	57	10	31
2	83	31	43	34	12	26
3	116	37	16	36	9	35
1	136	17	23	50	19	7
1	88	8	26	28	9	40
1	91	37	41	40	5	26
5	91	66	34	19	10	34
15	173	26	0	50	3	185
3	102	17	25	17	5	16
0	95	15	4	11	21	55
13	49	47	20	29	11	29
5	149	61	27	44	4	26
1	100	33	12	38	2	62
0	45	3	0	2	2	64
0	282	27	16	36	2	22
6	28	45	18	11	4	83
0	90	8	21	29	8	17
0	168	46	30	27	10	57
1	96	7	19	10	4	38
2	45	11	0	37	5	33
1	49	7	15	33	7	35
0	46	9	9	25	10	17
0	39	6	22	11	19	27
0	70	26	25	21	3	16
0	191	26	29	29	4	24
1	121	20	7	15	2	47
3	90	13	31	24	10	15
0	66	11	3	11	0	53
0	46	17	10	18	7	14
1	121	23	9	16	8	23
1	78	16	28	28	2	20
1	126	6	21	27	7	68
1	89	10	4	28	1	73
1	64	35	6	16	4	30
1	44	17	10	21	6	29
2	77	7	1	22	2	25

0	237	33	16	17	3	31
0	112	29	4	16	0	48
1	173	57	26	17	4	26
1	173	57	26	17	4	26
2	47	8	0	24	6	11
0	33	9	11	11	2	6
0	94	15	16	20	5	42
1	68	13	2	41	6	42
0	82	29	11	48	6	40
1	47	14	12	23	7	12
1	125	10	3	20	4	25
1	52	15	19	15	2	1
0	54	19	10	27	2	26
5	30	34	4	11	3	20
0	18	8	17	3	0	10
0	74	12	4	6	7	70
0	135	21	22	24	3	18
0	20	0	0	2	0	68
1	102	15	9	21	6	12
1	85	31	2	35	1	12
1	31	7	15	20	6	11
2	29	13	7	20	5	10
1	81	30	2	34	1	11
2	23	11	9	10	5	6
0	73	26	28	11	1	8
0	82	8	5	9	5	23
0	51	14	9	15	4	13
0	5	0	2	0	0	100
1	17	5	10	20	3	12
0	44	8	10	11	5	6
0	34	3	6	18	4	16
0	20	2	3	20	5	15
6	33	9	11	12	1	16
0	18	0	0	2	0	53
0	151	14	14	21	2	11
1	52	11	0	16	0	18
0	0	0	0	0	0	0
2	34	7	4	11	2	6
1	21	11	9	18	8	10
0	65	11	9	12	0	6
1	34	2	1	11	1	6
1	34	14	3	18	3	21
2	22	6	2	15	4	14
0	36	8	12	10	1	11
0	27	13	22	15	4	10
1	15	20	6	13	5	10
1	44	11	0	16	0	14
2	28	5	1	16	5	16
0	40	7	1	3	0	31
0	21	11	1	10	2	39
0	75	19	9	14	0	37
0	49	2	10	13	4	11
1	40	14	6	11	0	28
0	7	1	4	5	10	21
5	22	3	3	11	9	14
0	22	8	1	6	0	32
3	16	8	9	8	0	3
0	49	5	3	7	2	26
0	38	6	11	6	1	20

3	19	4	0	8	6	32
3	14	3	2	13	0	8
0	36	9	11	26	0	9
0	20	6	3	19	7	7
2	34	4	9	2	0	6
3	29	8	2	16	1	17
0	13	7	8	6	1	17
0	7	5	4	8	7	5
1	25	5	6	13	1	7
0	21	8	12	8	0	5
0	36	15	5	13	4	6
2	25	4	4	6	3	11
0	26	3	2	3	0	54
0	66	21	1	9	0	26
0	24	2	4	2	0	9
2	32	9	6	15	0	5
0	21	5	6	3	8	6
0	54	7	4	10	0	7
0	22	4	7	7	10	12
0	27	10	3	17	3	22
0	10	6	6	9	1	9
0	41	10	0	7	0	19
0	24	10	4	5	2	8
0	36	4	1	9	3	7
0	16	4	7	5	4	8
0	31	4	0	17	0	10
0	20	0	6	2	8	42
0	13	5	14	11	0	10
0	35	7	3	6	0	12
2	18	2	9	6	0	8
0	14	5	5	9	2	2
0	27	5	12	5	2	5
0	30	0	0	1	2	44
0	13	5	1	3	1	12
1	43	13	1	6	0	18
0	24	6	8	6	2	3
0	37	6	1	7	2	5
1	1	2	3	10	1	4
0	11	0	0	1	0	41
0	20	1	2	4	0	5
0	18	8	9	12	1	7
0	4	3	4	12	4	7
0	29	4	3	8	1	6
1	16	6	11	6	6	3
2	5	6	6	9	3	8
0	9	1	5	10	1	7
0	27	4	2	2	2	1
0	13	2	1	1	0	34
0	33	9	0	9	0	3
1	23	8	3	8	0	10
0	16	4	5	3	2	2
1	18	8	3	8	1	9
0	15	3	4	11	0	1
0	14	0	2	1	1	66
0	26	15	12	0	2	6
0	42	4	1	2	0	10
0	20	3	2	5	0	15
0	14	3	3	7	0	5
0	29	3	2	6	0	19

0	36	2	5	4	1	4
0	29	4	1	5	1	12
0	8	7	3	3	1	4
0	27	6	3	9	0	9
1	7	4	3	7	1	1
1	26	3	4	7	0	2
0	24	1	3	5	2	4
0	15	0	0	3	1	19
0	10	3	0	5	1	16
0	7	2	2	5	1	1
1	30	9	2	5	2	5
0	7	1	1	10	0	4
0	9	3	0	4	1	4
0	20	2	0	13	0	13
0	4	1	1	5	0	5
0	3	3	1	2	0	30
0	8	0	1	6	2	2
0	22	0	3	3	2	7
0	5	0	5	4	1	6
0	9	0	1	5	1	5
1	3	4	2	8	1	3
3	1	4	0	2	1	4
0	14	1	1	10	3	6
0	14	3	1	6	2	3
0	22	3	2	0	0	3
0	9	0	0	2	1	5
0	20	1	2	0	0	2
0	21	4	3	6	0	5
0	4	2	0	5	0	2
0	8	4	0	2	4	10
0	68	0	3	2	0	0
0	7	2	4	2	1	2
0	14	1	0	6	0	4
0	14	4	0	4	0	10
0	13	2	4	1	0	4
0	12	5	2	3	1	10
0	6	1	0	1	0	9
0	19	4	1	1	1	12
0	5	4	2	2	0	3
0	6	6	5	2	2	5
0	15	0	0	1	1	0
0	11	5	3	5	0	12
0	17	10	2	4	1	7
0	18	4	0	1	0	14
1	7	5	3	4	0	3
0	16	2	2	2	0	1
1	6	1	0	1	0	2
0	4	2	4	6	0	7
0	11	3	7	6	1	1
0	5	0	1	4	0	5
0	5	10	0	3	0	8
0	1	0	0	0	0	19
0	0	0	0	1	0	27
0	19	2	0	3	1	16
0	6	1	2	4	1	5
0	2	4	1	4	1	2
0	5	0	2	4	2	1
0	8	1	0	1	3	1
0	1	2	1	0	0	23

0	14	0	0	7	0	16
0	12	0	0	5	0	5
0	10	1	2	4	0	10
0	1	3	1	4	1	3
0	7	2	1	3	1	2
1	6	0	0	2	0	6
0	5	0	2	0	0	27
0	9	3	1	2	3	6
0	4	2	1	1	0	4
0	9	1	4	3	0	2
0	8	9	2	2	1	6
0	0	1	0	2	1	23
0	3	2	2	3	0	1
0	7	0	2	1	0	2
0	0	4	2	0	1	2
0	3	1	2	5	0	4
0	4	2	0	2	0	2
0	4	1	1	7	3	2
0	7	2	0	1	1	2
0	7	2	3	3	1	6
1	9	1	0	4	2	1
0	7	0	1	2	0	42
0	16	2	0	1	0	1
0	4	0	0	3	0	0
0	11	0	1	1	0	4
0	4	0	1	1	0	0
0	9	1	0	4	0	7
0	4	5	0	3	0	9
0	7	2	9	2	1	1
0	1	3	1	3	0	2
0	8	5	4	7	0	2
0	8	1	0	2	0	1
0	3	4	0	6	1	5
2	3	2	5	0	0	1
1	8	0	0	4	1	3
0	11	4	2	2	1	2
0	11	3	1	2	0	1
0	13	2	2	5	0	5
0	5	1	2	2	0	0
0	6	2	1	0	0	0
0	5	1	0	1	0	3
0	6	2	1	3	0	2
1	5	5	2	2	0	3
0	7	2	2	1	0	1
0	5	3	2	2	0	0
0	2	0	0	0	0	9
0	5	2	0	2	0	4
0	10	3	0	1	0	7
0	5	1	3	1	0	1
0	9	0	0	2	0	8
1	2	4	0	0	2	0
0	7	0	0	0	1	2
0	1	1	0	1	0	2
0	12	0	1	1	0	5
0	4	0	0	0	0	2
1	9	5	1	2	0	3
0	3	1	2	1	0	0
0	3	1	0	1	0	9
0	4	0	0	0	0	10

0	13	1	0	6	0	2
0	13	3	2	2	0	5
0	1	1	0	0	0	6
0	6	1	0	1	0	6
1	0	1	2	1	2	1
0	0	0	2	2	0	2
0	3	1	3	0	0	3
1	2	0	0	1	1	0
0	3	0	3	3	0	4
0	1	0	0	0	0	27
0	13	0	2	0	0	2
0	11	1	0	4	0	2
0	3	0	2	0	0	2
0	5	3	0	3	0	1
0	4	1	1	0	0	2
0	11	0	2	1	0	4
0	8	1	0	3	0	0
0	3	1	1	1	0	2
0	9	0	0	2	1	4
0	7	4	5	1	0	3
0	5	0	2	2	0	0
0	7	0	0	0	0	3
1	3	0	0	3	0	1
0	1	0	0	3	1	3
0	2	1	3	2	0	1
0	3	0	2	1	0	1
0	2	0	0	1	0	2
0	2	4	0	0	0	1
0	3	0	1	0	0	1
0	8	1	1	0	0	2
0	3	0	3	1	0	1
0	3	0	0	1	0	2
0	0	0	0	0	0	8
0	8	1	0	0	0	6
0	2	5	1	4	0	1
0	3	1	0	1	0	2
0	4	1	1	1	2	1
0	2	1	0	1	1	0
0	4	1	2	1	0	1
0	1	0	0	0	0	2
0	3	1	1	3	0	0
0	4	0	1	0	0	4
2	5	3	0	1	0	5
0	15	5	1	1	0	1
0	8	1	1	1	0	5
0	0	0	0	0	0	0
0	1	0	1	1	1	1
0	2	0	2	0	0	0
0	2	1	0	1	0	1
0	1	1	0	0	1	0
0	4	1	0	0	1	1
0	4	0	0	0	0	0
3957	141227	40732	32073	45917	10304	48172
8619	216569	63967	75813	77291	25466	64030
477	318	397	423	395	451	366
CHR_P	CHR_P	CHR_P	CHR_P	CHR_P	CHR_P	CHR_P
CHR_P	CHR_P	CHR_P	CHR_P	CHR_P	CHR_P	CHR_P

78	79	80	81	82	83	84
13	14	15	16	17	18	19
CHR_P	CHR_P	CHR_P	CHR_P	CHR_P	CHR_P	CHR_P
D_6	E_19	E_21	E_22	E_23	E_3	E_46
128375	4686947	1336672	1032612	1495548	331698	1653485
CHR_P	CHR_P	CHR_P	CHR_P	CHR_P	CHR_P	CHR_P
10.18	7.73	6.27	11.78	8.28	12.13	5.36
7.08	3.56	5.24	6.21	3.76	1.94	1.30
1.60	1.17	1.04	1.07	2.90	6.63	1.74
2.04	2.57	1.82	2.22	2.44	1.11	1.10
1.43	1.19	1.49	1.96	2.00	2.90	2.43
1.30	2.72	3.91	0.92	1.59	0.33	3.51
1.00	1.71	2.96	1.61	2.41	1.10	2.25
0.06	1.21	0.48	0.12	0.25	0.08	4.28
0.43	1.14	1.46	0.49	0.81	0.39	1.50
1.13	1.04	0.68	0.52	0.76	0.13	0.62
0.40	0.59	0.28	0.16	0.46	0.21	0.45
0.28	0.56	0.57	0.57	0.41	0.63	0.62
0.15	0.48	0.35	0.11	0.68	0.11	0.44
0.30	0.34	0.24	0.50	0.33	0.25	0.24
0.33	0.35	0.28	0.05	0.29	0.07	0.68
0.06	0.11	0.18	0.22	0.21	0.73	0.23
0.09	0.49	0.31	0.05	0.32	0.04	0.44
0.20	0.49	0.22	0.13	0.20	0.16	0.35
0.16	0.30	0.31	0.12	0.20	0.10	0.33
0.55	0.16	0.25	0.17	0.25	0.16	0.22
0.13	0.26	0.21	0.10	0.18	0.13	0.23
0.07	0.26	0.10	0.11	0.12	0.07	0.11
0.18	0.19	0.15	0.09	0.14	0.07	0.19
0.06	0.31	0.33	0.09	0.19	0.04	0.33
0.28	0.29	0.21	0.04	0.16	0.03	0.41
0.06	0.25	0.14	0.13	0.13	0.11	0.16
0.06	0.10	0.06	0.14	0.11	0.14	0.10
0.23	0.09	0.12	0.06	0.06	0.02	0.18
0.07	0.20	0.20	0.14	0.19	0.10	0.13
0.09	0.15	0.14	0.13	0.17	0.09	0.06
0.07	0.17	0.20	0.08	0.13	0.20	0.19
0.10	0.10	0.09	0.10	0.11	0.08	0.07
0.02	0.16	0.11	0.05	0.07	0.05	0.34
0.03	0.07	0.04	0.01	0.03	0.02	0.44
0.07	0.19	0.19	0.14	0.18	0.09	0.12
0.04	0.12	0.09	0.16	0.10	0.04	0.06
0.31	0.14	0.11	0.07	0.06	0.04	0.09
0.18	0.14	0.22	0.04	0.16	0.03	0.06
0.07	0.07	0.04	0.14	0.11	0.03	0.04
0.25	0.01	0.03	0.04	0.04	0.11	0.03
0.03	0.04	0.05	0.06	0.06	0.04	0.05
0.10	0.06	0.05	0.04	0.06	0.09	0.07
0.02	0.11	0.08	0.12	0.08	0.07	0.06
0.08	0.05	0.03	0.01	0.02	0.01	0.18
0.03	0.09	0.08	0.07	0.11	0.09	0.15
0.03	0.06	0.06	0.04	0.07	0.05	0.05
0.03	0.08	0.10	0.06	0.06	0.04	0.05
0.04	0.02	0.02	0.05	0.03	0.11	0.05
0.00	0.02	0.01	0.00	0.02	0.01	0.15
0.03	0.02	0.03	0.01	0.01	0.03	0.19
0.07	0.05	0.02	0.01	0.07	0.03	0.02
0.02	0.07	0.07	0.02	0.05	0.02	0.06

0.02	0.04	0.02	0.04	0.03	0.01	0.03
0.10	0.03	0.04	0.01	0.03	0.01	0.03
0.01	0.08	0.05	0.02	0.01	0.03	0.05
0.00	0.01	0.00	0.00	0.00	0.00	0.18
0.01	0.04	0.05	0.05	0.03	0.02	0.02
0.02	0.05	0.05	0.04	0.03	0.04	0.06
0.03	0.03	0.03	0.01	0.04	0.03	0.03
0.02	0.01	0.03	0.05	0.03	0.05	0.01
0.01	0.03	0.02	0.01	0.02	0.01	0.02
0.02	0.03	0.03	0.02	0.02	0.03	0.04
0.01	0.02	0.01	0.00	0.01	0.00	0.06
0.00	0.01	0.01	0.00	0.01	0.00	0.14
0.03	0.01	0.01	0.02	0.01	0.02	0.01
0.00	0.05	0.02	0.01	0.02	0.01	0.02
0.00	0.03	0.01	0.00	0.01	0.02	0.08
0.00	0.04	0.01	0.00	0.01	0.00	0.03
0.00	0.04	0.03	0.01	0.02	0.01	0.03
0.04	0.03	0.03	0.05	0.02	0.00	0.01
0.02	0.01	0.01	0.02	0.01	0.05	0.01
0.01	0.01	0.01	0.02	0.01	0.02	0.01
0.02	0.03	0.01	0.00	0.01	0.00	0.04
0.01	0.03	0.01	0.01	0.01	0.01	0.02
0.00	0.03	0.04	0.01	0.03	0.01	0.02
0.04	0.01	0.00	0.00	0.01	0.01	0.01
0.01	0.02	0.03	0.02	0.01	0.01	0.02
0.01	0.01	0.02	0.03	0.01	0.01	0.03
0.01	0.01	0.01	0.01	0.01	0.03	0.01
0.01	0.02	0.02	0.01	0.01	0.01	0.02
0.00	0.03	0.02	0.02	0.02	0.01	0.01
0.00	0.01	0.01	0.01	0.01	0.02	0.01
0.02	0.01	0.01	0.02	0.01	0.01	0.00
0.00	0.01	0.01	0.00	0.00	0.02	0.04
0.01	0.04	0.03	0.01	0.02	0.00	0.02
0.00	0.02	0.01	0.01	0.01	0.01	0.01
0.01	0.01	0.01	0.01	0.01	0.02	0.01
0.01	0.01	0.01	0.00	0.01	0.00	0.02
0.00	0.01	0.01	0.00	0.01	0.01	0.01
0.02	0.00	0.01	0.01	0.01	0.01	0.00
0.01	0.01	0.01	0.01	0.01	0.01	0.01
0.00	0.00	0.00	0.00	0.00	0.00	0.04
0.00	0.01	0.01	0.01	0.01	0.01	0.01
0.00	0.02	0.02	0.01	0.01	0.01	0.01
0.00	0.01	0.01	0.00	0.00	0.00	0.02
0.00	0.00	0.01	0.00	0.01	0.01	0.01
0.02	0.00	0.01	0.01	0.01	0.01	0.00
0.01	0.00	0.00	0.01	0.00	0.01	0.00
0.01	0.00	0.00	0.01	0.01	0.01	0.01
0.00	0.01	0.00	0.00	0.00	0.00	0.01
0.00	0.02	0.00	0.01	0.00	0.00	0.01
0.01	0.01	0.02	0.01	0.01	0.01	0.01
0.01	0.00	0.00	0.00	0.01	0.01	0.01
0.01	0.01	0.01	0.00	0.01	0.00	0.01
0.01	0.01	0.01	0.00	0.00	0.00	0.01
0.00	0.01	0.00	0.00	0.00	0.00	0.02
0.00	0.01	0.00	0.00	0.00	0.01	0.01
0.00	0.01	0.01	0.01	0.01	0.00	0.01
0.00	0.01	0.00	0.00	0.00	0.00	0.01
0.00	0.01	0.00	0.00	0.00	0.00	0.01
0.01	0.01	0.01	0.00	0.01	0.00	0.01

0.00	0.00	0.00	0.00	0.00	0.01	0.01
0.00	0.00	0.00	0.00	0.00	0.00	0.00
0.00	0.01	0.00	0.00	0.00	0.00	0.00
0.00	0.00	0.00	0.00	0.00	0.01	0.01
0.01	0.00	0.00	0.00	0.00	0.00	0.00
0.00	0.00	0.01	0.01	0.00	0.00	0.00
0.00	0.00	0.00	0.00	0.00	0.00	0.01
0.00	0.00	0.00	0.00	0.00	0.00	0.01
0.00	0.01	0.00	0.00	0.00	0.00	0.01
0.00	0.00	0.00	0.00	0.00	0.01	0.00
0.00	0.00	0.00	0.00	0.00	0.01	0.00
0.01	0.01	0.00	0.00	0.00	0.00	0.01
0.00	0.00	0.00	0.00	0.00	0.00	0.01
0.00	0.00	0.00	0.00	0.00	0.00	0.02
0.00	0.00	0.00	0.00	0.00	0.01	0.00
0.00	0.00	0.00	0.01	0.00	0.00	0.00
0.00	0.00	0.00	0.00	0.00	0.00	0.00
0.00	0.00	0.00	0.01	0.00	0.00	0.00
0.00	0.00	0.00	0.00	0.00	0.00	0.00
0.00	0.00	0.00	0.00	0.00	0.00	0.01
0.01	0.00	0.00	0.00	0.00	0.00	0.00
0.00	0.01	0.01	0.00	0.00	0.00	0.00
0.00	0.00	0.00	0.00	0.00	0.00	0.00
0.00	0.01	0.00	0.00	0.00	0.00	0.00
0.00	0.00	0.00	0.00	0.00	0.00	0.00
0.00	0.00	0.00	0.00	0.00	0.00	0.00
0.00	0.00	0.00	0.00	0.00	0.00	0.00
0.00	0.00	0.00	0.00	0.00	0.00	0.00
0.00	0.00	0.00	0.00	0.00	0.00	0.00
0.00	0.00	0.00	0.00	0.00	0.00	0.00
0.00	0.00	0.00	0.00	0.00	0.00	0.00
0.00	0.00	0.00	0.00	0.00	0.00	0.00
0.00	0.00	0.00	0.00	0.00	0.00	0.00
0.00	0.00	0.00	0.00	0.00	0.00	0.01
0.00	0.00	0.00	0.00	0.00	0.00	0.00
0.00	0.00	0.00	0.00	0.00	0.00	0.00
0.00	0.00	0.01	0.00	0.00	0.00	0.00
0.00	0.00	0.00	0.00	0.00	0.00	0.00
0.00	0.00	0.00	0.00	0.00	0.00	0.00
0.00	0.00	0.00	0.00	0.00	0.00	0.01
0.00	0.00	0.00	0.00	0.00	0.00	0.00
0.00	0.00	0.00	0.00	0.00	0.00	0.01
0.00	0.00	0.00	0.00	0.00	0.00	0.00
0.00	0.00	0.00	0.00	0.00	0.00	0.01
0.00	0.00	0.00	0.00	0.00	0.00	0.00
0.00	0.00	0.00	0.00	0.00	0.00	0.00
0.00	0.00	0.00	0.00	0.00	0.00	0.00
0.00	0.00	0.00	0.00	0.00	0.00	0.00
0.00	0.00	0.00	0.00	0.00	0.00	0.00
0.00	0.00	0.00	0.00	0.00	0.00	0.01
0.00	0.00	0.00	0.00	0.00	0.00	0.00
0.00	0.00	0.00	0.00	0.00	0.00	0.00
0.00	0.00	0.00	0.00	0.00	0.00	0.00
0.00	0.00	0.00	0.00	0.00	0.00	0.00
0.00	0.00	0.00	0.00	0.00	0.00	0.00
0.00	0.00	0.00	0.00	0.00	0.00	0.00
0.00	0.00	0.00	0.00	0.00	0.00	0.01
0.00	0.00	0.00	0.00	0.00	0.00	0.01
0.00	0.00	0.00	0.00	0.00	0.00	0.00

0.00	0.00	0.00	0.00	0.00	0.00	0.00
0.00	0.00	0.00	0.00	0.00	0.00	0.00
0.00	0.00	0.00	0.00	0.00	0.00	0.00
0.00	0.00	0.00	0.00	0.00	0.00	0.00
0.00	0.00	0.00	0.00	0.00	0.00	0.00
0.00	0.00	0.00	0.00	0.00	0.00	0.00
0.00	0.00	0.00	0.00	0.00	0.00	0.00
0.00	0.00	0.00	0.00	0.00	0.00	0.00
0.00	0.00	0.00	0.00	0.00	0.00	0.00
0.00	0.00	0.00	0.00	0.00	0.00	0.00
0.00	0.00	0.00	0.00	0.00	0.00	0.00
0.00	0.00	0.00	0.00	0.00	0.00	0.00
0.00	0.00	0.00	0.00	0.00	0.00	0.00
0.00	0.00	0.00	0.00	0.00	0.00	0.00
0.00	0.00	0.00	0.00	0.00	0.00	0.00
0.00	0.00	0.00	0.00	0.00	0.00	0.00
0.00	0.00	0.00	0.00	0.00	0.00	0.00
0.00	0.00	0.00	0.00	0.00	0.00	0.00
0.00	0.00	0.00	0.00	0.00	0.00	0.00
0.00	0.00	0.00	0.00	0.00	0.00	0.00
0.00	0.00	0.00	0.00	0.00	0.01	0.00
0.00	0.00	0.00	0.00	0.00	0.00	0.00
0.00	0.00	0.00	0.00	0.00	0.00	0.00
0.00	0.00	0.00	0.00	0.00	0.00	0.00
0.00	0.00	0.00	0.00	0.00	0.00	0.00
0.00	0.00	0.00	0.00	0.00	0.00	0.00
0.00	0.00	0.00	0.00	0.00	0.00	0.01
0.00	0.00	0.00	0.00	0.00	0.00	0.00
0.00	0.00	0.00	0.00	0.00	0.00	0.00
0.00	0.00	0.00	0.00	0.00	0.00	0.00
0.00	0.00	0.00	0.00	0.00	0.00	0.00
0.00	0.00	0.00	0.00	0.00	0.00	0.00
0.00	0.00	0.00	0.00	0.00	0.00	0.00
0.00	0.00	0.00	0.00	0.00	0.00	0.00
0.00	0.00	0.00	0.00	0.00	0.00	0.00
0.00	0.00	0.00	0.00	0.00	0.00	0.00
0.00	0.00	0.00	0.00	0.00	0.00	0.00
0.00	0.00	0.00	0.00	0.00	0.00	0.00
0.00	0.00	0.00	0.00	0.00	0.00	0.00
0.00	0.00	0.00	0.00	0.00	0.00	0.00
0.00	0.00	0.00	0.00	0.00	0.00	0.00
0.00	0.00	0.00	0.00	0.00	0.00	0.00
0.00	0.00	0.00	0.00	0.00	0.00	0.00
0.00	0.00	0.00	0.00	0.00	0.00	0.00
0.00	0.00	0.00	0.00	0.00	0.00	0.00
0.00	0.00	0.00	0.00	0.00	0.00	0.00
0.00	0.00	0.00	0.00	0.00	0.00	0.00
0.00	0.00	0.00	0.00	0.00	0.00	0.00
0.00	0.00	0.00	0.00	0.00	0.00	0.00
0.00	0.00	0.00	0.00	0.00	0.00	0.00
0.00	0.00	0.00	0.00	0.00	0.00	0.00
0.00	0.00	0.00	0.00	0.00	0.00	0.00
0.00	0.00	0.00	0.00	0.00	0.00	0.00
0.00	0.00	0.00	0.00	0.00	0.00	0.00
0.00	0.00	0.00	0.00	0.00	0.00	0.00
0.00	0.00	0.00	0.00	0.00	0.00	0.00
0.00	0.00	0.00	0.00	0.00	0.00	0.00
0.00	0.00	0.00	0.00	0.00	0.00	0.00
0.00	0.00	0.00	0.00	0.00	0.00	0.00

0.00	0.00	0.00	0.00	0.00	0.00	0.00
0.00	0.00	0.00	0.00	0.00	0.00	0.00
0.00	0.00	0.00	0.00	0.00	0.00	0.00
0.00	0.00	0.00	0.00	0.00	0.00	0.00
0.00	0.00	0.00	0.00	0.00	0.00	0.00
0.00	0.00	0.00	0.00	0.00	0.00	0.00
0.00	0.00	0.00	0.00	0.00	0.00	0.00
0.00	0.00	0.00	0.00	0.00	0.00	0.00
0.00	0.00	0.00	0.00	0.00	0.00	0.00
0.00	0.00	0.00	0.00	0.00	0.00	0.00
0.00	0.00	0.00	0.00	0.00	0.00	0.00
0.00	0.00	0.00	0.00	0.00	0.00	0.00
0.00	0.00	0.00	0.00	0.00	0.00	0.00
0.00	0.00	0.00	0.00	0.00	0.00	0.00
0.00	0.00	0.00	0.00	0.00	0.00	0.00
0.00	0.00	0.00	0.00	0.00	0.00	0.00
0.00	0.00	0.00	0.00	0.00	0.00	0.00
0.00	0.00	0.00	0.00	0.00	0.00	0.00
0.00	0.00	0.00	0.00	0.00	0.00	0.00
0.00	0.00	0.00	0.00	0.00	0.00	0.00
0.00	0.00	0.00	0.00	0.00	0.00	0.00
0.00	0.00	0.00	0.00	0.00	0.00	0.00
0.00	0.00	0.00	0.00	0.00	0.00	0.00
0.00	0.00	0.00	0.00	0.00	0.00	0.00
0.00	0.00	0.00	0.00	0.00	0.00	0.00
0.00	0.00	0.00	0.00	0.00	0.00	0.00
0.00	0.00	0.00	0.00	0.00	0.00	0.00
0.00	0.00	0.00	0.00	0.00	0.00	0.00
0.00	0.00	0.00	0.00	0.00	0.00	0.00
0.00	0.00	0.00	0.00	0.00	0.00	0.00

78	79	80	81	82	83	84
14	15	16	17	18	19	20
CHR_P	CHR_P	CHR_P	CHR_P	CHR_P	CHR_P	CHR_P
D_6	E_19	E_21	E_22	E_23	E_3	E_46
128375	4686947	1336672	1032612	1495548	331698	1653485
D_6	E_19	E_21	E_22	E_23	E_3	E_46
-0.32	-0.87	-1.20	0.04	-0.75	0.11	-1.40
1.12	0.08	0.58	0.86	0.14	-0.39	-0.58
-0.39	-0.71	-0.81	-0.78	0.59	3.40	-0.28
-0.40	0.12	-0.62	-0.23	0.00	-1.32	-1.33
-0.82	-1.07	-0.75	-0.23	-0.18	0.81	0.29
0.10	1.38	2.46	-0.25	0.36	-0.78	2.10
-0.27	0.87	2.86	0.71	1.99	-0.11	1.73
-0.51	1.56	0.25	-0.39	-0.17	-0.47	7.12

-0.31	1.09	1.73	-0.20	0.43	-0.39	1.80
0.96	0.72	-0.20	-0.61	0.00	-1.62	-0.35
-0.28	0.44	-0.71	-1.18	-0.03	-0.97	-0.08
-1.86	0.57	0.66	0.67	-0.73	1.28	1.16
-1.17	0.70	-0.03	-1.44	1.86	-1.43	0.48
-0.96	-0.74	-1.32	0.20	-0.79	-1.22	-1.32
0.51	0.61	0.28	-0.77	0.32	-0.69	2.11
-1.03	-0.80	-0.51	-0.34	-0.36	1.88	-0.29
-0.42	2.07	0.97	-0.71	1.01	-0.73	1.79
-0.12	2.68	0.10	-0.84	-0.11	-0.49	1.38
-0.21	1.07	1.18	-0.51	0.15	-0.74	1.32
3.15	-0.71	0.23	-0.60	0.17	-0.72	-0.14
-0.49	1.73	0.81	-0.94	0.44	-0.47	1.20
-1.23	1.72	-0.78	-0.68	-0.43	-1.32	-0.58
0.81	0.85	0.40	-0.41	0.29	-0.55	0.88
-0.58	1.76	1.95	-0.30	0.64	-0.79	1.97
0.87	0.96	0.48	-0.52	0.20	-0.57	1.68
-1.02	2.55	0.40	0.29	0.20	-0.17	0.83
-1.21	-0.67	-1.17	-0.01	-0.50	0.03	-0.62
1.12	-0.22	0.04	-0.46	-0.46	-0.89	0.61
-0.67	1.82	1.78	0.72	1.56	-0.20	0.40
-0.17	1.21	0.97	0.69	1.59	-0.40	-0.94
-0.39	1.93	2.51	-0.34	0.93	2.45	2.36
-0.47	-0.49	-0.60	-0.33	-0.17	-0.88	-1.09
-0.56	1.50	0.73	-0.21	0.18	-0.11	4.21
-0.25	-0.03	-0.19	-0.35	-0.24	-0.30	2.02
-0.61	2.09	2.10	0.93	1.81	-0.03	0.54
-0.63	-0.12	-0.34	0.13	-0.24	-0.65	-0.52
2.28	0.36	-0.01	-0.57	-0.60	-0.87	-0.30
0.92	0.49	1.31	-0.50	0.72	-0.69	-0.35
-0.34	-0.41	-0.68	0.44	0.12	-0.84	-0.66
1.22	-0.68	-0.54	-0.47	-0.49	0.11	-0.55
-1.01	-0.64	-0.50	-0.26	-0.30	-0.64	-0.45
0.94	-0.28	-0.65	-0.85	-0.32	0.76	0.11
-1.76	1.72	0.68	2.17	0.81	0.28	-0.14
0.97	0.38	-0.17	-0.69	-0.39	-0.74	3.65
-0.66	0.51	0.44	0.12	0.95	0.57	1.77
-1.04	1.23	0.93	-0.42	1.56	0.27	-0.07
-0.28	1.39	2.31	0.70	0.94	-0.19	0.45
-0.35	-1.11	-0.92	0.14	-0.65	1.86	0.09
-0.39	0.40	-0.19	-0.24	0.16	-0.21	4.72
-0.08	-0.17	-0.08	-0.53	-0.42	0.06	3.71
1.99	0.65	-0.66	-1.53	1.90	-0.23	-0.97
-0.42	0.95	0.94	-0.42	0.55	-0.38	0.88
-1.49	0.12	-0.83	0.69	-0.49	-1.72	-0.81
3.69	-0.11	0.80	-0.82	0.08	-1.03	0.12
-0.99	2.80	1.46	-0.38	-0.89	0.01	1.27

-0.27	0.17	-0.28	-0.31	-0.24	-0.22	7.00
-1.32	1.33	1.98	1.68	0.21	-0.99	-0.29
-0.71	2.33	2.48	1.12	0.81	1.16	2.75
0.30	0.19	-0.16	-1.42	0.91	-0.04	0.21
-0.48	-0.82	-0.33	0.26	-0.18	0.13	-0.69
-1.31	0.51	-0.38	-0.86	-0.25	-0.95	-0.03
-0.11	0.88	0.65	-0.20	-0.18	0.59	1.82
0.18	0.75	0.03	-0.42	-0.32	-0.64	4.01
-0.24	0.12	-0.04	-0.13	-0.06	-0.18	6.06
-0.24	-1.36	-1.38	-0.68	-0.92	-0.47	-1.30
-1.13	2.84	0.39	-0.62	-0.06	-0.81	0.80
-0.59	1.60	0.18	-0.58	0.03	0.58	5.08
-0.78	2.54	-0.08	-0.54	-0.33	-0.64	2.21
-1.22	2.70	1.54	-0.27	0.54	-0.31	2.41
0.09	0.01	0.00	0.25	-0.19	-0.40	-0.31
0.03	-0.95	-0.64	-0.36	-0.72	2.09	-0.63
-0.59	-0.68	-1.16	-0.24	-0.97	0.20	-1.08
0.81	1.50	0.42	-0.72	0.35	-0.72	2.76
-1.09	1.84	0.00	-0.93	-0.38	-1.02	0.71
-0.77	2.76	3.63	0.20	2.88	-0.08	1.23
1.67	-0.74	-1.04	-1.27	-0.40	-0.37	-0.45
-0.56	0.96	1.41	0.70	-0.18	-0.12	0.09
-0.17	-0.32	-0.12	0.18	-0.27	-0.32	0.21
-0.85	-1.00	-1.02	-0.71	-0.84	0.87	-0.90
-0.74	0.64	0.02	-0.24	-0.36	-0.33	0.53
-1.67	3.01	1.12	1.18	1.02	-0.72	0.55
-1.47	0.19	-0.47	0.26	-0.13	1.59	-0.49
0.60	-0.71	-0.64	0.23	-0.44	-0.26	-1.14
-0.46	-0.15	-0.24	-0.43	-0.35	0.22	1.57
-0.48	6.11	3.61	1.01	1.84	-0.68	2.04
-1.28	2.29	0.20	0.26	-0.20	-0.01	1.08
0.14	-1.47	-1.28	-0.02	-0.68	2.12	-1.55
-0.09	0.82	1.13	-0.63	-0.13	-0.58	2.69
-1.01	0.18	0.63	-1.25	0.88	-0.24	1.18
0.72	-0.93	-0.18	-0.23	-0.42	-0.57	-0.93
1.47	0.15	0.33	-0.65	0.45	0.03	0.42
-0.27	0.30	-0.20	-0.22	-0.10	-0.21	6.69
-1.43	0.43	0.50	0.74	2.02	0.97	0.64
-0.60	2.24	2.25	-0.05	0.34	0.17	0.73
-0.51	1.52	0.42	-0.29	0.16	-0.33	2.83
-0.44	-0.28	1.02	-0.77	0.01	0.45	1.57
2.58	-1.27	-0.68	-0.41	-0.46	0.52	-1.14
2.09	-1.25	-1.32	-0.41	-0.97	1.84	-0.83
0.60	-0.85	-0.80	-0.26	-0.24	1.07	-0.29
-0.41	0.68	0.04	-0.65	-0.37	-0.57	1.76
-0.97	1.99	-0.27	-0.05	-0.33	-0.90	-0.08
0.94	0.65	4.04	2.05	2.34	0.81	0.65
0.54	-1.02	-0.93	-1.03	-0.25	1.02	-0.24

-0.17	0.36	0.20	-0.30	-0.16	-0.54	-0.01
1.24	0.53	0.33	-0.53	-0.49	-0.56	1.47
-0.40	1.57	-0.09	-0.66	-0.36	-0.73	3.40
-0.64	-0.08	-0.33	-0.45	-0.20	0.94	0.08
-0.34	0.54	0.91	-0.10	0.55	-0.30	0.06
-1.03	1.65	0.01	-0.62	-0.34	-0.15	2.73
-0.77	1.69	-0.17	-0.45	-0.35	-0.61	3.05
0.38	2.25	0.97	-0.73	0.42	-0.54	2.86
-0.59	-0.22	-0.29	-0.31	-0.03	0.52	0.26
-1.03	-0.37	-0.53	-0.85	-0.57	0.21	0.03
-1.02	3.65	-0.04	0.02	-0.16	-0.78	0.64
-1.29	0.16	-0.43	-0.04	-0.18	0.69	1.83
0.64	-0.32	-0.94	-0.94	-0.83	-1.38	-0.40
-1.39	0.79	1.89	2.55	-0.70	0.51	0.62
0.04	0.64	0.69	-1.01	0.35	-0.33	1.64
0.04	0.64	0.69	-1.01	0.35	-0.33	1.64
-0.72	0.47	-0.29	-0.45	-0.54	-0.83	0.57
-1.28	0.17	-0.41	0.25	-0.18	0.57	0.14
-0.82	-0.38	-1.13	-0.69	-0.34	1.80	-0.61
2.79	1.44	-0.08	-0.85	0.52	-0.08	1.39
0.71	0.19	0.65	-0.26	-0.08	0.22	1.72
-0.21	-0.04	-0.20	-0.34	-0.17	-0.18	6.52
-0.90	-1.14	-0.68	-0.53	-0.58	0.51	-0.62
-0.64	0.73	0.31	1.25	1.07	-0.28	-0.02
0.57	-0.39	-0.42	-0.71	0.09	0.94	-0.22
-0.89	0.30	0.21	1.56	-0.03	-0.54	-0.71
-0.36	-0.20	-0.42	0.23	-0.40	-0.66	-0.67
-0.23	0.16	0.13	0.43	-0.12	-0.31	3.29
2.42	-0.37	0.04	-0.97	0.44	1.76	1.10
-0.99	2.25	2.99	0.32	0.42	-0.30	0.33
-0.71	0.35	0.09	0.60	-0.01	-0.53	-0.14
-0.72	2.73	0.56	-0.32	0.20	-0.08	1.18
-0.24	0.97	0.70	-0.37	0.20	-0.06	0.33

7	8	9	10	11	12	13
78	79	80	81	82	83	84
TAGCACCATCT(	GAATGTTGCTCG(	ATGCACCTGGGCA	CACCCGTAGAA(	TGCGGGGCTAG(	ATCACATTGCCA(	AATTGCACGGTAT
miR-29a-3p	miR-409-3p	miR-500a-3p	miR-99b-5p	miR-744-5p	miR-23b-3p	miR-363-3p
67482	67182	65049	56245	56410	53026	52323
-0.31	1.96	0.19	-0.29	-0.65	0.09	-0.38
-0.98	-1.03	-1.38	4.80	-1.18	-0.50	0.32
-0.02	-0.56	-0.24	-0.35	-1.00	-1.08	2.78
-0.65	-0.95	-0.46	-0.46	-0.95	-1.12	1.35
2.85	-0.18	-1.70	0.21	1.81	2.84	0.79
-0.78	0.24	-0.54	-0.42	0.20	-0.09	-0.86
-0.85	2.38	-0.83	-0.42	1.91	0.59	-1.21
-0.83	0.06	-0.71	-0.40	0.69	-0.77	-0.90
-1.06	-0.21	0.46	-0.28	-1.00	-0.80	1.71
-0.90	-0.70	-0.03	-0.10	-1.21	0.17	-0.28
0.15	-0.74	0.84	0.49	-0.98	1.04	0.74
-0.87	-0.74	-0.82	-0.41	1.97	-1.08	-1.29
0.89	-1.19	-0.43	-0.43	-0.38	1.46	0.27
-0.94	0.34	-0.66	-0.19	0.36	-0.46	-0.96
-0.19	-0.46	1.68	-0.26	-0.34	-0.59	0.26
1.67	0.03	0.81	-0.25	-0.28	2.25	0.68
0.05	-0.29	2.72	-0.19	-0.15	0.04	0.36
0.35	1.15	0.37	-0.34	1.42	1.06	0.82
0.53	-0.76	1.04	-0.31	-0.88	-0.57	-1.07
1.56	-1.14	0.09	-0.19	-0.79	-0.85	0.90

-0.42	-0.75	-0.77	-0.31	-0.75	-0.28	-0.60
-0.14	2.50	0.36	-0.11	1.07	-0.56	-0.83
-0.16	0.18	0.33	-0.27	0.67	-0.03	-0.34
-0.79	-0.12	0.07	-0.33	0.57	-0.11	-0.98
0.00	0.76	-1.63	0.78	0.10	-0.22	-0.84
1.88	0.06	-0.04	-0.03	0.62	0.45	-0.50
-0.02	0.13	1.27	0.07	-0.86	-0.90	0.09
0.53	-0.73	-0.61	-0.43	-1.22	-1.07	2.43
1.60	0.01	1.06	-0.11	-1.10	-0.73	-0.36
-0.40	-0.81	0.22	-0.07	-1.14	-1.05	-0.79
-1.23	0.08	-1.37	1.65	-1.10	-0.81	-0.67
-0.68	0.39	-1.44	0.59	-1.26	-1.08	-0.27
3.83	-0.66	-1.73	2.03	0.42	1.72	4.17
-0.48	-0.02	-0.75	-0.51	0.45	-0.49	-0.84
-0.41	-0.34	-0.96	-0.45	-0.20	0.51	-0.40
0.51	-1.09	-0.58	-0.44	-0.06	0.03	-0.52
-1.29	-0.78	-1.24	-0.48	0.37	-1.06	-1.15
-0.50	0.85	-0.67	-0.43	-1.13	2.57	-1.35
0.80	-0.17	-0.71	-0.28	-1.07	2.06	-0.10
0.79	-0.62	-0.34	-0.39	-0.29	-0.17	0.33
-0.82	0.48	2.99	-0.07	1.74	-0.47	-1.18
-0.92	2.56	5.49	1.30	3.41	0.73	-0.05
-0.09	0.10	0.07	-0.09	0.19	0.17	-0.55
0.79	-0.81	1.14	-0.20	-0.68	0.30	-0.62
-0.65	-0.15	-0.17	-0.21	0.48	-0.58	-1.57
-1.11	-0.40	2.21	-0.32	1.62	-0.96	-1.57
5.19	-0.81	0.78	-0.14	-0.90	-0.37	1.61
0.73	0.72	0.13	-0.39	0.18	0.33	-0.53
0.53	-1.14	-0.12	-0.30	-0.50	-0.26	0.10
2.18	-0.19	0.15	-0.41	-0.63	-0.58	0.30
1.43	0.64	-0.15	-0.37	-0.65	0.52	0.43
0.38	3.15	0.53	0.21	0.09	1.11	-0.98
-0.70	2.14	-0.02	0.42	-0.23	0.63	-0.84
1.66	-0.22	0.52	-0.02	0.40	0.65	-0.12
-0.36	-0.61	-0.44	-0.20	-0.02	-0.66	-0.88
0.14	1.11	0.68	0.53	-0.53	-0.05	-0.37
0.03	4.04	-0.34	0.32	-0.67	-0.35	0.15
2.18	-1.23	-0.28	-0.27	-0.92	-0.92	0.44
0.74	1.27	-0.83	-0.21	0.46	-0.58	-0.96
3.60	-0.71	-1.50	-0.22	0.74	0.53	0.37
5.59	2.18	-1.52	-0.03	0.38	0.52	0.46
0.90	-0.59	0.07	-0.29	-1.10	-1.15	1.17
-0.39	-0.98	0.87	0.28	-0.20	-1.03	-0.59
0.23	-0.20	1.22	0.16	-0.09	-0.89	-0.59
0.01	-1.20	1.34	-0.18	-0.88	-1.09	0.92
-0.88	-0.30	-0.27	-0.49	0.99	-1.24	-0.61
-0.02	-0.76	-0.20	-0.38	-1.41	-0.42	1.21
-0.31	-0.92	-1.76	2.88	-1.39	0.33	-1.21
-0.20	1.12	-1.68	-0.44	0.89	-0.04	3.19
2.06	1.53	-1.58	-0.33	0.34	-0.53	4.29
-0.52	0.04	-0.68	-0.41	-0.49	-1.06	-0.47
0.35	-0.47	-1.39	-0.54	-1.21	-1.16	-1.73
-0.57	-0.81	-0.38	-0.40	-0.74	-0.37	1.62
-0.20	0.31	-0.90	-0.43	-0.23	0.03	-0.35
-0.75	1.19	2.88	-0.14	4.02	0.68	-1.18
0.61	1.10	-0.24	-0.41	0.18	-0.44	0.22
0.21	-0.78	-0.29	-0.16	-1.01	-0.61	-1.14
-0.77	1.67	-0.56	-0.17	-0.85	-0.74	-1.67
2.76	-0.74	0.96	-0.32	-1.00	0.64	3.01

3.63	-1.04	1.41	-0.12	-1.02	0.02	1.12
0.20	-1.27	0.70	0.18	-0.71	-0.24	1.18
2.88	-0.40	-0.18	-0.27	-0.84	-0.36	1.02
-0.08	-0.37	-0.12	-0.32	0.87	-0.33	-0.72
1.23	-0.45	0.09	0.21	-0.90	0.53	0.55
0.49	-0.90	-0.38	-0.42	-1.28	-1.10	0.99
1.27	-1.01	0.28	-0.01	-0.48	-0.09	0.42
2.79	-0.59	0.25	-0.36	-0.64	-0.33	0.25
0.99	-1.22	-0.30	-0.36	-0.99	-0.55	1.41
0.92	-0.94	0.40	-0.40	-0.73	-0.55	-0.56
2.22	-0.96	-0.11	0.19	-0.32	-0.17	0.81
0.16	0.35	-0.12	-0.35	-0.62	-1.14	-0.04
-0.28	-0.31	-0.57	-0.33	-1.01	-0.74	-1.21
0.04	1.29	0.53	-0.20	0.54	-0.60	-0.01
-0.03	-0.97	1.73	0.03	-1.06	-0.53	-0.68
0.32	-0.17	2.33	-0.03	-1.04	0.31	-0.27
1	-2	-1	-1	-1	0	1
0.00	0.00	0.00	0.00	0.00	0.00	0.00
1.00	1.00	1.00	1.00	1.00	1.00	1.00
0.97	0.17	0.53	0.33	0.36	0.12	0.16

75	76	77	78	79	80	81
TAGCACCATCT(	GAATGTTGCTCG(	ATGCACCTGGGCA	CACCCGTAGAA(	TGCGGGGCTAG(	ATCACATTGCCA(	AATTGCACGGTAT
miR-29a-3p	miR-409-3p	miR-500a-3p	miR-99b-5p	miR-744-5p	miR-23b-3p	miR-363-3p
67482	67182	65049	56245	56410	53026	52323
-0.31	1.96	0.19	-0.29	-0.65	0.09	-0.38
-0.98	-1.03	-1.38	4.80	-1.18	-0.50	0.32
-0.02	-0.56	-0.24	-0.35	-1.00	-1.08	2.78
-0.65	-0.95	-0.46	-0.46	-0.95	-1.12	1.35
2.85	-0.18	-1.70	0.21	1.81	2.84	0.79
-0.78	0.24	-0.54	-0.42	0.20	-0.09	-0.86
-0.85	2.38	-0.83	-0.42	1.91	0.59	-1.21
-0.83	0.06	-0.71	-0.40	0.69	-0.77	-0.90
-1.06	-0.21	0.46	-0.28	-1.00	-0.80	1.71
-0.90	-0.70	-0.03	-0.10	-1.21	0.17	-0.28
0.15	-0.74	0.84	0.49	-0.98	1.04	0.74
-0.87	-0.74	-0.82	-0.41	1.97	-1.08	-1.29
0.89	-1.19	-0.43	-0.43	-0.38	1.46	0.27
-0.94	0.34	-0.66	-0.19	0.36	-0.46	-0.96
-0.19	-0.46	1.68	-0.26	-0.34	-0.59	0.26
1.67	0.03	0.81	-0.25	-0.28	2.25	0.68
0.05	-0.29	2.72	-0.19	-0.15	0.04	0.36
0.35	1.15	0.37	-0.34	1.42	1.06	0.82
0.53	-0.76	1.04	-0.31	-0.88	-0.57	-1.07
1.56	-1.14	0.09	-0.19	-0.79	-0.85	0.90
-0.42	-0.75	-0.77	-0.31	-0.75	-0.28	-0.60
-0.14	2.50	0.36	-0.11	1.07	-0.56	-0.83
-0.16	0.18	0.33	-0.27	0.67	-0.03	-0.34
-0.79	-0.12	0.07	-0.33	0.57	-0.11	-0.98
0.00	0.76	-1.63	0.78	0.10	-0.22	-0.84
1.88	0.06	-0.04	-0.03	0.62	0.45	-0.50
-0.02	0.13	1.27	0.07	-0.86	-0.90	0.09
0.53	-0.73	-0.61	-0.43	-1.22	-1.07	2.43

1.60	0.01	1.06	-0.11	-1.10	-0.73	-0.36
-0.40	-0.81	0.22	-0.07	-1.14	-1.05	-0.79
-1.23	0.08	-1.37	1.65	-1.10	-0.81	-0.67
-0.68	0.39	-1.44	0.59	-1.26	-1.08	-0.27
3.83	-0.66	-1.73	2.03	0.42	1.72	4.17
-0.48	-0.02	-0.75	-0.51	0.45	-0.49	-0.84
-0.41	-0.34	-0.96	-0.45	-0.20	0.51	-0.40
0.51	-1.09	-0.58	-0.44	-0.06	0.03	-0.52
-1.29	-0.78	-1.24	-0.48	0.37	-1.06	-1.15
-0.50	0.85	-0.67	-0.43	-1.13	2.57	-1.35
0.80	-0.17	-0.71	-0.28	-1.07	2.06	-0.10
0.79	-0.62	-0.34	-0.39	-0.29	-0.17	0.33
-0.82	0.48	2.99	-0.07	1.74	-0.47	-1.18
-0.92	2.56	5.49	1.30	3.41	0.73	-0.05
-0.09	0.10	0.07	-0.09	0.19	0.17	-0.55
0.79	-0.81	1.14	-0.20	-0.68	0.30	-0.62
-0.65	-0.15	-0.17	-0.21	0.48	-0.58	-1.57
-1.11	-0.40	2.21	-0.32	1.62	-0.96	-1.57
5.19	-0.81	0.78	-0.14	-0.90	-0.37	1.61
0.73	0.72	0.13	-0.39	0.18	0.33	-0.53
0.53	-1.14	-0.12	-0.30	-0.50	-0.26	0.10
2.18	-0.19	0.15	-0.41	-0.63	-0.58	0.30
1.43	0.64	-0.15	-0.37	-0.65	0.52	0.43
0.38	3.15	0.53	0.21	0.09	1.11	-0.98
-0.70	2.14	-0.02	0.42	-0.23	0.63	-0.84
1.66	-0.22	0.52	-0.02	0.40	0.65	-0.12
-0.36	-0.61	-0.44	-0.20	-0.02	-0.66	-0.88
0.14	1.11	0.68	0.53	-0.53	-0.05	-0.37
0.03	4.04	-0.34	0.32	-0.67	-0.35	0.15
2.18	-1.23	-0.28	-0.27	-0.92	-0.92	0.44
0.74	1.27	-0.83	-0.21	0.46	-0.58	-0.96
3.60	-0.71	-1.50	-0.22	0.74	0.53	0.37
5.59	2.18	-1.52	-0.03	0.38	0.52	0.46
0.90	-0.59	0.07	-0.29	-1.10	-1.15	1.17
-0.39	-0.98	0.87	0.28	-0.20	-1.03	-0.59
0.23	-0.20	1.22	0.16	-0.09	-0.89	-0.59
0.01	-1.20	1.34	-0.18	-0.88	-1.09	0.92
-0.88	-0.30	-0.27	-0.49	0.99	-1.24	-0.61
-0.02	-0.76	-0.20	-0.38	-1.41	-0.42	1.21
-0.31	-0.92	-1.76	2.88	-1.39	0.33	-1.21
-0.20	1.12	-1.68	-0.44	0.89	-0.04	3.19
2.06	1.53	-1.58	-0.33	0.34	-0.53	4.29
-0.52	0.04	-0.68	-0.41	-0.49	-1.06	-0.47
0.35	-0.47	-1.39	-0.54	-1.21	-1.16	-1.73
-0.57	-0.81	-0.38	-0.40	-0.74	-0.37	1.62
-0.20	0.31	-0.90	-0.43	-0.23	0.03	-0.35
-0.75	1.19	2.88	-0.14	4.02	0.68	-1.18
0.61	1.10	-0.24	-0.41	0.18	-0.44	0.22
0.21	-0.78	-0.29	-0.16	-1.01	-0.61	-1.14
-0.77	1.67	-0.56	-0.17	-0.85	-0.74	-1.67
2.76	-0.74	0.96	-0.32	-1.00	0.64	3.01
3.63	-1.04	1.41	-0.12	-1.02	0.02	1.12
0.20	-1.27	0.70	0.18	-0.71	-0.24	1.18
2.88	-0.40	-0.18	-0.27	-0.84	-0.36	1.02
-0.08	-0.37	-0.12	-0.32	0.87	-0.33	-0.72
1.23	-0.45	0.09	0.21	-0.90	0.53	0.55
0.49	-0.90	-0.38	-0.42	-1.28	-1.10	0.99
1.27	-1.01	0.28	-0.01	-0.48	-0.09	0.42
2.79	-0.59	0.25	-0.36	-0.64	-0.33	0.25

0.99	-1.22	-0.30	-0.36	-0.99	-0.55	1.41
0.92	-0.94	0.40	-0.40	-0.73	-0.55	-0.56
2.22	-0.96	-0.11	0.19	-0.32	-0.17	0.81
0.16	0.35	-0.12	-0.35	-0.62	-1.14	-0.04
-0.28	-0.31	-0.57	-0.33	-1.01	-0.74	-1.21
0.04	1.29	0.53	-0.20	0.54	-0.60	-0.01
-0.03	-0.97	1.73	0.03	-1.06	-0.53	-0.68
0	0	2	0	-1	0	0

75	76	77	78	79	80	81
32	99	2	87	132	44	128
AGAGGTAGTAG(	TGCCCTGTGGACT	CAACGGAATCCCA	CTGGCCCTCTC	CCTGTTCTCCA	CTGACCTATGAAT	TGAGCGCCTCGAC
let-7d-5p	miR-146b-3p	miR-191-5p	miR-328	miR-26b-3p	miR-192-5p	miR-339-3p
463804	27199	10497180	48416	11100	248983	12315.00
-0.85	-0.05	-0.77	-0.41	-0.35	-0.83	0.88
-1.63	2.39	3.44	0.21	-1.72	1.81	-1.52
0.95	-0.05	-0.58	-1.05	-0.62	0.07	-0.69
0.18	-0.45	-0.72	-1.30	-0.69	-0.61	-0.69
1.13	1.49	0.71	1.15	-0.58	-0.23	-1.02
-0.56	-1.03	-0.50	0.60	0.02	-0.70	-0.78
-0.06	-0.60	-0.74	1.82	-0.64	-0.79	0.66
-0.05	-0.43	-0.53	2.04	0.34	-0.79	0.73
-0.90	-1.26	-0.06	0.12	-1.16	0.01	-1.17
-1.10	-1.14	-0.19	-1.15	-1.32	1.07	-1.20
-0.72	0.08	0.29	-0.32	-0.95	1.19	-1.17
0.89	-0.45	-0.81	0.75	0.86	-0.72	-0.11
-0.36	-1.03	-0.57	-1.36	2.27	1.93	1.27
0.57	0.04	-0.55	0.66	-0.78	-0.73	0.43
-0.64	-0.85	0.07	-1.10	0.14	-0.32	0.01
-0.85	-0.77	-0.14	0.56	1.20	0.13	0.44
0.45	0.98	-0.02	0.21	0.27	-0.76	0.68
-0.56	-0.80	-0.53	-0.40	1.60	0.19	2.47
-1.27	0.03	0.88	-0.55	0.73	0.00	-0.04
-0.49	-0.75	-0.49	-1.30	0.91	2.41	-0.29
-0.99	0.13	-0.47	0.69	-0.73	-0.56	-0.16
2.08	1.01	-0.34	0.26	1.83	-0.83	1.08
1.72	1.26	-0.34	0.43	0.06	-0.70	1.28
0.64	0.74	-0.55	1.54	-1.05	-0.76	1.08
1.86	0.24	2.64	0.01	-0.25	-0.55	-1.52
0.79	2.14	0.82	-0.52	0.23	-0.69	-0.35
-0.24	-0.86	0.06	-1.57	0.38	1.77	-0.33
-0.53	-1.28	-0.73	-1.88	-0.88	4.11	-0.78
-1.63	1.91	-0.02	-0.87	1.54	0.22	-0.78
-1.96	0.47	0.40	-0.30	-1.02	0.63	-0.82
-1.42	-0.50	0.80	2.91	-1.49	-0.21	-1.07
-1.43	1.46	1.55	-0.94	0.10	5.41	-0.91
0.82	-1.02	0.91	-0.38	1.95	2.62	-1.40
-0.47	-1.10	-0.55	0.67	0.32	-0.69	-0.44
-0.80	-0.75	-0.20	1.05	0.71	-0.30	-0.04
-0.13	0.43	-0.61	-0.18	-0.27	-0.75	0.23
-0.21	-1.20	-0.85	0.89	-1.64	-0.87	-1.15
-1.57	0.07	-0.66	-1.33	-0.36	0.17	-0.06
-1.47	-0.83	-0.72	-1.54	1.08	1.53	-0.32

-0.21	1.32	-0.26	-0.88	1.10	-0.48	0.57
-1.15	3.26	-0.64	8.00	1.35	-0.67	-0.12
-0.81	3.64	-0.61	35.18	4.10	-0.52	1.11
-0.61	0.34	-0.28	1.26	-0.14	-0.80	0.44
-1.62	-0.84	-0.14	-0.62	1.29	0.01	-0.15
-1.22	1.95	0.28	2.34	2.78	-0.61	0.95
-1.54	2.12	-0.78	8.03	-0.33	-0.83	-0.67
-0.61	-0.07	0.36	-0.96	0.87	-0.31	0.34
0.24	0.49	-0.28	0.88	0.78	-0.71	0.82
-1.08	-0.59	-0.29	0.41	0.55	-0.46	0.46
-0.67	0.32	-0.37	-0.35	0.17	-0.71	1.06
-1.13	-0.04	-0.31	-0.37	1.61	-0.34	1.25
-0.18	0.43	0.20	1.29	-0.19	-0.82	0.50
0.68	-0.62	-0.06	0.08	-0.94	-0.82	0.00
0.07	-0.38	0.14	0.25	1.43	-0.65	0.88
-0.03	-0.36	-0.21	1.79	0.22	-0.76	-0.24
-0.02	0.29	0.14	-0.46	-0.84	-0.80	0.18
-0.72	-0.43	-0.27	-0.04	-0.08	-0.67	0.30
-0.21	-1.05	-0.61	-1.92	-0.20	3.52	-0.75
0.87	3.02	-0.03	0.25	0.52	-0.79	-0.12
0.79	1.40	0.48	0.39	2.99	-0.09	-0.44
0.83	1.84	0.42	0.17	1.83	-0.05	-0.94
-0.46	-1.26	-0.54	-1.83	-0.63	5.44	-1.01
0.84	-0.24	-0.22	-0.46	-0.15	-0.78	-0.88
1.27	0.05	-0.32	-0.53	0.43	-0.59	-0.37
0.68	-0.63	-0.10	-1.78	-0.83	-0.16	-1.07
0.49	0.13	-0.81	-1.71	2.28	0.07	-0.50
-1.92	-1.24	-0.78	-1.60	-0.05	0.08	-0.74
-1.94	2.62	2.04	-0.19	0.18	1.46	-1.52
5.63	1.64	-0.54	0.41	0.67	-0.20	-0.06
1.24	1.60	-0.36	0.18	4.25	-0.32	0.54
-0.40	-0.22	-0.73	-0.83	0.84	-0.67	0.20
-1.70	-0.22	-0.94	-0.10	5.37	2.05	-0.05
0.08	-1.00	-0.65	-1.10	-0.31	0.27	-0.45
0.30	-0.43	-0.66	-1.35	1.75	0.66	0.29
-0.98	5.57	-0.81	38.57	2.19	-0.84	1.36
-0.34	-0.71	-0.57	-0.28	1.31	-0.03	-0.56
-1.37	1.08	1.00	-0.78	0.48	0.82	-0.54
-0.47	0.60	1.12	0.14	2.42	0.97	0.57
-0.49	-0.85	0.08	-1.47	-0.37	0.38	-0.39
-0.60	-0.80	0.58	-1.28	0.04	-0.17	-0.42
-0.33	-0.26	0.86	-0.02	-0.97	-0.69	-0.71
-0.17	-0.24	0.14	-0.68	0.44	-0.39	0.09
-0.88	1.07	-0.39	2.12	1.76	-0.74	0.94
-1.09	-0.29	-0.58	-1.55	1.10	3.65	-0.22
-0.54	-1.37	-0.68	-2.01	-0.68	6.22	-0.87
-1.18	0.08	-0.19	-0.19	1.31	-0.61	0.89
-0.31	-0.03	-0.07	-1.15	1.18	-0.63	0.82
-0.66	-0.46	-0.30	-0.90	0.60	-0.67	-0.16
-0.97	-0.05	0.03	-1.40	0.40	0.24	0.14
-0.22	0.30	0.18	-0.16	0.73	-0.64	0.16
1.14	-0.15	-0.16	-1.97	-1.23	5.00	-1.22
-1.55	0.11	-0.17	0.08	-0.18	-0.68	-0.22
2.16	0.78	-0.31	0.52	0.69	-0.77	0.89
-0.42	-0.64	1.06	-1.03	-0.77	-0.18	-1.15
-1.26	0.60	-0.22	-1.21	0.24	-0.15	-0.92

0.34	0.35	0.35	0.37	0.39	0.40	0.40
0.26	0.19	0.46	0.22	0.38	0.40	0.85

4686947	1336672	1032612	1495548	331698	1653485	7479891

85	86	87	88	89	90	91
16	17	18	19	20	21	22
E_19	E_21	E_22	E_23	E_3	E_46	E_48
CHR_P	CHR_P	CHR_P	CHR_P	CHR_P	CHR_P	CHR_P
11896438	2298032	4000500	2560098	527532	2489824	12852326
0.88	0.37	0.10	0.84	0.42	0.19	0.96
E_19	E_21	E_22	E_23	E_3	E_46	E_48
7209491	961360	2967888	1064550	195834	836339	5372435
1091684	255189	377862	379984	124977	258293	997282
502157	213241	199130	172634	19968	62806	209441
164902	42264	34304	133004	68294	83643	138640
363634	74226	71113	112232	11484	53131	352710
168227	60585	62714	91720	29872	116972	246250
384358	159415	29437	72873	3409	169046	1441458
241542	120763	51665	110884	11308	108609	350839
170486	19447	3984	11486	855	206272	1126803
160818	59440	15638	37107	4040	72117	268023
146719	27738	16747	34871	1335	30042	179239
83057	11529	5049	21295	2197	21733	96561
78420	23046	18190	18821	6539	29913	90006
67728	14380	3469	31235	1127	21234	151565
47756	9645	16109	15141	2620	11470	56920
49746	11428	1692	13245	723	32632	240590
15960	7283	7002	9764	7497	11053	21272
68639	12706	1450	14569	432	21244	140819
68787	9163	4133	9311	1693	17088	56091
42508	12757	4006	9113	1012	15857	60966
22498	10337	5460	11379	1634	10414	23674
37407	8453	3170	8471	1321	11161	35615
37223	4087	3436	5650	671	5481	27571
26441	6142	2757	6541	768	9153	41399
43123	13258	2801	8605	361	15787	30680
41338	8570	1361	7492	350	19954	59425
35378	5537	4177	5771	1090	7670	26419
13570	2631	4416	4918	1441	4771	15113
12615	4758	2076	2971	210	8490	58871
28349	8096	4620	8607	1001	6149	36799
21739	5854	4227	7817	878	2975	12463
24465	8071	2430	5991	2016	9244	18471
13614	3701	3365	5191	787	3153	20715
22482	4396	1462	3296	542	16316	88289
9431	1539	315	1303	187	21344	86977
26556	7663	4366	8052	962	5712	33597
17481	3592	5270	4749	375	2813	12579
20296	4581	2089	2878	410	4251	16360
19821	8938	1413	7464	263	2794	14919
9234	1750	4360	5035	303	2132	5546
2083	1343	1343	1802	1161	1533	7478
6288	2100	2021	2809	461	2590	3516
8362	1914	1295	2664	966	3483	6831
15094	3308	3782	3872	735	2936	7229
7445	1250	307	1006	77	8899	63328
12184	3381	2185	4911	922	7026	7620

8964	2412	1302	3118	517	2193	5820
10643	3997	1868	2942	373	2509	8264
2285	887	1727	1377	1089	2514	4060
3209	249	142	737	55	7014	81989
3186	1092	221	537	337	9277	20102
6693	940	226	3241	320	843	1234
9428	2710	646	2436	221	3106	4450
5262	1015	1432	1345	139	1216	2965
3803	1802	434	1409	98	1507	5283
10682	2089	583	406	260	2310	14583
1912	105	56	159	42	8704	52418
6295	2142	1567	1409	163	1180	5085
7356	2196	1203	1553	392	2756	5368
4687	1156	368	1965	310	1606	2898
1394	1149	1634	1572	472	712	866
4365	807	446	987	131	1164	1891
4609	1215	630	910	301	2118	4266
2675	394	128	238	12	2919	19160
1293	226	118	235	27	6738	41406
832	225	565	622	220	332	2546
6500	838	323	732	83	1197	8508
4792	595	134	572	206	3920	12660
5083	392	158	326	41	1577	17584
5139	1069	333	801	103	1631	4640
4768	1358	1688	855	25	450	2545
1148	508	530	523	528	610	930
1909	353	577	485	231	456	1742
3695	594	77	635	25	1907	11559
3930	566	218	505	63	929	3626
4700	1642	406	1573	107	1008	3297
1158	185	57	566	131	563	1374
3160	1065	650	596	139	735	2498
1282	676	901	513	93	1403	1156
936	263	315	385	279	374	789
3055	613	391	500	116	987	660
3718	680	546	745	76	665	3532
1928	367	453	524	241	428	864
1251	391	610	539	141	204	1466
1104	241	54	164	162	2150	5159
6239	1194	447	866	40	965	3665
3066	404	328	350	90	715	827
818	275	419	448	245	261	684
1568	529	74	245	27	1080	5400
1279	438	117	537	77	618	1873
608	424	321	389	75	209	579
1204	375	173	445	83	460	784
460	25	16	54	6	1799	17637
1169	347	303	654	106	437	1639
2372	686	176	341	68	450	1658
1641	240	69	207	20	892	6124
649	453	68	278	85	668	1795
364	206	198	274	104	151	343
518	141	188	202	121	229	661
534	162	201	292	122	296	358
916	152	23	88	11	540	4941
2445	174	178	181	7	259	414
931	611	323	496	72	318	1011
394	130	87	294	127	309	423
1789	440	152	296	19	395	1224

876	229	97	144	30	436	2474
1251	126	35	98	9	733	3901
726	176	126	218	88	274	292
1460	525	192	478	47	341	677
1264	154	58	124	34	596	3702
1129	126	75	120	20	559	2947
1499	285	70	249	28	595	1928
601	168	131	213	59	251	447
425	102	50	110	49	203	140
1745	130	107	133	14	235	2359
620	114	124	160	60	427	1010
579	108	85	134	17	188	376
625	250	230	96	41	200	653
666	197	26	185	25	341	849
666	197	26	185	25	341	849
853	134	88	111	14	309	178
569	99	137	140	53	191	326
387	62	72	129	65	114	236
936	99	10	188	25	313	1198
441	169	68	116	33	316	625
137	21	5	28	6	913	3914
171	93	84	115	52	117	245
605	140	171	228	23	133	224
310	87	50	143	49	121	278
585	158	248	147	18	61	161
385	71	148	84	7	31	176
309	86	91	70	11	466	1273
245	92	31	127	46	174	294
757	261	84	127	18	127	303
522	124	139	128	15	118	205
885	101	30	85	14	171	1564
481	126	60	116	23	129	216
627	96	70	142	7	143	860
653	58	68	54	22	195	642
635	128	59	131	12	140	1010
318	99	57	112	20	106	241
353	141	100	94	28	135	395
370	103	102	119	27	128	297
81	31	21	39	46	66	181
318	130	66	99	24	122	402
130	8	5	21	1	319	2105
221	26	60	59	25	37	341
115	50	49	74	42	87	86
341	216	38	133	29	161	404
97	60	46	55	24	37	168
422	68	60	91	18	91	192
241	95	29	81	3	309	530
456	38	28	42	11	135	864
324	39	2	59	13	379	759
127	66	68	105	31	55	159
97	14	13	42	38	269	245
255	35	84	66	16	26	124
594	82	85	70	2	36	199
239	35	52	29	5	85	40
273	40	20	67	6	84	151
151	36	42	80	8	54	271
71	10	4	8	0	373	1934
209	61	39	62	16	79	74
193	39	68	77	18	47	161

209	41	8	46	3	139	640
419	108	31	94	5	108	284
83	34	50	53	35	46	116
415	59	69	93	11	53	147
31	10	12	10	25	266	33
178	47	12	37	5	307	1273
275	47	40	44	6	34	235
159	85	68	34	12	60	122
139	30	22	27	13	72	302
59	48	43	65	19	31	125
143	91	33	49	8	89	222
140	77	5	46	7	180	460
151	46	23	72	8	50	120
224	10	9	74	12	55	95
309	94	21	57	11	108	418
611	40	77	45	4	114	151
111	15	7	57	22	65	91
179	25	0	60	19	49	190
221	59	12	50	4	135	573
118	10	0	75	7	43	23
165	63	10	39	6	120	466
205	27	36	42	15	27	22
83	24	30	48	22	44	91
208	53	12	50	4	130	543
156	18	3	53	9	46	51
153	34	13	34	33	88	76
231	63	28	38	5	88	165
20	0	0	12	103	16	11
207	43	3	18	1	133	297
322	49	15	45	4	67	89
29	15	29	40	39	21	34
124	17	23	26	4	16	109
131	39	38	39	9	137	121
107	11	7	10	3	382	1148
124	25	25	20	8	45	237
268	30	14	33	4	60	64
39	21	17	40	2	38	235
114	66	70	64	4	19	109
63	93	17	24	9	31	57
93	22	31	51	2	17	108
144	17	25	53	6	13	61
314	16	11	22	2	114	551
94	19	0	57	10	31	46
83	31	43	34	12	26	170
116	37	16	36	9	35	70
136	17	23	50	19	7	17
88	8	26	28	9	40	190
91	37	41	40	5	26	73
91	66	34	19	10	34	48
173	26	0	50	3	185	489
102	17	25	17	5	16	64
95	15	4	11	21	55	356
49	47	20	29	11	29	56
149	61	27	44	4	26	140
100	33	12	38	2	62	120
45	3	0	2	2	64	1485
282	27	16	36	2	22	110
28	45	18	11	4	83	84
90	8	21	29	8	17	52

168	46	30	27	10	57	178
96	7	19	10	4	38	41
45	11	0	37	5	33	34
49	7	15	33	7	35	40
46	9	9	25	10	17	60
39	6	22	11	19	27	67
70	26	25	21	3	16	37
191	26	29	29	4	24	76
121	20	7	15	2	47	166
90	13	31	24	10	15	67
66	11	3	11	0	53	598
46	17	10	18	7	14	29
121	23	9	16	8	23	179
78	16	28	28	2	20	54
126	6	21	27	7	68	47
89	10	4	28	1	73	163
64	35	6	16	4	30	84
44	17	10	21	6	29	66
77	7	1	22	2	25	59
237	33	16	17	3	31	112
112	29	4	16	0	48	259
173	57	26	17	4	26	75
173	57	26	17	4	26	75
47	8	0	24	6	11	14
33	9	11	11	2	6	62
94	15	16	20	5	42	117
68	13	2	41	6	42	52
82	29	11	48	6	40	89
47	14	12	23	7	12	32
125	10	3	20	4	25	90
52	15	19	15	2	1	78
54	19	10	27	2	26	54
30	34	4	11	3	20	51
18	8	17	3	0	10	50
74	12	4	6	7	70	54
135	21	22	24	3	18	55
20	0	0	2	0	68	499
102	15	9	21	6	12	47
85	31	2	35	1	12	61
31	7	15	20	6	11	44
29	13	7	20	5	10	40
81	30	2	34	1	11	55
23	11	9	10	5	6	13
73	26	28	11	1	8	46
82	8	5	9	5	23	105
51	14	9	15	4	13	44
5	0	2	0	0	100	584
17	5	10	20	3	12	22
44	8	10	11	5	6	26
34	3	6	18	4	16	57
20	2	3	20	5	15	17
33	9	11	12	1	16	79
18	0	0	2	0	53	562
151	14	14	21	2	11	28
52	11	0	16	0	18	51
0	0	0	0	0	0	0
34	7	4	11	2	6	34
21	11	9	18	8	10	14
65	11	9	12	0	6	21

34	2	1	11	1	6	21
34	14	3	18	3	21	31
22	6	2	15	4	14	15
36	8	12	10	1	11	31
27	13	22	15	4	10	12
15	20	6	13	5	10	11
44	11	0	16	0	14	39
28	5	1	16	5	16	11
40	7	1	3	0	31	199
21	11	1	10	2	39	199
75	19	9	14	0	37	29
49	2	10	13	4	11	11
40	14	6	11	0	28	53
7	1	4	5	10	21	12
22	3	3	11	9	14	20
22	8	1	6	0	32	97
16	8	9	8	0	3	28
49	5	3	7	2	26	67
38	6	11	6	1	20	17
19	4	0	8	6	32	22
14	3	2	13	0	8	13
36	9	11	26	0	9	34
20	6	3	19	7	7	20
34	4	9	2	0	6	28
29	8	2	16	1	17	52
13	7	8	6	1	17	34
7	5	4	8	7	5	11
25	5	6	13	1	7	6
21	8	12	8	0	5	9
36	15	5	13	4	6	22
25	4	4	6	3	11	28
26	3	2	3	0	54	167
66	21	1	9	0	26	128
24	2	4	2	0	9	65
32	9	6	15	0	5	41
21	5	6	3	8	6	9
54	7	4	10	0	7	20
22	4	7	7	10	12	21
27	10	3	17	3	22	33
10	6	6	9	1	9	13
41	10	0	7	0	19	88
24	10	4	5	2	8	22
36	4	1	9	3	7	7
16	4	7	5	4	8	3
31	4	0	17	0	10	12
20	0	6	2	8	42	1
13	5	14	11	0	10	25
35	7	3	6	0	12	52
18	2	9	6	0	8	12
14	5	5	9	2	2	13
27	5	12	5	2	5	8
30	0	0	1	2	44	143
13	5	1	3	1	12	8
43	13	1	6	0	18	97
24	6	8	6	2	3	33
37	6	1	7	2	5	24
1	2	3	10	1	4	9
11	0	0	1	0	41	67
20	1	2	4	0	5	35

18	8	9	12	1	7	11
4	3	4	12	4	7	6
29	4	3	8	1	6	45
16	6	11	6	6	3	10
5	6	6	9	3	8	8
9	1	5	10	1	7	12
27	4	2	2	2	1	11
13	2	1	1	0	34	113
33	9	0	9	0	3	34
23	8	3	8	0	10	9
16	4	5	3	2	2	4
18	8	3	8	1	9	34
15	3	4	11	0	1	12
14	0	2	1	1	66	65
26	15	12	0	2	6	8
42	4	1	2	0	10	22
20	3	2	5	0	15	15
14	3	3	7	0	5	13
29	3	2	6	0	19	55
36	2	5	4	1	4	10
29	4	1	5	1	12	34
8	7	3	3	1	4	15
27	6	3	9	0	9	13
7	4	3	7	1	1	11
26	3	4	7	0	2	17
24	1	3	5	2	4	8
15	0	0	3	1	19	51
10	3	0	5	1	16	59
7	2	2	5	1	1	7
30	9	2	5	2	5	22
7	1	1	10	0	4	4
9	3	0	4	1	4	11
20	2	0	13	0	13	30
4	1	1	5	0	5	9
3	3	1	2	0	30	94
8	0	1	6	2	2	3
22	0	3	3	2	7	21
5	0	5	4	1	6	10
9	0	1	5	1	5	2
3	4	2	8	1	3	5
1	4	0	2	1	4	6
14	1	1	10	3	6	17
14	3	1	6	2	3	2
22	3	2	0	0	3	6
9	0	0	2	1	5	6
20	1	2	0	0	2	6
21	4	3	6	0	5	8
4	2	0	5	0	2	5
8	4	0	2	4	10	17
68	0	3	2	0	0	41
7	2	4	2	1	2	7
14	1	0	6	0	4	10
14	4	0	4	0	10	49
13	2	4	1	0	4	2
12	5	2	3	1	10	5
6	1	0	1	0	9	30
19	4	1	1	1	12	20
5	4	2	2	0	3	1
6	6	5	2	2	5	4

15	0	0	1	1	0	36
11	5	3	5	0	12	5
17	10	2	4	1	7	19
18	4	0	1	0	14	15
7	5	3	4	0	3	3
16	2	2	2	0	1	4
6	1	0	1	0	2	7
4	2	4	6	0	7	10
11	3	7	6	1	1	10
5	0	1	4	0	5	10
5	10	0	3	0	8	30
1	0	0	0	0	19	78
0	0	0	1	0	27	125
19	2	0	3	1	16	42
6	1	2	4	1	5	2
2	4	1	4	1	2	3
5	0	2	4	2	1	6
8	1	0	1	3	1	3
1	2	1	0	0	23	64
14	0	0	7	0	16	22
12	0	0	5	0	5	9
10	1	2	4	0	10	30
1	3	1	4	1	3	2
7	2	1	3	1	2	8
6	0	0	2	0	6	12
5	0	2	0	0	27	64
9	3	1	2	3	6	13
4	2	1	1	0	4	7
9	1	4	3	0	2	16
8	9	2	2	1	6	8
0	1	0	2	1	23	75
3	2	2	3	0	1	8
7	0	2	1	0	2	7
0	4	2	0	1	2	5
3	1	2	5	0	4	10
4	2	0	2	0	2	2
4	1	1	7	3	2	4
7	2	0	1	1	2	15
7	2	3	3	1	6	4
9	1	0	4	2	1	5
7	0	1	2	0	42	50
16	2	0	1	0	1	10
4	0	0	3	0	0	1
11	0	1	1	0	4	16
4	0	1	1	0	0	8
9	1	0	4	0	7	40
4	5	0	3	0	9	14
7	2	9	2	1	1	6
1	3	1	3	0	2	7
8	5	4	7	0	2	3
8	1	0	2	0	1	3
3	4	0	6	1	5	18
3	2	5	0	0	1	10
8	0	0	4	1	3	8
11	4	2	2	1	2	8
11	3	1	2	0	1	8
13	2	2	5	0	5	5
5	1	2	2	0	0	1
6	2	1	0	0	0	2

5	1	0	1	0	3	3
6	2	1	3	0	2	5
5	5	2	2	0	3	0
7	2	2	1	0	1	5
5	3	2	2	0	0	10
2	0	0	0	0	9	25
5	2	0	2	0	4	9
10	3	0	1	0	7	32
5	1	3	1	0	1	4
9	0	0	2	0	8	16
2	4	0	0	2	0	15
7	0	0	0	1	2	11
1	1	0	1	0	2	23
12	0	1	1	0	5	20
4	0	0	0	0	2	11
9	5	1	2	0	3	13
3	1	2	1	0	0	3
3	1	0	1	0	9	15
4	0	0	0	0	10	43
13	1	0	6	0	2	6
13	3	2	2	0	5	8
1	1	0	0	0	6	54
6	1	0	1	0	6	24
0	1	2	1	2	1	0
0	0	2	2	0	2	1
3	1	3	0	0	3	2
2	0	0	1	1	0	0
3	0	3	3	0	4	5
1	0	0	0	0	27	38
13	0	2	0	0	2	17
11	1	0	4	0	2	6
3	0	2	0	0	2	3
5	3	0	3	0	1	11
4	1	1	0	0	2	1
11	0	2	1	0	4	1
8	1	0	3	0	0	0
3	1	1	1	0	2	3
9	0	0	2	1	4	18
7	4	5	1	0	3	3
5	0	2	2	0	0	1
7	0	0	0	0	3	17
3	0	0	3	0	1	0
1	0	0	3	1	3	8
2	1	3	2	0	1	2
3	0	2	1	0	1	3
2	0	0	1	0	2	7
2	4	0	0	0	1	1
3	0	1	0	0	1	10
8	1	1	0	0	2	6
3	0	3	1	0	1	0
3	0	0	1	0	2	0
0	0	0	0	0	8	20
8	1	0	0	0	6	23
2	5	1	4	0	1	3
3	1	0	1	0	2	4
4	1	1	1	2	1	5
2	1	0	1	1	0	2
4	1	2	1	0	1	2
1	0	0	0	0	2	9

3	1	1	3	0	0	5
4	0	1	0	0	4	2
5	3	0	1	0	5	9
15	5	1	1	0	1	2
8	1	1	1	0	5	6
0	0	0	0	0	0	2
1	0	1	1	1	1	2
2	0	2	0	0	0	1
2	1	0	1	0	1	5
1	1	0	0	1	0	0
4	1	0	0	1	1	4
4	0	0	0	0	0	2
0	0	0	0	0	0	0
E_19	E_21	E_22	E_23	E_3	E_46	E_48
CHR_P	CHR_P	CHR_P	CHR_P	CHR_P	CHR_P	CHR_P
7209491	961360	2967888	1064550	195834	836339	5372435
20	14	23	14	12	11	14
-0.38	-1.62	0.43	-1.65	-1.96	-2.10	-1.56

0.05	0.73	0.98	0.95	0.87	0.42	0.25
26	8	1	2	4	14	18
1	1	1	1	1	1	1
85	86	87	88	89	90	91
20	21	22	23	24	25	26
E_48	E_7	E_8	E_9	F_40	F_41	F_42
CHR_P	CHR_P	CHR_P	CHR_P	CHR_P	CHR_P	CHR_P
7479891	1160935	2099567	1323600	3571995	3076499	1136044
E_48	E_7	E_8	E_9	F_40	F_41	F_42
997282	430892	567895	487095	1116571	1013554	241383
209441	93617	196075	92860	372466	369204	95275
138640	148736	270305	150760	144769	367770	14583
352710	30353	113550	79014	173851	170780	142685
246250	82596	155876	85452	187609	167992	65057
1441458	38241	80210	45678	265139	98721	59034
350839	69449	163721	80719	154960	157746	44478
1126803	15818	9548	3869	67671	13031	23608
268023	24670	55580	23927	105923	72153	21383
179239	17073	30178	26587	104997	54674	42363
96561	6552	22873	20929	47605	26249	28124
90006	20378	39596	18875	47949	43533	16743
151565	4948	31680	16258	45264	32383	56843
56920	9117	18724	14550	23025	27635	12557
240590	2835	13374	4201	59787	11354	52887
21272	10259	19506	12243	13077	24806	7630
140819	3697	21576	5753	49007	14030	13898
56091	7747	16607	7422	42871	22011	11271
60966	8106	13417	6278	35265	18210	6886

23674	7861	13955	6599	35791	18606	4800
35615	5428	11601	4615	30985	15512	7157
27571	3104	8290	5474	15659	11635	6148
41399	2483	9850	5079	41393	10738	7328
30680	5398	11011	3173	22523	23870	7963
59425	1975	7215	2028	30065	8975	23658
26419	6156	14455	7456	21998	15343	2634
15113	5195	7325	5344	8119	10887	6897
58871	602	2808	3748	16981	4214	16425
36799	5374	8404	7902	10130	15437	4288
12463	3316	8131	4344	13042	12622	3104
18471	6800	10459	4813	20057	13608	1778
20715	2173	6876	3633	7891	10504	6304
88289	2238	5921	2503	8198	5449	4144
86977	400	1536	507	13168	1871	3973
33597	5072	7830	7291	9354	14606	3939
12579	2375	4735	5382	6126	9559	1804
16360	4499	3898	2305	11683	5682	2563
14919	1807	4669	2725	9688	5919	3708
5546	1572	4467	2886	9818	4133	1843
7478	3962	3780	1108	6009	7634	2383
3516	3040	4636	2942	5063	7426	563
6831	2074	4725	2355	7359	5694	2415
7229	3507	4286	3015	3652	8135	945
63328	462	795	435	5207	1104	8344
7620	3969	6665	2790	3222	6505	1015
5820	2660	4605	2146	6160	5870	861
8264	1795	3923	1942	5275	5637	1408
4060	1890	2452	1926	4581	3481	1159
81989	101	608	528	411	357	1106
20102	1531	1660	560	10454	1263	3912
1234	577	2301	353	1459	1227	726
4450	1324	2938	1110	4484	4212	869
2965	997	1626	1168	2419	3051	573
5283	1007	2207	865	8034	3130	2145
14583	910	2869	901	4326	1250	894
52418	90	206	122	813	188	596
5085	1265	1894	1268	2534	3161	456
5368	1543	3472	985	3668	2730	1781
2898	641	2239	1399	3604	3307	980
866	2086	1142	1136	695	4455	592
1891	892	1525	788	1996	2164	364
4266	1013	1840	524	3786	1964	1108
19160	99	206	119	777	301	231
41406	87	159	86	157	188	82
2546	820	711	1147	1402	1428	847
8508	410	948	453	2657	1395	620
12660	445	1045	449	2256	962	279
17584	183	487	218	1561	626	1137
4640	623	1575	572	2504	2208	480
2545	125	676	1280	2557	1669	449
930	780	1069	561	1379	1561	138
1742	576	636	801	1705	1845	568
11559	100	330	140	1884	326	1700
3626	376	782	409	3583	1678	754
3297	760	2173	786	2036	2754	430
1374	177	654	101	642	530	748
2498	601	1074	495	1963	1290	471
1156	750	499	320	672	2716	279

789	441	690	278	1062	1337	379
660	494	727	366	980	1232	90
3532	470	797	751	900	1439	377
864	601	820	391	1207	1255	185
1466	481	584	586	1104	1268	265
5159	37	445	91	692	243	161
3665	418	946	336	962	1254	472
827	608	604	349	641	988	39
684	436	482	357	679	1179	115
5400	270	480	154	882	601	373
1873	430	823	406	1734	1317	320
579	311	493	223	491	1415	640
784	400	556	0	1298	985	445
17637	14	42	35	23	50	8
1639	369	883	293	784	656	198
1658	319	492	301	769	719	59
6124	144	209	122	729	266	118
1795	220	539	120	964	600	216
343	284	379	220	924	686	159
661	255	377	326	639	474	235
358	274	467	223	784	816	235
4941	51	94	43	997	155	367
414	261	303	99	547	740	11
1011	283	631	254	1150	929	107
423	191	358	245	392	625	151
1224	124	481	76	384	375	640
2474	144	207	166	798	328	71
3901	82	134	63	821	151	316
292	318	354	184	377	500	27
677	212	458	253	663	825	127
3702	102	225	132	358	334	195
2947	161	197	99	501	265	339
1928	210	442	97	1018	383	246
447	221	443	173	610	697	121
140	324	260	113	422	546	23
2359	68	146	94	485	245	182
1010	134	410	250	439	587	50
376	78	235	178	322	315	200
653	180	220	114	270	504	418
849	109	251	82	386	268	113
849	109	251	82	386	268	113
178	126	148	98	245	281	35
326	120	235	184	186	266	29
236	103	264	140	405	465	42
1198	32	241	24	248	77	199
625	120	213	96	806	222	284
3914	21	31	19	70	51	14
245	81	151	115	238	369	89
224	151	261	162	132	317	66
278	167	294	109	354	309	20
161	134	171	77	190	393	47
176	34	159	115	319	298	107
1273	37	72	50	266	357	34
294	139	241	123	299	299	22
303	228	130	57	116	357	60
205	170	162	82	285	406	21
1564	57	195	52	224	118	39
216	94	187	69	306	220	103
860	47	184	38	211	193	263

642	84	134	109	218	127	61
1010	102	212	111	144	235	152
241	109	167	94	131	306	22
395	77	167	90	161	131	85
297	131	159	107	213	387	39
181	77	118	38	112	143	18
402	78	183	100	111	266	93
2105	4	15	6	37	48	38
341	49	88	123	112	120	30
86	50	141	87	210	225	55
404	80	165	70	351	262	33
168	63	68	89	130	241	40
192	108	118	67	109	182	11
530	36	109	15	362	150	117
864	29	64	42	151	133	58
759	26	89	7	141	27	107
159	101	123	68	186	248	60
245	69	121	25	49	93	27
124	38	57	76	78	174	18
199	35	77	15	171	139	62
40	80	68	32	44	189	4
151	87	100	52	120	163	22
271	55	77	29	123	106	35
1934	4	10	3	38	13	13
74	33	90	45	143	181	16
161	53	91	45	110	150	19
640	30	84	24	278	102	42
284	48	113	35	290	207	35
116	48	77	73	78	100	27
147	62	128	59	149	192	21
33	37	50	13	37	52	8
1273	28	60	16	89	45	24
235	52	52	78	77	74	60
122	36	119	39	79	248	44
302	13	63	42	122	51	64
125	44	104	54	164	122	26
222	60	120	31	164	151	16
460	48	85	30	319	103	47
120	56	98	43	76	78	8
95	12	49	18	14	25	22
418	38	84	20	259	106	49
151	70	81	24	127	90	10
91	21	50	16	29	40	10
190	5	67	0	79	174	21
573	18	43	14	203	50	111
23	10	45	13	16	16	19
466	28	40	14	235	66	37
22	22	39	21	57	85	9
91	32	59	35	70	88	29
543	17	42	14	192	48	104
51	12	58	6	38	18	37
76	94	33	50	49	96	20
165	22	49	29	154	142	31
11	48	58	75	2	104	0
297	21	9	1	116	16	14
89	47	69	25	139	93	29
34	39	53	52	87	69	49
109	27	34	33	96	109	11
121	108	108	79	48	130	20

1148	34	54	5	142	20	80
237	20	17	24	111	33	25
64	34	46	25	63	53	4
235	10	53	51	147	94	24
109	53	88	19	70	114	26
57	28	12	23	61	87	4
108	29	45	21	100	73	18
61	18	43	39	118	73	25
551	12	28	10	81	30	15
46	14	80	8	23	35	19
170	33	42	35	83	107	19
70	32	75	22	72	80	3
17	76	18	14	36	78	8
190	29	43	33	32	51	30
73	42	68	42	55	124	20
48	27	71	28	107	114	19
489	4	40	5	84	17	81
64	40	26	37	36	37	3
356	24	46	27	123	38	6
56	34	46	24	123	88	12
140	38	45	30	100	97	20
120	32	56	19	97	57	27
1485	0	1	3	13	4	9
110	22	52	13	72	72	23
84	46	58	1	65	19	4
52	22	38	36	30	47	3
178	26	61	0	50	71	11
41	11	30	12	52	29	3
34	9	56	3	25	10	10
40	29	29	24	32	35	16
60	15	52	24	45	64	40
67	10	57	26	18	37	5
37	22	25	11	21	61	8
76	16	34	22	37	62	14
166	11	13	9	81	27	9
67	20	25	26	59	33	8
598	2	2	2	21	8	9
29	20	17	16	30	70	3
179	24	41	23	43	39	5
54	13	34	25	24	90	3
47	31	26	33	34	77	4
163	8	42	5	70	54	15
84	21	24	6	55	51	23
66	21	31	22	61	82	3
59	1	23	4	12	12	10
112	14	42	11	55	40	3
259	3	8	2	117	24	35
75	23	31	17	64	57	11
75	23	31	17	64	57	11
14	2	16	4	8	13	6
62	13	32	28	29	39	3
117	21	16	15	32	62	6
52	7	44	4	14	20	13
89	18	42	20	57	52	5
32	13	36	8	48	57	6
90	3	19	19	51	18	17
78	10	19	33	19	46	11
54	11	32	13	30	38	12
51	14	34	11	100	37	19

50	6	8	10	19	24	9
54	37	25	11	10	33	13
55	12	22	14	28	51	10
499	0	0	2	4	3	0
47	14	32	14	28	39	8
61	10	19	12	69	31	14
44	15	36	19	38	54	22
40	10	5	21	17	20	5
55	10	17	11	63	29	11
13	16	20	8	13	40	4
46	16	26	14	6	39	11
105	9	31	14	46	28	3
44	8	18	23	19	32	9
584	1	0	0	1	1	1
22	16	33	20	12	29	12
26	16	11	17	13	10	1
57	12	20	13	16	28	7
17	6	18	6	8	21	4
79	6	20	5	54	13	26
562	1	1	1	5	0	45
28	9	15	11	12	51	4
51	7	19	1	41	14	10
0	53	0	0	0	0	0
34	23	31	13	14	36	7
14	7	3	12	48	29	8
21	9	9	8	25	20	5
21	2	8	6	4	13	3
31	5	17	6	23	17	11
15	3	15	6	11	5	15
31	10	17	17	24	42	2
12	15	8	9	16	21	3
11	10	13	15	20	35	4
39	7	18	1	36	14	8
11	2	19	4	13	1	5
199	1	0	2	5	3	19
199	8	20	4	19	6	8
29	8	23	17	44	27	13
11	12	19	7	24	19	2
53	3	13	7	30	11	7
12	14	25	7	18	19	4
20	2	16	3	5	8	11
97	7	13	7	41	10	16
28	12	6	3	23	11	13
67	12	20	7	22	35	2
17	12	16	7	15	19	4
22	4	15	1	11	5	0
13	3	14	2	4	9	4
34	4	27	10	20	26	3
20	6	12	10	22	24	5
28	8	6	7	4	19	5
52	7	9	3	51	11	6
34	8	24	5	24	29	7
11	11	18	7	8	23	0
6	4	11	7	9	16	2
9	4	18	4	13	32	2
22	11	18	7	24	26	1
28	6	10	8	28	12	14
167	3	8	2	29	2	28
128	1	6	5	38	10	2

65	6	4	2	24	12	10
41	5	11	7	21	25	6
9	5	10	6	8	15	1
20	11	11	2	17	5	3
21	7	18	9	25	18	1
33	7	14	12	7	14	6
13	5	9	10	13	23	9
88	5	7	3	28	9	7
22	6	13	3	32	17	10
7	0	20	0	4	10	4
3	15	5	12	20	35	3
12	4	17	3	9	10	8
1	4	12	0	15	9	1
25	5	15	13	10	14	3
52	3	10	4	9	7	10
12	5	3	6	6	13	0
13	2	19	1	14	21	6
8	5	11	3	3	11	2
143	3	3	2	0	2	0
8	9	5	8	6	13	1
97	3	9	3	20	11	13
33	8	10	11	5	6	4
24	7	8	2	37	17	9
9	4	14	3	22	27	0
67	5	4	3	27	27	2
35	3	6	7	21	5	4
11	5	7	3	12	14	7
6	11	3	7	8	20	4
45	4	9	4	42	20	6
10	7	7	3	12	15	1
8	1	11	11	20	10	6
12	11	3	7	8	15	1
11	6	6	3	10	12	1
113	1	1	0	6	3	7
34	4	7	6	15	8	6
9	2	17	6	13	16	4
4	8	7	4	15	10	0
34	8	7	2	18	10	3
12	2	5	9	8	7	6
65	1	3	0	19	1	1
8	7	12	3	14	4	7
22	3	11	1	14	6	2
15	7	16	0	13	11	2
13	4	4	7	12	17	5
55	3	6	0	17	2	0
10	2	12	3	8	13	0
34	4	7	2	22	8	4
15	6	4	2	20	12	1
13	4	18	6	6	23	7
11	4	5	3	10	7	0
17	5	6	9	4	3	0
8	1	5	1	9	6	4
51	4	2	1	38	5	7
59	2	1	2	18	3	1
7	7	7	3	5	6	6
22	3	11	6	10	13	1
4	1	4	5	8	14	0
11	2	14	6	12	11	5
30	2	13	7	11	8	2

9	5	11	6	6	11	0
94	2	4	3	12	4	1
3	0	2	2	0	3	2
21	4	5	1	18	13	1
10	4	3	1	8	12	5
2	6	8	3	7	5	0
5	2	10	1	9	4	1
6	3	8	2	10	6	4
17	5	19	7	6	17	0
2	1	7	1	4	3	1
6	14	3	2	10	13	2
6	5	11	5	7	16	1
6	3	2	2	13	7	2
8	5	10	4	9	16	0
5	3	2	1	8	5	1
17	2	5	0	9	5	4
41	5	3	0	41	3	0
7	1	4	0	3	5	1
10	2	16	3	2	1	1
49	2	4	1	12	4	1
2	4	3	0	7	16	0
5	4	5	3	5	8	3
30	0	0	1	8	5	8
20	4	10	2	8	4	2
1	5	5	5	8	5	1
4	7	2	0	5	11	0
36	2	0	2	8	4	0
5	4	4	3	5	2	2
19	2	6	0	19	4	10
15	0	5	1	8	0	4
3	4	5	1	3	3	5
4	2	2	1	2	9	5
7	1	2	6	5	4	0
10	2	7	3	6	7	4
10	4	4	3	2	5	0
10	0	4	3	6	3	2
30	3	2	1	19	2	4
78	0	3	1	2	4	2
125	0	0	0	1	0	1
42	2	8	3	9	6	1
2	1	2	2	3	3	1
3	0	3	0	6	3	2
6	1	3	6	2	8	1
3	3	0	4	3	4	0
64	0	0	0	0	0	0
22	0	5	0	1	2	12
9	0	4	0	2	1	2
30	0	6	0	11	3	2
2	7	3	2	7	15	1
8	4	3	1	1	17	3
12	0	3	2	2	7	0
64	0	1	0	8	0	2
13	3	11	5	2	4	0
7	2	2	1	22	7	4
16	5	2	3	3	11	0
8	3	5	2	10	6	2
75	2	0	1	3	1	1
8	4	1	1	12	7	0
7	1	1	0	8	7	1

5	0	4	1	2	8	3
10	0	4	1	10	5	3
2	2	3	1	5	5	1
4	0	7	0	3	5	0
15	3	1	2	6	1	0
4	4	7	1	9	4	1
5	1	3	1	1	0	1
50	0	0	1	0	0	0
10	5	4	1	10	6	3
1	1	3	1	9	3	1
16	1	7	0	9	7	0
8	1	4	2	9	6	0
40	1	5	1	2	0	0
14	0	1	1	13	3	5
6	4	2	1	4	9	0
7	2	5	1	6	5	0
3	1	2	3	3	7	1
3	0	1	1	5	5	5
18	1	2	2	5	4	1
10	0	1	1	2	1	0
8	2	1	4	6	3	2
8	3	5	1	6	7	0
8	2	1	1	7	5	3
5	1	1	2	1	3	0
1	2	1	1	0	2	4
2	1	1	2	0	2	0
3	3	1	1	0	3	0
5	1	2	1	1	4	2
0	2	3	4	5	4	3
5	1	4	0	4	2	0
10	1	2	2	5	10	0
25	0	0	0	2	1	2
9	1	4	0	0	3	0
32	3	3	0	4	2	0
4	5	4	2	5	7	3
16	1	2	0	2	1	3
15	10	3	2	6	2	0
11	0	2	0	1	3	0
23	0	0	1	4	0	2
20	3	0	2	1	5	0
11	3	2	1	4	4	2
13	2	2	2	2	3	2
3	1	3	2	6	8	1
15	0	1	0	1	1	1
43	1	0	0	5	1	5
6	0	0	1	1	1	1
8	3	1	1	3	0	0
54	0	0	0	2	0	2
24	0	0	1	6	0	2
0	1	1	3	0	5	0
1	0	1	0	4	7	2
2	4	2	0	6	2	1
0	3	1	0	7	4	4
5	2	1	2	3	9	1
38	0	0	0	2	0	0
17	1	0	0	1	0	1
6	1	2	2	3	0	0
3	0	2	1	2	4	0
11	2	3	0	2	4	0

1	2	2	0	2	14	0
1	0	3	1	3	4	2
0	1	1	1	7	2	1
3	1	1	1	1	3	0
18	0	3	0	2	1	3
3	1	3	1	2	6	1
1	0	1	0	0	5	0
17	0	0	0	2	1	0
0	1	2	1	0	3	0
8	2	0	2	5	7	0
2	2	1	0	2	4	0
3	4	2	1	5	6	2
7	0	2	1	4	2	2
1	0	2	1	2	0	2
10	0	1	4	0	4	3
6	3	1	1	2	1	0
0	2	0	1	1	3	0
0	2	3	1	3	3	0
20	0	0	0	2	2	13
23	0	0	1	2	0	0
3	0	9	3	1	0	1
4	0	2	2	4	0	0
5	1	1	1	3	0	1
2	1	2	1	1	2	0
2	2	2	1	5	1	2
9	1	2	0	7	2	0
5	1	6	2	2	2	0
2	1	2	0	1	0	0
9	0	1	1	7	4	1
2	0	0	1	3	1	0
6	1	2	2	3	4	1
2	1	1	0	0	0	1
2	0	0	1	0	2	1
1	1	0	1	0	6	0
5	3	1	1	1	1	0
0	2	0	0	0	0	1
4	0	2	1	4	1	1
2	0	1	0	6	2	0

0.05	0.73	0.98	0.95	0.87	0.42	0.25
26	8	1	2	4	14	18
1	1	1	1	1	1	1
85	86	87	88	89	90	91
20	21	22	23	24	25	26
E_48	E_7	E_8	E_9	F_40	F_41	F_42
CHR_P	CHR_P	CHR_P	CHR_P	CHR_P	CHR_P	CHR_P
7479891	1160935	2099567	1323600	3571995	3076499	1136044
CHR_P	CHR_P	CHR_P	CHR_P	CHR_P	CHR_P	CHR_P
997282	430892	567895	487095	1116571	1013554	241383
209441	93617	196075	92860	372466	369204	95275
138640	148736	270305	150760	144769	367770	14583
352710	30353	113550	79014	173851	170780	142685
246250	82596	155876	85452	187609	167992	65057

1441458	38241	80210	45678	265139	98721	59034
350839	69449	163721	80719	154960	157746	44478
1126803	15818	9548	3869	67671	13031	23608
268023	24670	55580	23927	105923	72153	21383
179239	17073	30178	26587	104997	54674	42363
96561	6552	22873	20929	47605	26249	28124
90006	20378	39596	18875	47949	43533	16743
151565	4948	31680	16258	45264	32383	56843
56920	9117	18724	14550	23025	27635	12557
240590	2835	13374	4201	59787	11354	52887
21272	10259	19506	12243	13077	24806	7630
140819	3697	21576	5753	49007	14030	13898
56091	7747	16607	7422	42871	22011	11271
60966	8106	13417	6278	35265	18210	6886
23674	7861	13955	6599	35791	18606	4800
35615	5428	11601	4615	30985	15512	7157
27571	3104	8290	5474	15659	11635	6148
41399	2483	9850	5079	41393	10738	7328
30680	5398	11011	3173	22523	23870	7963
59425	1975	7215	2028	30065	8975	23658
26419	6156	14455	7456	21998	15343	2634
15113	5195	7325	5344	8119	10887	6897
58871	602	2808	3748	16981	4214	16425
36799	5374	8404	7902	10130	15437	4288
12463	3316	8131	4344	13042	12622	3104
18471	6800	10459	4813	20057	13608	1778
20715	2173	6876	3633	7891	10504	6304
88289	2238	5921	2503	8198	5449	4144
86977	400	1536	507	13168	1871	3973
33597	5072	7830	7291	9354	14606	3939
12579	2375	4735	5382	6126	9559	1804
16360	4499	3898	2305	11683	5682	2563
14919	1807	4669	2725	9688	5919	3708
5546	1572	4467	2886	9818	4133	1843
7478	3962	3780	1108	6009	7634	2383
3516	3040	4636	2942	5063	7426	563
6831	2074	4725	2355	7359	5694	2415
7229	3507	4286	3015	3652	8135	945
63328	462	795	435	5207	1104	8344
7620	3969	6665	2790	3222	6505	1015
5820	2660	4605	2146	6160	5870	861
8264	1795	3923	1942	5275	5637	1408
4060	1890	2452	1926	4581	3481	1159
81989	101	608	528	411	357	1106
20102	1531	1660	560	10454	1263	3912
1234	577	2301	353	1459	1227	726
4450	1324	2938	1110	4484	4212	869
2965	997	1626	1168	2419	3051	573
5283	1007	2207	865	8034	3130	2145
14583	910	2869	901	4326	1250	894
52418	90	206	122	813	188	596
5085	1265	1894	1268	2534	3161	456
5368	1543	3472	985	3668	2730	1781
2898	641	2239	1399	3604	3307	980
866	2086	1142	1136	695	4455	592
1891	892	1525	788	1996	2164	364
4266	1013	1840	524	3786	1964	1108
19160	99	206	119	777	301	231
41406	87	159	86	157	188	82

2546	820	711	1147	1402	1428	847
8508	410	948	453	2657	1395	620
12660	445	1045	449	2256	962	279
17584	183	487	218	1561	626	1137
4640	623	1575	572	2504	2208	480
2545	125	676	1280	2557	1669	449
930	780	1069	561	1379	1561	138
1742	576	636	801	1705	1845	568
11559	100	330	140	1884	326	1700
3626	376	782	409	3583	1678	754
3297	760	2173	786	2036	2754	430
1374	177	654	101	642	530	748
2498	601	1074	495	1963	1290	471
1156	750	499	320	672	2716	279
789	441	690	278	1062	1337	379
660	494	727	366	980	1232	90
3532	470	797	751	900	1439	377
864	601	820	391	1207	1255	185
1466	481	584	586	1104	1268	265
5159	37	445	91	692	243	161
3665	418	946	336	962	1254	472
827	608	604	349	641	988	39
684	436	482	357	679	1179	115
5400	270	480	154	882	601	373
1873	430	823	406	1734	1317	320
579	311	493	223	491	1415	640
784	400	556	0	1298	985	445
17637	14	42	35	23	50	8
1639	369	883	293	784	656	198
1658	319	492	301	769	719	59
6124	144	209	122	729	266	118
1795	220	539	120	964	600	216
343	284	379	220	924	686	159
661	255	377	326	639	474	235
358	274	467	223	784	816	235
4941	51	94	43	997	155	367
414	261	303	99	547	740	11
1011	283	631	254	1150	929	107
423	191	358	245	392	625	151
1224	124	481	76	384	375	640
2474	144	207	166	798	328	71
3901	82	134	63	821	151	316
292	318	354	184	377	500	27
677	212	458	253	663	825	127
3702	102	225	132	358	334	195
2947	161	197	99	501	265	339
1928	210	442	97	1018	383	246
447	221	443	173	610	697	121
140	324	260	113	422	546	23
2359	68	146	94	485	245	182
1010	134	410	250	439	587	50
376	78	235	178	322	315	200
653	180	220	114	270	504	418
849	109	251	82	386	268	113
849	109	251	82	386	268	113
178	126	148	98	245	281	35
326	120	235	184	186	266	29
236	103	264	140	405	465	42
1198	32	241	24	248	77	199

625	120	213	96	806	222	284
3914	21	31	19	70	51	14
245	81	151	115	238	369	89
224	151	261	162	132	317	66
278	167	294	109	354	309	20
161	134	171	77	190	393	47
176	34	159	115	319	298	107
1273	37	72	50	266	357	34
294	139	241	123	299	299	22
303	228	130	57	116	357	60
205	170	162	82	285	406	21
1564	57	195	52	224	118	39
216	94	187	69	306	220	103
860	47	184	38	211	193	263
642	84	134	109	218	127	61
1010	102	212	111	144	235	152
241	109	167	94	131	306	22
395	77	167	90	161	131	85
297	131	159	107	213	387	39
181	77	118	38	112	143	18
402	78	183	100	111	266	93
2105	4	15	6	37	48	38
341	49	88	123	112	120	30
86	50	141	87	210	225	55
404	80	165	70	351	262	33
168	63	68	89	130	241	40
192	108	118	67	109	182	11
530	36	109	15	362	150	117
864	29	64	42	151	133	58
759	26	89	7	141	27	107
159	101	123	68	186	248	60
245	69	121	25	49	93	27
124	38	57	76	78	174	18
199	35	77	15	171	139	62
40	80	68	32	44	189	4
151	87	100	52	120	163	22
271	55	77	29	123	106	35
1934	4	10	3	38	13	13
74	33	90	45	143	181	16
161	53	91	45	110	150	19
640	30	84	24	278	102	42
284	48	113	35	290	207	35
116	48	77	73	78	100	27
147	62	128	59	149	192	21
33	37	50	13	37	52	8
1273	28	60	16	89	45	24
235	52	52	78	77	74	60
122	36	119	39	79	248	44
302	13	63	42	122	51	64
125	44	104	54	164	122	26
222	60	120	31	164	151	16
460	48	85	30	319	103	47
120	56	98	43	76	78	8
95	12	49	18	14	25	22
418	38	84	20	259	106	49
151	70	81	24	127	90	10
91	21	50	16	29	40	10
190	5	67	0	79	174	21
573	18	43	14	203	50	111

23	10	45	13	16	16	19
466	28	40	14	235	66	37
22	22	39	21	57	85	9
91	32	59	35	70	88	29
543	17	42	14	192	48	104
51	12	58	6	38	18	37
76	94	33	50	49	96	20
165	22	49	29	154	142	31
11	48	58	75	2	104	0
297	21	9	1	116	16	14
89	47	69	25	139	93	29
34	39	53	52	87	69	49
109	27	34	33	96	109	11
121	108	108	79	48	130	20
1148	34	54	5	142	20	80
237	20	17	24	111	33	25
64	34	46	25	63	53	4
235	10	53	51	147	94	24
109	53	88	19	70	114	26
57	28	12	23	61	87	4
108	29	45	21	100	73	18
61	18	43	39	118	73	25
551	12	28	10	81	30	15
46	14	80	8	23	35	19
170	33	42	35	83	107	19
70	32	75	22	72	80	3
17	76	18	14	36	78	8
190	29	43	33	32	51	30
73	42	68	42	55	124	20
48	27	71	28	107	114	19
489	4	40	5	84	17	81
64	40	26	37	36	37	3
356	24	46	27	123	38	6
56	34	46	24	123	88	12
140	38	45	30	100	97	20
120	32	56	19	97	57	27
1485	0	1	3	13	4	9
110	22	52	13	72	72	23
84	46	58	1	65	19	4
52	22	38	36	30	47	3
178	26	61	0	50	71	11
41	11	30	12	52	29	3
34	9	56	3	25	10	10
40	29	29	24	32	35	16
60	15	52	24	45	64	40
67	10	57	26	18	37	5
37	22	25	11	21	61	8
76	16	34	22	37	62	14
166	11	13	9	81	27	9
67	20	25	26	59	33	8
598	2	2	2	21	8	9
29	20	17	16	30	70	3
179	24	41	23	43	39	5
54	13	34	25	24	90	3
47	31	26	33	34	77	4
163	8	42	5	70	54	15
84	21	24	6	55	51	23
66	21	31	22	61	82	3
59	1	23	4	12	12	10

112	14	42	11	55	40	3
259	3	8	2	117	24	35
75	23	31	17	64	57	11
75	23	31	17	64	57	11
14	2	16	4	8	13	6
62	13	32	28	29	39	3
117	21	16	15	32	62	6
52	7	44	4	14	20	13
89	18	42	20	57	52	5
32	13	36	8	48	57	6
90	3	19	19	51	18	17
78	10	19	33	19	46	11
54	11	32	13	30	38	12
51	14	34	11	100	37	19
50	6	8	10	19	24	9
54	37	25	11	10	33	13
55	12	22	14	28	51	10
499	0	0	2	4	3	0
47	14	32	14	28	39	8
61	10	19	12	69	31	14
44	15	36	19	38	54	22
40	10	5	21	17	20	5
55	10	17	11	63	29	11
13	16	20	8	13	40	4
46	16	26	14	6	39	11
105	9	31	14	46	28	3
44	8	18	23	19	32	9
584	1	0	0	1	1	1
22	16	33	20	12	29	12
26	16	11	17	13	10	1
57	12	20	13	16	28	7
17	6	18	6	8	21	4
79	6	20	5	54	13	26
562	1	1	1	5	0	45
28	9	15	11	12	51	4
51	7	19	1	41	14	10
0	53	0	0	0	0	0
34	23	31	13	14	36	7
14	7	3	12	48	29	8
21	9	9	8	25	20	5
21	2	8	6	4	13	3
31	5	17	6	23	17	11
15	3	15	6	11	5	15
31	10	17	17	24	42	2
12	15	8	9	16	21	3
11	10	13	15	20	35	4
39	7	18	1	36	14	8
11	2	19	4	13	1	5
199	1	0	2	5	3	19
199	8	20	4	19	6	8
29	8	23	17	44	27	13
11	12	19	7	24	19	2
53	3	13	7	30	11	7
12	14	25	7	18	19	4
20	2	16	3	5	8	11
97	7	13	7	41	10	16
28	12	6	3	23	11	13
67	12	20	7	22	35	2
17	12	16	7	15	19	4

22	4	15	1	11	5	0
13	3	14	2	4	9	4
34	4	27	10	20	26	3
20	6	12	10	22	24	5
28	8	6	7	4	19	5
52	7	9	3	51	11	6
34	8	24	5	24	29	7
11	11	18	7	8	23	0
6	4	11	7	9	16	2
9	4	18	4	13	32	2
22	11	18	7	24	26	1
28	6	10	8	28	12	14
167	3	8	2	29	2	28
128	1	6	5	38	10	2
65	6	4	2	24	12	10
41	5	11	7	21	25	6
9	5	10	6	8	15	1
20	11	11	2	17	5	3
21	7	18	9	25	18	1
33	7	14	12	7	14	6
13	5	9	10	13	23	9
88	5	7	3	28	9	7
22	6	13	3	32	17	10
7	0	20	0	4	10	4
3	15	5	12	20	35	3
12	4	17	3	9	10	8
1	4	12	0	15	9	1
25	5	15	13	10	14	3
52	3	10	4	9	7	10
12	5	3	6	6	13	0
13	2	19	1	14	21	6
8	5	11	3	3	11	2
143	3	3	2	0	2	0
8	9	5	8	6	13	1
97	3	9	3	20	11	13
33	8	10	11	5	6	4
24	7	8	2	37	17	9
9	4	14	3	22	27	0
67	5	4	3	27	27	2
35	3	6	7	21	5	4
11	5	7	3	12	14	7
6	11	3	7	8	20	4
45	4	9	4	42	20	6
10	7	7	3	12	15	1
8	1	11	11	20	10	6
12	11	3	7	8	15	1
11	6	6	3	10	12	1
113	1	1	0	6	3	7
34	4	7	6	15	8	6
9	2	17	6	13	16	4
4	8	7	4	15	10	0
34	8	7	2	18	10	3
12	2	5	9	8	7	6
65	1	3	0	19	1	1
8	7	12	3	14	4	7
22	3	11	1	14	6	2
15	7	16	0	13	11	2
13	4	4	7	12	17	5
55	3	6	0	17	2	0

10	2	12	3	8	13	0
34	4	7	2	22	8	4
15	6	4	2	20	12	1
13	4	18	6	6	23	7
11	4	5	3	10	7	0
17	5	6	9	4	3	0
8	1	5	1	9	6	4
51	4	2	1	38	5	7
59	2	1	2	18	3	1
7	7	7	3	5	6	6
22	3	11	6	10	13	1
4	1	4	5	8	14	0
11	2	14	6	12	11	5
30	2	13	7	11	8	2
9	5	11	6	6	11	0
94	2	4	3	12	4	1
3	0	2	2	0	3	2
21	4	5	1	18	13	1
10	4	3	1	8	12	5
2	6	8	3	7	5	0
5	2	10	1	9	4	1
6	3	8	2	10	6	4
17	5	19	7	6	17	0
2	1	7	1	4	3	1
6	14	3	2	10	13	2
6	5	11	5	7	16	1
6	3	2	2	13	7	2
8	5	10	4	9	16	0
5	3	2	1	8	5	1
17	2	5	0	9	5	4
41	5	3	0	41	3	0
7	1	4	0	3	5	1
10	2	16	3	2	1	1
49	2	4	1	12	4	1
2	4	3	0	7	16	0
5	4	5	3	5	8	3
30	0	0	1	8	5	8
20	4	10	2	8	4	2
1	5	5	5	8	5	1
4	7	2	0	5	11	0
36	2	0	2	8	4	0
5	4	4	3	5	2	2
19	2	6	0	19	4	10
15	0	5	1	8	0	4
3	4	5	1	3	3	5
4	2	2	1	2	9	5
7	1	2	6	5	4	0
10	2	7	3	6	7	4
10	4	4	3	2	5	0
10	0	4	3	6	3	2
30	3	2	1	19	2	4
78	0	3	1	2	4	2
125	0	0	0	1	0	1
42	2	8	3	9	6	1
2	1	2	2	3	3	1
3	0	3	0	6	3	2
6	1	3	6	2	8	1
3	3	0	4	3	4	0
64	0	0	0	0	0	0

22	0	5	0	1	2	12
9	0	4	0	2	1	2
30	0	6	0	11	3	2
2	7	3	2	7	15	1
8	4	3	1	1	17	3
12	0	3	2	2	7	0
64	0	1	0	8	0	2
13	3	11	5	2	4	0
7	2	2	1	22	7	4
16	5	2	3	3	11	0
8	3	5	2	10	6	2
75	2	0	1	3	1	1
8	4	1	1	12	7	0
7	1	1	0	8	7	1
5	0	4	1	2	8	3
10	0	4	1	10	5	3
2	2	3	1	5	5	1
4	0	7	0	3	5	0
15	3	1	2	6	1	0
4	4	7	1	9	4	1
5	1	3	1	1	0	1
50	0	0	1	0	0	0
10	5	4	1	10	6	3
1	1	3	1	9	3	1
16	1	7	0	9	7	0
8	1	4	2	9	6	0
40	1	5	1	2	0	0
14	0	1	1	13	3	5
6	4	2	1	4	9	0
7	2	5	1	6	5	0
3	1	2	3	3	7	1
3	0	1	1	5	5	5
18	1	2	2	5	4	1
10	0	1	1	2	1	0
8	2	1	4	6	3	2
8	3	5	1	6	7	0
8	2	1	1	7	5	3
5	1	1	2	1	3	0
1	2	1	1	0	2	4
2	1	1	2	0	2	0
3	3	1	1	0	3	0
5	1	2	1	1	4	2
0	2	3	4	5	4	3
5	1	4	0	4	2	0
10	1	2	2	5	10	0
25	0	0	0	2	1	2
9	1	4	0	0	3	0
32	3	3	0	4	2	0
4	5	4	2	5	7	3
16	1	2	0	2	1	3
15	10	3	2	6	2	0
11	0	2	0	1	3	0
23	0	0	1	4	0	2
20	3	0	2	1	5	0
11	3	2	1	4	4	2
13	2	2	2	2	3	2
3	1	3	2	6	8	1
15	0	1	0	1	1	1
43	1	0	0	5	1	5

6	0	0	1	1	1	1
8	3	1	1	3	0	0
54	0	0	0	2	0	2
24	0	0	1	6	0	2
0	1	1	3	0	5	0
1	0	1	0	4	7	2
2	4	2	0	6	2	1
0	3	1	0	7	4	4
5	2	1	2	3	9	1
38	0	0	0	2	0	0
17	1	0	0	1	0	1
6	1	2	2	3	0	0
3	0	2	1	2	4	0
11	2	3	0	2	4	0
1	2	2	0	2	14	0
1	0	3	1	3	4	2
0	1	1	1	7	2	1
3	1	1	1	1	3	0
18	0	3	0	2	1	3
3	1	3	1	2	6	1
1	0	1	0	0	5	0
17	0	0	0	2	1	0
0	1	2	1	0	3	0
8	2	0	2	5	7	0
2	2	1	0	2	4	0
3	4	2	1	5	6	2
7	0	2	1	4	2	2
1	0	2	1	2	0	2
10	0	1	4	0	4	3
6	3	1	1	2	1	0
0	2	0	1	1	3	0
0	2	3	1	3	3	0
20	0	0	0	2	2	13
23	0	0	1	2	0	0
3	0	9	3	1	0	1
4	0	2	2	4	0	0
5	1	1	1	3	0	1
2	1	2	1	1	2	0
2	2	2	1	5	1	2
9	1	2	0	7	2	0
5	1	6	2	2	2	0
2	1	2	0	1	0	0
9	0	1	1	7	4	1
2	0	0	1	3	1	0
6	1	2	2	3	4	1
2	1	1	0	0	0	1
2	0	0	1	0	2	1
1	1	0	1	0	6	0
5	3	1	1	1	1	0
0	2	0	0	0	0	1
4	0	2	1	4	1	1
2	0	1	0	6	2	0
219783	35733	64778	41274	110150	95123	34903
347971	81894	115766	91506	207787	197938	50068
314	404	377	412	350	356	410
CHR_P	CHR_P	CHR_P	CHR_P	CHR_P	CHR_P	CHR_P
CHR_P	CHR_P	CHR_P	CHR_P	CHR_P	CHR_P	CHR_P

85	86	87	88	89	90	91
20	21	22	23	24	25	26
CHR_P	CHR_P	CHR_P	CHR_P	CHR_P	CHR_P	CHR_P
E_48	E_7	E_8	E_9	F_40	F_41	F_42
7479891	1160935	2099567	1323600	3571995	3076499	1136044
CHR_P	CHR_P	CHR_P	CHR_P	CHR_P	CHR_P	CHR_P
4.54	12.06	8.77	11.80	10.14	10.66	6.92
0.95	2.62	3.03	2.25	3.38	3.88	2.73
0.63	4.16	4.17	3.65	1.31	3.87	0.42
1.60	0.85	1.75	1.91	1.58	1.80	4.09
1.12	2.31	2.41	2.07	1.70	1.77	1.86
6.56	1.07	1.24	1.11	2.41	1.04	1.69
1.60	1.94	2.53	1.96	1.41	1.66	1.27
5.13	0.44	0.15	0.09	0.61	0.14	0.68
1.22	0.69	0.86	0.58	0.96	0.76	0.61
0.82	0.48	0.47	0.64	0.95	0.57	1.21
0.44	0.18	0.35	0.51	0.43	0.28	0.81
0.41	0.57	0.61	0.46	0.44	0.46	0.48
0.69	0.14	0.49	0.39	0.41	0.34	1.63
0.26	0.26	0.29	0.35	0.21	0.29	0.36
1.09	0.08	0.21	0.10	0.54	0.12	1.52
0.10	0.29	0.30	0.30	0.12	0.26	0.22
0.64	0.10	0.33	0.14	0.44	0.15	0.40
0.26	0.22	0.26	0.18	0.39	0.23	0.32
0.28	0.23	0.21	0.15	0.32	0.19	0.20
0.11	0.22	0.22	0.16	0.32	0.20	0.14
0.16	0.15	0.18	0.11	0.28	0.16	0.21
0.13	0.09	0.13	0.13	0.14	0.12	0.18
0.19	0.07	0.15	0.12	0.38	0.11	0.21
0.14	0.15	0.17	0.08	0.20	0.25	0.23
0.27	0.06	0.11	0.05	0.27	0.09	0.68
0.12	0.17	0.22	0.18	0.20	0.16	0.08
0.07	0.15	0.11	0.13	0.07	0.11	0.20
0.27	0.02	0.04	0.09	0.15	0.04	0.47
0.17	0.15	0.13	0.19	0.09	0.16	0.12
0.06	0.09	0.13	0.11	0.12	0.13	0.09
0.08	0.19	0.16	0.12	0.18	0.14	0.05
0.09	0.06	0.11	0.09	0.07	0.11	0.18
0.40	0.06	0.09	0.06	0.07	0.06	0.12
0.40	0.01	0.02	0.01	0.12	0.02	0.11
0.15	0.14	0.12	0.18	0.08	0.15	0.11
0.06	0.07	0.07	0.13	0.06	0.10	0.05
0.07	0.13	0.06	0.06	0.11	0.06	0.07
0.07	0.05	0.07	0.07	0.09	0.06	0.11
0.03	0.04	0.07	0.07	0.09	0.04	0.05
0.03	0.11	0.06	0.03	0.05	0.08	0.07
0.02	0.09	0.07	0.07	0.05	0.08	0.02
0.03	0.06	0.07	0.06	0.07	0.06	0.07
0.03	0.10	0.07	0.07	0.03	0.09	0.03
0.29	0.01	0.01	0.01	0.05	0.01	0.24
0.03	0.11	0.10	0.07	0.03	0.07	0.03
0.03	0.07	0.07	0.05	0.06	0.06	0.02
0.04	0.05	0.06	0.05	0.05	0.06	0.04
0.02	0.05	0.04	0.05	0.04	0.04	0.03
0.37	0.00	0.01	0.01	0.00	0.00	0.03
0.09	0.04	0.03	0.01	0.09	0.01	0.11
0.01	0.02	0.04	0.01	0.01	0.01	0.02
0.02	0.04	0.05	0.03	0.04	0.04	0.02

0.01	0.03	0.03	0.03	0.02	0.03	0.02
0.02	0.03	0.03	0.02	0.07	0.03	0.06
0.07	0.03	0.04	0.02	0.04	0.01	0.03
0.24	0.00	0.00	0.00	0.01	0.00	0.02
0.02	0.04	0.03	0.03	0.02	0.03	0.01
0.02	0.04	0.05	0.02	0.03	0.03	0.05
0.01	0.02	0.03	0.03	0.03	0.03	0.03
0.00	0.06	0.02	0.03	0.01	0.05	0.02
0.01	0.02	0.02	0.02	0.02	0.02	0.01
0.02	0.03	0.03	0.01	0.03	0.02	0.03
0.09	0.00	0.00	0.00	0.01	0.00	0.01
0.19	0.00	0.00	0.00	0.00	0.00	0.00
0.01	0.02	0.01	0.03	0.01	0.02	0.02
0.04	0.01	0.01	0.01	0.02	0.01	0.02
0.06	0.01	0.02	0.01	0.02	0.01	0.01
0.08	0.01	0.01	0.01	0.01	0.01	0.03
0.02	0.02	0.02	0.01	0.02	0.02	0.01
0.01	0.00	0.01	0.03	0.02	0.02	0.01
0.00	0.02	0.02	0.01	0.01	0.02	0.00
0.01	0.02	0.01	0.02	0.02	0.02	0.02
0.05	0.00	0.01	0.00	0.02	0.00	0.05
0.02	0.01	0.01	0.01	0.03	0.02	0.02
0.02	0.02	0.03	0.02	0.02	0.03	0.01
0.01	0.00	0.01	0.00	0.01	0.01	0.02
0.01	0.02	0.02	0.01	0.02	0.01	0.01
0.01	0.02	0.01	0.01	0.01	0.03	0.01
0.00	0.01	0.01	0.01	0.01	0.01	0.01
0.00	0.01	0.01	0.01	0.01	0.01	0.00
0.02	0.01	0.01	0.02	0.01	0.02	0.01
0.00	0.02	0.01	0.01	0.01	0.01	0.01
0.01	0.01	0.01	0.01	0.01	0.01	0.01
0.02	0.00	0.01	0.00	0.01	0.00	0.00
0.02	0.01	0.01	0.01	0.01	0.01	0.01
0.00	0.02	0.01	0.01	0.01	0.01	0.00
0.00	0.01	0.01	0.01	0.01	0.01	0.00
0.02	0.01	0.01	0.00	0.01	0.01	0.01
0.01	0.01	0.01	0.01	0.02	0.01	0.01
0.00	0.01	0.01	0.01	0.00	0.01	0.02
0.00	0.01	0.01	0.00	0.01	0.01	0.01
0.08	0.00	0.00	0.00	0.00	0.00	0.00
0.01	0.01	0.01	0.01	0.01	0.01	0.01
0.01	0.01	0.01	0.01	0.01	0.01	0.00
0.03	0.00	0.00	0.00	0.01	0.00	0.00
0.01	0.01	0.01	0.00	0.01	0.01	0.01
0.00	0.01	0.01	0.01	0.01	0.01	0.00
0.00	0.01	0.01	0.01	0.01	0.00	0.01
0.00	0.01	0.01	0.01	0.01	0.01	0.01
0.02	0.00	0.00	0.00	0.01	0.00	0.01
0.00	0.01	0.00	0.00	0.00	0.01	0.00
0.00	0.01	0.01	0.01	0.01	0.01	0.00
0.00	0.01	0.01	0.01	0.00	0.01	0.00
0.01	0.00	0.01	0.00	0.00	0.00	0.02
0.01	0.00	0.00	0.00	0.01	0.00	0.00
0.02	0.00	0.00	0.00	0.01	0.00	0.01
0.00	0.01	0.01	0.00	0.00	0.01	0.00
0.00	0.01	0.01	0.01	0.01	0.01	0.00
0.02	0.00	0.00	0.00	0.00	0.00	0.01
0.01	0.00	0.00	0.00	0.00	0.00	0.01
0.01	0.01	0.01	0.00	0.01	0.00	0.01

0.00	0.01	0.01	0.00	0.01	0.01	0.00
0.00	0.01	0.00	0.00	0.00	0.01	0.00
0.01	0.00	0.00	0.00	0.00	0.00	0.01
0.00	0.00	0.01	0.01	0.00	0.01	0.00
0.00	0.00	0.00	0.00	0.00	0.00	0.01
0.00	0.01	0.00	0.00	0.00	0.01	0.01
0.00	0.00	0.00	0.00	0.00	0.00	0.00
0.00	0.00	0.00	0.00	0.00	0.00	0.00
0.00	0.00	0.00	0.00	0.00	0.00	0.00
0.00	0.00	0.00	0.00	0.00	0.00	0.00
0.00	0.00	0.00	0.00	0.00	0.00	0.00
0.01	0.00	0.00	0.00	0.00	0.00	0.01
0.00	0.00	0.00	0.00	0.01	0.00	0.01
0.02	0.00	0.00	0.00	0.00	0.00	0.00
0.00	0.00	0.00	0.00	0.00	0.00	0.00
0.00	0.00	0.00	0.00	0.00	0.00	0.00
0.00	0.00	0.00	0.00	0.00	0.00	0.00
0.00	0.00	0.00	0.00	0.00	0.00	0.00
0.00	0.00	0.00	0.00	0.00	0.00	0.00
0.01	0.00	0.00	0.00	0.00	0.00	0.00
0.00	0.00	0.00	0.00	0.00	0.00	0.00
0.00	0.01	0.00	0.00	0.00	0.00	0.00
0.00	0.00	0.00	0.00	0.00	0.00	0.00
0.01	0.00	0.00	0.00	0.00	0.00	0.00
0.00	0.00	0.00	0.00	0.00	0.00	0.00
0.00	0.00	0.00	0.00	0.00	0.00	0.01
0.00	0.00	0.00	0.00	0.00	0.00	0.00
0.00	0.00	0.00	0.00	0.00	0.00	0.00
0.00	0.00	0.00	0.00	0.00	0.00	0.00
0.00	0.00	0.00	0.00	0.00	0.00	0.00
0.00	0.00	0.00	0.00	0.00	0.00	0.00
0.00	0.00	0.00	0.00	0.00	0.00	0.00
0.00	0.00	0.00	0.00	0.00	0.00	0.00
0.01	0.00	0.00	0.00	0.00	0.00	0.00
0.00	0.00	0.00	0.00	0.00	0.00	0.00
0.00	0.00	0.00	0.00	0.00	0.00	0.00
0.00	0.00	0.00	0.00	0.00	0.00	0.00
0.00	0.00	0.00	0.00	0.00	0.00	0.00
0.00	0.00	0.00	0.00	0.00	0.00	0.00
0.00	0.00	0.00	0.00	0.00	0.00	0.00
0.00	0.00	0.00	0.00	0.00	0.00	0.00
0.00	0.00	0.00	0.00	0.00	0.00	0.00
0.00	0.00	0.00	0.00	0.00	0.00	0.00
0.00	0.00	0.00	0.00	0.00	0.00	0.00
0.00	0.00	0.00	0.00	0.00	0.00	0.00
0.00	0.00	0.00	0.00	0.00	0.00	0.00
0.00	0.00	0.00	0.00	0.00	0.00	0.00
0.00	0.00	0.00	0.00	0.00	0.00	0.00
0.00	0.00	0.00	0.00	0.00	0.00	0.00
0.01	0.00	0.00	0.00	0.00	0.00	0.00
0.00	0.00	0.00	0.00	0.00	0.00	0.00
0.00	0.00	0.00	0.00	0.00	0.00	0.00
0.00	0.00	0.00	0.00	0.00	0.00	0.00
0.00	0.00	0.00	0.00	0.00	0.00	0.00
0.00	0.00	0.00	0.00	0.00	0.00	0.00
0.00	0.00	0.00	0.00	0.00	0.00	0.00
0.00	0.00	0.00	0.00	0.00	0.00	0.00
0.01	0.00	0.00	0.00	0.00	0.00	0.00
0.00	0.00	0.00	0.00	0.00	0.00	0.00

0.00	0.00	0.00	0.00	0.00	0.00	0.00
0.00	0.00	0.00	0.00	0.00	0.00	0.00
0.00	0.00	0.00	0.00	0.00	0.00	0.00
0.00	0.00	0.00	0.00	0.00	0.00	0.00
0.00	0.00	0.00	0.00	0.00	0.00	0.00
0.00	0.00	0.00	0.00	0.00	0.00	0.00
0.00	0.00	0.00	0.00	0.00	0.00	0.00
0.00	0.00	0.00	0.00	0.00	0.00	0.00
0.00	0.00	0.00	0.00	0.00	0.00	0.00
0.00	0.00	0.00	0.00	0.00	0.00	0.00
0.00	0.00	0.00	0.00	0.00	0.00	0.00
0.00	0.00	0.00	0.00	0.00	0.00	0.00
0.00	0.00	0.00	0.00	0.00	0.00	0.00
0.00	0.00	0.00	0.00	0.00	0.00	0.00
0.00	0.00	0.00	0.00	0.00	0.00	0.00
0.00	0.00	0.00	0.00	0.00	0.00	0.00
0.00	0.00	0.00	0.00	0.00	0.00	0.00
0.00	0.00	0.00	0.00	0.00	0.00	0.00
0.00	0.00	0.00	0.00	0.00	0.00	0.00
0.00	0.00	0.00	0.00	0.00	0.00	0.00
0.00	0.00	0.00	0.00	0.00	0.00	0.00
0.00	0.00	0.00	0.00	0.00	0.00	0.00
0.00	0.00	0.00	0.00	0.00	0.00	0.00
0.00	0.00	0.00	0.00	0.00	0.00	0.00
0.00	0.00	0.00	0.00	0.00	0.00	0.00
0.00	0.00	0.00	0.00	0.00	0.00	0.00
0.01	0.00	0.00	0.00	0.00	0.00	0.00
0.00	0.00	0.00	0.00	0.00	0.00	0.00
0.00	0.00	0.00	0.00	0.00	0.00	0.00
0.00	0.00	0.00	0.00	0.00	0.00	0.00
0.00	0.00	0.00	0.00	0.00	0.00	0.00
0.00	0.00	0.00	0.00	0.00	0.00	0.00
0.00	0.00	0.00	0.00	0.00	0.00	0.00
0.00	0.00	0.00	0.00	0.00	0.00	0.00
0.00	0.00	0.00	0.00	0.00	0.00	0.00
0.00	0.00	0.00	0.00	0.00	0.00	0.00
0.00	0.00	0.00	0.00	0.00	0.00	0.00
0.00	0.00	0.00	0.00	0.00	0.00	0.00
0.00	0.00	0.00	0.00	0.00	0.00	0.00
0.00	0.00	0.00	0.00	0.00	0.00	0.00
0.00	0.00	0.00	0.00	0.00	0.00	0.00
0.00	0.00	0.00	0.00	0.00	0.00	0.00
0.00	0.00	0.00	0.00	0.00	0.00	0.00
0.00	0.00	0.00	0.00	0.00	0.00	0.00
0.00	0.00	0.00	0.00	0.00	0.00	0.00
0.00	0.00	0.00	0.00	0.00	0.00	0.00
0.00	0.00	0.00	0.00	0.00	0.00	0.00
0.00	0.00	0.00	0.00	0.00	0.00	0.00
0.01	0.00	0.00	0.00	0.00	0.00	0.00
0.00	0.00	0.00	0.00	0.00	0.00	0.00
0.00	0.00	0.00	0.00	0.00	0.00	0.00
0.00	0.00	0.00	0.00	0.00	0.00	0.00
0.00	0.00	0.00	0.00	0.00	0.00	0.00
0.00	0.00	0.00	0.00	0.00	0.00	0.00
0.00	0.00	0.00	0.00	0.00	0.00	0.00
0.00	0.00	0.00	0.00	0.00	0.00	0.00
0.00	0.00	0.00	0.00	0.00	0.00	0.00
0.00	0.00	0.00	0.00	0.00	0.00	0.00
0.00	0.00	0.00	0.00	0.00	0.00	0.00

0.00	0.00	0.00	0.00	0.00	0.00	0.00
0.00	0.00	0.00	0.00	0.00	0.00	0.00
0.00	0.00	0.00	0.00	0.00	0.00	0.00
0.00	0.00	0.00	0.00	0.00	0.00	0.00
0.00	0.00	0.00	0.00	0.00	0.00	0.00
0.00	0.00	0.00	0.00	0.00	0.00	0.00
0.00	0.00	0.00	0.00	0.00	0.00	0.00
0.00	0.00	0.00	0.00	0.00	0.00	0.00
0.00	0.00	0.00	0.00	0.00	0.00	0.00
0.00	0.00	0.00	0.00	0.00	0.00	0.00
0.00	0.00	0.00	0.00	0.00	0.00	0.00
0.00	0.00	0.00	0.00	0.00	0.00	0.00
0.00	0.00	0.00	0.00	0.00	0.00	0.00
0.00	0.00	0.00	0.00	0.00	0.00	0.00
0.00	0.00	0.00	0.00	0.00	0.00	0.00
0.00	0.00	0.00	0.00	0.00	0.00	0.00
0.00	0.00	0.00	0.00	0.00	0.00	0.00
0.00	0.00	0.00	0.00	0.00	0.00	0.00
0.00	0.00	0.00	0.00	0.00	0.00	0.00
0.00	0.00	0.00	0.00	0.00	0.00	0.00
0.00	0.00	0.00	0.00	0.00	0.00	0.00
0.00	0.00	0.00	0.00	0.00	0.00	0.00
0.00	0.00	0.00	0.00	0.00	0.00	0.00
0.00	0.00	0.00	0.00	0.00	0.00	0.00
0.00	0.00	0.00	0.00	0.00	0.00	0.00
0.00	0.00	0.00	0.00	0.00	0.00	0.00
0.00	0.00	0.00	0.00	0.00	0.00	0.00
0.00	0.00	0.00	0.00	0.00	0.00	0.00
0.00	0.00	0.00	0.00	0.00	0.00	0.00
0.00	0.00	0.00	0.00	0.00	0.00	0.00
85	86	87	88	89	90	91

21	22	23	24	25	26	27
CHR_PE_48 7479891	CHR_PE_7 1160935	CHR_PE_8 2099567	CHR_PE_9 1323600	CHR_PF_40 3571995	CHR_PF_41 3076499	CHR_PF_42 1136044
E_48	E_7	E_8	E_9	F_40	F_41	F_42
-1.59	0.10	-0.64	0.04	-0.33	-0.22	-1.06
-0.68	-0.19	-0.07	-0.30	0.03	0.18	-0.16
-1.11	1.55	1.55	1.16	-0.60	1.32	-1.27
-0.83	-1.58	-0.69	-0.53	-0.86	-0.65	1.62
-1.15	0.16	0.27	-0.10	-0.51	-0.44	-0.33
4.85	-0.11	0.04	-0.08	1.10	-0.14	0.45
0.68	1.24	2.17	1.26	0.38	0.78	0.17
8.65	0.18	-0.35	-0.45	0.49	-0.37	0.60

1.25	0.20	0.53	-0.02	0.74	0.33	0.04
0.14	-0.72	-0.75	-0.30	0.50	-0.47	1.16
-0.12	-1.08	-0.44	0.13	-0.15	-0.73	1.26
-0.74	0.70	1.07	-0.31	-0.50	-0.30	-0.11
1.91	-1.26	0.76	0.21	0.31	-0.10	7.32
-1.20	-1.22	-1.02	-0.66	-1.48	-1.02	-0.62
4.03	-0.64	-0.06	-0.54	1.49	-0.46	5.96
-0.87	-0.04	0.02	0.00	-0.77	-0.15	-0.34
3.05	-0.34	1.11	-0.11	1.81	-0.06	1.52
0.40	0.02	0.41	-0.34	1.72	0.17	1.07
0.86	0.40	0.23	-0.26	1.24	0.09	0.14
-1.22	-0.10	-0.15	-0.70	0.94	-0.35	-0.93
0.08	-0.09	0.35	-0.73	2.00	0.09	0.77
-0.40	-0.99	-0.36	-0.29	-0.14	-0.45	0.38
0.86	-0.61	0.41	0.05	3.18	-0.07	1.13
0.19	0.30	0.48	-0.40	0.80	1.24	1.03
0.83	-0.44	-0.11	-0.48	0.85	-0.21	3.24
0.10	1.08	2.04	1.24	1.60	0.87	-0.74
-1.11	0.11	-0.40	-0.14	-1.03	-0.38	0.95
1.49	-0.92	-0.67	-0.21	0.40	-0.66	3.43
1.17	0.84	0.44	1.64	-0.30	1.07	0.30
-1.06	-0.22	0.55	0.07	0.38	0.71	-0.31
-0.15	2.33	1.65	0.61	2.13	1.22	-0.92
-0.54	-1.18	-0.31	-0.66	-0.97	-0.22	1.14
5.16	0.05	0.48	0.02	0.22	-0.03	0.89
1.76	-0.34	-0.27	-0.33	0.25	-0.29	0.22
1.31	1.06	0.59	1.84	-0.21	1.32	0.41
-0.52	-0.47	-0.43	-0.08	-0.53	-0.26	-0.56
-0.46	0.15	-0.63	-0.68	-0.09	-0.63	-0.47
-0.25	-0.43	-0.21	-0.27	-0.05	-0.31	0.14
-0.89	-0.67	-0.37	-0.35	-0.12	-0.67	-0.56
-0.53	0.09	-0.33	-0.59	-0.36	-0.16	-0.25
-1.23	0.19	-0.09	-0.10	-0.61	0.04	-1.22
-1.13	-0.32	0.13	-0.35	-0.05	-0.26	0.02
-1.27	1.37	0.08	0.35	-1.26	0.86	-1.51
6.22	-0.61	-0.63	-0.67	0.24	-0.64	5.00
-0.59	1.03	0.86	0.11	-0.71	0.13	-0.71
-1.45	2.03	1.79	0.40	0.69	1.11	-1.58
-0.13	0.38	0.80	0.25	0.29	0.74	-0.02
-1.03	0.11	-0.39	-0.10	-0.27	-0.43	-0.55
12.72	-0.30	-0.07	0.05	-0.27	-0.27	0.71
1.40	0.29	-0.10	-0.38	1.48	-0.39	1.87
-1.61	-1.04	0.01	-1.45	-1.20	-1.21	-0.79
-0.41	0.08	0.32	-0.22	0.19	0.29	-0.28
-1.72	-0.60	-0.82	-0.57	-1.06	-0.28	-1.49
-0.27	-0.05	0.26	-0.43	2.31	0.20	1.71
2.29	0.03	1.07	-0.17	0.79	-0.66	0.03

9.36	-0.28	-0.25	-0.26	-0.08	-0.30	0.32
-0.40	0.59	0.09	0.21	-0.41	0.42	-1.21
0.03	1.59	2.45	-0.01	0.77	0.39	2.24
-1.29	-0.94	0.30	0.25	0.16	0.31	-0.19
-0.97	0.46	-0.61	-0.35	-0.91	0.16	-0.63
-1.29	0.03	-0.08	-0.44	-0.52	-0.15	-1.14
-0.22	0.52	0.53	-0.77	1.02	-0.11	0.80
6.09	-0.51	-0.48	-0.50	-0.18	-0.48	-0.21
8.26	-0.19	-0.18	-0.20	-0.23	-0.21	-0.19
-1.03	-0.37	-1.06	-0.09	-0.96	-0.83	-0.30
2.14	-0.49	-0.18	-0.53	0.73	-0.18	0.12
3.34	0.03	0.30	-0.09	0.61	-0.15	-0.30
6.90	-0.52	-0.29	-0.51	0.37	-0.38	2.20
0.95	0.53	1.32	0.13	1.14	1.19	0.11
-0.28	-0.38	-0.29	-0.03	-0.13	-0.20	-0.26
-1.22	0.02	-0.36	-0.56	-0.64	-0.36	-1.24
-1.23	-0.42	-1.05	-0.10	-0.49	-0.10	-0.41
3.98	-0.68	-0.47	-0.63	0.66	-0.62	3.61
0.35	-0.44	-0.24	-0.52	2.45	0.50	1.02
0.49	1.27	2.79	0.99	0.92	2.22	0.16
-0.90	-1.01	-0.59	-1.22	-0.94	-0.96	0.35
-0.38	0.28	0.25	-0.30	0.40	-0.11	-0.12
-0.42	-0.01	-0.36	-0.36	-0.40	0.19	-0.35
-1.28	-0.48	-0.64	-0.99	-0.73	-0.32	-0.62
-1.10	-0.09	-0.33	-0.55	-0.55	-0.17	-1.14
0.99	0.42	0.25	1.41	-0.56	0.81	-0.04
-1.20	0.65	0.05	-0.40	-0.19	0.13	-1.00
-0.91	-0.28	-0.70	-0.22	-0.60	-0.30	-0.83
0.58	-0.46	-0.19	-0.41	-0.22	-0.39	-0.30
1.47	0.64	1.13	0.04	0.14	0.89	0.94
-0.89	1.46	0.10	-0.06	-0.52	0.29	-1.36
-2.01	-0.19	-1.15	-0.90	-1.40	-0.16	-1.97
3.05	0.24	0.21	-0.39	0.31	0.03	0.75
0.04	0.97	1.15	0.39	1.95	1.45	0.21
-1.14	-0.39	-0.53	-0.80	-0.92	0.37	0.80
-1.12	0.84	0.17	-2.04	0.99	0.62	1.24
14.75	-0.24	-0.19	-0.16	-0.28	-0.22	-0.27
0.22	0.98	1.86	0.12	0.13	0.07	-0.26
0.36	0.64	0.37	0.31	0.25	0.37	-0.82
4.61	0.07	-0.08	-0.14	0.56	-0.17	-0.05
0.43	0.03	0.46	-0.61	0.55	0.06	0.04
-1.51	0.01	-0.49	-0.61	0.12	-0.16	-0.80
-1.50	0.08	-0.42	0.37	-0.43	-0.74	-0.07
-1.37	0.08	-0.03	-0.46	-0.05	0.30	-0.15
4.35	-0.49	-0.48	-0.57	1.26	-0.44	1.60
-0.69	0.25	-0.20	-0.60	-0.15	0.34	-0.96
-0.16	1.18	1.92	0.47	2.20	1.93	-0.78
-1.20	-0.47	-0.43	-0.35	-0.85	-0.21	-0.69

-0.23	-0.41	-0.08	-0.54	-0.41	-0.37	0.83
2.20	-0.19	-0.47	-0.20	0.87	-0.39	-0.85
4.13	-0.32	-0.38	-0.54	1.17	-0.52	1.63
-1.23	1.05	0.01	-0.29	-0.60	-0.05	-1.40
-0.52	-0.10	0.06	-0.08	-0.09	0.30	-0.44
4.15	-0.29	-0.09	-0.18	-0.16	-0.08	0.58
3.74	0.37	-0.19	-0.43	0.38	-0.29	2.34
1.60	0.58	0.91	-0.67	1.77	-0.08	0.99
-1.34	0.75	1.08	-0.26	0.42	1.32	-0.62
-1.15	1.63	-0.04	-0.46	-0.10	0.54	-1.14
3.00	-0.56	-0.42	-0.41	0.45	-0.29	0.77
0.24	-0.08	0.88	0.78	0.01	0.82	-0.94
-1.35	-1.14	-0.52	-0.22	-0.82	-0.66	0.39
-0.14	1.18	0.13	-0.27	-0.47	1.35	5.62
0.28	-0.06	0.29	-0.51	0.13	-0.16	0.02
0.28	-0.06	0.29	-0.51	0.13	-0.16	0.02
-0.98	-0.23	-0.57	-0.55	-0.59	-0.39	-0.93
-0.75	-0.07	0.02	0.32	-0.67	-0.28	-0.98
-1.40	-0.30	0.43	0.01	0.19	0.93	-1.32
1.01	-0.64	0.39	-0.75	-0.15	-0.67	1.10
0.06	0.29	0.26	-0.17	2.06	-0.16	2.43
6.10	-0.18	-0.22	-0.22	-0.16	-0.20	-0.25
-1.18	-0.69	-0.66	-0.46	-0.73	0.01	-0.56
-0.89	0.70	0.61	0.55	-0.80	0.26	-0.45
-0.87	0.89	0.82	-0.16	0.14	0.16	-1.22
-0.90	0.16	-0.23	-0.50	-0.55	0.30	-0.68
-0.63	-0.60	-0.26	-0.18	-0.16	-0.10	-0.12
1.67	-0.32	-0.29	-0.25	0.25	0.81	-0.35
-0.68	1.31	1.18	0.60	0.40	0.73	-1.23
-0.56	2.97	-0.11	-0.55	-0.79	1.12	-0.32
-0.74	0.77	-0.12	-0.32	-0.09	0.58	-0.87
3.22	0.05	0.87	-0.14	0.31	-0.15	-0.22
-1.15	0.29	0.51	-0.55	0.42	0.01	0.57

14	15	16	17	18	19	20
85	86	87	88	89	90	91
ACTCGGCGTGG	TATTGCACTCG	TTATAAAGCAAT	TTCACAGTGGCT	CCTCTGGGCCCTTC	CTGGCCCTCTCT	TCGTACCGTGAG
miR-1307-3p	miR-92b-3p	miR-340-5p	miR-27a-3p	miR-326	miR-328	miR-126-3p
51847	51252	50588	49172	49098	48416	41318
0.64	-1.14	2.86	-0.47	2.24	-0.41	-0.88
-1.76	3.28	-0.51	-1.07	-1.55	0.21	0.03
-0.83	-0.34	-0.44	-0.11	-0.84	-1.05	0.30
-0.60	-0.71	-0.46	-0.59	-0.78	-1.30	-0.25
-1.62	1.07	-0.31	-0.27	-0.87	1.15	0.27
-0.56	-0.12	-0.19	-0.61	-0.98	0.60	-0.83
0.99	-0.56	0.32	-0.80	0.64	1.82	-0.81
0.81	1.68	-0.46	-0.87	-0.03	2.04	-0.77
-0.82	1.27	-0.48	-0.62	-0.96	0.12	-0.53
-0.93	-0.11	-0.18	-0.49	-1.05	-1.15	-0.30
-0.37	0.42	-0.46	0.90	-0.63	-0.32	0.61
0.50	-0.11	-0.44	-1.03	-0.57	0.75	-0.72
0.10	-0.93	3.85	2.19	0.84	-1.36	2.36
1.12	0.52	-0.44	-1.02	0.29	0.66	-0.75
-0.55	-0.42	-0.28	-0.14	0.14	-1.10	-0.18
0.50	-0.58	0.11	0.90	0.33	0.56	1.49
0.57	-0.47	-0.06	0.38	2.07	0.21	-0.87
1.25	-1.01	-0.35	-0.21	1.42	-0.40	1.63
-0.43	-0.77	-0.33	0.50	-0.39	-0.55	0.09
-0.16	-0.78	0.27	2.05	-0.03	-1.30	2.28

0.05	0.02	-0.23	-0.82	-0.71	0.69	-0.48
1.82	-0.23	-0.34	-0.56	1.43	0.26	-0.73
1.08	0.15	-0.14	-0.57	0.88	0.43	-0.59
1.85	0.07	-0.17	-0.66	0.65	1.54	-0.85
-1.41	1.34	-0.47	0.35	-1.08	0.01	-0.61
-0.21	-0.88	-0.30	1.65	-0.12	-0.52	-0.45
-1.03	-0.69	-0.36	2.00	-0.34	-1.57	1.54
-1.23	-0.92	0.14	2.55	-0.80	-1.88	6.13
-0.75	0.05	0.00	1.41	-1.13	-0.87	0.34
-1.36	0.36	-0.37	0.42	-1.22	-0.30	-0.58
-1.51	4.70	-0.46	-0.30	-1.35	2.91	-0.77
-1.63	0.71	-0.23	5.02	-1.29	-0.94	0.10
-1.59	1.01	-0.42	0.29	-0.51	-0.38	1.59
1.04	-0.61	-0.34	-0.38	0.89	0.67	-0.63
0.77	-0.12	0.06	-0.26	2.96	1.05	-0.69
0.75	-0.42	-0.34	-0.47	0.97	-0.18	-0.64
-0.54	0.70	-0.50	-1.23	-1.16	0.89	-0.94
-0.48	-1.04	1.61	-0.75	0.76	-1.33	-0.47
-0.28	-1.27	3.62	2.20	3.53	-1.54	1.74
0.10	-0.39	-0.37	-0.28	0.39	-0.88	-0.43
3.97	-0.89	-0.44	0.05	19.65	8.00	-0.91
11.09	-1.34	-0.48	4.71	91.37	35.18	-0.83
0.77	-0.11	-0.35	-0.35	1.00	1.26	-0.55
0.07	-0.44	4.02	0.80	0.30	-0.62	-0.31
-0.04	-0.34	-0.47	-0.99	-0.53	2.34	-0.55
2.72	-0.68	-0.50	-0.92	8.68	8.03	-0.85
-0.14	-0.68	-0.16	3.52	0.53	-0.96	0.54
1.23	-0.14	0.07	-0.23	0.69	0.88	-0.51
0.47	-0.34	-0.37	-0.70	0.52	0.41	0.89
0.72	-0.44	-0.24	-0.32	0.41	-0.35	-0.39
0.27	-0.61	-0.08	-0.36	0.80	-0.37	0.88
1.56	0.30	-0.29	0.62	1.31	1.29	-0.79
0.13	0.23	-0.38	-0.28	0.33	0.08	-0.76
0.59	-0.52	0.20	0.64	1.02	0.25	-0.41
0.14	0.04	-0.35	-0.45	-0.86	1.79	-0.67
-0.21	0.16	-0.42	0.31	-0.14	-0.46	-0.67
-0.44	-0.60	-0.30	-0.35	-0.70	-0.04	-0.24
-1.08	-1.31	0.27	2.32	-0.62	-1.92	5.73
-0.11	-0.39	-0.43	-0.62	-0.17	0.25	-0.47
-1.29	0.04	0.04	1.06	-0.71	0.39	0.88
-1.49	-0.32	-0.35	0.43	-0.73	0.17	1.32
-1.23	-0.91	-0.03	1.39	-1.04	-1.83	3.73
-0.30	-0.06	-0.45	-0.05	-0.39	-0.46	-0.62
-0.74	-0.20	-0.32	-0.22	-0.68	-0.53	-0.53
-1.20	-0.88	-0.45	0.57	-0.96	-1.78	1.12
1.55	-1.18	-0.37	-0.64	-0.48	-1.71	0.60
-1.05	-1.17	0.37	0.46	-0.27	-1.60	1.95
-1.76	1.63	-0.34	1.34	-1.55	-0.19	-0.81
-0.92	2.55	-0.45	-0.65	-0.17	0.41	0.93
-0.17	0.51	-0.29	0.81	0.57	0.18	1.66
-0.13	-0.17	-0.40	-0.69	-0.08	-0.83	-0.30
-1.53	-1.26	-0.26	-0.67	-1.53	-0.10	0.94
-0.82	-0.43	-0.38	-0.61	-0.77	-1.10	0.36
0.11	-0.85	-0.29	-0.59	0.24	-1.35	1.87
9.10	-1.15	-0.50	-0.77	87.60	38.57	-0.79
-0.03	-0.70	0.10	-0.41	-0.36	-0.28	0.91
-1.08	-0.55	-0.37	0.50	-0.68	-0.78	-0.30
-1.47	0.60	-0.46	-0.48	-1.28	0.14	-0.09
0.19	-0.71	-0.15	6.11	2.29	-1.47	0.82

-0.47	-0.64	-0.24	3.61	0.20	-1.28	1.13
0.26	0.23	-0.43	1.01	0.26	-0.02	-0.63
-0.13	-0.44	-0.35	1.84	-0.20	-0.68	-0.13
1.59	-0.26	0.22	-0.68	-0.01	2.12	-0.58
-0.49	-1.14	1.57	2.04	1.08	-1.55	2.69
-1.20	-0.91	0.58	1.47	-0.89	-2.01	3.05
0.65	-0.28	-0.46	0.64	1.46	-0.19	0.24
0.05	-0.70	-0.19	1.13	0.10	-1.15	0.21
-0.40	-0.22	-0.41	0.04	-0.06	-0.90	-0.39
-0.19	-0.60	-0.22	0.14	-0.52	-1.40	0.31
0.13	-0.30	-0.39	0.89	0.29	-0.16	0.03
-1.00	-0.83	-0.30	0.94	-1.36	-1.97	0.75
0.00	-0.25	-0.28	-0.38	-1.01	0.08	-0.84
0.86	-0.39	-0.02	0.34	0.84	0.52	-0.76
-0.81	-0.65	-0.44	2.05	-0.83	-1.03	-0.20
-0.55	-0.74	0.80	0.45	0.86	-1.21	-0.18
1	-1	-2	1	1	0	3
0.00	0.00	0.00	0.00	0.00	0.00	0.00
1.00	1.00	1.00	1.00	1.00	1.00	1.00
0.69	0.49	0.31	0.68	0.93	0.74	0.59

82	83	84	85	86	87	88
ACTCGGCGTGG(	TATTGCACTCG'	TTATAAAGCAAT(	TTCACAGTGGCTA	CCTCTGGGCCCTTC(	CTGGCCCTCTCT(	TCGTACCGTGAGT
miR-1307-3p	miR-92b-3p	miR-340-5p	miR-27a-3p	miR-326	miR-328	miR-126-3p
51847	51252	50588	49172	49098	48416	41318
0.64	-1.14	2.86	-0.47	2.24	-0.41	-0.88
-1.76	3.28	-0.51	-1.07	-1.55	0.21	0.03
-0.83	-0.34	-0.44	-0.11	-0.84	-1.05	0.30
-0.60	-0.71	-0.46	-0.59	-0.78	-1.30	-0.25
-1.62	1.07	-0.31	-0.27	-0.87	1.15	0.27
-0.56	-0.12	-0.19	-0.61	-0.98	0.60	-0.83
0.99	-0.56	0.32	-0.80	0.64	1.82	-0.81
0.81	1.68	-0.46	-0.87	-0.03	2.04	-0.77
-0.82	1.27	-0.48	-0.62	-0.96	0.12	-0.53
-0.93	-0.11	-0.18	-0.49	-1.05	-1.15	-0.30
-0.37	0.42	-0.46	0.90	-0.63	-0.32	0.61
0.50	-0.11	-0.44	-1.03	-0.57	0.75	-0.72
0.10	-0.93	3.85	2.19	0.84	-1.36	2.36
1.12	0.52	-0.44	-1.02	0.29	0.66	-0.75
-0.55	-0.42	-0.28	-0.14	0.14	-1.10	-0.18
0.50	-0.58	0.11	0.90	0.33	0.56	1.49
0.57	-0.47	-0.06	0.38	2.07	0.21	-0.87
1.25	-1.01	-0.35	-0.21	1.42	-0.40	1.63
-0.43	-0.77	-0.33	0.50	-0.39	-0.55	0.09
-0.16	-0.78	0.27	2.05	-0.03	-1.30	2.28
0.05	0.02	-0.23	-0.82	-0.71	0.69	-0.48
1.82	-0.23	-0.34	-0.56	1.43	0.26	-0.73
1.08	0.15	-0.14	-0.57	0.88	0.43	-0.59
1.85	0.07	-0.17	-0.66	0.65	1.54	-0.85
-1.41	1.34	-0.47	0.35	-1.08	0.01	-0.61
-0.21	-0.88	-0.30	1.65	-0.12	-0.52	-0.45
-1.03	-0.69	-0.36	2.00	-0.34	-1.57	1.54
-1.23	-0.92	0.14	2.55	-0.80	-1.88	6.13

-0.75	0.05	0.00	1.41	-1.13	-0.87	0.34
-1.36	0.36	-0.37	0.42	-1.22	-0.30	-0.58
-1.51	4.70	-0.46	-0.30	-1.35	2.91	-0.77
-1.63	0.71	-0.23	5.02	-1.29	-0.94	0.10
-1.59	1.01	-0.42	0.29	-0.51	-0.38	1.59
1.04	-0.61	-0.34	-0.38	0.89	0.67	-0.63
0.77	-0.12	0.06	-0.26	2.96	1.05	-0.69
0.75	-0.42	-0.34	-0.47	0.97	-0.18	-0.64
-0.54	0.70	-0.50	-1.23	-1.16	0.89	-0.94
-0.48	-1.04	1.61	-0.75	0.76	-1.33	-0.47
-0.28	-1.27	3.62	2.20	3.53	-1.54	1.74
0.10	-0.39	-0.37	-0.28	0.39	-0.88	-0.43
3.97	-0.89	-0.44	0.05	19.65	8.00	-0.91
11.09	-1.34	-0.48	4.71	91.37	35.18	-0.83
0.77	-0.11	-0.35	-0.35	1.00	1.26	-0.55
0.07	-0.44	4.02	0.80	0.30	-0.62	-0.31
-0.04	-0.34	-0.47	-0.99	-0.53	2.34	-0.55
2.72	-0.68	-0.50	-0.92	8.68	8.03	-0.85
-0.14	-0.68	-0.16	3.52	0.53	-0.96	0.54
1.23	-0.14	0.07	-0.23	0.69	0.88	-0.51
0.47	-0.34	-0.37	-0.70	0.52	0.41	0.89
0.72	-0.44	-0.24	-0.32	0.41	-0.35	-0.39
0.27	-0.61	-0.08	-0.36	0.80	-0.37	0.88
1.56	0.30	-0.29	0.62	1.31	1.29	-0.79
0.13	0.23	-0.38	-0.28	0.33	0.08	-0.76
0.59	-0.52	0.20	0.64	1.02	0.25	-0.41
0.14	0.04	-0.35	-0.45	-0.86	1.79	-0.67
-0.21	0.16	-0.42	0.31	-0.14	-0.46	-0.67
-0.44	-0.60	-0.30	-0.35	-0.70	-0.04	-0.24
-1.08	-1.31	0.27	2.32	-0.62	-1.92	5.73
-0.11	-0.39	-0.43	-0.62	-0.17	0.25	-0.47
-1.29	0.04	0.04	1.06	-0.71	0.39	0.88
-1.49	-0.32	-0.35	0.43	-0.73	0.17	1.32
-1.23	-0.91	-0.03	1.39	-1.04	-1.83	3.73
-0.30	-0.06	-0.45	-0.05	-0.39	-0.46	-0.62
-0.74	-0.20	-0.32	-0.22	-0.68	-0.53	-0.53
-1.20	-0.88	-0.45	0.57	-0.96	-1.78	1.12
1.55	-1.18	-0.37	-0.64	-0.48	-1.71	0.60
-1.05	-1.17	0.37	0.46	-0.27	-1.60	1.95
-1.76	1.63	-0.34	1.34	-1.55	-0.19	-0.81
-0.92	2.55	-0.45	-0.65	-0.17	0.41	0.93
-0.17	0.51	-0.29	0.81	0.57	0.18	1.66
-0.13	-0.17	-0.40	-0.69	-0.08	-0.83	-0.30
-1.53	-1.26	-0.26	-0.67	-1.53	-0.10	0.94
-0.82	-0.43	-0.38	-0.61	-0.77	-1.10	0.36
0.11	-0.85	-0.29	-0.59	0.24	-1.35	1.87
9.10	-1.15	-0.50	-0.77	87.60	38.57	-0.79
-0.03	-0.70	0.10	-0.41	-0.36	-0.28	0.91
-1.08	-0.55	-0.37	0.50	-0.68	-0.78	-0.30
-1.47	0.60	-0.46	-0.48	-1.28	0.14	-0.09
0.19	-0.71	-0.15	6.11	2.29	-1.47	0.82
-0.47	-0.64	-0.24	3.61	0.20	-1.28	1.13
0.26	0.23	-0.43	1.01	0.26	-0.02	-0.63
-0.13	-0.44	-0.35	1.84	-0.20	-0.68	-0.13
1.59	-0.26	0.22	-0.68	-0.01	2.12	-0.58
-0.49	-1.14	1.57	2.04	1.08	-1.55	2.69
-1.20	-0.91	0.58	1.47	-0.89	-2.01	3.05
0.65	-0.28	-0.46	0.64	1.46	-0.19	0.24
0.05	-0.70	-0.19	1.13	0.10	-1.15	0.21

-0.40	-0.22	-0.41	0.04	-0.06	-0.90	-0.39
-0.19	-0.60	-0.22	0.14	-0.52	-1.40	0.31
0.13	-0.30	-0.39	0.89	0.29	-0.16	0.03
-1.00	-0.83	-0.30	0.94	-1.36	-1.97	0.75
0.00	-0.25	-0.28	-0.38	-1.01	0.08	-0.84
0.86	-0.39	-0.02	0.34	0.84	0.52	-0.76
-0.81	-0.65	-0.44	2.05	-0.83	-1.03	-0.20
-1	-1	1	0	1	-1	0

82	83	84	85	86	87	88
51	110	84	109	98	81	40
TCGGATCCGTC	TGGAGAGAAAG	TTATAAAGCAAT	TAAAGTGCTGAC	CCGCACTGTGGGTA	AATTGCACGGTA	TGAGGGGCAGAG
miR-127-3p	miR-185-5p	miR-340-5p	miR-106b-5p	miR-106b-3p	miR-363-3p	miR-423-5p
154285	23397	50588	23446	27350	52323	312449
2.23	-0.68	2.86	-0.42	0.24	-0.38	-0.27
0.39	-0.74	-0.51	-0.19	0.97	0.32	-0.54
-0.65	0.66	-0.44	0.84	-1.08	2.78	-0.18
-1.12	-0.60	-0.46	-0.19	-0.77	1.35	-0.22
0.66	-0.24	-0.31	0.96	-1.91	0.79	-0.64
-0.15	-0.93	-0.19	-0.28	-0.53	-0.86	-0.26
0.71	-0.93	0.32	-0.51	0.01	-1.21	-0.17
-0.53	-0.97	-0.46	-0.82	2.00	-0.90	1.03
0.44	-0.65	-0.48	-0.86	-1.08	1.71	-0.42
-0.33	0.96	-0.18	0.15	-1.09	-0.28	-0.65
-0.49	-0.13	-0.46	-0.49	-1.32	0.74	-0.42
-1.56	-1.13	-0.44	-0.95	-0.10	-1.29	4.21
-1.76	1.87	3.85	3.79	-0.40	0.27	-0.53
0.41	-0.87	-0.44	-0.92	0.69	-0.96	0.39
-0.25	1.16	-0.28	-0.15	-0.11	0.26	-0.57
-0.04	0.41	0.11	0.77	-0.05	0.68	-0.58
0.63	-0.46	-0.06	-0.31	0.29	0.36	-0.39
0.68	0.98	-0.35	1.00	1.93	0.82	-0.10
-0.40	-0.20	-0.33	-0.55	0.37	-1.07	-0.19
-1.63	2.88	0.27	1.44	-0.49	0.90	-0.22
-0.96	-0.77	-0.23	-0.48	-0.20	-0.60	-0.63
1.06	-0.29	-0.34	-0.83	1.40	-0.83	1.82
-0.36	-0.18	-0.14	-0.46	1.59	-0.34	0.09
-0.75	-0.49	-0.17	-0.36	0.76	-0.98	-0.07
1.78	-0.52	-0.47	-0.51	0.34	-0.84	0.25
1.21	0.38	-0.30	-0.22	-0.61	-0.50	-0.17
0.79	1.47	-0.36	0.55	-0.86	0.09	-0.58
-1.16	2.23	0.14	4.67	-1.86	2.43	-0.52
1.59	-0.60	0.00	-0.15	-0.77	-0.36	-0.44
-0.32	-0.63	-0.37	-0.65	-0.45	-0.79	-0.72
7.26	-1.01	-0.46	-0.82	0.81	-0.67	-0.65
3.08	-0.01	-0.23	-0.27	-0.19	-0.27	-0.45
-0.27	1.24	-0.42	1.79	-1.30	4.17	-0.56
-0.77	-0.80	-0.34	-0.29	0.66	-0.84	-0.09
-0.37	-0.40	0.06	0.13	1.08	-0.40	-0.55
-1.47	-0.87	-0.34	-0.65	-0.82	-0.52	0.14
-1.35	-1.24	-0.50	-1.04	-0.27	-1.15	3.28
0.17	-0.51	1.61	-0.29	0.10	-1.35	-0.70
-0.77	0.38	3.62	1.91	-1.17	-0.10	-0.71

-1.14	-0.65	-0.37	-0.65	-0.09	0.33	0.17
2.27	4.78	-0.44	0.26	4.93	-1.18	2.65
6.64	5.90	-0.48	-0.15	16.43	-0.05	3.67
1.22	-0.82	-0.35	-0.73	0.50	-0.55	-0.59
-0.99	2.47	4.02	1.60	0.85	-0.62	-0.70
-0.93	-0.98	-0.47	-1.05	2.29	-1.57	1.17
0.39	2.35	-0.50	-0.32	3.86	-1.57	3.29
0.07	-0.25	-0.16	0.19	-0.56	1.61	-0.30
0.95	-0.17	0.07	-0.37	2.49	-0.53	-0.18
-1.45	0.15	-0.37	0.20	0.70	0.10	-0.54
0.43	-0.34	-0.24	-0.26	0.02	0.30	-0.29
1.90	0.51	-0.08	0.42	-0.41	0.43	-0.57
10.02	-0.71	-0.29	-0.47	-0.16	-0.98	-0.40
5.09	-0.61	-0.38	-0.30	-0.24	-0.84	-0.45
-0.16	0.60	0.20	0.50	-0.11	-0.12	-0.49
-0.85	-1.04	-0.35	-0.71	0.60	-0.88	0.61
3.87	-0.75	-0.42	-0.62	-0.44	-0.37	-0.37
4.53	-0.45	-0.30	-0.07	-0.39	0.15	-0.63
-1.76	8.04	0.27	4.61	-1.70	0.44	-0.35
0.82	0.14	-0.43	-0.76	0.32	-0.96	0.40
-1.17	0.90	0.04	0.93	0.94	0.37	-0.33
-0.98	1.51	-0.35	1.08	-0.87	0.46	-0.45
-1.71	6.51	-0.03	3.12	-1.50	1.17	-0.30
-1.35	-0.52	-0.45	-0.66	0.17	-0.59	0.62
-0.22	0.28	-0.32	-0.30	-0.26	-0.59	0.45
-1.58	1.56	-0.45	0.11	-1.46	0.92	-0.16
-1.59	0.65	-0.37	-0.55	3.41	-0.61	0.66
-0.31	0.23	0.37	0.85	-1.66	1.21	-0.63
-0.99	-1.34	-0.34	-1.19	2.48	-1.21	-0.69
0.71	-0.07	-0.45	0.83	1.23	3.19	0.67
1.52	0.40	-0.29	1.04	1.63	4.29	-0.22
0.29	-0.47	-0.40	-0.34	0.27	-0.47	-0.21
-1.85	-0.56	-0.26	0.51	2.19	-1.73	-0.28
-1.24	-0.18	-0.38	-0.18	-0.61	1.62	-0.39
-0.67	1.75	-0.29	0.78	-0.20	-0.35	-0.39
4.92	-0.36	-0.50	-1.13	12.40	-1.18	6.68
0.71	0.96	0.10	0.69	-0.34	0.22	-0.40
-0.24	-0.52	-0.37	-0.75	-0.07	-1.14	-0.62
1.99	-0.77	-0.46	-1.03	2.09	-1.67	1.22
0.65	1.69	-0.15	1.65	-1.25	3.01	-0.68
-0.66	-0.17	-0.24	0.01	-1.32	1.12	-0.54
-1.53	-0.45	-0.43	-0.62	-0.41	1.18	-0.47
1.90	-0.35	-0.35	-0.34	-0.97	1.02	-0.49
-0.23	-0.61	0.22	-0.15	1.84	-0.72	0.11
-0.97	3.05	1.57	2.73	-0.83	0.55	-0.55
-1.61	3.74	0.58	4.15	-1.50	0.99	-0.53
-1.04	0.37	-0.46	-0.29	0.08	0.42	0.09
0.01	-0.19	-0.19	-0.09	-0.42	0.25	-0.33
-1.45	-0.43	-0.41	-0.18	0.37	1.41	-0.59
-1.20	0.38	-0.22	-0.16	-0.43	-0.56	-0.36
-1.21	-0.29	-0.39	-0.08	-0.74	0.81	-0.16
-0.79	2.34	-0.30	0.58	-0.07	-0.04	-0.25
0.19	-1.03	-0.28	-0.83	0.39	-1.21	-0.68
0.80	-0.62	-0.02	-0.20	0.59	-0.01	0.45
-0.76	0.70	-0.44	-0.56	-0.98	-0.68	-0.64
1.12	0.92	0.80	-0.49	-0.73	-0.27	-0.59

0.41	0.41	0.41	0.41	0.42	0.44	0.46
0.36	0.34	1.00	1.00	0.24	0.36	0.71

1160935	2099567	1323600	3571995	3076499	1136044	2580829

92	93	94	95	96	97	98
23	24	25	26	27	28	29
E_7	E_8	E_9	F_40	F_41	F_42	F_44
CHR_P	CHR_P	CHR_P	CHR_P	CHR_P	CHR_P	CHR_P
2333798	3006460	1323600	5508732	5304434	4176164	4502548
0.52	0.30	0.35	0.93	0.46	0.25	0.40
E_7	E_8	E_9	F_40	F_41	F_42	F_44
1172863	906893	0	1936737	2227935	3040120	1921719
430892	567895	487095	1116571	1013554	241383	1338069
93617	196075	92860	372466	369204	95275	219682
148736	270305	150760	144769	367770	14583	145667
30353	113550	79014	173851	170780	142685	146527
82596	155876	85452	187609	167992	65057	146024
38241	80210	45678	265139	98721	59034	23965
69449	163721	80719	154960	157746	44478	54262
15818	9548	3869	67671	13031	23608	2532
24670	55580	23927	105923	72153	21383	18985
17073	30178	26587	104997	54674	42363	66948
6552	22873	20929	47605	26249	28124	51409
20378	39596	18875	47949	43533	16743	22650
4948	31680	16258	45264	32383	56843	26369
9117	18724	14550	23025	27635	12557	18005
2835	13374	4201	59787	11354	52887	7585
10259	19506	12243	13077	24806	7630	13509
3697	21576	5753	49007	14030	13898	8712
7747	16607	7422	42871	22011	11271	15137
8106	13417	6278	35265	18210	6886	7645
7861	13955	6599	35791	18606	4800	19478
5428	11601	4615	30985	15512	7157	15258
3104	8290	5474	15659	11635	6148	10258
2483	9850	5079	41393	10738	7328	36115
5398	11011	3173	22523	23870	7963	2938
1975	7215	2028	30065	8975	23658	2871
6156	14455	7456	21998	15343	2634	13895
5195	7325	5344	8119	10887	6897	7480
602	2808	3748	16981	4214	16425	2518
5374	8404	7902	10130	15437	4288	4053
3316	8131	4344	13042	12622	3104	13764
6800	10459	4813	20057	13608	1778	4276
2173	6876	3633	7891	10504	6304	3427
2238	5921	2503	8198	5449	4144	2360
400	1536	507	13168	1871	3973	1637
5072	7830	7291	9354	14606	3939	3737
2375	4735	5382	6126	9559	1804	3723
4499	3898	2305	11683	5682	2563	3703
1807	4669	2725	9688	5919	3708	2694
1572	4467	2886	9818	4133	1843	6980
3962	3780	1108	6009	7634	2383	1265
3040	4636	2942	5063	7426	563	3486
2074	4725	2355	7359	5694	2415	5368
3507	4286	3015	3652	8135	945	3096
462	795	435	5207	1104	8344	842
3969	6665	2790	3222	6505	1015	1233

2660	4605	2146	6160	5870	861	3552
1795	3923	1942	5275	5637	1408	2688
1890	2452	1926	4581	3481	1159	4563
101	608	528	411	357	1106	262
1531	1660	560	10454	1263	3912	1640
577	2301	353	1459	1227	726	3186
1324	2938	1110	4484	4212	869	1227
997	1626	1168	2419	3051	573	1808
1007	2207	865	8034	3130	2145	3430
910	2869	901	4326	1250	894	1362
90	206	122	813	188	596	210
1265	1894	1268	2534	3161	456	751
1543	3472	985	3668	2730	1781	1108
641	2239	1399	3604	3307	980	2932
2086	1142	1136	695	4455	592	1721
892	1525	788	1996	2164	364	1111
1013	1840	524	3786	1964	1108	916
99	206	119	777	301	231	153
87	159	86	157	188	82	87
820	711	1147	1402	1428	847	2376
410	948	453	2657	1395	620	492
445	1045	449	2256	962	279	347
183	487	218	1561	626	1137	266
623	1575	572	2504	2208	480	563
125	676	1280	2557	1669	449	1158
780	1069	561	1379	1561	138	1184
576	636	801	1705	1845	568	1493
100	330	140	1884	326	1700	285
376	782	409	3583	1678	754	1266
760	2173	786	2036	2754	430	724
177	654	101	642	530	748	1106
601	1074	495	1963	1290	471	804
750	499	320	672	2716	279	724
441	690	278	1062	1337	379	540
494	727	366	980	1232	90	568
470	797	751	900	1439	377	399
601	820	391	1207	1255	185	1007
481	584	586	1104	1268	265	1139
37	445	91	692	243	161	400
418	946	336	962	1254	472	466
608	604	349	641	988	39	253
436	482	357	679	1179	115	1113
270	480	154	882	601	373	84
430	823	406	1734	1317	320	626
311	493	223	491	1415	640	403
400	556	0	1298	985	445	1575
14	42	35	23	50	8	15
369	883	293	784	656	198	442
319	492	301	769	719	59	168
144	209	122	729	266	118	77
220	539	120	964	600	216	136
284	379	220	924	686	159	866
255	377	326	639	474	235	653
274	467	223	784	816	235	639
51	94	43	997	155	367	68
261	303	99	547	740	11	279
283	631	254	1150	929	107	324
191	358	245	392	625	151	368
124	481	76	384	375	640	184

144	207	166	798	328	71	198
82	134	63	821	151	316	129
318	354	184	377	500	27	429
212	458	253	663	825	127	289
102	225	132	358	334	195	95
161	197	99	501	265	339	67
210	442	97	1018	383	246	214
221	443	173	610	697	121	432
324	260	113	422	546	23	230
68	146	94	485	245	182	102
134	410	250	439	587	50	136
78	235	178	322	315	200	164
180	220	114	270	504	418	156
109	251	82	386	268	113	148
109	251	82	386	268	113	148
126	148	98	245	281	35	79
120	235	184	186	266	29	87
103	264	140	405	465	42	394
32	241	24	248	77	199	101
120	213	96	806	222	284	168
21	31	19	70	51	14	18
81	151	115	238	369	89	202
151	261	162	132	317	66	106
167	294	109	354	309	20	207
134	171	77	190	393	47	197
34	159	115	319	298	107	286
37	72	50	266	357	34	111
139	241	123	299	299	22	162
228	130	57	116	357	60	44
170	162	82	285	406	21	130
57	195	52	224	118	39	66
94	187	69	306	220	103	189
47	184	38	211	193	263	63
84	134	109	218	127	61	74
102	212	111	144	235	152	48
109	167	94	131	306	22	70
77	167	90	161	131	85	90
131	159	107	213	387	39	107
77	118	38	112	143	18	35
78	183	100	111	266	93	75
4	15	6	37	48	38	10
49	88	123	112	120	30	109
50	141	87	210	225	55	152
80	165	70	351	262	33	59
63	68	89	130	241	40	116
108	118	67	109	182	11	74
36	109	15	362	150	117	91
29	64	42	151	133	58	17
26	89	7	141	27	107	47
101	123	68	186	248	60	114
69	121	25	49	93	27	7
38	57	76	78	174	18	126
35	77	15	171	139	62	54
80	68	32	44	189	4	35
87	100	52	120	163	22	67
55	77	29	123	106	35	49
4	10	3	38	13	13	10
33	90	45	143	181	16	80
53	91	45	110	150	19	76

30	84	24	278	102	42	62
48	113	35	290	207	35	72
48	77	73	78	100	27	74
62	128	59	149	192	21	88
37	50	13	37	52	8	34
28	60	16	89	45	24	8
52	52	78	77	74	60	61
36	119	39	79	248	44	22
13	63	42	122	51	64	75
44	104	54	164	122	26	146
60	120	31	164	151	16	75
48	85	30	319	103	47	44
56	98	43	76	78	8	60
12	49	18	14	25	22	83
38	84	20	259	106	49	67
70	81	24	127	90	10	52
21	50	16	29	40	10	43
5	67	0	79	174	21	118
18	43	14	203	50	111	27
10	45	13	16	16	19	92
28	40	14	235	66	37	14
22	39	21	57	85	9	91
32	59	35	70	88	29	48
17	42	14	192	48	104	26
12	58	6	38	18	37	95
94	33	50	49	96	20	24
22	49	29	154	142	31	52
48	58	75	2	104	0	26
21	9	1	116	16	14	2
47	69	25	139	93	29	71
39	53	52	87	69	49	141
27	34	33	96	109	11	78
108	108	79	48	130	20	30
34	54	5	142	20	80	31
20	17	24	111	33	25	34
34	46	25	63	53	4	19
10	53	51	147	94	24	69
53	88	19	70	114	26	31
28	12	23	61	87	4	39
29	45	21	100	73	18	42
18	43	39	118	73	25	167
12	28	10	81	30	15	10
14	80	8	23	35	19	25
33	42	35	83	107	19	25
32	75	22	72	80	3	63
76	18	14	36	78	8	15
29	43	33	32	51	30	34
42	68	42	55	124	20	58
27	71	28	107	114	19	63
4	40	5	84	17	81	32
40	26	37	36	37	3	20
24	46	27	123	38	6	37
34	46	24	123	88	12	63
38	45	30	100	97	20	35
32	56	19	97	57	27	51
0	1	3	13	4	9	0
22	52	13	72	72	23	27
46	58	1	65	19	4	33
22	38	36	30	47	3	28

26	61	0	50	71	11	12
11	30	12	52	29	3	24
9	56	3	25	10	10	20
29	29	24	32	35	16	29
15	52	24	45	64	40	33
10	57	26	18	37	5	16
22	25	11	21	61	8	23
16	34	22	37	62	14	11
11	13	9	81	27	9	7
20	25	26	59	33	8	41
2	2	2	21	8	9	1
20	17	16	30	70	3	9
24	41	23	43	39	5	16
13	34	25	24	90	3	17
31	26	33	34	77	4	9
8	42	5	70	54	15	22
21	24	6	55	51	23	4
21	31	22	61	82	3	32
1	23	4	12	12	10	31
14	42	11	55	40	3	9
3	8	2	117	24	35	7
23	31	17	64	57	11	16
23	31	17	64	57	11	16
2	16	4	8	13	6	31
13	32	28	29	39	3	18
21	16	15	32	62	6	13
7	44	4	14	20	13	15
18	42	20	57	52	5	18
13	36	8	48	57	6	9
3	19	19	51	18	17	76
10	19	33	19	46	11	9
11	32	13	30	38	12	9
14	34	11	100	37	19	20
6	8	10	19	24	9	14
37	25	11	10	33	13	2
12	22	14	28	51	10	6
0	0	2	4	3	0	1
14	32	14	28	39	8	7
10	19	12	69	31	14	14
15	36	19	38	54	22	24
10	5	21	17	20	5	21
10	17	11	63	29	11	12
16	20	8	13	40	4	26
16	26	14	6	39	11	9
9	31	14	46	28	3	11
8	18	23	19	32	9	8
1	0	0	1	1	1	0
16	33	20	12	29	12	8
16	11	17	13	10	1	15
12	20	13	16	28	7	11
6	18	6	8	21	4	16
6	20	5	54	13	26	11
1	1	1	5	0	45	0
9	15	11	12	51	4	13
7	19	1	41	14	10	5
53	0	0	0	0	0	0
23	31	13	14	36	7	10
7	3	12	48	29	8	8
9	9	8	25	20	5	9

2	8	6	4	13	3	9
5	17	6	23	17	11	15
3	15	6	11	5	15	14
10	17	17	24	42	2	8
15	8	9	16	21	3	12
10	13	15	20	35	4	12
7	18	1	36	14	8	5
2	19	4	13	1	5	13
1	0	2	5	3	19	3
8	20	4	19	6	8	4
8	23	17	44	27	13	5
12	19	7	24	19	2	10
3	13	7	30	11	7	12
14	25	7	18	19	4	2
2	16	3	5	8	11	15
7	13	7	41	10	16	8
12	6	3	23	11	13	10
12	20	7	22	35	2	3
12	16	7	15	19	4	12
4	15	1	11	5	0	4
3	14	2	4	9	4	13
4	27	10	20	26	3	12
6	12	10	22	24	5	15
8	6	7	4	19	5	8
7	9	3	51	11	6	9
8	24	5	24	29	7	3
11	18	7	8	23	0	12
4	11	7	9	16	2	13
4	18	4	13	32	2	10
11	18	7	24	26	1	9
6	10	8	28	12	14	22
3	8	2	29	2	28	1
1	6	5	38	10	2	4
6	4	2	24	12	10	21
5	11	7	21	25	6	8
5	10	6	8	15	1	10
11	11	2	17	5	3	26
7	18	9	25	18	1	8
7	14	12	7	14	6	7
5	9	10	13	23	9	9
5	7	3	28	9	7	5
6	13	3	32	17	10	24
0	20	0	4	10	4	11
15	5	12	20	35	3	7
4	17	3	9	10	8	4
4	12	0	15	9	1	7
5	15	13	10	14	3	7
3	10	4	9	7	10	9
5	3	6	6	13	0	4
2	19	1	14	21	6	6
5	11	3	3	11	2	3
3	3	2	0	2	0	1
9	5	8	6	13	1	5
3	9	3	20	11	13	2
8	10	11	5	6	4	7
7	8	2	37	17	9	16
4	14	3	22	27	0	17
5	4	3	27	27	2	3
3	6	7	21	5	4	8

5	7	3	12	14	7	12
11	3	7	8	20	4	13
4	9	4	42	20	6	10
7	7	3	12	15	1	5
1	11	11	20	10	6	8
11	3	7	8	15	1	9
6	6	3	10	12	1	1
1	1	0	6	3	7	4
4	7	6	15	8	6	6
2	17	6	13	16	4	8
8	7	4	15	10	0	9
8	7	2	18	10	3	5
2	5	9	8	7	6	17
1	3	0	19	1	1	1
7	12	3	14	4	7	2
3	11	1	14	6	2	6
7	16	0	13	11	2	3
4	4	7	12	17	5	7
3	6	0	17	2	0	3
2	12	3	8	13	0	3
4	7	2	22	8	4	7
6	4	2	20	12	1	7
4	18	6	6	23	7	12
4	5	3	10	7	0	7
5	6	9	4	3	0	14
1	5	1	9	6	4	5
4	2	1	38	5	7	1
2	1	2	18	3	1	2
7	7	3	5	6	6	21
3	11	6	10	13	1	4
1	4	5	8	14	0	23
2	14	6	12	11	5	7
2	13	7	11	8	2	10
5	11	6	6	11	0	3
2	4	3	12	4	1	2
0	2	2	0	3	2	7
4	5	1	18	13	1	5
4	3	1	8	12	5	5
6	8	3	7	5	0	3
2	10	1	9	4	1	7
3	8	2	10	6	4	2
5	19	7	6	17	0	5
1	7	1	4	3	1	5
14	3	2	10	13	2	6
5	11	5	7	16	1	5
3	2	2	13	7	2	3
5	10	4	9	16	0	1
3	2	1	8	5	1	3
2	5	0	9	5	4	0
5	3	0	41	3	0	1
1	4	0	3	5	1	6
2	16	3	2	1	1	0
2	4	1	12	4	1	0
4	3	0	7	16	0	7
4	5	3	5	8	3	2
0	0	1	8	5	8	1
4	10	2	8	4	2	9
5	5	5	8	5	1	3
7	2	0	5	11	0	0

2	0	2	8	4	0	3
4	4	3	5	2	2	2
2	6	0	19	4	10	3
0	5	1	8	0	4	2
4	5	1	3	3	5	5
2	2	1	2	9	5	1
1	2	6	5	4	0	8
2	7	3	6	7	4	2
4	4	3	2	5	0	8
0	4	3	6	3	2	7
3	2	1	19	2	4	3
0	3	1	2	4	2	0
0	0	0	1	0	1	0
2	8	3	9	6	1	0
1	2	2	3	3	1	2
0	3	0	6	3	2	5
1	3	6	2	8	1	5
3	0	4	3	4	0	3
0	0	0	0	0	0	1
0	5	0	1	2	12	1
0	4	0	2	1	2	1
0	6	0	11	3	2	0
7	3	2	7	15	1	2
4	3	1	1	17	3	0
0	3	2	2	7	0	4
0	1	0	8	0	2	0
3	11	5	2	4	0	1
2	2	1	22	7	4	6
5	2	3	3	11	0	5
3	5	2	10	6	2	2
2	0	1	3	1	1	0
4	1	1	12	7	0	1
1	1	0	8	7	1	6
0	4	1	2	8	3	1
0	4	1	10	5	3	0
2	3	1	5	5	1	5
0	7	0	3	5	0	5
3	1	2	6	1	0	2
4	7	1	9	4	1	2
1	3	1	1	0	1	3
0	0	1	0	0	0	0
5	4	1	10	6	3	3
1	3	1	9	3	1	4
1	7	0	9	7	0	10
1	4	2	9	6	0	7
1	5	1	2	0	0	1
0	1	1	13	3	5	0
4	2	1	4	9	0	0
2	5	1	6	5	0	1
1	2	3	3	7	1	1
0	1	1	5	5	5	6
1	2	2	5	4	1	1
0	1	1	2	1	0	2
2	1	4	6	3	2	5
3	5	1	6	7	0	1
2	1	1	7	5	3	2
1	1	2	1	3	0	3
2	1	1	0	2	4	1
1	1	2	0	2	0	2

3	1	1	0	3	0	0
1	2	1	1	4	2	2
2	3	4	5	4	3	2
1	4	0	4	2	0	1
1	2	2	5	10	0	0
0	0	0	2	1	2	1
1	4	0	0	3	0	2
3	3	0	4	2	0	0
5	4	2	5	7	3	6
1	2	0	2	1	3	0
10	3	2	6	2	0	2
0	2	0	1	3	0	1
0	0	1	4	0	2	1
3	0	2	1	5	0	3
3	2	1	4	4	2	1
2	2	2	2	3	2	0
1	3	2	6	8	1	3
0	1	0	1	1	1	0
1	0	0	5	1	5	1
0	0	1	1	1	1	0
3	1	1	3	0	0	0
0	0	0	2	0	2	0
0	0	1	6	0	2	0
1	1	3	0	5	0	5
0	1	0	4	7	2	3
4	2	0	6	2	1	1
3	1	0	7	4	4	3
2	1	2	3	9	1	0
0	0	0	2	0	0	1
1	0	0	1	0	1	1
1	2	2	3	0	0	0
0	2	1	2	4	0	1
2	3	0	2	4	0	2
2	2	0	2	14	0	0
0	3	1	3	4	2	0
1	1	1	7	2	1	1
1	1	1	1	3	0	3
0	3	0	2	1	3	0
1	3	1	2	6	1	0
0	1	0	0	5	0	2
0	0	0	2	1	0	1
1	2	1	0	3	0	1
2	0	2	5	7	0	0
2	1	0	2	4	0	1
4	2	1	5	6	2	5
0	2	1	4	2	2	3
0	2	1	2	0	2	1
0	1	4	0	4	3	1
3	1	1	2	1	0	1
2	0	1	1	3	0	1
2	3	1	3	3	0	0
0	0	0	2	2	13	0
0	0	1	2	0	0	0
0	9	3	1	0	1	3
0	2	2	4	0	0	3
1	1	1	3	0	1	2
1	2	1	1	2	0	0
2	2	1	5	1	2	2
1	2	0	7	2	0	2

1	6	2	2	2	0	1
1	2	0	1	0	0	2
0	1	1	7	4	1	0
0	0	1	3	1	0	3
1	2	2	3	4	1	0
1	1	0	0	0	1	0
0	0	1	0	2	1	0
1	0	1	0	6	0	0
3	1	1	1	1	0	3
2	0	0	0	0	1	1
0	2	1	4	1	1	1
0	1	0	6	2	0	1
0	0	1	0	0	0	0
E_7	E_8	E_9	F_40	F_41	F_42	F_44
CHR_P	CHR_P	CHR_P	CHR_P	CHR_P	CHR_P	CHR_P
1172863	906893	0	1936737	2227935	3040120	1921719
16	10	0	11	14	23	14
-1.08	-2.42	-4.49	-2.08	-1.63	0.36	-1.63

0.92	0.85	0.10	0.04
3	5	23	27
1	1	1	1
92	93	94	95
27	28	29	30
F_44	F_9	G_10	G_9
CHR_P	CHR_P	CHR_P	CHR_P
2580829	1797912	999373	336028
F_44	F_9	G_10	G_9
1338069	652973	273186	110265
219682	122940	215390	26137
145667	125931	14419	12206
146527	186789	94406	16565
146024	206210	54647	23910
23965	18035	33221	13330
54262	49594	58222	18835
2532	2158	5400	2740
18985	20381	24629	8586
66948	27101	30790	21344
51409	22088	14122	13068
22650	38405	10022	4659
26369	23357	12687	5899
18005	28494	9022	4547
7585	4309	7370	1032
13509	30687	2256	1015
8712	3110	7239	1515
15137	10154	4100	1477
7645	6488	7688	4923

19478	11759	8333	2437
15258	13778	2678	1067
10258	7317	5535	2832
36115	9239	10632	2095
2938	3395	4481	1400
2871	2070	6408	1265
13895	9703	2308	978
7480	9474	2078	1197
2518	1255	4845	1918
4053	4359	3250	924
13764	9512	6491	2815
4276	4603	2770	869
3427	12922	3134	588
2360	3220	1694	817
1637	1446	727	643
3737	4101	2969	836
3723	5538	3098	1501
3703	1877	4939	1697
2694	1720	4607	1218
6980	2197	1705	513
1265	8631	643	269
3486	7199	727	289
5368	7016	1233	671
3096	3313	1092	508
842	360	945	324
1233	2024	714	802
3552	3764	949	354
2688	2927	2751	1307
4563	2903	881	384
262	327	261	71
1640	981	551	116
3186	2777	666	582
1227	1077	852	255
1808	1598	871	640
3430	1413	900	66
1362	3332	947	355
210	143	97	88
751	1221	787	241
1108	2006	703	130
2932	1472	581	339
1721	2035	991	121
1111	1181	576	315
916	1186	720	152
153	69	254	135
87	100	35	18
2376	1183	538	156
492	442	577	193
347	444	187	479
266	297	384	67
563	816	222	80
1158	271	1598	459
1184	1228	272	239
1493	1379	153	72
285	130	829	122
1266	602	377	174
724	629	336	141
1106	1819	168	157
804	1044	896	349
724	766	699	209

540	1301	188	65
568	462	283	188
399	606	236	100
1007	1014	207	88
1139	683	295	88
400	579	46	292
466	549	626	110
253	747	128	141
1113	873	250	74
84	84	153	52
626	354	192	83
403	1253	261	24
1575	1101	237	84
15	9	18	13
442	589	127	25
168	278	152	48
77	67	67	39
136	198	80	26
866	664	149	24
653	469	136	52
639	587	145	102
68	45	119	30
279	246	51	87
324	423	121	51
368	515	53	77
184	226	493	95
198	115	95	68
129	68	90	27
429	357	71	42
289	211	228	89
95	174	62	23
67	106	168	62
214	309	124	31
432	351	83	33
230	206	91	75
102	76	210	30
136	301	29	14
164	411	102	49
156	110	300	21
148	153	120	54
148	153	120	54
79	114	79	73
87	244	55	94
394	208	48	22
101	288	46	20
168	139	95	19
18	39	4	0
202	312	44	17
106	179	77	68
207	258	22	12
197	232	78	16
286	305	73	1
111	78	193	131
162	171	38	26
44	109	105	47
130	187	21	21
66	135	41	28
189	139	72	53
63	122	72	32

74	98	35	29
48	208	25	22
70	209	23	14
90	195	135	18
107	109	28	26
35	248	7	4
75	196	54	5
10	16	11	5
109	101	37	15
152	134	37	6
59	72	37	11
116	70	53	21
74	83	24	28
91	26	28	24
17	32	36	16
47	101	17	18
114	148	38	12
7	90	6	2
126	55	32	29
54	60	102	12
35	68	21	52
67	94	17	16
49	82	29	11
10	9	12	1
80	66	23	26
76	100	29	20
62	37	27	5
72	38	25	6
74	84	33	8
88	81	37	10
34	58	21	171
8	43	9	4
61	116	36	10
22	46	29	14
75	48	31	23
146	94	43	23
75	53	45	14
44	24	40	5
60	136	16	4
83	66	17	33
67	55	31	13
52	45	16	20
43	91	5	13
118	76	21	16
27	18	24	6
92	92	7	16
14	17	49	11
91	73	16	12
48	101	13	4
26	17	23	6
95	81	4	11
24	68	18	19
52	55	38	11
26	73	2	0
2	6	29	4
71	50	15	0
141	106	26	0
78	26	41	23
30	46	40	4

31	19	16	1
34	51	23	8
19	45	10	14
69	46	8	5
31	125	28	0
39	17	7	7
42	139	17	4
167	62	33	15
10	9	10	6
25	153	9	3
25	80	29	14
63	49	23	6
15	12	23	8
34	27	15	11
58	77	8	4
63	41	13	8
32	31	7	5
20	40	13	6
37	34	13	9
63	44	27	1
35	41	14	3
51	43	18	6
0	0	0	0
27	35	25	3
33	1	40	3
28	23	9	14
12	51	4	7
24	26	10	21
20	43	5	0
29	66	4	2
33	47	7	3
16	38	4	3
23	32	9	7
11	17	6	4
7	7	21	7
41	30	10	5
1	1	2	1
9	33	4	2
16	28	6	3
17	19	9	13
9	18	4	3
22	27	9	3
4	16	5	2
32	36	5	7
31	17	6	8
9	28	5	7
7	4	19	1
16	24	12	2
16	24	12	2
31	41	1	1
18	40	12	2
13	18	12	5
15	35	3	4
18	8	13	3
9	24	7	2
76	8	5	6
9	28	10	2
9	54	2	0
20	7	12	3

14	16	41	1
2	37	3	2
6	12	5	2
1	2	1	0
7	25	7	4
14	7	15	3
24	41	5	1
21	22	10	4
12	6	14	3
26	42	5	4
9	28	6	0
11	19	4	7
8	35	6	4
0	1	0	2
8	35	0	2
15	14	7	3
11	13	4	0
16	16	1	3
11	13	11	1
0	0	1	0
13	10	7	2
5	9	6	2
0	1	53	0
10	26	5	2
8	16	4	0
9	21	8	1
9	36	2	3
15	31	8	2
14	24	3	0
8	15	1	5
12	15	7	2
12	12	7	0
5	8	5	2
13	26	0	6
3	1	8	2
4	5	4	1
5	5	12	5
10	26	2	1
12	20	6	6
2	23	0	0
15	22	2	3
8	8	2	2
10	10	6	2
3	11	6	5
12	27	3	1
4	3	2	0
13	31	4	1
12	34	9	1
15	11	2	0
8	14	4	1
9	15	7	2
3	28	0	0
12	13	1	1
13	14	1	3
10	13	5	2
9	1	0	2
22	15	9	6
1	1	1	0
4	0	4	1

21	4	6	6
8	4	10	0
10	8	4	5
26	23	1	3
8	11	2	1
7	27	0	3
9	23	1	3
5	3	4	4
24	13	3	0
11	17	1	1
7	23	3	1
4	14	2	1
7	4	4	6
7	16	2	0
9	3	3	1
4	7	4	5
6	14	2	0
3	12	1	0
1	3	1	2
5	14	3	2
2	0	5	1
7	14	9	3
16	26	0	0
17	17	0	0
3	0	6	3
8	9	0	0
12	0	7	0
13	15	2	0
10	13	1	0
5	10	0	0
8	15	4	1
9	12	4	0
1	8	7	3
4	1	3	5
6	3	8	1
8	12	2	0
9	7	2	0
5	17	0	2
17	6	1	1
1	1	0	0
2	11	0	1
6	3	2	1
3	6	0	0
7	7	2	0
3	2	1	2
3	2	3	0
7	6	2	1
7	4	2	0
12	6	2	1
7	10	3	2
14	4	4	0
5	8	1	1
1	0	0	0
2	1	1	0
21	5	3	1
4	7	2	1
23	2	1	2
7	9	3	0
10	4	2	0

3	12	0	1
2	2	0	0
7	13	0	1
5	4	1	0
5	10	1	0
3	7	1	0
7	6	2	0
2	7	4	0
5	5	2	2
5	9	1	0
6	7	0	4
5	6	3	2
3	3	0	0
1	10	0	0
3	1	4	0
0	2	0	1
1	4	0	0
6	6	2	0
0	7	1	0
0	1	1	0
7	6	1	0
2	3	0	0
1	0	5	1
9	5	2	0
3	10	1	0
0	8	1	0
3	2	1	2
2	8	2	0
3	1	0	0
2	4	0	1
5	8	0	1
1	3	1	0
8	6	0	0
2	4	1	0
8	11	2	0
7	2	2	0
3	0	2	0
0	0	0	0
0	0	0	0
0	3	1	0
2	0	0	0
5	3	0	0
5	4	2	1
3	4	1	1
1	0	0	0
1	1	0	1
1	4	1	3
0	0	0	1
2	8	3	0
0	8	1	0
4	3	0	0
0	0	0	0
1	8	0	1
6	1	4	0
5	5	1	0
2	0	1	0
0	0	0	0
1	4	1	0
6	5	0	0

1	9	1	1
0	5	2	0
5	3	2	1
5	2	1	0
2	0	1	2
2	6	0	0
3	2	0	0
0	2	0	0
3	2	0	0
4	7	0	1
10	6	2	0
7	2	0	0
1	4	1	0
0	2	0	0
0	3	0	1
1	5	1	1
1	7	2	0
6	2	1	0
1	0	2	0
2	0	0	0
5	1	3	0
1	3	0	0
2	2	0	1
3	0	1	1
1	2	2	0
2	1	2	1
0	6	1	1
2	3	1	1
2	2	3	0
1	1	0	1
0	2	0	2
1	0	0	0
2	5	0	2
0	2	0	0
6	3	0	0
0	0	0	1
2	3	0	0
1	4	0	1
1	0	1	0
3	1	3	0
1	1	1	1
0	1	0	1
3	3	1	0
0	3	3	0
1	0	0	0
0	0	0	1
0	1	0	0
0	0	0	0
0	0	0	0
5	1	1	0
3	5	0	0
1	3	1	0
3	0	0	0
0	2	0	1
1	1	0	0
1	1	0	0
0	2	0	1
1	5	0	0
2	3	0	0

0	0	0	0
0	3	0	0
1	1	0	1
3	5	0	2
0	3	1	0
0	2	0	0
2	8	0	0
1	2	1	0
1	2	3	0
0	5	0	0
1	1	1	0
5	0	0	0
3	4	3	1
1	6	0	0
1	6	0	2
1	2	0	0
1	4	0	1
0	1	0	0
0	0	0	0
0	0	0	2
3	0	0	1
3	0	1	1
2	2	0	0
0	3	0	1
2	1	0	0
2	3	0	0
1	2	0	0
2	1	0	0
0	2	0	0
3	3	0	0
0	1	1	0
0	2	0	0
0	2	0	0
0	2	1	0
3	1	0	1
1	8	0	0
1	2	1	0
1	0	0	0

0.92	0.85	0.10	0.04
3	5	23	27
1	1	1	1
92	93	94	95
27	28	29	30
F_44	F_9	G_10	G_9
CHR_P	CHR_P	CHR_P	CHR_P
2580829	1797912	999373	336028
CHR_P	CHR_P	CHR_P	CHR_P
1338069	652973	273186	110265
219682	122940	215390	26137
145667	125931	14419	12206
146527	186789	94406	16565
146024	206210	54647	23910

23965	18035	33221	13330
54262	49594	58222	18835
2532	2158	5400	2740
18985	20381	24629	8586
66948	27101	30790	21344
51409	22088	14122	13068
22650	38405	10022	4659
26369	23357	12687	5899
18005	28494	9022	4547
7585	4309	7370	1032
13509	30687	2256	1015
8712	3110	7239	1515
15137	10154	4100	1477
7645	6488	7688	4923
19478	11759	8333	2437
15258	13778	2678	1067
10258	7317	5535	2832
36115	9239	10632	2095
2938	3395	4481	1400
2871	2070	6408	1265
13895	9703	2308	978
7480	9474	2078	1197
2518	1255	4845	1918
4053	4359	3250	924
13764	9512	6491	2815
4276	4603	2770	869
3427	12922	3134	588
2360	3220	1694	817
1637	1446	727	643
3737	4101	2969	836
3723	5538	3098	1501
3703	1877	4939	1697
2694	1720	4607	1218
6980	2197	1705	513
1265	8631	643	269
3486	7199	727	289
5368	7016	1233	671
3096	3313	1092	508
842	360	945	324
1233	2024	714	802
3552	3764	949	354
2688	2927	2751	1307
4563	2903	881	384
262	327	261	71
1640	981	551	116
3186	2777	666	582
1227	1077	852	255
1808	1598	871	640
3430	1413	900	66
1362	3332	947	355
210	143	97	88
751	1221	787	241
1108	2006	703	130
2932	1472	581	339
1721	2035	991	121
1111	1181	576	315
916	1186	720	152
153	69	254	135
87	100	35	18

2376	1183	538	156
492	442	577	193
347	444	187	479
266	297	384	67
563	816	222	80
1158	271	1598	459
1184	1228	272	239
1493	1379	153	72
285	130	829	122
1266	602	377	174
724	629	336	141
1106	1819	168	157
804	1044	896	349
724	766	699	209
540	1301	188	65
568	462	283	188
399	606	236	100
1007	1014	207	88
1139	683	295	88
400	579	46	292
466	549	626	110
253	747	128	141
1113	873	250	74
84	84	153	52
626	354	192	83
403	1253	261	24
1575	1101	237	84
15	9	18	13
442	589	127	25
168	278	152	48
77	67	67	39
136	198	80	26
866	664	149	24
653	469	136	52
639	587	145	102
68	45	119	30
279	246	51	87
324	423	121	51
368	515	53	77
184	226	493	95
198	115	95	68
129	68	90	27
429	357	71	42
289	211	228	89
95	174	62	23
67	106	168	62
214	309	124	31
432	351	83	33
230	206	91	75
102	76	210	30
136	301	29	14
164	411	102	49
156	110	300	21
148	153	120	54
148	153	120	54
79	114	79	73
87	244	55	94
394	208	48	22
101	288	46	20

168	139	95	19
18	39	4	0
202	312	44	17
106	179	77	68
207	258	22	12
197	232	78	16
286	305	73	1
111	78	193	131
162	171	38	26
44	109	105	47
130	187	21	21
66	135	41	28
189	139	72	53
63	122	72	32
74	98	35	29
48	208	25	22
70	209	23	14
90	195	135	18
107	109	28	26
35	248	7	4
75	196	54	5
10	16	11	5
109	101	37	15
152	134	37	6
59	72	37	11
116	70	53	21
74	83	24	28
91	26	28	24
17	32	36	16
47	101	17	18
114	148	38	12
7	90	6	2
126	55	32	29
54	60	102	12
35	68	21	52
67	94	17	16
49	82	29	11
10	9	12	1
80	66	23	26
76	100	29	20
62	37	27	5
72	38	25	6
74	84	33	8
88	81	37	10
34	58	21	171
8	43	9	4
61	116	36	10
22	46	29	14
75	48	31	23
146	94	43	23
75	53	45	14
44	24	40	5
60	136	16	4
83	66	17	33
67	55	31	13
52	45	16	20
43	91	5	13
118	76	21	16
27	18	24	6

92	92	7	16
14	17	49	11
91	73	16	12
48	101	13	4
26	17	23	6
95	81	4	11
24	68	18	19
52	55	38	11
26	73	2	0
2	6	29	4
71	50	15	0
141	106	26	0
78	26	41	23
30	46	40	4
31	19	16	1
34	51	23	8
19	45	10	14
69	46	8	5
31	125	28	0
39	17	7	7
42	139	17	4
167	62	33	15
10	9	10	6
25	153	9	3
25	80	29	14
63	49	23	6
15	12	23	8
34	27	15	11
58	77	8	4
63	41	13	8
32	31	7	5
20	40	13	6
37	34	13	9
63	44	27	1
35	41	14	3
51	43	18	6
0	0	0	0
27	35	25	3
33	1	40	3
28	23	9	14
12	51	4	7
24	26	10	21
20	43	5	0
29	66	4	2
33	47	7	3
16	38	4	3
23	32	9	7
11	17	6	4
7	7	21	7
41	30	10	5
1	1	2	1
9	33	4	2
16	28	6	3
17	19	9	13
9	18	4	3
22	27	9	3
4	16	5	2
32	36	5	7
31	17	6	8

9	28	5	7
7	4	19	1
16	24	12	2
16	24	12	2
31	41	1	1
18	40	12	2
13	18	12	5
15	35	3	4
18	8	13	3
9	24	7	2
76	8	5	6
9	28	10	2
9	54	2	0
20	7	12	3
14	16	41	1
2	37	3	2
6	12	5	2
1	2	1	0
7	25	7	4
14	7	15	3
24	41	5	1
21	22	10	4
12	6	14	3
26	42	5	4
9	28	6	0
11	19	4	7
8	35	6	4
0	1	0	2
8	35	0	2
15	14	7	3
11	13	4	0
16	16	1	3
11	13	11	1
0	0	1	0
13	10	7	2
5	9	6	2
0	1	53	0
10	26	5	2
8	16	4	0
9	21	8	1
9	36	2	3
15	31	8	2
14	24	3	0
8	15	1	5
12	15	7	2
12	12	7	0
5	8	5	2
13	26	0	6
3	1	8	2
4	5	4	1
5	5	12	5
10	26	2	1
12	20	6	6
2	23	0	0
15	22	2	3
8	8	2	2
10	10	6	2
3	11	6	5
12	27	3	1

4	3	2	0
13	31	4	1
12	34	9	1
15	11	2	0
8	14	4	1
9	15	7	2
3	28	0	0
12	13	1	1
13	14	1	3
10	13	5	2
9	1	0	2
22	15	9	6
1	1	1	0
4	0	4	1
21	4	6	6
8	4	10	0
10	8	4	5
26	23	1	3
8	11	2	1
7	27	0	3
9	23	1	3
5	3	4	4
24	13	3	0
11	17	1	1
7	23	3	1
4	14	2	1
7	4	4	6
7	16	2	0
9	3	3	1
4	7	4	5
6	14	2	0
3	12	1	0
1	3	1	2
5	14	3	2
2	0	5	1
7	14	9	3
16	26	0	0
17	17	0	0
3	0	6	3
8	9	0	0
12	0	7	0
13	15	2	0
10	13	1	0
5	10	0	0
8	15	4	1
9	12	4	0
1	8	7	3
4	1	3	5
6	3	8	1
8	12	2	0
9	7	2	0
5	17	0	2
17	6	1	1
1	1	0	0
2	11	0	1
6	3	2	1
3	6	0	0
7	7	2	0
3	2	1	2

3	2	3	0
7	6	2	1
7	4	2	0
12	6	2	1
7	10	3	2
14	4	4	0
5	8	1	1
1	0	0	0
2	1	1	0
21	5	3	1
4	7	2	1
23	2	1	2
7	9	3	0
10	4	2	0
3	12	0	1
2	2	0	0
7	13	0	1
5	4	1	0
5	10	1	0
3	7	1	0
7	6	2	0
2	7	4	0
5	5	2	2
5	9	1	0
6	7	0	4
5	6	3	2
3	3	0	0
1	10	0	0
3	1	4	0
0	2	0	1
1	4	0	0
6	6	2	0
0	7	1	0
0	1	1	0
7	6	1	0
2	3	0	0
1	0	5	1
9	5	2	0
3	10	1	0
0	8	1	0
3	2	1	2
2	8	2	0
3	1	0	0
2	4	0	1
5	8	0	1
1	3	1	0
8	6	0	0
2	4	1	0
8	11	2	0
7	2	2	0
3	0	2	0
0	0	0	0
0	0	0	0
0	3	1	0
2	0	0	0
5	3	0	0
5	4	2	1
3	4	1	1
1	0	0	0

1	1	0	1
1	4	1	3
0	0	0	1
2	8	3	0
0	8	1	0
4	3	0	0
0	0	0	0
1	8	0	1
6	1	4	0
5	5	1	0
2	0	1	0
0	0	0	0
1	4	1	0
6	5	0	0
1	9	1	1
0	5	2	0
5	3	2	1
5	2	1	0
2	0	1	2
2	6	0	0
3	2	0	0
0	2	0	0
3	2	0	0
4	7	0	1
10	6	2	0
7	2	0	0
1	4	1	0
0	2	0	0
0	3	0	1
1	5	1	1
1	7	2	0
6	2	1	0
1	0	2	0
2	0	0	0
5	1	3	0
1	3	0	0
2	2	0	1
3	0	1	1
1	2	2	0
2	1	2	1
0	6	1	1
2	3	1	1
2	2	3	0
1	1	0	1
0	2	0	2
1	0	0	0
2	5	0	2
0	2	0	0
6	3	0	0
0	0	0	1
2	3	0	0
1	4	0	1
1	0	1	0
3	1	3	0
1	1	1	1
0	1	0	1
3	3	1	0
0	3	3	0
1	0	0	0

0	0	0	1
0	1	0	0
0	0	0	0
0	0	0	0
5	1	1	0
3	5	0	0
1	3	1	0
3	0	0	0
0	2	0	1
1	1	0	0
1	1	0	0
0	2	0	1
1	5	0	0
2	3	0	0
0	0	0	0
0	3	0	0
1	1	0	1
3	5	0	2
0	3	1	0
0	2	0	0
2	8	0	0
1	2	1	0
1	2	3	0
0	5	0	0
1	1	1	0
5	0	0	0
3	4	3	1
1	6	0	0
1	6	0	2
1	2	0	0
1	4	0	1
0	1	0	0
0	0	0	0
0	0	0	2
3	0	0	1
3	0	1	1
2	2	0	0
0	3	0	1
2	1	0	0
2	3	0	0
1	2	0	0
2	1	0	0
0	2	0	0
3	3	0	0
0	1	1	0
0	2	0	0
0	2	0	0
0	2	1	0
3	1	0	1
1	8	0	0
1	2	1	0
1	0	0	0
82077	55369	31195	10366
243271	124837	61898	20341
395	385	421	451
CHR_P	CHR_P	CHR_P	CHR_P
CHR_P	CHR_P	CHR_P	CHR_P

92	93	94	95
27	28	29	30
CHR_P	CHR_P	CHR_P	CHR_P
F_44	F_9	G_10	G_9
2580829	1797912	999373	336028
CHR_P	CHR_P	CHR_P	CHR_P
16.30	11.79	8.76	10.64
2.68	2.22	6.90	2.52
1.77	2.27	0.46	1.18
1.79	3.37	3.03	1.60
1.78	3.72	1.75	2.31
0.29	0.33	1.06	1.29
0.66	0.90	1.87	1.82
0.03	0.04	0.17	0.26
0.23	0.37	0.79	0.83
0.82	0.49	0.99	2.06
0.63	0.40	0.45	1.26
0.28	0.69	0.32	0.45
0.32	0.42	0.41	0.57
0.22	0.51	0.29	0.44
0.09	0.08	0.24	0.10
0.16	0.55	0.07	0.10
0.11	0.06	0.23	0.15
0.18	0.18	0.13	0.14
0.09	0.12	0.25	0.47
0.24	0.21	0.27	0.24
0.19	0.25	0.09	0.10
0.12	0.13	0.18	0.27
0.44	0.17	0.34	0.20
0.04	0.06	0.14	0.14
0.03	0.04	0.21	0.12
0.17	0.18	0.07	0.09
0.09	0.17	0.07	0.12
0.03	0.02	0.16	0.19
0.05	0.08	0.10	0.09
0.17	0.17	0.21	0.27
0.05	0.08	0.09	0.08
0.04	0.23	0.10	0.06
0.03	0.06	0.05	0.08
0.02	0.03	0.02	0.06
0.05	0.07	0.10	0.08
0.05	0.10	0.10	0.14
0.05	0.03	0.16	0.16
0.03	0.03	0.15	0.12
0.09	0.04	0.05	0.05
0.02	0.16	0.02	0.03
0.04	0.13	0.02	0.03
0.07	0.13	0.04	0.06
0.04	0.06	0.04	0.05
0.01	0.01	0.03	0.03
0.02	0.04	0.02	0.08
0.04	0.07	0.03	0.03
0.03	0.05	0.09	0.13
0.06	0.05	0.03	0.04
0.00	0.01	0.01	0.01
0.02	0.02	0.02	0.01
0.04	0.05	0.02	0.06
0.01	0.02	0.03	0.02

0.02	0.03	0.03	0.06
0.04	0.03	0.03	0.01
0.02	0.06	0.03	0.03
0.00	0.00	0.00	0.01
0.01	0.02	0.03	0.02
0.01	0.04	0.02	0.01
0.04	0.03	0.02	0.03
0.02	0.04	0.03	0.01
0.01	0.02	0.02	0.03
0.01	0.02	0.02	0.01
0.00	0.00	0.01	0.01
0.00	0.00	0.00	0.00
0.03	0.02	0.02	0.02
0.01	0.01	0.02	0.02
0.00	0.01	0.01	0.05
0.00	0.01	0.01	0.01
0.01	0.01	0.01	0.01
0.01	0.00	0.05	0.04
0.01	0.02	0.01	0.02
0.02	0.02	0.00	0.01
0.00	0.00	0.03	0.01
0.02	0.01	0.01	0.02
0.01	0.01	0.01	0.01
0.01	0.03	0.01	0.02
0.01	0.02	0.03	0.03
0.01	0.01	0.02	0.02
0.01	0.02	0.01	0.01
0.01	0.01	0.01	0.02
0.00	0.01	0.01	0.01
0.01	0.02	0.01	0.01
0.01	0.01	0.01	0.01
0.00	0.01	0.00	0.03
0.01	0.01	0.02	0.01
0.00	0.01	0.00	0.01
0.01	0.02	0.01	0.01
0.00	0.00	0.00	0.01
0.01	0.01	0.01	0.01
0.00	0.02	0.01	0.00
0.02	0.02	0.01	0.01
0.00	0.00	0.00	0.00
0.01	0.01	0.00	0.00
0.00	0.01	0.00	0.00
0.00	0.00	0.00	0.00
0.00	0.00	0.00	0.00
0.01	0.01	0.00	0.00
0.01	0.01	0.00	0.01
0.01	0.01	0.00	0.01
0.00	0.00	0.00	0.00
0.00	0.00	0.00	0.01
0.00	0.01	0.00	0.00
0.00	0.01	0.00	0.01
0.00	0.00	0.02	0.01
0.00	0.00	0.00	0.01
0.00	0.00	0.00	0.00
0.01	0.01	0.00	0.00
0.00	0.00	0.01	0.01
0.00	0.00	0.00	0.00
0.00	0.00	0.01	0.01
0.00	0.01	0.00	0.00

0.01	0.01	0.00	0.00
0.00	0.00	0.00	0.01
0.00	0.00	0.01	0.00
0.00	0.01	0.00	0.00
0.00	0.01	0.00	0.00
0.00	0.00	0.01	0.00
0.00	0.00	0.00	0.01
0.00	0.00	0.00	0.01
0.00	0.00	0.00	0.01
0.00	0.00	0.00	0.01
0.00	0.00	0.00	0.00
0.00	0.01	0.00	0.00
0.00	0.00	0.00	0.00
0.00	0.00	0.00	0.00
0.00	0.01	0.00	0.00
0.00	0.00	0.00	0.01
0.00	0.00	0.00	0.00
0.00	0.00	0.00	0.00
0.00	0.01	0.00	0.00
0.00	0.00	0.01	0.01
0.00	0.00	0.00	0.00
0.00	0.00	0.00	0.00
0.00	0.00	0.00	0.00
0.00	0.00	0.00	0.00
0.00	0.00	0.00	0.01
0.00	0.00	0.00	0.00
0.00	0.00	0.00	0.00
0.00	0.00	0.00	0.00
0.00	0.00	0.00	0.00
0.00	0.00	0.00	0.00
0.00	0.00	0.00	0.00
0.00	0.00	0.00	0.00
0.00	0.00	0.00	0.00
0.00	0.00	0.00	0.00
0.00	0.00	0.00	0.00
0.00	0.00	0.00	0.00
0.00	0.00	0.00	0.00
0.00	0.00	0.00	0.00
0.00	0.00	0.00	0.00
0.00	0.00	0.00	0.00
0.00	0.00	0.00	0.00
0.00	0.00	0.00	0.00
0.00	0.00	0.00	0.00
0.00	0.00	0.00	0.00
0.00	0.00	0.00	0.00
0.00	0.00	0.00	0.00
0.00	0.00	0.00	0.01
0.00	0.00	0.00	0.00
0.00	0.00	0.00	0.00
0.00	0.00	0.00	0.00
0.00	0.00	0.00	0.00
0.00	0.00	0.00	0.00
0.00	0.00	0.00	0.00
0.00	0.00	0.00	0.00
0.00	0.00	0.00	0.00
0.00	0.00	0.00	0.00
0.00	0.00	0.00	0.02
0.00	0.00	0.00	0.00
0.00	0.00	0.00	0.00

0.00	0.00	0.00	0.00
0.00	0.00	0.00	0.00
0.00	0.00	0.00	0.00
0.00	0.00	0.00	0.00
0.00	0.00	0.00	0.00
0.00	0.00	0.00	0.00
0.00	0.00	0.00	0.00
0.00	0.00	0.00	0.00
0.00	0.00	0.00	0.00
0.00	0.00	0.00	0.00
0.00	0.00	0.00	0.00
0.00	0.00	0.00	0.00
0.00	0.00	0.00	0.00
0.00	0.00	0.00	0.00
0.00	0.00	0.00	0.00
0.00	0.00	0.00	0.00
0.00	0.00	0.00	0.00
0.00	0.00	0.00	0.00
0.00	0.00	0.00	0.00
0.00	0.00	0.00	0.00
0.00	0.00	0.00	0.00
0.00	0.00	0.00	0.00
0.00	0.00	0.00	0.00
0.00	0.00	0.00	0.00
0.00	0.00	0.00	0.00
0.00	0.00	0.00	0.00
0.00	0.00	0.00	0.00
0.00	0.00	0.00	0.00
0.00	0.00	0.00	0.00
0.00	0.00	0.00	0.00
0.00	0.00	0.00	0.00
0.00	0.00	0.00	0.00
0.00	0.00	0.00	0.00
0.00	0.00	0.00	0.00
0.00	0.00	0.00	0.00
0.00	0.00	0.00	0.00
0.00	0.00	0.00	0.00
0.00	0.00	0.00	0.00
0.00	0.00	0.00	0.00
0.00	0.00	0.00	0.00
0.00	0.00	0.00	0.00
0.00	0.00	0.00	0.00
0.00	0.00	0.00	0.00
0.00	0.00	0.00	0.00
0.00	0.00	0.00	0.00
0.00	0.00	0.00	0.00
0.00	0.00	0.00	0.00
0.00	0.00	0.00	0.00
0.00	0.00	0.00	0.00
0.00	0.00	0.00	0.00
0.00	0.00	0.00	0.00
0.00	0.00	0.00	0.00
0.00	0.00	0.00	0.00
0.00	0.00	0.00	0.00
0.00	0.00	0.00	0.00
0.00	0.00	0.00	0.00
0.00	0.00	0.00	0.00
0.00	0.00	0.00	0.00
0.00	0.00	0.00	0.00

0.00	0.00	0.00	0.00
0.00	0.00	0.00	0.00
0.00	0.00	0.00	0.00
0.00	0.00	0.00	0.00
0.00	0.00	0.00	0.00
0.00	0.00	0.00	0.00
0.00	0.00	0.00	0.00
0.00	0.00	0.00	0.00
0.00	0.00	0.00	0.00
0.00	0.00	0.00	0.00
0.00	0.00	0.00	0.00
0.00	0.00	0.00	0.00
0.00	0.00	0.00	0.00
0.00	0.00	0.00	0.00
0.00	0.00	0.00	0.00
0.00	0.00	0.00	0.00
0.00	0.00	0.00	0.00
0.00	0.00	0.00	0.00
0.00	0.00	0.00	0.00
0.00	0.00	0.00	0.00
0.00	0.00	0.00	0.00
0.00	0.00	0.00	0.00
0.00	0.00	0.00	0.00
0.00	0.00	0.00	0.00
0.00	0.00	0.00	0.00
0.00	0.00	0.00	0.00
0.00	0.00	0.00	0.00
0.00	0.00	0.00	0.00
0.00	0.00	0.00	0.00
0.00	0.00	0.00	0.00

92	93	94	95		
28	29	30	31		
CHR_P	CHR_P	CHR_P	CHR_P		
F_44	F_9	G_10	G_9		
2580829	1797912	999373	336028		
F_44	F_9	G_10	G_9	ttest rank	NPvP ttest
1.05	0.04	-0.64	-0.22	15	0.17
-0.17	-0.31	1.06	-0.22	103	0.81
-0.25	0.12	-1.24	-0.70	99	0.78
-0.66	0.91	0.57	-0.84	85	0.64
-0.42	1.73	-0.45	0.16	9	0.15
-0.81	-0.78	-0.12	0.08	28	0.26
-0.80	-0.43	1.11	1.04	104	0.83
-0.56	-0.55	-0.31	-0.14	118	0.92

-0.71	-0.44	0.40	0.47	42	0.36
0.14	-0.69	0.58	3.33	3	0.07
0.58	-0.27	-0.07	2.97	66	0.55
-1.93	1.81	-1.52	-0.38	60	0.51
-0.21	0.37	0.28	1.22	16	0.17
-1.42	0.27	-1.02	-0.16	96	0.74
-0.58	-0.65	0.08	-0.55	2	0.06
-0.57	1.13	-0.98	-0.86	64	0.54
-0.32	-0.64	0.47	-0.07	32	0.29
-0.30	-0.31	-0.82	-0.71	4	0.07
-0.79	-0.57	0.58	2.62	105	0.83
0.07	-0.18	0.37	0.05	73	0.59
0.46	1.47	-1.15	-0.87	69	0.58
-0.40	-0.29	0.40	1.86	19	0.20
3.98	0.59	2.75	1.03	59	0.46
-0.79	-0.55	0.23	0.15	95	0.74
-0.56	-0.55	0.45	-0.05	83	0.64
1.02	1.14	-0.77	-0.39	45	0.37
-0.75	0.52	-1.14	-0.36	127	0.95
-0.79	-0.86	0.41	0.69	20	0.20
-1.13	-0.55	-0.06	-0.35	37	0.34
1.53	1.63	2.48	3.96	21	0.23
-0.90	-0.17	-0.04	-0.16	112	0.88
-1.55	2.16	-0.42	-1.26	77	0.62
-0.47	-0.02	-0.08	0.29	72	0.59
-0.29	-0.26	-0.27	-0.06	102	0.81
-1.10	-0.46	0.01	-0.31	51	0.41
-0.60	-0.26	-0.27	0.01	55	0.43
-0.81	-0.94	0.53	0.60	128	0.96
-0.61	-0.63	0.57	0.26	121	0.92
-0.17	-0.72	-0.54	-0.60	1	0.0052
-0.68	0.45	-0.64	-0.59	89	0.68
-0.68	1.11	-1.08	-0.98	88	0.67
-0.10	1.75	-0.88	-0.12	114	0.89
-1.07	-0.18	-1.18	-0.62	134	1.00
-0.68	-0.77	-0.18	-0.15	65	0.54
-1.01	-0.55	-0.84	0.32	39	0.35
-0.23	1.56	-1.16	-0.89	29	0.28
-0.32	0.49	1.91	3.44	61	0.52
0.20	0.09	-0.71	-0.42	131	0.99
-0.29	-0.19	-0.11	-0.16	87	0.67
-0.23	-0.29	-0.29	-0.43	33	0.29
0.19	0.80	-0.76	1.12	5	0.09
-0.57	-0.44	-0.21	-0.28	48	0.39
-1.06	-0.53	-0.60	2.02	107	0.84
0.67	-0.19	-0.01	-1.20	6	0.10
-0.46	1.95	0.30	0.51	30	0.28

-0.28	-0.28	-0.26	-0.04	111	0.88
-1.52	-0.48	-0.23	-0.39	7	0.13
-0.87	1.01	-0.12	-0.95	43	0.36
0.38	-0.30	-0.89	0.16	68	0.57
-0.53	-0.11	-0.24	-0.77	12	0.16
-0.89	-0.26	-0.49	0.47	11	0.16
-0.90	-0.05	0.09	-0.61	97	0.76
-0.58	-0.63	-0.09	0.29	41	0.35
-0.25	-0.21	-0.24	-0.22	117	0.91
-0.03	-0.46	-0.70	-0.83	135	1.00
-1.01	-0.82	0.19	0.20	98	0.76
-0.58	-0.30	-0.45	2.50	71	0.58
-0.71	-0.50	0.19	-0.39	110	0.88
-0.67	0.23	-0.64	-0.57	74	0.60
-0.25	-0.37	0.23	0.14	136	1.00
-0.50	0.04	-0.91	0.11	38	0.34
-0.22	0.44	-1.53	-1.33	108	0.84
-0.62	-0.72	1.54	0.16	79	0.63
0.20	-0.39	-0.23	0.38	25	0.25
-0.28	0.04	-0.03	0.32	126	0.95
-0.31	1.29	-0.97	-0.17	13	0.17
-0.57	0.53	1.73	2.33	94	0.73
-0.33	-0.20	0.03	-0.03	36	0.33
-1.01	0.54	-1.06	-1.04	34	0.31
-0.74	-0.60	-0.53	0.31	10	0.16
-1.21	-0.01	-0.68	-0.27	14	0.17
0.00	0.86	-0.81	-0.55	86	0.66
-0.25	-0.39	-0.65	-0.74	58	0.45
-0.28	-0.02	-0.44	0.80	52	0.41
-0.38	0.34	2.05	0.45	90	0.69
-1.01	0.84	-0.83	0.86	119	0.92
0.08	0.52	-1.03	-1.21	91	0.70
-0.84	-0.76	-0.20	-0.18	82	0.63
-0.20	-0.52	-0.59	-0.09	113	0.89
-0.86	1.34	-0.43	-1.18	53	0.43
2.90	3.08	-0.08	0.05	101	0.79
-0.28	-0.29	-0.21	-0.08	133	0.99
-0.33	1.06	-0.68	-1.12	76	0.61
-0.75	-0.15	-0.18	-0.23	49	0.39
-0.52	-0.47	-0.29	0.02	46	0.38
-0.86	-0.48	-0.68	-0.69	62	0.54
0.63	0.98	-0.75	-1.33	35	0.32
0.39	0.59	-0.98	-0.73	130	0.97
0.11	0.78	-0.64	0.60	106	0.84
-0.62	-0.63	0.06	-0.15	123	0.94
-0.43	-0.24	-0.73	0.44	116	0.90
-0.42	1.07	-0.45	-0.03	80	0.63
-0.65	0.37	-1.25	-0.03	44	0.37

-0.51	-0.36	0.62	0.07		93	0.73
-0.73	-0.84	-0.52	0.65		75	0.60
-0.53	-0.63	-0.15	-0.23		70	0.58
-0.06	0.31	-0.95	-0.41		81	0.63
-0.46	-0.41	0.10	0.28		47	0.38
-0.83	-0.20	-0.56	-0.49		92	0.73
-1.03	-0.62	0.70	0.92		40	0.35
-0.58	0.47	-0.09	-0.44		22	0.24
0.28	0.83	-1.03	-0.77		120	0.92
-0.44	-0.13	-0.40	1.03		84	0.64
-0.83	-0.77	1.39	-0.16		100	0.78
-0.86	0.55	-1.13	-0.98		17	0.18
-1.22	1.12	-0.68	-0.05		124	0.94
-0.82	-0.77	4.11	-0.74		54	0.43
-0.59	-0.18	0.27	0.85		26	0.25
-0.59	-0.18	0.27	0.85		26	0.25
-0.94	-0.64	-0.50	0.75		31	0.29
-0.90	0.30	-0.65	1.98		109	0.84
0.88	0.24	-1.12	-0.76		56	0.45
-0.52	0.92	-0.43	-0.26		129	0.97
-0.29	-0.09	0.15	-0.39		8	0.13
-0.31	-0.14	-0.35	-0.39		78	0.62
-0.60	0.77	-1.06	-0.96		18	0.19
-0.75	0.21	-0.17	1.86		125	0.94
-0.22	0.89	-1.15	-0.92		122	0.93
-0.31	0.32	-0.28	-0.61		132	0.99
-0.03	0.43	-0.28	-0.79		23	0.24
-0.19	-0.17	1.83	4.53		67	0.56
-0.18	0.69	-0.77	0.24		24	0.25
-1.15	-0.14	0.84	1.67		57	0.45
-0.48	0.22	-0.84	-0.31		50	0.40
-0.40	0.54	-0.11	0.69		115	0.90
0.00	0.19	0.01	2.46		63	0.54

21	22	23	24	25	26	27
92	93	94	95	2	13	71
TCACAGTGAACC(	TGAGGTAGGAGGT	ACCACTGACCGTTGAC	TACAGTATAG.	GCTGACTCCT.	CAGTGCAATG'	TAGCAGCACATAATGGTTT(
miR-128	let-7e-5p	miR-181a-2-3p	miR-144-3p	miR-345-5p	miR-130a-3]	miR-15a-5p
40544	37943	36605	36676	32906	30537	29005
0.18	-0.64	-0.46	-0.20	1.35	-0.48	-0.56
0.71	-0.49	-1.63	-0.32	-1.77	-0.85	-0.70
-0.79	-0.64	-0.70	-0.24	-0.13	-0.06	0.20
-0.96	-0.60	-1.06	-0.25	0.62	-0.43	-0.46
1.38	1.06	-0.67	-0.22	-1.25	-0.38	-0.24
1.46	-0.07	-0.61	-0.23	-0.10	-0.66	-0.44
1.06	1.44	0.20	-0.28	1.28	-0.46	-0.40
-0.86	-0.46	-0.28	-0.12	0.33	-0.87	-0.57
0.03	-0.92	-1.27	-0.25	-1.06	-0.70	-0.48
0.05	-1.03	-0.45	-0.19	-1.20	-0.65	-0.13
0.73	0.12	-0.50	-0.29	-0.86	-0.11	-0.04
-0.47	0.88	0.70	-0.31	1.37	-0.76	-0.50
2.44	-0.64	-0.40	0.13	0.40	0.74	3.99
-1.60	0.17	0.25	-0.24	-0.46	-0.89	-0.51
-0.26	-0.40	-0.65	0.07	0.05	0.84	0.00
0.86	-0.45	-0.11	-0.04	0.27	2.30	0.97
-1.35	-0.52	1.91	-0.25	1.93	-0.39	-0.41
0.75	0.02	0.91	-0.27	-0.14	3.20	0.47
-0.40	-0.87	0.81	-0.30	-0.63	-0.39	-0.14
1.19	-0.89	0.54	4.95	1.57	0.37	2.17

0.27	-0.54	1.52	-0.10	-0.92	-0.07	-0.32
-0.57	2.21	2.31	-0.31	0.65	-0.23	-0.56
-0.86	0.45	1.45	-0.27	1.08	0.34	-0.46
-1.29	-0.30	0.47	-0.23	-0.05	-0.55	-0.50
-0.21	3.11	-0.65	-0.21	-1.26	-0.51	-0.49
-0.85	0.59	-0.45	-0.30	-0.08	-0.09	-0.34
-0.65	-0.56	-1.18	0.27	-1.02	1.73	0.46
-0.52	-0.61	-1.35	9.91	-0.66	0.73	3.56
3.16	-0.37	-0.71	-0.30	0.08	-0.66	-0.24
0.02	-1.21	0.63	-0.27	-1.27	-0.73	-0.43
-0.98	-0.78	-0.99	-0.21	-1.69	-1.05	-0.59
0.49	0.02	3.75	-0.32	-1.14	-0.63	-0.25
3.52	3.63	-1.49	-0.14	-1.64	-0.02	-0.07
1.36	-0.99	-1.23	-0.25	1.81	-0.82	-0.31
-0.44	-1.25	-0.97	-0.13	-0.54	-0.66	-0.11
1.67	-0.45	0.85	-0.20	1.27	-0.53	-0.48
-1.78	-0.79	-1.48	-0.18	-1.26	-1.07	-0.61
-0.25	-1.01	-0.30	-0.15	-0.90	-0.15	-0.12
0.02	-1.09	0.06	0.18	-0.90	0.52	2.56
-0.08	-0.60	1.39	-0.29	0.86	-0.59	-0.38
-1.41	0.30	2.14	-0.13	1.86	-0.94	-0.33
-0.04	1.80	5.08	0.23	4.53	-0.61	-0.42
-1.18	-0.19	0.60	-0.14	-0.01	0.13	-0.38
0.65	-0.99	-0.51	-0.17	-0.23	0.26	1.02
-0.99	0.02	2.08	-0.29	-0.77	-0.69	-0.58
-1.96	-0.33	0.08	-0.16	0.68	-1.11	-0.44
2.40	-0.01	0.89	-0.22	2.73	0.86	0.41
1.10	-0.41	1.10	-0.24	0.90	-0.04	-0.31
0.90	-0.94	-0.50	-0.06	2.36	1.75	0.23
0.96	-0.94	-0.05	-0.23	1.62	0.22	-0.23
1.05	-0.93	2.81	-0.18	0.28	2.98	0.16
-0.14	0.21	0.21	-0.23	0.38	0.08	-0.50
-0.73	0.62	-0.12	-0.17	-0.71	-0.04	-0.41
1.81	-0.26	0.14	-0.26	1.01	0.61	0.09
1.22	0.59	1.71	-0.27	-0.21	-0.48	-0.51
-0.88	0.25	1.11	-0.26	-0.42	-0.13	-0.47
0.34	0.17	0.06	-0.24	-0.90	0.50	-0.22
-0.14	-0.70	-0.48	8.69	-0.36	1.25	2.75
0.66	1.06	0.28	-0.28	0.56	-0.29	-0.62
2.90	0.70	-0.07	-0.24	-0.68	0.47	-0.29
2.68	0.52	-0.46	-0.19	-0.85	0.47	-0.19
-0.29	-1.04	-0.70	8.22	-0.97	0.78	3.23
-1.03	-0.42	0.22	-0.06	-0.41	-0.62	-0.52
-0.86	-0.08	-0.72	-0.19	-0.04	-0.27	-0.39
-0.26	-0.74	-0.58	-0.14	-0.73	0.34	-0.18
-0.98	0.02	2.73	-0.24	-0.21	-0.63	-0.25
1.01	-1.26	-1.55	-0.17	-0.45	1.26	0.65
-0.60	-0.56	-0.16	-0.32	-1.44	0.32	-0.70
0.88	0.65	-0.08	-0.17	-0.76	-0.49	-0.36
3.09	0.41	0.03	-0.26	1.20	0.34	0.01
-0.42	0.04	-0.16	-0.23	0.44	-0.32	-0.40
4.46	-1.12	2.38	-0.21	-1.22	-0.24	2.99
-0.07	-0.72	-0.41	-0.12	-0.13	-0.35	0.11
1.06	-0.53	0.21	-0.16	1.41	0.18	1.07
-2.12	-0.59	1.49	0.07	2.97	-0.90	-0.66
1.03	0.19	-0.27	-0.13	0.47	1.13	0.76
-0.87	-0.63	-0.60	-0.30	-0.85	-0.54	-0.39
-1.01	0.72	1.47	-0.27	-1.43	-0.60	-0.51
0.18	-0.93	0.15	0.30	0.43	2.24	1.52

0.63	-0.18	0.33	-0.20	0.50	2.25	0.42
-1.25	-0.23	-0.65	-0.22	0.74	-0.05	-0.29
0.88	-0.42	0.45	-0.10	2.02	0.34	0.16
-0.24	-0.57	0.03	-0.21	0.97	0.17	-0.33
1.18	-0.93	0.42	6.69	0.64	0.73	2.83
0.04	-1.14	-1.12	14.75	0.22	0.36	4.61
0.97	-0.39	0.84	-0.24	0.98	0.64	0.07
1.15	-0.53	0.17	-0.19	1.86	0.37	-0.08
0.39	-0.80	-2.04	-0.16	0.12	0.31	-0.14
1.95	-0.92	0.99	-0.28	0.13	0.25	0.56
1.45	0.37	0.62	-0.22	0.07	0.37	-0.17
0.21	0.80	1.24	-0.27	-0.26	-0.82	-0.05
-0.20	-0.86	2.90	-0.28	-0.33	-0.75	-0.52
-0.52	1.34	3.08	-0.29	1.06	-0.15	-0.47
-0.59	-0.43	-0.08	-0.21	-0.68	-0.18	-0.29
-0.09	-1.18	0.05	-0.08	-1.12	-0.23	0.02
0	-1	-1	1	-1	1	0
0.00	0.00	0.00	0.00	0.00	0.00	0.00
1.00	1.00	1.00	1.00	1.00	1.00	1.00
0.85	0.52	0.76	0.97	0.50	0.36	0.37

89	90	91	92	93	94	95
TCACAGTGAACC(	TGAGGTAGGAGG1	ACCACTGACCGTTGAC	TACAGTATAG.	GCTGACTCCT.	CAGTGCAATG'	TAGCAGCACATAATGGTTT(
miR-128	let-7e-5p	miR-181a-2-3p	miR-144-3p	miR-345-5p	miR-130a-3	miR-15a-5p
40544	37943	36605	36676	32906	30537	29005
0.18	-0.64	-0.46	-0.20	1.35	-0.48	-0.56
0.71	-0.49	-1.63	-0.32	-1.77	-0.85	-0.70
-0.79	-0.64	-0.70	-0.24	-0.13	-0.06	0.20
-0.96	-0.60	-1.06	-0.25	0.62	-0.43	-0.46
1.38	1.06	-0.67	-0.22	-1.25	-0.38	-0.24
1.46	-0.07	-0.61	-0.23	-0.10	-0.66	-0.44
1.06	1.44	0.20	-0.28	1.28	-0.46	-0.40
-0.86	-0.46	-0.28	-0.12	0.33	-0.87	-0.57
0.03	-0.92	-1.27	-0.25	-1.06	-0.70	-0.48
0.05	-1.03	-0.45	-0.19	-1.20	-0.65	-0.13
0.73	0.12	-0.50	-0.29	-0.86	-0.11	-0.04
-0.47	0.88	0.70	-0.31	1.37	-0.76	-0.50
2.44	-0.64	-0.40	0.13	0.40	0.74	3.99
-1.60	0.17	0.25	-0.24	-0.46	-0.89	-0.51
-0.26	-0.40	-0.65	0.07	0.05	0.84	0.00
0.86	-0.45	-0.11	-0.04	0.27	2.30	0.97
-1.35	-0.52	1.91	-0.25	1.93	-0.39	-0.41
0.75	0.02	0.91	-0.27	-0.14	3.20	0.47
-0.40	-0.87	0.81	-0.30	-0.63	-0.39	-0.14
1.19	-0.89	0.54	4.95	1.57	0.37	2.17
0.27	-0.54	1.52	-0.10	-0.92	-0.07	-0.32
-0.57	2.21	2.31	-0.31	0.65	-0.23	-0.56
-0.86	0.45	1.45	-0.27	1.08	0.34	-0.46
-1.29	-0.30	0.47	-0.23	-0.05	-0.55	-0.50
-0.21	3.11	-0.65	-0.21	-1.26	-0.51	-0.49
-0.85	0.59	-0.45	-0.30	-0.08	-0.09	-0.34
-0.65	-0.56	-1.18	0.27	-1.02	1.73	0.46
-0.52	-0.61	-1.35	9.91	-0.66	0.73	3.56

3.16	-0.37	-0.71	-0.30	0.08	-0.66	-0.24
0.02	-1.21	0.63	-0.27	-1.27	-0.73	-0.43
-0.98	-0.78	-0.99	-0.21	-1.69	-1.05	-0.59
0.49	0.02	3.75	-0.32	-1.14	-0.63	-0.25
3.52	3.63	-1.49	-0.14	-1.64	-0.02	-0.07
1.36	-0.99	-1.23	-0.25	1.81	-0.82	-0.31
-0.44	-1.25	-0.97	-0.13	-0.54	-0.66	-0.11
1.67	-0.45	0.85	-0.20	1.27	-0.53	-0.48
-1.78	-0.79	-1.48	-0.18	-1.26	-1.07	-0.61
-0.25	-1.01	-0.30	-0.15	-0.90	-0.15	-0.12
0.02	-1.09	0.06	0.18	-0.90	0.52	2.56
-0.08	-0.60	1.39	-0.29	0.86	-0.59	-0.38
-1.41	0.30	2.14	-0.13	1.86	-0.94	-0.33
-0.04	1.80	5.08	0.23	4.53	-0.61	-0.42
-1.18	-0.19	0.60	-0.14	-0.01	0.13	-0.38
0.65	-0.99	-0.51	-0.17	-0.23	0.26	1.02
-0.99	0.02	2.08	-0.29	-0.77	-0.69	-0.58
-1.96	-0.33	0.08	-0.16	0.68	-1.11	-0.44
2.40	-0.01	0.89	-0.22	2.73	0.86	0.41
1.10	-0.41	1.10	-0.24	0.90	-0.04	-0.31
0.90	-0.94	-0.50	-0.06	2.36	1.75	0.23
0.96	-0.94	-0.05	-0.23	1.62	0.22	-0.23
1.05	-0.93	2.81	-0.18	0.28	2.98	0.16
-0.14	0.21	0.21	-0.23	0.38	0.08	-0.50
-0.73	0.62	-0.12	-0.17	-0.71	-0.04	-0.41
1.81	-0.26	0.14	-0.26	1.01	0.61	0.09
1.22	0.59	1.71	-0.27	-0.21	-0.48	-0.51
-0.88	0.25	1.11	-0.26	-0.42	-0.13	-0.47
0.34	0.17	0.06	-0.24	-0.90	0.50	-0.22
-0.14	-0.70	-0.48	8.69	-0.36	1.25	2.75
0.66	1.06	0.28	-0.28	0.56	-0.29	-0.62
2.90	0.70	-0.07	-0.24	-0.68	0.47	-0.29
2.68	0.52	-0.46	-0.19	-0.85	0.47	-0.19
-0.29	-1.04	-0.70	8.22	-0.97	0.78	3.23
-1.03	-0.42	0.22	-0.06	-0.41	-0.62	-0.52
-0.86	-0.08	-0.72	-0.19	-0.04	-0.27	-0.39
-0.26	-0.74	-0.58	-0.14	-0.73	0.34	-0.18
-0.98	0.02	2.73	-0.24	-0.21	-0.63	-0.25
1.01	-1.26	-1.55	-0.17	-0.45	1.26	0.65
-0.60	-0.56	-0.16	-0.32	-1.44	0.32	-0.70
0.88	0.65	-0.08	-0.17	-0.76	-0.49	-0.36
3.09	0.41	0.03	-0.26	1.20	0.34	0.01
-0.42	0.04	-0.16	-0.23	0.44	-0.32	-0.40
4.46	-1.12	2.38	-0.21	-1.22	-0.24	2.99
-0.07	-0.72	-0.41	-0.12	-0.13	-0.35	0.11
1.06	-0.53	0.21	-0.16	1.41	0.18	1.07
-2.12	-0.59	1.49	0.07	2.97	-0.90	-0.66
1.03	0.19	-0.27	-0.13	0.47	1.13	0.76
-0.87	-0.63	-0.60	-0.30	-0.85	-0.54	-0.39
-1.01	0.72	1.47	-0.27	-1.43	-0.60	-0.51
0.18	-0.93	0.15	0.30	0.43	2.24	1.52
0.63	-0.18	0.33	-0.20	0.50	2.25	0.42
-1.25	-0.23	-0.65	-0.22	0.74	-0.05	-0.29
0.88	-0.42	0.45	-0.10	2.02	0.34	0.16
-0.24	-0.57	0.03	-0.21	0.97	0.17	-0.33
1.18	-0.93	0.42	6.69	0.64	0.73	2.83
0.04	-1.14	-1.12	14.75	0.22	0.36	4.61
0.97	-0.39	0.84	-0.24	0.98	0.64	0.07
1.15	-0.53	0.17	-0.19	1.86	0.37	-0.08

0.39	-0.80	-2.04	-0.16	0.12	0.31	-0.14
1.95	-0.92	0.99	-0.28	0.13	0.25	0.56
1.45	0.37	0.62	-0.22	0.07	0.37	-0.17
0.21	0.80	1.24	-0.27	-0.26	-0.82	-0.05
-0.20	-0.86	2.90	-0.28	-0.33	-0.75	-0.52
-0.52	1.34	3.08	-0.29	1.06	-0.15	-0.47
-0.59	-0.43	-0.08	-0.21	-0.68	-0.18	-0.29
0	-1	0	0	-1	0	0

89	90	91	92	93	94	95
83	65	112	62	123	130	108
TATTGCACTCGT(	GCCCTGACCTGT(	TCCCCCAGGTGTGATT	TGAGACCTCT(	AATATAACAC.	TGGGTCTTTG(	TTATCAGAATCTCCAGGGG
miR-92b-3p	miR-4732-3p	miR-361-3p	miR-769-5p	miR-410	miR-193a-5	miR-361-5p
51252	93386	20718	102201	14130	11818.00	23449
-1.14	-0.14	1.41	0.08	0.77	-0.63	-0.40
3.28	-0.42	0.02	-0.65	-0.96	-0.46	1.33
-0.34	-0.06	-0.04	-0.69	0.09	-0.30	-0.45
-0.71	0.11	-0.51	-1.15	-0.66	-0.16	-0.76
1.07	-1.30	-0.17	-0.42	-0.96	1.71	4.06
-0.12	1.41	-1.56	-0.62	-0.67	-0.45	-0.25
-0.56	-0.02	0.50	-0.13	-0.07	0.04	-0.57
1.68	2.11	-0.74	0.09	-0.71	-0.29	-0.60
1.27	1.34	-1.54	-1.16	-0.04	-0.64	-0.51
-0.11	0.03	-1.36	-0.45	-0.68	-0.71	-0.21
0.42	-0.98	-0.64	0.18	-0.08	-0.63	0.14
-0.11	1.69	-0.03	0.09	-0.85	-0.01	-0.76
-0.93	-1.19	-0.82	2.44	-0.71	-0.44	0.10
0.52	0.59	-0.03	-0.22	-0.50	0.18	-0.66
-0.42	-0.39	0.25	-0.04	0.58	-0.20	-0.27
-0.58	-0.45	0.04	0.74	0.61	-0.44	-0.06
-0.47	-0.80	0.92	0.39	0.27	-0.01	-0.12
-1.01	0.05	-0.36	0.12	2.22	-0.33	-0.54
-0.77	-1.28	0.41	3.16	0.14	-0.53	0.22
-0.78	-0.66	-0.35	1.19	-0.37	-0.48	0.02
0.02	1.94	-0.11	-0.94	-0.82	-0.50	-0.39
-0.23	-0.25	1.91	0.33	1.00	0.55	-0.53
0.15	0.23	2.64	-0.12	-0.05	0.73	-0.38
0.07	0.88	1.15	-0.40	-0.49	0.06	-0.47
1.34	-0.36	-0.19	-1.44	-0.77	4.30	1.75
-0.88	-1.42	0.47	0.10	0.29	-0.07	0.62
-0.69	-0.64	-1.29	-0.47	3.42	-0.31	-0.30
-0.92	-1.17	-1.54	-0.76	1.28	-0.60	-0.61
0.05	-0.95	-0.17	0.07	0.84	-0.45	0.50
0.36	-0.05	-0.88	-0.53	-0.09	-0.69	-0.32
4.70	7.78	-0.16	-1.44	-0.54	-0.75	-0.20
0.71	-0.09	-0.90	0.31	0.10	-0.68	0.27
1.01	-1.13	-1.42	-0.67	-0.79	0.36	1.81
-0.61	-0.09	0.37	0.43	-0.09	0.89	-0.52
-0.12	-0.58	-0.13	0.95	-0.62	0.33	0.05
-0.42	-1.24	1.91	0.53	-0.81	-0.06	-0.48
0.70	4.89	-1.43	-1.57	-0.84	-0.66	-0.90
-1.04	-1.26	-0.17	1.14	1.01	-0.76	-0.08
-1.27	-1.41	-0.16	2.86	0.14	-0.59	0.21

-0.39	-1.23	0.66	1.21	-0.47	-0.26	-0.09
-0.89	-1.06	1.57	1.32	-0.81	0.02	-0.52
-1.34	-0.73	2.86	8.27	-0.96	-0.28	-0.68
-0.11	-0.71	0.39	-0.57	-0.39	0.12	-0.26
-0.44	-0.78	0.20	1.53	-0.53	-0.22	0.47
-0.34	0.04	0.82	0.09	-0.12	0.17	-0.33
-0.68	-0.45	0.74	-0.27	-0.72	0.43	-0.77
-0.68	-1.32	0.35	0.95	1.33	-0.09	1.10
-0.14	-0.19	0.90	0.98	1.35	0.05	0.14
-0.34	0.17	-0.05	0.07	-0.51	-0.27	-0.22
-0.44	-0.62	2.50	1.14	1.64	-0.30	-0.10
-0.61	-0.73	0.45	0.41	3.74	-0.42	-0.08
0.30	-1.05	1.07	-0.89	0.80	0.08	-0.14
0.23	-0.28	-0.04	-1.00	-0.06	0.57	-0.19
-0.52	-1.16	2.02	-0.07	-0.22	0.06	0.79
0.04	0.47	0.24	-1.02	-0.55	0.26	-0.51
0.16	-0.70	0.09	-0.80	0.11	-0.11	-0.25
-0.60	0.53	-0.34	-1.04	1.44	-0.23	-0.22
-1.31	-1.25	-0.72	0.42	-0.43	-0.47	-0.44
-0.39	-0.68	1.59	-0.17	0.13	1.06	0.17
0.04	-0.43	0.34	0.15	-0.75	1.00	0.85
-0.32	-0.93	0.81	-0.50	-0.32	0.61	1.61
-0.91	-0.96	-1.36	-0.47	1.32	-0.69	-0.49
-0.06	0.32	-0.68	-0.43	-0.81	-0.40	-0.44
-0.20	0.19	-0.68	-0.87	0.04	-0.38	-0.54
-0.88	-0.56	-1.21	-0.77	-0.70	-0.37	-0.31
-1.18	2.38	0.88	1.27	-0.44	0.15	-0.85
-1.17	-1.19	-1.29	-0.81	0.15	-0.77	-0.41
1.63	0.48	-0.52	-1.22	-0.52	0.01	0.28
2.55	4.06	1.54	-0.99	-0.74	-0.15	-0.46
0.51	0.04	2.17	-0.01	-0.06	0.44	-0.17
-0.17	1.30	-0.08	-0.84	-0.18	-0.26	-0.68
-1.26	0.30	-1.41	-0.68	-0.33	-0.76	-0.27
-0.43	-0.10	-1.68	-1.01	-0.57	-0.57	-0.56
-0.85	0.01	-0.36	0.80	0.24	-0.34	-0.30
-1.15	-0.21	8.02	2.31	-0.96	1.07	-0.73
-0.70	-0.25	0.26	0.22	1.14	-0.22	-0.56
-0.55	-1.25	-0.16	1.40	-0.11	-0.26	0.10
0.60	-0.24	-0.59	-0.11	2.79	-0.36	-0.34
-0.71	-1.36	-0.22	0.88	1.44	-0.20	0.54
-0.64	-1.38	-0.29	0.65	-0.08	-0.42	0.91
0.23	-0.68	-0.31	-0.20	-0.85	0.23	-0.10
-0.44	-0.92	-0.03	-0.18	0.52	-0.40	0.55
-0.26	-0.47	0.52	0.59	-0.08	-0.66	-0.30
-1.14	-1.30	0.26	1.82	1.39	-0.67	0.06
-0.91	-1.03	-1.34	-0.22	1.01	-0.63	-0.52
-0.28	-0.37	0.75	0.52	-0.64	-0.60	-0.10
-0.70	-1.06	1.08	0.53	0.39	-0.26	0.06
-0.22	-0.09	-0.26	-0.77	-0.75	-0.18	-0.08
-0.60	-0.96	0.42	1.02	-0.15	-0.16	-0.09
-0.30	-0.83	1.32	-0.11	-0.67	-0.10	0.30
-0.83	-0.30	-0.62	0.80	1.10	-0.12	-0.44
-0.25	-0.03	0.28	-0.90	-0.52	-0.03	-0.46
-0.39	-0.46	0.83	-0.05	0.92	0.43	-0.41
-0.65	-0.70	-1.03	0.09	-0.43	-0.28	0.10
-0.74	-0.83	-0.77	-0.61	-0.26	-0.79	0.28
0.48	0.49	0.50	0.52	0.53	0.56	0.57
0.66	0.73	1.00	0.38	0.76	0.46	1.00

1797912	999373	336028			
99	100	101			
30	31	32			
F_9	G_10	G_9			
CHR_P	CHR_P	CHR_P			
3840464	4043278	1669856			
0.90	0.43	0.87			
F_9	G_10	G_9	UC ave with disca	UC ave	UC sd
2042552	3043905	1333828	2863493	2583054	3181205.95
652973	273186	110265	466300	419901	368585.80
122940	215390	26137	95796	86241	105619.81
125931	14419	12206	69946	63028	56233.40
186789	94406	16565	85100	76611	64336.83
206210	54647	23910	81120	73047	63402.07
18035	33221	13330	53412	48107	74155.61
49594	58222	18835	42864	38603	[illegible]
2158	5400	2740	17282	15577	[illegible]
20381	24629	8586	28322	25496	[illegible]
27101	30790	21344	30961	27946	[illegible]
22088	14122	13068	18817	16971	[illegible]
38405	10022	4659	18941	17062	[illegible]
23357	12687	5899	14434	12998	[illegible]
28494	9022	4547	16876	15204	[illegible]
4309	7370	1032	10192	9185	[illegible]
30687	2256	1015	11545	10395	[illegible]
3110	7239	1515	7170	6456	[illegible]
10154	4100	1477	8726	7858	[illegible]
6488	7688	4923	8026	7228	[illegible]
11759	8333	2437	8456	7694	[illegible]
13778	2678	1067	6721	6054	6928.25
7317	5535	2832	6417	5779	6397.18
9239	10632	2095	5348	4830	6128.55
3395	4481	1400	4390	3957	6049.89
2070	6408	1265	4054	3652	4813.05
9703	2308	978	5128	4631	4740.10
9474	2078	1197	5305	4780	4768.48
1255	4845	1918	4530	4219	5069.74
4359	3250	924	3844	3465	3276.12
9512	6491	2815	4063	3660	3163.25
4603	2770	869	3875	3490	4029.58
12922	3134	588	4500	4051	3603.89
3220	1694	817	2845	2563	4722.29
1446	727	643	4300	3873	14035.22
4101	2969	836	3453	3112	2993.69
5538	3098	1501	4093	3688	3376.31
1877	4939	1697	3832	3462	3654.15
1720	4607	1218	2734	2468	2665.63
2197	1705	513	2980	2685	3020.05
8631	643	269	3365	3030	3933.04
7199	727	289	2595	2337	2469.68
7016	1233	671	2518	2268	1995.26
3313	1092	508	2157	1943	1668.74
360	945	324	1536	1385	2268.67
2024	714	802	1776	1605	1229.63

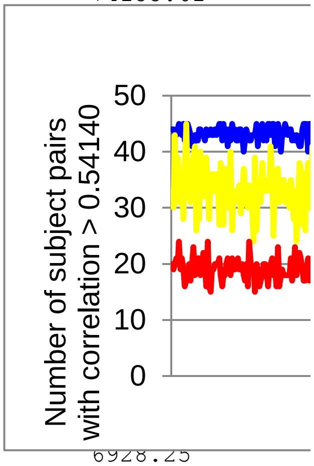

3764	949	354	1827	1645	1467.17
2927	2751	1307	1663	1498	1621.42
2903	881	384	1986	1788	1729.09
327	261	71	547	492	1429.96
981	551	116	1547	1408	2976.66
2777	666	582	1175	1058	1060.27
1077	852	255	1261	1137	1875.41
1598	871	640	1308	1179	1160.26
1413	900	66	1333	1242	1470.12
3332	947	355	1171	1055	1174.40
143	97	88	470	426	1249.72
1221	787	241	1077	971	1121.42
2006	703	130	1030	927	1073.85
1472	581	339	1048	943	844.50
2035	991	121	1092	984	918.42
1181	576	315	916	824	998.44
1186	720	152	949	855	1050.44
69	254	135	438	395	749.43
100	35	18	325	292	1091.22
1183	538	156	1072	965	904.89
442	577	193	695	626	809.87
444	187	479	565	509	996.69
297	384	67	477	430	667.92
816	222	80	536	483	713.88
271	1598	459	800	724	946.36
1228	272	239	694	625	550.03
1379	153	72	719	647	592.17
130	829	122	473	426	795.89
602	377	174	615	558	660.75
629	336	141	411	370	428.72
1819	168	157	601	542	624.91
1044	896	349	670	605	728.77
766	699	209	453	414	371.75
1301	188	65	593	534	517.00
462	283	188	499	449	561.68
606	236	100	443	399	427.29
1014	207	88	493	444	399.50
683	295	88	531	478	465.15
579	46	292	521	470	1364.69
549	626	110	340	307	404.15
747	128	141	362	327	338.37
873	250	74	456	411	328.89
84	153	52	266	240	383.98
354	192	83	311	280	314.77
1253	261	24	388	349	374.56
1101	237	84	309	283	256.41
9	18	13	88	79	265.97
589	127	25	278	250	243.47
278	152	48	247	223	307.00
67	67	39	191	172	384.02
198	80	26	240	216	355.39
664	149	24	282	254	235.11
469	136	52	260	235	210.16
587	145	102	236	212	187.33
45	119	30	188	169	335.27
246	51	87	226	204	250.80
423	121	51	206	186	186.64
515	53	77	247	222	184.97
226	493	95	217	196	202.95

115	95	68	190	171	220.97
68	90	27	158	142	236.42
357	71	42	176	159	157.54
211	228	89	182	164	164.12
174	62	23	158	143	243.22
106	168	62	161	145	209.37
309	124	31	181	163	214.05
351	83	33	183	165	165.40
206	91	75	164	148	178.53
76	210	30	149	134	178.95
301	29	14	138	125	127.43
411	102	49	174	156	152.17
110	300	21	141	127	128.85
153	120	54	127	114	156.86
153	120	54	127	114	156.86
114	79	73	139	126	127.46
244	55	94	131	118	104.99
208	48	22	136	123	118.10
288	46	20	116	105	155.45
139	95	19	133	120	169.07
39	4	0	59	53	148.94
312	44	17	129	116	107.47
179	77	68	87	78	65.90
258	22	12	123	110	115.92
232	78	16	106	95	91.23
305	73	1	106	95	101.92
78	193	131	68	62	95.71
171	38	26	94	85	101.18
109	105	47	91	82	91.09
187	21	21	88	80	82.21
135	41	28	72	65	122.55
139	72	53	94	84	87.11
122	72	32	79	72	64.15
98	35	29	85	76	75.38
208	25	22	72	65	70.37
209	23	14	72	64	61.35
195	135	18	85	77	62.27
109	28	26	76	69	81.11
248	7	4	98	89	88.43
196	54	5	63	56	55.18
16	11	5	34	31	70.23
101	37	15	83	74	77.19
134	37	6	67	60	57.98
72	37	11	57	51	64.81
70	53	21	60	54	44.55
83	24	28	57	51	57.99
26	28	24	62	56	88.76
32	36	16	52	47	58.69
101	17	18	50	45	55.60
148	38	12	55	49	46.13
90	6	2	63	58	88.23
55	32	29	57	51	48.86
60	102	12	72	65	86.09
68	21	52	52	47	43.66
94	17	16	52	47	41.62
82	29	11	67	61	65.51
9	12	1	24	22	63.75
66	23	26	48	43	45.86
100	29	20	53	48	44.02

37	27	5	50	45	91.92
38	25	6	46	41	73.56
84	33	8	59	53	43.21
81	37	10	44	39	35.60
58	21	171	61	55	89.46
43	9	4	32	29	55.79
116	36	10	54	49	44.35
46	29	14	39	35	53.98
48	31	23	43	39	42.64
94	43	23	54	49	40.24
53	45	14	53	48	70.30
24	40	5	45	40	85.17
136	16	4	48	43	50.48
66	17	33	39	35	44.83
55	31	13	40	36	39.41
45	16	20	43	39	47.05
91	5	13	39	35	36.99
76	21	16	45	40	38.53
18	24	6	34	31	59.01
92	7	16	34	30	34.62
17	49	11	35	31	43.98
73	16	12	40	37	35.88
101	13	4	43	39	32.79
17	23	6	33	29	55.70
81	4	11	35	31	43.56
68	18	19	41	37	44.51
55	38	11	35	32	39.85
73	2	0	15	14	23.33
6	29	4	38	34	78.82
50	15	0	36	32	37.50
106	26	0	42	38	37.86
26	41	23	39	36	33.60
46	40	4	28	25	24.33
19	16	1	26	24	62.16
51	23	8	41	37	35.45
45	10	14	33	29	41.15
46	8	5	35	31	37.94
125	28	0	36	32	36.63
17	7	7	29	27	25.78
139	17	4	36	32	29.52
62	33	15	29	28	29.80
9	10	6	23	21	33.65
153	9	3	26	24	29.83
80	29	14	34	30	30.36
49	23	6	32	29	30.23
12	23	8	34	31	39.00
27	15	11	34	31	28.02
77	8	4	28	25	26.28
41	13	8	29	26	34.27
31	7	5	19	17	25.93
40	13	6	31	28	26.81
34	13	9	27	24	27.88
44	27	1	27	24	24.36
41	14	3	24	21	23.89
43	18	6	24	22	26.57
0	0	0	7	6	18.13
35	25	3	23	20	21.01
1	40	3	25	22	32.11
23	9	14	25	22	22.23

51	4	7	19	17	18.35
26	10	21	23	21	26.09
43	5	0	17	16	22.66
66	4	2	22	20	16.12
47	7	3	22	19	18.63
38	4	3	23	21	17.27
32	9	7	19	17	15.20
17	6	4	20	18	20.56
7	21	7	17	16	24.35
30	10	5	24	21	22.04
1	2	1	9	9	20.31
33	4	2	19	17	16.59
28	6	3	15	14	16.11
19	9	13	16	15	15.63
18	4	3	13	11	11.69
27	9	3	12	11	23.51
16	5	2	16	15	24.66
36	5	7	16	14	13.55
17	6	8	12	11	14.88
28	5	7	13	12	16.87
4	19	1	14	13	20.88
24	12	2	12	11	11.28
24	12	2	12	11	11.28
41	1	1	14	13	15.41
40	12	2	16	14	14.28
18	12	5	12	11	13.15
35	3	4	12	11	15.37
8	13	3	13	12	12.41
24	7	2	15	14	14.98
8	5	6	11	10	13.88
28	10	2	12	11	12.13
54	2	0	12	11	12.72
7	12	3	14	12	23.16
16	41	1	14	12	19.78
37	3	2	14	13	14.56
12	5	2	15	13	16.31
2	1	0	4	4	12.17
25	7	4	13	12	11.82
7	15	3	15	14	31.13
41	5	1	14	13	15.17
22	10	4	14	12	13.51
6	14	3	14	13	30.34
42	5	4	13	12	13.19
28	6	0	13	12	13.47
19	4	7	13	11	16.72
35	6	4	13	11	9.38
1	0	2	4	4	13.29
35	0	2	12	11	11.74
14	7	3	13	12	12.53
13	4	0	11	10	11.04
16	1	3	9	8	8.99
13	11	1	11	10	11.63
0	1	0	4	4	8.36
10	7	2	10	9	7.82
9	6	2	9	8	14.13
1	53	0	19	18	52.85
26	5	2	12	11	10.45
16	4	0	11	10	13.13
21	8	1	10	9	9.67

36	2	3	10	9	10.12
31	8	2	10	9	8.26
24	3	0	9	8	10.02
15	1	5	10	9	8.31
15	7	2	10	9	8.75
12	7	0	11	10	9.10
8	5	2	8	8	12.97
26	0	6	9	8	9.99
1	8	2	7	6	15.31
5	4	1	7	6	12.35
5	12	5	8	7	9.44
26	2	1	10	9	9.84
20	6	6	9	8	11.48
23	0	0	12	11	15.04
22	2	3	8	7	8.33
8	2	2	9	8	13.14
10	6	2	12	11	11.52
11	6	5	8	7	9.91
27	3	1	8	7	8.20
3	2	0	5	5	7.42
31	4	1	8	7	9.02
34	9	1	8	7	6.79
11	2	0	8	7	8.22
14	4	1	9	8	7.05
15	7	2	8	7	9.43
28	0	0	9	8	9.83
13	1	1	9	9	9.97
14	1	3	9	8	8.51
13	5	2	6	6	5.77
1	0	2	6	6	7.28
15	9	6	8	7	6.57
1	1	0	6	5	10.15
0	4	1	6	6	11.77
4	6	6	7	6	6.12
4	10	0	7	7	8.00
8	4	5	7	6	7.04
23	1	3	8	7	8.94
11	2	1	7	6	7.12
27	0	3	6	5	6.59
23	1	3	7	6	6.67
3	4	4	6	6	10.93
13	3	0	7	6	7.21
17	1	1	6	5	6.38
23	3	1	6	6	6.66
14	2	1	5	5	5.61
4	4	6	6	6	8.00
16	2	0	7	6	5.21
3	3	1	6	5	5.92
7	4	5	6	5	4.82
14	2	0	6	5	5.25
12	1	0	7	6	6.95
3	1	2	4	3	7.27
14	3	2	5	5	5.72
0	5	1	4	4	5.47
14	9	3	5	5	6.54
26	0	0	5	5	5.73
17	0	0	6	5	5.27
0	6	3	3	3	6.41
9	0	0	6	6	6.43

0	7	0	7	7	5.33
15	2	0	7	6	5.82
13	1	0	5	5	5.84
10	0	0	5	5	4.95
15	4	1	5	5	4.84
12	4	0	6	6	7.13
8	7	3	6	6	5.89
1	3	5	3	2	4.57
3	8	1	5	4	6.32
12	2	0	4	4	4.08
7	2	0	5	4	4.11
17	0	2	4	4	5.35
6	1	1	5	5	5.37
1	0	0	5	4	13.57
11	0	1	4	4	4.12
3	2	1	5	4	5.65
6	0	0	3	3	4.46
7	2	0	4	4	4.56
2	1	2	3	3	5.94
2	3	0	4	4	4.28
6	2	1	3	3	5.61
4	2	0	4	4	6.11
6	2	1	3	3	2.36
10	3	2	4	4	3.49
4	4	0	4	4	4.64
8	1	1	4	3	3.53
0	0	0	3	2	5.44
1	1	0	4	3	10.92
5	3	1	4	4	4.95
7	2	1	3	3	3.37
2	1	2	2	2	1.80
9	3	0	3	3	3.52
4	2	0	2	2	2.78
12	0	1	4	4	4.15
2	0	0	2	2	3.43
13	0	1	3	3	3.93
4	1	0	3	3	4.20
10	1	0	3	3	3.52
7	1	0	4	3	3.38
6	2	0	3	3	3.31
7	4	0	4	3	5.77
5	2	2	2	2	2.23
9	1	0	3	3	3.57
7	0	4	3	2	2.75
6	3	2	3	3	2.34
3	0	0	3	3	3.57
10	0	0	2	2	2.68
1	4	0	3	3	3.30
2	0	1	2	2	3.74
4	0	0	2	2	3.99
6	2	0	3	3	2.73
7	1	0	2	2	1.95
1	1	0	3	2	4.66
6	1	0	2	2	2.55
3	0	0	3	2	2.71
0	5	1	3	3	3.94
5	2	0	3	2	3.20
10	1	0	3	3	2.82
8	1	0	3	3	5.00

2	1	2	2	2	2.91
8	2	0	2	2	2.46
1	0	0	3	2	4.70
4	0	1	1	1	2.44
8	0	1	2	2	2.17
3	1	0	3	2	3.18
6	0	0	3	3	3.49
4	1	0	2	2	4.23
11	2	0	2	2	2.65
2	2	0	2	2	2.05
0	2	0	2	2	4.53
0	0	0	1	1	3.32
0	0	0	1	1	2.55
3	1	0	2	2	3.47
0	0	0	3	3	3.06
3	0	0	2	2	2.57
4	2	1	2	2	2.05
4	1	1	2	2	2.02
0	0	0	1	1	3.37
1	0	1	1	1	1.18
4	1	3	2	1	1.48
0	0	1	2	2	4.93
8	3	0	3	2	3.67
8	1	0	2	2	2.18
3	0	0	3	2	2.64
0	0	0	2	2	4.58
8	0	1	2	2	2.71
1	4	0	2	2	2.96
5	1	0	2	2	2.28
0	1	0	2	2	2.28
0	0	0	1	1	3.08
4	1	0	3	2	3.11
5	0	0	3	2	2.31
9	1	1	3	2	3.23
5	2	0	2	2	3.59
3	2	1	2	2	3.18
2	1	0	2	2	1.78
0	1	2	2	2	1.95
6	0	0	2	2	2.44
2	0	0	2	2	2.05
2	0	0	1	1	1.78
2	0	0	1	1	1.35
7	0	1	2	2	1.70
6	2	0	2	2	2.75
2	0	0	2	2	2.11
4	1	0	1	1	1.06
2	0	0	2	2	4.69
3	0	1	2	2	1.92
5	1	1	2	2	1.82
7	2	0	2	2	1.87
2	1	0	2	2	2.36
0	2	0	2	1	2.39
0	0	0	2	2	2.95
1	3	0	1	1	2.64
3	0	0	2	1	2.30
2	0	1	1	1	1.66
0	1	1	2	2	2.41
2	2	0	2	2	2.42
1	2	1	2	2	2.44

6	1	1	1	1	1.63
3	1	1	2	2	1.80
2	3	0	2	2	1.59
1	0	1	1	1	1.52
2	0	2	2	2	2.97
0	0	0	1	1	2.97
5	0	2	1	1	1.63
2	0	0	1	1	2.41
3	0	0	1	1	1.94
0	0	1	1	1	1.46
3	0	0	1	1	1.87
4	0	1	1	1	2.10
0	1	0	1	1	2.76
1	3	0	1	1	1.62
1	1	1	1	1	1.65
1	0	1	1	1	1.22
3	1	0	1	1	1.51
3	3	0	1	1	1.83
0	0	0	1	1	2.77
0	0	1	1	1	1.67
1	0	0	1	1	1.64
0	0	0	1	1	1.41
0	0	0	1	1	2.66
1	1	0	2	1	1.36
5	0	0	1	1	1.81
3	1	0	1	1	1.38
0	0	0	2	2	2.39
2	0	1	1	1	1.53
1	0	0	1	1	1.68
1	0	0	1	1	1.18
2	0	1	1	0	1.22
5	0	0	1	1	1.37
3	0	0	1	1	1.32
0	0	0	1	1	1.33
3	0	0	1	1	1.10
1	0	1	1	1	1.31
5	0	2	1	1	1.58
3	1	0	1	1	1.74
2	0	0	1	1	1.63
8	0	0	2	2	1.90
2	1	0	1	1	1.35
2	3	0	1	1	1.78
5	0	0	1	1	1.54
1	1	0	1	1	1.39
0	0	0	1	1	1.61
4	3	1	1	1	1.76
6	0	0	1	1	1.85
6	0	2	1	1	2.12
2	0	0	1	1	1.37
4	0	1	1	1	1.10
1	0	0	1	1	1.72
0	0	0	1	0	0.68
0	0	2	1	1	0.97
0	0	1	1	1	0.82
0	1	1	1	1	1.10
2	0	0	1	1	1.52
3	0	1	1	1	1.15
1	0	0	1	1	1.07
3	0	0	1	1	1.49

2	0	0	1	1	1.68
1	0	0	1	1	1.20
2	0	0	1	1	1.89
3	0	0	1	1	1.42
1	1	0	1	1	1.07
2	0	0	1	1	1.55
2	0	0	1	1	1.41
2	1	0	1	1	2.13
1	0	1	1	1	1.03
8	0	0	1	1	2.58
2	1	0	1	1	0.94
0	0	0	1	1	1.34
0	0	0			
F_9	G_10	G_9			
CHR_P	CHR_P	CHR_P			
2042552	3043905	1333828			
17	24	25		21	4.75
-0.89	0.50	0.80			

	UC ave	UC sd	sd/ave
	466300	359409	0.77
	95796	107213	1.12
	69946	55047	0.79
	85100	62197	0.73
	81120	61700	0.76
	53412	76428	1.43
	42864	41027	0.96
	17282	34968	2.02
	28322	36116	1.28
	30961	31822	1.03
	18817	18275	0.97
	18941	13984	0.74
	14434	14370	1.00
	16876	12748	0.76
	10192	17544	1.72
	11545	10903	0.94
	7170	11995	1.67
	8726	9877	1.13
	8026	9388	1.17

8456	7531	0.89
6721	6994	1.04
6417	6435	1.00
5348	6254	1.17
4390	6235	1.42
4054	4916	1.21
5128	4743	0.92
5305	4743	0.89
4530	5233	1.16
3844	3237	0.84
4063	3078	0.76
3875	4071	1.05
4500	3521	0.78
2845	4904	1.72
4300	14759	3.43
3453	2965	0.86
4093	3319	0.81
3832	3670	0.96
2734	2682	0.98
2980	3044	1.02
3365	4011	1.19
2595	2473	0.95
2518	1947	0.77
2157	1622	0.75
1536	2347	1.53
1776	1176	0.66
1827	1435	0.79
1663	1628	0.98
1986	1712	0.86
547	1500	2.74
1547	3111	2.01
1175	1055	0.90
1261	1940	1.54
1308	1153	0.88
1333	1516	1.14
1171	1183	1.01
470	1312	2.79
1077	1134	1.05
1030	1085	1.05
1048	826	0.79
1092	905	0.83
916	1013	1.11
949	1067	1.12
438	779	1.78
325	1148	3.54
1072	891	0.83
695	826	1.19
565	1037	1.84
477	689	1.44
536	734	1.37
800	968	1.21
694	537	0.77
719	581	0.81
473	827	1.75
615	673	1.09
411	433	1.05
601	631	1.05
670	741	1.11
453	371	0.82

593	512	0.86
499	571	1.15
443	429	0.97
493	391	0.79
531	461	0.87
521	1432	2.75
340	413	1.21
362	338	0.93
456	315	0.69
266	397	1.49
311	317	1.02
388	375	0.97
309	257	0.83
88	279	3.17
278	241	0.87
247	314	1.27
191	401	2.10
240	367	1.53
282	231	0.82
260	206	0.79
236	183	0.78
188	349	1.86
226	255	1.13
206	186	0.90
247	179	0.72
217	203	0.93
190	225	1.19
158	245	1.55
176	157	0.89
182	164	0.90
158	252	1.59
161	215	1.33
181	219	1.21
183	164	0.90
164	181	1.11
149	183	1.23
138	127	0.92
174	151	0.87
141	129	0.92
127	161	1.27
127	161	1.27
139	127	0.91
131	103	0.78
136	117	0.86
116	160	1.38
133	174	1.30
59	156	2.67
129	106	0.82
87	64	0.74
123	116	0.94
106	90	0.86
106	102	0.97
68	99	1.45
94	103	1.09
91	92	1.02
88	82	0.93
72	127	1.76
94	87	0.93
79	64	0.81

85	75	0.88
72	71	0.98
72	61	0.85
85	60	0.71
76	82	1.07
98	88	0.90
63	55	0.87
34	73	2.16
83	77	0.93
67	57	0.86
57	66	1.16
60	43	0.72
57	58	1.03
62	92	1.48
52	60	1.14
50	57	1.13
55	45	0.83
63	92	1.46
57	48	0.85
72	88	1.22
52	43	0.83
52	41	0.79
67	66	0.98
24	67	2.77
48	46	0.96
53	43	0.81
50	96	1.90
46	76	1.67
59	42	0.70
44	35	0.80
61	92	1.52
32	58	1.83
54	44	0.81
39	56	1.43
43	43	0.99
54	39	0.72
53	72	1.36
45	89	1.99
48	51	1.06
39	46	1.17
40	40	0.99
43	48	1.10
39	37	0.96
45	38	0.85
34	61	1.79
34	35	1.04
35	45	1.30
40	36	0.90
43	32	0.73
33	58	1.78
35	45	1.28
41	45	1.11
35	41	1.15
15	24	1.60
38	82	2.19
36	38	1.07
42	38	0.90
39	34	0.87
28	24	0.87

26	65	2.47
41	35	0.86
33	42	1.30
35	38	1.10
36	37	1.02
29	26	0.87
36	29	0.81
29	30	1.05
23	35	1.49
26	30	1.16
34	30	0.89
32	30	0.95
34	40	1.15
34	28	0.80
28	26	0.93
29	35	1.23
19	27	1.40
31	27	0.87
27	28	1.06
27	24	0.90
24	24	1.01
24	27	1.13
7	19	2.81
23	21	0.93
25	33	1.35
25	22	0.90
19	18	0.98
23	27	1.14
17	23	1.34
22	15	0.69
22	18	0.85
23	17	0.71
19	15	0.78
20	21	1.03
17	25	1.44
24	22	0.93
9	21	2.26
19	16	0.87
15	16	1.07
16	16	0.95
13	12	0.92
12	25	2.03
16	26	1.57
16	13	0.84
12	15	1.22
13	17	1.36
14	22	1.51
12	11	0.97
12	11	0.97
14	16	1.12
16	14	0.92
12	13	1.09
12	16	1.29
13	12	0.97
15	15	0.99
11	14	1.26
12	12	0.99
12	13	1.07
14	24	1.76

14	20	1.47
14	15	1.06
15	17	1.11
4	13	2.97
13	12	0.90
15	33	2.14
14	15	1.08
14	14	0.99
14	32	2.19
13	13	0.99
13	14	1.04
13	17	1.37
13	9	0.72
4	14	3.63
12	12	0.97
13	13	0.96
11	11	1.01
9	9	0.99
11	12	1.07
4	9	2.18
10	8	0.80
9	15	1.62
19	55	2.85
12	10	0.87
11	13	1.24
10	10	0.92
10	10	1.06
10	8	0.82
9	10	1.11
10	8	0.82
10	9	0.84
11	9	0.83
8	13	1.60
9	10	1.20
7	16	2.29
7	13	1.95
8	10	1.24
10	10	1.01
9	12	1.38
12	15	1.32
8	8	1.06
9	14	1.48
12	12	0.96
8	10	1.25
8	8	1.04
5	8	1.46
8	9	1.15
8	7	0.86
8	8	1.08
9	7	0.77
8	10	1.26
9	10	1.10
9	10	1.06
9	9	0.98
6	6	0.88
6	7	1.15
8	6	0.84
6	11	1.81
6	12	1.96

7	6	0.90
7	8	1.12
7	7	1.05
8	9	1.20
7	7	1.06
6	7	1.14
7	7	0.99
6	11	1.77
7	7	1.03
6	7	1.17
6	7	1.05
5	6	1.11
6	8	1.34
7	5	0.76
6	6	1.02
6	5	0.83
6	5	0.92
7	7	1.01
4	8	2.03
5	6	1.11
4	6	1.37
5	7	1.25
5	6	1.12
6	5	0.90
3	7	2.56
6	7	1.01
7	5	0.77
7	6	0.84
5	6	1.18
5	5	0.98
5	5	0.89
6	7	1.17
6	6	0.92
3	5	1.76
5	6	1.34
4	4	0.94
5	4	0.88
4	5	1.22
5	5	1.05
5	14	3.10
4	4	0.94
5	6	1.27
3	5	1.52
4	5	1.11
3	6	1.97
4	4	1.06
3	6	1.67
4	6	1.50
3	2	0.76
4	3	0.77
4	5	1.10
4	4	0.91
3	6	2.16
4	11	3.16
4	5	1.28
3	3	1.19
2	2	0.88
3	4	1.04
2	3	1.22

4	4	1.01
2	4	1.72
3	4	1.19
3	4	1.42
3	4	1.11
4	3	0.88
3	3	1.07
4	6	1.58
2	2	1.01
3	4	1.23
3	3	1.06
3	2	0.77
3	4	1.28
2	3	1.26
3	3	0.95
2	4	1.63
2	4	2.16
3	3	0.92
2	2	1.02
3	5	1.82
2	3	1.28
3	3	1.01
3	4	1.28
3	3	1.26
3	3	0.87
3	5	1.73
2	3	1.32
2	2	1.10
3	5	1.78
1	3	1.75
2	2	0.98
3	3	1.26
3	4	1.11
2	4	1.99
2	3	1.08
2	2	0.99
2	5	2.33
1	3	2.69
1	3	4.03
2	4	1.62
3	3	1.10
2	3	1.20
2	2	0.92
2	2	0.86
1	4	2.81
1	1	1.66
2	1	0.95
2	5	2.44
3	4	1.48
2	2	1.08
3	3	1.06
2	5	2.59
2	3	1.34
2	3	1.56
2	2	1.29
2	2	1.35
1	3	2.81
3	3	1.26
3	2	0.87

3	3	1.24
2	4	1.98
2	3	1.36
2	2	1.05
2	2	1.02
2	2	1.14
2	2	1.26
1	2	2.40
1	1	1.12
2	2	0.71
2	3	1.67
2	2	1.18
1	1	1.48
2	5	2.55
2	2	1.07
2	2	0.89
2	2	1.04
2	2	1.08
2	2	1.63
2	3	1.34
1	3	1.90
2	2	1.49
1	2	1.17
2	2	1.31
2	2	1.40
2	2	1.11
1	2	1.25
2	2	1.07
2	2	0.96
1	2	1.36
2	3	1.63
1	3	2.40
1	2	1.22
1	3	2.53
1	2	1.34
1	2	1.64
1	2	2.03
1	2	1.50
1	3	2.22
1	2	1.62
1	2	1.48
1	1	1.30
1	2	1.19
1	2	3.47
1	3	2.80
1	2	1.50
1	2	1.42
1	1	2.49
1	3	2.59
2	1	0.84
1	2	1.25
1	1	1.22
2	2	1.48
1	2	1.32
1	2	2.79
1	1	1.57
1	1	2.47
1	1	1.14
1	1	1.54

1	1	1.43
1	1	1.26
1	1	1.21
1	2	1.64
1	2	2.13
1	2	1.37
2	2	1.01
1	1	1.57
1	2	1.31
1	2	1.18
1	1	0.99
1	2	1.44
1	2	1.40
1	2	1.43
1	2	1.53
1	1	1.04
1	1	1.03
1	2	1.37
1	1	1.35
1	1	1.70
1	1	1.53
1	1	1.26
1	2	1.77
1	1	1.53
1	1	1.13
1	2	1.37
1	2	2.05
1	1	1.07
1	2	2.05
1	1	1.66
1	1	1.89
1	2	1.55
1	1	1.22
1	2	2.15
1	1	1.43
1	3	2.02
1	1	1.11
1	1	1.09

UC ave	UC sd	sd/ave
466300	359409	0.77
95796	107213	1.12
69946	55047	0.79
85100	62197	0.73
81120	61700	0.76

53412	76428	1.43
42864	41027	0.96
17282	34968	2.02
28322	36116	1.28
30961	31822	1.03
18817	18275	0.97
18941	13984	0.74
14434	14370	1.00
16876	12748	0.76
10192	17544	1.72
11545	10903	0.94
7170	11995	1.67
8726	9877	1.13
8026	9388	1.17
8456	7531	0.89
6721	6994	1.04
6417	6435	1.00
5348	6254	1.17
4390	6235	1.42
4054	4916	1.21
5128	4743	0.92
5305	4743	0.89
4530	5233	1.16
3844	3237	0.84
4063	3078	0.76
3875	4071	1.05
4500	3521	0.78
2845	4904	1.72
4300	14759	3.43
3453	2965	0.86
4093	3319	0.81
3832	3670	0.96
2734	2682	0.98
2980	3044	1.02
3365	4011	1.19
2595	2473	0.95
2518	1947	0.77
2157	1622	0.75
1536	2347	1.53
1776	1176	0.66
1827	1435	0.79
1663	1628	0.98
1986	1712	0.86
547	1500	2.74
1547	3111	2.01
1175	1055	0.90
1261	1940	1.54
1308	1153	0.88
1333	1516	1.14
1171	1183	1.01
470	1312	2.79
1077	1134	1.05
1030	1085	1.05
1048	826	0.79
1092	905	0.83
916	1013	1.11
949	1067	1.12
438	779	1.78
325	1148	3.54

1072	891	0.83
695	826	1.19
565	1037	1.84
477	689	1.44
536	734	1.37
800	968	1.21
694	537	0.77
719	581	0.81
473	827	1.75
615	673	1.09
411	433	1.05
601	631	1.05
670	741	1.11
453	371	0.82
593	512	0.86
499	571	1.15
443	429	0.97
493	391	0.79
531	461	0.87
521	1432	2.75
340	413	1.21
362	338	0.93
456	315	0.69
266	397	1.49
311	317	1.02
388	375	0.97
309	257	0.83
88	279	3.17
278	241	0.87
247	314	1.27
191	401	2.10
240	367	1.53
282	231	0.82
260	206	0.79
236	183	0.78
188	349	1.86
226	255	1.13
206	186	0.90
247	179	0.72
217	203	0.93
190	225	1.19
158	245	1.55
176	157	0.89
182	164	0.90
158	252	1.59
161	215	1.33
181	219	1.21
183	164	0.90
164	181	1.11
149	183	1.23
138	127	0.92
174	151	0.87
141	129	0.92
127	161	1.27
127	161	1.27
139	127	0.91
131	103	0.78
136	117	0.86
116	160	1.38

133	174	1.30
59	156	2.67
129	106	0.82
87	64	0.74
123	116	0.94
106	90	0.86
106	102	0.97
68	99	1.45
94	103	1.09
91	92	1.02
88	82	0.93
72	127	1.76
94	87	0.93
79	64	0.81
85	75	0.88
72	71	0.98
72	61	0.85
85	60	0.71
76	82	1.07
98	88	0.90
63	55	0.87
34	73	2.16
83	77	0.93
67	57	0.86
57	66	1.16
60	43	0.72
57	58	1.03
62	92	1.48
52	60	1.14
50	57	1.13
55	45	0.83
63	92	1.46
57	48	0.85
72	88	1.22
52	43	0.83
52	41	0.79
67	66	0.98
24	67	2.77
48	46	0.96
53	43	0.81
50	96	1.90
46	76	1.67
59	42	0.70
44	35	0.80
61	92	1.52
32	58	1.83
54	44	0.81
39	56	1.43
43	43	0.99
54	39	0.72
53	72	1.36
45	89	1.99
48	51	1.06
39	46	1.17
40	40	0.99
43	48	1.10
39	37	0.96
45	38	0.85
34	61	1.79

34	35	1.04
35	45	1.30
40	36	0.90
43	32	0.73
33	58	1.78
35	45	1.28
41	45	1.11
35	41	1.15
15	24	1.60
38	82	2.19
36	38	1.07
42	38	0.90
39	34	0.87
28	24	0.87
26	65	2.47
41	35	0.86
33	42	1.30
35	38	1.10
36	37	1.02
29	26	0.87
36	29	0.81
29	30	1.05
23	35	1.49
26	30	1.16
34	30	0.89
32	30	0.95
34	40	1.15
34	28	0.80
28	26	0.93
29	35	1.23
19	27	1.40
31	27	0.87
27	28	1.06
27	24	0.90
24	24	1.01
24	27	1.13
7	19	2.81
23	21	0.93
25	33	1.35
25	22	0.90
19	18	0.98
23	27	1.14
17	23	1.34
22	15	0.69
22	18	0.85
23	17	0.71
19	15	0.78
20	21	1.03
17	25	1.44
24	22	0.93
9	21	2.26
19	16	0.87
15	16	1.07
16	16	0.95
13	12	0.92
12	25	2.03
16	26	1.57
16	13	0.84
12	15	1.22

13	17	1.36
14	22	1.51
12	11	0.97
12	11	0.97
14	16	1.12
16	14	0.92
12	13	1.09
12	16	1.29
13	12	0.97
15	15	0.99
11	14	1.26
12	12	0.99
12	13	1.07
14	24	1.76
14	20	1.47
14	15	1.06
15	17	1.11
4	13	2.97
13	12	0.90
15	33	2.14
14	15	1.08
14	14	0.99
14	32	2.19
13	13	0.99
13	14	1.04
13	17	1.37
13	9	0.72
4	14	3.63
12	12	0.97
13	13	0.96
11	11	1.01
9	9	0.99
11	12	1.07
4	9	2.18
10	8	0.80
9	15	1.62
19	55	2.85
12	10	0.87
11	13	1.24
10	10	0.92
10	10	1.06
10	8	0.82
9	10	1.11
10	8	0.82
10	9	0.84
11	9	0.83
8	13	1.60
9	10	1.20
7	16	2.29
7	13	1.95
8	10	1.24
10	10	1.01
9	12	1.38
12	15	1.32
8	8	1.06
9	14	1.48
12	12	0.96
8	10	1.25
8	8	1.04

5	8	1.46
8	9	1.15
8	7	0.86
8	8	1.08
9	7	0.77
8	10	1.26
9	10	1.10
9	10	1.06
9	9	0.98
6	6	0.88
6	7	1.15
8	6	0.84
6	11	1.81
6	12	1.96
7	6	0.90
7	8	1.12
7	7	1.05
8	9	1.20
7	7	1.06
6	7	1.14
7	7	0.99
6	11	1.77
7	7	1.03
6	7	1.17
6	7	1.05
5	6	1.11
6	8	1.34
7	5	0.76
6	6	1.02
6	5	0.83
6	5	0.92
7	7	1.01
4	8	2.03
5	6	1.11
4	6	1.37
5	7	1.25
5	6	1.12
6	5	0.90
3	7	2.56
6	7	1.01
7	5	0.77
7	6	0.84
5	6	1.18
5	5	0.98
5	5	0.89
6	7	1.17
6	6	0.92
3	5	1.76
5	6	1.34
4	4	0.94
5	4	0.88
4	5	1.22
5	5	1.05
5	14	3.10
4	4	0.94
5	6	1.27
3	5	1.52
4	5	1.11
3	6	1.97

4	4	1.06
3	6	1.67
4	6	1.50
3	2	0.76
4	3	0.77
4	5	1.10
4	4	0.91
3	6	2.16
4	11	3.16
4	5	1.28
3	3	1.19
2	2	0.88
3	4	1.04
2	3	1.22
4	4	1.01
2	4	1.72
3	4	1.19
3	4	1.42
3	4	1.11
4	3	0.88
3	3	1.07
4	6	1.58
2	2	1.01
3	4	1.23
3	3	1.06
3	2	0.77
3	4	1.28
2	3	1.26
3	3	0.95
2	4	1.63
2	4	2.16
3	3	0.92
2	2	1.02
3	5	1.82
2	3	1.28
3	3	1.01
3	4	1.28
3	3	1.26
3	3	0.87
3	5	1.73
2	3	1.32
2	2	1.10
3	5	1.78
1	3	1.75
2	2	0.98
3	3	1.26
3	4	1.11
2	4	1.99
2	3	1.08
2	2	0.99
2	5	2.33
1	3	2.69
1	3	4.03
2	4	1.62
3	3	1.10
2	3	1.20
2	2	0.92
2	2	0.86
1	4	2.81

1	1	1.66
2	1	0.95
2	5	2.44
3	4	1.48
2	2	1.08
3	3	1.06
2	5	2.59
2	3	1.34
2	3	1.56
2	2	1.29
2	2	1.35
1	3	2.81
3	3	1.26
3	2	0.87
3	3	1.24
2	4	1.98
2	3	1.36
2	2	1.05
2	2	1.02
2	2	1.14
2	2	1.26
1	2	2.40
1	1	1.12
2	2	0.71
2	3	1.67
2	2	1.18
1	1	1.48
2	5	2.55
2	2	1.07
2	2	0.89
2	2	1.04
2	2	1.08
2	2	1.63
2	3	1.34
1	3	1.90
2	2	1.49
1	2	1.17
2	2	1.31
2	2	1.40
2	2	1.11
1	2	1.25
2	2	1.07
2	2	0.96
1	2	1.36
2	3	1.63
1	3	2.40
1	2	1.22
1	3	2.53
1	2	1.34
1	2	1.64
1	2	2.03
1	2	1.50
1	3	2.22
1	2	1.62
1	2	1.48
1	1	1.30
1	2	1.19
1	2	3.47
1	3	2.80

1	2	1.50
1	2	1.42
1	1	2.49
1	3	2.59
2	1	0.84
1	2	1.25
1	1	1.22
2	2	1.48
1	2	1.32
1	2	2.79
1	1	1.57
1	1	2.47
1	1	1.14
1	1	1.54
1	1	1.43
1	1	1.26
1	1	1.21
1	2	1.64
1	2	2.13
1	2	1.37
2	2	1.01
1	1	1.57
1	2	1.31
1	2	1.18
1	1	0.99
1	2	1.44
1	2	1.40
1	2	1.43
1	2	1.53
1	1	1.04
1	1	1.03
1	2	1.37
1	1	1.35
1	1	1.70
1	1	1.53
1	1	1.26
1	2	1.77
1	1	1.53
1	1	1.13
1	2	1.37
1	2	2.05
1	1	1.07
1	2	2.05
1	1	1.66
1	1	1.89
1	2	1.55
1	1	1.22
1	2	2.15
1	1	1.43
1	3	2.02
1	1	1.11
1	1	1.09

ave UCs	sd UCs	sd/ave
11.62	4.46	0.38
3.27	3.41	1.04
2.11	1.33	0.63
2.45	1.01	0.41
2.16	0.90	0.42
1.19	1.11	0.93
1.17	0.63	0.54
0.34	0.55	1.62
0.59	0.50	0.85
0.76	0.39	0.51
0.47	0.27	0.56
0.49	0.11	0.23
0.36	0.17	0.49
0.47	0.17	0.37
0.22	0.22	0.99
0.30	0.23	0.77
0.16	0.16	1.01
0.21	0.10	0.47
0.18	0.11	0.62
0.23	0.10	0.44
0.16	0.06	0.40
0.15	0.07	0.43
0.12	0.08	0.68
0.12	0.11	0.89
0.13	0.17	1.30
0.11	0.05	0.46
0.14	0.06	0.45
0.11	0.10	0.92
0.11	0.05	0.48
0.10	0.04	0.42
0.09	0.04	0.47
0.12	0.05	0.42
0.06	0.07	1.11
0.07	0.18	2.50
0.09	0.04	0.47
0.14	0.16	1.14
0.11	0.08	0.75
0.09	0.10	1.05
0.10	0.08	0.84
0.10	0.12	1.24
0.08	0.05	0.64
0.07	0.03	0.48
0.06	0.02	0.38
0.04	0.04	1.08
0.06	0.05	0.75
0.05	0.01	0.30
0.04	0.02	0.61
0.05	0.03	0.61
0.01	0.03	2.50
0.03	0.04	1.45
0.04	0.02	0.52
0.03	0.03	1.00

0.04	0.01	0.36
0.03	0.02	0.65
0.02	0.02	0.72
0.01	0.02	2.61
0.03	0.01	0.44
0.02	0.01	0.50
0.03	0.01	0.44
0.04	0.04	0.93
0.02	0.01	0.51
0.02	0.01	0.55
0.01	0.01	1.37
0.01	0.02	3.38
0.03	0.02	0.59
0.02	0.01	0.63
0.01	0.01	1.13
0.01	0.01	0.97
0.01	0.01	0.69
0.03	0.08	2.34
0.02	0.01	0.66
0.02	0.01	0.50
0.01	0.01	1.06
0.01	0.01	0.55
0.01	0.01	0.73
0.02	0.01	0.70
0.01	0.01	0.57
0.02	0.04	1.79
0.02	0.01	0.62
0.01	0.01	0.72
0.01	0.01	0.46
0.01	0.01	0.57
0.02	0.01	0.65
0.01	0.02	1.96
0.01	0.01	0.75
0.01	0.01	0.64
0.01	0.01	0.38
0.01	0.01	0.99
0.01	0.00	0.45
0.01	0.01	0.68
0.01	0.00	0.49
0.00	0.01	3.17
0.01	0.00	0.57
0.01	0.00	0.86
0.00	0.01	1.43
0.01	0.01	0.84
0.01	0.00	0.53
0.01	0.00	0.38
0.01	0.00	0.57
0.00	0.00	1.23
0.01	0.01	0.98
0.00	0.00	0.50
0.01	0.00	0.62
0.01	0.01	1.44
0.00	0.00	0.65
0.00	0.00	1.02
0.01	0.00	0.61
0.01	0.01	1.03
0.00	0.00	0.84
0.00	0.00	0.75
0.00	0.00	0.67

0.00	0.00	0.42
0.00	0.00	0.73
0.00	0.00	0.75
0.00	0.00	0.68
0.00	0.00	0.48
0.00	0.00	0.49
0.00	0.00	0.74
0.00	0.00	0.74
0.00	0.00	0.83
0.00	0.00	0.78
0.00	0.00	0.49
0.00	0.00	1.04
0.00	0.00	0.83
0.00	0.00	2.55
0.00	0.00	0.60
0.00	0.00	0.72
0.00	0.00	0.66
0.00	0.00	0.87
0.00	0.00	1.23
0.00	0.00	1.32
0.00	0.00	0.58
0.00	0.00	0.65
0.00	0.00	0.90
0.00	0.00	1.16
0.00	0.00	0.50
0.00	0.00	0.55
0.00	0.00	0.61
0.00	0.00	0.51
0.00	0.00	0.53
0.00	0.00	0.48
0.00	0.00	0.47
0.00	0.00	1.08
0.00	0.00	0.71
0.00	0.00	1.62
0.00	0.00	0.57
0.00	0.00	0.60
0.00	0.00	0.72
0.00	0.00	0.73
0.00	0.00	0.73
0.00	0.00	0.96
0.00	0.00	0.73
0.00	0.00	1.09
0.00	0.00	0.45
0.00	0.00	1.24
0.00	0.00	0.50
0.00	0.00	1.21
0.00	0.00	0.68
0.00	0.00	0.61
0.00	0.00	0.65
0.00	0.00	2.63
0.00	0.00	0.55
0.00	0.00	0.73
0.00	0.00	1.10
0.00	0.00	1.00
0.00	0.00	0.66
0.00	0.00	0.61
0.00	0.00	1.21
0.00	0.00	1.56
0.00	0.00	0.70

0.00	0.00	0.88
0.00	0.00	0.64
0.00	0.00	0.38
0.00	0.00	0.79
0.00	0.00	1.44
0.00	0.00	0.99
0.00	0.00	1.21
0.00	0.00	0.55
0.00	0.00	0.85
0.00	0.00	0.81
0.00	0.00	0.62
0.00	0.00	1.25
0.00	0.00	1.10
0.00	0.00	0.83
0.00	0.00	0.62
0.00	0.00	0.55
0.00	0.00	1.23
0.00	0.00	1.09
0.00	0.00	0.76
0.00	0.00	0.76
0.00	0.00	2.37
0.00	0.00	1.51
0.00	0.00	0.63
0.00	0.00	0.92
0.00	0.00	0.49
0.00	0.00	0.70
0.00	0.00	2.46
0.00	0.00	0.46
0.00	0.00	0.81
0.00	0.00	0.85
0.00	0.00	0.90
0.00	0.00	1.08
0.00	0.00	0.62
0.00	0.00	0.53
0.00	0.00	1.00
0.00	0.00	1.13
0.00	0.00	0.53
0.00	0.00	0.52
0.00	0.00	0.76
0.00	0.00	0.57
0.00	0.00	0.87
0.00	0.00	0.72
0.00	0.00	1.37
0.00	0.00	0.58
0.00	0.00	0.72
0.00	0.00	0.57
0.00	0.00	0.63
0.00	0.00	0.86
0.00	0.00	3.05
0.00	0.00	0.62
0.00	0.00	1.46
0.00	0.00	0.52
0.00	0.00	0.75
0.00	0.00	0.78
0.00	0.00	0.98
0.00	0.00	0.58
0.00	0.00	0.59
0.00	0.00	0.87
0.00	0.00	0.79

0.00	0.00	0.78
0.00	0.00	0.94
0.00	0.00	0.51
0.00	0.00	1.88
0.00	0.00	0.66
0.00	0.00	0.70
0.00	0.00	0.78
0.00	0.00	0.74
0.00	0.00	1.33
0.00	0.00	1.17
0.00	0.00	0.48
0.00	0.00	1.06
0.00	0.00	0.87
0.00	0.00	1.14
0.00	0.00	0.97
0.00	0.00	0.97
0.00	0.00	0.99
0.00	0.00	0.70
0.00	0.00	0.75
0.00	0.00	1.01
0.00	0.00	0.89
0.00	0.00	0.58
0.00	0.00	0.78
0.00	0.00	0.84
0.00	0.00	1.02
0.00	0.00	1.06
0.00	0.00	1.26
0.00	0.00	0.78
0.00	0.00	0.90
0.00	0.00	2.79

all 95 samples

B_33vC_2	4465
B_33vC_2	4465
B_33vC_2	0.61

ave UCs	sd UCs
0.00	1.00
0.00	1.00
0.00	1.00
0.00	1.00
0.00	1.00
0.00	1.00
0.00	1.00
0.00	1.00

1

	A_11
A_11	
A_16	-0.24
A_2	-0.10
A_4	0.20
B_29	-0.20
B_32	0.02
B_34	0.32
C_1	0.15

0.00	1.00	C_40	-0.10
0.00	1.00	C_41	-0.01
0.00	1.00	C_42	-0.37
0.00	1.00	C_44	-0.07
0.00	1.00	C_46	-0.16
0.00	1.00	C_47	0.24
0.00	1.00	D_16	0.05
0.00	1.00	D_17	-0.27
0.00	1.00	D_18	0.37
0.00	1.00	D_36	0.23
0.00	1.00	D_4	-0.03
0.00	1.00	E_47	-0.33
0.00	1.00	F_43	-0.11
0.00	1.00	F_6	0.17
0.00	1.00	F_7	0.19
0.00	1.00	F_8	0.28
0.00	1.00	G_33	-0.20
0.00	1.00	G_34	0.11
0.00	1.00	G_8	-0.26
0.00	1.00	A_1	-0.29
0.00	1.00	A_13	-0.13
0.00	1.00	A_14	-0.10
0.00	1.00	A_15	0.08
0.00	1.00	A_17	-0.22
0.00	1.00	B_30	-0.34
0.00	1.00	B_33	0.37
0.00	1.00	C_2	0.41
0.00	1.00	C_37	0.13
0.00	1.00	C_38	0.03
0.00	1.00	C_39	0.25
0.00	1.00	C_43	0.10
0.00	1.00	C_48	-0.03
0.00	1.00	D_10	0.43
0.00	1.00	D_12	0.38
0.00	1.00	D_13	0.30
0.00	1.00	D_14	0.11
0.00	1.00	D_3	-0.04
0.00	1.00	D_9	0.36
0.00	1.00	E_20	-0.18
0.00	1.00	E_29	0.13
0.00	1.00	E_4	0.03
0.00	1.00	E_5	0.13
0.00	1.00	E_6	0.02
0.00	1.00	F_1	0.30
0.00	1.00	F_35	0.28
0.00	1.00	F_36	0.05
0.00	1.00	F_37	-0.05

0.00	1.00
0.00	1.00
0.00	1.00
0.00	1.00
0.00	1.00
0.00	1.00
0.00	1.00
0.00	1.00
0.00	1.00
0.00	1.00
0.00	1.00
0.00	1.00
0.00	1.00
0.00	1.00
0.00	1.00
0.00	1.00
0.00	1.00
0.00	1.00
0.00	1.00
0.00	1.00
0.00	1.00
0.00	1.00
0.00	1.00
0.00	1.00
0.00	1.00
0.00	1.00
0.00	1.00
0.00	1.00
0.00	1.00
0.00	1.00
0.00	1.00
0.00	1.00
0.00	1.00
0.00	1.00
0.00	1.00
0.00	1.00
0.00	1.00
0.00	1.00
0.00	1.00
0.00	1.00
0.00	1.00
0.00	1.00
0.00	1.00
0.00	1.00
0.00	1.00
0.00	1.00
0.00	1.00
0.00	1.00

F_38	0.21
F_39	0.19
G_3	-0.31
G_30	0.06
G_31	-0.25
G_32	-0.26
G_4	-0.36
G_5	0.05
G_6	-0.11
G_7	-0.26
A_10	0.00
A_12	0.18
A_18	-0.06
A_3	0.13
A_5	0.11
A_6	0.32
B_31	-0.26
C_4	-0.14
C_45	-0.06
D_11	0.41
D_35	-0.15
D_5	-0.17
D_6	-0.21
E_19	-0.07
E_21	-0.31
E_22	-0.03
E_23	-0.16
E_3	0.09
E_46	-0.24
E_48	-0.29
E_7	0.01
E_8	-0.05
E_9	-0.09
F_40	-0.26
F_41	-0.12
F_42	-0.32
F_44	0.08
F_9	0.17
G_10	-0.26
G_9	0.23

	0.00	1.00
	0.00	1.00
	0.00	1.00
	0.00	1.00
	0.00	1.00
	0.00	1.00
	0.00	1.00
	0.00	1.00
	0.00	1.00
	0.00	1.00
	0.00	1.00
	0.00	1.00
	0.00	1.00
	0.00	1.00
	0.00	1.00
	0.00	1.00
	0.00	1.00
	0.00	1.00
	0.00	1.00
	0.00	1.00
	0.00	1.00
	0.00	1.00
	0.00	1.00
	0.00	1.00
	0.00	1.00
	0.00	1.00
	0.00	1.00
	0.00	1.00
	0.00	1.00
	0.00	1.00
	0.00	1.00
	0.00	1.00
	0.00	1.00

28	29	30	31	32	33
96	97	98	99	100	101
AAGGAGCTC	TCCGGTTCTCAGG	CCGCACTGTGGGTACTTGCTGC	TGCCCTGTGGACTCAGT	TAGTGCAATATT	GCCCCTGGGCCTATC
miR-28-5p	miR-671-3p	miR-106b-3p	miR-146b-3p	miR-454-3p	miR-331-3p
28163	28118	27350	27199	2.68E+04	26325.00
-0.76	-1.03	0.24	-0.05	-0.69	4.12
0.69	-0.76	0.97	2.39	-0.81	-1.02
-0.20	-1.30	-1.08	-0.05	-0.15	0.00
-0.78	-1.25	-0.77	-0.45	-0.60	-0.06
1.80	1.93	-1.91	1.49	-0.03	0.08
-0.03	2.02	-0.53	-1.03	-0.48	-0.99
-0.24	1.44	0.01	-0.60	-0.61	-0.14
-0.50	1.26	2.00	-0.43	-0.68	-0.46
-0.88	-0.98	-1.08	-1.26	-0.41	-0.51
-0.73	-1.16	-1.09	-1.14	0.65	-0.14
0.54	-0.71	-1.32	0.08	0.26	-0.59
-0.10	0.50	-0.10	-0.45	-0.68	-0.95
3.20	-0.51	-0.40	-1.03	3.65	-0.08
-0.81	0.18	0.69	0.04	-0.62	0.11
-0.33	-0.15	-0.11	-0.85	0.82	0.56
1.85	0.04	-0.05	-0.77	1.32	-0.43
-0.46	0.15	0.29	0.98	-0.62	0.47
0.08	-1.06	1.93	-0.80	0.04	1.30
-0.02	-0.14	0.37	0.03	-0.10	1.24
1.51	-0.77	-0.49	-0.75	1.58	-0.39

-0.39	0.58	-0.20	0.13	-0.24	-0.77
-0.67	0.92	1.40	1.01	-0.70	-0.27
-0.41	0.85	1.59	1.26	-0.55	-0.11
-0.58	0.84	0.76	0.74	-0.65	-0.20
-0.60	0.69	0.34	0.24	-0.50	-0.59
-0.69	-0.56	-0.61	2.14	-0.44	0.03
-0.48	-1.03	-0.86	-0.86	1.26	-0.23
0.93	-1.52	-1.86	-1.28	2.44	-0.50
0.38	-0.16	-0.77	1.91	-0.36	-0.38
-0.84	-0.90	-0.45	0.47	-0.50	-0.08
-1.13	-0.21	0.81	-0.50	-0.61	-0.46
-0.49	-0.56	-0.19	1.46	-0.34	-0.05
1.90	-0.59	-1.30	-1.02	1.18	0.58
-0.32	0.18	0.66	-1.10	-0.56	1.00
-0.30	0.25	1.08	-0.75	-0.49	2.00
-0.26	0.25	-0.82	0.43	-0.60	0.67
-1.08	0.03	-0.27	-1.20	-0.77	-0.94
0.02	-0.64	0.10	0.07	-0.06	0.54
0.60	-1.46	-1.17	-0.83	2.27	1.55
0.73	0.18	-0.09	1.32	-0.58	0.32
0.04	0.80	4.93	3.26	-0.71	6.21
0.48	3.37	16.43	3.64	-0.65	6.75
-0.57	0.31	0.50	0.34	-0.53	-0.07
0.51	-0.10	0.85	-0.84	1.26	0.59
-0.29	3.01	2.29	1.95	-0.56	-0.57
-0.48	1.26	3.86	2.12	-0.78	2.37
1.04	-0.61	-0.56	-0.07	-0.12	-0.23
0.16	1.21	2.49	0.49	-0.61	-0.24
0.18	-0.49	0.70	-0.59	-0.11	-0.34
0.61	-0.41	0.02	0.32	-0.59	-0.36
0.78	-0.45	-0.41	-0.04	0.00	-0.33
-0.70	0.75	-0.16	0.43	-0.65	0.48
-0.80	0.05	-0.24	-0.62	-0.55	0.50
0.54	0.03	-0.11	-0.38	-0.12	0.01
-0.51	1.40	0.60	-0.36	-0.64	-0.73
-0.79	0.02	-0.44	0.29	-0.63	0.14
-0.38	-0.26	-0.39	-0.43	-0.11	-0.59
0.51	-1.38	-1.70	-1.05	5.38	-0.62
-0.41	0.58	0.32	3.02	-0.60	-0.21
1.07	1.25	0.94	1.40	0.34	-0.30
1.65	0.18	-0.87	1.84	0.19	0.07
-0.05	-1.48	-1.50	-1.26	6.59	-0.65
-0.97	-0.75	0.17	-0.24	-0.64	-0.34
-0.79	-0.29	-0.26	0.05	-0.48	-0.65
-0.65	-1.23	-1.46	-0.63	0.21	-0.63
-0.01	-0.44	3.41	0.13	-0.53	-0.61
-0.33	-1.61	-1.66	-1.24	0.14	0.83
-0.22	-0.14	2.48	2.62	-0.81	-0.59
-0.28	-0.42	1.23	1.64	-0.44	2.93
1.22	-0.11	1.63	1.60	-0.29	1.23
-0.66	-0.51	0.27	-0.22	-0.66	-0.18
2.93	-1.41	2.19	-0.22	0.38	-0.93
-0.10	-1.08	-0.61	-1.00	-0.12	-0.65
1.72	-0.87	-0.20	-0.43	1.49	-0.33
-1.15	4.27	12.40	5.57	-0.69	15.97
1.15	-0.16	-0.34	-0.71	0.96	-0.67
-0.02	0.05	-0.07	1.08	0.04	0.37
-0.44	2.58	2.09	0.60	-0.41	-0.97
-0.28	-1.27	-1.25	-0.85	0.68	1.99

1.02	-0.68	-1.32	-0.80	0.04	-0.27
-0.77	-0.41	-0.41	-0.26	-0.65	-0.05
0.01	-0.46	-0.97	-0.24	-0.37	-0.33
0.45	0.52	1.84	1.07	-0.57	-0.90
1.57	-1.14	-0.83	-0.29	1.76	-0.08
0.43	-1.51	-1.50	-1.37	4.35	-0.69
0.03	0.01	0.08	0.08	-0.49	0.25
0.46	-0.49	-0.42	-0.03	-0.48	-0.20
-0.61	-0.61	0.37	-0.46	-0.57	-0.60
0.55	0.12	-0.43	-0.05	1.26	-0.15
0.06	-0.16	-0.74	0.30	-0.44	0.34
0.04	-0.80	-0.07	-0.15	1.60	-0.96
-0.86	0.63	0.39	0.11	-0.62	-0.43
-0.48	0.98	0.59	0.78	-0.63	-0.24
-0.68	-0.75	-0.98	-0.64	0.06	-0.73
-0.69	-1.33	-0.73	0.60	-0.15	0.44
1	1	-1	0	2.12E-01	-0.15
0.00	0.00	0.00	0.00	0.00	0.00
1.00	1.00	1.00	1.00	1.00	1.00
0.46	0.39	0.98	0.81	0.96	0.90

96	97	98	99	100	101
AAGGAGCTC	TCCGGTTCTCAGG	CCGCACTGTGGGTACTTGCTGC	TGCCCTGTGGACTCAGT	TAGTGCAATATT	GCCCCTGGGCCTATC
miR-28-5p	miR-671-3p	miR-106b-3p	miR-146b-3p	miR-454-3p	miR-331-3p
28163	28118	27350	27199	2.68E+04	26325.00
-0.76	-1.03	0.24	-0.05	-0.69	4.12
0.69	-0.76	0.97	2.39	-0.81	-1.02
-0.20	-1.30	-1.08	-0.05	-0.15	0.00
-0.78	-1.25	-0.77	-0.45	-0.60	-0.06
1.80	1.93	-1.91	1.49	-0.03	0.08
-0.03	2.02	-0.53	-1.03	-0.48	-0.99
-0.24	1.44	0.01	-0.60	-0.61	-0.14
-0.50	1.26	2.00	-0.43	-0.68	-0.46
-0.88	-0.98	-1.08	-1.26	-0.41	-0.51
-0.73	-1.16	-1.09	-1.14	0.65	-0.14
0.54	-0.71	-1.32	0.08	0.26	-0.59
-0.10	0.50	-0.10	-0.45	-0.68	-0.95
3.20	-0.51	-0.40	-1.03	3.65	-0.08
-0.81	0.18	0.69	0.04	-0.62	0.11
-0.33	-0.15	-0.11	-0.85	0.82	0.56
1.85	0.04	-0.05	-0.77	1.32	-0.43
-0.46	0.15	0.29	0.98	-0.62	0.47
0.08	-1.06	1.93	-0.80	0.04	1.30
-0.02	-0.14	0.37	0.03	-0.10	1.24
1.51	-0.77	-0.49	-0.75	1.58	-0.39
-0.39	0.58	-0.20	0.13	-0.24	-0.77
-0.67	0.92	1.40	1.01	-0.70	-0.27
-0.41	0.85	1.59	1.26	-0.55	-0.11
-0.58	0.84	0.76	0.74	-0.65	-0.20
-0.60	0.69	0.34	0.24	-0.50	-0.59
-0.69	-0.56	-0.61	2.14	-0.44	0.03
-0.48	-1.03	-0.86	-0.86	1.26	-0.23
0.93	-1.52	-1.86	-1.28	2.44	-0.50

0.38	-0.16	-0.77	1.91	-0.36	-0.38
-0.84	-0.90	-0.45	0.47	-0.50	-0.08
-1.13	-0.21	0.81	-0.50	-0.61	-0.46
-0.49	-0.56	-0.19	1.46	-0.34	-0.05
1.90	-0.59	-1.30	-1.02	1.18	0.58
-0.32	0.18	0.66	-1.10	-0.56	1.00
-0.30	0.25	1.08	-0.75	-0.49	2.00
-0.26	0.25	-0.82	0.43	-0.60	0.67
-1.08	0.03	-0.27	-1.20	-0.77	-0.94
0.02	-0.64	0.10	0.07	-0.06	0.54
0.60	-1.46	-1.17	-0.83	2.27	1.55
0.73	0.18	-0.09	1.32	-0.58	0.32
0.04	0.80	4.93	3.26	-0.71	6.21
0.48	3.37	16.43	3.64	-0.65	6.75
-0.57	0.31	0.50	0.34	-0.53	-0.07
0.51	-0.10	0.85	-0.84	1.26	0.59
-0.29	3.01	2.29	1.95	-0.56	-0.57
-0.48	1.26	3.86	2.12	-0.78	2.37
1.04	-0.61	-0.56	-0.07	-0.12	-0.23
0.16	1.21	2.49	0.49	-0.61	-0.24
0.18	-0.49	0.70	-0.59	-0.11	-0.34
0.61	-0.41	0.02	0.32	-0.59	-0.36
0.78	-0.45	-0.41	-0.04	0.00	-0.33
-0.70	0.75	-0.16	0.43	-0.65	0.48
-0.80	0.05	-0.24	-0.62	-0.55	0.50
0.54	0.03	-0.11	-0.38	-0.12	0.01
-0.51	1.40	0.60	-0.36	-0.64	-0.73
-0.79	0.02	-0.44	0.29	-0.63	0.14
-0.38	-0.26	-0.39	-0.43	-0.11	-0.59
0.51	-1.38	-1.70	-1.05	5.38	-0.62
-0.41	0.58	0.32	3.02	-0.60	-0.21
1.07	1.25	0.94	1.40	0.34	-0.30
1.65	0.18	-0.87	1.84	0.19	0.07
-0.05	-1.48	-1.50	-1.26	6.59	-0.65
-0.97	-0.75	0.17	-0.24	-0.64	-0.34
-0.79	-0.29	-0.26	0.05	-0.48	-0.65
-0.65	-1.23	-1.46	-0.63	0.21	-0.63
-0.01	-0.44	3.41	0.13	-0.53	-0.61
-0.33	-1.61	-1.66	-1.24	0.14	0.83
-0.22	-0.14	2.48	2.62	-0.81	-0.59
-0.28	-0.42	1.23	1.64	-0.44	2.93
1.22	-0.11	1.63	1.60	-0.29	1.23
-0.66	-0.51	0.27	-0.22	-0.66	-0.18
2.93	-1.41	2.19	-0.22	0.38	-0.93
-0.10	-1.08	-0.61	-1.00	-0.12	-0.65
1.72	-0.87	-0.20	-0.43	1.49	-0.33
-1.15	4.27	12.40	5.57	-0.69	15.97
1.15	-0.16	-0.34	-0.71	0.96	-0.67
-0.02	0.05	-0.07	1.08	0.04	0.37
-0.44	2.58	2.09	0.60	-0.41	-0.97
-0.28	-1.27	-1.25	-0.85	0.68	1.99
1.02	-0.68	-1.32	-0.80	0.04	-0.27
-0.77	-0.41	-0.41	-0.26	-0.65	-0.05
0.01	-0.46	-0.97	-0.24	-0.37	-0.33
0.45	0.52	1.84	1.07	-0.57	-0.90
1.57	-1.14	-0.83	-0.29	1.76	-0.08
0.43	-1.51	-1.50	-1.37	4.35	-0.69
0.03	0.01	0.08	0.08	-0.49	0.25
0.46	-0.49	-0.42	-0.03	-0.48	-0.20

-0.61	-0.61	0.37	-0.46	-0.57	-0.60
0.55	0.12	-0.43	-0.05	1.26	-0.15
0.06	-0.16	-0.74	0.30	-0.44	0.34
0.04	-0.80	-0.07	-0.15	1.60	-0.96
-0.86	0.63	0.39	0.11	-0.62	-0.43
-0.48	0.98	0.59	0.78	-0.63	-0.24
-0.68	-0.75	-0.98	-0.64	0.06	-0.73
-1	-1	-1	1	-1.49E-01	0.44

96	97	98	99	100	101
37	28	8	79	5	22
CTAGACTGA	TTTGGCAATGGTA	AAACCGTTACCATTACTGAGTT	TGCGGGGCTAGGGCTAA	AACATTCAACGC	CAAAGTGCTGTTCGT
miR-151a-	miR-182-5p	miR-451a	miR-744-5p	miR-181a-5p	miR-93-5p
393199	501331	3762724	56410	8658137	679306
-0.32	-0.73	-0.50	-0.65	-0.18	1.24
2.84	2.95	-0.26	-1.18	-1.73	-1.25
-0.21	0.51	0.10	-1.00	-0.76	2.48
-0.91	-0.18	-0.40	-0.95	-1.23	1.64
0.42	-0.74	-0.49	1.81	-0.08	-0.94
-0.66	-0.97	-0.60	0.20	-0.44	-1.32
-0.55	-0.81	-0.58	1.91	1.03	0.26
-0.95	-0.91	-0.56	0.69	0.65	-0.80
0.50	0.19	-0.23	-1.00	-1.26	-0.23
1.26	1.05	-0.03	-1.21	-1.64	0.26
2.90	1.63	0.30	-0.98	-1.00	-0.93
-0.74	-0.90	-0.54	1.97	0.27	-1.03
0.28	-0.11	2.47	-0.38	-0.24	0.95
-0.71	-0.82	-0.53	0.36	0.88	-0.38
-0.40	0.37	-0.08	-0.34	-0.49	1.27
0.15	0.30	0.07	-0.28	-0.42	-0.06
-0.80	-0.76	-0.52	-0.15	1.77	0.22
-0.04	-0.15	0.68	1.42	0.05	1.62
0.81	0.57	0.02	-0.88	0.11	-0.44
-0.23	0.84	3.77	-0.79	-0.29	-0.54
-0.32	0.18	-0.51	-0.75	0.23	-0.27
-0.77	-0.60	-0.56	1.07	1.38	-0.39
-0.57	-0.56	-0.47	0.67	1.21	-0.20
-0.90	-0.80	-0.54	0.57	1.19	-0.71
0.16	-0.74	-0.52	0.10	0.34	-1.27
-0.52	-0.77	-0.49	0.62	1.77	0.19
0.32	1.97	0.97	-0.86	-1.13	0.61
0.29	1.52	5.14	-1.22	-1.79	-0.21
1.54	-0.11	-0.37	-1.10	-1.47	-0.53
1.58	1.28	-0.14	-1.14	-1.71	0.28
1.04	0.47	-0.53	-1.10	-1.76	1.77
6.29	4.08	0.53	-1.26	-1.76	0.04
1.58	-0.61	0.31	0.42	-1.26	-1.04
-0.84	-0.58	-0.47	0.45	1.07	0.09
-0.26	0.02	0.14	-0.20	0.40	0.76
-0.68	-0.99	-0.52	-0.06	0.31	-0.65
-1.17	-1.00	-0.60	0.37	-1.01	-1.72
0.75	0.00	0.00	-1.13	0.48	0.49
1.50	0.77	10.00	-1.07	0.26	2.86

-0.52	-0.76	-0.41	-0.29	2.00	-0.56
-0.75	-0.46	-0.41	1.74	2.23	-1.02
0.05	-0.36	0.15	3.41	4.38	-0.94
-0.61	-0.84	-0.55	0.19	0.78	-0.17
0.01	0.25	0.38	-0.68	-0.78	1.30
-0.04	-0.78	-0.51	0.48	0.14	-1.23
-1.03	-0.79	-0.51	1.62	3.61	-1.70
-0.11	-0.36	-0.04	-0.90	-0.03	-0.60
-0.17	-0.60	-0.46	0.18	1.96	-0.63
-0.39	0.17	0.28	-0.50	0.15	-0.22
-0.66	-0.48	-0.29	-0.63	0.70	-0.56
-0.01	-0.33	0.11	-0.65	1.01	-0.42
-0.54	-0.87	-0.55	0.09	-0.55	-0.24
-0.47	-0.40	-0.53	-0.23	-1.08	0.04
-0.58	-0.70	-0.42	0.40	-0.49	0.46
-0.53	-0.51	-0.57	-0.02	-0.24	-1.14
-0.61	-0.95	-0.55	-0.53	-0.11	-0.52
-0.48	-0.38	-0.50	-0.67	-0.83	-0.27
-0.48	1.33	6.18	-0.92	-0.57	0.02
-0.52	-0.78	-0.52	0.46	2.54	0.31
0.45	-0.33	-0.19	0.74	1.40	0.22
0.30	-0.08	-0.08	0.38	1.09	-0.67
-0.19	2.29	4.07	-1.10	-0.97	0.60
-0.98	-0.40	-0.45	-0.20	0.62	-0.01
-0.81	-0.24	-0.41	-0.09	-0.19	-0.18
-0.22	-0.66	0.00	-0.88	-0.36	0.28
-0.51	2.73	-0.09	0.99	1.09	2.75
0.44	0.19	1.39	-1.41	-1.78	1.74
5.63	1.31	-0.36	-1.39	-1.67	0.80
0.04	0.00	-0.28	0.89	0.55	4.05
0.19	0.08	-0.15	0.34	0.95	3.41
-0.78	-0.68	-0.45	-0.49	-0.87	1.24
0.70	1.99	-0.15	-1.21	-1.31	-1.31
-0.58	0.23	-0.20	-0.74	-0.99	0.15
-0.11	0.23	1.13	-0.23	-0.06	0.58
-1.00	-0.94	-0.37	4.02	2.07	-1.85
-0.58	-0.41	-0.19	0.18	-0.32	-0.09
1.53	0.84	-0.01	-1.01	-0.77	-0.41
2.28	1.12	-0.51	-0.85	-0.82	-1.23
0.36	-0.22	1.56	-1.00	-1.07	1.72
-0.01	0.04	0.25	-1.02	-0.75	-0.78
-0.57	-0.46	-0.39	-0.71	-0.23	-0.68
-0.60	-0.46	-0.17	-0.84	-0.18	-0.43
-0.87	-0.89	-0.47	0.87	0.81	-1.32
-0.30	0.61	7.12	-0.90	0.29	-0.58
-0.46	1.49	8.65	-1.28	-1.15	-0.40
0.15	-0.92	0.18	-0.48	0.16	-0.99
-0.63	-0.67	-0.35	-0.64	0.27	-0.36
-0.68	-0.21	-0.45	-0.99	-0.10	-0.29
-0.09	0.40	0.49	-0.73	-0.51	-0.14
-0.63	-0.66	-0.37	-0.32	-0.44	-0.45
-0.47	3.43	0.60	-0.62	-0.33	0.38
-0.81	-0.79	-0.56	-1.01	-0.42	-0.40
-0.94	-0.86	-0.55	0.54	1.73	-0.29
0.53	0.41	-0.31	-1.06	-0.45	0.40
0.60	0.69	-0.14	-1.04	0.16	1.86
0.58	0.60	0.62	0.65	0.66	0.67
0.53	1.00	1.00	1.00	0.45	0.78

reselects		
typical UC	typical NP	typical P
43.19	33.63	18.79

Student
trials
1000
100
10

no relabeling_but_resele

sd/ave
1.23
0.88
1.22
0.89
0.84
0.87
1.54

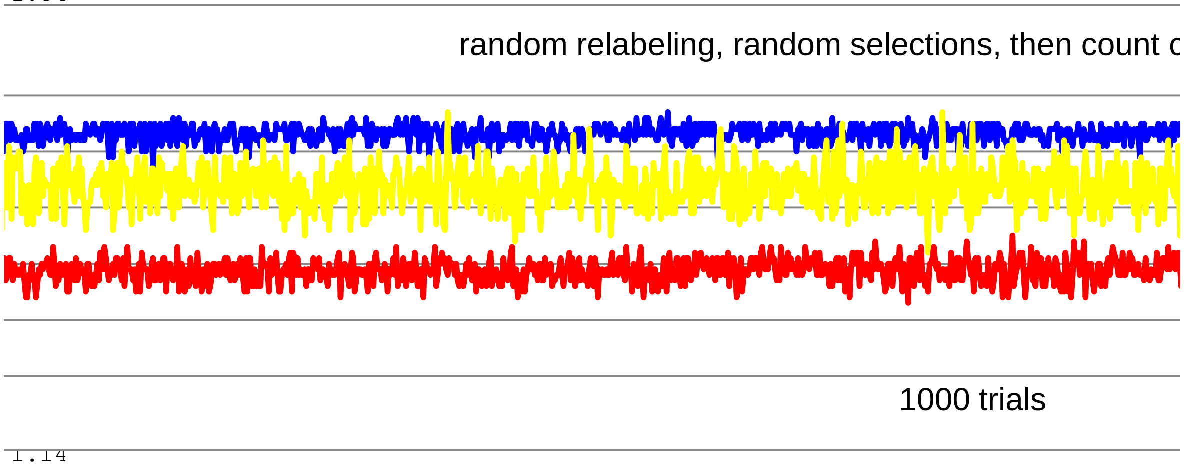

1.14
1.11
1.27
1.53
1.32
1.02
1.00
1.20
0.95
0.86
1.15
0.89
1.84
3.62
0.96
0.92
1.06
1.08
1.12
1.30
1.06
0.88
0.86
1.64
0.77

0.89
1.08
0.97
2.91
2.11
1.00
1.65
0.98
1.18
1.11
2.94
1.15
1.16
0.90
0.93
1.21
1.23
1.90
3.74
0.94
1.29
1.96
1.55
1.48
1.31
0.88
0.92
1.87
1.18
1.16
1.15
1.20
0.90
0.97
1.25
1.07
0.90
0.97
2.90
1.32
1.04
0.80
1.60
1.13
1.07
0.91
3.35
0.97
1.38
2.24
1.65
0.93
0.90
0.88
1.98
1.23
1.01
0.83
1.03

1.29		
1.66		
0.99		
1.00		
1.71		
1.44		
1.31		
1.00		
1.21		
1.33	selection criteria:	
1.02		
0.97		
1.01	min	max
1.37	4	685
1.37	4	685
1.02	2	548
0.89	1	399
0.96	0	518
1.49	0	664
1.41	3	657
2.83		
0.92	2	443
0.84		
1.05	0	434
0.96		
1.07		
1.53		
1.19		
1.11		
1.03		
1.88		
1.03		
0.90		
0.99		
1.08		
0.95		
0.81		
1.18		
1.00		
0.98		
2.30		
1.04		
0.96		
1.27		
0.83		
1.13		
1.59		
1.25		
1.23		
0.93		
1.53		
0.96		
1.33		
0.93		
0.89		
1.08		
2.93		
1.06		
0.92		

2.03
1.79
0.81
0.91
1.63
1.96
0.91
1.54
1.09
0.83
1.46
2.12
1.16
1.27
1.09
1.21
1.06
0.96
1.91
1.14
1.41
0.98
0.84
1.90
1.39
1.21
1.25
1.72
2.33
1.17
1.00
0.93
0.97
2.57
0.97
1.40
1.21
1.13
0.97
0.91
1.08
1.60
1.27
1.00
1.05
1.26
0.90
1.03
1.34
1.50
0.97
1.17
1.00
1.11
1.23
2.97
1.03
1.44
1.01

1.09
1.24
1.45
0.80
0.96
0.81
0.89
1.14
1.54
1.03
2.39
0.97
1.17
1.06
1.03
2.15
1.69
0.94
1.33
1.47
1.62
1.06
1.06
1.22
1.02
1.18
1.40
1.08
1.09
1.34
1.10
1.18
1.88
1.59
1.15
1.22
3.15
1.00
2.27
1.19
1.10
2.33
1.08
1.14
1.48
0.83
3.80
1.07
1.06
1.11
1.09
1.18
2.32
0.91
1.74
3.02
0.97
1.35
1.03

1.16
0.93
1.21
0.92
0.95
0.94
1.72
1.29
2.42
2.07
1.34
1.11
1.49
1.42
1.15
1.60
1.06
1.36
1.15
1.57
1.26
0.97
1.19
0.87
1.35
1.21
1.17
1.09
0.99
1.25
0.95
1.91
2.09
1.00
1.22
1.15
1.30
1.16
1.25
1.09
1.89
1.13
1.28
1.15
1.22
1.44
0.87
1.12
0.93
1.03
1.12
2.16
1.22
1.48
1.36
1.23
1.00
2.56
1.10

0.82
0.94
1.29
1.08
0.98
1.28
1.02
1.88
1.45
1.03
0.99
1.33
1.15
3.28
1.05
1.38
1.63
1.22
2.10
1.17
1.79
1.59
0.86
0.86
1.20
1.02
2.30
3.34
1.39
1.30
0.98
1.15
1.33
1.12
1.84
1.30
1.54
1.21
0.98
1.18
1.70
1.11
1.34
1.16
0.88
1.39
1.36
1.05
1.75
2.30
1.03
1.12
1.94
1.39
1.11
1.39
1.37
0.97
1.85

1.43
1.21
1.91
1.87
1.08
1.34
1.22
2.12
1.19
1.06
2.47
2.76
4.26
1.74
1.21
1.30
1.03
0.96
2.97
1.78
1.05
2.59
1.60
1.19
1.16
2.75
1.45
1.67
1.40
1.46
2.98
1.37
0.95
1.35
2.11
1.47
1.16
1.12
1.22
1.36
2.55
1.23
0.82
1.79
1.29
1.59
2.71
1.18
0.99
1.14
1.18
1.75
1.45
2.03
1.60
1.28
1.42
1.51
1.22

1.36
1.17
1.06
1.47
1.75
2.55
1.32
2.68
1.45
1.76
2.16
1.62
2.36
1.73
1.60
1.41
1.29
3.67
2.96
1.62
1.54
2.64
2.75
0.95
1.36
1.33
1.59
1.43
2.96
1.68
2.62
1.25
1.65
1.54
1.37
1.31
1.76
2.26
1.48
1.12
1.69
1.37
1.28
1.10
1.56
1.51
1.54
1.59
1.15
1.14
1.48
1.46
1.82
1.64
1.37
1.90
1.64
1.24
1.49

2.18
1.13
2.18
1.78
2.01
1.66
1.32
2.28
1.54
2.15
1.22
1.15

0.22

1.00	2	3	4
XX UCs,	counts shown are exact	0	each miR ranked by total
0.00	0	0	0
index ty	total of totals of =100	139641984	total all miRNA reads =1
1.00	UC	A_11	479320
2.00	UC	A_16	21319
3.00	UC	A_2	1102300
4.00	UC	A_4	1969971
5.00	UC	B_29	71054
6.00	UC	B_32	356562
7.00	UC	B_34	1363063
8.00	UC	C_1	824884
9.00	UC	C_40	2206918
10.00	UC	C_41	623719
11.00	UC	C_42	774447
12.00	UC	C_44	627333
13.00	UC	C_46	2772220
14.00	UC	C_47	1217409
15.00	UC	D_16	4032681
16.00	UC	D_17	1739788
17.00	UC	D_18	1227867
18.00	UC	D_36	121631
19.00	UC	D_4	2029037
20.00	UC	E_47	1734052
21.00	UC	F_43	1026638
22.00	UC	F_6	1873959
23.00	UC	F_7	1687678
24.00	UC	F_8	1238990
25.00	UC	G_33	121924
26.00	UC	G_34	416015
27.00	UC	G_8	1782397
1.00	CHR_NP	A_1	2570804
2.00	CHR_NP	A_13	371228
3.00	CHR_NP	A_14	359180
4.00	CHR_NP	A_15	111957
5.00	CHR_NP	A_17	117100
6.00	CHR_NP	B_30	148505
7.00	CHR_NP	B_33	1922117
8.00	CHR_NP	C_2	685397
9.00	CHR_NP	C_37	2915691
10.00	CHR_NP	C_38	932164
11.00	CHR_NP	C_39	148130
12.00	CHR_NP	C_43	2018570
13.00	CHR_NP	C_48	2072241
14.00	CHR_NP	D_10	727386
15.00	CHR_NP	D_12	206047
16.00	CHR_NP	D_13	2758841
17.00	CHR_NP	D_14	2173631
18.00	CHR_NP	D_3	852941
19.00	CHR_NP	D_9	239935
20.00	CHR_NP	E_20	1893208
21.00	CHR_NP	E_29	1240451
22.00	CHR_NP	E_4	2304775
23.00	CHR_NP	E_5	1597470

24.00	CHR_NP	E_6	1583268
25.00	CHR_NP	F_1	2592561
26.00	CHR_NP	F_35	3565545
27.00	CHR_NP	F_36	2585170
28.00	CHR_NP	F_37	976547
29.00	CHR_NP	F_38	1738984
30.00	CHR_NP	F_39	1856730
31.00	CHR_NP	G_3	2304707
32.00	CHR_NP	G_30	924323
33.00	CHR_NP	G_31	555209
34.00	CHR_NP	G_32	358914
35.00	CHR_NP	G_4	3926455
36.00	CHR_NP	G_5	1257195
37.00	CHR_NP	G_6	488065
38.00	CHR_NP	G_7	1421920
1.00	CHR_P	A_10	492624
2.00	CHR_P	A_12	721119
3.00	CHR_P	A_18	27468
4.00	CHR_P	A_3	816795
5.00	CHR_P	A_5	972175
6.00	CHR_P	A_6	1468719
7.00	CHR_P	B_31	409595
8.00	CHR_P	C_4	1862148
9.00	CHR_P	C_45	4820581
10.00	CHR_P	D_11	198165
11.00	CHR_P	D_35	2718285
12.00	CHR_P	D_5	905697
13.00	CHR_P	D_6	128375
14.00	CHR_P	E_19	4686947
15.00	CHR_P	E_21	1336672
16.00	CHR_P	E_22	1032612
17.00	CHR_P	E_23	1495548
18.00	CHR_P	E_3	331698
19.00	CHR_P	E_46	1653485
20.00	CHR_P	E_48	7479891
21.00	CHR_P	E_7	1160935
22.00	CHR_P	E_8	2099567
23.00	CHR_P	E_9	1323600
24.00	CHR_P	F_40	3571995
25.00	CHR_P	F_41	3076499
26.00	CHR_P	F_42	1136044
27.00	CHR_P	F_44	2580829
28.00	CHR_P	F_9	1797912
29.00	CHR_P	G_10	999373
30.00	CHR_P	G_9	336028
0.00	0	0	0
0.00	0	0	0
0.00	0	0	0

rank 73
percenti 0.98

2 3 4 5

A_16	*A_2*	*A_4*	*B_29*
0.08			
-0.04	0.66		
0.21	-0.07	-0.29	
-0.14	-0.29	-0.05	0.04
-0.34	-0.47	-0.16	-0.08
-0.10	-0.41	-0.07	-0.14

0.32	0.42	0.34	-0.11
0.36	0.37	0.30	0.06
0.39	0.10	-0.18	0.08
-0.19	-0.31	-0.03	-0.10
-0.21	0.08	-0.03	-0.09
-0.09	-0.32	0.00	-0.11
-0.14	0.46	0.30	-0.26
-0.23	0.08	-0.22	-0.07
-0.33	-0.24	-0.11	-0.14
-0.30	0.15	0.09	-0.22
-0.03	-0.12	-0.18	-0.21
-0.08	0.20	0.06	-0.23
-0.02	-0.01	0.17	-0.18
-0.31	-0.41	-0.20	-0.18
-0.34	-0.36	-0.13	-0.13
-0.22	-0.34	0.00	-0.12
0.34	-0.18	-0.28	0.49
-0.16	-0.18	-0.35	0.27
0.03	0.42	0.13	-0.29
0.02	0.27	0.15	-0.18
0.23	0.04	-0.02	-0.13
0.47	0.22	0.20	-0.20
0.42	0.08	0.14	-0.02
0.45	0.15	-0.03	-0.10
0.25	0.20	-0.11	0.67
-0.23	-0.32	0.00	-0.13
-0.12	-0.16	-0.03	0.05
-0.16	-0.27	-0.10	0.04
0.05	-0.08	0.26	-0.17
-0.13	-0.08	0.01	-0.16
-0.13	0.11	-0.02	-0.13
-0.20	-0.34	-0.36	0.13
-0.12	-0.32	-0.18	0.00
-0.18	-0.26	-0.18	-0.02
-0.20	-0.41	-0.29	-0.05
-0.19	0.01	0.01	-0.18
-0.03	-0.47	-0.32	-0.09
-0.13	-0.35	-0.19	0.03
-0.17	0.01	-0.22	0.07
-0.31	-0.46	-0.39	-0.15
-0.21	0.04	0.01	-0.14
-0.28	-0.19	-0.18	-0.03
-0.28	-0.07	-0.25	-0.13
-0.07	-0.26	-0.24	0.00
-0.02	-0.07	0.02	-0.02
-0.26	-0.07	-0.19	0.22
-0.12	-0.28	-0.02	-0.14

0.04	-0.10	-0.06	-0.01
-0.07	0.05	0.03	-0.06
-0.06	0.21	0.09	-0.17
-0.11	-0.22	-0.27	0.14
-0.07	-0.03	-0.33	0.40
0.06	0.00	-0.33	0.61
0.00	0.36	0.16	-0.19
0.05	0.07	0.12	-0.13
-0.05	0.14	0.09	-0.06
-0.06	0.54	0.26	-0.14
-0.15	0.17	0.32	-0.41
-0.01	0.54	0.46	-0.23
0.61	0.09	0.00	0.12
0.01	0.43	0.39	0.22
-0.12	0.33	0.17	0.14
-0.17	0.17	0.64	-0.41
0.02	0.02	-0.02	-0.10
-0.04	0.60	0.66	-0.15
-0.13	0.14	0.08	-0.03
-0.14	-0.24	-0.14	0.02
-0.37	0.09	0.03	-0.17
0.24	0.01	-0.15	-0.04
0.24	-0.13	-0.21	-0.16
-0.16	0.40	0.14	-0.05
-0.08	0.15	-0.20	0.06
0.03	0.09	-0.04	0.08
-0.08	0.09	-0.09	0.23
-0.25	-0.50	-0.34	-0.06
-0.05	0.08	-0.03	-0.17
0.00	0.23	0.15	-0.17
-0.19	-0.22	-0.32	0.00
-0.30	-0.08	-0.13	0.01
-0.06	0.20	0.20	0.15
-0.12	0.14	0.04	-0.26
-0.14	-0.04	-0.17	0.18
0.00	0.26	0.15	-0.16
-0.12	-0.25	0.08	-0.29
-0.41	-0.33	-0.12	-0.18
0.06	0.18	-0.02	-0.21
-0.02	0.19	0.10	-0.18

34	1	2	3
102	103	104	105
CAGTGCA/	AGGGGTGCTATCTGTGATTGA	TACCCTGTAGATCCGAATTTGTG	TGTAACAGCAACTCCATGTGGA
miR-130k	miR-342-5p	miR-10a-5p	miR-194-5p
#######	2.60E+04	25811	25540
-1.01	0.47	-0.49	-0.50
-2.02	-1.27	4.54	1.09
0.01	-0.50	-0.12	1.23
-0.74	-0.89	-0.20	0.17
-0.64	1.39	-0.01	-0.08
0.55	1.06	-0.31	-0.08
-0.75	0.53	-0.52	-0.76
-0.71	-0.48	-0.61	-0.73
-1.10	-1.25	-0.31	-0.31
-1.20	-0.68	-0.03	0.90
-0.03	-0.54	1.44	0.17
-1.43	2.99	-0.46	-1.22
1.17	-0.39	-0.33	2.42
-0.90	0.06	-0.52	-0.90
-0.34	-0.67	-0.42	-0.13
1.65	-0.05	0.50	1.51
0.94	0.32	-0.37	-0.73
1.53	-0.47	-0.21	0.68
1.22	-0.87	-0.17	-0.19
0.92	-0.85	-0.29	0.85

0.42	0.02	-0.48	-0.14
0.53	0.40	-0.26	-1.30
1.29	0.67	-0.28	-0.92
-0.50	1.27	-0.54	-0.82
-0.16	-0.57	0.38	-1.17
0.61	1.44	-0.10	-0.88
0.71	-1.13	0.18	1.85
-0.59	-1.04	0.45	8.06
-0.22	-0.04	0.29	1.27
-1.33	-1.27	-0.07	0.76
-1.54	-1.36	-0.21	-0.56
0.11	-1.30	1.08	2.25
0.17	-0.41	0.73	1.82
-1.22	-1.09	-0.57	-0.71
-1.11	-0.93	-0.50	1.25
-0.28	1.26	-0.35	-0.90
-1.58	-0.25	-0.67	-1.26
0.10	-0.68	-0.26	1.43
-0.53	-0.64	0.43	0.76
-0.10	0.68	-0.09	-0.49
-1.16	3.48	-0.59	-1.05
-0.32	13.87	-0.39	-0.98
0.23	0.03	-0.33	-0.94
0.26	-1.02	-0.26	1.76
-0.40	0.08	-0.43	-0.39
-1.07	5.20	-0.65	-0.94
3.27	-0.58	-0.25	0.16
1.53	0.21	-0.45	-0.77
0.95	-0.80	-0.44	0.47
1.47	0.00	-0.45	-0.61
1.40	-0.46	-0.29	0.45
1.56	0.24	-0.43	-1.04
1.66	-0.88	-0.40	-0.95
1.87	0.22	-0.22	-0.39
0.37	0.27	-0.47	-0.86
1.43	-0.41	-0.45	-0.88
-0.19	-0.31	-0.28	0.17
0.44	-0.59	0.72	1.99
1.03	1.55	-0.23	-1.09
2.11	1.19	0.01	0.51
0.31	1.42	0.61	-0.33
0.84	-1.03	0.09	2.90
0.09	-0.46	-0.39	-1.18
0.35	0.77	-0.26	-0.55
1.18	-0.82	-0.27	0.15
-1.80	-0.42	-0.11	-0.94
-0.14	-0.86	-0.45	2.71
-1.52	-1.35	1.23	0.09
-0.46	0.17	-0.01	0.49
0.53	0.06	0.18	1.91
-0.93	-0.63	-0.40	-0.10
2.17	-1.04	-0.34	4.86
0.17	-0.56	-0.35	0.52
-0.85	-0.28	-0.15	3.26
-1.79	9.27	-0.70	-1.53
-0.15	0.48	-0.42	0.03
-0.37	-0.66	-0.31	0.42
0.94	0.54	-0.17	1.24
0.65	-1.02	0.36	0.53

4.04	-0.93	0.20	0.33
2.05	-1.03	-0.30	-0.53
2.34	-0.25	-0.16	-0.49
0.81	1.02	-0.54	-0.56
0.65	-0.24	-0.01	1.47
-0.16	-1.20	-0.23	2.20
1.18	-0.47	-0.41	-0.19
1.92	-0.43	-0.08	-0.47
0.47	-0.35	-0.54	-0.20
2.20	-0.85	-0.41	0.87
1.93	-0.21	-0.37	-0.39
-0.78	-0.69	0.83	-0.85
-0.42	-0.65	-0.51	-0.73
1.07	0.37	-0.36	-0.84
-0.45	-1.25	0.62	-0.52
-0.03	-0.03	0.07	0.65
-0.52	2.72E-01	1	0
0.00	0.00	0.00	0.00
1.00	1.00	1.00	1.00
0.60	0.38	0.78	0.62

102	103	104	105
CAGTGCAA	AGGGGTGCTATCTGTGATTGA	TACCCTGTAGATCCGAATTTGTG	TGTAACAGCAACTCCATGTGGA
miR-130b	miR-342-5p	miR-10a-5p	miR-194-5p
#######	2.60E+04	25811	25540
-1.01	0.47	-0.49	-0.50
-2.02	-1.27	4.54	1.09
0.01	-0.50	-0.12	1.23
-0.74	-0.89	-0.20	0.17
-0.64	1.39	-0.01	-0.08
0.55	1.06	-0.31	-0.08
-0.75	0.53	-0.52	-0.76
-0.71	-0.48	-0.61	-0.73
-1.10	-1.25	-0.31	-0.31
-1.20	-0.68	-0.03	0.90
-0.03	-0.54	1.44	0.17
-1.43	2.99	-0.46	-1.22
1.17	-0.39	-0.33	2.42
-0.90	0.06	-0.52	-0.90
-0.34	-0.67	-0.42	-0.13
1.65	-0.05	0.50	1.51
0.94	0.32	-0.37	-0.73
1.53	-0.47	-0.21	0.68
1.22	-0.87	-0.17	-0.19
0.92	-0.85	-0.29	0.85
0.42	0.02	-0.48	-0.14
0.53	0.40	-0.26	-1.30
1.29	0.67	-0.28	-0.92
-0.50	1.27	-0.54	-0.82
-0.16	-0.57	0.38	-1.17
0.61	1.44	-0.10	-0.88
0.71	-1.13	0.18	1.85
-0.59	-1.04	0.45	8.06

-0.22	-0.04	0.29	1.27
-1.33	-1.27	-0.07	0.76
-1.54	-1.36	-0.21	-0.56
0.11	-1.30	1.08	2.25
0.17	-0.41	0.73	1.82
-1.22	-1.09	-0.57	-0.71
-1.11	-0.93	-0.50	1.25
-0.28	1.26	-0.35	-0.90
-1.58	-0.25	-0.67	-1.26
0.10	-0.68	-0.26	1.43
-0.53	-0.64	0.43	0.76
-0.10	0.68	-0.09	-0.49
-1.16	3.48	-0.59	-1.05
-0.32	13.87	-0.39	-0.98
0.23	0.03	-0.33	-0.94
0.26	-1.02	-0.26	1.76
-0.40	0.08	-0.43	-0.39
-1.07	5.20	-0.65	-0.94
3.27	-0.58	-0.25	0.16
1.53	0.21	-0.45	-0.77
0.95	-0.80	-0.44	0.47
1.47	0.00	-0.45	-0.61
1.40	-0.46	-0.29	0.45
1.56	0.24	-0.43	-1.04
1.66	-0.88	-0.40	-0.95
1.87	0.22	-0.22	-0.39
0.37	0.27	-0.47	-0.86
1.43	-0.41	-0.45	-0.88
-0.19	-0.31	-0.28	0.17
0.44	-0.59	0.72	1.99
1.03	1.55	-0.23	-1.09
2.11	1.19	0.01	0.51
0.31	1.42	0.61	-0.33
0.84	-1.03	0.09	2.90
0.09	-0.46	-0.39	-1.18
0.35	0.77	-0.26	-0.55
1.18	-0.82	-0.27	0.15
-1.80	-0.42	-0.11	-0.94
-0.14	-0.86	-0.45	2.71
-1.52	-1.35	1.23	0.09
-0.46	0.17	-0.01	0.49
0.53	0.06	0.18	1.91
-0.93	-0.63	-0.40	-0.10
2.17	-1.04	-0.34	4.86
0.17	-0.56	-0.35	0.52
-0.85	-0.28	-0.15	3.26
-1.79	9.27	-0.70	-1.53
-0.15	0.48	-0.42	0.03
-0.37	-0.66	-0.31	0.42
0.94	0.54	-0.17	1.24
0.65	-1.02	0.36	0.53
4.04	-0.93	0.20	0.33
2.05	-1.03	-0.30	-0.53
2.34	-0.25	-0.16	-0.49
0.81	1.02	-0.54	-0.56
0.65	-0.24	-0.01	1.47
-0.16	-1.20	-0.23	2.20
1.18	-0.47	-0.41	-0.19
1.92	-0.43	-0.08	-0.47

0.47	-0.35	-0.54	-0.20
2.20	-0.85	-0.41	0.87
1.93	-0.21	-0.37	-0.39
-0.78	-0.69	0.83	-0.85
-0.42	-0.65	-0.51	-0.73
1.07	0.37	-0.36	-0.84
-0.45	-1.25	0.62	-0.52
-0.03	-2.73E-02	0	1

102	103	104	105
23	50	30	45
TCAGTGCA	TGAGATGAAGCACTGTAGCTC	TCAGTGCATCACAGAACTTTGT	TCTCACACAGAAATCGCACCCGT
miR-148a	miR-143-3p	miR-148b-3p	miR-342-3p
664224	177234	497718	229733
0.38	-0.43	0.26	1.43
-1.27	-0.55	-1.50	2.87
-0.65	-0.51	-0.73	-0.15
-0.45	-0.54	-0.75	-0.52
-1.13	-0.61	-1.43	2.74
1.18	0.03	0.67	-0.91
-0.16	0.01	1.48	-0.48
-0.75	-0.44	-0.75	-0.94
-0.85	-0.50	-0.70	-0.81
-0.72	-0.42	-0.75	1.01
0.10	0.48	0.67	0.50
-0.69	-0.32	-1.22	-0.39
2.66	3.41	0.15	-0.10
-1.14	-0.50	-1.16	-0.53
-0.28	-0.29	-0.65	-0.73
0.58	0.23	0.51	-0.38
-0.54	-0.45	0.80	0.22
-0.70	0.08	-0.89	-0.36
2.30	0.81	0.21	-0.14
0.81	3.08	-0.79	-0.15
0.86	-0.40	1.51	-0.91
0.22	-0.39	1.68	-0.22
0.12	-0.19	1.17	-0.34
-0.61	-0.53	0.47	-0.49
-0.66	-0.65	-0.61	-0.50
0.20	-0.42	1.57	0.92
1.18	0.04	0.79	-0.65
0.64	1.96	-0.98	-0.52
4.72	0.99	1.01	0.10
0.93	-0.08	-0.19	0.13
-0.75	-0.66	0.86	-0.65
2.64	0.02	0.85	0.65
-0.90	-0.52	-1.74	0.24
0.24	0.89	-0.20	-0.85
-0.44	0.19	-0.94	-0.11
0.05	-0.18	0.23	0.23
-1.35	-0.66	-1.72	-1.22
1.63	0.97	-0.38	-0.47
1.31	1.38	-0.27	2.50

-0.62	-0.27	-0.91	1.08
-0.90	0.12	5.56	0.20
-0.86	1.24	7.72	3.57
-0.56	-0.48	1.36	-0.48
4.26	1.07	1.23	-0.75
-0.77	-0.32	-0.73	-0.31
-1.32	-0.53	1.28	-0.71
1.09	0.53	0.91	0.49
0.62	0.12	1.35	0.13
-0.59	-0.08	-0.69	-0.24
-0.63	-0.20	-0.52	0.82
-0.48	-0.24	0.59	0.83
0.65	-0.48	4.20	-0.54
0.23	-0.46	3.01	-0.98
1.01	-0.12	1.71	-0.08
0.55	-0.45	1.37	-0.91
0.40	-0.53	1.36	-0.49
0.32	-0.51	1.59	-0.76
0.21	0.77	-0.83	-0.58
-0.07	-0.53	0.97	0.01
-0.18	-0.44	0.65	-0.23
-0.77	-0.58	-0.59	0.44
0.77	0.96	-0.76	-0.88
-0.22	-0.55	0.30	-0.74
-0.58	-0.50	0.48	-0.61
-0.11	-0.46	-0.09	-0.63
-0.38	1.64	-0.83	0.03
0.26	-0.33	0.00	-0.06
0.43	-0.47	0.07	1.34
-1.02	-0.65	-0.84	-0.06
0.07	-0.37	-0.06	0.49
0.15	-0.38	0.19	-0.45
1.49	4.63	-0.63	-0.33
-0.51	-0.48	-0.63	-0.83
-0.82	0.72	-1.33	0.04
-1.38	-0.56	-0.57	2.81
0.02	0.01	0.05	-0.51
0.87	0.06	-0.28	0.15
0.81	-0.08	-0.17	-0.66
0.85	-0.17	1.21	0.51
0.40	-0.08	0.97	0.44
-0.41	-0.53	0.69	0.12
0.29	-0.42	1.59	0.95
-0.55	0.06	-0.40	0.57
0.88	3.71	-0.94	1.77
0.86	1.40	-1.06	-0.59
-0.61	0.29	-0.22	1.03
0.41	-0.10	0.55	0.86
0.05	-0.38	0.07	0.11
3.18	1.48	0.38	-0.71
-0.07	-0.39	0.71	0.13
1.13	1.87	-0.31	-0.71
3.98	-0.23	1.53	-1.01
0.59	-0.29	1.63	-0.55
2.75	-0.29	2.48	-0.84
1.03	-0.43	3.96	0.32
0.68	0.69	0.73	0.74
1.00	0.65	0.64	0.78

UC	NP	P
#REF!	#REF!	#REF!

using all thresholds = 0.54140, corresponding to 1E-3 frequency of corrs > 0.54140

only reselection of 29s		
PvUC ttest	NPvP ttest	NPvUC ttest
0.00E+00	0.00E+00	0.00E+00
7.43E-158	6.16E-63	1.81E-44
7.48E-14	9.29E-07	1.03E-04

ction of 29s #REF!

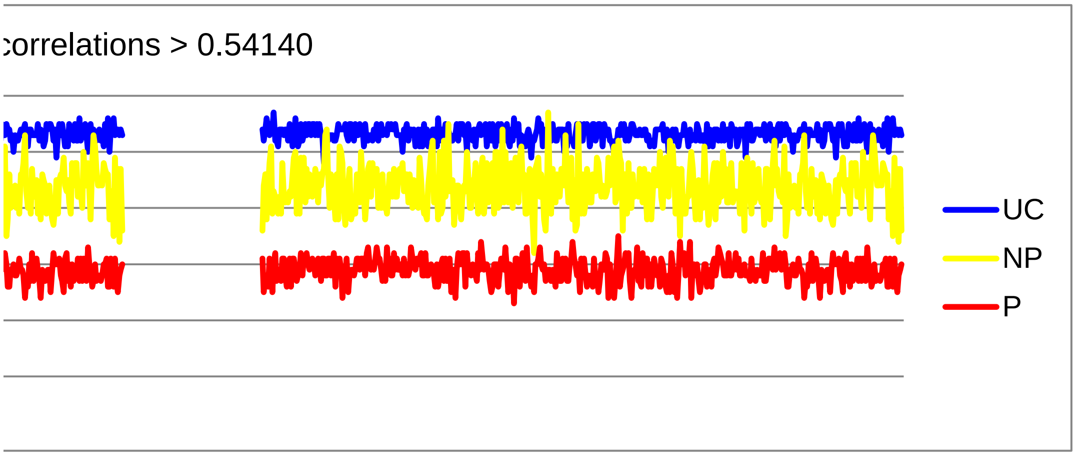

total UC reads after discards
16189
16189
15375
15296
15271
14130
13677
13338
12972
12390
12316
12206
11818
11405
11117
11010
10801
10703
10601
9806
9782

5	6	7	8
259 mature miRNAs:	TATTGCACTTGTCCCGGCC	CAACGGAATCCCAAAAGCAGCTG	TCTCCCAACCCTTGTACCAGTG
ID	miR-92a-3p	miR-191-5p	miR-150-5p
total all samples =100:	46389442	10497180	8994745
UC	17.08	0.63	1.27
UC	3.76	15.01	1.45
UC	11.64	1.30	0.42
UC	17.41	0.82	0.80
UC	1.87	5.70	5.29
UC	14.85	1.55	1.00
UC	14.41	0.74	2.47
UC	17.33	1.47	2.54
UC	16.56	3.05	0.50
UC	15.83	2.63	1.21
UC	11.24	4.26	1.63
UC	13.86	0.50	6.11
UC	6.82	1.31	1.56
UC	16.32	1.40	2.74
UC	12.20	3.50	1.17
UC	9.31	2.79	2.16
UC	11.44	3.20	2.40
UC	11.57	1.47	2.57
UC	9.38	6.27	1.99
UC	7.62	1.61	1.45
UC	15.29	1.68	1.29
UC	11.98	2.11	3.70
UC	12.33	2.10	2.91
UC	14.79	1.41	2.57
UC	3.24	12.29	1.57
UC	6.65	6.06	3.14
UC	8.93	3.47	1.02
CHR_NP	6.98	0.77	0.26
CHR_NP	13.17	3.20	1.08
CHR_NP	15.91	4.64	0.59
CHR_NP	15.47	5.99	0.38
CHR_NP	6.54	8.55	0.54
CHR_NP	1.16	6.37	1.55
CHR_NP	16.96	1.39	0.90
CHR_NP	15.55	2.58	1.29
CHR_NP	12.34	1.18	5.81
CHR_NP	23.63	0.37	0.61
CHR_NP	12.30	1.02	3.01
CHR_NP	6.39	0.83	1.28
CHR_NP	9.26	2.38	6.02
CHR_NP	14.47	1.08	2.96
CHR_NP	5.12	1.20	4.43
CHR_NP	14.32	2.33	2.67
CHR_NP	11.69	2.81	1.26
CHR_NP	14.35	4.24	3.73
CHR_NP	16.84	0.60	1.69
CHR_NP	6.82	4.50	2.37
CHR_NP	11.42	2.31	2.75
CHR_NP	12.56	2.27	2.36
CHR_NP	10.93	2.02	5.36

CHR_NP	9.29	2.20	4.15
CHR_NP	12.83	3.95	2.72
CHR_NP	15.87	3.08	0.70
CHR_NP	9.89	3.75	2.46
CHR_NP	15.48	2.54	1.67
CHR_NP	14.23	3.73	1.80
CHR_NP	13.95	2.34	1.90
CHR_NP	4.86	1.19	1.04
CHR_NP	8.19	3.15	4.06
CHR_NP	3.59	4.90	2.58
CHR_NP	2.52	4.71	4.61
CHR_NP	6.08	1.42	0.67
CHR_NP	14.88	2.50	1.80
CHR_NP	12.94	2.18	2.03
CHR_NP	8.98	2.95	0.83
CHR_P	12.54	0.50	0.66
CHR_P	11.66	0.60	0.34
CHR_P	7.89	10.22	0.92
CHR_P	5.48	1.42	1.48
CHR_P	6.88	2.04	1.78
CHR_P	17.52	0.79	1.13
CHR_P	4.55	0.06	9.21
CHR_P	14.33	1.06	1.13
CHR_P	10.06	1.01	3.52
CHR_P	12.27	0.52	5.04
CHR_P	10.92	1.31	3.42
CHR_P	8.96	6.69	2.97
CHR_P	10.18	7.08	1.60
CHR_P	7.73	3.56	1.17
CHR_P	6.27	5.24	1.04
CHR_P	11.78	6.21	1.07
CHR_P	8.28	3.76	2.90
CHR_P	12.13	1.94	6.63
CHR_P	5.36	1.30	1.74
CHR_P	4.54	0.95	0.63
CHR_P	12.06	2.62	4.16
CHR_P	8.77	3.03	4.17
CHR_P	11.80	2.25	3.65
CHR_P	10.14	3.38	1.31
CHR_P	10.66	3.88	3.87
CHR_P	6.92	2.73	0.42
CHR_P	16.30	2.68	1.77
CHR_P	11.79	2.22	2.27
CHR_P	8.76	6.90	0.46
CHR_P	10.64	2.52	1.18
0	0.00	0.00	0.00
0	0.00	0.00	0.00
0	0.00	0.00	0.00

6	7	8	9
B_32	*B_34*	*C_1*	*C_40*
0.54			
0.20	0.33		

-0.03	-0.34	0.01	
-0.10	-0.40	-0.32	0.55
-0.12	-0.42	-0.43	0.33
0.29	0.41	0.53	-0.23
-0.08	-0.22	-0.36	-0.18
0.00	0.32	0.74	0.00
-0.35	-0.33	-0.29	0.24
-0.01	-0.16	-0.42	-0.12
-0.14	0.20	0.17	-0.27
-0.35	-0.07	0.00	-0.04
-0.18	-0.27	-0.18	-0.18
-0.24	-0.41	-0.29	0.04
0.54	0.28	0.08	0.09
0.07	0.56	0.39	-0.41
0.09	0.50	0.38	-0.47
0.16	0.51	0.57	-0.29
-0.05	-0.07	0.09	0.04
-0.10	0.09	-0.20	-0.43
-0.22	-0.47	-0.50	0.29
-0.15	-0.36	-0.31	0.20
0.11	-0.22	-0.33	0.08
-0.06	-0.36	-0.20	0.42
0.08	-0.04	0.31	0.54
-0.07	-0.33	-0.33	0.26
-0.15	-0.35	-0.30	0.14
0.08	0.33	0.48	-0.17
-0.20	0.00	0.29	-0.12
0.07	0.39	0.05	-0.39
0.27	0.11	0.63	0.32
0.07	0.09	-0.26	-0.14
-0.22	-0.19	-0.38	-0.06
-0.18	0.08	0.11	-0.48
-0.11	0.19	0.30	-0.24
-0.08	0.18	0.19	-0.22
0.09	0.43	0.25	-0.25
-0.03	-0.18	-0.28	-0.08
0.07	0.19	0.58	-0.28
-0.02	0.22	0.42	-0.21
-0.13	-0.25	-0.37	-0.19
0.15	0.46	0.29	-0.45
-0.21	-0.13	0.13	-0.11
-0.14	0.11	0.04	-0.37
-0.19	-0.01	-0.19	-0.23
0.15	0.39	-0.01	-0.08
0.17	0.29	0.04	0.11
0.04	0.13	-0.27	-0.41
0.58	0.49	0.35	-0.17

0.03	0.11	0.01	0.02
0.21	0.26	-0.14	0.19
-0.18	-0.35	-0.32	0.07
0.00	0.35	0.10	-0.47
0.07	0.01	-0.23	-0.41
-0.10	-0.11	-0.31	-0.30
-0.20	-0.43	-0.36	0.20
-0.09	-0.10	0.24	0.05
0.18	-0.01	-0.07	0.02
-0.16	-0.42	-0.47	0.29
0.02	0.12	0.10	-0.03
-0.16	-0.40	-0.42	0.33
-0.07	-0.26	-0.15	0.20
-0.05	0.06	0.11	0.11
-0.13	0.04	-0.07	-0.18
0.21	0.28	0.30	0.09
0.08	-0.18	-0.11	-0.04
0.07	-0.28	-0.21	0.44
-0.24	-0.19	-0.14	-0.17
-0.13	0.14	0.25	-0.17
0.12	0.19	-0.23	-0.12
-0.20	-0.35	-0.30	-0.05
0.18	-0.03	0.06	0.06
-0.36	-0.41	-0.60	0.06
-0.17	-0.42	-0.52	-0.02
-0.19	-0.26	0.01	0.16
0.01	-0.14	-0.45	-0.11
0.16	0.33	0.52	-0.34
-0.25	-0.35	-0.32	-0.03
-0.16	-0.34	-0.27	0.15
-0.21	-0.13	0.00	-0.21
-0.12	-0.04	-0.27	-0.40
0.00	-0.19	-0.16	0.04
-0.08	-0.28	-0.37	-0.11
-0.11	-0.12	-0.21	-0.21
-0.08	-0.15	-0.35	0.10
0.33	0.24	0.16	-0.09
0.14	0.51	0.30	-0.42
-0.05	-0.30	-0.43	0.21
0.01	-0.11	-0.39	0.08

4	5	6	7
106	107	108	109
TACCCATTGCATATCGGAGTTG	ACCCCACTCCTGGTACC	TTATCAGAATCTCCAGGGGTAC	TAAAGTGCTGACAGTGCAGAT
miR-660-5p	miR-4286	miR-361-5p	miR-106b-5p
23949	23822	23449	23446
-0.51	1.23	-0.40	-0.42
-0.07	-1.16	1.33	-0.19
0.33	-1.17	-0.45	0.84
0.13	-1.14	-0.76	-0.19
-0.56	2.46	4.06	0.96
-0.70	-0.88	-0.25	-0.28
-0.55	-0.14	-0.57	-0.51
-0.65	0.97	-0.60	-0.82
-0.56	-1.28	-0.51	-0.86
-0.50	-0.78	-0.21	0.15
-0.19	-0.54	0.14	-0.49
-0.64	-0.42	-0.76	-0.95
2.94	0.65	0.10	3.79
-0.77	-0.31	-0.66	-0.92
0.19	-0.35	-0.27	-0.15
0.72	0.21	-0.06	0.77
-0.53	0.06	-0.12	-0.31
2.16	1.82	-0.54	1.00
0.36	0.08	0.22	-0.55
1.97	-0.38	0.02	1.44

-0.61	-0.82	-0.39	-0.48
-0.79	0.43	-0.53	-0.83
-0.51	0.17	-0.38	-0.46
-0.73	-0.14	-0.47	-0.36
-0.67	1.54	1.75	-0.51
-0.57	1.08	0.62	-0.22
1.32	-1.18	-0.30	0.55
5.07	-1.19	-0.61	4.67
0.23	-0.35	0.50	-0.15
0.16	-0.90	-0.32	-0.65
-0.39	-0.93	-0.20	-0.82
2.14	-0.14	0.27	-0.27
-0.03	2.83	1.81	1.79
-0.37	1.84	-0.52	-0.29
-0.12	4.22	0.05	0.13
-0.66	0.21	-0.48	-0.65
-0.93	-1.15	-0.90	-1.04
-0.16	-0.78	-0.08	-0.29
4.48	-0.17	0.21	1.91
-0.50	0.42	-0.09	-0.65
-0.63	12.46	-0.52	0.26
-0.24	39.75	-0.68	-0.15
-0.75	0.45	-0.26	-0.73
0.45	0.75	0.47	1.60
-0.54	0.84	-0.33	-1.05
-0.90	10.03	-0.77	-0.32
-0.31	0.59	1.10	0.19
-0.59	0.71	0.14	-0.37
-0.29	0.63	-0.22	0.20
-0.50	0.21	-0.10	-0.26
-0.18	0.32	-0.08	0.42
-0.73	0.85	-0.14	-0.47
-0.72	0.33	-0.19	-0.30
-0.50	1.12	0.79	0.50
-0.72	-0.26	-0.51	-0.71
-0.70	-0.35	-0.25	-0.62
-0.52	-0.28	-0.22	-0.07
5.80	-0.69	-0.44	4.61
-0.52	-0.15	0.17	-0.76
0.00	0.84	0.85	0.93
0.01	0.61	1.61	1.08
4.37	-1.24	-0.49	3.12
-0.47	-0.82	-0.44	-0.66
-0.52	-0.48	-0.54	-0.30
0.22	-1.15	-0.31	0.11
4.09	-1.28	-0.85	-0.55
2.19	-0.75	-0.41	0.85
0.08	1.31	0.28	-1.19
0.48	-0.23	-0.46	0.83
0.67	0.81	-0.17	1.04
-0.19	-0.52	-0.68	-0.34
-0.59	-0.49	-0.27	0.51
-0.13	-0.94	-0.56	-0.18
0.92	0.25	-0.30	0.78
-0.82	37.19	-0.73	-1.13
0.49	-0.45	-0.56	0.69
0.14	-0.37	0.10	-0.75
-0.40	-0.64	-0.34	-1.03
1.57	-0.08	0.54	1.65

-0.09	-0.33	0.91	0.01
-0.66	-0.45	-0.10	-0.62
-0.36	-0.20	0.55	-0.34
-0.73	0.94	-0.30	-0.15
3.40	0.08	0.06	2.73
4.13	-1.23	-0.52	4.15
-0.32	1.05	-0.10	-0.29
-0.38	0.01	0.06	-0.09
-0.54	-0.29	-0.08	-0.18
1.17	-0.60	-0.09	-0.16
-0.52	-0.05	0.30	-0.08
1.63	-1.40	-0.44	0.58
-0.53	-0.06	-0.46	-0.83
-0.63	0.31	-0.41	-0.20
-0.15	-0.95	0.10	-0.56
-0.23	-0.41	0.28	-0.49
1	1	1	-2
0.00	0.00	0.00	0.00
1.00	1.00	1.00	1.00
0.55	0.66	0.32	0.79

106	107	108	109
TACCCATTGCATATCGGAGTTG	ACCCCACTCCTGGTACC	TTATCAGAATCTCCAGGGGTAC	TAAAGTGCTGACAGTGCAGAT
miR-660-5p	miR-4286	miR-361-5p	miR-106b-5p
23949	23822	23449	23446
-0.51	1.23	-0.40	-0.42
-0.07	-1.16	1.33	-0.19
0.33	-1.17	-0.45	0.84
0.13	-1.14	-0.76	-0.19
-0.56	2.46	4.06	0.96
-0.70	-0.88	-0.25	-0.28
-0.55	-0.14	-0.57	-0.51
-0.65	0.97	-0.60	-0.82
-0.56	-1.28	-0.51	-0.86
-0.50	-0.78	-0.21	0.15
-0.19	-0.54	0.14	-0.49
-0.64	-0.42	-0.76	-0.95
2.94	0.65	0.10	3.79
-0.77	-0.31	-0.66	-0.92
0.19	-0.35	-0.27	-0.15
0.72	0.21	-0.06	0.77
-0.53	0.06	-0.12	-0.31
2.16	1.82	-0.54	1.00
0.36	0.08	0.22	-0.55
1.97	-0.38	0.02	1.44
-0.61	-0.82	-0.39	-0.48
-0.79	0.43	-0.53	-0.83
-0.51	0.17	-0.38	-0.46
-0.73	-0.14	-0.47	-0.36
-0.67	1.54	1.75	-0.51
-0.57	1.08	0.62	-0.22
1.32	-1.18	-0.30	0.55
5.07	-1.19	-0.61	4.67

0.23	-0.35	0.50	-0.15
0.16	-0.90	-0.32	-0.65
-0.39	-0.93	-0.20	-0.82
2.14	-0.14	0.27	-0.27
-0.03	2.83	1.81	1.79
-0.37	1.84	-0.52	-0.29
-0.12	4.22	0.05	0.13
-0.66	0.21	-0.48	-0.65
-0.93	-1.15	-0.90	-1.04
-0.16	-0.78	-0.08	-0.29
4.48	-0.17	0.21	1.91
-0.50	0.42	-0.09	-0.65
-0.63	12.46	-0.52	0.26
-0.24	39.75	-0.68	-0.15
-0.75	0.45	-0.26	-0.73
0.45	0.75	0.47	1.60
-0.54	0.84	-0.33	-1.05
-0.90	10.03	-0.77	-0.32
-0.31	0.59	1.10	0.19
-0.59	0.71	0.14	-0.37
-0.29	0.63	-0.22	0.20
-0.50	0.21	-0.10	-0.26
-0.18	0.32	-0.08	0.42
-0.73	0.85	-0.14	-0.47
-0.72	0.33	-0.19	-0.30
-0.50	1.12	0.79	0.50
-0.72	-0.26	-0.51	-0.71
-0.70	-0.35	-0.25	-0.62
-0.52	-0.28	-0.22	-0.07
5.80	-0.69	-0.44	4.61
-0.52	-0.15	0.17	-0.76
0.00	0.84	0.85	0.93
0.01	0.61	1.61	1.08
4.37	-1.24	-0.49	3.12
-0.47	-0.82	-0.44	-0.66
-0.52	-0.48	-0.54	-0.30
0.22	-1.15	-0.31	0.11
4.09	-1.28	-0.85	-0.55
2.19	-0.75	-0.41	0.85
0.08	1.31	0.28	-1.19
0.48	-0.23	-0.46	0.83
0.67	0.81	-0.17	1.04
-0.19	-0.52	-0.68	-0.34
-0.59	-0.49	-0.27	0.51
-0.13	-0.94	-0.56	-0.18
0.92	0.25	-0.30	0.78
-0.82	37.19	-0.73	-1.13
0.49	-0.45	-0.56	0.69
0.14	-0.37	0.10	-0.75
-0.40	-0.64	-0.34	-1.03
1.57	-0.08	0.54	1.65
-0.09	-0.33	0.91	0.01
-0.66	-0.45	-0.10	-0.62
-0.36	-0.20	0.55	-0.34
-0.73	0.94	-0.30	-0.15
3.40	0.08	0.06	2.73
4.13	-1.23	-0.52	4.15
-0.32	1.05	-0.10	-0.29
-0.38	0.01	0.06	-0.09

-0.54	-0.29	-0.08	-0.18
1.17	-0.60	-0.09	-0.16
-0.52	-0.05	0.30	-0.08
1.63	-1.40	-0.44	0.58
-0.53	-0.06	-0.46	-0.83
-0.63	0.31	-0.41	-0.20
-0.15	-0.95	0.10	-0.56
0	0	0	0

106	107	108	109
20	31	111	26
CACTAGATTGTGAGCTCCTGGA	TGTAAACATCCTACACTC[illegible]	ACCATCGACCGTTGATTGTACC	TACCACAGGGTAGAACCACGG
miR-28-3p	miR-30c-5p	miR-181a-3p	miR-140-3p
903691	492626	23176	605614
0.42	-0.41	-0.85	1.34
3.14	-0.86	-0.38	-1.78
-0.33	-0.23	-0.11	0.50
-0.84	-0.87	-0.91	1.67
-0.57	0.52	-0.47	-1.76
0.22	-1.19	-1.21	-0.75
0.15	-1.14	-0.83	-0.36
-0.99	-0.58	-0.40	-0.61
-0.89	-0.54	-0.80	-0.73
-0.17	-0.57	-0.84	-0.61
1.11	0.31	0.90	-0.21
-0.82	-0.23	-0.28	-0.89
-0.26	2.45	1.90	-0.37
-1.04	-1.11	-0.64	-0.34
-0.22	0.13	0.54	0.91
0.03	1.73	1.25	0.36
-0.02	0.02	-0.18	1.35
-0.70	-0.16	2.26	0.53
2.92	1.63	1.80	1.12
-0.41	2.06	1.94	1.12
-0.01	-0.88	-0.22	-0.27
0.20	0.11	-0.25	1.34
0.04	-0.20	-0.33	0.69
-0.70	-1.01	-0.94	0.24
0.09	-0.32	-0.16	-1.91
0.02	0.38	-0.34	-0.47
-0.36	0.94	-0.44	-0.12
-0.75	-0.66	0.32	-0.18
6.39	0.33	1.55	1.20
3.15	-0.83	-0.07	-0.52
0.86	-1.55	-1.09	-1.39
7.78	0.49	0.77	-1.40
-1.27	1.79	0.08	-1.82
0.17	0.32	-0.59	0.62
-0.58	1.10	0.28	-0.26
0.44	0.41	-0.07	1.10
-1.95	-1.76	-1.26	-1.47
1.25	0.87	0.50	1.04
0.68	0.83	2.67	-0.11

0.13	1.28	1.27	-0.17
0.78	-0.94	-1.40	0.18
0.78	-0.99	-0.92	2.26
-0.02	-0.81	-0.38	0.79
1.61	1.48	0.55	1.09
0.55	0.76	1.16	-0.03
-0.43	-1.56	-1.45	-0.90
0.34	4.26	0.92	1.33
0.89	1.25	0.65	0.71
-1.03	2.98	0.74	1.23
-0.28	2.36	0.99	1.94
-0.53	2.46	2.30	1.12
0.20	-0.72	-0.77	1.49
-0.67	-1.18	-1.08	1.04
-0.30	1.54	-0.29	0.93
-0.41	-0.52	-0.39	0.30
0.06	-0.96	-0.36	1.34
-0.90	0.20	-0.75	0.62
-0.74	0.51	1.39	-0.52
1.00	0.33	0.68	-0.79
0.70	3.47	1.48	-1.37
0.13	2.90	0.96	-1.43
-0.78	0.17	0.22	-0.95
-1.04	-0.54	-1.20	-0.48
-0.79	0.38	-0.82	-0.93
-0.82	0.06	-0.28	-0.22
-0.20	-0.83	1.43	-0.29
-0.48	-0.50	-0.60	1.31
7.04	-1.06	0.23	-1.66
-1.10	0.01	-0.52	-0.87
0.12	2.01	1.40	0.11
-0.35	-0.47	-0.87	1.16
1.34	1.58	1.52	-0.07
-1.13	-0.07	-0.56	-0.18
-0.70	1.86	0.95	-0.02
-0.71	-1.86	-1.10	1.25
-0.85	0.52	0.38	-0.28
2.53	0.66	1.73	0.21
3.15	-0.39	0.38	-1.02
-0.71	1.93	2.25	2.55
0.23	2.51	0.97	0.40
-0.60	-0.34	-0.73	0.29
0.17	0.93	0.42	0.20
-0.72	2.45	-0.54	-0.17
-0.14	2.36	2.86	0.83
-1.22	-0.15	1.60	0.10
-0.10	2.33	0.58	1.08
-0.15	1.65	0.91	2.04
-0.70	0.61	-0.67	1.24
0.94	2.13	1.77	1.60
-0.35	1.22	-0.08	0.87
-0.93	-0.92	0.99	-0.74
0.07	-0.90	-0.58	1.02
-0.18	-0.17	0.47	1.14
0.37	-0.04	-0.09	-0.77
0.05	-0.16	-0.44	-0.39
0.75	0.77	0.86	0.88
1.00	0.55	1.00	1.00

	reselects	
	UC	NP
	44	33
	44	30
	43	43
	43	31
	44	39
	45	37
	44	31
	43	34
	45	28
	42	37
	45	45
2.96E+13	45	34
	44	32
	43	31
	41	38
	41	39
	43	41
	43	26
	42	29
	44	28
	44	40
	43	32
	43	32
	42	33
	44	39
	43	34
	43	28
	44	35
	43	36
	43	36
	44	33
	43	36
	44	35
	45	27
	45	38
	42	30
	45	27
	43	37
	44	32
	41	31
	44	30
	42	40
	45	26
	43	32
	44	31
	42	33
	42	33
	44	34
	44	29
	43	32
	40	37

42	34
44	32
43	30
42	34
42	32
43	30
43	24
43	39
45	26
41	32
44	35
43	31
45	38
42	33
44	33
42	35
45	34
45	33
43	41
45	36
45	25
41	35
43	35
45	37
44	31
40	35
43	33
43	35
45	31
44	33
43	34
44	30
44	35
42	33
43	28
42	33
43	24
42	30
41	38
41	37
44	27
45	36
45	26
45	34
40	38
45	30
45	30
45	41
42	38
45	28
44	39
44	37
42	39
42	37
42	40
42	40
43	29
40	34
41	32

44	30
44	27
42	34
42	29
42	31
42	27
45	36
43	39
45	31
41	29
45	34
44	38
41	32
43	35
43	34
45	34
44	34
42	34
44	28
44	37
43	34
45	28
42	37
44	38
46	34
43	39
44	32
45	27
43	35
39	41
43	34
44	35
42	36
43	33
43	31
42	37
43	36
43	39
42	32
43	37
42	32
42	30
45	26
44	30
44	31
43	31
44	34
45	35
45	34
43	36
44	34
44	37
42	36
43	38
45	32
44	33
45	30
45	36
39	33

44	34
45	38
39	26
44	29
42	36
45	38
45	30
43	33
43	40
43	37
45	36
44	37
43	34
40	30
45	35
44	27
45	34
41	35
44	36
44	39
43	34
45	28
40	36
43	35
45	39
40	31
42	34
43	35
45	29
44	37
37	37
45	31
41	34
43	29
42	39
45	32
41	38
43	35
43	36
45	36
42	33
45	36
41	30
43	36
46	28
43	34
42	37
41	30
46	30
43	36
45	39
42	41
45	32
41	34
44	34
43	35
43	32
45	33
45	34

41	31
43	32
42	35
43	38
44	35
44	39
43	30
45	34
40	33
44	38
44	34
43	32
40	28
44	26
45	39
43	34
44	34
40	34
42	32
43	33
42	31
44	39
42	37
42	32
44	34
45	39
44	29
45	36
42	36
40	34
42	29
42	30
42	38
44	32
42	33
44	35
40	40
44	34
39	30
43	36
44	35
44	37
43	36
42	32
42	33
43	37
41	33
43	30
42	42
45	33
45	30
43	36
42	36
43	38
43	33
43	32
42	39
45	34
44	38

44	31
44	35
44	28
44	36
45	26
42	41
42	28
43	29
44	34
45	29
40	29
45	35
44	31
43	33
45	36
44	30
43	31
43	35
42	25
42	33
43	31
44	29
41	31
44	30
42	31
43	28
41	36
42	32
44	36
43	31
42	39
46	30
44	28
44	32
44	34
43	26
43	30
42	36
44	36
40	31
41	36
44	38
41	36
44	32
43	37
41	36
41	39
43	34
45	32
40	42
45	26
46	36
43	32
45	29
44	34
44	34
44	36
44	35
44	35

44	27
43	37
46	27
41	34
44	28
41	39
45	32
44	37
44	29
42	36
44	34
43	40
43	34
44	36
41	29
44	35
41	33
42	34
44	32
44	33
43	30
43	36
43	36
45	39
46	37
44	37
43	32
45	29
43	31
45	33
46	33
43	36
40	39
43	31
44	32
46	33
44	35
43	36
46	32
40	37
45	26
45	37
42	36
44	31
45	32
45	32
39	39
42	31
44	34
44	32
44	32
45	27
44	40
42	37
42	32
40	32
44	27
45	26
43	35

44	47
44	39
42	37
44	35
40	35
43	30
44	33
45	37
40	39
43	34
43	38
45	31
42	39
43	32
41	30
42	35
43	32
44	35
43	36
39	36
44	31
44	41
43	34
46	29
42	35
42	35
43	31
42	40
44	40
39	36
44	28
45	37
42	34
43	32
44	32
45	28
40	32
43	35
41	31
43	35
42	28
42	36
43	37
43	32
45	29
42	37
43	34
45	24
42	36
45	26
41	28
44	34
45	26
43	36
43	35
45	33
42	37
42	32
42	32

41	34
43	39
45	32
45	33
44	29
42	30
44	26
44	31
42	36
43	30
40	39
42	30
44	33
43	32
45	33
43	40
43	37
42	34
40	30
42	30
42	33
45	30
43	31
44	34
42	30
43	36
41	31
40	36
43	33
43	43
42	32
44	35
41	30
44	35
43	28
44	33
43	31
40	35
43	37
43	35
43	44
43	39
44	31
43	33
45	31
45	34
45	26
44	35
43	34
44	36
44	33
45	32
44	39
42	31
42	36
45	25
44	29
43	35
44	34

43	32
43	32
43	34
43	33
42	32
44	31
43	30
43	41
41	36
45	31
43	32
43	37
43	31
42	37
46	29
43	29
43	31
44	31
44	34
43	29
46	37
43	35
46	28
45	31
43	34
44	29
43	38
43	34
42	36
42	34
44	36
46	28
44	34
43	38
45	41
44	29
47	33
42	32
42	31
41	29
44	32
44	29
44	38
43	32
44	31
44	32
42	31
45	33
44	32
41	36
42	39
46	40
42	29
41	34
42	29
45	39
42	34
43	39

45	31
43	37
45	32
45	36
43	33
45	32
45	33
43	37
45	35
43	31
45	36
43	34
43	36
38	37
43	43
43	44
42	36
43	30
43	36
43	32
42	32
42	28
43	36
45	28
44	41
44	40
44	38
44	30
45	27
43	31
42	34
45	37
45	28
44	29
42	32
45	29
43	36
43	35
45	31
44	40
44	35
41	32
45	28
42	32
42	37
43	38
43	32
42	38
44	34
43	35
45	37
42	30
44	34
45	30
43	36
44	30
44	31
43	29
45	32

44	36
45	34
44	32
44	37
45	32
43	32
43	35
43	37
43	36
40	38
44	33
44	36
45	36
42	31
44	36
44	34
43	30
44	35
41	33
42	32
44	30
41	39
44	35
41	31
45	29
42	29
44	28
41	34
42	37
41	40
44	42
44	31
44	31
41	33
46	28
42	40
43	29
44	30
43	42
45	32
44	34
44	45
43	33
42	34
42	35
43	27
43	31
45	34
42	34
45	31
45	28
45	32
43	34
44	33
40	40
45	36
43	30
43	30
42	35

44	34
41	38
44	32
44	29
45	32
43	37
44	39
44	29
45	32
41	38
44	31
45	33
42	36
45	38
43	29
41	40
43	40
45	30
42	31
45	31
43	44
42	31
44	40
45	35
42	31
41	32
44	38
42	30
46	36
43	39
44	38
45	31
41	39
43	41
41	35
42	36
41	29
43	29
44	34
43	37
39	33
42	28
42	22
43	37
44	38
46	39
44	32
45	36
41	33
44	28
43	26
43	33
44	47
42	33
42	29
45	37
43	32
42	31
42	36

44	34
44	32
44	37
44	37
40	34
44	43
41	35
45	31
42	33
42	39
42	28
41	35
43	26
45	27
43	45
45	30
42	29
42	36
45	29
44	31
45	37
44	35
41	31
43	31
45	36
45	34
42	33
43	39
45	38
45	35
44	32
41	34
45	32
44	32
43	33
44	39
42	34
42	35
41	36
40	41
42	31
42	32
44	42
43	39
45	38
43	26
45	36
42	36
44	29
42	38
43	30
45	30
45	36
44	32
43	33
43	33
43	32
44	37
43	35

43	31
44	37
44	33
42	28
43	36
41	35
42	28
42	33
41	37
44	34
44	35
44	34
44	40
44	32
45	30
42	36
43	39
44	37
40	32
43	42
43	38
44	41
42	35
44	29
42	37
41	35
43	25
43	37
43	31
45	29
43	29
43	32
41	32
44	37
42	40
43	37
43	32
42	28
45	32
44	35
42	28
42	34
43	30
43	41
41	35
45	32
41	27
44	29
42	33
41	36
44	38
44	28
44	38
42	31
43	33
42	32
45	40
42	36
43	37

44	31
44	31
41	36
45	38
44	31
43	32
44	33
41	32
42	33
44	29
43	38
44	36
44	26
39	36
45	39
44	35
45	33
42	29
42	38
43	31
44	32
44	33
44	37
43	32
44	31
43	32
45	27
42	37
43	37
43	31
45	28
42	34
44	38
42	42
41	34
44	37
45	33
42	32
45	34
43	31
43	41
45	25
44	28
44	36
42	30
43	31
40	31
43	34
42	30
42	33
43	29
44	36
43	36
44	38
45	43
41	33
41	30
44	32
44	29

43	37
43	32
43	33
43	35
45	29
42	33
44	28
43	36
41	35
42	34
45	29
44	32
45	34
45	30
44	28
42	27
44	30
39	35
43	29
45	35
43	36
45	36
43	39
41	34
42	33
41	35
45	31
43	29
44	38
42	34
45	38
42	38
42	32
46	32
42	33
43	30
43	40
45	34
43	34
40	38
44	33
45	28
42	38
44	43
43	41
42	37
43	34
44	34
42	35
43	34
41	38
45	37
42	36
46	36
40	28
44	33
44	33
46	25
43	39

44	30
44	37
43	24
44	37
43	26

44	26
42	34
44	36
46	28
44	34
43	38
45	41
44	29
47	33
42	32
42	31
41	29
44	32
44	29
44	38
43	32

44	31
44	32
42	31
45	33
44	32
41	36
42	39
46	40
42	29
41	34
42	29
45	39
42	34
43	39
45	31
43	37
45	32
45	36
43	33
45	32
45	33
43	37
45	35
43	31
45	36
43	34
43	36
38	37
43	43
43	44
42	36
43	30
43	36
43	32
42	32
42	28
43	36
45	28
44	41
44	40
44	38
44	30
45	27
43	31
42	34
45	37
45	28
44	29
42	32
45	29
43	36
43	35
45	31
44	40
44	35
41	32
45	28
42	32
42	37

43	38
43	32
42	38
44	34
43	35
45	37
42	30
44	34
45	30
43	36
44	30
44	31
43	29
45	32
44	36
45	34
44	32
44	37
45	32
43	32
43	35
43	37
43	36
40	38
44	33
44	36
45	36
42	31
44	36
44	34
43	30
44	35
41	33
42	32
44	30
41	39
44	35
41	31
45	29
42	29
44	28
41	34
42	37
41	40
44	42
44	31
44	31
41	33
46	28
42	40
43	29
44	30
43	42
45	32
44	34
44	45
43	33
42	34
42	35

43	27
43	31
45	34
42	34
45	31
45	28
45	32
43	34
44	33
40	40
45	36
43	30
43	30
42	35
44	34
41	38
44	32
44	29
45	32
43	37
44	39
44	29
45	32
41	38
44	31
45	33
42	36
45	38
43	29
41	40
43	40
45	30
42	31
45	31
43	44
42	31
44	40
45	35
42	31
41	32
44	38
42	30
46	36
43	39
44	38
45	31
41	39
43	41
41	35
42	36
41	29
43	29
44	34
43	37
39	33
42	28
42	22
43	37
44	38

46	39
44	32
45	36
41	33
44	28
43	26
43	33
44	47
42	33
42	29
45	37
43	32
42	31
42	36
44	34
44	32
44	37
44	37
40	34
44	43
41	35
45	31
42	33
42	39
42	28
41	35
43	26
45	27
43	45
45	30
42	29
42	36
45	29
44	31
45	37
44	35
41	31
43	31
45	36
45	34
42	33
43	39
45	38
45	35
44	32
41	34
45	32
44	32
43	33
44	39
42	34
42	35
41	36
40	41
42	31
42	32
44	42
43	39
45	38

43	26
45	36
42	36
44	29
42	38
43	30
45	30
45	36
44	32
43	33
43	33
43	32
44	37
43	35
43	31
44	37
44	33
42	28
43	36
41	35
42	28
42	33
41	37
44	34
44	35
44	34
44	40
44	32
45	30
42	36
43	39
44	37
40	32
43	42
43	38
44	41
42	35
44	29
42	37
41	35
43	25
43	37
43	31
45	29
43	29
43	32
41	32
44	37
42	40
43	37
43	32
42	28
45	32
44	35
42	28
42	34
43	30
43	41
41	35

45	32
41	27
44	29
42	33
41	36
44	38
44	28
44	38
42	31
43	33
42	32
45	40
42	36
43	37
44	31
44	31
41	36
45	38
44	31
43	32
44	33
41	32
42	33
44	29
43	38
44	36
44	26
39	36
45	39
44	35
45	33
42	29
42	38
43	31
44	32
44	33
44	37
43	32
44	31
43	32
45	27
42	37
43	37
43	31
45	28
42	34
44	38
42	42
41	34
44	37
45	33
42	32
45	34
43	31
43	41
45	25
44	28
44	36
42	30

43	31
40	31
43	34
42	30
42	33
43	29
44	36
43	36
44	38
45	43
41	33
41	30
44	32
44	29
43	37
43	32
43	33
43	35
45	29
42	33
44	28
43	36
41	35
42	34
45	29
44	32
45	34
45	30
44	28
42	27
44	30
39	35
43	29
45	35
43	36
45	36
43	39
41	34
42	33
41	35
45	31
43	29
44	38
42	34
45	38
42	38
42	32
46	32
42	33
43	30
43	40
45	34
43	34
40	38
44	33
45	28
42	38
44	43
43	41

42	37
43	34
44	34
42	35
43	34
41	38
45	37
42	36
46	36
40	28
44	33
44	33
46	25
43	39
44	30
44	37
43	24
44	37
43	26

9	10	11
TGAGGTAGTAGGTTGTATAGTT	AACATTCAACGCTGTCGGTGAGT	TAGCAGCACGTAAATATTGGCG
let-7a-5p	miR-181a-5p	miR-16-5p
8839402	8658137	7939871
1.72	2.00	0.25
1.83	0.60	1.32
3.13	1.47	2.02
2.31	1.05	0.56
5.24	2.09	1.80
3.37	1.76	0.60
2.83	3.10	0.18
1.76	2.75	0.20
2.32	1.02	1.00
1.92	0.68	1.06
1.58	1.26	1.62
2.12	2.41	0.15
2.00	1.94	2.99
2.23	2.96	0.24
2.05	1.72	1.88
1.73	1.78	2.67
2.82	3.77	0.40
1.28	2.21	2.45
1.15	2.27	1.26
1.32	1.90	4.28
2.15	2.37	0.86
2.69	3.41	0.16
2.95	3.26	0.28
2.67	3.24	0.24
5.40	2.47	0.33
2.89	3.76	0.49
2.64	1.14	2.91
2.05	0.54	5.71
1.42	0.83	0.96
1.11	0.62	0.55
2.15	0.57	0.20
1.73	0.57	1.06
4.58	1.02	5.05
1.77	3.13	0.37
1.48	2.53	0.72
2.05	2.45	0.25
1.28	1.25	0.13
1.42	2.60	0.71
1.05	2.40	1.63
1.79	3.97	0.43
0.55	4.18	0.13
0.37	6.13	0.07
1.83	2.87	0.50
1.12	1.46	1.60
0.83	2.29	0.48
0.28	5.43	0.18
1.55	2.13	2.43
2.20	3.94	0.50
1.10	2.30	1.80
1.35	2.80	0.87

1.14	3.08	2.09
2.44	1.66	0.28
3.06	1.19	0.51
2.56	1.72	0.99
2.44	1.95	0.48
2.43	2.06	0.60
2.17	1.41	1.29
1.73	1.65	4.97
2.68	4.46	0.36
3.80	3.43	1.44
3.81	3.15	1.79
1.68	1.28	5.69
2.71	2.72	0.37
3.21	1.99	0.70
3.43	1.84	2.27
2.49	3.15	0.36
0.63	0.55	3.85
1.50	0.65	0.55
6.97	2.66	0.86
3.38	3.02	1.34
1.98	1.38	0.38
0.64	0.98	6.05
2.35	1.27	1.89
1.97	2.11	1.97
0.48	4.04	0.01
2.10	1.87	1.76
1.42	1.47	1.19
2.04	1.43	1.30
2.57	1.19	2.72
1.82	1.49	3.91
2.22	1.96	0.92
2.44	2.00	1.59
1.11	2.90	0.33
1.10	2.43	3.51
1.60	1.12	6.56
0.85	2.31	1.07
1.75	2.41	1.24
1.91	2.07	1.11
1.58	1.70	2.41
1.80	1.77	1.04
4.09	1.86	1.69
1.79	1.78	0.29
3.37	3.72	0.33
3.03	1.75	1.06
1.60	2.31	1.29
0.00	0.00	0.00
0.00	0.00	0.00
0.00	0.00	0.00

10	11	12
C_41	*C_42*	*C_44*

0.40		
-0.37	-0.35	
0.11	0.10	-0.24
-0.26	-0.40	0.44
0.26	-0.01	-0.36
-0.05	0.32	-0.31
-0.36	-0.28	0.07
0.02	-0.24	-0.17
-0.04	0.25	-0.19
0.12	0.16	-0.20
0.05	-0.06	0.17
-0.63	-0.41	0.52
-0.66	-0.47	0.38
-0.39	-0.52	0.48
-0.15	0.01	-0.06
-0.28	0.03	-0.05
0.31	0.32	-0.45
0.26	0.24	-0.24
0.12	0.52	-0.24
0.56	0.49	-0.28
0.24	-0.05	-0.09
0.41	0.63	-0.30
0.15	0.27	-0.29
-0.25	-0.29	0.11
0.03	-0.25	-0.16
-0.31	-0.17	0.40
0.03	-0.25	0.50
0.19	0.07	-0.18
0.27	0.18	-0.33
-0.40	-0.03	0.34
-0.17	-0.28	0.00
-0.16	-0.19	-0.01
-0.39	-0.11	0.03
0.17	0.06	-0.36
-0.35	-0.20	0.45
-0.16	-0.31	0.16
-0.23	0.33	-0.27
-0.63	-0.23	0.22
-0.18	-0.05	-0.04
-0.39	-0.11	0.15
-0.24	0.05	-0.13
-0.26	-0.10	-0.13
-0.05	-0.15	-0.22
-0.28	-0.04	-0.18
-0.34	-0.21	0.44

-0.13	-0.09	-0.14
-0.01	-0.07	-0.16
0.23	0.14	-0.21
-0.50	-0.24	0.32
-0.33	0.01	-0.05
-0.14	0.13	-0.12
0.37	0.20	-0.27
-0.03	-0.23	0.11
-0.08	-0.13	0.16
0.22	0.23	-0.29
-0.04	-0.19	0.27
0.51	0.18	-0.43
0.36	0.38	-0.23
-0.04	-0.33	0.01
-0.26	-0.15	-0.13
-0.01	-0.37	0.22
0.14	0.13	0.04
0.49	0.05	-0.09
0.20	0.01	-0.04
-0.13	-0.22	0.00
-0.14	-0.14	0.16
0.20	0.45	-0.23
0.02	0.19	0.13
0.15	0.30	-0.51
-0.07	0.49	-0.41
-0.11	0.15	-0.24
-0.11	0.27	-0.25
-0.46	-0.25	0.51
0.15	0.14	-0.20
0.26	0.11	-0.19
-0.30	0.17	-0.04
-0.36	0.08	-0.07
-0.06	0.09	-0.05
0.08	0.22	-0.21
-0.24	0.16	-0.08
0.17	0.19	-0.08
-0.07	-0.14	0.13
-0.62	-0.43	0.35
0.16	0.43	-0.35
0.34	0.16	-0.38

8	9	10
110	111	112
TGGAGAGAAAGGCAGTTCCTGA	ACCATCGACCGTTGATTGTACC	TCCCCCAGGTGTGATTCTGATTT
miR-185-5p	miR-181a-3p	miR-361-3p
23397	23176	20718
-0.68	-0.85	1.41
-0.74	-0.38	0.02
0.66	-0.11	-0.04
-0.60	-0.91	-0.51
-0.24	-0.47	-0.17
-0.93	-1.21	-1.56
-0.93	-0.83	0.50
-0.97	-0.40	-0.74
-0.65	-0.80	-1.54
0.96	-0.84	-1.36
-0.13	0.90	-0.64
-1.13	-0.28	-0.03
1.87	1.90	-0.82
-0.87	-0.64	-0.03
1.16	0.54	0.25
0.41	1.25	0.04
-0.46	-0.18	0.92
0.98	2.26	-0.36
-0.20	1.80	0.41
2.88	1.94	-0.35

-0.77	-0.22	-0.11
-0.29	-0.25	1.91
-0.18	-0.33	2.64
-0.49	-0.94	1.15
-0.52	-0.16	-0.19
0.38	-0.34	0.47
1.47	-0.44	-1.29
2.23	0.32	-1.54
-0.60	1.55	-0.17
-0.63	-0.07	-0.88
-1.01	-1.09	-0.16
-0.01	0.77	-0.90
1.24	0.08	-1.42
-0.80	-0.59	0.37
-0.40	0.28	-0.13
-0.87	-0.07	1.91
-1.24	-1.26	-1.43
-0.51	0.50	-0.17
0.38	2.67	-0.16
-0.65	1.27	0.66
4.78	-1.40	1.57
5.90	-0.92	2.86
-0.82	-0.38	0.39
2.47	0.55	0.20
-0.98	1.16	0.82
2.35	-1.45	0.74
-0.25	0.92	0.35
-0.17	0.65	0.90
0.15	0.74	-0.05
-0.34	0.99	2.50
0.51	2.30	0.45
-0.71	-0.77	1.07
-0.61	-1.08	-0.04
0.60	-0.29	2.02
-1.04	-0.39	0.24
-0.75	-0.36	0.09
-0.45	-0.75	-0.34
8.04	1.39	-0.72
0.14	0.68	1.59
0.90	1.48	0.34
1.51	0.96	0.81
6.51	0.22	-1.36
-0.52	-1.20	-0.68
0.28	-0.82	-0.68
1.56	-0.28	-1.21
0.65	1.43	0.88
0.23	-0.60	-1.29
-1.34	0.23	-0.52
-0.07	-0.52	1.54
0.40	1.40	2.17
-0.47	-0.87	-0.08
-0.56	1.52	-1.41
-0.18	-0.56	-1.68
1.75	0.95	-0.36
-0.36	-1.10	8.02
0.96	0.38	0.26
-0.52	1.73	-0.16
-0.77	0.38	-0.59
1.69	2.25	-0.22

-0.17	0.97	-0.29
-0.45	-0.73	-0.31
-0.35	0.42	-0.03
-0.61	-0.54	0.52
3.05	2.86	0.26
3.74	1.60	-1.34
0.37	0.58	0.75
-0.19	0.91	1.08
-0.43	-0.67	-0.26
0.38	1.77	0.42
-0.29	-0.08	1.32
2.34	0.99	-0.62
-1.03	-0.58	0.28
-0.62	0.47	0.83
0.70	-0.09	-1.03
0.92	-0.44	-0.77
-1	-2	-2
0.00	0.00	0.00
1.00	1.00	1.00
0.33	0.20	0.85

110	111	112
TGGAGAGAAAGGCAGTTCCTGA	ACCATCGACCGTTGATTGTACC	TCCCCCAGGTGTGATTCTGATTT
miR-185-5p	miR-181a-3p	miR-361-3p
23397	23176	20718
-0.68	-0.85	1.41
-0.74	-0.38	0.02
0.66	-0.11	-0.04
-0.60	-0.91	-0.51
-0.24	-0.47	-0.17
-0.93	-1.21	-1.56
-0.93	-0.83	0.50
-0.97	-0.40	-0.74
-0.65	-0.80	-1.54
0.96	-0.84	-1.36
-0.13	0.90	-0.64
-1.13	-0.28	-0.03
1.87	1.90	-0.82
-0.87	-0.64	-0.03
1.16	0.54	0.25
0.41	1.25	0.04
-0.46	-0.18	0.92
0.98	2.26	-0.36
-0.20	1.80	0.41
2.88	1.94	-0.35
-0.77	-0.22	-0.11
-0.29	-0.25	1.91
-0.18	-0.33	2.64
-0.49	-0.94	1.15
-0.52	-0.16	-0.19
0.38	-0.34	0.47
1.47	-0.44	-1.29
2.23	0.32	-1.54

-0.60	1.55	-0.17
-0.63	-0.07	-0.88
-1.01	-1.09	-0.16
-0.01	0.77	-0.90
1.24	0.08	-1.42
-0.80	-0.59	0.37
-0.40	0.28	-0.13
-0.87	-0.07	1.91
-1.24	-1.26	-1.43
-0.51	0.50	-0.17
0.38	2.67	-0.16
-0.65	1.27	0.66
4.78	-1.40	1.57
5.90	-0.92	2.86
-0.82	-0.38	0.39
2.47	0.55	0.20
-0.98	1.16	0.82
2.35	-1.45	0.74
-0.25	0.92	0.35
-0.17	0.65	0.90
0.15	0.74	-0.05
-0.34	0.99	2.50
0.51	2.30	0.45
-0.71	-0.77	1.07
-0.61	-1.08	-0.04
0.60	-0.29	2.02
-1.04	-0.39	0.24
-0.75	-0.36	0.09
-0.45	-0.75	-0.34
8.04	1.39	-0.72
0.14	0.68	1.59
0.90	1.48	0.34
1.51	0.96	0.81
6.51	0.22	-1.36
-0.52	-1.20	-0.68
0.28	-0.82	-0.68
1.56	-0.28	-1.21
0.65	1.43	0.88
0.23	-0.60	-1.29
-1.34	0.23	-0.52
-0.07	-0.52	1.54
0.40	1.40	2.17
-0.47	-0.87	-0.08
-0.56	1.52	-1.41
-0.18	-0.56	-1.68
1.75	0.95	-0.36
-0.36	-1.10	8.02
0.96	0.38	0.26
-0.52	1.73	-0.16
-0.77	0.38	-0.59
1.69	2.25	-0.22
-0.17	0.97	-0.29
-0.45	-0.73	-0.31
-0.35	0.42	-0.03
-0.61	-0.54	0.52
3.05	2.86	0.26
3.74	1.60	-1.34
0.37	0.58	0.75
-0.19	0.91	1.08

-0.43	-0.67	-0.26
0.38	1.77	0.42
-0.29	-0.08	1.32
2.34	0.99	-0.62
-1.03	-0.58	0.28
-0.62	0.47	0.83
0.70	-0.09	-1.03
1	0	-1

110	111	112
46	101	80
AGCTACATCTGGCTACTGGGT	GCCCCTGGGCCTATCCTAGAA	ATCACATTGCCAGGGATTACC
miR-222-3p	miR-331-3p	miR-23b-3p
221365	26325.00	53026
0.56	4.12	0.09
-0.96	-1.02	-0.50
-0.09	0.00	-1.08
-0.40	-0.06	-1.12
-0.45	0.08	2.84
1.34	-0.99	-0.09
1.65	-0.14	0.59
-0.92	-0.46	-0.77
-1.72	-0.51	-0.80
-1.64	-0.14	0.17
-0.47	-0.59	1.04
0.20	-0.95	-1.08
0.88	-0.08	1.46
-1.51	0.11	-0.46
-0.48	0.56	-0.59
1.01	-0.43	2.25
-0.21	0.47	0.04
-0.88	1.30	1.06
0.37	1.24	-0.57
0.37	-0.39	-0.85
0.72	-0.77	-0.28
1.82	-0.27	-0.56
1.48	-0.11	-0.03
0.47	-0.20	-0.11
-0.96	-0.59	-0.22
0.45	0.03	0.45
-0.62	-0.23	-0.90
-1.11	-0.50	-1.07
1.40	-0.38	-0.73
-0.92	-0.08	-1.05
-1.38	-0.46	-0.81
0.51	-0.05	-1.08
-1.78	0.58	1.72
1.29	1.00	-0.49
0.40	2.00	0.51
2.02	0.67	0.03
-1.26	-0.94	-1.06
-0.22	0.54	2.57
0.02	1.55	2.06

0.42	0.32	-0.17
-0.04	6.21	-0.47
7.71	6.75	0.73
0.33	-0.07	0.17
-0.99	0.59	0.30
-0.07	-0.57	-0.58
-1.28	2.37	-0.96
3.70	-0.23	-0.37
3.47	-0.24	0.33
1.02	-0.34	-0.26
1.87	-0.36	-0.58
2.38	-0.33	0.52
3.13	0.48	1.11
1.13	0.50	0.63
1.27	0.01	0.65
4.19	-0.73	-0.66
0.59	0.14	-0.05
0.81	-0.59	-0.35
-1.18	-0.62	-0.92
1.34	-0.21	-0.58
3.04	-0.30	0.53
0.71	0.07	0.52
-1.74	-0.65	-1.15
-1.13	-0.34	-1.03
0.12	-0.65	-0.89
-1.15	-0.63	-1.09
-0.33	-0.61	-1.24
-0.94	0.83	-0.42
-0.36	-0.59	0.33
0.79	2.93	-0.04
4.54	1.23	-0.53
0.98	-0.18	-1.06
-0.94	-0.93	-1.16
-1.21	-0.65	-0.37
-0.07	-0.33	0.03
2.97	15.97	0.68
-0.06	-0.67	-0.44
-0.84	0.37	-0.61
-1.04	-0.97	-0.74
1.23	1.99	0.64
0.93	-0.27	0.02
-0.42	-0.05	-0.24
1.56	-0.33	-0.36
0.27	-0.90	-0.33
-0.07	-0.08	0.53
-1.45	-0.69	-1.10
2.03	0.25	-0.09
1.79	-0.20	-0.33
0.40	-0.60	-0.55
0.69	-0.15	-0.55
1.11	0.34	-0.17
-1.58	-0.96	-1.14
-0.23	-0.43	-0.74
1.56	-0.24	-0.60
-1.16	-0.73	-0.53
-0.89	0.44	0.31

0.90	1.00	1.00
0.53	0.33	1.00

P	ave
20	sd
19	
20	
21	
20	
24	
19	
21	
19	
16	
17	
19	
20	
17	
20	
23	
18	
19	
21	
21	
18	
19	
22	
20	
16	
24	
16	
15	
19	
19	
20	
20	
20	
21	
18	
16	
18	
18	
20	
21	
20	
18	
21	
19	
19	
20	
21	
19	
19	
20	
18	

17
20
16
24
20
19
19
15
20
19
16
17
18
20
20
19
16
19
20
21
18
19
16
23
16
18
19
18
18
18
18
18
21
17
19
23
18
20
22
21
19
17
17
19
21
17
19
21
21
18
17
20
18
19
18
19
18
20
17

14
14
18
18
20
17
18
14
16
19
18
20
19
20
20
21
19
18
18
23
20
16
18
17
20
18
19
18
20
15
15
18
20
18
17
21
17
18
20
19
19
18
15
20
20
17
16
18
16
18
20
17
22
20
19
23
21
19
17

19
18
20
18
21
21
19
19
17
21
16
19
23
19
19
19
17
19
15
18
20
15
22
20
19
19
19
16
17
20
21
17
17
18
20
17
19
21
21
15
19
20
20
18
17
18
18
23
15
20
20
19
15
16
19
16
21
18
19

17
16
22
18
19
15
19
16
17
20
15
16
18
20
20
18
19
20
18
20
19
21
18
21
19
17
18
20
17
18
20
18
21
19
17
15
21
15
21
21
16
16
19
16
18
16
16
23
20
20
20
19
15
21
18
20
19
22
18

15
15
18
19
19
20
18
21
22
15
22
21
18
21
18
19
18
18
19
16
19
19
17
17
21
20
20
19
21
17
18
19
18
18
16
20
19
18
18
18
19
21
22
14
19
18
20
18
18
16
18
22
21
15
17
19
19
19
18

18
19
18
15
20
19
20
16
21
22
19
19
19
17
18
20
19
15
19
18
19
21
18
23
16
18
19
17
21
19
16
18
20
20
17
18
22
19
16
16
19
19
14
21
20
19
19
18
19
19
23
16
18
21
20
22
20
21
19

18
21
20
20
20
18
20
17
16
18
18
16
18
20
18
21
19
17
18
20
15
19
19
17
16
17
19
16
18
20
22
16
18
17
19
18
16
17
16
21
18
19
17
18
22
23
16
18
19
14
18
18
15
19
15
18
19
21
19

19
20
18
19
17
20
20
17
17
21
18
19
19
20
16
17
17
18
18
19
21
18
16
19
19
20
22
17
21
20
16
17
20
21
17
19
18
17
18
16
19
21
17
16
21
19
14
19
18
19
18
18
19
18
18
20
22
18
20

19
18
21
19
21
18
17
23
18
19
19
15
20
18
18
16
21
23
18
14
18
19
18
17
20
15
18
19
17
15
18
17
16
21
19
15
20
22
17
18
19
17
21
21
18
16
19
21
16
21
21
19
17
18
19
22
18
21

20
22
20
19
20
21
20
18
19
21
20
17
21
21
18
18
21
21
18
20
22
16
21
19
21
19
14
19
18
21
15
18
19
19
18
19
21
21
19
21
21
21
18
22
23
20
19
20
19
21
23
21
21
19
17
19
17
23
20

21
18
18
22
19
21
19
19
20
18
21
18
20
18
19
23
21
20
19
21
20
21
22
18
18
22
18
18
19
20
19
18
16
20
16
18
20
21
17
21
17
17
21
15
19
19
14
19
22
20
20
22
22
16
22
19
19
18
20

19
18
17
22
19
24
21
19
20
20
19
17
15
16
20
20
20
16
19
21
21
19
23
19
15
20
19
19
13
21
21
20
16
20
22
18
17
23
18
18
16
18
15
20
20
21
23
20
19
20
17
18
19
19
17
19
18
16
22

20
17
17
21
19
21
17
17
20
21
24
21
20
18
20
15
21
19
21
18
16
18
17
18
16
21
17
18
15
17
20
19
19
22
21
14
20
16
15
14
17
21
25
16
18
19
20
22
20
22
17
14
18
19
18
23
17
17
16

22
18
21
21
16
20
16
19
21
19
18
19
16
21
20
18
16
15
18
15
22
20
15
19
14
18
24
20
21
22
18
19
20
24
14
20
20
18
20
17
15
17
17
20
20
16
18
19
16
20
21
18
20
23
22
21
20
18
18

18
22
18
18
20
19
22
19
21
18
18
19
20
18
18
18
17
21
18
19
17
19
18
18
18
22
17
17
19
21
21
20
19
23
19
21
20
22
19
20
22
20
16
16
19
19
20
18
18
19
21
19
19
18
14
17
16
20
17

22
17
19
21
19
17
14
19
17
17
18
19
19
15
19
22
20
20
20
21
18
17
15
21
22
17
20
16
20
17
19
18
21
17
21
17
21
19
17
23
20
19
16
17
20
17
18
18
17
18
20
20
21
16
16
21
19
16
21

17
15
18
19
20

21
15
18
17
16
21
19
15
20
22
17
18
19
17
21
21

18
16
19
21
16
21
21
19
17
18
19
22
18
21
20
22
20
19
20
21
20
18
19
21
20
17
21
21
18
18
21
21
18
20
22
16
21
19
21
19
14
19
18
21
15
18
19
19
18
19
21
21
19
21
21
21
18
22
23

20
19
20
19
21
23
21
21
19
17
19
17
23
20
21
18
18
22
19
21
19
19
20
18
21
18
20
18
19
23
21
20
19
21
20
21
22
18
18
22
18
18
19
20
19
18
16
20
16
18
20
21
17
21
17
17
21
15
19

19
14
19
22
20
20
22
22
16
22
19
19
18
20
19
18
17
22
19
24
21
19
20
20
19
17
15
16
20
20
20
16
19
21
21
19
23
19
15
20
19
19
13
21
21
20
16
20
22
18
17
23
18
18
16
18
15
20
20

21
23
20
19
20
17
18
19
19
17
19
18
16
22
20
17
17
21
19
21
17
17
20
21
24
21
20
18
20
15
21
19
21
18
16
18
17
18
16
21
17
18
15
17
20
19
19
22
21
14
20
16
15
14
17
21
25
16
18

19
20
22
20
22
17
14
18
19
18
23
17
17
16
22
18
21
21
16
20
16
19
21
19
18
19
16
21
20
18
16
15
18
15
22
20
15
19
14
18
24
20
21
22
18
19
20
24
14
20
20
18
20
17
15
17
17
20
20

16
18
19
16
20
21
18
20
23
22
21
20
18
18
18
22
18
18
20
19
22
19
21
18
18
19
20
18
18
18
17
21
18
19
17
19
18
18
18
22
17
17
19
21
21
20
19
23
19
21
20
22
19
20
22
20
16
16
19

19
20
18
18
19
21
19
19
18
14
17
16
20
17
22
17
19
21
19
17
14
19
17
17
18
19
19
15
19
22
20
20
20
21
18
17
15
21
22
17
20
16
20
17
19
18
21
17
21
17
21
19
17
23
20
19
16
17
20

17
18
18
17
18
20
20
21
16
16
21
19
16
21
17
15
18
19
20

12	13	14
TTCAAGTAATCCAGGATAGGCT	AAACCGTTACCATTACTGAGTT	AAGCTGCCAGTTGAAGAACTGT
miR-26a-5p	miR-451a	miR-22-3p
5953261	3762724	3462453
0.46	0.07	0.40
1.01	0.20	0.12
1.17	0.40	1.47
0.58	0.12	0.60
2.83	0.07	0.09
1.46	0.01	0.26
0.78	0.02	0.21
0.55	0.03	0.14
0.67	0.22	0.58
0.80	0.33	0.42
1.87	0.51	0.60
0.54	0.05	0.24
2.15	1.71	1.44
0.47	0.05	0.15
0.77	0.30	0.98
1.59	0.38	1.69
1.25	0.05	0.36
0.86	0.72	1.21
1.34	0.35	0.50
2.01	2.43	1.37
0.72	0.06	0.43
0.97	0.03	0.20
0.87	0.08	0.29
0.48	0.04	0.24
1.38	0.06	0.08
1.99	0.07	0.47
1.92	0.88	1.42
1.73	3.19	1.67
1.76	0.14	0.55
0.62	0.26	0.38
0.28	0.05	0.12
1.04	0.63	0.30
3.61	0.51	0.17
0.72	0.08	0.25
0.53	0.42	0.23
1.05	0.06	0.23
0.14	0.01	0.07
1.45	0.34	0.41
1.53	5.87	0.83
1.64	0.12	0.34
1.03	0.12	0.20
1.24	0.42	0.46
1.01	0.04	0.31
1.56	0.55	0.86
0.65	0.06	0.34
0.84	0.06	0.20
3.31	0.32	1.06
1.47	0.09	0.53
1.24	0.50	1.12
1.57	0.18	0.62

1.54	0.40	1.37
1.14	0.04	0.27
0.81	0.05	0.29
1.94	0.11	0.85
0.88	0.03	0.35
0.96	0.04	0.31
1.09	0.07	0.81
2.16	3.76	1.95
1.52	0.05	0.49
2.91	0.24	0.63
3.09	0.30	0.35
2.14	2.59	1.83
0.96	0.09	0.26
1.92	0.11	0.54
1.94	0.34	1.97
0.32	0.29	0.38
0.70	1.11	2.22
0.79	0.14	0.11
0.91	0.18	0.28
1.80	0.26	0.63
0.76	0.09	0.44
1.41	0.26	0.14
1.04	0.23	0.78
1.32	0.97	0.93
0.20	0.14	0.13
1.04	0.23	1.38
1.34	0.34	0.37
1.00	0.06	0.43
1.71	1.21	1.14
2.96	0.48	1.46
1.61	0.12	0.49
2.41	0.25	0.81
1.10	0.08	0.39
2.25	4.28	1.50
1.60	5.13	1.22
1.94	0.44	0.69
2.53	0.15	0.86
1.96	0.09	0.58
1.41	0.61	0.96
1.66	0.14	0.76
1.27	0.68	0.61
0.66	0.03	0.23
0.90	0.04	0.37
1.87	0.17	0.79
1.82	0.26	0.83
0.00	0.00	0.00
0.00	0.00	0.00
0.00	0.00	0.00

13	14	15
C_46	*C_47*	*D_16*

-0.50		
0.13	-0.22	
0.54	-0.57	0.16
-0.36	0.36	0.10
0.09	-0.04	0.45
0.24	-0.21	0.24
0.52	-0.37	0.18
-0.10	-0.01	-0.19
-0.41	0.41	-0.27
-0.40	0.42	-0.22
-0.49	0.65	-0.28
-0.33	0.17	-0.31
-0.15	-0.05	-0.22
0.35	-0.50	0.36
0.42	-0.29	0.12
0.20	-0.43	0.03
0.01	-0.21	0.23
-0.41	0.31	-0.02
0.08	-0.28	0.03
0.15	-0.31	-0.02
-0.03	0.28	0.01
0.07	0.24	0.11
-0.10	0.09	-0.25
-0.32	0.56	-0.23
0.38	-0.24	0.10
0.63	-0.32	0.23
0.05	0.13	-0.15
-0.21	0.27	-0.05
-0.13	0.16	-0.07
-0.38	0.31	-0.22
0.58	-0.37	0.39
-0.25	0.38	-0.18
-0.32	0.35	-0.13
0.30	-0.50	0.05
-0.12	0.10	-0.21
0.20	-0.14	0.15
-0.03	-0.03	0.03
0.18	-0.23	0.07
-0.34	0.08	-0.16
-0.36	0.19	-0.08
0.20	-0.37	0.03
-0.23	0.16	-0.38

-0.46	0.18	-0.06
-0.19	-0.10	-0.07
0.47	-0.31	0.15
-0.34	0.17	-0.30
0.23	-0.40	-0.21
0.16	-0.36	-0.20
0.44	-0.39	0.25
-0.31	0.26	-0.04
-0.17	-0.11	-0.14
0.21	-0.29	0.35
0.06	0.18	0.19
0.38	-0.43	0.37
-0.14	-0.17	0.02
-0.22	0.22	-0.03
0.14	-0.16	0.07
-0.11	0.21	0.05
0.44	-0.32	-0.03
0.31	-0.27	0.24
0.56	-0.19	0.17
-0.21	0.24	-0.06
0.41	-0.21	0.16
0.19	-0.27	0.25
-0.11	-0.03	-0.13
0.41	-0.54	0.46
0.35	-0.55	0.19
-0.24	0.13	0.07
0.14	-0.48	-0.06
-0.13	0.33	-0.32
0.55	-0.37	0.09
0.40	-0.30	0.14
0.08	-0.09	0.02
0.26	-0.37	0.02
0.03	-0.25	0.00
0.56	-0.51	0.38
0.05	-0.18	-0.01
0.35	-0.22	0.18
-0.06	0.08	-0.06
-0.32	0.31	-0.25
0.20	-0.32	0.20
0.10	-0.34	0.11

11	12	13
113	114	115
GTCCAGTTTTCCCAGGAATCCCT	CAAAGTGCTCATAGTGCAGGTAG	AGGCAAGATGCTGGCATAGCT
miR-145-5p	miR-20b-5p	miR-31-5p
20235	19721	18520
1.29	-0.65	-0.50
-1.36	-1.33	-1.48
-0.50	2.10	-0.21
-0.60	0.65	-0.93
-0.24	0.43	1.96
-1.07	-0.97	-0.74
-0.22	-0.90	0.74
0.54	-1.06	-0.46
-0.77	0.14	-1.00
-0.92	0.25	-0.84
-0.71	0.86	0.28
-0.88	-1.20	0.64
1.49	0.32	-0.20
0.50	-0.91	-0.23
-0.06	1.15	-0.73
-0.32	0.59	-0.07
0.83	-0.20	0.82
0.22	0.93	0.41
2.10	-0.28	-0.83
-0.52	1.59	0.21

-0.99	-0.46	-0.98
-0.01	-0.92	2.31
-0.08	-0.55	0.71
-0.75	-0.74	0.41
0.51	-0.78	-0.47
2.78	-0.29	2.20
-0.27	2.22	-1.01
-0.80	4.25	-0.43
-0.30	0.11	-0.45
-0.32	0.59	-1.21
-0.88	0.91	-1.37
-0.97	3.39	-1.15
0.19	3.13	-0.43
2.55	-0.72	-1.09
8.36	-0.44	-1.18
0.31	-1.07	4.13
-1.19	-1.22	-1.28
1.23	-0.54	1.78
2.63	0.85	1.65
0.24	-0.79	1.57
9.01	-0.80	-0.25
18.44	-0.89	5.93
1.13	-0.59	-0.44
1.89	0.18	-1.00
1.54	-1.07	-0.17
1.13	-1.00	-0.51
0.21	-0.02	0.00
-0.04	-0.90	1.19
0.07	-0.29	-0.20
-0.42	-0.49	2.30
-0.45	0.15	1.10
0.23	-0.85	0.22
0.17	-0.30	-1.19
1.06	-0.25	0.43
-1.05	-0.98	-0.40
-0.07	-0.39	-0.30
-0.72	-0.41	-0.03
-0.81	3.54	0.81
1.63	-0.55	2.36
1.23	0.24	1.36
1.52	0.28	1.12
-0.70	6.13	-0.05
0.89	-0.58	-0.47
-0.49	-0.32	0.04
-0.63	1.52	-0.94
-0.36	-0.21	-0.96
-0.18	0.94	-1.25
-0.55	2.11	-0.11
1.38	2.51	-0.71
1.99	3.05	0.41
-0.03	-0.43	-0.72
-0.52	-0.56	-0.89
-0.88	-0.37	-0.61
0.83	-0.07	0.43
23.97	-1.33	-0.09
-0.61	0.04	1.16
0.80	0.59	0.44
-1.03	-1.02	-1.29
-0.37	3.65	0.16

-0.53	-0.04	-0.43
-0.85	0.02	-0.04
-0.57	-0.16	-0.18
0.21	-0.78	0.69
0.03	0.64	1.83
-1.15	3.00	0.24
1.63	-0.56	-0.08
-0.04	-0.42	0.88
-0.46	-0.41	0.78
-0.10	0.45	0.01
0.54	-0.29	0.82
-1.14	0.77	-0.94
-0.44	-0.83	-0.86
-0.13	-0.77	0.55
-0.40	1.39	-1.13
1.03	-0.16	-0.98
-1	1	0
0.00	0.00	0.00
1.00	1.00	1.00
0.64	0.75	0.21

113	114	115
GTCCAGTTTTCCCAGGAATCCCT	CAAAGTGCTCATAGTGCAGGTAG	AGGCAAGATGCTGGCATAGCT
miR-145-5p	miR-20b-5p	miR-31-5p
20235	19721	18520
1.29	-0.65	-0.50
-1.36	-1.33	-1.48
-0.50	2.10	-0.21
-0.60	0.65	-0.93
-0.24	0.43	1.96
-1.07	-0.97	-0.74
-0.22	-0.90	0.74
0.54	-1.06	-0.46
-0.77	0.14	-1.00
-0.92	0.25	-0.84
-0.71	0.86	0.28
-0.88	-1.20	0.64
1.49	0.32	-0.20
0.50	-0.91	-0.23
-0.06	1.15	-0.73
-0.32	0.59	-0.07
0.83	-0.20	0.82
0.22	0.93	0.41
2.10	-0.28	-0.83
-0.52	1.59	0.21
-0.99	-0.46	-0.98
-0.01	-0.92	2.31
-0.08	-0.55	0.71
-0.75	-0.74	0.41
0.51	-0.78	-0.47
2.78	-0.29	2.20
-0.27	2.22	-1.01
-0.80	4.25	-0.43

-0.30	0.11	-0.45
-0.32	0.59	-1.21
-0.88	0.91	-1.37
-0.97	3.39	-1.15
0.19	3.13	-0.43
2.55	-0.72	-1.09
8.36	-0.44	-1.18
0.31	-1.07	4.13
-1.19	-1.22	-1.28
1.23	-0.54	1.78
2.63	0.85	1.65
0.24	-0.79	1.57
9.01	-0.80	-0.25
18.44	-0.89	5.93
1.13	-0.59	-0.44
1.89	0.18	-1.00
1.54	-1.07	-0.17
1.13	-1.00	-0.51
0.21	-0.02	0.00
-0.04	-0.90	1.19
0.07	-0.29	-0.20
-0.42	-0.49	2.30
-0.45	0.15	1.10
0.23	-0.85	0.22
0.17	-0.30	-1.19
1.06	-0.25	0.43
-1.05	-0.98	-0.40
-0.07	-0.39	-0.30
-0.72	-0.41	-0.03
-0.81	3.54	0.81
1.63	-0.55	2.36
1.23	0.24	1.36
1.52	0.28	1.12
-0.70	6.13	-0.05
0.89	-0.58	-0.47
-0.49	-0.32	0.04
-0.63	1.52	-0.94
-0.36	-0.21	-0.96
-0.18	0.94	-1.25
-0.55	2.11	-0.11
1.38	2.51	-0.71
1.99	3.05	0.41
-0.03	-0.43	-0.72
-0.52	-0.56	-0.89
-0.88	-0.37	-0.61
0.83	-0.07	0.43
23.97	-1.33	-0.09
-0.61	0.04	1.16
0.80	0.59	0.44
-1.03	-1.02	-1.29
-0.37	3.65	0.16
-0.53	-0.04	-0.43
-0.85	0.02	-0.04
-0.57	-0.16	-0.18
0.21	-0.78	0.69
0.03	0.64	1.83
-1.15	3.00	0.24
1.63	-0.56	-0.08
-0.04	-0.42	0.88

-0.46	-0.41	0.78
-0.10	0.45	0.01
0.54	-0.29	0.82
-1.14	0.77	-0.94
-0.44	-0.83	-0.86
-0.13	-0.77	0.55
-0.40	1.39	-1.13
1	0	-1

113	114	115
14	18	54
TGAGGTAGTAGGTTGTGTGGTT	AGCAGCATTGTACAGGGCTATGA	CTTTCAGTCGGATGTTTACAGC
let-7b-5p	miR-103a-3p	miR-30e-3p
1666971	1030775	138740
2.23	-0.02	-0.84
-1.07	0.44	-0.78
1.24	0.76	0.98
1.04	-0.33	-0.11
0.48	1.38	-0.71
-0.45	-1.13	-0.26
-0.41	-0.23	-0.23
0.48	-1.13	-0.11
-0.41	-1.03	-0.63
-0.23	-0.64	-0.81
-1.34	-0.83	1.28
-0.25	-0.68	0.01
-0.88	1.13	1.70
1.60	-0.92	-0.45
-0.34	1.53	0.33
-1.43	1.05	0.87
0.63	-0.43	-0.73
0.27	2.86	-0.44
-1.58	0.86	3.16
-0.97	0.72	1.62
-0.67	-0.17	-0.02
-0.06	-0.09	-0.26
0.73	0.11	-0.44
1.83	-0.49	-1.03
0.38	-1.15	-1.02
-0.14	-0.73	-0.81
-0.67	-0.82	-0.28
-0.97	0.06	1.10
-1.69	-0.14	5.47
-1.30	0.20	2.51
1.11	-0.06	-0.58
-1.17	1.21	3.78
-0.06	1.17	-0.43
0.14	-0.75	0.68
1.18	0.28	-0.35
-1.14	-0.77	0.15
2.07	-1.84	-1.32
-1.12	0.40	1.26
-1.06	0.90	0.81

-1.01	-0.21	0.56
1.50	0.41	-1.32
3.71	2.61	-1.18
-0.60	-0.28	-0.63
-1.59	1.57	1.23
-1.23	-0.39	0.95
1.47	-1.04	-1.41
-1.66	0.06	1.22
-0.98	0.33	0.38
-0.77	0.95	-0.62
-0.95	0.15	-0.45
-1.23	1.00	-0.57
-0.59	-0.13	-0.40
0.56	-0.25	-0.87
-0.76	1.50	-0.12
-0.60	-0.67	0.10
0.13	-0.59	-0.37
-0.62	-0.15	-0.61
-1.21	-0.66	-0.72
0.12	-0.80	-0.64
-0.12	0.71	-0.56
-0.54	0.50	-1.07
-1.03	-0.58	-0.60
1.15	-1.46	-1.10
0.18	-1.29	-1.16
0.18	-1.08	-0.93
0.24	1.76	5.98
-0.27	-0.36	-0.63
-1.02	2.03	1.81
6.59	3.48	-0.39
1.48	3.75	2.28
-0.02	0.11	0.65
-2.12	-0.96	5.13
0.21	-0.55	-0.17
0.87	1.06	0.11
8.15	0.14	-1.28
-0.75	0.57	0.05
-1.54	0.84	2.62
-0.96	-0.12	3.69
-0.74	2.68	-0.11
-1.32	0.10	0.80
0.20	-0.84	-0.82
-0.79	-0.11	0.08
-1.22	-0.49	-1.03
-1.32	1.38	0.12
-1.20	0.40	-0.27
-1.22	0.02	-0.05
-1.02	0.41	0.26
-0.66	-0.34	-0.43
-1.48	1.72	2.31
-1.02	0.17	0.20
-0.62	1.07	1.71
-1.42	-0.30	0.67
0.27	-0.31	-0.19
-1.02	-0.82	-0.01
-0.16	-0.71	-1.20
1.03	1.03	1.04
0.78	1.00	1.00

NP	P	UC
43.20	33.67	18.85
1.44	3.92	2.06

gamma, beta fits of random label, random selection

NP	P	UC
K-S	K-S	K-S
A-D	A-D	A-D
alpha	alpha	alpha
74.81	85.72	878.84
beta	beta	beta
0.90	0.44	0.10
p-val of ave of observed		
0.00	0.17	0.00
observed aves		
43.20	33.67	18.85

15	16	17
CATTGCACTTGTCTCGGTCTGA	TGAGGTAGTAGTTTGTGCTGTT	TGTAAACATCCCCGACTGGAAG
miR-25-3p	let-7i-5p	miR-30d-5p
3413660	2186845	2131733
1.21	0.86	0.64
0.87	0.24	0.36
1.77	0.71	0.57
1.29	0.82	0.43
0.40	0.27	0.45
0.67	1.09	0.32
0.37	0.79	0.53
0.34	0.22	0.58
1.13	0.32	0.39
1.27	0.49	0.33
0.78	0.21	0.45
0.32	0.52	0.42
0.80	0.98	0.49
0.44	0.18	0.46
1.05	0.43	0.45
0.69	0.49	0.59
0.49	0.34	0.58
1.06	0.41	0.68
0.99	0.30	0.37
0.63	0.27	0.65
0.85	0.84	0.32
0.35	0.30	0.61
0.35	0.39	0.67
0.26	0.34	0.56
0.35	0.09	0.38
0.60	0.38	0.54
1.18	0.45	0.47
1.20	0.54	0.37
1.18	0.50	0.28
1.43	0.50	0.22
1.16	0.67	0.37
2.07	0.73	0.26
0.82	0.24	0.41
0.70	0.32	0.45
1.13	0.39	0.44
0.67	0.35	0.40
0.21	0.22	0.27
1.60	1.28	0.41
1.94	1.26	0.47
0.37	0.20	0.55
0.29	1.11	0.40
0.21	3.48	0.41
0.44	0.41	0.54
1.52	0.97	0.33
0.41	0.13	0.42
0.13	0.60	0.40
0.58	0.26	0.58
0.43	0.31	0.70
0.47	0.28	0.89
0.42	0.24	0.70

0.52	0.34	0.82
0.42	0.47	0.57
0.55	0.53	0.40
0.48	0.60	0.58
0.49	0.46	0.41
0.48	0.27	0.45
0.66	0.72	0.51
0.80	0.54	0.37
0.66	0.32	0.57
0.60	0.53	0.77
0.42	0.27	0.63
1.07	0.49	0.36
0.49	0.23	0.52
0.52	0.65	0.46
1.00	0.62	0.43
1.85	2.07	0.55
3.58	1.08	0.40
2.20	0.82	0.25
1.53	1.10	1.09
1.40	0.83	1.11
1.14	0.80	0.48
0.79	1.15	0.38
1.37	0.89	0.41
0.72	0.49	0.60
0.15	0.86	0.37
0.60	0.80	0.45
1.12	0.34	0.32
1.13	0.40	0.28
1.04	0.59	0.56
0.68	0.28	0.57
0.52	0.16	0.57
0.76	0.46	0.41
0.13	0.21	0.63
0.62	0.45	0.62
0.82	0.44	0.41
0.48	0.18	0.57
0.47	0.35	0.61
0.64	0.51	0.46
0.95	0.43	0.44
0.57	0.28	0.46
1.21	0.81	0.48
0.82	0.63	0.28
0.49	0.40	0.69
0.99	0.45	0.32
2.06	1.26	0.45
0.00	0.00	0.00
0.00	0.00	0.00
0.00	0.00	0.00

16	17	18
D_17	*D_18*	*D_36*

-0.24		
0.19	0.08	
0.18	0.07	0.03
0.30	-0.28	0.05
0.00	-0.19	-0.17
-0.28	0.46	-0.01
-0.19	0.54	0.00
-0.41	0.44	-0.04
-0.31	-0.07	-0.32
0.00	0.36	-0.12
0.43	-0.27	0.11
0.28	-0.38	0.00
0.23	-0.25	-0.23
-0.06	-0.32	-0.09
-0.37	-0.13	-0.11
0.06	-0.33	-0.16
0.17	-0.37	-0.07
-0.20	0.20	0.02
-0.15	0.16	0.19
-0.16	0.29	-0.17
-0.41	-0.14	-0.13
0.17	-0.05	0.10
0.31	-0.16	0.19
0.01	0.39	-0.05
-0.25	0.48	0.14
-0.10	0.43	0.16
0.01	0.50	-0.04
0.38	-0.08	0.11
-0.18	0.18	0.00
-0.30	0.44	0.12
0.51	0.07	-0.01
0.09	0.37	0.04
0.40	0.03	0.36
0.16	0.39	0.19
0.56	0.11	0.42
0.00	0.28	-0.05
-0.13	0.16	-0.03
0.46	0.12	0.04
-0.07	-0.02	-0.25

-0.21	0.43	-0.06
0.15	-0.10	0.09
0.27	-0.34	0.02
-0.26	0.31	-0.14
0.36	-0.16	-0.10
0.26	-0.21	-0.10
0.28	-0.41	0.05
-0.34	0.31	-0.12
-0.03	0.13	-0.24
0.21	-0.04	0.02
-0.13	-0.05	0.14
0.31	-0.30	0.30
-0.18	-0.24	-0.13
-0.29	-0.09	0.04
0.16	-0.10	0.16
-0.24	-0.09	0.06
0.29	-0.36	0.09
0.13	-0.37	0.14
0.26	-0.24	0.29
-0.19	0.42	0.17
0.45	-0.24	0.23
0.11	-0.14	-0.07
-0.01	-0.21	-0.09
0.47	-0.01	0.25
0.64	-0.01	0.02
-0.04	0.40	-0.09
0.35	0.01	-0.08
0.02	0.27	0.05
0.27	-0.26	0.07
0.15	-0.35	0.01
0.32	0.28	0.10
0.41	0.22	0.08
0.16	0.00	-0.03
0.41	-0.18	0.15
0.28	0.17	0.01
0.09	-0.28	-0.05
-0.13	0.08	-0.11
-0.20	0.60	0.00
0.29	-0.06	-0.22
0.24	0.08	-0.04

14	15	16
116	117	118
TGAGGTAGTAGGTTGTATGGTT	TGTAAACATCCTCGACTGGAAG	ACAGTAGTCTGCACATTGGTTA
let-7c	miR-30a-5p	miR-199a-3p
18147	16585	16186
0.13	0.81	-0.55
1.32	-2.04	-0.68
0.55	-0.73	-0.47
-0.11	-1.10	-0.58
-0.41	-1.73	1.93
-0.54	-0.61	-0.07
0.83	0.33	0.30
-1.01	-0.27	-0.86
-0.58	-0.07	-0.96
-1.00	-1.41	-0.85
-1.11	0.21	0.00
1.17	-0.32	-0.78
-0.27	1.06	2.25
-0.49	1.66	-1.02
-0.64	0.53	-0.64
-0.41	0.27	2.80
0.37	1.58	-0.92
-0.71	0.35	0.00
-1.29	0.95	-0.66
-1.18	1.77	0.90

-0.27	-0.79	-0.15
2.87	0.19	-0.20
0.97	0.65	0.98
0.48	-0.93	-0.17
1.76	-0.83	0.47
0.32	0.02	-0.33
-0.76	0.47	0.26
-0.32	5.68	1.89
-0.66	-0.22	1.02
-1.47	-0.89	-0.86
-0.70	-0.17	-0.98
-0.95	2.08	0.75
-0.26	-1.44	4.41
-0.59	-0.52	-0.46
-0.64	-0.26	-0.52
4.97	-0.37	-0.77
-0.38	-1.11	-1.22
-0.49	0.33	-0.34
-0.98	3.52	0.64
-0.36	1.24	-0.61
-0.91	-1.11	0.13
0.59	0.29	1.26
-0.01	0.15	-0.09
-1.50	2.85	2.03
-0.69	-1.20	-0.76
-1.73	-1.25	-0.54
-1.07	0.94	0.93
0.38	0.20	0.16
-1.24	-0.05	0.44
-1.23	-0.17	-0.38
-1.00	0.93	1.69
1.16	-1.08	0.17
0.25	-0.87	-0.17
0.59	-0.47	2.07
0.36	-0.85	0.18
0.09	-1.08	-0.43
0.43	-0.66	0.58
-1.15	4.39	1.95
1.64	-0.74	-0.39
1.58	-0.13	2.51
0.48	-0.97	2.77
-1.27	-0.13	0.92
-1.04	-1.21	-0.93
0.07	-0.82	0.18
-0.19	4.46	-0.36
0.78	4.44	-0.55
-1.28	-0.61	0.55
1.60	0.30	-0.84
2.96	0.11	0.80
1.34	1.46	1.87
0.14	-0.71	-0.45
-1.78	-0.12	2.16
-0.83	-1.13	-0.33
-0.55	-0.20	0.00
0.72	-1.56	-1.12
0.14	1.81	0.77
-1.07	0.29	-0.63
0.64	-1.39	0.04
-0.32	0.79	0.64

-0.94	1.89	0.69
-0.94	2.55	-1.01
-0.83	-0.70	0.35
-1.38	0.51	-0.33
-0.40	0.62	1.64
-1.35	-0.14	0.28
-1.14	1.18	-0.06
-0.52	0.13	0.29
-0.22	-0.27	-0.51
-0.82	-0.47	0.13
-0.66	1.35	-0.16
0.39	5.62	0.02
-1.22	-0.82	-0.59
1.12	-0.77	-0.18
-0.68	4.11	0.27
-0.05	-0.74	0.85
-1	0	0
0.00	0.00	0.00
1.00	1.00	1.00
0.92	0.38	0.21

116	117	118
TGAGGTAGTAGGTTGTATGGTT	TGTAAACATCCTCGACTGGAAG	ACAGTAGTCTGCACATTGGTTA
let-7c	miR-30a-5p	miR-199a-3p
18147	16585	16186
0.13	0.81	-0.55
1.32	-2.04	-0.68
0.55	-0.73	-0.47
-0.11	-1.10	-0.58
-0.41	-1.73	1.93
-0.54	-0.61	-0.07
0.83	0.33	0.30
-1.01	-0.27	-0.86
-0.58	-0.07	-0.96
-1.00	-1.41	-0.85
-1.11	0.21	0.00
1.17	-0.32	-0.78
-0.27	1.06	2.25
-0.49	1.66	-1.02
-0.64	0.53	-0.64
-0.41	0.27	2.80
0.37	1.58	-0.92
-0.71	0.35	0.00
-1.29	0.95	-0.66
-1.18	1.77	0.90
-0.27	-0.79	-0.15
2.87	0.19	-0.20
0.97	0.65	0.98
0.48	-0.93	-0.17
1.76	-0.83	0.47
0.32	0.02	-0.33
-0.76	0.47	0.26
-0.32	5.68	1.89

-0.66	-0.22	1.02
-1.47	-0.89	-0.86
-0.70	-0.17	-0.98
-0.95	2.08	0.75
-0.26	-1.44	4.41
-0.59	-0.52	-0.46
-0.64	-0.26	-0.52
4.97	-0.37	-0.77
-0.38	-1.11	-1.22
-0.49	0.33	-0.34
-0.98	3.52	0.64
-0.36	1.24	-0.61
-0.91	-1.11	0.13
0.59	0.29	1.26
-0.01	0.15	-0.09
-1.50	2.85	2.03
-0.69	-1.20	-0.76
-1.73	-1.25	-0.54
-1.07	0.94	0.93
0.38	0.20	0.16
-1.24	-0.05	0.44
-1.23	-0.17	-0.38
-1.00	0.93	1.69
1.16	-1.08	0.17
0.25	-0.87	-0.17
0.59	-0.47	2.07
0.36	-0.85	0.18
0.09	-1.08	-0.43
0.43	-0.66	0.58
-1.15	4.39	1.95
1.64	-0.74	-0.39
1.58	-0.13	2.51
0.48	-0.97	2.77
-1.27	-0.13	0.92
-1.04	-1.21	-0.93
0.07	-0.82	0.18
-0.19	4.46	-0.36
0.78	4.44	-0.55
-1.28	-0.61	0.55
1.60	0.30	-0.84
2.96	0.11	0.80
1.34	1.46	1.87
0.14	-0.71	-0.45
-1.78	-0.12	2.16
-0.83	-1.13	-0.33
-0.55	-0.20	0.00
0.72	-1.56	-1.12
0.14	1.81	0.77
-1.07	0.29	-0.63
0.64	-1.39	0.04
-0.32	0.79	0.64
-0.94	1.89	0.69
-0.94	2.55	-1.01
-0.83	-0.70	0.35
-1.38	0.51	-0.33
-0.40	0.62	1.64
-1.35	-0.14	0.28
-1.14	1.18	-0.06
-0.52	0.13	0.29

-0.22	-0.27	-0.51
-0.82	-0.47	0.13
-0.66	1.35	-0.16
0.39	5.62	0.02
-1.22	-0.82	-0.59
1.12	-0.77	-0.18
-0.68	4.11	0.27
0	-1	1

116	117	118
10	121	41
CATTGCACTTGTCTCGGTCTGA	ACTGCCCCAGGTGCTGCTGG	TTCACCACCTTCTCCACCCAGC
miR-25-3p	miR-324-3p	miR-197-3p
3413660	15277	286288
1.16	3.54	0.21
0.27	-1.28	1.16
2.58	-0.31	-0.97
1.36	0.36	-0.75
-0.92	-0.93	1.99
-0.22	-0.48	-1.22
-0.99	0.27	-0.04
-1.07	-0.09	1.10
0.96	-0.44	-0.71
1.31	0.13	-0.46
0.04	-0.69	-0.38
-1.13	-0.69	-0.84
0.10	0.22	-0.01
-0.82	-0.23	0.25
0.75	-0.14	-0.10
-0.18	0.06	-0.52
-0.68	0.68	0.19
0.77	2.45	-0.47
0.59	-0.56	-0.50
-0.34	-0.33	-0.50
0.24	-0.45	-0.98
-1.04	-0.12	1.65
-1.05	0.15	0.92
-1.29	0.48	0.42
-1.05	-1.18	2.62
-0.41	0.18	-0.84
1.07	-0.61	-1.23
1.12	-0.64	-1.13
1.07	-0.80	0.08
1.73	-0.96	-0.63
1.03	-1.17	0.23
3.37	-0.96	-0.55
0.17	-0.77	1.34
-0.15	0.71	3.08
0.94	1.95	1.82
-0.23	-0.38	0.77
-1.41	-0.43	-1.16
2.16	0.05	-0.43
3.03	0.83	-0.36

-1.00	-0.56	0.56
-1.21	4.30	8.40
-1.40	17.41	8.63
-0.82	0.38	-0.21
1.96	0.44	0.21
-0.90	-0.63	-0.10
-1.61	4.19	4.35
-0.45	-0.17	0.48
-0.85	-0.17	0.83
-0.73	0.86	-0.35
-0.88	0.38	-0.23
-0.61	0.15	-0.68
-0.86	-0.23	0.00
-0.55	-0.07	-0.22
-0.73	0.26	0.06
-0.69	-0.67	-0.84
-0.73	-0.18	-0.50
-0.26	0.14	-0.89
0.10	-0.48	-1.33
-0.27	-0.37	0.37
-0.41	-0.48	0.33
-0.88	-0.95	0.60
0.80	-0.75	-1.31
-0.70	0.27	-0.58
-0.60	-0.45	-1.09
0.61	-0.18	-1.18
2.79	-0.12	-0.80
7.24	3.06	-0.90
3.69	-0.40	1.29
1.97	-0.07	1.37
1.63	-0.06	2.17
0.99	-0.02	0.09
0.07	-0.43	-1.43
1.55	-0.02	-1.05
-0.11	0.94	0.00
-1.57	21.70	15.37
-0.42	-0.23	-0.85
0.93	-0.92	-0.70
0.96	-1.28	-1.01
0.72	0.17	-0.64
-0.20	-0.41	-0.50
-0.61	0.25	-0.26
0.00	-0.18	-0.30
-1.62	0.57	-0.64
-0.35	0.14	-0.45
0.14	-0.75	-1.23
-0.72	-0.07	0.19
-0.75	0.02	-0.09
-0.30	0.32	-0.10
0.50	-0.67	-0.61
-0.47	-0.28	0.04
1.16	-0.98	-1.22
0.14	-0.90	-0.68
-0.69	0.30	1.11
0.58	-0.65	-1.08
3.33	1.98	-0.98
1.08	1.24	1.26
1.00	0.47	0.26

ave	43.20	33.67

	beta fits	
	NP	P
	K-S	K-S
	A-D	A-D
	alpha1	alpha1
	17.02	12.28
	alpha2	alpha2
	27.07	10.94
	a	a
	25.85	16.43
	b	b
	133.12	56.43
	p-val of ave of observed	
	0.00	0.17
	observed aves	
	43.20	33.67

18	19	20
TGAGGTAGTAGTTTGTACAGTT	TGAGGTAGTAGGTTGTGTGGTT	TGAGGTAGTAGATTGTATAGTT
let-7g-5p	let-7b-5p	let-7f-5p
1899491	1666971	1418336
0.24	0.85	0.04
0.15	0.28	0.19
0.51	0.68	0.30
0.54	0.65	0.14
0.73	0.55	0.29
0.40	0.39	0.21
0.60	0.40	0.09
0.12	0.55	0.03
0.18	0.40	0.13
0.30	0.43	0.26
0.20	0.23	0.27
0.34	0.42	0.24
0.79	0.31	1.07
0.14	0.74	0.04
0.30	0.41	0.25
0.43	0.22	0.51
0.20	0.58	0.03
0.26	0.51	0.23
0.20	0.19	0.19
0.41	0.30	0.56
0.51	0.35	0.19
0.39	0.46	0.07
0.41	0.59	0.09
0.26	0.79	0.04
0.27	0.53	0.09
0.37	0.44	0.06
0.42	0.35	0.31
0.64	0.30	1.02
0.38	0.17	0.44
0.16	0.24	0.20
0.13	0.66	0.06
0.31	0.26	0.52
0.57	0.46	0.57
0.16	0.49	0.06
0.13	0.67	0.07
0.54	0.27	0.09
0.06	0.83	0.02
0.48	0.27	0.26
0.48	0.28	0.47
0.34	0.29	0.16
0.22	0.73	0.03
0.31	1.11	0.04
0.30	0.36	0.06
0.44	0.19	0.26
0.09	0.25	0.08
0.13	0.72	0.02
0.37	0.18	0.21
0.31	0.30	0.13
0.21	0.33	0.16
0.26	0.30	0.08

0.28	0.25	0.18
0.35	0.36	0.05
0.29	0.56	0.07
0.58	0.34	0.22
0.34	0.36	0.15
0.19	0.49	0.04
0.49	0.36	0.18
0.88	0.26	0.78
0.61	0.49	0.09
0.88	0.45	0.40
0.86	0.37	0.32
0.69	0.29	0.71
0.22	0.67	0.04
0.50	0.50	0.12
0.63	0.50	0.25
0.65	0.51	0.75
0.27	0.42	0.18
0.17	0.29	0.23
1.27	1.61	0.39
0.87	0.72	0.34
0.47	0.46	0.14
0.79	0.10	1.30
0.54	0.50	0.35
0.44	0.62	0.52
0.07	1.88	0.01
0.74	0.34	0.59
0.25	0.20	0.20
0.15	0.30	0.33
0.48	0.34	0.35
0.35	0.24	0.28
0.11	0.50	0.05
0.68	0.33	0.29
0.11	0.25	0.07
0.44	0.24	0.68
0.69	0.26	1.09
0.14	0.26	0.08
0.49	0.29	0.21
0.39	0.35	0.10
0.41	0.21	0.54
0.34	0.29	0.12
1.63	0.36	1.52
0.32	0.22	0.09
0.42	0.51	0.08
0.41	0.29	0.24
0.57	0.44	0.10
0.00	0.00	0.00
0.00	0.00	0.00
0.00	0.00	0.00

19	20	21
D_4	*E_47*	*F_43*

0.18		
-0.08	-0.14	
-0.08	-0.38	0.00
-0.16	-0.37	0.13
-0.36	-0.41	0.21
-0.21	-0.30	-0.22
0.08	-0.24	-0.21
0.09	0.48	-0.15
-0.03	0.72	-0.08
0.52	0.13	0.11
0.46	0.07	0.24
-0.20	-0.28	0.24
0.36	0.09	0.11
-0.13	0.05	-0.25
0.15	-0.13	-0.19
0.22	-0.16	-0.25
0.05	-0.17	0.04
-0.37	-0.15	0.28
0.40	0.04	0.07
0.31	0.34	-0.17
0.38	0.00	-0.23
-0.03	-0.27	-0.10
-0.06	-0.23	-0.07
-0.09	-0.39	0.12
0.52	0.23	-0.01
0.28	-0.22	0.12
-0.15	-0.29	-0.04
0.40	0.30	-0.18
0.19	-0.17	0.06
0.07	0.27	-0.15
0.20	0.05	-0.20
0.13	0.16	-0.04
-0.12	-0.35	0.07
-0.22	-0.34	0.18
0.08	-0.05	0.03
-0.13	-0.22	0.61

-0.06	-0.28	0.09
-0.23	-0.19	0.23
-0.01	0.83	-0.10
-0.02	-0.36	0.00
0.03	-0.01	-0.03
-0.01	0.02	-0.20
-0.03	0.80	-0.07
-0.12	-0.02	-0.01
-0.29	0.01	0.07
0.00	0.39	-0.06
0.17	0.05	0.28
0.07	0.27	-0.04
0.26	-0.16	0.10
-0.44	-0.26	0.08
-0.02	-0.05	-0.11
-0.10	-0.14	0.35
0.27	0.33	0.16
-0.17	0.26	0.22
0.12	0.35	-0.23
-0.07	-0.26	-0.10
-0.06	0.24	0.09
0.79	0.15	-0.08
0.35	-0.09	0.21
0.20	0.40	-0.22
0.34	0.35	-0.15
-0.04	0.00	-0.20
0.14	0.13	0.08
-0.01	-0.10	-0.04
0.15	0.92	-0.18
-0.03	0.87	-0.05
0.27	0.11	-0.17
0.31	0.23	-0.16
-0.07	0.11	0.08
0.65	0.43	0.11
0.24	0.07	0.02
0.14	0.33	0.06
0.31	-0.09	0.59
-0.09	-0.30	0.10
0.31	0.25	0.07
0.09	-0.01	0.15

17	18	19
119	120	121
ACAGTAGTCTGCACATTGGTTA	TATTCATTTATCCCCAGCCTACA	ACTGCCCCAGGTGCTGCTGG
miR-199b-3p	miR-664a-3p	miR-324-3p
16186	15369	15277
-0.55	0.57	3.54
-0.68	-0.33	-1.28
-0.47	-0.35	-0.31
-0.58	-0.58	0.36
1.93	4.31	-0.93
-0.07	-0.83	-0.48
0.30	-0.68	0.27
-0.86	-0.16	-0.09
-0.96	-0.48	-0.44
-0.85	0.04	0.13
0.00	0.21	-0.69
-0.78	-0.78	-0.69
2.25	0.02	0.22
-1.02	0.37	-0.23
-0.64	0.00	-0.14
2.80	-0.01	0.06
-0.92	0.15	0.68
0.00	-0.47	2.45
-0.66	0.61	-0.56
0.90	-0.51	-0.33

-0.15	-0.84	-0.45
-0.20	-0.60	-0.12
0.98	-0.39	0.15
-0.17	-0.71	0.48
0.47	0.89	-1.18
-0.33	0.90	0.18
0.26	-0.34	-0.61
1.89	-0.48	-0.64
1.02	-0.49	-0.80
-0.86	-0.51	-0.96
-0.98	-0.80	-1.17
0.75	-0.48	-0.96
4.41	4.67	-0.77
-0.46	1.13	0.71
-0.52	2.86	1.95
-0.77	0.33	-0.38
-1.22	-1.03	-0.43
-0.34	0.00	0.05
0.64	2.22	0.83
-0.61	1.44	-0.56
0.13	-0.99	4.30
1.26	-1.00	17.41
-0.09	-0.36	0.38
2.03	0.22	0.44
-0.76	0.08	-0.63
-0.54	-1.02	4.19
0.93	-0.38	-0.17
0.16	-0.62	-0.17
0.44	-0.40	0.86
-0.38	-0.59	0.38
1.69	-0.53	0.15
0.17	-0.50	-0.23
-0.17	-0.51	-0.07
2.07	-0.14	0.26
0.18	-0.96	-0.67
-0.43	-0.77	-0.18
0.58	-0.74	0.14
1.95	-0.28	-0.48
-0.39	-0.22	-0.37
2.51	0.57	-0.48
2.77	1.88	-0.95
0.92	-0.91	-0.75
-0.93	-0.64	0.27
0.18	-0.39	-0.45
-0.36	-0.66	-0.18
-0.55	-0.53	-0.12
0.55	0.23	3.06
-0.84	0.15	-0.40
0.80	1.22	-0.07
1.87	0.67	-0.06
-0.45	-0.68	-0.02
2.16	-0.90	-0.43
-0.33	-0.34	-0.02
0.00	0.82	0.94
-1.12	-0.85	21.70
0.77	-0.63	-0.23
-0.63	0.14	-0.92
0.04	-0.72	-1.28
0.64	0.47	0.17

0.69	-0.29	-0.41
-1.01	-0.45	0.25
0.35	-0.54	-0.18
-0.33	-0.83	0.57
1.64	0.57	0.14
0.28	-0.98	-0.75
-0.06	-0.23	-0.07
0.29	-0.57	0.02
-0.51	-0.55	0.32
0.13	-0.59	-0.67
-0.16	-0.39	-0.28
0.02	-0.93	-0.98
-0.59	-0.94	-0.90
-0.18	-0.64	0.30
0.27	-0.50	-0.65
0.85	0.75	1.98
-1	-1	-1
0.00	0.00	0.00
1.00	1.00	1.00
0.21	0.22	0.88

119	120	121
ACAGTAGTCTGCACATTGGTTA	TATTCATTTATCCCCAGCCTACA	ACTGCCCCAGGTGCTGCTGG
miR-199b-3p	miR-664a-3p	miR-324-3p
16186	15369	15277
-0.55	0.57	3.54
-0.68	-0.33	-1.28
-0.47	-0.35	-0.31
-0.58	-0.58	0.36
1.93	4.31	-0.93
-0.07	-0.83	-0.48
0.30	-0.68	0.27
-0.86	-0.16	-0.09
-0.96	-0.48	-0.44
-0.85	0.04	0.13
0.00	0.21	-0.69
-0.78	-0.78	-0.69
2.25	0.02	0.22
-1.02	0.37	-0.23
-0.64	0.00	-0.14
2.80	-0.01	0.06
-0.92	0.15	0.68
0.00	-0.47	2.45
-0.66	0.61	-0.56
0.90	-0.51	-0.33
-0.15	-0.84	-0.45
-0.20	-0.60	-0.12
0.98	-0.39	0.15
-0.17	-0.71	0.48
0.47	0.89	-1.18
-0.33	0.90	0.18
0.26	-0.34	-0.61
1.89	-0.48	-0.64

1.02	-0.49	-0.80
-0.86	-0.51	-0.96
-0.98	-0.80	-1.17
0.75	-0.48	-0.96
4.41	4.67	-0.77
-0.46	1.13	0.71
-0.52	2.86	1.95
-0.77	0.33	-0.38
-1.22	-1.03	-0.43
-0.34	0.00	0.05
0.64	2.22	0.83
-0.61	1.44	-0.56
0.13	-0.99	4.30
1.26	-1.00	17.41
-0.09	-0.36	0.38
2.03	0.22	0.44
-0.76	0.08	-0.63
-0.54	-1.02	4.19
0.93	-0.38	-0.17
0.16	-0.62	-0.17
0.44	-0.40	0.86
-0.38	-0.59	0.38
1.69	-0.53	0.15
0.17	-0.50	-0.23
-0.17	-0.51	-0.07
2.07	-0.14	0.26
0.18	-0.96	-0.67
-0.43	-0.77	-0.18
0.58	-0.74	0.14
1.95	-0.28	-0.48
-0.39	-0.22	-0.37
2.51	0.57	-0.48
2.77	1.88	-0.95
0.92	-0.91	-0.75
-0.93	-0.64	0.27
0.18	-0.39	-0.45
-0.36	-0.66	-0.18
-0.55	-0.53	-0.12
0.55	0.23	3.06
-0.84	0.15	-0.40
0.80	1.22	-0.07
1.87	0.67	-0.06
-0.45	-0.68	-0.02
2.16	-0.90	-0.43
-0.33	-0.34	-0.02
0.00	0.82	0.94
-1.12	-0.85	21.70
0.77	-0.63	-0.23
-0.63	0.14	-0.92
0.04	-0.72	-1.28
0.64	0.47	0.17
0.69	-0.29	-0.41
-1.01	-0.45	0.25
0.35	-0.54	-0.18
-0.33	-0.83	0.57
1.64	0.57	0.14
0.28	-0.98	-0.75
-0.06	-0.23	-0.07
0.29	-0.57	0.02

-0.51	-0.55	0.32
0.13	-0.59	-0.67
-0.16	-0.39	-0.28
0.02	-0.93	-0.98
-0.59	-0.94	-0.90
-0.18	-0.64	0.30
0.27	-0.50	-0.65
1	1	2

119	120	121
57	71	52
AATGACACGATCACTCCCGTTGA	CTATACGACCTGCTGCCTTTCT	ATCACATTGCCAGGGATTTCC
miR-425-5p	let-7d-3p	miR-23a-3p
127210	71988	151875
-0.26	2.00	-0.46
0.03	-0.96	-0.31
1.92	-1.06	-0.42
-0.63	-1.08	-0.72
2.63	1.76	3.79
-0.62	2.96	-0.43
-1.13	1.52	-0.49
-0.89	0.51	-0.64
0.01	-0.69	-0.57
-0.20	-0.60	-0.39
0.29	-0.34	0.41
-1.51	-0.40	-0.66
0.92	-0.14	2.51
-1.00	-0.45	-0.67
1.11	-0.39	-0.02
1.17	0.00	0.47
0.70	0.45	-0.19
0.16	-0.47	0.09
-0.43	-0.61	0.37
0.05	-0.72	0.06
-1.06	-0.43	-0.53
-1.06	0.11	-0.42
-0.72	0.04	-0.47
-1.10	-0.19	-0.67
0.54	0.40	0.38
0.82	-0.16	0.35
0.23	-1.04	-0.36
0.53	-1.28	-0.27
-0.31	-0.73	0.10
-1.03	-0.94	-0.39
-0.97	-0.76	-0.88
-0.42	-0.98	-0.17
4.83	0.55	4.70
0.16	1.63	-0.22
1.49	0.28	1.20
-0.51	0.33	-0.23
-1.51	-0.01	-0.95
-0.80	0.55	0.70
1.79	-0.02	1.83

-0.28	-0.33	0.46
0.22	1.22	0.03
3.05	0.55	0.23
0.14	0.23	-0.33
1.17	-0.07	1.56
-0.77	0.24	-0.40
-0.98	1.15	-0.73
1.83	-0.23	0.97
-0.49	0.47	0.02
0.75	0.02	-0.17
0.57	-0.31	-0.19
0.13	-0.21	-0.14
-0.04	0.31	-0.22
-0.40	0.12	-0.22
1.67	0.24	1.39
-0.86	0.32	-0.61
0.15	-0.19	-0.42
-0.17	-0.38	-0.41
0.07	-1.23	-0.33
0.25	-0.33	-0.19
1.94	0.48	0.86
2.73	0.37	1.11
0.27	-1.31	-0.52
0.01	-0.53	-0.72
-0.09	-0.25	-0.61
0.87	-1.02	-0.46
-1.72	-1.12	-0.68
0.94	-0.55	-0.34
0.10	0.29	0.10
0.88	0.05	-0.20
1.67	-0.18	0.60
-1.09	-0.70	-0.70
0.47	0.94	-0.61
-0.34	-0.75	-0.23
0.28	-0.44	0.58
-0.39	3.01	0.02
-0.40	-0.39	-0.27
-0.34	-0.71	0.40
-1.32	0.03	-0.42
1.33	-0.95	0.95
1.98	-0.64	0.94
1.68	-0.36	-0.42
0.21	-0.72	0.55
-0.99	2.09	-0.38
-0.29	-0.63	0.88
-0.40	-1.22	-0.41
0.59	0.02	0.08
0.09	-0.36	0.32
0.21	-0.56	-0.22
-0.41	-0.64	0.19
0.42	-0.36	0.29
-1.21	-1.24	-0.28
-1.52	-0.50	-0.57
-0.48	0.04	-0.44
-0.23	-0.91	-0.21
-0.39	0.11	-0.28
1.32	1.34	1.41
0.80	0.70	1.00

18.85

UC
K-S

A-D

alpha1
48.20
alpha2
8.30
a
33.20
b
95.54

#NUM!

18.85

Student
pseudo data
trials
1000
100
10

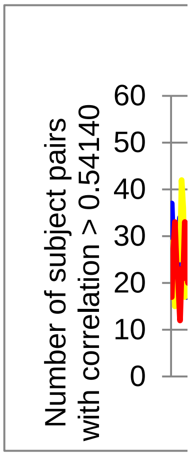

21	22	23
AGCTCGGTCTGAGGCCCCTCAGT	TTCAAGTAATTCAGGATAGGT	AGCAGCATTGTACAGGGCTATGA
miR-423-3p	miR-26b-5p	miR-103a-3p
1093922	1034642	1030775
0.30	0.03	0.21
0.02	0.13	0.26
0.10	0.17	0.29
0.15	0.11	0.18
0.05	0.40	0.35
0.65	0.10	0.10
0.84	0.11	0.19
0.38	0.03	0.10
0.17	0.06	0.11
0.12	0.10	0.15
0.17	0.16	0.13
0.66	0.05	0.14
0.22	0.70	0.33
0.34	0.03	0.12
0.20	0.16	0.37
0.37	0.29	0.32
0.28	0.03	0.17
0.30	0.12	0.50
0.06	0.25	0.30
0.27	0.47	0.29
0.28	0.11	0.20
0.69	0.04	0.21
0.53	0.04	0.23
0.63	0.02	0.16
0.05	0.10	0.10
0.09	0.13	0.14
0.07	0.32	0.13
0.09	0.43	0.22
0.11	0.27	0.20
0.04	0.11	0.23
0.03	0.03	0.21
0.02	0.28	0.34
0.03	0.70	0.33
0.56	0.08	0.14
0.15	0.05	0.24
0.43	0.11	0.14
0.46	0.00	0.03
0.10	0.15	0.25
0.09	0.36	0.31
0.20	0.15	0.19
0.41	0.04	0.26
0.86	0.03	0.48
0.42	0.05	0.19
0.22	0.28	0.37
0.22	0.05	0.18
0.60	0.02	0.11
0.25	0.29	0.22
0.52	0.09	0.25
0.43	0.14	0.31
0.43	0.10	0.23

0.27	0.19	0.32
0.37	0.07	0.20
0.25	0.06	0.19
0.40	0.20	0.37
0.43	0.07	0.15
0.18	0.05	0.15
0.27	0.12	0.20
0.07	0.68	0.15
0.16	0.10	0.13
0.08	0.38	0.29
0.05	0.40	0.26
0.07	0.55	0.16
0.19	0.05	0.07
0.25	0.06	0.08
0.09	0.21	0.11
0.38	0.08	0.39
0.07	0.18	0.18
0.01	0.11	0.42
0.12	0.23	0.57
0.14	0.45	0.60
0.27	0.12	0.23
0.03	0.23	0.12
0.21	0.17	0.16
0.25	0.32	0.32
0.76	0.01	0.23
0.41	0.28	0.27
0.03	0.19	0.30
0.06	0.09	0.20
0.11	0.49	0.49
0.18	0.31	0.22
0.22	0.05	0.13
0.21	0.32	0.20
0.73	0.04	0.16
0.23	0.44	0.35
0.10	0.64	0.26
0.29	0.10	0.22
0.30	0.33	0.26
0.30	0.14	0.18
0.12	0.44	0.39
0.26	0.15	0.23
0.22	0.40	0.32
0.16	0.11	0.18
0.55	0.06	0.18
0.07	0.23	0.13
0.10	0.15	0.14
0.00	0.00	0.00
0.00	0.00	0.00
0.00	0.00	0.00

22	23	24
F_6	*F_7*	*F_8*

0.78		
0.56	0.74	
0.13	0.07	0.01
0.19	0.14	0.04
-0.42	-0.47	-0.54
-0.46	-0.46	-0.40
-0.21	-0.32	-0.46
-0.42	-0.49	-0.40
-0.13	-0.12	0.12
-0.33	-0.40	-0.46
-0.36	-0.37	-0.42
0.27	0.21	0.22
-0.05	-0.04	0.06
0.51	0.36	0.24
0.09	0.10	0.47
-0.11	-0.19	-0.30
-0.38	-0.41	-0.47
0.34	0.26	0.03
0.26	0.28	0.39
0.20	0.23	0.34
0.42	0.46	0.39
-0.33	-0.27	-0.40
0.42	0.31	0.31
0.25	0.32	0.55
-0.06	-0.09	-0.41
0.66	0.61	0.34
-0.04	0.10	-0.04
0.35	0.34	0.13
0.14	0.10	-0.15
0.40	0.28	0.19
0.26	0.21	0.21
0.12	0.22	-0.07
0.43	0.44	0.41

0.32	0.24	0.21
0.19	0.01	-0.03
-0.43	-0.41	-0.37
0.57	0.50	0.35
0.01	0.10	-0.17
-0.11	-0.09	-0.26
-0.49	-0.49	-0.43
0.10	0.15	0.28
0.11	0.12	0.07
-0.39	-0.30	-0.40
0.09	0.10	0.09
-0.57	-0.56	-0.47
-0.26	-0.28	-0.27
0.03	0.20	0.24
0.06	0.09	-0.15
0.23	0.12	0.16
-0.22	-0.29	-0.39
-0.45	-0.43	-0.33
-0.24	-0.31	-0.31
0.18	0.23	0.38
0.02	-0.06	-0.18
-0.26	-0.35	-0.46
0.11	-0.10	-0.17
-0.36	-0.34	-0.47
-0.26	-0.22	-0.52
-0.03	0.11	0.06
-0.08	-0.11	-0.35
0.44	0.41	0.39
-0.35	-0.38	-0.38
-0.41	-0.42	-0.33
0.15	0.17	-0.07
0.17	0.14	-0.20
-0.08	-0.03	-0.09
-0.20	-0.24	-0.47
0.10	0.11	-0.16
-0.19	-0.20	-0.33
0.21	0.15	0.11
0.83	0.77	0.55
-0.30	-0.30	-0.45
-0.24	-0.23	-0.22

20	21	22
122	123	124
TAACAGTCTACAGCCATGGTCG	AATATAACACAGATGGCCTGT	AAAGTTCTGAGACACTCCGACT
miR-132-3p	miR-410	miR-148a-5p
15261	14130	13673
0.91	0.77	-0.12
-2.06	-0.96	-0.50
-0.75	0.09	0.06
0.24	-0.66	-0.29
-0.87	-0.96	-0.34
0.62	-0.67	-0.69
1.64	-0.07	-0.70
0.91	-0.71	-0.69
-0.97	-0.04	-0.55
0.27	-0.68	-0.82
-0.59	-0.08	0.11
0.78	-0.85	-0.40
1.88	-0.71	2.24
1.28	-0.50	-0.41
-0.47	0.58	0.05
0.46	0.61	0.89
0.45	0.27	-0.01
0.38	2.22	-0.97
0.22	0.14	3.48
-0.57	-0.37	1.35

0.09	-0.82	-0.66
0.35	1.00	0.65
-0.24	-0.05	0.22
0.31	-0.49	-0.49
-2.06	-0.77	-0.73
-0.72	0.29	-0.65
-1.52	3.42	-0.04
-1.24	1.28	0.44
-0.10	0.84	1.50
-0.31	-0.09	0.91
-0.99	-0.54	-0.69
-0.63	0.10	2.19
-1.63	-0.79	-0.47
1.49	-0.09	-0.25
1.57	-0.62	-0.22
1.30	-0.81	-0.20
-0.96	-0.84	-1.10
-0.46	1.01	0.74
0.79	0.14	1.94
0.57	-0.47	0.65
-1.57	-0.81	-0.58
-1.17	-0.96	0.34
0.33	-0.39	-0.56
0.76	-0.53	1.53
1.00	-0.12	0.69
-1.55	-0.72	-1.02
-0.04	1.33	0.67
1.09	1.35	0.98
1.03	-0.51	0.35
1.08	1.64	0.24
1.43	3.74	0.15
-0.70	0.80	-0.53
0.75	-0.06	-0.63
1.32	-0.22	0.56
1.58	-0.55	-0.46
0.34	0.11	-0.21
0.08	1.44	-0.16
-1.54	-0.43	-0.56
-0.77	0.13	-0.42
-1.22	-0.75	0.13
-1.15	-0.32	-0.29
-1.83	1.32	-0.18
-0.97	-0.81	-0.58
-1.33	0.04	-0.77
-1.15	-0.70	-0.51
1.76	-0.44	1.76
-0.12	0.15	-0.11
0.18	-0.52	1.52
-0.11	-0.74	-0.71
1.86	-0.06	0.51
1.47	-0.18	-0.15
0.02	-0.33	0.88
0.53	-0.57	-0.54
2.38	0.24	-0.02
-1.60	-0.96	-0.54
0.37	1.14	0.07
-0.16	-0.11	2.29
-0.82	2.79	0.71
-0.38	1.44	0.19

-1.13	-0.08	0.65
-0.69	-0.85	-0.26
-0.34	0.52	-0.08
1.80	-0.08	0.22
-0.61	1.39	1.72
-1.40	1.01	0.06
-0.30	-0.64	0.29
0.43	0.39	0.26
0.01	-0.75	-0.17
0.19	-0.15	2.06
0.93	-0.67	-0.16
-1.32	1.10	2.43
0.88	-0.52	-0.29
0.24	0.92	-0.09
-1.12	-0.43	0.15
-0.76	-0.26	-0.39
1	-2	1
0.00	0.00	0.00
1.00	1.00	1.00
0.39	0.93	0.11

122	123	124
TAACAGTCTACAGCCATGGTCG	AATATAACACAGATGGCCTGT	AAAGTTCTGAGACACTCCGACT
miR-132-3p	miR-410	miR-148a-5p
15261	14130	13673
0.91	0.77	-0.12
-2.06	-0.96	-0.50
-0.75	0.09	0.06
0.24	-0.66	-0.29
-0.87	-0.96	-0.34
0.62	-0.67	-0.69
1.64	-0.07	-0.70
0.91	-0.71	-0.69
-0.97	-0.04	-0.55
0.27	-0.68	-0.82
-0.59	-0.08	0.11
0.78	-0.85	-0.40
1.88	-0.71	2.24
1.28	-0.50	-0.41
-0.47	0.58	0.05
0.46	0.61	0.89
0.45	0.27	-0.01
0.38	2.22	-0.97
0.22	0.14	3.48
-0.57	-0.37	1.35
0.09	-0.82	-0.66
0.35	1.00	0.65
-0.24	-0.05	0.22
0.31	-0.49	-0.49
-2.06	-0.77	-0.73
-0.72	0.29	-0.65
-1.52	3.42	-0.04
-1.24	1.28	0.44

-0.10	0.84	1.50
-0.31	-0.09	0.91
-0.99	-0.54	-0.69
-0.63	0.10	2.19
-1.63	-0.79	-0.47
1.49	-0.09	-0.25
1.57	-0.62	-0.22
1.30	-0.81	-0.20
-0.96	-0.84	-1.10
-0.46	1.01	0.74
0.79	0.14	1.94
0.57	-0.47	0.65
-1.57	-0.81	-0.58
-1.17	-0.96	0.34
0.33	-0.39	-0.56
0.76	-0.53	1.53
1.00	-0.12	0.69
-1.55	-0.72	-1.02
-0.04	1.33	0.67
1.09	1.35	0.98
1.03	-0.51	0.35
1.08	1.64	0.24
1.43	3.74	0.15
-0.70	0.80	-0.53
0.75	-0.06	-0.63
1.32	-0.22	0.56
1.58	-0.55	-0.46
0.34	0.11	-0.21
0.08	1.44	-0.16
-1.54	-0.43	-0.56
-0.77	0.13	-0.42
-1.22	-0.75	0.13
-1.15	-0.32	-0.29
-1.83	1.32	-0.18
-0.97	-0.81	-0.58
-1.33	0.04	-0.77
-1.15	-0.70	-0.51
1.76	-0.44	1.76
-0.12	0.15	-0.11
0.18	-0.52	1.52
-0.11	-0.74	-0.71
1.86	-0.06	0.51
1.47	-0.18	-0.15
0.02	-0.33	0.88
0.53	-0.57	-0.54
2.38	0.24	-0.02
-1.60	-0.96	-0.54
0.37	1.14	0.07
-0.16	-0.11	2.29
-0.82	2.79	0.71
-0.38	1.44	0.19
-1.13	-0.08	0.65
-0.69	-0.85	-0.26
-0.34	0.52	-0.08
1.80	-0.08	0.22
-0.61	1.39	1.72
-1.40	1.01	0.06
-0.30	-0.64	0.29
0.43	0.39	0.26

0.01	-0.75	-0.17
0.19	-0.15	2.06
0.93	-0.67	-0.16
-1.32	1.10	2.43
0.88	-0.52	-0.29
0.24	0.92	-0.09
-1.12	-0.43	0.15
-1	0	0

122	123	124
36	136	55
TAGCAGCACATCATGGTTTACA	AACATTCATTGTTGTCGGTGGGT	CAACACCAGTCGATGGGCTGT
miR-15b-5p	miR-181d	miR-21-3p
436288	10591	137573
-0.31	-0.30	0.08
0.05	-2.01	-1.38
0.45	-1.01	-0.98
-0.12	-1.41	-0.29
4.79	0.11	-1.36
-0.37	1.20	0.30
-0.45	2.09	0.40
-0.20	-0.45	-1.03
-0.18	-1.03	-0.83
0.44	0.06	-0.82
-0.43	0.61	0.16
-0.72	-0.61	-0.39
-0.30	1.09	0.73
-0.14	-0.26	-0.89
-0.12	0.37	0.25
-0.34	1.71	0.74
0.06	-0.03	2.00
-0.04	-0.15	-0.26
-0.26	0.60	0.37
-0.52	-0.52	1.61
-0.52	0.10	-0.83
-0.48	0.32	0.55
-0.27	0.17	0.90
-0.19	-0.18	0.46
0.47	-1.77	-1.37
-0.03	1.62	-0.46
-0.27	-0.34	2.35
-0.56	-0.93	0.80
-0.57	-0.84	1.52
-0.34	-0.68	-1.07
0.53	-1.24	-1.37
-0.24	-0.22	-1.37
4.82	-0.78	-1.34
0.13	0.36	1.20
1.63	1.97	-0.49
-0.44	0.06	-0.04
-0.40	-1.54	-1.18
-0.40	1.42	0.41
-0.17	3.90	-0.19

-0.38	0.55	-0.31
-0.27	0.53	3.05
-0.68	7.55	5.66
-0.18	1.04	0.15
0.02	1.99	1.96
-0.60	-0.13	-0.83
-0.40	-0.09	2.24
-0.28	1.16	1.52
-0.39	0.47	1.39
-0.31	-0.52	-0.50
-0.46	0.01	1.24
-0.46	-0.03	-0.07
-0.34	0.94	0.61
0.03	-0.12	-0.17
-0.09	1.11	-0.09
-0.61	-0.29	-0.39
-0.01	-0.41	0.16
-0.31	-0.11	-0.39
-0.56	0.22	1.09
-0.19	0.17	-1.19
0.11	0.76	-1.32
0.83	0.10	-1.36
-0.36	-0.88	0.74
0.21	-0.99	-0.17
-0.30	-1.26	1.03
-0.25	-0.81	1.13
-0.55	1.10	-0.44
-0.20	-1.15	-0.44
0.56	-2.01	-1.38
2.96	-0.96	-1.36
0.29	0.96	-1.30
-0.33	-0.43	-0.49
-0.65	-1.87	-1.00
-0.22	-0.68	-0.77
-0.30	-0.02	-0.90
-0.72	2.86	-0.43
-0.54	0.80	-0.25
-0.30	0.99	-0.60
-0.63	-0.24	-0.99
-0.12	0.97	2.80
-0.34	0.70	1.46
0.13	-0.37	-0.38
-0.24	0.20	-0.89
-0.65	-0.06	0.01
-0.52	0.33	1.27
-0.52	-1.15	2.29
-0.47	0.29	0.03
-0.43	0.51	1.07
-0.08	-0.55	-0.17
-0.53	0.42	0.79
-0.26	0.01	-0.66
-0.56	0.57	0.03
-0.60	0.00	-0.46
-0.26	0.19	1.95
-0.27	0.01	0.30
0.01	2.46	0.51
1.50	1.61	1.69
0.85	0.69	1.00

relabeling+reselection of 29s

Extended trials start at MA1.

PvUC ttest	NPvP ttest	NPvUC ttest
relabeling+reselection of 29s		
PvUC ttest	NPvP ttest	NPvUC ttest
3.80E-02	5.54E-01	1.37E-01
5.23E-01	5.53E-01	9.65E-01
2.38E-01	1.64E-01	6.95E-01

random relabeling, rand

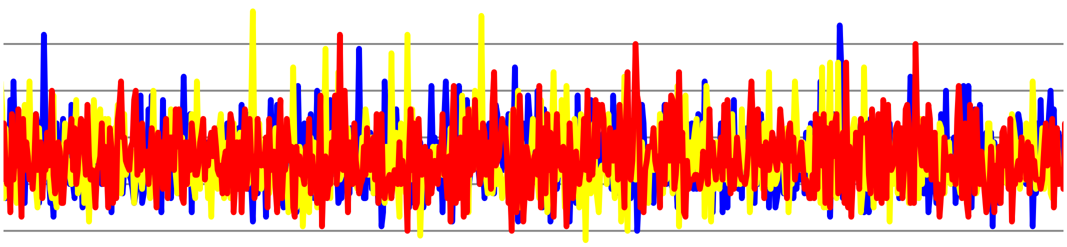

24	25	26
CAAAGAATTCTCCTTTTGGGCT	CACTAGATTGTGAGCTCCTGGA	ACTGGACTTGGAGTCAGAAGG
miR-186-5p	miR-28-3p	miR-378a-3p
979581	903691	738230
0.19	0.27	0.18
0.18	0.55	0.06
0.26	0.20	0.12
0.11	0.15	0.12
0.13	0.17	0.20
0.07	0.25	0.12
0.06	0.25	0.20
0.08	0.13	0.14
0.16	0.14	0.10
0.24	0.21	0.12
0.34	0.34	0.11
0.09	0.15	0.15
0.47	0.20	0.29
0.08	0.13	0.13
0.23	0.21	0.24
0.39	0.23	0.14
0.15	0.23	0.27
0.13	0.16	0.22
0.27	0.52	0.21
0.30	0.19	0.18
0.09	0.23	0.12
0.10	0.25	0.22
0.11	0.23	0.23
0.06	0.16	0.15
0.08	0.24	0.06
0.18	0.23	0.11
0.35	0.19	0.07
0.44	0.16	0.07
0.33	0.87	0.12
0.17	0.55	0.13
0.07	0.32	0.10
0.33	1.01	0.14
0.22	0.10	0.15
0.11	0.25	0.17
0.14	0.17	0.17
0.17	0.27	0.22
0.02	0.04	0.04
0.20	0.36	0.22
0.44	0.30	0.26
0.21	0.24	0.26
0.03	0.31	0.29
0.02	0.31	0.72
0.12	0.23	0.10
0.33	0.39	0.20
0.12	0.29	0.14
0.02	0.19	0.11
0.36	0.26	0.19
0.13	0.32	0.21
0.23	0.13	0.12
0.17	0.20	0.23

0.23	0.18	0.15
0.10	0.25	0.17
0.07	0.16	0.13
0.21	0.20	0.20
0.09	0.19	0.11
0.10	0.24	0.19
0.15	0.14	0.12
0.53	0.16	0.10
0.21	0.33	0.17
0.31	0.30	0.16
0.30	0.24	0.12
0.59	0.15	0.08
0.10	0.13	0.10
0.19	0.15	0.10
0.25	0.15	0.09
0.11	0.21	0.14
0.42	0.18	0.11
0.15	0.94	0.18
0.11	0.12	0.14
0.30	0.24	0.21
0.12	0.20	0.10
0.21	0.37	0.13
0.19	0.12	0.16
0.30	0.16	0.20
0.02	0.16	0.52
0.22	0.15	0.16
0.20	0.48	0.23
0.16	0.55	0.13
0.30	0.16	0.26
0.31	0.25	0.21
0.12	0.17	0.10
0.20	0.25	0.18
0.10	0.16	0.13
0.33	0.22	0.23
0.28	0.11	0.16
0.23	0.22	0.15
0.21	0.22	0.18
0.15	0.16	0.11
0.32	0.32	0.28
0.19	0.20	0.16
0.20	0.14	0.21
0.09	0.24	0.19
0.12	0.21	0.25
0.25	0.27	0.09
0.47	0.24	0.10
0.00	0.00	0.00
0.00	0.00	0.00
0.00	0.00	0.00

25	26	27
G_33	*G_34*	*G_8*

0.22		
-0.19	-0.10	
-0.17	-0.25	0.59
-0.07	-0.06	0.25
-0.13	-0.28	0.23
0.27	-0.23	-0.05
0.04	-0.14	0.33
0.43	0.04	0.10
-0.01	-0.04	-0.33
0.01	0.16	-0.23
-0.06	0.27	-0.45
0.03	-0.33	-0.22
-0.38	0.06	0.10
-0.33	0.05	0.30
-0.11	0.37	-0.36
0.09	0.21	-0.30
0.03	0.23	-0.24
0.12	0.41	-0.31
-0.32	-0.07	0.26
0.05	0.02	-0.41
0.11	0.16	-0.35
-0.09	0.27	0.21
-0.11	0.18	-0.33
-0.30	-0.16	0.00
-0.31	0.23	-0.18
-0.30	0.11	0.20
0.20	0.32	-0.09
0.25	0.06	-0.04
-0.07	0.30	-0.10
-0.03	-0.16	-0.35

0.21	0.24	-0.08
0.02	0.04	0.18
-0.20	-0.14	0.57
0.27	0.64	-0.38
0.22	0.45	-0.06
0.30	0.46	-0.03
-0.20	-0.22	0.70
0.08	0.12	0.03
0.07	0.20	0.29
-0.14	-0.05	0.59
-0.30	-0.27	-0.06
-0.32	-0.20	0.53
0.20	-0.09	-0.01
0.19	-0.07	-0.19
-0.02	0.09	-0.10
-0.23	-0.27	-0.12
-0.22	-0.28	0.19
-0.35	-0.42	0.32
-0.33	-0.13	0.14
0.10	0.20	-0.28
-0.36	-0.08	0.26
-0.11	0.09	0.15
0.07	-0.13	0.12
-0.26	0.07	0.54
-0.10	0.17	0.41
0.20	0.16	0.00
0.00	0.34	0.14
-0.18	0.10	-0.36
-0.28	-0.13	0.42
-0.19	-0.25	0.50
-0.13	0.22	-0.09
-0.24	0.32	0.05
-0.13	0.13	-0.01
-0.42	-0.20	0.30
-0.05	0.28	-0.13
-0.14	-0.18	0.37
-0.18	-0.10	-0.14
0.05	0.19	-0.34
-0.03	0.09	0.57
-0.21	0.27	0.38

23	24	25
125	126	127
TGTAGTGTTTCCTACTTTATGGA	ACTCTTTCCCTGTTGCACTAC	CTGCCCTGGCCCGAGGGACCGA
miR-142-3p	miR-130b-5p	miR-874
13338	12970	12358
-0.37	0.32	0.68
-0.39	-0.98	-1.39
-0.34	-0.91	-0.16
-0.35	-0.74	-0.78
-0.22	2.95	1.98
-0.33	2.28	-0.77
-0.29	0.25	-0.91
-0.34	0.67	-0.85
-0.37	-0.85	-0.79
-0.34	-0.46	0.19
-0.33	-1.02	0.94
-0.32	0.63	-0.80
1.68	0.71	-0.77
-0.37	-0.38	-0.77
-0.23	-0.87	-0.44
0.41	0.33	0.01
-0.30	-0.35	1.84
-0.30	-0.63	0.33
-0.27	-0.86	-0.36
4.54	-0.56	-0.49

-0.27	-0.09	-0.30
-0.35	0.59	-0.16
-0.27	1.14	0.00
-0.30	0.00	0.26
-0.39	0.67	1.29
0.04	-0.92	2.72
0.34	-0.89	-0.50
4.34	-1.01	-0.59
-0.23	-0.43	-0.11
-0.36	-1.36	-0.55
-0.39	-0.91	-0.96
-0.39	-1.03	-0.52
-0.39	-0.14	-0.69
-0.24	-0.47	-1.11
-0.27	-0.51	-1.04
-0.05	-0.47	0.13
-0.34	-0.88	-1.09
-0.15	-0.06	-0.85
0.22	-0.59	0.08
-0.23	-0.26	0.20
0.27	0.28	7.67
1.00	3.76	54.32
-0.18	0.09	1.01
0.29	-0.16	-0.42
-0.20	-0.12	-0.49
-0.24	0.05	7.11
0.02	-0.74	0.78
-0.18	0.28	-0.31
-0.18	0.20	-0.56
-0.20	0.02	0.22
-0.06	-0.70	-0.34
-0.24	0.05	0.91
-0.32	0.25	-0.39
-0.06	0.75	0.68
-0.33	1.40	-0.71
-0.29	-0.63	1.78
-0.25	-0.12	0.11
11.42	-1.02	-0.37
-0.30	-0.35	0.49
-0.15	1.20	0.33
-0.19	0.50	0.96
5.97	-1.07	-0.66
-0.23	-0.71	0.10
-0.18	0.21	0.27
-0.24	-1.06	-0.01
-0.37	-1.13	-1.20
-0.34	-1.09	0.20
-0.39	-0.61	-0.18
-0.36	1.45	-0.47
-0.33	1.66	-0.17
-0.28	-0.12	-0.85
-0.25	-0.88	-0.88
-0.27	-0.39	-0.80
-0.07	-0.34	-0.95
-0.12	0.58	59.63
0.01	-0.26	-0.73
-0.29	-1.17	-0.26
-0.21	-0.90	-0.64
-0.04	-1.14	0.73

-0.20	-0.68	0.31
-0.34	-0.53	1.25
-0.17	-0.58	1.07
-0.18	0.51	-0.28
6.52	-0.62	-0.02
6.10	-1.18	-0.89
-0.18	-0.69	0.70
-0.22	-0.66	0.61
-0.22	-0.46	0.55
-0.16	-0.73	-0.80
-0.20	0.01	0.26
-0.25	-0.56	-0.45
-0.31	-0.60	-0.75
-0.14	0.77	0.21
-0.35	-1.06	-0.17
-0.39	-0.96	1.86
0	0	0
0.00	0.00	0.00
1.00	1.00	1.00
0.66	0.23	0.94

125	126	127
TGTAGTGTTTCCTACTTTATGGA	ACTCTTTCCCTGTTGCACTAC	CTGCCCTGGCCCGAGGGACCGA
miR-142-3p	miR-130b-5p	miR-874
13338	12970	12358
-0.37	0.32	0.68
-0.39	-0.98	-1.39
-0.34	-0.91	-0.16
-0.35	-0.74	-0.78
-0.22	2.95	1.98
-0.33	2.28	-0.77
-0.29	0.25	-0.91
-0.34	0.67	-0.85
-0.37	-0.85	-0.79
-0.34	-0.46	0.19
-0.33	-1.02	0.94
-0.32	0.63	-0.80
1.68	0.71	-0.77
-0.37	-0.38	-0.77
-0.23	-0.87	-0.44
0.41	0.33	0.01
-0.30	-0.35	1.84
-0.30	-0.63	0.33
-0.27	-0.86	-0.36
4.54	-0.56	-0.49
-0.27	-0.09	-0.30
-0.35	0.59	-0.16
-0.27	1.14	0.00
-0.30	0.00	0.26
-0.39	0.67	1.29
0.04	-0.92	2.72
0.34	-0.89	-0.50
4.34	-1.01	-0.59

-0.23	-0.43	-0.11
-0.36	-1.36	-0.55
-0.39	-0.91	-0.96
-0.39	-1.03	-0.52
-0.39	-0.14	-0.69
-0.24	-0.47	-1.11
-0.27	-0.51	-1.04
-0.05	-0.47	0.13
-0.34	-0.88	-1.09
-0.15	-0.06	-0.85
0.22	-0.59	0.08
-0.23	-0.26	0.20
0.27	0.28	7.67
1.00	3.76	54.32
-0.18	0.09	1.01
0.29	-0.16	-0.42
-0.20	-0.12	-0.49
-0.24	0.05	7.11
0.02	-0.74	0.78
-0.18	0.28	-0.31
-0.18	0.20	-0.56
-0.20	0.02	0.22
-0.06	-0.70	-0.34
-0.24	0.05	0.91
-0.32	0.25	-0.39
-0.06	0.75	0.68
-0.33	1.40	-0.71
-0.29	-0.63	1.78
-0.25	-0.12	0.11
11.42	-1.02	-0.37
-0.30	-0.35	0.49
-0.15	1.20	0.33
-0.19	0.50	0.96
5.97	-1.07	-0.66
-0.23	-0.71	0.10
-0.18	0.21	0.27
-0.24	-1.06	-0.01
-0.37	-1.13	-1.20
-0.34	-1.09	0.20
-0.39	-0.61	-0.18
-0.36	1.45	-0.47
-0.33	1.66	-0.17
-0.28	-0.12	-0.85
-0.25	-0.88	-0.88
-0.27	-0.39	-0.80
-0.07	-0.34	-0.95
-0.12	0.58	59.63
0.01	-0.26	-0.73
-0.29	-1.17	-0.26
-0.21	-0.90	-0.64
-0.04	-1.14	0.73
-0.20	-0.68	0.31
-0.34	-0.53	1.25
-0.17	-0.58	1.07
-0.18	0.51	-0.28
6.52	-0.62	-0.02
6.10	-1.18	-0.89
-0.18	-0.69	0.70
-0.22	-0.66	0.61

-0.22	-0.46	0.55
-0.16	-0.73	-0.80
-0.20	0.01	0.26
-0.25	-0.56	-0.45
-0.31	-0.60	-0.75
-0.14	0.77	0.21
-0.35	-1.06	-0.17
0	-1	2

125	126	127
120	16	89
TATTCATTTATCCCCAGCCTACA	AGCTCGGTCTGAGGCCCCTCAGT	TCACAGTGAACCGGTCTCTTT
miR-664a-3p	miR-423-3p	miR-128
15369	1093922	40544
0.57	0.03	0.18
-0.33	-1.22	0.71
-0.35	-0.87	-0.79
-0.58	-0.64	-0.96
4.31	-1.06	1.38
-0.83	1.52	1.46
-0.68	2.37	1.06
-0.16	0.36	-0.86
-0.48	-0.53	0.03
0.04	-0.77	0.05
0.21	-0.54	0.73
-0.78	1.58	-0.47
0.02	-0.34	2.44
0.37	0.20	-1.60
0.00	-0.42	-0.26
-0.01	0.34	0.86
0.15	-0.08	-1.35
-0.47	0.02	0.75
0.61	-1.01	-0.40
-0.51	-0.13	1.19
-0.84	-0.06	0.27
-0.60	1.71	-0.57
-0.39	1.01	-0.86
-0.71	1.46	-1.29
0.89	-1.06	-0.21
0.90	-0.90	-0.85
-0.34	-0.98	-0.65
-0.48	-0.88	-0.52
-0.49	-0.80	3.16
-0.51	-1.13	0.02
-0.80	-1.15	-0.98
-0.48	-1.23	0.49
4.67	-1.14	3.52
1.13	1.15	1.36
2.86	-0.63	-0.44
0.33	0.59	1.67
-1.03	0.71	-1.78
0.00	-0.86	-0.25
2.22	-0.90	0.02

1.44	-0.42	-0.08
-0.99	0.49	-1.41
-1.00	2.47	-0.04
-0.36	0.55	-1.18
0.22	-0.32	0.65
0.08	-0.34	-0.99
-1.02	1.32	-1.96
-0.38	-0.19	2.40
-0.62	0.98	1.10
-0.40	0.58	0.90
-0.59	0.57	0.96
-0.53	-0.12	1.05
-0.50	0.32	-0.14
-0.51	-0.20	-0.73
-0.14	0.43	1.81
-0.96	0.59	1.22
-0.77	-0.53	-0.88
-0.74	-0.12	0.34
-0.28	-0.98	-0.14
-0.22	-0.60	0.66
0.57	-0.93	2.90
1.88	-1.05	2.68
-0.91	-1.00	-0.29
-0.64	-0.46	-1.03
-0.39	-0.19	-0.86
-0.66	-0.88	-0.26
-0.53	0.38	-0.98
0.23	-0.97	1.01
0.15	-1.25	-0.60
1.22	-0.76	0.88
0.67	-0.70	3.09
-0.68	-0.12	-0.42
-0.90	-1.16	4.46
-0.34	-0.39	-0.07
0.82	-0.22	1.06
-0.85	2.03	-2.12
-0.63	0.50	1.03
0.14	-1.14	-0.87
-0.72	-1.03	-1.01
0.47	-0.80	0.18
-0.29	-0.51	0.63
-0.45	-0.34	-1.25
-0.54	-0.36	0.88
-0.83	1.88	-0.24
0.57	-0.29	1.18
-0.98	-0.87	0.04
-0.23	-0.04	0.97
-0.57	0.02	1.15
-0.55	0.00	0.39
-0.59	-0.77	1.95
-0.39	-0.15	1.45
-0.93	-0.34	0.21
-0.94	-0.57	-0.20
-0.64	1.13	-0.52
-0.50	-0.98	-0.59
0.75	-0.86	-0.09
1.73	1.78	1.80
0.43	1.00	1.00

1000 relabeling+reselect.
UC
37
26
25
19
25
34
32
26
30
17

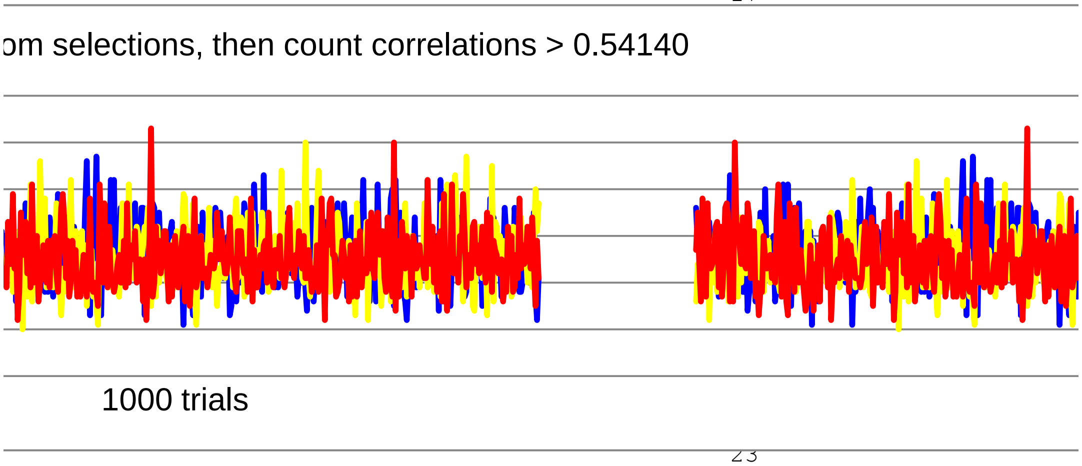

23
27
32
27
35
33
16
23
26
52
34
27
23
16
19
13
23
33
22
25
23
34
27
27
21
20
37

20
17
22
34
21
22
15
27
22
27
28
23
24
19
24
26
25
24
20
23
21
26
27
29
14
30
21
24
29
27
22
18
23
23
26
22
21
19
16
18
20
29
39
16
18
28
22
36
22
39
24
30
31
23
21
14
38
22
24

29
29
17
24
26
24
25
18
29
29
43
25
25
25
35
14
32
34
22
29
22
23
23
24
23
29
23
26
23
26
21
32
26
20
22
32
21
33
22
22
20
23
28
26
32
23
37
19
22
32
19
35
20
12
28
32
18
22
27

28
26
13
24
27
38
24
26
25
37
24
16
31
15
26
20
24
23
23
25
22
14
41
26
29
30
33
22
17
25
35
22
17
26
40
22
17
22
18
25
35
23
38
22
29
24
26
16
17
17
35
22
29
31
18
32
26
26
24

28
49
24
22
17
17
29
22
31
19
30
28
22
16
32
11
14
42
30
26
31
21
28
29
36
29
24
28
26
20
29
15
15
23
27
16
20
22
19
29
17
15
25
23
25
28
41
25
30
23
29
19
24
14
34
42
29
25
32

12
25
23
21
41
22
20
38
26
38
19
22
30
24
32
33
24
20
26
24
17
31
32
33
32
24
18
37
35
27
26
25
23
31
31
35
28
26
29
45
23
12
28
17
33
19
25
39
34
32
26
22
22
40
20
21
15
36
28

23
33
12
24
16
27
19
23
26
26
23
32
20
23
12
24
23
22
16
39
21
20
24
25
31
20
32
38
35
25
29
28
18
27
30
31
23
23
31
22
19
39
25
18
30
21
26
29
34
22
23
29
17
20
25
29
10
17
32

37
33
23
25
23
20
20
16
27
27
22
33
25
23
37
20
26
29
20
19
25
22
15
20
14
23
26
26
33
31
27
33
19
27
19
35
27
29
21
42
25
25
26
23
14
26
23
24
26
33
14
20
28
15
28
31
22
38
22

21
19
30
17
28
21
32
28
27
27
27
16
28
20
27
35
28
23
29
27
15
30
23
27
15
17
20
28
25
18
26
28
21
28
27
17
24
19
28
21
21
27
18
31
20
24
33
21
31
24
30
21
42
19
27
29
28
25
13

30
35
25
28
23
54
45
17
27
17
25
24
30
22
22
23
27
30
33
34
14
26
18
14
34
20
19
17
20
28
30
28
25
26
26
21
33
27
22
27
20
28
17
29
30
30
26
20
27
43
27
34
17
21
30
22
33
18

18
30
14
18
20
25
21
16
23
22
35
27
22
40
23
27
23
28
30
16
20
22
30
18
41
21
25
41
19
15
30
36
34
34
37
21
21
26
31
24
28
31
11
29
25
24
16
28
23
27
24
28
20
23
32
23
22
33
35

33
24
26
26
20
30
29
30
11
23
18
33
23
38
24
25
24
28
35
40
28
36
22
28
31
23
30
24
32
24
25
25
30
16
24
31
19
25
31
37
29
25
28
22
26
20
19
30
31
28
20
18
29
18
34
31
17
24
28

39
27
17
37
22
27
34
23
20
23
32
23
25
27
29
25
23
36
46
26
13
35
25
28
47
29
22
13
34
21
29
28
21
42
37
42
19
29
29
36
26
31
36
21
20
29
28
33
37
22
24
23
36
36
13
34
31
30
17

37
36
21
27
35
24
25
23
25
22
24
31
33
22
24
28
28
21
27
11
24
22
17
19
17
13
23
31
32
18
17
35
29
27
27
28
32
25
28
36
25
25
35
28
21
21
29
30
13
15
31
24
16
23
24
20
23
37
34

19
21
21
23
41
28
32
28
32
18
43
21
24
26
22
23
20
19
24
19
22
23
28
21
23
35
35
22
23
30
25
17
21
22
20
31
19
14
29
35
36
16
22
23
22
29
29
29
33
20
26
29
26
28
22
34
37
20
28

27
37
29
17
30
28
34
31
34
23
30
23
31
42
22
22
23
16
25
26
33
16
41
24
30
28
26
21
21
27
38
40
15
42
26
19
35
22
18
16
12
21
24
18
23
34
19
21
27
26
21
25
28
25
32
25
21
28
25

30
14
42
25
16
31
21
26
15
24
38
26
26
34
21
31
23
17
18
31
25
20
29
18
26
26
24
29
15
22
25
32
24
38
19
34
23
22
27
26
17
23
36
30
22
18
27
21
36
31
24
18
18
20
29
23
27
20
29

20
35
18
12
21

36
28
30
28
25
26
26
21
33
27
22
27
20
28
17
29

30
30
26
20
27
43
27
34
17
21
30
22
33
18
18
30
14
18
20
25
21
16
23
22
35
27
22
40
23
27
23
28
30
16
20
22
30
18
41
21
25
41
19
15
30
36
34
34
37
21
21
26
31
24
28
31
11
29
25

24
16
28
23
27
24
28
20
23
32
23
22
33
35
33
24
26
26
20
30
29
30
11
23
18
33
23
38
24
25
24
28
35
40
28
36
22
28
31
23
30
24
32
24
25
25
30
16
24
31
19
25
31
37
29
25
28
22
26

20
19
30
31
28
20
18
29
18
34
31
17
24
28
39
27
17
37
22
27
34
23
20
23
32
23
25
27
29
25
23
36
46
26
13
35
25
28
47
29
22
13
34
21
29
28
21
42
37
42
19
29
29
36
26
31
36
21
20

29
28
33
37
22
24
23
36
36
13
34
31
30
17
37
36
21
27
35
24
25
23
25
22
24
31
33
22
24
28
28
21
27
11
24
22
17
19
17
13
23
31
32
18
17
35
29
27
27
28
32
25
28
36
25
25
35
28
21

21
29
30
13
15
31
24
16
23
24
20
23
37
34
19
21
21
23
41
28
32
28
32
18
43
21
24
26
22
23
20
19
24
19
22
23
28
21
23
35
35
22
23
30
25
17
21
22
20
31
19
14
29
35
36
16
22
23
22

29
29
29
33
20
26
29
26
28
22
34
37
20
28
27
37
29
17
30
28
34
31
34
23
30
23
31
42
22
22
23
16
25
26
33
16
41
24
30
28
26
21
21
27
38
40
15
42
26
19
35
22
18
16
12
21
24
18
23

34
19
21
27
26
21
25
28
25
32
25
21
28
25
30
14
42
25
16
31
21
26
15
24
38
26
26
34
21
31
23
17
18
31
25
20
29
18
26
26
24
29
15
22
25
32
24
38
19
34
23
22
27
26
17
23
36
30
22

18
27
21
36
31
24
18
18
20
29
23
27
20
29
20
35
18
12
21

27	28	29
CAAAGTGCTGTTCGTGCAGGTAG	TCAGTGCACTACAGAACTTTGT	TGTCAGTTTGTCAAATACCCCA
miR-93-5p	miR-148a-3p	miR-223-3p
679306	664224	624773
0.23	0.15	0.10
0.07	0.02	0.16
0.31	0.07	0.11
0.26	0.08	0.07
0.09	0.03	0.20
0.06	0.21	0.02
0.17	0.11	0.02
0.10	0.06	0.03
0.14	0.05	0.04
0.17	0.06	0.06
0.09	0.13	0.28
0.08	0.06	0.02
0.21	0.33	0.20
0.13	0.03	0.04
0.23	0.10	0.08
0.15	0.17	0.15
0.17	0.08	0.05
0.26	0.06	0.15
0.12	0.30	0.45
0.12	0.18	0.25
0.13	0.19	0.02
0.13	0.14	0.08
0.14	0.13	0.08
0.10	0.07	0.02
0.07	0.07	0.26
0.16	0.14	0.22
0.19	0.21	0.06
0.14	0.17	0.23
0.12	0.50	0.21
0.17	0.19	0.10
0.27	0.06	0.02
0.15	0.33	0.14
0.08	0.05	0.58
0.16	0.14	0.17
0.20	0.08	0.17
0.11	0.12	0.14
0.04	0.01	0.00
0.18	0.25	0.07
0.34	0.22	0.27
0.11	0.07	0.20
0.08	0.05	0.02
0.09	0.05	0.04
0.14	0.07	0.07
0.24	0.46	0.20
0.07	0.06	0.06
0.04	0.01	0.01
0.11	0.21	0.54
0.11	0.17	0.11
0.14	0.07	0.17
0.11	0.07	0.10

0.12	0.08	0.08
0.14	0.17	0.08
0.15	0.14	0.05
0.18	0.20	0.21
0.08	0.16	0.04
0.12	0.15	0.06
0.13	0.14	0.05
0.15	0.14	0.22
0.17	0.11	0.20
0.17	0.10	0.32
0.11	0.06	0.43
0.19	0.18	0.15
0.15	0.10	0.09
0.14	0.07	0.04
0.17	0.11	0.10
0.33	0.09	0.06
0.27	0.14	0.14
0.20	0.15	0.13
0.42	0.04	0.08
0.37	0.12	0.31
0.23	0.13	0.08
0.07	0.24	0.05
0.16	0.08	0.04
0.19	0.05	0.16
0.03	0.01	0.02
0.15	0.12	0.07
0.12	0.19	0.18
0.07	0.18	0.06
0.26	0.19	0.31
0.10	0.15	0.33
0.11	0.09	0.09
0.12	0.14	0.19
0.07	0.07	0.04
0.11	0.19	0.33
0.13	0.19	0.14
0.09	0.07	0.15
0.13	0.15	0.17
0.13	0.12	0.08
0.14	0.38	0.20
0.12	0.11	0.25
0.18	0.21	0.23
0.12	0.44	0.04
0.13	0.17	0.06
0.18	0.34	0.14
0.27	0.20	0.14
0.00	0.00	0.00
0.00	0.00	0.00
0.00	0.00	0.00

28	29	30
A_1	*A_13*	*A_14*

0.13		
0.17	0.67	
-0.11	0.01	0.33
0.25	0.64	0.78
0.11	0.01	-0.07
-0.28	-0.05	-0.13
-0.19	-0.20	-0.05
-0.28	-0.01	-0.22
-0.05	-0.28	-0.02
0.02	0.27	0.25
0.33	0.09	0.18
-0.24	-0.02	-0.23
-0.29	-0.20	-0.17
-0.22	-0.19	-0.18
-0.35	-0.17	-0.27
0.14	0.36	0.27
-0.31	0.03	0.06
-0.28	-0.27	-0.22
0.02	0.33	-0.07
-0.42	0.03	-0.33
-0.02	-0.16	-0.27
-0.27	-0.05	-0.32
-0.01	0.03	-0.20
-0.30	0.06	-0.15
-0.25	-0.08	-0.09
-0.26	0.03	-0.30
-0.27	0.04	-0.09

-0.31	0.04	-0.01
-0.06	0.08	-0.05
0.86	0.03	0.05
-0.40	-0.07	-0.32
-0.11	0.03	-0.33
-0.05	0.03	-0.27
0.86	0.06	0.15
-0.16	-0.25	-0.11
-0.04	-0.07	-0.23
0.40	0.06	0.04
0.03	0.03	0.20
0.44	0.21	0.33
-0.02	0.45	0.69
-0.12	-0.36	-0.23
-0.09	0.03	-0.18
-0.10	0.09	0.15
0.22	0.39	0.27
0.32	-0.01	0.20
0.22	-0.04	-0.06
-0.23	-0.22	-0.17
0.24	0.02	-0.22
0.07	0.59	0.65
0.01	0.48	0.47
0.30	0.16	0.08
0.17	0.29	0.03
-0.06	-0.17	-0.14
-0.02	0.30	-0.02
-0.29	-0.23	-0.38
0.69	0.07	0.04
0.81	0.03	0.10
-0.15	0.04	-0.18
-0.11	0.20	-0.24
-0.03	0.01	-0.17
0.15	0.53	0.38
-0.15	0.09	-0.12
0.34	0.15	0.18
-0.15	0.35	0.36
-0.43	-0.20	-0.44
0.30	0.39	0.34
0.04	0.15	0.19

26	27	28
128	129	130
TGAGCGCCTCGACGACAGAGCCG	ACTCCAGCCCCACAGCCTCAGC	TGGGTCTTTGCGGGCGAGATGA
miR-339-3p	miR-766-3p	miR-193a-5p
12315	12195	11818
0.88	-0.44	-0.63
-1.52	0.51	-0.46
-0.69	-0.56	-0.30
-0.69	-0.77	-0.16
-1.02	1.23	1.71
-0.78	-1.00	-0.45
0.66	-0.48	0.04
0.73	0.13	-0.29
-1.17	-0.24	-0.64
-1.20	-0.47	-0.71
-1.17	0.12	-0.63
-0.11	-1.03	-0.01
1.27	-0.56	-0.44
0.43	0.98	0.18
0.01	-0.19	-0.20
0.44	-0.13	-0.44
0.68	0.31	-0.01
2.47	-0.31	-0.33
-0.04	0.04	-0.53
-0.29	-0.39	-0.48

-0.16	-0.70	-0.50
1.08	0.33	0.55
1.28	0.37	0.73
1.08	0.36	0.06
-1.52	4.15	4.30
-0.35	-0.58	-0.07
-0.33	-0.70	-0.31
-0.78	-0.69	-0.60
-0.78	0.10	-0.45
-0.82	0.29	-0.69
-1.07	2.23	-0.75
-0.91	2.03	-0.68
-1.40	1.16	0.36
-0.44	-0.13	0.89
-0.04	0.09	0.33
0.23	-0.02	-0.06
-1.15	-1.01	-0.66
-0.06	-0.92	-0.76
-0.32	-0.34	-0.59
0.57	-0.46	-0.26
-0.12	3.48	0.02
1.11	14.49	-0.28
0.44	0.41	0.12
-0.15	-0.39	-0.22
0.95	0.52	0.17
-0.67	1.69	0.43
0.34	0.12	-0.09
0.82	-0.18	0.05
0.46	-0.38	-0.27
1.06	-0.40	-0.30
1.25	-0.08	-0.42
0.50	1.62	0.08
0.00	0.85	0.57
0.88	-0.05	0.06
-0.24	-0.60	0.26
0.18	0.04	-0.11
0.30	-0.67	-0.23
-0.75	-1.00	-0.47
-0.12	-0.38	1.06
-0.44	-0.54	1.00
-0.94	-0.73	0.61
-1.01	-1.04	-0.69
-0.88	-0.61	-0.40
-0.37	-0.92	-0.38
-1.07	-0.98	-0.37
-0.50	-0.65	0.15
-0.74	-0.65	-0.77
-1.52	0.99	0.01
-0.06	1.32	-0.15
0.54	0.81	0.44
0.20	-0.27	-0.26
-0.05	-1.10	-0.76
-0.45	-0.80	-0.57
0.29	-0.57	-0.34
1.36	17.98	1.07
-0.56	-0.82	-0.22
-0.54	0.05	-0.26
0.57	-0.89	-0.36
-0.39	0.30	-0.20

-0.42	0.21	-0.42
-0.71	1.56	0.23
0.09	-0.03	-0.40
0.94	-0.54	-0.66
-0.22	-0.71	-0.67
-0.87	-0.90	-0.63
0.89	0.16	-0.60
0.82	-0.23	-0.26
-0.16	-0.50	-0.18
0.14	-0.55	-0.16
0.16	0.30	-0.10
-1.22	-0.68	-0.12
-0.22	-0.31	-0.03
0.89	0.32	0.43
-1.15	-0.28	-0.28
-0.92	-0.61	-0.79
-1	0	-1
0.00	0.00	0.00
1.00	1.00	1.00
0.81	0.97	0.31

128	129	130
TGAGCGCCTCGACGACAGAGCCG	ACTCCAGCCCCACAGCCTCAGC	TGGGTCTTTGCGGGCGAGATGA
miR-339-3p	miR-766-3p	miR-193a-5p
12315.00	12195.00	11818.00
0.88	-0.44	-0.63
-1.52	0.51	-0.46
-0.69	-0.56	-0.30
-0.69	-0.77	-0.16
-1.02	1.23	1.71
-0.78	-1.00	-0.45
0.66	-0.48	0.04
0.73	0.13	-0.29
-1.17	-0.24	-0.64
-1.20	-0.47	-0.71
-1.17	0.12	-0.63
-0.11	-1.03	-0.01
1.27	-0.56	-0.44
0.43	0.98	0.18
0.01	-0.19	-0.20
0.44	-0.13	-0.44
0.68	0.31	-0.01
2.47	-0.31	-0.33
-0.04	0.04	-0.53
-0.29	-0.39	-0.48
-0.16	-0.70	-0.50
1.08	0.33	0.55
1.28	0.37	0.73
1.08	0.36	0.06
-1.52	4.15	4.30
-0.35	-0.58	-0.07
-0.33	-0.70	-0.31
-0.78	-0.69	-0.60

-0.78	0.10	-0.45
-0.82	0.29	-0.69
-1.07	2.23	-0.75
-0.91	2.03	-0.68
-1.40	1.16	0.36
-0.44	-0.13	0.89
-0.04	0.09	0.33
0.23	-0.02	-0.06
-1.15	-1.01	-0.66
-0.06	-0.92	-0.76
-0.32	-0.34	-0.59
0.57	-0.46	-0.26
-0.12	3.48	0.02
1.11	14.49	-0.28
0.44	0.41	0.12
-0.15	-0.39	-0.22
0.95	0.52	0.17
-0.67	1.69	0.43
0.34	0.12	-0.09
0.82	-0.18	0.05
0.46	-0.38	-0.27
1.06	-0.40	-0.30
1.25	-0.08	-0.42
0.50	1.62	0.08
0.00	0.85	0.57
0.88	-0.05	0.06
-0.24	-0.60	0.26
0.18	0.04	-0.11
0.30	-0.67	-0.23
-0.75	-1.00	-0.47
-0.12	-0.38	1.06
-0.44	-0.54	1.00
-0.94	-0.73	0.61
-1.01	-1.04	-0.69
-0.88	-0.61	-0.40
-0.37	-0.92	-0.38
-1.07	-0.98	-0.37
-0.50	-0.65	0.15
-0.74	-0.65	-0.77
-1.52	0.99	0.01
-0.06	1.32	-0.15
0.54	0.81	0.44
0.20	-0.27	-0.26
-0.05	-1.10	-0.76
-0.45	-0.80	-0.57
0.29	-0.57	-0.34
1.36	17.98	1.07
-0.56	-0.82	-0.22
-0.54	0.05	-0.26
0.57	-0.89	-0.36
-0.39	0.30	-0.20
-0.42	0.21	-0.42
-0.71	1.56	0.23
0.09	-0.03	-0.40
0.94	-0.54	-0.66
-0.22	-0.71	-0.67
-0.87	-0.90	-0.63
0.89	0.16	-0.60
0.82	-0.23	-0.26

-0.16	-0.50	-0.18
0.14	-0.55	-0.16
0.16	0.30	-0.10
-1.22	-0.68	-0.12
-0.22	-0.31	-0.03
0.89	0.32	0.43
-1.15	-0.28	-0.28
-0.92	-0.61	-0.79

128	129	130
105	86	58
TGTAACAGCAACTCCATGTGGA	CCTCTGGGCCCTTCCTCCAG	AACATTCAACCTGTCGGTGAGT
miR-194-5p	miR-326	miR-181c-5p
25540	49098	125038
-0.50	2.24	-0.50
1.09	-1.55	-1.47
1.23	-0.84	-0.55
0.17	-0.78	-0.98
-0.08	-0.87	0.26
-0.08	-0.98	-0.88
-0.76	0.64	-0.27
-0.73	-0.03	0.06
-0.31	-0.96	-1.04
0.90	-1.05	-1.47
0.17	-0.63	0.04
-1.22	-0.57	0.05
2.42	0.84	2.83
-0.90	0.29	-0.52
-0.13	0.14	-0.10
1.51	0.33	1.24
-0.73	2.07	0.00
0.68	1.42	0.46
-0.19	-0.39	0.62
0.85	-0.03	1.60
-0.14	-0.71	-1.20
-1.30	1.43	1.50
-0.92	0.88	0.66
-0.82	0.65	-0.36
-1.17	-1.08	-0.69
-0.88	-0.12	0.64
1.85	-0.34	0.09
8.06	-0.80	-0.39
1.27	-1.13	-0.81
0.76	-1.22	-1.31
-0.56	-1.35	-1.85
2.25	-1.29	-1.36
1.82	-0.51	0.51
-0.71	0.89	3.03
1.25	2.96	0.77
-0.90	0.97	0.44
-1.26	-1.16	-1.63
1.43	0.76	0.01
0.76	3.53	1.78

-0.49	0.39	1.51
-1.05	19.65	-1.56
-0.98	91.37	-1.01
-0.94	1.00	-0.49
1.76	0.30	0.39
-0.39	-0.53	-0.49
-0.94	8.68	-1.50
0.16	0.53	3.44
-0.77	0.69	2.39
0.47	0.52	0.87
-0.61	0.41	2.72
0.45	0.80	0.56
-1.04	1.31	-0.70
-0.95	0.33	-1.22
-0.39	1.02	0.24
-0.86	-0.86	-0.80
-0.88	-0.14	-0.76
0.17	-0.70	-1.17
1.99	-0.62	0.93
-1.09	-0.17	0.55
0.51	-0.71	1.93
-0.33	-0.73	0.98
2.90	-1.04	-0.32
-1.18	-0.39	-0.55
-0.55	-0.68	-1.02
0.15	-0.96	-0.54
-0.94	-0.48	-0.60
2.71	-0.27	-1.08
0.09	-1.55	-1.28
0.49	-0.17	-0.73
1.91	0.57	1.85
-0.10	-0.08	-0.67
4.86	-1.53	-0.96
0.52	-0.77	-0.67
3.26	0.24	0.92
-1.53	87.60	-1.84
0.03	-0.36	0.47
0.42	-0.68	-0.13
1.24	-1.28	-0.71
0.53	2.29	2.33
0.33	0.20	2.48
-0.53	0.26	1.12
-0.49	-0.20	0.81
-0.56	-0.01	1.16
1.47	1.08	2.75
2.20	-0.89	0.03
-0.19	1.46	1.59
-0.47	0.10	2.45
-0.20	-0.06	-0.01
0.87	-0.52	0.77
-0.39	0.29	0.39
-0.85	-1.36	2.24
-0.73	-1.01	-0.87
-0.84	0.84	1.01
-0.52	-0.83	-0.12
0.65	0.86	-0.95
1.95	2.07	2.25
1.00	0.54	0.59

ion of 29s

NP	P
26	17
29	27
15	33
32	24
30	21
25	12
42	23
36	21
17	33
32	25

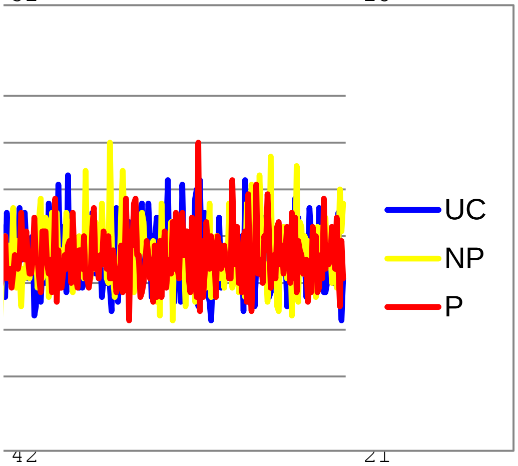

42	21
25	23
27	19
19	25
19	35
15	23
32	25
21	29
24	17
21	23
26	23
29	31
30	24
19	29
17	40
39	22
17	18
15	21
24	29
25	27
23	16
21	16
33	23
25	29
25	27
22	30
20	34

26	23
30	32
38	20
18	23
28	30
19	34
32	33
16	27
18	22
22	37
12	26
23	21
19	23
38	21
30	15
25	23
26	24
36	26
25	33
25	20
34	28
21	24
34	15
17	32
30	28
28	16
32	27
31	17
37	34
24	37
18	42
26	30
35	30
22	25
24	24
25	23
23	28
27	29
16	38
22	40
27	22
21	26
22	23
19	33
25	24
22	26
23	27
24	20
17	26
25	32
40	26
21	22
31	15
25	31
22	23
26	29
28	19
29	28
26	34

24	17
24	30
36	30
25	30
29	23
22	36
18	28
31	36
23	29
30	17
17	16
28	29
23	20
20	29
27	23
35	33
20	29
17	22
42	22
27	24
19	29
31	31
24	34
25	21
24	28
17	24
24	29
13	31
26	26
22	32
26	28
19	23
32	22
35	24
28	18
17	25
20	27
29	18
28	29
17	35
29	33
33	14
26	20
25	29
26	19
31	29
21	14
26	28
23	36
22	19
20	24
24	34
37	25
57	26
27	17
24	19
25	34
33	27
19	24

21	25
29	24
21	30
29	15
18	34
23	23
28	25
29	27
32	19
31	14
24	30
35	38
27	17
30	24
20	23
22	34
14	30
22	25
24	28
45	15
24	13
15	28
23	23
28	26
26	22
11	22
18	20
24	28
27	22
14	34
30	18
22	26
29	20
22	22
15	16
18	25
21	39
30	11
25	22
49	26
20	17
30	20
17	20
31	29
26	23
23	39
24	20
44	26
21	52
22	24
30	23
29	40
31	27
32	14
25	29
24	26
30	20
22	33
20	25

19	22
21	18
23	20
30	21
28	28
36	22
33	32
22	24
29	23
30	28
18	30
24	21
23	35
28	16
18	31
18	35
22	18
16	17
28	21
24	21
17	22
48	17
24	21
25	23
31	22
19	20
13	29
33	20
20	25
27	21
18	22
52	10
26	27
21	36
36	23
26	33
27	15
25	16
22	34
9	24
20	22
26	27
26	28
27	23
25	16
21	27
22	18
28	21
26	25
28	23
23	20
25	28
24	25
22	35
22	22
23	22
23	17
25	26
32	12

24	32
21	41
18	16
27	20
19	17
19	30
39	27
25	13
31	34
24	14
14	36
36	22
33	24
23	23
40	38
25	25
27	31
23	20
56	22
29	33
26	31
21	22
18	28
19	29
28	19
18	37
21	44
26	24
20	25
23	26
22	30
17	34
21	30
19	32
22	26
27	16
26	19
35	10
24	36
30	18
29	14
40	20
15	39
29	27
23	12
20	18
24	28
20	26
27	17
28	17
34	24
31	26
30	27
20	22
17	41
38	15
24	28
26	32
26	18

14	35
21	23
29	31
33	24
44	13
25	26
21	35
33	24
22	26
38	23
25	28
15	24
41	11
27	18
26	33
21	24
21	17
34	21
27	27
21	21
15	20
35	23
31	25
22	34
8	23
32	40
29	21
18	24
29	22
33	28
20	38
17	26
14	37
23	33
24	37
23	34
25	23
20	23
35	22
26	32
25	28
21	25
17	31
31	31
26	21
32	37
12	28
23	26
43	15
21	34
10	44
33	23
26	21
30	25
30	34
21	50
31	39
28	37
24	15

29	21
19	14
24	23
27	25
16	28
26	28
28	28
23	32
24	25
30	23
23	26
35	15
24	33
18	26
27	20
21	37
29	28
31	25
27	39
18	38
37	19
22	22
41	28
11	44
28	25
19	31
28	14
39	13
23	25
23	19
29	21
21	21
29	21
32	22
34	22
22	21
30	19
28	21
33	27
13	24
41	24
35	20
24	36
12	24
25	29
19	24
29	21
29	25
19	25
23	30
26	21
31	37
21	24
25	38
20	25
23	18
35	25
24	19
25	24

20	30
27	26
20	22
36	21
24	21
29	20
22	22
26	27
14	35
22	42
30	32
31	19
20	29
27	36
28	26
27	24
19	28
33	17
23	29
20	25
44	23
19	26
22	31
27	29
20	25
21	23
24	27
27	36
26	31
24	25
18	28
32	17
14	31
21	33
20	25
30	30
42	21
35	24
24	23
27	26
23	29
29	24
28	19
25	21
23	19
32	17
20	17
19	22
30	17
24	35
19	19
21	34
16	29
45	24
15	35
29	16
15	32
34	22
46	15

18	33
18	26
18	33
17	35
46	18
19	19
21	28
26	31
27	16
27	46
31	15
16	28
20	13
32	21
34	22
22	31
26	24
23	19
14	34
18	31
45	31
25	32
19	33
27	22
20	18
21	36
15	30
27	24
31	17
24	35
18	24
24	16
19	38
17	27
22	17
37	37
12	25
28	23
20	24
27	26
32	32
18	33
27	19
28	32
21	17
25	23
22	36
29	37
16	33
21	16
27	17
20	16
17	50
25	33
17	31
34	28
20	24
32	34

33	24
22	23
25	37
23	34
26	21
24	24
29	31
20	17
18	20
33	13
32	18
25	18
18	30
24	25
25	23
29	24
20	26
22	21
24	22
24	20
30	34
20	41
18	33
23	17
17	28
28	33
31	16
20	13
23	37
23	33
25	33
25	18
18	36
20	23
25	20
22	27
26	22
23	18
15	14
33	16
33	20
18	28
29	21
21	14
24	24
23	21
26	28
23	16
25	31
31	32
28	24
20	27
30	19
24	34
35	12
33	20
30	20
23	23
29	25

19	21
26	30
30	26
21	28
33	21
27	29
31	18
24	28
42	24
31	25
19	21
23	21
23	21
26	19
32	21
19	28
23	33
27	24
29	29
18	25
17	34
27	15
30	25
30	32
25	20
24	22
23	23
21	19
23	33
21	24
30	24
18	39
30	23
21	29
28	12
31	18
23	35
10	26
26	26
20	33
17	22
23	32
41	19
16	41
25	20
19	24
23	30
28	16
46	20
27	21
30	28
38	24
19	20
25	29
28	19
23	29
19	29
26	30
37	22

29	18
19	27
13	36
23	39
18	33
21	21
19	29
30	17
42	20
21	29
19	21
31	27
21	17
27	19
26	17
31	22
18	26
28	20
15	17
24	25
22	38
27	19
17	18
21	20
15	18
11	15
28	41
32	32
34	20
24	37
30	21
23	19
19	32
20	21
27	21
24	18
22	20
21	24
17	26
27	20
37	20
21	29
30	19
20	37
41	20
23	28
30	27
21	25
21	31
32	20
30	23
24	25
24	24
29	16
32	25
31	12
36	21
17	30
15	53

24	17
21	20
17	32
32	24
20	29
26	22
27	25
23	31
22	31
20	23
28	16
18	28
27	17
29	23
19	30
31	22
28	19
24	23
28	28
39	32
38	16
21	25
28	30
31	15
27	22
37	26
18	38
11	19
22	21
27	28
30	30
23	21
28	23
21	23
25	19
36	22
21	26
19	25
29	23
22	25
15	35
23	28
21	25
24	31
26	25
21	22
24	22
23	25
26	34
27	25
19	25
34	21
38	18
21	31
30	31
34	31
33	24
17	22
19	25

35	18
31	25
22	38
35	16
25	27
25	19
27	19
24	22
27	26
35	23
28	28
33	29
24	20
18	35
23	25
25	21
27	19
30	27
25	27
29	23
21	30
44	21
25	21
34	19
21	22
29	32
23	36
33	23
25	25
23	21
24	26
37	24
27	31
30	25
21	23
20	30
50	21
31	21
21	27
17	23
22	18
22	23
23	19
35	28
44	18
33	21
21	38
20	26
29	12
30	31
27	28
18	37
20	38
22	26
25	26
29	17
35	18
33	20
26	24

22	29
23	26
27	21
25	25
29	16
24	28
16	17
26	24
13	29
37	17
27	26
26	31
30	19
23	24
25	25
33	22
12	33
30	23
25	35
29	23
26	21
24	33
22	35
27	26
15	31
24	31
28	21
31	18
22	34
31	28
16	20
26	17
18	50
17	14
21	29
22	17
25	22
19	33
24	27
37	23
17	30
28	27
24	26
30	17
30	30
21	28
22	23
26	24
19	28
26	24
23	24
37	21
27	21
19	42
21	29
22	24
28	32
18	20
20	27

25	18
28	30
17	31
37	16
28	39
28	20
41	14
31	25
23	24
30	41
29	22
43	22
32	25
25	20
24	30
23	27
16	39
21	30
47	19
21	24
27	26
30	21
15	32
14	33
28	24
23	28
32	22
26	26
21	32
22	26
29	20
13	35
31	21
20	34
45	18
16	29
33	27
32	26
22	22
30	22
24	25
29	16
24	20
26	18
22	32
23	26
17	30
32	18
23	19
23	26
32	21
27	38
34	23
24	28
27	24
28	25
20	32
21	27
23	23

19	34
26	24
40	15
31	29
37	21

16	27
24	35
18	24
24	16
19	38
17	27
22	17
37	37
12	25
28	23
20	24
27	26
32	32
18	33
27	19
28	32

21	17
25	23
22	36
29	37
16	33
21	16
27	17
20	16
17	50
25	33
17	31
34	28
20	24
32	34
33	24
22	23
25	37
23	34
26	21
24	24
29	31
20	17
18	20
33	13
32	18
25	18
18	30
24	25
25	23
29	24
20	26
22	21
24	22
24	20
30	34
20	41
18	33
23	17
17	28
28	33
31	16
20	13
23	37
23	33
25	33
25	18
18	36
20	23
25	20
22	27
26	22
23	18
15	14
33	16
33	20
18	28
29	21
21	14
24	24

23	21
26	28
23	16
25	31
31	32
28	24
20	27
30	19
24	34
35	12
33	20
30	20
23	23
29	25
19	21
26	30
30	26
21	28
33	21
27	29
31	18
24	28
42	24
31	25
19	21
23	21
23	21
26	19
32	21
19	28
23	33
27	24
29	29
18	25
17	34
27	15
30	25
30	32
25	20
24	22
23	23
21	19
23	33
21	24
30	24
18	39
30	23
21	29
28	12
31	18
23	35
10	26
26	26
20	33
17	22
23	32
41	19
16	41
25	20

19	24
23	30
28	16
46	20
27	21
30	28
38	24
19	20
25	29
28	19
23	29
19	29
26	30
37	22
29	18
19	27
13	36
23	39
18	33
21	21
19	29
30	17
42	20
21	29
19	21
31	27
21	17
27	19
26	17
31	22
18	26
28	20
15	17
24	25
22	38
27	19
17	18
21	20
15	18
11	15
28	41
32	32
34	20
24	37
30	21
23	19
19	32
20	21
27	21
24	18
22	20
21	24
17	26
27	20
37	20
21	29
30	19
20	37
41	20

23	28
30	27
21	25
21	31
32	20
30	23
24	25
24	24
29	16
32	25
31	12
36	21
17	30
15	53
24	17
21	20
17	32
32	24
20	29
26	22
27	25
23	31
22	31
20	23
28	16
18	28
27	17
29	23
19	30
31	22
28	19
24	23
28	28
39	32
38	16
21	25
28	30
31	15
27	22
37	26
18	38
11	19
22	21
27	28
30	30
23	21
28	23
21	23
25	19
36	22
21	26
19	25
29	23
22	25
15	35
23	28
21	25
24	31
26	25

21	22
24	22
23	25
26	34
27	25
19	25
34	21
38	18
21	31
30	31
34	31
33	24
17	22
19	25
35	18
31	25
22	38
35	16
25	27
25	19
27	19
24	22
27	26
35	23
28	28
33	29
24	20
18	35
23	25
25	21
27	19
30	27
25	27
29	23
21	30
44	21
25	21
34	19
21	22
29	32
23	36
33	23
25	25
23	21
24	26
37	24
27	31
30	25
21	23
20	30
50	21
31	21
21	27
17	23
22	18
22	23
23	19
35	28
44	18

33	21
21	38
20	26
29	12
30	31
27	28
18	37
20	38
22	26
25	26
29	17
35	18
33	20
26	24
22	29
23	26
27	21
25	25
29	16
24	28
16	17
26	24
13	29
37	17
27	26
26	31
30	19
23	24
25	25
33	22
12	33
30	23
25	35
29	23
26	21
24	33
22	35
27	26
15	31
24	31
28	21
31	18
22	34
31	28
16	20
26	17
18	50
17	14
21	29
22	17
25	22
19	33
24	27
37	23
17	30
28	27
24	26
30	17
30	30

21	28
22	23
26	24
19	28
26	24
23	24
37	21
27	21
19	42
21	29
22	24
28	32
18	20
20	27
25	18
28	30
17	31
37	16
28	39
28	20
41	14
31	25
23	24
30	41
29	22
43	22
32	25
25	20
24	30
23	27
16	39
21	30
47	19
21	24
27	26
30	21
15	32
14	33
28	24
23	28
32	22
26	26
21	32
22	26
29	20
13	35
31	21
20	34
45	18
16	29
33	27
32	26
22	22
30	22
24	25
29	16
24	20
26	18
22	32

23	26
17	30
32	18
23	19
23	26
32	21
27	38
34	23
24	28
27	24
28	25
20	32
21	27
23	23
19	34
26	24
40	15
31	29
37	21

30	31	32
TGAGAACTGAATTCCATAGGCT	TACCACAGGGTAGAACCACGG	AAAAGCTGGGTTGAGAGGGCGA
miR-146b-5p	miR-140-3p	miR-320a
614674	605614	549148
0.03	0.19	0.17
0.47	0.02	0.07
0.14	0.14	0.12
0.06	0.20	0.12
0.21	0.02	0.11
0.06	0.08	0.11
0.01	0.10	0.20
0.01	0.08	0.12
0.09	0.08	0.08
0.27	0.08	0.09
0.80	0.10	0.04
0.04	0.07	0.15
0.18	0.10	0.07
0.02	0.10	0.17
0.06	0.16	0.16
0.10	0.13	0.11
0.05	0.19	0.19
0.05	0.14	0.17
0.16	0.17	0.07
0.25	0.17	0.11
0.07	0.10	0.13
0.02	0.19	0.19
0.03	0.15	0.25
0.02	0.13	0.34
0.06	0.01	0.13
0.12	0.09	0.17
0.12	0.11	0.09
0.41	0.11	0.05
0.61	0.18	0.05
0.32	0.09	0.05
0.03	0.04	0.17
1.21	0.04	0.04
0.15	0.02	0.09
0.02	0.15	0.12
0.02	0.10	0.12
0.07	0.17	0.11
0.00	0.04	0.17
0.18	0.17	0.07
0.17	0.11	0.06
0.14	0.11	0.09
0.06	0.12	0.38
0.12	0.24	1.43
0.04	0.16	0.12
0.06	0.17	0.06
0.06	0.11	0.13
0.02	0.07	0.43
0.21	0.19	0.07
0.06	0.15	0.17
0.05	0.18	0.12
0.06	0.22	0.12

0.12	0.17	0.10
0.03	0.19	0.21
0.02	0.17	0.20
0.08	0.16	0.15
0.03	0.13	0.15
0.03	0.19	0.16
0.05	0.15	0.14
0.32	0.09	0.09
0.13	0.07	0.26
0.15	0.04	0.21
0.26	0.04	0.19
0.22	0.06	0.08
0.03	0.09	0.19
0.07	0.07	0.13
0.25	0.10	0.12
0.11	0.10	0.36
0.13	0.18	0.07
0.37	0.03	0.07
0.06	0.07	0.37
0.19	0.12	0.15
0.05	0.18	0.12
0.21	0.11	0.02
0.08	0.11	0.10
0.30	0.11	0.10
0.01	0.18	1.74
0.08	0.10	0.18
0.30	0.13	0.06
0.28	0.06	0.06
0.29	0.25	0.10
0.21	0.14	0.06
0.04	0.13	0.14
0.16	0.13	0.11
0.03	0.11	0.14
0.41	0.16	0.10
0.27	0.12	0.07
0.06	0.17	0.15
0.11	0.22	0.11
0.05	0.18	0.13
0.27	0.20	0.07
0.09	0.16	0.11
0.68	0.08	0.20
0.03	0.17	0.09
0.04	0.18	0.17
0.21	0.07	0.07
0.12	0.09	0.12
0.00	0.00	0.00
0.00	0.00	0.00
0.00	0.00	0.00

31	32	33
A_15	*A_17*	*B_30*

0.25		
-0.02	0.07	
-0.07	-0.29	-0.17
0.00	-0.17	0.02
-0.33	-0.25	-0.24
0.44	-0.20	-0.18
-0.20	0.18	-0.21
-0.25	0.19	0.01
-0.47	-0.17	-0.10
0.05	-0.23	-0.16
-0.01	-0.19	-0.15
0.05	-0.24	-0.22
-0.19	0.16	0.03
-0.01	-0.05	-0.27
0.08	-0.29	-0.19
-0.38	0.06	0.23
-0.21	-0.24	-0.34
-0.26	-0.28	0.04
-0.32	-0.32	-0.21
-0.28	-0.06	-0.06
0.31	-0.04	-0.16
0.43	-0.06	-0.09
-0.35	-0.25	0.20
0.03	-0.12	-0.28

0.33	-0.04	-0.12
0.31	0.01	-0.03
-0.19	0.12	0.07
-0.13	-0.21	-0.16
-0.31	-0.08	0.39
-0.29	-0.07	0.58
-0.13	0.20	0.13
0.12	-0.26	-0.15
-0.02	-0.22	-0.06
-0.15	0.13	0.12
0.02	0.14	-0.33
-0.03	0.24	0.08
0.36	0.70	0.12
0.31	-0.15	0.18
-0.06	-0.03	0.21
0.27	-0.06	-0.33
-0.16	0.26	0.09
-0.04	0.07	0.12
-0.33	-0.01	0.11
0.05	-0.21	-0.14
-0.26	-0.11	-0.06
-0.05	0.61	-0.01
0.16	0.52	-0.14
-0.22	0.18	0.19
-0.34	0.18	0.28
0.05	-0.09	0.10
-0.16	0.14	0.22
-0.20	-0.37	-0.26
-0.29	0.06	0.03
-0.13	0.11	0.06
-0.32	-0.14	0.08
-0.47	-0.16	-0.01
-0.20	-0.23	0.16
-0.34	0.28	-0.06
-0.32	-0.13	0.18
-0.13	0.35	0.02
0.12	0.17	-0.38
-0.19	-0.35	-0.40
-0.03	0.44	0.04
0.01	0.13	-0.11

29	30	31
131	132	133
AATGCACCCGGGCAAGGATTCT	CCTGTTCTCCATTACTTGGCTC	CCCAGTGTTCAGACTACCTGTTC
miR-501-3p	miR-26b-3p	miR-199a-5p
11405	11100	11007
-0.50	-0.35	-0.34
3.21	-1.72	-0.42
1.10	-0.62	-0.25
-0.41	-0.69	-1.00
-0.35	-0.58	-0.84
-0.76	0.02	-0.20
-0.67	-0.64	-0.75
-0.41	0.34	-1.14
-0.46	-1.16	-0.25
0.04	-1.32	-0.80
-0.76	-0.95	2.66
-0.65	0.86	-1.06
-0.56	2.27	0.93
-0.22	-0.78	-0.60
-0.12	0.14	0.03
-0.12	1.20	2.67
0.37	0.27	0.33
-0.64	1.60	-0.96
-0.16	0.73	-0.34
2.52	0.91	-0.08

-0.07	-0.73	-0.18
-0.22	1.83	0.08
-0.35	0.06	0.50
-0.42	-1.05	-0.47
-0.64	-0.25	0.18
-0.53	0.23	0.68
1.79	0.38	1.62
-0.29	-0.88	2.05
-0.05	1.54	1.23
-0.01	-1.02	-0.20
0.83	-1.49	-1.12
-0.63	0.10	1.36
-0.76	1.95	0.79
-0.61	0.32	-0.82
-0.42	0.71	-0.89
-0.56	-0.27	-0.56
-0.61	-1.64	-1.39
-0.67	-0.36	0.16
-0.27	1.08	1.75
-0.49	1.10	-0.19
2.77	1.35	-0.54
2.67	4.10	4.25
0.03	-0.14	1.35
-0.51	1.29	0.08
-0.54	2.78	-0.86
1.20	-0.33	-1.53
0.02	0.87	1.79
-0.34	0.78	-0.08
-0.24	0.55	0.09
-0.19	0.17	-0.49
-0.34	1.61	1.41
1.63	-0.19	1.74
2.03	-0.94	0.73
1.55	1.43	2.06
0.30	0.22	-0.56
2.79	-0.84	0.23
0.58	-0.08	0.42
0.92	-0.20	0.66
-0.55	0.52	0.28
-0.71	2.99	1.59
-0.76	1.83	0.37
1.92	-0.63	0.48
5.07	-0.15	-0.67
3.01	0.43	-0.37
2.10	-0.83	-0.17
-0.67	2.28	-0.46
-0.38	-0.05	0.36
-0.25	0.18	0.19
-0.69	0.67	-0.48
-0.68	4.25	0.55
-0.52	0.84	-0.72
-0.69	5.37	-1.25
-0.06	-0.31	-0.66
-0.46	1.75	-0.47
4.14	2.19	0.17
-0.58	1.31	-0.19
-0.38	0.48	0.08
-0.23	2.42	-0.99
0.16	-0.37	2.25

0.13	0.04	2.99
0.43	-0.97	0.32
-0.12	0.44	0.42
-0.31	1.76	-0.30
3.29	1.10	0.33
1.67	-0.68	-0.56
-0.32	1.31	2.97
-0.29	1.18	-0.11
-0.25	0.60	-0.55
0.25	0.40	-0.79
0.81	0.73	1.12
-0.35	-1.23	-0.32
-0.19	-0.18	-1.15
-0.17	0.69	-0.14
1.83	-0.77	0.84
4.53	0.24	1.67
-1	0	-1
0.00	0.00	0.00
1.00	1.00	1.00
0.27	0.23	0.35

131	132	133
AATGCACCCGGGCAAGGATTCT	CCTGTTCTCCATTACTTGGCTC	CCCAGTGTTCAGACTACCTGTTC
miR-501-3p	miR-26b-3p	miR-199a-5p
11405	11100	11007
-0.50	-0.35	-0.34
3.21	-1.72	-0.42
1.10	-0.62	-0.25
-0.41	-0.69	-1.00
-0.35	-0.58	-0.84
-0.76	0.02	-0.20
-0.67	-0.64	-0.75
-0.41	0.34	-1.14
-0.46	-1.16	-0.25
0.04	-1.32	-0.80
-0.76	-0.95	2.66
-0.65	0.86	-1.06
-0.56	2.27	0.93
-0.22	-0.78	-0.60
-0.12	0.14	0.03
-0.12	1.20	2.67
0.37	0.27	0.33
-0.64	1.60	-0.96
-0.16	0.73	-0.34
2.52	0.91	-0.08
-0.07	-0.73	-0.18
-0.22	1.83	0.08
-0.35	0.06	0.50
-0.42	-1.05	-0.47
-0.64	-0.25	0.18
-0.53	0.23	0.68
1.79	0.38	1.62
-0.29	-0.88	2.05

-0.05	1.54	1.23
-0.01	-1.02	-0.20
0.83	-1.49	-1.12
-0.63	0.10	1.36
-0.76	1.95	0.79
-0.61	0.32	-0.82
-0.42	0.71	-0.89
-0.56	-0.27	-0.56
-0.61	-1.64	-1.39
-0.67	-0.36	0.16
-0.27	1.08	1.75
-0.49	1.10	-0.19
2.77	1.35	-0.54
2.67	4.10	4.25
0.03	-0.14	1.35
-0.51	1.29	0.08
-0.54	2.78	-0.86
1.20	-0.33	-1.53
0.02	0.87	1.79
-0.34	0.78	-0.08
-0.24	0.55	0.09
-0.19	0.17	-0.49
-0.34	1.61	1.41
1.63	-0.19	1.74
2.03	-0.94	0.73
1.55	1.43	2.06
0.30	0.22	-0.56
2.79	-0.84	0.23
0.58	-0.08	0.42
0.92	-0.20	0.66
-0.55	0.52	0.28
-0.71	2.99	1.59
-0.76	1.83	0.37
1.92	-0.63	0.48
5.07	-0.15	-0.67
3.01	0.43	-0.37
2.10	-0.83	-0.17
-0.67	2.28	-0.46
-0.38	-0.05	0.36
-0.25	0.18	0.19
-0.69	0.67	-0.48
-0.68	4.25	0.55
-0.52	0.84	-0.72
-0.69	5.37	-1.25
-0.06	-0.31	-0.66
-0.46	1.75	-0.47
4.14	2.19	0.17
-0.58	1.31	-0.19
-0.38	0.48	0.08
-0.23	2.42	-0.99
0.16	-0.37	2.25
0.13	0.04	2.99
0.43	-0.97	0.32
-0.12	0.44	0.42
-0.31	1.76	-0.30
3.29	1.10	0.33
1.67	-0.68	-0.56
-0.32	1.31	2.97
-0.29	1.18	-0.11

-0.25	0.60	-0.55
0.25	0.40	-0.79
0.81	0.73	1.12
-0.35	-1.23	-0.32
-0.19	-0.18	-1.15
-0.17	0.69	-0.14
1.83	-0.77	0.84
4.53	0.24	1.67

131	132	133
67	93	107
TGTAAACATCCTACACTCAGCT	GCTGACTCCTAGTCCAGGGCTC	ACCCCACTCCTGGTACC
miR-30b-5p	miR-345-5p	miR-4286
82798	32906	23822
0.64	1.35	1.23
-0.89	-1.77	-1.16
-0.45	-0.13	-1.17
-0.52	0.62	-1.14
-0.18	-1.25	2.46
-0.72	-0.10	-0.88
-0.47	1.28	-0.14
-0.61	0.33	0.97
-0.71	-1.06	-1.28
0.46	-1.20	-0.78
-0.11	-0.86	-0.54
-0.47	1.37	-0.42
3.84	0.40	0.65
-0.66	-0.46	-0.31
-0.42	0.05	-0.35
0.82	0.27	0.21
-0.28	1.93	0.06
-0.07	-0.14	1.82
-0.06	-0.63	0.08
2.06	1.57	-0.38
-0.40	-0.92	-0.82
-0.53	0.65	0.43
-0.29	1.08	0.17
-0.41	-0.05	-0.14
-0.63	-1.26	1.54
0.17	-0.08	1.08
0.87	-1.02	-1.18
0.85	-0.66	-1.19
-0.31	0.08	-0.35
-0.67	-1.27	-0.90
-0.80	-1.69	-0.93
-0.05	-1.14	-0.14
0.13	-1.64	2.83
-0.38	1.81	1.84
1.87	-0.54	4.22
-0.45	1.27	0.21
-0.81	-1.26	-1.15
2.90	-0.90	-0.78
4.35	-0.90	-0.17

-0.04	0.86	0.42
-0.28	1.86	12.46
0.25	4.53	39.75
-0.60	-0.01	0.45
0.95	-0.23	0.75
-0.57	-0.77	0.84
-0.70	0.68	10.03
0.35	2.73	0.59
-0.02	0.90	0.71
0.42	2.36	0.63
0.03	1.62	0.21
1.25	0.28	0.32
-0.43	0.38	0.85
-0.61	-0.71	0.33
0.11	1.01	1.12
-0.57	-0.21	-0.26
-0.61	-0.42	-0.35
-0.07	-0.90	-0.28
3.23	-0.36	-0.69
-0.36	0.56	-0.15
0.66	-0.68	0.84
0.37	-0.85	0.61
1.93	-0.97	-1.24
-0.48	-0.41	-0.82
-0.08	-0.04	-0.48
-0.32	-0.73	-1.15
-0.32	-0.21	-1.28
1.20	-0.45	-0.75
-0.62	-1.44	1.31
-0.28	-0.76	-0.23
0.07	1.20	0.81
-0.52	0.44	-0.52
1.79	-1.22	-0.49
-0.01	-0.13	-0.94
1.35	1.41	0.25
-0.72	2.97	37.19
0.19	0.47	-0.45
-0.02	-0.85	-0.37
-0.59	-1.43	-0.64
1.60	0.43	-0.08
0.18	0.50	-0.33
-0.58	0.74	-0.45
0.03	2.02	-0.20
0.58	0.97	0.94
5.08	0.64	0.08
3.34	0.22	-1.23
0.03	0.98	1.05
0.30	1.86	0.01
-0.09	0.12	-0.29
0.61	0.13	-0.60
-0.15	0.07	-0.05
-0.30	-0.26	-1.40
-0.58	-0.33	-0.06
-0.30	1.06	0.31
-0.45	-0.68	-0.95
2.50	-1.12	-0.41
2.25	2.35	2.38
1.00	1.00	0.39

	NP	P	UC	NP
mean	25.79	25.42	25.27	25.79
sd	6.69	6.62	6.62	
	gamma fits			beta f
	NP	P	UC	NP
	K-S	K-S	K-S	K-S
	0.02	0.02	0.02	0.02
	A-D	A-D	A-D	A-D
	0.05	0.05	0.20	0.05
	alpha	alpha	alpha	alpha1
	14.20	14.43	14.71	12.46
	beta	beta	beta	alpha2
	1.79	1.76	1.75	#####
	p-val of ave			a
	0.56	0.54	0.51	1.73
	relabel+reselect aves			b
	25.79	25.42	25.27	#####
	p-val of ave			p-val
	0.56	0.54	0.51	0.56
	0.44	0.46	0.49	observ
	NP	P	UC	25.79
	obs: no relabel but reselect			p-val
	43.20	33.67	18.85	0.56
	p-val of observed ave of only reselect			0.44
	0.99	0.89	0.15	NP
	0.01	0.11	0.85	obs: r
				43.20
				p-val
				0.99
				0.01

33	34	35	36
TTTGGCAATGGTAGAACTCACACT	TCGAGGAGCTCACAGTCT	TCAGTGCATCACAGAACTTTGT	TGTAAACATCCTACACTC
miR-182-5p	miR-151b	miR-148b-3p	miR-30c-5p
501331	500966	497718	#####
0.04	0.06	0.11	0.07
0.42	0.17	0.04	0.05
0.17	0.19	0.07	0.08
0.09	0.09	0.07	0.05
0.04	0.26	0.04	0.11
0.01	0.08	0.13	0.04
0.03	0.07	0.17	0.04
0.02	0.03	0.07	0.07
0.13	0.11	0.07	0.07
0.22	0.10	0.07	0.07
0.28	0.19	0.13	0.10
0.02	0.06	0.05	0.08
0.10	0.12	0.11	0.20
0.03	0.05	0.05	0.04
0.15	0.10	0.07	0.10
0.14	0.17	0.12	0.16
0.03	0.07	0.14	0.09
0.10	0.10	0.06	0.08
0.17	0.07	0.11	0.16
0.20	0.14	0.07	0.18
0.13	0.13	0.17	0.05
0.05	0.08	0.17	0.10
0.05	0.10	0.15	0.08
0.03	0.07	0.12	0.05
0.04	0.12	0.08	0.08
0.03	0.09	0.17	0.11
0.32	0.10	0.14	0.13
0.27	0.19	0.06	0.06
0.10	0.12	0.15	0.10
0.25	0.09	0.09	0.05
0.16	0.10	0.14	0.02
0.54	0.17	0.14	0.11
0.05	0.33	0.03	0.17
0.05	0.03	0.09	0.10
0.12	0.04	0.06	0.14
0.01	0.06	0.11	0.11
0.01	0.02	0.03	0.01
0.11	0.05	0.09	0.13
0.19	0.08	0.09	0.13
0.03	0.07	0.06	0.15
0.06	0.06	0.34	0.05
0.08	0.09	0.43	0.05
0.02	0.12	0.16	0.06
0.14	0.08	0.15	0.15
0.03	0.06	0.07	0.12
0.03	0.04	0.16	0.02
0.08	0.19	0.14	0.27
0.05	0.12	0.16	0.14
0.13	0.13	0.07	0.22
0.06	0.10	0.08	0.19

0.08	0.13	0.13	0.20
0.02	0.15	0.28	0.06
0.07	0.16	0.23	0.04
0.04	0.17	0.18	0.16
0.06	0.11	0.16	0.07
0.01	0.12	0.16	0.05
0.07	0.15	0.17	0.10
0.25	0.08	0.07	0.11
0.03	0.07	0.14	0.10
0.08	0.14	0.13	0.24
0.10	0.15	0.08	0.21
0.35	0.12	0.07	0.10
0.07	0.06	0.11	0.07
0.09	0.08	0.12	0.11
0.04	0.13	0.10	0.09
0.40	0.10	0.07	0.06
0.13	0.09	0.10	0.07
0.25	0.15	0.10	0.05
0.11	0.27	0.07	0.09
0.12	0.18	0.10	0.18
0.04	0.06	0.11	0.07
0.32	0.02	0.07	0.16
0.14	0.10	0.08	0.09
0.14	0.10	0.05	0.17
0.01	0.05	0.08	0.01
0.07	0.10	0.10	0.11
0.20	0.09	0.09	0.12
0.23	0.07	0.09	0.07
0.09	0.20	0.15	0.17
0.12	0.20	0.14	0.20
0.06	0.14	0.13	0.08
0.06	0.19	0.17	0.13
0.02	0.10	0.09	0.20
0.18	0.13	0.06	0.19
0.27	0.17	0.06	0.08
0.02	0.15	0.09	0.19
0.04	0.13	0.13	0.16
0.09	0.19	0.11	0.12
0.15	0.09	0.12	0.18
0.04	0.16	0.13	0.14
0.47	0.12	0.09	0.05
0.03	0.05	0.17	0.05
0.02	0.08	0.17	0.08
0.16	0.10	0.21	0.09
0.19	0.09	0.27	0.08
0.00	0.00	0.00	0.00
0.00	0.00	0.00	0.00
0.00	0.00	0.00	0.00

34	35	36	37	38	39
B_33	*C_2*	*C_37*	*C_38*	*C_39*	*C_43*

0.61					
0.24	0.02				
0.10	-0.02	-0.12			
0.04	0.21	0.16	-0.30		
-0.03	0.25	-0.06	-0.33	0.55	
0.17	0.10	0.54	-0.31	0.18	0.09
0.37	0.52	0.11	0.03	-0.05	-0.08
0.21	0.39	0.12	-0.01	-0.02	0.02
0.21	0.25	0.16	-0.09	-0.09	-0.19
0.13	0.29	-0.17	-0.36	0.50	0.50
0.24	0.23	0.20	0.17	-0.03	-0.24
0.31	0.36	0.04	0.21	-0.19	-0.23
0.08	-0.06	0.11	-0.47	0.01	0.13
0.42	0.04	0.40	-0.19	0.06	-0.22
0.23	0.16	-0.02	-0.13	-0.10	0.03
0.23	0.00	0.43	-0.28	0.09	-0.06
-0.11	-0.09	0.07	-0.38	0.16	0.14
0.02	-0.03	0.09	-0.14	-0.02	-0.23
0.02	0.04	-0.14	0.07	-0.13	-0.29
0.08	0.12	0.22	-0.45	0.02	0.02
0.14	-0.19	0.30	0.32	-0.22	-0.41

-0.08	-0.09	0.00	-0.01	-0.17	-0.35
-0.23	-0.22	-0.15	-0.03	0.04	-0.19
-0.28	-0.19	-0.22	-0.08	0.02	0.38
0.06	0.00	0.50	-0.13	-0.03	-0.25
-0.09	-0.02	0.18	-0.37	-0.01	0.05
-0.14	0.00	0.11	-0.34	-0.07	0.05
-0.32	-0.22	-0.32	-0.07	0.01	0.32
0.06	0.08	-0.11	0.26	-0.30	-0.25
-0.26	-0.31	-0.05	0.12	-0.21	-0.27
-0.37	-0.28	-0.28	-0.15	-0.08	0.17
0.00	-0.07	-0.05	0.11	0.18	0.15
-0.14	0.07	-0.29	-0.17	0.28	0.46
-0.12	0.02	-0.09	-0.11	0.15	0.05
-0.08	0.04	-0.10	0.23	-0.26	-0.20
0.13	0.15	0.07	-0.31	-0.04	0.05
0.28	0.06	0.10	0.30	0.09	-0.11
-0.09	-0.10	-0.03	-0.14	0.30	0.14
-0.23	-0.13	-0.25	0.09	0.14	0.16
0.07	0.26	-0.01	-0.21	0.25	0.40
0.25	0.43	0.10	0.05	-0.09	-0.04
-0.21	-0.30	0.07	-0.14	0.17	0.17
-0.10	0.11	0.00	-0.38	0.45	0.34
-0.20	-0.22	-0.12	0.03	0.19	-0.14
-0.16	-0.04	-0.14	-0.44	0.10	0.44
-0.19	-0.19	-0.11	-0.45	0.00	0.22
0.01	-0.01	-0.12	-0.04	-0.37	-0.15
-0.20	-0.26	0.08	-0.44	-0.05	0.01
0.28	0.07	0.27	0.10	-0.07	-0.24
-0.14	-0.08	-0.11	-0.17	0.19	0.50
-0.24	-0.18	-0.24	-0.02	0.02	0.31
0.23	0.19	0.21	-0.25	-0.03	0.08
0.11	-0.10	0.34	-0.47	0.10	0.07
-0.04	-0.10	0.20	-0.13	-0.07	-0.10
-0.02	-0.11	0.06	-0.37	0.40	0.34
0.00	-0.01	0.33	-0.33	-0.08	0.00
-0.29	-0.31	-0.17	-0.13	0.12	0.23
0.12	-0.05	0.16	0.03	0.24	-0.08
0.23	-0.08	0.36	0.00	-0.14	-0.38
-0.28	-0.24	-0.28	-0.25	0.07	0.23
-0.19	0.08	-0.17	-0.25	0.32	0.30

32	33	34
134	135	136
CACGCTCATGCACACACCCACA	TCGACCGGACCTCGACCGGCT	AACATTCATTGTTGTCGGTGGGT
miR-574-3p	miR-1307-5p	miR-181d
10799.00	10674.00	10591.00
0.38	1.73	-0.30
0.14	-0.86	-2.01
-0.52	0.40	-1.01
-0.57	0.00	-1.41
4.45	-0.86	0.11
-0.33	-0.40	1.20
0.07	-0.19	2.09
-0.43	-0.55	-0.45
-0.33	-0.66	-1.03
0.30	-0.62	0.06
-0.62	-0.47	0.61
-0.95	-0.62	-0.61
0.59	3.76	1.09
-0.28	-0.65	-0.26
-0.22	-0.33	0.37
-0.35	0.91	1.71
0.70	-0.24	-0.03
0.99	0.06	-0.15
-0.33	-0.28	0.60
-0.38	1.46	-0.52

-0.58	-0.45	0.10
0.03	-0.37	0.32
-0.04	-0.33	0.17
-0.17	-0.24	-0.18
-0.36	-0.71	-1.77
-0.30	-0.23	1.62
-0.88	0.75	-0.34
-0.70	3.75	-0.93
-0.58	1.44	-0.84
-0.79	-0.50	-0.68
-0.30	-0.86	-1.24
-0.53	-0.53	-0.22
2.24	-0.46	-0.78
0.15	-0.46	0.36
0.16	-0.37	1.97
0.15	-0.15	0.06
-0.73	-0.82	-1.54
0.70	-0.36	1.42
0.21	0.89	3.90
-0.47	-0.29	0.55
6.01	3.68	0.53
14.50	23.08	7.55
-0.39	-0.43	1.04
0.80	0.57	1.99
-0.48	-0.62	-0.13
5.29	1.27	-0.09
0.28	1.23	1.16
-0.08	0.27	0.47
-0.21	-0.12	-0.52
-0.19	-0.02	0.01
-0.19	0.51	-0.03
0.58	-0.17	0.94
0.41	-0.57	-0.12
0.55	0.04	1.11
-0.55	-0.28	-0.29
0.13	-0.60	-0.41
-0.35	-0.13	-0.11
-0.92	3.36	0.22
-0.48	-0.40	0.17
-0.35	-0.21	0.76
-0.12	-0.54	0.10
-0.84	1.84	-0.88
-0.38	-0.34	-0.99
-0.90	0.08	-1.26
-0.84	0.00	-0.81
-0.82	-0.26	1.10
-0.30	2.98	-1.15
0.82	-0.86	-2.01
0.27	-0.36	-0.96
0.46	1.70	0.96
-0.39	-0.23	-0.43
-0.61	-0.36	-1.87
-0.56	-0.31	-0.68
-0.49	-0.14	-0.02
8.27	12.56	2.86
-0.51	0.48	0.80
-0.76	-0.53	0.99
-0.71	-0.72	-0.24
0.35	2.73	0.97

0.09	0.56	0.70
0.60	-0.32	-0.37
-0.01	0.20	0.20
-0.53	-0.08	-0.06
-0.14	1.18	0.33
-0.74	3.22	-1.15
0.77	0.05	0.29
-0.12	0.87	0.51
-0.32	-0.14	-0.55
-0.09	0.31	0.42
0.58	-0.15	0.01
-0.87	-0.22	0.57
-0.48	-0.40	0.00
0.22	0.54	0.19
-0.84	-0.11	0.01
-0.31	0.69	2.46
0.11	-1.40	-1.59
0.00	0.00	0.00
1.00	1.00	1.00
0.42	0.90	0.38

134	135	136
CACGCTCATGCACACACCCACA	TCGACCGGACCTCGACCGGCT	AACATTCATTGTTGTCGGTGGGT
miR-574-3p	miR-1307-5p	miR-181d
10799	10674	10591
0.38	1.73	-0.30
0.14	-0.86	-2.01
-0.52	0.40	-1.01
-0.57	0.00	-1.41
4.45	-0.86	0.11
-0.33	-0.40	1.20
0.07	-0.19	2.09
-0.43	-0.55	-0.45
-0.33	-0.66	-1.03
0.30	-0.62	0.06
-0.62	-0.47	0.61
-0.95	-0.62	-0.61
0.59	3.76	1.09
-0.28	-0.65	-0.26
-0.22	-0.33	0.37
-0.35	0.91	1.71
0.70	-0.24	-0.03
0.99	0.06	-0.15
-0.33	-0.28	0.60
-0.38	1.46	-0.52
-0.58	-0.45	0.10
0.03	-0.37	0.32
-0.04	-0.33	0.17
-0.17	-0.24	-0.18
-0.36	-0.71	-1.77
-0.30	-0.23	1.62
-0.88	0.75	-0.34
-0.70	3.75	-0.93

-0.58	1.44	-0.84
-0.79	-0.50	-0.68
-0.30	-0.86	-1.24
-0.53	-0.53	-0.22
2.24	-0.46	-0.78
0.15	-0.46	0.36
0.16	-0.37	1.97
0.15	-0.15	0.06
-0.73	-0.82	-1.54
0.70	-0.36	1.42
0.21	0.89	3.90
-0.47	-0.29	0.55
6.01	3.68	0.53
14.50	23.08	7.55
-0.39	-0.43	1.04
0.80	0.57	1.99
-0.48	-0.62	-0.13
5.29	1.27	-0.09
0.28	1.23	1.16
-0.08	0.27	0.47
-0.21	-0.12	-0.52
-0.19	-0.02	0.01
-0.19	0.51	-0.03
0.58	-0.17	0.94
0.41	-0.57	-0.12
0.55	0.04	1.11
-0.55	-0.28	-0.29
0.13	-0.60	-0.41
-0.35	-0.13	-0.11
-0.92	3.36	0.22
-0.48	-0.40	0.17
-0.35	-0.21	0.76
-0.12	-0.54	0.10
-0.84	1.84	-0.88
-0.38	-0.34	-0.99
-0.90	0.08	-1.26
-0.84	0.00	-0.81
-0.82	-0.26	1.10
-0.30	2.98	-1.15
0.82	-0.86	-2.01
0.27	-0.36	-0.96
0.46	1.70	0.96
-0.39	-0.23	-0.43
-0.61	-0.36	-1.87
-0.56	-0.31	-0.68
-0.49	-0.14	-0.02
8.27	12.56	2.86
-0.51	0.48	0.80
-0.76	-0.53	0.99
-0.71	-0.72	-0.24
0.35	2.73	0.97
0.09	0.56	0.70
0.60	-0.32	-0.37
-0.01	0.20	0.20
-0.53	-0.08	-0.06
-0.14	1.18	0.33
-0.74	3.22	-1.15
0.77	0.05	0.29
-0.12	0.87	0.51

-0.32	-0.14	-0.55
-0.09	0.31	0.42
0.58	-0.15	0.01
-0.87	-0.22	0.57
-0.48	-0.40	0.00
0.22	0.54	0.19
-0.84	-0.11	0.01
-0.31	0.69	2.46

134	135	136
69	53	113
TGGCTCAGTTCAGCAGGAACAG	AATGGCGCCACTAGGGTTGTG	GTCCAGTTTTCCCAGGAATCCCT
miR-24-3p	miR-652-3p	miR-145-5p
78156	143334	20235
0.04	2.20	1.29
0.17	0.05	-1.36
-0.36	-0.25	-0.50
-0.38	-0.49	-0.60
1.53	1.26	-0.24
-1.10	-0.85	-1.07
-0.71	-0.38	-0.22
-0.94	-0.64	0.54
-1.07	-1.34	-0.77
-0.09	0.17	-0.92
-0.03	-1.24	-0.71
-0.96	-2.15	-0.88
3.38	0.09	1.49
-0.73	-0.53	0.50
0.21	0.56	-0.06
0.40	0.05	-0.32
0.11	1.12	0.83
0.28	1.82	0.22
0.76	0.80	2.10
2.03	-1.00	-0.52
-0.57	-0.70	-0.99
-0.22	-0.56	-0.01
-0.34	0.49	-0.08
-0.44	1.04	-0.75
-0.65	0.41	0.51
0.39	0.72	2.78
-0.69	-0.61	-0.27
-0.10	-1.78	-0.80
-0.30	-0.81	-0.30
-0.30	-0.29	-0.32
-1.09	1.85	-0.88
0.01	0.14	-0.97
1.63	0.63	0.19
0.33	0.02	2.55
3.37	3.90	8.36
-0.04	-1.21	0.31
-1.41	-1.77	-1.19
0.73	-0.06	1.23
2.86	1.03	2.63

0.14	-1.03	0.24
4.04	8.39	9.01
9.21	28.19	18.44
0.42	0.57	1.13
2.26	2.20	1.89
-0.96	-0.32	1.54
0.38	6.32	1.13
2.15	-0.61	0.21
0.20	-0.23	-0.04
0.64	-0.12	0.07
0.68	-0.21	-0.42
0.94	-0.32	-0.45
0.19	0.57	0.23
0.13	0.90	0.17
2.62	0.80	1.06
-0.87	-1.33	-1.05
-0.15	0.42	-0.07
-0.60	0.02	-0.72
0.66	-1.10	-0.81
-0.36	0.24	1.63
0.29	1.20	1.23
1.73	0.66	1.52
-0.75	-1.50	-0.70
-0.96	0.44	0.89
-0.93	-0.76	-0.49
-0.34	-0.83	-0.63
-0.29	-1.46	-0.36
0.11	1.19	-0.18
-0.34	3.19	-0.55
0.53	0.96	1.38
2.33	0.63	1.99
-0.55	-1.14	-0.03
-0.27	-1.92	-0.52
-0.29	-0.82	-0.88
4.27	-0.19	0.83
0.26	36.59	23.97
-0.40	-0.72	-0.61
0.44	0.87	0.80
-1.22	-1.49	-1.03
2.70	0.12	-0.37
1.54	-0.83	-0.53
-0.27	0.69	-0.85
0.54	-0.49	-0.57
-0.31	-1.72	0.21
2.41	-0.81	0.03
0.95	-1.72	-1.15
0.53	-0.60	1.63
1.32	-0.82	-0.04
0.13	-0.57	-0.46
1.14	-1.06	-0.10
1.19	-0.28	0.54
0.11	-1.49	-1.14
-0.67	-1.06	-0.44
0.23	-0.53	-0.13
-0.64	-0.60	-0.40
-0.57	2.02	1.03
3.04	3.88	5.81
0.82	0.99	0.53

P	UC
25.42	25.27
its	
P	UC
K-S	K-S
0.02	0.02
A-D	A-D
0.20	0.20
alpha1	alpha1
9.99	13.46
alpha2	alpha2
#####	#####
a	a
3.21	0.01
b	b
#####	#####
of ave	
0.54	0.51
ed aves	
25.42	25.27
of observed ave	
0.54	0.51
0.46	0.49
P	UC
o relabel but reselect	
33.67	18.85
of observed ave	
0.89	0.15
0.11	0.85

reselects		
typica	typica	typical P
43.19	33.63	####

reselects		
UC	NP	P
37	33	19
43	28	18
42	30	19
43	35	19
38	28	18
40	30	18
43	32	18
43	28	19
44	25	18
40	32	17
43	31	15
43	40	20
40	29	14
44	29	18
43	34	20
41	33	19
42	33	16
42	28	18
42	31	19
37	31	19
40	34	17
39	34	21
41	33	19
44	33	22
42	37	17
45	27	18
41	36	14
44	28	18
43	32	21
36	37	18
41	30	18
44	33	18
44	24	21
42	35	14
42	32	19
40	39	19
44	40	17
40	41	23
43	27	21
41	37	21
39	32	20
39	32	17
41	35	16
43	38	17
42	31	18
41	29	17
41	34	15
42	37	15
41	35	16
40	38	20
39	29	19

The Wilcoxon Rank-Sum Test ('

100 trials

	UCtru	UCpseudo
Obser	100	100
Mean:	43	25
Media	43	25
Stand	1	7

The Test Procedure

Hyp 0
Nb. 33
Ave 6
Ran ####
Ran 101
Tes ####
Nom 0

Two-Tailed Test (Normal Appr

Cri -1.95996398454005 and 1
Dec Reject H0 if |Z-Stat| >
Z S 12
Z S 12
Z S 12
P-V 0
Fin The Null Hypothesis Mus

41	32	15
41	32	21
41	36	16
43	33	17
40	29	12
42	34	15
43	26	17
39	35	17
44	29	20
43	28	17
40	32	18
41	36	17
42	32	15
42	35	19
44	29	19
39	35	16
41	32	19
41	38	22
42	32	14
41	29	17
40	39	21
39	38	17
42	32	14
43	29	21
44	35	19
43	29	20
43	30	19
40	34	17
42	29	16
42	32	17
41	26	18
44	31	19
42	30	17
43	37	18
42	27	19
43	33	20
43	33	18
42	36	21
43	33	20
41	27	21
43	28	17
37	36	18
43	34	16
43	29	23
43	30	21
41	34	20
38	38	17
38	31	20
42	27	17
42	34	20
43	36	16
44	35	21
45	34	18
42	26	18
42	32	15
39	25	17
41	30	20
43	28	17
42	29	17

43	27	17
42	33	20
44	32	15
42	39	19
44	26	18
42	30	18
43	41	20
43	26	17
42	29	19
41	33	20
42	38	18
40	30	19
42	33	17
38	28	23
40	30	14
40	39	17
43	29	17
42	29	18
42	35	17
41	27	18
41	33	18
39	34	17
42	40	19
42	27	19
42	33	15
41	31	19
41	37	17
43	32	19
43	34	18
40	33	17
43	32	16
42	34	16
43	35	20
43	31	18
44	29	18
44	31	16
45	32	18
43	33	16
36	35	19
44	29	19
45	27	14
43	33	16
39	31	16
43	30	20
44	34	20
41	31	20
43	31	18
42	28	17
41	36	18
39	33	15
38	32	17
41	29	16
42	32	15
42	37	16
43	34	19
44	34	19
41	30	16
42	35	16
42	27	18

45	33	21
38	31	15
44	30	18
42	37	15
42	31	15
41	31	15
43	32	16
42	27	16
38	34	18
42	35	19
44	28	20
42	30	19
44	34	17
44	30	16
43	38	17
41	35	19
38	41	15
41	33	17
40	28	18
43	32	21
39	30	17
40	35	20
42	29	19
41	35	19
42	28	16
41	33	13
43	32	21
38	32	19
44	37	18
43	28	17
42	34	15
41	29	15
44	27	17
42	30	15
45	38	16
38	31	14
41	26	20
42	33	22
41	31	18
42	32	17
42	27	19
39	31	18
43	33	19
44	33	18
40	36	17
43	30	18
44	31	14
38	39	23
43	28	19
45	38	20
42	34	22
42	30	14
42	29	17
43	34	19
43	32	19
42	32	20
41	31	19
42	31	19
44	32	16

41	32	17
42	28	19
42	36	15
41	31	13
42	35	16
43	35	19
41	34	19
41	32	16
43	34	15
42	28	19
42	29	20
42	30	14
44	48	18
42	32	17
40	32	20
42	32	20
44	29	19
43	33	15
40	39	19
41	23	18
43	31	19
42	31	17
42	35	15
42	39	17
43	33	18
41	29	17
43	30	20
41	35	18
43	30	17
43	28	15
44	33	16
45	28	18
43	31	16
42	33	17
43	27	16
38	27	17
42	34	20
43	32	16
42	31	19
40	32	14
42	35	16
42	35	21
41	37	21
43	28	19
44	32	17
44	38	22
40	33	18
45	27	16
44	28	18
44	30	20
44	29	16
43	33	19
40	34	20
44	30	20
44	36	17
42	35	19
44	27	18
41	38	18
41	34	22

45	31	16
42	27	17
42	30	19
37	35	20
43	29	16
42	35	15
42	26	21
42	33	18
42	33	20
40	34	18
43	27	16
42	31	19
42	37	20
42	35	20
44	28	20
43	27	20
43	32	16
44	36	16
41	26	18
43	34	14
44	27	19
42	30	15
39	32	18
42	25	16
44	25	19
39	34	14
39	35	20
40	35	18
40	35	18
41	31	19
44	23	18
44	27	14
42	36	15
40	32	20
42	32	16
38	27	17
42	33	17
43	31	20
46	25	15
42	34	17
42	34	18
42	34	17
44	30	16
41	34	15
41	28	23
42	32	14
44	31	14
42	30	17
44	30	18
42	34	19
38	34	17
43	38	17
43	32	19
42	32	17
43	32	17
43	33	20
43	35	16
42	37	16
41	35	18

45	41	21
45	31	17
43	41	17
43	29	20
43	31	16
44	25	17
37	33	16
44	30	18
42	30	13
41	33	19
43	35	22
42	33	17
45	32	17
41	32	20
44	32	16
41	35	19
43	34	21
38	39	24
43	39	17
41	34	22
43	33	16
42	27	17
41	33	17
43	30	18
43	28	19
42	36	18
43	29	16
45	27	18
39	31	18
43	32	20
46	30	17
38	37	20
41	27	15
40	31	17
44	36	18
39	29	17
39	26	17
43	34	20
42	33	18
44	36	20
41	32	20
43	28	19
45	26	18
40	33	15
41	29	18
43	27	16
41	32	19
41	33	19
42	34	15
43	34	17
44	34	19
38	32	19
42	32	18
41	30	19
41	33	15
42	37	16
42	32	19
43	28	19
41	34	15

37	33	19
43	36	17
38	30	20
43	32	19
39	30	17
41	28	17
41	33	18
38	33	16
42	35	16
43	30	20
41	27	17
41	39	15
42	31	19
43	31	17
42	34	19
41	32	17
43	35	21
44	30	17
43	31	19
41	25	18
42	38	17
39	32	19
40	35	18
40	29	16
40	34	16
41	30	18
42	34	18
40	31	19
40	34	18
45	35	18
43	31	19
43	31	23
42	33	17
42	36	20
42	36	15
42	23	17
42	35	19
42	28	16
42	31	17
41	29	15
43	25	16
44	34	20
42	32	20
41	36	17
40	41	21
43	30	19
43	34	19
42	35	21
43	36	20
44	27	17
43	31	22
41	32	17
42	36	20
41	27	17
41	36	18
43	28	15
44	32	20
45	36	14
41	25	15

42	31	20
41	34	19
43	34	20
42	30	17
42	32	18
41	31	17
43	30	18
44	30	14
40	34	13
39	34	19
44	28	21
38	35	20
39	32	18
42	31	17
43	35	17
41	30	19
42	34	15
42	34	18
42	30	18
42	38	15
43	30	18
40	36	14
43	32	19
44	31	19
42	33	19
41	36	18
43	30	20
44	33	18
39	33	16
45	33	17
42	31	15
45	30	20
41	30	19
43	34	16
43	28	20
42	27	14
43	36	16
42	30	18
44	38	18
39	34	20
38	40	16
44	37	17
45	27	19
43	29	22
45	35	18
40	29	19
42	39	15
43	37	17
42	28	16
44	32	18
44	32	16
43	35	20
44	39	19
45	30	21
44	33	17
43	20	20
44	35	17
42	28	15
45	35	17

45	32	15
42	36	14
40	31	15
40	34	19
44	29	19
39	31	17
42	29	15
41	39	17
43	34	17
44	29	16
45	33	15
41	30	16
38	33	20
44	29	15
42	31	18
40	27	20
44	30	16
42	34	17
42	35	18
42	34	20
38	35	19
42	32	17
43	34	19
43	30	16
41	27	16
40	27	12
41	37	19
41	35	16
42	33	21
42	34	16
38	34	20
41	28	19
44	33	16
40	31	16
44	32	17
42	36	18
44	32	17
43	39	19
44	28	19
42	33	19
43	31	15
44	32	16
43	33	17
45	34	15
41	32	21
38	32	19
40	29	22
44	27	17
42	28	18
43	32	16
41	34	15
43	32	15
41	26	18
41	32	15
42	27	16
43	25	19
41	30	16
43	32	15

41	32	18
43	33	17
44	27	16
41	33	15
44	26	20
43	35	18
41	38	20
38	31	19
41	36	21
44	35	18
45	35	16
44	29	19
42	29	20
44	32	17
41	32	16
45	27	20
42	33	18
42	37	16
44	40	19
43	31	19
41	33	14
42	29	17
38	36	18
41	25	17
43	29	18
41	28	18
41	27	17
37	36	20
42	33	15
39	33	14
42	37	19
43	28	17
42	32	18
41	31	18
44	33	18
42	32	19
42	31	20
41	37	19
40	30	19
44	30	17
38	33	17
38	33	19
43	36	17
45	31	17
43	31	18
41	39	21
43	32	17
42	29	16
44	29	18
40	29	15
41	33	19
42	29	15
41	30	17
41	28	17
41	28	15
37	40	17
41	35	16
43	30	15
43	32	16

43	23	17
44	32	20
42	29	18
40	33	20
42	29	18
43	32	17
43	27	22
39	35	17
42	36	17
42	38	16
41	39	16
41	23	22
41	32	19
40	34	18
39	31	20
41	33	20
38	33	19
43	35	19
41	30	24
42	41	19
43	33	20
43	32	21
40	37	21
43	40	21
42	40	21
39	27	18
41	29	17
42	38	18
43	34	18
43	28	19
44	32	20
42	30	18
44	32	18
40	33	15
43	35	19
44	32	16
43	30	19
41	27	20
44	26	16
43	28	14
42	36	16
43	28	21
42	32	19
40	32	14
43	36	18
42	29	16
39	35	16
43	30	20
38	35	16
44	30	17
38	30	21
42	29	19
41	35	18
41	34	20
40	41	20
39	36	20
44	30	18
43	36	16
45	33	19

42	35	20
43	30	19
42	33	18
39	32	16
42	28	19
44	33	19
38	32	17
44	32	17
43	36	19
43	30	21
41	26	17
41	28	17
39	30	15
45	37	18
45	31	18
43	38	19
44	42	16
43	30	14
43	30	15
42	28	19
40	31	16
43	32	18
41	38	19
43	33	16
42	31	18
43	33	20
40	26	18
42	36	16
42	28	15
44	38	19
42	27	17
43	31	16
42	31	18
41	29	21
43	35	16
44	30	16
40	38	20
43	31	17
44	33	17
41	30	18
42	31	22
43	29	20
43	30	19
43	31	19
44	33	19
44	37	18
41	33	22
44	37	21
43	34	15
44	27	16
45	27	16
44	30	20
43	36	18
40	37	19
41	33	21
41	33	18
41	27	17
43	30	19
42	25	14

42	31	16
43	34	21
43	37	16
42	25	21
42	36	20
42	30	12
44	40	17
43	28	22
42	29	18
39	29	17
41	36	18
44	33	18
41	28	22
42	37	19
43	24	19
43	33	19
40	37	19
40	37	19
40	31	17
44	34	16
42	34	18
42	38	18
43	36	18
45	31	19
45	29	19
41	35	17
43	32	19
42	25	18
42	36	17
39	33	18
42	31	18
41	35	20
42	31	20
43	32	19
42	31	18
44	30	18
43	34	21
46	27	14
42	30	20
43	29	21
43	30	19
43	32	17
39	30	18
41	35	17
41	30	20
42	36	21
43	25	19
40	34	18
42	34	21
42	34	21
39	32	23
44	28	21
43	33	19
43	34	13
42	33	17
39	32	16
45	37	20
43	36	20
43	40	19

42	31	16
42	31	19
42	34	20
44	26	17
40	31	16
44	40	18
41	35	19
42	34	17
42	33	16
41	34	21
39	30	16
41	28	16
42	29	17
42	33	18
42	32	19
42	27	17
42	29	15
42	35	20
41	28	16
43	28	14
41	36	17
43	42	20
43	34	20
41	34	15
42	36	20
40	30	20
41	29	14
45	26	19
43	27	20
44	28	19
41	31	19
42	28	19
41	40	17
42	41	18
42	31	17
38	31	17
40	28	16
43	31	19
42	32	15
42	31	19
45	27	14
42	31	17
42	31	20
43	31	19
42	31	17
43	24	17
45	36	17
43	35	21
44	27	19
42	27	17
41	31	13
43	28	16
40	36	19
44	38	17
42	29	19
43	29	16
42	32	17
43	28	17
43	29	18

42	25	17
42	36	18
41	28	17
43	30	20
44	36	19
41	36	15
43	28	18
44	31	18
41	29	16
42	39	17
42	33	20
43	39	20
46	31	19
38	32	19
38	35	20
42	29	22
41	32	17
41	27	18
43	34	20
44	33	15
43	34	17
43	32	20
37	31	19
43	25	17
42	33	18
43	35	17
39	35	18
42	28	21
42	32	15
43	31	16
43	31	17
44	34	19
41	35	17
43	29	19
40	34	19
43	21	19
42	26	14
45	33	18
45	30	19
43	31	21
42	22	18
42	28	19
43	38	17
43	36	16
43	30	18
41	37	20
44	30	16
41	29	17
43	37	17
43	30	18
43	29	18
42	30	20
38	26	19
42	31	16
44	33	19
44	38	19
40	32	16
39	28	23
43	34	19

42	31	21
42	28	21
41	31	17
43	31	16
43	32	19
39	31	20
41	30	15
41	29	18
42	25	19
43	33	21
44	30	18
42	33	21
44	32	19
45	38	20
43	26	16
41	31	18
41	34	13
42	37	17
42	30	18
44	35	19
42	31	18
44	25	14
41	26	19
42	31	19
40	32	18
44	27	18
45	29	19
41	35	18
45	27	17
42	38	17
40	25	17
42	30	19
43	36	16
43	32	15
42	29	16
41	38	24
42	30	17
42	31	19
42	31	16
44	31	19
43	27	23
44	27	20
42	30	13
40	36	17
39	37	16
43	27	16
43	27	17
42	31	16
43	26	19
42	30	16
42	35	20
44	31	20
42	31	16
44	34	20
40	32	16
42	31	16
40	35	20
43	32	20
42	31	14

42	33	15
44	36	19
42	32	20
42	32	18
43	32	22
40	34	16
38	32	17
42	34	16
43	36	20
41	31	16
42	27	24
41	33	17
42	38	18
43	29	20
43	30	19

44	27	20
42	34	16
38	34	20
41	28	19
44	33	16
40	31	16
44	32	17
42	36	18
44	32	17
43	39	19
44	28	19
42	33	19
43	31	15
44	32	16
43	33	17
45	34	15

41	32	21
38	32	19
40	29	22
44	27	17
42	28	18
43	32	16
41	34	15
43	32	15
41	26	18
41	32	15
42	27	16
43	25	19
41	30	16
43	32	15
41	32	18
43	33	17
44	27	16
41	33	15
44	26	20
43	35	18
41	38	20
38	31	19
41	36	21
44	35	18
45	35	16
44	29	19
42	29	20
44	32	17
41	32	16
45	27	20
42	33	18
42	37	16
44	40	19
43	31	19
41	33	14
42	29	17
38	36	18
41	25	17
43	29	18
41	28	18
41	27	17
37	36	20
42	33	15
39	33	14
42	37	19
43	28	17
42	32	18
41	31	18
44	33	18
42	32	19
42	31	20
41	37	19
40	30	19
44	30	17
38	33	17
38	33	19
43	36	17
45	31	17
43	31	18

41	39	21
43	32	17
42	29	16
44	29	18
40	29	15
41	33	19
42	29	15
41	30	17
41	28	17
41	28	15
37	40	17
41	35	16
43	30	15
43	32	16
43	23	17
44	32	20
42	29	18
40	33	20
42	29	18
43	32	17
43	27	22
39	35	17
42	36	17
42	38	16
41	39	16
41	23	22
41	32	19
40	34	18
39	31	20
41	33	20
38	33	19
43	35	19
41	30	24
42	41	19
43	33	20
43	32	21
40	37	21
43	40	21
42	40	21
39	27	18
41	29	17
42	38	18
43	34	18
43	28	19
44	32	20
42	30	18
44	32	18
40	33	15
43	35	19
44	32	16
43	30	19
41	27	20
44	26	16
43	28	14
42	36	16
43	28	21
42	32	19
40	32	14
43	36	18

42	29	16
39	35	16
43	30	20
38	35	16
44	30	17
38	30	21
42	29	19
41	35	18
41	34	20
40	41	20
39	36	20
44	30	18
43	36	16
45	33	19
42	35	20
43	30	19
42	33	18
39	32	16
42	28	19
44	33	19
38	32	17
44	32	17
43	36	19
43	30	21
41	26	17
41	28	17
39	30	15
45	37	18
45	31	18
43	38	19
44	42	16
43	30	14
43	30	15
42	28	19
40	31	16
43	32	18
41	38	19
43	33	16
42	31	18
43	33	20
40	26	18
42	36	16
42	28	15
44	38	19
42	27	17
43	31	16
42	31	18
41	29	21
43	35	16
44	30	16
40	38	20
43	31	17
44	33	17
41	30	18
42	31	22
43	29	20
43	30	19
43	31	19
44	33	19

44	37	18
41	33	22
44	37	21
43	34	15
44	27	16
45	27	16
44	30	20
43	36	18
40	37	19
41	33	21
41	33	18
41	27	17
43	30	19
42	25	14
42	31	16
43	34	21
43	37	16
42	25	21
42	36	20
42	30	12
44	40	17
43	28	22
42	29	18
39	29	17
41	36	18
44	33	18
41	28	22
42	37	19
43	24	19
43	33	19
40	37	19
40	37	19
40	31	17
44	34	16
42	34	18
42	38	18
43	36	18
45	31	19
45	29	19
41	35	17
43	32	19
42	25	18
42	36	17
39	33	18
42	31	18
41	35	20
42	31	20
43	32	19
42	31	18
44	30	18
43	34	21
46	27	14
42	30	20
43	29	21
43	30	19
43	32	17
39	30	18
41	35	17
41	30	20

42	36	21
43	25	19
40	34	18
42	34	21
42	34	21
39	32	23
44	28	21
43	33	19
43	34	13
42	33	17
39	32	16
45	37	20
43	36	20
43	40	19
42	31	16
42	31	19
42	34	20
44	26	17
40	31	16
44	40	18
41	35	19
42	34	17
42	33	16
41	34	21
39	30	16
41	28	16
42	29	17
42	33	18
42	32	19
42	27	17
42	29	15
42	35	20
41	28	16
43	28	14
41	36	17
43	42	20
43	34	20
41	34	15
42	36	20
40	30	20
41	29	14
45	26	19
43	27	20
44	28	19
41	31	19
42	28	19
41	40	17
42	41	18
42	31	17
38	31	17
40	28	16
43	31	19
42	32	15
42	31	19
45	27	14
42	31	17
42	31	20
43	31	19
42	31	17

43	24	17
45	36	17
43	35	21
44	27	19
42	27	17
41	31	13
43	28	16
40	36	19
44	38	17
42	29	19
43	29	16
42	32	17
43	28	17
43	29	18
42	25	17
42	36	18
41	28	17
43	30	20
44	36	19
41	36	15
43	28	18
44	31	18
41	29	16
42	39	17
42	33	20
43	39	20
46	31	19
38	32	19
38	35	20
42	29	22
41	32	17
41	27	18
43	34	20
44	33	15
43	34	17
43	32	20
37	31	19
43	25	17
42	33	18
43	35	17
39	35	18
42	28	21
42	32	15
43	31	16
43	31	17
44	34	19
41	35	17
43	29	19
40	34	19
43	21	19
42	26	14
45	33	18
45	30	19
43	31	21
42	22	18
42	28	19
43	38	17
43	36	16
43	30	18

41	37	20
44	30	16
41	29	17
43	37	17
43	30	18
43	29	18
42	30	20
38	26	19
42	31	16
44	33	19
44	38	19
40	32	16
39	28	23
43	34	19
42	31	21
42	28	21
41	31	17
43	31	16
43	32	19
39	31	20
41	30	15
41	29	18
42	25	19
43	33	21
44	30	18
42	33	21
44	32	19
45	38	20
43	26	16
41	31	18
41	34	13
42	37	17
42	30	18
44	35	19
42	31	18
44	25	14
41	26	19
42	31	19
40	32	18
44	27	18
45	29	19
41	35	18
45	27	17
42	38	17
40	25	17
42	30	19
43	36	16
43	32	15
42	29	16
41	38	24
42	30	17
42	31	19
42	31	16
44	31	19
43	27	23
44	27	20
42	30	13
40	36	17
39	37	16

43	27	16
43	27	17
42	31	16
43	26	19
42	30	16
42	35	20
44	31	20
42	31	16
44	34	20
40	32	16
42	31	16
40	35	20
43	32	20
42	31	14
42	33	15
44	36	19
42	32	20
42	32	18
43	32	22
40	34	16
38	32	17
42	34	16
43	36	20
41	31	16
42	27	24
41	33	17
42	38	18
43	29	20
43	30	19

:TCAGC

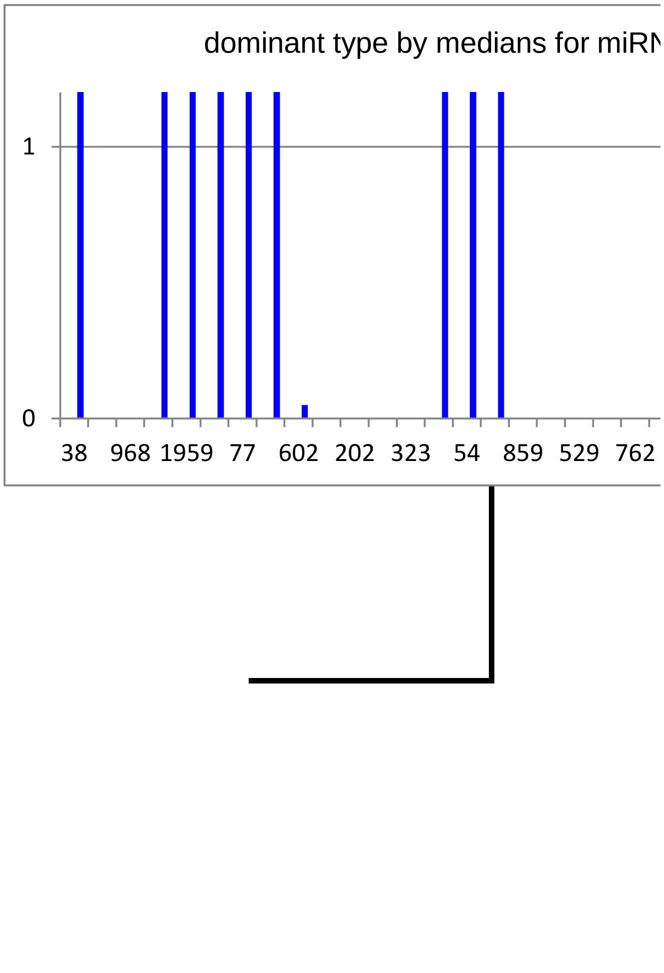

dominant type by medians for miRN
1
0
38
968
1959
77
602
202
323
54
859
529
762

40	41	42	43	44	45	46	47	48	49	50	51	52	53	54	55
C_48	*D_10*	*D_12*	*D_13*	*D_14*	*D_3*	*D_9*	*E_20*	*E_29*	*E_4*	*E_5*	*E_6*	*F_1*	*F_35*	*F_36*	*F_37*

0.03														
0.03	0.88													
0.09	0.49	0.46												
-0.03	0.03	-0.01	-0.11											
0.40	0.22	0.14	0.29	-0.10										
-0.01	0.89	0.80	0.44	-0.09	0.29									
0.34	-0.13	-0.06	0.01	0.24	-0.15	-0.24								
0.44	0.17	0.16	0.42	-0.06	0.40	0.16	0.36							
0.18	-0.02	0.02	0.06	0.11	0.07	-0.02	0.44	0.34						
0.61	0.01	0.04	0.15	-0.04	0.18	-0.01	0.54	0.64	0.52					
0.32	-0.11	-0.03	0.18	0.07	0.07	-0.19	0.49	0.45	0.55	0.59				
-0.15	0.27	0.24	0.62	-0.19	0.01	0.19	0.07	0.38	-0.17	0.13	0.21			
-0.46	0.23	0.15	0.51	-0.18	-0.12	0.15	-0.12	0.14	-0.21	-0.15	-0.01	0.83		
0.12	0.14	0.16	0.36	0.29	-0.09	-0.01	0.53	0.33	0.36	0.35	0.37	0.30	0.22	
-0.04	-0.02	0.00	0.24	-0.25	0.37	0.06	-0.01	0.47	0.05	0.09	0.07	0.23	0.27	0.16

-0.15	0.21	0.16	0.50	-0.24	0.03	0.17	0.03	0.20	-0.20	0.11	0.07	0.76	0.78	0.18	0.21
-0.34	-0.08	-0.04	0.26	-0.12	-0.14	-0.10	0.01	0.13	-0.08	0.02	0.33	0.69	0.66	0.18	0.19
-0.15	-0.25	-0.21	-0.37	0.20	-0.30	-0.24	0.05	-0.39	0.01	-0.18	0.03	-0.34	-0.31	-0.20	-0.29
0.46	0.15	0.10	0.28	-0.33	0.32	0.16	0.04	0.42	-0.13	0.31	0.08	0.28	0.03	0.14	0.18
0.30	-0.14	-0.08	-0.02	0.04	0.01	-0.17	0.44	0.24	0.21	0.19	0.27	-0.04	-0.23	0.47	0.08
0.29	-0.13	-0.10	-0.09	-0.02	-0.09	-0.16	0.41	0.01	0.11	0.17	0.22	-0.08	-0.24	0.41	-0.15
-0.27	-0.28	-0.25	-0.44	0.17	-0.33	-0.27	0.02	-0.45	0.00	-0.25	0.00	-0.34	-0.26	-0.23	-0.32
-0.08	0.21	0.09	0.14	-0.20	0.06	0.26	-0.16	-0.07	-0.05	-0.08	-0.26	0.02	0.21	-0.02	0.05
-0.13	-0.02	-0.04	0.03	-0.18	-0.13	0.05	0.00	-0.03	-0.13	-0.05	-0.07	0.14	0.19	0.11	0.15
-0.18	-0.28	-0.23	-0.30	0.22	-0.50	-0.28	0.20	-0.37	0.01	-0.18	0.01	-0.29	-0.13	-0.01	-0.28
0.04	-0.12	-0.10	-0.19	0.12	0.13	-0.10	-0.26	0.00	-0.09	-0.16	-0.11	-0.28	-0.21	-0.25	0.08
-0.36	-0.14	-0.06	-0.31	0.39	-0.39	-0.19	0.07	-0.46	0.11	-0.21	0.09	-0.22	-0.13	-0.08	-0.34
-0.14	-0.06	-0.07	-0.14	0.09	0.11	-0.07	-0.14	-0.20	-0.28	-0.32	-0.26	-0.12	-0.08	-0.22	-0.12
-0.26	-0.03	-0.05	-0.11	-0.35	-0.25	-0.05	-0.32	-0.17	-0.06	-0.24	-0.20	-0.03	0.17	-0.04	0.02
0.16	-0.07	-0.04	-0.03	0.01	-0.09	-0.20	0.28	0.21	0.32	0.20	0.29	0.03	-0.06	0.33	0.03
-0.21	-0.02	-0.05	0.05	0.01	0.11	-0.08	-0.22	0.08	0.05	-0.06	-0.06	0.11	0.22	-0.06	0.37
0.12	-0.23	-0.20	-0.34	0.30	0.16	-0.25	0.15	-0.05	0.14	0.01	0.20	-0.29	-0.34	-0.01	0.03
-0.29	-0.35	-0.27	-0.48	0.12	-0.36	-0.35	-0.07	-0.46	0.07	-0.25	-0.10	-0.40	-0.16	-0.13	-0.11
0.23	-0.16	-0.14	-0.34	0.23	-0.07	-0.26	0.16	-0.10	0.39	0.18	0.31	-0.37	-0.38	0.12	-0.29
0.02	0.87	0.96	0.43	-0.07	0.19	0.82	-0.13	0.10	0.01	0.02	-0.10	0.20	0.15	0.09	-0.03
0.09	-0.28	-0.18	-0.25	0.19	-0.14	-0.27	0.11	0.04	0.11	0.17	0.39	-0.07	-0.20	0.12	0.01
0.29	-0.15	-0.13	-0.19	0.41	0.24	-0.25	0.20	-0.06	-0.13	0.05	0.01	-0.16	-0.29	-0.06	-0.29
0.00	-0.18	-0.18	-0.12	-0.02	0.46	-0.13	-0.11	0.09	-0.34	-0.18	0.01	0.08	0.00	-0.29	0.16
-0.05	-0.10	-0.01	-0.16	0.32	-0.50	-0.27	0.56	-0.13	0.20	0.16	0.33	-0.03	-0.06	0.27	-0.36
0.14	-0.24	-0.14	-0.06	0.27	-0.31	-0.34	0.86	0.08	0.34	0.29	0.41	-0.02	-0.11	0.43	-0.18
-0.02	0.13	0.15	0.27	-0.05	-0.17	0.16	0.31	0.05	0.24	0.14	-0.04	0.05	0.14	0.12	-0.10
0.18	-0.15	-0.06	0.08	0.02	-0.25	-0.24	0.75	0.21	0.14	0.38	0.38	0.32	0.14	0.49	0.04
0.48	0.16	0.13	0.35	-0.13	0.56	0.26	0.11	0.58	0.46	0.57	0.33	0.07	-0.12	0.16	0.32
0.05	-0.22	-0.17	-0.36	0.22	-0.20	-0.25	0.20	-0.17	0.18	0.06	0.19	-0.30	-0.34	-0.06	-0.29
-0.20	-0.25	-0.22	-0.38	0.10	-0.27	-0.24	0.00	-0.36	0.02	-0.17	-0.02	-0.30	-0.23	-0.24	-0.24
0.45	0.21	0.22	0.33	0.11	0.22	0.10	0.65	0.43	0.58	0.56	0.49	0.07	-0.11	0.46	0.06
0.52	-0.10	-0.04	0.09	0.17	-0.04	-0.20	0.83	0.52	0.47	0.77	0.57	0.12	-0.14	0.55	0.04
0.14	-0.08	0.00	0.04	0.03	-0.16	-0.09	0.44	0.15	0.39	0.40	0.14	-0.07	-0.09	0.37	0.06
0.17	-0.30	-0.26	-0.40	0.52	0.00	-0.40	0.45	0.11	0.22	0.23	0.26	-0.23	-0.29	0.20	-0.01
0.38	-0.01	0.02	0.20	0.10	0.03	-0.12	0.69	0.26	0.37	0.50	0.37	0.08	0.02	0.64	0.18
-0.06	-0.34	-0.26	-0.43	0.16	-0.32	-0.34	0.01	-0.23	-0.20	-0.23	-0.11	-0.30	-0.26	-0.19	-0.19
0.05	0.03	-0.03	0.16	0.27	0.36	0.00	-0.05	0.23	-0.19	0.01	0.00	0.21	0.19	0.03	0.46
0.30	0.26	0.20	0.41	-0.29	0.28	0.25	0.06	0.64	-0.01	0.37	0.20	0.38	0.27	0.13	0.45
-0.16	-0.21	-0.18	-0.10	0.42	-0.33	-0.27	0.25	-0.23	-0.20	-0.30	-0.04	-0.06	0.01	0.05	-0.16
-0.20	0.14	0.14	0.17	0.35	-0.28	0.05	0.06	-0.21	-0.16	-0.21	0.04	0.21	0.22	0.21	-0.18

Two Independent Samples) | The Wilcoxon Rank-Sum Test (Two Independent Samples

INPUT DATA & RANKS | 1000 trials | INPUT DATA

RANKS | NPtru NPpseudo

	UCtru	UCpseuc	UCtrue	JCpseudo	Obser 1000 1000	NPtru
	44	**37**	166	***95.5***	Mean: 34 25	33
	44	**26**	166	60	Media 34 25	30
	43	**25**	140	53	Stand 4 7	43
	43	**19**	140	16	The Test Procedure	31
	44	**25**	166	53	Hyp 0	39
	45	**34**	189	91	Nb. 46	37
	44	**32**	166	86	Ave 43	31
	43	**26**	140	60	Ran ####	34
	45	**30**	189	83	Ran 1001	28
	42	**17**	117	10	Tes ####	37
	45	**28**	189	76	Nom 0	45
	45	**22**	189	33		34
oximation)	44	**38**	166	97	Two-Tailed Test (Normal Approximation)	32
..9599639845	43	**19**	140	16	Cri -1.95996398454005 and 1.95996398454005	31
› 1.95996398	41	**42**	105	117	Dec Reject H0 if |Z-Stat| > 1.95996398454005	38
	41	**27**	105	69	Z S 28	39
	43	**29**	140	80	Z S 28	41
	43	**22**	140	33	Z S 28	26
	42	**26**	117	60	P-V 0	29
st be Reject	44	**30**	166	83	Fin The Null Hypothesis Must be Rejected	28
	44	**26**	166	60		40
	43	**16**	140	6		32
	43	**28**	140	76		32
	42	**25**	117	53		33
	44	**23**	166	42		39
	43	**27**	140	69		34
	43	32	140	86		28
	44	27	166	69		35
	43	35	140	93		36
	43	33	140	88		36
	44	16	166	6		33
	43	23	140	42		36
	44	26	166	60		35
	45	52	189	200		27
	45	34	189	91		38
	42	27	117	69		30
	45	23	189	42		27
	43	16	140	6		37
	44	19	166	16		32
	41	13	105	1		31
	44	23	166	42		30
	42	33	117	88		40
	45	22	189	33		26
	43	25	140	53		32
	44	23	166	42		31
	42	34	117	91		33
	42	27	117	69		33
	44	27	166	69		34
	44	21	166	25		29

43	20	140	21	32
40	37	100	96	37
42	20	117	21	34
44	17	166	10	32
43	22	140	33	30
42	34	117	91	34
42	21	117	25	32
43	22	140	33	30
43	15	140	3	24
43	27	140	69	39
45	22	189	33	26
41	27	105	69	32
44	28	166	76	35
43	23	140	42	31
45	24	189	49	38
42	19	117	16	33
44	24	166	49	33
42	26	117	60	35
45	25	189	53	34
45	24	189	49	33
43	20	140	21	41
45	23	189	42	36
45	21	189	25	25
41	26	105	60	35
43	27	140	69	35
45	29	189	80	37
44	14	166	2	31
40	30	100	83	35
43	21	140	25	33
43	24	140	49	35
45	29	189	80	31
44	27	166	69	33
43	22	140	33	34
44	18	166	12	30
44	23	166	42	35
42	23	117	42	33
43	26	140	60	28
42	22	117	33	33
43	21	140	25	24
42	19	117	16	30
41	16	105	6	38
41	18	105	12	37
44	20	166	21	27
45	29	189	80	36
45	39	189	98	26
45	16	189	6	34
40	18	100	12	38
45	28	189	76	30
45	22	189	33	30
45	36	189	94	41
42	22	117	33	38
				28
				39
				37
				39
				37
				40
				40
				29

34
32
30
27
34
29
31
27
36
39
31
29
34
38
32
35
34
34
34
34
28
37
34
28
37
38
34
39
32
27
35
41
34
35
36
33
31
37
36
39
32
37
32
30
26
30
31
31
34
35
34
36
34
37
36
38
32
33
30

36
33
34
38
26
29
36
38
30
33
40
37
36
37
34
30
35
27
34
35
36
39
34
28
36
35
39
31
34
35
29
37
37
31
34
29
39
32
38
35
36
36
33
36
30
36
28
34
37
30
30
36
39
41
32
34
34
35
32

33
34
31
32
35
38
35
39
30
34
33
38
34
32
28
26
39
34
34
34
32
33
31
39
37
32
34
39
29
36
36
34
29
30
38
32
33
35
40
34
30
36
35
37
36
32
33
37
33
30
42
33
30
36
36
38
33
32
39

34
38
31
35
28
36
26
41
28
29
34
29
29
35
31
33
36
30
31
35
25
33
31
29
31
30
31
28
36
32
36
31
39
30
28
32
34
26
30
36
36
31
36
38
36
32
37
36
39
34
32
42
26
36
32
29
34
34
36

35
35
27
37
27
34
28
39
32
37
29
36
34
40
34
36
29
35
33
34
32
33
30
36
36
39
37
37
32
29
31
33
33
36
39
31
32
33
35
36
32
37
26
37
36
31
32
32
39
31
34
32
32
27
40
37
32
32
27

26
35
47
39
37
35
35
30
33
37
39
34
38
31
39
32
30
35
32
35
36
36
31
41
34
29
35
35
31
40
40
36
28
37
34
32
32
28
32
35
31
35
28
36
37
32
29
37
34
24
36
26
28
34
26
36
35
33
37

32
32
34
39
32
33
29
30
26
31
36
30
39
30
33
32
33
40
37
34
30
30
33
30
31
34
30
36
31
36
33
43
32
35
30
35
28
33
31
35
37
35
44
39
31
33
31
34
26
35
34
36
33
32
39
31
36
25
29

35
34
32
32
34
33
32
31
30
41
36
31
32
37
31
37
29
29
31
31
34
29
37
35
28
31
34
29
38
36
26
34
36
28
34
38
41
29
33
32
31
29
32
29
38
32
31
32
31
33
32
36
39
40
29
34
29
39

34
39
31
37
32
36
33
32
33
37
35
31
36
34
36
37
43
44
36
30
36
32
32
28
36
28
41
40
38
30
27
31
34
37
28
29
32
29
36
35
31
40
35
32
28
32
37
38
32
38
34
35
37
30
34
30
36
30
31

29
32
36
34
32
37
32
32
35
37
36
38
33
36
36
31
36
34
30
35
33
32
30
39
35
31
29
29
28
34
37
40
42
31
31
33
28
40
29
30
42
32
34
45
33
34
35
27
31
34
34
31
28
32
34
33
40
36
30

30
35
34
38
32
29
32
37
39
29
32
38
31
33
36
38
29
40
40
30
31
31
44
31
40
35
31
32
38
30
36
39
38
31
39
41
35
36
29
29
34
37
33
28
22
37
38
39
32
36
33
28
26
33
47
33
29
37
32

31
36
34
32
37
37
34
43
35
31
33
39
28
35
26
27
45
30
29
36
29
31
37
35
31
31
36
34
33
39
38
35
32
34
32
32
33
39
34
35
36
41
31
32
42
39
38
26
36
36
29
38
30
30
36
32
33
33
32

37
35
31
37
33
28
36
35
28
33
37
34
35
34
40
32
30
36
39
37
32
42
38
41
35
29
37
35
25
37
31
29
29
32
32
37
40
37
32
28
32
35
28
34
30
41
35
32
27
29
33
36
38
28
38
31
33
32
40

36
37
31
31
36
38
31
32
33
32
33
29
38
36
26
36
39
35
33
29
38
31
32
33
37
32
31
32
27
37
37
31
28
34
38
42
34
37
33
32
34
31
41
25
28
36
30
31
31
34
30
33
29
36
36
38
43
33
30

32
29
37
32
33
35
29
33
28
36
35
34
29
32
34
30
28
27
30
35
29
35
36
36
39
34
33
35
31
29
38
34
38
38
32
32
33
30
40
34
34
38
33
28
38
43
41
37
34
34
35
34
38
37
36
36
28
33
33

25
39
30
37
24
37
26

34
36
26
34
36
28
34
38
41
29
33
32
31
29
32
29

38
32
31
32
31
33
32
36
39
40
29
34
29
39
34
39
31
37
32
36
33
32
33
37
35
31
36
34
36
37
43
44
36
30
36
32
32
28
36
28
41
40
38
30
27
31
34
37
28
29
32
29
36
35
31
40
35
32
28

32
37
38
32
38
34
35
37
30
34
30
36
30
31
29
32
36
34
32
37
32
32
35
37
36
38
33
36
36
31
36
34
30
35
33
32
30
39
35
31
29
29
28
34
37
40
42
31
31
33
28
40
29
30
42
32
34
45
33

34
35
27
31
34
34
31
28
32
34
33
40
36
30
30
35
34
38
32
29
32
37
39
29
32
38
31
33
36
38
29
40
40
30
31
31
44
31
40
35
31
32
38
30
36
39
38
31
39
41
35
36
29
29
34
37
33
28
22

37
38
39
32
36
33
28
26
33
47
33
29
37
32
31
36
34
32
37
37
34
43
35
31
33
39
28
35
26
27
45
30
29
36
29
31
37
35
31
31
36
34
33
39
38
35
32
34
32
32
33
39
34
35
36
41
31
32
42

39
38
26
36
36
29
38
30
30
36
32
33
33
32
37
35
31
37
33
28
36
35
28
33
37
34
35
34
40
32
30
36
39
37
32
42
38
41
35
29
37
35
25
37
31
29
29
32
32
37
40
37
32
28
32
35
28
34
30

41
35
32
27
29
33
36
38
28
38
31
33
32
40
36
37
31
31
36
38
31
32
33
32
33
29
38
36
26
36
39
35
33
29
38
31
32
33
37
32
31
32
27
37
37
31
28
34
38
42
34
37
33
32
34
31
41
25
28

36
30
31
31
34
30
33
29
36
36
38
43
33
30
32
29
37
32
33
35
29
33
28
36
35
34
29
32
34
30
28
27
30
35
29
35
36
36
39
34
33
35
31
29
38
34
38
38
32
32
33
30
40
34
34
38
33
28
38

43
41
37
34
34
35
34
38
37
36
36
28
33
33
25
39
30
37
24
37
26

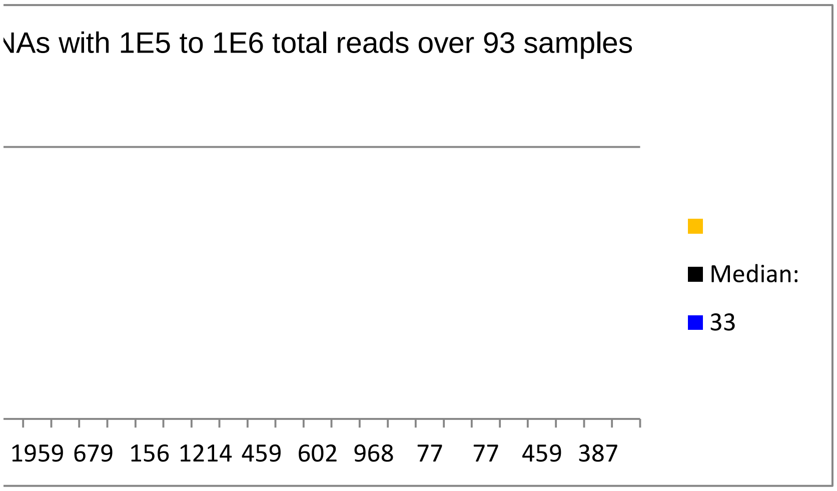
NAs with 1E5 to 1E6 total reads over 93 samples
Median:
33
1959 679 156 1214 459 602 968 77 77 459 387

56	57	58	59	60	61	62	63	64	65	66	67	68	69	70	71
F_38	*F_39*	*G_3*	*G_30*	*G_31*	*G_32*	*G_4*	*G_5*	*G_6*	*G_7*	*A_10*	*A_12*	*A_18*	*A_3*	*A_5*	*A_6*

0.55															
-0.34	-0.13														
0.18	0.07	-0.30													
-0.24	-0.06	-0.01	0.48												
-0.20	0.01	0.06	0.41	0.82											
-0.30	-0.08	0.93	-0.36	-0.07	0.01										
0.39	-0.09	-0.08	0.11	-0.13	-0.15	-0.03									
0.26	0.21	0.05	0.16	0.14	0.10	0.10	0.61								
-0.11	-0.04	0.44	-0.25	0.03	0.04	0.46	0.19	0.38							
-0.28	-0.12	0.03	-0.04	-0.15	-0.29	-0.01	-0.05	-0.11	0.10						
-0.21	0.07	0.36	-0.42	-0.14	-0.11	0.43	-0.14	-0.08	0.39	0.04					
-0.05	-0.13	-0.13	-0.08	-0.04	-0.04	-0.07	-0.15	-0.25	-0.07	0.08	0.15				
-0.05	0.06	-0.18	0.12	0.19	0.16	-0.15	0.07	0.06	0.04	0.19	-0.08	-0.03			
-0.16	0.08	-0.13	0.21	0.54	0.41	-0.14	-0.24	-0.16	-0.03	0.14	0.06	-0.02	0.55		
0.07	0.25	-0.15	-0.04	-0.24	-0.37	-0.15	0.00	0.01	-0.17	0.44	0.14	-0.01	0.28	0.29	
-0.30	-0.02	0.19	-0.16	0.15	0.13	0.24	-0.21	-0.06	0.07	0.24	0.20	0.10	-0.20	0.06	0.05
-0.26	0.03	0.27	-0.43	-0.13	-0.13	0.38	0.01	0.14	0.41	0.24	0.57	-0.04	0.17	0.05	0.29
-0.44	-0.15	0.26	-0.15	0.15	0.23	0.25	-0.25	-0.23	0.07	0.12	0.26	-0.16	-0.01	0.30	0.03
0.17	-0.08	-0.23	0.13	-0.11	-0.10	-0.26	0.15	-0.07	-0.25	-0.12	-0.08	-0.04	0.01	-0.06	-0.06
-0.30	0.30	0.32	0.05	0.20	0.18	0.27	-0.34	0.05	0.28	0.30	0.14	-0.34	-0.07	0.17	0.09
-0.12	-0.21	0.05	-0.02	0.02	0.04	0.07	-0.25	-0.33	-0.02	0.13	0.12	0.56	-0.40	-0.06	-0.18
0.08	0.18	-0.08	0.07	-0.06	-0.11	-0.02	-0.16	-0.01	-0.20	0.20	-0.07	0.41	-0.24	-0.16	0.05
-0.04	0.01	0.33	-0.34	0.09	0.13	0.35	-0.20	-0.09	0.42	-0.13	0.41	-0.06	-0.08	0.23	-0.17
0.00	-0.02	0.18	-0.17	0.34	0.32	0.17	-0.15	0.03	0.46	-0.24	0.21	-0.06	-0.30	0.11	-0.38
0.27	-0.17	-0.10	-0.10	-0.06	-0.11	-0.11	0.29	0.04	0.31	-0.24	-0.08	-0.02	-0.03	-0.06	-0.26
0.26	0.27	0.01	0.15	0.38	0.39	0.00	-0.15	0.12	0.17	-0.27	0.05	-0.07	-0.14	0.22	-0.13
-0.01	-0.05	-0.24	0.24	0.06	-0.02	-0.31	0.04	0.01	-0.34	-0.04	-0.40	-0.30	-0.24	-0.02	0.00
-0.27	-0.15	0.82	-0.28	0.00	0.05	0.76	-0.05	-0.01	0.24	-0.02	0.27	-0.15	-0.31	-0.12	-0.20
-0.26	-0.09	0.87	-0.36	-0.13	-0.05	0.89	-0.04	0.03	0.31	0.00	0.31	-0.09	-0.15	-0.17	-0.10
0.05	-0.12	-0.11	0.09	0.27	0.19	-0.18	-0.05	-0.13	-0.02	-0.18	-0.10	-0.17	-0.27	0.18	-0.15
0.06	0.02	-0.01	0.20	0.39	0.35	-0.09	-0.14	0.04	0.10	-0.17	-0.05	-0.29	-0.27	0.30	-0.06
-0.02	0.04	-0.03	0.00	0.23	0.20	-0.03	0.01	0.14	0.20	-0.14	0.07	-0.10	0.03	0.18	0.04
-0.17	-0.13	0.21	-0.17	0.10	0.01	0.25	-0.22	-0.18	0.20	0.17	0.25	0.02	-0.37	0.07	0.00
0.16	-0.04	-0.11	0.15	0.35	0.34	-0.18	0.01	-0.02	0.16	-0.21	-0.09	-0.14	-0.15	0.24	-0.13
-0.32	-0.06	0.37	-0.14	0.04	0.04	0.38	-0.17	-0.01	0.47	0.47	0.08	0.02	0.08	0.03	-0.03
0.29	0.18	-0.14	0.04	-0.24	-0.33	-0.15	0.03	-0.10	-0.22	0.22	-0.07	0.10	-0.29	-0.18	0.41
0.38	0.16	-0.39	0.46	0.02	-0.13	-0.44	0.16	0.18	-0.29	0.02	-0.47	-0.38	0.06	0.08	0.16
0.00	0.01	0.30	-0.25	-0.01	-0.05	0.31	0.12	0.25	0.67	0.07	0.23	0.19	-0.25	-0.17	-0.17
0.21	0.25	0.08	-0.06	0.01	-0.03	0.13	0.30	0.39	0.30	-0.04	0.48	0.07	-0.14	-0.10	0.01

)
& RANKS

	RANKS	
NPpseu	NPtru	NPpseudo
26	1335	602
29	968	859
15	1970	38
32	1084	1214
30	1877	968
25	1752	529
42	1084	1959
36	1447	1654
17	762	77
32	1752	1214
26	1985	602
21	1447	260
20	1214	202
32	1084	1214
22	1824	323
20	1877	202
16	1942	54
18	602	114
29	859	859
28	762	762
25	1917	529
37	1214	1752
28	1214	762
27	1335	679
42	1877	1959
25	1447	529
27	762	679
19	1553	156
19	1654	156
15	1654	38
32	1335	1214
21	1654	260
24	1553	459
21	679	260
26	1824	602
29	968	859
30	679	968
19	1752	156
17	1214	77
39	1084	1877
17	968	77
15	1917	38
24	602	459
25	1214	529
23	1084	387
21	1335	260
33	1335	1335
25	1447	529
25	859	529

The Wilcoxon Rank-Sum Test (Two Independent Samples)

100 trials

	NPtru	NPpseudo
Observ	100	100
Mean:	33	25
Mediar	33	25
Standa	4	7

The Test Procedure

Hypc	0
Nb.	31
Aver	6
Rank	####
Rank	101
Test	####
Nomi	0

Two-Tailed Test (Normal Approximation)

Crit	-1.95996398454005 and 1.9599639845
Deci	Reject H0 if |Z-Stat| > 1.95996398
Z St	8
Z St	8
Z St	8
P-Va	0
Fina	The Null Hypothesis Must be Reject

INPUT DATA & RANKS

		RANKS	
NPtru	NPpseu	NPtru	NPpseudo
33	26	136	67
30	29	99	89
43	15	199	3
31	32	110	122
39	30	189	99
37	25	175	56
31	42	110	198
34	36	148	167
28	17	83	10
37	32	175	122
45	26	200	67
34	21	148	29
32	20	122	24
31	32	110	122
38	22	183	35
39	20	189	24
41	16	195	6
26	18	67	15
29	29	89	89
28	28	83	83
40	25	193	56
32	37	122	175
32	28	122	83
33	27	136	75
39	42	189	198
34	25	148	56
28	27	83	75
35	19	159	20
36	19	167	20
36	15	167	3
33	32	136	122
36	21	167	29
35	24	159	46
27	21	75	29
38	26	183	67
30	29	99	89
27	30	75	99
37	19	175	20
32	17	122	10
31	39	110	189
30	17	99	10
40	15	193	3
26	24	67	46
32	25	122	56
31	23	110	41
33	21	136	29
33	33	136	136
34	25	148	56
29	25	89	56

22	1214	323
20	1752	202
26	1447	602
30	1214	968
38	968	1824
18	1447	114
28	1214	762
19	968	156
32	459	1214
16	1877	54
18	602	114
22	1214	323
12	1553	11
23	1084	387
19	1824	156
38	1335	1824
30	1335	968
25	1553	529
26	1447	602
36	1335	1654
25	1942	529
25	1654	529
34	529	1447
21	1553	260
34	1553	1447
17	1752	77
30	1084	968
28	1553	762
32	1335	1214
31	1553	1084
37	1084	1752
24	1335	459
18	1447	114
26	968	602
35	1553	1553
22	1335	323
24	762	459
25	1335	529
23	459	387
27	968	679
16	1824	54
22	1752	323
27	679	679
21	1654	260
22	602	323
19	1447	156
25	1824	529
22	968	323
23	968	387
24	1942	459
17	1824	77
25	762	529
40	1877	1917
21	1752	260
31	1877	1084
25	1752	529
22	1917	323
26	1917	602
28	859	762

32	22	122	35
37	20	175	24
34	26	148	67
32	30	122	99
30	38	99	183
34	18	148	15
32	28	122	83
30	19	99	20
24	32	46	122
39	16	189	6
26	18	67	15
32	22	122	35
35	12	159	1
31	23	110	41
38	19	183	20
33	38	136	183
33	30	136	99
35	25	159	56
34	26	148	67
33	36	136	167
41	25	195	56
36	25	167	56
25	34	56	148
35	21	159	29
35	34	159	148
37	17	175	10
31	30	110	99
35	28	159	83
33	32	136	122
35	31	159	110
31	37	110	175
33	24	136	46
34	18	148	15
30	26	99	67
35	35	159	159
33	22	136	35
28	24	83	46
33	25	136	56
24	23	46	41
30	27	99	75
38	16	183	6
37	22	175	35
27	27	75	75
36	21	167	29
26	22	67	35
34	19	148	20
38	25	183	56
30	22	99	35
30	23	99	41
41	24	195	46
38	17	183	10

29	1447	859
26	1214	602
24	968	459
24	679	459
36	1447	1654
25	859	529
29	1084	859
22	679	323
18	1654	114
31	1877	1084
23	1084	387
30	859	968
17	1447	77
28	1824	762
23	1214	387
20	1553	202
27	1447	679
35	1447	1553
20	1447	202
17	1447	77
42	762	1959
27	1752	679
19	1447	156
31	762	1084
24	1752	459
25	1824	529
24	1447	459
17	1877	77
24	1214	459
13	679	17
26	1553	602
22	1942	323
26	1447	602
19	1553	156
32	1654	1214
35	1335	1553
28	1084	762
17	1752	77
20	1654	202
29	1877	859
28	1214	762
17	1752	77
29	1214	859
33	968	1335
26	602	602
25	968	529
26	1084	602
31	1084	1084
21	1447	260
26	1553	602
23	1447	387
22	1654	323
20	1447	202
24	1752	459
37	1654	1752
57	1824	2000
27	1214	679
24	1335	459
25	968	529

33	1654	1335
19	1335	156
21	1447	260
29	1824	859
21	602	260
29	859	859
18	1654	114
23	1824	387
28	968	762
29	1335	859
32	1917	1214
31	1752	1084
24	1654	459
35	1752	1553
27	1447	679
30	968	968
20	1553	202
22	679	323
14	1447	24
22	1553	323
24	1654	459
45	1877	1985
24	1447	459
15	762	38
23	1654	387
28	1553	762
26	1877	602
11	1084	7
18	1447	114
24	1553	459
27	859	679
14	1752	24
30	1752	968
22	1084	323
29	1447	859
22	859	323
15	1877	38
18	1214	114
21	1824	260
30	1553	968
25	1654	529
49	1654	1996
20	1335	202
30	1654	968
17	968	77
31	1654	1084
26	762	602
23	1447	387
24	1752	459
44	968	1978
21	968	260
22	1654	323
30	1877	968
29	1942	859
31	1214	1084
32	1447	1214
25	1447	529
24	1553	459
30	1214	968

22	1335	323
20	1447	202
19	1084	156
21	1214	260
23	1553	387
30	1824	968
28	1553	762
36	1877	1654
33	968	1335
22	1447	323
29	1335	859
30	1824	968
18	1447	114
24	1214	459
23	762	387
28	602	762
18	1877	114
18	1447	114
22	1447	323
16	1447	54
28	1214	762
24	1335	459
17	1084	77
48	1877	1995
24	1752	459
25	1214	529
31	1447	1084
19	1877	156
13	859	17
33	1654	1335
20	1654	202
27	1447	679
18	859	114
52	968	1998
26	1824	602
21	1214	260
36	1335	1654
26	1553	602
27	1917	679
25	1447	529
22	968	323
9	1654	2
20	1553	202
26	1752	602
26	1654	602
27	1214	679
25	1335	529
21	1752	260
22	1335	323
28	968	762
26	1959	602
28	1335	762
23	968	387
25	1654	529
24	1654	459
22	1824	323
22	1335	323
23	1214	387
23	1877	387

25	1447	529
32	1824	1214
24	1084	459
21	1553	260
18	762	114
27	1654	679
19	602	156
19	1942	156
39	762	1877
25	859	529
31	1447	1084
24	859	459
14	859	24
36	1553	1654
33	1084	1335
23	1335	387
40	1654	1917
25	968	529
27	1084	679
23	1553	387
56	529	1999
29	1335	859
26	1084	602
21	859	260
18	1084	114
19	968	156
28	1084	762
18	762	114
21	1654	260
26	1214	602
20	1654	202
23	1084	387
22	1877	323
17	968	77
21	762	260
19	1214	156
22	1447	323
27	602	679
26	968	602
35	1654	1553
24	1654	459
30	1084	968
29	1654	859
40	1824	1917
15	1654	38
29	1214	859
23	1752	387
20	1654	202
24	1877	459
20	1447	202
27	1214	679
28	1959	762
34	602	1447
31	1654	1084
30	1214	968
20	859	202
17	1447	77
38	1447	1824
24	1654	459

26	1553	602
26	1553	602
14	679	24
21	1752	260
29	679	859
33	1447	1335
44	762	1978
25	1877	529
21	1214	260
33	1752	1335
22	859	323
38	1654	1824
25	1447	529
15	1917	38
41	1447	1942
27	1654	679
26	859	602
21	1553	260
21	1335	260
34	1447	1447
27	1214	679
21	1335	260
15	968	38
35	1654	1553
31	1654	1084
22	1877	323
8	1752	1
32	1752	1214
29	1214	859
18	859	114
29	1084	859
33	1335	1335
20	1335	202
17	1654	77
14	1877	24
23	1084	387
24	1214	459
23	1335	387
25	1553	529
20	1654	202
35	1214	1553
26	1752	602
25	602	529
21	1752	260
17	1654	77
31	1084	1084
26	1214	602
32	1214	1214
12	1877	11
23	1084	387
43	1447	1970
21	1214	260
10	1214	4
33	679	1335
26	1917	602
30	1752	968
30	1214	968
21	1214	260
31	679	1084

28	602	762
24	1553	459
29	1993	859
19	1877	156
24	1752	459
27	1553	679
16	1553	54
26	968	602
28	1335	762
23	1752	387
24	1877	459
30	1447	968
23	1824	387
35	1084	1553
24	1877	459
18	1214	114
27	968	679
21	1553	260
29	1214	859
31	1553	1084
27	1654	679
18	1654	114
37	1084	1752
22	1942	323
41	1447	1942
11	859	7
28	1553	762
19	1553	156
28	1084	762
39	1917	1877
23	1917	387
23	1654	387
29	762	859
21	1752	260
29	1447	859
32	1214	1214
34	1214	1447
22	762	323
30	1214	968
28	1553	762
33	1084	1335
13	1553	17
41	762	1942
35	1654	1553
24	1752	459
12	1214	11
25	859	529
19	1752	156
29	1447	859
29	459	859
19	1654	156
23	602	387
26	762	602
31	1447	1084
21	602	260
25	1654	529
20	1553	202
23	1335	387
35	1752	1553

24	1214	459
25	1214	529
20	1447	202
27	1877	679
20	1214	202
36	1335	1654
24	859	459
29	968	859
22	602	323
26	1084	602
14	1654	24
22	968	323
30	1877	968
31	968	1084
20	1335	202
27	1214	679
28	1335	762
27	1917	679
19	1752	156
33	1447	1335
23	968	387
20	968	202
44	1335	1978
19	968	156
22	1084	323
27	1447	679
20	968	202
21	1654	260
24	1084	459
27	1654	679
26	1335	602
24	1970	459
18	1214	114
32	1553	1214
14	968	24
21	1553	260
20	762	202
30	1335	968
42	1084	1959
35	1553	1553
24	1752	459
27	1553	679
23	1978	387
29	1877	859
28	1084	762
25	1335	529
23	1084	387
32	1447	1214
20	602	202
19	1553	156
30	1447	968
24	1654	459
19	1335	156
21	1214	260
16	1877	54
45	1084	1985
15	1654	38
29	529	859
15	859	38

34	1553	1447
46	1447	1990
18	1214	114
18	1214	114
18	1447	114
17	1335	77
46	1214	1990
19	1084	156
21	968	260
26	1942	602
27	1654	679
27	1084	679
31	1214	1084
16	1752	54
20	1084	202
32	1752	1214
34	859	1447
22	859	323
26	1084	602
23	1084	387
14	1447	24
18	859	114
45	1752	1985
25	1553	529
19	762	156
27	1084	679
20	1447	202
21	859	260
15	1824	38
31	1654	1084
16	602	54
24	1447	459
18	1654	114
24	762	459
19	1447	156
17	1824	77
22	1942	323
37	859	1752
12	1335	11
28	1214	762
20	1084	202
27	859	679
32	1214	1214
18	859	114
27	1824	679
28	1214	762
21	1084	260
25	1214	529
22	1084	323
29	1335	859
16	1214	54
21	1654	260
27	1877	679
20	1917	202
17	859	77
25	1447	529
17	859	77
34	1877	1447

20	1447	202
32	1877	1214
33	1084	1335
22	1752	323
25	1214	529
23	1654	387
26	1335	602
24	1214	459
29	1335	859
20	1752	202
18	1553	114
33	1084	1335
32	1654	1214
25	1447	529
18	1654	114
24	1752	459
25	1970	529
29	1978	859
20	1654	202
22	968	323
24	1654	459
24	1214	459
30	1214	968
20	762	202
18	1654	114
23	762	387
17	1942	77
28	1917	762
31	1824	1084
20	968	202
23	679	387
23	1084	387
25	1447	529
25	1752	529
18	762	114
20	859	202
25	1214	529
22	859	323
26	1654	602
23	1553	387
15	1084	38
33	1917	1335
33	1553	1335
18	1214	114
29	762	859
21	1214	260
24	1752	459
23	1824	387
26	1214	602
23	1824	387
25	1447	529
31	1553	1084
28	1752	762
20	968	202
30	1447	968
24	968	459
35	1654	1553
33	968	1335
30	1084	968

23	859	387
29	1214	859
19	1654	156
26	1447	602
30	1214	968
21	1752	260
33	1214	1335
27	1214	679
31	1553	1084
24	1752	459
42	1654	1959
31	1824	1084
19	1335	156
23	1654	387
23	1654	387
26	1084	602
32	1654	1214
19	1447	156
23	968	387
27	1553	679
29	1335	859
18	1214	114
17	968	77
27	1877	679
30	1553	968
30	1084	968
25	859	529
24	859	459
23	762	387
21	1447	260
23	1752	387
21	1917	260
30	1959	968
18	1084	114
30	1084	968
21	1335	260
28	762	762
31	1917	1084
23	859	387
10	968	4
26	1959	602
20	1214	202
17	1447	77
23	1985	387
41	1335	1942
16	1447	54
25	1553	529
19	679	156
23	1084	387
28	1447	762
46	1447	1990
27	1084	679
30	762	968
38	1214	1824
19	1447	156
25	1335	529
28	1917	762
23	1654	387
19	968	156

26	968	602
37	1553	1752
29	1447	859
19	1824	156
13	1214	17
23	859	387
18	1214	114
21	1752	260
19	1877	156
30	859	968
42	1214	1959
21	1824	260
19	1084	156
31	1335	1084
21	1654	260
27	1824	679
26	859	602
31	1917	1084
18	1917	114
28	968	762
15	1084	38
24	1084	459
22	1978	323
27	1084	679
17	1917	77
21	1553	260
15	1084	38
11	1214	7
28	1824	762
32	968	1214
34	1654	1447
24	1877	459
30	1824	968
23	1084	387
19	1877	156
20	1942	202
27	1553	679
24	1654	459
22	859	323
21	859	260
17	1447	77
27	1752	679
37	1335	1752
21	762	260
30	323	968
20	1752	202
41	1824	1942
23	1877	387
30	1214	968
21	1654	260
21	1335	260
32	762	1214
30	602	968
24	1335	459
24	1993	459
29	1335	859
32	859	1214
31	1752	1084
36	1214	1654

17	1084	77
15	1654	38
24	1447	459
21	1214	260
17	1752	77
32	1752	1214
20	1447	202
26	1970	602
27	1553	679
23	1084	387
22	1335	323
20	1877	202
28	762	762
18	1553	114
27	602	679
29	679	859
19	1985	156
31	968	1084
28	859	762
24	1654	459
28	859	762
39	1084	1877
38	1752	1824
21	1553	260
28	1084	762
31	1084	1084
27	1654	679
37	1447	1752
18	1335	114
11	1877	7
22	1824	323
27	1553	679
30	1214	968
23	1447	387
28	1214	762
21	1214	260
25	1335	529
36	1877	1654
21	1447	260
19	1553	156
29	1654	859
22	1942	323
15	1084	38
23	1214	387
21	1959	260
24	1877	459
26	1824	602
21	602	260
24	1654	459
23	1654	387
26	859	602
27	1824	679
19	968	156
34	968	1447
38	1654	1824
21	1214	260
30	1335	968
34	1335	1447
33	1214	1335

17	1752	77
19	1553	156
35	1084	1553
31	1752	1084
22	1335	323
35	762	1553
25	1654	529
25	1553	529
27	762	679
24	1335	459
27	1752	679
35	1447	1553
28	1553	762
33	1447	1335
24	1917	459
18	1214	114
23	968	387
25	1654	529
27	1877	679
30	1752	968
25	1214	529
29	1959	859
21	1824	260
44	1942	1978
25	1553	529
34	859	1447
21	1752	260
29	1553	859
23	529	387
33	1752	1335
25	1084	529
23	859	387
24	859	459
37	1214	1752
27	1214	679
30	1752	968
21	1917	260
20	1752	202
50	1214	1997
31	762	1084
21	1214	260
17	1553	77
22	762	323
22	1447	323
23	968	387
35	1942	1553
44	1553	1978
33	1214	1335
21	679	260
20	859	202
29	1335	859
30	1654	968
27	1824	679
18	762	114
20	1824	202
22	1084	323
25	1335	529
29	1214	859
35	1917	1553

33	1654	1335
26	1752	602
22	1084	323
23	1084	387
27	1654	679
25	1824	529
29	1084	859
24	1214	459
16	1335	54
26	1214	602
13	1335	17
37	859	1752
27	1824	679
26	1654	602
30	602	968
23	1654	387
25	1877	529
33	1553	1335
12	1335	11
30	859	968
25	1824	529
29	1084	859
26	1214	602
24	1335	459
22	1752	323
27	1214	679
15	1084	38
24	1214	459
28	679	762
31	1752	1084
22	1752	323
31	1084	1084
16	762	54
26	1447	602
18	1824	114
17	1959	77
21	1447	260
22	1752	323
25	1335	529
19	1214	156
24	1447	459
37	1084	1752
17	1942	77
28	529	762
24	762	459
30	1654	968
30	968	968
21	1084	260
22	1084	323
26	1447	602
19	968	156
26	1335	602
23	859	387
37	1654	1752
27	1654	679
19	1824	156
21	1970	260
22	1335	323
28	968	762

18	1214	114
20	859	202
25	1752	529
28	1214	762
17	1335	77
37	1553	1752
28	859	762
28	1335	762
41	762	1942
31	1654	1084
23	1553	387
30	1447	968
29	859	859
43	1214	1970
32	1447	1214
25	968	529
24	762	459
23	679	387
16	968	54
21	1553	260
47	859	1993
21	1553	260
27	1654	679
30	1654	968
15	1877	38
14	1447	24
28	1335	762
23	1553	387
32	1084	1214
26	859	602
21	1824	260
22	1447	323
29	1824	859
13	1824	17
31	1214	1084
20	1214	202
45	1335	1985
16	968	54
33	1917	1335
32	1447	1214
22	1447	323
30	1824	968
24	1335	459
29	762	859
24	1824	459
26	1970	602
22	1942	323
23	1752	387
17	1447	77
32	1447	1214
23	1553	387
23	1447	387
32	1824	1214
27	1752	679
34	1654	1447
24	1654	459
27	762	679
28	1335	762
20	1335	202

21	529	260
23	1877	387
19	968	156
26	1752	602
40	459	1917
31	1752	1084
37	602	1752

27	1447	679
31	1654	1084
16	602	54
24	1447	459
18	1654	114
24	762	459
19	1447	156
17	1824	77
22	1942	323
37	859	1752
12	1335	11
28	1214	762
20	1084	202
27	859	679
32	1214	1214
18	859	114

27	1824	679
28	1214	762
21	1084	260
25	1214	529
22	1084	323
29	1335	859
16	1214	54
21	1654	260
27	1877	679
20	1917	202
17	859	77
25	1447	529
17	859	77
34	1877	1447
20	1447	202
32	1877	1214
33	1084	1335
22	1752	323
25	1214	529
23	1654	387
26	1335	602
24	1214	459
29	1335	859
20	1752	202
18	1553	114
33	1084	1335
32	1654	1214
25	1447	529
18	1654	114
24	1752	459
25	1970	529
29	1978	859
20	1654	202
22	968	323
24	1654	459
24	1214	459
30	1214	968
20	762	202
18	1654	114
23	762	387
17	1942	77
28	1917	762
31	1824	1084
20	968	202
23	679	387
23	1084	387
25	1447	529
25	1752	529
18	762	114
20	859	202
25	1214	529
22	859	323
26	1654	602
23	1553	387
15	1084	38
33	1917	1335
33	1553	1335
18	1214	114
29	762	859

21	1214	260
24	1752	459
23	1824	387
26	1214	602
23	1824	387
25	1447	529
31	1553	1084
28	1752	762
20	968	202
30	1447	968
24	968	459
35	1654	1553
33	968	1335
30	1084	968
23	859	387
29	1214	859
19	1654	156
26	1447	602
30	1214	968
21	1752	260
33	1214	1335
27	1214	679
31	1553	1084
24	1752	459
42	1654	1959
31	1824	1084
19	1335	156
23	1654	387
23	1654	387
26	1084	602
32	1654	1214
19	1447	156
23	968	387
27	1553	679
29	1335	859
18	1214	114
17	968	77
27	1877	679
30	1553	968
30	1084	968
25	859	529
24	859	459
23	762	387
21	1447	260
23	1752	387
21	1917	260
30	1959	968
18	1084	114
30	1084	968
21	1335	260
28	762	762
31	1917	1084
23	859	387
10	968	4
26	1959	602
20	1214	202
17	1447	77
23	1985	387
41	1335	1942

16	1447	54
25	1553	529
19	679	156
23	1084	387
28	1447	762
46	1447	1990
27	1084	679
30	762	968
38	1214	1824
19	1447	156
25	1335	529
28	1917	762
23	1654	387
19	968	156
26	968	602
37	1553	1752
29	1447	859
19	1824	156
13	1214	17
23	859	387
18	1214	114
21	1752	260
19	1877	156
30	859	968
42	1214	1959
21	1824	260
19	1084	156
31	1335	1084
21	1654	260
27	1824	679
26	859	602
31	1917	1084
18	1917	114
28	968	762
15	1084	38
24	1084	459
22	1978	323
27	1084	679
17	1917	77
21	1553	260
15	1084	38
11	1214	7
28	1824	762
32	968	1214
34	1654	1447
24	1877	459
30	1824	968
23	1084	387
19	1877	156
20	1942	202
27	1553	679
24	1654	459
22	859	323
21	859	260
17	1447	77
27	1752	679
37	1335	1752
21	762	260
30	323	968

20	1752	202
41	1824	1942
23	1877	387
30	1214	968
21	1654	260
21	1335	260
32	762	1214
30	602	968
24	1335	459
24	1993	459
29	1335	859
32	859	1214
31	1752	1084
36	1214	1654
17	1084	77
15	1654	38
24	1447	459
21	1214	260
17	1752	77
32	1752	1214
20	1447	202
26	1970	602
27	1553	679
23	1084	387
22	1335	323
20	1877	202
28	762	762
18	1553	114
27	602	679
29	679	859
19	1985	156
31	968	1084
28	859	762
24	1654	459
28	859	762
39	1084	1877
38	1752	1824
21	1553	260
28	1084	762
31	1084	1084
27	1654	679
37	1447	1752
18	1335	114
11	1877	7
22	1824	323
27	1553	679
30	1214	968
23	1447	387
28	1214	762
21	1214	260
25	1335	529
36	1877	1654
21	1447	260
19	1553	156
29	1654	859
22	1942	323
15	1084	38
23	1214	387
21	1959	260

24	1877	459
26	1824	602
21	602	260
24	1654	459
23	1654	387
26	859	602
27	1824	679
19	968	156
34	968	1447
38	1654	1824
21	1214	260
30	1335	968
34	1335	1447
33	1214	1335
17	1752	77
19	1553	156
35	1084	1553
31	1752	1084
22	1335	323
35	762	1553
25	1654	529
25	1553	529
27	762	679
24	1335	459
27	1752	679
35	1447	1553
28	1553	762
33	1447	1335
24	1917	459
18	1214	114
23	968	387
25	1654	529
27	1877	679
30	1752	968
25	1214	529
29	1959	859
21	1824	260
44	1942	1978
25	1553	529
34	859	1447
21	1752	260
29	1553	859
23	529	387
33	1752	1335
25	1084	529
23	859	387
24	859	459
37	1214	1752
27	1214	679
30	1752	968
21	1917	260
20	1752	202
50	1214	1997
31	762	1084
21	1214	260
17	1553	77
22	762	323
22	1447	323
23	968	387

35	1942	1553
44	1553	1978
33	1214	1335
21	679	260
20	859	202
29	1335	859
30	1654	968
27	1824	679
18	762	114
20	1824	202
22	1084	323
25	1335	529
29	1214	859
35	1917	1553
33	1654	1335
26	1752	602
22	1084	323
23	1084	387
27	1654	679
25	1824	529
29	1084	859
24	1214	459
16	1335	54
26	1214	602
13	1335	17
37	859	1752
27	1824	679
26	1654	602
30	602	968
23	1654	387
25	1877	529
33	1553	1335
12	1335	11
30	859	968
25	1824	529
29	1084	859
26	1214	602
24	1335	459
22	1752	323
27	1214	679
15	1084	38
24	1214	459
28	679	762
31	1752	1084
22	1752	323
31	1084	1084
16	762	54
26	1447	602
18	1824	114
17	1959	77
21	1447	260
22	1752	323
25	1335	529
19	1214	156
24	1447	459
37	1084	1752
17	1942	77
28	529	762
24	762	459

30	1654	968
30	968	968
21	1084	260
22	1084	323
26	1447	602
19	968	156
26	1335	602
23	859	387
37	1654	1752
27	1654	679
19	1824	156
21	1970	260
22	1335	323
28	968	762
18	1214	114
20	859	202
25	1752	529
28	1214	762
17	1335	77
37	1553	1752
28	859	762
28	1335	762
41	762	1942
31	1654	1084
23	1553	387
30	1447	968
29	859	859
43	1214	1970
32	1447	1214
25	968	529
24	762	459
23	679	387
16	968	54
21	1553	260
47	859	1993
21	1553	260
27	1654	679
30	1654	968
15	1877	38
14	1447	24
28	1335	762
23	1553	387
32	1084	1214
26	859	602
21	1824	260
22	1447	323
29	1824	859
13	1824	17
31	1214	1084
20	1214	202
45	1335	1985
16	968	54
33	1917	1335
32	1447	1214
22	1447	323
30	1824	968
24	1335	459
29	762	859
24	1824	459

26	1970	602
22	1942	323
23	1752	387
17	1447	77
32	1447	1214
23	1553	387
23	1447	387
32	1824	1214
27	1752	679
34	1654	1447
24	1654	459
27	762	679
28	1335	762
20	1335	202
21	529	260
23	1877	387
19	968	156
26	1752	602
40	459	1917
31	1752	1084
37	602	1752

72	73	74	75	76	77	78	79	80	81	82	83	84	85	86	87
B_31	*C_4*	*C_45*	*D_11*	*D_35*	*D_5*	*D_6*	*E_19*	*E_21*	*E_22*	*E_23*	*E_3*	*E_46*	*E_48*	*E_7*	*E_8*

0.33															
0.39	0.32														
-0.22	-0.29	-0.17													
0.36	0.27	0.35	-0.25												
0.30	-0.08	0.15	-0.12	-0.04											
0.37	-0.06	-0.09	-0.17	0.03	0.46										
-0.04	0.16	0.21	-0.09	0.15	0.18	-0.31									
0.14	0.07	0.10	-0.21	0.12	0.23	-0.10	0.65								
-0.31	-0.14	-0.33	0.17	-0.34	-0.12	-0.36	0.19	0.42							
0.09	0.07	0.01	-0.11	0.18	0.12	-0.02	0.46	0.67	0.22						
0.10	-0.32	0.05	0.13	0.12	-0.14	-0.02	-0.31	-0.09	0.07	-0.02					
0.27	0.20	0.36	-0.20	0.23	0.15	-0.07	0.35	0.25	-0.11	0.09	-0.08				
0.22	0.33	0.23	-0.23	0.21	0.05	-0.04	0.30	0.11	-0.11	0.01	-0.23	0.85			
0.07	-0.29	0.09	0.19	-0.06	0.07	-0.23	0.26	0.53	0.40	0.37	0.46	0.07	-0.16		
0.16	-0.11	0.23	-0.10	0.25	0.14	-0.16	0.44	0.64	0.20	0.71	0.31	0.17	-0.04	0.62	
0.06	0.21	0.03	-0.02	0.07	-0.05	-0.30	0.24	0.37	0.37	0.48	0.21	0.04	0.01	0.41	0.53
0.49	0.23	0.35	-0.31	0.27	0.53	0.19	0.40	0.44	-0.20	0.27	-0.12	0.35	0.21	0.15	0.44
0.06	-0.12	0.04	-0.01	0.05	0.09	-0.27	0.31	0.60	0.42	0.56	0.26	-0.01	-0.17	0.70	0.65
0.28	0.33	0.19	-0.29	0.47	0.21	0.12	0.21	0.14	-0.14	0.08	-0.34	0.27	0.33	-0.28	-0.02
0.19	0.00	-0.21	-0.06	0.01	0.20	0.34	-0.16	-0.17	-0.20	0.06	0.09	-0.11	-0.09	-0.09	0.04
-0.24	-0.34	-0.24	0.16	-0.05	-0.34	0.04	-0.18	-0.12	0.02	0.04	0.33	-0.30	-0.35	0.10	0.25
0.05	0.08	-0.16	-0.24	0.11	0.30	0.08	0.38	0.46	0.27	0.21	-0.34	0.15	0.18	-0.03	0.06
0.00	0.22	-0.10	0.09	-0.01	0.09	-0.04	0.22	0.17	0.01	0.17	-0.27	0.07	0.02	-0.06	0.00

The Wilcoxon Rank-Sum Test (Two Independent Samples)

100 trials

	Ptrue	Ppseudo
Obser	100	100
Mean:	26	36
Media	25	36
Stand	6	4

The Test Procedure

Hyp 0
Nb. 31
Ave 6
Ran 20100
Ran 101
Tes 5966
Nom 0

Two-Tailed Test (Normal Approximation)

Cri -1.95996398454005 and 1.9599639845
Dec Reject H0 if |Z-Stat| > 1.959963984
Z S -10
Z S 10
Z S -10
P-V 0
Fin The Null Hypothesis Must be Rejecte

INPUT DATA & RANKS

		RANKS	
Ptrue	Ppseudo	Ptrue	Ppseudo
17	38	10	163
27	36	64	142
33	38	104	163
24	38	45	163
21	36	21	142
12	36	1	142
23	36	34	142
21	38	21	163
33	36	104	142
25	34	52	119
20	30	16	87
25	40	52	179
14	28	3	72
35	36	132	142
21	40	21	179
27	38	64	163
24	32	45	97
36	36	142	142
25	38	52	163
13	38	2	163
34	34	119	119
23	42	34	191
29	38	79	163
26	44	58	198
21	34	21	119
23	36	34	142
19	28	13	72
25	36	52	142
35	42	132	191
23	36	34	142
25	36	52	142
29	36	79	142
17	42	10	191
23	28	34	72
23	38	34	163
31	38	92	163
24	34	45	119
29	46	79	200
40	42	179	191
22	42	26	191
18	40	12	179
21	34	21	119
29	32	79	97
27	34	64	119
16	36	7	142
16	34	7	119
23	30	34	87
29	30	79	87
27	32	64	97

30	40	87	179
34	38	119	163
23	30	34	87
32	42	97	191
20	32	16	97
23	34	34	119
30	24	87	45
34	30	119	87
33	34	104	119
27	34	64	119
22	40	26	179
37	34	153	119
26	36	58	142
21	34	21	119
23	30	34	87
21	38	21	163
15	38	5	163
23	32	34	97
24	38	45	163
26	44	58	198
33	28	104	72
20	34	16	119
28	42	72	191
24	34	45	119
15	28	5	72
32	42	97	191
28	38	72	163
16	40	7	179
27	38	64	163
17	34	10	119
34	32	119	97
37	34	153	119
42	36	191	142
30	38	87	163
30	34	87	119
25	36	52	142
24	38	45	163
23	40	34	179
28	36	72	142
29	42	79	191
38	40	163	179
40	42	179	191
22	34	26	119
26	36	58	142
23	32	34	97
33	46	104	200
24	42	45	191
26	40	58	179
27	34	64	119
20	40	16	179
26	34	58	119

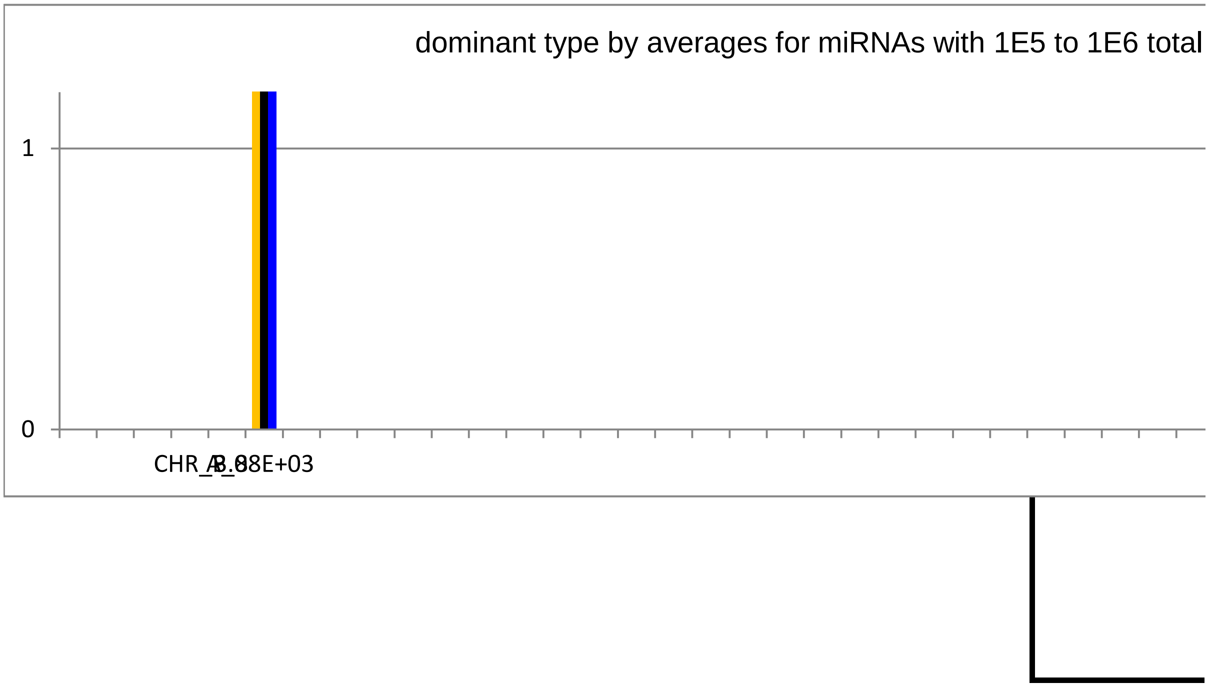
dominant type by averages for miRNAs with 1E5 to 1E6 total
1
0
CHR_A
8.98E+03

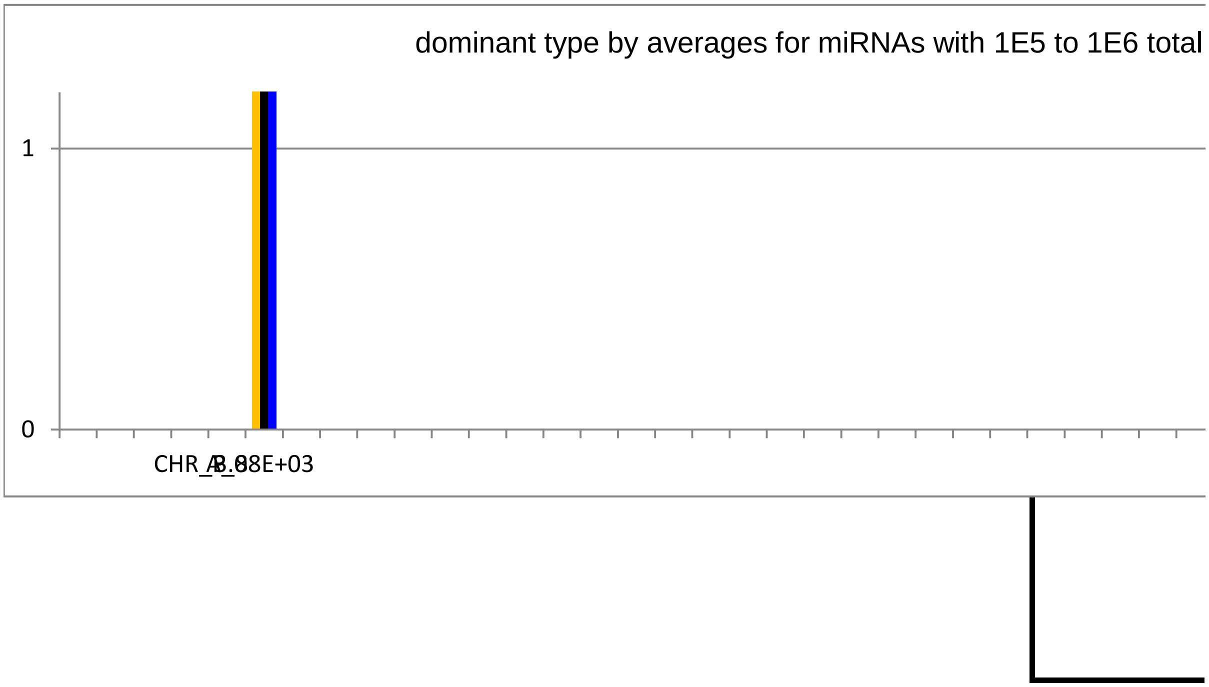

dominant type by averages for miRNAs with 1E5 to 1E6 total
1
0
CHR_A
8.98E+03

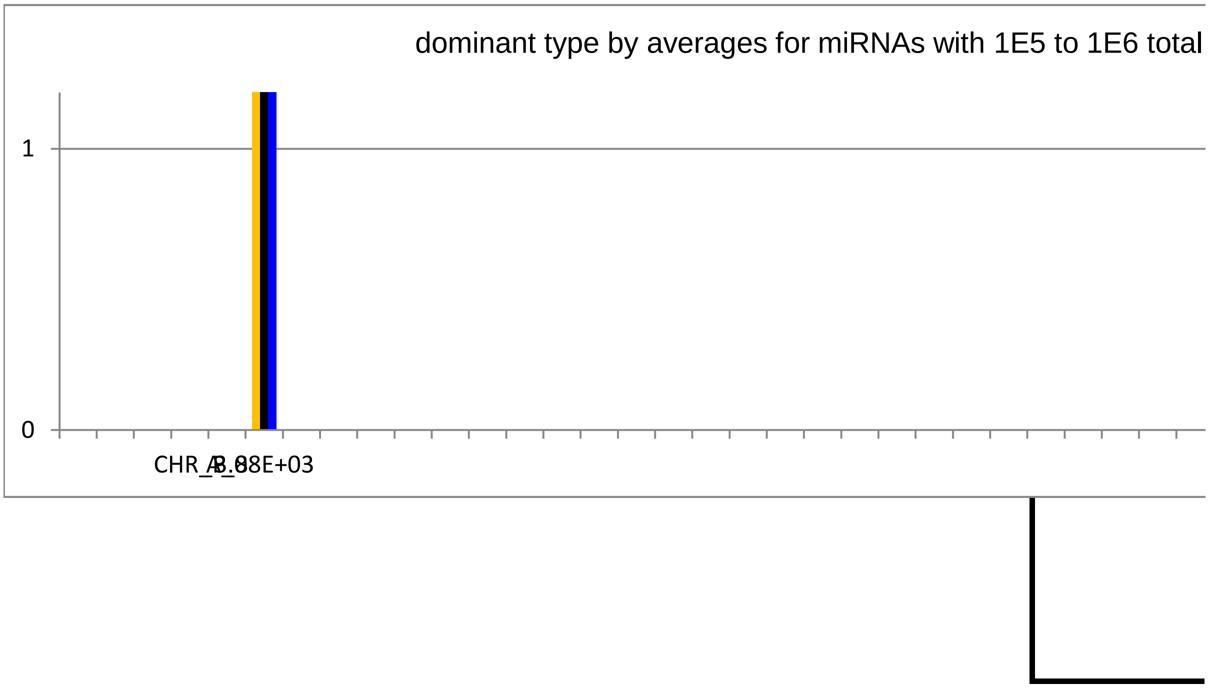

dominant type by averages for miRNAs with 1E5 to 1E6 total
1
0
CHR_A_8
3.98E+03

88	89	90	91	92	93	94	95
E_9	*F_40*	*F_41*	*F_42*	*F_44*	*F_9*	*G_10*	*G_9*

0.07						
0.55	0.26					
-0.14	0.34	-0.15				
-0.10	0.37	0.04	-0.01			
-0.11	-0.11	0.08	-0.18	0.29		
-0.01	0.29	0.11	0.45	0.16	-0.26	
0.03	0.06	-0.01	0.01	0.10	-0.15	0.48

	UC	UC	UC	UC	UC
ave	A_11	A_16	A_2	A_4	A_7
3.59E+06	1.65E+06	5.16E+05	5.58E+06	8.89E+06	1.42E+05
3.96E-02					

UC	CHR_P	UC	CHR_P	CHR_NP
F_2	A_8	D_15	A_9	C_5
8.99E+02	3.08E+03	6.83E+04	8.23E+04	1.41E+05

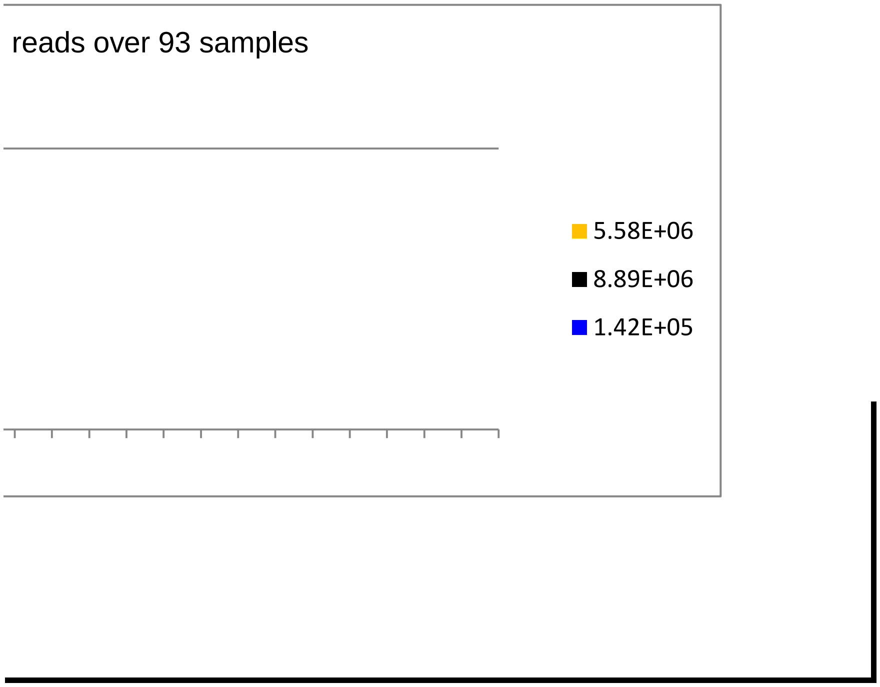

reads over 93 samples
5.58E+06
8.89E+06
1.42E+05

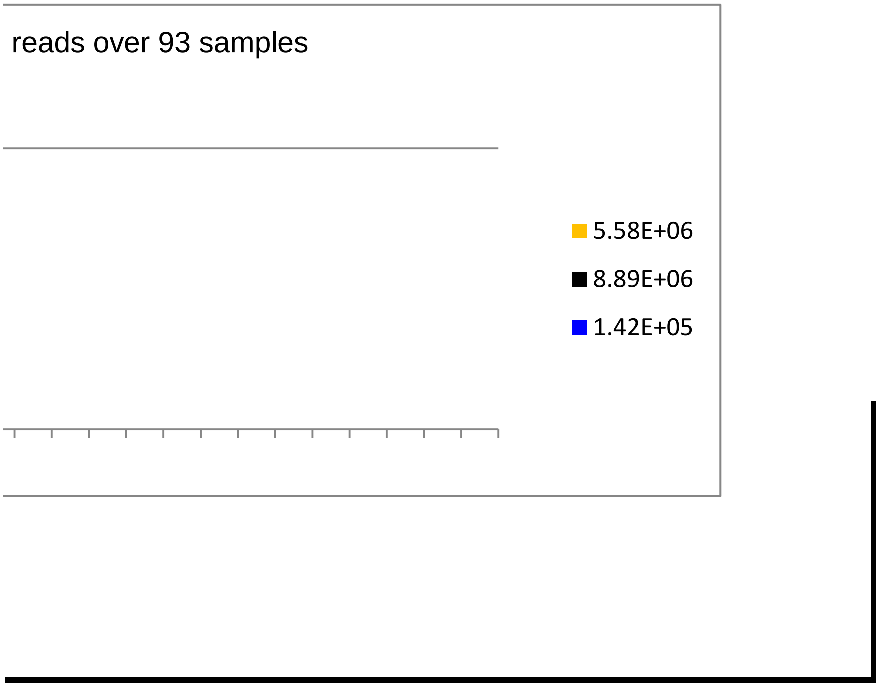
reads over 93 samples
5.58E+06
8.89E+06
1.42E+05

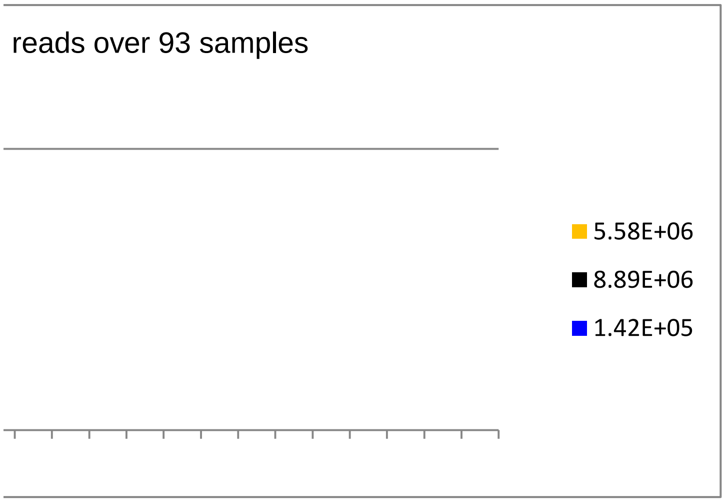
reads over 93 samples
5.58E+06
8.89E+06
1.42E+05

consistent sign test	UC ave	NP ave	P ave	UCvPttest	NPvPttest
miR-92a-3p	0.00	-0.08	-0.38	0.11	0.17
miR-191-5p	0.00	-0.13	-0.09	0.69	0.81
miR-150-5p	0.00	0.12	0.21	0.54	0.78
let-7a-5p	0.00	-0.48	-0.36	0.23	0.64
miR-181a-5p	0.00	0.22	-0.20	0.42	0.15
miR-16-5p	0.00	0.12	0.51	0.13	0.26
miR-26a-5p	0.00	0.45	0.39	0.16	0.83
miR-451a	0.00	0.44	0.50	0.25	0.92

miR-22-3p	0.00	0.06	0.28	0.29	0.36
miR-25-3p	0.00	-0.02	0.69	0.08	0.07
let-7i-5p	0.00	0.41	0.67	0.05	0.55
miR-30d-5p	0.00	-0.13	0.13	0.73	0.51
let-7g-5p	0.00	0.18	0.76	0.07	0.17
let-7b-5p	0.00	-0.28	-0.13	0.77	0.74
let-7f-5p	0.00	0.03	0.71	0.06	0.06
miR-423-3p	0.00	-0.17	-0.29	0.24	0.54
miR-26b-5p	0.00	0.17	0.45	0.10	0.29
miR-103a-3p	0.00	0.02	0.53	0.08	0.07
miR-186-5p	0.00	0.21	0.27	0.29	0.83
miR-28-3p	0.00	0.38	0.16	0.66	0.59
miR-378a-3p	0.00	0.17	0.37	0.23	0.58
miR-93-5p	0.00	-0.15	0.24	0.47	0.20
miR-148a-3p	0.00	0.27	0.49	0.09	0.46
miR-223-3p	0.00	0.30	0.21	0.40	0.74
miR-146b-5p	0.00	0.14	0.27	0.29	0.64
miR-140-3p	0.00	0.14	0.36	0.17	0.37
miR-320a	0.00	0.56	0.63	0.49	0.95
miR-182-5p	0.00	-0.08	0.25	0.36	0.20
miR-151b	0.00	0.05	0.30	0.27	0.34
miR-148b-3p	0.00	0.71	0.26	0.36	0.23
miR-30c-5p	0.00	0.51	0.56	0.07	0.88
let-7d-5p	0.00	-0.44	-0.29	0.38	0.62
miR-30e-5p	0.00	0.30	0.47	0.12	0.59
miR-21-5p	0.00	0.02	-0.01	0.96	0.81
miR-151a-5p	0.00	0.06	0.29	0.29	0.41
miR-15b-5p	0.00	-0.06	-0.21	0.36	0.43
miR-151a-3p	0.00	0.05	0.06	0.84	0.96
miR-146a-5p	0.00	0.13	0.15	0.55	0.92
miR-941	0.00	0.14	-0.39	0.08	0.01
miR-423-5p	0.00	0.10	-0.03	0.93	0.68
miR-197-3p	0.00	0.48	0.20	0.73	0.67
miR-181b-5p	0.00	0.00	-0.03	0.90	0.89
miR-484	0.00	0.26	0.26	0.53	1.00
miR-192-5p	0.00	0.19	0.45	0.23	0.54
miR-342-3p	0.00	-0.06	0.14	0.56	0.35
miR-222-3p	0.00	0.75	0.31	0.34	0.28
miR-27b-3p	0.00	0.48	0.28	0.29	0.52
miR-3615	0.00	0.13	0.13	0.69	0.99
miR-144-5p	0.00	0.34	0.57	0.25	0.67
miR-143-3p	0.00	0.04	0.31	0.31	0.29
miR-127-3p	0.00	0.78	-0.12	0.72	0.09
miR-23a-3p	0.00	0.14	-0.02	0.91	0.39
miR-652-3p	0.00	1.16	0.86	0.50	0.84
miR-30e-3p	0.00	0.06	0.73	0.06	0.10
miR-21-3p	0.00	0.32	-0.01	0.96	0.28

miR-101-3p	0.00	0.37	0.44	0.32	0.88
miR-425-5p	0.00	0.39	-0.02	0.93	0.13
miR-181c-5p	0.00	0.09	0.39	0.20	0.36
miR-155-5p	0.00	-0.17	-0.05	0.83	0.57
miR-125a-5p	0.00	0.08	-0.35	0.11	0.16
miR-221-3p	0.00	0.61	-0.05	0.87	0.16
miR-769-5p	0.00	0.24	0.14	0.58	0.76
miR-126-5p	0.00	0.85	0.20	0.55	0.35
miR-142-5p	0.00	0.25	0.30	0.45	0.91
miR-4732-3p	0.00	-0.25	-0.25	0.39	1.00
miR-17-5p	0.00	0.14	0.23	0.41	0.76
miR-30b-5p	0.00	0.27	0.44	0.16	0.58
miR-20a-5p	0.00	0.31	0.38	0.27	0.88
miR-24-3p	0.00	0.70	0.50	0.10	0.60
miR-183-5p	0.00	-0.11	-0.11	0.59	1.00
let-7d-3p	0.00	-0.05	-0.24	0.34	0.34
miR-486-3p	0.00	-0.23	-0.17	0.60	0.84
miR-98-5p	0.00	0.28	0.45	0.13	0.63
miR-532-5p	0.00	-0.07	0.18	0.47	0.25
miR-29a-3p	0.00	0.64	0.62	0.04	0.95
miR-409-3p	0.00	0.14	-0.23	0.37	0.17
miR-500a-3p	0.00	0.10	0.00	0.99	0.73
miR-99b-5p	0.00	0.00	-0.14	0.52	0.33
miR-744-5p	0.00	-0.15	-0.40	0.15	0.31
miR-23b-3p	0.00	-0.11	-0.36	0.10	0.16
miR-363-3p	0.00	-0.13	0.31	0.34	0.17
miR-1307-3p	0.00	0.23	0.01	0.98	0.66
miR-92b-3p	0.00	-0.20	-0.37	0.14	0.45
miR-340-5p	0.00	0.00	-0.15	0.49	0.41
miR-27a-3p	0.00	0.50	0.64	0.06	0.69
miR-326	0.00	3.19	2.79	0.35	0.92
miR-328	0.00	1.27	0.63	0.64	0.70
miR-126-3p	0.00	0.26	0.42	0.13	0.63
miR-128	0.00	0.34	0.39	0.21	0.89
let-7e-5p	0.00	-0.19	-0.35	0.14	0.43
miR-181a-2-3p	0.00	0.33	0.42	0.16	0.79
miR-144-3p	0.00	0.54	0.54	0.35	0.99
miR-345-5p	0.00	0.09	0.24	0.37	0.61
miR-130a-3p	0.00	0.00	0.17	0.49	0.39
miR-15a-5p	0.00	0.09	0.34	0.25	0.38
miR-28-5p	0.00	0.01	0.14	0.60	0.54
miR-671-3p	0.00	0.04	-0.24	0.41	0.32
miR-106b-3p	0.00	0.57	0.55	0.29	0.97
miR-146b-3p	0.00	0.31	0.24	0.45	0.84
miR-454-3p	0.00	0.16	0.13	0.64	0.94
miR-331-3p	0.00	0.38	0.46	0.44	0.90
miR-130b-3p	0.00	0.29	0.44	0.17	0.63
miR-342-5p	0.00	0.37	-0.12	0.77	0.37

miR-10a-5p	0.00	-0.16	-0.13	0.54	0.73
miR-194-5p	0.00	0.25	0.44	0.17	0.60
miR-660-5p	0.00	0.25	0.46	0.16	0.58
miR-4286	0.00	1.81	1.01	0.43	0.63
miR-361-5p	0.00	-0.04	-0.15	0.46	0.38
miR-106b-5p	0.00	0.26	0.16	0.59	0.73
miR-185-5p	0.00	0.69	0.29	0.32	0.35
miR-181a-3p	0.00	0.17	0.47	0.09	0.24
miR-361-3p	0.00	0.21	0.24	0.51	0.92
miR-145-5p	0.00	1.21	0.74	0.39	0.64
miR-20b-5p	0.00	0.26	0.36	0.25	0.78
miR-31-5p	0.00	0.30	-0.10	0.69	0.18
let-7c	0.00	-0.22	-0.24	0.39	0.94
miR-30a-5p	0.00	0.25	0.58	0.12	0.43
miR-199a-3p	0.00	0.44	0.15	0.53	0.25
miR-199b-3p	0.00	0.44	0.15	0.53	0.25
miR-664a-3p	0.00	-0.03	-0.27	0.23	0.29
miR-324-3p	0.00	0.52	0.70	0.37	0.84
miR-132-3p	0.00	-0.13	0.07	0.79	0.45
miR-410	0.00	0.08	0.09	0.71	0.97
miR-148a-5p	0.00	0.09	0.41	0.11	0.13
miR-142-3p	0.00	0.43	0.20	0.58	0.62
miR-130b-5p	0.00	-0.15	-0.41	0.09	0.19
miR-874	0.00	1.72	1.90	0.35	0.94
miR-339-3p	0.00	-0.14	-0.13	0.59	0.93
miR-766-3p	0.00	0.44	0.43	0.51	0.99
miR-193a-5p	0.00	-0.10	-0.23	0.28	0.24
miR-501-3p	0.00	0.52	0.32	0.32	0.56
miR-26b-3p	0.00	0.47	0.86	0.01	0.25
miR-199a-5p	0.00	0.38	0.17	0.54	0.45
miR-574-3p	0.00	0.55	0.09	0.79	0.40
miR-1307-5p	0.00	0.86	0.76	0.13	0.90
miR-181d	0.00	0.33	0.13	0.64	0.54

UC	UC	UC	UC	UC	UC	UC	UC	UC	UC	UC
B_29	B_32	B_34	C_1	C_40	C_41	C_42	C_44	C_46	C_47	D_15
2.16E+05	7.93E+05	2.42E+06	2.85E+06	1.72E+07	4.59E+06	2.63E+06	1.41E+06	4.41E+06	7.78E+06	6.83E+04

UC	UC	CHR_NP	UC	CHR_P	CHR_P	UC	CHR_P	CHR_NP	CHR_NP	UC
A_7	B_29	C_39	D_36	A_18	D_6	A_16	E_3	A_13	A_17	B_32
1.42E+05	2.16E+05	#######	2.91E+05	3.57E+05	#######	#######	5.28E+05	6.98E+05	7.19E+05	#######

NP<P?	expect
0	0
1	1

1	1
0	0
1	1

0 0

0	0
0	0

UC	UC	UC	UC	UC	UC	UC	UC	UC	UC	UC
D_16	D_17	D_18	D_36	D_4	E_47	F_2	F_43	F_6	F_7	F_8
1.15E+07	3.05E+06	4.01E+06	2.91E+05	4.27E+06	3.42E+06	8.99E+02	2.10E+06	3.63E+06	3.80E+06	3.41E+06

CHR_NP	CHR_NP	CHR_NP	UC	CHR_P	CHR_NP	CHR_NP	UC	CHR_P	UC	CHR_NP
B_30	G_32	D_9	G_33	B_31	G_31	D_12	G_34	E_9	C_44	G_6
8.52E+05	#######	#######	#######	1.10E+06	1.17E+06	1.21E+06	1.23E+06	1.32E+06	#######	#######

UC	UC	UC	CHR_NP	CHR_NP	CHR_NP	CHR_NP	CHR_NP	CHR_NP	CHR_NP	CHR_NP
G_33	G_34	G_8	A_1	A_13	A_14	A_15	A_17	B_30	B_33	C_2
9.88E+05	1.23E+06	8.08E+06	5.81E+06	6.98E+05	2.23E+06	4.03E+06	7.19E+05	8.52E+05	4.58E+06	2.34E+06

CHR_P	CHR_NP	UC	CHR_P	CHR_NP	CHR_NP	UC	CHR_NP	CHR_P	CHR_P	CHR_P
D_11	F_37	A_11	G_9	D_3	E_29	F_43	A_14	A_12	E_21	E_7
#######	1.61E+06	1.65E+06	#######	1.81E+06	2.09E+06	2.10E+06	2.23E+06	2.27E+06	#######	2.33E+06

CHR_NP C_37	CHR_NP C_38	CHR_NP C_39	CHR_NP C_43	CHR_NP C_48	CHR_NP C_5	CHR_NP D_10	CHR_NP D_12	CHR_NP D_13	CHR_NP D_14	CHR_NP D_3
4.57E+06	3.63E+06	2.59E+05	5.68E+06	3.19E+06	1.41E+05	2.63E+06	1.21E+06	6.37E+06	4.72E+06	1.81E+06

CHR_NP C_2	UC B_34	CHR_P E_46	CHR_P D_5	CHR_NP E_5	CHR_P E_23	CHR_NP E_20	CHR_NP E_6	CHR_NP D_10	UC C_42	CHR_NP G_30
#######	#######	2.49E+06	#######	2.55E+06	#######	#######	2.62E+06	2.63E+06	#######	2.67E+06

CHR_NP	CHR_NP	CHR_NP	CHR_NP	CHR_NP	CHR_NP	CHR_NP	CHR_NP	CHR_NP	CHR_NP	CHR_NP
D_9	E_20	E_29	E_4	E_5	E_6	F_1	F_35	F_36	F_37	F_38
9.71E+05	2.60E+06	2.09E+06	4.83E+06	2.55E+06	2.62E+06	3.93E+06	8.30E+06	3.68E+06	1.61E+06	4.57E+06

UC	CHR_P	UC	CHR_P	CHR_NP	UC	UC	UC	CHR_NP	CHR_NP	CHR_NP
C_1	E_8	D_17	A_5	C_48	F_8	E_47	F_6	C_38	F_36	F_39
2.85E+06	3.01E+06	3.05E+06	#######	3.19E+06	3.41E+06	3.42E+06	#######	3.63E+06	3.68E+06	3.69E+06

CHR_NP	CHR_NP	CHR_NP	CHR_NP	CHR_NP	CHR_NP	CHR_NP	CHR_NP	CHR_NP	CHR_P	CHR_P
F_39	G_3	G_30	G_31	G_32	G_4	G_5	G_6	G_7	A_10	A_12
3.69E+06	4.45E+06	2.67E+06	1.17E+06	8.64E+05	1.13E+07	6.41E+06	1.46E+06	6.65E+06	3.90E+06	2.27E+06

UC	CHR_P	CHR_P	CHR_NP	CHR_P	UC	CHR_NP	CHR_P	CHR_P	UC	UC
F_7	F_9	A_10	F_1	E_22	D_18	A_15	G_10	F_42	D_4	C_46
3.80E+06	#######	3.90E+06	3.93E+06	4.00E+06	4.01E+06	4.03E+06	#######	#######	#######	4.41E+06

CHR_P	CHR_P	CHR_P	CHR_P	CHR_P	CHR_P	CHR_P	CHR_P	CHR_P	CHR_P
A_18	A_3	A_5	A_6	A_8	A_9	B_31	C_4	C_45	D_11
3.57E+05	5.81E+06	3.12E+06	4.49E+06	3.08E+03	8.23E+04	1.10E+06	5.44E+06	1.14E+07	1.53E+06

CHR_NP	CHR_P	CHR_P	CHR_NP	CHR_NP	CHR_NP	UC	CHR_NP	CHR_NP	CHR_P
G_3	A_6	F_44	F_38	C_37	B_33	C_41	D_14	E_4	F_41
#######	4.49E+06	4.50E+06	4.57E+06	4.57E+06	4.58E+06	4.59E+06	4.72E+06	4.83E+06	5.30E+06

CHR_P	CHR_P	CHR_P	CHR_P	CHR_P	CHR_P	CHR_P	CHR_P	CHR_P	CHR_P
D_35	D_5	D_6	E_19	E_21	E_22	E_23	E_3	E_46	E_48
5.41E+06	2.50E+06	3.77E+05	1.19E+07	2.30E+06	4.00E+06	2.56E+06	5.28E+05	2.49E+06	1.29E+07

CHR_P	CHR_P	CHR_P	UC	CHR_NP	CHR_NP	CHR_P	CHR_NP	CHR_NP	CHR_NP
D_35	C_4	F_40	A_2	C_43	A_1	A_3	D_13	G_5	G_7
5.41E+06	5.44E+06	5.51E+06	5.58E+06	5.68E+06	5.81E+06	5.81E+06	6.37E+06	6.41E+06	6.65E+06

CHR_P	CHR_P	CHR_P	CHR_P	CHR_P	CHR_P	CHR_P	CHR_P	CHR_P	CHR_P
E_7	E_8	E_9	F_40	F_41	F_42	F_44	F_9	G_10	G_9
2.33E+06	3.01E+06	1.32E+06	5.51E+06	5.30E+06	4.18E+06	4.50E+06	3.84E+06	4.04E+06	1.67E+06

UC	UC	CHR_NP	UC	CHR_NP	CHR_P	UC	CHR_P	CHR_P	UC
C_47	G_8	F_35	A_4	G_4	C_45	D_16	E_19	E_48	C_40
7.78E+06	8.08E+06	8.30E+06	8.89E+06	1.13E+07	1.14E+07	1.15E+07	1.19E+07	1.29E+07	1.72E+07

relabelin
NP
40
48
48
48
51
47
39
36
41
38
45
44
58
36
42
39
34
34
50
39
26
40
36
35
48
32
40
35
47
34
40
33
53
34
36
29
45
46
30
43
39
45
37
51
34
34
39
41
39
38
41

45
47
34
38
42
36
43
42
40
38
31
45
49
63
29
30
42
37
37
44
46
53
43
30
37
44
42
36
32
60
46
76
40
29
30
34
38
45
44
36
39
41
32
39
40
55
50
40
34
46
24
33
41
38
33
36
43
48
40

40
36
51
47
41
44
37
46
38
30
41
35
39
43
25
51
44
40
45
45
33
40
39
40
42
34
38
38
48
47
42
42
35
41
47
57
39
45
35
54
30
40
61
59
36
44
50
36
37
45
40
49
40
42
56
44
60
42
34

41
39
48
36
49
56
34
35
38
51
42
47
38
48
39
51
32
36
38
37
43
35
36
35
46
35
44
43
59
33
38
46
47
29
39
36
50
38
40
42
33
43
50
38
36
35
41
48
39
36
36
41
53
36
41
50
44
55
38

59
36
36
39
44
43
40
43
38
38
47
53
48
37
48
48
31
37
47
39
37
43
44
40
42
42
43
36
40
45
48
42
40
50
49
40
35
46
40
40
34
48
37
47
47
47
37
40
32
47
43
35
26
36
35
48
47
36
30

40
38
42
56
49
31
49
37
58
26
41
48
31
20
48
25
34
33
41
27
37
32
42
46
37
37
27
37
43
48
33
43
26
53
49
57
31
35
40
41
33
32
48
40
42
41
49
24
47
59
40
64
44
41
38
39
36
62
51

35
40
31
43
39
40
39
38
48
34
40
39
57
44
37
52
37
49
40
41
41
35
63
43
34
54
44
57
64
48
43
40
35
49
40
37
60
58
45
41
45
36
54
31
33
42
52
27
41
44
38
62
42
37
46
38
52
42
36

36
53
36
46
29
43
38
32
35
48
36
44
51
33
42
47
48
33
39
40
31
47
42
46
24
47
34
30
37
66
50
35
52
43
43
44
43
32
29
34
38
31
23
51
45
44
40
42
27
38
34
47
35
45
47
34
45
42
48

47
37
42
44
32
48
35
37
46
37
34
49
40
40
24
53
34
32
50
32
35
35
44
49
52
46
33
44
46
40
42
36
35
45
53
36
33
41
43
43
42
34
51
59
52
51
46
43
43
45
31
28
40
35
46
20
42
48
35

32
37
42
44
26
55
43
32
54
30
33
30
49
45
38
46
35
40
39
49
53
49
42
45
37
43
41
49
55
41
41
46
39
35
41
31
31
40
28
39
48
42
40
42
50
50
38
47
43
40
27
58
50
44
47
38
35
33

46
44
35
33
56
41
36
31
40
39
26
30
43
34
36
34
51
40
41
37
23
33
33
41
30
42
48
44
46
39
32
42
45
29
31
33
41
51
29
31
48
38
37
48
38
36
58
47
44
40
51
46
37
36
41
46
28
31
43

41
29
41
35
50
41
41
44
45
41
44
53
45
38
61
31
32
32
44
55
42
45
46
53
58
40
32
40
35
47
43
48
42
38
49
41
38
50
49
48
41
41
39
49
34
29
43
42
47
50
58
38
37
45
53
41
38
37
36

36
48
33
45
49
39
29
31
42
43
36
42
43
43
59
33
47
48
48
44
49
50
42
41
35
39
52
36
45
38
35
27
36
43
37
42
37
38
43
44
49
47
42
48
39
46
53
38
51
31
32
41
40
49
41
40
35
36
41

53
47
32
28
29
44
41
40
39
32
39
42
36
36
32
31
34
32
41
43
38
54
44
28
52
41
49
44
35
41
52
45
37
67
48
46
44
37
39
48
36
43
41
46
40
39
35
43
41
32
45
46
29
37
34
33
35
43
49

43
35
35
43
36
43
53
44
47
46
34
32
36
33
41
27
41
37
33
36
31
45
37
36
35
36
38
38
53
41
47
57
42
35
52
25
45
48
39
36
33
44
51
41
35
36
26
40
45
37
39
51
41
36
48
42
49
47
35

32
45
28
35
54
51
34
38
34
50
49
44
51
40
47
47
39
31
60
28
31
42
33
30
49
31
47
45
49
39
55
35
45
32
34
36
50
30
46
50
40
36
41
35
40
34
40
44
45
57
38
33
40
43
39
42
46
38
41

39
40
38
40
54
39
40
45
39
32
34
48
40
44
38
41
38
55
38
31
38
48
43
40
49
38
43
62
43
55
51
40
55
36
52
34
43
33
42
43
46
45
39
42
36
49
26
37
34
49
28
38
42
46
68
31
40
40
33

37
34
29
37
41
28
58
37
60
34
25
28
40
43
39
42
42
33
41
52
48
41
37
40
45
38
44
39
30
50
31
36
47
36
43
40
40
43
29
37
40
35
39
33
41
39
36
43
38
23
30
32
34
51
45
41
43
38
43

44
34
39
41
42
35
39
51
40
57
35
43
32
43
49
35
50
43
31
41
40
49
32
41
42
42
35
41
35
35
41
35
34
53
46
45
37
47

36
41
41
46
39
35
41
31
31
40
28
39
48
42
40
42

50
50
38
47
43
40
27
58
50
44
47
38
35
33
46
44
35
33
56
41
36
31
40
39
26
30
43
34
36
34
51
40
41
37
23
33
33
41
30
42
48
44
46
39
32
42
45
29
31
33
41
51
29
31
48
38
37
48
38

36
58
47
44
40
51
46
37
36
41
46
28
31
43
41
29
41
35
50
41
41
44
45
41
44
53
45
38
61
31
32
32
44
55
42
45
46
53
58
40
32
40
35
47
43
48
42
38
49
41
38
50
49
48
41
41
39
49
34

29
43
42
47
50
58
38
37
45
53
41
38
37
36
36
48
33
45
49
39
29
31
42
43
36
42
43
43
59
33
47
48
48
44
49
50
42
41
35
39
52
36
45
38
35
27
36
43
37
42
37
38
43
44
49
47
42
48
39

46
53
38
51
31
32
41
40
49
41
40
35
36
41
53
47
32
28
29
44
41
40
39
32
39
42
36
36
32
31
34
32
41
43
38
54
44
28
52
41
49
44
35
41
52
45
37
67
48
46
44
37
39
48
36
43
41
46
40

39
35
43
41
32
45
46
29
37
34
33
35
43
49
43
35
35
43
36
43
53
44
47
46
34
32
36
33
41
27
41
37
33
36
31
45
37
36
35
36
38
38
53
41
47
57
42
35
52
25
45
48
39
36
33
44
51
41
35

36
26
40
45
37
39
51
41
36
48
42
49
47
35
32
45
28
35
54
51
34
38
34
50
49
44
51
40
47
47
39
31
60
28
31
42
33
30
49
31
47
45
49
39
55
35
45
32
34
36
50
30
46
50
40
36
41
35
40

34
40
44
45
57
38
33
40
43
39
42
46
38
41
39
40
38
40
54
39
40
45
39
32
34
48
40
44
38
41
38
55
38
31
38
48
43
40
49
38
43
62
43
55
51
40
55
36
52
34
43
33
42
43
46
45
39
42
36

49
26
37
34
49
28
38
42
46
68
31
40
40
33
37
34
29
37
41
28
58
37
60
34
25
28
40
43
39
42
42
33
41
52
48
41
37
40
45
38
44
39
30
50
31
36
47
36
43
40
40
43
29
37
40
35
39
33
41

39
36
43
38
23
30
32
34
51
45
41
43
38
43
44
34
39
41
42
35
39
51
40
57
35
43
32
43
49
35
50
43
31
41
40
49
32
41
42
42
35
41
35
35
41
35
34
53
46
45
37
47
38
45
40
45
37
40
50

49
35
35
54
38
49
41
46
41
49
36
40
37
38
44
52
45
45
24
54
68
34
48
31
53
32
41
50
37
32
42
44
37
32
33
34
43
27
39
45
41
44
30
36
20
36
38
39
41
36
47
40
40
40
38
45
39
34
55

	30
	39
	39
	55
	53
	56
	56
	32
	33
	42
	23
	49
	32
	37
	42
	26
	38
	31
	47
	41
	50
	50
	33
	31
	45
	31
	39
	50
	35
	33
	32
	37
	39
	41
	41
	43
	30
	36
	36
	45
37	44
40	34
31	53
32	47
44	37
36	53
40	36
43	37
51	30
26	44
41	27
38	53
42	39
34	32
46	46
41	33
51	30
55	33
45	41

61	28
41	32
40	53
42	52
31	32
38	38
41	33
32	39
35	42
44	59
36	38
43	24
38	52
44	39
56	54
39	45
48	32
27	45
33	39
47	38
47	35
40	41
39	42
39	33
43	51
41	32
45	34
42	53
39	37
35	41
39	52
51	39
40	50
32	44
43	34
40	49
52	38
51	31
38	56
49	44
37	57
36	42
44	52
47	44
29	36
39	49
52	38
36	40
40	48
42	41
45	36
45	32
42	50
41	42
40	50
38	32
39	40
43	40
61	33

49	42
38	41
55	29
42	45
40	31
29	55
43	42
55	46
34	50
38	33
33	40
30	42
34	37
47	36
33	69
47	46
50	22
32	51
44	36
28	40
38	49
38	36
37	41
37	48
43	45
43	39
34	52
44	35
49	43
27	47
39	29
51	36
41	37
32	37
30	45
40	45
37	44
45	40
48	41
47	41
50	38
36	50
33	44
35	41
56	28
37	38
40	43
37	35
41	40
30	51
39	46
31	42
35	45
37	58
41	31
38	33
29	55
38	36
50	56

39	39
35	34
47	35
34	40
33	54
42	40
39	52
33	45
27	40
37	53
32	51
41	46
40	41
35	50
41	33
43	28
33	38
38	43
54	37
35	40
31	34
47	38
35	48
33	35
38	35
44	34
51	47
35	37
35	41
29	41

.g+reselection of 29s

P	UC
41	39
38	36
51	30
43	41
41	39
31	41
42	29
47	31
38	36
46	39
47	41
27	40
50	34
35	53
40	50
44	34
41	41
45	44
44	48
40	56
39	43
42	52
33	57
40	39
29	44
46	45
43	35
44	51
32	38
33	48
46	30
54	45
39	26
42	44
61	41
42	59
49	32
46	33
35	42
41	48
32	40
42	54
31	52
46	29
48	50
39	37
40	29
36	38
54	48
61	38
49	33

45	51
31	37
51	46
39	42
46	47
30	52
47	36
39	49
40	40
44	55
43	58
43	51
43	25
27	52
39	55
32	51
44	29
53	31
46	50
42	39
20	42
29	42
40	34
44	48
43	37
39	43
54	33
51	32
41	55
37	28
31	46
43	38
42	36
52	47
61	54
41	50
32	39
34	46
42	39
36	45
35	52
36	54
46	31
49	34
36	39
36	45
39	28
52	34
42	42
31	50
55	43
54	36
55	38
37	68
36	39
50	35
45	36
29	58
44	44

41	47
40	34
53	30
44	33
44	40
53	40
34	40
38	35
36	54
39	27
44	32
36	35
52	33
35	54
46	40
30	38
35	38
62	32
37	52
45	47
56	46
26	43
61	37
43	31
45	44
26	48
39	44
38	45
38	41
47	46
38	51
46	41
45	46
43	38
32	45
41	49
49	35
34	39
50	37
26	49
42	43
50	51
33	41
39	37
41	42
39	31
44	30
42	33
50	39
40	37
31	44
51	39
51	42
37	35
28	43
48	43
41	23
43	45
54	44

33	50
42	50
45	39
46	40
35	48
39	45
45	35
31	49
72	21
41	34
36	45
49	44
52	42
34	29
48	38
45	43
36	51
46	38
50	39
36	53
49	29
40	30
62	36
42	49
27	55
43	40
43	42
38	32
39	39
56	42
59	28
43	27
40	34
45	41
46	50
31	42
45	23
41	48
41	39
41	34
64	35
42	39
23	59
42	38
38	42
30	48
37	52
49	37
30	46
35	42
49	34
40	36
49	40
43	37
36	41
39	38
42	48
36	47
37	31

31	38
54	49
24	58
38	54
32	38
37	32
37	42
38	55
38	39
36	38
45	32
40	37
38	36
51	35
40	44
36	36
54	37
36	33
38	48
39	54
28	44
44	48
29	34
34	36
40	36
41	44
45	55
47	36
36	48
35	45
50	42
36	37
41	47
37	35
44	28
35	41
37	36
29	43
37	50
42	32
44	49
42	34
36	44
50	41
42	35
38	39
35	31
51	39
51	36
35	47
37	44
42	42
36	56
45	36
39	40
36	40
44	43
38	41
42	38

39	41
40	46
32	39
37	26
48	52
41	59
36	34
50	43
35	37
50	47
34	38
41	51
36	40
50	37
37	41
52	44
44	40
27	48
34	55
39	36
36	45
31	39
44	54
39	33
41	54
35	46
45	53
51	37
42	34
35	46
40	51
37	45
46	37
32	31
31	39
32	42
32	56
37	45
42	42
43	31
38	37
39	52
46	35
41	32
35	52
49	44
59	26
46	41
36	50
38	31
45	32
35	30
34	50
33	44
39	44
49	36
31	52
24	35
45	32

34	51
43	37
55	37
44	39
51	38
39	37
32	47
37	41
43	44
46	40
48	41
43	44
38	44
30	51
41	42
36	40
48	33
31	42
33	49
33	49
33	33
40	43
29	45
44	31
48	48
40	36
34	49
34	32
43	33
48	31
43	34
47	41
45	51
42	43
36	56
51	31
34	33
44	30
46	31
42	41
34	48
45	32
46	35
49	43
42	34
40	40
48	24
48	53
43	31
55	42
47	32
36	37
35	30
41	43
40	41
38	48
47	37
35	41
50	52

45	43
37	44
38	42
36	36
38	40
34	46
44	45
47	44
55	50
42	34
45	43
40	43
29	41
47	40
35	41
33	37
34	36
59	38
37	43
37	45
35	45
34	48
45	42
35	45
32	48
32	37
33	40
68	34
38	52
38	30
35	40
48	29
31	37
39	39
47	43
54	25
42	39
52	30
47	60
42	28
39	41
36	45
33	58
41	46
42	55
34	32
32	36
48	37
48	45
40	40
35	66
38	49
38	48
37	44
46	35
41	46
35	32
39	43
29	42

36	37
34	47
44	39
50	43
50	37
33	42
39	44
37	36
42	42
29	43
26	37
41	34
46	35
27	61
58	41
53	33
44	30
38	46
36	25
43	52
46	39
42	41
44	32
52	34
35	30
39	38
33	41
29	35
42	47
54	29
41	32
46	31
52	30
50	31
42	31
40	50
37	40
48	36
23	49
53	39
44	37
44	47
32	37
37	39
44	34
34	36
47	43
46	39
35	40
39	41
40	40
41	40
37	38
43	38
49	44
60	35
32	43
44	29
44	28

42	58
38	41
43	31
40	51
48	48
28	54
35	57
41	45
31	29
47	40
34	44
48	45
36	33
50	33
41	44
46	32
45	49
28	49
38	38
33	45
32	59
48	39
37	48
37	51
36	36
39	42
42	28
31	41
33	40
39	38
34	51
45	38
54	38
44	36
43	41
54	46
48	50
52	36
50	38
47	44
28	44
46	34
39	37
46	46
39	37
50	30
37	50
38	42
40	44
33	43
55	49
27	34
30	43
39	43
46	36
36	39
47	42
37	58

46	43
43	41
48	37
40	43
33	31
30	45
46	43
46	42
38	38
42	38
46	51
44	44
39	52
36	58
38	30
37	50
29	52
42	38
37	40
61	29
41	62
50	40
39	37
38	38
34	51
43	47
37	48
37	40
46	50
46	44
48	45
45	45
37	43
49	42
35	33
33	65
49	47
32	34
41	48
44	42
34	33
42	41
28	50
40	45
55	28
38	42
42	30
37	70
47	37
45	29
35	29
42	43
42	44
40	30
36	41
34	42
37	53
36	53
32	41

38	27
56	43
33	45
49	43
39	32
33	34
49	49
39	51
53	38
37	38
42	50
26	44
40	42
32	42
32	32
41	44
40	36
58	37
47	46
45	33
37	48
47	45
32	37
37	39
37	43
35	25
52	35
30	45
61	41
48	30
39	46
43	34
45	45
44	42
44	44
36	50
37	36
40	48
48	45
49	39
40	49
34	38
30	45
45	34
29	49
36	58
32	37
35	35
31	39
46	30
54	30
54	32
36	48
41	52
32	45
30	36
43	42
46	44
49	36

42	45
26	47
38	53
34	45
37	49
38	46
38	66
50	28
41	44
61	36
62	34
41	30
35	32
49	45
49	25
36	42
37	35
29	33
30	42
41	37
47	53
47	42
39	49
48	38
30	43
55	41
32	33
32	42
46	43
51	37
50	35
48	40
54	32
33	44
42	39
54	32
36	41
32	54
51	28
41	42
47	42
40	37
34	34
36	27
34	53
48	43
40	39
51	41
38	43
34	46
37	44
35	50
42	41
34	37
49	41
41	53
48	39
35	45
40	37

42	34
29	42
47	33
42	38
59	40
34	31
54	46
28	35
42	42
54	44
40	27
49	44
58	42
48	37
41	43
46	39
33	38
46	33
44	37
43	37
42	35
35	40
38	46
38	51
36	42
52	38
48	36
43	31
36	34
47	47
37	32
38	34
46	44
27	32
39	49
58	36
41	32
44	48
44	39
39	42
41	38
29	47
44	34
47	44
33	44
36	43
46	35
35	32
44	33
47	56
50	35
36	39
48	42
46	36
43	47
34	43
36	42
31	36
40	42

55	31
47	45
52	43
41	47
31	53
30	60
45	30
44	35
34	32
47	51
44	33
37	49
49	38
50	47
31	47
41	36
33	47
28	53
44	35
43	30
47	46
46	38
41	42
47	49
34	35
47	50
50	40
39	45
34	40
49	42
44	28
27	52
52	46
35	41
37	42
51	39
36	30
41	35
43	35
37	45
58	50
43	39
29	41
36	26
32	40
27	44
35	54
31	43
41	41
35	52
46	38
43	29
49	38
50	31
44	32
33	40
33	38
49	33
40	45

39	51
39	33
38	56
35	42
42	29
30	37
34	50
34	37
36	47
42	31
32	41
40	49
31	39
39	33
30	52
41	37
61	39
42	52
45	36
44	33
41	47
44	48
43	40
43	53
36	50
45	39
39	45
35	39
41	52
27	61
32	37
48	38
39	52
37	40
42	32
40	36
38	32
50	31
50	31
44	29
30	31
39	51
42	52
43	36
37	47
43	36
39	30
33	51
37	32
30	46
38	51
43	44
40	31
40	39
41	36
28	45
29	52
53	43
22	47

30	38
49	43
33	54
45	37
41	37
38	38
45	35
41	36
43	37
32	60
53	39
33	52
37	44
43	43
39	36
41	46
45	45
34	43
39	39
41	43
41	48
36	43
45	33
28	53
44	32
38	51
22	45
32	42
41	51
39	30
39	49
38	36
38	46
31	43
31	47
42	34
37	33
37	59
30	37
40	44
44	40
49	31
42	40
36	39
43	47
43	33
48	42
43	46
44	38
25	40
46	33
56	40
45	43
33	53
28	40
37	44
34	44
34	43
37	58

62	42
54	50
30	50
47	42
43	47
46	33
24	39
50	35
35	44
50	44
56	46
40	42
38	35
55	46
58	37
43	32
45	41
47	51
48	47
38	44
31	46
42	44
52	32
40	42
43	36
62	37
50	44
37	48
51	38
22	54
38	45
34	58
34	50
38	51
45	28
42	47
30	43
39	33
51	30
36	39
47	37
42	47
34	28
35	45
38	51
39	40
41	47
42	40
37	42
42	34
45	45
48	41
41	48
34	46
42	33
48	28
39	47
43	47
44	48

41	44
47	47
55	42
49	38
26	34
54	33
47	45
38	36
31	61
51	26
33	43
27	41
47	49
43	47
40	33
45	50
39	50
45	32
43	43
55	30
55	42
38	42
56	41
41	28
36	33
44	37
37	44
37	37
29	56
45	52
38	53
63	41
43	37
35	49
42	32
44	43
43	38
30	48

47	38
39	38
34	51
45	38
54	38
44	36
43	41
54	46
48	50
52	36
50	38
47	44
28	44
46	34
39	37
46	46

39	37
50	30
37	50
38	42
40	44
33	43
55	49
27	34
30	43
39	43
46	36
36	39
47	42
37	58
46	43
43	41
48	37
40	43
33	31
30	45
46	43
46	42
38	38
42	38
46	51
44	44
39	52
36	58
38	30
37	50
29	52
42	38
37	40
61	29
41	62
50	40
39	37
38	38
34	51
43	47
37	48
37	40
46	50
46	44
48	45
45	45
37	43
49	42
35	33
33	65
49	47
32	34
41	48
44	42
34	33
42	41
28	50
40	45
55	28

38	42
42	30
37	70
47	37
45	29
35	29
42	43
42	44
40	30
36	41
34	42
37	53
36	53
32	41
38	27
56	43
33	45
49	43
39	32
33	34
49	49
39	51
53	38
37	38
42	50
26	44
40	42
32	42
32	32
41	44
40	36
58	37
47	46
45	33
37	48
47	45
32	37
37	39
37	43
35	25
52	35
30	45
61	41
48	30
39	46
43	34
45	45
44	42
44	44
36	50
37	36
40	48
48	45
49	39
40	49
34	38
30	45
45	34
29	49

36	58
32	37
35	35
31	39
46	30
54	30
54	32
36	48
41	52
32	45
30	36
43	42
46	44
49	36
42	45
26	47
38	53
34	45
37	49
38	46
38	66
50	28
41	44
61	36
62	34
41	30
35	32
49	45
49	25
36	42
37	35
29	33
30	42
41	37
47	53
47	42
39	49
48	38
30	43
55	41
32	33
32	42
46	43
51	37
50	35
48	40
54	32
33	44
42	39
54	32
36	41
32	54
51	28
41	42
47	42
40	37
34	34
36	27
34	53

48	43
40	39
51	41
38	43
34	46
37	44
35	50
42	41
34	37
49	41
41	53
48	39
35	45
40	37
42	34
29	42
47	33
42	38
59	40
34	31
54	46
28	35
42	42
54	44
40	27
49	44
58	42
48	37
41	43
46	39
33	38
46	33
44	37
43	37
42	35
35	40
38	46
38	51
36	42
52	38
48	36
43	31
36	34
47	47
37	32
38	34
46	44
27	32
39	49
58	36
41	32
44	48
44	39
39	42
41	38
29	47
44	34
47	44
33	44

36	43
46	35
35	32
44	33
47	56
50	35
36	39
48	42
46	36
43	47
34	43
36	42
31	36
40	42
55	31
47	45
52	43
41	47
31	53
30	60
45	30
44	35
34	32
47	51
44	33
37	49
49	38
50	47
31	47
41	36
33	47
28	53
44	35
43	30
47	46
46	38
41	42
47	49
34	35
47	50
50	40
39	45
34	40
49	42
44	28
27	52
52	46
35	41
37	42
51	39
36	30
41	35
43	35
37	45
58	50
43	39
29	41
36	26
32	40

27	44
35	54
31	43
41	41
35	52
46	38
43	29
49	38
50	31
44	32
33	40
33	38
49	33
40	45
39	51
39	33
38	56
35	42
42	29
30	37
34	50
34	37
36	47
42	31
32	41
40	49
31	39
39	33
30	52
41	37
61	39
42	52
45	36
44	33
41	47
44	48
43	40
43	53
36	50
45	39
39	45
35	39
41	52
27	61
32	37
48	38
39	52
37	40
42	32
40	36
38	32
50	31
50	31
44	29
30	31
39	51
42	52
43	36
37	47

43	36
39	30
33	51
37	32
30	46
38	51
43	44
40	31
40	39
41	36
28	45
29	52
53	43
22	47
30	38
49	43
33	54
45	37
41	37
38	38
45	35
41	36
43	37
32	60
53	39
33	52
37	44
43	43
39	36
41	46
45	45
34	43
39	39
41	43
41	48
36	43
45	33
28	53
44	32
38	51
22	45
32	42
41	51
39	30
39	49
38	36
38	46
31	43
31	47
42	34
37	33
37	59
30	37
40	44
44	40
49	31
42	40
36	39
43	47

43	33
48	42
43	46
44	38
25	40
46	33
56	40
45	43
33	53
28	40
37	44
34	44
34	43
37	58
62	42
54	50
30	50
47	42
43	47
46	33
24	39
50	35
35	44
50	44
56	46
40	42
38	35
55	46
58	37
43	32
45	41
47	51
48	47
38	44
31	46
42	44
52	32
40	42
43	36
62	37
50	44
37	48
51	38
22	54
38	45
34	58
34	50
38	51
45	28
42	47
30	43
39	33
51	30
36	39
47	37
42	47
34	28
35	45
38	51

39	40
41	47
42	40
37	42
42	34
45	45
48	41
41	48
34	46
42	33
48	28
39	47
43	47
44	48
41	44
47	47
55	42
49	38
26	34
54	33
47	45
38	36
31	61
51	26
33	43
27	41
47	49
43	47
40	33
45	50
39	50
45	32
43	43
55	30
55	42
38	42
56	41
41	28
36	33
44	37
37	44
37	37
29	56
45	52
38	53
63	41
43	37
35	49
42	32
44	43
43	38
30	48
43	36
53	39
50	32
44	44
45	36
29	54
44	36

32	35
39	50
32	33
41	43
51	47
37	27
59	41
39	39
46	49
33	35
43	55
51	39
50	29
34	42
40	40
30	44
29	36
36	32
52	44
44	38
34	35
37	47
47	37
44	58
40	50
42	34
35	35
47	31
48	35
44	52
39	48
32	35
42	35
48	29
48	40
44	43
46	34
46	39
34	43
39	51
39	34
50	32
47	53
31	44
63	41
35	60
33	47
40	60
41	41
36	38
49	34
42	34
35	33
46	43
42	52
37	34
35	54
42	36
44	34

48	35
35	50
48	38
25	35
27	41
35	37
42	36
31	49
39	47
36	38
50	47
38	39
36	41
47	47
34	39
41	40
41	41
49	42
28	43
41	41
29	49
38	40
42	49
37	40
42	46
38	31
37	50
34	41
40	44
25	50
54	35
49	47
40	52
30	41
37	44
37	40
38	47
33	44
50	39
37	50
39	
54	
39	
31	
42	
43	
38	
45	
48	
48	
44	
42	
48	
42	
34	
45	
45	
37	
33	

38
39
38
48
42
43
42
45
41
27
40
47
45
41
27
31
42
53
42
43
47
45
40
37
31
42
37
38
46
50
36
44
30
41
41
46
37
35
33
28
29
43
41
44
43
40
39
51
37
45
35
34
36
51
57
63
37
34
31

30
44
41
35
48
42
39
24
44
50
44
46
41
43
40
50
45
38
51
42
42
44
45
51
37
34
42
43
33
39
45
41
38
43
36
46
51
43
35
46
28
37
37
44
45
40
35
41
42
47
42
44
35
39
46
52
47
35
32

38
45
43
36
34
46
48
37
50
45
38
40
40
42
36
40
51
38
40
33
46
42
30
47
33
53
43
44
47
45
